# Catalogue of Texas spiders

**DOI:** 10.3897/zookeys.570.6095

**Published:** 2016-03-02

**Authors:** David Allen Dean

**Affiliations:** 1 Department of Entomology, Texas A&M University, College Station, Texas, United States of America

**Keywords:** Distribution, Locality, Caves, Time of activity, Habitat, Method, Type, Collection, Etymology, History of collecting, Thesis

## Abstract

This catalogue lists 1,084 species of spiders (three identified to genus only) in 311 genera from 53 families currently recorded from Texas and is based on the “Bibliography of Texas Spiders” published by Bea Vogel in 1970. The online list of species can be found at http://pecanspiders.tamu.edu/spidersoftexas.htm. Many taxonomic revisions have since been published, particularly in the families Araneidae, Gnaphosidae and Leptonetidae. Many genera in other families have been revised. The Anyphaenidae, Ctenidae, Hahniidae, Nesticidae, Sicariidae and Tetragnathidae were also revised. Several families have been added and others split up. Several genera of Corinnidae were transferred to Phrurolithidae and Trachelidae. Two genera from Miturgidae were transferred to Eutichuridae. Zoridae was synonymized under Miturgidae. A single species formerly in Amaurobiidae is now in the Family Amphinectidae. Some trapdoor spiders in the family Ctenizidae have been transferred to Euctenizidae.

Gertsch and Mulaik started a list of Texas spiders in 1940. In a letter from Willis J. Gertsch dated October 20, 1982, he stated “Years ago a first listing of the Texas fauna was published by me based largely on Stanley Mulaik material, but it had to be abandoned because of other tasks.” This paper is a compendium of the spiders of Texas with distribution, habitat, collecting method and other data available from revisions and collections. This includes many records and unpublished data (including data from three unpublished studies). One of these studies included 16,000 adult spiders belonging to 177 species in 29 families. All specimens in that study were measured and results are in the appendix. Hidalgo County has 340 species recorded with Brazos County at 323 and Travis County at 314 species. These reflect the amount of collecting in the area.

## Introduction

Gertsch and Mulaik (1940) published the first list of spiders in Texas. In a letter from Willis J. Gertsch dated October 20, 1982, he stated “Years ago a first listing of the Texas fauna was published by me based largely on Stanley Mulaik material, but it had to be abandoned because of other tasks.” They described 17 new species in nine families and provided distributions in select families (Ctenizidae, Dipluridae, Euctenizidae, Theraphosidae, Caponiidae, Dictynidae, Diguetidae, Dysderidae, Filistatidae, Mimetidae, Oecobiidae, Oonopidae, Pholcidae, Scytodidae, Segestriidae, Sicariidae, and Uloboridae). Bea Vogel published a “Bibliography of Texas Spiders” in 1970 based on literature records. The current paper is an update of her work and includes data from revisions and labels from specimens and many new records. Her list included 582 species, but she underestimated the diversity of spiders occurring in Texas partly because of more recent collecting in many areas of the state. Fifty-seven names in her list have been synonymized, 17 are not found in Texas, five are nomen dubium, one is undescribed, and three are duplicates resulting in 499 species (Table 4). Many revisions have since been published and much additional collecting has more than doubled the number of species recorded from Texas. Texas is a transition zone which includes extreme range-limits of many species and also has part of its border adjoining Mexico. The climate varies from subtropical in South Texas, to temperate conditions in the panhandle; and from desert in the west, to swamp in the east.

References are listed that mention Texas for each species. Some checklists have been published, which remain the only reference to a species’ occurrence in Texas. Illustrations of the genitalia of a species not included in published reports of a Texas occurrence are included as a reference in brackets. Counties listed are those in which published reports include a species occurring in Texas and includes unpublished records from collections. A species listed as “widespread” is widely distributed across Texas. Several species are listed as “Texas.” The latest name of a species is given with synonymy included where Texas is listed. [T] is a transfer. [S] is synonymy.

Collecting data from locality labels is provided where available. This was taken from collections and revisions. The collections at Texas A&M University, the author’s collection and that at Midwestern State University were searched. Records from West Texas A&M were donated. Cave records from the Texas Memorial Museum are included. The South West Arthropod Network (http://symbiota4.acis.ufl.edu/scan/portal/collections/) was accessed September 13, 2014. It includes records from Denver Museum of Nature & Science, Museum of Comparative Zoology, New Mexico State University, Texas Memorial Museum, and Texas Tech University.

Catalogs of Banks (1910), Bonnet (1955, 1956, 1957, 1958, 1959), Buckle et al. (2001), Crosby (1905), Marx (1890), Petrunkevitch (1911), Roewer (1942, 1955), Roth (1988), Roth and Brown (1986), and Vogel (1962, 1967) were searched. See Brignoli (1983) and Platnick (1989, 1993, 1998, 2000, 2001, and 2003) for updates on new family classifications and current status of species. NMBE – World Spider Catalog (http://www.wsc.nmbe.ch/) was used for recent changes in names. Distribution in Petrunkevitch (1911) listed as all states, North America, East of Rocky Mountains or United States are not included here.

Several spider species have been listed as endangered by the US Fish & Wildlife Service (Federal Register 1998, 2000, 2002, 2003). These are mostly in the families Dictynidae and Leptonetidae.

**Table 1.** Number of species recorded from Texas described by time period.

Years	Number of species	Authors with most species
1755–1799	19	Clerck-9
1800–1824	10	Walckenaer-6
1825–1849	145	Hentz-73 Walckenaer-41
1850–1874	52	Hentz-24
1875–1899	240	Banks-56 O. P.-Cambridge-29 Emerton-40 Keyserling-52 Peckham & Peckham-22 Simon-16
1900–1924	130	Banks-28 F. O. P.-Cambridge-14 Chamberlin-39 Peckham & Peckham-19
1925–1949	257	Chamberlin-26 Chamberlin & others-43 Gertsch-71 Gertsch & Mulaik-45 Gertsch & others-39
1950–1974	72	Gertsch-16 Levi-15
1975–1999	113	Gertsch-56 Gertsch & others-6 Platnick & Shadab-12
2000–2013	43	Ledford et al.-10

**Table 2.** Number of species described by Chamberlin and Gertsch and co-authors in Texas.

	Ch	Ch & I	G	G & D	G & I	G & M	G & W
<1922	12						
1922–1932	37		1				
1933		1	9				
1934			10				
1935		8	20				6
1936	10		17	15	8	28	1
1937–1939		2	1				1
1940	6	5	2	1		17	
1941–1947		16	11	3			
1950’s			5				
1960’s			3				
1970’s			8				
1980’s			5				
1990’s			51				
Total	65	32	143	19	8	45	8

Ch=Chamberlin, Ch & I=Chamberlin & Ivie, G=Gertsch, G & D=Gertsch & Davis, G & I=Gertsch & Ivie, G & M=Gertsch & Mulaik, G & W=Gertsch & Wallace

**Table 3.** Collectors of holotypes in Texas.

Year	S. Mulaik	S. & D. Mulaik	L. I. Davis	J. R. Reddell	Other	Unknown	Total
earlier					7	2	9
1933	8				3		11
1934	35	1	6		3		45
1935	27		6		4		37
1936	5		10		2		17
1937	2				2		4
1938		1				2	3
1939	2	8			1		11
1940					2		2
1941		1			1		2
1942–48					4	1	5
1950					2		2
1952					4		4
1956–59					5		5
1960					3		3
1961					2		2
1962				2	1		3
1963				12	4		16
1964				8	2		10
1965				4			4
1966				3	1		4
1967–69				1	6		7
1970’s				2	12		14
1980’s				1	12		13
1990’s				3	7		10
2000-				2	9		11
no date	9				11	42	62
Total	88	11	22	38	110	47	316

**Table 4.** Comparison of number of genera and species in this publication versus Vogel (1970b).

	This publication		Vogel 1970b	
Family	Number genera	Number species	Number genera	Number species
Atypidae	1	2		
Ctenizidae	1	7	1	5
Dipluridae	1	2	1	2
Euctenizidae	3	4	2	2
Theraphosidae	1	18	1	11
Agelenidae	5	15	5	11
Amphinectidae	1	1		
Anyphaenidae	5	19	1	2
Araneidae	28	94	17	34
Caponiidae	2	2	2	2
Clubionidae	2	12	2	12
Corinnidae	4	15	1	1
Ctenidae	3	3	1	1
Dictynidae	12	115	11	48
Diguetidae	1	4	1	4
Dysderidae	1	1	1	1
Eutichuridae	2	3	2	2
Filistatidae	3	4	3	4
Gnaphosidae	22	104	15	33
Hahniidae	2	7	1	2
Hersiliidae	1	1	1	1
Leptonetidae	3	21	1	1
Linyphiidae	27	74	12	25
Liocranidae	1	1		
Lycosidae	17	86	14	37
Mimetidae	2	7	2	4
Miturgidae	3	3	1	1
Mysmenidae	1	1	1	1
Nephilidae	1	1		
Nesticidae	2	8	2	2
Oecobiidae	1	3	1	2
Oonopidae	6	9	5	7
Oxyopidae	3	15	3	12
Philodromidae	6	38	5	13
Pholcidae	10	18	8	12
Phrurolithidae	4	11	3	5
Pisauridae	3	8	2	3
Plectreuridae	1	1		
Prodidomidae	1	1	1	1
Salticidae	49	147	32	62
Scytodidae	1	6	1	5
Segestriidae	1	1	1	1
Selenopidae	1	1	1	1
Sicariidae	1	5	1	3
Sparassidae	3	3		
Symphytognathidae	1	1		
Tetragnathidae	6	17	3	7
Theridiidae	34	96	31	75
Thomisidae	11	45	8	29
Titanoecidae	1	3	1	2
Trachelidae	2	5	1	1
Uloboridae	5	9	4	6
Zoropsidae	2	6	1	3
Total	311	1084	215	499

## History of collecting in Texas

**General**: Some areas of Texas have been heavily collected (Rio Grande Valley, Austin, College Station, Wichita Falls) while many areas remain little collected.

**Sampling of counties**: Many studies of spiders have been undertaken in Texas. Those based on a particular county include: Brazos (Dean and Sterling 1990, Henderson 2007), Dallas (Jones 1936), Ellis (Hunter 1988), Erath (Agnew et al. 1985), Galveston (Rapp 1984), Nacogdoches (Brown 1974), Potter (Roberts 2001), Smith (Rydzak and Killebrew 1982), Travis (Vincent and Frankie 1985), Walker (Dean and Sterling 1990; Dean et al. 1982), and Wichita (Carpenter 1972). Dean and Sterling (1987) and Gertsch and Mulaik (1940) attempted to study spiders across the state. Broussard and Horner (2006) studied a remote area of western Texas. Salmon and Horner (1977) studied ballooning spiders but did not identify them to species.

**Sampling of agroecosystems**: Many agroecosystems have been studied: cabbage (Irungu 2007), citrus (Breene et al. 1993a), corn (Knutson and Gilstrap 1989), cotton (Breene et al. 1993c, Dean et al. 1982, Dean and Sterling 1987, Kagan 1943, Pamanes-Guerrero 1975), guar (Rogers and Horner 1977), peanut (Agnew et al. 1985), pecan (Bumroongsook et al. 1992, Calixto et al. 2013, Liao et al. 1984), rice (Woods and Harrel 1976), saltcedar (Knutson et al. 2010), sugarcane (Breene et al. 1993b), wildflowers (Dean and Eger 1986), and woolly croton (Breene et al. 1988).

Cokendolpher et al. (2008) studied playas in the Texas panhandle. Yantis (2005) studied the spiders under trees (pine and post oak) in unmanaged habitats. Quinn (2000) and Wharton et al. (1996) studied the potential prey of the golden-cheeked warbler in juniper, oak and pine.

Jackman et al. (2008) studied the spiders collected from a large web at Lake Tawakoni State Park that received worldwide attention. A website (http://www.texasento.net/Social_Spider.htm) maintains the history of this story and mentions other webs. The major species involved was *Tetragnatha guatemalensis* O. P.-Cambridge. Two orb-weaver species that contributed to the web included *Larinioides cornutus* (Clerck) and *Metazygia wittfeldae* (McCook). A large web was found in 2010 at the Nails Creek Unit of Lake Somerville State Park in Lee County and another one in 2015 at Lakeside Park South in Dallas County. Both of these webs included the same species.

**Sampling of families**: Studies of specific families of spiders include: Gnaphosidae (Bowen et al. 2004, Zolnerowich and Horner 1985), Salticidae (Carpenter 1972, Hunter 1988), and crab spiders (Cokendolpher et al. 1979, Rydzak and Killebrew 1982).

**Miscellaneous sampling**: Spiders collected by mud dauber wasps were studied by Dean et al. (1988). A survey of ballooning spiders in east Texas was done by Dean and Sterling (1985, 1990). Reddell (1965, 1970) investigated the cave fauna. More recently, Cokendolpher (2004a), Cokendolpher and Reddell (2001a, b), Reddell and Cokendolpher (2004) have studied the fauna of select caves. Goetze and Flores (2001a, b) sampled spiders in Laredo but only identified them to family. Yantis (2005) sampled two major vegetation types: evergreen forest (pine) versus deciduous woodland (post oak woodland) to examine the influence of vegetation and soil on the occurrence of plant and animal species. In each plot, the percentage of trees was determined and noted here under habitat.

**Theses and dissertations on Texas spiders**: An online search of colleges and universities in Texas has turned up 46 theses and dissertations on Texas spiders that were identified either as the focus of the study or part of it. That includes 12 different colleges/universities (Lamar University in Beaumont [2], Midwestern State University in Wichita Falls [16], North Texas State University in Denton [1], Southern Methodist University in Dallas [1], Texas A&M University in College Station [13], Texas Tech University in Lubbock [2], Texas A&M International University in Laredo [1], Texas Christian University in Fort Worth [2], University of Houston [1], University of Texas at Arlington [3], University of Texas in Austin [3], and West Texas A&M University at Canyon [1].

Seventeen did not publish their work: Brady 1959, Cate 1992, Hanss 2000, Henderson 2007, Hunter 1988, Irungu 2007, Li 1990, Matelski 1982, Matts 1978, Pamanes-Guerrero 1975, Powell 2014, Quinn 2000, Reddick 1996, Roberts 2001, Trevino 2014, Yantis 2005, and Zaltsberg 1977.

An additional twenty-nine published their work [citation in brackets]: Agnew 1981 [Agnew et al. 1982], Barron 1995 [Barron et al. 1999], Bowen 2002 [Bowen et al. 2004], Breene 1988 [Breene et al. 1988, Breene et al. 1989], Broussard 2002 [Broussard and Horner 2006], Brown 1984 [Cokendolpher and Brown 1985], Bumroongsook 1986 [Bumroongsook et al. 1992], Carpenter 1969 [Carpenter 1972], Cokendolpher 1978 [Cokendolpher et al. 1979], Gann 2014 [Gann et al. 2015], Hamilton 2008 [Hamilton and Craig 2008, Hamilton et al. 2012], Hamilton 2009 [Hamilton et al. 2011], Harwood 1970 [Harwood 1974], Higgins 1988 [Higgins 1989, Higgins 1990, Higgins 1992b, Higgins and McGuinness 1990], Hoffmaster 1983 [Hoffmaster 1985], Horner 1967 [Horner and Stewart 1967], Janowski-Bell 1995 [Janowski-Bell and Horner 1999], Jones 1935 [Jones 1936], Kagan 1942 [Kagan 1943], Knutson 1987 [Knutson and Gilstrap 1989], Liao 1984 [Liao et al. 1984], Pickett 1985 [Pickett and Gilstrap 1986], Pritchett 1904a [Pritchett 1904b], Salmon 1976 [Salmon and Horner 1977], Steffenson 2014 [Steffenson et al. 2014], Tugman 1987 [Tugman et al. 1990], Woods 1974 [Woods and Harrel 1976], Zhang 2002 [Zhang et al. 2004], and Zolnerowich 1983 [Zolnerowich and Horner 1985].

**Collectors**: Many people have collected spiders in Texas. Among the earliest were Stanley and Dorothea Mulaik who collected many spiders from 1933–1940, mostly from 1934–1935, and holotypes of 99 species. They were counselors at several camps in the summer and Stanley taught at several institutions. They collected spiders, scorpions, turtles, and other small invertebrates mostly from the Rio Grande Valley toward Laredo and were paid a few cents each by the American Museum of Natural History. They moved to Utah in 1939 where Stanley pursued his PhD with Dr. Ralph Chamberlin. He described new taxa of isopods in his dissertation. He taught for many years and he and his wife were involved in several organizations. L. Irby Davis collected mostly in Cameron Co. from 1934–1936 with 22 holotypes collected. He went on to become a noted ornithologist. James Reddell studied cave fauna for many years and collected 38 holotypes from caves from 1962–2001 and an additional 7 species with colleagues, and greatly improved the knowledge of the fauna of Texas caves. The author has collected in more than one-half of Texas counties. A table containing numbers of species by county is in the appendix.

General keys to spiders include Kaston’s (1978) well-illustrated book. Jackman (1997) is a good field guide with color pictures of the more common spiders in Texas. Ubick et al. (2005) is the best illustrated general guide with chapters on all families and genera in the United States. Spiders of Connecticut (Kaston 1981) includes illustrations of species that are hard to find elsewhere.

Lowrie (1987) reported on the distribution and time periods of taxonomists who described spiders from Wisconsin.

Early workers were Europeans who described American species: Baron Charles A. Walckenaer from France (total of 47 species, 19 in 1837 and 22 in 1841), Count Eugen Keyserling from Germany (total of 54 species, 16 in 1880 and 12 in 1884), and others. Octavius P.-Cambridge from England (35 species from 1861–1902) and his nephew F. O. P.-Cambridge from England (15 species from 1899–1904) described many new spiders from Central America.

Twelve countries outside of the United States are represented mostly in the nineteenth century including England-66 species (5 workers), France-83 species (9 workers), and Germany-79 (7 workers). Bonnet (1945, in French) contains biographies of all arachnologists before 1940.

Early workers from America include: Nicholas M. Hentz (total of 98 species from 1821–1850, 11 in 1844, 15 in 1846, 32 in 1847, and 24 in 1850), James H. Emerton (total of 51 species from 1875–1924, 22 in 1882, 5 in 1884, 6 in 1890, and 4 in 1913), George W. and Elizabeth Peckham (total of 41 species from 1883–1909, 10 in 1888, 10 in 1901, and 9 in 1909), and Nathan Banks (total of 85 species from 1892–1926, 10 in 1892, 12 in 1895, 15 in 1896, 13 in 1898, 5 in 1901, and 13 in 1904). Henry C. McCook described 9 species from 1887–1894 and Thomas H. Montgomery described 9 species from 1902–1904.

Later American authors include: Ralph V. Chamberlin (total of 65 species from 1908–1940, 8 in 1919, 11 in 1922, 13 in 1924, 10 in 1936). He collaborated with three authors: Gertsch (11 species), Ivie (32 species from 1933–1945, 8 in 1935, 6 in 1944), and Angus M. Woodbury (3 species in 1929).

Willis J. Gertsch described 143 species from 1932–1992, 9 in 1933, 10 in 1934, 20 in 1935, 17 in 1936, 6 in 1941, 8 in 1974, 5 in 1984, and 51 in 1992. During the 1930’s, he collaborated with L. Irby Davis (19 species, 15 in 1936) and Stanley Mulaik (45 species, 28 in 1936, 17 in 1940). Gertsch also co-authored papers with Allan F. Archer (4 species), Wilton Ivie (8 species), Howard K. Wallace (8 species), Franklin Ennik (2 species), Norman I. Platnick (1 species), and Susan E. Riechert (3 species). Gertsch also collected in many localities in the United States and Mexico.

Herbert W. Levi described 23 species of araneids and theridiids from 1953–2003. Norman I. Platnick described 7 species and 12 with Mohammed Shadab from 1975–1988. James C. Cokendolpher described 9 species and 7 with other authors. Joel Ledford and coauthors described 10 species of leptonetids in 2012.

A total of 316 species were described from Texas and named from the following categories: location (11 city/town, 16 county, 21 state, 10 other); person (16 collector, 7 arachnologist, 40 other); appearance (18 morphology, 6 eyes, 3 color, 2 markings, 10 size); 2 Indian; 9 name of cave; 5 mountains; and 140 miscellaneous. Nine species were named after Stanley and Dorothea Mulaik who collected many spiders from Texas in the 1930’s.

### Stanley and Dorothea Mulaik

Counties and number of species collected include: Brewster (1), Brooks (1), Cameron (6), Hays (2), Hidalgo (58), Jeff Davis (1), Jim Wells (1), Kendall (1), Kerr (3), Kleberg (1), Matagorda (1), Randall (1), Starr (7), Terrell (5), Tom Green (1), Val Verde (3), Webb (1), and Zapata (5).

### L. Irby Davis

Counties and number of species collected include: Bexar (3), Brewster (1), Cameron (10), Kendall (1), Llano (6), and Travis (1).

### J. R. Reddell

Counties and number of species collected include: Bandera (1), Bell (2), Bexar (5), Burnet (1), Childress (1), Coryell (1), Culberson (1), Hays (2), Kendall (1), Medina (2), Menard (1), Real (1), San Saba (2), Sutton (1), Travis (4), Uvalde (4), Val Verde (6), and Williamson (2).

Vogel (1970b) listed 582 species (57 synonyms, 17 are not found in Texas, 5 are nomen dubium, one is undescribed, and three are duplicates) resulting in 499 valid species.

Listing under each species where data is available:

**Distribution.** general distribution followed by Texas counties in which it occurs

**Locality.** parks, forests, caves, etc.

**Caves.** caves by county

**Time of activity.** month (s) of year males and females were collected, a range in “” is a period with no month specified

**Habitat.** habitat (divided by category: crops, grass, landscape features, littoral, nest/prey, objects, orchard, plants, soil/woodland, structures, web)

**Method.** collecting method with sex (m=male, f=female) of spider(s) collected by each method

**Eggs/spiderlings.** number of eggs found in an eggsac or number of spiderlings found in an eggsac or on a female spider (i.e., collected from pitfall trap)

**Type.** data on species type specimen

**Male/Female.** noted if only one sex is known

**Etymology.** origin of species name

**Collection.** museums where collection data was obtained

**Note.** note on location or species

These books (Jaeger 1959, Webster’s New Universal Unabridged Dictionary 1996, Woods 1944) were used to determine etymology not listed in description or revision.

Localities listed as “the Basin” in Brewster Co. are listed here as Chisos Basin.

Collection abbreviations are: JCC (James C. Cokendolpher, personal collection), MSU (Midwestern State University, Wichita Falls), TAMU (Texas A&M University Insect Collection, College Station, part of author’s personal collection has been donated), TMM (Texas Memorial Museum, Austin- now named Texas Natural History Collections), TTU (Texas Tech University, Lubbock), WTAM (West Texas A&M University, Canyon), AMNH (American Museum of Natural History, New York), DMNS (Denver Museum of Nature & Science), FSCA (Florida State Collection of Arthropods, Gainesville), MCZ (Museum of Comparative Zoology, Boston), NMSU (New Mexico State University, Las Cruces), SIUC (Southern Illinois University at Carbondale), and USNM (United States National Museum, Washington, D. C.).

Spiders are divided by suborder, then alphabetical by family, genus and species.

## Taxonomy

### Suborder Mygalomorphae

#### Family Antrodiaetidae Gertsch, 1940

**Note.** species incorrectly reported from Texas

*Antrodiaetus robustus* (Simon, 1891); Coyle 1971: 345 [disproved Texas as locality (Starr Co.) because specimen was collected by George Marx who was notorious for inaccurate label data]; Gertsch and Mulaik 1940: 311; Vogel 1962: 246; Vogel 1970b: 28 [not in Texas]

*Brachybothrium robustum* Simon, 1891; Petrunkevitch 1911: 52; Roewer 1942: 190

#### Family Atypidae Thorell, 1870

##### Genus *Sphodros* Walckenaer, 1835

###### Sphodros paisano

Taxon classificationAnimaliaAraneaeAtypidae

Gertsch & Platnick, 1980

Sphodros paisano Gertsch and Platnick 1980: 20, f, desc. (figs 30–31); Jackman 1997: 24, 160; Platnick 1986: 140, m, desc. (figs 1–2)

####### Distribution.

Cameron, Travis

####### Locality.

Sabal Palm Audubon Sanctuary

####### Time of activity.

Male (May 31-August 10); female (March)

####### Type.

Mexico, Tamaulipas, Rancho El Milagro, Cruillas

####### Etymology.

Spanish, noun, countryman

###### Sphodros rufipes

Taxon classificationAnimaliaAraneaeAtypidae

(Latreille, 1829)

Sphodros rufipes Bradley 2013: 103; Gertsch and Platnick 1980: 21 [S], mf, desc. (figs 2, 5–6, 11–12, 32–36); Jackman 1997: 24, 160
Atypus bicolor Lucas, 1836; Gertsch, 1979: 124

####### Distribution.

Liberty

####### Time of activity.

Female (January)

####### Type.

Pennsylvania, Philadelphia

####### Etymology.

Latin, color

#### Family Ctenizidae Thorell, 1887

**Note.** species incorrectly reported from Texas

*Bothriocyrtum californicum* O. P.-Cambridge, 1874; Banks 1910: 2 [misidentified] [not in Texas]

##### Genus *Ummidia* Thorell, 1875

###### Ummidia absoluta

Taxon classificationAnimaliaAraneaeCtenizidae

(Gertsch & Mulaik, 1940)

Ummidia absoluta Brignoli 1983: 117 [T]; Jackman 1997: 160
Pachylomerides absolutus Gertsch and Mulaik 1940: 311, f, desc. (fig. 10); Vogel 1962: 246; Vogel 1970b: 28

####### Distribution.

Bandera

####### Time of activity.

Female (“July-August”)

####### Type.

Texas (female, Bandera Co., Bandera, July-August 1937, B. Hale, holotype, AMNH)

[male unknown]

####### Etymology.

Latin, easily separated species

###### Ummidia audouini

Taxon classificationAnimaliaAraneaeCtenizidae

(Lucas, 1835)

Ummidia audouini Jackman 1997: 160 [Roddy 1957: 286 [T] (figs 5–6)]
Pachylomerides audouini (Lucas, 1835); Gertsch and Mulaik 1940: 311; Vogel 1962: 246; Vogel 1970b: 28

####### Distribution.

East Texas

####### Type.

Unknown

####### Etymology.

Person (arachnologist)

###### Ummidia beatula

Taxon classificationAnimaliaAraneaeCtenizidae

(Gertsch & Mulaik, 1940)

Ummidia beatula Brignoli 1983: 117 [T]; Jackman 1997: 160
Pachylomerides beatulus Gertsch and Mulaik 1940: 312, f, desc. (fig. 11); Vogel 1962: 246; Vogel 1970b: 29

####### Distribution.

Dallas

####### Time of activity.

Female (December)

####### Type.

Texas (female, Dallas Co., 5–6 miles S Dallas, December 1937, J. C. Sanders, holotype, AMNH)

[male unknown]

####### Etymology.

Latin, fine spider

###### Ummidia celsa

Taxon classificationAnimaliaAraneaeCtenizidae

(Gertsch & Mulaik, 1940)

Ummidia celsa Brignoli 1983: 117 [T]; Jackman 1997: 160
Pachylomerides celsus Gertsch and Mulaik, 1940; Gertsch and Mulaik 1940: 313, m, desc. (figs 14–15) [see note below]; Vogel 1962: 246; Vogel 1970b: 29

####### Distribution.

Zapata

####### Time of activity.

Male (August)

####### Type.

Texas (male, Zapata Co., 32 miles SW Laredo, August 4, 1935, S. Mulaik, holotype)

[female unknown]

####### Etymology.

Latin, chelicerae nearly black, prominent, rugose

####### Note.

32 miles SW Laredo should be 32 miles SE Laredo in Zapata Co. based on other records from this date.

###### Ummidia funerea

Taxon classificationAnimaliaAraneaeCtenizidae

(Gertsch, 1936)

Ummidia funerea Platnick 2000 [spelling]
Pachylomerus funereus Gertsch, 1936; Bonnet 1958: 3291; Gertsch 1936: 1, m, desc. (figs 1–2)
Pachylomerides funereus (Gertsch, 1936); Gertsch and Mulaik 1940: 312 [T]; Roewer 1942: 149; Vogel 1962: 246; Vogel 1970b: 29.
Ummidia funereus (Gertsch, 1936); Jackman 1997: 160; Roewer 1955: 1712 [T]

####### Distribution.

Hidalgo, Webb, Wichita

####### Time of activity.

Male (April – June, September)

####### Type.

Texas (male, Hidalgo Co., Edinburg, June 1, 1935, S. Mulaik, holotype, AMNH)

[female unknown]

####### Etymology.

Latin, funereal

####### Collection.

MSU

###### Ummidia pygmaea

Taxon classificationAnimaliaAraneaeCtenizidae

(Chamberlin & Ivie, 1945)

Ummidia pygmaea Brignoli 1983: 117 [T]
Pachylomerides pygmaeus Chamberlin and Ivie 1945 [Chamberlin and Ivie 1945a: 558, m, desc. (figs 11–12)]

####### Distribution.

Wichita

####### Type.

Oklahoma, Eagletown

####### Etymology.

Latin, pygmy

####### Collection.

MSU

###### Ummidia tuobita

Taxon classificationAnimaliaAraneaeCtenizidae

(Chamberlin, 1917)

Ummidia tuobita Platnick 1998: 123 [T]
Pachylomerus tuobitus Chamberlin, 1917 [Chamberlin 1917: 33, m, desc. (pl. 1, figs 6–8)]
Pachylomerides tuobitus (Chamberlin, 1917) [Chamberlin and Ivie 1945a: 556, mf (figs 13–15)]

####### Distribution.

Brewster

####### Type.

Illinois

####### Etymology.

Latin, a tube

####### Collection.

MSU

#### Family Dipluridae Simon, 1889

##### Genus *Euagrus* Ausserer, 1875

###### Euagrus chisoseus

Taxon classificationAnimaliaAraneaeDipluridae

Gertsch, 1939

Euagrus chisoseus Coyle 1988: 267 [S], mf, desc. (figs 24–26, 39–43, 50, 223–251); Gertsch 1939b: 21, desc.; Gertsch and Mulaik 1940: 308, m (fig. 9); Jackman 1997: 27, 160; Taber and Fleenor 2003: 239; Vogel 1962: 247; Vogel 1970b: 29
Euagrus ravenus Gertsch & Mulaik, 1940; Gertsch and Mulaik 1940: 308, mf, desc. (figs 8, 13); Reddell 1970: 405; Vogel 1962: 247; Vogel 1970b: 29
Euagrus apacheus Gertsch & Mulaik, 1940; Gertsch and Mulaik 1940: 309, mf, desc. (figs 7, 12); Vogel 1962: 247; Vogel 1970b: 29

####### Distribution.

Central and west Texas; Bandera, Bastrop, Bell, Bexar, Blanco, Brewster, Burnet, Comal, Crockett, Culberson, Edwards, Hays, Jeff Davis, Kendall, Kerr, Kimble, Llano, Presidio, Sutton, Terrell, Travis, Uvalde, Wichita

####### Locality.

Bastrop State Park, Big Bend National Park, Big Bend Ranch State Park, Chisos Basin, Chisos Mountains, Davis Mountains, Guadalupe Mountains National Park, Inks Lake State Park, Lake Travis, Mo Ranch, Pedernales Falls State Park, Raven Ranch, Travis Park, Zilker Park

####### Caves.

**Edwards** (Punkin Cave)

####### Time of activity.

Male (March – October, December); female (February – December)

####### Habitat.

(landscape features: cave, crevices in steep road bank, under [rock, stone, stones at edge of limestone creek in disturbed area]); (littoral: by creek at light, creek); (soil/woodland: oak woods, oak-juniper woods, oak-pine litter, under log); (web: tubular-maze webs in crevices in steep road bank, web in duff covered ravine bank)

####### Method.

Berlese funnel [f]; carrion trap [m]

####### Type.

Texas (male, Brewster Co., Chisos Mountains, Chisos Basin, August 2, 1938, no collector, holotype, AMNH)

####### Etymology.

locality (mountains)

####### Collection.

MSU, NMSU, TMM, TTU

###### Euagrus comstocki

Taxon classificationAnimaliaAraneaeDipluridae

Gertsch, 1935

Euagrus comstocki Bradley 2013: 122; Coyle 1988: 273, mf, desc. (figs 252–259) [see note below]; Gertsch and Mulaik 1940: 309 [see note below]; Jackman 1997: 27, 160, desc.; Kaston 1953: 33, desc. (fig. 62); Kaston 1972: 65, desc. (fig. 150); Kaston 1978: 68, desc. (figs 165–167); Roewer 1942: 203; Taber and Fleenor 2003: 240; Vogel 1970b: 29
Evagrus comstocki Gertsch, 1935; Bonnet 1956: 1848; Comstock 1940: 246, desc.; Gertsch 1935a: 3, mf, desc. (figs 2, 7–8) [Terrell & Travis Co. are Evagrus chisoseus]

####### Distribution.

Hidalgo, Starr, Webb, Zapata

####### Time of activity.

Male (March – April, September, November); female (January, March – April, June – July, September – November)

####### Habitat.

(soil/woodland: under shrub)

####### Type.

Texas (male, Hidalgo Co., Edinburg, November 11, 1934, S. Mulaik, holotype, AMNH)

####### Etymology.

Person (arachnologist)

####### Note.

32 miles E Laredo and 32 miles SW Laredo should be 32 miles SE Laredo in Zapata Co. based on other records from this date.

#### Family Euctenizidae Raven, 1985

**Note.**genera transferred from Cyrtaucheniidae by Bond & Hedin, in Bond et al. 2012: 8

##### Genus *Entychides* Simon, 1888

###### Entychides arizonicus

Taxon classificationAnimaliaAraneaeCyrtaucheniidae

Gertsch & Wallace, 1936

Entychides arizonicus Bond 2005: 47
Eutychides arizonicus Gertsch & Wallace, 1936 [Gertsch and Wallace 1936: 20, m, desc. (figs 26–31)]
Entychides arizonica Gertsch & Wallace, 1936; Bond and Opell 2002: 516, f, desc. (figs 12A–B)

####### Distribution.

Archer, Bell, Brazos, Brewster, Brown, Erath, San Patricio, Travis, Wichita.

####### Locality.

Big Bend National Park, Chisos Basin, Lick Creek Park

####### Time of activity.

Male (January, April, August – October, December); female (March, December)

####### Method.

Flight intercept trap on ground [m]

####### Type.

Arizona, Santa Catalina Mountains, Sabino Basin

####### Etymology.

locality (state)

####### Collection.

MSU, TAMU

##### Genus *Eucteniza* Ausserer, 1875

###### Eucteniza relata

Taxon classificationAnimaliaAraneaeCyrtaucheniidae

(O. P.-Cambridge, 1895)

Eucteniza relata Bond and Godwin 2013: 45 [S], mf, desc. (figs 23–30)
Astrosoga rex Chamberlin, 1940; Brignoli 1983: 111; Chamberlin 1940b: 5, m, desc.; Chamberlin and Ivie 1945a: 556, m, desc. (figs 8–10); Gertsch 1979: 109; Gertsch and Mulaik 1940: 310; Reddell 1965: 170; Vogel 1962: 246; Vogel 1970b: 28
Eucteniza rex (Chamberlin, 1940); Bond 2005: 46, 47; Bond and Hedin 2006: 81; Bond and Opell 2002: 495, 509 [T], 511, 534 (figs 8A-E); Jackman 1997: 160
Myrmekiaphila comstocki Bishop & Crosby, 1926; Gertsch 1935a: 3, f (fig. 3) [misidentified, see Gertsch and Mulaik 1940: 310]
Astrosoga stolida Gertsch & Mulaik, 1940; Gertsch and Mulaik 1940: 310, mf, desc. (figs 1–4, 26); Vogel 1962: 246
Astrosoga solida Gertsch & Mulaik, 1940; Vogel 1970b: 28
Eucteniza stolida (Gertsch & Mulaik, 1940); Bond 2005: 46, 47; Bond and Opell 2002: 495, 513 [T], 534; Jackman 1997: 160

####### Distribution.

Atascosa, Bastrop, Bell, Bexar, Dimmit, Duval, Hidalgo, Houston, Kendall, Kenedy, Kerr, Kleberg, La Salle, Midland, Nueces, Sabine, San Patricio, Starr, Sutton, Travis, Ward, Webb, Zapata

####### Locality.

Bastrop State Park, Raven Ranch

####### Caves.

**Travis** (Austin Caverns)

####### Time of activity.

Male (January – February, June – July, September – December); female (February – September, November – December)

####### Habitat.

(landscape features: cave)

####### Type.

Mexico, Amula in Guerrero

####### Etymology.

Latin, returned

####### Collection.

MSU, TAMU, TMM

###### Eucteniza ronnewtoni

Taxon classificationAnimaliaAraneaeCyrtaucheniidae

Bond & Godwin, 2013

Eucteniza ronnewtoni Bond and Godwin 2013: 57, m, desc. (figs 58–63)

####### Distribution.

Brewster, Val Verde

####### Time of activity.

Male (September – October)

####### Habitat.

(landscape features: on rocks)

####### Type.

Texas (male, Val Verde Co., at bridge on Pecos River, September 2, 1968, J. A. Brubaker, F. J. Moore, holotype, AMNH)

####### Etymology.

Person (The specific epithet is a patronym in honor of Dr. Ronald Newton, biologist and Texas native, Bond and Godwin 2013).

##### Genus *Myrmekiaphila* Atkinson, 1886

###### Myrmekiaphila comstocki

Taxon classificationAnimaliaAraneaeCyrtaucheniidae

Bishop & Crosby, 1926

Myrmekiaphila comstocki Bishop and Crosby 1926: 168, m, desc. (figs 7–8); Bond and Opell 2002: 495; Bond and Platnick 2007: 11, mf, desc. (figs 5, 15, 25, 41–47) [see note below]; Bradley 2013: 114; Gertsch 1935a: 3, f (fig. 3); Gertsch and Mulaik 1940: 310; Jackman 1997: 160; Roewer 1942: 168; Vogel 1970b: 28
Myrmekiaphila fluviatilis (Hentz, 1850); Bishop and Crosby 1926: 166; Gertsch 1935a: 3; Gertsch and Mulaik 1940: 310; Henderson 2007: 37, 52–54, 74–76, 79, 82; Jackman 1997: 160; Kaston 1953: 60, desc. (fig. 142); Vogel 1970b: 28; Yantis 2005: 66, 197, 201 [all misidentified]
Myrmeciophila fluviatilis (Hentz, 1850); Comstock 1912: 239; Comstock 1940: 234 [misidentified]
Myrmeciophila comstocki Bishop & Crosby, 1926; Brown 1974: 233

####### Distribution.

Brazos, Cherokee, Clay, Coryell, Grimes, Hardeman, Hidalgo, Houston, Hunt, Kimble, Kleberg, Leon, Madison, Montague, Nacogdoches, Travis, Trinity, Walker, Wichita

####### Locality.

Lick Creek Park, Riley Estate

####### Time of activity.

Male (February – May, October – November); female (April, May, July)

####### Habitat.

(grass: sandy grassland, short grass); (littoral: sandy area, sandy by water); (soil/woodland: disturbed habitat, pine woods [%: 66, 82, 86, 97], post oak savanna with pasture, post oak woodland, post oak woods [%: 41, 49, 56, 74, 77, 82, 84, 92, 96], upland woods); (structures: front porch, under newspaper in garage)

####### Method.

5 gallon bucket trap [m]; pitfall trap [m]

####### Type.

Texas (male, Travis Co., Austin, March 12-18, 1903, J. H. Comstock, holotype, AMNH)

####### Etymology.

Person (collector)

####### Collection.

MSU, TAMU

####### Note.

Palp keys out to *Myrmeciophila foliata* Atkinson, 1886 because the distal dilation of metatarsus I is large (see fig. 14 in Bond and Platnick 2007) and the embolus is thick. However, specimens from Texas that were not seen for their revision have the distal dilation large but is *Myrmeciophila comstocki*. This is based on a Texas population (from several counties) not seen in their revision (Bond and Platnick, pers. comm.).

#### Family Nemesiidae Simon, 1889

**Note.** species incorrectly reported from Texas

*Brachythele longitarsis* Simon, 1891; Gertsch and Mulaik 1940: 310 [Webb Co., recorded by G. Marx]; Petrunkevitch 1911: 53; Roewer 1942: 197; Simon 1891: 319; Smith 1908: 226; Vogel 1970b: 29 [not in Texas]

#### Family Theraphosidae Thorell, 1869

**Note.** species incorrectly reported from Texas

*Aphonopelma seemanni* (Ausserer, 1875) [not in Texas]

*Eurypelma seemanni* Ausserer, 1875; Petrunkevitch 1911: 64; Roewer 1942: 241

**nomen dubium**

*Aphonopelma californicum* Ausserer, 1871; Prentice 1997: 147 [T]

*Eurypelma californicum* Ausserer, 1871; Banks 1910: 4; Comstock 1912: 245; Comstock 1940: 243; Roewer 1942: 239

*Aphonopelma pseudoroseum* (Strand, 1907); Breene et al. 1996: 22, 23; Gertsch and Mulaik 1940: 314 [T]; Jackman 1997: 160; Prentice 1997: 147 [T]; Smith 1995: 131; Vogel 1962: 248; Vogel 1970b: 29

*Eurypelma pseudoroseum* Strand, 1907; Banks 1910: 4; Roewer 1942: 241

*Delopelma pseudoroseum* (Strand, 1907); Bonnet 1956: 1383

*Tapinauchenius texensis* Simon, 1891; Banks 1910: 4; Bonnet 1959: 4240; Breene et al. 1996: 14; Comstock 1912: 246; Comstock 1940: 244; Gertsch and Mulaik 1940: 314 [Maverick Co. by Marx]; Petrunkevitch 1911: 91; Roewer 1942: 257; Vogel 1970b: 29

##### Genus *Aphonopelma* Pocock, 1901

###### Aphonopelma anax

Taxon classificationAnimaliaAraneaeTheraphosidae

(Chamberlin, 1940)

Aphonopelma anax Breene et al. 1996: 16, 23; Jackman 1997: 160; Smith 1995: 71 [T], mf, desc. (figs 83–100)
Dugesiella anax Chamberlin, 1940; Chamberlin 1940a: 34, mf, desc.; Gertsch and Mulaik 1940: 315; McCoy and Clapper 1979: 450; Vogel 1962: 249; Vogel 1970b: 29

####### Distribution.

Cameron, Kleberg, Zapata

####### Locality.

Falcon International Reservoir

####### Type.

Texas (male, Kleberg Co., Kingsville, no date, J. C. Cross, holotype, AMNH)

####### Etymology.

Greek, regal

####### Collection.

DMNS

###### Aphonopelma armada

Taxon classificationAnimaliaAraneaeTheraphosidae

(Chamberlin, 1940)

Aphonopelma armada Breene et al. 1996: 16, 23; Jackman 1997: 160; Smith 1995: 74 [T], f, desc. (figs 118–123)
Dugesiella armada Chamberlin, 1940; Chamberlin 1940a: 32, f, desc.; Gertsch and Mulaik 1940: 315; Vogel 1962: 249; Vogel 1970b: 29

####### Distribution.

Travis

####### Time of activity.

Female (September)

####### Type.

Texas (female, Travis Co., Austin, September 1909, A. Petrunkevitch, holotype, AMNH)

[male unknown]

####### Etymology.

Latin, character of armature of coxae

###### Aphonopelma arnoldi

Taxon classificationAnimaliaAraneaeTheraphosidae

Smith, 1995

Aphonopelma arnoldi Breene et al. 1996: 17, 23; Jackman 1997: 160; Smith 1995: 74, m, desc. (figs 124–134)

####### Distribution.

Crosby

####### Time of activity.

Male (June)

####### Type.

Texas (male, Crosby Co., Crosbyton, June 17, 1963, P. Keathley, holotype, Oklahoma State University)

[female unknown]

####### Etymology.

Person (Named after D. C. Arnold of the Oklahoma State University Entomology Department, Smith 1995).

###### Aphonopelma breenei

Taxon classificationAnimaliaAraneaeTheraphosidae

Smith, 1995

Aphonopelma breenei Breene et al. 1996: 17, 23; Jackman 1997: 160; Smith 1995: 78, f, desc. (figs 151–158)

####### Distribution.

Cameron

####### Time of activity.

Female (November)

####### Type.

Texas (female, Cameron Co., Harlingen, November 1939, B. Brown, holotype, AMNH)

[male unknown]

####### Etymology.

Person (Named after the late Dr. Robert Breene who with Barbara Moore founded the American Tarantula Society in 1991, Smith 1995).

###### Aphonopelma clarki

Taxon classificationAnimaliaAraneaeTheraphosidae

Smith, 1995

Aphonopelma clarki Breene et al. 1996: 18, 23; Jackman 1997: 160; Smith 1995: 87, mf, desc. (figs 211–230)

####### Distribution.

Dallas

####### Time of activity.

Female (January)

####### Type.

Texas (female, Dallas Co., Dallas, January 25, 1959, H. J. Berman, holotype, BMNH)

####### Etymology.

Person (Named after the late Douglas John Clark, curator of arachnology, BMNH, [1931–1971] who died at the tragically young age of 41. A theraphosid enthusiast, he had many live tarantulas in his office. Over the years, as I have worked through the specimen jars, one by one, I have often found him there before me, Smith 1995)

###### Aphonopelma echinum

Taxon classificationAnimaliaAraneaeTheraphosidae

(Chamberlin, 1940)

Aphonopelma echinum [Smith 1995: 96 [T], m, desc. (figs 289–298)]
Dugesiella echina Chamberlin, 1940; Punzo 1991: 277 [Chamberlin 1940a: 36, m, desc.]

####### Distribution.

Brewster, Kerr, Presidio

####### Locality.

Big Bend National [State] Park

####### Time of activity.

Male (March, November)

####### Type.

Colorado, Arkansas Valley

[female unknown]

####### Etymology.

Greek, spiny, hedge-hog like

####### Collection.

MSU

###### Aphonopelma gurleyi

Taxon classificationAnimaliaAraneaeTheraphosidae

Smith, 1995

Aphonopelma gurleyi Breene et al. 1996: 18, 23; Jackman 1997: 160; Smith 1995: 104, m, desc. (figs 359–367)

####### Distribution.

Cooke

####### Type.

Texas (male, Cooke Co., Sherman, Moss Lake, no date, R. Gurley, BMNH)

[female unknown]

####### Etymology.

Person (Named after the collector, amateur entomologist/arachnologist and naturalist, Russ Gurley, Smith 1995).

###### Aphonopelma harlingenum

Taxon classificationAnimaliaAraneaeTheraphosidae

(Chamberlin, 1940)

Aphonopelma harlingenum Breene et al. 1996: 19, 23; Smith 1995: 106 [T], f, desc. (figs 378–383)
Dugesiella harlingena Chamberlin, 1940; Chamberlin 1940a: 37, f, desc.; Gertsch and Mulaik 1940: 315; Vogel 1962: 249
Dugesiella harlingen (Chamberlin, 1940); Vogel 1970b: 29
Aphonopelma harlingena (Chamberlin, 1940); Cokendolpher 1993: 39; Jackman 1997: 160

####### Distribution.

Cameron, Hidalgo

####### Type.

Texas (female, Cameron Co., Harlingen, no date, B. Brown, holotype, AMNH)

[male unknown]

####### Etymology.

locality (city)

###### Aphonopelma hentzi

Taxon classificationAnimaliaAraneaeTheraphosidae

(Girard, 1852)

Aphonopelma hentzi Breene et al. 1996: 22, 23; Cokendolpher et al. 2008: 10, 52, (photos 37–38); Jackman 1997: 160; Janowski-Bell and Horner 1999: 504; Roberts 2001: 48 [Smith 1995: 107 [T], mf, desc. (figs 393–411)]
Mygale hentzii Girard, 1852; Lincecum 1867a: 138; Lincecum 1867b: 409
Eurypelma hentzii (Girard, 1852); Banks 1892: 148; Marx 1890: 502; Rau 1925: 1
Eurypelma hentzi (Girard, 1852); Jones 1936: 69
Dugesiella hentzi (Girard, 1852); Bonnet 1956: 1612; Brown 1974: 237; Gertsch and Mulaik 1940: 315; Petrunkevitch 1911: 60; Roewer 1942: 238; Vogel 1970b: 29
Rhechostica hentzi (Girard, 1852); Formanowicz and Ducey 1991: 2916

####### Distribution.

Archer, Brown, Carson, Clay, Dallas, Nacogdoches, Potter, Starr, Taylor, Travis, Wichita, Wilbarger

####### Locality.

Pantex Lake, Wildcat Bluff Nature Center, W. J. Wagoneer Estate

####### Time of activity.

Male (June – September); female (April – June, September – October, December)

####### Habitat.

(grass: grassland); (landscape features: under rock); (littoral: near playa); (structures: lawn, service station)

####### Type.

unknown

####### Etymology.

Person (arachnologist)

####### Collection.

MSU, TTU

###### Aphonopelma heterops

Taxon classificationAnimaliaAraneaeTheraphosidae

Chamberlin, 1940

Aphonopelma heterops Breene et al. 1996: 19, 23; Chamberlin 1940a: 29, f, desc.; Gertsch and Mulaik 1940: 314; Jackman 1997: 160; Smith 1995: 113, f, desc. (figs 416–422); Vogel 1962: 248; Vogel 1970b: 29

####### Distribution.

Hidalgo

####### Time of activity.

Female (“September-December”)

####### Type.

Texas (female, Hidalgo Co., Edinburg, September-December, 1933, S. Mulaik, holotype, AMNH)

[male unknown]

####### Etymology.

Greek, different, mixed (heter-) + eyes (-ops)

###### Aphonopelma hollyi

Taxon classificationAnimaliaAraneaeTheraphosidae

Smith, 1995

Aphonopelma hollyi Breene et al. 1996: 20, 23; Jackman 1997: 160; Smith 1995: 114, m, desc. (figs 423–434)

####### Distribution.

Lubbock

####### Time of activity.

Male (August)

####### Type.

Texas (male, Lubbock Co., Lubbock, August 1981, C. Moody, holotype, Oklahoma State University)

[female unknown]

####### Etymology.

Person (Named after the singer Buddy Holly who was born in Lubbock, Smith 1995).

###### Aphonopelma marxi

Taxon classificationAnimaliaAraneaeTheraphosidae

(Simon, 1891)

Aphonopelma marxi Prentice 1997: 147 [S, T]
Eurypelma marxi Simon, 1891; Gertsch 1935a: 4; Roewer 1942: 240 [Petrunkevitch 1929: 517, m, desc. (fig. 13)]
Aphonopelma simulatum Chamberlin & Ivie, 1939; Gertsch and Mulaik 1940: 314; Vogel 1962: 248; Vogel 1970b: 29

####### Distribution.

Hidalgo

####### Type.

unknown

[female unknown]

####### Etymology.

Person (arachnologist)

####### Note.

Smith 1995: 119, 120 does not believe it is this species.

###### Aphonopelma moderatum

Taxon classificationAnimaliaAraneaeTheraphosidae

(Chamberlin & Ivie, 1939)

Aphonopelma moderatum Bradley 2013: 222; Breene et al. 1996: 20, 23; Gertsch and Mulaik 1940: 314 [T] [see note below]; Jackman 1997: 160; Smith 1995: 122, m, desc. (figs 500–507); Vogel 1962: 248
Delopelma moderatum Chamberlin & Ivie, 1939; Bonnet 1956: 1382; Chamberlin and Ivie 1939: 9, m, desc. (fig. 5); Vogel 1962: 249
Delopelma modoratum Chamberlin & Ivie, 1939; Vogel 1970b: 29

####### Distribution.

Maverick, Starr, Zapata

####### Time of activity.

Male (March, May); female (September)

####### Type.

Texas (male, Starr Co., 5 miles E Rio Grande City, May 1, 1937, S. Mulaik, holotype, AMNH)

####### Etymology.

Latin, moderate

####### Collection.

DMNS

####### Note.

32 miles SW Laredo should be 32 miles SE Laredo in Zapata Co. based on other records from this date.

###### Aphonopelma mordax

Taxon classificationAnimaliaAraneaeTheraphosidae

(Ausserer, 1871)

Aphonopelma mordax Smith 1995: 124 [T], f, desc. (figs 518–523)
Eurypelma mordax Ausserer, 1871; Ausserer 1871: 211, m, desc. (fig. 14); Marx 1890: 502

####### Distribution.

Texas

####### Type.

unknown

[male unknown]

####### Etymology.

Latin, biting

###### Aphonopelma rusticum

Taxon classificationAnimaliaAraneaeTheraphosidae

(Simon, 1891)

Aphonopelma rusticum Platnick 1993: 100 [T]; Vogel 1970b: 29 [Smith 1995: 137, m, desc. (figs 650–659)]
Eurypelma rusticum Simon, 1891; F. O. P.-Cambridge 1897: 24, m (pl. 1, figs 20–20a); Petrunkevitch 1911: 64; Roewer 1942: 241

####### Distribution.

Texas

####### Type.

Mexico

[female unknown]

####### Etymology.

Latin, rusty abdominal color

###### Aphonopelma steindachneri

Taxon classificationAnimaliaAraneaeTheraphosidae

(Ausserer, 1875)

Aphonopelma steindachneri Breene et al. 1996: 21, 23; Gertsch and Mulaik 1940: 314 [T]; Jackman 1997: 160; Punzo 2007: 66; Smith 1995: 147, m, desc. (figs 744–753); Vogel 1962: 248; Vogel 1970b: 29
Eurypelma steindachneri Ausserer, 1875; Ausserer 1875: 199, mf, desc. (figs 43–44); Comstock 1912: 245, desc.; Comstock 1940: 243, desc.; Gertsch 1939b: 21; Petrunkevitch 1911: 65

####### Distribution.

Brewster, Dallas, Pecos

####### Locality.

Big Bend National Park, Chisos Basin, Chisos Mountains

####### Type.

unknown

####### Etymology.

Person

####### Collection.

MCZ

####### Note.

Hamilton et al. (2011) stated that this species occurs only in California.

###### Aphonopelma texense

Taxon classificationAnimaliaAraneaeTheraphosidae

(Simon, 1891)

Aphonopelma texense Breene et al. 1996: 21, 23; Pérez-Miles et al. 1996: 42, m (fig. 7); Platnick 1998: 151 [spelling]; Smith 1995: 152 [T]
Eurypelma texense Simon, 1891; Banks 1910: 4
Rhechostica texense (Simon, 1891); Comstock 1912: 243, desc.; Comstock 1940: 241, desc; Gertsch and Mulaik 1940: 314 [record by Marx]
Rhechostica texensis (Simon, 1891); Bonnet 1958: 3855; Petrunkevitch 1911: 87; Roewer 1942: 245; Vogel 1970b: 29
Aphonopelma texensis (Simon, 1891); Jackman 1997: 160; Smith 1995: 152 [T], m, desc. (figs 791–799)

####### Distribution.

Maverick, Starr, Zapata

####### Type.

Texas (male, no location, 1880’s, G. Marx, holotype, USNM)

[female unknown]

####### Etymology.

locality (state)

###### Aphonopelma waconum

Taxon classificationAnimaliaAraneaeTheraphosidae

(Chamberlin, 1940)

Aphonopelma waconum Breene et al. 1996: 22, 23; Jackman 1997: 160; Smith 1995: 156 [T], m, desc. (figs 832a–832i)
Dugesiella wacona Chamberlin, 1940; Chamberlin 1940a: 38, m, desc.; Gertsch and Mulaik 1940: 315; Vogel 1962: 249; Vogel 1970b: 29

####### Distribution.

McLennan

####### Time of activity.

Male (July)

####### Type.

Texas (male, McLennan Co., Waco, July 5, 1931, no collector, holotype, AMNH)

[female unknown]

####### Etymology.

locality (city)

### Suborder Araneomorphae

#### Family Agelenidae C. L. Koch, 1837

##### Genus *Agelenopsis* Giebel, 1869

###### Agelenopsis aleenae

Taxon classificationAnimaliaAraneaeAgelenidae

Chamberlin & Ivie, 1935

Agelenopsis aleenae Ayoub et al. 2005: 44; Guarisco 2014: 82, f, desc.; Jackman 1997: 160; Reddell 1965: 168; Reddell 1973: 41; Vogel 1970b: 2; Whitman-Zai et al. 2015: 12, mf, desc. (figs 1–2)

####### Distribution.

Blanco, Briscoe, Clay, Dallas, Howard, Jeff Davis, Llano, San Saba

####### Locality.

Caprock Canyons State Park, Davis Mountains Resort, Lake Arrowhead State Park

####### Caves.

**San Saba** (Dove Cave)

####### Time of activity.

Male (May, October); female (September)

####### Habitat.

(landscape features: cave); (soil/woodland: saltcedar)

####### Method.

Malaise trap [f]

####### Type.

New Mexico, Suwanee

####### Etymology.

Person (Named for Aleen Ivie, wife of arachnologist Wilton Ivie, who collected the specimen, Whitman-Zai et al. 2015).

####### Collection.

NMSU, TAMU, TMM

###### Agelenopsis aperta

Taxon classificationAnimaliaAraneaeAgelenidae

(Gertsch, 1934)

Agelenopsis aperta Ayoub and Riechert 2004: 3465; Ayoub et al. 2005: 44; Bradley 2013: 65; Cokendolpher and Reddell 2001b: 36; Jackman 1997: 160; Kaston 1978: 170; Maupin and Riechert 2001: 570; Reddell 1965: 168; Reddell and Cokendolpher 2004: 76; Reddell and Finch 1963: 50; Riechert 1993: 344; Roewer 1955: 41; Roth and Brown 1986: 4 [S]; Vogel 1970b: 2; Whitman-Zai et al. 2015: 13, mf, desc. (figs 23–24, 41, 56–57)
Agelena aperta Gertsch, 1934; Gertsch 1934d: 25, mf, desc. (fig. 10); Jones 1936: 69
Agelenopsis apertus (Gertsch, 1934); Gertsch 1939b: 25 [T]
Agelenopsis aperta guttata Chamberlin & Ivie, 1941; Chamberlin and Ivie 1941: 595, mf, desc. (fig. 22); Jackman 1997: 160; Roewer 1955: 41; Vogel 1967: 4; Vogel 1970b: 2

####### Distribution.

Bandera, Bastrop, Bell, Bexar, Blanco, Brazos, Brewster, Burleson, Dallas, Edwards, El Paso, Fort Bend, Hidalgo, Kerr, Liberty, Pecos, Randall, Reeves, San Patricio, Tom Green, Travis, Uvalde, Val Verde, Walker, Wichita, Williamson

####### Locality.

Amistad National Recreational Area, Chisos Basin, Chisos Mountains, Fort Hood, Lick Creek Park, Palo Duro Canyon State Park, Raven Ranch

####### Caves.

**Bell** (Rock Ring Sink [Fort Hood]); **Bexar** (Cave of the Half-Snake, Logan’s Cave); **Travis** (Root Cave); **Williamson** (Three-Mile Cave)

####### Time of activity.

Male (May – September); female (June – December)

####### Habitat.

(landscape features: cave); (littoral: grassy field, near water, palmetto-cypress swamp); (soil/woodland: upland deciduous forest)

####### Method.

Flight intercept trap on ground [m]; pitfall trap [f]

####### Type.

Colorado, east of Boulder, Valmont Buttes

####### Etymology.

Latin, opened

####### Collection.

DMNS, MCZ, TAMU, TMM

###### Agelenopsis emertoni

Taxon classificationAnimaliaAraneaeAgelenidae

Chamberlin & Ivie, 1935

Agelenopsis emertoni Ayoub et al. 2005: 44; Chamberlin and Ivie 1935b: 33, m, desc. (fig. 110); Chamberlin and Ivie 1941: 593, mf, desc. (figs 5, 28, 30); Guarisco 2008b: 5; Henderson 2007: 52, 76, 79, 82; Jackman 1997: 160; Roewer 1955: 41; Roth and Brown 1986: 5; Vogel 1967: 4; Vogel 1970b: 2; Whitman-Zai et al. 2015: 14, mf, desc. (figs 25–26, 42, 58); Yantis 2005: 66, 196, 199
Agelenopsis aperta (Gertsch, 1934); Yantis 2005: 196, 199 [misidentified]
Agelenopsis sp. nr emertoni Chamberlin & Ivie, 1935; Jackman et al. 2007: 199 [misidentified]
Agelenopsis sp. nr pennsylvanica (C. L. Koch, 1843); Henderson 2007: 55, 76, 79, 82 [misidentified]

####### Distribution.

Anderson, Bastrop, Bell, Brazos, Burleson, Dallas, Grayson, Grimes, Houston, Hunt, Leon, McLennan, Madison, Nueces, San Patricio, Trinity, Walker, Wichita

####### Locality.

Lake Tawakoni State Park, Lick Creek Park, Welder Wildlife Refuge, White Rock Lake

####### Time of activity.

Male (April, July – November); female (April – June, August – October)

####### Habitat.

(grass: grass); (littoral: moist salt beach); (soil/woodland: disturbed habitat, forest, pine woods [%: 60, 66, 69, 77, 80, 84, 86, 95, 97], post oak savanna with pasture, post oak woods [%: 60, 76, 82, 85, 93, 100], sandy area, sandy brushland, upland woods); (web: large spider web)

####### Method.

5 gallon bucket trap [mf]; beating [mf]; pitfall trap [mf]

####### Type.

Texas (male, Bell Co., Belton, September 1, 1933, W. Ivie, holotype, AMNH)

####### Etymology.

Person (Named for arachnologist James H. Emerton, Whitman-Zai et al. 2015).

####### Collection.

DMNS, MCZ, MSU, TAMU

###### Agelenopsis kastoni

Taxon classificationAnimaliaAraneaeAgelenidae

Chamberlin & Ivie, 1941

Agelenopsis kastoni Yantis 2005: 66, 196, 199 [Whitman-Zai et al. 2015: 15, mf, desc. (figs 19–20, 39, 54)]

####### Distribution.

Cherokee, Grimes, Harris, Leon, Madison, Rusk, Sabine, Trinity, Tyler, Walker

####### Locality.

Kirby State Forest

####### Time of activity.

Male (March 26-April 4, April, April 24-May 3)

####### Habitat.

(soil/woodland: beech-magnolia forest, pine woods [%: 66, 86, 97], post oak woods [%: 49, 71, 91, 92, 94, 96])

####### Method.

5 gallon bucket trap [m]; flight intercept trap/malaise trap [m]; flight intercept trap on ground [m]; malaise trap [m]

####### Type.

Connecticut, Haddam

####### Etymology.

Person (Named for arachnologist Benjamin J. Kaston who collected the holotype, Whitman-Zai et al. 2015).

####### Collection.

MSU, TAMU

###### Agelenopsis longistyla

Taxon classificationAnimaliaAraneaeAgelenidae

(Banks, 1901)

Agelenopsis longistyla Platnick 1998: 618 [spelling]; Whitman-Zai et al. 2015: 16, mf, desc. (figs 15–16, 40, 65)
Agelenopsis longistylus (Banks, 1901); Chamberlin and Ivie 1941: 592, mf, desc. (figs 10, 20, 33); Jackman 1997: 160; Roewer 1955: 42; Vogel 1970b: 2

####### Distribution.

McCulloch, Oldham

####### Time of activity.

Female (September – October)

####### Type.

New Mexico, White Mountains

####### Etymology.

Latin, long stylus on palp

###### Agelenopsis naevia

Taxon classificationAnimaliaAraneaeAgelenidae

(Walckenaer, 1841)

Agelenopsis naevia Ayoub et al. 2005: 44; Broussard and Horner 2006: 253; Brown 1974: 231; Chamberlin and Ivie 1941: 597, mf, desc. (figs 9, 25, 36); Jackman 1997: 93, desc., 160 (photo 24b); Kaston 1953: 131, desc.; Kaston 1972: 178, desc.; Kaston 1978: 169, desc.; Knutson et al. 2010: 515; Richman et al. 2011a: 47; Roth and Brown 1986: 5 [T]; Vogel 1970b: 2; Whitman-Zai et al. 2015: 16, mf, desc. (figs 21–22, 33, 48); Yantis 2005: 66, 196, 199
Agelena naevia Walckenaer, 1841; Jones 1936: 69

####### Distribution.

Anderson, Angelina, Bastrop, Brazos, Brown, Dallas, Fort Bend, Grimes, Henderson, Hidalgo, Houston, Howard, Jeff Davis, Leon, Madison, Montgomery, Nacogdoches, Polk, Presidio, Rusk, Smith, Walker, Waller, Wichita, Wise

####### Locality.

Chihuahuan desert, Dalquest Research Site, Decker’s Prairie, Lick Creek Park, Santa Ana National Wildlife Refuge, Texas A&M University Rangeland Area

####### Time of activity.

Male (March – July, October); female (February – March, June – October)

####### Habitat.

(grass: short grass); (landscape features: under rock); (soil/woodland: pine woods [%: 73, 74, 77, 80, 83, 100], post oak woods [%: 48, 70, 75, 76, 80, 85, 90, 100], saltcedar, tree bark); (web: base of house in web, web across creek bed)

####### Method.

5 gallon bucket trap [mf]; pitfall trap [m]

####### Type.

Georgia

####### Etymology.

Latin, spotted

####### Collection.

DMNS, MCZ, MSU, NMSU, TAMU

###### Agelenopsis oklahoma

Taxon classificationAnimaliaAraneaeAgelenidae

(Gertsch, 1936)

Agelenopsis oklahoma Ayoub et al. 2005: 44 [Whitman-Zai et al. 2015: 17, mf, desc. (figs 17–18, 38, 53)]
Agelenopsis sp. nr oklahoma (Gertsch, 1936); Henderson 2007: 53, 76, 79, 82 [misidentified]

####### Distribution.

Brazos, Clay

####### Locality.

Lake Arrowhead State Park, Lick Creek Park

####### Time of activity.

Male (April)

####### Habitat.

(soil/woodland: upland woods)

####### Method.

pitfall trap [m]

####### Type.

Oklahoma, Stillwater

####### Etymology.

locality (Named for the state from which the species was described, Whitman-Zai et al. 2015).

####### Collection.

TAMU

###### Agelenopsis spatula

Taxon classificationAnimaliaAraneaeAgelenidae

Chamberlin & Ivie, 1935

Agelenopsis spatula Agnew et al. 1985: 4, 9; Ayoub et al. 2005: 45; Chamberlin and Ivie 1935b: 32, mf, desc. (fig. 109); Chamberlin and Ivie 1941: 596, mf, desc. (figs 6, 26, 32); Jackman 1997: 160; Roewer 1955: 43; Vogel 1970b: 2; Whitman-Zai et al. 2015: 21, mf, desc. (figs 13–14, 35, 50); Yantis 2005: 196; Young and Edwards 1990: 14
Agelena spathula (Chamberlin & Ivie, 1935); Bonnet 1955: 201

####### Distribution.

Archer, Brazos, Briscoe, Clay, Dallam, Erath, Frio, Houston, Liberty, Roberts, Travis, Wichita, Williamson

####### Locality.

Caprock Canyons State Park, Lake Kickapoo

####### Time of activity.

Male (September – October); female (February, May, September – November)

####### Habitat.

(crops: peanuts); (grass: short grass); (littoral: rocks near water, under rock); (soil/woodland: on ground, pine woods [%: 88])

####### Method.

5 gallon bucket trap [f]; pitfall trap [mf]

####### Type.

Texas (male, Wichita Co., Wichita Falls, September 3, 1933, W. Ivie, holotype, AMNH)

####### Etymology.

Latin, spoon shaped palp

####### Collection.

DMNS, MCZ, MSU, TAMU

##### Genus *Barronopsis* Chamberlin & Ivie, 1941

###### Barronopsis texana

Taxon classificationAnimaliaAraneaeAgelenidae

(Gertsch, 1934)

Barronopsis texana Ayoub et al. 2005: 45; Guarisco 2008b: 5; Jackman 1997: 160; Lehtinen 1967: 218 [T]; Roth and Brown 1986: 5; Stocks 2009: 17, mf, desc. (figs 2, 5, 16, 18–23, 48, 55–59); Yantis 2005: 196
Agelena texana Gertsch, 1934; Bonnet 1955: 202; Gertsch 1934d: 24, m, desc.; Jones 1936: 69
Agelenopsis texana (Gertsch, 1934); Brown 1974: 231; Chamberlin and Ivie 1941: 601 [T], m, desc. (figs 46–47); Roewer 1955: 43; Roth 1954: 5, m (fig. 4); Vogel 1970b: 2

####### Distribution.

Anderson, Aransas, Blanco, Brazoria, Brazos, Cameron, Dallas, Denton, Fannin, Harris, Hidalgo, Hunt, Kerr, Nacogdoches, Sabine, Travis, Trinity

####### Locality.

Lake Dallas, Lake Tawakoni State Park, Lick Creek Park, Thurmond Lake, White Rock Lake, Zilker Park

####### Time of activity.

Male (March, October – December, December 2-January 17); female (March – May, October – December, December 2-January 17)

####### Habitat.

(grass: in grass near woods); (soil/woodland: ground, mix-pine forest, oak forest, oak woods, palm, pine woods [%: 69], under [bark, log])

####### Method.

5 gallon bucket trap [m]; flight intercept trap [m]; malaise trap [mf]

####### Type.

Texas (male, Hidalgo Co., Edinburg, no date, S. Mulaik, holotype, AMNH)

####### Etymology.

locality (state)

####### Collection.

MCZ, MSU, TAMU

##### Genus *Coras* Simon, 1898

**Note.** genus transferred here from Amaurobiidae (Miller et al. 2010: 802)

###### Coras alabama

Taxon classificationAnimaliaAraneaeAmaurobiidae

Muma, 1946

Coras alabama Brown 1974: 231; Jackman 1997: 160 [Muma 1946: 9, mf, desc. (figs 11, 39–40)]

####### Distribution.

Nacogdoches

####### Time of activity.

Female (March)

####### Habitat.

(objects: under board in empty lot)

####### Type.

Alabama, Madison Co., Monte Sano

####### Etymology.

locality (state)

###### Coras lamellosus

Taxon classificationAnimaliaAraneaeAmaurobiidae

(Keyserling, 1887)

Coras lamellosus [Muma 1946: 6, mf, desc. (figs 7, 27–30)]

####### Distribution.

Anderson, Denton, Grayson, Hardin, Kleberg

####### Locality.

Padre Island

####### Time of activity.

Male (November); female (March – April)

####### Habitat.

(soil/woodland: wooded area)

####### Type.

Virginia, Fort Monroe; Pennsylvania, Altoona; Lake Superior

####### Etymology.

Latin, refers to a thin plate

####### Collection.

MCZ, MSU

###### Coras medicinalis

Taxon classificationAnimaliaAraneaeAmaurobiidae

(Hentz, 1821)

Coras medicinalis Bonnet 1956: 1201; Jackman 1997: 160; Jones 1936: 69; Kaston 1972: 181, desc. (fig. 399); Kaston 1978: 172 (fig. 430); Vogel 1970b: 2 [Muma 1946: 4, mf, desc. (figs 1–3, 21–24)]

####### Distribution.

Dallas

####### Type.

unknown

####### Etymology.

Latin, web used as narcotic in cases of fever

##### Genus *Tegenaria* Latreille, 1804

###### Tegenaria domestica

Taxon classificationAnimaliaAraneaeAgelenidae

(Clerck, 1757)

Tegenaria domestica Jackman, 1997: 94, desc., 160; Reddell and Cokendolpher 2004: 76; Roewer 1955: 77 [S]; Vogel 1970b: 3 [Roth 1968: 11, mf, desc. (figs 13–18)]
Tegenaria derhami (Scopoli, 1763); Jones 1936: 69

####### Distribution.

Bexar, Dallas, Lubbock

####### Caves.

**Bexar** (Cave With A View)

####### Time of activity.

Female (June)

####### Habitat.

(landscape features: cave)

####### Type.

Sweden

####### Etymology.

Greek, “of the house”

####### Collection.

JCC, TMM

###### Tegenaria pagana

Taxon classificationAnimaliaAraneaeAgelenidae

C. L. Koch, 1840

Tegenaria pagana Jackman 1997: 160; Roth 1968: 26 [S], mf, desc. (figs 30–35); Vogel 1970b: 3
Tegenaria antrias Crosby, 1926; Roewer 1955: 79; Roth 1952: 284
Tegenaria simplex Bryant, 1936; Bonnet 1959: 4302; Bryant 1936: 90, f, desc. (fig. 9); Jones 1936: 69

####### Distribution.

Central and northeast Texas; Coryell, Dallas, Fannin, Hays, San Saba, Travis, Wichita

####### Caves.

**Hays** (Ezell’s Cave); **San Saba** (Bremer Cave)

####### Time of activity.

Male (November); female (February, April, November)

####### Habitat.

(landscape features: cave)

####### Type.

Greece

####### Etymology.

Latin, rustic

####### Collection.

MSU, TMM

##### Genus *Tortolena* Chamberlin & Ivie, 1941

###### Tortolena dela

Taxon classificationAnimaliaAraneaeAgelenidae

Chamberlin & Ivie, 1941

Tortolena dela Bennett and Ubick 2005: 59; Chamberlin and Ivie 1941: 615, f, desc. (fig. 79); Jackman 1997: 160; Roewer 1955: 83; Roth 1982: 7–5, 7–6; Roth 1985: B-1–4, B-1–5; Roth 1994: 50, 51; Roth and Brame 1972: 50; Roth and Brown 1986: 11; Vogel 1967: 15; Vogel 1970b: 3

####### Distribution.

Hidalgo

####### Time of activity.

Female (October)

####### Type.

Texas (female, Hidalgo Co., 7 miles E Edinburg, October 14, 1934, S. Mulaik, holotype, AMNH)

[male unknown]

####### Etymology.

undetermined

#### Family Amphinectidae Forster & Wilton, 1973

**Note.** genus transferred here from Amaurobiidae (Davies 1998: 242)

##### Genus *Metaltella* Mello-Leitão, 1931

###### Metaltella simoni

Taxon classificationAnimaliaAraneaeAmphinectidae

(Keyserling, 1878)

Metaltella simoni Cutler 2005a: 63; Jackman 1997: 99, desc., 160 (photo 27a); Reddell and Cokendolpher 2004: 76; Yantis 2005: 197 [Leech 1972: 107, mf, desc. (figs 194–195, 390)]

####### Distribution.

Bexar, Brazos, Colorado, Galveston, Harris, Hidalgo, Leon (imm.), Montgomery, Orange, San Patricio, Wichita

####### Locality.

Attwater Prairie Chicken National Wildlife Refuge, Lick Creek Park

####### Caves.

**Bexar** (Robber Baron Cave)

####### Time of activity.

Male (May – June, August, October – December); female (April – June, August, October – November)

####### Habitat.

(landscape features: cave); (littoral: near water); (objects: wood pile); (soil/woodland: debris under banana trees, leaf litter, post oak savanna, post oak woods [%: 70]); (structures: bathroom, indoors, in structure [bit collector causing reaction], on bed in house)

####### Method.

5 gallon bucket trap [imm.]; pitfall trap [mf]

####### Type.

Uruguay

####### Etymology.

Person (arachnologist)

####### Collection.

MSU, TAMU, TMM

#### Family Anyphaenidae Bertkau, 1878

##### Genus *Anyphaena* Sundevall, 1833

###### Anyphaena celer

Taxon classificationAnimaliaAraneaeAnyphaenidae

(Hentz, 1847)

Anyphaena celer Bryant 1931: 111; Dondale and Redner 1982: 175, mf, desc. (figs 320–324, 326); Jackman 1997: 160; Kaston 1972: 232, desc. (fig. 524); Kaston 1978: 223, desc. (fig. 57); Platnick 1974: 214, mf, desc. (figs 1, 9–10, 18); Rapp 1984: 7

####### Distribution.

East Texas; Galveston, Wichita

####### Habitat.

(grass: grass and shrub area)

####### Type.

Alabama and North Carolina

####### Etymology.

Latin, swift

####### Collection.

MSU

###### Anyphaena dixiana

Taxon classificationAnimaliaAraneaeAnyphaenidae

(Chamberlin & Woodbury, 1929)

Anyphaena dixiana Agnew et al. 1985: 8; Jackman 1997: 160; Platnick 1974: 221, mf, desc. (figs 4, 23–25)

####### Distribution.

Brewster, Erath, Hays, Kerr

####### Time of activity.

Male (December 16-January 26); female (January 27-February 24, April, December 16-January 26)

####### Habitat.

(landscape features: under rock); (soil/woodland: *Juniperus* managed plot, upland deciduous forest)

####### Method.

Flight intercept trap on ground [f]; flight intercept trap elevated [m]

####### Type.

Utah, St. George

####### Etymology.

New Latin, apart

####### Collection.

TAMU

###### Anyphaena fraterna

Taxon classificationAnimaliaAraneaeAnyphaenidae

(Banks, 1896)

Anyphaena fraterna Agnew et al. 1985: 8; Jackman 1997: 160; Kaston 1978: 224, desc.; Platnick 1974: 233, mf, desc. (figs 52, 56, 60, 77–78)

####### Distribution.

Central and north Texas; Brazos, Burleson/Lee, Erath, Kerr, Montgomery, Sabine, Travis, Wichita

####### Locality.

Lick Creek Park

####### Time of activity.

Male (March – May, May 22-June 4); female (March – May, June 23-July 2)

####### Habitat.

(soil/woodland: beech-magnolia forest, bottomland forest, upland deciduous forest, *Quercus buckleyi*, *Ulmus crassifolia*); (structures: house)

####### Method.

Flight intercept trap [f]; flight intercept trap elevated [m]; malaise trap [mf]; pitfall trap [m]; sweeping [f]

####### Type.

New York, Sea Cliff

####### Etymology.

Latin, brotherly

####### Collection.

DMNS, MSU, TAMU

###### Anyphaena lacka

Taxon classificationAnimaliaAraneaeAnyphaenidae

Platnick, 1974

Anyphaena lacka Jackman 1997: 160; Platnick 1974: 233, m, desc. (figs 54, 58, 62)

####### Distribution.

San Patricio

####### Locality.

Lake Corpus Christi State Park

####### Time of activity.

Male (June)

####### Type.

Texas (male, San Patricio Co., SW Mathis, Lake Corpus Christi State Park, June 28, 1962, J. A. Beatty, holotype, MCZ)

[female unknown]

####### Etymology.

arbitrary combination of letters

###### Anyphaena maculata

Taxon classificationAnimaliaAraneaeAnyphaenidae

(Banks, 1896)

Anyphaena maculata [Platnick 1974: 216, mf, desc. (figs 2, 11–12, 19)]

####### Distribution.

Brazos

####### Locality.

Lick Creek Park

####### Time of activity.

Female (December 2-January 17)

####### Method.

Malaise trap [f]

####### Type.

Washington D. C.

####### Etymology.

Latin, black spots on body

####### Collection.

TAMU

###### Anyphaena pectorosa

Taxon classificationAnimaliaAraneaeAnyphaenidae

L. Koch, 1866

Anyphaena pectorosa Bradley 2013: 74; Dondale and Redner 1982: 176; Jackman 1997: 160; Kaston 1978: 224, desc.; Platnick 1974: 230, mf, desc. (figs 51, 55, 59, 74–75); Woods and Harrel 1976: 43; Young and Edwards 1990: 14

####### Distribution.

North-central Texas; Brewster, Gonzalez, Jefferson, Polk, Travis

####### Locality.

Palmetto State Park

####### Time of activity.

Male (May – June); female (May)

####### Habitat.

(crops: rice); (soil/woodland: *Quercus virginiana*, *Ulmus crassifolia*)

####### Method.

Beating [m]; sweeping [m]

####### Type.

Maryland, Baltimore

####### Etymology.

Latin, breast

####### Collection.

MSU, TAMU

###### Anyphaena rita

Taxon classificationAnimaliaAraneaeAnyphaenidae

Platnick, 1974

Anyphaena rita Broussard and Horner 2006: 253; Richman et al. 2011a: 47 [Platnick 1974: 225, mf, desc. (figs 7, 28, 37, 44)]

####### Distribution.

Brewster, Presidio

####### Locality.

Chihuahuan desert, Dalquest Research Site

####### Time of activity.

Male (“November/December”)

####### Method.

pitfall trap [m]

####### Type.

Arizona, Santa Catalina Mountains, Bear Canyon

####### Etymology.

locality (The specific name is a noun in apposition derived from the Santa Rita Mountains, where the species is abundant, Platnick 1974).

####### Collection.

MSU

##### Genus *Hibana* Brescovit, 1991

###### Hibana arunda

Taxon classificationAnimaliaAraneaeAnyphaenidae

(Platnick, 1974)

Hibana arunda Brescovit 1991: 743 [T]; Jackman 1997: 160; Pfannenstiel 2008a: 204; Taylor and Pfannenstiel 2008: 997
Aysha arunda Platnick, 1974; Platnick 1974: 259, mf, desc. (figs 118–119, 139, 142)

####### Distribution.

Cameron, Falls, Hidalgo

####### Locality.

Bentsen-Rio Grande Valley State Park, Frontera Audubon, Resaca de la Palma State Park, Sabal Palm Audubon Sanctuary

####### Time of activity.

Male (March, May- October); female (February, April – November)

####### Habitat.

(crops: cotton, soybean); (grass: grass); (orchard: grapefruit, orange, tangerine); (soil-woodland: palm forest margin [resaca bank])

####### Method.

D-vac suction [m]

####### Type.

Texas (male, Hidalgo Co., Edinburg, May 1934, S. Mulaik, holotype, AMNH)

####### Etymology.

arbitrary combination of letters

####### Collection.

MSU, TAMU

###### Hibana cambridgei

Taxon classificationAnimaliaAraneaeAnyphaenidae

(Bryant, 1931)

Hibana cambridgei Brescovit 1991: 743 [T]; Jackman 1997: 160
Aysha cambridgei Bryant, 1931; Platnick 1974: 254, mf, desc. (figs 120–121, 138, 141)

####### Distribution.

North-central, central and west Texas; Bastrop, Brewster, Edwards, Hays, Henderson, Jeff Davis, Real, Sabine, Travis, Wichita

####### Locality.

Bastrop State Park

####### Time of activity.

Male (April – June); female (May – June)

####### Habitat.

(plants: roadside vegetation); (soil/woodland: beech-magnolia forest, *Juniperus* managed plot, roadside vegetation, trees, *Juniperus ashei*, *Quercus buckleyi*, *Quercus virginiana*, *Ulmus crassifolia*)

####### Method.

Beating [mf]; flight intercept trap elevated [f]; malaise trap [f]; sweeping [mf]

####### Type.

Mexico, Guanajuato

####### Etymology.

Person (arachnologist)

####### Collection.

MSU, TAMU

###### Hibana futilis

Taxon classificationAnimaliaAraneaeAnyphaenidae

(Banks, 1898)

Hibana futilis Armstrong and Richman 2007: 395; Brescovit 1991: 742 [S, T]; Brescovit 1993: 138; Calixto et al. 2013: 181; Jackman 1997: 161; Patt and Pfannenstiel 2008: 65; Patt and Pfannenstiel 2009: 14; Pfannenstiel 2008a: 204; Taylor and Pfannenstiel 2008: 997
Anyphaena decepta Banks, 1899; Banks 1899: 190, f, desc
Aysha decepta (Banks, 1899); Agnew et al. 1985: 8; Breene et al. 1993b: 647; Brown 1974: 231; Dean and Sterling 1990: 402; Jones 1936: 70; Li 1990: 137, 142, 144; Platnick 1974: 256 [T], mf, desc. (figs 112–113, 123–124); Rapp 1984: 7; Roewer 1955: 534; Vincent and Frankie 1985: 380; Vogel 1970b: 5

####### Distribution.

Eastern ½ Texas; Bexar, Brazos, Burleson, Cameron, Coryell, Dallas, Erath, Falls, Galveston, Hidalgo, Kenedy, Mason, Medina, Nacogdoches, Robertson, Sabine, Travis, Washington, Wichita, Zavala

####### Locality.

Adriance Pecan Orchard, Bentsen-Rio Grande Valley State Park, Frontera Audubon, Holmes Pecan Orchard, Kenedy Ranch, Laguna Atascosa National Wildlife Refuge, Lick Creek Park, Russell Farm, Storey Pecan Orchard, Texas A&M University Rangeland Area

####### Time of activity.

Male (January – December); female (January – December)

####### Habitat.

(crops: cotton, sugarcane); (grass: grass, grasses, grassy and shrub area); (littoral: salt marsh area); (nest/prey: mud dauber nest); (orchard: citrus, grapefruit, orange, pecan, sour orange); (plants: miscellaneous vegetation, *Amaranthus palmeri*); (soil/woodland: live oak, post oak savanna with pasture, sandy area, thorn thicket, trees/shrubs); (structures: house, indoors)

####### Method.

Beating [mf]; boll weevil pheromone trap [mf]; cardboard band [mf]; D-Vac suction [m]; flight intercept trap [mf]; fogging [mf]; irrigation tubing [mf]; malaise trap [m]; pitfall trap [mf]; suction trap [f]; sweeping [mf]

####### Type.

Mexico, Baja California

####### Etymology.

Latin, vain

####### Collection.

DMNS, MSU, TAMU

###### Hibana gracilis

Taxon classificationAnimaliaAraneaeAnyphaenidae

(Hentz, 1847)

Hibana gracilis Bradley 2013: 75; Brescovit 1991: 742 [T]; Calixto et al. 2013: 181; Jackman 1997: 106, desc., 161 (photo 30a)
Aysha gracilis (Hentz, 1847); Agnew et al. 1985: 4, 10; Bonnet 1955: 837; Breene 1988: 15, 17, 23–26, 35, 41; Breene et al. 1988: 180–181; Breene et al. 1989: 162; Breene et al. 1993c: 9, 47, 74, mf (figs 65A-C); Bumroongsook et al. 1992: 17; Dean and Eger 1986: 142; Dean and Sterling 1987: 6; Dean and Sterling 1990: 402, 405; Dean et al. 1982: 255; Dean et al. 1987: 268; Dean et al. 1988: 287; Dondale and Redner 1982: 167, mf, desc. (figs 307–312); Glick 1957: 5; Jones 1936: 70; Kagan 1942: 55; Kagan 1943: 258; Kaston 1972: 231, desc. (fig. 522); Kaston 1978: 222, desc. (fig. 568); Liao et al. 1984: 410; McDaniel et al. 1981: 104; Nuessly and Sterling 1984: 96; Pamanes-Guerrero 1975: 37, 41, 81; Platnick 1974: 252, mf, desc. (figs 116–117, 140, 143); Vogel 1970b: 5; Young and Edwards 1990: 14

####### Distribution.

Eastern ½ Texas; Angelina, Archer, Bexar, Brazos, Burleson, Cameron, Colorado, Comanche, Coryell, Dallas, Delta, Denton, Dickens, Duval, Erath, Fannin, Frio, Hidalgo, Houston, Karnes, Kenedy, McLennan, Robertson, Sabine, Stephens, Travis, Walker, Webb, Wichita, Young

####### Locality.

Adriance Pecan Orchard, Angelina National Forest, Attwater Prairie Chicken National Wildlife Refuge, Bentsen-Rio Grande Valley State Park, Bill Haney Pecan Orchard, Ellis Prison Unit, Hoblitzelle Farms, Holmes Pecan Orchard, Sam Houston State Park

####### Time of activity.

Male (January, March – September); female (March – December)

####### Habitat.

(crops: cotton, peanuts, soybean); (grass: grass, grassland); (landscape features: under rock); (nest/prey: mud dauber nest); (orchard: pecan); (plants: bluebonnets, croton, herbs, miscellaneous vegetation); (soil/woodland: beech-magnolia forest, post oak savanna with pasture, *Juniperus ashei*, *Quercus buckleyi*, *Quercus virginiana*, *Ulmus crassifolia*); (structures: indoors)

####### Method.

Beating [mf]; boll weevil pheromone trap [m]; cardboard band [mf]; D-Vac suction [m]; fogging [mf]; malaise trap [mf]; pitfall trap [mf]; suction trap [mf]; sweeping [mf]

####### Type.

North Carolina and Alabama

####### Etymology.

Latin, slender

####### Collection.

DMNS, MSU, TAMU

###### Hibana incursa

Taxon classificationAnimaliaAraneaeAnyphaenidae

(Chamberlin, 1919)

Hibana incursa Brescovit 1991: 742 [T]; Jackman 1997: 161
Aysha incursa (Chamberlin, 1919); Platnick 1974: 257, mf, desc. (figs 114–115, 126–127)

####### Distribution.

Brewster, El Paso, Presidio

####### Locality.

Big Bend National Park, Big Bend Ranch State Park

####### Time of activity.

Male (March – April, July); female (May – June)

####### Habitat.

(orchard: pecan); (soil/woodland: cottonwood)

####### Method.

Beating [mf]; malaise trap [mf]

####### Type.

California, Claremont

####### Etymology.

Latin, attack

####### Collection.

NMSU, TAMU

###### Hibana velox

Taxon classificationAnimaliaAraneaeAnyphaenidae

(Becker, 1879)

Hibana velox Brescovit 1991: 743 [T]; Jackman 1997: 161
Aysha velox (Becker, 1879); Kaston 1978: 222; Platnick 1974: 258, mf, desc. (figs 110–111, 122, 125)

####### Distribution.

Southeast Texas; Angelina, Brazos, Colorado, Fort Bend, Harris, Jefferson

####### Locality.

Attwater Prairie Chicken National Wildlife Refuge

####### Time of activity.

Male (June, August); female (June, August)

####### Method.

sweeping [m]

####### Type.

Mississippi, Pascagoula

####### Etymology.

Latin, speedy

####### Collection.

MSU, TAMU

##### Genus *Lupettiana* Brescovit, 1997

###### Lupettiana mordax

Taxon classificationAnimaliaAraneaeAnyphaenidae

(O. P.-Cambridge, 1896)

Lupettiana mordax Bradley 2013: 75; Brescovit 1997: 68, mf, desc. (figs 157–162 [T]); Calixto et al. 2013: 181
Teudis mordax (O. P.-Cambridge, 1896); Breene et al. 1993c: 9, 47, 75, mf (figs 67A–C); Dean and Eger 1986: 142; Dean and Sterling 1990: 402; Dean et al. 1982: 255; Jackman 1997: 161; Kaston 1978: 224, desc. (fig. 572); Liao et al. 1984: 410; Platnick 1974: 263, mf, desc. (figs 131–133); Vincent and Frankie 1985: 380; Young and Edwards 1990: 14
Anyphaena sp. prob. celer (Hentz, 1847); Dean et al. 1982: 255 [misidentified]
Anyphaena celer (Hentz, 1847); Young and Edwards 1990: 14 [misidentified]

####### Distribution.

East Texas; Bastrop, Brazos, Burleson, Goliad, Robertson, Sabine, Travis, Walker

####### Locality.

Bastrop State Park, Ellis Prison Unit, Holmes Pecan Orchard, Lick Creek Park, Somerville Lake, Stetz Pecan Orchard, Texas A&M University Rangeland Area

####### Time of activity.

Male (March – August); female (April – August)

####### Habitat.

(crops: cotton); (grass: tall grass prairie); (orchard: pecan); (plants: bluebonnets, Indian paintbrush, miscellaneous vegetation); (soil/woodland: beech-magnolia forest, live oak, trees, *Juniperus ashei*, *Quercus buckleyi*, *Quercus virginiana*, *Ulmus crassifolia*)

####### Method.

Beating [mf]; cardboard band [mf]; malaise trap [f]; pitfall trap [m]; suction trap [m]; sweeping [mf]

####### Type.

Mexico, Guerrero, Omiltemi

####### Etymology.

Latin, biting

####### Collection.

TAMU

##### Genus *Pippuhana* Brescovit, 1997

###### Pippuhana calcar

Taxon classificationAnimaliaAraneaeAnyphaenidae

(Bryant, 1931)

Pippuhana calcar Bradley 2013: 76; Brescovit 1997: 113 [T], mf, desc. (figs 305–308); Richman and Ubick 2005: 67
Teudis calcar Bryant, 1931; Jackman 1997: 161; Platnick 1974: 265 [S], mf, desc. (figs 128–130)
Anyphaena schwarzi Gertsch, 1933; Gertsch 1933c: 10, f, desc. (fig. 12); Roewer 1955: 529

####### Distribution.

South Texas; Brazos, Cameron, Hidalgo, San Patricio

####### Time of activity.

Male (April); female (January, March)

####### Type.

Florida, Dunedin

####### Etymology.

Latin, spur on patella

####### Collection.

TAMU

##### Genus *Wulfila* O. P.-Cambridge, 1895

###### Wulfila albens

Taxon classificationAnimaliaAraneaeAnyphaenidae

(Hentz, 1847)

Wulfila albens Platnick 2000 [spelling]
Wulfila alba (Hentz, 1847); Jackman 1997: 161; Kaston 1978: 223; Platnick 1974: 245, mf, desc. (figs 83–84, 90, 100)

####### Distribution.

North-central Texas; Brazos, Gonzales, Sabine, Walker

####### Locality.

Lick Creek Park, Palmetto State Park

####### Time of activity.

Male (April – May); female (April 29-May 3, May 22–June 4, June)

####### Habitat.

(soil/woodland: beech-magnolia forest, trees)

####### Method.

Beating [m]; beating/sweeping [m]; malaise trap [f]

####### Type.

Alabama

####### Etymology.

Latin, lack of dark markings

####### Collection.

TAMU

###### Wulfila bryantae

Taxon classificationAnimaliaAraneaeAnyphaenidae

Platnick, 1974

Wulfila bryantae Jackman 1997: 161; Platnick 1974: 249, mf, desc. (figs 92–93, 96, 102)

####### Distribution.

Cameron, Hidalgo, Jim Wells

####### Locality.

Frontera Audubon, Sabal Palm Audubon Sanctuary, Santa Ana National Wildlife Refuge

####### Time of activity.

Male (April – June); female (March 3-April 4, April – December)

####### Habitat.

(orchard: grapefruit, orange, organic citrus grove); (soil/woodland: forest)

####### Method.

Flight intercept trap [f]

####### Type.

Texas (male, Hidalgo Co., 5 miles E Edinburg, April 20, 1937, S. Mulaik, holotype, AMNH)

####### Etymology.

Person (The specific name is a patronym in honor of Miss Elizabeth Bryant, in recognition of her pioneering work on North American anyphaenids, Platnick 1974).

####### Collection.

TAMU

###### Wulfila saltabundus

Taxon classificationAnimaliaAraneaeAnyphaenidae

(Hentz, 1847)

Wulfila saltabundus Bradley 2013: 76; Breene et al. 1993c: 9, 47, 74, mf (figs 66A-C); Dondale and Redner 1982: 170 [spelling], mf, desc. (figs 313–319); Jackman 1997: 161
Wulfila saltabunda (Hentz, 1847); Dean et al. 1982: 255; Dean et al. 1988: 287; Kaston 1978: 223, desc. (fig. 570); Platnick 1974: 243, mf, desc. (figs 81–82, 89, 99); Rapp 1984: 7; Young and Edwards 1990: 14

####### Distribution.

East and north-central Texas; Brazos, Galveston, Houston, Walker

####### Locality.

Ellis Prison Unit, Sam Houston National Forest, Stubblefield Lake

####### Time of activity.

Male (April, July – August); female (April, June – July)

####### Habitat.

(crops: cotton); (grass: grassland); (structures: indoors)

####### Method.

pitfall trap [m]

####### Type.

Alabama

####### Etymology.

Latin, continuous in forest

####### Collection.

TAMU

###### Wulfila tantillus

Taxon classificationAnimaliaAraneaeAnyphaenidae

Chickering, 1940

Wulfila tantillus Calixto et al. 2013: 181; Jackman 1997: 161; Platnick 1993: 597 [spelling]; Reddell and Cokendolpher 2004: 76
Wulfila tantilla Chickering, 1940; Platnick 1974: 246, mf, desc. (figs 85–86, 91, 101)

####### Distribution.

Central and south Texas; Bexar, Cameron, Hidalgo, Montague, Robertson, Travis, Webb, Wichita

####### Locality.

Bentsen-Rio Grande Valley State Park, Holmes Pecan Orchard

####### Caves.

**Bexar** (Kick Start Cave)

####### Time of activity.

Male (April, July); female (May, August, October)

####### Habitat.

(landscape features: cave); (orchard: pecan); (soil/woodland: *Quercus virginiana*, *Ulmus crassifolia*)

####### Method.

Beating [m]; cardboard band [f]; sweeping [m]

####### Type.

Panama, El Valle

####### Etymology.

Latin, so little

####### Collection.

MSU, TAMU, TMM

#### Family Araneidae Clerck, 1775

**Note.** species incorrectly reported from Texas

*Eustala rosae* Chamberlin & Ivie, 1935; Kaston 1972: 149; Kaston 1978: 143 [not in Texas]

*Hypsosinga pygmaea* (Sundevall, 1831); Young and Edwards 1990: 15 [not in Texas]

*Mastophora bisaccata* (Emerton, 1884); Brown 1974: 232; Jackman 1997: 161 [not in Texas] [probably misidentified]

*Neoscona moreli* (Vinson, 1863) [not in Texas]

*Neoscona neotheis* (Petrunkevitch, 1911); Gertsch and Mulaik 1936b: 21 (Nueces Co.); Vogel 1970b: 4 [not in U.S., probably *oaxacensis*]

*Aranea neotheis* Petrunkevitch, 1911; Roewer 1942: 848

**nomen dubium**

*Neoscona benjamina* (Walckenaer, 1841); Brown 1974: 232; Reddell 1965: 170; Reddell and Finch 1963: 54; Vogel 1970b: 4

*Epeira benjamina* Walckenaer, 1837; McCook 1889: 116; McCook 1893: 147

##### Genus *Acacesia* Simon, 1895

###### Acacesia hamata

Taxon classificationAnimaliaAraneaeAraneidae

(Hentz, 1847)

Acacesia hamata Agnew et al. 1985: 6; Bradley 2013: 77; Breene et al. 1993b: 647; Breene et al. 1993c: 10, 47, 104, mf (figs 157A-C); Brown 1974: 231; Calixto et al. 2013: 181; Dean et al. 1982: 254; Glueck 1994: 69, mf, desc. (figs 1, 4–8); Jackman 1997: 72, desc., 161 (photo 21a); Kaston 1953: 183, desc. (fig. 453); Kaston 1972: 151, desc. (fig. 339); Kaston 1978: 144, desc. (fig. 362); Levi 1976: 375 [S], mf, desc. (figs 74–87); Rice 1986: 124; Roth 1982: 11–1; Roth 1985: B-6–5, B-6–11; Roth 1994: 70, 74; Young and Edwards 1990: 14
Epeira foliata Hentz, 1847; McCook 1893: 154
Acacesia foliata (Hentz, 1847); Petrunkevitch 1911: 274

####### Distribution.

Southern ½ Texas; Brazos, Brewster, Cameron, Erath, Hidalgo, Kenedy, Nacogdoches, Robertson, San Patricio, Shelby, Travis (imm.), Walker

####### Locality.

Ellis Prison Unit, Holmes Pecan Orchard, Kenedy Ranch, Laguna Atascosa National Wildlife Refuge, Lake Corpus Christi State Park, Lick Creek Park, Sabal Palm Audubon Sanctuary, Santa Ana National Wildlife Refuge

####### Time of activity.

Male (January, March – May, July – August, October); female (April, June, August – October)

####### Habitat.

(crops: cotton, sugarcane); (grass: grass, meadow); (nest/prey: mud dauber nest [f]); (orchard: pecan); (plants: vegetation); (soil/woodland: palm forest margin [resaca bank], trees, woods, *Juniperus ashei*, *Quercus buckleyi*, *Quercus virginiana*, *Ulmus crassifolia*)

####### Method.

Beating [m]; cardboard band [imm.]; D-Vac suction [m]; malaise trap [m]; sweeping [mf]

####### Type.

Alabama

####### Etymology.

Latin, hooked

####### Collection.

MSU, TAMU

##### Genus *Acanthepeira* Marx, 1883

###### Acanthepeira cherokee

Taxon classificationAnimaliaAraneaeAraneidae

Levi, 1976

Acanthepeira cherokee Breene et al. 1993c: 10, 47, 103, mf (figs 153A-B); Dean et al. 1982: 254; Jackman 1997: 161; Levi 1976: 366, mf, desc. (figs 24, 29–35, 42–43); Young and Edwards 1990: 14

####### Distribution.

Southeast Texas; Brazos, Colorado, Jefferson, Wichita

####### Locality.

Attwater Prairie Chicken National Wildlife Refuge, Lick Creek Park

####### Time of activity.

Male (March, November); female (April – May, September, November)

####### Habitat.

(crops: cotton); (littoral: sedge meadow); (soil/woodland: post oak savanna)

####### Method.

Beating [f]; sweeping [mf]

####### Type.

North Carolina, Mud Creek

####### Etymology.

Indian tribe (The name is a noun in apposition, after the southeastern Indian tribe, Levi 1976).

####### Collection.

MSU, TAMU

###### Acanthepeira marion

Taxon classificationAnimaliaAraneaeAraneidae

Levi, 1976

Acanthepeira marion [Levi 1976: 368, mf, desc. (figs 25, 36–41, 44)]

####### Distribution.

Fannin

####### Type.

Florida, Marion Co.

####### Etymology.

locality (The name is a noun in apposition, after the type locality, Levi 1976).

####### Collection.

MSU

###### Acanthepeira stellata

Taxon classificationAnimaliaAraneaeAraneidae

(Walckenaer, 1805)

Acanthepeira stellata Agnew et al. 1982: 631; Agnew et al. 1985: 3; Breene et al. 1993c: 10, 47, 103, mf (figs 152A-C); Brown 1974: 231; Calixto et al. 2013: 181; Cokendolpher et al. 2008: 8, 13 (fig. 2); Dean and Eger 1986: 141; Dean and Sterling 1985: 116; Dean and Sterling 1987: 6; Dean and Sterling 1990: 402, 404; Dean and Sterling 1992: 3–4; Dean et al. 1982: 254; Dean et al. 1987: 268; Dean et al. 1988: 285–286; Dondale et al. 2003: 309, mf, desc. (figs 726–732); Jackman 1997: 72, desc., 161 (photo 21b); Kagan 1942: 34; Kagan 1943: 258; Levi 1976: 364, mf, desc. (figs 12–23); McDaniel et al. 1981: 104; Nuessly and Sterling 1984: 97; Nyffeler and Sterling 1994: 1295, 1298; Nyffeler et al. 1987a: 356; Nyffeler et al. 1987b: 1119; Nyffeler et al. 1987c: 368; Nyffeler et al. 1988a: 55; Nyffeler et al. 1989: 374, 377; Nyffeler et al. 1992c: 2; Pamanes-Guerrero 1975: 16, 34, 37, 41, 59, 63, 78, 81; Rapp 1984: 4; Roberts 2001: 48; Rogers and Horner 1977: 523; Sterling et al. 1979: 979; Vogel 1970b: 3; Woods and Harrel 1976: 43; Young and Edwards 1990: 14
Marxia stellata (Hentz, 1805); Jones 1936: 70
Acanthepeira stellata (Marx); Kaston 1972: 148, desc. (fig. 333)

####### Distribution.

Eastern 2/3 Texas; Archer, Bastrop, Bee, Brazoria, Brazos, Burleson, Cameron, Carson, Clay, Collin, Colorado, Dallas, Delta, Erath, Fannin, Fayette, Fort Bend, Galveston, Goliad, Grayson, Grimes, Houston, Hunt, Jefferson, Jim Wells, Kenedy, Limestone, McLennan, Nacogdoches, Nueces, Potter, Robertson, Sabine, Travis, Victoria, Walker, Wharton, Wichita, Willacy, Williamson, Young

####### Locality.

Attwater Prairie Chicken National Wildlife Refuge, Ellis Prison Unit, Galveston Island State Park, Holmes Pecan Orchard, Pantex Plant, Ramsey Prison Farm, Sam Houston State Park, Texas A&M University Rangeland Area, Wildcat Bluff Nature Center

####### Time of activity.

Male (March – September, November – December); female (February – December)

####### Habitat.

(crops: cotton, guar, peanuts, rice); (grass: grassland, grassy and shrub area, pasture, shrubs and tall grass); (littoral: playa, near playa, salt marsh area); (nest/prey: mud dauber nest [mf]); (orchard: pecan); (plants: bluebonnets, croton, Indian paintbrush, miscellaneous vegetation, *Coreopsis* sp., *Monarda citriodora*); (soil/woodland: beech-magnolia forest, pine); (structures: around house)

####### Method.

cardboard band [m]; malaise trap [m]; pitfall trap [m]; suction trap [m]; sweeping [mf]

####### Type.

Carolina (of 1805)

####### Etymology.

Latin, starred

####### Collection.

DMNS, MSU, TAMU, TTU

##### Genus *Allocyclosa* Levi, 1999

###### Allocyclosa bifurca

Taxon classificationAnimaliaAraneaeAraneidae

(McCook, 1887)

Allocyclosa bifurca Bradley 2013: 78; Levi 1999: 304 [T], mf, desc. (figs 3–22)
Cyclosa bifurca (McCook, 1887); Jackman 1997: 161; Levi 1977a: 86, mf, desc. (figs 78–89)

####### Distribution.

Aransas, Cameron, Hidalgo, Kenedy, San Patricio

####### Locality.

Goose Island State Park, Lake Corpus Christi Dam

####### Time of activity.

Female (May – June, November – December)

####### Habitat.

(nest/prey: mud dauber nest [f] of *Chalybion californicum*); (orchard: grapefruit)

####### Type.

Florida, Merrit’s Island on Indian River, Fairyland

####### Etymology.

Latin, forked abdomen

####### Collection.

TAMU

##### Genus *Araneus* Clerck, 1757

###### Araneus bicentenarius

Taxon classificationAnimaliaAraneaeAraneidae

(McCook, 1888)

Araneus bicentenarius Dondale et al. 2003: 209, mf, desc. (figs 428–435); Jackman 1997: 73, 161 (photo 21c); Levi 1971a: 143 [S], mf, desc. (figs 15–26); Taber and Fleenor 2003: 231; Taber and Fleenor 2005: 276 (figs 12–14)
Aranea kisatchia Archer, 1951; Archer 1951a: 27, f, desc. (fig. 69)
Araneus kisatcheus Archer, 1951; Vogel 1970b: 3

####### Distribution.

Central and southeast Texas; Brazos, Freestone, Gonzalez, Hays, Orange, Walker

####### Locality.

Lick Creek Park, Palmetto State Park

####### Time of activity.

Male (May); female (May – August)

####### Habitat.

(littoral: wetlands); (soil/woodland: oak)

####### Method.

Beating/sweeping [f]

####### Type.

Ohio, northwestern and Allegheny Mountains

####### Etymology.

bicentennial of Philadelphia

####### Collection.

DMNS, TAMU

###### Araneus bonsallae

Taxon classificationAnimaliaAraneaeAraneidae

(McCook, 1894)

Araneus bonsallae Jackman 1997: 161; Levi 1973: 524, mf, desc. (figs 265–294, 453–454)

####### Distribution.

North-central Texas; Dallas, Wichita

####### Time of activity.

Female (May)

####### Habitat.

(plants: vegetation); (soil/woodland: tree)

####### Method.

sweeping

####### Type.

California

####### Etymology.

Person (Miss Elizabeth F. Bonsall, who made the original drawings for nearly all the plates contained in the atlas by McCook)

####### Collection.

MCZ, MSU, TAMU

###### Araneus cavaticus

Taxon classificationAnimaliaAraneaeAraneidae

(Keyserling, 1881)

Araneus cavaticus Bradley 2013: 79; Dondale et al. 2003: 244, mf, desc. (figs 539–545); Jackman 1997: 73, 161; Kaston 1978: 157, desc. (fig. 392); Levi 1971a: 170, mf, desc. (figs 187–194)

####### Distribution.

East Texas; Harris

####### Type.

Kentucky, cave in Carter Co.

####### Etymology.

Latin, cave

####### Note.

Hoffman (1982: 93) states that this species does not occur in Texas because of the habitat it has been associated with and the distance from other collecting sites.

###### Araneus cingulatus

Taxon classificationAnimaliaAraneaeAraneidae

(Walckenaer, 1841)

Araneus cingulatus Dondale et al. 2003: 256, mf, desc. (figs 575–582); Jackman 1997: 161; Kaston 1978: 160, desc.; Levi 1973: 526, mf, desc. (figs 301–313, 455–462)

####### Distribution.

North-central Texas; Travis, Walker

####### Locality.

Ellis Prison Unit

####### Time of activity.

Male (April – July); female (May – July, September)

####### Habitat.

(soil/woodland: *Quercus virginiana*, *Ulmus crassifolia*)

####### Method.

Beating [mf]; sweeping [mf]

####### Type.

Georgia

####### Etymology.

Latin, girdled

####### Collection.

TAMU

###### Araneus cochise

Taxon classificationAnimaliaAraneaeAraneidae

Levi, 1973

Araneus cochise Agnew et al. 1985: 6; Dean et al. 1989: 126, m, desc. (figs 1–2); Jackman 1997: 161; Levi 1991: 278, mf, desc. (figs 445–448)

####### Distribution.

Erath, Kerr, Travis

####### Time of activity.

Male (March – May); female (March – June)

####### Habitat.

(soil/woodland: juniper, *Juniperus ashei*, *Quercus virginiana*)

####### Method.

Beating [mf]; sweeping [mf]

####### Eggs/spiderlings.

Erath [7 spiderlings in eggsac] [TAMU]

####### Type.

Arizona, Cochise Co., Chiricahua Mountains, Southwestern Research Station

####### Etymology.

locality (The name is a noun in apposition after the type locality, Levi, 1973).

####### Collection.

MCZ, MSU, TAMU

###### Araneus detrimentosus

Taxon classificationAnimaliaAraneaeAraneidae

(O. P.-Cambridge, 1889)

Araneus detrimentosus Agnew et al. 1985: 7; Bradley 2013: 81; Jackman 1997: 73, desc., 161 (photo 21d); Levi 1973: 538 [T], mf, desc. (figs 398–414); Levi 1991: 269
Cambridgepeira detrimentosa (O. P.-Cambridge, 1889); Archer 1951b: 2 (fig. 9)

####### Distribution.

Eastern ½ Texas; Atascosa, Bastrop, Bell, Brazos, Burleson, Cameron, Duval, Erath, Gillespie, Goliad, Hidalgo, Lee, Leon, Liberty, Navarro, Starr, Travis, Williamson

####### Locality.

Bentsen-Rio Grande Valley State Park, Falcon Lake State Park, Lake Somerville State Park [Nails Creek Unit], Riley Estate

####### Time of activity.

Male (April – June, August – September); female (April – October)

####### Habitat.

(nest/prey: mud dauber nest [f]); (orchard: grapefruit, Valley lemon); (plants: Indian paintbrush); (soil/woodland: juniper, rock elm, shrubs, trees, *Juniperus* sp., *Quercus virginiana*, *Ulmus* sp.); (web: web in live oak, web on mesquite [*Prosopis juliflora*])

####### Method.

Beating [mf]; suction trap [m]; sweeping [m]

####### Type.

Guatemala

####### Etymology.

Latin, prone to detritus

####### Collection.

DMNS, MCZ, TAMU

###### Araneus gemma

Taxon classificationAnimaliaAraneaeAraneidae

(McCook, 1888)

Araneus gemma Kaston 1972: 163, desc. (fig. 362); Reddell 1965: 170; Vogel 1970b: 3 [Levi 1971a: 172, mf, desc. (figs 203–214)]

####### Distribution.

Bastrop, Brewster

####### Caves.

**Brewster** (O.T.L. Cave)

####### Time of activity.

Female (May)

####### Habitat.

(landscape features: cave)

####### Type.

California

####### Etymology.

Latin, bud or gem

####### Collection.

DMNS, TMM

###### Araneus guttulatus

Taxon classificationAnimaliaAraneaeAraneidae

(Walckenaer, 1841)

Araneus guttulatus [Levi 1973: 530, mf, desc. (figs 3, 332–361, 470–474)]

####### Distribution.

Shelby

####### Time of activity.

Male (August)

####### Habitat.

(plants: vegetation)

####### Method.

sweeping [m]

####### Type.

Georgia

####### Etymology.

Latin, for speckled

####### Collection.

TAMU

###### Araneus illaudatus

Taxon classificationAnimaliaAraneaeAraneidae

(Gertsch & Mulaik, 1936)

Araneus illaudatus Jackman 1997: 73, 161; Levi 1971a: 176, m, desc. (figs 233–240); Levi 1975b: 268 [S], m (figs 3–4)
Aranea illaudata Gertsch & Mulaik, 1936; Gertsch and Mulaik 1936b: 19, m, desc. (figs 36–37); Roewer 1942: 861
Araneus iliaudatus (Gertsch & Mulaik, 1936); Vogel 1970b: 3
Araneus pima Levi, 1971; Levi 1971a: 176, mf, desc. (figs 218–232)

####### Distribution.

Brewster, Dallam, Galveston, Hidalgo, Jeff Davis, Kerr

####### Caves.

**Brewster** (O.T.L. Cave)

####### Time of activity.

Female (September – October)

####### Habitat.

(landscape features: cave); (structures: barns, under house eave); (soil/woodland: trees)

####### Type.

Texas (male, Hidalgo Co., Edinburg, September-December 1933, S. Mulaik, holotype, AMNH)

####### Etymology.

Greek, referring to a rope or band

####### Collection.

DMNS, TAMU, TMM

####### Note.

Hoffman (1982: 93) stated that this species does not occur in Texas because of the habitat it has been associated with and the distance from other collecting sites.

###### Araneus juniperi

Taxon classificationAnimaliaAraneaeAraneidae

(Emerton, 1884)

Araneus juniperi Dondale et al. 2003: 254, mf, desc. (figs 568–574); Jackman 1997: 161; Kaston 1978: 158, desc. (fig. 397); Levi 1973: 522 [S], mf, desc. (figs 248–264, 447–452)
Conepeira llano Archer, 1951; Archer 1951b: 24, mf, desc. (figs 52, 55); Vogel 1967: 24; Vogel 1970b: 3

####### Distribution.

Brazos, Comanche, Llano, Robertson

####### Locality.

Bill Haney Pecan Orchard, Holmes Pecan Orchard

####### Time of activity.

Male (June – September); female (September)

####### Method.

Fogging [mf]

####### Type.

Maine, Portland, Peaks Island

####### Etymology.

collected in junipers

####### Collection.

TAMU

###### Araneus kerr

Taxon classificationAnimaliaAraneaeAraneidae

Levi, 1981

Araneus kerr Jackman 1997: 161; Levi 1981b: 254, f, desc. (figs 1–4)

####### Distribution.

Kerr

####### Locality.

Raven Ranch

####### Time of activity.

Female (June)

####### Type.

Texas (female, Kerr Co., Raven Ranch, June 1941, J. Stillwagon, holotype, AMNH)

[male unknown]

####### Etymology.

locality (The specific name is a noun in apposition taken from the type locality, Levi 1981b).

###### Araneus marmoreus

Taxon classificationAnimaliaAraneaeAraneidae

Clerck, 1757

Araneus marmoreus Brown 1974: 231; Dondale et al. 2003: 221, mf, desc. (figs 466–474); Jackman 1997: 73–74, 161, desc.; Kaston 1972: 165, desc. (fig. 366); Kaston 1978: 158, desc. (fig. 394); Levi 1971a: 156, mf, desc. (figs 1–6, 100–105, 107–113, 183); Taber and Fleenor 2005: 277 (fig. 12–5)

####### Distribution.

Southeast and east Texas; Brazos, Gonzales, Nacogdoches, Rusk, San Jacinto

####### Locality.

Big Creek Scenic Area, Lick Creek Park, Palmetto State Park

####### Time of activity.

Female (September, November)

####### Habitat.

(littoral: sedge meadow, wetlands); (nest/prey: mud dauber nest [imm.])

####### Type.

Sweden

####### Etymology.

Greek, marbled

####### Collection.

TAMU

###### Araneus miniatus

Taxon classificationAnimaliaAraneaeAraneidae

(Walckenaer, 1841)

Araneus miniatus Bradley 2013: 82; Hoffmaster 1985: 627; Jackman 1997: 161; Kaston 1978: 158, desc. (fig. 396); Levi 1973: 506 [S], mf, desc. (figs 158–171); Petrunkevitch 1911: 303
Epeira miniata Walckenaer, 1837; McCook 1893: 177
Larinia nigrofoliata Keyserling, 1884; Petrunkevitch 1911: 354; Roewer 1942: 772

####### Distribution.

North-central Texas; Brazos, Cameron, Denton, Fannin, Houston, Hunt, Morris, Polk, Sabine, San Patricio, Travis, Walker

####### Locality.

Lick Creek Park, Resaca de la Palma State Park, Welder Wildlife Refuge

####### Time of activity.

Male (February – April, June – July); female (March – May, July – August, November)

####### Habitat.

(grass: pasture); (plants: vegetation); (soil/woodland: trees, *Juniperus ashei*, *Quercus buckleyi*, *Quercus virginiana*, *Ulmus crassifolia*)

####### Method.

Beating [mf]; beating/sweeping [m]; sweeping [mf]

####### Type.

Georgia

####### Etymology.

Latin, colored with vermillion

####### Collection.

MCZ, MSU, TAMU

###### Araneus nashoba

Taxon classificationAnimaliaAraneaeAraneidae

Levi, 1973

Araneus nashoba Agnew et al. 1985: 7; Jackman 1997: 161; Levi 1973: 534, mf, desc. (figs 380–397)

####### Distribution.

Erath, Fayette, Kimble, Travis

####### Time of activity.

Male (April – June); female (April – July)

####### Habitat.

(soil/woodland: *Quercus buckleyi*, *Quercus virginiana*)

####### Method.

Beating [mf]; sweeping [mf]

####### Type.

Massachusetts, Pepperell

####### Etymology.

locality (The specific name is a noun in apposition, after the Nashoba region of Massachusetts, Levi 1973).

####### Collection.

TAMU

###### Araneus nordmanni

Taxon classificationAnimaliaAraneaeAraneidae

(Thorell, 1870)

Araneus nordmanni Dondale et al. 2003: 219 (figs 458–465); Jackman 1997: 73, 161, desc.; Levi 1971a: 150 [S], mf, desc. (figs 61–94, 96–99)
Epeira angulata (Clerck, 1757); McCook 1893: 186

####### Distribution.

South Texas; Bastrop

####### Time of activity.

Female (June)

####### Type.

Sweden, Uppland

####### Etymology.

Person (arachnologist)

####### Collection.

MSU

####### Note.

Hoffman (1982: 93) stated that this species does not occur in Texas because of the habitat it has been associated with and the distance from other collecting sites.

###### Araneus pegnia

Taxon classificationAnimaliaAraneaeAraneidae

(Walckenaer, 1841)

Araneus pegnia Agnew et al. 1985: 7; Brown 1974: 231; Dondale et al. 2003: 213, mf, desc. (figs 444–450); Jackman 1997: 161; Levi 1973: 546 [S], mf, desc. (figs 426–438); Vogel 1970b: 3
Neosconella pegnia (Walckenaer, 1841); Knutson et al. 2010: 515
Araneus globosus (Keyserling, 1865); Jones 1936: 70

####### Distribution.

Eastern ½ Texas; Brazos, Cameron, Comanche, Dallas, Erath, Hidalgo, Howard, Menard, Nacogdoches, Sutton, Travis, Wichita, Williamson

####### Locality.

Bentsen-Rio Grande Valley State Park, Resaca de la Palma State Park, Riley Estate, Sabal Palm Audubon Sanctuary, Santa Ana National Wildlife Refuge

####### Time of activity.

Male (April – June, August – November); female (May – November)

####### Habitat.

(nest/prey: mud dauber nest [mf]); (orchard: grapefruit, orange, tangerine); (plants: goldenrod); (soil/woodland: saltcedar, trees, trees/shrubs, *Juniperus ashei*, *Quercus buckleyi*); (web: orbweb)

####### Method.

Beating [mf]; suction trap [m]; sweeping [m]

####### Type.

Georgia

####### Etymology.

undetermined

####### Collection.

DMNS, MSU, TAMU

###### Araneus pratensis

Taxon classificationAnimaliaAraneaeAraneidae

(Emerton, 1884)

Araneus pratensis Dean and Eger 1986: 141; Dondale et al. 2003: 237, mf, desc. (figs 517–523); Jackman 1997: 73, desc., 161 (photo 21f); Kaston 1978: 160, desc. (fig. 399); Levi 1973: 492 [T], mf, desc. (figs 2, 21–31); Rapp 1984: 4
Neoscona pratensis Emerton, 1884; Woods and Harrel 1976: 43; Young and Edwards 1990: 15

####### Distribution.

Southeast, central and east Texas; Bexar, Brazos, Fayette, Galveston, Jefferson, Kerr, Lavaca, Leon, Refugio, San Patricio, Victoria

####### Time of activity.

Male (April – May, August, October); female (April – May, August, November)

####### Habitat.

(crops: rice); (plants: bluebonnets, Indian paintbrush, miscellaneous vegetation)

####### Method.

sweeping [mf]

####### Type.

Connecticut, New Haven

####### Etymology.

Latin, pertaining to a meadow

####### Collection.

MCZ, TAMU

###### Araneus texanus

Taxon classificationAnimaliaAraneaeAraneidae

(Archer, 1951)

Araneus texanus Dean and Eger 1986: 141; Jackman 1997: 161; Levi 1973: 534 [T], mf, desc. (figs 362–374)
Conepeira texana Archer, 1951; Archer 1951b: 20, mf, desc. (figs 48, 69); Vogel 1967: 25; Vogel 1970b: 3

####### Distribution.

Brazos, Edwards, Freestone, Gillespie, Limestone

####### Locality.

Riley Estate

####### Time of activity.

Male (April); female (May – June)

####### Habitat.

(nest/prey: mud dauber nest [f]); (plants: bluebonnets, Indian paintbrush); (soil/woodland: trees)

####### Method.

Beating [f]; sweeping [m]

####### Type.

Texas (male, Limestone Co., Mexia, M. Kagan, holotype, AMNH)

####### Etymology.

locality (state)

####### Collection.

TAMU

###### Araneus thaddeus

Taxon classificationAnimaliaAraneaeAraneidae

(Hentz, 1847)

 Dondale et al. 2003 : 211, mf, desc. (figs 436–443) [ Levi 1973: 543, mf, desc. (figs 415–425)]

####### Distribution.

close to Rio Grande Valley, South Texas

####### Type.

Alabama

####### Etymology.

one of twelve apostles

##### Genus *Araniella* Chamberlin & Ivie, 1942

###### Araniella displicata

Taxon classificationAnimaliaAraneaeAraneidae

(Hentz, 1847)

Araniella displicata Agnew et al. 1985: 7; Breene 1988: 35; Breene et al. 1989: 162; Breene et al. 1993a: 169; Breene et al. 1993b: 647; Breene et al. 1993c: 10, 47, 107, mf (figs 166A-C); Dean et al. 1987: 268; Jackman 1997: 75, desc., 161; Rapp 1984: 4; Woods and Harrel 1976: 43; Young and Edwards 1990: 14 [Levi 1974: 294 [S, T], mf, desc. (figs 1–21)]
Epeira cucurbitina (Clerck, 1757); McCook 1893: 149
Epeira displicata Hentz, 1847; Marx 1890: 544

####### Distribution.

Burleson, Cameron, Erath, Galveston, Hidalgo, Jefferson, Travis

####### Locality.

Galveston Island State Park

####### Time of activity.

Male (March – April); female (February – May)

####### Habitat.

(crops: cotton, rice, sugarcane); (grass: grassy and shrub area); (orchard: citrus); (soil/woodland: *Quercus buckleyi*, *Quercus virginiana*, *Ulmus crassifolia*)

####### Method.

Beating [f]; suction trap [imm.]; sweeping [mf]

####### Type.

Alabama

####### Etymology.

Latin, scattered

####### Collection.

TAMU

##### Genus *Argiope* Audouin, 1826

###### Argiope argentata

Taxon classificationAnimaliaAraneaeAraneidae

(Fabricius, 1775)

Argiope argentata Bradley 2013: 86; Jackman 1997: 161; Levi 1968: 345, mf, desc. (figs 42, 73, 112–136); Levi 2004: 58; McCook 1893: 220; Marx 1890: 541; Vogel 1970b: 4

####### Distribution.

Southern 1/4 Texas; Cameron, Nueces, Zapata

####### Locality.

Corpus Christi Botanical Gardens

####### Time of activity.

Male (May); female (May, October)

####### Habitat.

(web: in web)

####### Type.

unknown

####### Etymology.

Latin, silver

####### Collection.

TAMU

###### Argiope aurantia

Taxon classificationAnimaliaAraneaeAraneidae

Lucas, 1833

Argiope aurantia Agnew et al. 1985: 3; Barron et al. 1999: 550; Bonnet 1955: 675; Breene et al. 1993c: 10, 47, 101, mf (figs 147A-C); Brown 1974: 232; Bumroongsook et al. 1992: 18; Calixto et al. 2013: 181; Cokendolpher and Reddell 2001b: 37; Dean and Sterling 1990: 404; Dean et al. 1982: 254; Dean et al. 1987: 268; Dondale et al. 2003: 155, mf, desc. (figs 323–328); Harwood 1974: 131; Hoffmaster 1985: 627; Jackman 1997: 75, desc., 161 (photo 21h); Jackman et al. 2007: 199; Jones 1936: 70; Kagan 1942: 23; Kagan 1943: 258; Levi 1968: 338 [S], mf, desc. (figs 43–57); Levi 2004: 52; Nyffeler et al. 1986: 200; Nyffeler et al. 1987c: 368; Nyffeler et al. 1988a: 55; Nyffeler et al. 1992a: 1181; Reddell 1965: 170; Reddell and Finch 1963: 48; Roberts 2001: 48; Sterling et al. 1979: 979; Taber and Fleenor 2003: 236; Taber and Fleenor 2005: 275 (figs 12–13); Vogel 1970b: 4; Young and Edwards 1990: 14
Argiope cophinaria (Walckenaer, 1841); McCook 1893: 217
Epeira riparia Hentz, 1847; Hentz 1875: 106

####### Distribution.

Eastern 2/3 Texas; Archer, Atascosa, Bastrop, Bell, Bexar, Brazos, Burleson, Clay, Collin, Colorado, Comal, Comanche, Cooke, Coryell, Dallas, DeWitt, Denton, Erath, Fannin, Galveston, Gonzales, Grimes, Harris, Hays, Houston, Hunt, Johnson, Kendall, Kerr, Leon, Liberty, Matagorda, McLennan, Nacogdoches, Navarro, Potter, Robertson, Sabine, San Patricio, Travis, Victoria, Walker, Washington, Wichita, Williamson, Wilson

####### Locality.

Adriance Pecan Orchard, Attwater Prairie Chicken National Wildlife Refuge, Bill Haney Pecan Orchard, Brison Pecan Orchard, Ellis Prison Unit, Fort Hood, Fort Sam Houston, Holmes Pecan Orchard, Lackland Air Force Base, Lake Grapevine, Lake Tawakoni State Park, Lick Creek Park, Palmetto State Park, Stubblefield Lake, Texas A&M University Rangeland Area, Welder Wildlife Refuge, Wildcat Bluff Nature Center, Williams Lake

####### Caves.

**Bell** (Medusa Cave [Fort Hood], Road Side Sink [Fort Hood], Seven Cave [Fort Hood]); **Coryell** (Brokeback Cave [Fort Hood], Mixmaster Cave [Fort Hood]); **Hays** (Ezell’s Cave, Fern Cave); **Kendall** (Cueva de los Tres Bobos); **Williamson** (Steam Cave)

####### Time of activity.

Male (June – September); female (June – November)

####### Habitat.

(crops: cotton, peanuts); (grass: grass, grassland); (landscape features: barns, cave); (littoral: wetlands); (nest/prey: mud dauber nest); (orchard: pecan); (plants: miscellaneous vegetation, vegetation); (soil/woodland: hackberry woodland, trees); (structures: under picnic table); (web: large spider web)

####### Method.

Beating [mf]; cardboard band [imm.]; fogging [m]; pitfall trap [imm.]; suction trap [mf]; sweeping [mf]

####### Type.

North America

####### Etymology.

New Latin, orange

####### Collection.

DMNS, MCZ, MSU, TAMU, TMM

###### Argiope blanda

Taxon classificationAnimaliaAraneaeAraneidae

O. P.-Cambridge, 1898

Argiope blanda Jackman 1997: 161; Levi 1968: 348, mf, desc. (figs 137–153); Levi 2004: 60; Vogel 1970b: 4

####### Distribution.

South Texas; Cameron

####### Locality.

Sabal Palm Audubon Sanctuary

####### Time of activity.

Male (May)

####### Type.

Guatemala, Santa Ana

####### Etymology.

Latin, smooth

####### Collection.

MCZ

###### Argiope trifasciata

Taxon classificationAnimaliaAraneaeAraneidae

(Forskål, 1775)

Argiope trifasciata Agnew et al. 1985: 3; Breene 1988: 23–24; Breene et al. 1988: 180; Breene et al. 1993c: 10, 47, 101, mf (figs 148A-C); Cokendolpher et al. 2008: 8, 13 (fig. 3, photo 13); Dean et al. 1982: 254; Dean et al. 1988: 285–286; Dondale et al. 2003: 157, mf, desc. (figs 329–335); Jackman 1997: 76, desc., 161 (photo 21i); Jäger 2012: 294; Jones 1936: 70; Knutson et al. 2010: 515; Levi 1968: 340 [S], mf, desc. (figs 58–72, 74–91); Levi 2004: 54; Nyffeler et al. 1986: 200; Nyffeler et al. 1987c: 370; Rapp 1984: 4; Roberts 2001: 48; Rogers and Horner 1977: 523; Taber and Fleenor 2003: 236; Vogel 1970b: 4; Young and Edwards 1990: 14
Argiope avara Thorell, 1859; McCook 1893: 222, pl. XIV

####### Distribution.

Widespread; Archer, Bastrop, Bell, Bexar, Borden, Brazos, Brown, Burleson, Burleson/Lee, Burnet, Caldwell, Carson, Clay, Collin, Concho, Coryell, Dallas, Denton, Ector, Erath, Fannin, Fayette, Galveston, Garza, Houston, Howard, Lubbock, Martin, Nueces, Oldham, Pecos, Potter, Presidio, Rains, Reagan, Runnels, Travis, Upton, Walker, Ward, Wichita, Young

####### Locality.

Ellis Prison Unit, Lake Dallas, Lick Creek Park, Pantex Lake (edge), Texas A&M University Rangeland Area, Wildcat Bluff Nature Center

####### Time of activity.

Male (June – October); female (January, July, September – November)

####### Habitat.

(crops: cotton, guar, peanuts); (grass: broom weed, grassland, pasture); (nest/prey: mud dauber nest [imm.]); (plants: bush, miscellaneous vegetation, roadside vegetation, vegetation, *Baccharis*); (soil/woodland: oak, post oak savanna, post oak savanna with pasture, saltcedar, trees/shrubs); (web: in web)

####### Method.

Beating [m]; suction trap [m]; sweeping [mf]

####### Type.

Egypt

####### Etymology.

Latin, three stripes on abdomen of immature

####### Collection.

DMNS, JCC, MSU, TAMU, TTU

##### Genus *Colphepeira* Archer, 1941

###### Colphepeira catawba

Taxon classificationAnimaliaAraneaeAraneidae

(Banks, 1911)

Colphepeira catawba Jackman 1997: 161; Levi 1978: 422, mf, desc. (figs 1–15); Roth 1982: 11–2; Roth 1985: B-6–6, B-6–11; Roth 1994: 69

####### Distribution.

Brazos, Val Verde, Wilbarger

####### Locality.

Seminole Canyon State Park

####### Time of activity.

Male (May, October)

####### Type.

North Carolina, Asheville

####### Etymology.

Indian tribe

####### Collection.

TAMU

##### Genus *Cyclosa* Menge, 1866

###### Cyclosa berlandi

Taxon classificationAnimaliaAraneaeAraneidae

Levi, 1999

Cyclosa berlandi Levi 1999: 358, mf, desc. (figs 322–332)
Cyclosa walckenaeri (O. P.-Cambridge, 1889); Levi 1977a: 84 [west Texas record]

####### Distribution.

Brewster

####### Locality.

Big Bend National Park, Chisos Mountains

####### Time of activity.

Female (September)

####### Type.

Ecuador, 20 km N Cuenca

####### Etymology.

Person (Berland described spiders from the mountains of Ecuador, illustrated the abdomen of the male, with three posterior tubercles and a nondiagnostic view of the male palpus. As there is only one common species in the area with triforked abdomen in males; the identification is easy, Levi 1999).

###### Cyclosa caroli

Taxon classificationAnimaliaAraneaeAraneidae

(Hentz, 1850)

Cyclosa caroli Jackman 1997: 161; Levi 1977a: 82, mf, desc. (figs 51–63); Levi 1999: 336, mf, desc. (figs 162–180)

####### Distribution.

East and south Texas; McLennan

####### Type.

Alabama

####### Etymology.

undetermined

####### Collection.

MSU

###### Cyclosa conica

Taxon classificationAnimaliaAraneaeAraneidae

(Pallas, 1772)

Cyclosa conica Rapp 1984: 4; Woods and Harrel 1976: 43; Young and Edwards 1990: 15 [Levi 1977a: 78, mf, desc. (figs 1–19); page 80: many specimens of Cyclosa turbinata erroneously labeled as Cyclosa conica]

####### Distribution.

Galveston, Jefferson

####### Habitat.

(crops: rice)

####### Type.

Germany

####### Etymology.

Greek, conical

###### Cyclosa turbinata

Taxon classificationAnimaliaAraneaeAraneidae

(Walckenaer, 1841)

Cyclosa turbinata Agnew et al. 1985: 3; Breene et al. 1993c: 11, 47, 105, mf (figs 159A-C); Brown 1974: 232; Bumroongsook et al. 1992: 17; Calixto et al. 2013: 181; Dean and Eger 1986: 141; Dean and Sterling 1987: 6; Dean and Sterling 1990: 404; Dean et al. 1982: 254; Dean et al. 1987: 268; Dean et al. 1988: 286; Dondale et al. 2003: 164, mf, desc. (figs 347–354); Jackman 1997: 77, desc., 161; Levi 1977a: 80, mf, desc. (figs 20, 38–50); Levi 1999: 356, mf, desc. (figs 314–321); Liao et al. 1984: 410; Nyffeler and Sterling 1994: 1295, 1298; Nyffeler et al. 1986: 196; Nyffeler et al. 1988a: 55; Nyffeler et al. 1992a: 1181; Nyffeler et al. 1992c: 2; Vincent and Frankie 1985: 380; Vogel 1970b: 3; Young and Edwards 1990: 15

####### Distribution.

Widespread; Bandera, Brazos, Burleson, Cameron, Collin, Comanche, Delta, Erath, Fannin, Goliad, Houston, Hunt, Kaufman, McLennan, Nacogdoches, Presidio, Robertson, Travis, Val Verde, Walker, Wharton, Wichita, Williamson

####### Locality.

Adriance Pecan Orchard, Bill Haney Pecan Orchard, Ellis Prison Unit, Goliad State Park, Holmes Pecan Orchard, Lost Maples State Park, South Padre Island, Texas A&M University Rangeland Area

####### Time of activity.

Male (March – September); female (March – October)

####### Habitat.

(crops: cotton, peanuts); (grass: grassland, pasture); (littoral: behind sand dune, past dunes, shrub); (orchard: pecan); (plants: bluebonnets, croton, miscellaneous vegetation, prickly pear, *Baccharis*, *Monarda citriodora*); (soil/woodland: live oak, *Quercus buckleyi*, *Quercus virginiana*, *Ulmus crassifolia*); (web: web in hollow sycamore tree, web in shrub)

####### Method.

Beating [m]; cardboard band [m]; D-Vac suction [m]; fogging [mf]; suction trap [m]; sweeping [mf]

####### Type.

Georgia

####### Etymology.

Latin, top-shaped

####### Collection.

DMNS, MSU, TAMU

###### Cyclosa walckenaeri

Taxon classificationAnimaliaAraneaeAraneidae

(O. P.-Cambridge, 1889)

Cyclosa walckenaeri Jackman 1997: 161; Levi 1977a: 84, mf, desc. (figs 64–77 [see note below]); Levi 1999: 360, mf, desc. (figs 38, 333–343)

####### Distribution.

Cameron, Hidalgo, Starr

####### Locality.

Laguna Atascosa National Wildlife Refuge

####### Time of activity.

Female (April, June, September)

####### Habitat.

(grass: grasses); (nest/prey: mud dauber nest [f] from *Chalybion californicum*); (soil/woodland: savanna with native grasses)

####### Method.

Lindgren flight trap [f]; sweeping [f]

####### Type.

Guatemala, Volcan de Fuego

####### Etymology.

Person (arachnologist)

####### Collection.

TAMU

####### Note.

West Texas record is *Cyclosa berlandi*.

##### Genus *Eriophora* Simon, 1864

###### Eriophora edax

Taxon classificationAnimaliaAraneaeAraneidae

(Blackwall, 1863)

Eriophora edax Jackman 1997: 161; Kaston 1972: 150, desc. (figs 337–338); Kaston 1978: 143, desc. (figs 360–361); Levi 1971b: 296, mf, desc. (figs 35–48)

####### Distribution.

South Texas; Cameron, Hidalgo

####### Locality.

Anzalduas County Park, Bentsen-Rio Grande Valley State Park, Frontera Audubon, Russell Farm, Sabal Palm Audubon Sanctuary

####### Time of activity.

Male (March – April, August, October); female (February, May – June, August, December)

####### Habitat.

(orchard: grapefruit, orange); (structures: on pavement)

####### Type.

Brazil, Rio de Janeiro

####### Etymology.

Latin, greedy or devouring

####### Collection.

MSU, NMSU, TAMU

###### Eriophora ravilla

Taxon classificationAnimaliaAraneaeAraneidae

(C. L. Koch, 1844)

Eriophora ravilla Bradley 2013: 89; Breene et al. 1993c: 11, 47, 103, mf (figs 154A-D); Dean et al. 1982: 254; Jackman 1997: 77, desc., 161 (photo 21k); Levi 1971b: 286 [S, T], mf, desc. (figs 7–24); Roewer 1942: 866; Young and Edwards 1990: 15
Epeira ravilla C. L. Koch, 1844; Banks 1910: 43; Marx 1890: 547; McCook 1893: 161
Araneus ravillus (C. L. Koch, 1844); Petrunkevitch 1911: 312
Epeira balaustina McCook, 1888; McCook 1893: 155
Epeira bivariolata O. P.-Cambridge, 1889; McCook 1893: 159
Araneus balaustinus (McCook, 1888); Petrunkevitch 1911: 281
Eriophora variolata O. P.-Cambridge, 1889; F. O. P.-Cambridge 1903: 464
Araneus variolatus (O. P.-Cambridge, 1889); Petrunkevitch 1911: 323; Vogel 1970b: 3

####### Distribution.

Southeast and south Texas; Aransas, Brazoria, Brazos, Cameron, Harris, Hidalgo, Nacogdoches, Nueces, Walker

####### Locality.

Ellis Prison Unit, Frontera Audubon, Lick Creek Park, Resaca de la Palma State Park, Russell Farm, Sabal Palm Audubon Sanctuary, Santa Ana National Wildlife Refuge

####### Time of activity.

Male (March – April, November); female (March – June, August, October – November)

####### Habitat.

(crops: cotton); (grass: grasses); (orchard: orange, grapefruit); (soil/woodland: forest, palm forest); (structures: around house)

####### Method.

Beating [mf]; sweeping [m]

####### Type.

Mexico

####### Etymology.

Latin, gray-yellow

####### Collection.

NMSU, TAMU

##### Genus *Eustala* Simon, 1895

###### Eustala anastera

Taxon classificationAnimaliaAraneaeAraneidae

(Walckenaer, 1841)

Eustala anastera Agnew et al. 1985: 3; Armstrong and Richman 2007: 395; Bonnet 1956: 1837; Breene et al. 1993c: 11, 47, 104, mf (figs 155A-C); Brown 1974: 232; Calixto et al. 2013: 181; Dean and Eger 1986: 141; Dean and Sterling 1987: 6; Dean and Sterling 1990: 402, 404; Dean et al. 1982: 254; Dean et al. 1988: 285–286; Dondale et al. 2003: 267, mf, desc. (figs 600–611); Jackman 1997: 78, desc., 161 (photo 21l); Kagan 1942: 30; Kagan 1943: 258; Knutson et al. 2010: 515; Levi 1977a: 114 [S], mf, desc. (figs 205–232, 280–285, 298–302, 314–315); Liao et al. 1984: 410; Rapp 1984: 4; Rogers and Horner 1977: 523; Young and Edwards 1990: 15
Epeira anastera Walckenaer, 1841; McCook 1893: 172
Eustala anestera (Walckenaer, 1841); Vogel 1970b: 4
Eustala prompta (Hentz, 1847); Jones 1936: 70

####### Distribution.

Widespread; Archer, Atascosa, Bastrop, Baylor, Bee, Blanco, Brazos, Brown, Burleson, Cameron, Clay, Collin, Colorado, Comanche, Dallas, Erath, Galveston, Gillespie, Hidalgo, Houston, Howard, Hunt, McLennan, Montague, Nacogdoches, Nueces, Orange, Presidio, Robertson, Scurry, Travis, Walker, Wichita

####### Locality.

Attwater Prairie Chicken National Wildlife Refuge, Big Bend Ranch State Park, Bill Haney Pecan Orchard, Ellis Prison Unit, Frontera Audubon, Galveston Island State Park, Holmes Pecan Orchard, Lick Creek Park, Proctor Lake, Russell Farm, Sabal Palm Audubon Sanctuary

####### Time of activity.

Male (March – October); female (April – December)

####### Habitat.

(crops: cotton, guar, peanuts); (grass: grass, grassland, grassy and shrub area, pasture); (littoral: salt marsh area, sandy area); (nest/prey: mud dauber nest [f]); (objects: croton cage); (orchard: grapefruit, orange, pecan, sour orange, tangerine); (plants: bluebonnets, Indian paintbrush, vegetation); (soil/woodland: brush, mesquite, saltcedar, trees/shrubs, *Juniperus ashei*, *Quercus virginiana*, *Ulmus crassifolia*)

####### Method.

Beating [m]; beating/sweeping [f]; black light trap [m]; boll weevil pheromone trap [mf]; cardboard band [mf]; D-Vac suction [f]; fogging [mf]; pitfall trap [mf]; suction trap [mf]; sweeping [mf]

####### Type.

Georgia

####### Etymology.

Greek, solid throughout

####### Collection.

DMNS, MSU, NMSU, TAMU

###### Eustala bifida

Taxon classificationAnimaliaAraneaeAraneidae

F. O. P.-Cambridge, 1904

Eustala bifida Armstrong and Richman 2007: 395; Jackman 1997: 161; Levi 1977a: 108, mf, desc. (figs 167–175, 178)

####### Distribution.

Cameron, Wichita

####### Locality.

Russell Farm, Sabal Palm Audubon Sanctuary

####### Time of activity.

Male (March); female (February)

####### Habitat.

(soil/woodland: palm grove)

####### Method.

Boll weevil pheromone trap [m]

####### Type.

Costa Rica, San Jose

####### Etymology.

Latin, female abdomen with two conical tubercles at end

####### Collection.

MSU, NMSU

###### Eustala brevispina

Taxon classificationAnimaliaAraneaeAraneidae

Gertsch & Davis, 1936

Eustala brevispina Armstrong and Richman 2007: 395; Bonnet 1956: 1839; Gertsch and Davis 1936: 12, mf, desc. (figs 9–10); Jackman 1997: 161; Levi 1977a: 106, mf, desc. (figs 149–158); Roewer 1942: 768; Vogel 1970b: 4

####### Distribution.

Cameron

####### Locality.

Russell Farm

####### Time of activity.

Male (December); female (March, May – June)

####### Method.

Boll weevil pheromone trap [f]

####### Type.

Texas (male, Cameron Co., December 1934, L. I. Davis, holotype, AMNH)

####### Etymology.

Latin, short spines

####### Collection.

NMSU

###### Eustala cameronensis

Taxon classificationAnimaliaAraneaeAraneidae

Gertsch & Davis, 1936

Eustala cameronensis Bonnet 1956: 1839; Gertsch and Davis 1936: 13, m, desc. (fig. 13); Jackman 1997: 161; Levi 1977a: 112, m, desc. (figs 189–191); Roewer 1942: 768
Eustala cameronsis Gertsch & Davis, 1936; Vogel 1970b: 4

####### Distribution.

Cameron, Hidalgo

####### Time of activity.

Male (“January-March”, September)

####### Type.

Texas (male, Cameron Co., January-March 1936, L. I. Davis, holotype, AMNH)

[female unknown]

####### Etymology.

locality (county)

###### Eustala cepina

Taxon classificationAnimaliaAraneaeAraneidae

(Walckenaer, 1841)

Eustala cepina Breene et al. 1993c: 11, 47, 104, mf (figs 156A-C); Calixto et al. 2013: 181; Dean and Eger 1986: 141; Dean and Sterling 1987: 6; Dean and Sterling 1990: 404; Dondale et al. 2003: 271, mf, desc. (figs 622–631); Jackman 1997: 161; Levi 1977a: 118, mf, desc. (figs 233–252, 286–290, 303–308, 316)

####### Distribution.

Eastern ½ Texas; Archer, Brazos, Cameron, Clay, Colorado, Comanche, Dickens, Fayette (imm.), Hunt, Montague, Robertson, Throckmorton, Travis, Walker, Wichita, Willacy, Williamson

####### Locality.

Attwater Prairie Chicken National Wildlife Refuge, Bill Haney Pecan Orchard, Ellis Prison Unit, Holmes Pecan Orchard

####### Time of activity.

Male (March – July); female (March – August)

####### Habitat.

(crops: cotton); (nest/prey: mud dauber nest); (orchard: pecan); (plants: bluebonnets, Indian paintbrush, vegetation); (soil/woodland: tree, trees/shrubs, *Quercus buckleyi*)

####### Method.

Beating [f]; cardboard band [f]; fogging [mf]; suction trap [m]; sweeping [mf]

####### Type.

Georgia

####### Etymology.

Latin, field

####### Collection.

MSU, TAMU

###### Eustala clavispina

Taxon classificationAnimaliaAraneaeAraneidae

(O. P.-Cambridge, 1889)

Eustala clavispina Jackman 1997: 161; Levi 1977a: 106, mf, desc. (figs 159–166, 176–177)
Eustala rosae Chamberlin & Ivie, 1935; Gertsch and Davis 1936: 14; Vogel 1970b: 4 [Texas records]

####### Distribution.

Cameron, Hidalgo

####### Locality.

Hoblitzelle Farms

####### Time of activity.

Male (February)

####### Type.

Guatemala, Vera Paz, Cubilguitz

####### Etymology.

Latin, upper side of abdomen with claviform spines

####### Collection.

TAMU

###### Eustala conchlea

Taxon classificationAnimaliaAraneaeAraneidae

(McCook, 1888)

Eustala conchlea [Levi 1977a: 122, mf, desc. (figs 269–279, 296, 312, 318)]

####### Distribution.

Clay

####### Type.

California

####### Etymology.

Greek, shell-like

####### Collection.

MSU

###### Eustala devia

Taxon classificationAnimaliaAraneaeAraneidae

(Gertsch & Mulaik, 1936)

Eustala devia Jackman 1997: 161; Levi 1977a: 101 [T], mf, desc. (figs 118–127)
Neosconella devia Gertsch & Mulaik, 1936; Bonnet 1958: 3061; Gertsch and Mulaik 1936b: 16, f, desc. (fig. 38); Vogel 1970b: 4
Aranea devia (Gertsch & Mulaik, 1936); Roewer 1942: 860

####### Distribution.

Hidalgo

####### Time of activity.

Female (August)

####### Type.

Texas (female, Hidalgo Co., Edinburg, August 25, 1935, S. Mulaik, holotype, AMNH)

####### Etymology.

Latin, out of the way

###### Eustala emertoni

Taxon classificationAnimaliaAraneaeAraneidae

(Banks, 1904)

Eustala emertoni Dondale et al. 2003: 269, mf, desc. (figs 612–621); Jackman 1997: 161; Jackman et al. 2007: 199; Levi 1977a: 120, mf, desc. (figs 253–268, 291–295, 309–311, 317); Tugmon et al. 1990: 44

####### Distribution.

Eastern 2/3 Texas; Archer, Bastrop, Bell, Bosque, Brazoria, Brazos, Brown, Cameron, Colorado, Denton, Hunt, Kaufman, Robertson, Travis, Walker, Wichita

####### Locality.

Ellis Prison Unit, Holmes Pecan Orchard, Lacuna Park, Lake Tawakoni State Park, Lick Creek Park, Nash Prairie, South Padre Island

####### Time of activity.

Male (April – July, September – November); female (March – October)

####### Habitat.

(crops: cotton); (grass: grass, grass marsh, grassland); (littoral: behind dune, dune vegetation, low dune grass); (nest/prey: mud dauber nest); (orchard: pecan); (plants: croton, miscellaneous vegetation, vegetation); (soil/woodland: woods, *Quercus buckleyi*, *Quercus virginiana*, *Ulmus crassifolia*); (web: large spider web)

####### Method.

Beating [f]; beating/sweeping [f]; D-Vac suction [f]; sweeping [mf]

####### Type.

Georgia

####### Etymology.

Person (arachnologist)

####### Collection.

DMNS, MSU, TAMU

##### Genus *Gasteracantha* Sundevall, 1833

###### Gasteracantha cancriformis

Taxon classificationAnimaliaAraneaeAraneidae

(Linnaeus, 1758)

Gasteracantha cancriformis Bonnet 1957: 1945; Brown 1974: 232; Bumroongsook et al. 1992: 18; Jackman 1997: 78, desc., 161 (photo 21m); Levi 1978: 437, mf, desc. (figs 69–84); Liao et al. 1984: 410; McCook 1893: 211; Marx 1890: 539; Petrunkevitch 1911: 343; Roth 1982: 11–2; Roth 1985: B-6–2, B-6–8; Roth 1994: 68; Taber and Fleenor 2003: 237; Taber and Fleenor 2005: 281; Vogel 1970b: 5; Yantis 2005: 197

####### Distribution.

Eastern 2/3 Texas; Bastrop, Bell, Bexar, Brazoria, Brazos, Burleson, Cameron, Denton, Galveston, Gonzalez, Grimes, Harris, Hidalgo, Leon, McLennan, Nacogdoches, Sabine, San Patricio, Shelby, Travis, Van Zandt, Walker, Wharton, Wichita, Zapata

####### Locality.

5-Eagle Ranch, Adriance Pecan Orchard, Lick Creek Park, Lower Rio Grande Valley National Wildlife Refuge, Nash Prairie, Palmetto State Park, Sabal Palm Audubon Sanctuary, Texas A&M University Rangeland Area

####### Time of activity.

Male (April, June – July, September – October); female (January – December)

####### Habitat.

(grass: grassland); (littoral: sedge meadow); (nest/prey: mud dauber nest [imm.] from *Chalybion californicum*); (orchard: pecan); (plants: bush, miscellaneous vegetation, vegetation); (soil/woodland: beech-magnolia forest, oak pine forest, post oak savanna, post oak woods [%: 85], re-vegetated site, trees, woods); (web: web near creek)

####### Method.

5 gallon bucket trap [f]; beating [mf]; beating/sweeping [f]; malaise trap [f]; sweeping [f]; uv light [m]

####### Type.

Jamaica

####### Etymology.

Latin, crab-like

####### Collection.

DMNS, MSU, TAMU

####### Note.

Color variation of abdomen of female includes white, yellow, orange and red.

##### Genus *Gea* C. L. Koch, 1843

###### Gea heptagon

Taxon classificationAnimaliaAraneaeAraneidae

(Hentz, 1850)

Gea heptagon Agnew et al. 1985: 7; Breene et al. 1993c: 11, 47, 101, mf (figs 146A-C); Brown 1974: 232; Dean and Eger 1986: 141; Dean and Sterling 1985: 116; Dean and Sterling 1987: 6; Dean and Sterling 1992: 3–4; Dean et al. 1982: 254; Dean et al. 1987: 268; Dean et al. 1988: 285–286; Dondale et al. 2003: 151, mf, desc. (figs 314–322); Jackman 1997: 79, desc., 161; Kagan 1942: 37; Kagan 1943: 258; Kaston 1972: 143, desc. (fig. 320); Kaston 1978: 137, desc. (fig. 343); Knutson et al. 2010: 515; Levi 1968: 324, mf, desc. (figs 1–24); Nyffeler and Sterling 1994: 1295, 1298; Nyffeler et al. 1987c: 372; Nyffeler et al. 1988a: 55; Nyffeler et al. 1989: 374, 377; Nyffeler et al. 1992c: 2; Rapp 1984: 5; Roth 1982: 11–2; Roth 1985: B-6–2, B-6–8; Roth 1994: 67; Vogel 1970b: 4; Woods and Harrel 1976: 43; Young and Edwards 1990: 15

####### Distribution.

East and south Texas; Brazos, Burleson, Caldwell, Colorado, Comal, Erath, Fayette, Fort Bend, Galveston, Houston, Howard, Jefferson, Kerr, Madison, Matagorda, McLennan, Nacogdoches, Nueces, Polk, San Patricio, Travis, Van Zandt, Walker, Wichita

####### Locality.

Attwater Prairie Chicken National Wildlife Refuge, Ellis Prison Unit, Lick Creek Park, Texas A&M University Rangeland Area

####### Time of activity.

Male (March – November); female (March, May – September)

####### Habitat.

(crops: cotton, rice); (grass: grassland, grassy and shrub area, pasture); (littoral: salt marsh area); (nest/prey: mud dauber nest [mf]); (plants: Indian paintbrush, miscellaneous vegetation, vegetation, yarrow, *Monarda citriodora*); (soil/woodland: forest, saltcedar, *Quercus virginiana*); (structures: indoors)

####### Method.

Beating/sweeping [f]; D-Vac suction [f]; pitfall trap [mf]; suction trap [imm.]; sweeping [mf]

####### Type.

North Carolina and Alabama

####### Etymology.

Greek, seven-sided

####### Collection.

DMNS, MSU, NMSU, TAMU

##### Genus *Hypsosinga* Ausserer, 1871

###### Hypsosinga funebris

Taxon classificationAnimaliaAraneaeAraneidae

(Keyserling, 1892)

Hypsosinga funebris Cokendolpher et al. 2008: 8, 13; Dean and Eger 1986: 141; Dondale et al. 2003: 292, mf, desc. (figs 688–696); Jackman 1997: 161; Levi 1975b: 273 [S]
Hypsosinga singaeformis (Scheffer, 1904); Cokendolpher and Reddell 2001b: 37; Levi 1972: 246, mf, desc. (figs 58–71)

####### Distribution.

Andrews, Atascosa, Bell, Bexar, Brazos, Burleson, Cameron, Carson, Fayette, Glasscock, Hidalgo, Howard, Kerr, Motley, Sterling, Uvalde, Val Verde

####### Locality.

5-Eagle Ranch, Fort Hood, Garner State Park, NK Ranch, South Padre Island, Seminole Canyon State Park

####### Caves.

**Bell** (Canyon Side Sink [Fort Hood])

####### Time of activity.

Male (March 30-April 6, April – July, September); female (April – July, September)

####### Habitat.

(grass: dune, grassland); (landscape features: cave); (littoral: dune, near playa); (plants: Indian paintbrush, miscellaneous vegetation, roadside vegetation); (soil/woodland: post oak savanna with pasture, trees/shrubs)

####### Method.

Beating [mf]; pitfall trap [mf]; sweeping [mf]

####### Type.

Florida, Crescent City

####### Etymology.

Latin, of a funeral

####### Collection.

TAMU

###### Hypsosinga rubens

Taxon classificationAnimaliaAraneaeAraneidae

(Hentz, 1847)

Hypsosinga rubens Agnew et al. 1985: 3; Breene et al. 1993c: 11, 47, 107, mf (figs 165A-C); Dean et al. 1982: 254; Dondale et al. 2003: 289, mf, desc. (figs 675–687); Jackman 1997: 161; Kaston 1978: 152, desc. (fig. 380); Levi 1972: 248 [S], mf, desc. (figs 72–88); Young and Edwards 1990: 15
Singa nigripes Keyserling, 1884; McCook 1893: 232; Marx 1890: 549
Araneus nigripes (Keyserling, 1884); Petrunkevitch 1911: 306

####### Distribution.

North-central and central Texas; Aransas, Brazos, Brown, Erath, Fannin, Hunt, Kenedy, Montague, Montgomery, San Saba, Travis, Walker, Young

####### Locality.

Ellis Prison Unit, Goose Island State Park, Jones State Forest, Lick Creek Park, NK Ranch

####### Time of activity.

Male (March – May, August), female (March – June, August)

####### Habitat.

(crops: cotton, peanuts); (plants: miscellaneous vegetation); (soil/woodland: juniper, woods, *Juniperus ashei*, *Ulmus crassifolia*)

####### Method.

Beating [f]; hanging carrion trap [f]; pitfall trap [f]; sweeping [f]

####### Type.

Alabama

####### Etymology.

Latin, red

####### Collection.

MSU, TAMU

##### Genus *Kaira* O. P.-Cambridge, 1889

###### Kaira alba

Taxon classificationAnimaliaAraneaeAraneidae

(Hentz, 1850)

Kaira alba Jackman 1997: 161; Levi 1977b: 216, mf, desc. (figs 117–129); Levi 1993c: 216, mf, desc. (figs 1- 2, 23–28)

####### Distribution.

North-central and south Texas; Brazos, Denton, Hidalgo, Travis, Uvalde

####### Locality.

Garner State Park, Lick Creek Park

####### Time of activity.

Male (May, July)

####### Habitat.

(soil/woodland: *Quercus virginiana*)

####### Method.

Beating [m]; beating/sweeping [m]

####### Type.

North Carolina

####### Etymology.

Latin, white

####### Collection.

TAMU

###### Kaira altiventer

Taxon classificationAnimaliaAraneaeAraneidae

O. P.-Cambridge, 1889

Kaira altiventer Jackman 1997: 161; Levi 1977b: 218, mf, desc. (figs 130–137); Levi 1993c: 213, mf, desc. (figs 3–22)

####### Distribution.

Cameron, Hidalgo

####### Locality.

Frontera Audubon

####### Time of activity.

Male (March, August); female (December)

####### Habitat.

(orchard: grapefruit, sour orange); (soil/woodland: low shrubs)

####### Type.

Panama, Veragux

####### Etymology.

Latin, high belly

####### Collection.

TAMU

###### Kaira hiteae

Taxon classificationAnimaliaAraneaeAraneidae

Levi, 1977

Kaira hiteae Jackman 1997: 161; Levi 1977b: 220, m, desc. (figs 138–140); Levi 1993c: 222, f, desc. (figs 82–85)

####### Distribution.

Brazos, Cameron, Colorado, Dallas, Grayson, Travis

####### Locality.

Attwater Prairie Chicken National Wildlife Refuge, South Padre Island, Texas A&M University Rangeland Area

####### Time of activity.

Male (July – August); female (September – November)

####### Habitat.

(plants: vegetation)

####### Method.

sweeping [f]

####### Type.

Arkansas, Boston Mountains, Cove Creek Valley

####### Etymology.

Person (The species is named after M. Hite, the collector of several specimens of this rare species, Levi 1993c).

####### Collection.

MCZ, TAMU

##### Genus *Larinia* Simon, 1874

###### Larinia directa

Taxon classificationAnimaliaAraneaeAraneidae

(Hentz, 1847)

Larinia directa Agnew et al. 1985: 4; Armstrong and Richman 2007: 395; Banks 1894: 8; Breene et al. 1993b: 647; Brown 1974: 232; Dean and Eger 1986: 141; Dean and Sterling 1990: 404; Hoffmaster 1985: 627; Jackman 1997: 79, desc., 161; Knutson et al. 2010: 515; Levi 1975a: 105 [T], mf, desc. (figs 1–12, 31, 34, 37–41); Young and Edwards 1990: 15
Drexelia directa (Hentz, 1847); Woods and Harrel 1976: 43

####### Distribution.

Southern ½ Texas, west Texas; Archer, Bosque, Brazos, Brewster, Burleson, Cameron, Colorado, Erath, Fayette, Freestone, Goliad, Hidalgo, Hopkins, Howard, Hunt, Jefferson, Kenedy, Nacogdoches, Presidio, San Patricio, Victoria, Walker, Wichita, Willacy

####### Locality.

Attwater Prairie Chicken National Wildlife Refuge, Bentsen-Rio Grande Valley State Park, Ellis Prison Unit, Lacuna Park, Lick Creek Park, Russell Farm, Sabal Palm Audubon Sanctuary, Santa Ana National Wildlife Refuge, Somerville Lake, South Padre Island, Welder Wildlife Refuge

####### Time of activity.

Male (February – August, October, December); female (February – September)

####### Habitat.

(crops: peanuts, rice, sugarcane); (littoral: dune); (nest/prey: mud dauber nest [f]); (orchard: grapefruit, orange); (plants: bluebonnets, Indian paintbrush, miscellaneous vegetation, vegetation, next to cotton field); (soil/woodland: saltcedar)

####### Method.

Boll weevil pheromone trap [mf]; moth pheromone trap [f]; suction trap [m]; sweeping [mf]

####### Type.

South Carolina and Alabama

####### Etymology.

Latin, straight

####### Collection.

DMNS, MSU, NMSU, TAMU

##### Genus *Larinioides* Caporiacco, 1934

###### Larinioides cornutus

Taxon classificationAnimaliaAraneaeAraneidae

(Clerck, 1757)

Larinioides cornutus Dondale et al. 2003: 184, mf, desc. (figs 395–401); Grasshoff 1983: 227 [T]; Guarisco 2008b: 5; Jackman 1997: 80, desc., 161; Jackman et al. 2007: 199; Roberts 2001: 48
Nuctenea cornuta (Clerck, 1757); Agnew et al. 1985: 7; Levi 1974: 306 [S, T], mf, desc. (figs 61–62, 67–76, 94, 97–98, 110–111, 118–119, 126); Rapp 1984: 5
Araneus cornutus Clerck, 1757; Vogel 1970b: 3
Epeira strix Hentz, 1847; Jones 1936: 70

####### Distribution.

Eastern 2/3 Texas; Archer, Baylor, Brown, Burnet, Clay, Comanche, Cooke, Dallas, Denton, Galveston, Grayson, Hood, Hunt, Lee, Palo Pinto, Potter, Travis, Wichita

####### Locality.

Galveston Island State Park, Inks Lake State Park, Lake Somerville State Park [Nails Creek Unit], Lake Tawakoni State Park, Lakeside Park South, Proctor Lake, Wildcat Bluff Nature Center

####### Time of activity.

Male (March – April, June, August – September, November); female (January – May, July – December)

####### Habitat.

(grass: grassy and shrub area, pasture); (littoral: salt marsh area); (soil/woodland: sandy area, tree, under bark); (structures: house); (web: communal web, dead in web, large spider web)

####### Method.

Beating [mf]; pitfall trap; sweeping

####### Type.

Sweden

####### Etymology.

Latin, referring to horn or projection

####### Collection.

DMNS, MSU, TAMU

###### Larinioides patagiatus

Taxon classificationAnimaliaAraneaeAraneidae

(Clerck, 1757)

Larinioides patagiatus Dondale et al. 2003: 186, mf, desc. (figs 402–408); Grasshoff 1983: 227 [T]; Jackman 1997: 161
Nuctenea patagiata (Clerck, 1757); Levi 1974: 309, mf, desc. (figs 77–84, 100–102, 107, 112–113, 120–123, 127)

####### Distribution.

South Texas

####### Type.

Sweden

####### Etymology.

Latin, gold-bordered

###### Larinioides sclopetarius

Taxon classificationAnimaliaAraneaeAraneidae

(Clerck, 1757)

Larinioides sclopetarius Grasshoff 1983: 227 [T]; Jackman 1997: 161
Nuctenea sclopetaria (Clerck, 1757) [Levi 1974: 310 [S], mf, desc. (figs 85–88, 103–104, 108, 114–115, 124–125, 128)]
Araneus sericatus Clerck, 1757; Brown 1974: 232

####### Distribution.

Nacogdoches

####### Locality.

Lake Rayburn

####### Time of activity.

Male (August); female (August)

####### Type.

Sweden

####### Etymology.

Greek, pointed

##### Genus *Mangora* O. P.-Cambridge, 1889

###### Mangora calcarifera

Taxon classificationAnimaliaAraneaeAraneidae

F. O. P.-Cambridge, 1904

Mangora calcarifera Jackman 1997: 161; Levi 1975a: 132, mf, desc. (figs 131–144); Levi 2005a: 150

####### Distribution.

South Texas; Cameron

####### Time of activity.

Male (March, September); female (October)

####### Habitat.

(soil/woodland: palm grove)

####### Type.

Guatemala, Petexbatún

####### Etymology.

Latin, spur on palp

###### Mangora fascialata

Taxon classificationAnimaliaAraneaeAraneidae

Franganillo, 1936

Mangora fascialata Agnew et al. 1985: 7; Breene et al. 1993c: 11, 47, 102, mf (figs 150A-C); Dean and Sterling 1987: 6; Jackman 1997: 161; Levi 1975a: 128, mf, desc. (figs 110–117); Levi 2005a: 162

####### Distribution.

South Texas; Brazos, Brewster, Comal, Coryell, Erath, Frio, Hidalgo, Uvalde

####### Locality.

Big Bend National Park, Garner State Park, Texas A&M University Rangeland Area

####### Time of activity.

Male (June – July); female (May – July)

####### Habitat.

(crops: cotton); (littoral: cane and mesquite along river); (soil/woodland: post oak savanna with pasture)

####### Method.

sweeping

####### Type.

Cuba

####### Etymology.

Latin, a band

####### Collection.

TAMU

###### Mangora gibberosa

Taxon classificationAnimaliaAraneaeAraneidae

(Hentz, 1847)

Mangora gibberosa Agnew et al. 1985: 7; Breene et al. 1993c: 12, 47, 102, mf (figs 151A-C); Brown 1974: 232; Dean et al. 1982: 254; Dean et al. 1988: 286; Dondale et al. 2003: 134, mf, desc. (figs 271–277); Hoffmaster 1985: 627; Jackman 1997: 161; Kagan 1942: 36; Kagan 1943: 258; Kaston 1978: 140, desc. (fig. 352); Knutson et al. 2010: 515; Levi 1975a: 130, mf, desc. (figs 118–130); Levi 2005a: 161; Nyffeler et al. 1988a: 55; Vogel 1970b: 4; Young and Edwards 1990: 15

####### Distribution.

Eastern ½ Texas; Anderson, Bastrop, Brazos, Burleson, Cherokee, DeWitt, Erath, Fannin, Goliad, Gonzales, Henderson, Houston, Howard, Hunt, Kerr, Lavaca, Limestone, McLennan, Nacogdoches, Polk, Presidio, Rains, San Patricio, Travis, Uvalde, Van Zandt, Walker, Wichita, Williamson

####### Locality.

Bastrop State Park, Ellis Prison Unit, Garner State Park, Lick Creek Park, Palmetto State Park, Riley Estate, Texas A&M University Rangeland Area, Welder Wildlife Refuge

####### Time of activity.

Male (May – November); female (April – November)

####### Habitat.

(crops: cotton); (grass: grass, grassland, meadow, pasture); (nest/prey: mud dauber nest in garage [f]); (plants: miscellaneous vegetation, roadside vegetation, vegetation, *Baccharis*); (soil/woodland: post oak savanna, saltcedar, willow)

####### Method.

sweeping [mf]

####### Type.

Alabama

####### Etymology.

Latin, humped

####### Collection.

DMNS, MSU, TAMU

###### Mangora maculata

Taxon classificationAnimaliaAraneaeAraneidae

(Keyserling, 1865)

Mangora maculata Agnew et al. 1985: 7; Dondale et al. 2003: 139, mf, desc. (figs 285–290); Henderson 2007: 65, 76, 79, 82; Jackman 1997: 161; Kaston 1978: 140, desc. (fig. 353); Levi 1975a: 122, mf, desc. (figs 58–68); Levi 1975a: 122

####### Distribution.

Southeast Texas; Brazos, Comanche, Erath, Gonzales, Travis, Walker

####### Locality.

Lick Creek Park, Nabor’s Lake, Palmetto State Park

####### Time of activity.

Male (May – August); female (June – July, July 15-August 15)

####### Habitat.

(nest/prey: mud dauber nest [f]); (plants: vegetation); (soil/woodland: upland woods, woods, *Quercus buckleyi*)

####### Method.

Beating [m]; pitfall trap [f]; sweeping [m]

####### Type.

Maryland, Baltimore

####### Etymology.

Latin, markings

####### Collection.

TAMU

###### Mangora placida

Taxon classificationAnimaliaAraneaeAraneidae

(Hentz, 1847)

Mangora placida Agnew et al. 1985: 7; Brown 1974: 232; Dondale et al. 2003: 136, mf, desc. (figs 278–284); Jackman 1997: 161; Kaston 1972: 146, desc. (fig. 328); Kaston 1978: 140, desc. (fig. 351); Levi 1975a: 126, mf, desc. (figs 80–81, 90–101); Levi 2005a: 164

####### Distribution.

Eastern 2/3 Texas; Bastrop, Brazos, Burleson, Comal, Erath, Fannin, Gonzales, Kerr, Montague, Montgomery, Nacogdoches, Polk, Travis, Walker, Wichita

####### Locality.

5-Eagle Ranch, Bastrop State Park, Holmes Pecan Orchard, Jones State Forest, Lick Creek Park, Palmetto State Park, Sam Houston National Forest, Stubblefield Lake

####### Time of activity.

Male (February – July), female (March – October)

####### Habitat.

(littoral: creek bank, near creek, sedge meadow); (nest/prey: mud dauber nest [imm.]); (orchard: pecan); (plants: miscellaneous vegetation, vegetation); (soil/woodland: brush, old field, trees, woods, *Juniperus ashei*, *Quercus buckleyi*, *Quercus virginiana*, *Ulmus crassifolia*)

####### Method.

Beating [mf]; beating/sweeping [f]; cardboard band [mf]; fogging [mf]; sweeping [mf]

####### Type.

Alabama

####### Etymology.

Latin, mild or gentle, placid

####### Collection.

DMNS, MSU, TAMU

###### Mangora spiculata

Taxon classificationAnimaliaAraneaeAraneidae

(Hentz, 1847)

Mangora spiculata Jackman 1997: 161; Levi 1975a: 125, mf, desc. (figs 82–89); Levi 2005a: 164

####### Distribution.

East and south Texas; Hunt, Montgomery, Orange, Walker

####### Locality.

Jones State Forest

####### Time of activity.

Male (June, August); female (April, August)

####### Habitat.

(plants: vegetation)

####### Method.

sweeping [mf]

####### Type.

Alabama

####### Etymology.

Latin, a point

####### Collection.

MSU, TAMU

##### Genus *Mastophora* Holmberg, 1876

###### Mastophora alvareztoroi

Taxon classificationAnimaliaAraneaeAraneidae

Ibarra & Jiménez, 2003

Mastophora alvareztoroi Levi 2003: 360, mf, desc. (figs 296–307)

####### Distribution.

Hidalgo

####### Locality.

Santa Ana National Wildlife Refuge

####### Time of activity.

Female (December)

####### Type.

Mexico, Chiapas, Rancho Alejandria, Municipio Estacion Juarez

####### Etymology.

Person (The species was named after the collector, the late Miguel Alvarez del Toro, who dedicated his life to the study and protection of the Chiapas fauna and is the author of a book on Chiapas spiders, Levi 2003).

###### Mastophora cornigera

Taxon classificationAnimaliaAraneaeAraneidae

(Hentz, 1850)

Mastophora cornigera Gertsch 1955: 233, mf, desc. (figs 1–5, 37, 41–42); Jackman 1997: 161; Levi 2003: 344, mf, desc. (figs 169–182, 455); Liao et al. 1984: 410; Vogel 1970b: 4

####### Distribution.

Brazos, Cameron, Duval, Galveston, Hidalgo, Robertson, San Patricio, Travis, Wilson

####### Locality.

Frontera Audubon, Lick Creek Park, Santa Ana National Wildlife Refuge, Welder Wildlife Refuge

####### Time of activity.

Male (March – April, June – July, October – December); female (January – February, June – July, October, December)

####### Habitat.

(orchard: grapefruit); (soil/woodland: scrub live oak, *Quercus buckleyi*, *Quercus virginiana*, *Ulmus crassifolia*)

####### Method.

Beating [m]; beating/sweeping [m]; fogging [m]; sweeping [m]

####### Eggs/spiderlings.

Brazos [eggsac collected July 24, 2002, hatched week of August 26, 34 males, 65 immatures]; Cameron [62 males, 64 immatures, emerged June; 63 males, 64 immatures, emerged February; eggsac collected February 10, 1980, hatch March 15, 18 males, 25 immatures]; Hidalgo [59 males, 70 immatures, emerged April] [TAMU]

####### Type.

Alabama

####### Etymology.

Latin, horned

####### Collection.

TAMU

###### Mastophora leucabulba

Taxon classificationAnimaliaAraneaeAraneidae

(Gertsch, 1955)

Mastophora leucabulba Levi 2003: 358, mf, desc. (figs 288–295)
Agathostichus leucabulba Gertsch, 1955; Jackman 1997: 161
Agatostichus leucabulba Gertsch, 1955; Gertsch 1955: 250, f, desc. (figs 34, 38, 40); Roth 1982: 11–1; Roth 1985: B-6–3, B-6–8; Roth 1994: 68; Vogel 1967: 22; Vogel 1970b: 3

####### Distribution.

Cameron, Duval, Hidalgo, Wilson

####### Locality.

Santa Ana National Wildlife Refuge

####### Time of activity.

Male (April)

####### Type.

Texas (female, Cameron Co., E of Harlingen, January-March, 1936, L. I. Davis, holotype, AMNH)

####### Etymology.

Latin, white bulbous processes on carapace

####### Collection.

TAMU

###### Mastophora phrynosoma

Taxon classificationAnimaliaAraneaeAraneidae

Gertsch, 1955

Mastophora phrynosoma Bradley 2013: 96; Jackman 1997: 161; Levi 2003: 336, mf, desc. (figs 86–99, 449–450)

####### Distribution.

Walker

####### Locality.

Huntsville State Park

####### Time of activity.

Female (September)

####### Habitat.

(soil/woodland: bush, elm)

####### Type.

North Carolina, Burlington

####### Etymology.

Greek, toad-like

####### Collection.

TAMU

###### Mastophora stowei

Taxon classificationAnimaliaAraneaeAraneidae

Levi, 2003

Mastophora stowei Levi 2003: 334, mf, desc. (figs 63–74, 447)

####### Distribution.

Harrison

####### Time of activity.

Female (July)

####### Type.

Florida, Gainesville

####### Etymology.

Person (The species is named after the collector, Mark Stowe, who has contributed much to our knowledge of *Mastophora*, Levi 2003).

##### Genus *Mecynogea* Simon, 1903

###### Mecynogea lemniscata

Taxon classificationAnimaliaAraneaeAraneidae

(Walckenaer, 1841)

Mecynogea lemniscata Agnew et al. 1985: 4; Breene et al. 1993c: 12, 47, 105, mf (figs 158A-C); Dean and Sterling 1990: 404; Dean et al. 1982: 254; Jackman 1997: 80, desc., 161 (photo 21q); Knutson et al. 2010: 515; Levi 1980: 13 [S, T], mf, desc. (figs 1–15); Roth 1982: 11–2; Roth 1985: B-6–4, B-6–9; Roth 1994: 69, 72; Yantis 2005: 197; Young and Edwards 1990: 15
Allepeira lemniscata (Walckenaer, 1841); Exline 1948: 311
Epeira basilica McCook, 1878; McCook 1878: 133; McCook 1889: 164
Hentzia basilica (McCook, 1878); Comstock 1912: 417; Comstock 1940: 431; McCook 1893: 244; Petrunkevitch 1911: 349; Vogel 1970b: 5
Argiope basilica McCook, 1878; Marx 1890: 541
Allepeira basilica (McCook, 1878); Bryant 1940: 358; Roewer 1942: 778
Mecynogea basilica (McCook, 1878); Brown 1974: 232

####### Distribution.

Eastern 2/3 Texas; Archer, Bexar, Brazos, Burleson, Cameron, Comanche, Erath, Garza, Gonzales, Hidalgo, Houston, Howard, Hunt, Hutchinson, Nacogdoches, Sabine, Travis, Walker, Wichita

####### Locality.

5-Eagle Ranch, Bentsen-Rio Grande Valley State Park, Ellis Prison Unit, Johnson Ranch, Lake Tawakoni State Park, Lick Creek Park, Palmetto State Park, Proctor Lake, Santa Ana National Wildlife Refuge

####### Time of activity.

Male (May – July); female (May – August)

####### Habitat.

(crops: cotton, peanuts); (nest/prey: mud dauber nest [mf in *Chalybion californicum*, f in *Sceliphron caementarium*]); (soil/woodland: beech-magnolia forest, pine woods [%: 99], saltcedar, willow, woods); (web: in web, web in oak tree)

####### Method.

5 gallon bucket trap [m]; beating [m]; malaise trap [m]; suction trap [m]; sweeping [mf]

####### Type.

Georgia

####### Etymology.

Latin, adorned with ribbons

####### Collection.

MCZ, MSU, NMSU, TAMU

##### Genus *Metazygia* F. O. P.-Cambridge, 1904

###### Metazygia wittfeldae

Taxon classificationAnimaliaAraneaeAraneidae

(McCook, 1894)

Metazygia wittfeldae Armstrong and Richman 2007: 395; Breene et al. 1993c: 12, 47, 106, mf (figs 162A-C); Dean et al. 1982: 254; Guarisco 2008b: 5; Jackman 1997: 161; Jackman et al. 2007: 199; Kaston 1953: 190, desc. (fig. 467); Kaston 1972: 158, desc. (fig. 352); Kaston 1978: 150, desc. (fig. 376); Levi 1977a: 92, mf, desc. (figs 90–103); Levi 1995: 81; Rice 1986: 124; Young and Edwards 1990: 15

####### Distribution.

Central, southeast and south Texas; Brazos, Burnet, Cameron, Dallas, Fort Bend, Goliad, Hood, Hunt, Lee, Montgomery, San Patricio, Walker, Washington

####### Locality.

Ellis Prison Unit, Goliad State Park, Lake Buchanan, Lake Corpus Christi State Park, Lake Somerville State Park [Nails Creek Unit], Lake Tawakoni State Park, Lakeside Park South, Russell Farm

####### Time of activity.

Male (March – April, June – August); female (March – May, July – November)

####### Habitat.

(crops: cotton); (web: large spider web)

####### Method.

Boll weevil pheromone trap [mf]

####### Type.

Florida

####### Etymology.

Person (after the late Miss Anna Wittfeld, of Merrit Island, Florida)

####### Collection.

MSU, NMSU, TAMU

###### Metazygia zilloides

Taxon classificationAnimaliaAraneaeAraneidae

(Banks, 1898)

Metazygia zilloides Armstrong and Richman 2007: 395; Breene et al. 1993a: 169; Breene et al. 1993b: 647; Jackman 1997: 161; Levi 1977a: 92, mf, desc. (figs 104–111); Levi 1995: 86; Rice 1986: 124

####### Distribution.

Central and south Texas; Bell, Cameron, Hidalgo, Hunt, Lee, Montgomery, San Patricio, Starr, Travis, Willacy

####### Locality.

Bentsen-Rio Grande Valley State Park, Frontera Audubon, Lake Corpus Christi State Park, Lake Somerville State Park [Nails Creek Unit], Russell Farm, Sabal Palm Audubon Sanctuary

####### Time of activity.

Male (March, October, December); female (March – April, July – December)

####### Habitat.

(crops: sugarcane); (orchard: citrus, grapefruit, orange, sour orange); (web: large spider web)

####### Method.

Boll weevil pheromone trap [mf]

####### Type.

Mexico, Tepic

####### Etymology.

like *Zilla californica* Banks, 1896 = *Zygiella x-notata* (Clerck, 1758)

####### Collection.

DMNS, MSU, NMSU, TAMU

##### Genus *Metepeira* F. O. P.-Cambridge, 1903

###### Metepeira arizonica

Taxon classificationAnimaliaAraneaeAraneidae

Chamberlin & Ivie, 1942

Metepeira arizonica Jackman 1997: 161; Levi 1977b: 200, mf, desc. (figs 12–13, 39–46); Piel 2001: 66, mf, desc. (figs 206–212)

####### Distribution.

West Texas; Brewster, Presidio

####### Locality.

Big Bend National Park

####### Time of activity.

Female (March)

####### Type.

Arizona, Canyon Lake

####### Etymology.

locality (state)

####### Collection.

MSU

###### Metepeira comanche

Taxon classificationAnimaliaAraneaeAraneidae

Levi, 1977

Metepeira comanche Jackman 1997: 161; Knutson et al. 2010: 515; Levi 1977b: 204, mf, desc. (figs 61–69); Piel 2001: 62, mf, desc. (figs 185–191)
Metepeira n. sp.; Rogers and Horner 1977: 523

####### Distribution.

Widespread; Andrews, Archer, Bastrop, Baylor, Borden, Brewster, Collin, Crane, Gaines, Garza, Haskell, Howard, Jim Wells, Jones, Kent, Kimble, Kinney, Motley, Nacogdoches, Reagan, Taylor, Upton, Val Verde, Ward, Webb, Wichita

####### Locality.

Seminole Canyon State Park

####### Time of activity.

Male (February, April – July); female (February, May – July, September – November)

####### Habitat.

(crops: guar); (grass: grass); (plants: miscellaneous vegetation); (soil/woodland: juniper, saltcedar, tree, trees/shrubs); (web: in web)

####### Method.

Beating [mf]; sweeping [mf]

####### Type.

Texas (male, Haskell Co., 9.7 km W O’Brien, February 3, 1971, C. E. Rogers, holotype, MCZ)

####### Etymology.

Indian tribe (The name is a noun in apposition after the Indian tribe of the Texas plains, Levi 1977b).

####### Collection.

DMNS, MCZ, MSU, NMSU, TAMU

####### Note.

Levi 1977 lists Wells Co. but it is Jim Wells Co.

###### Metepeira foxi

Taxon classificationAnimaliaAraneaeAraneidae

Gertsch & Ivie, 1936

Metepeira foxi Dondale et al. 2003: 320, mf, desc. (figs 749–757); Jackman 1997: 161; Levi 1977b: 210, mf, desc. (figs 87–96)

####### Distribution.

West Texas; Hudspeth

####### Time of activity.

Female (May)

####### Type.

Utah, Richfield

####### Etymology.

Person

####### Collection.

MCZ

###### Metepeira labyrinthea

Taxon classificationAnimaliaAraneaeAraneidae

(Hentz, 1847)

Metepeira labyrinthea Agnew et al. 1985: 4; Bradley 2013: 97; Brown 1974: 232; Dean and Sterling 1990: 405; Dondale et al. 2003: 317, mf, desc. (figs 740–748); Gertsch 1939b: 25; Jackman 1997: 81, desc., 161; Levi 1977b: 196, mf, desc. (figs 1–11, 14–20); Piel 2001: 14, 17; Roberts 2001: 49; Young and Edwards 1990: 15
Metepeira labyrinthica (Hentz, 1847); Reddell 1965: 170; Vogel 1970b: 4

####### Distribution.

Widespread; Archer, Bosque, Brazos, Brewster, Brown, Comanche, Erath, Maverick, Nacogdoches, Potter, Sutton, Walker, Wichita

####### Locality.

Bill Haney Pecan Orchard, Chisos Mountains, Ellis Prison Unit, Lick Creek Park, Nabor’s Lake, Wildcat Bluff Nature Center

####### Caves.

**Sutton** (Felton Cave)

####### Time of activity.

Male (May – August); female (May – August, October)

####### Habitat.

(crops: peanuts); (landscape features: cave); (nest/prey: mud dauber nest [mf]); (orchard: pecan); (soil/woodland: trees, woods); (structures: indoors, porch); (web: in web, web in oak tree)

####### Method.

Beating [m]; fogging [f]; suction trap [m]

####### Type.

North Carolina and Alabama

####### Etymology.

Greek, type of web

####### Collection.

MSU, TAMU, TMM

###### Metepeira minima

Taxon classificationAnimaliaAraneaeAraneidae

Gertsch, 1936

Metepeira minima Armstrong and Richman 2007: 395; Gertsch 1936: 10, m, desc. (fig. 31); Jackman 1997: 161; Levi 1977b: 206, mf, desc. (figs 70–77); Piel 2001: 82, mf, desc. (figs 278–285); Roewer 1942: 869; Vogel 1970b: 4

####### Distribution.

South Texas; Bastrop, Cameron, Hidalgo, Kenedy

####### Locality.

Santa Ana National Wildlife Refuge

####### Time of activity.

Male (May); female (October – November)

####### Method.

Beating [f]; boll weevil pheromone trap [m]

####### Type.

Texas (male, Hidalgo Co., Edinburg, May 27, 1935, S. Mulaik, holotype, AMNH)

####### Etymology.

Latin, petite shape, small

####### Collection.

DMNS, TAMU

##### Genus *Micrathena* Sundevall, 1833

###### Micrathena gracilis

Taxon classificationAnimaliaAraneaeAraneidae

(Walckenaer, 1805)

Micrathena gracilis Agnew et al. 1985: 7; Bonnet 1957: 2868; Breene et al. 1993c: 12, 47, 100, mf (figs 144A-C); Brown 1974: 232; Dean et al. 1982: 254; Dondale et al. 2003: 146, mf, desc. (figs 299–306); Jackman 1997: 161; Jones 1936: 69; Levi 1978: 433, mf, desc. (figs 55–68); Taber and Fleenor 2005: 281 (fig. 12–11); Vogel 1970b: 5; Young and Edwards 1990: 15

####### Distribution.

Eastern ½ Texas; Aransas, Archer, Bastrop, Brazos, Burleson, Cameron, Comanche, Dallas, Erath, Goliad, Gonzalez, Grayson, Hardin, Harris, Houston, Jim Wells, Liberty, Montgomery, Nacogdoches, Red River, San Patricio, Travis, Walker (imm.), Wichita

####### Locality.

5-Eagle Ranch, Buescher State Park, Decker’s Prairie, Ellis Prison Unit, Goose Island State Park, Lick Creek Park, Nabor’s Lake, Palmetto State Park

####### Time of activity.

Male (May – July); female (January, May – November)

####### Habitat.

(crops: cotton); (grass: pasture); (littoral: along creek, creek bank, on tree fungus and marsh edge); (nest/prey: mud dauber nest [pen f] *Chalybion californicum*); (soil/woodland: forest, woods, *Quercus buckleyi*); (web: web by creek)

####### Method.

Beating/sweeping [mf]; sweeping [m]

####### Type.

Carolina (of 1805)

####### Etymology.

Latin, slender

####### Collection.

DMNS, MCZ, MSU, TAMU

###### Micrathena mitrata

Taxon classificationAnimaliaAraneaeAraneidae

(Hentz, 1850)

Micrathena mitrata Brown 1974: 232; Dondale et al. 2003: 148, mf, desc. (figs 307–313); Jackman 1997: 161; Levi 1978: 428, mf, desc. (figs 28–40); Levi 1985: 486

####### Distribution.

East Texas; Nacogdoches, Sabine

####### Time of activity.

Female (October)

####### Habitat.

(soil/woodland: beech-magnolia forest); (web: web near creek)

####### Method.

Malaise trap [f]

####### Type.

North Carolina and Alabama

####### Etymology.

Latin, abdomen above resembles a bishop’s mitre

####### Collection.

TAMU

###### Micrathena sagittata

Taxon classificationAnimaliaAraneaeAraneidae

(Walckenaer, 1841)

Micrathena sagittata Breene et al. 1993c: 12, 47, 100, mf (figs 145A-C); Brown 1974: 232; Dean et al. 1982: 254; Dondale et al. 2003: 143, mf, desc. (figs 291–298); Jackman 1997: 81, desc., 161 (photo 21a); Kaston 1972: 139, desc. (fig. 311); Kaston 1978: 133, desc. (fig. 334); Levi 1978: 430, mf, desc. (figs 41–54); Magalhaes and Santos 2012: 52; Taber and Fleenor 2005: 281 (fig. 12–10); Vogel 1970b: 5

####### Distribution.

Central, east and south Texas; Brazos, Cameron, Gonzales, Hardin, Hidalgo, Nacogdoches, Walker

####### Locality.

Bentsen-Rio Grande Valley State Park, Ellis Prison Unit, Frontera Audubon, Palmetto State Park, Resaca de la Palma State Park, Sabal Palm Audubon Sanctuary

####### Time of activity.

Male (June – August, October – November); female (April – May, August, October)

####### Habitat.

(crops: cotton); (littoral: near creek, wetlands); (nest/prey: mud dauber nest [imm.]); (orchard: grapefruit); (soil/woodland: palm forest, woods)

####### Method.

Beating [f]

####### Type.

Georgia

####### Etymology.

Latin, arrow- (head) like

####### Collection.

MSU, TAMU

##### Genus *Neoscona* Simon, 1864

###### Neoscona arabesca

Taxon classificationAnimaliaAraneaeAraneidae

(Walckenaer, 1841)

Neoscona arabesca Agnew et al. 1985: 4; Armstrong and Richman 2007: 395; Berman and Levi 1971: 474 [S, T], mf, desc. (figs 1–3, 5–6, 8, 10, 14–42, 125–126); Bonnet 1958: 3055; Breene et al. 1993a: 169; Breene et al. 1993b: 647; Breene et al. 1993c: 12, 47, 106, mf (figs 164A-C); Breene et al. 1994: 8; Brown 1974: 232; Bumroongsook et al. 1992: 17; Calixto et al. 2013: 181; Dean and Eger 1986: 141; Dean and Sterling 1987: 6; Dean and Sterling 1990: 405; Dean et al. 1982: 254; Dean et al. 1987: 268; Dean et al. 1988: 285–286; Dondale et al. 2003: 171, mf, desc. (figs 363–371); Hoffmaster 1985: 627; Jackman 1997: 82, desc., 161 (photo 21t); Li 1990: 137, 142, 144; Liao et al. 1984: 410; Nyffeler and Sterling 1994: 1295, 1298; Nyffeler et al. 1987a: 357; Nyffeler et al. 1989: 374, 377; Nyffeler et al. 1992a: 1181; Nyffeler et al. 1992c: 2; Rapp 1984: 5; Rice 1986: 124; Vogel 1970b: 4; Woods and Harrel 1976: 43; Young and Edwards 1990: 15
Epeira arabesca Walckenaer, 1841; McCook 1893: 148; Marx 1890: 542
Epeira trivittata Keyserling, 1864; Jones 1936: 70
Araneus trivittatus (Keyserling, 1864); Jones 1936: 70
Neoscona minima F. O. P.-Cambridge, 1904; Bonnet 1958: 3058; Brown 1974: 233; Gertsch and Mulaik 1936b: 20, m (fig. 30); Kagan 1942: 27; Kagan 1943: 258; Vogel 1970b: 4; Woods and Harrel 1976: 43
Aranea minima (F. O. P.-Cambridge, 1904); Roewer 1942: 847

####### Distribution.

Widespread; Atascosa, Bee, Brazoria, Brazos, Burleson, Cameron, Collin, Colorado, Comanche, Dallas, Erath, Falls, Fannin, Fayette, Galveston, Gillespie, Hays, Henderson, Hidalgo, Houston, Hunt, Jefferson, Jim Wells, Matagorda, McLennan, Montague, Nacogdoches, Polk, Rains, Robertson, San Patricio, Travis, Victoria, Walker, Webb, Wichita, Willacy, Williamson

####### Locality.

Adriance Pecan Orchard, Attwater Prairie Chicken National Wildlife Refuge, Bentsen-Rio Grande Valley State Park, Ellis Prison Unit, Galveston Island State Park, Holmes Pecan Orchard, Lake Corpus Christi State Park, Lake Tawakoni State Park, Lick Creek Park, Proctor Lake, Ramsey Prison Farm, Resaca de la Palma State Park, Russell Farm, Santa Ana National Wildlife Refuge, Texas A&M University Rangeland Area, Welder Wildlife Refuge

####### Time of activity.

Male (January – December); female (January – December)

####### Habitat.

(crops: corn, cotton, peanuts, rice, sugarcane, watermelon); (grass: grass, grassland, grassy and shrub area, pasture); (littoral: salt marsh area); (nest/prey: mud dauber nest [mf], nest of *Chalybion californicum*); (orchard: citrus, orange, pecan, tangerine, Valley lemon); (plants: bluebonnets, croton, garden, Indian paintbrush, miscellaneous vegetation, pepper, roadside vegetation, *Hibiscus* sp., *Monarda citriodora*); (soil/woodland: brushy area, hibiscus, mesquite, oak, post oak savanna); (structures: fence next to cotton field)

####### Method.

Beating [mf]; boll weevil pheromone trap [mf]; cardboard band [mf]; D-Vac suction [mf]; fogging [mf]; pitfall trap [f]; suction trap [mf]; sweeping [mf]

####### Type.

Georgia

####### Etymology.

Spanish, Arabic-like pattern

####### Collection.

DMNS, MSU, NMSU, TAMU

###### Neoscona crucifera

Taxon classificationAnimaliaAraneaeAraneidae

(Lucas, 1838)

Neoscona crucifera Calixto et al. 2013: 181; Dondale et al. 2003: 173, mf, desc, (figs 372–377); Guarisco 2008b: 5; Jackman 1997: 161; Jackman et al. 2007: 199; Knutson et al. 2010: 515; Levi 1993a: 231 [S]; Roberts 2001: 49; Yantis 2005: 197
Neoscona hentzii (Keyserling, 1864); Agnew et al. 1985: 7; Berman and Levi 1971: 478, mf, desc. (figs 51–58, 128); Hoffmaster 1985: 627

####### Distribution.

Widespread; Archer, Bastrop, Bell, Brazos, Brown, Clay, Comanche, Erath, Gillespie, Howard, Hunt, Leon, Nacogdoches, Potter, Presidio, Robertson, Runnels, San Patricio, Tarrant, Tom Green, Travis, Walker, Washington, Wheeler, Wichita

####### Locality.

Big Bend Ranch State Park, Holmes Pecan Orchard, Lake Tawakoni State Park, Riley Estate, Stubblefield Lake Recreation Area, Texas A&M University Rangeland Area, Welder Wildlife Refuge, Wildcat Bluff Nature Center

####### Time of activity.

Male (April, July – October); female (June – November)

####### Habitat.

(littoral: palmetto-cypress swamp); (orchard: pecan, pecan orchard); (soil/woodland: juniper, post oak woods [%: 90], saltcedar, wetland/woodland park); (structures: bedroom, outside house, under house eave); (web: in web, in web in woods, large spider web, on web in bosque, web under eave of house)

####### Method.

5 gallon bucket trap [f]; beating [mf]; black light trap [m]; cardboard band [f]; fogging [mf]; suction trap [f]; tile trap [m]

####### Eggs/spiderlings.

Comanche [eggsac laid June 1, 2001, hatched July 12; 533 spiderlings] [TAMU]

####### Type.

Canary Islands

####### Etymology.

Latin, cross-bearing

####### Collection.

DMNS, MSU, NMSU, TAMU

###### Neoscona domiciliorum

Taxon classificationAnimaliaAraneaeAraneidae

(Hentz, 1847)

Neoscona domiciliorum Berman and Levi 1971: 477, mf, desc. (figs 43–50, 127); Jackman 1997: 161; Kaston 1972: 157, desc. (fig. 350); Kaston 1978: 149, desc. (fig. 374); Woods and Harrel 1976: 43; Young and Edwards 1990: 15

####### Distribution.

Central and east Texas; Cameron, Hidalgo, Jefferson, Montgomery, Runnels, Travis, Wichita, Williamson

####### Locality.

Frontera Audubon

####### Caves.

**Williamson** (Williams Cave)

####### Time of activity.

Male (November); female (June, October – November)

####### Habitat.

(crops: rice); (grass: grass); (landscape features: cave); (littoral: flood plain); (orchard: grapefruit, Valley lemon); (soil/woodland: trees/shrubs)

####### Method.

Beating [f]

####### Type.

Alabama

####### Etymology.

Latin, refers to a house

####### Collection.

DMNS, MSU, TAMU

###### Neoscona nautica

Taxon classificationAnimaliaAraneaeAraneidae

(L. Koch, 1875)

Neoscona nautica Berman and Levi 1971: 498 [S], mf, desc. (figs 13, 111–120, 132); Jackman 1997: 161; Levi 1993a: 228
Epeira volucripes Keyserling, 1885; Marx 1890: 548

####### Distribution.

Central Texas; Galveston, Travis

####### Time of activity.

Male (September); female (August – September)

####### Habitat.

(structures: warehouse)

####### Type.

Sudan

####### Etymology.

Greek, for sailor

####### Collection.

MCZ, TAMU

###### Neoscona oaxacensis

Taxon classificationAnimaliaAraneaeAraneidae

(Keyserling, 1864)

Neoscona oaxacensis Berman and Levi 1971: 486 [S], mf, desc. (figs 4, 9, 11, 78–90, 129); Cokendolpher et al. 2008: 8, 16 (photo 14); Jackman 1997: 83, 161, desc.; Kaston 1972: 157, desc. (fig. 351); Kaston 1978: 149, desc. (fig. 375); Knutson et al. 2010: 515; Roberts 2001: 49; Rogers and Horner 1977: 523; Young and Edwards 1990: 15
Neoscona vertebrata (McCook, 1888); Kagan 1942: 26; Kagan 1943: 258

####### Distribution.

Western 2/3 Texas; Archer, Bastrop, Bell, Borden, Brazos, Brewster, Burleson, Carson, Clay, Coleman, Ector, Fisher, Guadalupe, Hidalgo, Howard, Hunt, Kendall, Lubbock, Martin, McLennan, Montague, Potter, Presidio, Randall, Reagan, Scurry, Upton, Ward, Wichita, Wilbarger

####### Locality.

Lake Thomas, Palo Duro Canyon State Park, Pantex Lake (edge), Wildcat Bluff Nature Center

####### Time of activity.

Male (June – October); female (February, June – December)

####### Habitat.

(crops: cotton, guar); (grass: grass, shrubs and tall grass); (orchard: pecan, pecan orchard); (littoral: near playa); (plants: roadside vegetation, vegetation, *Baccharis*); (soil/woodland: juniper, saltcedar, trees/shrubs)

####### Method.

Beating [m]; D-Vac suction [m]; sweeping [mf]

####### Type.

Mexico, Oaxaca

####### Etymology.

locality (Mexican state)

####### Collection.

DMNS, MSU, TAMU, TTU

###### Neoscona utahana

Taxon classificationAnimaliaAraneaeAraneidae

(Chamberlin, 1919)

Neoscona utahana Agnew et al. 1985: 7; Berman and Levi 1971: 485 [S], mf, desc. (figs 68–77, 135); Breene et al. 1993a: 169; Breene et al. 1993b: 647; Breene et al. 1993c: 13, 47, 106, mf (figs 163A-C); Dean and Sterling 1987: 6; Hoffmaster 1985: 627; Jackman 1997: 161; Young and Edwards 1990: 15
Neoscona eximia Gertsch & Mulaik, 1936; Bonnet 1958: 3058; Gertsch and Mulaik 1936b: 19, mf, desc. (fig. 32); Kagan 1942: 28; Kagan 1943: 258; Vogel 1970b: 4
Aranea eximia (Gertsch and Mulaik, 1936); Roewer 1942: 860

####### Distribution.

Widespread; Brazos, Cameron, Erath, Hidalgo, McLennan, Nueces, San Patricio, Travis, Walker, Winkler

####### Locality.

Bentsen-Rio Grande Valley State Park, Ellis Prison Unit, Welder Wildlife Refuge

####### Time of activity.

Male (July – August); female (June, August – December)

####### Habitat.

(crops: cotton, sugarcane); (orchard: citrus); (structures: under house eave)

####### Method.

suction trap [m]

####### Type.

Utah, Fillmore

####### Etymology.

locality (state)

####### Collection.

DMNS, TAMU

##### Genus *Ocrepeira* Marx, 1883

###### Ocrepeira ectypa

Taxon classificationAnimaliaAraneaeAraneidae

(Walckenaer, 1841)

Ocrepeira ectypa Jackman 1997: 161; Levi 1993b: 56 [T]
Wixia ectypa (Walckenaer, 1841); Bonnet 1959: 4828; Vogel 1970b: 4 [Levi 1976: 380 [S], mf, desc. (figs 88–100, 110, 113, 123)]
Wixia infumata (Hentz, 1850); Jones 1936: 70

####### Distribution.

Cameron, Dallas

####### Locality.

Sabal Palm Audubon Sanctuary

####### Time of activity.

Female (October)

####### Type.

Georgia

####### Etymology.

Greek, carved

####### Collection.

TAMU

###### Ocrepeira georgia

Taxon classificationAnimaliaAraneaeAraneidae

(Levi, 1976)

Ocrepeira georgia Jackman 1997: 161; Levi 1993b: 56 [T]
Wixia georgia Levi, 1976; Dean and Eger 1986: 141 [Levi 1976: 382, mf, desc. (figs 101–109, 111, 114, 124)]

####### Distribution.

Bandera, Brazos, Cameron, Hidalgo, Travis

####### Locality.

Bentsen-Rio Grande Valley State Park, Laguna Atascosa National Wildlife Refuge, Lost Maples State Park

####### Time of activity.

Male (April, May, October); female (April – May, October)

####### Habitat.

(plants: bluebonnets, Indian paintbrush); (soil/woodland: brushy area, savanna with native grasses, *Quercus buckleyi*, *Ulmus crassifolia*)

####### Method.

Beating [f]; sweeping [mf]

####### Type.

Georgia, Athens

####### Etymology.

locality (The specific name is a noun in apposition after the state of the type locality, Levi, 1976).

####### Collection.

TAMU

###### Ocrepeira globosa

Taxon classificationAnimaliaAraneaeAraneidae

(F. O. P.-Cambridge, 1904)

Ocrepeira globosa Jackman 1997: 161; Levi 1993b: 75 [T], f, desc. (figs 36–40)
Wixia globosa F. O. P.-Cambridge, 1904; Agnew et al. 1985: 7; Levi 1976: 382, f, desc. (figs 116–120)

####### Distribution.

Brown, Dallas, Erath

####### Time of activity.

Female (October – November)

####### Habitat.

(plants: vegetation)

####### Method.

suction trap [f]

####### Type.

Mexico, Guerrero, Tepetlapa

[male unknown]

####### Etymology.

Latin, globe or ball-like

####### Collection.

MCZ, MSU, TAMU

###### Ocrepeira redempta

Taxon classificationAnimaliaAraneaeAraneidae

(Gertsch & Mulaik, 1936)

Ocrepeira redempta Jackman 1997: 161; Levi 1993b: 84 [T], mf, desc. (figs 88–93)
Aranea redempta Gertsch & Mulaik, 1936; Gertsch and Mulaik 1936b: 18, f, desc. (fig. 39); Roewer 1942: 862
Araneus redemptus Gertsch & Mulaik, 1936; Bonnet 1955: 581; Vogel 1970b: 3
Neoscona redempta (Gertsch & Mulaik, 1936); Berman and Levi 1971: 499, f, desc. (figs 121–124)

####### Distribution.

Hidalgo

####### Time of activity.

Female (October)

####### Type.

Texas (female, Hidalgo Co., Edinburg, October 10, 1935, C. Rutherford, holotype, AMNH)

####### Etymology.

Latin, redeemed

##### Genus *Scoloderus* Simon, 1887

###### Scoloderus nigriceps

Taxon classificationAnimaliaAraneaeAraneidae

(O. P.-Cambridge, 1895)

Scoloderus nigriceps Bradley 2013: 100; Traw 1996: 64 [S], mf, desc. (figs 18–26)
Scoloderus cordatus (Taczanowski, 1879); Jackman 1997: 161; Levi 1976: 386, mf, desc. (figs 126–136); Roth 1982: 11–2; Roth 1985: B-6–3, B-6–8; Roth 1994: 68

####### Distribution.

Cameron, Hidalgo

####### Locality.

Santa Ana National Wildlife Refuge

####### Time of activity.

Female (February, April)

####### Type.

Mexico, Teapa

####### Etymology.

Latin, markings on abdomen

####### Collection.

TAMU

##### Genus *Singa* C. L. Koch, 1836

###### Singa eugeni

Taxon classificationAnimaliaAraneaeAraneidae

Levi, 1972

Singa eugeni [Levi 1972: 236, mf, desc. (figs 25–34)]

####### Distribution.

Jim Wells

####### Type.

Wisconsin, Iowa Co.

####### Etymology.

Person (The species is named after arachnologist Count Eugen Keyserling, Levi 1972).

####### Collection.

MSU

###### Singa keyserlingi

Taxon classificationAnimaliaAraneaeAraneidae

McCook, 1894

Singa keyserlingi [Levi 1972: 232, mf, desc. (figs 9–24)]

####### Distribution.

Bee

####### Type.

Missouri, St. Louis

####### Etymology.

Person (The species is named after arachnologist Count Eugen Keyserling)

####### Collection.

MSU

###### Singa

Taxon classificationAnimaliaAraneaeAraneidae

sp.

Singa Brown 1974: 233; Jones 1936: 70; Rogers and Horner 1977: 523

####### Distribution.

Nacogdoches, Rolling Plains

####### Habitat.

(crops: guar); (nest/prey: mud dauber nest [f])

##### Genus *Verrucosa* McCook, 1888

###### Verrucosa arenata

Taxon classificationAnimaliaAraneaeAraneidae

(Walckenaer, 1841)

Verrucosa arenata Brown 1974: 233; Jackman 1997: 161; Kaston 1978: 143, desc. (fig. 359); Levi 1976: 358, mf, desc. (figs 1–11); Lise et al. 2015: 11, mf, desc. (figs 8–38); Rapp 1984: 5; Roth 1982: 11–2; Roth 1985: B-6–6, B-6–11; Roth 1994: 69; Vogel 1970b: 4

####### Distribution.

Eastern ½ Texas; Bastrop, Brazos, Galveston, Gonzalez, Grayson, Nacogdoches, Sabine, San Jacinto, Tyler, Walker

####### Locality.

Buescher State Park, Galveston Island State Park, Kirby State Forest, Lick Creek Park, Palmetto State Park, Stubblefield Lake Recreation Area

####### Time of activity.

Male (May – August); female (May – July, September – November)

####### Habitat.

(littoral: near creek, salt marsh, sedge meadow); (nest/prey: mud dauber nest [mf]); (soil/woodland: beech-magnolia forest, tree, woods)

####### Method.

Beating [m]; beating/sweeping [f]; Lindgren funnel trap [m]; malaise trap [m]

####### Type.

Georgia

####### Etymology.

Latin, sandy

####### Collection.

DMNS, MSU, TAMU

##### Genus *Wagneriana* F. O. P.-Cambridge, 1904

###### Wagneriana tauricornis

Taxon classificationAnimaliaAraneaeAraneidae

(O. P.-Cambridge, 1889)

Wagneriana tauricornis Bradley 2013: 102; Jackman 1997: 161; Levi 1976: 370, mf, desc. (figs 57–73); Roth 1982: 11–2; Roth 1985: B-6–5, B-6–11; Roth 1994: 69

####### Distribution.

Southeast and south Texas; Brooks, Cameron, DeWitt, Hidalgo

####### Locality.

Lower Rio Grande Valley National Wildlife Refuge, Sabal Palm Audubon Sanctuary

####### Time of activity.

Male (July); female (September – November)

####### Habitat.

(plants: miscellaneous vegetation); (soil/woodland: ebony-guayacan association)

####### Method.

pitfall trap [f]; sweeping [m]

####### Type.

Guatemala

####### Etymology.

Latin, bull-horned

####### Collection.

TAMU

#### Family Caponiidae Simon, 1890

##### Genus *Orthonops* Chamberlin, 1924

###### Orthonops lapanus

Taxon classificationAnimaliaAraneaeCaponiidae

Gertsch & Mulaik, 1940

Orthonops lapanus Broussard and Horner 2006: 253; Gertsch and Mulaik 1940: 324, mf, desc. (fig. 16); Jackman 1997: 161; Platnick 1995: 15, mf, desc. (figs 36–38); Richman et al. 2011a: 46; Vogel 1967: 32; Vogel 1970b: 5
Orthonops gertschi Chamberlin, 1928; Gertsch 1935a: 31; Roewer 1942: 316; Vogel 1970b: 5 [Texas records]

####### Distribution.

Brewster, Hays, Hidalgo, Kerr, Presidio, Starr, Travis, Webb

####### Locality.

Big Bend National Park, Big Bend Ranch State Park, Chihuahuan desert, Chisos Basin, Chisos Mountains, Dalquest Research Site, La Mesa Ranch, Raven Ranch

####### Caves.

**Travis** (Dobie Shelter)

####### Time of activity.

Male (January, June, August – November); female (January – February, May – June, August – December)

####### Habitat.

(landscape features: under rock); (soil/woodland: *Juniperus* managed plot, leaf litter, upland deciduous forest)

####### Method.

Flight intercept trap on ground [mf]; pitfall trap [mf]

####### Type.

Texas (male, Starr Co., 3 miles E Rio Grande City, January 21, 1939, S. Mulaik, holotype, AMNH)

####### Etymology.

Latin, with shorter legs

####### Collection.

MSU, NMSU, TAMU, TMM

##### Genus *Tarsonops* Chamberlin, 1924

###### Tarsonops systematicus

Taxon classificationAnimaliaAraneaeCaponiidae

Chamberlin, 1924

Tarsonops systematicus Bond and Taylor 2013: 60; Comstock 1940: 305, desc.; Gertsch 1935a: 31, f (fig. 35); Gertsch and Mulaik 1940: 324; Jackman 1997: 161; Ubick 2005a: 76 (fig. 18.10); Vogel 1970b: 5 [Chamberlin 1924b: 601, f, desc. (fig. 37)]

####### Distribution.

Cameron, Hidalgo, Llano, Starr, Webb

####### Locality.

Laguna Atascosa National Wildlife Refuge

####### Time of activity.

Male (February – March); female (January – February, July, September – November)

####### Habitat.

(soil/woodland: dense coastal brush)

####### Method.

pitfall trap [m]

####### Type.

Mexico, Sonora, San Pedro Bay

####### Etymology.

Greek, systematic

####### Collection.

TAMU

#### Family Clubionidae Wagner, 1887

**Note.** Species incorrectly reported from Texas

*Clubiona johnsoni* Gertsch, 1941; Woods and Harrel 1976: 43; Young and Edwards 1990: 16 [not in Texas]

*Clubiona plumbi* Gertsch, 1941; Woods and Harrel 1976: 43; Young and Edwards 1990: 16 [not in Texas]

*Clubiona riparia* L. Koch, 1866; Woods and Harrel 1976: 43; Young and Edwards 1990: 16 [not in Texas]

##### Genus *Clubiona* Latreille, 1804

###### Clubiona abboti

Taxon classificationAnimaliaAraneaeClubionidae

L. Koch, 1866

Clubiona abboti Bonnet 1956: 1107; Breene et al. 1993c: 14, 47, 85, mf (figs 99A-B); Calixto et al. 2013: 181; Dean and Sterling 1987: 6; Dean and Sterling 1990: 405; Dondale and Redner 1982: 41, mf, desc. (figs 45–42–45, 47); Gertsch 1941b: 15, mf (figs 32–36); Henderson 2007: 61, 76, 79, 82; Jackman 1997: 161; Nyffeler et al. 1992c: 2; Vogel 1970b: 5
Clubiona abbotti L. Koch, 1866; Agnew et al. 1985: 4; Cokendolpher et al. 2008: 8, 16; Jones 1936: 69; Rapp 1984: 7; Young and Edwards 1990: 16
Clubiona abbotii abbotii L. Koch, 1866; Edwards 1958: 417, mf, desc. (figs 42–43, 83, 181–182, 236)
Clubiona abboti abboti L. Koch, 1866; Vogel 1970b: 5

####### Distribution.

Brazos, Brewster, Burleson, Carson, Colorado, Comal, Dallas, Delta, Erath, Floyd, Freestone, Galveston, Goliad, Harris, Jefferson, Kerr, Liberty, Nueces, Orange, Robertson, Travis, Walker, Wichita

####### Locality.

Attwater Prairie Chicken National Wildlife Refuge, Ellis Prison Unit, Holmes Pecan Orchard, Lick Creek Park, Zilker Park

####### Time of activity.

Male (January, May – December); female (February, April – December)

####### Habitat.

(crops: cotton, peanuts); (grass: grass, grassland, grassy and shrub area); (littoral: near playa, near water); (orchard: pecan); (plants: *Monarda citriodora*); (soil/woodland: post oak woodland)

####### Method.

cardboard band [mf]; D-Vac suction [mf]; fogging [f]; pitfall trap [mf]; suction trap [m]; sweeping

####### Type.

Maryland, Baltimore

####### Etymology.

Person (naturalist)

####### Collection.

DMNS, MSU, TAMU

###### Clubiona adjacens

Taxon classificationAnimaliaAraneaeClubionidae

Gertsch & Davis, 1936

Clubiona adjacens Bonnet 1956: 1108; Edwards 1958: 408, m, desc. (figs 54–55, 160); Gertsch 1941b: 8, m (figs 30–31); Gertsch and Davis 1936: 19, m, desc. (fig. 35); Jackman 1997: 161; Roewer 1955: 513; Vogel 1970b: 5

####### Distribution.

Cameron

####### Time of activity.

Male (May)

####### Type.

Texas (male, Cameron Co., May 1–2, 1936, L. I. Davis, holotype, AMNH)

[female unknown]

####### Etymology.

Latin, species closely related to *Clubiona abboti* L. Koch, 1866

###### Clubiona catawba

Taxon classificationAnimaliaAraneaeClubionidae

Gertsch, 1941

Clubiona catawba Dean and Eger 1986: 142; Dean and Sterling 1990: 405; Dean et al. 1988: 287; Dondale and Redner 1982: 53, mf, desc. (figs 68–71); Edwards 1958: 426, mf, desc. (figs 76–77, 92, 194, 244); Jackman 1997: 162; Vogel 1970b: 5

####### Distribution.

Brazos, Burleson, Cameron, Colorado, Gillespie, Goliad, Houston, Starr, Travis, Victoria, Walker

####### Locality.

Attwater Prairie Chicken National Wildlife Refuge, Ellis Prison Unit, Lick Creek Park, South Padre Island

####### Time of activity.

Male (May – October); female (January, April, August)

####### Habitat.

(grass: dunes, grassland, pasture); (plants: bluebonnets); (soil/woodland: forest, live oak forest, post oak savanna, post oak savanna with pasture)

####### Method.

pitfall trap [m]; suction trap [m]; sweeping [mf]

####### Type.

Tennessee, Kingston

####### Etymology.

Indian tribe

####### Collection.

MSU, TAMU

###### Clubiona kagani

Taxon classificationAnimaliaAraneaeClubionidae

Gertsch, 1941

Clubiona kagani Dean and Sterling 1990: 405; Edwards 1958: 425, f, desc. (figs 89, 200, 243); Gertsch 1941b: 6, f, desc. (fig. 16); Irungu 2007: 30; Jackman 1997: 162; Kagan 1942: 57 (desc.); Kagan 1943: 258; Roewer 1955: 515; Vogel 1967: 34; Vogel 1970b: 5; Young and Edwards 1990: 16

####### Distribution.

Harris, Hidalgo, McLennan, Walker

####### Locality.

Ellis Prison Unit

####### Time of activity.

Female (March 30-April 5, July)

####### Habitat.

(crops: cabbage, cotton)

####### Method.

pitfall trap [f]; suction trap [f]

####### Type.

Texas (female, McLennan Co., Riesel, July 26, 1940, M. Kagan, holotype, AMNH)

[male unknown]

####### Etymology.

Person (collector)

####### Collection.

TAMU

###### Clubiona kiowa

Taxon classificationAnimaliaAraneaeClubionidae

Gertsch, 1941

Clubiona kiowa Edwards 1958: 428, mf, desc. (figs 62–63, 90, 186, 245); Gertsch 1941b: 12, m, desc. (figs 23–24); Jackman 1997: 162; Pfannenstiel 2008a: 204; Roewer 1955: 515; Vogel 1967: 35; Vogel 1970b: 5

####### Distribution.

Cameron, Colorado, Dallas, Hidalgo

####### Locality.

Attwater Prairie Chicken National Wildlife Refuge

####### Time of activity.

Male (April 28-May 5, June – August); female (April 28-May 5, May – June, August – September)

####### Habitat.

(crops: cotton, soybean); (orchard: grapefruit, sour orange, tangerine)

####### Method.

pitfall trap [mf]; sweeping [m]

####### Type.

Texas (male, Dallas Co., Dallas, 1936, J. H. Robinson, holotype, AMNH)

####### Etymology.

Indian tribe

####### Collection.

TAMU

###### Clubiona maritima

Taxon classificationAnimaliaAraneaeClubionidae

L. Koch, 1867

Clubiona maritima Dondale and Redner 1982: 35, mf, desc. (figs 38–41); Edwards 1958: 432 [S], mf, desc. (figs 131–133, 139, 180, 214); Jackman 1997: 162; Jones 1936: 69; Pfannenstiel 2008a: 204; Vogel 1970b: 5
Clubiona transversa Bryant, 1936; Bonnet 1956: 1161; Bryant 1936: 97, f, desc. (fig. 8); Jones 1936: 69; Roewer 1955: 518

####### Distribution.

Archer, Cameron, Dallas, Hidalgo

####### Locality.

White Rock Lake

####### Time of activity.

Male (June); female (March, June)

####### Habitat.

(crops: cotton)

####### Type.

Virgin Islands, St. Thomas

####### Etymology.

Latin, maritime

####### Collection.

MCZ, MSU, TAMU

###### Clubiona pygmaea

Taxon classificationAnimaliaAraneaeClubionidae

Banks, 1892

Clubiona pygmaea Dondale and Redner 1982: 34, mf, desc. (figs 34–37); Edwards 1958: 392, mf, desc. (figs 97–98, 135, 173, 226); Jackman 1997: 162; Vogel 1970b: 6

####### Distribution.

Hidalgo

####### Type.

New York, Ithaca, Fall Creek

####### Etymology.

Latin, pygmy

##### Genus *Elaver* O. P.-Cambridge, 1898

###### Elaver chisosa

Taxon classificationAnimaliaAraneaeClubionidae

(Roddy, 1966)

Elaver chisosa Brescovit et al. 1994: 36 [T]; Jackman 1997: 162
Clubionoides chisosa Roddy, 1966; Roddy 1966: 401, f, desc. (fig. 5); Vogel 1967: 36; Vogel 1970b: 6

####### Distribution.

Brewster

####### Locality.

Big Bend National Park, Chisos Basin, Chisos Mountains

####### Time of activity.

Female (September)

####### Type.

Texas (female, Brewster Co., Big Bend National Park, Chisos Mountains, September 28, 1950, W. J. Gertsch, holotype, AMNH)

[male unknown]

####### Etymology.

locality (Chisos Mountains)

###### Elaver dorotheae

Taxon classificationAnimaliaAraneaeClubionidae

(Gertsch, 1935)

^1^

Elaver dorotheae Brescovit et al. 1994: 36 [T, spelling]; Jackman 1997: 162
Clubiona dorothea Gertsch, 1935; Roewer 1955: 514
Clubiona dorotheae Gertsch, 1935; Bonnet 1956: 1122; Gertsch 1935b: 12, f, desc. (fig. 25)
Clubionoides dorothea (Gertsch, 1935); Edwards 1958: 381, mf, desc. (figs 22, 34–36, 208); Vogel 1970b: 6

####### Distribution.

Hidalgo

####### Time of activity.

Female (“September-December”)

####### Type.

Texas (female, Hidalgo Co., Edinburg, September-December 1933, S. Mulaik, holotype, AMNH)

####### Etymology.

Person (first name of collector’s wife, Dorothea)

###### Elaver excepta

Taxon classificationAnimaliaAraneaeClubionidae

(L. Koch, 1866)

Elaver excepta Brescovit et al. 1994: 36 [T]; Calixto et al. 2013: 181; Jackman 1997: 162; Yantis 2005: 200
Clubionoides excepta (L. Koch, 1866); Agnew et al. 1985: 8; Brown 1974: 233; Dondale and Redner 1982: 98, mf, desc. (figs 7, 178–181); Edwards 1958: 377, mf, desc. (figs 19, 31–33, 211); Vogel 1970b: 6
Elaver expecta (L. Koch, 1866); Trevino 2014: 11

####### Distribution.

Bell, Brazos, Cameron, Comal, Denton, Erath, Gonzales, Harris, Hidalgo, Hunt, Kaufman, Kerr, Madison, Nacogdoches, Robertson, Sabine, Walker, Webb

####### Locality.

Holmes Pecan Orchard, Huntsville State Park, Lake Tawakoni State Park, Lick Creek Park, Parson’s Slough, Riley Estate

####### Time of activity.

Male (March – July, July 24-August 6, September – October); female (January, March – October)

####### Habitat.

(grass: short grass, sandy-prairie grass, tall grass prairie); (littoral: sedge meadow); (orchard: pecan); (soil/woodland: beech magnolia forest, leaf litter, old field, post oak woods [%: 76], sandy area, sandy by water, tree, upland deciduous forest); (structures: bedroom ceiling, on [wall, wall in house])

####### Method.

5 gallon bucket trap [f]; beating [f]; cardboard band [mf]; flight intercept trap on ground [m]; malaise trap [mf]; pitfall trap [m]

####### Type.

Maryland, Baltimore

####### Etymology.

Latin, to exclude

####### Collection.

DMNS, MCZ, MSU, TAMU

###### Elaver mulaiki

Taxon classificationAnimaliaAraneaeClubionidae

(Gertsch, 1935)

Elaver mulaiki Brescovit et al. 1994: 37 [T]; Jackman 1997: 162
Clubiona mulaiki Gertsch, 1935; Bonnet 1956: 1135; Gertsch 1935b: 11, mf, desc. (figs 22–24); Roewer 1955: 516
Clubionoides mulaiki (Gertsch, 1935); Edwards 1958: 379, mf, desc. (figs 20, 24–26, 207); Vogel 1970b: 6

####### Distribution.

Cameron, Hidalgo, Starr

####### Locality.

Sabal Palm Audubon Sanctuary

####### Time of activity.

Male (September); female (February)

####### Habitat.

(orchard: grapefruit)

####### Type.

Texas (female, Hidalgo Co., 7 miles E Edinburg, February 8, 1935, S. Mulaik, holotype, AMNH)

####### Etymology.

Person (collector)

####### Collection.

TAMU

###### Elaver texana

Taxon classificationAnimaliaAraneaeClubionidae

(Gertsch, 1933)

Elaver texana Brescovit et al. 1994: 37 [T]; Jackman 1997: 162
Clubiona texana Gertsch, 1933; Bonnet 1956: 1160; Gertsch 1933c: 7, f, desc. (fig. 16); Roewer 1955: 517
Clubionoides texana (Gertsch, 1933); Edwards 1958: 380, mf, desc. (figs 23, 27–30, 210); Rapp 1984: 7; Vogel 1970b: 6

####### Distribution.

Cameron, Galveston, Hidalgo, Nueces, Starr

####### Locality.

Frontera Audubon, Laguna Madre

####### Time of activity.

Male (October); female (January)

####### Habitat.

(orchard: grapefruit, orange)

####### Type.

Texas (female, Cameron Co., Brownsville, January 3–11, 1928, Lutz, holotype, AMNH)

####### Etymology.

locality (state)

####### Collection.

TAMU

#### Family Corinnidae Karsch, 1880

**Note.** *Phrurolithus*, *Phruronellus*, *Phrurotimpus* and *Scotinella* transferred to Phrurolithidae (Ramírez 2014: 342). *Meriola* and *Trachelas* transferred to Trachelidae (Ramírez 2014: 342).

**Note.** Species incorrectly reported from Texas

*Castianeira cingulata* (C. K. Koch, 1842) [not in Texas]

*Thargalia zonoria* Hentz, 1847; Marx 1890: 514 [not in Texas]

##### Genus *Castianeira* Keyserling, 1879

###### Castianeira alteranda

Taxon classificationAnimaliaAraneaeCorinnidae

Gertsch, 1942

Castianeira alteranda Agnew et al. 1985: 4, 10; Jackman 1997: 162; Young and Edwards 1990: 16 [Reiskind 1969: 206, mf, desc. (figs 66–69, 83)]

####### Distribution.

Brazos, Coryell, Erath, Knox, Williamson

####### Locality.

Stiles Farm Foundation, Texas A&M University Rangeland Area

####### Time of activity.

Male (May, July – September); female (May, July – August, September 28-October 5, October)

####### Habitat.

(crops: cotton, peanuts); (soil/woodland: post oak savanna with pasture); (structures: indoors)

####### Method.

pitfall trap [mf]

####### Eggs/spiderlings.

Erath [29 eggs in eggsac] [TAMU]

####### Type.

Montana, Hamilton

####### Etymology.

Latin, similar in coloration and general appearance to *Castianeira amoena* (C. L. Koch, 1841)

####### Collection.

FSCA, MSU, TAMU

###### Castianeira amoena

Taxon classificationAnimaliaAraneaeCorinnidae

(C. L. Koch, 1841)

Castianeira amoena Agnew et al. 1985: 4; Broussard and Horner 2006: 253; Calixto et al. 2013: 181; Jackman 1997: 112, 162; Kaston 1972: 227, desc. (fig. 511); Kaston 1978: 218, desc. (fig. 557); Petrunkevitch 1911: 452; Reiskind 1969: 204 [S], mf, desc. (figs 70–73, 84–85); Richman et al. 2011a: 47; Yantis 2005: 66, 196, 199; Young and Edwards 1990: 16
Thargalia amoena C. L. Koch, 1847; Marx 1890: 513

####### Distribution.

Eastern 2/3 Texas; Brazos, Comanche, Cooke, Coryell, Erath, Grimes, Hidalgo, Houston, Leon, Madison, Parker, Presidio, Robertson, Travis, Uvalde

####### Locality.

Bill Haney Pecan Orchard, Chihuahuan desert, Dalquest Research Site, Holmes Pecan Orchard, Lick Creek Park, Riley Estate

####### Time of activity.

Male (June – September, December); female (July – November)

####### Habitat.

(crops: peanuts); (grass: grass); (orchard: pecan); (soil/woodland: leaf litter, pine woods [%: 74], post oak savanna with pasture, post oak woods [%: 60, 76, 80, 100], sandy area, woods, *Ulmus crassifolia*); (structures: in building, indoors)

####### Method.

5 gallon bucket trap [mf]; pitfall trap [mf] (in sand [m]) sweeping [f]; tile trap [m]

####### Eggs/spiderlings.

Erath [21 eggs in eggsac] [TAMU]

####### Type.

Carolina (of 1841)

####### Etymology.

Latin, lovely

####### Collection.

MSU, NMSU, TAMU

###### Castianeira crocata

Taxon classificationAnimaliaAraneaeCorinnidae

(Hentz, 1847)

Castianeira crocata Breene et al. 1993c: 13, 47, 83, mf (figs 92A-B); Jackman 1997: 112, 162; Reiskind 1969: 200, mf, desc. (figs 44–45, 56); Roberts 2001: 50 [male probably floridana (Banks, 1904)]; [Reiskind 1969: 201, m, desc. (figs 42–43, 59); page 200 – male of Castianeira floridana probably very close to crocata]; [Reiskind 1981: 173, m (fig. 3)]

####### Distribution.

Southeast and south Texas; Brazos, Burleson, Cameron, Clay, Colorado, Coryell, Kenedy, Lubbock, Montague, Potter, Williamson

####### Locality.

Attwater Prairie Chicken National Wildlife Refuge, Kenedy Ranch, Laguna Atascosa National Wildlife Refuge, Stiles Farm Foundation, Wildcat Bluff Nature Center

####### Time of activity.

Male (July 28-August 8); female (April – August)

####### Habitat.

(crops: cotton); (soil/woodland: post oak savanna with pasture)

####### Method.

pitfall trap [mf]

####### Type.

Alabama

####### Etymology.

Latin, saffron-yellow

####### Collection.

MSU, TAMU, TTU

###### Castianeira cubana

Taxon classificationAnimaliaAraneaeCorinnidae

(Banks, 1926)

Castianeira cubana Jackman 1997: 162; Reiskind 1969: 247 [S], mf, desc. (figs 216–219, 276)
Myrmecotypus cubanus Banks, 1926; Bonnet 1957: 3020; Bryant 1933: 190, f, desc. (pl. 4, figs 43, 45); Bryant 1940: 445; Comstock 1940: 592; Roewer 1955: 634

####### Distribution.

Cameron, Kenedy

####### Locality.

Kenedy Ranch, Laguna Atascosa National Wildlife Refuge

####### Time of activity.

Male (April); female (October)

####### Habitat.

(littoral: dense coastal brush, sand dune under live oak)

####### Method.

Beating [f]; yellow pan trap [m]

####### Type.

Cuba, Soledad

####### Etymology.

locality (country)

####### Collection.

TAMU

###### Castianeira descripta

Taxon classificationAnimaliaAraneaeCorinnidae

(Hentz, 1847)

Castianeira descripta Agnew et al. 1985: 4; Breene et al. 1993b: 647; Calixto et al. 2013: 181; Cokendolpher et al. 2008: 8, 16 (photo 15); Dondale and Redner 1982: 114, mf, desc. (figs 211–217); Jackman 1997: 112, desc., 162 (photo 34d); Kaston 1972: 226, desc. (fig. 508); Kaston 1978: 217, desc. (fig. 554); Reiskind 1969: 208, mf, desc. (figs 88–91, 121); Trevino 2014: 11; Vogel and Durden 1972: 1; Young and Edwards 1990: 16

####### Distribution.

East, central, and south Texas; Archer, Brazos, Brown, Burleson, Cameron, Carson, Comanche, Coryell, Erath, Hays, Hidalgo, Kerr, Knox, Robertson, Tom Green (imm.), Travis, Webb, Wichita

####### Locality.

Bill Haney Pecan Orchard, Holmes Pecan Orchard, NK Ranch, Pantex Lake (edge), Texas A&M University Rangeland Area

####### Time of activity.

Male (March – September); female (April – October)

####### Habitat.

(crops: cotton, peanuts, sugarcane); (landscape features: rocks); (littoral: near playa); (orchard: pecan); (plants: miscellaneous vegetation); (soil/woodland: post oak savanna with pasture, sandy area); (structures: indoors, in lab)

####### Method.

pitfall trap [mf] (in sand [f]); ramp trap [f]; sweeping [f]; tile trap [f]

####### Type.

North Carolina

####### Etymology.

Latin, descriptive

####### Collection.

AMNH, DMNS, MSU, TAMU, TTU

###### Castianeira gertschi

Taxon classificationAnimaliaAraneaeCorinnidae

Kaston, 1945

Castianeira gertschi Breene et al. 1993c: 13, 47, 83, mf (figs 93A-C); Dean et al. 1982: 255; Dondale and Redner 1982: 109, mf, desc. (figs 196–200); Jackman 1997: 162; Kaston 1972: 226, desc. (fig. 510); Kaston 1978: 218, desc. (fig. 556); Reiskind 1969: 217, mf, desc. (figs 104–107, 120); Young and Edwards 1990: 16

####### Distribution.

South Texas; Brazos, Hunt, Walker

####### Locality.

Ellis Prison Unit

####### Time of activity.

Male (July); female (February)

####### Habitat.

(crops: cotton)

####### Method.

pitfall trap [m]

####### Type.

Connecticut, Indian Neck

####### Etymology.

Person (arachnologist)

####### Collection.

TAMU

###### Castianeira longipalpa

Taxon classificationAnimaliaAraneaeCorinnidae

(Hentz, 1847)

Castianeira longipalpa Calixto et al. 2013: 181; Cokendolpher et al. 2008: 16; Irungu 2007: 30; Jackman 1997: 112, 162; Platnick 2000 [spelling]; Trevino 2014: 11; Yantis 2005: 66, 196, 199
Castianeira longipalpus (Hentz, 1847); Agnew et al. 1985: 4; Breene et al. 1993c: 13, 47, 84, mf (figs 94A-C); Dean et al. 1982: 255; Reiskind 1969: 186, mf, desc. (figs 7–10, 50–53); Young and Edwards 1990: 16

####### Distribution.

South Texas; Anderson, Archer, Bee, Bexar, Brazos, Burleson, Colorado, Comanche, Cooke, Coryell, Erath, Goliad, Hidalgo, Houston, Jeff Davis, Kenedy, Leon, Lubbock, Robertson, Walker, Webb, Wichita

####### Locality.

Attwater Prairie Chicken National Wildlife Refuge, Bentsen-Rio Grande Valley State Park, Ellis Prison Unit, Goliad State Park, Holmes Pecan Orchard, Kenedy Ranch, Nabor’s Lake, Somerville Lake, Texas A&M University Rangeland Area

####### Time of activity.

Male (March – August, October); female (May – August, October – November)

####### Habitat.

(crops: cabbage, cotton, peanuts); (grass: pasture); (littoral: near playa, sand dune area); (orchard: pecan); (plants: Compositae); (soil/woodland: pine woods [%: 60, 69, 74, 84], post oak savanna with pasture, post oak woods [%: 41, 92], sand dune area, sandy area); (structures: in [building, lab])

####### Method.

5 gallon bucket trap [mf]; pitfall trap [mf] (in sand [m]); ramp trap [m]; tile trap [m]

####### Type.

Alabama

####### Etymology.

Latin, long neck on palp

####### Collection.

FSCA, MSU, TAMU

###### Castianeira nanella

Taxon classificationAnimaliaAraneaeCorinnidae

Gertsch, 1933

Castianeira nanella Broussard and Horner 2006: 253; Richman et al. 2011a: 48 [Reiskind 1969: 225, mf, desc. (figs 129–132, 149–150)]

####### Distribution.

Brewster, Presidio

####### Locality.

Chihuahuan desert, Dalquest Research Site

####### Method.

pitfall trap [mf]

####### Type.

Utah, Salt Lake City, City Creek Canyon

####### Etymology.

Greek, dwarfish

####### Collection.

MSU

###### Castianeira occidens

Taxon classificationAnimaliaAraneaeCorinnidae

Reiskind, 1969

Castianeira occidens Agnew et al. 1985: 4; Broussard and Horner 2006: 253; Jackman 1997: 162; Richman et al. 2011a: 48; Trevino 2014: 11; Young and Edwards 1990: 16 [Reiskind 1969: 211, mf, desc. (figs 96–99, 113–115)]

####### Distribution.

Brewster, Erath, Presidio, Webb, Wichita

####### Locality.

Chihuahuan desert, Dalquest Research Site

####### Time of activity.

Male (September); female (March)

####### Habitat.

(crops: peanuts); (landscape features: under rock)

####### Method.

pitfall trap [mf]

####### Type.

Arizona, Lakeside

####### Etymology.

noun, the West (The specific name is a noun in apposition meaning the West, Reiskind 1969).

####### Collection.

FSCA, MSU

###### Castianeira peregrina

Taxon classificationAnimaliaAraneaeCorinnidae

(Gertsch, 1935)

Castianeira peregrina Jackman 1997: 162; Reiskind 1969: 251 [T], f, desc. (fig. 207)
Mazax peregrina Gertsch, 1935; Bonnet 1957: 2741; Gertsch 1935b: 15, f, desc. (fig. 30) [not male]; Vogel 1970b: 6
Apochinomma peregrinum (Gertsch, 1935); Roewer 1955: 608

####### Distribution.

Cameron, Hidalgo

####### Time of activity.

Female (February – March, November)

####### Type.

Texas (female, Hidalgo Co., 5 miles S San Juan, February 22, 1935, S. Mulaik, holotype, AMNH)

[male unknown]

####### Etymology.

Latin, pilgrim

###### Castianeira trilineata

Taxon classificationAnimaliaAraneaeCorinnidae

(Hentz, 1847)

Castianeira trilineata Agnew et al. 1985: 8; Dondale and Redner 1982: 104, mf, desc. (figs 182–186); Henderson 2007: 58, 76, 79, 82; Jackman 1997: 162 (photo 34a); Reiskind 1969: 219, mf, desc. (figs 108–110, 119)

####### Distribution.

Central and southeast Texas; Brazos, Coryell, Erath, Hidalgo, Hunt, Montgomery, Robertson

####### Locality.

Holmes Pecan Orchard, Lick Creek Park

####### Time of activity.

Male (March – June, October); female (April – July, September)

####### Habitat.

(grass: grass); (soil/woodland: disturbed habitat, leaf litter, post oak savanna with pasture, sandy area, woods); (structures: on floor in lab, sink in house)

####### Method.

pitfall trap [mf]

####### Type.

Alabama

####### Etymology.

Latin, three horizontal light bands on abdomen

####### Collection.

MSU, TAMU

##### Genus *Falconina* Brignoli, 1985

###### Falconina gracilis

Taxon classificationAnimaliaAraneaeCorinnidae

(Keyserling, 1891)

Falconina gracilis Bonaldo 2000: 79, mf, desc. (figs 36–38, 41–42, 63, 101, 215–228); Calixto et al. 2013: 181, 188–189; Henderson 2007: 61, 63, 65–66, 76, 79, 82; Irungu 2007: 30; Reddell and Cokendolpher 2004: 77; Trevino 2014: 11; Ubick and Richman 2005a: 82; Yantis 2005: 200
Corinna sp.; Jackman 1997: 162

####### Distribution.

Bexar, Brazoria, Brazos, Burleson, Colorado, Coryell, Fayette, Fort Bend, Goliad, Grimes, Harris, Hays, Henderson, Hidalgo, Polk, Robertson, San Patricio, Travis, Victoria, Washington, Webb, Williamson

####### Locality.

5-Eagle Ranch, Attwater Prairie Chicken National Wildlife Refuge, Brazos Bend State Park, Goliad State Park, Holmes Pecan Orchard, Lick Creek Park, NK Ranch, Somerville Lake, Stiles Farm Foundation, Welder Wildlife Refuge

####### Caves.

**Bexar** (Crownridge Canyon Cave); **Travis** (Five Pocket Cave)

####### Time of activity.

Male (January, January 26-February 22, March – November); female (January, March – November)

####### Habitat.

(crops: cabbage, cotton); (grass: grassland); (landscape features: cave); (nest/prey: pocket gopher burrows); (orchard: pecan); (soil/woodland: buckeye-sycamore forest, *Juniperus* unmanaged plot, open field, post oak savanna with pasture, post oak woodland, post oak woods [%: 60], sandy area, upland woods); (structures: around house, bathroom floor, in house, indoors, on floor in house)

####### Method.

5 gallon bucket trap [imm.]; cardboard band [mf]; flight intercept trap [m]; flight intercept trap on ground [m]; pitfall trap [mf]; tile trap [m]

####### Type.

Brazil, Rio Grande do Sul

####### Etymology.

Latin, slender

####### Collection.

TAMU, TMM

####### Note.

Ubick and Richman (2005a) noted that this species has been associated with *Solenopsis invicta* Buren, red imported fire ant, in Texas (page 82, Cokendolpher, pers. comm.). In a study in a post oak savanna with pasture habitat by Calixto (2008), a yearly total of *Falconina* and ants indicates that *Falconina* was most abundant in pitfall traps that contained the most *Solenopsis invicta*. Also the following ant genera were most abundant in traps with *Falconina gracilis*: *Diplorhoptrum*, *Forelius*, *Monomorium*, and *Paratrechina*. Two genera of ants, *Brachymyrmex* and *Strumigenys*, were only found in traps that contained the most *Forelius gracilis*. Both *Strumigenys invicta* and *Forelius gracilis* were more abundant in 2006 than 2007 at all three locations. More rain occurred in 2007 than 2006. Thanks to Alejandro Calixto for identifying the ants.

##### Genus *Mazax* O. P.-Cambridge, 1898

###### Mazax kaspari

Taxon classificationAnimaliaAraneaeCorinnidae

Cokendolpher, 1978

Mazax kaspari Cokendolpher 1978b: 230, mf, desc. (figs 1–7); Jackman 1997: 162; Ubick and Richman 2005a: 80

####### Distribution.

Presidio

####### Time of activity.

Male (March); female (March)

####### Habitat.

(grass: grass along river)

####### Type.

Texas (male, Presidio Co., 4 km W Lajitas, March 28, 1975, T. C. Kaspar, holotype, AMNH)

####### Etymology.

Person (The specific name is in honor of the biologist Mr . T. C . Kaspar, who collected the type specimens, Cokendolpher 1978b).

###### Mazax pax

Taxon classificationAnimaliaAraneaeCorinnidae

Reiskind, 1969

Mazax pax Jackman 1997: 162; Reiskind 1969: 264 [S], mf, desc. (figs 233–236, 285); Roth 1982: 13–2; Ubick and Richman 2005a: 80
Mazax spinosa O. P.-Cambridge, 1898; Comstock 1940: 592

####### Distribution.

Cameron, Hidalgo, Starr

####### Locality.

Sabal Palm Audubon Sanctuary

####### Time of activity.

Male (September)

####### Habitat.

(soil/woodland: palm forest)

####### Method.

Flight intercept trap on ground [m]

####### Type.

Mexico, Tabasco, Teapa

####### Etymology.

Latin, peace

####### Collection.

TAMU

##### Genus *Septentrinna* Bonaldo, 2000

###### Septentrinna bicalcarata

Taxon classificationAnimaliaAraneaeCorinnidae

(Simon, 1896)

Septentrinna bicalcarata Bonaldo 2000: 85, mf, desc. (figs 229–233); Trevino 2014: 11; Ubick and Richman 2005a: 82

####### Distribution.

Brewster, Hudspeth, Webb

####### Locality.

Big Bend National Park, Guadalupe Pass, Signal Peak

####### Time of activity.

Male (May); female (April – May)

####### Type.

Arizona

####### Etymology.

Latin, two-spurred

####### Collection.

MSU

#### Family Ctenidae Keyserling, 1877

##### Genus *Anahita* Karsch, 1879

###### Anahita punctulata

Taxon classificationAnimaliaAraneaeCtenidae

(Hentz, 1844)

Anahita punctulata Jackman 1997: 162; Peck 1981: 158 [T], mf, desc. (figs 1–4); Roth 1982: 14–1; Roth 1985: B-9–1; Roth 1994: 86; Sissom et al. 1999: 260, mf, desc.; Ubick and Dávilla 2005: 84
Ctenus punctulatus Hentz, 1844; Marx 1890: 567

####### Distribution.

Harris (Houston), Tyler

####### Locality.

Kirby State Forest

####### Time of activity.

Male (April 27-May 8)

####### Method.

Flight intercept trap on ground [m]

####### Type.

Alabama

####### Etymology.

Latin, minute white dots on abdomen

####### Collection.

TAMU

##### Genus *Ctenus* Walckenaer, 1805

###### Ctenus valverdiensis

Taxon classificationAnimaliaAraneaeCtenidae

Peck, 1981

Ctenus valverdiensis Jackman 1997: 162; Peck 1981: 164, f, desc. (figs 18–19); Sissom et al. 1999: 261, m, desc. (figs 3–5, 7–9)
Ctenus sp.; Reddell 1965: 170; Reddell 1970: 405

####### Distribution.

Val Verde

####### Caves.

**Val Verde** (Cave 8, Diablo Cave, East Gypsum Cave, Ladder Cave, Langtry East Gypsum Cave, Tarantula Cave)

####### Time of activity.

Male (May, September); female (January, September)

####### Habitat.

(landscape features: cave)

####### Type.

Texas (female, Val Verde Co., East Gypsum Cave, January 25, 1964, J. Reddell, D. McKenzie, J. Porter, holotype, AMNH)

####### Etymology.

locality (The specific name refers to the type locality, Peck 1981).

####### Collection.

TMM

##### Genus *Leptoctenus* L. Koch, 1878

###### Leptoctenus byrrhus

Taxon classificationAnimaliaAraneaeCtenidae

Simon, 1888

Leptoctenus byrrhus Broussard and Horner 2006: 253; Cokendolpher 1993: 39; Gertsch 1935b: 24, mf (figs 56–60); Gertsch 1939b: 25; Jackman 1997: 162; Peck 1981: 166, mf, desc. (figs 20–21, 24–25); Polotow and Brescovit 2014: Appendix S1; Reddell and Cokendolpher 2004: 77; Richman et al. 2011a: 47; Roth 1982: 14–1; Roth 1985: B-9–1; Roth 1994: 86; Sissom et al. 1999: 261, f, desc. (figs 1–2); Ubick and Dávilla 2005: 84
Ctenus byrrhus (Simon, 1888); Bonnet 1956: 1277; Comstock 1940: 569, desc.; Reddell 1965: 170 [part]; Vogel 1970b: 7

####### Distribution.

Central and south Texas; Bandera, Bexar, Brewster, Cameron, Hidalgo, Kendall, Kerr, Medina, Presidio, Starr, Terrell, Val Verde

####### Locality.

Bentsen-Rio Grande Valley State Park, Big Bend National Park, Chihuahuan desert, Chisos Basin, Chisos Mountains, Dalquest Research Site, Lost Maples State Park, Lower Rio Grande Valley National Wildlife Refuge, Sabal Palm Audubon Sanctuary

####### Caves.

**Bexar** (Get A Rope Cave, Up the Creek Cave); **Medina** (Haby Bat Cave); **Terrell** (Longley Cave); **Val Verde** (Diablo Cave, Ladder Cave, Langtry East Gypsum Cave, Unnamed Cave No. 8)

####### Time of activity.

Male (February 28-March 13, March 26-April 1, April – October); female (July – September, November)

####### Habitat.

(grass: grass); (landscape features: cave, under rock); (soil/woodland: forest litter, palm forest, re-vegetated site, upland deciduous forest)

####### Method.

carrion trap [m]; flight intercept trap on ground [m]; pitfall trap [mf]; yellow pan trap [m]

####### Type.

Mexico

####### Etymology.

Latin, red

####### Collection.

MCZ, MSU, TAMU, TMM

#### Family Cybaeidae Banks, 1892

nomen dubium

*Cybaeus austinensis* (Chamberlin, 1924); Bonnet 1956: 1301; Bonnet 1958: 3339; Roewer 1955: 89; Roth and Brown 1986: 15

*Parauximus austinensis* Chamberlin 1924; Chamberlin 1924a: 2; Roth 1985: 10; Chamberlin and Ivie 1932: 7

**Locality.** Texas: Austin, R. V. Chamberlin, August, 1909

**Note.** Described in Dictynidae (Chamberlin 1924a: 2), transferred to Agelenidae (Chamberlin and Ivie 1932: 7), transferred to Cybaeidae (Brignoli 1983: 467). Listed as **nomen dubium** (Roth and Brown 1986: 15).

#### Family Dictynidae O. P.-Cambridge, 1871

These are federally endangered (US Fish and Wildlife Service 2010). All are from Bexar Co.

*Cicurina baronia* Gertsch, 1992 Robber Baron Cave

*Cicurina madla* Gertsch, 1992 Madla’s Cave

*Cicurina venii* Gertsch, 1992 Braken Bat Cave

*Cicurina vespera* Gertsch, 1992 Government Canyon Bat Cave

**Note.** species incorrectly reported from Texas

*Emblyna altamira* (Gertsch & Davis, 1942); Jackman 1997: 163 [not in Texas]

*Dictyna altamira* Gertsch & Davis, 1942; Vogel 1970b: 7

*Dictyna crosbyi* Gertsch & Mulaik, 1940; Roewer 1955: 1320 [not in Texas]

**nomen nudum**

*Dictyna texana* Banks, 1898; Banks 1910: 18; F. O. P.-Cambridge 1902: 359, errata [Texas record] [see Chamberlin and Gertsch 1958: 133, *Dictyna iviei* Gertsch & Mulaik, 1936]

##### Genus *Argennina* Gertsch & Mulaik, 1936

###### Argennina unica

Taxon classificationAnimaliaAraneaeDictynidae

Gertsch & Mulaik, 1936

Argennina unica Bennett 2005a: 99; Chamberlin and Gertsch 1958: 17, f, desc. (pl. 2, fig. 10); Gertsch and Mulaik 1936a: 2, f, desc. (fig. 5); Gertsch and Mulaik 1940: 326; Jackman 1997: 162; Lehtinen 1967: 216; Roewer 1955: 1303; Roth 1982: 15–1; Roth 1985: B-11–1; Roth 1994: 89; Vogel 1970b: 7

####### Distribution.

Hidalgo

####### Type.

Texas (female, Hidalgo Co., Edinburg, spring 1933, S. Mulaik, holotype, AMNH)

[male unknown]

####### Etymology.

Latin, singular

##### Genus *Brommella* Tullgren, 1948

###### Brommella lactea

Taxon classificationAnimaliaAraneaeDictynidae

(Chamberlin & Gertsch, 1958)

Brommella lactea Brignoli 1983: 518 [T]; Jackman 1997: 162
Pagomys lactea Chamberlin & Gertsch, 1958; Chamberlin and Gertsch 1958: 16, f, desc.; Vogel 1967: 59; Vogel 1970b: 8

####### Distribution.

Randall

####### Locality.

Palo Duro Canyon

####### Time of activity.

Female (December)

####### Type.

Texas (female, Randall Co., Palo Duro Canyon, near Amarillo, December 1934, D. & S. Mulaik, holotype, AMNH)

[male unknown]

####### Etymology.

Latin, of milk

##### Genus *Cicurina* Menge, 1871

**Note.** transferred from Agelenidae to Dictynidae (Lehtinen 1967: 223)

###### Cicurina aenigma

Taxon classificationAnimaliaAraneaeDictynidae

Gertsch, 1992

Cicurina aenigma Gertsch 1992: 94, f, desc. (figs 29–30); Jackman 1997: 162

####### Distribution.

Hays

####### Time of activity.

Female (April)

####### Type.

Texas (female, Hays Co., April 13, 1939, D. & S. Mulaik, holotype, AMNH)

[male unknown]

####### Etymology.

Latin, enigma, secret

###### Cicurina arcuata

Taxon classificationAnimaliaAraneaeDictynidae

Keyserling, 1887

Cicurina arcuata Agnew et al. 1985: 7; Bonnet 1956: 1086; Jackman 1997: 162; Jones 1936: 69; Vogel 1970b: 2 [Chamberlin and Ivie 1940: 63, mf, desc. (figs 46–47, 84–85)]

####### Distribution.

Dallas, Erath

####### Time of activity.

Female (March)

####### Habitat.

(soil/woodland: under [log, log in woods], woods)

####### Type.

United States

####### Etymology.

Latin, an arch

####### Collection.

TAMU

###### Cicurina armadillo

Taxon classificationAnimaliaAraneaeDictynidae

Gertsch, 1992

Cicurina armadillo Gertsch 1992: 95, f, desc. (figs 33–34, chart 1); Jackman 1997: 162

####### Distribution.

Travis

####### Time of activity.

Female (January)

####### Habitat.

(nest/prey: armadillo nest)

####### Type.

Texas (female, Travis Co., near Austin, January 8, 1948, Chelden, holotype, AMNH)

[male unknown]

####### Etymology.

Spanish, animal

###### Cicurina bandera

Taxon classificationAnimaliaAraneaeDictynidae

Gertsch, 1992

Cicurina bandera Gertsch 1992: 111, f, desc. (figs 113–114); Jackman 1997: 162; Paquin and Dupérré 2009: 14, f, desc. (figs 8–9, 135)

####### Distribution.

Bandera

####### Caves.

**Bandera** (Fossil Cave)

####### Time of activity.

Female (March, July)

####### Habitat.

(landscape features: cave)

####### Type.

Texas (female, Bandera Co., Fossil Cave, July 23, 1966, J. Reddell, D. McKenzie, holotype, AMNH)

[male unknown]

####### Etymology.

locality (Named for Bandera County, Gertsch 1992).

####### Collection.

TMM

###### Cicurina bandida

Taxon classificationAnimaliaAraneaeDictynidae

Gertsch, 1992

Cicurina bandida Culver et al. 2003: 463; Gertsch 1992: 107, f, desc. (figs 79–80); Jackman 1997: 162, 171; Paquin and Dupérré 2009: 14 f, desc. (figs 10–11, 132); Paquin and Hedin 2005b: 2–7; Paquin and Hedin 2006: 165; Paquin et al. 2008: 142 [S], mf, desc. (figs 2–3, 4a–g, 5a)
Cicurina cueva Gertsch, 1992; Culver et al. 2003: 464; Gertsch 1992: 107, f, desc. (figs 81–82); Jackman 1997: 162; Paquin and Dupérré 2009: 22, f, desc. (figs 32–33, 132); Paquin and Hedin 2005b: 2–7
Cicurina reyesi Gertsch, 1992; Culver et al. 2003: 464; Gertsch 1992: 107, f, desc. (figs 85–86); Jackman 1997: 162; Paquin and Dupérré 2009: 41, f, desc. (figs 88–89, 132); Paquin and Hedin 2004: 3254; Paquin and Hedin 2005b: 2, 4–7

####### Distribution.

Travis

####### Caves.

**Travis** (Airman’s Cave, Bandit Cave, Blowing Sink, Cave X, Driskill Cave, Flint Ridge Cave, Get Down Cave, Ireland’s Cave, Lost Gold Cave, Lost Oasis Cave, Maple Run Cave)

####### Time of activity.

Female (March – June, September)

####### Habitat.

(landscape features: cave)

####### Type.

Texas (female, Travis Co., Bandit Cave, May 26, 1966, J. Reddell, J. Fish, holotype, AMNH)

####### Etymology.

locality (Specific name from Spanish bandido, bandit, named for Bandit Cave, Gertsch 1992).

####### Collection.

TMM

###### Cicurina baronia

Taxon classificationAnimaliaAraneaeDictynidae

Gertsch, 1992

Cicurina baronia Cokendolpher 2004a: 38, f, desc. (figs 28–31); Federal Register 1998: 71855–71856, 71858, 71860, 71866; Federal Register 2000: 81419–81421, 81425, 81428, 81433; Federal Register 2002: 55064, 55066–55067, 55075, 55086–55087, 55089; Federal Register 2003: 17156, 17158, 17176, 17191, 17203; Gertsch 1992: 109, mf, desc. (figs 89–90, 155–156); Jackman 1997: 162, 171; NABN 2001: 8; Paquin and Dupérré 2009: 15, f, desc. (figs 12–13, 134); Reddell and Cokendolpher 2004: 79; SWCA Environmental Consultants 2007: 3

####### Distribution.

Bexar

####### Caves.

**Bexar** (Robber Baron Cave)

####### Time of activity.

Male (June, December); female (April)

####### Habitat.

(landscape features: cave)

####### Type.

Texas (female, Bexar Co., Robber Barron Cave, April 1969, R. Bartholomew, holotype, AMNH)

####### Etymology.

locality (Specific name for Robber Baron Cave, Gertsch 1992).

####### Collection.

TMM, TTU

###### Cicurina barri

Taxon classificationAnimaliaAraneaeDictynidae

Gertsch, 1992

Cicurina barri Culver et al. 2003: 463; Gertsch 1992: 117, f, desc. (figs 141–142); Jackman 1997: 162; Paquin and Dupérré 2009: 15, f, desc. (figs 14–15, 137)

####### Distribution.

Sutton

####### Caves.

**Sutton** (Caverns of Sonora [=Mayfield Cave])

####### Time of activity.

Female (August)

####### Habitat.

(landscape features: cave)

####### Type.

Texas (female, Sutton Co., Caverns of Sonora, August 29, 1959, T. Barr, holotype, AMNH)

[male unknown]

####### Etymology.

Person (Specific name for Thomas Barr, dean of American speleologists, Gertsch 1992).

####### Collection.

TMM

###### Cicurina blanco

Taxon classificationAnimaliaAraneaeDictynidae

Gertsch, 1992

Cicurina blanco Gertsch 1992: 95, f, desc. (figs 7–8); Jackman 1997: 162

####### Distribution.

Blanco

####### Time of activity.

Female (February)

####### Type.

Texas (female, Blanco Co., 10 miles E Johnson City, February 23, 1986, S. J. Harden, holotype, AMNH)

[male unknown]

####### Etymology.

Spanish, white (refers to Blanco Co.)

###### Cicurina browni

Taxon classificationAnimaliaAraneaeDictynidae

Gertsch, 1992

Cicurina browni Culver et al. 2003: 463; Gertsch 1992: 98, f, desc. (figs 53–54); Jackman 1997: 162; Paquin and Dupérré 2009: 16, f, desc. (figs 16–17, 130); Paquin and Hedin 2005b: 10; SWCA Environmental Consultants 2007: 1–2, 3–32

####### Distribution.

Williamson

####### Caves.

**Williamson** (Brown’s Cave)

####### Time of activity.

Female (April)

####### Habitat.

(landscape features: cave)

####### Type.

Texas (female, Williamson Co., Brown’s Cave, April 23, 1989, W. Elliott, J. Reddell, M. Reyes, holotype, AMNH)

[male unknown]

####### Etymology.

locality (Named for Brown’s Cave, Gertsch 1992).

####### Collection.

TMM

###### Cicurina brunsi

Taxon classificationAnimaliaAraneaeDictynidae

Cokendolpher, 2004

Cicurina brunsi Cokendolpher 2004a: 38, f, desc. (figs 32–33); Paquin and Dupérré 2009: 16, f, desc. (figs 18–19, 134); Paquin and Hedin 2004: 3253; Reddell and Cokendolpher 2004: 79

####### Distribution.

Bexar

####### Locality.

Camp Bullis

####### Caves.

**Bexar** (Stahl Cave)

####### Time of activity.

Female (November)

####### Habitat.

(landscape features: cave)

####### Type.

Texas (female, Bexar Co., Stahl Cave, Camp Bullis, November 1, 2001, J. R. Reddell & M. Reyes (molted December 14, 2001, August 2, 2002), holotype, AMNH)

[male unknown]

####### Etymology.

Person (The specific name is honoring Dusty Bruns for his efforts in promoting cave research and sound cave management at Camp Bullis, Cokendolpher 2004a).

####### Collection.

TMM

###### Cicurina bullis

Taxon classificationAnimaliaAraneaeDictynidae

Cokendolpher, 2004

Cicurina bullis Cokendolpher 2004a: 39, f, desc. (figs 34–36); Paquin and Dupérré 2009: 17, f, desc. (figs 20–21, 134); Paquin and Hedin 2004: 3253; Reddell and Cokendolpher 2004: 80

####### Distribution.

Bexar

####### Locality.

Camp Bullis

####### Caves.

**Bexar** ([all Camp Bullis] Eagles Nest Cave, Hilger Hole, Isocow Cave, Platypus Pit, Root Canal Cave)

####### Time of activity.

Female (March – April, November – December)

####### Habitat.

(landscape features: cave)

####### Type.

Texas (female, Bexar Co., Isocow Cave, Zone 3, Camp Bullis, March 2, 1994, W. Elliott & G. Veni, holotype, AMNH)

[male unknown]

####### Etymology.

locality (The specific name is a noun in apposition; taken from Camp Bullis, Cokendolpher 2004a).

####### Collection.

TMM, TTU

###### Cicurina buwata

Taxon classificationAnimaliaAraneaeDictynidae

Chamberlin & Ivie, 1940

Cicurina buwata Chamberlin and Ivie 1940: 74, immature, desc. (fig. 94); Cokendolpher 2004a: 32 [S], f, desc. (figs 19–20); Gertsch 1992: 78, 120; Hedin 2015: 348; Jackman 1997: 162; Nicholas 1960: 156; Paquin and Dupérré 2009: 18, f, desc. (figs 22–23, 131); Paquin and Hedin 2004: 3253; Paquin and Hedin 2005b: 10; Reddell 1965: 169; Reddell and Finch 1963: 40; Roth and Brown 1986: 7; SWCA Environmental Consultants 2007: 1–2, 3–32; Vogel 1967: 7; Vogel 1970b: 2
Cicurina elliotti Gertsch, 1992; Culver et al. 2003: 464; Gertsch 1992: 101, f, desc. (figs 73–74); Jackman 1997: 162

####### Distribution.

Travis, Williamson

####### Caves.

**Travis** (Backyard Cave, Cotterell Cave, Fossil Garden Cave, Gallifer Cave, McNeil Bat Cave); **Williamson** (Beck’s Sewer Cave, Bev’s Grotto, Buttercup River Cave, Good Friday Cave, McNeil Quarry Cave, Marigold Cave, Rattlesnake Filled Cave, Testudo Tube, T.W.A.S. A Cave, Underline Cave)

####### Time of activity.

Female (January – June, August – September)

####### Habitat.

(landscape features: cave)

####### Type.

Texas (immature, Travis Co., cave near Austin, March 12–18, 1903, J. H. Comstock, type, AMNH)

[male unknown]

####### Etymology.

undetermined

####### Collection.

TMM

###### Cicurina caliga

Taxon classificationAnimaliaAraneaeDictynidae

Cokendolpher & Reddell, 2001

Cicurina caliga Cokendolpher and Reddell 2001b: 38, f, desc. (figs 1–2, 3A); Paquin and Dupérré 2009: 19, f, desc. (figs 24–25, 129); Paquin and Hedin 2004: 3253

####### Distribution.

Bell

####### Locality.

Fort Hood

####### Caves.

**Bell** ([all Fort Hood] Buchanan Cave, Streak Cave, Triple J Cave)

####### Time of activity.

Female (May – June, November)

####### Habitat.

(landscape features: cave)

####### Type.

Texas (female, Bell Co., Triple J Cave, November 1994, M. Warton, holotype, AMNH)

[male unknown]

####### Etymology.

Latin, noun for army boot (army base)

####### Collection.

TMM

###### Cicurina caverna

Taxon classificationAnimaliaAraneaeDictynidae

Gertsch, 1992

Cicurina caverna Culver et al. 2003: 463; Gertsch 1992: 115, f, desc. (figs 131–132); Jackman 1997: 162; Paquin and Dupérré 2009: 19, f, desc. (figs 26–27, 137)

####### Distribution.

Kimble

####### Caves.

**Kimble** (Flemming’s Bat Cave)

####### Time of activity.

Female (February)

####### Habitat.

(landscape features: cave)

####### Type.

Texas (female, Kimble Co., Flemming’s Bat Cave, February 21, 1964, W. H. Russell, holotype, AMNH)

[male unknown]

####### Etymology.

Latin, a cavern

####### Collection.

TMM

###### Cicurina coryelli

Taxon classificationAnimaliaAraneaeDictynidae

Gertsch, 1992

Cicurina coryelli Cokendolpher 2004b: 61; Cokendolpher and Reddell 2001b: 40, f, desc. (figs 3 B, 4–7); Gertsch 1992: 103, f, desc. (figs 71–72); Jackman 1997: 162; Paquin and Dupérré 2009: 22, f, desc. (figs 30–31, 129); Paquin and Hedin 2004: 3253

####### Distribution.

Coryell

####### Locality.

Fort Hood

####### Caves.

**Coryell** ([all Fort Hood] Big Red Cave, Egypt Cave, Tippit Cave)

####### Time of activity.

Female (January, April – May)

####### Habitat.

(landscape features: cave)

####### Type.

Texas (female, Coryell Co., Tippit Cave, January 31, 1992, J. Reddell, M. Reyes, holotype, AMNH)

[male unknown]

####### Etymology.

locality (Specific name for Coryell County, Texas, Gertsch 1992).

####### Collection.

TMM, TTU

###### Cicurina davisi

Taxon classificationAnimaliaAraneaeDictynidae

Exline, 1936

Cicurina davisi Chamberlin and Ivie 1940: 59, mf, desc. (figs 41, 83); Exline 1936: 18, f, desc. (fig. 24); Jackman 1997: 162; Roth and Brown 1986: 7; Vogel 1970b: 2

####### Distribution.

Concho, Kerr, Llano

####### Locality.

Raven Ranch

####### Time of activity.

Male (December); female (December)

####### Type.

Texas (female, Llano Co., December 1934, L. I. Davis, holotype, AMNH)

####### Etymology.

Person (collector)

###### Cicurina delrio

Taxon classificationAnimaliaAraneaeDictynidae

Gertsch, 1992

Cicurina delrio Gertsch 1992: 96, f, desc. (figs 21–22, chart 1); Jackman 1997: 162; Paquin and Dupérré 2009: 23, f, desc. (figs 34–35, 138)

####### Distribution.

Val Verde

####### Locality.

Ellison Brite Ranch

####### Caves.

**Val Verde** (Cave No. 8, Diablo Cave, Sunset Cave)

####### Time of activity.

Female (August, December)

####### Habitat.

(landscape features: under rock); (objects: under rotting shirt)

####### Type.

Texas (female, Val Verde Co., 12 miles NW Del Rio, Sunset Cave, December 14, 1962, J. Reddell, W. Russell, holotype, AMNH)

[male unknown]

####### Etymology.

locality (Specific name for Del Rio, Texas, used in apposition, Gertsch 1992).

####### Collection.

TMM

###### Cicurina dorothea

Taxon classificationAnimaliaAraneaeDictynidae

Gertsch, 1992

Cicurina dorothea Gertsch 1992: 94 [S], f, desc. (figs 35–36); Jackman 1997: 162
Cicurina texana (Gertsch, 1935); Chamberlin and Ivie 1940: 78, f, desc. (fig. 64) [part]; Vogel 1970b: 2
Cicurina minorata (Gertsch & Davis, 1936); Henderson 2007: 68, 76, 79, 82 [misidentified]

####### Distribution.

Brazos, Kerr

####### Locality.

Lick Creek Park, Raven Ranch

####### Time of activity.

Female (August, September 17-October 20, December)

####### Habitat.

(soil/woodland: post oak woodland)

####### Method.

pitfall trap [f]

####### Type.

Texas (female, Kerr Co., Raven Ranch, August 1939, D. & S. Mulaik, holotype, AMNH)

[male unknown]

####### Etymology.

Person (Specific name for Dorothea Mulaik, collector of many Texas spiders, Gertsch 1992).

####### Collection.

TAMU

###### Cicurina ezelli

Taxon classificationAnimaliaAraneaeDictynidae

Gertsch, 1992

Cicurina ezelli Culver et al. 2003: 464; Gertsch 1992: 99, f, desc. (figs 61–62); Jackman 1997: 162; Paquin and Dupérré 2009: 23, f, desc. (figs 36–37, 133); Paquin and Hedin 2004: 3253

####### Distribution.

Hays

####### Caves.

**Hays** (Ezell’s Cave, Grapevine Cave)

####### Time of activity.

Female (July, September)

####### Habitat.

(landscape features: cave)

####### Type.

Texas (female, Hays Co., Ezell’s Cave, September 7, 1963, J. Reddell, D. McKenzie, R. Ballinger, holotype, AMNH)

[male unknown]

####### Etymology.

locality (Specific name for Ezell’s Cave, Gertsch 1992).

####### Collection.

TMM

###### Cicurina gruta

Taxon classificationAnimaliaAraneaeDictynidae

Gertsch, 1992

Cicurina gruta Culver et al. 2003: 464; Gertsch 1992: 117, f, desc. (figs 147–148); Jackman 1997: 162; Paquin and Dupérré 2009: 24, f, desc. (figs 38–39, 138 [sic 28–29])

####### Distribution.

Edwards

####### Caves.

**Edwards** (Dunbar Cave)

####### Time of activity.

Female (September)

####### Habitat.

(landscape features: cave)

####### Type.

Texas (female, Edwards Co., Dunbar Cave, September 29, 1956, W. McAlister, holotype, AMNH)

[male unknown]

####### Etymology.

Spanish, cave

####### Collection.

TMM

###### Cicurina hexops

Taxon classificationAnimaliaAraneaeDictynidae

Chamberlin & Ivie, 1940

Cicurina hexops Chamberlin and Ivie 1940: 79, m, desc. (fig. 92); Gertsch 1992: 90, m, desc. (figs 49–50); Jackman 1997: 162; Roth and Brown 1986: 7; Vogel 1967: 7; Vogel 1970b: 2

####### Distribution.

Tom Green

####### Time of activity.

Male (December)

####### Type.

Texas (male, Tom Green Co., Water Valley, December 1939, S. & D. Mulaik, holotype, AMNH)

[female unknown]

####### Etymology.

Latin, 6 eyes

###### Cicurina holsingeri

Taxon classificationAnimaliaAraneaeDictynidae

Gertsch, 1992

Cicurina holsingeri Culver et al. 2003: 464; Gertsch 1992: 98, f, desc. (figs 57–58); Jackman 1997: 162; Paquin and Dupérré 2009: 24, f, desc. (figs 40–41, 138)

####### Distribution.

Val Verde

####### Locality.

Seminole Canyon State Park

####### Caves.

**Val Verde** (Seminole Canyon Cave)

####### Time of activity.

Female (March)

####### Habitat.

(landscape features: cave)

####### Type.

Texas (female, Val Verde Co., Seminole Canyon State Park, March 4, 1983, W. R. Elliott, holotype, AMNH)

[male unknown]

####### Etymology.

Person (Named for Dr. John R. Holsinger of Old Dominion University, specialist on many cave animals, Gertsch 1992).

####### Collection.

TMM

###### Cicurina hoodensis

Taxon classificationAnimaliaAraneaeDictynidae

Cokendolpher & Reddell, 2001

Cicurina hoodensis Cokendolpher 2004b: 61; Cokendolpher and Reddell 2001b: 43, f, desc. (figs 3C, 10–11); Paquin and Dupérré 2009: 25, f, desc. (figs 42–43, 129); Paquin and Hedin 2004: 3253

####### Distribution.

Bell

####### Locality.

Fort Hood

####### Caves.

**Bell** ([all Fort Hood] Buchanan Cave, Camp 6 Cave No. 1, Peep in the Deep Cave, Talking Crows Cave, Treasure Cave, Triple J Cave)

####### Time of activity.

Female (April – June, November)

####### Habitat.

(landscape features: cave)

####### Type.

Texas (female, Bell Co., Buchanan Cave, May 7, 1998, L. J. Graves, J. Reddell & M. Reyes, holotype, AMNH)

[male unknown]

####### Etymology.

locality (This species is named for its occurrence on Fort Hood, Cokendolpher and Reddell 2001b).

####### Collection.

TMM, TTU

###### Cicurina joya

Taxon classificationAnimaliaAraneaeDictynidae

Gertsch, 1992

Cicurina joya Gertsch 1992: 96, f, desc. (figs 13–14, chart 1); Jackman 1997: 162; Reddell and Cokendolpher 2004: 80

####### Distribution.

Comal

####### Caves.

**Comal** (Brehmmer Cave, Heidrich’s Cave)

####### Time of activity.

Female (March)

####### Habitat.

(landscape features: cave)

####### Type.

Texas (female, Comal Co., Heidrich’s Cave, March 19, 1960, W. J. Gertsch, W. Ivie, holotype, AMNH)

[male unknown]

####### Etymology.

Spanish, jewel

####### Collection.

TMM

###### Cicurina loftini

Taxon classificationAnimaliaAraneaeDictynidae

Cokendolpher, 2004

Cicurina loftini Cokendolpher 2004a: 41, f, desc. (figs 37–39); Paquin and Dupérré 2009: 27, f, desc. (figs 46–47, 134); Paquin and Hedin 2004: 3253; Paquin and Hedin 2005b: 10; Reddell and Cokendolpher 2004: 80; White et al. 2009: 341

####### Distribution.

Bexar

####### Caves.

**Bexar** (Caracol Creek Coon Cave, SBC Cave)

####### Time of activity.

Female (February, June)

####### Habitat.

(landscape features: cave)

####### Type.

Texas (female, Bexar Co., Caracol Creek Coon Cave, June 15, 1993, J. Loftin, J. R. Reddell, M. Reyes & G. Veni, holotype, AMNH)

[male unknown]

####### Etymology.

Person (The species is named after James Loftin of San Antonio, for his years of cave explorations, Cokendolpher 2004a).

####### Collection.

TMM, TTU

###### Cicurina machete

Taxon classificationAnimaliaAraneaeDictynidae

Gertsch, 1992

Cicurina machete Gertsch 1992: 114, f, desc. (figs 125–126); Jackman 1997: 162; Paquin and Dupérré 2009: 28, f, desc. (figs 48–49, 130)

####### Distribution.

San Saba

####### Caves.

**San Saba** (Whiteface Cave)

####### Time of activity.

Female (February)

####### Habitat.

(landscape features: cave)

####### Type.

Texas (female, San Saba Co., Whiteface Cave, February 9, 1964, J. Reddell, D. McKenzie, K. Garrett, holotype, AMNH)

[male unknown]

####### Etymology.

Spanish, cutlass

####### Collection.

TMM

###### Cicurina madla

Taxon classificationAnimaliaAraneaeDictynidae

Gertsch, 1992

Cicurina madla Cokendolpher 2004a: 42, f, desc. (figs 40–46); Federal Register 1998: 71855–71856, 71858, 71860, 71866; Federal Register 2000: 81419–81421, 81425, 81428, 81433; Federal Register 2002: 55064, 55066–55067, 55074– 55075, 55086–55087, 55089; Federal Register 2003: 17156, 17158, 17175–17176, 17190–17191, 17195; Gertsch 1992: 109, f, desc. (figs 91–92); Jackman 1997: 162, 171; NABN 2001: 8; Paquin and Dupérré 2009: 28, f, desc. (figs 50–51, 134–135); Paquin and Hedin 2004: 3253–3254; Reddell and Cokendolpher 2004: 80; SWCA Environmental Consultants 2007: 3

####### Distribution.

Bexar, Uvalde

####### Locality.

Camp Bullis

####### Caves.

**Bexar** (Christmas Cave, Headquarters Cave [Camp Bullis], Helotes Blowhole, Hills and Dales Pit, Logan’s Cave, Lost Pothole (=Lost Pot), Madla’s Cave, Madla’s Drop Cave, Robber’s Cave)

####### Time of activity.

Female (February, June – July, September – October)

####### Habitat.

(landscape features: cave)

####### Type.

Texas (female, Bexar Co., Madlas’s Cave, October 4, 1963, J. Reddell, D. McKenzie, holotype, AMNH)

[male unknown]

####### Etymology.

locality (Specific name for Madlas’s Cave, Gertsch 1992).

####### Collection.

TMM, TTU

###### Cicurina marmorea

Taxon classificationAnimaliaAraneaeDictynidae

Gertsch, 1992

Cicurina marmorea Gertsch 1992: 90, f, desc. (figs 11–12); Jackman 1997: 162

####### Distribution.

Burnet

####### Time of activity.

Female (November)

####### Type.

Texas (female, Burnet Co., 8 miles N Marble Falls, November 8, 1964, J. Reddell, holotype, AMNH)

[male unknown]

####### Etymology.

Latin, marble for Marble Falls

###### Cicurina mckenziei

Taxon classificationAnimaliaAraneaeDictynidae

Gertsch, 1992

Cicurina mckenziei Gertsch 1992: 115, f, desc. (figs 139–140); Jackman 1997: 162; Paquin and Dupérré 2009: 30, f, desc. (figs 54–55, 136)

####### Distribution.

Bandera

####### Caves.

**Bandera** (Fog Fissure)

####### Time of activity.

Female (October)

####### Habitat.

(landscape features: cave)

####### Type.

Texas (female, Bandera Co., Fog Fissure, October 30, 1963, D. McKenzie, holotype, AMNH)

[male unknown]

####### Etymology.

Person (Named for David McKenzie, student of caves, Gertsch 1992).

####### Collection.

TMM

###### Cicurina medina

Taxon classificationAnimaliaAraneaeDictynidae

Gertsch, 1992

Cicurina medina Culver et al. 2003: 464; Gertsch 1992: 117, m, desc. (figs 149–150); Jackman 1997: 162; Paquin and Dupérré 2009: 30, m, desc. (figs 56–57, 135)

####### Distribution.

Medina

####### Caves.

**Medina** (Boehme’s Cave)

####### Time of activity.

Male (February)

####### Habitat.

(landscape features: cave)

####### Type.

Texas (male, Medina Co., Boehme’s Cave, February 16, 1964, J. Reddell, D. McKenzie, J. Porter, holotype, AMNH)

[female unknown]

####### Etymology.

locality (Named for Medina County, Gertsch 1992).

####### Collection.

TMM

###### Cicurina menardia

Taxon classificationAnimaliaAraneaeDictynidae

Gertsch, 1992

Cicurina menardia Culver et al. 2003: 464; Gertsch 1992: 98, mf, desc. (figs 59–60, 157–158); Jackman 1997: 162; Paquin and Dupérré 2009: 32, f, desc. (figs 58–59, 137)

####### Distribution.

Menard

####### Caves.

**Menard** (Powell’s Cave)

####### Time of activity.

Male (September); female (September)

####### Habitat.

(landscape features: cave)

####### Type.

Texas (female, Menard Co., Powell’s Cave, September 16, 1978, J. Reddell, holotype, AMNH)

####### Etymology.

locality (Specific name for Menard County, Gertsch 1992).

####### Collection.

TMM

###### Cicurina microps

Taxon classificationAnimaliaAraneaeDictynidae

Chamberlin & Ivie, 1940

Cicurina microps Chamberlin and Ivie 1940: 77, mf, desc. (figs 61–62, 91); Gertsch 1992: 97, mf, desc. (figs 16, 51–52, chart 1); Jackman 1997: 162; Roewer 1955: 51; Roth and Brown 1986: 7; Vogel 1967: 8; Vogel 1970b: 2

####### Distribution.

Kerr, McCulloch, Travis

####### Locality.

Raven Ranch

####### Time of activity.

Male (November – December); female (December)

####### Type.

Texas (male, Kerr Co., Raven Ranch, December 16, 1939, D. & S. Mulaik, holotype, AMNH)

####### Etymology.

Greek, small eyes

####### Collection.

DMNS

###### Cicurina minorata

Taxon classificationAnimaliaAraneaeDictynidae

(Gertsch & Davis, 1936)

Cicurina minorata Chamberlin and Ivie 1940: 80, f, desc. (figs 63, 96); Cokendolpher 2004a: 46, f, desc. (figs 48–51); Gertsch 1992: 92 [T], f, desc. (fig. 15); Jackman 1997: 162; Roewer 1955: 51; Roth and Brown 1986: 7; Vogel 1970b: 2
Chorizomma minorata Gertsch & Davis, 1936; Gertsch and Davis 1936: 6, f, desc. (fig. 8)
Chorizomma minoratum Gertsch & Davis, 1936; Bonnet 1956: 1076

####### Distribution.

Bexar

####### Time of activity.

Female (December)

####### Type.

Texas (female, Bexar Co., San Antonio, December 1934, L. I. Davis, holotype, AMNH)

[male unknown]

####### Etymology.

Latin, very small

###### Cicurina mirifica

Taxon classificationAnimaliaAraneaeDictynidae

Gertsch, 1992

Cicurina mirifica Gertsch 1992: 88, f, desc. (figs 5–6, chart 1); Jackman 1997: 162; Paquin and Dupérré 2009: 32, f, desc. (figs 60–61, 137)

####### Distribution.

Pecos

####### Caves.

**Pecos** (Amazing Maze Cave)

####### Time of activity.

Female (March)

####### Habitat.

(landscape features: cave)

####### Type.

Texas (female, Pecos Co., Amazing Maze Cave, March 1, 1986, A. Cobb, holotype, AMNH)

[male unknown]

####### Etymology.

Latin, wonder, amazing

####### Collection.

TMM

###### Cicurina mixmaster

Taxon classificationAnimaliaAraneaeDictynidae

Cokendolpher & Reddell, 2001

Cicurina mixmaster Cokendolpher and Reddell 2001b: 41, f, desc. (figs 8–9); Paquin and Dupérré 2009: 33, f, desc. (figs 62–63, 129); Paquin and Hedin 2004: 3254

####### Distribution.

Coryell

####### Caves.

**Coryell** (Mixmaster Cave)

####### Time of activity.

Female (November)

####### Habitat.

(landscape features: cave)

####### Type.

Texas (female, Coryell Co., Mixmaster Cave, November 5, 1998, J. Cokendolpher, J. Krejca, J. Reddell & M. Reyes, holotype, AMNH)

[male unknown]

####### Etymology.

locality (Noun in apposition; referring to the type locality, Cokendolpher and Reddell 2001b).

###### Cicurina modesta

Taxon classificationAnimaliaAraneaeDictynidae

Gertsch, 1992

Cicurina modesta Gertsch 1992: 90, m, desc. (figs 45–46); Jackman 1997: 162

####### Distribution.

Kerr

####### Method.

pitfall trap [m]

####### Type.

Texas (male, Kerr Co., Camp Verde, no date, W. Rogers, holotype, AMNH)

[female unknown]

####### Etymology.

Latin, modest

###### Cicurina neovespera

Taxon classificationAnimaliaAraneaeDictynidae

Cokendolpher, 2004

Cicurina neovespera Cokendolpher 2004a: 47, f, desc. (figs 52–53); Paquin and Dupérré 2009: 33, f, desc. (figs 64–65, 134); Reddell and Cokendolpher 2004: 80

####### Distribution.

Bexar

####### Caves.

**Bexar** (Elm Springs Cave [=Grubbs Cave ES], La Cantera Sink [=Grubbs Cave No. 23])

####### Time of activity.

Female (October)

####### Habitat.

(landscape features: cave)

####### Type.

Texas (female, Bexar Co., Elm Springs Cave, no date, A. G. Grubbs, holotype, AMNH)

[male unknown]

####### Etymology.

Greek, meaning new kin of *Cicurina vespera* Gertsch, 1992

####### Collection.

TMM

###### Cicurina obscura

Taxon classificationAnimaliaAraneaeDictynidae

Gertsch, 1992

Cicurina obscura Gertsch 1992: 113, f, desc. (figs 115–116); Jackman 1997: 162; Paquin and Dupérré 2009: 34, f, desc. (figs 66–67, 135)

####### Distribution.

Bandera

####### Caves.

**Bandera** (Sutherland Hollow Cave)

####### Time of activity.

Female (August)

####### Habitat.

(landscape features: cave)

####### Type.

Texas (female, Bandera Co., Sutherland Hollow Cave, August 4, 1974, S. Sweet, holotype, AMNH)

[male unknown]

####### Etymology.

Latin, obscure

####### Collection.

TMM

###### Cicurina orellia

Taxon classificationAnimaliaAraneaeDictynidae

Gertsch, 1992

Cicurina orellia Culver et al. 2003: 464; Gertsch 1992: 105, f, desc. (figs 107–108); Jackman 1997: 162; Paquin and Dupérré 2009: 34, f, desc. (figs 68–69, 135)

####### Distribution.

Real

####### Caves.

**Real** (Orell Crevice Cave, Ramsey Bat Cave)

####### Time of activity.

Female (August)

####### Habitat.

(landscape features: cave)

####### Type.

Texas (female, Real Co., Orell Crevice Cave, August 18, 1963, J. Reddell, D. McKenzie, holotype, AMNH)

[male unknown]

####### Etymology.

locality (Named for Orell Crevice Cave, Gertsch 1992).

####### Collection.

TMM

###### Cicurina pablo

Taxon classificationAnimaliaAraneaeDictynidae

Gertsch, 1992

Cicurina pablo Culver et al. 2003: 464; Gertsch 1992: 105, f, desc. (figs 105–106); Jackman 1997: 162; Paquin and Dupérré 2009: 35, f, desc. (figs 70–71, 135)

####### Distribution.

Uvalde

####### Caves.

**Uvalde** (Pablo’s Cave)

####### Time of activity.

Female (April)

####### Habitat.

(landscape features: cave)

####### Type.

Texas (female, Uvalde Co., Pablo’s Cave, April 5, 1963, J. Reddell, D. McKenzie, holotype, AMNH)

[male unknown]

####### Etymology.

locality (Specific name for Pablo’s Cave, used in apposition, Gertsch 1992).

####### Collection.

TMM

###### Cicurina pampa

Taxon classificationAnimaliaAraneaeDictynidae

Chamberlin & Ivie, 1940

Cicurina pampa Chamberlin and Ivie 1940: 79, f, desc. (fig. 60); Cokendolpher 2004a: 48 [S], f, desc. (figs 54–60); Gertsch 1992: 90, f, desc. (figs 23–24); Jackman 1997: 162; Paquin and Hedin 2004: 3254; Reddell and Cokendolpher 2004: 80; Roewer 1955: 51; Roth and Brown 1986: 8; Vogel 1967: 8; Vogel 1970b: 2
Cicurina gatita Gertsch, 1992; Gertsch 1992: 92, f, desc. (figs 27–28); Jackman 1997: 162

####### Distribution.

Bexar, Kendall

####### Locality.

Camp Bullis

####### Caves.

**Bexar** (Black Cat Cave, Cherry Hollow Cave (20b) [=Cave No. 19], Cross the Creek Cave [Camp Bullis], Karst Feature 471–4, Porcupine Squeeze Cave [=Grubs Cave No. 189], Stone Oak Parkway Pit, Up the Creek Cave [Camp Bullis], Vera Cruz Shaft [Camp Bullis])

####### Time of activity.

Female (January – April, October – December)

####### Habitat.

(landscape features: cave)

####### Type.

Texas (female, Kendall Co., December 1939, D. & S. Mulaik, holotype, AMNH)

[male unknown]

####### Etymology.

Spanish, grassy plain

####### Collection.

TMM

###### Cicurina pastura

Taxon classificationAnimaliaAraneaeDictynidae

Gertsch, 1992

Cicurina pastura Gertsch, 1992: 114, f, desc. (figs 123–124); Jackman 1997: 162; Paquin and Dupérré 2009: 35, f, desc. (figs 72–73, 136)

####### Distribution.

Kerr

####### Caves.

**Kerr** (Water Pond Pasture Cave)

####### Time of activity.

Female (October)

####### Habitat.

(landscape features: cave)

####### Type.

Texas (female, Kerr Co., Water Pond Pasture Cave, October 16, 1976, D. Pate, R. Fieseler, C. Yates, holotype, AMNH)

[male unknown]

####### Etymology.

Latin, pasture

####### Collection.

TMM

###### Cicurina patei

Taxon classificationAnimaliaAraneaeDictynidae

Gertsch, 1992

Cicurina patei Culver et al. 2003: 464; Gertsch 1992: 113, f, desc. (figs 117–118); Jackman 1997: 162; Paquin and Dupérré 2009: 36, f, desc. (figs 74–75, 138)

####### Distribution.

Val Verde

####### Caves.

**Val Verde** (Fawcett’s Cave)

####### Time of activity.

Female (April, August)

####### Habitat.

(landscape features: cave)

####### Type.

Texas (female, Val Verde Co., Fawcett’s Cave, August 8, 1987, D. Pate, holotype, AMNH)

[male unknown]

####### Etymology.

Person (Named for Dale Pate, Gertsch 1992).

####### Collection.

TMM

###### Cicurina platypus

Taxon classificationAnimaliaAraneaeDictynidae

Cokendolpher, 2004

Cicurina platypus Cokendolpher 2004a: 51, f, desc. (figs 61–62); Paquin and Dupérré 2009: 38, f, desc. (figs 76–77, 134); Paquin and Hedin 2004: 3254; Reddell and Cokendolpher 2004: 80

####### Distribution.

Bexar

####### Locality.

Camp Bullis

####### Caves.

**Bexar** (MARS Pit [Camp Bullis], Platypus Pit)

####### Time of activity.

Female (March, October)

####### Habitat.

(landscape features: cave)

####### Type.

Texas (female, Bexar Co., Platypus Pit, March 30, 1995, J. R. Reddell & M. Reyes, holotype, AMNH)

[male unknown]

####### Etymology.

locality (The specific name is a noun in apposition; taken from the type locality Platypus Pit, Cokendolpher 2004a).

####### Collection.

TMM

###### Cicurina porteri

Taxon classificationAnimaliaAraneaeDictynidae

Gertsch, 1992

Cicurina porteri Culver et al. 2003: 464; Gertsch 1992: 115, f, desc. (figs 133–134); Jackman 1997: 162; Paquin and Dupérré 2009: 38, f, desc. (figs 78–79, 138)

####### Distribution.

Val Verde

####### Caves.

**Val Verde** (Oriente Milestone Molasses Bat Cave)

####### Time of activity.

Female (January)

####### Habitat.

(landscape features: cave)

####### Type.

Texas (female, Val Verde Co., Oriente Milestone Molasses Bat Cave, January 25, 1964, J. Reddell, D. McKenzie, J. Porter, holotype, AMNH)

[male unknown]

####### Etymology.

Person (Named for John Porter, student of caves, Gertsch 1992).

####### Collection.

TMM

###### Cicurina puentecilla

Taxon classificationAnimaliaAraneaeDictynidae

Gertsch, 1992

Cicurina puentecilla Gertsch 1992: 111, f, desc. (figs 99–100); Jackman 1997: 162; Paquin and Dupérré 2009: 39, f, desc. (figs 80–81, 134); Paquin and Hedin 2004: 3254; Reddell and Cokendolpher 2004: 81; White et al. 2009: 341

####### Distribution.

Bexar, Comal

####### Caves.

**Bexar** (B-52 Cave, Black Cat Cave); **Comal** (Natural Bridge Caverns)

####### Time of activity.

Female (September)

####### Habitat.

(landscape features: cave)

####### Type.

Texas (female, Comal Co., Natural Bridge Caverns, September 2, 1978, A. G. Grubbs, holotype, AMNH)

[male unknown]

####### Etymology.

Spanish, little bridge

####### Collection.

TMM, TTU

###### Cicurina rainesi

Taxon classificationAnimaliaAraneaeDictynidae

Gertsch, 1992

Cicurina rainesi Culver et al. 2003: 464; Gertsch 1992: 117, f, desc. (figs 143–144); Jackman 1997: 162; Paquin and Dupérré 2009: 39, f, desc. (figs 82–83, 138)

####### Distribution.

Edwards

####### Caves.

**Edwards** (3-Bounce Pit)

####### Time of activity.

Female (February, July)

####### Habitat.

(landscape features: cave)

####### Type.

Texas (female, Edwards Co., 3-Bounce Pit, February 1974, T. Raines, J. Lewis, R. Fieseler, holotype, AMNH)

[male unknown]

####### Etymology.

Person (Named for Terry Raines, student of caves, Gertsch 1992).

####### Collection.

TMM

###### Cicurina reclusa

Taxon classificationAnimaliaAraneaeDictynidae

Gertsch, 1992

Cicurina reclusa Gertsch 1992: 111, f, desc. (figs 97–98); Jackman 1997: 162; Paquin and Dupérré 2009: 40, f, desc. (figs 84–85, 134); Reddell and Cokendolpher 2004: 81

####### Distribution.

Comal

####### Caves.

**Comal** (Kappelman Cave, Kappelman Salamander Cave)

####### Time of activity.

Female (March)

####### Habitat.

(landscape features: cave)

####### Type.

Texas (female, Comal Co., Kappelman Salamander Cave, March 15, 1964, W. Russell, J. Reddell, holotype, AMNH)

[male unknown]

####### Etymology.

Latin, recluse

####### Collection.

TMM

###### Cicurina riogrande

Taxon classificationAnimaliaAraneaeDictynidae

Gertsch & Mulaik, 1940

Cicurina riogrande Chamberlin and Ivie 1940: 76, f, desc. (figs 57–58); Gertsch 1992: 97, f, desc. (figs 3–4); Jackman 1997: 162; Roth and Brown 1986: 8; Vogel 1967: 9; Vogel 1970b: 2
Cicurina riogranda Gertsch & Mulaik, 1940; Roewer 1955: 52

####### Distribution.

Starr

####### Time of activity.

Female (January)

####### Type.

Texas (female, Starr Co., 5 miles E Rio Grande City, January 12, 1939, S. Mulaik, holotype, AMNH)

[male unknown]

####### Etymology.

locality (city)

###### Cicurina robusta

Taxon classificationAnimaliaAraneaeDictynidae

Simon, 1886

Cicurina robusta Exline 1936: 20, mf, desc. (figs 21, 21a); Jackman 1997: 162; Vogel 1970b: 2 [Chamberlin and Ivie 1940: 68, mf, desc. (figs 53, 87)]

####### Distribution.

Travis

####### Type.

Colorado

####### Etymology.

Latin, hard, strong

###### Cicurina rosae

Taxon classificationAnimaliaAraneaeDictynidae

Gertsch, 1992

Cicurina rosae Gertsch 1992: 94, f, desc. (figs 31–32); Jackman 1997: 162

####### Distribution.

Kimble

####### Time of activity.

Female (November)

####### Type.

Texas (female, Kimble Co., 7 miles E Junction, November 19, 1967, R. Carpenter, holotype, AMNH)

[male unknown]

####### Etymology.

Person (Specific name for Rose Carpenter, friend and collector of many Texas spiders, Gertsch 1992).

###### Cicurina rudimentops

Taxon classificationAnimaliaAraneaeDictynidae

Chamberlin & Ivie, 1940

Cicurina rudimentops Chamberlin and Ivie 1940: 76, f, desc. (fig. 59); Gertsch 1992: 95, f, desc. (figs 19–20, chart 1); Jackman 1997: 162; Roewer 1955: 52; Roth and Brown 1986: 8; Vogel 1967: 9; Vogel 1970b: 2

####### Distribution.

Jim Wells

####### Time of activity.

Female (December)

####### Type.

Texas (female, Jim Wells Co., 17 miles N Alice, December 1939, D. & S. Mulaik, holotype, AMNH)

[male unknown]

####### Etymology.

Latin, rudimentary eyes

####### Note.

Duval is wrong county listed in Gertsch 1992

###### Cicurina russelli

Taxon classificationAnimaliaAraneaeDictynidae

Gertsch, 1992

Cicurina russelli Culver et al. 2003: 464; Gertsch 1992: 107, f, desc. (figs 83–84); Jackman 1997: 162; Paquin and Dupérré 2009: 41, f, desc. (figs 90–91, 133)

####### Distribution.

Hays

####### Caves.

**Hays** (Boyett’s Cave)

####### Time of activity.

Female (March)

####### Habitat.

(landscape features: cave)

####### Type.

Texas (female, Hays Co., Boyett’s Cave, March 30, 1963, J. Reddell, W. Russell, holotype, AMNH)

[male unknown]

####### Etymology.

Person (Named for William Russell, Gertsch 1992).

####### Collection.

TMM

###### Cicurina sansaba

Taxon classificationAnimaliaAraneaeDictynidae

Gertsch, 1992

Cicurina sansaba Gertsch 1992: 114, mf, desc. (figs 127–128, 153–154); Jackman 1997: 162; Paquin and Dupérré 2009: 42, f, desc. (figs 92–93, 130)

####### Distribution.

San Saba

####### Caves.

**San Saba** (Gorman Cave, Lemons Ranch Cave)

####### Time of activity.

Male (June); female (March, June)

####### Habitat.

(landscape features: cave)

####### Type.

Texas (female, San Saba Co., Gorman Cave, March 15, 1963, J. Reddell, D. McKenzie, holotype, AMNH)

####### Etymology.

locality (Specific name for San Saba County, Gertsch 1992).

####### Collection.

TMM

###### Cicurina selecta

Taxon classificationAnimaliaAraneaeDictynidae

Gertsch, 1992

Cicurina selecta Culver et al. 2003: 464; Gertsch 1992: 105, f, desc. (figs 111–112); Jackman 1997: 162; Paquin and Dupérré 2009: 42, f, desc. (figs 94–95, 135)

####### Distribution.

Uvalde

####### Caves.

**Uvalde** (Sandtleben Cave [=Davy Crockett Cave])

####### Time of activity.

Female (October)

####### Habitat.

(landscape features: cave)

####### Type.

Texas (female, Uvalde Co., Sandtleben Cave, October 18, 1964, J. Reddell, holotype, AMNH)

[male unknown]

####### Etymology.

Latin, to choose

####### Collection.

TMM

###### Cicurina serena

Taxon classificationAnimaliaAraneaeDictynidae

Gertsch, 1992

Cicurina serena Culver et al. 2003: 464; Gertsch 1992: 105, f, desc. (figs 109–110); Jackman 1997: 162; Paquin and Dupérré 2009: 43, f, desc. (figs 96–97, 135)

####### Distribution.

Uvalde

####### Caves.

Uvalde (North Well Cave, Picture Cave No. 1)

####### Time of activity.

Female (April, November)

####### Habitat.

(landscape features: cave)

####### Type.

Texas (female, Uvalde Co., Picture Cave No. 1, November 3, 1962, J. Reddell, holotype, AMNH)

[male unknown]

####### Etymology.

Latin, serene

####### Collection.

TMM

###### Cicurina sheari

Taxon classificationAnimaliaAraneaeDictynidae

Gertsch, 1992

Cicurina sheari Culver et al. 2003: 464; Gertsch 1992: 115, f, desc. (figs 135–136); Jackman 1997: 162; Paquin and Dupérré 2009: 44, f, desc. (figs 98–99, 136)

####### Distribution.

Real

####### Caves.

**Real** (Ramsey Bat Cave)

####### Time of activity.

Female (October)

####### Habitat.

(landscape features: cave)

####### Type.

Texas (female, Real Co., Ramsey Bat Cave, October 2, 1976, D. Pate, R. Hemperly, K. Heuss, holotype, AMNH)

[male unknown]

####### Etymology.

Person (Named for William A. Shear, student of spider behavior and evolution, Gertsch 1992).

####### Collection.

TMM

###### Cicurina sintonia

Taxon classificationAnimaliaAraneaeDictynidae

Gertsch, 1992

Cicurina sintonia Gertsch 1992: 95, mf, desc. (figs 25–26, 47–48, chart 1); Jackman 1997: 162

####### Distribution.

San Patricio

####### Time of activity.

Male (November); female (November)

####### Type.

Texas (female, San Patricio Co., Sinton, November 20, 1959, H. E. Laughlin, holotype, AMNH)

####### Etymology.

locality (Specific name for Sinton, Texas, Gertsch 1992).

###### Cicurina sprousei

Taxon classificationAnimaliaAraneaeDictynidae

Gertsch, 1992

Cicurina sprousei Culver et al. 2003: 464; Gertsch 1992: 113, f, desc. (figs 119–120); Jackman 1997: 162; Paquin and Dupérré 2009: 44, f, desc. (figs 100–101, 136)

####### Distribution.

Bandera, Bexar, Travis, Williamson

####### Caves.

**Bandera** (Station “C” Cave)

####### Time of activity.

Female (June, August – October)

####### Habitat.

(landscape features: cave)

####### Type.

Texas (female, Bandera Co., Station “C” Cave, September 4, 1988, P. Sprouse, holotype, AMNH)

[male unknown]

####### Etymology.

Person (Specific name for Peter Sprouse, student of caves, Gertsch 1992).

####### Collection.

TMM, TTU

###### Cicurina stowersi

Taxon classificationAnimaliaAraneaeDictynidae

Gertsch, 1992

Cicurina stowersi Gertsch 1992: 113, f, desc. (figs 121–122); Jackman 1997: 162; Paquin and Dupérré 2009: 46, f, desc. (figs 102–103, 136)

####### Distribution.

Kerr

####### Caves.

**Kerr** (Stowers Cave)

####### Time of activity.

Female (May)

####### Habitat.

(landscape features: cave)

####### Type.

Texas (female, Kerr Co., Stowers Cave, May 3, 1969, R. Bartholomew, holotype, AMNH)

[male unknown]

####### Etymology.

locality (Specific name for Stowers Cave, Gertsch 1992).

####### Collection.

TMM

###### Cicurina suttoni

Taxon classificationAnimaliaAraneaeDictynidae

Gertsch, 1992

Cicurina suttoni Culver et al. 2003: 464; Gertsch 1992: 115, mf, desc. (figs 137–138, 151–152); Jackman 1997: 162; Paquin and Dupérré 2009: 46, f, desc. (figs 104–105, 137)

####### Distribution.

Sutton

####### Caves.

**Sutton** (Felton Cave)

####### Time of activity.

Male (October); female (July)

####### Habitat.

(landscape features: cave, rotting root in cave)

####### Type.

Texas (female, Sutton Co., Felton Cave, July 4, 1964, J. Reddell, holotype, AMNH)

####### Etymology.

locality (Specific name for Sutton County, Gertsch 1992).

####### Collection.

TMM

###### Cicurina texana

Taxon classificationAnimaliaAraneaeDictynidae

(Gertsch, 1935)

Cicurina texana Calixto et al. 2013: 181; Chamberlin and Ivie 1940: 78, m, desc. (fig. 90) [part – not f, fig. 64, see Cicurina dorothea]; Gertsch 1992: 92 [T], m, desc. (figs 43–44); Jackman 1997: 162; Roewer 1955: 53; Roth and Brown 1986: 8; Vogel 1970b: 2
Chorizomma texana Gertsch, 1935; Gertsch 1935a: 15, m, desc. (figs 36–37); Vogel 1970b: 2
Chorizomma texanum Gertsch, 1935; Bonnet 1956: 1077

####### Distribution.

Llano, Robertson

####### Locality.

Holmes Pecan Orchard

####### Time of activity.

Male (October, December)

####### Habitat.

(orchard: pecan)

####### Method.

pitfall trap [m]

####### Type.

Texas (male, Llano Co., Llano, December 1934, L. I. Davis, holotype, AMNH)

[female unknown]

####### Etymology.

locality (state)

####### Collection.

TAMU

###### Cicurina travisae

Taxon classificationAnimaliaAraneaeDictynidae

Gertsch, 1992

Cicurina travisae Culver et al. 2003: 464; Gertsch 1992: 101, f, desc. (figs 63–70); Hedin 2015: 348, 354 [S]; Jackman 1997: 162; Paquin and Dupérré 2009: 47, f, desc. (figs 106–107, 131); Paquin and Hedin 2005b: 10; SWCA Environmental Consultants 2007: 1–2, 3–32
Cicurina reddelli Gertsch, 1992; Culver et al. 2003: 464; Gertsch 1992: 105, f, desc. (figs 77–78); Jackman 1997: 162; Paquin and Dupérré 2009: 40, f, desc. (figs 86–87, 131); Paquin and Hedin 2004: 3254
Cicurina wartoni Gertsch, 1992; Culver et al. 2003: 464; Gertsch 1992: 101, f, desc. (figs 75–76); Jackman 1997: 162, 171; Paquin and Dupérré 2009: 55, f, desc. (figs 122–123–131)

####### Distribution.

Travis, Williamson

####### Caves.

**Travis** (Amber Cave, Broken Arrow Cave, Cotterell Cave, Kretschmarr Cave, Kretschmarr Double Pit, McDonald Cave (=Schulze Cave), North Root Cave, Pickle Pit, Pisarowicz Cave, Root Cave, Salamander Cave, Spider Cave, Tooth Cave); **Williamson** (Testudo Cave)

####### Time of activity.

Female (January – June, August, December)

####### Habitat.

(landscape features: cave)

####### Type.

Texas (female, Travis Co., Tooth Cave, August 5, 1963, J. Reddell, holotype, AMNH)

[male unknown]

####### Etymology.

Person (Specific name for Mrs. Nevenna Tsanoff Travis, tireless sponsor for preservation of caves, Gertsch 1992).

####### Collection.

TMM

###### Cicurina troglobia

Taxon classificationAnimaliaAraneaeDictynidae

Cokendolpher, 2004

Cicurina troglobia Cokendolpher 2004b: 60, f, desc. (figs 1–3); Paquin and Dupérré 2009: 47, f, desc. (figs 108–109, 130); Paquin and Hedin 2004: 3254
Cicurina spp.; Cokendolpher and Reddell 2001b: 37

####### Distribution.

Bell

####### Caves.

**Bell** (Seven Mile Mountain Cave)

####### Time of activity.

Female (June)

####### Habitat.

(landscape features: cave)

####### Type.

Texas (female, Bell Co., Seven Mile Mountain Cave, June 28, 2000, J. Reddell, M. Reyes, molted to maturity July 14, 2001, holotype, AMNH)

[male unknown]

####### Etymology.

Greek, cave and life

####### Collection.

TTU

###### Cicurina ubicki

Taxon classificationAnimaliaAraneaeDictynidae

Gertsch, 1992

Cicurina ubicki Culver et al. 2003: 464; Gertsch 1992: 109, f, desc. (figs 87–88); Jackman 1997: 162; Paquin and Dupérré 2009: 48, f, desc. (figs 110–111, 133)

####### Distribution.

Hays

####### Caves.

**Hays** (Fern Cave, McGlothlin Cave)

####### Time of activity.

Female (May, September)

####### Habitat.

(landscape features: cave)

####### Type.

Texas (female, Hays Co., Fern Cave, September 2, 1989, D. Ubick, S. Fend, S. Renkes, holotype, AMNH)

[male unknown]

####### Etymology.

Person (Specific name for Darrell Ubick, collector of many cave spiders, Gertsch 1992).

####### Collection.

TMM

###### Cicurina uvalde

Taxon classificationAnimaliaAraneaeDictynidae

Gertsch, 1992

Cicurina uvalde Culver et al. 2003: 464; Gertsch 1992: 103, f, desc. (figs 101–102); Jackman 1997: 162; Paquin and Dupérré 2009: 48, f, desc. (figs 112–113, 135)

####### Distribution.

Uvalde

####### Caves.

**Uvalde** (Rambie’s Cave)

####### Time of activity.

Female (April, August – September)

####### Habitat.

(landscape features: cave)

####### Type.

Texas (female, Uvalde Co., Rambie’s Cave, April 6, 1963, J. Reddell, D. McKenzie, holotype, AMNH)

[male unknown]

####### Etymology.

locality (Specific name for Uvalde County, Gertsch 1992).

####### Collection.

TMM

###### Cicurina varians

Taxon classificationAnimaliaAraneaeDictynidae

Gertsch & Mulaik, 1940

Cicurina varians Barr and Reddell 1967: 260; Broussard and Horner 2006: 253; Chamberlin and Ivie 1940: 57, mf, desc. (figs 42, 82, 93, 95); Cokendolpher 2004a: 33, f (figs 12, 21–27); Cokendolpher and Polyak 2004: 189; Cokendolpher and Reddell 2001b: 44; Gertsch 1992: 81, (chart 1); Jackman 1997: 162; Kunath and Smith 1968: 37–38, 44, 80, 103; McKenzie and Reddell 1964: 7, 15, 22, 47, 49; Paquin and Hedin 2004: 3255; Reddell 1964: 7, 11, 33–35, 38, 41; Reddell 1965: 169; Reddell 1967: 14, 23, 26–27, 50, 54; Reddell 1970: 404; Reddell 1973: 29, 56, 69, 79, 111, 115; Reddell 1994: 6; Reddell and Cokendolpher 2004: 82; Reddell and Finch 1963: 8–9, 21, 25, 28, 30, 40–41, 43, 48, 50, 53–54; Reddell and Smith 1965: 20, 33, 46; Richman et al. 2011a: 47; Roberts 2001: 49; Smith and Reddell 1971: 21, 24–25, 29, 31, 41; Vogel 1967: 9; Vogel 1970b: 2; White et al. 2009: 341; Yantis 2005: 196

####### Distribution.

Widespread in caves; Anderson, Bandera, Bee, Bell, Bexar, Blanco, Brewster, Burnet, Cherokee, Childress, Comal, Concho, Coryell, Crockett, Culberson, Dallas, Edwards, Gillespie, Hardeman, Hays, Irion, Jeff Davis, Jim Wells, Kendall, Kerr, Kimble, King, Kinney, Lampasas, Llano, Mason, Medina, Menard, Pecos, Potter, Presidio, Randall, Real, San Saba, Schleicher, Sutton, Terrell, Travis, Uvalde, Val Verde, Wheeler, Wichita, Williamson

####### Locality.

Camp Bullis, Chihuahuan desert, Dalquest Research Site, Fort Hood, Lost Maples State Park, Raven Ranch, Wildcat Bluff Nature Center

####### Caves.

**Bandera** (Emmett Wilson Cave, Fog Fissure, Fossil Cave, Garrison Hilltop Cave, Station “C” Cave No. 1); **Bell** (Adam’s Gold Mine, Black Cave, Camp 6 Cave No. 1 [Fort Hood], Figure 8 Cave [Fort Hood], Fools Cave [Fort Hood], Gnarla Cave [Fort Hood], Hill’s Cave, Jagged Walls Cave [Fort Hood], Moffatt Pit Cave [Fort Hood], Nolan Creek Cave [Fort Hood], Price Pit Cave [Fort Hood], Root Sink [Fort Hood], Rugger’s Rift Cave, Sledgehammer Cave [Fort Hood], Sparta Cave [Fort Hood], Streak Cave [Fort Hood], Talking Crows Cave [Fort Hood], Tres Dedos Cave [Fort Hood], Valentine Cave [Fort Hood], Viper Den Cave [Fort Hood]); **Bexar** (Assassin Cave, B-52 Cave [Camp Bullis], Banzai Mud Dauber Cave [Camp Bullis], Bear Cave, Black Cat Cave, Boneyard Pit [Camp Bullis], Breached Dam Cave, Bunny Hole [Camp Bullis], Caracol Creek Coon Cave, Constant Sorrow Cave, Cross the Creek Cave [Camp Bullis], Dangerfield Cave [Camp Bullis], Dirtwater Cave, Dogleg Cave [Camp Bullis], Eagles Nest Cave [Camp Bullis], Friesenhahn Cave, Glinn’s Gloat Hole [Camp Bullis], Goat Cave [Government Canyon State Natural Area], Government Canyon Bat Cave [Government Canyon State Natural Area], Han’s Grotto, Headquarters Cave [Camp Bullis], Hector’s Hole [Camp Bullis], Hilger Hole [Camp Bullis], Hills and Dales Pit, Hitzfelder’s Bone Hole [=Hitzfelder Cave], Hold Me Back Cave [Camp Bullis], Hornet’s Last Laugh Pit, Isocow Cave [Camp Bullis], Isopit, Kamikazi Cricket Cave, Lone Gunman Pit [Camp Bullis], Low Priority Cave [Camp Bullis], MARS Shaft [Camp Bullis], Madla’s Cave, Mattke Cave, Max and Roberts Cave, Niche Cave, One Formation Cave [Government Canyon State Natural Area], Peace Pipe Cave, Platypus Pit [Camp Bullis], Porcupine Parlor Cave, Raging Cajun Cave [=Rajin’ Cajun Cave], Robber Baron Cave, Robbers Cave, Root Canal Cave [Camp Bullis], Some Monk Chanted Evening Cave, Stevens Ranch Cave No. 1, Stone Oak Parkway Pit, Strange Little Cave [Camp Bullis], Sunless City Cave, Tall Tales Cave, Twin Pits, Up the Creek Cave [Camp Bullis], Vera Cruz Shaft [Camp Bullis], Well Done Cave, Winston’s Cave [Camp Bullis], Wurzbach Bat Cave); **Blanco** (Davis Blowout Cave, Llewellyn Cave, T Cave); **Brewster** (O.T.L. Cave, Split Tank Cave); **Burnet** (Beaver Creek Bat Cave, Crossing Cave, Duncan’s Flea Cave, Fenceline Sink, Longhorn Caverns, Marble Falls Cave No. 3, Persimon Sink, Pie Cave, Porcupine Cave, Shin Oak Sink, Simon Says Sink No. 2, Simons 1174 Sink, Simons Rattlesnake Well, Simons Squeeze-Down Pit, Simons Squirm-Around Cave, Snake Pit Sink, Snelling’s Cave, Tree Ladder Sink, Wagon Trail Cave); **Childress** (Black Hand Cave, Buzzard Wall Cave); **Comal** (Bear Creek Cave, Bracken Bat Cave, Brehmmer-Heidrich Cave, Camp Bullis Cave No. 3, Coreth Bat Cave, Deepwater Cave, Ebert Cave, Fischer Cave, Hitzfielder’s Cave, Kappelman Cave, Kappelman Salamander Cave, Klar’s Cave, Lewis Cave, Little Gem Cave, Natural Bridge Caverns, Startzville Bat Cave, Washington Cave, Wyley’s Cave); **Coryell** (Brokeback Cave [Fort Hood], Chigiouxs’ Cave [Fort Hood], Copperhead Cave No. 2 [Fort Hood], Diamond Cave, Egypt Cave [Fort Hood], Gann Cave [Fort Hood], Mixmaster Cave [Fort Hood], Oxygen Bottle Cave, Rocket River Cave System (Double Tree Cave, Rocket River Cave) [Fort Hood], Rocket River Cave System [Fort Hood], Runoff Cave [Fort Hood], Saltpeter Cave [Fort Hood], Shell Mountain Bat Cave [Fort Hood], Tippit Cave [Fort Hood]); **Crockett** (Dudley Cave, Ketchum Cave); **Culberson** (Decent Cave, East Mill Cave); **Edwards** (Deep Cave, Devil’s Sinkhole, Dunbar Cave, Hughes Cave, Jacoby Cave, Punkin Cave, 3-Bounce Pit); **Gillespie** (Cave Creek Mosquito Cave); **Hardeman** (Walkup Cave); **Hays** (Boggus Cave, Boyett’s Cave, Donaldson Cave, Ezell’s Cave, Halifax Bat Cave, Hunter Uncave, McCarty Cave, Morton’s Cave, Nance Bat Cave, Wimberly Bat Cave); **Irion** (Arden Cave); **Jeff Davis** (Bloys Camp Cave); **Kendall** (Cascade Caverns, Cave-Without-A-Name–Dead Man’s Cave System, Century Caverns, Cricket Cave, Forget-Me-Not Cave, Gertrude’s Unknown Cave, Kohl Ranch Cave No. 1, Pfeiffer Crawlway Cave, Schneider Ranch Cave, Schwarz Cave, Swaglet Cave); **Kerr** (East Trap Cave, Goat Trap Cave, Mingus Root Cave, Old Morris Cave, Secrest Cave, Seven Room Cave, Stowers Cave); **Kimble** (Fleming Bat Cave, Garter Snake Cave, The Hole, Live Dog Cave, Lizard Cave, Llewelyn Rose Cave, Top Dog Cave); **King** (River Styx Cave); **Kinney** (Bader Cave, Cricket Siphon Cave, Rattlesnake Cave, Webb Cave); **Lampasas** (Enough Cave); **Llano** (Miller’s Cave); **Mason** (Kothmann Cave, Mill Creek Cavern); **Medina** (Coontop Tip, Haby Bat Cave, Koch Cave, Lutz Cave, Ney Cave, Valdina Farms Sinkhole, Weynand Cave); **Menard** (Celery Creek Cave, Kearney’s Dead Goat Cave, Neel Cave and Powell’s Cave); **Pecos** (Amazing Maze Cave); **Real** (Cave of the Lakes, Emmett Wilson Cave, Haby Cave, Orell Bat Cave, Ramsey Bat Cave, Section 6 Cave, Skeleton Cave, Tucker Hollow Cave); **San Saba** (Cicurina Cave, Gorman Cave, Harrell’s Cave, Lemon’s Cave, Puberty Pit, Springdale Ranch Cave, Upper Cave, Whiteface Cave); **Schleicher** (Cave Y); **Sutton** (Felton Cave, Harrison Cave, Silky Cave); **Terrell** (Goode Cave, Longley Cave, Pasotex Pit, Wizard’s Well); **Travis** (Adobe Springs Cave, Airman’s Cave, Amber Cave, Arrow Cave, Bandit Cave, Beckett’s Cave, Bee Creek Cave, Beer Bottle Cave, Brew Pot Sink, Broken Arrow Cave, Cave Y, Cold Cave, Dead Dog Cave No. 1, Driskill Cave, Fossil Cave, Gallifer Cave, Goat Cave, Grove Sinks Cave, Hideout Cave, Hole in the Road, Ireland’s Cave, Jest John Cave, Ken Harrell Cave, Kretschmarr Fluted Sink, Kretschmarr Salamander Cave, Kretschmarr Sink, LaCrosse Cave, Lost Gold Cave, Lunsford’s Cave, McDonald Cave, McNeil Bat Cave, Maple Run Cave, Moss Pit, New Comanche Trail Cave, Night Sink, No Rent Cave, Northwoods Cave, Rolling Rock Cave, Schulze Cave, Spanish Wells, Stark’s North Mine, Stovepipe Cave, Three-Holer Cave, Tooth Cave, Twin Dig Pit, Wade Sink, Weldon Cave, Weldon West Cave, Whirlpool Cave); **Uvalde** (BFS Cave, Burial Cave, Carson Cave, Grape Hollow Cave, Indian Creek Cave [questionable], Maybe Stream Cave, Picture Cave No. 1, Rambie’s Cave, Sandtleben Cave, Tampke Ranch Cave, West Holler Cave); **Val Verde** (Arledge Bat Cave, Cave Hollow Cave, Centipede Cave, Emerald Sink, Fern Cave, H. T. Miers Cave, Langtry Lead Cave, Langtry Quarry Cave, Litter Barrel Cave, Oriente Milestone Molasses Bat Cave, Robertson Mill Dirt Cave, Twin Tree Cave); **Wheeler** (Small Mouth Cave); **Williamson** (Ballroom Cave No. 2 [questionable], Bat Well, Beck Bat Cave, Beck Horse Cave, Beck Ranch Cave, Beck’s Sewer Cave, Blue Wasp Cave, Bone Cave [questionable], Broken Knife Sink, Chinaberry Cave [questionable], Cobb Caverns, Coffin Cave, Core Barrel Cave, Cricket Cave, Dead Ash Cave, Desert Dune Cave, Elm Cave, Elm Bat Cave, Elm Water Cave, Fern Bluff Cave, Flint Wash Cave, Four-Corners Cave, Jug Cave, Life Station Cave, Lorfing’s Unseen Rattler Cave, Marigold Cave, Man-With-A-Spear Cave, McNeil Quarry Cave, Mosquito Cave, Muscle Sink, Pussy Cat Cave, Raccoon Cave, Ramsel’s Corral Cave, Rattlesnake Filled Cave, Steam Cave, Sunless City Cave, Susana Cave, Temples of Thor Cave, Terrell’s Cave, Texella Cave, The Chimney, Three-Mile Cave, Walsh Ranch Cave, Williams Cave, Wolf Cave, Wolf’s Rattlesnake Cave)

####### Time of activity.

Male (January – May, August – December); female (January – June, August – December)

####### Habitat.

(landscape features: cave); (nest/prey: bird nest); (soil/woodland: leaf litter, pine woods [%: 60])

####### Method.

5 gallon bucket trap [m]; berlese funnel; pitfall trap [mf]

####### Type.

Texas (female, Kerr Co., Raven Ranch, December 1939, D. & S. Mulaik, holotype)

####### Etymology.

Latin, spines vary from typical formula

####### Collection.

MSU, TAMU, TMM, TTU

###### Cicurina venefica

Taxon classificationAnimaliaAraneaeDictynidae

Gertsch, 1992

Cicurina venefica Gertsch 1992: 114, f, desc. (figs 129–130); Jackman 1997: 162; Paquin and Dupérré 2009: 50, f, desc. (figs 114–115, 137)

####### Distribution.

Terrell

####### Caves.

**Terrell** (Wizard’s Well)

####### Time of activity.

Female (February)

####### Habitat.

(landscape features: cave)

####### Type.

Texas (female, Terrell Co., Wizard’s Well, February 12–13, 1983, E. Short, R. Waters, holotype, AMNH)

[male unknown]

####### Etymology.

Latin, a witch

####### Collection.

TMM

###### Cicurina venii

Taxon classificationAnimaliaAraneaeDictynidae

Gertsch, 1992

Cicurina venii Cokendolpher 2004a: 52, f, desc. (figs 63–64); Culver et al. 2003: 464; Federal Register 1998: 71855–71856, 71858, 71860, 71866; Federal Register 2000: 81419–81420, 81425, 81428, 81433; Federal Register 2002: 55064, 55067, 55073, 55075, 55086–55087; Federal Register 2003: 17156–17158, 17175–17176, 17190–17191, 17193; Gertsch 1992: 111, f, desc. (figs 95–96); Jackman 1997: 162, 171; NABN 2001: 8; Paquin and Dupérré 2009: 52, f, desc. (figs 116–117, 134); Reddell and Cokendolpher 2004: 81; SWCA Environmental Consultants 2007: 3

####### Distribution.

Bexar

####### Caves.

**Bexar** (Braken Bat Cave)

####### Time of activity.

Female (November)

####### Habitat.

(landscape features: cave)

####### Type.

Texas (female, Bexar Co., Braken Bat Cave, November 22, 1980, G. Veni, holotype, AMNH)

[male unknown]

####### Etymology.

Person (Specific name for George Veni, student of Texas caves, Gertsch 1992).

####### Collection.

TMM

###### Cicurina vespera

Taxon classificationAnimaliaAraneaeDictynidae

Gertsch, 1992

Cicurina vespera Cokendolpher 2004a: 53, f, desc. (figs 65–66); Culver et al. 2003: 464; Federal Register 1998: 71855–71856, 71858, 71860, 71866; Federal Register 2000: 81419–81421, 81425, 81428, 81433; Federal Register 2002: 55064, 55067, 55073–55074, 55086–55087, 55089; Federal Register 2003: 17156–17158, 17176, 17190; Gertsch 1992: 111, f, desc. (figs 93–94); Jackman 1997: 162, 171; NABN 2001: 8; Paquin and Dupérré 2009: 53, f, desc. (figs 118–119, 134); Paquin and Hedin 2004: 3254; Reddell and Cokendolpher 2004: 81; SWCA Environmental Consultants 2007: 3

####### Distribution.

Bexar

####### Caves.

**Bexar** (Government Canyon Bat Cave [Government Canyon State Natural Area])

####### Time of activity.

Female (August)

####### Habitat.

(landscape features: cave)

####### Type.

Texas (female, Bexar Co., Government Canyon Bat Cave, August 11, 1965, J. Reddell, J. Fish, holotype, AMNH)

[male unknown]

####### Etymology.

Latin, in the evening

####### Collection.

TMM

###### Cicurina vibora

Taxon classificationAnimaliaAraneaeDictynidae

Gertsch, 1992

Cicurina vibora Culver et al. 2003: 464; Gertsch 1992: 98, f, desc. (figs 55–56); Jackman 1997: 162; Paquin and Dupérré 2009: 53, f, desc. (figs 120–121, 130); Paquin and Hedin 2004: 3254; Paquin and Hedin 2005b: 10; SWCA Environmental Consultants 2007: 1–2, 3–32

####### Distribution.

Williamson

####### Caves.

**Williamson** (Rattlesnake Filled Cave, Sunless City Cave, Temples of Thor Cave)

####### Time of activity.

Female (April – May, August)

####### Habitat.

(landscape features: cave)

####### Type.

Texas (female, Williamson Co., Rattlesnake Filled Cave, August 24, 1963, J. Reddell, W. Russell, holotype, AMNH)

[male unknown]

####### Etymology.

Mexican, viper

####### Collection.

TMM

####### Note.

Sunless City Cave is in Williamson Co. not Bexar Co. as in Paquin and Hedin 2004: 3254.

###### Cicurina watersi

Taxon classificationAnimaliaAraneaeDictynidae

Gertsch, 1992

Cicurina watersi Culver et al. 2003: 464; Gertsch 1992: 103, f, desc. (figs 103–104); Jackman 1997: 163; Paquin and Dupérré 2009: 55, f, desc. (figs 124–125, 135)

####### Distribution.

Uvalde

####### Caves.

**Uvalde** (Frio Queen Cave)

####### Habitat.

(landscape features: cave)

####### Type.

Texas (female, Uvalde Co., Frio Queen Cave, summer 1983, R. M. Waters, holotype, AMNH)

[male unknown]

####### Etymology.

Person (Specific name for the collector, Randy M. Waters, Gertsch 1992).

####### Collection.

TMM

##### Genus *Dictyna* Sundevall, 1833

###### Dictyna annexa

Taxon classificationAnimaliaAraneaeDictynidae

Gertsch & Mulaik, 1936

Dictyna annexa Agnew et al. 1985: 3; Bonnet 1956: 1426; Breene et al. 1993a: 169; Breene et al. 1993b: 647; Breene et al. 1993c: 14, 47, 53, mf, (figs 10A-B); Breene et al. 1994: 8; Calixto et al. 2013: 181; Chamberlin and Gertsch 1958: 92, mf, desc. (pl. 25, figs 7–9); Dean and Sterling 1987: 6; Gertsch and Mulaik 1936a: 6, m, desc. (fig. 8); Jackman 1997: 163; Roewer 1955: 1318; Vogel 1970b: 7; Young and Edwards 1990: 16
Dictyna idahoana Chamberlin & Ivie, 1933; Gertsch and Mulaik 1940: 331; Roewer 1955: 1321 [Texas records]
Dictyna anexa Gertsch & Mulaik, 1936; Knutson et al. 2010: 515

####### Distribution.

Widespread; Archer, Baylor, Brown, Cameron, Collingsworth, Comanche, Coryell, Erath, Frio, Hidalgo, Howard, Jim Wells, La Salle, Llano, Reagan, Runnels, Scurry, Starr, Travis, Wichita, Zapata

####### Locality.

Bill Haney Pecan Orchard, Lake Thomas

####### Time of activity.

Male (March – October); female (March – November)

####### Habitat.

(crops: cotton, peanuts, sugarcane, watermelon); (grass: grass); (orchard: citrus, pecan); (plants: miscellaneous vegetation, roadside vegetation, *Baccharis*); (soil/woodland: juniper, saltcedar, post oak savanna with pasture, *Hibiscus* sp., *Ulmus crassifolia*); (structures: near blacklight trap)

####### Method.

D-Vac suction [m]; fogging [mf]; pitfall trap [mf]; suction trap [m]; sweeping [mf]

####### Eggs/spiderlings.

Erath [7 spiderlings in eggsac] [TAMU]

####### Type.

Texas (male, Hidalgo Co., 5 miles W Edinburg, July 4, 1935, S. Mulaik, holotype, AMNH)

####### Etymology.

Latin, bind

####### Collection.

DMNS, MSU, TAMU

###### Dictyna bellans

Taxon classificationAnimaliaAraneaeDictynidae

Chamberlin, 1919

Dictyna bellans Agnew et al. 1985: 3; Breene et al. 1993b: 647; Breene et al. 1994: 8; Chamberlin and Gertsch 1958: 62, mf, desc. (pl. 16, figs 4–7); Jackman 1997: 163; Knutson et al. 2010: 515; Pfannenstiel 2008a: 204; Young and Edwards 1990: 16
Dictyna longispina Emerton, 1888; Gertsch and Mulaik 1940: 329; Jones 1936: 69; Kagan 1942: 11; Kagan 1943: 258; Vogel 1970b: 7; Young and Edwards 1990: 16 [misidentified, Texas records]

####### Distribution.

Eastern 2/3 Texas; Archer, Baylor, Brazos, Cameron, Clay, Coleman, Comal, Comanche, Dallas, Erath, Fannin, Hidalgo, Howard, Hunt, Leon, Llano, McLennan, Navarro, Palo Pinto, Runnels, San Saba, Scurry, Travis, Val Verde, Wichita, Willacy

####### Locality.

Bentsen-Rio Grande Valley State Park, Lake Thomas

####### Caves.

**San Saba** (Copperhead Cave)

####### Time of activity.

Male (February, April – December); female (April – May, July – December)

####### Habitat.

(crops: cotton, peanuts, sugarcane); (grass: grass); (landscape features: cave); (plants: miscellaneous vegetation, *Baccharis*); (soil/woodland: juniper, saltcedar, trees/shrubs, woods, *Hibiscus* sp., *Ulmus crassifolia*)

####### Method.

Beating [m]; D-Vac suction [mf]; pitfall trap [f]; sweeping [m]

####### Type.

Mississippi, Canton

####### Etymology.

Latin, behavior, film

####### Collection.

DMNS, MSU, TAMU, TMM

###### Dictyna bostoniensis

Taxon classificationAnimaliaAraneaeDictynidae

Emerton, 1888

Dictyna bostoniensis Agnew et al. 1985: 3; Chamberlin and Gertsch 1958: 78, mf, desc. (pl. 20, figs 1–11); Gertsch and Mulaik 1940: 332; Jackman 1997: 163; Vogel 1970b: 7; Young and Edwards 1990: 16

####### Distribution.

Comanche, Dallam, Hemphill, Travis

####### Time of activity.

Male (July); female (March, May, July)

####### Habitat.

(crops: peanuts); (soil/woodland: *Juniperus ashei*, *Ulmus crassifolia*)

####### Method.

Beating [f]; sweeping [f]

####### Type.

Massachusetts, Boston

####### Etymology.

locality (city)

####### Collection.

TAMU

###### Dictyna calcarata

Taxon classificationAnimaliaAraneaeDictynidae

Banks, 1904

Dictyna calcarata Chamberlin and Gertsch 1958: 64, mf, desc. (pl. 17, figs 1–6); Gertsch and Mulaik 1940: 330; Jackman 1997: 163; Jones 1936: 69; Jones 1948: 30; Kaston 1972: 82, desc.; Kaston 1978: 83, desc.; Roewer 1955: 1319
Dictyna calcerata Banks, 1904; Vogel 1970b: 7

####### Distribution.

Widespread; Archer, Baylor, Brazos, Burnet, Cameron, Comal, Dallas, Hidalgo, Kleberg, Llano, Lubbock, Reeves, Robertson, Rusk, Terrell, Travis, Webb, Wichita

####### Locality.

Holmes Pecan Orchard, Lake Buchanan

####### Time of activity.

Male (April – May, July – September); female (July – August, October – December)

####### Habitat.

(orchard: pecan); (structures: outside wall)

####### Type.

California, San Pedro

####### Etymology.

Latin, furnished with a spur (on palpus)

####### Collection.

MSU, TAMU, TTU

###### Dictyna cholla

Taxon classificationAnimaliaAraneaeDictynidae

Gertsch & Davis, 1942

Dictyna cholla Chamberlin and Gertsch 1958: 68 [S], mf, desc. (pl. 17, figs 7–9); Jackman 1997: 163; Roewer 1955: 1316; Vogel 1967: 49; Vogel 1970b: 7
Dictyna hardyi Gertsch, 1946; Gertsch 1946a: 17, mf, desc. (figs 19–20)

####### Distribution.

Cameron

####### Locality.

Laguna Madre

####### Time of activity.

Male (August); female (August)

####### Habitat.

(nest/prey: nest of *Neotoma micropus* [mf])

####### Type.

Mexico, Sonora, 27 miles S Nogales

####### Etymology.

Probably after cactus common name, *cholla* for species

###### Dictyna coloradensis

Taxon classificationAnimaliaAraneaeDictynidae

Chamberlin, 1919

Dictyna coloradensis Bradley 2013: 118; Chamberlin and Gertsch 1958: 89 [S], mf, desc. (pl. 26, figs 4–7); Cokendolpher et al. 2008: 8, 18 (photo 16, fig. 5); Jackman 1997: 163; Knutson et al. 2010: 515; Vogel 1970b: 7
Dictyna marxi Jones, 1947; Jones 1948: 30, mf, desc. (figs 82–85, 87)

####### Distribution.

North-central, east, and southeast Texas; Anderson, Archer, Bastrop, Baylor, Bosque, Brown, Burleson, Carson, Howard, Kenedy, Lampasas, Palo Pinto, Runnels, Scurry, Travis, Wichita

####### Locality.

Lake Thomas

####### Time of activity.

Male (March, May); female (February, April – August)

####### Habitat.

(littoral: near playa); (grass: grassland); (plants: miscellaneous vegetation, roadside vegetation); (soil/woodland: juniper, saltcedar, trees/shrubs, willow)

####### Method.

Beating [f]; pitfall trap; sweeping [mf]

####### Type.

Colorado, Colorado Springs

####### Etymology.

locality (state)

####### Collection.

DMNS, MSU, NMSU, TAMU

###### Dictyna foliacea

Taxon classificationAnimaliaAraneaeDictynidae

(Hentz, 1850)

Dictyna foliacea Bradley 2013: 118; Chamberlin and Gertsch 1958: 73 [S], mf, desc. (pl. 19, figs 8–13); Gertsch and Mulaik 1940: 329; Jackman 1997: 163; Kaston 1972: 80, desc. (fig. 185); Kaston 1978: 82, desc. (fig. 203); Vogel 1970b: 7
Dictyna frondea Emerton, 1888; Gertsch and Mulaik 1940: 332

####### Distribution.

East and north-central Texas; Angelina, Collingsworth, Dallas, Nacogdoches, Robertson, San Augustine, Young

####### Time of activity.

Female (May – July)

####### Type.

Alabama

####### Etymology.

Latin, found to make web in hollow of leaves

####### Collection.

MSU

###### Dictyna formidolosa

Taxon classificationAnimaliaAraneaeDictynidae

Gertsch & Ivie, 1936

Dictyna formidolosa Agnew et al. 1985: 6; Chamberlin and Gertsch 1958: 66, mf, desc. (pl. 16, figs 8–10, pl. 17, fig. 12); Gertsch and Mulaik 1940: 328; Jackman 1997: 163; Roewer 1955: 1317; Vogel 1970b: 7; Yantis 2005: 200

####### Distribution.

Angelina, Bandera, Burleson, Erath, Fort Bend, Jasper, Leon, Montgomery

####### Locality.

Lost Maples State Park

####### Time of activity.

Male (March 30-April 6, April – May); female (August)

####### Habitat.

(soil/woodland: loblolly pine unmanaged, post oak savanna with pasture, post oak woods [%: 92], under oak)

####### Method.

5 gallon bucket trap [m]; carrion pitfall trap; pitfall trap [m] (under oak [m])

####### Type.

North Carolina, Black Mountain

####### Etymology.

Latin, causing fear

####### Collection.

TAMU

###### Dictyna personata

Taxon classificationAnimaliaAraneaeDictynidae

Gertsch & Mulaik, 1936

Dictyna personata Bonnet 1956: 1447; Chamberlin and Gertsch 1958: 95, mf, desc. (pl. 28, figs 1–4); Gertsch and Mulaik 1936a: 9, f, desc. (fig. 3); Gertsch and Mulaik 1940: 329, m (figs 20–21); Jackman 1997: 163; Roewer 1955: 1323; Vogel 1970b: 8

####### Distribution.

Hidalgo, Llano, Zapata

####### Time of activity.

Male (July); female (July – September)

####### Type.

Texas (female, Zapata Co., 30 miles SE Laredo, August 4, 1935, S. Mulaik, holotype, AMNH)

####### Etymology.

Latin, of a person

###### Dictyna secuta

Taxon classificationAnimaliaAraneaeDictynidae

Chamberlin, 1924

Dictyna secuta Chamberlin and Gertsch 1958: 97 [S], mf, desc. (pl. 27, figs 1–6); Jackman 1997: 163; Roewer 1955: 1317; Vogel 1967: 46; Vogel 1970b: 8
Dictyna bishopi Gertsch & Mulaik, 1940; Gertsch and Mulaik 1940: 328, f, desc. (figs 30, 32)

####### Distribution.

Brewster, El Paso

####### Time of activity.

Female (June)

####### Type.

Mexico, Gulf of California, San Esteban Island

####### Etymology.

Latin, followed

###### Dictyna sylvania

Taxon classificationAnimaliaAraneaeDictynidae

Chamberlin & Ivie, 1944

Dictyna sylvania Chamberlin and Gertsch 1958: 98, m, desc. (pl. 26, figs 9–11); Jackman 1997: 163

####### Distribution.

Panola

####### Time of activity.

Male (May)

####### Type.

Georgia, 1 mile N Sylvania

[female unknown]

####### Etymology.

locality (city)

###### Dictyna terrestris

Taxon classificationAnimaliaAraneaeDictynidae

Emerton, 1911

Dictyna terrestris Chamberlin and Gertsch 1958: 70, mf, desc. (pl. 18, figs 6–8); Dean and Sterling 1990: 402; Jackman 1997: 163; Vogel 1970b: 8

####### Distribution.

Brazos, Hunt

####### Time of activity.

Male (March, August)

####### Method.

suction trap [m]

####### Type.

New Hampshire, Lake Winnipesaukee, Three Mile Island

####### Etymology.

Latin, of the earth, ground

####### Collection.

TAMU

###### Dictyna volucripes

Taxon classificationAnimaliaAraneaeDictynidae

Keyserling, 1881

Dictyna volucripes Agnew et al. 1985: 3; Bradley 2013: 118; Breene et al. 1993b: 647; Breene et al. 1993c: 15, 47, 54, mf (figs 16A-B); Chamberlin and Gertsch 1958: 87, mf, desc. (pl. 26, figs 1–3); Dean and Sterling 1987: 6; Dean et al. 1982: 254; Gertsch and Mulaik 1940: 331; Jackman 1997: 97, 163; Jones 1936: 69; Jones 1948: 32; Rogers and Horner 1977: 523; Vogel 1970b: 8; Young and Edwards 1990: 17

####### Distribution.

Eastern 2/3 Texas; Anderson, Archer, Bastrop, Bee, Bowie, Brown, Cameron, Clay, Collin, Comanche, Coryell, Dallas, Erath, Harris, Hidalgo, Hunt, Kerr, Llano, Navarro, Scurry, Travis, Walker, Webb, Wichita, Wilbarger, Willacy, Williamson, Zapata

####### Locality.

Bentsen-Rio Grande Valley State Park, Ellis Prison Unit, La Gringa Resaca, Lake Thomas, Lake Travis, Proctor Lake

####### Time of activity.

Male (February – December); female (March – December)

####### Habitat.

(crops: cotton, guar, peanuts, sugarcane); (grass: grass); (landscape features: rocky area); (plants: miscellaneous vegetation, vegetation, *Baccharis*); (soil/woodland: juniper, post oak savanna with pasture)

####### Method.

D-Vac suction [m]; pitfall trap [m]; suction trap [m]; sweeping [mf]

####### Type.

Massachusetts, Blue Hills

####### Etymology.

Latin, swift

####### Collection.

DMNS, MSU, TAMU

##### Genus *Emblyna* Chamberlin, 1948

###### Emblyna annulipes

Taxon classificationAnimaliaAraneaeDictynidae

(Blackwall, 1846)

Emblyna annulipes Platnick 1993: 556 [T]
Dictyna annulipes Blackwall, 1888 [Chamberlin and Gertsch 1958: 123 [S], mf, desc. (pl. 37, figs 1–5)]
Dictyna muraria Emerton, 1888; Jones 1936: 69

####### Distribution.

Comanche, Dallas, Wichita

####### Locality.

Bill Haney Pecan Orchard

####### Time of activity.

Female (July)

####### Habitat.

(orchard: pecan)

####### Method.

Fogging [f]

####### Type.

Canada: Ontario, Toronto

####### Etymology.

Latin, ring, annulus

####### Collection.

MSU, TAMU

###### Emblyna callida

Taxon classificationAnimaliaAraneaeDictynidae

(Gertsch & Ivie, 1936)

Emblyna callida Jackman 1997: 163; Platnick 1993: 556 [T]
Dictyna callida Gertsch & Ivie, 1936; Bonnet 1956: 1432; Chamberlin and Gertsch 1958: 110, mf, desc. (pl. 31, figs 10–13); Gertsch and Ivie 1936: 4, m, desc. (figs 6–8); Gertsch and Mulaik 1940: 331; Roewer 1955: 1319; Vogel 1970b: 7

####### Distribution.

Cameron, Hidalgo, Travis

####### Time of activity.

Male (April – July, October); female (April, June)

####### Habitat.

(soil/woodland: *Juniperus ashei*, *Ulmus crassifolia*)

####### Method.

Beating [mf]; sweeping [m]

####### Type.

Texas (male, Hidalgo Co., Edinburg, October 22, 1934, S. Mulaik, holotype, AMNH)

####### Etymology.

Latin, cunning

####### Collection.

TAMU

###### Emblyna completa

Taxon classificationAnimaliaAraneaeDictynidae

(Chamberlin & Gertsch, 1929)

Emblyna completa [Chamberlin and Gertsch 1958: 138, mf, desc. (pl. 42, figs 9–11, pl. 43, figs 1–3)]

####### Distribution.

Hutchinson

####### Locality.

Johnson Ranch

####### Time of activity.

Female (July)

####### Habitat.

(soil/woodland: saltcedar)

####### Type.

Utah, Moab

####### Etymology.

Latin, encircle

####### Collection.

NMSU

###### Emblyna consulta

Taxon classificationAnimaliaAraneaeDictynidae

(Gertsch & Ivie, 1936)

Emblyna consulta Jackman 1997: 163; Platnick 1993: 557 [T]
Dictyna consulta Gertsch and Ivie, 1936; Agnew et al. 1985: 3; Breene et al. 1993c: 14, 47, 53, mf (figs 11A-B); Chamberlin and Gertsch 1958: 146 [S], mf, desc. (pl. 45, figs 9–11); Dean and Sterling 1987: 6; Gertsch and Ivie 1936: 6, m, desc. (figs 12–13); Gertsch and Mulaik 1940: 329; Roewer 1955: 1320; Vogel 1967: 51; Vogel 1970b: 7; Young and Edwards 1990: 16
Dictyna montgomeryi Gertsch and Mulaik, 1940; Gertsch and Mulaik 1940: 328, f, desc. (fig. 31)

####### Distribution.

Brewster, Comanche, Coryell, Erath, Floyd, Hale, Howard, Lubbock, Martin, Midland, Mitchell, Nolan, Parmer, Reeves, Terry, Tom Green

####### Time of activity.

Male (January, May – October); female (April, June – September)

####### Habitat.

(crops: corn, cotton, peanuts); (littoral: creek bank); (soil/woodland: post oak savanna with pasture)

####### Method.

D-Vac suction [mf]; pitfall trap [m]; suction trap [mf]; sweeping [m]

####### Type.

Minnesota, near Minneapolis, Lake Minnetonka

####### Etymology.

Latin, considered

####### Collection.

MSU, TAMU

###### Emblyna cruciata

Taxon classificationAnimaliaAraneaeDictynidae

(Emerton, 1888)

Emblyna cruciata Jackman 1997: 163; Platnick 1993: 557 [T]
Dictyna cruciata Emerton, 1888; Chamberlin and Gertsch 1958: 111, mf, desc. (pl. 33, figs 1–4); Gertsch and Mulaik 1940: 328
Dictyna crucita Emerton, 1888; Vogel 1970b: 7

####### Distribution.

San Augustine

####### Time of activity.

Female (June)

####### Type.

Connecticut, New Haven

####### Etymology.

Latin, torment

###### Emblyna evicta

Taxon classificationAnimaliaAraneaeDictynidae

(Gertsch & Mulaik, 1940)

Emblyna evicta Calixto et al. 2013: 181; Jackman 1997: 163; Platnick 1993: 557 [T]
Dictyna evicta Gertsch and Mulaik, 1940; Chamberlin and Gertsch 1958: 122, mf, desc. (pl. 36, figs 1–4); Gertsch and Mulaik 1940: 332, m, desc. (fig. 18); Roewer 1955: 1320; Vogel 1967: 48; Vogel 1970b: 7

####### Distribution.

East Texas; Hays, Robertson

####### Locality.

Holmes Pecan Orchard

####### Time of activity.

Male (March – April)

####### Habitat.

(orchard: pecan)

####### Method.

cardboard band [m]

####### Type.

Texas (male, Hays Co., April 15, 1939, D. and S. Mulaik, holotype, AMNH)

####### Etymology.

Latin, expel

####### Collection.

TAMU

###### Emblyna hentzi

Taxon classificationAnimaliaAraneaeDictynidae

(Kaston, 1945)

Emblyna hentzi Calixto et al. 2013: 181; Jackman 1997: 163; Platnick 1993: 557 [T]
Dictyna hentzi Kaston, 1945; Chamberlin and Gertsch 1958: 115, mf, desc. (pl. 34, figs 11–15); Kaston 1972: 81; Kaston 1978: 82; Vogel 1970b: 7
Dictyna muraria Emerton, 1888; Jones 1936: 69 [Texas record]

####### Distribution.

Dallas, Fort Bend, Nacogdoches, Robertson, Wichita

####### Locality.

Holmes Pecan Orchard

####### Time of activity.

Female (February, May – July)

####### Habitat.

(orchard: pecan)

####### Method.

pitfall trap [f]

####### Type.

Connecticut, Cheshire

####### Etymology.

Person (arachnologist)

####### Collection.

MSU, TAMU

###### Emblyna iviei

Taxon classificationAnimaliaAraneaeDictynidae

(Gertsch & Mulaik, 1936)

Emblyna iviei Jackman 1997: 163; Platnick 1993: 557 [T]
Dictyna iviei Gertsch and Mulaik, 1936; Agnew et al. 1985: 3; Bonnet 1956: 1440; Chamberlin and Gertsch 1958: 133 [S], mf, desc. (pl. 40, figs 9–12); Gertsch and Mulaik 1936a: 7, m, desc. (figs 6–7); Gertsch and Mulaik 1940: 327; Roewer 1955: 1322; Vogel 1967: 55; Vogel 1970b: 7; Young and Edwards 1990: 16
Dictyna texana Jones, 1948; Jones 1948: 42, mf, desc. (figs 7, 30–32)

####### Distribution.

South Texas; Erath, Hidalgo, Jim Wells, Starr, Travis

####### Locality.

Mount Barker

####### Time of activity.

Male (April – September); female (January, May, December)

####### Habitat.

(crops: peanuts); (soil/woodland: sandy area)

####### Method.

pitfall trap [m]

####### Type.

Texas (male, Hidalgo Co., Edinburg, May 2, 1935, S. Mulaik, holotype, AMNH)

####### Etymology.

Person (collector of other spiders, Wilton Ivie)

####### Collection.

TAMU

###### Emblyna littoricolens

Taxon classificationAnimaliaAraneaeDictynidae

(Chamberlin & Ivie, 1935)

Emblyna littoricolens Platnick 1993: 557 [T]
Dictyna littoricolens Chamberlin and Gertsch, 1958 [Chamberlin and Gertsch 1958: 147, mf, desc. (pl. 46, figs 1–6)]

####### Distribution.

Wichita

####### Type.

Utah, Utah Lake

####### Etymology.

Latin, seashore

####### Collection.

MSU

###### Emblyna melva

Taxon classificationAnimaliaAraneaeDictynidae

(Chamberlin & Gertsch, 1958)

Emblyna melva Brignoli 1983: 514 [T]
Dictyna melva Chamberlin and Gertsch, 1958; Agnew et al. 1985: 6; Jackman 1997: 163 [Chamberlin and Gertsch 1958: 108, mf, desc. (pl. 32, figs 6–9)]

####### Distribution.

Erath, Travis

####### Time of activity.

Male (March – June); female (May)

####### Habitat.

(soil/woodland: juniper, *Juniperus ashei*, *Quercus buckleyi*, *Ulmus crassifolia*)

####### Method.

Beating [m]; suction trap [m]; sweeping [m]

####### Type.

Arizona, Cienega

####### Etymology.

Latin, color rusty red-brown

####### Collection.

TAMU

###### Emblyna orbiculata

Taxon classificationAnimaliaAraneaeDictynidae

(Jones, 1947)

Emblyna orbiculata Jackman 1997: 163; Platnick 1993: 558 [T]
Dictyna orbiculata Jones, 1947; Chamberlin and Gertsch 1958: 131, m, desc. (pl. 39, figs 10–13); Jones 1947: 5, m, desc. (figs 10–13); Roewer 1955: 1323; Vogel 1967: 51; Vogel 1970b: 8

####### Distribution.

Dallas

####### Time of activity.

Male (January)

####### Habitat.

(soil/woodland: forest herbs)

####### Type.

Texas (male, Dallas Co., Elm Fork of Trinity River, January 20, 1940, S. Jones, holotype, MCZ)

[female unknown]

####### Etymology.

Latin, palp with orbiculate coil

###### Emblyna reticulata

Taxon classificationAnimaliaAraneaeDictynidae

(Gertsch & Ivie, 1936)

Emblyna reticulata Jackman 1997: 163; Knutson et al. 2010: 515; Platnick 1993: 558 [T]
Dictyna reticulata Gertsch and Ivie, 1936; Breene et al. 1993c: 14, 47, 54, mf (figs 13A-B); Chamberlin and Gertsch 1958: 148 [S], mf, desc. (pl. 46, fig. 12, pl. 47, figs 1–7); Dean and Sterling 1987: 6; Gertsch and Mulaik 1940: 329; Kaston 1972: 81, desc.; Kaston 1978: 82, desc.; Vogel 1970b: 8
Dictyna declarata Gertsch and Mulaik, 1936; Bonnet 1956: 1434; Gertsch and Mulaik 1936a: 9, f, desc. (fig. 11); Gertsch and Mulaik 1940: 331

####### Distribution.

West and south Texas; Cameron, Howard, Reeves, Travis, Zapata

####### Time of activity.

Male (“January-March”, April, June – September); female (“January-March”, April – August)

####### Habitat.

(crops: cotton); (soil/woodland: saltcedar, *Juniperus ashei*, *Quercus virginiana*)

####### Method.

Beating [mf]; D-Vac suction [mf]; sweeping [mf]

####### Type.

Utah, Richfield

####### Etymology.

Latin, dorsum of abdomen with fine dark reticulations

####### Collection.

NMSU, TAMU

###### Emblyna roscida

Taxon classificationAnimaliaAraneaeDictynidae

(Hentz, 1850)

Emblyna roscida Jackman 1997: 163; Platnick 1993: 558 [T]
Dictyna roscida (Hentz, 1850); Breene et al. 1993c: 15, 47, 54, mf (figs 14A-B); Chamberlin and Gertsch 1958: 100 [S], mf, desc. (pl. 29, figs 8–12); Vogel 1970b: 8
Dictyna rubra Emerton, 1888; Gertsch and Mulaik 1940: 328
Dictyna florens Ivie and Barrows, 1935; Gertsch and Mulaik 1940: 328 [Texas records – see Chamberlin and Gertsch 1958: 103]

####### Distribution.

Clay, Harris, Hunt, Scurry, Titus, Travis, Walker, Wichita

####### Locality.

Ellis Prison Unit, Lake Thomas, Zilker Park

####### Time of activity.

Male (June, October); female (May – June, August, October)

####### Habitat.

(crops: cotton); (plants: miscellaneous vegetation, *Baccharis*)

####### Method.

sweeping [f]

####### Type.

Alabama

####### Etymology.

Latin, rose colored

####### Collection.

MSU, TAMU

###### Emblyna stulta

Taxon classificationAnimaliaAraneaeDictynidae

(Gertsch & Mulaik, 1936)

Emblyna stulta Jackman 1997: 163; Platnick 1993: 558 [T]
Dictyna stulta (Gertsch and Mulaik, 1936); Bonnet 1956: 1450; Chamberlin and Gertsch 1958: 106, mf, desc. (pl. 31, figs 1–5); Gertsch and Mulaik 1936a: 7, m, desc. (fig. 9); Gertsch and Mulaik 1940: 328; Roewer 1955: 1325; Vogel 1970b: 8

####### Distribution.

Jeff Davis

####### Time of activity.

Male (July)

####### Type.

Texas (male, Jeff Davis Co., Fort Davis, July 1934, S. Mulaik, holotype, AMNH)

####### Etymology.

Latin, foolish

###### Emblyna sublata

Taxon classificationAnimaliaAraneaeDictynidae

(Hentz, 1850)

Emblyna sublata Bradley 2013: 119; Jackman 1997: 98, desc., 163; Platnick 1993: 558 [T]
Dictyna sublata (Hentz, 1850); Agnew et al. 1985: 3; Bonnet 1956: 1450; Chamberlin and Gertsch 1958: 127 [S], mf, desc. (pl. 38, figs 1–8, pl. 39, figs 1–5); Gertsch and Mulaik 1940: 328; Kaston 1972: 80, desc. (fig. 183); Kaston 1978: 81, desc. (fig. 201); Vogel 1970b: 8; Young and Edwards 1990: 17
Dictyna volupis Keyserling, 1881; Jones 1936: 69

####### Distribution.

North-central, central and east Texas; Brazos, Clay, Comanche, Dallas, Denton, Erath, Fort Bend, Hunt, Jasper, Kerr, Montgomery, Robertson, Sutton, Tarrant, Wichita

####### Locality.

Jones State Forest, Lake Tawakoni State Park, Lick Creek Park, Riley Estate

####### Time of activity.

Male (March – June, August, October); female (March – August)

####### Habitat.

(crops: peanuts); (grass: grass); (plants: miscellaneous vegetation, vegetation); (soil/woodland: bottomland forest)

####### Method.

Flight intercept trap [m]; pitfall trap [mf]; sweeping [mf]

####### Type.

Alabama

####### Etymology.

Latin, raised aloft

####### Collection.

MSU, TAMU

##### Genus *Lathys* Simon, 1884

###### Lathys delicatula

Taxon classificationAnimaliaAraneaeDictynidae

(Gertsch & Mulaik, 1936)

Lathys delicatula Chamberlin and Gertsch 1958: 31 [T], mf, desc. (pl. 7, figs 5–9); Gertsch 1946a: 3, f (fig. 14); Jackman 1997: 163; Roewer 1955: 1329; Vogel 1970b: 8
Scotolathys delicatulus Gertsch and Mulaik, 1936; Gertsch and Mulaik 1936a: 4, f, desc. (fig. 4) [see note below]; Gertsch and Mulaik 1940: 326
Scotolathys delicatula Gertsch and Mulaik, 1936; Bonnet 1958: 3963

####### Distribution.

Widespread; Brazos, Brewster, Burleson, Cameron, Collin, Coryell, Gonzalez, Hidalgo, Houston, Hunt, Jeff Davis, Sabine, San Augustine, Smith, Tyler, Wichita, Zapata

####### Locality.

Big Bend National Park, Big Slough Wild Area, Big Thicket National Preserve, Chisos Basin, Chisos Mountains, Laguna Madre, Texas A&M University Rangeland Area, Tyler State Park

####### Time of activity.

Male (January – March, May, August – October, October 30-November 6, December); female (January – December)

####### Habitat.

(grass: grass); (nest/prey: nest of *Neotoma micropus* [mf]); (soil/woodland: beech-magnolia litter, leaf litter, mixed hardwood litter, post oak savanna with pasture)

####### Method.

Berlese funnel [mf]; pitfall trap [mf]

####### Type.

Texas (female, Cameron Co., 15 miles SW Harlingen, November 18, 1934, S. Mulaik, holotype, AMNH)

####### Etymology.

Latin, dainty

####### Collection.

DMNS, TAMU

####### Note.

32 miles E Laredo should be 32 miles SE Laredo in Zapata Co. based on other records from this date.

###### Lathys maculina

Taxon classificationAnimaliaAraneaeDictynidae

Gertsch, 1946

Lathys maculina Bradley 2013: 119; Chamberlin and Gertsch 1958: 32 [T], mf, desc. (pl. 6, figs 9–12); Gertsch 1946a: 4, f (fig. 15); Jackman 1997: 163; Kaston 1972: 78 (fig. 177); Kaston 1978: 79 (fig. 195); Vogel 1970b: 8
Dictyolathys maculata Banks, 1900; Bryant 1943: 85, f, desc. (figs A, B)
Scotolathys maculatus Banks, 1900; Gertsch and Mulaik 1940: 326 [T]

####### Distribution.

Harris

####### Time of activity.

Female (June)

####### Type.

Alabama, Mobile

####### Etymology.

Latin, derivation of *Dictyolathys maculata* Banks, 1900, preoccupied

##### Genus *Mallos* O. P.-Cambridge, 1902

###### Mallos blandus

Taxon classificationAnimaliaAraneaeDictynidae

Chamberlin & Gertsch, 1958

Mallos blandus Bond and Opell 1997: 421, mf, desc. (figs 58–63)

####### Distribution.

Culberson

####### Locality.

Guadalupe Mountains National Park

####### Time of activity.

Female (August)

####### Type.

New Mexico, Whites City

####### Etymology.

Latin, friendly, mild

###### Mallos niveus

Taxon classificationAnimaliaAraneaeDictynidae

O. P.-Cambridge, 1902

Mallos niveus [Bond and Opell 1997: 428, mf, desc. (figs 9, 15, 75–80)]

####### Distribution.

Wichita

####### Type.

Mexico, Morelos, Cuernavaca

####### Etymology.

Latin, snowy

####### Collection.

MSU

###### Mallos pallidus

Taxon classificationAnimaliaAraneaeDictynidae

(Banks, 1904)

Mallos pallidus Chamberlin and Gertsch 1958: 42, mf, desc. (pl. 9, figs 3–8); Jackman 1997: 163; Kaston 1978: 83; Vogel 1970b: 8

####### Distribution.

Presidio, Travis

####### Locality.

Big Bend Ranch State Park, Mount Barker

####### Time of activity.

Male (October); female (March)

####### Habitat.

(plants: grape vine)

####### Method.

Beating [f]

####### Type.

California, Mount Shasta

####### Etymology.

Latin, pale (pallid)

####### Collection.

NMSU

##### Genus *Mexitlia* Lehtinen, 1967

###### Mexitlia trivittata

Taxon classificationAnimaliaAraneaeDictynidae

(Banks, 1901)

Mexitlia trivittata [Bond and Opell 1997: 439, mf, desc. (figs 3, 11, 13, 100–103)]

####### Distribution.

Brewster, Wichita

####### Type.

New Mexico, Albuquerque

####### Etymology.

Latin, three bands

####### Collection.

MSU

##### Genus *Phantyna* Chamberlin, 1948

###### Phantyna bicornis

Taxon classificationAnimaliaAraneaeDictynidae

(Emerton, 1915)

Phantyna bicornis Calixto et al. 2013: 182; Jackman 1997: 163; Lehtinen 1967: 257 [T]
Dictyna bicornis Emerton, 1915; Agnew et al. 1985: 3; Chamberlin and Gertsch 1958: 59 [S], mf, desc. (pl. 15, figs 1–7); Vogel 1967: 45; Vogel 1970b: 7; Young and Edwards 1990: 16
Dictyna annamae Gertsch and Mulaik, 1940; Gertsch and Mulaik 1940: 330, m, desc. (figs 24–25)

####### Distribution.

Comanche, Dallam, Erath, Kendall, Randall, Walker, Wilbarger, Zavala

####### Locality.

Bill Haney Pecan Orchard, Ellis Prison Unit, Palo Duro Canyon, Proctor Lake

####### Time of activity.

Male (May – September); female (January, April, June – September)

####### Habitat.

(crops: peanuts); (orchard: pecan); (soil/woodland: sandy area)

####### Method.

Fogging [f]; irrigation tubing [f]; pitfall trap [mf]; suction trap [mf]

####### Type.

Massachusetts, Ipswich

####### Etymology.

Latin, male chelicera has basal horns distinctly developed

####### Collection.

MSU, TAMU

###### Phantyna mulegensis

Taxon classificationAnimaliaAraneaeDictynidae

(Chamberlin, 1924)

Phantyna mulegensis Jackman 1997: 163; Lehtinen 1967: 257 [T]
Dictyna mulegensis Chamberlin, 1924; Breene et al. 1993c: 14, 47, 53, mf (figs 12A-B); Chamberlin and Gertsch 1958: 56, mf, desc. (pl. 14, figs 9–13, pl. 15, figs 8–9); Dean and Sterling 1987: 6; Gertsch and Mulaik 1940: 327; Roewer 1955: 1317; Vogel 1970b: 7

####### Distribution.

West and south Texas; Hidalgo, Kleberg, Nueces, Starr, Val Verde

####### Time of activity.

Male (February, May – June, October – November); female (January – August, October – November)

####### Habitat.

(crops: cotton)

####### Method.

D-Vac suction [f]

####### Type.

Mexico, Baja California, Mulegé

####### Etymology.

locality (town)

####### Collection.

MCZ, TAMU

###### Phantyna provida

Taxon classificationAnimaliaAraneaeDictynidae

(Gertsch & Mulaik, 1936)

Phantyna provida Jackman 1997: 163; Lehtinen 1967: 257 [T]
Dictyna provida (Gertsch and Mulaik, 1936); Bonnet 1956: 1447; Chamberlin and Gertsch 1958: 55 [S], mf, desc. (pl. 13, figs 2–5); Gertsch and Mulaik 1936a: 8, f, desc. (fig. 10); Gertsch and Mulaik 1940: 327; Roewer 1955: 1324; Vogel 1967: 49; Vogel 1970b: 8
Dictyna ingenuata Gertsch and Mulaik, 1940; Gertsch and Mulaik 1940: 332, m, desc. (fig. 19); Roewer 1955: 1321

####### Distribution.

Hidalgo, Llano

####### Time of activity.

Male (May); female (May, September)

####### Type.

Texas (female, Hidalgo Co., Edinburg, May 2, 1935, S. Mulaik, holotype, AMNH)

####### Etymology.

Latin, provided with distinct features

###### Phantyna segregata

Taxon classificationAnimaliaAraneaeDictynidae

(Gertsch & Mulaik, 1936)

Phantyna segregata Breene et al. 1994: 8; Calixto et al. 2013: 180, 182, 185, 187; Irungu 2007: 30; Jackman 1997: 98, desc., 163; Lehtinen 1967: 257 [T]; Pfannenstiel 2008a: 204
Dictyna segregata Gertsch and Mulaik, 1936; Agnew et al. 1985: 3; Bonnet 1956: 1450; Breene 1988: 34, 36; Breene et al. 1989: 162; Breene et al. 1993c: 15, 47, 54, mf (figs 15A-C); Chamberlin and Gertsch 1958: 57 [S], mf, desc. (pl. 14, figs 1–5); Dean and Eger 1986: 141; Dean and Sterling 1987: 6; Dean and Sterling 1990: 402, 405; Dean and Sterling 1992: 3–4; Dean et al. 1982: 254; Dean et al. 1988: 286; Gertsch and Mulaik 1936a: 4, m, desc. (figs 13–14); Gertsch and Mulaik 1940: 327; Kagan 1942: 12; Kagan 1943: 258; Nyffeler and Sterling 1994: 1295, 1298; Nyffeler et al. 1988a: 55; Nyffeler et al. 1988b: 215; Nyffeler et al. 1992a: 1181; Nyffeler et al. 1992c: 2; Pamanes-Guerrero 1975: 41, 59, 63, 78, 81; Roewer 1955: 1317; Vogel 1967: 52; Vogel 1970b: 8; Young and Edwards 1990: 17
Dictyna patellaris Jones, 1947; Jones 1947: 1, m, desc. (figs 1–4)

####### Distribution.

Eastern ½ Texas; Archer, Bexar, Brazos, Burleson, Cameron, Comanche, Coryell, Dallas, Denton, Erath, Harris, Hidalgo, Houston, Hunt, Kaufman, Kerr, La Salle, McLennan, Nueces, Robertson, San Patricio, Travis, Victoria, Walker, Washington, Wichita, Williamson

####### Locality.

5-Eagle Ranch, Bill Haney Pecan Orchard, Ellis Prison Unit, Frontera Audubon, Holmes Pecan Orchard, Lake Tawakoni State Park, NK Ranch, Stiles Farm Foundation

####### Time of activity.

Male (February – December); female (February – December)

####### Habitat.

(crops: cabbage, cotton, peanuts, soybean, watermelon); (grass: grass, pasture); (littoral: edge of pond, near pond); (orchard: grapefruit, Mexican lime, orange, pecan, sour orange, tangerine); (plants: bluebonnets, herbs, *Hibiscus* sp., *Monarda citriodora*); (soil/woodland: post oak savanna with pasture)

####### Method.

cardboard band [mf]; D-Vac suction [m]; fogging [mf]; pitfall trap [mf] (edge of pond [f], near pond [f]); ramp trap [mf]; suction trap [mf]; sweeping [mf]; tile trap [mf]

####### Eggs/spiderlings.

Hidalgo [17 eggs in eggsac] [TAMU]

####### Type.

Texas (male, Hidalgo Co., northwest of Edinburg, June 15, 1935, S. Mulaik, holotype, AMNH)

####### Etymology.

Latin, separated

####### Collection.

DMNS, MSU, TAMU

##### Genus *Thallumetus* Simon, 1893

###### Thallumetus pineus

Taxon classificationAnimaliaAraneaeDictynidae

(Chamberlin & Ivie, 1944)

Thallumetus pineus Bennett 2005a: 100; Chamberlin and Gertsch 1958: 36, mf, desc. (pl. 7, figs 11–14); Jackman 1997: 163; Roth 1982: 15–2; Roth 1985: B-11–2; Roth 1994: 90; Vogel 1970b: 8

####### Distribution.

Panola

####### Time of activity.

Male (May)

####### Habitat.

(plants: roadside plants)

####### Method.

sweeping [m]

####### Type.

Georgia, 3 miles SE Savannah

####### Etymology.

Latin, habitat (tree)

##### Genus *Tivyna* Chamberlin, 1948

###### Tivyna petrunkevitchi

Taxon classificationAnimaliaAraneaeDictynidae

(Gertsch & Mulaik, 1940)

Tivyna petrunkevitchi Jackman 1997: 163; Lehtinen 1967: 271 [T]
Dictyna petrunkevitchi Gertsch and Mulaik, 1940; Chamberlin and Gertsch 1958: 52, f, desc. (pl. 11, figs 7–8) [see note below]; Gertsch and Mulaik 1940: 330, f, desc. (figs 22–23) [see note below]; Roewer 1955: 1323; Vogel 1967: 52; Vogel 1970b: 8

####### Distribution.

Zapata

####### Time of activity.

Female (November)

####### Type.

Texas (female, Zapata Co., 32 miles E Laredo, November 11, 1934, S. Mulaik, holotype, AMNH)

[male unknown]

####### Etymology.

Person (arachnologist, Alexander Petrunkevitch)

####### Note.

32 miles E Laredo should be 32 miles SE Laredo in Zapata Co. based on other records from this date.

##### Genus *Tricholathys* Chamberlin & Ivie, 1935

###### Tricholathys knulli

Taxon classificationAnimaliaAraneaeDictynidae

Gertsch & Mulaik, 1936

Tricholathys knulli Bonnet 1959: 4686; Chamberlin and Gertsch 1958: 20, f, desc. (pl. 3, fig. 6); Gertsch and Mulaik 1936a: 1, f, desc. (fig. 1); Gertsch and Mulaik 1940: 326; Jackman 1997: 163; Roewer 1955: 1335; Vogel 1970b: 8

####### Distribution.

Cameron

####### Time of activity.

Female (June)

####### Type.

Texas (female, Cameron Co., Brownsville, June 1, 1934, J. N. Knull, holotype, AMNH)

[male unknown]

####### Etymology.

Person (collector)

#### Family Diguetidae F. O. P.-Cambridge, 1899

##### Genus *Diguetia* Simon, 1895

###### Diguetia albolineata

Taxon classificationAnimaliaAraneaeDiguetidae

(O. P.-Cambridge, 1895)

Diguetia albolineata Gertsch 1958a: 16, mf, desc. (figs 6, 14–15); Jackman 1997: 40, 163; Kaston 1978: 90, desc.; Vogel 1970b: 8
Diguetia caudata Gertsch, 1935; Gertsch and Mulaik 1940: 317 [Texas record]; Vogel 1970b: 21

####### Distribution.

Brewster, Val Verde

####### Locality.

Big Bend National Park, Chisos Basin, Chisos Mountains, Seminole Canyon State Park

####### Time of activity.

Male (July, September); female (July, September)

####### Type.

Mexico, Guerrero, Vente de Zopilote

####### Etymology.

Latin, white lines

####### Collection.

TAMU

###### Diguetia canities

Taxon classificationAnimaliaAraneaeDiguetidae

(McCook, 1889)

Diguetia canities Agnew et al. 1985: 6; Chamberlin 1924b: 591; Gertsch 1958a: 6, mf, desc. (figs 16–19); Gertsch and Mulaik 1940: 317 [part, see note below]; Jackman 1997: 40, desc., 163; Kaston 1953: 41, desc. (fig. 83); Kaston 1972: 89, desc. (fig. 201); Kaston 1978: 90, desc. (fig. 219); Milstead 1958: 445; Petrunkevitch 1911: 117; Roewer 1942: 323; Vogel 1970b: 9, 20

####### Distribution.

Brewster, El Paso, Erath, Presidio, Randall, Terrell

####### Locality.

Big Bend National Park, Big Bend Ranch State Park, Black Gap Wildlife Management Area, Blackstone Ranch, Chisos Basin, Palo Duro Canyon

####### Time of activity.

Male (August, October); female (March – April, August – October)

####### Habitat.

(landscape features: under rock); (nest/prey: stomach of *Cnemidophorus perplexus*, stomach of *Cnemidophorus sacki*); (web: web in cactus)

####### Type.

California, near San Bernardino

####### Etymology.

Latin, grayish hairs

####### Collection.

NMSU, TAMU

####### Note.

SE Laredo is 32 miles SE of Laredo in Zapata Co. based on collecting records from this date.

###### Diguetia canities mulaiki

Taxon classificationAnimaliaAraneaeDiguetidae

Gertsch, 1958

Diguetia canities mulaiki Banks 1898b: 209; Gertsch 1935a: 6; Gertsch 1958a: 12, mf, desc. (figs 1–4, 11–13); Jackman 1997: 40, 163; Vogel 1967: 61; Vogel 1970b: 9
Diguetia canities McCook 1889; Gertsch and Mulaik 1940: 317 [part]

####### Distribution.

Cameron, Dimmitt, Hidalgo, Hudspeth, Jim Wells, Zapata

####### Locality.

Laguna Madre

####### Time of activity.

Female (May, August – September, November – December)

####### Habitat.

(nest/prey: mud dauber nest [mf], nest of *Neotoma* sp.)

####### Type.

Texas (male, Hidalgo Co., Edinburg, 1933, S. Mulaik, holotype, AMNH)

####### Etymology.

Person (collector)

####### Collection.

MCZ

###### Diguetia imperiosa

Taxon classificationAnimaliaAraneaeDiguetidae

Gertsch & Mulaik, 1940

Diguetia imperiosa Gertsch 1958a: 18, mf, desc. (figs 7–10); Gertsch and Mulaik 1940: 317, mf, desc.; Jackman 1997: 163; Vogel 1967: 62; Vogel 1970b: 9, 21
Diguetia canities McCook, 1889; Gertsch and Mulaik 1940: 317 [part]

####### Distribution.

Brewster, Hidalgo, Presidio, Terrell, Val Verde

####### Locality.

Big Bend National Park, Big Bend Ranch State Park

####### Time of activity.

Male (August); female (May – December)

####### Habitat.

(web: in web)

####### Type.

Texas (male, Val Verde Co., Langtry, Santa Elena Canyon, August 18, 1935, S. Mulaik, holotype, AMNH)

####### Etymology.

Latin, authority

####### Collection.

TAMU

#### Family Dysderidae C. L. Koch, 1837

##### Genus *Dysdera* Latreille, 1804

###### Dysdera crocata

Taxon classificationAnimaliaAraneaeDysderidae

C. L. Koch, 1838

Dysdera crocata Gertsch and Mulaik 1940: 323; Jones 1936: 70; Vogel 1970b: 9 [Kaston 1948: 62, m, desc. (figs 7–10); Roberts 1985: 60, mf (figs 19b, d, f, h)]
Dysdera crocota C. L. Koch, 1838; Jackman 1997: 42, desc., 163

####### Distribution.

Dallas, El Paso, Tarrant

####### Time of activity.

Male (August, November); female (June, August)

####### Habitat.

(structures: outside house)

####### Type.

Greece

####### Etymology.

Latin, saffron-yellow

####### Collection.

NMSU, TAMU

#### Family Eutichuridae Lehtinen, 1967

**Note.** raised to family (Ramírez 2014: 340)

##### Genus *Cheiracanthium* C. L. Koch, 1839

**Note.** transferred from Clubionidae to Miturgidae: Eutichurinae (Ramirez et al. 1997: 44) and here Ramírez 2014: 341. Spelling of genus changed from *Chiracanthium* (Platnick 1989: 438).

###### Cheiracanthium inclusum

Taxon classificationAnimaliaAraneaeMiturgidae

(Hentz, 1847)

Cheiracanthium inclusum Armstrong and Richman 2007: 395; Breene 1988: 23–26, 35, 41; Breene et al. 1988: 180–181; Breene et al. 1989: 162; Breene et al. 1993a: 169; Breene et al. 1993b: 647; Breene et al. 1993c: 13, 47, 85, mf (figs 98A-C); Calixto et al. 2013: 183; Dean and Sterling 1990: 403, 405; Dean and Sterling 1992: 3–4; Dean et al. 1988: 287; Jackman 1997: 109, desc., 161 (photo 33a); Knutson et al. 2010: 515; Pfannenstiel 2008a: 204; Pfannenstiel 2008b: 419; Platnick 1989: 438 [spelling]; Taylor and Pfannenstiel 2008: 997; Taylor and Pfannenstiel 2009: 1380; Trevino 2014: 13
Chiracanthium inclusum (Hentz, 1847); Agnew et al. 1982: 631; Agnew et al. 1985: 4, 10; Bonnet 1956: 1057; Brown 1974: 233; Dean and Eger 1986: 142; Dean and Sterling 1985: 119; Dean and Sterling 1987: 6; Dean et al. 1982: 255; Dean et al. 1987: 268; Edwards 1958: 368, mf, desc. (figs 10–13, 17, 202) [see note below]; Jones 1936: 69; Kagan 1942: 59; Kagan 1943: 258; Liao et al. 1984: 410; McDaniel et al. 1981: 104; Nuessly and Sterling 1984: 97; Nyffeler et al. 1987a: 356; Pamanes-Guerrero 1975: 16, 34, 37, 41, 59, 63, 78, 81; Rogers and Horner 1977: 523; Sterling et al. 1979: 979; Vogel 1970b: 5; Young and Edwards 1990: 16

####### Distribution.

Atascosa, Bastrop, Baylor, Bee, Bexar, Brazoria, Brazos, Burleson, Cameron, Cherokee, Clay, Colorado, Comanche, Coryell, Dallas, Delta, Erath, Fayette, Galveston, Gillespie, Gonzalez, Harris, Henderson, Hidalgo, Houston, Howard, Hunt, Kaufman, Kent, Kerr, Kinney, Knox, Llano, Martin, McLennan, Menard, Midland, Nacogdoches, Newton, Nolan, Polk, Presidio, Robertson, Scurry, Starr, Sutton, Travis, Val Verde, Victoria, Walker, Webb, Wharton, Wichita, Willacy, Young, Zapata

####### Locality.

Adriance Pecan Orchard, Attwater Prairie Chicken National Wildlife Refuge, Bill Haney Pecan Orchard, Buescher State Park, Ellis Prison Unit, Holmes Pecan Orchard, Lake Thomas, Lick Creek Park, Lower Rio Grande Valley National Wildlife Refuge, Palmetto State Park, Ramsey Prison Farm, Russell Farm, Zilker Park

####### Time of activity.

Male (January – October, December); female (January – December)

####### Habitat.

(crops: cotton, guar, peanuts, sugarcane); (grass: grass, grassland, pasture); (nest/prey: mud dauber nest [f]); (orchard: citrus, pecan); (plants: bluebonnets, croton, Indian paintbrush, roadside vegetation, vegetation, yucca, *Baccharis*); (soil/woodland: juniper, post oak savanna with pasture, riparian mesquite forest, saltcedar, trees, trees/shrubs, *Juniperus ashei*, *Quercus virginiana*); (structures: around house)

####### Method.

Beating [mf]; boll weevil pheromone trap [mf]; cardboard band [mf]; D-Vac suction [mf]; fogging [mf]; malaise trap [m]; pitfall trap [m]; suction trap [mf]; sweeping [mf]; uv light [m]

####### Eggs/spiderlings.

Walker [eggsac laid in lab April 18, 1978, hatched May 2, 32 spiderlings] [TAMU]

####### Type.

Carolina (of 1847)

####### Etymology.

Latin, to include

####### Collection.

DMNS, MSU, NMSU, TAMU

####### Note.

32 miles SW Laredo should be 32 miles SE Laredo in Zapata Co. based on other records from this date.

##### Genus *Strotarchus* Simon, 1888

**Note.** transferred from Clubionidae to Miturgidae (Lehtinen 1967: 321) and here Ramírez (2014: 341). Bonaldo et al. (2012) revised *Strotarchus* but did not study any specimens of *piscatorius* from Texas and only the type of *planeticus* where the male remains unknown.

###### Strotarchus piscatorius

Taxon classificationAnimaliaAraneaeMiturgidae

(Hentz, 1847)

Strotarchus piscatorius Brown 1974: 233; Jackman 1997: 166; Yantis 2005: 66, 198, 202 [Edwards 1958: 373, mf, desc. (figs 1–3, 15, 205)]

####### Distribution.

Brazos, Brewster, Houston, Leon, Nacogdoches, Sabine, San Patricio, Trinity, Walker

####### Locality.

Welder Wildlife Refuge

####### Time of activity.

Male (April – May, July); female (May – June, August)

####### Habitat.

(soil/woodland: beech-magnolia forest, hackberry woodland, mix-pine forest, on ground, pine woods [%: 66, 82, 84, 86, 95], post oak woods [%: 44, 56, 71, 77, 91, 94])

####### Method.

5 gallon bucket trap [mf]; flight intercept trap [m]; malaise trap [mf]; pitfall trap [f]

####### Type.

Alabama

####### Etymology.

Latin, fisherman

####### Collection.

MSU, TAMU

###### Strotarchus planeticus

Taxon classificationAnimaliaAraneaeMiturgidae

Edwards, 1958

Strotarchus planeticus Edwards 1958: 374, f, desc. (figs 159, 179, 206); Jackman 1997: 166; Ubick and Richman 2005c: 174; Vogel 1967: 43; Vogel 1970b: 6

####### Distribution.

Cameron, San Patricio

####### Locality.

Laguna Madre, Welder Wildlife Refuge

####### Time of activity.

Female (June)

####### Habitat.

(nest/prey: nest of *Neotoma micropus* [f]); (soil/woodland: hackberry woodland)

####### Method.

pitfall trap [f]

####### Type.

Texas (female, Cameron Co., Laguna Madre, 25 miles SE Harlingen, June 13, 1945, D. E. Hardy, V. L. Wooley, holotype, AMNH)

[male unknown]

####### Etymology.

Greek, wanderer

####### Collection.

TAMU

#### Family Filistatidae Ausserer, 1867

##### Genus *Filistatinella* Gertsch & Ivie, 1936

###### Filistatinella crassipalpis

Taxon classificationAnimaliaAraneaeFilistatidae

(Gertsch, 1935)

Filistatinella crassipalpis Bonnet 1957: 1908; Platnick 2000 [spelling]; Reddell 1963: 35; Reddell 1965: 171; Ubick 2005b: 105; Vogel 1970b: 9
Filistata crassipalpus Gertsch, 1935; Gertsch 1935a: 5, mf, desc. (figs 4–6) [see note below]
Filistatinella crassipalpus (Gertsch, 1935); Brown 1974: 233; Comstock 1940: 301, desc.; Gertsch 1979: 132; Gertsch and Ivie 1936: 1 [T]; Gertsch and Mulaik 1940: 315; Jackman 1997: 163; Lehtinen 1967: 235; Roewer 1955: 1282

####### Distribution.

Archer, Baylor, Caldwell, Fannin, Grayson, Kerr, Nacogdoches, Sutton, Val Verde, Wichita, Zapata

####### Caves.

**Val Verde** (Marshall Bat Cave)

####### Time of activity.

Male (November); female (May, July, November)

####### Habitat.

(landscape features: cave); (structures: in barn)

####### Type.

Texas (male, Webb Co., 32 miles E Laredo, November 11, 1934, S. Mulaik, holotype, AMNH)

####### Etymology.

Latin, palps

####### Collection.

MSU, TMM

####### Note.

32 miles E Laredo should be 32 miles SE Laredo in Zapata Co. based on other records from this date.

##### Genus *Filistatoides* F. O. P.-Cambridge, 1899

###### Filistatoides insignis

Taxon classificationAnimaliaAraneaeFilistatidae

(O. P.-Cambridge, 1896)

Filistatoides insignis Comstock 1940: 301, desc.; Gertsch and Mulaik 1940: 316 [see note below]; Jackman 1997: 163; Ubick 2005b: 105; Vogel 1970b: 9 [Ramírez and Grismado, 1997: 346, m (figs 104–106)]

####### Distribution.

Brewster, Hidalgo, Starr, Zapata

####### Time of activity.

Female (February, June, November)

####### Type.

Guatemala

####### Etymology.

Latin, remarkable

####### Note.

32 miles E Laredo should be 32 miles SE Laredo in Zapata Co. based on other records from this date.

##### Genus *Kukulcania* Lehtinen, 1967

###### Kukulcania arizonica

Taxon classificationAnimaliaAraneaeFilistatidae

(Chamberlin & Ivie, 1935)

Kukulcania arizonica Jackman 1997: 163; Lehtinen 1967: 242 [T]
Filistata arizonica Chamberlin and Ivie, 1935; Gertsch 1939b: 23; Gertsch and Mulaik 1940: 315; Milstead 1958: 445; Reddell 1965: 171; Vogel 1970b: 9 [Chamberlin and Ivie 1935b: 4, mf, desc. of m (pl. 4, figs 24–25)]

####### Distribution.

Brewster, Cameron, Hidalgo, Presidio, Real, Terrell

####### Locality.

Chisos Basin, Chisos Mountains, La Mota Mountains, Laguna Atascosa National Wildlife Refuge, Santa Ana National Wildlife Refuge

####### Caves.

**Real** (Orell Crevice Cave)

####### Time of activity.

Male (April 26-May 14, July, October – November, November 14-February 8); female (July – August, December)

####### Habitat.

(landscape features: cave, under rock); (littoral: dense coastal brush); (nest/prey: stomach of *Cnemidophorus perplexus*, stomach of *Cnemidophorus tessellatus*)

####### Method.

Flight intercept trap on ground [m]; pitfall trap [m]

####### Type.

Arizona, near Roosevelt Dam

####### Etymology.

locality (state)

####### Collection.

TAMU, TMM

###### Kukulcania hibernalis

Taxon classificationAnimaliaAraneaeFilistatidae

(Hentz, 1842)

Kukulcania hibernalis Bradley 2013: 124; Breene et al. 1993c: 15, 47, 52, m (figs 7A-B); Broussard and Horner 2006: 253; Jackman 1997: 31, desc., 163 (photo 6a); Lehtinen 1967: 242 [T]; Rice 1986: 124; Richman et al. 2011a: 46; Taber and Fleenor 2005: 278 (figs 12–6, 12–7); Vetter 2015: 27; Young and Edwards 1990: 17 [Ramirez and Grismado 1997: 348, mf (figs 107–111)]
Filistata hibernalis Hentz, 1842; Agnew et al. 1985: 6; Brown 1974: 233; Comstock 1912: 291, desc.; Comstock 1940: 294, desc.; Dean et al. 1982: 254; Gertsch and Mulaik 1940: 315; Kaston 1972: 73 (fig. 166); Kaston 1978: 74 (fig. 183); Vogel 1970b: 9

####### Distribution.

Archer, Atascosa, Bastrop, Bexar, Brazos, Brown, Calhoun, Cameron, Clay, Culberson, Edwards, Erath, Gonzales, Harris, Henderson, Hidalgo, Jack, Jeff Davis, Llano, Midland, Montgomery, Morris, Nacogdoches, Nueces, Presidio, Robertson, San Patricio, Shelby, Starr, Titus, Tom Green, Travis, Walker, Wichita, Williamson, Zapata

####### Locality.

Bentsen-Rio Grande Valley State Park, Chihuahuan desert, Dalquest Research Site, Ellis Prison Unit, Lake Corpus Christi State Park, Palmetto State Park, Sabal Palm Audubon Sanctuary, Santa Ana National Wildlife Refuge, Welder Wildlife Refuge

####### Time of activity.

Male (January – July, September – December); female (January – December)

####### Habitat.

(crops: cotton); (landscape features: overpass, under [bridge, rock]); (littoral: wetlands); (nest/prey: pack rat nest); (objects: under rafter); (soil/woodland: anacua groves, palm forest); (structures: back of TV, bit person in bed, cellar, elementary school, garage, house, in [barn, bedroom, building, house, lab, lab on ceiling, lakehouse], indoors, store room, under house, warehouse)

####### Method.

Boll weevil pheromone trap [imm.]; flight intercept trap on ground [m]; pitfall trap [mf]

####### Eggs/spiderlings.

Titus [146 spiderlings] [TAMU]

####### Type.

South Carolina and Alabama

####### Etymology.

Latin, winter

####### Collection.

DMNS, MSU, TAMU

#### Family Gnaphosidae Pocock, 1898

**nomen dubium**

*Micaria aurata* (Hentz, 1847); Kaston 1953: 81; Kaston 1972: 218; Kaston 1978: 211; Marx 1890: 506; Petrunkevitch 1911: 493

##### Genus *Callilepis* Westring, 1874

###### Callilepis chisos

Taxon classificationAnimaliaAraneaeGnaphosidae

Platnick, 1975

Callilepis chisos Agnew et al. 1985: 7; Bowen et al. 2004: 187; Broussard and Horner 2006: 253; Jackman 1997: 163; Platnick 1975a: 15, mf, desc. (figs 29–35); Richman et al. 2011a: 48; Zolnerowich and Horner 1985: 80

####### Distribution.

Brewster, Comanche, Erath, Hidalgo, Presidio, Smith, Wichita

####### Locality.

Bentsen-Rio Grande Valley State Park, Big Bend National Park, Chihuahuan desert, Chisos Basin, Chisos Mountains, Dalquest Research Site, Nabor’s Lake, Tyler State Park

####### Time of activity.

Male (May); female (April – June, August – September)

####### Habitat.

(littoral: bark debris along small lake); (soil/woodland: sandy area, under bark, woods); (structures: indoors)

####### Method.

pitfall trap [mf]

####### Type.

Texas (male, Brewster Co., Big Bend National Park, Chisos Mountains, the Basin, May 28, 1952, M. Cazier, W. Gertsch, R. Schrammel, holotype, AMNH)

####### Etymology.

locality (The specific name is a noun in apposition taken from the type locality, Platnick 1975a).

####### Collection.

MSU, TAMU

###### Callilepis gertschi

Taxon classificationAnimaliaAraneaeGnaphosidae

Platnick, 1975

Callilepis gertschi Broussard and Horner 2006: 253; Jackman 1997: 163; Platnick 1975a: 27, mf, desc. (figs 60–66); Richman et al. 2011a: 48

####### Distribution.

Brewster, Cameron, Comal, Coryell, Hidalgo, Presidio, Starr, Terrell, Travis, Val Verde, Zapata

####### Locality.

Bentsen-Rio Grande Valley State Park, Chihuahuan desert, Chisos Mountains, Dalquest Research Site, Falcon State Park, Green Island Bird Refuge, Stockton Plateau

####### Time of activity.

Male (January, May – June, August); female (March – July, September)

####### Habitat.

(soil/woodland: post oak savanna with pasture)

####### Method.

pitfall trap [mf]; sweeping [f]

####### Type.

Arizona, Santa Catalina Mountains, Sabine Canyon

####### Etymology.

Person (The specific name is a patronym in honor of Dr. Willis J. Gertsch, who collected the type specimens and many other *Callilepis*, Platnick 1975a).

####### Collection.

DMNS, MSU, TAMU

###### Callilepis imbecilla

Taxon classificationAnimaliaAraneaeGnaphosidae

(Keyserling, 1887)

Callilepis imbecilla Agnew et al. 1985: 7; Bowen et al. 2004: 187; Jackman 1997: 163; Kaston 1978: 202; Platnick 1975a: 13 [S], mf, desc. (figs 22–28); Platnick and Dondale 1992: 195, desc. (figs 294–296); Trevino 2014: 11; Yantis 2005: 196, 199
Callilepis munda Chamberlin, 1936; Chamberlin 1936a: 16, mf, desc. (figs 22–24); Milstead 1958: 445; Vogel 1970b: 9

####### Distribution.

Central and south Texas; Aransas, Bell, Brewster, Brooks, Brown, Cameron, Erath, Fayette, Hidalgo, Houston, Kenedy, Kleberg, Leon, Nueces, Presidio, San Patricio, Sutton, Travis, Webb, Wichita, Williamson

####### Locality.

Black Gap Wildlife Management Area, Goose Island State Park, Kenedy Ranch, Santa Ana National Wildlife Refuge

####### Time of activity.

Male (March – July, August – September); female (April – May, July – September)

####### Habitat.

(littoral: near pond under oak, sand dune under live oak); (nest/prey: stomach of *Cnemidophorus perplexus*, stomach of *Cnemidophorus tigris*); (soil/woodland: leaf litter, pine woods [%: 67], post oak woods [%: 43])

####### Method.

5 gallon bucket trap [mf]; pitfall trap [mf] (near pond [m], under oak [m]); yellow pan trap [m]

####### Type.

Kentucky, Bee Spring

####### Etymology.

Latin, feeble

####### Collection.

DMNS, MSU, TAMU

###### Callilepis mumai

Taxon classificationAnimaliaAraneaeGnaphosidae

Platnick, 1975

Callilepis mumai [Platnick 1975a: 21, mf, desc. (figs 46–52)]

####### Distribution.

Ward

####### Time of activity.

Male (July)

####### Habitat.

(soil/woodland: on ground)

####### Type.

New Mexico, White Sands National Monument

####### Etymology.

Person (The specific name is a patronym in honor of Dr. Martin H. Muma, who collected the type specimens as well as many other *Callilepis* from the southwestern United States, Platnick 1975a).

####### Collection.

TAMU

##### Genus *Camillina* Berland, 1919

**Note.** Trevino (2014: 11) recorded *Camillina elegans* (Bryant, 1940) from Webb Co. Because of the distance between collecting sites (Florida), it is not included in this list. See Platnick and Shadab (1982b: 4).

###### Camillina pulchra

Taxon classificationAnimaliaAraneaeGnaphosidae

(Keyserling, 1891)

Camillina pulchra Calixto et al. 2013: 180, 182, 186–189; Platnick 2001 [spelling]; Ubick 2005c: 108
Camillina pulcher (Keyserling, 1891); Trevino 2014: 11 [Platnick and Shadab 1982b: 22, mf, desc. (figs 61–64)]

####### Distribution.

Brazos, Burleson, Colorado, Comanche, Coryell, Robertson, Webb, Williamson

####### Locality.

5-Eagle Ranch, Attwater Prairie Chicken National Wildlife Refuge, Bill Haney Pecan Orchard, Holmes Pecan Orchard, NK Ranch, Stiles Farm Foundation

####### Time of activity.

Male (March – August, September 28-October 4, October – November); female (April – December)

####### Habitat.

(crops: cotton); (orchard: pecan); (soil/woodland: post oak savanna with pasture, woods)

####### Method.

cardboard band [mf]; pitfall trap [mf]; tile trap [m]

####### Type.

Brazil, Rio Grande

####### Etymology.

Latin, beautiful

####### Collection.

TAMU

####### Note.

This species was introduced into the United States from Brazil. It was recorded in Alabama on December 6, 1975. The earliest Texas county records include: Brazos (m, November 5–12, 2002), (mf, June 13–20, 2003); Burleson (mf, June 12–19, 2003); Colorado (f, May 22–29, 2007); Comanche (mf, June 14–21, 2000); Coryell (m, May 11–31, 2006), (f, July 5–12, 2006); Robertson (m, June 27-July 3, 2000), (f, August 15–23, 2000); Williamson (m, May 9, 2001), (f, June 13, 2001).

##### Genus *Cesonia* Simon, 1893

###### Cesonia bilineata

Taxon classificationAnimaliaAraneaeGnaphosidae

(Hentz, 1847)

Cesonia bilineata Agnew et al. 1985: 7; Bowen et al. 2004: 188; Brown 1974: 233; Calixto et al. 2013: 182; Jackman 1997: 163; Platnick and Shadab 1980b: 342, mf, desc. (figs 1–7); Trevino 2014: 11; Yantis 2005: 199

####### Distribution.

Widespread; Anderson, Angelina, Bastrop, Bexar, Brazos, Brown, Burleson, Cameron, Colorado, Comanche, Coryell, Dallas, Edwards, Erath, Fort Bend, Hays, Hidalgo, Kenedy, Kerr, Kleberg, Lee, Montague, Nacogdoches, Sabine, San Patricio, Starr, Travis, Trinity, Tyler, Webb, Wichita

####### Locality.

Angelina National Forest, Attwater Prairie Chicken National Wildlife Refuge, Bastrop State Park, Bill Haney Pecan Orchard, Brazos Bend State Park, Falcon State Park, Kenedy Ranch, Kirby State Forest, Lake Somerville State Park [Nails Creek Unit], Lick Creek Park, Lower Rio Grande Valley National Wildlife Refuge

####### Time of activity.

Male (February 25-March 30, March – September); female (March – September)

####### Habitat.

(grass: grass); (littoral: sedge meadow); (orchard: citrus, grapefruit, pecan, tangerine); (plants: miscellaneous vegetation); (soil/woodland: beech magnolia litter, buckeye-sycamore forest, forest litter, *Juniperus* managed plot, *Juniperus* unmanaged plot, loblolly pine managed, mesquite thicket, old field, pine woods [%: 84], post oak savanna with pasture, riparian mesquite forest, sandy area, upland deciduous forest); (structures: bathroom, indoors); (web: large spider web)

####### Method.

5 gallon bucket trap [m]; cardboard band [mf]; D-Vac suction [m]; flight intercept trap [m]; malaise trap [m]; pitfall trap [mf]; sweeping [mf]

####### Type.

North Carolina, Alabama

####### Etymology.

Latin, two longitudinal black lines on abdomen

####### Collection.

MSU, TAMU

###### Cesonia sincera

Taxon classificationAnimaliaAraneaeGnaphosidae

Gertsch & Mulaik, 1936

Cesonia sincera Agnew et al. 1985: 4; Bonnet 1956: 1026; Bowen et al. 2004: 188; Broussard and Horner 2006: 253; Gertsch and Mulaik 1936a: 10, mf, desc. (figs 12, 16); Jackman 1997: 163; Platnick and Shadab 1980b: 343, mf, desc. (figs 8–11); Richman et al. 2011a: 48; Roewer 1955: 412; Trevino 2014: 11; Tugmon et al. 1990: 44; Vogel 1970b: 9; Young and Edwards 1990: 17; Zolnerowich and Horner 1985: 80

####### Distribution.

Widespread; Archer, Brewster, Cameron, Childress, Colorado, Comanche, Hidalgo, Mason, Nueces, Presidio, San Patricio, Starr, Terrell, Tom Green, Val Verde, Van Zandt, Webb, Wichita

####### Locality.

Attwater Prairie Chicken National Wildlife Refuge, Big Bend National Park, Chihuahuan desert, Dalquest Research Site, Lake Wichita

####### Time of activity.

Male (March – April, July – September); female (March – October, December)

####### Habitat.

(crops: peanuts); (grass: grass); (landscape features: under rock); (littoral: along margin of reservoir and pond); (objects: on tarp); (orchard: grapefruit, tangerine)

####### Method.

pitfall trap [mf]; sweeping [mf]; yellow pan trap [m]

####### Type.

Texas (male, Starr Co., Rio Grande City, July 1934, S. Mulaik, holotype, AMNH)

####### Etymology.

Latin, genuine

####### Collection.

MSU, TAMU

##### Genus *Drassodes* Westring, 1851

###### Drassodes auriculoides

Taxon classificationAnimaliaAraneaeGnaphosidae

Barrows, 1919

Drassodes auriculoides Cokendolpher et al. 2008: 8, 18 [Platnick and Shadab 1976a: 18, mf, desc. (figs 49–56)]

####### Distribution.

Carson

####### Locality.

Pantex Plant

####### Habitat.

(littoral: near playa)

####### Method.

pitfall trap

####### Type.

Ohio, Rockbridge

####### Etymology.

Latin, epigynum consists of 3 lobes, middle nearly square, side ones each resembling a human ear

####### Collection.

WTAM

###### Drassodes gosiutus

Taxon classificationAnimaliaAraneaeGnaphosidae

Chamberlin, 1919

Drassodes gosiutus Agnew et al. 1985: 7; Bowen et al. 2004: 188; Cokendolpher et al. 2008: 8, 18; Jackman 1997: 163; Kaston 1978: 203, desc.; Platnick and Dondale 1992: 144, mf, desc. (figs 220–223); Platnick and Shadab 1976a: 12, mf, desc. (figs 29–36); Trevino 2014: 11; Zolnerowich and Horner 1985: 80

####### Distribution.

Archer, Bandera, Brewster, Brown, Carson, Dallam, Erath, Kimble, McCulloch, Swisher, Tarrant, Webb, Wichita

####### Locality.

Pantex Plant

####### Time of activity.

Male (October – November); female (February – May, July – August, October – November)

####### Habitat.

(grass: grassland, short grass); (landscape features: under rock); (littoral: near playa)

####### Method.

pitfall trap [mf]

####### Eggs/spiderlings.

Wichita [eggs in sac (60, 63, 82, 83, 95, 98, 105, 106, 116, 147)] [Zolnerowich and Horner 1985: 80]

####### Type.

Utah, Fillmore

####### Etymology.

referring to desert

####### Collection.

DMNS, MSU, TAMU, WTAM

###### Drassodes saccatus

Taxon classificationAnimaliaAraneaeGnaphosidae

(Emerton, 1890)

Drassodes saccatus Agnew et al. 1985: 7; Jackman 1997: 163; Platnick and Shadab 1976a: 15 [S], mf, desc. (figs 2, 37–48); Roberts 2001: 50; Zolnerowich and Horner 1985: 81
Drassodes robinsoni Chamberlin, 1919; Chamberlin 1936b: 8; Vogel 1970b: 9

####### Distribution.

Archer, Brewster, Briscoe, Clay, Erath, Jeff Davis, Potter, Wichita

####### Locality.

Lake McKenzie Park, Mount Locke Observatory, Wildcat Bluff Nature Center

####### Time of activity.

Male (February – May); female (March – May, July)

####### Habitat.

(landscape features: under [rock, stone]); (soil/woodland: under oak)

####### Method.

pitfall trap [m] (under oak [m])

####### Eggs/spiderlings.

Wichita [111 first instar in sac] [Zolnerowich and Horner 1985: 81]

####### Type.

Massachusetts, Melrose

####### Etymology.

Latin, a sack

####### Collection.

MSU, TAMU, WTAM

##### Genus *Drassyllus* Chamberlin, 1922

###### Drassyllus antonito

Taxon classificationAnimaliaAraneaeGnaphosidae

Platnick & Shadab, 1982

Drassyllus antonito Jackman 1997: 163; Platnick and Shadab 1982a: 35, mf, desc. (figs 83, 90–93); Trevino 2014: 11

####### Distribution.

Brewster, Coryell, Hardeman, Kimble, San Patricio, Webb

####### Locality.

Big Bend National Park, Medicine Mounds Ranch

####### Time of activity.

Male (March – June, August – September); female (February – April, June – July, November)

####### Habitat.

(soil/woodland: post oak savanna with pasture)

####### Method.

pitfall trap [mf]

####### Type.

New Mexico, Antonito

####### Etymology.

locality (The specific name is a noun in apposition taken from the type locality, Platnick and Shadab 1982a).

####### Collection.

TAMU, WTAM

###### Drassyllus aprilinus

Taxon classificationAnimaliaAraneaeGnaphosidae

(Banks, 1904)

Drassyllus aprilinus Agnew et al. 1985: 7; Bowen et al. 2004: 188; Calixto et al. 2013: 182; Cokendolpher and Reddell 2001b: 45; Henderson 2007: 52–53, 76, 79, 83; Jackman 1997: 115, 163; Platnick and Dondale 1992: 130, mf, desc. (figs 200–203); Platnick and Shadab 1982a: 48, mf, desc. (figs 128–133); Yantis 2005: 66, 196, 200

####### Distribution.

Eastern 2/3 Texas; Anderson, Angelina, Bandera, Bell, Brazos, Brewster, Burleson, Colorado, Comanche, Coryell, DeWitt, Erath, Gonzales, Grayson, Grimes, Hays, Houston, Jeff Davis, Kerr, Leon, Llano, Madison, Montague, Real, Refugio, Robertson, Sabine, San Patricio, Taylor, Travis, Trinity, Tyler, Walker, Wichita, Wood

####### Locality.

Angelina National Forest, Big Bend National Park, Big Thicket National Preserve, Bill Haney Pecan Orchard, Fort Hood, Holmes Pecan Orchard, Kirby State Forest, Lick Creek Park, Lost Maples State Park, Palmetto State Park, Texas A&M University Rangeland Area

####### Caves.

**Bell** (Price Pit Cave [Fort Hood])

####### Time of activity.

Male (January 27-February 24, January, March – May, September, October 27-November 11, November, December 16-January 26); female (January 27 – February 24, March – June, September, October 27 – November 11, November – December)

####### Habitat.

(landscape features: cave); (orchard: pecan); (soil/woodland: beech magnolia litter, disturbed habitat, forest litter, hardwood litter, *Juniperus* managed plot, *Juniperus* unmanaged plot, leaf litter, loblolly pine managed, loblolly pine unmanaged, magnolia litter, mixed hardwood leaf litter, oak leaf litter, old field, pine woods [%: 66, 80, 82, 86, 88, 95, 99, 100], post oak savanna with pasture, post oak woods [%: 49, 56, 77, 84, 91, 92, 94, 96], sandy area, under oak, upland deciduous forest, upland woods, woods)

####### Method.

5 gallon bucket trap [mf]; berlese funnel [mf]; flight intercept trap on ground [mf]; pitfall trap [mf] (in leaves [mf], in sand [f], under oak [f])

####### Type.

Maryland, Chevy Chase

####### Etymology.

Latin, month collected

####### Collection.

DMNS, MSU, TAMU

###### Drassyllus broussardi

Taxon classificationAnimaliaAraneaeGnaphosidae

Platnick & Horner, 2007

Drassyllus broussardi Platnick and Horner 2007: 197, mf, desc. (figs 1–4)

####### Distribution.

Brewster, Presidio

####### Locality.

Dalquest Research Site

####### Time of activity.

Male (July – September); female (July – September)

####### Method.

pitfall trap [mf]

####### Type.

Texas (male, Presidio Co., Dalquest Research Site, August 8, 2005, N. Horner, J. Rogers, holotype, AMNH)

####### Etymology.

Person (The specific name is a patronym in honor of Greg H. Broussard, in recognition of the many valuable specimens taken in his survey work at the type locality, Platnick and Horner 2007).

###### Drassyllus cerrus

Taxon classificationAnimaliaAraneaeGnaphosidae

Platnick & Shadab, 1982

Drassyllus cerrus Cokendolpher et al. 2008: 8, 20; Jackman 1997: 163; Platnick and Shadab 1982a: 42, m, desc. (figs 114–115); Trevino 2014: 11

####### Distribution.

Carson, Val Verde, Webb

####### Time of activity.

Male (February – March, November)

####### Habitat.

(grass: grassland); (littoral: near playa)

####### Type.

Texas (male, Val Verde Co., Langtry, March 19, 1960, W. J. Gertsch, W. Ivie, R. Schrammel, holotype, AMNH)

[female unknown]

####### Etymology.

arbitrary combination of letters

###### Drassyllus conformans

Taxon classificationAnimaliaAraneaeGnaphosidae

Chamberlin, 1936

Drassyllus conformans Trevino 2014: 11 [Platnick and Shadab 1982a: 28, mf, desc. (figs 66–71)]

####### Distribution.

Webb

####### Time of activity.

Male (June)

####### Method.

pitfall trap [m]

####### Type.

California, Santa Monica

####### Etymology.

Latin, conforming

###### Drassyllus covensis

Taxon classificationAnimaliaAraneaeGnaphosidae

Exline, 1962

Drassyllus covensis Jackman 1997: 163 [Platnick and Shadab 1982a: 49, mf, desc. (figs 134–139)]

####### Distribution.

Houston, Sabine, Tyler, Walker

####### Locality.

Big Slough Wild Area, Big Thicket National Preserve, Huntsville State Park

####### Time of activity.

Female (April – May)

####### Habitat.

(soil/woodland: beech-magnolia litter, leaf litter)

####### Type.

Arkansas, Cove Creek

####### Etymology.

locality (creek)

####### Collection.

TAMU

###### Drassyllus creolus

Taxon classificationAnimaliaAraneaeGnaphosidae

Chamberlin & Gertsch, 1940

Drassyllus creolus Jackman 1997: 163; Platnick and Dondale 1992: 133, mf, desc. (figs 208–211); Platnick and Shadab 1982a: 56, mf, desc. (figs 152–153, 158–161); Yantis 2005: 200

####### Distribution.

Colorado, Jefferson, San Patricio, Trinity, Walker

####### Locality.

Attwater Prairie Chicken National Wildlife Refuge, Welder Wildlife Refuge

####### Time of activity.

Male (May); female (May, July)

####### Habitat.

(soil/woodland: live-oak woodland, pine woods [%: 84])

####### Method.

5 gallon bucket trap [m]; pitfall trap [f]

####### Type.

Louisiana, Baton Rouge

####### Etymology.

type of people in Louisiana

####### Collection.

TAMU

###### Drassyllus depressus

Taxon classificationAnimaliaAraneaeGnaphosidae

(Emerton, 1890)

Drassyllus depressus Cokendolpher et al. 2008: 8, 20; Trevino 2014: 11 [Platnick and Shadab 1982a: 12, mf, desc. (figs 18–23)]

####### Distribution.

Carson, Webb

####### Locality.

Pantex Plant

####### Time of activity.

Female (June)

####### Habitat.

(littoral: near playa)

####### Method.

pitfall trap

####### Type.

Massachusetts, Medford

####### Etymology.

Latin, pressed down low

####### Collection.

WTAM

###### Drassyllus dixinus

Taxon classificationAnimaliaAraneaeGnaphosidae

Chamberlin, 1922

Drassyllus dixinus Jackman 1997: 163; Platnick and Shadab 1982a: 24, mf, desc. (figs 54–59); Yantis 2005: 66, 197, 200
Drassyllus sp. nr dixinus Chamberlin, 1922; Henderson 2007: 64, 76, 79, 83 [misidentified]

####### Distribution.

Anderson, Angelina, Brazos, Coryell, Culberson, Harris, Harrison, Houston, Leon, Tyler

####### Locality.

Angelina National Forest, Kirby State Forest, Lick Creek Park

####### Time of activity.

Male (April – June); female (April 27-May 18, May – June, June 30-July 15)

####### Habitat.

(soil/woodland: loblolly pine managed, longleaf pine managed, old field, pine woods [%: 73, 74, 80, 83, 84, 88, 95, 100], post oak savanna with pasture, post oak woodland, post oak woods [%: 92], sandy area)

####### Method.

5 gallon bucket trap [mf]; pitfall trap [mf]

####### Type.

Louisiana, Mandeville

####### Etymology.

location (region)

####### Collection.

MSU, TAMU

###### Drassyllus dromeus

Taxon classificationAnimaliaAraneaeGnaphosidae

Chamberlin, 1922

Drassyllus dromeus Agnew et al. 1985: 7; Bowen et al. 2004: 188; Chamberlin 1922: 169, m, desc.; Chamberlin 1936a: 23; Jackman 1997: 115, 163; Platnick and Dondale 1992: 135, mf, desc. (figs 212–215); Platnick and Shadab 1982a: 62 [S], mf, desc. (figs 178–183); Roewer 1955: 414; Trevino 2014: 11; Vogel 1970b: 9; Yantis 2005: 197; Zolnerowich and Horner 1985: 81
Drassyllus devexus Chamberlin, 1936; Brown 1974: 233; Reddell 1970: 405; Smith and Reddell 1971: 24; Vogel 1970b: 9

####### Distribution.

Western 2/3 Texas; Archer, Brazos, Cameron, Comal, Erath, Hardeman, Hays, Hidalgo, Houston, Jeff Davis, Kerr, Kimble, Llano, Lubbock, Nacogdoches, Presidio, San Patricio, Starr, Travis, Tyler, Webb, Wichita

####### Locality.

Angelina National Forest, Kirby State Forest, Laguna Atascosa National Wildlife Refuge, Lower Rio Grande Valley National Wildlife Refuge, Medicine Mounds Ranch, Texas A&M University Rangeland Area

####### Caves.

**Kimble** (Garter Snake Cave)

####### Time of activity.

Male (January – May, November 20-December 4, December); female (February – June, October, December, December 17-January 8)

####### Habitat.

(grass: grass); (landscape features: cave, under rock); (objects: under [board, brick, sheet metal]); (soil/woodland: on ground in woods, *Juniperus* managed plot, *Juniperus* unmanaged plot, loblolly pine managed, pine woods [%: 95], post oak savanna with pasture, riparian mesquite forest, thorn thicket, under [bark, juniper, oak], willow tree bark); (structures: indoors, on floor in house)

####### Method.

5 gallon bucket trap [f]; carrion trap [m]; flight intercept trap on ground [m]; pitfall trap [mf] (under Juniper [m], under oak [f]); sweeping

####### Type.

Texas (male, Travis Co., Austin, no date, no collector, holotype, MCZ)

####### Etymology.

Greek, running

####### Collection.

DMNS, MSU, TAMU, TMM, WTAM

###### Drassyllus ellipes

Taxon classificationAnimaliaAraneaeGnaphosidae

Chamberlin & Gertsch, 1940

Drassyllus ellipes [Platnick and Shadab 1982a: 53, mf, desc. (figs 146–151)]

####### Distribution.

Tyler

####### Locality.

Kirby State Forest

####### Time of activity.

Male (March 30-April 27)

####### Method.

pitfall trap [m]

####### Type.

Alabama, Duncanville

####### Etymology.

Greek, elliptical

####### Collection.

TAMU

###### Drassyllus eremitus

Taxon classificationAnimaliaAraneaeGnaphosidae

Chamberlin, 1922

Drassyllus eremitus [Platnick and Shadab 1982a: 11, mf, desc. (figs 12–17)]

####### Distribution.

Tyler

####### Locality.

Kirby State Forest

####### Time of activity.

Male (March 30-April 27)

####### Method.

pitfall trap [m]

####### Type.

Tennessee, Glenraven

####### Etymology.

Greek, hermit

####### Collection.

TAMU

###### Drassyllus gynosaphes

Taxon classificationAnimaliaAraneaeGnaphosidae

Chamberlin, 1936

Drassyllus gynosaphes Agnew et al. 1985: 7; Bowen et al. 2004: 188; Chamberlin 1936b: 16, mf, desc. (figs 26–28); Cokendolpher and Reddell 2001b: 45; Jackman 1997: 163; Platnick and Shadab 1982a: 17, mf, desc. (figs 36–41); Reddell and Cokendolpher 2004: 85; Roewer 1955: 415; Trevino 2014: 11; Vogel 1970b: 9; Yantis 2005: 197, 200

####### Distribution.

Anderson, Angelina, Bell, Bexar, Cameron, Erath, Gonzales, Grimes, Hidalgo, Houston, Madison, Travis, Webb, Wichita

####### Locality.

Angelina National Forest, Fort Hood, Palmetto State Park

####### Caves.

**Bell** (Price Pit Cave [Fort Hood]); **Bexar** (Backhole)

####### Time of activity.

Male (March – May, December); female (February, April – June, October)

####### Habitat.

(landscape features: cave); (littoral: edge of pond, near pond); (soil/woodland: edge of woods, hardwood bottomland, leaf litter, loblolly pine managed, longleaf pine managed, oak leaf litter, pine woods [%: 67, 95], post oak woods [%: 56, 94], woods)

####### Method.

5 gallon bucket trap [mf]; berlese funnel [f]; pitfall trap [mf] (edge of pond [mf], edge of woods [m], in dead leaves [m], in woods [f], near pond [m])

####### Type.

Texas (female, Hidalgo Co., Edinburg, April 1934, S. Mulaik, holotype, AMNH)

####### Etymology.

Greek, female

####### Collection.

MSU, TAMU, TMM

###### Drassyllus inanus

Taxon classificationAnimaliaAraneaeGnaphosidae

Chamberlin & Gertsch, 1940

Drassyllus inanus Breene et al. 1993c: 16, 47, 87, mf (figs 105A-B); Calixto et al. 2013: 182; Cokendolpher et al. 2008: 8, 20; Jackman 1997: 163; Platnick and Shadab 1982a: 33, mf, desc. (figs 78–82)

####### Distribution.

Blanco, Brazos, Burleson, Caldwell, Cameron, Carson, Coleman, Comanche, Coryell, Hidalgo, Houston, Robertson, San Patricio

####### Locality.

Bentsen-Rio Grande Valley State Park, Bill Haney Pecan Orchard, Browning Ranch, Holmes Pecan Orchard, Horne Ranch, NK Ranch, Pantex Plant

####### Time of activity.

Male (March – October, December); female (March 30-April 6, April – August)

####### Habitat.

(crops: cotton); (grass: grassland); (littoral: near playa); (orchard: pecan); (soil/woodland: ground litter, mesquite woods, post oak savanna with pasture)

####### Method.

pitfall trap [mf]

####### Type.

Utah, Bluff

####### Etymology.

Latin, empty

####### Collection.

DMNS, TAMU, WTAM

###### Drassyllus lepidus

Taxon classificationAnimaliaAraneaeGnaphosidae

(Banks, 1899)

Drassyllus lepidus Agnew et al. 1985: 4; Bowen et al. 2004: 188; Calixto et al. 2013: 182, 187; Cokendolpher et al. 2008: 8, 20 (fig. 6); Irungu 2007: 30; Jackman 1997: 115, 163; Platnick and Shadab 1982a: 92 [S], mf, desc. (figs 270–271, 276–279); Roberts 2001: 50; Trevino 2014: 11; Young and Edwards 1990: 17; Zolnerowich and Horner 1985: 81
Drassyllus mephisto Chamberlin, 1936; Brown 1974: 233; Chamberlin 1936b: 14, mf, desc. (figs 3–5); Roewer 1955: 416; Vogel 1970b: 9

####### Distribution.

Widespread; Blanco, Bosque, Bowie, Brazos, Brewster, Brown, Burleson, Cameron, Carson, Clay, Comanche, Coryell, Erath, Hidalgo, Jeff Davis, Jim Wells, Kerr, Kimble, Montague, Potter, Randall, San Patricio, Shelby, Starr, Taylor, Travis, Webb, Wichita, Williamson

####### Locality.

5-Eagle Ranch, Bill Haney Pecan Orchard, Browning Ranch, Canoncita Ranch, Lake Meredith National Recreation Area, Palo Duro Canyon State Park, Pantex Lake (edge), Pantex Plant, Stiles Farm Foundation, Welder Wildlife Refuge, Wildcat Bluff Nature Center

####### Time of activity.

Male (January, March – August); female (March – October)

####### Habitat.

(crops: cabbage, cotton, peanuts); (grass: grass, grassland); (landscape features: under [concrete, rock]); (littoral: playa); (objects: under [board, sheet metal]); (orchard: pecan); (soil/woodland: oak leaf litter, post oak savanna with pasture, sandy area, under juniper); (structures: indoors, warehouse)

####### Method.

pitfall trap [mf] (in sand [m], under juniper [mf]); swine feces pitfall trap [m]

####### Type.

Louisiana, Shreveport

####### Etymology.

Greek, scales

####### Collection.

DMNS, MSU, TAMU, WTAM

###### Drassyllus mexicanus

Taxon classificationAnimaliaAraneaeGnaphosidae

(Banks, 1898)

Drassyllus mexicanus Trevino 2014: 11 [Platnick and Shadab 1982a: 65, mf, desc. (figs 196–199)]

####### Distribution.

Webb

####### Time of activity.

Male (October – November)

####### Type.

Mexico, Orizaba

####### Etymology.

locality (country)

###### Drassyllus mormon

Taxon classificationAnimaliaAraneaeGnaphosidae

Chamberlin, 1936

Drassyllus mormon Jackman 1997: 163 [Platnick and Shadab 1982a: 63, mf, desc. (figs 184–189)]

####### Distribution.

Jeff Davis

####### Time of activity.

Female (March)

####### Type.

Utah, St. George

####### Etymology.

Latin, religion

####### Collection.

MSU

###### Drassyllus mumai

Taxon classificationAnimaliaAraneaeGnaphosidae

Gertsch & Riechert, 1976

Drassyllus mumai Jackman 1997: 163; Platnick and Shadab 1982a: 70, mf, desc. (figs 206–211)

####### Distribution.

Brewster, Presidio

####### Type.

New Mexico, Carrizozo

####### Etymology.

Person (Named for Dr. Martin Muma of Silver City, New Mexico, student of solpugids and spiders, who has collected numerous examples of this distinctive species, Gertsch and Riechert 1976).

###### Drassyllus notonus

Taxon classificationAnimaliaAraneaeGnaphosidae

Chamberlin, 1928

Drassyllus notonus Agnew et al. 1985: 4; Bowen et al. 2004: 188; Breene et al. 1993c: 16, 47, 87, mf (figs 106A-B); Calixto et al. 2013: 182; Cokendolpher et al. 2008: 8, 20; Dean and Sterling 1990: 403; Dean et al. 1982: 255; Irungu 2007: 30; Jackman 1997: 115, 163; Platnick and Shadab 1982a: 30, mf, desc. (figs 72–77); Young and Edwards 1990: 17; Zolnerowich and Horner 1985: 81

####### Distribution.

Archer, Brazos, Brewster, Brown, Burleson, Carson, Comanche, Coryell, Dallas, Erath, Floyd, Grayson, Haskell, Hidalgo, Houston, Knox, Robertson, Walker, Wichita, Williamson

####### Locality.

Big Bend National Park, Bill Haney Pecan Orchard, Ellis Prison Unit, Holmes Pecan Orchard, Pantex Plant, Stiles Farm Foundation

####### Time of activity.

Male (February – August); female (March – September)

####### Habitat.

(crops: cabbage, cotton, peanuts); (grass: grass); (landscape features: under stone); (littoral: near playa); (objects: under board); (orchard: pecan); (plants: emergent vegetation, vegetation); (soil/woodland: on ground, post oak savanna with pasture); (structures: garage floor)

####### Method.

Boll weevil pheromone trap [f]; D-Vac suction [m]; pitfall trap [mf]; suction trap [m]; sweeping [m]

####### Type.

Utah, Noton

####### Etymology.

locality (town)

####### Collection.

DMNS, MSU, TAMU, WTAM

###### Drassyllus orgilus

Taxon classificationAnimaliaAraneaeGnaphosidae

Chamberlin, 1922

Drassyllus orgilus Agnew et al. 1985: 4; Bowen et al. 2004: 188; Calixto et al. 2013: 182; Chamberlin 1922: 169, f, desc.; Chamberlin 1936b: 13; Jackman 1997: 115, 163; Platnick and Shadab 1982a: 60, mf, desc. (figs 172–177); Roewer 1955: 416; Trevino 2014: 12; Vogel 1970b: 9; Yantis 2005: 197, 200; Young and Edwards 1990: 17; Zolnerowich and Horner 1985: 81

####### Distribution.

Eastern 2/3 Texas; Anderson, Archer, Bexar, Brazos, Brown, Burleson, Cameron, Clay, Dallas, Denton, DeWitt, Erath, Galveston, Gonzales, Hardeman, Hays, Hidalgo, Houston, Kerr, Kimble, Llano, McCulloch, Montgomery, Robertson, San Patricio, Starr, Sutton, Tarrant, Taylor, Tom Green, Travis, Webb, Wichita

####### Locality.

Holmes Pecan Orchard, Lick Creek Park, Medicine Mounds Ranch

####### Time of activity.

Male (February – May, July, September – December); female (January – June, August, October – December, December 16 – January 26)

####### Habitat.

(crops: peanuts); (grass: grass); (landscape features: under rock); (littoral: near [pond, near water]); (objects: under board); (orchard: pecan); (plants: vegetation); (soil/woodland: *Juniperus* managed plot, pine woods [%: 69, 85], post oak savanna with pasture, woods); (structures: indoors)

####### Method.

5 gallon bucket trap [m]; flight intercept trap on ground [f]; pitfall trap [mf] (near pond [f])

####### Type.

Texas (female, Travis Co., Austin, no date, no collector, holotype, MCZ)

####### Etymology.

Greek, irritable

####### Collection.

DMNS, MSU, TAMU, WTAM

###### Drassyllus prosaphes

Taxon classificationAnimaliaAraneaeGnaphosidae

Chamberlin, 1936

Drassyllus prosaphes Broussard and Horner 2006: 253; Chamberlin 1936b: 16, m, desc. (figs 29–30); Jackman 1997: 115, 163; Platnick and Shadab 1982a: 89, mf, desc. (figs 264–269); Richman et al. 2011a: 48; Roewer 1955: 416; Trevino 2014: 12; Vogel 1970b: 9

####### Distribution.

Angelina, Aransas, Bee, Bexar, Brewster, Brooks, Hidalgo, Kleberg, Presidio, San Patricio, Taylor, Webb

####### Locality.

Angelina National Forest, Bentsen-Rio Grande Valley State Park, Chihuahuan desert, Dalquest Research Site

####### Caves.

**Brewster** (O.T.L. Cave)

####### Time of activity.

Male (April – June); female (April – June)

####### Habitat.

(landscape features: cave); (plants: *Opuntia* sp.); (soil/woodland: ground litter, hardwood bottomland, leaf litter, mesquite woods)

####### Method.

pitfall trap [mf]

####### Type.

Texas (male, Hidalgo Co., Edinburg, May 2, 1935, S. Mulaik, holotype, AMNH)

####### Etymology.

Greek, face

####### Collection.

MSU, TAMU, TMM

###### Drassyllus rufulus

Taxon classificationAnimaliaAraneaeGnaphosidae

(Banks, 1892)

Drassyllus rufulus Jackman 1997: 163; Platnick and Dondale 1992: 126, mf, desc. (figs 192–195); Platnick and Shadab 1982a: 44, mf, desc. (figs 122–127); Trevino 2014: 12; Yantis 2005: 197

####### Distribution.

Anderson, Brazos, Colorado, Hardin, Webb

####### Locality.

Lick Creek Park

####### Time of activity.

Male (July, November); female (October – November)

####### Habitat.

(soil/woodland: forest, pine woods [%: 69], woods)

####### Method.

5 gallon bucket trap [mf]; pitfall trap [f]

####### Type.

New York, Ithaca

####### Etymology.

Latin, color reddish

####### Collection.

TAMU

###### Drassyllus sinton

Taxon classificationAnimaliaAraneaeGnaphosidae

Platnick & Shadab, 1982

Drassyllus sinton Jackman 1997: 163; Platnick and Shadab 1982a: 34, mf, desc. (figs 84–89)

####### Distribution.

Cameron, Hays, Hidalgo, San Patricio

####### Time of activity.

Male (April – May, September – October, December); female (September, December)

####### Type.

Texas (male, San Patricio Co., 8 miles NE Sinton, September 4, 1959, H. E. Laughlin, holotype, AMNH)

####### Etymology.

locality (The specific name is a noun in apposition taken from the type locality, Platnick and Shadab 1982a).

###### Drassyllus texamans

Taxon classificationAnimaliaAraneaeGnaphosidae

Chamberlin, 1936

Drassyllus texamans Agnew et al. 1985: 7; Bowen et al. 2004: 189; Calixto et al. 2013: 182; Chamberlin 1936b: 17, f, desc. (fig. 25); Cokendolpher and Reddell 2001b: 45; Jackman 1997: 115, 163; Platnick and Shadab 1982a: 26 [S], mf, desc. (figs 60–65); Reddell 1965: 171; Roewer 1955: 417; Vogel 1970b: 9; Zolnerowich and Horner 1985: 81
Drassyllus finium Chamberlin, 1936; Chamberlin 1936b: 15, m, desc. (figs 33–34); Roewer 1955: 415; Vogel 1970b: 9

####### Distribution.

Widespread; Bandera, Bell, Brazos, Brewster, Brown, Burleson, Cameron, Colorado, Comanche, Coryell, Dallas, Edwards, Erath, Hardeman, Harris, Hays, Hidalgo, Kerr, Kleberg, Pecos, Potter, Presidio, Randall, Robertson, San Patricio, Sutton, Terrell, Throckmorton, Tom Green, Travis, Wichita, Williamson

####### Locality.

Attwater Prairie Chicken National Wildlife Refuge, Bill Haney Pecan Orchard, Canoncita Ranch, Fort Hood, Holmes Pecan Orchard, Lake Meredith, Lost Maples State Park, Palo Duro Canyon State Park, Perkins Scout Reservation, Raven Ranch, Santa Ana National Wildlife Refuge, Stiles Farm Foundation, Texas A&M University Rangeland Area, Welder Wildlife Refuge

####### Caves.

**Bell** (Newby Cave [Fort Hood]); **Brewster** (O.T.L. Cave); **Hardeman** (Campsey Cave)

####### Time of activity.

Male (March – July); female (March 3-April 4, April – August, December)

####### Habitat.

(crops: cotton); (grass: grass, grasses); (landscape features: cave); (littoral: near pond); (objects: under barrel); (orchard: pecan); (soil/woodland: anacua groves, forest, ground, *Juniperus* unmanaged plot, leaf litter, live oak woodland, oak litter, open field, pine litter, post oak savanna with pasture, *Quercus* litter, sandy area, sandy terrace along river, under [juniper, oak], woods)

####### Method.

Berlese funnel [mf]; carrion pitfall trap [m]; flight intercept trap [f]; flight intercept trap on ground [m]; pitfall trap [mf] (in sand [mf], in woods [m], near pond [m], under juniper [mf], under oak [mf]); ramp trap [f]; tile trap [f]

####### Type.

Texas (female, Terrell Co., Sanderson, July 4, 1934, S. Mulaik, holotype, AMNH)

####### Etymology.

locality (state)

####### Collection.

DMNS, MSU, TAMU, TMM, WTAM

##### Genus *Eilica* Keyserling, 1891

###### Eilica bicolor

Taxon classificationAnimaliaAraneaeGnaphosidae

Banks, 1896

Eilica bicolor Jackman 1997: 163; Platnick 1975b: 10, mf, desc. (figs 1, 20–23); Roth 1982: 20–2; Roth 1985: B-16–2; Roth 1994: 97; Ubick 2005c: 109

####### Distribution.

Cameron, Hidalgo, Starr

####### Locality.

Laguna Atascosa National Wildlife Refuge, Lower Rio Grande Valley National Wildlife Refuge, Resaca de la Palma State Park, Santa Ana National Wildlife Refuge

####### Time of activity.

Male (March 3-April 4, April); female (January 28 – March 1, March 4-April 3, November)

####### Habitat.

(soil/woodland: forest, riparian mesquite forest, thorn thicket)

####### Method.

carrion flight intercept trap [f]; flight intercept trap [mf]

####### Type.

Florida, Punta Gorda

####### Etymology.

Latin, cephalothorax and abdomen two colors

####### Collection.

TAMU

##### Genus *Gertschosa* Platnick & Shadab, 1981

###### Gertschosa amphiloga

Taxon classificationAnimaliaAraneaeGnaphosidae

(Chamberlin, 1936)

Gertschosa amphiloga Jackman 1997: 163; Platnick and Shadab 1981a: 180 [T], f, desc. (figs 11–12); Roth 1982: 20–3; Roth 1985: B-16–3; Roth 1994: 98; Ubick 2005c: 110
Sergiolus amphilogus Chamberlin, 1936; Chamberlin 1936b: 4, f, desc. (fig. 18); Roewer 1955: 438; Vogel 1970b: 10

####### Distribution.

Cameron

####### Time of activity.

Female (June)

####### Type.

Texas (female, Cameron Co., Brownsville, June 1, 1934, J. N. Knull, holotype, AMNH)

[male unknown]

####### Etymology.

Greek, gather around

##### Genus *Gnaphosa* Latreille, 1804

###### Gnaphosa altudona

Taxon classificationAnimaliaAraneaeGnaphosidae

Chamberlin, 1922

Gnaphosa altudona Bonnet 1957: 2003; Breene et al. 1993c: 16, 47, 88, mf (figs 108A–B); Chamberlin 1922: 157, f, desc.; Cokendolpher et al. 2008: 8, 20; Irungu 2007: 30; Jackman 1997: 163; Platnick and Shadab 1975a: 30, mf, desc. (figs 59–64); Roewer 1955: 369; Trevino 2014: 12; Zolnerowich and Horner 1985: 81
Gnaphosa altadora Chamberlin, 1922; Vogel 1970b: 9
Gnaphosa sp.; Milstead 1958: 446

####### Distribution.

Brewster, Burleson, Carson, Coryell, Hidalgo, Presidio, San Patricio, Tom Green, Webb, Wichita, Williamson

####### Locality.

Chisos Basin, Chisos Mountains, La Mota Mountains, Pantex Plant, Stiles Farm Foundation

####### Time of activity.

Male (April – July); female (March, June – August)

####### Habitat.

(crops: cabbage, cotton); (landscape features: under [rock, stone]); (littoral: near playa); (nest/prey: stomach of *Cnemidophorus tigris*); (soil/woodland – post oak savanna with pasture)

####### Method.

pitfall trap [mf]

####### Eggs/spiderlings.

Wichita [27 first instar in sac] [Zolnerowich and Horner 1985: 81]

####### Type.

Texas (female, Brewster Co., Altudo, no date, no collector, holotype, MCZ)

####### Etymology.

locality (town)

####### Collection.

MSU, TAMU, WTAM

###### Gnaphosa clara

Taxon classificationAnimaliaAraneaeGnaphosidae

(Keyserling, 1887)

Gnaphosa clara Jackman 1997: 163; Platnick and Dondale 1992: 168, mf, desc. (figs 252–255); Platnick and Shadab 1975a: 12 [S], mf, desc. (figs 11–16); Zolnerowich and Horner 1985: 82
Gnaphosa mulaiki Chamberlin, 1936; Chamberlin 1936b: 6, f, desc. (fig. 24); Roewer 1955: 370

####### Distribution.

Cameron, Lubbock, Lynn, Wichita, Williamson

####### Locality.

Green Island Bird Refuge, Stiles Farm Foundation

####### Time of activity.

Male (June); female (May – June)

####### Habitat.

(crops: cotton); (objects: under wood)

####### Method.

pitfall trap [m]

####### Eggs/spiderlings.

Wichita [59 eggs in sac] [Zolnerowich and Horner 1985: 82]

####### Type.

Utah, Bridger Basin

####### Etymology.

Latin, clear

####### Collection.

JCC, MSU, TAMU, WTAM

###### Gnaphosa fontinalis

Taxon classificationAnimaliaAraneaeGnaphosidae

Keyserling, 1887

Gnaphosa fontinalis Agnew et al. 1985: 4; Bowen et al. 2004: 189; Bradley 2013: 127; Broussard and Horner 2006: 254; Chamberlin 1922: 157; Cokendolpher and Reddell 2001b: 45; Jackman 1997: 163; Platnick and Dondale 1992: 163, mf, desc. (figs 244–247); Platnick and Shadab 1975a: 54 [S], mf, desc. (figs 127–134, 150); Richman et al. 2011a: 48; Roberts 2001: 50; Vogel 1970b: 10; Yantis 2005: 66, 197; Young and Edwards 1990: 17; Zolnerowich and Horner 1985: 82
Gnaphosa texana Chamberlin, 1922; Bonnet 1957: 2022; Chamberlin 1922: 157, m, desc.; Roewer 1955: 371; Vogel 1970b: 10

####### Distribution.

Anderson, Angelina, Bell, Brown, Coryell, Dallas, Erath, Hays, Houston, Kerr, Leon, Llano, Montague, Potter, Presidio, Sabine, Smith, Travis, Wichita

####### Locality.

Angelina National Forest, Chihuahuan desert, Dalquest Research Site, Lake Meredith National Recreation Area, Raven Ranch, Tyler State Park, White Rock Lake, Wildcat Bluff Nature Center

####### Caves.

**Bell** (Cub Cave); **Hays** (Ezell’s Cave)

####### Time of activity.

Male (April – July, October); female (April – August)

####### Habitat.

(crops: peanuts); (landscape features: cave, under rock); (littoral: near pond); (plants: herbs near water); (soil/woodland: beech-magnolia forest, *Juniperus* managed plot, *Juniperus* unmanaged plot, leaf litter, loblolly pine managed, loblolly pine unmanaged, pine woods [%: 79, 83, 99], post oak savanna with pasture, post oak woods [%: 44, 56, 82, 91], sandy area, under [juniper, oak], upland deciduous forest)

####### Method.

5 gallon bucket trap [mf]; flight intercept trap on ground [mf]; malaise trap [f]; pitfall trap [mf] (in leaves [mf], in sand [m], near pond [m], under juniper [mf], under oak [mf])

####### Type.

Kentucky, Bee Spring

####### Etymology.

Latin, of a spring

####### Collection.

DMNS, MCZ, MSU, TAMU, TMM, WTAM

###### Gnaphosa saxosa

Taxon classificationAnimaliaAraneaeGnaphosidae

Platnick & Shadab, 1975

Gnaphosa saxosa [Platnick and Shadab 1975a: 17, mf, desc. (figs 29–34)]

####### Distribution.

Hardeman, Knox, Presidio

####### Locality.

Big Bend Ranch State Park, Medicine Mounds Ranch

####### Time of activity.

Male (July – August); female (March, July)

####### Habitat.

(crops: cotton); (landscape features: under rock)

####### Method.

pitfall trap [mf]

####### Type.

Colorado, Boone

####### Etymology.

Latin, saxosus (rocky) and refers to one habitat of this species under rocks

####### Collection.

NMSU, TAMU, WTAM

###### Gnaphosa sericata

Taxon classificationAnimaliaAraneaeGnaphosidae

(L. Koch, 1866)

Gnaphosa sericata Agnew et al. 1985: 4; Bowen et al. 2004: 189; Breene et al. 1993c: 16, 47, 88, mf (figs 109A-B); Brown 1974: 234; Calixto et al. 2013: 182; Chamberlin 1922: 157; Dean et al. 1982: 255; Henderson 2007: 63–64, 76, 79, 83; Irungu 2007: 30; Jackman 1997: 163; Kaston 1953: 75, desc. (fig. 182); Platnick and Shadab 1975a: 61, mf, desc. (figs 143–149); Ramirez 2014: 363; Roberts 2001: 50; Trevino 2014: 12; Vogel 1970b: 10; Yantis 2005: 197; Young and Edwards 1990: 17; Zolnerowich and Horner 1985: 82

####### Distribution.

Widespread; Bastrop, Brazos, Brewster, Brown, Burleson, Coleman, Colorado, Comal, Comanche, Coryell, Cottle, Denton, Erath, Frio, Garza, Hidalgo, Houston, Jeff Davis, Kenedy, Kerr, La Salle, Nacogdoches, Nolan, Potter, San Patricio, Somervell, Tom Green, Travis, Walker, Webb, Wichita, Wilbarger, Zapata

####### Locality.

Attwater Prairie Chicken National Wildlife Refuge, Bentsen-Rio Grande Valley State Park, Bill Haney Pecan Orchard, Chaparral Wildlife Management Area, Ellis Prison Unit, Falcon Reservoir, Horne Ranch, Kenedy Ranch, Lick Creek Park, Matador Wildlife Management Area, Raven Ranch, Somerville Lake, Welder Wildlife Refuge, Wildcat Bluff Nature Center

####### Time of activity.

Male (March – October); female (March – October)

####### Habitat.

(crops: cabbage, cotton, peanuts); (grass: grass); (landscape features: under rock); (orchard: pecan); (soil/woodland: acacia area, disturbed habitat, pine woods [%: 79], post oak savanna with pasture, post oak woodland, sandy brushland, sandy open prairie, sandy area, under [cow manure, oak], upland woods); (structures: in house, on ground near house)

####### Method.

5 gallon bucket trap [f]; pitfall trap [mf] (in sand [m], under oak [f]); swine feces pitfall trap [m]

####### Type.

Maryland, Baltimore

####### Etymology.

Greek, silk

####### Collection.

DMNS, MSU, TAMU, WTAM

##### Genus *Haplodrassus* Chamberlin, 1922

###### Haplodrassus chamberlini

Taxon classificationAnimaliaAraneaeGnaphosidae

Platnick & Shadab, 1975

Haplodrassus chamberlini Bowen et al. 2004: 189; Broussard and Horner 2006: 254; Jackman 1997: 163; Platnick and Dondale 1992: 213, mf, desc. (figs 317–321); Richman et al. 2011a: 48; Zolnerowich and Horner 1985: 82 [Platnick and Shadab 1975b: 27, mf, desc. (figs 63–70)]

####### Distribution.

Brown, Culberson, Deaf Smith, Parker, Presidio, Reeves, Wichita

####### Locality.

Chihuahuan desert, Dalquest Research Site

####### Time of activity.

Male (March – May); female (March – May)

####### Habitat.

(grass: grassy pasture); (landscape features: under [rock, stone])

####### Method.

pitfall trap [mf]

####### Type.

Oklahoma, Felt

####### Etymology.

Person (The specific name is a patronym in honor of the late Dr. R. V. Chamberlin, in recognition of his pioneering work on *Haplodrassus* and *Orodrassus*, Platnick and Shadab 1975b).

####### Collection.

MSU, WTAM

###### Haplodrassus dixiensis

Taxon classificationAnimaliaAraneaeGnaphosidae

Chamberlin & Woodbury, 1929

Haplodrassus dixiensis Jackman 1997: 163 [Platnick and Shadab 1975b: 23, mf, desc. (figs 39–46)]

####### Distribution.

Brown, Presidio

####### Time of activity.

Female (March)

####### Habitat.

(soil/woodland: ground)

####### Type.

Utah, St. George

####### Etymology.

Latin, region

####### Collection.

MSU

###### Haplodrassus signifer

Taxon classificationAnimaliaAraneaeGnaphosidae

(C. L. Koch, 1839)

Haplodrassus signifer Agnew et al. 1985: 4; Bowen et al. 2004: 189; Jackman 1997: 163; Platnick and Shadab 1975b: 11, mf, desc. (figs 11–22); Roberts 2001: 50; Young and Edwards 1990: 17; Zolnerowich and Horner 1985: 82

####### Distribution.

Baylor, Brown, Coryell, Erath, Hardeman, Hutchinson, Kerr, McLennan, Potter, Tom Green, Travis, Wichita

####### Locality.

Lake Meredith National Recreation Area, Medicine Mounds Ranch, Wildcat Bluff Nature Center

####### Time of activity.

Male (February – May, September); female (February – May)

####### Habitat.

(crops: peanuts); (landscape features: under [rock, stone]); (littoral: near pond); (soil/woodland: edge of woods, post oak savanna with pasture, under juniper, upland deciduous forest)

####### Method.

Flight intercept trap [m]; pitfall trap [mf] (border/edge of woods [mf], near pond [f], under juniper [m])

####### Eggs/spiderlings.

Wichita [232 second instar, 50 first instar spiderlings; 94 first instar, 24 eggs; 60 second instar, 26 first instar] [Zolnerowich and Horner 1985: 82]

####### Type.

Czechoslovakia, Bohemia, near Karlsbad

####### Etymology.

Latin, a sign

####### Collection.

DMNS, MSU, TAMU, WTAM

##### Genus *Herpyllus* Hentz, 1832

###### Herpyllus bubulcus

Taxon classificationAnimaliaAraneaeGnaphosidae

Chamberlin, 1922

Herpyllus bubulcus Bonnet 1957: 2171; Broussard and Horner 2006: 254; Chamberlin 1922: 150, f, desc.; Jackman 1997: 163; Platnick and Shadab 1977: 29, mf, desc. (figs 85–90); Richman et al. 2011a: 48; Roewer 1955: 422; Trevino 2014: 12; Vogel 1970b: 10; Zolnerowich and Horner 1985: 82

####### Distribution.

Armstrong, Brewster, Culberson, El Paso, Hardeman, Jeff Davis, Kendall, Llano, Parmer, Pecos, Potter, Presidio, Randall, Reeves, Sutton, Travis, Webb

####### Locality.

Big Bend National Park, Chihuahuan desert, Chisos Basin, Chisos Mountains, Dalquest Research Site, Lake Meredith National Recreation Area, Lake Tanglewood, Medicine Mounds Ranch, Palo Duro Canyon

####### Time of activity.

Male (February, November); female (March)

####### Habitat.

(landscape features: rock pile, under rock)

####### Method.

pitfall trap [mf]

####### Type.

Texas (female, Brewster Co., Altudo, no date, no collector, holotype, MCZ)

####### Etymology.

Latin, herdsman

####### Collection.

MSU, WTAM

###### Herpyllus cockerelli

Taxon classificationAnimaliaAraneaeGnaphosidae

(Banks, 1901)

Herpyllus cockerelli Jackman 1997: 163; Platnick and Shadab 1977: 15, mf, desc. (figs 31–36)

####### Distribution.

Brewster, Brown, Coryell, Jeff Davis, Sutton

####### Locality.

Big Bend National Park, Chisos Mountains

####### Time of activity.

Male (December); female (February, March)

####### Habitat.

(soil/woodland: under bark)

####### Type.

New Mexico, Mesilla Park

####### Etymology.

Person (arachnologist/entomologist Theodore Dru Alison Cockerell, professor at New Mexico Agricultural College (now New Mexico State University, Las Cruces)

####### Collection.

MSU

###### Herpyllus ecclesiasticus

Taxon classificationAnimaliaAraneaeGnaphosidae

Hentz, 1832

Herpyllus ecclesiasticus Agnew et al. 1985: 7; Bumroongsook et al. 1992: 18; Calixto et al. 2013: 182, 189; Jackman 1997: 115, desc., 163 (photo 35c); Lombardini et al. 2005: 1378; Platnick and Dondale 1992: 273, mf, desc. (figs 2–3, 14, 433–436); Platnick and Shadab 1977: 7 [S], mf, desc. (figs 1–8); Roberts 2001: 50; Yantis 2005: 197; Zolnerowich and Horner 1985: 82
Herpyllus vasifer (Walckenaer, 1837); Brown 1974: 234
Herpyllus cratus Chamberlin, 1922; Chamberlin 1936b: 1; Roewer 1955: 422; Vogel 1970b: 10

####### Distribution.

Widespread; Archer, Bailey, Bastrop, Bexar, Brazos, Brewster, Burleson, Carson, Clay, Comanche, Dallas, Denton, Eastland, Erath, Galveston, Gray, Grayson, Hardeman, Hardin, Hays, Hidalgo, Houston, Hutchinson, Kendall, Kerr, Llano, Lubbock, Lynn, Nacogdoches, Parmer, Potter, Randall, Robertson, Sutton, Swisher, Taylor, Travis, Uvalde, Wichita

####### Locality.

Bastrop State Park, Bill Haney Pecan Orchard, Holmes Pecan Orchard, Medicine Mounds Ranch, Santa Ana National Wildlife Refuge, Somerville Lake, Storey Pecan Orchard, Wildcat Bluff Nature Center

####### Time of activity.

Male (February – March, May – July, September – November); female (January – September, November)

####### Habitat.

(grass: grass); (landscape features: under rock); (orchard: pecan); (soil/woodland: *Juniperus* managed plot, on tree, pine woods [%: 88], under bark, woods); (structures: garage, house, indoors, on [floor in house, wall in house])

####### Method.

5 gallon bucket trap [f]; cardboard band [mf]; flight intercept trap elevated [f]; fogging [f]; malaise trap [m]; pitfall trap [mf]; ramp trap [m]; suction trap [m]

####### Type.

United States

####### Etymology.

Greek, assembly

####### Collection.

MSU, TAMU, TTU, WTAM

###### Herpyllus gertschi

Taxon classificationAnimaliaAraneaeGnaphosidae

Platnick & Shadab, 1977

Herpyllus gertschi Jackman 1997: 163; Platnick and Shadab 1977: 35, mf, desc. (figs 57–58, 103–106)

####### Distribution.

Brewster

####### Locality.

Big Bend National Park, Chisos Basin, Chisos Mountains

####### Type.

Arizona, Southwestern Research Station

####### Etymology.

Person (The specific name is a patronym in honor of Dr. Willis J. Gertsch, who first recognized the species as new, Platnick and Shadab 1977).

###### Herpyllus hesperolus

Taxon classificationAnimaliaAraneaeGnaphosidae

Chamberlin, 1928

Herpyllus hesperolus Bradley 2013: 128; Jackman 1997: 163; Platnick and Dondale 1992: 271, mf, desc. (figs 429–432); Platnick and Shadab 1977: 23, mf, desc. (figs 63–68); Zolnerowich and Horner 1985: 82

####### Distribution.

Brewster, Hudspeth, Pecos, Sutton, Wichita

####### Time of activity.

Male (May); female (February – March)

####### Habitat.

(landscape features: rocky hillside, rock pile, under rock)

####### Type.

California, Los Angeles

####### Etymology.

Greek, western

####### Collection.

MSU

###### Herpyllus propinquus

Taxon classificationAnimaliaAraneaeGnaphosidae

(Keyserling, 1887)

Herpyllus propinquus Jackman 1997: 163; Platnick and Shadab 1977: 9, mf, desc. (figs 9–14)

####### Distribution.

El Paso, Hudspeth, Presidio

####### Locality.

Big Bend Ranch State Park

####### Time of activity.

Male (March)

####### Type.

California, Santa Barbara

####### Etymology.

Latin, near

####### Collection.

MSU, NMSU, WTAM

###### Herpyllus regnans

Taxon classificationAnimaliaAraneaeGnaphosidae

Chamberlin, 1936

Herpyllus regnans Chamberlin 1936b: 2, f, desc. (fig. 14); Jackman 1997: 163; Platnick and Shadab 1977: 31, mf, desc. (figs 91–94); Roewer 1955: 423; Vogel 1970b: 10

####### Distribution.

Brown, Crockett, Grayson, Kendall, Kerr, Llano, Sutton, Zapata

####### Locality.

Lake Texoma, Raven Ranch

####### Time of activity.

Male (December); female (February, May, December)

####### Habitat.

(landscape features: under rock); (soil/woodland: under bark)

####### Type.

Texas (female, Zapata Co., Arroyo Solado, no date, S. Mulaik, holotype, AMNH)

####### Etymology.

Latin, reign

####### Collection.

MSU

##### Genus *Litopyllus* Chamberlin, 1922

###### Litopyllus temporarius

Taxon classificationAnimaliaAraneaeGnaphosidae

Chamberlin, 1922

Litopyllus temporarius Henderson 2007: 60, 62, 76, 79, 83; Yantis 2005: 66, 197 [Platnick and Shadab 1980a: 17, mf, desc. (figs 27–30)]

####### Distribution.

Anderson, Angelina, Brazos, Burleson, Houston, Leon, Sabine, Tyler

####### Locality.

Angelina National Forest, Kirby State Forest, Lick Creek Park

####### Time of activity.

Male (March 30-April 27, April – June)

####### Habitat.

(soil/woodland: beech-magnolia forest, disturbed habitat, loblolly pine unmanaged, pine woods [%: 80, 84, 100], post oak savanna with pasture, post oak woods [%: 56, 71], upland woods)

####### Method.

5 gallon bucket trap [m]; flight intercept trap [m]; malaise trap [m]; pitfall trap [m]

####### Type.

Kentucky, near Mammoth Cave

####### Etymology.

Latin, time

####### Collection.

TAMU

##### Genus *Micaria* Westring, 1851

###### Micaria deserticola

Taxon classificationAnimaliaAraneaeGnaphosidae

Gertsch, 1933

Micaria deserticola Breene et al. 1993c: 16, 47, 86, mf (figs 103A-B); Calixto et al. 2013: 182; Irungu 2007: 30; Jackman 1997: 163; Platnick and Shadab 1988: 59, mf, desc. (figs 150–153)
Micaria sp.; Agnew et al. 1985: 4 [part]; Dean and Sterling 1987: 6 [part]; Young and Edwards 1990: 17 [part]

####### Distribution.

Brewster, Burleson, Colorado, Comanche, Coryell, Hidalgo, Howard, Kenedy

####### Locality.

Attwater Prairie Chicken National Wildlife Refuge, Big Bend National Park, Bill Haney Pecan Orchard, Kenedy Ranch

####### Time of activity.

Male (March – August, October); female (March – August, October 26 – November 2, November – December)

####### Habitat.

(crops: cabbage, cotton, peanuts); (orchard: pecan); (soil/woodland: forest litter, post oak savanna with pasture)

####### Method.

pitfall trap [mf]; yellow pan trap [m]

####### Type.

Arizona, Scottsdale

####### Etymology.

Latin, place, deserts (habitat); -cola Latin suffix meaning inhabitant of

####### Collection.

TAMU

###### Micaria emertoni

Taxon classificationAnimaliaAraneaeGnaphosidae

Gertsch, 1935

Micaria emertoni Broussard and Horner 2006: 254; Jackman 1997: 163; Platnick and Shadab 1988: 56, mf, desc. (figs 142–145); Richman et al. 2011a: 48

####### Distribution.

Brewster, Presidio

####### Locality.

Chihuahuan desert, Dalquest Research Site

####### Method.

pitfall trap [f]

####### Type.

Massachusetts

####### Etymology.

Person (arachnologist)

####### Collection.

MSU

###### Micaria gertschi

Taxon classificationAnimaliaAraneaeGnaphosidae

Barrows & Ivie, 1942

Micaria gertschi Irungu 2007: 30; Jackman 1997: 163; Knutson et al. 2010: 515; Platnick and Dondale 1992: 52, mf, desc. (figs 64–67); Platnick and Shadab 1988: 12, mf, desc. (figs 14–17)

####### Distribution.

Colorado, Erath, Hidalgo, Howard, Rusk, Scurry

####### Locality.

Attwater Prairie Chicken National Wildlife Refuge, Lake Thomas

####### Time of activity.

Male (May, October); female (June, September)

####### Habitat.

(crops: cabbage, peanuts); (soil/woodland: saltcedar, sandy area)

####### Method.

pitfall trap [mf] (in sand [f])

####### Type.

Ohio, Columbus

####### Etymology.

Person (honor arachnologist)

####### Collection.

NMSU, TAMU

###### Micaria imperiosa

Taxon classificationAnimaliaAraneaeGnaphosidae

Gertsch, 1935

Micaria imperiosa Bonnet 1957: 2841; Broussard and Horner 2006: 254; Gertsch 1935b: 16, m, desc. (fig. 37); Jackman 1997: 163; Platnick and Shadab 1988: 43, mf, desc. (figs 106–109); Richman et al. 2011a: 48; Roewer 1955: 630; Vogel 1970b: 6

####### Distribution.

Borden, Culberson, Jeff Davis, Presidio, Terrell

####### Locality.

Chihuahuan desert, Dalquest Research Site

####### Time of activity.

Male (September); female (September – October)

####### Method.

pitfall trap [mf]

####### Type.

Texas (male, Terrell Co., 5 miles E Dryden, summer 1934, S. Mulaik, holotype, AMNH)

####### Etymology.

Latin, authority

####### Collection.

MSU

###### Micaria langtry

Taxon classificationAnimaliaAraneaeGnaphosidae

Platnick & Shadab, 1988

Micaria langtry Broussard and Horner 2006: 254; Jackman 1997: 163; Platnick and Shadab 1988: 45, m, desc. (figs 46–47); Richman et al. 2011a: 48

####### Distribution.

Brewster, Presidio, Val Verde

####### Locality.

Big Bend National Park, Chihuahuan desert, Dalquest Research Site

####### Time of activity.

Male (March, June)

####### Method.

pitfall trap [m]; yellow pan trap [m]

####### Type.

Texas (male, Val Verde Co., Langtry, June 3, 1941, S. and D. Mulaik, holotype, AMNH)

[female known but not described, deposited at TAMU]

####### Etymology.

locality (The specific name is a noun in apposition taken from the type locality, Platnick and Shadab 1988).

####### Collection.

MSU, TAMU

###### Micaria longipes

Taxon classificationAnimaliaAraneaeGnaphosidae

Emerton, 1890

Micaria longipes Breene et al. 1993c: 16, 47, 86, mf (figs 101A-C); Broussard and Horner 2006: 254; Jackman 1997: 116, 163; Platnick and Shadab 1988: 49, mf, desc. (figs 122–125); Richman et al. 2011a: 48; Trevino 2014: 12
Micaria sp.; Agnew et al. 1985: 4 [part]; Dean and Sterling 1987: 6 [part]; Young and Edwards 1990: 17 [part]

####### Distribution.

Widespread; Borden, Brewster, Colorado, Comanche, Coryell, Culberson, Denton, Erath, Frio, Hidalgo, Jeff Davis, Lynn, Presidio, Reeves, San Patricio, Taylor, Tom Green, Travis, Webb

####### Locality.

Attwater Prairie Chicken National Wildlife Refuge, Chihuahuan desert, Dalquest Research Site

####### Time of activity.

Male (July – September); female (April, June – July, October)

####### Habitat.

(crops: cotton, peanuts); (soil/woodland: on ground, post oak savanna with pasture, woods)

####### Method.

D-Vac suction [f]; pitfall trap [mf] (in sand in woods [m])

####### Type.

Massachusetts, Medford

####### Etymology.

Latin, cephalothorax twice as long as wide

####### Collection.

MSU, TAMU

###### Micaria mormon

Taxon classificationAnimaliaAraneaeGnaphosidae

Gertsch, 1935

Micaria mormon Jackman 1997: 163; Platnick and Shadab 1988: 17, mf, desc. (figs 26–29)

####### Distribution.

Winkler

####### Time of activity.

Male (June)

####### Habitat.

(grass: perennial broomweed)

####### Type.

Utah, Salt Lake City, City Creek Canyon

####### Etymology.

Latin, religion

###### Micaria nanella

Taxon classificationAnimaliaAraneaeGnaphosidae

Gertsch, 1935

Micaria nanella Agnew et al. 1985: 7; Bonnet 1957: 2843; Calixto et al. 2013: 182; Gertsch 1935b: 19, m, desc. (figs 47–48); Jackman 1997: 116, 163, desc.; Platnick and Shadab 1988: 47, mf, desc. (figs 118–121) [see note below]; Roewer 1955: 631; Trevino 2014: 12; Vogel 1970b: 6

####### Distribution.

Bandera, Burleson, Cameron, Colorado, Comanche, Coryell, DeWitt, Erath, Hidalgo, Jim Wells, Kenedy, Kerr, Llano, Nueces, San Patricio, Terrell, Webb

####### Locality.

Attwater Prairie Chicken National Wildlife Refuge, Bill Haney Pecan Orchard, Green Island Bird Refuge, Kenedy Ranch, Raven Ranch, Welder Wildlife Refuge

####### Time of activity.

Male (February – September, November – December); female (March – October)

####### Habitat.

(grass: pasture); (orchard: pecan); (soil/woodland: juniper, post oak savanna with pasture, sandy area, savanna, tree bark)

####### Method.

pitfall trap [mf] (in sand [f], under oak); yellow pan trap [m]

####### Type.

Texas (male, Terrell Co., Sanderson, July 4, 1934, S. Mulaik, holotype, AMNH)

####### Etymology.

Greek, dwarfish

####### Collection.

TAMU

####### Note.

Webb Co.: 54 miles S Laredo is in Nuevo Leon, Mexico based on the map in Platnick and Shadab (1988).

###### Micaria nye

Taxon classificationAnimaliaAraneaeGnaphosidae

Platnick & Shadab, 1988

Micaria nye Broussard and Horner 2006: 254; Jackman 1997: 164; Platnick and Shadab 1988: 42, mf, desc. (figs 102–105); Richman et al. 2011a: 48; Trevino 2014: 12

####### Distribution.

Brewster, Burleson, Coryell, Culberson, Erath, Presidio, San Patricio, Starr, Webb

####### Locality.

Big Bend National Park, Chihuahuan desert, Dalquest Research Site, Guadalupe Mountains

####### Time of activity.

Male (March 29 – April 5, April – July, September); female (April – June, September – October)

####### Habitat.

(soil/woodland: post oak savanna with pasture)

####### Method.

pitfall trap [mf]

####### Type.

Nevada, Nye Co., Mercury

####### Etymology.

locality (The specific name is a noun in apposition taken from the type locality, Platnick and Shadab 1988).

####### Collection.

MSU, TAMU

###### Micaria palliditarsa

Taxon classificationAnimaliaAraneaeGnaphosidae

Banks, 1896

Micaria palliditarsa Platnick 2000 [spelling]; Trevino 2014: 12
Micaria palliditarsus Banks, 1896; Jackman 1997: 164; Platnick and Shadab 1988: 38, mf, desc. (figs 90–93)

####### Distribution.

Crockett, Edwards, Webb

####### Time of activity.

Female (May – July)

####### Habitat.

(landscape features: under rock)

####### Type.

California, Los Angeles

####### Etymology.

Latin, pale tarsi

###### Micaria pasadena

Taxon classificationAnimaliaAraneaeGnaphosidae

Platnick & Shadab, 1988

Micaria pasadena Jackman 1997: 164; Platnick and Shadab 1988: 39, mf, desc. (figs 94–97)

####### Distribution.

Hudspeth

####### Type.

California, Pasadena

####### Etymology.

locality (The specific name is a noun in apposition taken from the type locality, Platnick and Shadab 1988).

###### Micaria pulicaria

Taxon classificationAnimaliaAraneaeGnaphosidae

(Sundevall, 1831)

Micaria pulicaria Bradley 2013: 129; Jackman 1997: 164; Platnick and Dondale 1992: 32, mf, desc. (figs 24–27); Platnick and Shadab 1988: 7, mf, desc. (figs 2–5)

####### Distribution.

Howard, Lubbock

####### Type.

Sweden

####### Etymology.

Latin, a flea

####### Collection.

NMSU

###### Micaria punctata

Taxon classificationAnimaliaAraneaeGnaphosidae

Banks, 1896

Micaria punctata Jackman 1997: 164; Platnick and Shadab 1988: 21 [S], mf, desc. (figs 38–41)
Micaria swansoni Gertsch and Mulaik, 1936; Bonnet 1957: 2850; Gertsch and Mulaik 1936b: 21, m, desc. (fig. 29); Roewer 1955: 632; Vogel 1970b: 6

####### Distribution.

Harris, Kerr

####### Time of activity.

Male (August, November); female (August)

####### Habitat.

(grass: pasture); (soil/woodland: juniper, oak)

####### Method.

pitfall trap [mf]

####### Type.

Florida, Punta Gorda

####### Etymology.

Latin, white spots on abdomen

###### Micaria seminola

Taxon classificationAnimaliaAraneaeGnaphosidae

Gertsch, 1942

Micaria seminola Jackman 1997: 164; Platnick and Shadab 1988: 53, mf, desc. (figs 134–137); Trevino 2014: 12

####### Distribution.

San Patricio, Travis, Webb

####### Time of activity.

Male (March – April, July)

####### Type.

Florida, Saint Augustine

####### Etymology.

Indian tribe in Florida

###### Micaria triangulosa

Taxon classificationAnimaliaAraneaeGnaphosidae

Gertsch, 1935

Micaria triangulosa Agnew et al. 1985: 4, 10; Bonnet 1957: 2850; Gertsch 1935b: 20, mf, desc. (figs 44–46); Jackman 1997: 116, 164; Platnick and Shadab 1988: 61, mf, desc. (figs 158–161); Roewer 1955: 632; Trevino 2014: 12; Vogel 1970b: 6; Young and Edwards 1990: 17

####### Distribution.

Western 2/3 Texas; Cameron, Clay, Eastland, Ector, Erath, Hays, Hidalgo, Kleberg, San Patricio, Terrell, Tom Green, Webb

####### Locality.

Green Island Bird Refuge, Laguna Madre

####### Time of activity.

Male (March – May, July, September – October); female (February, July, September – November)

####### Habitat.

(crops: peanuts)

####### Method.

pitfall trap [m]

####### Type.

Texas (male, Hidalgo Co., 10 miles SE Edinburg, October 20, 1934, S. Mulaik, holotype, AMNH)

####### Etymology.

Latin, triangle

####### Collection.

MSU, TAMU

###### Micaria vinnula

Taxon classificationAnimaliaAraneaeGnaphosidae

Gertsch & Davis, 1936

Micaria vinnula Bonnet 1957: 2850; Breene et al. 1993c: 16, 47, 86, mf (figs 102A-B); Gertsch and Davis 1936: 18, mf, desc. (figs 22–24); Jackman 1997: 116, 164; Platnick and Shadab 1988: 23, mf, desc. (figs 42–45); Roewer 1955: 632; Trevino 2014: 12; Vogel 1970b: 6

####### Distribution.

Central and southeast Texas; Bandera, Bexar, Brazos, Burleson, Coleman, Colorado, Coryell, Harris, Houston, Kerr, Victoria, Webb, Williamson

####### Locality.

Attwater Prairie Chicken National Wildlife Refuge, Horne Ranch, Lick Creek Park

####### Time of activity.

Male (February, May – July, December); female (January, March, May – July, October – December)

####### Habitat.

(crops: cotton); (grass: dead grass, pasture)

####### Method.

pitfall trap [mf]

####### Type.

Texas (male, Bexar Co., San Antonio, December 28, 1935, L. I. Davis, holotype, AMNH)

####### Etymology.

Latin, delightful

####### Collection.

MSU, TAMU

##### Genus *Nodocion* Chamberlin, 1922

###### Nodocion eclecticus

Taxon classificationAnimaliaAraneaeGnaphosidae

Chamberlin, 1924

Nodocion eclecticus Jackman 1997: 164; Platnick and Dondale 1992: 256, mf, desc. (figs 399–403); Platnick and Shadab 1980a: 10, mf, desc. (figs 13–16, 31)

####### Distribution.

Knox, Medina, Sutton, Taylor, Wichita, Zavala

####### Time of activity.

Male (January); female (January – February, April, August)

####### Habitat.

(landscape features: under [rock, rock pile]); (orchard: pecan); (soil/woodland: under bark)

####### Method.

irrigation tubing [mf]

####### Type.

Mexico, Sonora, Guaymus

####### Etymology.

Greek, choosing

####### Collection.

MSU, TAMU

###### Nodocion floridanus

Taxon classificationAnimaliaAraneaeGnaphosidae

(Banks, 1896)

Nodocion floridanus Bowen et al. 2004: 189; Breene et al. 1993c: 16, 47, 89, mf (figs 112A-B); Calixto et al. 2013: 182; Dean et al. 1982: 255; Jackman 1997: 164; Li 1990: 137, 142, 144; Lombardini et al. 2005: 1378; Platnick and Dondale 1992: 259, mf, desc. (figs 22–23, 405–409); Platnick and Shadab 1980a: 14 [S], mf, desc. (figs 21–26, 33); Trevino 2014: 12; Tugmon et al. 1990: 44; Young and Edwards 1990: 17; Zolnerowich and Horner 1985: 82
Liodrassus deceptus Gertsch and Mulaik, 1936; Bonnet 1957: 2545; Gertsch and Mulaik 1936a: 12, mf, desc. (figs 22–24); Roewer 1955: 424; Vogel 1970b: 10

####### Distribution.

Eastern 2/3 Texas; Baylor, Burleson, Cameron, Comanche, Grayson, Hidalgo, Jim Wells, Kenedy, Kerr, Robertson, San Patricio, San Saba, Travis, Walker, Webb, Wichita

####### Locality.

Bentsen-Rio Grande Valley State Park, Bill Haney Pecan Orchard, Buddy Adams Pecan Orchard, Ellis Prison Unit, Holmes Pecan Orchard, Lake Corpus Christi State Park, Lake Wichita, Storey Pecan Orchard

####### Time of activity.

Male (April – August, October – December); female (January, April – October)

####### Habitat.

(crops: cotton); (nest/prey: mud dauber nest [m]); (orchard: pecan); (soil/woodland: post oak savanna with pasture, tamarisk bower, under bark)

####### Method.

Boll weevil pheromone trap [f]; cardboard band [mf]; pitfall trap [f]

####### Type.

Florida, Punta Gorda

####### Etymology.

locality (state)

####### Collection.

DMNS, MSU, TAMU

###### Nodocion rufithoracicus

Taxon classificationAnimaliaAraneaeGnaphosidae

Worley, 1928

Nodocion rufithoracicus Agnew et al. 1985: 4; Jackman 1997: 164; Trevino 2014: 12; Young and Edwards 1990: 17; Zolnerowich and Horner 1985: 83 [Platnick and Shadab 1980a: 6, mf, desc. (figs 5–8)]

####### Distribution.

Brown, Erath, Jeff Davis, Webb, Wichita

####### Time of activity.

Male (March, May, August – September); female (April, June)

####### Habitat.

(crops: peanuts); (landscape features: under rock); (soil/woodland: leaf litter, mixed hardwood leaf litter, on ground)

####### Method.

Berlese funnel [m]

####### Type.

Nebraska, Mitchell

####### Etymology.

Latin, red on thorax

####### Collection.

MSU, TAMU

###### Nodocion utus

Taxon classificationAnimaliaAraneaeGnaphosidae

(Chamberlin, 1936)

Nodocion utus Cokendolpher et al. 2008: 9, 67 [Platnick and Shadab 1980a: 9, mf, desc. (figs 9–12)]

####### Distribution.

Brewster, Carson

####### Locality.

Pantex Plant

####### Habitat.

(grass: grassland)

####### Method.

pitfall trap

####### Type.

Utah, Richfield

####### Etymology.

locality (state)

####### Collection.

MSU, WTAM

##### Genus *Scopoides* Platnick, 1989

###### Scopoides cambridgei

Taxon classificationAnimaliaAraneaeGnaphosidae

(Gertsch & Davis, 1940)

Scopoides cambridgei Broussard and Horner 2006: 254; Jackman 1997: 164; Platnick 1989: 482 [new generic name]; Richman et al. 2011a: 48
Scopodes cambridgei (Gertsch and Davis, 1940); Platnick and Shadab 1976b: 23, mf, desc. (figs 58–63)

####### Distribution.

Brewster, Hudspeth, Presidio, Terrell, Val Verde

####### Locality.

Big Bend National Park, Big Bend Ranch State Park, Chihuahuan desert, Dalquest Research Site

####### Time of activity.

Male (March, May, August, October); female (March, May, August)

####### Habitat.

(landscape features: under [rock, rock near parking lot]); (soil/woodland: forest litter)

####### Method.

pitfall trap [mf]; yellow pan trap [m]

####### Type.

Mexico, Durango, 1 mile W Lerdo

####### Etymology.

Person (arachnologist)

####### Collection.

MSU, NMSU, TAMU

##### Genus *Scotophaeus* Simon, 1893

###### Scotophaeus blackwalli

Taxon classificationAnimaliaAraneaeGnaphosidae

(Thorell, 1871)

Scotophaeus blackwalli Jackman 1997: 164; Reddell and Cokendolpher 2004: 85 [Platnick and Shadab 1977: 41 [T], mf, desc. (figs 123–129)]
Herpyllus blackwalli (Thorell, 1871); Eads et al. 1957: 238; Reddell 1964: 11; Reddell 1961: 13; Reddell 1965: 171; Reddell and Russell 1961: 13; Vogel 1970b: 10

####### Distribution.

Comal, Uvalde

####### Caves.

**Comal** (Bracken Bat Cave); **Uvalde** (Frio Bat Cave)

####### Habitat.

(landscape features: cave)

####### Type.

England

####### Etymology.

Person (arachnologist in England)

####### Collection.

TMM

####### Note.

a record from Frio Bat Cave in Uvalde Co. is unconfirmed [Reddell 1965: 171].

##### Genus *Sergiolus* Simon, 1891

###### Sergiolus angustus

Taxon classificationAnimaliaAraneaeGnaphosidae

(Banks, 1904)

Sergiolus angustus Jackman 1997: 164; Platnick and Shadab 1981b: 37, mf, desc. (figs 99–103); Zolnerowich and Horner 1985: 83

####### Distribution.

North-central Texas; Kleberg, Wichita

####### Time of activity.

Female (March)

####### Habitat.

(landscape features: rocky hillside, under rock); (plants: *Opuntia* sp.)

####### Type.

California, San Pedro

####### Etymology.

Latin, narrow

####### Collection.

MSU

###### Sergiolus bicolor

Taxon classificationAnimaliaAraneaeGnaphosidae

Banks, 1900

Sergiolus bicolor Jackman 1997: 164; Platnick and Dondale 1992: 251, mf, desc. (figs 393–398); Platnick and Shadab 1981b: 26 [S], mf, desc. (figs 3–4, 66–71); Trevino 2014: 12; Zolnerowich and Horner 1985: 83
Sergiolus bellior Chamberlin, 1936; Chamberlin 1936b: 4, f, desc. (fig. 17); Roewer 1955: 438
Sergiolus bellion Chamberlin, 1936; Vogel 1970b: 10

####### Distribution.

Goliad, Hidalgo, Kaufman, Kenedy, Tyler, Walker, Webb, Wichita

####### Locality.

Kirby State Forest

####### Time of activity.

Male (April – June, August); female (August – September)

####### Habitat.

(landscape features: under [rock, stone]); (plants: miscellaneous vegetation); (soil/woodland: woods); (structures: house, indoors)

####### Method.

pitfall trap [f]; sweeping [m]

####### Type.

Louisiana, Covington

####### Etymology.

Latin, cephalothorax and abdomen two colors

####### Collection.

MSU, TAMU

###### Sergiolus capulatus

Taxon classificationAnimaliaAraneaeGnaphosidae

(Walckenaer, 1837)

Sergiolus capulatus Jackman 1997: 164; Platnick and Dondale 1992: 243, mf, desc. (figs 369–374); Platnick and Shadab 1981b: 10 [S], mf, desc. (figs 1, 2, 12–17); Yantis 2005: 202
Sergiolus variegatus (Hentz, 1847); Bonnet 1958: 4033; Jones 1936: 69; Vogel 1970b: 10

####### Distribution.

Archer, Brazos, Colorado, Dallas, Denton, Fort Bend, Madison, Sabine, Tyler, Wichita

####### Locality.

Attwater Prairie Chicken National Wildlife Refuge, Brazos Bend State Park, Kirby State Forest, Lick Creek Park

####### Time of activity.

Male (April – May, May 19-June 7); female (April 27-May 18)

####### Habitat.

(soil/woodland: beech-magnolia forest, buckeye-sycamore forest, old field, post oak woods [%: 96])

####### Method.

5 gallon bucket trap [m]; flight intercept trap [f]; flight intercept trap on ground [m]; malaise trap [m]; pitfall trap [m]; sweeping [m]

####### Type.

Georgia

####### Etymology.

Latin, handle

####### Collection.

MSU, TAMU

###### Sergiolus cyaneiventris

Taxon classificationAnimaliaAraneaeGnaphosidae

Simon, 1893

Sergiolus cyaneiventris Jackman 1997: 164; Platnick and Shadab 1981b: 24, mf, desc. (figs 60–65)

####### Distribution.

Galveston, Hays, San Patricio, Tyler, Walker, Waller

####### Locality.

Kirby State Forest

####### Time of activity.

Male (April 27-May 18, May 19-June 7, June)

####### Habitat.

(soil/woodland: *Juniperus* unmanaged plot)

####### Method.

Flight intercept trap on ground [m]

####### Type.

Florida

####### Etymology.

Latin, color on venter

###### Sergiolus lowelli

Taxon classificationAnimaliaAraneaeGnaphosidae

Chamberlin & Woodbury, 1929

Sergiolus lowelli Agnew et al. 1985: 4; Breene et al. 1993a: 169; Jackman 1997: 164; Platnick and Shadab 1981b: 15 [S], mf, desc. (figs 30–35); Young and Edwards 1990: 17; Zolnerowich and Horner 1985: 83
Sergiolus segregatus Chamberlin, 1936; Chamberlin 1936b: 5, mf, desc. (figs 11–12); Roewer 1955: 439; Vogel 1970b: 10

####### Distribution.

Archer, Baylor, Caldwell, Cameron, Erath, Gonzales, Hidalgo, Kenedy, Presidio, San Patricio, Travis, Wichita

####### Locality.

Lake Wichita, Padre Island National Seashore, Shipp Farm

####### Time of activity.

Male (January, March – September); female (April, June – October, December)

####### Habitat.

(crops: peanuts); (grass: under board in damp pasture, grass); (nest/prey: bird nest); (objects: on tarpaulin, under tarpaulin); (orchard: citrus); (soil/woodland: ground, in hackberry, leaf litter, tamarisk bower); (structures: in house, in building, indoors)

####### Method.

pitfall trap [m]; sweeping [m]

####### Type.

Utah, St. George

####### Etymology.

Person (collector, Lowell A. Woodbury)

####### Collection.

DMNS, MSU, TAMU

###### Sergiolus minutus

Taxon classificationAnimaliaAraneaeGnaphosidae

(Banks, 1898)

Sergiolus minutus Chamberlin 1922: 153; Jackman 1997: 164; Platnick and Shadab 1981b: 20 [T], mf, desc. (figs 48–53); Vogel 1970b: 10
Poecilochroa minuta Banks, 1898; Banks 1898a: 185, m, desc.; Banks 1910: 8; Comstock 1912: 316, desc.; Petrunkevitch 1911: 146; Roewer 1955: 432

####### Distribution.

Brazos, Shelby

####### Type.

Texas (male, Brazos Co., no date, no collector, holotype, MCZ)

####### Etymology.

Latin, size

###### Sergiolus montanus

Taxon classificationAnimaliaAraneaeGnaphosidae

(Emerton, 1890)

Sergiolus montanus Jackman 1997: 164; Platnick and Shadab 1981b: 28, mf, desc. (figs 72–76)

####### Distribution.

Grayson, Lubbock

####### Type.

New Hampshire, Mt. Washington

####### Etymology.

Latin, montain

####### Collection.

JCC

###### Sergiolus ocellatus

Taxon classificationAnimaliaAraneaeGnaphosidae

(Walckenaer, 1837)

Sergiolus ocellatus Breene et al. 1993c: 16, 47, 89, mf (figs 111A-C); Dean et al. 1982: 255; Jackman 1997: 164; Platnick and Dondale 1992: 245, mf, desc. (figs 375–380); Platnick and Shadab 1981b: 11 [S, T], mf, desc. (figs 18–23); Trevino 2014: 12; Young and Edwards 1990: 17
Poecilochroa ocellata (Walckenaer, 1837); Kaston 1978: 208; Rapp 1984: 6
Sergiolus decipiens Chamberlin, 1922; Chamberlin 1922: 151, m, desc.; Chamberlin 1936a: 10 [Texas records]; Roewer 1955: 438

####### Distribution.

Anderson, Angelina, Burleson, Galveston, Harrison, Travis, Walker, Webb

####### Locality.

Angelina National Forest, Ellis Prison Unit, Galveston Island State Park, Somerville Lake

####### Time of activity.

Male (May, July – September); female (August)

####### Habitat.

(crops: cotton); (littoral: salt marsh); (soil/woodland: loblolly pine unmanaged)

####### Method.

pitfall trap [m]

####### Type.

Georgia

####### Etymology.

Latin, little eyes, marked with spots

####### Collection.

DMNS, TAMU

###### Sergiolus stella

Taxon classificationAnimaliaAraneaeGnaphosidae

Chamberlin, 1922

Sergiolus stella Bowen et al. 2004: 189; Broussard and Horner 2006: 254; Chamberlin 1922: 152, f, desc.; Chamberlin 1936a: 8; Jackman 1997: 164; Platnick and Shadab 1981b: 32, mf, desc. (figs 83–87); Richman et al. 2011a: 48; Roewer 1955: 439; Vogel 1970b: 10; Zolnerowich and Horner 1985: 83

####### Distribution.

Brewster, Cameron, Denton, Presidio, Travis, Wichita

####### Locality.

Chihuahuan desert, Dalquest Research Site

####### Time of activity.

Female (March, May, July, September)

####### Habitat.

(landscape features: stony hillside, under rock); (structures: house)

####### Method.

pitfall trap [mf]

####### Type.

Texas (female, Travis Co., Austin, no date, no collector, holotype)

####### Etymology.

Latin, column

####### Collection.

MSU

###### Sergiolus tennesseensis

Taxon classificationAnimaliaAraneaeGnaphosidae

Chamberlin, 1922

Sergiolus tennesseensis Agnew et al. 1985: 8; Jackman 1997: 164 [Platnick and Shadab 1981b: 34, mf, desc. (figs 88–93)]

####### Distribution.

Erath

####### Time of activity.

Female (August)

####### Habitat.

(soil/woodland: woods)

####### Method.

pitfall trap [f] (in sand in woods [f])

####### Type.

Tennessee, Glenraven

####### Etymology.

locality (state)

####### Collection.

TAMU

##### Genus *Sosticus* Chamberlin, 1922

###### Sosticus insularis

Taxon classificationAnimaliaAraneaeGnaphosidae

(Banks, 1895)

Sosticus insularis Bradley 2013: 131; Jackman 1997: 164; Platnick and Dondale 1992: 199, mf, desc. (figs 297–300); Platnick and Shadab 1976b: 11, mf, desc. (figs 19–26); Yantis 2005: 66, 198, 202

####### Distribution.

Brown, Dallas, Houston, Leon, Trinity

####### Time of activity.

Male (April – May); female (April – May)

####### Habitat.

(soil/woodland: pine woods [%: 66, 69, 82, 84, 88], post oak woods [%: 56, 92], under bark)

####### Method.

5 gallon bucket trap [mf]

####### Type.

New York, Long Island, Sea Cliff

####### Etymology.

Latin, from island

####### Collection.

MSU, TAMU

##### Genus *Synaphosus* Platnick & Shadab, 1980

###### Synaphosus paludis

Taxon classificationAnimaliaAraneaeGnaphosidae

(Chamberlin & Gertsch, 1940)

Synaphosus paludis Bowen et al. 2004: 189; Breene et al. 1993c: 16, 47, 89, mf (figs 113A-B); Cokendolpher et al. 2008: 9, 22; Dean et al. 1982: 255; Jackman 1997: 164; Platnick and Shadab 1980a: 24, mf, desc. (figs 44–48); Yantis 2005: 198; Young and Edwards 1990: 17

####### Distribution.

Angelina, Brazos, Burleson, Carson, Gonzales, Hidalgo, Houston, Kerr, San Patricio, Walker, Wichita

####### Locality.

Angelina National Forest, Ellis Prison Unit, Lick Creek Park, Palmetto State Park, Pantex Lake, Pantex Plant

####### Time of activity.

Male (March, May – August, August 28 – September 4); female (May 30 – June 8, June – July, July 27 – August 3, August 31 – September 7, September – October)

####### Habitat.

(crops: cotton, sorghum); (grass: grass); (soil/woodland: carrion in palm thicket, loblolly pine unmanaged, pine woods [%: 95], post oak savanna with pasture)

####### Method.

5 gallon bucket trap [m]; pitfall trap [mf]

####### Type.

Georgia, Okefenokee Swamp

####### Etymology.

Latin, a marsh

####### Collection.

MSU, TAMU, WTAM

###### Synaphosus syntheticus

Taxon classificationAnimaliaAraneaeGnaphosidae

(Chamberlin, 1924)

Synaphosus syntheticus Jackman 1997: 164; Platnick and Shadab 1980a: 23, mf, desc. (figs 40–43)

####### Distribution.

Brewster, Dallas

####### Locality.

Big Bend National Park

####### Time of activity.

Male (May); female (May, August)

####### Habitat.

(soil/woodland: cottonwood, mesquite litter, saltcedar); (structures: mule barn, rock and adobe houses)

####### Type.

Mexico, Baja California, Isla Raza

####### Etymology.

Latin, synthetic

##### Genus *Talanites* Simon, 1893

###### Talanites captiosus

Taxon classificationAnimaliaAraneaeGnaphosidae

(Gertsch & Davis, 1936)

Talanites captiosus Breene et al. 1993c: 16, 47, 88, mf (figs 110A-B); Jackman 1997: 164; Platnick and Ovtsharenko 1991: 116 [T]
Drassyllochemmis captiosus Gertsch and Davis, 1936; Bonnet 1956: 1601; Comstock 1940: 591; Gertsch and Davis 1936: 17, m, desc. (fig. 34); Roewer 1955: 620; Vogel 1970b: 6
Rachodrassus captiosus (Gertsch and Davis, 1936); Platnick and Shadab 1976b: 8 [T], mf, desc. (figs 15–18); Zolnerowich and Horner 1985: 83

####### Distribution.

Angelina, Burleson, Cameron, Coleman, Coryell, Houston, San Patricio, Wichita, Williamson

####### Locality.

Horne Ranch, La Gringa Resaca, Stiles Farm Foundation

####### Time of activity.

Male (May – September); female (July – September)

####### Habitat.

(crops: cotton); (objects: under [railroad tie, wood]); (soil/woodland: loblolly pine unmanaged, post oak savanna with pasture)

####### Method.

pitfall trap [m]

####### Type.

Texas (male, Cameron Co., May 1–2, 1936, L. I. Davis, holotype, AMNH)

####### Etymology.

Latin, deception

####### Collection.

MSU, TAMU

###### Talanites exlineae

Taxon classificationAnimaliaAraneaeGnaphosidae

(Platnick & Shadab, 1976)

Talanites exlineae Bradley, 2013: 132; Henderson 2007: 34, 54–59, 62, 76, 79, 83; Jackman 1997: 164; Platnick and Ovtsharenko 1991: 116 [T]; Yantis 2005: 66, 198, 202
Rachodrassus exlineae Platnick & Shadab, 1976; Agnew et al. 1985: 8; Platnick and Shadab 1976b: 7, mf, desc. (figs 9–14)

####### Distribution.

Angelina, Brazos, Burleson, Caldwell, Coryell, Erath, Gonzalez, Grayson, Grimes, Houston, Kerr, Leon, Madison, Sabine, Smith, Trinity, Tyler, Wichita, Williamson

####### Locality.

Angelina National Forest, Big Slough Wild Area, Big Thicket National Preserve, Kirby State Forest, Lick Creek Park, Raven Ranch, Stiles Farm Foundation, Texas A&M University Rangeland Area, Tyler State Park

####### Time of activity.

Male (April – August); female (March – August, September 27-October 6)

####### Habitat.

(crops: cotton); (littoral: near pond); (soil/woodland: beech-magnolia litter, disturbed habitat, hardwood bottomland, leaf litter, loblolly pine managed, loblolly pine unmanaged, longleaf pine managed, longleaf pine unmanaged, magnolia litter, pine woods [%: 66, 67, 69, 80, 82, 83, 84, 88, 92, 95], post oak savanna with pasture, post oak woods [%: 41, 44, 56, 71, 91, 93, 96], post oak woodland, sandy area, under [juniper, oak], upland woods)

####### Method.

5 gallon bucket trap [mf]; berlese funnel [f]; carrion trap [m]; flight intercept trap [m]; flight intercept trap on ground [m]; pitfall trap [mf] (near pond [m], under juniper [m], under oak [m])

####### Type.

Arkansas, 1.7 mile S Lapile

####### Etymology.

Person (The specific name is a patronym in honor of the late Harriet Exline, who first recognized the species as new, Platnick and Shadab 1976b).

####### Collection.

DMNS, MSU, TAMU

##### Genus *Trachyzelotes* Lohmander, 1944

###### Trachyzelotes lyonneti

Taxon classificationAnimaliaAraneaeGnaphosidae

(Audouin, 1826)

Trachyzelotes lyonneti Bowen et al. 2004: 189; Irungu 2007: 30; Jackman 1997: 164; Platnick and Murphy 1984: 6 [S], mf, desc. (figs 7–10); Trevino 2014: 12; Zolnerowich and Horner 1985: 83
Nodocion agilis Bryant, 1936; Bonnet 1958: 3105; Bryant 1936: 93, m, desc. (fig. 3); Jones 1936: 69; Roewer 1955: 427; Vogel 1970b: 10
Drassyllus agilis (Bryant, 1936); Kaston 1978: 206
Nodocion zelotoides Chamberlin, 1936; Chamberlin 1936b: 6, f, desc. (fig. 20); Vogel 1970b: 10
Nodocion chamberlini Roewer 1951; Roewer 1955: 427

####### Distribution.

Baylor, Cameron, Dallas, Hidalgo, Kleberg, Potter, San Patricio, Travis, Webb, Wichita

####### Locality.

Bentsen-Rio Grande Valley State Park, Big Bend National Park, Green Island Bird Refuge, Welder Wildlife Refuge

####### Time of activity.

Male (March – May, July – September); female (March 30-April 5, April – May, September, December)

####### Habitat.

(crops: cabbage); (grass: grass); (soil/woodland: ground, live oak forest, woods); (structures: house, mule barn)

####### Method.

carrion trap [m]; pitfall trap [mf] (in woods [m]); snake carrion pit [m]

####### Type.

Egypt or Syria

####### Etymology.

Person (honor arachnologist)

####### Collection.

MSU, TAMU

##### Genus *Urozelotes* Mello-Leitão, 1938

###### Urozelotes rusticus

Taxon classificationAnimaliaAraneaeGnaphosidae

(L. Koch, 1872)

Urozelotes rusticus Agnew et al. 1985: 8; Jackman 1997: 164; Platnick and Murphy 1984: 24 [S, T], mf, desc. (figs 55–58); Trevino 2014: 12; Zolnerowich and Horner 1985: 83
Zelotes rusticus (L. Koch, 1872); Brown 1974: 234
Drassyllus liopus Chamberlin, 1922; Chamberlin 1922: 170, m, desc.; Vogel 1970b: 9

####### Distribution.

Blanco, Dallas, Erath, Garza, Hidalgo, Kleberg, Lubbock, Martin, Medina, Nacogdoches, Parker, Travis, Webb, Wichita

####### Caves.

**Blanco** (Davis Blowout Cave); **Medina** (Ney Cave)

####### Time of activity.

Male (May – September); female (May, August)

####### Habitat.

(landscape features: cave); (soil/woodland: debris under banana trees); (structures: house, indoors, on ground near house, on floor in house)

####### Method.

pitfall trap [f]

####### Type.

Italy

####### Etymology.

Latin, rural

####### Collection.

JCC, MSU, TAMU, TMM

##### Genus *Zelotes* Gistel, 1848

**Note.** Trevino (2014: 13) recorded *Zelotes pallidus* (O. P.-Cambridge, 1874) and *Zelotes sula* Lowrie and Gertsch, 1955 from Webb Co. Because of the distance between collecting sites for *Zelotes pallidus* (California) and *Zelotes sula* (Colorado), they are not included in this list. See Platnick and Shadab (1983: 109, 185).

###### Zelotes aiken

Taxon classificationAnimaliaAraneaeGnaphosidae

Platnick & Shadab, 1983

Zelotes aiken Bowen et al. 2004: 190; Cokendolpher et al. 2008: 9, 67; Jackman 1997: 164; Platnick and Shadab 1983: 128, mf, desc. (figs 64–69); Trevino 2014: 12; Zolnerowich and Horner 1985: 83

####### Distribution.

Brazos, Burleson, Carson, Clay, Coryell, Hardeman, Hays, Montague, Walker, Webb, Wichita

####### Locality.

Ellis Prison Unit, Lick Creek Park, Medicine Mounds Ranch, Pantex Plant

####### Time of activity.

Male (March – May, May 30-June 6, July, September); female (April – June, September)

####### Habitat.

(grass: Bermuda grass, grass, grassland, sandy grassland); (landscape features: under rock); (littoral: lake shore); (soil/woodland: *Juniperus* unmanaged plot, next to cotton field, post oak savanna with pasture)

####### Method.

Flight intercept trap on ground [m]; pitfall trap [mf]

####### Type.

South Carolina, Aiken Co., Savannah River Plant

####### Etymology.

locality (The specific name is a noun in apposition taken from the type locality, Platnick and Shadab 1983).

####### Collection.

MSU, TAMU, WTAM

###### Zelotes anglo

Taxon classificationAnimaliaAraneaeGnaphosidae

Gertsch & Riechert, 1976

Zelotes anglo Bowen et al. 2004: 190; Jackman 1997: 164; Platnick and Shadab 1983: 126, mf, desc. (figs 58–63); Zolnerowich and Horner 1985: 83

####### Distribution.

Archer, Burleson, Coryell, Jeff Davis, Presidio, Terrell, Travis, Wichita, Wilbarger

####### Locality.

Big Bend Ranch State Park

####### Time of activity.

Male (April, June 28-July 2, September – October); female (June, September – October)

####### Habitat.

(grass: pasture); (landscape features: under rock); (soil/woodland: post oak savanna with pasture)

####### Method.

pitfall trap [mf]; swine feces pitfall trap [m]

####### Type.

New Mexico, Carizozo

####### Etymology.

Latin, people of European descent in American southwest

####### Collection.

DMNS, MSU, TAMU

###### Zelotes duplex

Taxon classificationAnimaliaAraneaeGnaphosidae

Chamberlin, 1922

Zelotes duplex Henderson 2007: 34, 55–61, 66, 76, 79, 83; Jackman 1997: 164; Platnick and Dondale 1992: 91, mf, desc. (figs 136–139); Platnick and Shadab 1983: 168, mf, desc. (figs 203–208, 269); Trevino 2014: 12; Yantis 2005: 66, 199, 202

####### Distribution.

Angelina, Brazos, Harris, Hidalgo, Houston, Leon, Madison, Sabine, Trinity, Tyler, Webb

####### Locality.

Angelina National Forest, Kirby State Forest, Lick Creek Park

####### Time of activity.

Male (April – July); female (April 27-May 18, May – July, August 15-September 17, September)

####### Habitat.

(littoral: sedge meadow); (soil/woodland: beech-magnolia forest, disturbed habitat, loblolly pine managed, loblolly pine unmanaged, longleaf pine unmanaged, pine woods [%: 79, 83, 84, 88, 92], post oak woodland, post oak woods [%: 56, 77], sandy area, upland woods)

####### Method.

5 gallon bucket trap [mf]; blue pan trap [m]; flight intercept trap [m]; pitfall trap [mf]

####### Type.

Virginia, Fairfax Co.

####### Etymology.

Latin, double

####### Collection.

TAMU

###### Zelotes gertschi

Taxon classificationAnimaliaAraneaeGnaphosidae

Platnick & Shadab, 1983

Zelotes gertschi Agnew et al. 1985: 4; Bowen et al. 2004: 190; Calixto et al. 2013: 182; Cokendolpher et al. 2008: 9, 22 (photo 17); Irungu 2007: 30; Jackman 1997: 116, 164; Platnick and Shadab 1983: 131, mf, desc. (figs 76–81); Roberts 2001: 50; Trevino 2014: 12; Young and Edwards 1990: 17; Zolnerowich and Horner 1985: 83

####### Distribution.

Western 2/3 Texas; Archer, Bandera, Brown, Carson, Clay, Comanche, Coryell, Cottle, Erath, Hardeman, Hays, Hidalgo, Kerr, Kimble, Kleberg, Knox, Lubbock, Pecos, Potter, Randall, San Patricio, Travis, Val Verde, Webb, Wichita, Williamson

####### Locality.

Bill Haney Pecan Orchard, Buffalo Lake, Canoncita Ranch, Lake Meredith National Recreation Area, Matador Wildlife Management Area, Medicine Mounds Ranch, Palo Duro Canyon State Park, Pantex Lake (edge), Pantex Plant, Raven Ranch, Stiles Farm Foundation, Wildcat Bluff Nature Center

####### Time of activity.

Male (March – August, October – December); female (February – December)

####### Habitat.

(crops: cabbage, cotton, peanuts); (grass: grassland); (landscape features: on rocky ground, under [rock, stone]); (littoral: playa, edge of pond); (objects: under [board, cardboard, railroad tie]); (orchard: pecan); (soil/woodland: post oak savanna with pasture)

####### Method.

pitfall trap [mf] (edge of pond [mf])

####### Type.

Texas (male, San Patricio Co., 8 miles NE Sinton, August 4, 1960, H. E. Laughlin, holotype, AMNH)

####### Etymology.

Person (The specific name is a patronym in honor of Dr. Willis J. Gertsch, who first recognized the species as new, Platnick and Shadab 1983).

####### Collection.

DMNS, MSU, TAMU, WTAM

###### Zelotes hentzi

Taxon classificationAnimaliaAraneaeGnaphosidae

Barrows, 1945

Zelotes hentzi Brown 1974: 234; Jackman 1997: 116, 164; Platnick and Dondale 1992: 84, mf, desc. (figs 120–123); Platnick and Shadab 1983: 112, mf, desc. (figs 19–24); Trevino 2014: 12; Yantis 2005: 66, 199, 202

####### Distribution.

Anderson, Angelina, Brazos, Burleson, Collin, Colorado, Coryell, Grimes, Hardin, Harris, Houston, Hunt, Knox, Leon, Madison, Milam, Nacogdoches, San Jacinto, Trinity, Tyler, Walker, Webb, Wichita

####### Locality.

Angelina National Forest, Attwater Prairie Chicken National Wildlife Refuge, Kirby State Forest, Lick Creek Park, Texas A&M University Rangeland Area

####### Time of activity.

Male (March – June, August 24-September 28, September – November); female (March – September)

####### Habitat.

(grass: short grass); (landscape features: under rock); (soil/woodland: bottomland hardwood, pine woods [%: 69, 79, 80, 82, 84, 86, 97, 100], post oak woods [%: 41, 56, 74, 77, 80, 84, 85, 92, 94, 96], sandy area, post oak savanna with pasture, longleaf pine unmanaged); (structures: on patio)

####### Method.

5 gallon bucket trap [mf]; pitfall trap [mf]

####### Type.

Ohio, Rockbridge

####### Etymology.

Person (honor arachnologist)

####### Collection.

MSU, TAMU

###### Zelotes laccus

Taxon classificationAnimaliaAraneaeGnaphosidae

(Barrows, 1919)

Zelotes laccus [Platnick and Shadab 1983: 173, mf, desc. figs (219–224)]

####### Distribution.

Colorado

####### Locality.

Attwater Prairie Chicken National Wildlife Refuge

####### Time of activity.

Male (May – June); female (May – June)

####### Method.

pitfall trap [mf]

####### Type.

Ohio, Colombus

####### Etymology.

Latin, milk

####### Collection.

TAMU

###### Zelotes laetus

Taxon classificationAnimaliaAraneaeGnaphosidae

(O. P.-Cambridge, 1872)

Zelotes laetus FitzPatrick 2007: 108 [S]
Zelotes reformans Chamberlin, 1924; Jackman 1997: 164; Platnick and Shadab 1983: 182, mf, desc. (figs 253–258); Trevino 2014: 13

####### Distribution.

El Paso, Webb

####### Time of activity.

Male (April); female (March-April, July-September)

####### Type.

Jordan

####### Etymology.

Latin, pleasant

###### Zelotes lasalanus

Taxon classificationAnimaliaAraneaeGnaphosidae

Chamberlin, 1928

Zelotes lasalanus Bowen et al. 2004: 190; Broussard and Horner 2006: 254; Cokendolpher et al. 2008: 9, 67; Jackman 1997: 116, 164; Platnick and Dondale 1992: 86, mf, desc. (figs 124–127); Platnick and Shadab 1983: 114, mf, desc. (figs 25–30); Richman et al. 2011a: 48; Trevino 2014: 12

####### Distribution.

Western 2/3 Texas; Bailey, Brewster, Brown, Cameron, Carson, Clay, Colorado, Coryell, Culberson, Dimmit, Ector, Hays, Jeff Davis, Kenedy, Kerr, Kleberg, Presidio, Reeves, Tarrant, Terrell, Travis, Webb, Wichita

####### Locality.

Attwater Prairie Chicken National Wildlife Refuge, Chaparral Wildlife Management Area, Chihuahuan desert, Dalquest Research Site, Pantex Plant

####### Time of activity.

Male (March – April, September – October); female (May – June, September, September 11-October 10)

####### Habitat.

(grass: grassland); (soil/woodland: ground, *Juniperus* managed plot, paloverde upland area, post oak savanna with pasture)

####### Method.

pitfall trap [mf]

####### Type.

Utah, La Sal Mountains

####### Etymology.

locality (mountains)

####### Collection.

MSU, TAMU, WTAM

###### Zelotes lymnophilus

Taxon classificationAnimaliaAraneaeGnaphosidae

Chamberlin, 1936

Zelotes lymnophilus Jackman 1997: 164; Platnick and Shadab 1983: 172, mf, desc. (figs 210, 215–218); Yantis 2005: 199

####### Distribution.

Anderson, Angelina, Coryell, Kerr

####### Locality.

Angelina National Forest, Raven Ranch

####### Time of activity.

Male (April – June); female (May – June)

####### Habitat.

(soil/woodland: longleaf pine managed, pine woods [%: 83, 100], post oak savanna with pasture)

####### Method.

5 gallon bucket trap [mf]; pitfall trap [mf]

####### Type.

Georgia, Okefenokee Swamp

####### Etymology.

Latin, water or lake-loving

####### Collection.

TAMU

###### Zelotes monachus

Taxon classificationAnimaliaAraneaeGnaphosidae

Chamberlin, 1924

Zelotes monachus Trevino 2014: 13 [Platnick and Shadab 1983: 129, mf, desc. (figs 70–75)]

####### Distribution.

Webb

####### Type.

Mexico, Baja California Norte

####### Etymology.

Greek, solitary

###### Zelotes monodens

Taxon classificationAnimaliaAraneaeGnaphosidae

Chamberlin, 1936

Zelotes monodens Chamberlin 1936b: 9, f, desc. (fig. 36); Jackman 1997: 164; Platnick and Shadab 1983: 179, mf, desc. (figs 243–246); Roewer 1955: 471; Vogel 1970b: 10

####### Distribution.

Hidalgo

####### Time of activity.

Male (April); female (May)

####### Type.

Texas (female, Hidalgo Co., Edinburg, May 2, 1935, S. Mulaik, holotype, AMNH)

####### Etymology.

Greek, one, L, tooth

###### Zelotes pseustes

Taxon classificationAnimaliaAraneaeGnaphosidae

Chamberlin, 1922

Zelotes pseustes Agnew et al. 1985: 4; Bonnet 1959: 4944; Bowen et al. 2004: 190; Calixto et al. 2013: 182; Chamberlin 1922: 164, m, desc.; Henderson 2007: 53, 76, 79, 83; Jackman 1997: 116, 164; Platnick and Shadab 1983: 119, mf, desc. (figs 37–42); Reddell and Cokendolpher 2004: 85; Roewer 1955: 471; Trevino 2014: 13; Vogel 1970b: 10; Yantis 2005: 199; Young and Edwards 1990: 17; Zolnerowich and Horner 1985: 83
Zelotes subterraneus (C. Koch, 1833); Chamberlin 1936a: 18; Vogel 1970b: 10 [Texas records]

####### Distribution.

North-central, central, and south Texas; Archer, Baylor, Bexar, Brazos, Brown, Burleson, Comanche, Coryell, Erath, Gonzales, Haskell, Hidalgo, Jeff Davis, Kenedy, Kerr, Kleberg, La Salle, Leon, Nueces, Palo Pinto, Parker, San Patricio, San Saba, Sutton, Travis, Webb, Wichita

####### Locality.

Bentsen-Rio Grande Valley State Park, Bill Haney Pecan Orchard, Chaparral Wildlife Management Area, Kenedy Ranch, Lick Creek Park, Raven Ranch, Santa Ana National Wildlife Refuge, Welder Wildlife Refuge

####### Caves.

**Bexar** (Vera Cruz Shaft)

####### Time of activity.

Male (January – December); female (January, March – June, June 28-July 2, August, October, October 30-November 6, December)

####### Habitat.

(crops: peanuts); (grass: grass, short grass); (landscape features: cave, under rock); (littoral: edge of pond, sand dune area); (objects: under board); (orchard: pecan); (plants: under dead yucca, *Opuntia* sp.); (soil/woodland: acacia area, anacua groves, dead leaves, forest, hackberry matte, hackberry woodland, leaf litter, litter, live oak forest, live oak woodland, mesquite woods, post oak savanna with pasture, post oak woods [%: 44, 71], sandy area, sandy brushland, upland woods, woods); (structures: garage)

####### Method.

5 gallon bucket trap [mf]; carrion trap [m]; flight intercept trap [f]; pitfall trap [mf] (edge of pond [m], in dead leaves [m], in leaves [mf], in sand [m], in woods [m]); swine feces pitfall trap [f]

####### Type.

Texas (male, Travis Co., Austin, no date, no collector, holotype, MCZ)

####### Etymology.

Greek, false

####### Collection.

DMNS, MSU, TAMU, TMM

###### Zelotes tuobus

Taxon classificationAnimaliaAraneaeGnaphosidae

Chamberlin, 1919

Zelotes tuobus Bowen et al. 2004: 190; Calixto et al. 2013: 182; Jackman 1997: 164; Platnick and Shadab 1983: 124, mf, desc. (figs 52–57); Trevino 2014: 13; Zolnerowich and Horner 1985: 83

####### Distribution.

Coryell, Robertson, Webb, Wichita

####### Locality.

Holmes Pecan Orchard

####### Time of activity.

Male (April, September – October, October 27 – November 2); female (August – September, October 27 – November 2, December)

####### Habitat.

(landscape features: under rock); (orchard: pecan); (soil/woodland: post oak savanna with pasture)

####### Method.

pitfall trap [mf]

####### Type.

Utah, Fillmore

####### Etymology.

Latin, a tube

####### Collection.

MSU, TAMU

#### Family Hahniidae Bertkau, 1878

##### Genus *Hahnia* C. L. Koch, 1841

###### Hahnia arizonica

Taxon classificationAnimaliaAraneaeHahniidae

Chamberlin & Ivie, 1942

Hahnia arizonica Jackman 1997: 164; Opell and Beatty 1976: 424, mf, desc. (figs 84–88)
Hahnia sanjuanensis Exline, 1938; Opell 1974: 57 [Texas record]

####### Distribution.

Brewster

####### Locality.

Big Bend National Park, Chisos Basin

####### Time of activity.

Female (May)

####### Type.

Arizona, Oak Creek Canyon, 20 miles S Flagstaff

####### Etymology.

locality (state)

###### Hahnia cinerea

Taxon classificationAnimaliaAraneaeHahniidae

Emerton, 1890

Hahnia cinerea Agnew et al. 1985: 7; Bradley 2013: 134; Calixto et al. 2013: 182; Cokendolpher et al. 2008: 9, 68; Jackman 1997: 164; Opell and Beatty 1976: 423, mf, desc. (figs 11–12, 78–83)

####### Distribution.

East Texas; Archer, Brazos, Burleson, Carson, Colorado, Coryell, Erath, Robertson, Travis, Wichita

####### Locality.

5-Eagle Ranch, Holmes Pecan Orchard, NK Ranch, Texas A&M University Rangeland Area

####### Time of activity.

Male (January – April, November); female (January – August, October – November)

####### Habitat.

(grass: grass, grassland); (orchard: pecan); (soil/woodland: dead leaves, forest litter, leaf litter, post oak savanna with pasture)

####### Method.

Berlese funnel [mf]; pitfall trap [mf]

####### Type.

Massachusetts, Swampscott

####### Etymology.

Latin, gray

####### Collection.

MSU, TAMU, TTU

###### Hahnia flaviceps

Taxon classificationAnimaliaAraneaeHahniidae

Emerton, 1913

Hahnia flaviceps Agnew et al. 1985: 7; Cokendolpher and Reddell 2001b: 46; Henderson 2007: 70, 76, 79, 83; Jackman 1997: 164; Opell 1974: 40; Opell and Beatty 1976: 430, mf, desc. (figs 110–113); Reddell and Cokendolpher 2004: 85

####### Distribution.

Archer, Bell, Bexar, Brazos, Burleson, Caldwell, Colorado, Coryell, Dallas, Erath, Gonzales, Hays, Houston, Matagorda, Travis, Tyler, Wichita

####### Locality.

Big Slough Wild Area, Big Thicket National Preserve, Caine’s Ranch, Fort Hood, Lick Creek Park, Palmetto State Park, Texas A&M University Rangeland Area, White Rock Lake

####### Caves.

**Bell** ([all Fort Hood] Big Crevice, Jagged Walls Cave, Price Pit Cave); **Bexar** (Stone Oak Parkway Pit); **Coryell** (Porter Cave [Fort Hood])

####### Time of activity.

Male (January – April, November – December); female (January – July, October – December)

####### Habitat.

(landscape features: cave); (soil/woodland: bottomland forest litter, forest litter, hardwood litter, *Juniperus* managed plot, leaf litter, old field, post oak savanna with pasture, under oak, upland woods)

####### Method.

Berlese funnel [mf]; pitfall trap [mf] (under oak [f])

####### Type.

New Jersey, Farmingdale

####### Etymology.

Latin, yellow head

####### Collection.

MSU, TAMU, TMM

##### Genus *Neoantistea* Gertsch, 1934

###### Neoantistea agilis

Taxon classificationAnimaliaAraneaeHahniidae

(Keyserling, 1887)

Neoantistea agilis Gertsch 1934c: 19, mf, desc. (figs 29, 41); Henderson 2007: 32, 62, 67–72, 74, 76, 79, 83; Jackman 1997: 95, 164; Opell 1974: 74; Opell and Beatty 1976: 404, mf, desc. (figs 1–3, 14–21); Rapp 1984: 6; Vogel 1970b: 10

####### Distribution.

Southeast Texas; Brazos, Burleson, Coryell, Galveston, Walker, Waller, Wichita

####### Locality.

5-Eagle Ranch, Lick Creek Park

####### Time of activity.

Male (February 15-March 15, March – April, June, August, August 15-September 17, September 17-October 20, October, October 20-November 15, November, 21, December 21-January 15); female (March, March 30-April 6, October)

####### Habitat.

(littoral: near water); (soil/woodland: disturbed habitat, post oak savanna, post oak savanna with pasture, post oak woodland, sandy area, upland woods)

####### Method.

pitfall trap [mf]

####### Type.

South Dakota, Fort Stevenson

####### Etymology.

Latin, agile

####### Collection.

MSU, TAMU

###### Neoantistea alachua

Taxon classificationAnimaliaAraneaeHahniidae

Gertsch, 1946

Neoantistea alachua [Opell and Beatty 1976: 413, mf, desc. (figs 58–61)]

####### Distribution.

Nacogdoches

####### Time of activity.

Female (October)

####### Habitat.

(web: in web on ground)

####### Type.

Florida, Alachua Co., 5 miles W Gainesville

####### Etymology.

locality (county)

####### Collection.

TAMU

###### Neoantistea mulaiki

Taxon classificationAnimaliaAraneaeHahniidae

Gertsch, 1946

Neoantistea mulaiki Agnew et al. 1985: 4, 9; Breene et al. 1993c: 16, 47, 89, mf (figs 115A-B); Calixto et al. 2013: 182; Cokendolpher et al. 2008: 9, 22; Dean et al. 1982: 254; Gertsch 1946b: 34, mf, desc. (pl. 1, figs 5–6); Irungu 2007: 30; Jackman 1997: 164; Opell 1974: 112 [see note below]; Opell and Beatty 1976: 409, mf, desc. (figs 38–41); Reddell and Cokendolpher 2004: 85; Vogel 1967: 86; Vogel 1970b: 10; Young and Edwards 1990: 17
Neoantistea sp. nr riparia (Keyserling, 1887); Irungu 2007: 30 [misidentified]

####### Distribution.

Central, east, and south Texas; Archer, Bexar, Blanco, Brazos, Burleson, Cameron, Carson, Colorado, Comanche, Erath, Galveston, Hidalgo, Kendall, Kerr, La Salle, Llano, Montgomery, Victoria, Walker, Williamson, Zapata

####### Locality.

Attwater Prairie Chicken National Wildlife Refuge, Bill Haney Pecan Orchard, Browning Ranch, Ellis Prison Unit, Green Island Bird Refuge, Pantex Lake, Stiles Farm Foundation

####### Caves.

**Bexar** (Droll Cave, Obvious Little Cave)

####### Time of activity.

Male (March – October, December); female (January, April – November)

####### Habitat.

(crops: cabbage, cotton, peanuts); (grass: grassland); (landscape features: cave); (littoral: near playa); (nest/prey: mud dauber nest [f]); (orchard: pecan); (soil/woodland: post oak savanna with pasture)

####### Method.

pitfall trap [mf]

####### Type.

Mexico, Monterrey

####### Etymology.

Person (collector of paratypes in Texas, Stanley Mulaik)

####### Collection.

MSU, TAMU, TMM

####### Note.

32 miles SE Laredo is in Zapata Co., not Webb Co.

###### Neoantistea oklahomensis

Taxon classificationAnimaliaAraneaeHahniidae

Opell & Beatty, 1976

Neoantistea oklahomensis Henderson 2007: 32, 52, 61, 67–73, 76, 79, 83; Jackman 1997: 164; Yantis 2005: 66, 201 [Opell and Beatty 1976: 409, mf, desc. (figs 42–45)]

####### Distribution.

Angelina, Brazos, Burleson, Colorado, Coryell, Grimes, Madison, Trinity, Tyler, Walker

####### Locality.

Angelina National Forest, Kirby State Forest, Lick Creek Park, Texas A&M University Rangeland Area

####### Time of activity.

Male (January – February, July, August 15-September 17, September – December); female (January – May, May 27-June 15, September 23-October 2, October – December)

####### Habitat.

(soil/woodland: bottomland forest litter, disturbed habitat, forest litter, leaf litter, loblolly pine unmanaged, longleaf pine unmanaged, pine woods [%: 66, 77, 97], post oak savanna with pasture, post oak woods [%: 60, 84, 94, 100], post oak woodland, upland woods)

####### Method.

5 gallon bucket trap [mf]; berlese funnel [mf]; flight intercept trap [f]; pitfall trap [mf]

####### Type.

Oklahoma, near Ripley

####### Etymology.

locality (The name of this species is derived from the state where the type specimens were collected, Opell and Beatty 1976).

####### Collection.

TAMU

##### Family Hersiliidae Thorell, 1870 Genus *Neotama* Baehr & Baehr, 1993

###### Neotama mexicana

Taxon classificationAnimaliaAraneaeHersiliidae

(O. P.-Cambridge, 1893)

Neotama mexicana Bradley 2013: 135; Cutler 2005b: 116, 117; Rheims and Brescovit 2004: 211 [T], mf, desc. (figs 55–61)
Tama mexicana O. P.-Cambridge, 1893; Comstock 1940: 634, desc. (figs 709–710); Gertsch 1935a: 20; Jackman 1997: 46, 164 (photo 15); Roth 1982: 22–1; Roth 1985: B-18–1; Roth 1994: 103; Vogel 1970b: 10

####### Distribution.

Cameron, Hidalgo

####### Locality.

Bentsen-Rio Grande Valley State Park, Sabal Palm Audubon Sanctuary

####### Time of activity.

Male (March, September – November); female (March, July, September – October)

####### Habitat.

(nest/prey: mud dauber nest [m]); (soil/woodland: palm forest, tree trunk at night [mf])

####### Method.

Beating [mf]

####### Type.

Mexico

####### Etymology.

locality (country)

####### Collection.

NMSU, TAMU

#### Family Leptonetidae Simon, 1890

**Note.** *Tayshaneta microps* (Gertsch, 1974) and *Tayshaneta myopica* (Gertsch, 1974), are federally endangered species (listed as *Neoleptoneta* by US Fish and Wildlife Service 2010).

**Note.** species incorrectly reported from Texas

*Leptoneta californica* Banks, 1904; Gertsch 1935a: 21; Roewer 1942: 313; Vogel 1970b: 11 [Texas records]

##### Genus *Chisoneta* Ledford & Griswold, 2011

###### Chisoneta chisosea

Taxon classificationAnimaliaAraneaeLeptonetidae

(Gertsch, 1974)

Chisoneta chisosea Ledford et al. 2011: 339, 371 [T]
Leptoneta chisosea Gertsch, 1974; Gertsch 1974: 175, f, desc. (figs 59, 121)
Neoleptoneta chisosea (Gertsch, 1974); Brignoli 1977: 216 [T]; Jackman 1997: 164

####### Distribution.

Brewster

####### Locality.

Big Bend National Park, Chisos Basin, Chisos Mountains

####### Time of activity.

Female (September, November)

####### Habitat.

(landscape features: ground detritus in ravine)

####### Type.

Texas (female, Brewster Co., Big Bend National Park, Chisos Mountains, September 28, 1950, W. J. Gertsch, holotype, AMNH)

[male unknown]

####### Etymology.

locality (Named for Chisos Mountains of Texas, Gertsch 1974).

##### Genus *Darkoneta* Ledford & Griswold, 2010

###### Darkoneta garza

Taxon classificationAnimaliaAraneaeLeptonetidae

(Gertsch, 1974)

Darkoneta garza Ledford and Griswold, 2010: 16 [T]
Archoleptoneta garza Gertsch, 1974; Gertsch 1974: 201, f, desc.; Jackman 1997: 164; Ledford et al. 2005: 123

####### Distribution.

Garza

####### Time of activity.

Female (October)

####### Type.

Texas (female, Garza Co., 7 miles E Justiceburg, October 12, 1972, V. Roth, B. Firstman, holotype, AMNH)

[male unknown]

####### Etymology.

locality (Named for Garza County, Texas, Gertsch 1974).

##### Genus *Tayshaneta* Ledford & Griswold, 2011

**nomen dubium**

*Leptoneta furtiva* Gertsch, 1974; Gertsch 1974: 176; Jackman 1997: 164; Ledford et al. 2012: 26

*Leptoneta uvaldea* Gertsch, 1974; Gertsch 1974: 172; Ledford et al. 2012: 26

###### Tayshaneta anopica

Taxon classificationAnimaliaAraneaeLeptonetidae

(Gertsch, 1974)

Tayshaneta anopica Ledford et al. 2011: 340–341, 375–376, 386 [T]; Ledford et al. 2012: 28, m, desc. (figs 2D, 12A–F, 33A–F, 52A–B)
Leptoneta spp.; Reddell 1965: 172 [part]
Leptoneta anopica Gertsch, 1974; Gertsch 1974: 172, f, desc. (figs 51, 78); Gertsch 1979: 151
Neoleptoneta anopica (Gertsch, 1974); Brignoli 1977: 216 [T]; Culver et al. 2003: 464; Gertsch 1992: 78; Jackman 1997: 164; Ledford et al. 2005: 123; SWCA Environmental Consultants 2007: 1–2, 3–32

####### Distribution.

Williamson

####### Caves.

**Williamson** (Cobb Cavern [=Cobb’s Caverns], Corn Cobb’s Cave)

####### Time of activity.

Male (March, September); female (March, July, October – November)

####### Habitat.

(landscape features: cave)

####### Type.

Texas (female, Williamson Co., Cobb Cave (= Cobb’s Cavern), March 31, 1963, J. Reddell, D. Mc Kenzie, holotype, AMNH)

####### Etymology.

Greek, without eyes

####### Collection.

TMM, TTU

####### Note.

Cobb Cave is also known as Cobb’s Caverns and located on Cobb Ranch in northern Williamson County.

###### Tayshaneta archambaulti

Taxon classificationAnimaliaAraneaeLeptonetidae

Ledford et al., 2012

Tayshaneta archambaulti Ledford et al. 2012: 30, mf, desc. (figs 13A–F, 34A–F, 52C)

####### Distribution.

Hays

####### Caves.

**Hays** (Burnett Ranch Cave, Grapevine Cave)

####### Time of activity.

Male (April – May, November); female (April, November)

####### Habitat.

(landscape features: cave)

####### Type.

Texas (male, Hays Co., Grapevine Cave, Nov. 18, 2009, J. Ledford, K. O’Connor, holotype, CASC)

####### Etymology.

Person (This species is named in honor of Martin Archambault, fellow caver and friend who helped collect many leptonetids in Texas and Mexico, Ledford et al. 2012).

####### Collection.

TMM

###### Tayshaneta bullis

Taxon classificationAnimaliaAraneaeLeptonetidae

(Cokendolpher, 2004)

Tayshaneta bullis Ledford et al. 2011: 340–341, 376–377, 386 [T]; Ledford et al. 2012: 32 (figs 14A.F, 35A.F, 52D)
Neoleptoneta bullis Cokendolpher, 2004; Cokendolpher 2004c: 65, mf, desc. (figs 1–9); Reddell and Cokendolpher 2004: 85

####### Distribution.

Bexar, Hays, Kerr

####### Locality.

Camp Bullis

####### Caves.

**Bexar** (Hill’s and Dale’s Pit, Up the Creek Cave [Camp Bullis]); **Hays** (Pulpit Cave)

####### Time of activity.

Male (January, September – November); female (January, March, August – November)

####### Habitat.

(landscape features: cave)

####### Type.

Texas (male, Bexar Co., Up the Creek Cave, Camp Bullis, September 10, 1998, J. Cokendolpher, J. Reddell, J. Krejca, M. Reyes, holotype, AMNH)

####### Etymology.

locality (The specific name is a noun in apposition taken from the type locality, Camp Bullis, Cokendolpher 2004c).

####### Collection.

TMM, TTU

###### Tayshaneta coeca

Taxon classificationAnimaliaAraneaeLeptonetidae

(Chamberlin & Ivie, 1942)

Tayshaneta coeca Ledford et al. 2011: 337, 340–341, 377–380, 386 [T] (figs 13A–F 17A–F, 31); Ledford et al. 2012: 34 (figs 10C, 15A–F, 36A–F, 52E)
Leptoneta spp.; Reddell 1965: 172 [part]
Leptoneta coeca Chamberlin and Ivie, 1942; Chamberlin and Ivie 1942: 10, m, desc. (fig. 9); Gertsch 1974: 170, mf, desc. (figs 50, 67–68, 80); Nicholas 1960: 156; Reddell 1965: 172; Vogel 1967: 87; Vogel 1970b: 11
Neoleptoneta coeca (Chamberlin and Ivie, 1942); Brignoli 1977: 216 [T]; Culver et al. 2003: 464; Gertsch 1992: 78; Jackman 1997: 164; Reddell and Cokendolpher 2004: 85
Tayshaneta undet.; Ledford et al. 2011: 342 [part]

####### Distribution.

Comal, Hays, Travis, Williamson

####### Caves.

**Comal** (Brehmmer Cave [=Heidrich’s Cave], Coreth Bat Cave, Natural Bridge Caverns); **Hays** (Freeman Crawl, Hackberry Cave, McCarty Cave, McGlothlin Sink, Root Beard Cave, Wiseman’s Sink, Wiseman’s Sink No. 2); **Williamson** (Flat Rock Cave, Prairie’s Flats Cave)

####### Time of activity.

Male (January, March – June, October); female (January – May, July, September – November)

####### Habitat.

(landscape features: cave)

####### Type.

Texas (male, Comal Co., Brehmmer Cave, June 20, 1938, no collector, holotype, AMNH)

####### Etymology.

Latin, blind, hidden

####### Collection.

TMM, TTU

###### Tayshaneta concinna

Taxon classificationAnimaliaAraneaeLeptonetidae

(Gertsch, 1974)

Tayshaneta concinna Ledford et al. 2011: 340–341, 380–381, 386 [T]; Ledford et al. 2012: 36 (figs 16A–C, 37A–F, 52F)
Leptoneta sp.; Reddell 1965: 171
Leptoneta concinna Gertsch, 1974; Gertsch 1974: 169, mf, desc. (figs 52, 71–72, 76)
Neoleptoneta concinna (Gertsch, 1974); Brignoli 1977: 216 [T]; Culver et al. 2003: 464; Gertsch 1992: 78; Jackman 1997: 164

####### Distribution.

Travis

####### Caves.

**Travis** (County Line Bat Cave, Lost Gold Cave, Seibert Sink [Stinkin Sink], Stark’s North Mine)

####### Time of activity.

Male (May, November); female (January, March, May, August – September, November)

####### Habitat.

(landscape features: cave)

####### Type.

Texas (female, Travis Co., Lost Gold Cave, May 27, 1963, J. Reddell, B. Frank, holotype, AMNH)

####### Etymology.

Latin, pleasing

####### Collection.

TMM, TTU

###### Tayshaneta devia

Taxon classificationAnimaliaAraneaeLeptonetidae

(Gertsch, 1974)

Tayshaneta devia Ledford et al. 2011: 340–341, 381, 385, 386 [T]; Ledford et al. 2012: 38, m, desc. (figs 11B, C, 17A–E, 31B, 32B, 38A–F, 53A)
Leptoneta spp.; Reddell 1965: 172 [part]
Leptoneta devia Gertsch, 1974; Gertsch 1974: 171, f, desc. (figs 54, 81)
Neoleptoneta devia (Gertsch, 1974); Brignoli 1977: 216 [T]; Culver et al. 2003: 464; Gertsch 1992: 78; Jackman 1997: 164

####### Distribution.

Travis, Williamson

####### Caves.

**Travis** (9K-2 Cave [=Moonmilk Cave], Brewpot Sink, Hammett’s Crossing, MacDonald Cave [=Schultz Cave], Stovepipe Cave, Tooth Cave surface); **Williamson** (Village Idiot Cave)

####### Time of activity.

Male (February, September – November); female (January – February, April, August – November)

####### Habitat.

(landscape features: cave); (soil/woodland: leaf litter)

####### Method.

sifting [mf]

####### Type.

Texas (female, Travis Co., Schulze Cave, August 21, 1963, W. Russell, holotype, AMNH)

####### Etymology.

Latin, devius, out of the way

####### Collection.

TMM

###### Tayshaneta emeraldae

Taxon classificationAnimaliaAraneaeLeptonetidae

Ledford et al., 2012

Tayshaneta emeraldae Ledford et al. 2012: 40, mf, desc. (figs 18A–F, 39A–F, 53B)

####### Distribution.

Val Verde

####### Caves.

**Val Verde** (Emerald Sink)

####### Time of activity.

Male (November); female (November)

####### Habitat.

(landscape features: cave)

####### Type.

Texas (male, Val Verde Co., Emerald Sink, November 3, 1984, J. Reddell, M. Reyes, holotype, AMNH)

####### Etymology.

locality (The species name is taken in apposition to the type locality, Ledford et al. 2012).

###### Tayshaneta fawcetti

Taxon classificationAnimaliaAraneaeLeptonetidae

Ledford et al., 2012

Tayshaneta fawcetti Ledford et al. 2012: 42 [S], mf, desc. (figs 2B, 19A–F, 31D, 32D, 40A–F, 53C)
Leptoneta spp.; Reddell 1965: 172 [part]
Leptoneta valverdae Gertsch 1974; Gertsch 1974: 174, mf, desc.
Tayshaneta valverdae (Gertsch, 1974); Ledford et al. 2011: 337

####### Distribution.

Val Verde

####### Caves.

**Val Verde** (Fawcett’s Cave [Devil’s River State Natural Area])

####### Time of activity.

Male (November); female (April, November)

####### Habitat.

(landscape features: cave)

####### Type.

Texas (male, Val Verde Co., Fawcett’s Cave, Devil’s River State Natural Area, November 10, 2009, J. Ledford, J. Kennedy, M. Sanders, T. Garot, K. Wardlaw, holotype, CASC)

####### Etymology.

locality (The species name is taken in apposition to the type locality and honors the Fawcett family, who owned Fawcett’s Cave and the surrounding Fawcett Ranch prior to its transition as a State Natural Area in 1988, Ledford et al. 2012).

####### Collection.

TMM

###### Tayshaneta grubbsi

Taxon classificationAnimaliaAraneaeLeptonetidae

Ledford et al., 2012

Tayshaneta grubbsi Ledford et al. 2012: 45, m, desc. (figs 20A–C, 32E, 41A–F)

####### Distribution.

Val Verde

####### Caves.

**Val Verde** (Litterbarrel Cave)

####### Time of activity.

Male (September)

####### Habitat.

(landscape features: cave)

####### Type.

Texas (male, Val Verde Co., Litterbarrel Cave, September 1, 1974, S. Sweet, M. Reaka, holotype, AMNH)

[female unknown]

####### Etymology.

Person (This species is named in honor of Andy Grubbs, a remarkable collector of several new *Tayshaneta* species throughout Texas, Ledford et al. 2012).

###### Tayshaneta madla

Taxon classificationAnimaliaAraneaeLeptonetidae

Ledford et al., 2012

Tayshaneta madla Ledford et al. 2012: 45, mf, desc. (figs 21A–C, 31F, 32F, 42A–F, 53D)

####### Distribution.

Bexar

####### Caves.

**Bexar** (Cave No. 18, Cave No. 189, Madla’s Cave, Madla’s Drop, Scorpion Cave, Young Cave No. 1)

####### Time of activity.

Male (January, March, June, September, December); female (January, March, June, December)

####### Habitat.

(landscape features: cave)

####### Type.

Texas (male, Bexar Co., Madla’s Cave, December 18, 2003, K. White, holotype, CASC)

####### Etymology.

locality (This species name is taken in apposition to the type locality and honors the Madla family, owners of Madla’s Cave and the surrounding property, Ledford et al. 2012).

####### Collection.

TMM, TTU

###### Tayshaneta microps

Taxon classificationAnimaliaAraneaeLeptonetidae

(Gertsch, 1974)

Tayshaneta microps Ledford et al. 2011: 340, 385, 386 [T]; Ledford et al. 2012: 48, m, desc. (figs 10A–B, 22A–F, 31C, 32C, 43A–F, 53E)
Leptoneta sp.; Reddell 1970: 405
Leptoneta microps Gertsch, 1974; Gertsch 1974: 172, f, desc. (figs 53, 77)
Neoleptoneta microps (Gertsch, 1974); Brignoli 1977: 216 [T]; Culver et al. 2003: 464; Federal Register 1998: 71855–71856, 71858, 71860, 71866; Federal Register 2000: 81419–81421, 81425, 81428, 81433; Federal Register 2002: 55064, 55067, 55073–55074, 55086–55087, 55089; Federal Register 2003: 17156–17158, 17176, 17191; Gertsch 1992: 78; Jackman 1997: 164, 171; NABN 2001: 8; Reddell and Cokendolpher 2004: 86; SWCA Environmental Consultants 2007: 3

####### Distribution.

Bexar

####### Caves.

**Bexar** (Government Canyon Bat Cave)

####### Time of activity.

Male (March, November); female (March – May, August)

####### Habitat.

(landscape features: cave)

####### Type.

Texas (female, Bexar Co., Government Canyon Bat Cave, August 11, 1965, J. Reddell, J. Fish, holotype, AMNH)

####### Etymology.

Greek, small and small eyed

####### Collection.

TMM, TTU

###### Tayshaneta myopica

Taxon classificationAnimaliaAraneaeLeptonetidae

(Gertsch, 1974)

Tayshaneta myopica Ledford et al. 2011: 337, 340–341, 374, 385–386 [T] (figs 30A-D); Ledford et al. 2012: 50 (figs 2A, 2C, 10D, 10E, 11A, 23A–F, 44A–F, 53F)
Leptoneta spp.; Reddell 1965: 172 [part]
Leptoneta myopica Gertsch, 1974; Gertsch 1974: 168, mf, desc. (figs 48–49, 56, 61–62, 65–66, 73); SWCA Environmental Consultants 2007: 3
Neoleptoneta myopica (Gertsch, 1974); Bradley 2013: 137; Brignoli 1977: 216 [T]; Culver et al. 2003: 464; Gertsch 1992: 78; Jackman 1997: 164, 171

####### Distribution.

Travis, Williamson

####### Caves.

**Travis** (Cortaña Cave, Gallifer Cave, Geode Cave, Jester Estate’s Cave, McNeil Bat Cave, New Comanche Trail Cave, Root Cave, Steiner Telephone Pole Cave, Tight Pit, Tooth Cave); **Williamson** (Goat Cave, McNeil Bat Cave, Steiner Telephone Pole Cave)

####### Time of activity.

Male (January – April, June – October); female (January – March, May – October)

####### Habitat.

(landscape features: cave)

####### Type.

Texas (male, Travis Co., Tooth Cave, March 30, 1965, J. Reddell, holotype, AMNH)

####### Etymology.

Greek, nearsighted

####### Collection.

TMM, TTU

###### Tayshaneta oconnorae

Taxon classificationAnimaliaAraneaeLeptonetidae

Ledford et al., 2012

Tayshaneta oconnorae Ledford et al. 2012: 53, m, desc. (figs 24A-C, 45A-F)

####### Distribution.

Hays

####### Caves.

**Hays** (Cathy’s Cave, Fern Cave)

####### Time of activity.

Male (March, May)

####### Habitat.

(landscape features: cave)

####### Type.

Texas (male, Hays Co., Fern Cave, May 26, 1989, A. Grubbs, J. Reddell, M. Reyes, holotype, AMNH)

[female unknown]

####### Etymology.

Person (This species is named in honor of Kathleen O’ Connor, fellow caver and biologist who helped collect many exciting *Tayshaneta* specimens, Ledford et al. 2012).

###### Tayshaneta paraconcinna

Taxon classificationAnimaliaAraneaeLeptonetidae

(Cokendolpher & Reddell, 2001)

Tayshaneta paraconcinna Ledford et al. 2011: 337, 340–341, 385–386 [T]; Ledford et al. 2012: 55 (figs 25A–F, 31A, 32A, 46A–F, 54A)
Neoleptoneta paraconcinna Cokendolpher and Reddell, 2001; Cokendolpher and Reddell 2001b: 46, mf, desc. (figs 12–22)

####### Distribution.

Bell, Blanco, Burnet, Travis, Williamson

####### Locality.

Flat Creek Ranch, Fort Hood, Moon Rocks Ranch, Pedernales State Park

####### Caves.

**Bell** ([all in Fort Hood] Camp 6 Cave No. 1, Figure 8 Cave, Hidden Pit Cave, Peep in the Deep Cave, Talking Crows Cave); **Williamson** (Fissure F-8 [The Sanctuary], Lizard’s Lounge Cave [F-11], On Campus Cave, Salt Lick Cave [The Sanctuary], Scoot Over Cave, Serta Cave, Short Stack Cave, Three Miles Cave [= Three Mile Bat Cave], Twin Springs Cave [= Whitney West Cave])

####### Time of activity.

Male (January, April – May, August, October – December); female (January, March – June, August – December)

####### Habitat.

(landscape features: cave)

####### Type.

Texas (male, Bell Co., Peep in the Deep Cave, May 8, 1998, J. Reddell, M. Reyes, holotype, AMNH)

####### Etymology.

Greek, near concinna (similar species, *Neoleptoneta concinna* (Gertsch, 1974))

####### Collection.

TMM, TTU

###### Tayshaneta sandersi

Taxon classificationAnimaliaAraneaeLeptonetidae

Ledford et al., 2012

Tayshaneta sandersi Ledford et al. 2012: 57, mf, desc. (figs 26A–C, 47A–F, 54B)

####### Distribution.

Travis

####### Caves.

**Travis** (District Park Cave, Slaughter Creek Cave, Whirlpool Cave)

####### Time of activity.

Male (March); female (November)

####### Habitat.

(landscape features: cave)

####### Type.

Texas (female, Travis Co., District Park Cave, November 19, 2009, J. Ledford, M. Sanders, holotype, CASC)

####### Etymology.

Person (This species is named in honor of Mark Sanders, fellow caver, biologist, and collector of several *Tayshaneta* species in Texas, Ledford et al. 2012).

####### Collection.

TMM

###### Tayshaneta sprousei

Taxon classificationAnimaliaAraneaeLeptonetidae

Ledford et al., 2012

Tayshaneta sprousei Ledford et al. 2012: 59, m, desc. (figs 27A–C, 48A–F)

####### Distribution.

Bexar

####### Locality.

Camp Bullis

####### Caves.

**Bexar** (Breached Dam Cave, Constant Sorrow Cave)

####### Time of activity.

Male (March, November)

####### Habitat.

(landscape features: cave)

####### Type.

Texas (male, Bexar Co., Constant Sorrow Cave, Camp Bullis, March 6, 2001, G. Veni, holotype, AMNH)

[female unknown]

####### Etymology.

Person (This species is named in honor of Peter Sprouse, fellow caver, biologist and collector of several *Tayshaneta* species in Texas caves, Ledford et al. 2012).

####### Collection.

TMM

###### Tayshaneta valverdae

Taxon classificationAnimaliaAraneaeLeptonetidae

(Gertsch, 1974)

Tayshaneta valverdae Ledford et al. 2011: 337, 341, 385–386 [T] (fig. 1D); Ledford et al. 2012: 60 (figs 28A–F, 49A–F, 54C)
Leptoneta spp.; Reddell 1965: 172 [part]
Leptoneta valverdae Gertsch, 1974; Gertsch 1974: 173, mf, desc. (figs 57–58, 69–70, 75)
Neoleptoneta valverdae (Gertsch, 1974); Brignoli 1977: 216 [T]; Culver et al. 2003: 464; Gertsch 1992: 78; Jackman 1997: 164

####### Distribution.

Bandera, Uvalde, Val Verde

####### Locality.

Love Creek Ranch, Marneldo Ranch

####### Caves.

**Bandera** (Harvestman Cave [Hill Country State Natural Area], Melanie’s Cave [Hill Country State Natural Area]); **Uvalde** (Big Fucking Snake Cave); **Val Verde** (Oriente Milestone Molasses Bat Cave)

####### Time of activity.

Male (January, April, June – July, October); female (June – July, October)

####### Habitat.

(landscape features: cave)

####### Type.

Texas (male, Val Verde Co., Oriente Milestone Molasses Bat Cave, January 25, 1964, J. Reddell, D. McKenzie, J. Porter, holotype, AMNH)

####### Etymology.

locality (Named for Val Verde County, Texas, Gertsch 1974).

####### Collection.

TMM

###### Tayshaneta vidrio

Taxon classificationAnimaliaAraneaeLeptonetidae

Ledford et al., 2012

Tayshaneta vidrio Ledford et al. 2012: 62, mf, desc. (figs 29A–C, 31E, 50A–F, 54D)

####### Distribution.

Brewster

####### Caves.

**Brewster** (400 Foot Cave)

####### Habitat.

(landscape features: cave)

####### Type.

Texas (male, Brewster Co., 400 Foot Cave, Glass Mountains, no date, no collector, holotype, AMNH)

####### Etymology.

locality (This species name is derived from the Spanish name for the Glass Mountains “Sierra del Vidrio” in West Texas. The name is to be treated as a noun in apposition, Ledford et al. 2012).

###### Tayshaneta whitei

Taxon classificationAnimaliaAraneaeLeptonetidae

Ledford et al., 2012

Tayshaneta whitei Ledford et al. 2012: 63, mf, desc. (figs 30A–F, 51A–F, 54E)

####### Distribution.

Bexar, Medina

####### Caves.

**Bexar** (Caracol Creek Coon Cave, Cave site #801, Lithic Ridge Cave [Government Canyon State Natural Area]); **Medina** (Medina Dam Cave, Nisbet Cave)

####### Time of activity.

Male (March, November); female (March, June, November)

####### Habitat.

(landscape features: cave)

####### Type.

Texas (male, Bexar Co., Lithic Ridge Cave, Government Canyon State Natural Area, November 6, 2002, Engelhard, J. Krejca, holotype, AMNH)

####### Etymology.

Person (This species is named in honor of Kemble White, fellow caver, geologist and collector of many *Tayshaneta* species in Texas, Ledford et al. 2012).

####### Collection.

TMM

#### Family Linyphiidae Blackwall, 1859

**Note.** species incorrectly reported from Texas

*Agyneta fabra* (Keyserling, 1886); Buckle et al. 2001: 100; Dupérré 2013: 120 [not in Texas]

*Meioneta fabra* (Keyserling, 1886); Jackman 1997: 165; Kaston 1953: 206; Kaston 1972: 124; Kaston 1978: 120; Roth 1988: 42

*Erigone fabra* Keyserling, 1886; Marx 1890: 533; Petrunkevitch 1911: 234

*Ceraticelus minutus* Emerton, 1882; Glick 1957: 5 [not in Texas]

*Ceratinopsis interpres* (O. P.-Cambridge, 1874) [not in Texas]

*Erigone interpres* (O. P.-Cambridge, 1874); Marx 1890: 534 [not in Texas]

*Lepthyphantes minutus* (Blackwall, 1833); Jones 1936: 69; Vogel 1970b: 12 [not in Texas]

*Scylaceus pallidus* (Emerton, 1882) [not in Texas]

*Erigone minutissima* Keyserling, 1886; Banks 1910: 31; Petrunkevitch 1911: 236 [not in Texas]

##### Genus *Agyneta* Hull, 1911

###### Agyneta chiricahua

Taxon classificationAnimaliaAraneaeLinyphiidae

Dupérré, 2013

Agyneta chiricahua Dupérré 2013: 107, mf, desc. (figs 336–345)
Meioneta sp. nr llanoensis (Gertsch and Davis, 1936); Calixto et al. 2013: 182, 186–187 [part]; Henderson 2007: 29, 77, 80, 83 [part]
Meioneta sp. nr unimaculata (Banks, 1892); Calixto et al. 2013: 182 [part]; Henderson 2007: 55 [part]; Irungu 2007: 31
Meioneta sp.; Agnew et al. 1985: 3 [part]

####### Distribution.

Bandera, Bexar, Brazos, Brooks, Burleson, Colorado, Comanche, Coryell, Erath, Fayette, Hidalgo, Houston, Robertson, San Patricio, Starr, Titus, Walker, Williamson

####### Locality.

Attwater Prairie Chicken National Wildlife Refuge, Bill Haney Pecan Orchard, Ellis Prison Unit, Holmes Pecan Orchard, Lick Creek Park, NK Ranch, Stiles Farm Foundation

####### Time of activity.

Male (January, April – December); female (April – December)

####### Habitat.

(crops: cabbage, cotton, peanuts); (orchard: pecan); (soil/woodland: post oak savanna with pasture, post oak woodland, rotten logs, upland woods)

####### Method.

D-Vac suction [m]; malaise trap [m]; pitfall trap [mf]; suction trap [mf]; sweeping [m]

####### Type.

Arizona, Cochise Co., Chiricahua Mountains

####### Etymology.

locality (The specific name is a noun in apposition taken from the type locality Chiricahua Mountains, Arizona, USA, Dupérré 2013).

####### Collection.

TAMU, TMM

###### Agyneta crista

Taxon classificationAnimaliaAraneaeLinyphiidae

Dupérré, 2013

Agyneta crista Dupérré 2013: 113, mf, desc. (figs 361–367)
Meioneta sp. nr llanoensis (Gertsch and Davis, 1936); Calixto et al. 2013: 182, 186–187 [part]
Meioneta sp.; Agnew et al. 1985: 3 [part]; Calixto et al. 2013: 182 [part]

####### Distribution.

Brazos, Burleson, Comanche, Coryell, Erath, Kendall, Robertson

####### Locality.

5-Eagle Ranch, Bill Haney Pecan Orchard, Holmes Pecan Orchard, NK Ranch

####### Time of activity.

Male (March – December); female (May – June, August, December)

####### Habitat.

(crops: peanuts); (orchard: pecan); (soil/woodland: post oak savanna with pasture)

####### Method.

pitfall trap [mf]

####### Type.

Utah, 6 miles N Greenriver

####### Etymology.

Latin, rooster-comb, in reference to the shape of the embolus prong

####### Collection.

TAMU

###### Agyneta flax

Taxon classificationAnimaliaAraneaeLinyphiidae

Dupérré, 2013

Agyneta flax Dupérré 2013: 89, mf, desc. (figs 265–273)
Meioneta sp. nr llanoensis (Gertsch and Davis, 1936); Calixto et al. 2013: 182, 186–187 [part]

####### Distribution.

Bastrop, Cameron, Comanche, Coryell, Fayette, Hidalgo, Montague, San Patricio, Starr, Travis

####### Locality.

Bastrop State Park, Bill Haney Pecan Orchard, Sabal Palm Audubon Sanctuary

####### Caves.

**Travis** (Three-Holer Cave)

####### Time of activity.

Male (March – November); female (February – May, July – September, November)

####### Habitat.

(landscape features: cave); (soil/woodland: oak-pine litter, post oak savanna with pasture)

####### Method.

Berlese funnel [f]; pitfall trap [m]

####### Type.

Arizona, Cochise Co., Chiricahua Mountains

####### Etymology.

noun in apposition, sickle-shaped lamella characteristica

####### Collection.

TAMU, TMM

###### Agyneta llanoensis

Taxon classificationAnimaliaAraneaeLinyphiidae

(Gertsch & Davis, 1936)

Agyneta llanoensis Buckle et al. 2001: 100; Calixto et al. 2013: 182; Dupérré 2013: 17, 130, mf, desc. (figs 425–431); Paquin et al. 2009: 39 [T], mf, desc. (figs 1–11, 25–26); Reddell and Cokendolpher 2004: 87
Meioneta llanoensis (Gertsch and Davis, 1936); Cokendolpher and Reddell 2001b: 51; Jackman 1997: 165; Roth 1988: 42
Microneta llanoensis Gertsch and Davis, 1936; Bonnet 1957: 2900; Gertsch and Davis 1936: 11, m, desc. (figs 14–16); Roewer 1942: 522; Vogel 1970b: 12
Meioneta sp. nr llanoensis (Gertsch and Davis, 1936); Calixto et al. 2013: 186–187 [part]; Henderson 2005: 29, 77, 80, 83 [part]
Meioneta sp. nr unimaculata (Banks,1892); Calixto et al. 2013: 182 [part]
Meioneta sp.; Agnew et al. 1985: 3 [part]

####### Distribution.

Angelina, Bandera, Bell, Bexar, Blanco, Brazos, Burleson, Burnet, Childress, Comal, Comanche, Coryell, Edwards, Erath, Gillespie, Hays, Irion, Kendall, Kerr, Kinney, Lampasas, Llano, Mason, Medina, Real, Robertson, San Saba, Schleicher, Sutton, Terrell, Travis, Uvalde, Val Verde, Williamson

####### Locality.

5-Eagle Ranch, Bill Haney Pecan Orchard, Camp Bullis, Fort Hood, Lick Creek Park, Sattler and Hoffman Ranch

####### Caves.

**Bandera** (Bob Clark Cave); **Bell** ([all Fort Hood] Afternoon Cave, Awesome Entrance Cave, Big Crevice, Blue Bottle Sink, Blue Green Hole Cave, Boca Verde Cave, Born Again Cave, Buchanan Cave, Bumelia Well Cave, C. B. Cave, Camp 6 Cave No. 1, Cellular Cave, Chupacabra Pit Cave, Cicurina Cave, Copperdead Cave, Corkscrew Cave, Craggy Rock Cave, Deceiving Sink, Deep in Dis Bear Cave, Dual Sinks Cave, Dying Oak Cave, Endless Pit Cave, Estes Cave, Falling Hat Cave, Falling Turtle Cave, Fellers Cave, Figure 8 Cave, Fire Break Cave, Fools Cave, Forbidden Chasm Cave, Forgotten Cave, Forgotten Sink, Geocache Cave, Gnarla Cave, Green Carpet Cave, Hammer Crack Cave, Hidey Ho Cave, Hope Well Sink, Humpty Cave, Jagged Walls Cave, L. Z. Sid Cave, Legless Visitor Cave, Leopard Frog Cave, Long Joint Sink, Lost Chasm Cave, Lucky Rock Cave, Marcelino’s Cave, Molly Hatchet Cave, Nolan Creek Cave, Owl Mountain Cave, Peep in the Deep Cave, Plethodon Cave, Plethodon Pit Cave, Raining Rattler Cave, Road Side Sink, Rugger’s Rift Cave, Rusty Cans Cave, Sanford Pit Cave, Seven Mile Mountain Cave, Skeeter Cave, Sledgehammer Cave, Sleepy Hollow Cave, Sleepy Hollow Pit, Slotsky Pit Cave, Soldiers Cave, Southern Cross Cave, Stand-Off Sink, Stone Eyes Sink, Streak Cave, Talking Crows Cave, Thumbs Up Cave, Tinaja Cave, Tony’s Can Cave, Treasure Cave, Triple J Cave, Tweedledum Cave, Valentine Cave, Vine Cave, Violet Cave, Viper Den Cave, Weep Hole Cave, West Corral Cave No. 1, West Corral Cave No. 2, West Corral Cave No. 4, West Corral Sink); **Bexar** (B-52 Cave [Camp Bullis], Bexar (=Bear) Cave, Black Cat Cave, Bunny Hole [Camp Bullis], Cannonball Cave [Camp Bullis], Cave site #602, Cave site #603, Christmas Cave, Dangerfield Cave [Camp Bullis], Dogleg Cave [Camp Bullis], Droll Cave, Eagles Nest Cave [Camp Bullis], Elm Springs Cave, Elm Water Hole Cave, Flying Buzzworm Cave [Camp Bullis], Forked Pit, Game Pasture Cave No. 1, Government Canyon Bat Cave, Hairy Tooth Cave, King Toad Cave, La Cantera Cave No. 3, Linda’s First Cave Find, Lone Gunman Pit [Camp Bullis], Low Priority Cave [Camp Bullis], Max and Roberts Cave [=SWCA cave site No. 3007], Meusebach Flats Cave, Obvious Little Cave, Peace Pipe Cave [Camp Bullis], Plethodon Pit (Stone Oak Karst Region), Porcupine Parlor Cave [Camp Bullis], Raging Cajun Cave, Rattlesnake Cave, Root Canal Cave [Camp Bullis], Root Toupee Cave [Camp Bullis], Stevens Ranch Trash Hole Cave, Strange Little Cave [Camp Bullis], SWCA Cave 3, Tin Pot Cave [Camp Bullis], Wurzbach Bat Cave, Yellow Ball Cave [Camp Bullis]); **Blanco** (Wells Sink); **Burnet** (Cricket City Sink, Eckhardt Root Cave, Fenceline Sink, Longhorn Caverns, Pie Cave, Railroad Cave, Resurrection Well, Simons Pretty Pit, Simons Water Cave, Taylor Water Cave, Washout Cave); **Childress** (Windmill Crack Cave); **Comal** (Bad Weather Pit, Camp Bullis Cave No. 1 [Camp Bullis], Ebert Cave, Fisher’s Pit, Kappelman Salamander Cave, Klar’s Cave, Snake Skin Pit [Camp Bullis]); **Coryell** ([all Fort Hood] Big Red Cave, Chigiouxs’ Cave, Copperhead Cave, Cornelius Cave, Diamond Cave, Dionne Cave, Egypt Cave, Formation Cave, Ingram Cave, Keyhole Cave, Lucky Day Cave, New Cave, Plateau Cave No. 2, Porter Cave, Sperry Cave, Tippit Cave, Wagontop Spring Cave); **Edwards** (Jenkins Skylight Stream Cave, Killer Frog Cave, Wyatt Cave); **Gillespie** (Cave Creek Mosquito Cave); **Hays** (Boyett’s Cave, McCarty Cave, Taylor Bat Cave, Wimberly Bat Cave); **Irion** (Arden Cave, Murphy Wells Cave); **Kendall** (474 Cave, Behr’s Cave, Charley’s Downclimb Cave, Covered Hole, Pfeiffer’s Water Cave, Sattler’s Deep Pit, Schroeder Bat Cave); **Kerr** (Seiker’s Cave, Wilson Ranch Cave); **Kinney** (Kelley Cave, Webb Cave); **Lampasas** (Battery Cave); **Mason** (Kothmann Cave, Mill Creek Cavern, Zesch Ranch Cave); **Medina** (Haby Bat Cave, Koch Cave); **Real** (Red Arrow Cave); **San Saba** (Gorman Cave, Harrell’s Cave, Lemon’s Cave, Whiteface Cave); **Schleicher** (Cave Y); **Sutton** (Felton Cave Root, Harrison Cave); **Terrell** (Goode Cave, Pasotex Pit, The Crack); **Travis** (Amber Cave, Armadillo Ranch Sink, Broken Arrow Cave, Cave site #401, Ceiling Slot Cave, Chuck’s Joint, Coon Slide Cave, Cotterell Cave, Driskill Cave, GCWA Cave, Jack’s Joint Cave, Jest John Cave, Jollyville Plateau Cave, Kretschmarr Double Pit, Lunsford’s Cave, Midden Sink, No Rent Cave, Rolling Rock Cave, Two Trunks Cave, Weldon Cave, Windmill Cave); **Uvalde** (Barn-sized Fissure Cave, Tampke Ranch Cave, Whitecotton Bat Cave); **Val Verde** (H.T. Miers Cave, Powers Ranch Bat Cave, Wren Cave); **Williamson** (A. J. and B. L. Wilcox Cave, Avant Ranch Cave, Avery Ranch Cave, Avery Stairstep Cave, Ballroom #2 Cave, Bat Well Cave, Beck Bat Cave, Beck Creek Cave, Beck Crevice Cave, Beck Horse Cave, Beck Pride Cave, Beck Ranch Cave, Beck Rattlesnake Cave, Beck Sewer Cave, Behren’s Ranch Cave, Blowhole Cave, Boyd’s Void Cave, Broken Plate Cave, Brown’s Cave, Buttercup Blow Hole Cave, Cat Cave, Cat Hollow Bat Cave, Cat Hollow Cave No. 3, Cave Coral Cave, Chagas Cave, Clan Cave, Cobb Cavern, Cricket Cave, Dion Cave, Double Nickel Cave, Duckworth Bat Cave, Feature No. 1, Fern Cave, Fortune 500 Cave, Godwin’s Goat Grave Cave (=Lift Station Cave), Grimace Cave, Hatchet Cave, Holler Hole Cave, Hook Cave, Ilex Cave, Joker Cave, Jug Cave, Killian Caver, LakeLine Cave, LakeLine Mall Well Trap No. 3, Leaning Tree Cave, Man-With-A-Spear Cave, Maverick Cave, Mayfield Cave, Medicine Man Cave, Millennium Cave, Mongo Cave, Mustard Cave, Near Miss Cave, O’Connor Cave, Off Campus Cave, Paleospring Cave, Pemmican Cave, Prairie Flats Cave, Price Is Right Cave, Prospectors Cave, Raccoon Cave, Rattlesnake Filled Cave, Rock Ridge Cave, Rockfall Cave, Rootin Tootin Cave, Salamander Squeeze Cave, Snowmelt Cave, Squeeze-Down Cave, Stepstone Cave, Testudo Tube, Texella Cave, The Abyss, The Chimney, Thin Roof Cave, Two Hole Cave, Underline Cave, Vault Cave, Velcro Cave, Venom Cave, Village Idiot Cave, Water Tank Cave, Water Tower Cave, Waterfall Canyon Cave, White Wall Cave, Wild Card Cave, Zapata Cave)

####### Time of activity.

Male (January – December); female (January -December)

####### Habitat.

(crops: peanuts); (landscape features: cave wall and guano); (orchard: pecan); (soil/woodland: leaf litter, longleaf pine managed, upland woods)

####### Method.

Berlese funnel [f]; pitfall trap [m]; suction trap [m]

####### Type.

Texas (male, Llano Co., Llano, December 1934, L. I. Davis, holotype, AMNH)

####### Etymology.

locality (city)

####### Collection.

JCC, TAMU, TMM

###### Agyneta micaria

Taxon classificationAnimaliaAraneaeLinyphiidae

(Emerton, 1882)

Agyneta micaria Dupérré 2013: 118 [T], mf, desc. (figs 28, 380–389)
Microneta micaria (Emerton, 1882); Vogel 1970b: 12
Meioneta sp. nr llanoensis (Gertsch and Davis, 1936); Calixto et al. 2013: 186–187 [part]; Henderson 2007: 54, 77, 80, 83 [part]
Meioneta sp. nr unimaculata (Banks,1892); Calixto et al. 2013: 182 [part]; Henderson 2007: 54–57, 65–66, 69, 75 [part]
Meioneta sp.; Agnew et al. 1985: 3 [part]; Calixto et al. 2013: 182 [part]; Dean and Sterling 1987: 6; Dean et al. 1982: 254

####### Distribution.

Bexar, Brazos, Burleson, Caldwell, Comanche, Coryell, Erath, Harris, Houston, Red River, Robertson, San Patricio, Travis, Walker

####### Locality.

Bill Haney Pecan Orchard, Ellis Prison Unit, Holmes Pecan Orchard, Lick Creek Park, Lockhart State Park

####### Caves.

**Travis** (Backhole)

####### Time of activity.

Male (March – July, September – December); female (March – June, August)

####### Habitat.

(crops: cotton, peanuts); (orchard: pecan); (soil/woodland: disturbed habitat, leaf litter, post oak savanna with pasture, riverine forest floor, woods)

####### Method.

Fogging [m]; pitfall trap [f]; ramp trap [m]; suction trap [mf]; tile trap [m]

####### Type.

Connecticut, New Haven

####### Etymology.

Latin, crumb

####### Collection.

TAMU, TMM

###### Agyneta parva

Taxon classificationAnimaliaAraneaeLinyphiidae

(Banks, 1896)

Agyneta parva Dupérré 2013: 96, mf, desc. (figs 23, 290–299)
Meioneta sp. nr llanoensis (Gertsch and Davis, 1936); Calixto et al. 2013: 186–187 [part]
Meioneta sp. nr meridionalis (Crosby and Bishop 1936); Henderson 2007: 54, 77, 80, 83 [part]
Meioneta sp. nr unimaculata (Banks,1892); Calixto et al. 2013: 182 [part]; Henderson 2007: 54, 65, 77, 80, 83 [part]
Meioneta sp.; Calixto et al. 2013: 182 [part]; Henderson 2007: 54, 77, 80, 83 [part]
nr Meioneta sp. ; Henderson 2007: 73, 77 [part]

####### Distribution.

Angelina, Brazos, Burleson, Comanche, Coryell, Erath, Robertson, Walker

####### Locality.

Bill Haney Pecan Orchard, Ellis Prison Unit, Holmes Pecan Orchard, Lick Creek Park

####### Time of activity.

Male (March – June); female (January 15-February 15, April – July)

####### Habitat.

(orchard: pecan); (soil/woodland: bottomland hardwood, disturbed habitat, post oak savanna with pasture, post oak woodland, sandy area, sedge meadow, woods)

####### Method.

pitfall trap [mf] (near pond [f]); suction trap [mf]

####### Type.

Washington D. C.

####### Etymology.

Latin, little

####### Collection.

TAMU

###### Agyneta regina

Taxon classificationAnimaliaAraneaeLinyphiidae

(Chamberlin & Ivie, 1944)

Agyneta regina Dupérré 2013: 109, mf, desc. (figs 346–353)
Meioneta sp. nr llanoensis (Gertsch and Davis, 1936); Calixto et al. 2013: 182, 186–187 [part]
Meioneta sp. nr unimaculata (Banks,1892); Calixto et al. 2013: 182 [part]; Henderson 2007: 69, 77, 80, 83 [part]
Meioneta sp.; Agnew et al. 1985: 3 [part]; Calixto et al. 2013: 182 [part]

####### Distribution.

Brazos, Burleson, Colorado, Comanche, Coryell, Erath, Robertson, Wharton

####### Locality.

Attwater Prairie Chicken National Wildlife Refuge, Bill Haney Pecan Orchard, Holmes Pecan Orchard, Lick Creek Park

####### Time of activity.

Male (March, May – October); female (March – September)

####### Habitat.

(crops: cotton, peanuts); (orchard: pecan); (soil/woodland: post oak savanna with pasture, upland woods)

####### Method.

pitfall trap [mf]; suction trap [mf]

####### Type.

Georgia, 3 miles SE Savannah

####### Etymology.

Latin, queen

####### Collection.

TAMU

###### Agyneta sandia

Taxon classificationAnimaliaAraneaeLinyphiidae

Dupérré, 2013

Agyneta sandia Dupérré 2013: 92, mf, desc. (figs 283–290)
Meioneta sp. nr llanoensis (Gertsch and Davis, 1936); Calixto et al. 2013: 186–187 [part]
Meioneta sp.; Calixto et al. 2013: 182 [part]

####### Distribution.

Bastrop, Bexar, Burleson, Caldwell, Comanche, Erath, Travis, Walker

####### Locality.

Bastrop State Park, Bill Haney Pecan Orchard, Camp Bullis, Ellis Prison Unit

####### Caves.

**Bexar** (Constant Sorrow Cave [Camp Bullis], Get A Rope Cave [Camp Bullis], Mastodon Pit)

####### Time of activity.

Male (April – May); female (April – August, October)

####### Habitat.

(orchard: pecan orchard); (soil/woodland: oak woods, post oak savanna with pasture)

####### Method.

pitfall trap [mf]; suction trap [mf]

####### Type.

New Mexico, Bernalillo Co., Sandia Mountains

####### Etymology.

locality (The specific name is a noun in apposition taken from the type locality Sandia Mountains, New Mexico, USA, Dupérré 2013).

####### Collection.

JCC, TAMU, TMM

###### Agyneta serrata

Taxon classificationAnimaliaAraneaeLinyphiidae

(Emerton, 1909)

Agyneta serrata Dupérré 2013: 136, mf, desc. (figs 443–449)
Meioneta sp. nr unimaculata (Banks, 1892); Henderson 2007: 54, 57, 66, 75, 77, 80, 83 [part]
Meioneta sp.; Agnew et al. 1985: 3 [part]

####### Distribution.

Angelina, Bexar, Brazos, Burleson, Cameron, Colorado, Comal, Coryell, Erath, Fayette, Harris, Hidalgo, San Patricio, Starr, Travis, Walker, Williamson

####### Locality.

Attwater Prairie Chicken National Wildlife Refuge, Camp Bullis, Ellis Prison Unit, Fresnos Resaca, Lick Creek Park, NK Ranch, Sabal Palm Audubon Sanctuary, Somerville Lake

####### Caves.

**Bexar** (Backhole [Camp Bullis], Wurzbach Bat Cave); **Comal** (Ebert Cave); **Williamson** (Valley Cave)

####### Time of activity.

Male (March – September, November – December); female (March – July, September, December)

####### Habitat.

(crops: peanuts); (soil/woodland: loblolly pine unmanaged, post oak savanna with pasture, post oak woodland, upland woods)

####### Method.

pitfall trap [mf]; suction trap [mf]

####### Type.

Massachusetts, Boston

####### Etymology.

Latin, ridge on tarsus of palp

####### Collection.

JCC, TAMU, TMM

###### Agyneta spicula

Taxon classificationAnimaliaAraneaeLinyphiidae

Dupérré, 2013

Agyneta spicula Dupérré 2013: 102, mf, desc. (figs 316–325)

####### Distribution.

Erath, Hardeman, Hidalgo, Kendall, Travis

####### Caves.

**Hardeman** (Walkup Cave)

####### Time of activity.

Male (July); female (January, May, July – August)

####### Type.

Texas (male, Kendall Co., Comfort, July 8, 1936, L. I. Davis, holotype, AMNH)

####### Etymology.

noun in apposition, spine-like retrolateral tibial apophysis

####### Collection.

TAMU

###### Agyneta tuberculata

Taxon classificationAnimaliaAraneaeLinyphiidae

Dupérré, 2013

Agyneta tuberculata Dupérré 2013: 115, mf, desc. (figs 368–375)
Meioneta sp. nr llanoensis (Gertsch and Davis, 1936); Calixto et al. 2013: 182, 186–187 [part]

####### Distribution.

Brazos, Hidalgo, Kerr, Lubbock, Robertson, Starr, Travis

####### Locality.

Holmes Pecan Orchard, Raven Ranch

####### Time of activity.

Male (January, April – May, July, December); female (June)

####### Habitat.

(orchard: pecan); (soil/woodland: *Juniperus ashei*)

####### Method.

suction trap [m]

####### Type.

Arizona, Cochise Co., Portal

####### Etymology.

Latin, tuberculate cymbium

####### Collection.

TAMU

##### Genus *Centromerus* Dahl, 1886

###### Centromerus latidens

Taxon classificationAnimaliaAraneaeLinyphiidae

(Emerton, 1882)

Centromerus latidens Buckle et al. 2001: 105; Helsdingen 1973: 21 mf, desc. (figs 14–17); Jackman 1997: 164; Roth 1988: 38

####### Distribution.

Brazos

####### Locality.

Texas A&M University Rangeland Area

####### Time of activity.

Female (April, December)

####### Habitat.

(soil/woodland: on ground)

####### Method.

pitfall trap [f]

####### Type.

Connecticut, New Haven

####### Etymology.

Latin, tarsus of male palpus wide

####### Collection.

TAMU

##### Genus *Ceraticelus* Simon, 1884

###### Ceraticelus creolus

Taxon classificationAnimaliaAraneaeLinyphiidae

Chamberlin, 1925

Ceraticelus creolus [Crosby and Bishop 1925: 19, mf, desc. (figs 27–31)]
Ceraticelus spp.; Dean and Eger 1986: 141 [part]

####### Distribution.

Brazos, Walker

####### Locality.

Ellis Prison Unit

####### Time of activity.

Male (May); female (April – May)

####### Habitat.

(crops: cotton); (plants: bluebonnets, Indian paintbrush)

####### Method.

sweeping [mf]

####### Type.

Louisiana, Benton

####### Etymology.

type of people in Louisiana

####### Collection.

TAMU

###### Ceraticelus emertoni

Taxon classificationAnimaliaAraneaeLinyphiidae

(O. P.-Cambridge, 1874)

Ceraticelus emertoni Jackman 1997: 164; Jones 1936: 69; Vogel 1970b: 11 [Crosby and Bishop 1925: 20, mf, desc. (figs 32–36, 112)]

####### Distribution.

Dallas, Walker

####### Locality.

Ellis Prison Unit

####### Time of activity.

Female (May)

####### Habitat.

(crops: cotton)

####### Type.

Massachusetts

####### Etymology.

Person (arachnologist)

####### Collection.

TAMU

###### Ceraticelus laetus

Taxon classificationAnimaliaAraneaeLinyphiidae

(O. P.-Cambridge, 1874)

Ceraticelus laetus [Crosby and Bishop 1925: 29, mf, desc. (pl. 6, figs 52–55)]

####### Distribution.

Coryell

####### Time of activity.

Male (May)

####### Habitat.

(soil/woodland: post oak savanna with pasture)

####### Method.

pitfall trap [m]

####### Type.

Massachusetts, Cambridge

####### Etymology.

Latin, pleasant

####### Collection.

TAMU

###### Ceraticelus paludigenus

Taxon classificationAnimaliaAraneaeLinyphiidae

Crosby & Bishop, 1925

Ceraticelus paludigenus Buckle et al. 2001: 107 [spelling]; Dean and Sterling 1990: 401; Jackman 1997: 164
Ceraticelus paludigena Crosby and Bishop, 1925; Dean and Eger 1986: 141 [Crosby and Bishop 1925: 39, mf, desc. (figs 86–91)]

####### Distribution.

Brazos, Victoria

####### Time of activity.

Male (April); female (August)

####### Habitat.

(plants: Indian paintbrush)

####### Method.

suction trap [f]; sweeping [m]

####### Type.

Georgia, Okefenokee Swamp, Billy’s Island

####### Etymology.

Latin, birth in stream

####### Collection.

TAMU

###### Ceraticelus paschalis

Taxon classificationAnimaliaAraneaeLinyphiidae

Crosby & Bishop, 1925

Ceraticelus paschalis Buckle et al. 2001: 107; Dean and Sterling 1990: 401, 404; Jackman 1997: 164 [Crosby and Bishop 1925: 40, mf, desc. (figs 92–94)]

####### Distribution.

Brazos, Walker

####### Locality.

Ellis Prison Unit

####### Time of activity.

Female (April, August, November)

####### Method.

suction trap [f]

####### Type.

United States

####### Etymology.

Latin, of Easter

####### Collection.

TAMU

###### Ceraticelus phylax

Taxon classificationAnimaliaAraneaeLinyphiidae

Ivie & Barrows, 1935

Ceraticelus phylax Prentice and Redak 2009: 42, mf, desc. (figs 1–13)

####### Distribution.

Hidalgo, Kerr, Wichita

####### Time of activity.

Female (December)

####### Type.

Oklahoma

####### Etymology.

Greek, preserve

####### Collection.

MSU

###### Ceraticelus similis

Taxon classificationAnimaliaAraneaeLinyphiidae

(Banks, 1892)

Ceraticelus similis Calixto et al. 2013: 182; Rogers and Horner 1977: 523; Young and Edwards 1990: 18 [Crosby and Bishop 1925: 42, mf, desc. (figs 98–102)]
Ceraticelus spp.; Dean and Eger 1986: 141 [part]
Ceraticelus sp. B; Agnew et al. 1985: 3 [part]; Dean and Sterling 1987: 6 [part]; Nyffeler et al. 1992c: 2; Young and Edwards 1990: 18 [sp. not sp. B]

####### Distribution.

North-central and south Texas; Brazos, Burleson, Cameron, Collin, Colorado, Delta, Erath, Fort Bend, Hidalgo, Kaufman, Nueces, Robertson, Walker, Wharton, Williamson

####### Locality.

5-Eagle Ranch, Attwater Prairie Chicken National Wildlife Refuge, Ellis Prison Unit, Holmes Pecan Orchard, NK Ranch, Stiles Farm Foundation

####### Time of activity.

Male (February – September, November – December); female (January – December)

####### Habitat.

(crops: cotton, guar, peanuts); (grass: grass); (orchard: pecan); (plants: bluebonnets, Indian paintbrush, *Monarda citriodora*)

####### Method.

D-Vac suction [mf]; fogging [mf]; pitfall trap [mf]; suction trap [mf]; sweeping [mf]

####### Type.

New York, Ithaca, South Hill, Six Mile Creek

####### Etymology.

Latin, similar to another species

####### Collection.

TAMU

##### Genus *Ceratinella* Emerton, 1882

###### Ceratinella brunnea

Taxon classificationAnimaliaAraneaeLinyphiidae

Emerton, 1882

Ceratinella brunnea Cokendolpher et al. 2008: 9, 68 (fig. 7) [Kaston 1948: 158, mf, desc. (figs 405–407)]

####### Distribution.

Burleson, Carson, Coryell

####### Time of activity.

Male (March – October); female (March – July, July 27-August 3, September – October)

####### Habitat.

(grass: grassland); (soil/woodland: post oak savanna with pasture)

####### Method.

pitfall trap [mf]

####### Type.

New Hampshire, Mt. Washington; Massachusetts, Salem and Sangus; Connecticut, New Haven

####### Etymology.

Latin, color dark brown

####### Collection.

TAMU

###### Ceratinella playa

Taxon classificationAnimaliaAraneaeLinyphiidae

Cokendolpher, Torrence, Smith & Dupérré, 2007

Ceratinella playa Cokendolpher et al. 2007: 52, mf, desc. (figs 4–6, 8, 10–16); Cokendolpher et al. 2008: 23 (fig. 7)

####### Distribution.

Briscoe

####### Time of activity.

Male (June), female (June)

####### Habitat.

(littoral: playa); (plants: emergent plants)

####### Type.

Texas (male, Briscoe Co., Playa BR13, June 15, 2005, S. M. Torrence, L. M. Smith, holotype, TTU)

####### Etymology.

noun in apposition, depressional wetlands, shallow

####### Collection.

TTU

##### Genus *Ceratinops* Banks, 1905

###### Ceratinops crenatus

Taxon classificationAnimaliaAraneaeLinyphiidae

(Emerton, 1882)

Ceratinops crenatus Buckle et al. 2001: 108 [spelling]; Calixto et al. 2013: 182, 186–187 [see note below], Dean and Sterling 1990: 402, 404; Henderson 2007: 68, 77, 79, 83; Jackman 1997: 164
Ceratinops crenata (Emerton, 1882); Agnew et al. 1985: 3, 9; Young and Edwards 1990: 18 [Crosby and Bishop 1933: 111, mf, desc. (figs 10–16)]

####### Distribution.

Brazos, Caldwell, Comanche, Coryell, Erath, Walker, Wichita

####### Locality.

Bill Haney Pecan Orchard, Ellis Prison Unit, Lick Creek Park

####### Time of activity.

Male (March – October); female (April – October)

####### Habitat.

(crops: peanuts); (orchard: pecan); (plants: miscellaneous vegetation); (soil/woodland: post oak savanna with pasture, post oak woodland, sandy area, under juniper)

####### Method.

Fogging [mf]; pitfall trap [mf] (in sand [mf], under juniper [m]); suction trap [mf]; sweeping [f]

####### Type.

Massachusetts, Beverly; Connecticut, New Haven

####### Etymology.

Latin, rounded projection

####### Collection.

MSU, TAMU

####### Note.

*Ceraticelus* erroneously used in Calixto et al. 2013 (p. 182).

###### Ceratinops latus

Taxon classificationAnimaliaAraneaeLinyphiidae

(Emerton, 1882)

Ceratinops latus Platnick 1998: 336 [spelling]
Ceratinops lata (Emerton, 1882) [Crosby and Bishop 1933: 114, mf, desc. (figs 22–28)]
Ceratinops sp.; Agnew et al. 1985: 6

####### Distribution.

Colorado, Erath

####### Locality.

Attwater Prairie Chicken National Wildlife Refuge

####### Time of activity.

Male (April – July); female (July)

####### Habitat.

(soil/woodland: post oak savanna with pasture)

####### Method.

pitfall trap [m]; suction trap [m]

####### Type.

Massachusetts, Watertown

####### Etymology.

Latin, wide

####### Collection.

TAMU

###### Ceratinops rugosus

Taxon classificationAnimaliaAraneaeLinyphiidae

(Emerton, 1909)

Ceratinops rugosus Jackman 1997: 164; Platnick 1998: 336 [spelling]
Ceratinops rugosa (Emerton, 1909); Dean and Eger 1986: 141 [Crosby and Bishop 1933: 116, mf, desc. (figs 35–39)]

####### Distribution.

Brazos

####### Time of activity.

Female (April)

####### Habitat.

(plants: bluebonnets)

####### Method.

sweeping [f]

####### Type.

Massachusetts, Grafton; New Hampshire, Lake Winnipesaukee, Three-mile Island

####### Etymology.

Latin, cephalothorax and sternum rough

####### Collection.

TAMU

##### Genus *Ceratinopsis* Emerton, 1882

###### Ceratinopsis laticeps

Taxon classificationAnimaliaAraneaeLinyphiidae

Emerton, 1882

Ceratinopsis laticeps Buckle et al. 2001: 109; Dean and Sterling 1990: 402; Jackman 1997: 164 [Bishop and Crosby 1930: 21, mf, desc. (figs 18–21)]

####### Distribution.

Brazos, Colorado

####### Locality.

Attwater Prairie Chicken National Wildlife Refuge

####### Time of activity.

Male (April – May)

####### Method.

pitfall trap [m]; suction trap [m]

####### Type.

Massachusetts, Danvers; Connecticut, New Haven

####### Etymology.

Latin, side of head

####### Collection.

TAMU

##### Genus *Erigone* Audouin, 1826

###### Erigone autumnalis

Taxon classificationAnimaliaAraneaeLinyphiidae

Emerton, 1882

Erigone autumnalis Agnew et al. 1985: 3, 9; Breene et al. 1993c: 17, 47, 108, mf (figs 170A-C); Brown 1974: 234; Bryant 1940: 326; Buckle et al. 2001: 115; Calixto et al. 2013: 182, 182, 185, 187, 190; Cokendolpher and Reddell 2001b: 50; Crosby and Bishop 1928: 19, mf, desc. (figs 18–20); Dean and Eger 1986: 141; Dean and Sterling 1987: 6; Dean and Sterling 1990: 402, 404; Dean et al. 1982: 254; Dean et al. 1988: 286; Henderson 2007: 29, 52–54, 56, 59, 66, 77, 79, 83; Jackman 1997: 164; Jones 1936: 70; Liao et al. 1984: 410; Nyffeler et al. 1992c: 2; Pamanes-Guerrero 1975: 41, 59, 78, 81; Roth 1988: 8; Vogel 1970b: 11; Young and Edwards 1990: 18

####### Distribution.

Angelina, Atascosa, Bastrop, Bee, Bell, Brazos, Burleson, Collin, Colorado, Comanche, Coryell, Dallas, Delta, Erath, Fayette, Hidalgo, Houston, Kerr, Nacogdoches, Robertson, Travis, Walker, Wichita, Williamson

####### Locality.

5-Eagle Ranch, Attwater Prairie Chicken National Wildlife Refuge, Bastrop State Park, Bill Haney Pecan Orchard, Ellis Prison Unit, Fort Hood, Holmes Pecan Orchard, Lick Creek Park, NK Ranch, Sam Houston National Forest, Somerville Lake, Stiles Farm Foundation, Stubblefield Lake, Texas A&M University Rangeland Area

####### Caves.

**Bell** (Fellers Cave [Fort Hood]); **Coryell** (Fossil Spring Cave [Fort Hood])

####### Time of activity.

Male (January – December); female (January – December)

####### Habitat.

(crops: alfalfa, cotton, peanuts); (grass: grass, grassland); (landscape features: cave); (orchard: pecan); (plants: bluebonnets, Indian paintbrush, *Monarda citriodora*, *Solanum elaeagnifolium*); (soil/woodland: leaf litter, longleaf pine managed, rotting pine log, post oak savanna with pasture, post oak woodland, sandy area, upland woods, woods, *Juniperus ashei*, *Quercus buckleyi*, *Quercus virginiana*, *Ulmus crassifolia*)

####### Method.

Beating [mf]; cardboard band [m]; D-Vac suction [m]; fogging [mf]; pitfall trap [mf]; ramp trap [m]; suction trap [mf]; sweeping [mf]; tile trap [mf]

####### Type.

Massachusetts, Boston; Connecticut, New Haven

####### Etymology.

Latin, season collected

####### Collection.

MSU, TAMU, TMM

####### Note.

Males were collected in a suction trap from 10:00 to 12:00 hours and 14:00 to 16:00 hours.

###### Erigone barrowsi

Taxon classificationAnimaliaAraneaeLinyphiidae

Crosby & Bishop, 1928

Erigone barrowsi Agnew et al. 1985: 3; Dean and Sterling 1990: 404; Jackman 1997: 164; Jones 1936: 70; Vogel 1970b: 11; Young and Edwards 1990: 18 [Crosby and Bishop 1928: 21, mf, desc. (figs 21–25)]

####### Distribution.

Coleman, Dallas, Erath, Walker

####### Locality.

Horne Ranch, Ellis Prison Unit

####### Time of activity.

Male (July, September); female (August)

####### Habitat.

(crops: peanuts)

####### Method.

pitfall trap [m]; suction trap [f]

####### Type.

Florida, Apalachicola

####### Etymology.

Person (collector, W. M. Barrows)

####### Collection.

TAMU

###### Erigone canthognatha

Taxon classificationAnimaliaAraneaeLinyphiidae

Chamberlin & Ivie, 1935

Erigone canthognatha [Chamberlin and Ivie 1935b: 13, m, desc. (pl. 7, figs 54–56)]

####### Distribution.

Wichita

####### Type.

Utah, Moab

[female unknown]

####### Etymology.

Greek, jaw edge

####### Collection.

MSU

###### Erigone denticulata

Taxon classificationAnimaliaAraneaeLinyphiidae

Chamberlin & Ivie, 1939

Erigone denticulata Cokendolpher et al. 2007: 56, mf, desc. (figs 7, 9, 17–23); Cokendolpher et al. 2008: 23 (photo 18–9, fig. 8)

####### Distribution.

Briscoe, Lubbock, Swisher

####### Time of activity.

Male (June); female (June, September)

####### Habitat.

(littoral: playa); (structures: greenhouse next to pond)

####### Type.

Utah, Mirror Lake

####### Etymology.

Latin, teeth

####### Collection.

TTU

###### Erigone dentigera

Taxon classificationAnimaliaAraneaeLinyphiidae

O. P.-Cambridge, 1874

Erigone dentigera Breene et al. 1993c: 17, 47, 108, m (figs 169A-C); Dean and Sterling 1990: 404; Dean et al. 1982: 254; Jackman 1997: 164; Young and Edwards 1990: 18 [Crosby and Bishop 1928: 25, mf, desc. (figs 38–41)]

####### Distribution.

Walker

####### Locality.

Ellis Prison Unit

####### Time of activity.

Male (May – June, August)

####### Habitat.

(crops: cotton)

####### Method.

D-Vac suction [m]; pitfall trap [m]; suction trap [m]

####### Type.

Massachusetts, Beverly

####### Etymology.

Latin, tooth-like spine on palp

####### Collection.

TAMU

###### Erigone dentosa

Taxon classificationAnimaliaAraneaeLinyphiidae

O. P.-Cambridge, 1894

Erigone dentosa [Crosby and Bishop 1928: 27, mf, desc. (figs 42–45)]

####### Distribution.

Lubbock

####### Type.

Guatemala, Antigua

####### Etymology.

Latin, teeth on face of chelicerae

####### Collection.

MSU

###### Erigone personata

Taxon classificationAnimaliaAraneaeLinyphiidae

Gertsch & Davis, 1936

Erigone personata Bonnet 1956: 1771; Buckle et al. 2001: 117; Gertsch and Davis 1936: 1, m, desc. (figs 1–2); Jackman 1997: 164; Roewer 1942: 727; Roth 1988: 8; Vogel 1970b: 11

####### Distribution.

Llano

####### Time of activity.

Male (December)

####### Type.

Texas (male, Llano Co., Llano, December 24, 1935, L. I. Davis, holotype, AMNH)

[female unknown]

####### Etymology.

Latin, of a person

##### Genus *Eulaira* Chamberlin & Ivie, 1933

###### Eulaira suspecta

Taxon classificationAnimaliaAraneaeLinyphiidae

Gertsch & Mulaik, 1936

Eulaira suspecta Bonnet 1956: 1812; Buckle et al. 2001: 118; Chamberlin and Ivie 1945b: 10; Gertsch and Mulaik 1936b: 1, mf, desc. (figs 1–3); Jackman 1997: 164; Reddell 1970: 406; Roewer 1942: 728; Roth 1988: 39; Vogel 1970b: 11

####### Distribution.

Hidalgo, Val Verde

####### Caves.

**Val Verde** (Four-Mile Cave)

####### Time of activity.

Male (February); female (February)

####### Habitat.

(landscape features: cave)

####### Type.

Texas (male, Hidalgo Co., 7 miles E Edinburg, February 17, 1935, S. Mulaik, holotype, AMNH)

####### Etymology.

Latin, female paratype eyes abnormal

####### Collection.

TMM

##### Genus *Floricomus* Crosby & Bishop, 1925

###### Floricomus mulaiki

Taxon classificationAnimaliaAraneaeLinyphiidae

Gertsch & Davis, 1936

Floricomus mulaiki Bonnet 1957: 1912; Buckle et al. 2001: 119; Gertsch and Davis 1936: 4, mf, desc. (figs 28–31); Jackman 1997: 164; Roewer 1942: 610; Roth 1988: 9; Vogel 1970b: 11

####### Distribution.

Cameron

####### Time of activity.

Male (May); female (May)

####### Type.

Texas (male, Cameron Co., May 1–2, 1936, [L. I.] Davis, holotype, AMNH)

####### Etymology.

Person (collector of many species of Texas spiders, Stanley Mulaik)

###### Floricomus ornatulus

Taxon classificationAnimaliaAraneaeLinyphiidae

Gertsch & Ivie, 1936

Floricomus ornatulus Bonnet 1957: 1912; Buckle et al. 2001: 119; Gertsch and Ivie 1936: 13, mf, desc. (figs 16–18); Jackman 1997: 164; Roewer 1942: 610; Roth 1988: 9; Vogel 1970b: 11

####### Distribution.

Cameron, Hidalgo

####### Time of activity.

Male (January – February); female (January – February, November)

####### Type.

Texas (male, Hidalgo Co., Edinburg, January 10–20, 1935, S. Mulaik, holotype, AMNH)

####### Etymology.

Latin, ornate

###### Floricomus rostratus

Taxon classificationAnimaliaAraneaeLinyphiidae

(Emerton, 1882)

Floricomus rostratus Dean and Sterling 1990: 404; Jackman 1997: 164 [Bishop and Crosby 1935: 40, mf, desc. (figs 29–34)]

####### Distribution.

Walker

####### Locality.

Ellis Prison Unit

####### Time of activity.

Male (May – June); female (May – June)

####### Method.

suction trap [mf]

####### Type.

Massachusetts, Walthom and Watertown

####### Etymology.

Latin, horn on male extends forward

####### Collection.

TAMU

##### Genus *Florinda* O. P.-Cambridge, 1896

###### Florinda coccinea

Taxon classificationAnimaliaAraneaeLinyphiidae

(Hentz, 1850)

Florinda coccinea Brown 1974: 234; Buckle et al. 2001: 119; Gertsch and Davis 1946: 5 [T]; Jackman 1997: 164; Kaston 1953: 209, desc. (fig. 517); Kaston 1972: 129, desc. (fig. 290); Kaston 1978: 124, desc. (fig. 310); Roth 1988: 39; Roth 1994: 111
Frontinella coccinea Hentz, 1850; Blauvelt 1936: 149, mf (figs 96–100)

####### Distribution.

Fannin, Harris, Nacogdoches, Nueces, San Patricio (imm.), Walker

####### Locality.

Ellis Prison Unit, Welder Wildlife Refuge

####### Time of activity.

Male (May – June); female (May)

####### Habitat.

(plants: in bush); (soil/woodland: hackberry matte)

####### Method.

suction trap [imm.]

####### Type.

North Carolina

####### Etymology.

Latin, scarlet

####### Collection.

MSU, TAMU

##### Genus *Frontinella* F. O. P.-Cambridge, 1902

###### Frontinella communis

Taxon classificationAnimaliaAraneaeLinyphiidae

(Hentz, 1850)

Frontinella communis Blauvelt 1936: 145, mf, desc. (figs 90–95); Brown 1974: 234; Platnick 1998: 355 [S] [Kaston 1948: 120, mf, desc. (figs 254–260)]
Frontinella pyramitela (Walckenaer, 1841); Agnew et al. 1985: 6, 11; Breene et al. 1993c: 17, 47, 111, mf (figs 178A-C); Dean and Sterling 1990: 404; Dean et al. 1982: 254; Jackman 1997: 63, desc., 164 (photo 19a); Nyffeler and Sterling 1994: 1295, 1298; Nyffeler et al. 1988b: 215; Taber and Fleenor 2003: 230; Young and Edwards 1990: 18

####### Distribution.

Archer, Bastrop, Bexar, Brazos, Brown, Erath, Galveston, Harris, Jack, Medina, Montgomery, Nacogdoches, Presidio, Travis, Walker, Wichita, Young

####### Locality.

Big Bend Ranch State Park, Buescher State Park, Ellis Prison Unit, Jones State Forest, Lick Creek Park, Sam Houston National Forest, Stubblefield Lake, Zilker Park

####### Caves.

**Medina** (Ney Cave)

####### Time of activity.

Male (March – May, July – October); female (March – May, July – November)

####### Habitat.

(crops: cotton); (landscape features: cave); (littoral: sedge meadow); (plants: cactus, vegetation); (soil/woodland: palm grove, juniper, woods, *Juniperus ashei*, *Quercus buckleyi*); (web: in web)

####### Method.

Beating [m]; suction trap [mf]; sweeping [mf]

####### Type.

United States

####### Etymology.

Latin, common

####### Collection.

DMNS, MSU, NMSU, TAMU, TMM

##### Genus *Grammonota* Emerton, 1882

###### Grammonota inornata

Taxon classificationAnimaliaAraneaeLinyphiidae

Emerton, 1882

Grammonota inornata Buckle et al. 2001: 121; Dean and Sterling 1990: 402; Jackman 1997: 164 [Bishop and Crosby 1932: 397, mf, desc. (figs 9–13)]

####### Distribution.

Brazos

####### Time of activity.

Male (June)

####### Method.

suction trap [m]

####### Type.

Massachusetts, Saugus and Woods Hole; Connecticut, New Haven

####### Etymology.

Latin, unadorned

####### Collection.

TAMU

###### Grammonota maculata

Taxon classificationAnimaliaAraneaeLinyphiidae

Banks, 1896

Grammonota maculata Banks 1896: 68, mf, desc.; Bishop and Crosby 1932: 401, mf, desc. (figs 24–26); Buckle et al. 2001: 121; Jackman 1997: 164; Petrunkevitch 1911: 241; Roth 1988: 10

####### Distribution.

Brazos, Harris

####### Time of activity.

Male (December); female (December)

####### Type.

Florida, Runneymeade; Texas, Brazos Co.

####### Etymology.

Latin, spots around spinnerets

###### Grammonota nigrifrons

Taxon classificationAnimaliaAraneaeLinyphiidae

Gertsch & Mulaik, 1936

Grammonota nigrifrons Bonnet 1957: 2055; Buckle et al. 2001: 121; Gertsch and Mulaik 1936b: 2, m, desc. (figs 8–9); Jackman 1997: 164; Roewer 1942: 731; Roth 1988: 10; Vogel 1970b: 11

####### Distribution.

Bexar, Cameron

####### Time of activity.

Male (December)

####### Type.

Texas (male, Cameron Co., December 1934, L. I. Davis, holotype, AMNH)

[female unknown]

####### Etymology.

Latin, black hairs

###### Grammonota suspiciosa

Taxon classificationAnimaliaAraneaeLinyphiidae

Gertsch & Mulaik, 1936

Grammonota suspiciosa Bonnet 1957: 2056; Buckle et al. 2001: 122; Gertsch and Mulaik 1936b: 2, m, desc. (figs 6–7); Jackman 1997: 164; Roewer 1942: 731; Roth 1988: 10; Vogel 1970b: 11

####### Distribution.

Terrell

####### Time of activity.

Male (July)

####### Type.

Texas (male, Terrell Co., Sanderson, July 4, 1934, S. Mulaik, holotype, AMNH)

[female unknown]

####### Etymology.

Latin, mistrustful

###### Grammonota texana

Taxon classificationAnimaliaAraneaeLinyphiidae

(Banks, 1899)

Grammonota texana Agnew et al. 1985: 3; Bishop and Crosby 1932: 409 [T], mf, desc. (figs 43–47); Breene 1988: 15, 17, 23–26, 35; Breene et al. 1988: 180–181; Breene et al. 1989: 162; Breene et al. 1993c: 17, 47, 108, mf (figs 171A-D); Buckle et al. 2001: 122; Bumroongsook et al. 1992: 18; Calixto et al. 2013: 182, 189; Dean and Eger 1986: 141; Dean and Sterling 1987: 6; Dean and Sterling 1990: 402; Dean et al. 1982: 254; Dean et al. 1987: 268; Dean et al. 1988: 286; Dondale 1959: 235; Jackman 1997: 164; Kagan 1942: 20; Kagan 1943: 258; Nyffeler et al. 1992c: 2; Pamanes-Guerrero 1975: 16, 34, 37, 41, 59, 63, 78, 81; Pfannenstiel 2008a: 204; Rapp 1984: 4; Roewer 1942: 731; Roth 1988: 10; Vogel 1970b: 11; Young and Edwards 1990: 18
Acartauchenius texana Banks, 1899; Banks 1899: 192, mf, desc.
Acartauchenius texanus Banks, 1899; Petrunkevitch 1911: 215

####### Distribution.

Atascosa, Bee, Bexar, Bowie, Brazos, Brooks, Burleson, Cameron, Camp, Collin, Colorado, Comanche, Delta, Erath, Fayette, Freestone, Galveston, Gillespie, Goliad, Harris, Hays, Hidalgo, Houston, Jim Wells, Kaufman, Kerr, Llano, Marion, McLennan, Nueces, Polk, Refugio, Robertson, San Patricio, Shelby, Stephens, Walker, Webb, Willacy, Williamson, Wood, Zapata

####### Locality.

Adriance Pecan Orchard, Attwater Prairie Chicken National Wildlife Refuge, Ellis Prison Unit, Holmes Pecan Orchard, Proctor Lake, Santa Ana National Wildlife Refuge, Stiles Farm Foundation, Texas A&M University Rangeland Area

####### Time of activity.

Male (February – October); female (February – October)

####### Habitat.

(crops: cotton, peanuts); (grass: grass, grassland, pasture); (orchard: pecan, pecan tree); (plants: bluebonnets, clover, croton, Indian paintbrush, miscellaneous vegetation, roadside vegetation, *Cassia* sp., *Monarda citriodora*); (structures: around house); (soil/woodland: post oak savanna with pasture, trees)

####### Method.

Beating [mf]; cardboard band [mf]; D-Vac suction [mf]; fogging [mf]; pitfall trap [mf]; ramp trap [mf]; suction trap [mf]; sweeping [mf]

####### Type.

Louisiana, Shreveport; Mississippi, Holly Springs; Texas, Brazos Co.

####### Etymology.

locality (state)

####### Collection.

MSU, TAMU

###### Grammonota vittata

Taxon classificationAnimaliaAraneaeLinyphiidae

Barrows, 1919

Grammonota vittata Dean and Sterling 1990: 404; Jackman 1997: 164 [Bishop and Crosby 1932: 412, mf, desc. (figs 50–52)]

####### Distribution.

Walker

####### Locality.

Ellis Prison Unit

####### Time of activity.

Male (June)

####### Method.

suction trap [m]

####### Type.

Ohio, Hebron

####### Etymology.

Latin, striped

####### Collection.

TAMU

##### Genus *Idionella* Banks, 1893

###### Idionella anomala

Taxon classificationAnimaliaAraneaeLinyphiidae

(Gertsch & Ivie, 1936)

Idionella anomala Ivie 1967: 128 [T]; Jackman 1997: 165; Roth 1988: 12
Ceraticelus anomalus Gertsch and Ivie, 1936; Bonnet 1956: 1003; Buckle et al. 2001: 106; Gertsch and Ivie 1936: 14, m, desc. (figs 14–15); Roewer 1942: 604 [Barnes 1953: 7, f, desc. (fig. 9)]
Ceraticelus anomalas Gertsch and Ivie, 1936; Vogel 1970b: 11

####### Distribution.

Hidalgo, Wichita

####### Time of activity.

Male (February)

####### Type.

Texas (male, Hidalgo Co., 7 miles E Edinburg, February 17, 1935, S. Mulaik, holotype, AMNH)

####### Etymology.

Greek, abnormal example

####### Collection.

MSU

###### Idionella deserta

Taxon classificationAnimaliaAraneaeLinyphiidae

(Gertsch & Ivie, 1936)

Idionella deserta Ivie 1967: 128 [T]; Jackman 1997: 165; Roth 1988: 12
Ceraticelus desertus Gertsch and Ivie, 1936; Bonnet 1956: 1004; Buckle et al. 2001: 106; Gertsch and Ivie 1936: 15, m, desc. (fig. 21); Roewer 1942: 605; Vogel 1970b: 11

####### Distribution.

Hidalgo

####### Time of activity.

Male (November)

####### Type.

Texas (male, Hidalgo Co., Edinburg, November 27, 1935, S. Mulaik, holotype, AMNH)

[female unknown]

####### Etymology.

Latin, solitary

###### Idionella formosa

Taxon classificationAnimaliaAraneaeLinyphiidae

(Banks, 1892)

Idionella formosa Ivie 1967: 128 [T]; Jackman 1997: 165
Ceraticelus formosus (Banks, 1892); Jones 1936: 69; Vogel 1970b: 11 [Crosby and Bishop 1925: 25, mf (figs 44–47)]

####### Distribution.

Dallas

####### Type.

New York

####### Etymology.

Latin, beautiful

###### Idionella sclerata

Taxon classificationAnimaliaAraneaeLinyphiidae

(Ivie & Barrows, 1935)

Idionella sclerata Calixto et al. 2013: 182, 186–187; Ivie 1967: 128 [T]; Jackman 1997: 165
Grammonota sclerata Ivie and Barrows, 1935; Buckle et al. 2001: 121; Dondale 1959: 236 [S], mf, desc. (fig. 17) [Ivie and Barrows 1935: 14, mf, desc. (figs 48–51)]
Grammonota confusa Gertsch and Mulaik, 1936; Bonnet 1957: 2054; Gertsch and Mulaik 1936b: 3, m, desc. (figs 4–5); Roewer 1942: 730; Vogel 1970b: 11
Ceratinopsis spp.; Dean and Eger 1986: 141 [part]

####### Distribution.

Brazos, Comanche, San Jacinto, San Patricio, Starr, Webb, Wichita

####### Locality.

Bill Haney Pecan Orchard

####### Time of activity.

Male (April – August, October); female (April – August)

####### Habitat.

(orchard: pecan); (plants: bluebonnets, Indian paintbrush); (soil/woodland: post oak savanna with pasture)

####### Method.

pitfall trap [mf]; sweeping [mf]

####### Type.

Florida, Fort Meyers

####### Etymology.

Greek, tough

####### Collection.

MSU, TAMU

##### Genus *Islandiana* Braendegaard, 1932

###### Islandiana flaveola

Taxon classificationAnimaliaAraneaeLinyphiidae

(Banks, 1892)

Islandiana flaveola Buckle et al. 2001: 126; Ivie 1965: 13, mf, desc. (figs 22–25); Jackman 1997: 165; Roth 1988: 12; Vogel 1970b: 11

####### Distribution.

Hartley

####### Time of activity.

Male (July); female (July)

####### Type.

New York, Ithaca, South Hill

####### Etymology.

Latin, yellow

###### Islandiana unicornis

Taxon classificationAnimaliaAraneaeLinyphiidae

Ivie, 1965

Islandiana unicornis Buckle et al. 2001: 126; Culver et al. 2003: 464; Draney and Buckle 2005: 142; Gertsch 1992: 78; Ivie 1965: 20, mf, desc. (figs 40–45); Jackman 1997: 165; Reddell 1994: 6; Roth 1988: 12; Vogel 1967: 74; Vogel 1970b: 11
Islandiana sp.; Reddell 1965: 172 [part]
Centromerus sp. nr latidens Emerton, 1882; Reddell 1965: 172 [part]

####### Distribution.

Childress, Wheeler

####### Caves.

**Childress** (Black Hand Cave); **Wheeler** (Big Mouth Cave)

####### Time of activity.

Male (May); female (May)

####### Habitat.

(landscape features: cave)

####### Type.

Texas (male, Childress Co., Black Hand Cave, May 1963, J. Reddell, B. Russell, holotype, AMNH)

####### Etymology.

Latin, hornlike, projection

####### Collection.

TMM

##### Genus *Jalapyphantes* Gertsch & Davis, 1946

###### Jalapyphantes puebla

Taxon classificationAnimaliaAraneaeLinyphiidae

Gertsch & Davis, 1946

Jalapyphantes puebla [Gertsch and Davis 1946: 8, f, desc. (fig. 16)]

####### Distribution.

Jeff Davis

####### Caves.

**Jeff Davis** (Bloys Camp Cave)

####### Habitat.

(landscape features: cave)

####### Type.

Mexico, Pueblo, Riofrio

[male unknown]

####### Etymology.

locality (state)

####### Collection.

TMM

##### Genus *Masoncus* Chamberlin, 1949

###### Masoncus conspectus

Taxon classificationAnimaliaAraneaeLinyphiidae

(Gertsch & Davis, 1936)

Masoncus conspectus Buckle et al. 2001: 131; Calixto et al. 2013: 182; Ivie 1967: 129 [S, T]; Jackman 1997: 165; Roth 1988: 13
Tapinocyba conspecta Gertsch and Davis, 1936; Bonnet 1959: 4242; Gertsch and Davis 1936: 4, m, desc. (figs 5–7); Roewer 1942: 702; Vogel 1970b: 12
Masoncus conspecta (Gertsch and Davis, 1936); Reddell 1965: 173; Vogel 1970b: 12
Masoncus nogales Chamberlin, 1948; Chamberlin 1948: 537, mf, desc. (figs 93–98); Vogel 1967: 75; Vogel 1970b: 12

####### Distribution.

Comanche, Culberson, Hidalgo, Llano, Tom Green, Val Verde

####### Locality.

Bill Haney Pecan Orchard

####### Caves.

**Culberson** (Plateau Cave); **Val Verde** (Popcorn Ball Cave)

####### Time of activity.

Male (May 25-June 1, June – July, December); female (June, December)

####### Habitat.

(landscape features: cave); (orchard: pecan)

####### Method.

pitfall trap [mf]

####### Type.

Texas (male, Llano Co., Llano, December 24, 1935, L. I. Davis, holotype, AMNH)

####### Etymology.

Latin, survey

####### Collection.

TAMU, TMM

##### Genus *Mermessus* O. P-Cambridge, 1899

###### Mermessus albulus

Taxon classificationAnimaliaAraneaeLinyphiidae

(Zorsch & Crosby, 1934)

Mermessus albulus Miller 2007: 255 [T]
Eperigone albula Zorsch and Crosby, 1934; Cokendolpher and Buckle 2004: 71 f, desc. (fig. 1); Reddell and Cokendolpher 2004: 87; Zorsch and Crosby, 1934: 245, mf, desc. (figs 1A–D)
Eperigone n. sp.; Cokendolpher and Reddell 2001b: 50

####### Distribution.

Bell, Bexar, Comal, Coryell, Hays, Travis, Williamson

####### Locality.

Camp Bullis, Fort Hood

####### Caves.

**Bell** ([all Fort Hood] Big Crevice, Figure 8 Cave, Fools Cave, Hidden Pit Cave, Keilman Cave, Peep in the Deep Cave, Poison Ivy Pit, Price Pit, Soldiers Cave, Viper Den Cave); **Bexar** (Bob Wire Cave, Cave No. 194, Eagles Nest Cave [Camp Bullis], Elm Water Hole Cave, Leon Hill Cave [Camp Bullis], Record Fire 1 Pit [Camp Bullis], Toad Cave, Up the Creek Cave [Camp Bullis]); **Comal** (Washington Cave); **Coryell** ([all Fort Hood] Copperhead Sink No. 2, Porter Cave, Rocket River C System [B. R.’s Secret Cave]); **Hays** (Wimberly Bat Cave); **Travis** (3-Holer Cave, District Park Cave, Moss Pit, No Rent Cave, Wade Sink); **Williamson** (Avery Ranch Cave, Beck Crevice Cave, Beck Horse Cave, Core Barrel Cave, Lobo’s Lair, Susana Cave, Testudo Cave, Texella Cave Karst Park, Venturi Cave)

####### Time of activity.

Male (January, March – June, August – October, December); female (January – June, August – September, November – December)

####### Habitat.

(landscape features: cave); (soil/woodland: leaf litter)

####### Method.

Berlese funnel [mf]

####### Type.

Louisiana, Tallulah

####### Etymology.

Latin, white

####### Collection.

TMM

###### Mermessus antraeus

Taxon classificationAnimaliaAraneaeLinyphiidae

(Crosby, 1926)

Mermessus antraeus Miller 2007: 255 [T]
Eperigone antrea (Crosby, 1926); Buckle et al. 2001: 113; Cokendolpher and Polyak 2004: 189; Jackman 1997: 164; Millidge 1987: 26, mf, desc. (figs 73–79); Reddell 1965: 172; Reddell 1970: 405; Smith and Reddell 1971: 21; Vogel 1970b: 11

####### Distribution.

Brewster, Culberson, Kimble

####### Caves.

**Brewster** (400-Foot Cave); **Culberson** (Border Cave, Cutoff Cave, Gyp Joint, New Cave, Olive’s Cave); **Kimble** (Fleming Bat Cave)

####### Habitat.

(landscape features: cave)

####### Type.

New Mexico, Carlsbad Cave

####### Etymology.

Greek, cavity

####### Collection.

TMM

###### Mermessus bryantae

Taxon classificationAnimaliaAraneaeLinyphiidae

(Ivie & Barrows, 1935)

Mermessus bryantae Miller 2007: 255 [T]
Eperigone bryantae Ivie and Barrows, 1935; Buckle et al. 2001: 113; Dean and Sterling 1990: 402, 404; Jackman 1997: 164; Millidge 1987: 17 [S], mf, desc. (figs 29–35)
Eperigone credula Gertsch and Davis, 1936; Bonnet 1956: 1707; Gertsch and Davis 1936: 2, m, desc. (figs 3–4); Roewer 1942: 717; Roth 1988: 7; Vogel 1970b: 11

####### Distribution.

Brazos, Burleson, Colorado, Dallas, Duval, Harris, Houston, Llano, Panola, Walker, Webb, Wichita

####### Locality.

5-Eagle Ranch, Attwater Prairie Chicken National Wildlife Refuge, Ellis Prison Unit, NK Ranch, Texas A&M University Rangeland Area

####### Time of activity.

Male (February, April – June, September – December); female (March – July, November)

####### Habitat.

(crops: cotton); (plants: Indian paintbrush)

####### Method.

pitfall trap [mf]; suction trap [mf]; sweeping [f]

####### Type.

Florida, Marco Island

####### Etymology.

Person (arachnologist)

####### Collection.

MSU, TAMU

###### Mermessus denticulatus

Taxon classificationAnimaliaAraneaeLinyphiidae

(Banks, 1898)

Mermessus denticulatus Calixto et al. 2013: 180, 182, 185, 187, 189–190; Cokendolpher et al. 2007: 56; Cokendolpher et al. 2008: 26; Miller 2007: 258 [S]
Eperigone eschatologica (Crosby, 1924); Breene et al. 1993c: 17, 47, 109, mf (figs 175A-D); Buckle et al. 2001: 114; Dean and Eger 1986: 141; Dean and Sterling 1990: 402, 404; Dean et al. 1988: 286; Irungu 2007: 31; Jackman 1997: 164; Millidge 1987: 37, mf, desc. (figs 132–136); Nyffeler et al. 1992c: 2
Eperigone sp.; Agnew et al. 1985: 3, 9; Young and Edwards 1990: 18

####### Distribution.

Widespread; Bee, Brazos, Brewster, Burleson, Cameron, Colorado, Comanche, Coryell, Dallas, Erath, Floyd, Frio, Gonzales, Hidalgo, Houston, Kimble, Kleberg, Knox, Leon, Lipscomb, Llano, Nueces, Potter, Robertson, San Patricio, San Saba, Victoria, Walker, Webb, Wichita, Williamson

####### Locality.

5-Eagle Ranch, Attwater Prairie Chicken National Wildlife Refuge, Bill Haney Pecan Orchard, Ellis Prison Unit, Holmes Pecan Orchard, NK Ranch, Palmetto State Park, Stiles Farm Foundation

####### Time of activity.

Male (January – December); female (January, March – October, November 30 – December 7)

####### Habitat.

(crops: cabbage, cotton, peanuts); (grass: grass, grassland, pasture); (landscape features: under rock); (littoral: near pond, playa); (orchard: pecan); (plants: bluebonnets, emergent plants, emergent vegetation, *Monarda citriodora*); (soil/woodland: brushy area, juniper, post oak savanna with pasture, under litter, woods); (structures: indoors)

####### Method.

D-Vac suction [mf]; fogging [mf]; pitfall trap [mf] (near pond [mf]); suction trap [mf]; sweeping [m]; tile trap [mf]

####### Type.

Mexico, Tepic

####### Etymology.

Latin, prominent tooth on mandibles

####### Collection.

MSU, TAMU, TTU

####### Note.

A male and female were collected in a suction trap 10:00 to 12:00 hours.

###### Mermessus fradeorum

Taxon classificationAnimaliaAraneaeLinyphiidae

(Berland, 1932)

Mermessus fradeorum Miller 2007: 255 [T]
Eperigone fradeorum Millidge, 1987 [Millidge 1987: 35 [S], mf, desc. (figs 124–131)]
Eperigone banksi (Ivie and Barrows, 1935); Rogers and Horner 1977: 523; Young and Edwards 1990: 18

####### Distribution.

Knox

####### Type.

Azores, Furnas, San Miguel

####### Etymology.

undetermined

####### Collection.

MSU

###### Mermessus maculatus

Taxon classificationAnimaliaAraneaeLinyphiidae

(Banks, 1892)

Mermessus maculatus Calixto et al. 2013: 182; Henderson 2007: 52, 54, 56, 60, 74, 77, 79, 83; Miller 2007: 256 [T]
Eperigone maculata (Banks, 1892); Buckle et al. 2001: 114; Cokendolpher and Reddell 2001b: 50; Dean and Sterling 1990: 402; Jackman 1997: 164; Millidge 1987: 30, mf, desc. (figs 106–111); Reddell 1965: 172; Reddell 1970: 405; Reddell and Cokendolpher 2004: 87; Reddell and Smith 1965: 20; Vogel 1970b: 11

####### Distribution.

Bell, Bexar, Brazoria, Brazos, Comal, Coryell, Edwards, Erath, Harris, Hays, Jasper, Kerr, Lampasas, Leon, Newton, Panola, Robertson, Val Verde

####### Locality.

Camp Bullis, Fort Hood, Holmes Pecan Orchard, Lick Creek Park, Texas A&M University Rangeland Area

####### Caves.

**Bell** (Keilman Cave [Fort Hood], Plasma Cave); **Bexar** (Backhole, Haz Mat Pit, Kamikazi Cricket Cave, Madla’s Cave, Madla’s Drop Cave, Persimmon Pit, Stevens Ranch Cave No. 1, Stone Oak Parkway Pit); **Comal** (Camp Bullis Bad Air Cave, Washington Cave); **Coryell** ([all Fort Hood] Chigiouxs’ Cave, Copperhead Sink No. 2, Plateau Cave No. 2, Porter Cave, Runoff Cave); **Edwards** (Devil’s Sinkhole); **Hays** (Ezell’s Cave); **Lampasas** (Enough Cave); **Val Verde** (H. T. Miers Cave)

####### Time of activity.

Male (February 15 – March 15, March – July, September, September 28-October 4, November); female (January – February, April – June, September – November)

####### Habitat.

(grass: short grass); (landscape features: cave); (littoral: sedge meadow); (orchard: pecan); (soil/woodland: leaf litter, post oak woodland, upland woods)

####### Method.

Berlese funnel [mf]; pitfall trap [mf]; suction trap [mf]

####### Type.

New York, Ithaca, Coy Glen

####### Etymology.

Latin, several pairs of transverse indistinct white spots on abdomen

####### Collection.

TAMU, TMM, TTU

###### Mermessus paulus

Taxon classificationAnimaliaAraneaeLinyphiidae

(Millidge, 1987)

Mermessus paulus Miller 2007: 256 [T]
Eperigone paula Millidge, 1987; Buckle et al. 2001: 114; Jackman 1997: 164; Millidge 1987: 38, f, desc. (figs 137–138)

####### Distribution.

Hidalgo

####### Time of activity.

Female (October)

####### Type.

Texas (female, Hidalgo Co., 5 miles E Rio Grande City, October 31, 1936, S. Mulaik, holotype, AMNH)

[male unknown]

####### Etymology.

adjective meaning small

###### Mermessus tibialis

Taxon classificationAnimaliaAraneaeLinyphiidae

(Millidge, 1987)

Mermessus tibialis Miller 2007: 256 [T]
Eperigone tibialis Millidge, 1987 [Millidge 1987: 21, mf, desc. (figs 49–52)]

####### Distribution.

Clay, Wichita

####### Type.

New Mexico, Sierra Co., San Fidel

####### Etymology.

Latin, prominent palpal tibia

####### Collection.

MSU

###### Mermessus tridentatus

Taxon classificationAnimaliaAraneaeLinyphiidae

(Emerton, 1882)

Mermessus tridentatus Miller 2007: 256 [T]
Eperigone tridentata (Emerton, 1882); Brown 1974: 234; Buckle et al. 2001: 114; Dean and Sterling 1990: 402, 404; Irungu 2007: 31; Jackman 1997: 164; Jones 1936: 69; Millidge 1987: 21, mf, desc. (figs 53–58); Vogel 1970b: 11

####### Distribution.

Brazos, Cherokee, Dallas, Harris, Hidalgo, Jefferson, Nacogdoches, Walker

####### Locality.

Ellis Prison Unit, Lake Striker

####### Time of activity.

Male (June, November); female (April – July, October)

####### Habitat.

(crops: cabbage); (structures: on wall in kitchen)

####### Method.

pitfall trap [m]; suction trap [mf]

####### Type.

Rhode Island, Providence; Connecticut, New Haven

####### Etymology.

Latin, palpal organ has three teeth

####### Collection.

TAMU

###### Mermessus trilobatus

Taxon classificationAnimaliaAraneaeLinyphiidae

(Emerton, 1882)

Mermessus trilobatus Miller 2007: 256 [T]
Eperigone trilobata (Emerton, 1882) [Millidge 1987: 8, mf, desc. (figs 1–8, 165, 167–168)]

####### Distribution.

Colorado

####### Locality.

Attwater Prairie Chicken National Wildlife Refuge

####### Time of activity.

Female (May)

####### Method.

pitfall trap [f]

####### Type.

Massachusetts, Cambridge; Connecticut, New Haven

####### Etymology.

Latin, palpal organ has three teeth

####### Collection.

TAMU

##### Genus *Neriene* Blackwall, 1833

###### Neriene radiata

Taxon classificationAnimaliaAraneaeLinyphiidae

(Walckenaer, 1841)

Neriene radiata Buckle et al. 2001: 133 [S, T]; Cokendolpher and Reddell 2001b: 52; Jackman 1997: 63, desc., 165; Roth 1988: 44; Young and Edwards 1990: 19 [Helsdingen 1969: 223, mf, desc. (figs 315–324)]
Linyphia marginata C. L. Koch, 1834; Blauvelt 1936: 110, mf, desc. (figs 26–31)
Prolinyphia marginata (C. L. Koch, 1834); Agnew et al. 1985: 3

####### Distribution.

Archer, Bastrop, Bell, Brazos, Clay, Comanche, Erath, Harrison, Hidalgo, Montague, Montgomery, Nacogdoches, Red River, Shelby, Travis, Tyler, Walker

####### Locality.

Bastrop State Park, Fort Hood, Jones State Forest, Lick Creek Park, Nabor’s Lake, Sam Houston National Forest, Stubblefield Lake

####### Caves.

**Bell** (Long Joint Sink [Fort Hood])

####### Time of activity.

Male (April – October); female (March – October)

####### Habitat.

(crops: peanuts); (landscape features: cave); (littoral: near water, sedge meadow); (plants: vegetation); (soil/woodland: bottomland forest, trees/shrubs, woods); (web: in web)

####### Method.

Beating [mf]; flight intercept trap [m]; pitfall trap [m]; sweeping [mf]

####### Type.

Georgia

####### Etymology.

Latin, radius of web

####### Collection.

DMNS, MSU, TAMU, TMM

##### Genus *Oaphantes* Chamberlin & Ivie, 1943

###### Oaphantes pallidulus

Taxon classificationAnimaliaAraneaeLinyphiidae

(Banks, 1904)

Oaphantes pallidulus [Chamberlin and Ivie 1943: 8, mf, desc. (figs 10–11)]

####### Distribution.

Wichita

####### Type.

California, Claremont

####### Etymology.

Latin, color

####### Collection.

MSU

##### Genus *Scylaceus* Bishop & Crosby, 1938

###### Scylaceus

Taxon classificationAnimaliaAraneaeLinyphiidae

sp.

Scylaceus Reddell 1965: 173

####### Distribution.

King

####### Caves.

**King** (River Styx Cave)

####### Habitat.

(landscape features: cave)

####### Collection.

TMM

##### Genus *Soulgas* Crosby & Bishop, 1936

###### Soulgas corticarius

Taxon classificationAnimaliaAraneaeLinyphiidae

(Emerton, 1909)

Soulgas corticarius Agnew et al. 1985: 6; Jackman 1997: 165; Roth 1985: B-22–5; Roth 1994: 115 [Paquin and Dupérré 2003: 121, mf (figs 1262–1264)]

####### Distribution.

Erath

####### Time of activity.

Male (December)

####### Method.

suction trap [m]

####### Type.

Massachusetts, Cambridge and Boston; Connecticut, New Haven; Rhode Island, Providence

####### Etymology.

Latin, covered with bark

####### Collection.

TAMU

##### Genus *Styloctetor* Simon, 1884

###### Styloctetor purpurescens

Taxon classificationAnimaliaAraneaeLinyphiidae

(Keyserling, 1886)

Styloctetor purpurescens Marusik and Tanasevitch 1998: 154 [T]
Ceratinopsis purpurescens Keyserling, 1886; Agnew et al. 1985: 6; Jackman 1997: 164 [Bishop and Crosby 1930: 25, mf, desc. (figs 31–35)]
Ceratinopsis sp. C; Agnew et al. 1985: 3 [misidentified]

####### Distribution.

Brazos, Erath, Kerr, Travis, Walker

####### Locality.

Ellis Prison Unit, Lick Creek Park

####### Time of activity.

Male (February – June); female (March – August)

####### Habitat.

(crops: cotton, peanuts); (objects: croton cage); (soil/woodland: bottomland forest, edge of woods, *Juniperus ashei*, *Quercus buckleyi*, *Quercus virginiana*, *Ulmus crassifolia*)

####### Method.

Beating [mf]; D-Vac suction [m]; flight intercept trap [m]; pitfall trap [m] (edge of woods [m]); suction trap [f]; sweeping [mf]

####### Type.

Washington D. C.

####### Etymology.

Latin, purple

####### Collection.

MSU, TAMU

##### Genus *Tapinocyba* Simon, 1884

###### Tapinocyba hortensis

Taxon classificationAnimaliaAraneaeLinyphiidae

(Emerton, 1924)

Tapinocyba hortensis Agnew et al. 1985: 6; Jackman 1997: 165 [Crosby and Bishop 1933: 119, m, desc. (figs 45–48)]

####### Distribution.

Erath

####### Time of activity.

Male (May)

####### Method.

pitfall trap [m]

####### Type.

Massachusetts, Holliston

####### Etymology.

Latin, garden

####### Collection.

TAMU

##### Genus *Tennesseellum* Petrunkevitch, 1925

###### Tennesseellum formicum

Taxon classificationAnimaliaAraneaeLinyphiidae

(Emerton, 1882)

Tennesseellum formicum Agnew et al. 1985: 3, 9; Breene et al. 1993c: 17, 47, 110, mf (figs 177A-C); Buckle et al. 2001: 147 [spelling]; Bumroongsook et al. 1992: 17; Dean and Eger 1986: 141; Dean and Sterling 1987: 6; Dean and Sterling 1990: 402, 404; Dupérré 2013: 174, mf, desc. (figs 15–20, 33, 42, 43, 570–579); Hormiga 1994: 69; Rogers and Horner 1977: 523; Young and Edwards 1990: 19
Tennesseellum formica (Emerton, 1882); Calixto et al. 2013: 180, 183, 186–187, 189; Cokendolpher et al. 2008: 9, 26 (photo 20); Irungu 2007: 31; Ivie 1969: 6 [T]; Jackman 1997: 64, desc., 165
Bathyphantes formica Emerton, 1882; Jones 1936: 69; Vogel 1970b: 12

####### Distribution.

Baylor, Blanco, Brazos, Burleson, Carson, Collin, Colorado, Comanche, Coryell, Dallam, Dallas, Erath, Hidalgo, Houston, Lubbock, Nueces, Robertson, San Patricio, Travis, Uvalde, Walker, Wichita, Williamson

####### Locality.

5-Eagle Ranch, Attwater Prairie Chicken National Wildlife Refuge, Bill Haney Pecan Orchard, Browning Ranch, Ellis Prison Unit, Holmes Pecan Orchard, NK Ranch, Pantex Lake (edge), Stiles Farm Foundation

####### Time of activity.

Male (January, March – December); female (March – December)

####### Habitat.

(crops: cabbage, cotton, guar, peanuts); (grass: grass); (orchard: pecan); (plants: bluebonnets, Indian paintbrush); (soil/woodland: post oak savanna with pasture)

####### Method.

cardboard band [mf]; D-Vac suction [m]; fogging [m]; pitfall trap [mf]; suction trap [mf]; sweeping [mf]; tile trap [m]

####### Type.

Connecticut, New Haven

####### Etymology.

Latin, referring to ants

####### Collection.

MSU, TAMU

##### Genus *Tenuiphantes* Saaristo & Tanasevitch, 1996

###### Tenuiphantes sabulosus

Taxon classificationAnimaliaAraneaeLinyphiidae

(Keyserling, 1886)

Tenuiphantes sabulosus Saaristo and Tanasevitch 2000: 264 [T]
Lepthyphantes sabulosa (Keyserling, 1886); Vogel 1970b: 12; Zorsch 1937: 890, mf desc. (figs 73–78)
Lepthyphantes sabulosus (Keyserling, 1886); Buckle et al. 2001: 128; Cokendolpher and Reddell 2001b: 50; Jackman 1997: 165; Roth 1988: 41

####### Distribution.

Bell, Brazos, Dallas

####### Locality.

Fort Hood, Lick Creek Park

####### Caves.

**Bell** (Treasure Cave [Fort Hood])

####### Time of activity.

Male (November); female (November)

####### Habitat.

(landscape features: cave, under rock); (soil/woodland: bottomland forest litter)

####### Method.

Berlese funnel [m]

####### Type.

Utah, Salt Lake

####### Etymology.

Latin, sandy

####### Collection.

TAMU

###### Tenuiphantes zebra

Taxon classificationAnimaliaAraneaeLinyphiidae

(Emerton, 1882)

Tenuiphantes zebra Saaristo and Tanasevitch 1996: 182 [T]
Lepthyphantes zebra (Emerton, 1882); Dean and Sterling 1990: 404; Jackman 1997: 165 [Zorsch 1937: 887, mf, desc. (figs 67–72)]

####### Distribution.

Walker, Williamson

####### Locality.

Ellis Prison Unit, Stiles Farm Foundation

####### Time of activity.

Female (May – June)

####### Habitat.

(crops: cotton)

####### Method.

pitfall trap [f]; suction trap [f]

####### Type.

Massachusetts, eastern; Connecticut, New Haven

####### Etymology.

Latin, gray stripes on side

####### Collection.

TAMU

##### Genus *Tutaibo* Chamberlin, 1916

###### Tutaibo anglicanus

Taxon classificationAnimaliaAraneaeLinyphiidae

(Hentz, 1850)

Tutaibo anglicanus Buckle et al. 2001: 148; Jackman 1997: 165; Millidge 1991: 165 [T, spelling], mf (figs 693–694); Roth 1994: 115
Ceratinopsis anglicana (Hentz, 1850); Bishop and Crosby 1930: 15, mf, desc. (figs 1–4); Bumroongsook et al. 1992: 17; Dean and Sterling 1987: 6; Dean and Sterling 1990: 402; Li 1990: 137, 142, 144; Liao et al. 1984: 410; Roth 1988: 4

####### Distribution.

Brazos, Burleson, Cameron, Colorado, Harris, Matagorda, Polk, Robertson, San Patricio, Travis, Victoria, Walker

####### Locality.

Adriance Pecan Orchard, Attwater Prairie Chicken National Wildlife Refuge, Brison Pecan Orchard, Ellis Prison Unit, Holmes Pecan Orchard, South Padre Island

####### Time of activity.

Male (May – August, October – December); female (March – August, October – December)

####### Habitat.

(crops: cotton); (grass: grassland); (orchard: pecan); (soil/woodland: trees, *Juniperus ashei*, *Quercus virginiana*, *Ulmus crassifolia*)

####### Method.

Beating [f]; fogging [mf]; pitfall trap [f]; suction trap [mf]; sweeping [f]

####### Type.

Alabama

####### Etymology.

Latin, anglican

####### Collection.

MSU, TAMU

##### Genus *Walckenaeria* Blackwall, 1833

###### Walckenaeria puella

Taxon classificationAnimaliaAraneaeLinyphiidae

Millidge, 1983

Walckenaeria puella Agnew et al. 1985: 3; Buckle et al. 2001: 151; Calixto et al. 2013: 183; Irungu 2007: 31; Jackman 1997: 165; Millidge 1983: 142, f, desc. (figs 108, 132); Roth 1988: 21; Young and Edwards 1990: 19

####### Distribution.

Coryell, Erath, Hidalgo, Jim Wells, Robertson, Williamson

####### Locality.

Holmes Pecan Orchard, Stiles Farm Foundation

####### Time of activity.

Female (March – August)

####### Habitat.

(crops: cabbage, cotton, peanuts); (orchard: pecan); (soil/woodland: on ground, post oak savanna with pasture)

####### Method.

pitfall trap [f]

####### Type.

Texas (female, Jim Wells Co., Alice, May 15–30, 1961, R. O. Albert, holotype, MCZ)

[male unknown]

####### Etymology.

Latin, girl

####### Collection.

MCZ, TAMU

###### Walckenaeria spiralis

Taxon classificationAnimaliaAraneaeLinyphiidae

(Emerton, 1882)

Walckenaeria spiralis Agnew et al. 1985: 3; Breene et al. 1993c: 18, 47, 110, mf (figs 176A-B); Buckle et al. 2001: 151; Calixto et al. 2013: 183; Dean and Eger 1986: 141; Dean and Sterling 1987: 6; Dean and Sterling 1990: 402, 404; Henderson 2007: 61, 77, 80, 83; Jackman 1997: 165; Young and Edwards 1990: 19 [Millidge 1983: 113, mf, desc. (figs 2, 4, 8–9, 14–16, 22–23, 28, 67, 109, 112)]

####### Distribution.

Brazos, Burleson, Colorado, Delta, Erath, Robertson, Tyler, Walker, Wichita

####### Locality.

Attwater Prairie Chicken National Wildlife Refuge, Big Thicket National Preserve, Ellis Prison Unit, Holmes Pecan Orchard, Lick Creek Park

####### Time of activity.

Male (January, March – November); female (April – September, November – December)

####### Habitat.

(crops: alfalfa, cotton, peanuts); (orchard: pecan); (plants: bluebonnets, Indian paintbrush); (soil/woodland: hardwood litter, post oak woodland)

####### Method.

D-Vac suction [f]; pitfall trap [mf]; suction trap [mf]; sweeping [mf]

####### Type.

Connecticut, New Haven

####### Etymology.

Latin, palpal organ very large, tube stiff, coiled in two large spirals

####### Collection.

MSU, TAMU

#### Family Liocranidae Simon, 1897

##### Genus *Neoanagraphis* Gertsch & Mulaik, 1936

###### Neoanagraphis chamberlini

Taxon classificationAnimaliaAraneaeLiocranidae

Gertsch & Mulaik, 1936

Neoanagraphis chamberlini Bradley 2013: 147; Broussard and Horner 2006: 254; Ramirez 2014: 367; Richman et al. 2011a: 47; Trevino 2014: 13; Ubick and Richman 2005b: 163; Vetter 2001: 4, mf, desc. (figs 2–5)

####### Distribution.

Brewster, Culberson, Hudspeth, Presidio, Webb

####### Locality.

Chihuahuan desert, Dalquest Research Site

####### Caves.

**Culberson** (Granado Cave)

####### Time of activity.

Male (September, “November/December”); female (August, “November/December”)

####### Habitat.

(landscape features: cave); (nest/prey: nest of *Cratageomys castanops*)

####### Method.

pitfall trap [mf]

####### Type.

New Mexico, White Sands

####### Etymology.

Person (arachnologist)

####### Collection.

MSU, TMM

#### Family Lycosidae Sundevall, 1833

**Note.** Species incorrectly reported from Texas

*Pardosa montgomeryi* Gertsch, 1934; Bonnet 1958: 3389; Gertsch 1934a: 24 [Edinburg, misidentified]; Gertsch and Wallace 1935: 3; Roewer 1955: 193; Vogel 1964: 15; Vogel 1970b: 13; Vogel 2004: 64 [type locality incorrect, should be New Mexico]

*Pardosa mulaiki* Gertsch, 1934; Bonnet 1958: 3394; Dondale and Redner 1986: 828 [type locality of Texas incorrect, actually Colorado]; Gertsch 1934a: 22 [type from Edinburg]; Roewer 1955: 193; Vogel 1970b: 13

*Pardosa prolifica* F. O. P.-Cambridge, 1902; Jones 1936: 69 [not in United States]

*Pirata piraticus* (Clerck, 1757); Jackman 1997: 165; Vogel 1970b: 14 [not in Texas]

*Pirata febriculosa* (Becker, 1881); Chamberlin 1908: 311; Petrunkevitch 1911: 576 [not in Texas]

*Sosippus mimus* Chamberlin, 1924; Comstock 1940: 639 [not in Texas]

##### Genus *Allocosa* Banks, 1900

###### Allocosa absoluta

Taxon classificationAnimaliaAraneaeLycosidae

(Gertsch, 1934)

Allocosa absoluta Agnew et al. 1985: 4; Breene et al. 1993c: 18, 47, 94, mf (figs 129A-C); Calixto et al. 2013: 183; Dondale and Redner 1983b: 943 [T], mf, desc. (figs 5–6, 38–41); Jackman 1997: 165; Young and Edwards 1990: 19
Arctosa absoluta Gertsch, 1934; Gertsch 1934d: 9, m, desc. (fig. 1); Vogel 1970b: 12
Arctosa floridiana (Banks, 1896); Gertsch and Wallace 1937: 3 [Texas records]
Arctosa floridana (Banks, 1896); Vogel 1970b: 12 [Texas records]

####### Distribution.

Eastern ½ Texas; Brazos, Burleson, Comanche, Erath, Hidalgo, Kenedy, Walker

####### Locality.

5-Eagle Ranch, Bentsen-Rio Grande Valley State Park, Bill Haney Pecan Orchard, Ellis Prison Unit, Kenedy Ranch, NK Ranch

####### Time of activity.

Male (April – June, August); female (March – July, October)

####### Habitat.

(crops: cotton, peanuts); (littoral: under rock by creek, edge of pond, near pond, sand dune area); (orchard: pecan); (structures: indoors)

####### Method.

pitfall trap [mf] (edge of pond [m], near pond [mf])

####### Type.

Texas (male, Hidalgo Co., Edinburg, no date, S. Mulaik, holotype, AMNH)

####### Etymology.

Latin, differs from several other species [*chamberlini*, *funerea*, *furtiva*]

####### Collection.

MSU, TAMU

###### Allocosa apora

Taxon classificationAnimaliaAraneaeLycosidae

(Gertsch, 1934)

Allocosa apora Dondale and Redner 1983b: 945, mf, desc. (figs 21, 67–71); Jackman 1997: 165

####### Distribution.

Hidalgo

####### Type.

Costa Rica, San Jose

####### Etymology.

Latin, hard to get

###### Allocosa floridiana

Taxon classificationAnimaliaAraneaeLycosidae

(Chamberlin, 1908)

Allocosa floridiana [Dondale and Redner 1983b: 944, mf, desc. (figs 7, 42–44)]

####### Distribution.

Burleson, Cameron

####### Locality.

Russell Farm

####### Time of activity.

Male (March, September); female (September)

####### Method.

Boll weevil pheromone trap [m]; pitfall trap [mf]

####### Type.

Florida, Punta Gorda

####### Etymology.

locality (state)

####### Collection.

NMSU, TAMU

###### Allocosa funerea

Taxon classificationAnimaliaAraneaeLycosidae

(Hentz, 1844)

Allocosa funerea Agnew et al. 1985: 7; Dondale and Redner 1983b: 938, mf, desc. (figs 1–4, 35–37); Dondale and Redner 1990: 233, mf, desc. (figs 343–349); Jackman 1997: 165

####### Distribution.

East, central and north-central Texas; Coryell, Erath, Wichita

####### Time of activity.

Male (May); female (May – June)

####### Habitat.

(littoral: near pond); (soil/woodland: sandy area); (structures: indoors); (soil/woodland: post oak savanna with pasture)

####### Method.

pitfall trap [mf] (in sand [m], near pond [f])

####### Type.

Alabama

####### Etymology.

Latin, funereal

####### Collection.

MSU, TAMU

###### Allocosa furtiva

Taxon classificationAnimaliaAraneaeLycosidae

(Gertsch, 1934)

Allocosa furtiva Dondale and Redner 1983b: 943, mf, desc. (figs 12–13, 56–59); Jackman 1997: 165

####### Distribution.

Hidalgo

####### Type.

Florida, Lake Co.

####### Etymology.

Latin, stealthy

###### Allocosa mulaiki

Taxon classificationAnimaliaAraneaeLycosidae

(Gertsch, 1934)

Allocosa mulaiki Dondale and Redner 1983b: 938 [T], mf desc. (figs 8–9, 45–50); Jackman 1997: 165
Arctosa mulaiki Gertsch, 1934; Bonnet 1955: 655; Gertsch 1934a: 10, mf, desc.; Roewer 1955: 231; Vogel 1970b: 12

####### Distribution.

Central, southeast, and south Texas; Hidalgo

####### Type.

Texas (female, Hidalgo Co., Edinburg, no date, S. Mulaik, holotype, AMNH)

####### Etymology.

Person (Female holotype, and male allotype from Edinburgh, Texas, collected by Mr. Stanley Mulaik for whom the species is named, Gertsch 1934a).

###### Allocosa noctuabunda

Taxon classificationAnimaliaAraneaeLycosidae

(Montgomery, 1904)

Allocosa noctuabunda Dondale and Redner 1983b: 947 [T], mf, desc. (figs 14–15, 60–61); Henderson 2007: 58–59, 77, 80, 83; Jackman 1997: 165
Trochosa noctuabunda Montgomery, 1904; Montgomery 1904: 301, mf, desc. (pl. 18, figs 9–10)
Arctosa noctuabunda (Montgomery, 1904); Gertsch 1934a: 7; Vogel 1970b: 12
Allocosa noctuabunda (Montgomery, 1904); Roewer 1955: 211 [S]
Allocosa degesta Chamberlin, 1904; Petrunkevitch 1911: 550

####### Distribution.

Brazos, Caldwell, Jeff Davis, Kerr, Llano, Travis

####### Locality.

Davis Mountains, Lick Creek Park, Raven Ranch

####### Time of activity.

Male (April – May); female (May)

####### Habitat.

(soil/woodland: disturbed habitat)

####### Method.

pitfall trap [mf]

####### Type.

Texas (male, female, Travis Co., Austin, no date, no collector, syntypes, AMNH)

####### Etymology.

Latin, traveling by night

####### Collection.

DMNS, TAMU

###### Allocosa pylora

Taxon classificationAnimaliaAraneaeLycosidae

Chamberlin, 1925

Allocosa pylora Chamberlin 1925: 226, m, desc.; Dondale and Redner 1983b: 942, mf desc. (figs 16–17, 62–63); Jackman 1997: 165

####### Distribution.

El Paso, Travis

####### Type.

Texas (male, El Paso Co., El Paso, no date, no collector, holotype, MCZ)

####### Etymology.

Greek, a gate or entrance keeper

###### Allocosa retenta

Taxon classificationAnimaliaAraneaeLycosidae

(Gertsch & Wallace, 1935)

Allocosa retenta Roewer 1955: 211 [T]; Yantis 2005: 196, 199
Lycosa retenta Gertsch and Wallace, 1935; Bonnet 1957: 2661; Gertsch 1939b: 26; Gertsch and Wallace 1935: 17, m, desc. (fig. 30); Milstead 1958: 445; Vogel 1970b: 13; Wallace 1942b: 8, m (figs 3–4)
Hogna retenta (Gertsch and Wallace, 1935); Jackman 1997: 165

####### Distribution.

Anderson, Brewster, Crosby, Culberson, Leon, Presidio, Terrell, Travis, Val Verde

####### Locality.

Black Gap Wildlife Management Area, Blackstone Ranch, Chisos Basin, Chisos Mountains, La Mota Mountains

####### Time of activity.

Male (June, September 27-October 6, November)

####### Habitat.

(nest/prey: stomach of *Cnemidophorus sacki*, stomach of *Cnemidophorus tessellatus*, stomach of *Cnemidophorus tigris*); (soil/woodland: pine woods [%: 69], post oak woods [%: 93])

####### Method.

5 gallon bucket trap [m]

####### Type.

Texas (male, Travis Co., Austin, no date, J. H. Montgomery, holotype, AMNH)

[female unknown]

####### Etymology.

Latin, hold back

####### Collection.

MSU, TAMU

##### Genus *Alopecosa* Simon, 1885

###### Alopecosa aculeata

Taxon classificationAnimaliaAraneaeLycosidae

(Clerck, 1757)

Alopecosa aculeata Richman et al. 2011a: 47 [Dondale and Redner 1979: 1038, mf, desc. (figs 1, 4–5, 17, 22–24, 26)]
Alopecosa aculeate (Clerck, 1757); Broussard and Horner 2006: 254

####### Distribution.

Brewster, Presidio, Sutton

####### Locality.

Chihuahuan desert, Dalquest Research Site

####### Method.

pitfall trap [mf]

####### Type.

Sweden

####### Etymology.

Latin, aculeate (pointed)

####### Collection.

MSU

###### Alopecosa kochi

Taxon classificationAnimaliaAraneaeLycosidae

(Keyserling, 1877)

Alopecosa kochi Bradley 2013: 149; Dondale and Redner 1979: 1039, mf, desc. (figs 2–3, 7–8, 25, 27–32); Jackman 1997: 165; Platnick 1989: 362 [spelling]; Roberts 2001: 49
Alopecosa kochii (Keyserling, 1877); Dondale and Redner 1990: 307, mf, desc. (figs 500–510)

####### Distribution.

Cameron, Potter

####### Locality.

Wildcat Bluff Nature Center

####### Type.

North America

####### Etymology.

Person (arachnologist)

##### Genus *Arctosa* C. L. Koch, 1847

###### Arctosa littoralis

Taxon classificationAnimaliaAraneaeLycosidae

(Hentz, 1844)

Arctosa littoralis Agnew et al. 1985: 4; Barron et al. 1999: 550; Dondale and Redner 1983a: 24 [S, T], mf, desc. (figs 65–74); Gertsch 1934a: 7; Henderson 2007: 55, 77, 80, 83; Jackman 1997: 86, desc., 165; Jones 1936: 69; Rapp 1984: 6; Vogel 1970b: 12; Young and Edwards 1990: 19
Trochosa cinerea (Fabricius, 1793); Montgomery 1904: 305, mf, desc. (pl. 20, figs 43)
Lycosa cinerea (Fabricius, 1793); Chamberlin 1908: 281, mf (pl. 20, figs 5–6)
Arctosa cinerea (Fabricius, 1793); Vogel 1970b: 12
Arctosa trifida F. O. P.-Cambridge, 1902; Gertsch and Wallace 1935: 5; Roewer 1955: 231; Vogel 1970b: 12

####### Distribution.

Widespread; Archer, Brazos, Brewster, Cameron, Coke, Comanche, Dallas, Erath, Galveston, Hidalgo, Hunt, Kerr, Randall, Travis, Uvalde, Wichita, Williamson

####### Locality.

Bentsen-Rio Grande Valley State Park, Chisos Pass, Lick Creek Park, Palo Duro Canyon State Park

####### Time of activity.

Male (March – May, July, October); female (April – August)

####### Habitat.

(crops: peanuts); (littoral: beach dune at night, creek bank, edge of pond, near [pond, water]); (nest/prey: feeding on *Cophosaurus texana*); (soil/woodland: post oak woodland, sandy area)

####### Method.

at night; pitfall trap [mf] (edge of pond [mf], near pond [m])

####### Type.

North Carolina

####### Etymology.

Latin, place, edge of river

####### Collection.

DMNS, MSU, TAMU

###### Arctosa minuta

Taxon classificationAnimaliaAraneaeLycosidae

F. O. P.-Cambridge, 1902

Arctosa minuta Dondale and Redner 1983a: 21, mf, desc. (figs 50–56); Jackman 1997: 165

####### Distribution.

South Texas

####### Type.

Guatemala

####### Etymology.

Latin, size

##### Genus *Camptocosa* Dondale, Jiménez & Nieto, 2005

###### Camptocosa parallela

Taxon classificationAnimaliaAraneaeLycosidae

(Banks, 1898)

Camptocosa parallela Dondale et al. 2005: 43 [T], mf, desc. (figs 1–3)
Schizocosa parallela (Banks, 1898); Milstead 1958: 445

####### Distribution.

Bexar, Brewster, Culberson, Presidio, Terrell, Travis, Val Verde

####### Locality.

Big Bend National Park, Blackstone Ranch, Chisos Basin, La Mota Mountains

####### Caves.

**Bexar** (Cave of the Bearded Tree, Cave of the Half-Snake); **Culberson** (Hully Gully Cave); **Travis** (Lunsford Cave); **Val Verde** (Wren Cave)

####### Time of activity.

Male (May); female (May, July – August)

####### Habitat.

(landscape features: cave); (nest/prey: stomach of *Cnemidophorus tessellatus*)

####### Type.

Mexico, Baja California Sur

####### Etymology.

Latin, stripe on each side of cephalothorax

####### Collection.

TMM

###### Camptocosa texana

Taxon classificationAnimaliaAraneaeLycosidae

Dondale, Jiménez & Nieto, 2005

Camptocosa texana Dondale et al. 2005: 44, m, desc. (fig. 4) [Slowik and Cushing 2008: 543, f, desc. (figs 1–3, 5)]

####### Distribution.

Culberson, Hidalgo, Kleberg

####### Caves.

**Culberson** (Hully Gully Cave)

####### Time of activity.

Male (April, April 30-May 7, July, August, September)

####### Habitat.

(grass: grass); (landscape features: cave)

####### Method.

pitfall trap [m]

####### Type.

Texas (male, Kleberg Co., 2 miles S Riviera, 14 April 1963, W. J. Gertsch and W. Ivie, holotype, AMNH)

####### Etymology.

locality (The specific epithet is derived from the name of the State of Texas, Dondale et al. 2005).

####### Collection.

TAMU

##### Genus *Geolycosa* Montgomery, 1904

###### Geolycosa fatifera

Taxon classificationAnimaliaAraneaeLycosidae

(Hentz, 1842)

Geolycosa fatifera Bonnet 1957: 1988; Jackman 1997: 165; Petrunkevitch 1911: 554; Vogel 1970b: 12 [Wallace 1942a: 9 [T], mf, desc. (figs 3–4, 33, 46–49, 75, 85, 96, 106)]
Lycosa fatifera (Hentz, 1842); Chamberlin 1908: 241, f, desc. (pl. 20, fig. 8)

####### Distribution.

Texas

####### Type.

Alabama

####### Etymology.

Latin, fatal

###### Geolycosa latifrons

Taxon classificationAnimaliaAraneaeLycosidae

Montgomery, 1904

Geolycosa latifrons Jackman 1997: 165; Montgomery 1904: 295, mf (pl. 19, figs 15–18); Vogel 1970b: 12; Wallace 1942a: 45, mf, desc. (figs 5–6, 36, 74)

####### Distribution.

Travis

####### Type.

Texas (male, female, Travis Co., no date, J. H. Montgomery, holotype, AMNH)

####### Etymology.

Latin, cephalothorax high at posterior eyes

####### Collection.

MCZ

###### Geolycosa missouriensis

Taxon classificationAnimaliaAraneaeLycosidae

(Banks, 1895)

Geolycosa missouriensis Dondale and Redner 1990: 30, mf, desc. (figs 12–14, 16); Jackman 1997: 165; Kaston 1972: 197, desc.; Kaston 1978: 187, desc.; Vogel 1970b: 13; Wallace 1942a: 13, mf, desc. (figs 1–2, 34, 50–51, 76, 86, 97, 107)

####### Distribution.

Brazos

####### Time of activity.

Female (May)

####### Type.

Missouri, Springfield

####### Etymology.

locality (state)

###### Geolycosa riograndae

Taxon classificationAnimaliaAraneaeLycosidae

Wallace, 1942

Geolycosa riograndae Agnew et al. 1985: 4; Jackman 1997: 165; Wallace 1942a: 49, mf, desc. (figs 15–16, 35, 52, 82, 92, 100, 110); Young and Edwards 1990: 19
Geolycosa riogrande Wallace, 1942; Vogel 1967: 101; Vogel 1970b: 13
Scaptocosa riograndae (Wallace, 1942); Roewer 1955: 293

####### Distribution.

Erath, Hidalgo, Zapata

####### Time of activity.

Male (September – October); female (November)

####### Habitat.

(crops: peanuts)

####### Type.

Texas (male, Hidalgo Co., Edinburg, October 1934, S. Mulaik, holotype, AMNH)

####### Etymology.

locality (river in Texas)

####### Collection.

TAMU

##### Genus *Gladicosa* Brady, 1987

###### Gladicosa euepigynata

Taxon classificationAnimaliaAraneaeLycosidae

(Montgomery, 1904)

Gladicosa euepigynata Brady 1987: 312 [T], mf, desc. (figs 5, 15–17, 43–46); Jackman 1997: 165
Lycosa euepigynata Montgomery, 1904; Banks 1910: 55; Gertsch 1934d: 8; Gertsch and Wallace 1935: 22 (figs 44–45); Montgomery 1904: 279, mf, desc. (figs 1–2); Vogel 1970b: 13
Hogna euepigynata (Montgomery, 1904); Roewer 1955: 258

####### Distribution.

Bandera, Hays, Kendall, Kerr, Tom Green, Travis

####### Locality.

Raven Ranch

####### Time of activity.

Male (January, December); female (April, December)

####### Habitat.

(littoral: under stone near water)

####### Type.

Texas (female, Travis Co., Austin, no date, T. H. Montgomery, holotype, AMNH)

####### Etymology.

Greek, copulatory

###### Gladicosa gulosa

Taxon classificationAnimaliaAraneaeLycosidae

(Walckenaer, 1837)

Gladicosa gulosa Brady 1987: 290 [T], mf, desc. (figs 4, 6–9, 35–36); Dondale and Redner 1990: 88, mf, desc. (figs 105–107); Jackman 1997: 165
Lycosa gulosa Walckenaer, 1837; Chamberlin 1908: 265, m, desc. (pl. 21, figs 6–7); Petrunkevitch 1911: 560; Vogel 1970b: 13
Alopecosa gulosa (Walckenaer, 1837); Vogel 1970b: 12

####### Distribution.

Dallas, Grayson, Jasper, Wichita

####### Time of activity.

Male (January, October); female (October, December)

####### Type.

North America

####### Etymology.

Latin, gluttonous

###### Gladicosa huberti

Taxon classificationAnimaliaAraneaeLycosidae

(Chamberlin, 1924)

Gladicosa huberti Yantis 2005: 200 [Brady 1987: 305, mf, desc. (figs 1, 18–20, 27–28)]

####### Distribution.

Walker

####### Time of activity.

Female (April)

####### Habitat.

(soil/woodland: pine woods [%: 86])

####### Method.

5 gallon bucket trap [f]

####### Type.

Louisiana, Talisheek

####### Etymology.

Person (collector, H. E. Hubert)

####### Collection.

TAMU

###### Gladicosa pulchra

Taxon classificationAnimaliaAraneaeLycosidae

(Keyserling, 1877)

Gladicosa pulchra Bradley 2013: 152; Brady 1987: 299 [S, T], mf, desc. (figs 3, 10–14, 37–42); Jackman 1997: 165; Yantis 2005: 66, 197, 200
Lycosa pulchra (Keyserling, 1877); Gertsch 1934d: 8; Gertsch and Wallace 1935: 21 (figs 38, 41); Jones 1936: 69; Roble 1986: 135; Vogel 1970b: 13
Scaptocosa pulchra (Keyserling, 1877); Roewer 1955: 293
Lycosa insopita Montgomery, 1904; Montgomery 1904: 280, mf, desc. (figs 3–4)

####### Distribution.

Anderson, Bandera, Brazos, Comal, Dallas, DeWitt, Grimes, Harris, Hays (not Hale), Houston, Kerr, Leon, Madison, Smith, Travis, Walker

####### Locality.

Raven Ranch, Tyler State Park

####### Time of activity.

Male (April, October – December); female (March – May, September – December)

####### Habitat.

(landscape features: under stone); (soil/woodland: pine woods [%: 60, 66, 69, 77, 83, 84], post oak woods [%: 44, 49, 56, 84, 91, 94])

####### Method.

5 gallon bucket trap [mf]

####### Type.

North America

####### Etymology.

Latin, beautiful

####### Collection.

TAMU

##### Genus *Hesperocosa* Gertsch & Wallace, 1937

###### Hesperocosa unica

Taxon classificationAnimaliaAraneaeLycosidae

(Gertsch & Wallace, 1935)

Hesperocosa unica Bradley 2013: 153; Comstock 1940: 653, desc.; Dondale 2005: 167, 169; Gertsch and Wallace 1937: 5 [T]; Jackman 1997: 165; Milstead 1958: 445; Roberts 2001: 49; Roth 1982: 28–3; Roth 1985: B-24–3, B-24–4; Roth 1994: 125; Vogel 1970b: 13
Schizocosa unica Gertsch and Wallace, 1935; Gertsch and Wallace 1935: 9, mf, desc. (figs 21–22); Vogel 1970b: 14

####### Distribution.

Jeff Davis, Potter, Presidio

####### Locality.

Wildcat Bluff Nature Center

####### Time of activity.

Female (July)

####### Habitat.

(nest/prey: stomach of *Cnemidophorus perplexus*, stomach of *Cnemidophorus sacki*)

####### Type.

New Mexico, Hope

####### Etymology.

Latin, unique

##### Genus *Hogna* Simon, 1885

###### Hogna antelucana

Taxon classificationAnimaliaAraneaeLycosidae

(Montgomery, 1904)

Hogna antelucana Breene et al. 1993c: 18, 47, 98, mf (figs 140A-C); Calixto et al. 2013: 183, 190; Cokendolpher et al. 2008: 9, 26 (photo 22–24, figs 9–10); Jackman 1997: 165; Roberts 2001: 49; Roewer 1955: 257 [S, T]
Lycosa antelucana Montgomery, 1904; Agnew et al. 1985: 4, 10–11; Banks 1910: 55; Brown 1974: 234; Gertsch and Wallace 1935: 13, mf, desc. (figs 25–26); Jones 1936: 69; Milstead 1958: 445; Montgomery 1904: 282, mf, desc. (figs 5–6); Petrunkevitch 1911: 554; Reddell 1970: 406; Vogel 1970b: 13; Young and Edwards 1990: 19
Lycosa apicata Banks, 1904; Banks 1904: 114; Chamberlin 1908: 232, f, desc. (pl. 17, figs 1–2); Comstock 1912: 629
Allocosa sp. nr georgicola (Walckenaer, 1837); Henderson 2007: 55, 80, 83 [misidentified]; Yantis 2005: 196 [misidentified]
Hogna helluo (Walckenaer, 1837); Henderson 2007: 57–58, 77, 80, 83 [misidentified]; Irungu 2007: 28, 31[misidentified]; Yantis 2005: 197, 200 [misidentified]
Hogna sp. nr helluo (Walckenaer, 1837); Henderson 2007: 61–63, 65, 77, 80, 83 [misidentified]
Hogna sp. nr lenta (Hentz, 1844); Yantis 2005: 197, 200 [misidentified]
Hogna sp. nr frondicola (Emerton, 1885); Henderson 2007: 55, 60, 65, 77, 80, 83 [misidentified]; Irungu 2007: 31 [misidentified]
Hogna sp. nr annexa (Chamberlin and Ivie, 1944); Henderson 2007: 67, 77, 80, 83 [misidentified]

####### Distribution.

Anderson, Archer, Baylor, Bexar, Brazos, Burleson, Calhoun, Cameron, Carson, Clay, Colorado, Comanche, Coryell, Dallas, Edwards, Erath, Floyd, Haskell, Hidalgo, Houston, Hunt, Jeff Davis, Kimble, Kleberg, Knox, Leon, Lubbock, Madison, Nacogdoches, Potter, Presidio, Randall, Robertson, San Patricio, Travis, Trinity, Val Verde, Walker, Washington, Wichita, Williamson

####### Locality.

Attwater Prairie Chicken National Wildlife Refuge, Bill Haney Pecan Orchard, Ellis Prison Unit, Holmes Pecan Orchard, Lick Creek Park, Palo Duro Canyon State Park, Pantex Lake, Stiles Farm Foundation, Welder Wildlife Refuge, Wildcat Bluff Nature Center

####### Caves.

**Edwards** (Punkin Cave); **Val Verde** (Fern Cave)

####### Time of activity.

Male (January, March – December); female (January – December)

####### Habitat.

(crops: cabbage, cotton, peanuts, soybean); (grass: grassland, pasture); (landscape features: cave); (littoral: near playa); (nest/prey: stomach of *Cnemidophorus sacki*); (orchard: pecan); (soil/woodland: disturbed habitat, hackberry woodland, juniper, pine woods [%: 69, 77, 80, 82, 88, 95, 97, 100], post oak savanna with grassland, post oak woodland, post oak woods [%: 92], upland woods, woods); (structures: indoors)

####### Method.

5 gallon bucket trap [mf]; pitfall trap [mf] (in woods [m], near pond [m], pasture [m], under oak [m])

####### Eggs/spiderlings.

Coryell [222 spiderlings]; Williamson [55, 74, 108, 108, 158, 193, 263, 429 spiderlings] [TAMU]

####### Type.

Texas (male, female, Travis Co., Austin, no date, no collector, holotype)

####### Etymology.

Latin, common in autumn, none in winter

####### Collection.

DMNS, JCC, MSU, TAMU, TMM

###### Hogna baltimoriana

Taxon classificationAnimaliaAraneaeLycosidae

(Keyserling, 1877)

Hogna baltimoriana Bradley 2013: 154; Platnick 2006 [S, T] [Dondale and Redner 1990: 47 [T], mf, desc. (figs 36–38)]
Geolycosa baltimoriana (Keyserling, 1877); Montgomery 1904: 297, mf, desc. (pl. 19, fig. 19)
Lycosa baltimoriana (Keyserling, 1877); Petrunkevitch 1911: 556
Lycosa lenta baltimoriana Keyserling, 1877; Chamberlin 1908: 246; Comstock 1912: 629
Lycosa benedicta Chamberlin, 1925; Chamberlin 1925: 227, mf, desc.; Vogel 1970b: 13
Hogna benedicta (Chamberlin, 1925); Jackman 1997: 165; Roewer 1955: 258

####### Distribution.

Dallas, Travis

####### Type.

North America

####### Etymology.

of city of Baltimore

###### Hogna carolinensis

Taxon classificationAnimaliaAraneaeLycosidae

(Walckenaer, 1805)

Hogna carolinensis Barron et al. 1999: 550; Broussard and Horner 2006: 254; Cokendolpher et al. 2008: 9, 29; Dondale and Redner 1990: 45, mf, desc. (figs 33–35); Jackman 1997: 87, desc., 165; Punzo 2003: 399; Punzo 2007: 66; Richman et al. 2011a: 47; Roberts 2001: 49; Roewer 1955: 257 [S, T]
Lycosa carolinensis Walckenaer, 1805; Agnew et al. 1985: 7; Bishop and Crosby 1926: 206; Bonnet 1957: 2605; Brown 1974: 234; Chamberlin 1908: 246, mf, desc. (pl. 21, figs 1–2); Kunath and Smith 1968: 37; Reddell 1965: 173; Vogel 1970b: 13
Geolycosa texana Montgomery, 1904; Montgomery 1904: 293, mf, desc. (pl. 18, figs 13–14); Pritchett 1904b: 860

####### Distribution.

North-central Texas; Brazos, Brewster, Burleson, Carson, Culberson, Edwards, Erath, Jasper, Potter, Presidio, Rusk, Terrell, Travis, Uvalde, Val Verde, Washington, Wichita, Williamson

####### Locality.

Big Bend National Park, Chihuahuan desert, Dalquest Research Site, Pantex Lake, Wildcat Bluff Nature Center

####### Caves.

**Edwards** (Devil’s Sinkhole); **Terrell** (Goode Cave); **Val Verde** (Fern Cave); **Williamson** (Little Lake Cave)

####### Time of activity.

Male (April, June – September); female (March – April, August, September 28-October 5, October)

####### Habitat.

(grass: grassland); (landscape features: cave); (littoral: near playa); (soil/woodland: burrow in rocky ground, post oak savanna with pasture)

####### Method.

pitfall trap [mf]

####### Eggs/spiderlings.

Brazos [245 spiderlings] [TAMU]

####### Type.

Carolina (of 1805)

####### Etymology.

locality (state)

####### Collection.

DMNS, MCZ, MSU, TAMU, TMM

###### Hogna coloradensis

Taxon classificationAnimaliaAraneaeLycosidae

(Banks, 1894)

Hogna coloradensis Jackman 1997: 165; Roewer 1955: 258 [T]; Slowik and Cushing 2007: 46, mf, desc. (figs 1–21)
Lycosa coloradensis Banks, 1894; Schoenly 1983: 793

####### Distribution.

El Paso, Pecos, Ward

####### Locality.

Monahans Sandhills State Park

####### Time of activity.

Male (July); female (July)

####### Type.

Colorado, Fort Collins

####### Etymology.

locality (state)

###### Hogna lenta

Taxon classificationAnimaliaAraneaeLycosidae

(Hentz, 1844)

Hogna lenta Calixto et al. 2013: 183; Platnick 2000 [T]
Lycosa lenta Hentz, 1844; Kaston 1978: 196, desc. (fig. 502); Vogel 1970b: 13; Wallace 1942b: 5, mf, desc. (figs 1–2, 18)
Lycosa lenta texana Montgomery, 1904; Banks 1910: 56
Isohogna lenta (Hentz, 1844); Jackman 1997: 165

####### Distribution.

Comanche, Travis

####### Locality.

Bill Haney Pecan Orchard

####### Time of activity.

Male (October)

####### Habitat.

(orchard: pecan)

####### Method.

pitfall trap [m]

####### Type.

North Carolina

####### Etymology.

Latin, slow

####### Collection.

TAMU

###### Hogna tigana

Taxon classificationAnimaliaAraneaeLycosidae

(Gertsch & Wallace, 1935)

Hogna tigana Platnick 2000 [T]
Lycosa tigana Gertsch and Wallace, 1935; Bonnet 1957: 2667; Gertsch and Wallace 1935: 14, m, desc. (fig. 32); Gertsch and Wallace 1937: 6, f, desc. (fig. 6); Vogel 1970b: 13; Wallace 1942b: 5, mf (figs 16, 17, 25)
Isohogna tigana (Gertsch and Wallace, 1935); Jackman 1997: 165; Roewer 1955: 263

####### Distribution.

Hidalgo, Kenedy, Nueces, Presidio, San Patricio, Starr, Webb, Williamson

####### Locality.

Big Bend Ranch State Park, Corpus Christi State Park, Kenedy Ranch, Stiles Farm Foundation

####### Time of activity.

Male (March – April, June, October, December); female (March – April, June – July, October, December)

####### Habitat.

(crops: cotton); (littoral: sand dune area)

####### Method.

pitfall trap [mf]

####### Eggs/spiderlings.

Williamson [28, 75, 85, 87 spiderlings] [TAMU]

####### Type.

Texas (male, Hidalgo Co., Edinburg, no date, S. Mulaik, holotype, AMNH)

####### Etymology.

Latin, a stalk

####### Collection.

NMSU, TAMU

###### Hogna watsoni

Taxon classificationAnimaliaAraneaeLycosidae

(Gertsch, 1934)

Hogna watsoni Roewer 1955: 259 [T]
Lycosa watsoni Gertsch, 1934; Rapp 1984: 6 [Gertsch 1934d: 6, f, desc. (fig. 7)]

####### Distribution.

Galveston (imm.)

####### Habitat.

(grass: grassy and shrub area); (soil/woodland: sandy area)

####### Type.

Georgia, Valdosta

[male unknown]

####### Etymology.

Person (collector, F. E. Watson)

##### Genus *Pardosa* C. L. Koch, 1847

###### Pardosa atlantica

Taxon classificationAnimaliaAraneaeLycosidae

Emerton, 1913

Pardosa atlantica Breene et al. 1993c: 18, 47, 95, m (fig. 133); Dondale and Redner 1984: 88, mf, desc. (fig. 17); Jackman 1997: 88, desc., 165; Nyffeler et al. 1987b: 1119; Vogel 2004: 75, mf, desc. (figs 50, 52)

####### Distribution.

East Texas; Brazos, Burleson, Houston

####### Locality.

Lick Creek Park

####### Time of activity.

Male (March, June – September); female (July – September)

####### Habitat.

(crops: cotton); (grass: pasture)

####### Method.

pitfall trap [mf]

####### Type.

New Jersey, Lakehurst

####### Etymology.

Greek, place, ocean

####### Collection.

TAMU

###### Pardosa delicatula

Taxon classificationAnimaliaAraneaeLycosidae

Gertsch & Wallace, 1935

Pardosa delicatula Agnew et al. 1985: 7; Armstrong and Richman 2007: 395; Bonnet 1958: 3365; Breene et al. 1993a: 169; Breene et al. 1993b: 647; Breene et al. 1993c: 18, 47, 96, mf (figs 135A-B); Brown 1974: 234; Calixto et al. 2013: 180, 183, 187; Dean and Sterling 1987: 6; Dean et al. 1982: 254; Dondale and Redner 1984: 77, mf, desc. (figs 3, 6, 45–47); Gertsch and Wallace 1935: 4, mf, desc. (figs 13, 17); Irungu 2007: 28, 31; Jackman 1997: 88, desc., 165; Roewer 1955: 189; Rogers and Horner 1977: 523; Vogel 1970b: 13; Vogel 2004: 76, mf, desc. (figs 45, 59); Young and Edwards 1990: 19

####### Distribution.

Widespread; Aransas, Archer, Bastrop, Baylor, Brazos, Brewster, Burleson, Caldwell, Cameron, Colorado, Comanche, Coryell, Dickens, Erath, Hidalgo, Houston, Hunt, Jefferson, Kenedy, Knox, Nueces, Robertson, San Saba, Travis, Walker, Wichita, Williamson

####### Locality.

5-Eagle Ranch, Attwater Prairie Chicken National Wildlife Refuge, Bentsen-Rio Grande Valley State Park, Bill Haney Pecan Orchard, Ellis Prison Unit, Holmes Pecan Orchard, Kenedy Ranch, Lick Creek Park, NK Ranch, Russell Farm

####### Time of activity.

Male (February – December); female (February – December)

####### Habitat.

(crops: cabbage, cotton, guar, soybean, sugarcane); (grass: grass, in grass by house, pasture); (littoral: on water in ditch by cotton, sand dune area, sand dune under live oak); (orchard: citrus, pecan); (plants: bluebonnets, vegetation); (soil/woodland: ground, post oak savanna with pasture, trees/shrubs); (structures: around house)

####### Method.

Beating [f]; boll weevil pheromone trap [mf]; fogging [mf]; pitfall trap [mf]; yellow pan trap [m]; sweeping [f]

####### Eggs/spiderlings.

Walker [28 spiderlings] [TAMU]

####### Type.

Texas (male, Hidalgo Co., Edinburg, no date, S. Mulaik, holotype, AMNH)

####### Etymology.

Latin, delicate

####### Collection.

DMNS, MSU, NMSU, TAMU

###### Pardosa distincta

Taxon classificationAnimaliaAraneaeLycosidae

(Blackwall, 1846)

Pardosa distincta Rapp 1984: 6; Woods and Harrel 1976: 43; Young and Edwards 1990: 19 [Vogel 1964: 10, mf (figs 5–6, 17, 20)]

####### Distribution.

Galveston, Jeff Davis, Jefferson

####### Habitat.

(crops: rice); (littoral: salt marsh area)

####### Type.

Canada, Toronto

####### Etymology.

Latin, distinct

####### Collection.

MSU

###### Pardosa falcifera

Taxon classificationAnimaliaAraneaeLycosidae

F. O. P.-Cambridge, 1902

Pardosa falcifera Agnew et al. 1985: 7; Jackman 1997: 165; Roberts 2001: 49; Vogel 1970a: 15, 20–21, mf, desc. (figs 62–68, 70, 73–74, 91); Vogel 1970b: 13; Vogel 2004: 67, mf, desc. (figs 24, 26)

####### Distribution.

Comanche, Dallas, Jeff Davis, Kerr, Llano, Lubbock, Potter, Presidio, Reeves, Somervell, Travis, Uvalde, Williamson

####### Locality.

Big Bend Ranch State Park, Davis Mountains Resort, Proctor Lake, Raven Ranch, Wildcat Bluff Nature Center

####### Time of activity.

Male (February – April, June – August, December); female (March – April, June – August, October, December)

####### Habitat.

(littoral: along shore, on ground under falls)

####### Method.

yellow pan trap [mf]

####### Type.

Mexico, Guerrero, Omilteme

####### Etymology.

Latin, referring to a sickle

####### Collection.

DMNS, JCC, NMSU, TAMU

###### Pardosa hamifera

Taxon classificationAnimaliaAraneaeLycosidae

F. O. P.-Cambridge, 1902

Pardosa hamifera [Dondale and Redner 1984: 79, mf, desc. (figs 4, 7, 48–49)]

####### Distribution.

Hidalgo

####### Type.

Guatemala

####### Etymology.

Latin, a hook

####### Collection.

DMNS

###### Pardosa lapidicina

Taxon classificationAnimaliaAraneaeLycosidae

Emerton, 1885

Pardosa lapidicina Chamberlin 1908: 195, mf, desc. (pl. 14, figs 7–8); Kaston 1953: 144, desc. (fig. 362); Kaston 1972: 457, desc. (fig. 201); Kaston 1978: 191, desc. (fig. 489) [Barnes 1959: 4, mf, desc. (figs 6–10)]

####### Distribution.

Blanco, Brazos, Brown, Burnet, Clay, Comal, Montague, Travis, Wichita, Williamson

####### Time of activity.

Male (March, November); female (May, October)

####### Type.

Massachusetts and Connecticut

####### Etymology.

Latin, stone trace

####### Collection.

DMNS, MSU, TAMU

###### Pardosa littoralis

Taxon classificationAnimaliaAraneaeLycosidae

Banks, 1896

Pardosa littoralis Dondale and Redner 1984: 91, mf, desc. (figs 22, 25, 60–61); Dondale and Redner 1990: 162, mf, desc. (figs 206–209); Jackman 1997: 165; Vogel 2004: 77, mf, desc. (figs 44, 55)

####### Distribution.

Southeast Texas; Nueces

####### Time of activity.

Male (April); female (April)

####### Type.

New York, Long Island, Mill Neck

####### Etymology.

Latin, place, edge of river

####### Collection.

MSU

###### Pardosa mercurialis

Taxon classificationAnimaliaAraneaeLycosidae

Montgomery, 1904

Pardosa mercurialis Agnew et al. 1985: 4, 10; Banks 1910: 59; Barnes 1959: 5 [S], mf, desc. (figs 11–15); Gertsch 1934a: 19 [includes most of the previous records of lapidicina]; Jackman 1997: 165; Jones 1936: 69; Montgomery 1904: 270, mf, desc. (pl. 19, figs 20–21); Pritchett 1904b: 860; Roberts 2001: 49; Roewer 1955: 189; Vogel 1970b: 13; Vogel 2004: 71, mf, desc. (figs 35, 41); Young and Edwards 1990: 19
Pardosa lapidicina Emerton, 1885; Chamberlin 1908: 195; Petrunkevitch 1911: 571 [Texas records]
Pardosa texana Banks, 1904; Banks 1904: 115, f, desc.; Banks 1910: 60

####### Distribution.

Widespread; Brazos, Comanche, Coryell, Dallas, Erath, Hardeman, Jack, Kerr, Montague, Palo Pinto, Potter, San Patricio, Travis, Webb, Wichita, Williamson, Zapata

####### Locality.

Proctor Lake, Texas A&M University Rangeland Area, Wildcat Bluff Nature Center

####### Time of activity.

Male (April – August); female (March – August, October – November)

####### Habitat.

(crops: peanuts); (soil/woodland: post oak savanna with pasture, sandy area); (structures: greenhouse)

####### Method.

pitfall trap [mf]

####### Eggs/spiderlings.

Erath [51, 92 spiderlings] [TAMU]

####### Type.

Texas (male, Travis Co., Austin, no date, no collector, holotype, AMNH)

####### Etymology.

of Mercury, mercurial

####### Collection.

DMNS, MSU, TAMU

###### Pardosa milvina

Taxon classificationAnimaliaAraneaeLycosidae

(Hentz, 1844)

Pardosa milvina Agnew et al. 1985: 7; Banks 1904: 115; Breene 1988: 35–36; Breene et al. 1989: 162; Breene et al. 1993c: 19, 47, 96, mf (figs 136A-C); Brown 1974: 235; Dean and Sterling 1990: 405; Dean et al. 1982: 255; Dondale and Redner 1984: 97 [S], mf, desc. (figs 30–32, 70–71); Dondale and Redner 1990: 165, mf, desc. (figs 201–205); Jackman 1997: 88, desc., 165; Vogel 2004: 77, mf, desc. (figs 49, 56); Woods and Harrel 1976: 43; Young and Edwards 1990: 19
Pardosa nigropalpis Emerton, 1885; Montgomery 1904: 275

####### Distribution.

Eastern ½ Texas; Archer, Brazos, Brewster, Burleson, Clay, Colorado, Coryell, Erath, Houston, Hunt, Jefferson, Lee, Nacogdoches, Nueces, San Saba, Travis, Walker, Wichita, Williamson

####### Locality.

Attwater Prairie Chicken National Wildlife Refuge, Ellis Prison Unit, Lick Creek Park, Stiles Farm Foundation, Texas A&M University Rangeland Area

####### Time of activity.

Male (February – September); female (February – October)

####### Habitat.

(crops: cotton, rice); (littoral: edge of pond, near pond, post oak savanna with pasture, sandy shore, sedge meadow)

####### Method.

pitfall trap [mf] (near pond [f]); suction trap [m]

####### Eggs/spiderlings.

Erath [40 spiderlings] [TAMU]

####### Type.

Alabama

####### Etymology.

Latin, rapacious

####### Collection.

DMNS, MSU, TAMU

###### Pardosa pauxilla

Taxon classificationAnimaliaAraneaeLycosidae

Montgomery, 1904

Pardosa pauxilla Agnew et al. 1985: 4, 9; Breene et al. 1993a: 169; Breene et al. 1993c: 19, 47, 96, mf (figs 134A-B); Calixto et al. 2013: 183, 186–187; Chamberlin 1908: 180, f, desc. (pl. 13, fig. 9); Cokendolpher et al. 2008: 29 (photo 25); Dean and Sterling 1987: 6; Dean et al. 1982: 255; Dean et al. 1987: 264; Dean et al. 1988: 285; Dondale and Redner 1984: 94, mf, desc. (figs 24, 27, 65–67); Gertsch and Wallace 1935: 4, mf (figs 11, 15); Irungu 2007: 28, 31; Jackman 1997: 165 (photo 22d); Jones 1936: 69; Milstead 1958: 445; Montgomery 1904: 268, mf, desc. (pl. 19, figs 22–23); Nyffeler et al. 1987b: 1121; Petrunkevitch 1911: 574; Rogers and Horner 1977: 523; Vogel 1970b: 13; Vogel 2004: 78, mf, desc. (figs 46, 58); Vogel and Durden 1972: 1; Young and Edwards 1990: 20
Pardosa uncatula F Cambridge, 1902; Gertsch 1934a: 20; Roewer 1955: 190; Vogel 1970b: 14 [Texas records]

####### Distribution.

Widespread; Archer, Bastrop, Baylor, Blanco, Brazos, Briscoe, Burleson, Caldwell, Cameron, Clay, Colorado, Comal, Comanche, Cooke, Coryell, Dallas, Erath, Frio, Guadalupe, Hale, Hidalgo, Hopkins, Houston, Kerr, Kleberg, Knox, Llano, Lubbock, Nueces, Palo Pinto, Robertson, San Saba, Terrell, Travis, Walker, Wichita, Williamson, Wilson

####### Locality.

5-Eagle Ranch, Attwater Prairie Chicken National Wildlife Refuge, Bill Haney Pecan Orchard, Blackstone Ranch, Browning Ranch, Ellis Prison Unit, Holmes Pecan Orchard, Lake Travis, Lick Creek Park, NK Ranch, Proctor Lake, Stiles Farm Foundation

####### Time of activity.

Male (February – October, December); female (January – October)

####### Habitat.

(crops: cabbage, cotton, guar, peanuts, soybean); (grass: grass, pasture, sandy-prairie grass); (littoral: edge of pond, near pond, playa, stream or pond margin); (nest/prey: mud dauber nest [f], stomach of *Cnemidophorus sacki*); (orchard: citrus, pecan); (plants: bluebonnets, miscellaneous vegetation); (soil/woodland: on ground, post oak savanna with pasture, sandy area, *Juniperus ashei*)

####### Method.

Fogging [f]; pitfall trap [mf] (edge of pond [mf], in sand [f], near pond [mf]); suction trap [mf]; sweeping [mf]

####### Eggs/spiderlings.

Erath [62 spiderlings] [TAMU]

####### Type.

Texas (male, Travis Co., Austin, no date, no collector, lectotype, AMNH)

####### Etymology.

Latin, near water

####### Collection.

DMNS, MSU, TAMU

###### Pardosa saxatilis

Taxon classificationAnimaliaAraneaeLycosidae

(Hentz, 1844)

Pardosa saxatilis Woods and Harrel 1976: 43; Young and Edwards 1990: 19 [Dondale and Redner 1984: 87, mf, desc. (figs 16, 19–21, 58–59)]

####### Distribution.

Colorado, Jefferson, Nueces, Orange

####### Locality.

Attwater Prairie Chicken National Wildlife Refuge

####### Time of activity.

Male (May 29-June 5, June); female (April – June)

####### Habitat.

(crops: rice)

####### Method.

pitfall trap [mf]

####### Type.

Alabama

####### Etymology.

Latin, living among rocks

####### Collection.

MSU, TAMU

###### Pardosa sierra

Taxon classificationAnimaliaAraneaeLycosidae

Banks, 1898

Pardosa sierra Barnes 1959: 14, mf, desc. (figs 34–41); Jackman 1997: 165; Vogel 1970b: 14; Vogel 2004: 72, mf, desc. (figs 32, 38)

####### Distribution.

West Texas

####### Type.

Mexico, Baja California, Sierra Laguna

####### Etymology.

locality (place)

###### Pardosa sternalis

Taxon classificationAnimaliaAraneaeLycosidae

(Thorell, 1877)

Pardosa sternalis Bonnet 1958: 3423; Bradley 2013: 158; Breene et al. 1993c: 19, 47, 95, mf (figs 132A-B); Cokendolpher et al. 2008: 29 (photos 26–27); Dean and Sterling 1987: 6; Gertsch 1939b: 26; Jackman 1997: 165; Jones 1936: 69; Knutson and Gilstrap 1989: 514; Vogel 1970a: 8, 16–18, mf, desc. (figs 1–5, 13–18, 22–27, 37, 47, 51, 78–80, 82, 87); Vogel 1970b: 14; Vogel 2004: 102, mf, desc. (figs 125, 127, 130)

####### Distribution.

Brewster, Briscoe, Castro, Culberson, Dallas, Floyd, Jeff Davis, Lipscomb, Lubbock, Presidio, Reeves

####### Locality.

Chisos Mountains

####### Time of activity.

Male (June – September); female (June – September)

####### Habitat.

(crops: corn, cotton); (littoral: playa)

####### Type.

Colorado, Boulder

####### Etymology.

Latin, referring to sternum

####### Collection.

DMNS, TAMU

###### Pardosa sura

Taxon classificationAnimaliaAraneaeLycosidae

Chamberlin & Ivie, 1941

Pardosa sura Correa-Ramírez et al. 2010: 545, mf, desc. (figs 4, 7)

####### Distribution.

Terrell

####### Time of activity.

Female (May)

####### Type.

California, Big Sur

####### Etymology.

locality (region)

###### Pardosa vadosa

Taxon classificationAnimaliaAraneaeLycosidae

Barnes, 1959

Pardosa vadosa Barnes 1959: 7, mf, desc. (figs 16–19); Jackman 1997: 165; Vogel 1967: 106; Vogel 1970b: 14; Vogel 2004: 72, mf, desc. (figs 31, 37)

####### Distribution.

Central and west Texas; Anderson, Brewster, Hudspeth, Jeff Davis, Llano, Travis

####### Locality.

Big Bend National Park, Davis Mountains Resort, Indio Mountain Research Station

####### Time of activity.

Male (April); female (April, December)

####### Method.

yellow pan trap [mf]

####### Type.

Arizona, Virgin Narrows

####### Etymology.

Latin, shallows in water

####### Collection.

DMNS, MSU, TAMU

###### Pardosa xerophila

Taxon classificationAnimaliaAraneaeLycosidae

Vogel, 1964

Pardosa xerophila [Vogel 2004: 66, mf, desc. (figs 17–18)]

####### Distribution.

Culberson

####### Locality.

Guadalupe Mountains National Park

####### Time of activity.

Male (June)

####### Type.

Arizona, White Mountain Reservoir

####### Etymology.

Greek, dry-loving

####### Collection.

NMSU

###### Pardosa zionis

Taxon classificationAnimaliaAraneaeLycosidae

Chamberlin & Ivie, 1942

Pardosa zionis Jackman 1997: 165; Vogel 1970a: 15, 22, mf, desc. (figs 69, 71–72, 75–76, 91); Vogel 1970b: 14; Vogel 2004: 68, mf, desc. (figs 25, 27)

####### Distribution.

Hays

####### Time of activity.

Male (March); female (March – April)

####### Habitat.

(littoral: near river south of springs)

####### Type.

Utah, Zion National Park

####### Etymology.

locality (Zion Park)

####### Collection.

TAMU

##### Genus *Pirata* Sundevall, 1833

###### Pirata alachuus

Taxon classificationAnimaliaAraneaeLycosidae

Gertsch & Wallace, 1935

Pirata alachuus Henderson 2007: 53, 57, 77, 80, 83; Yantis 2005: 198 [Wallace and Exline 1978: 82 [spelling], mf, desc. (figs 8, 169–174)]
Pirata alachua Gertsch and Wallace, 1935 [Gertsch and Wallace 1935: 9, mf, desc. (figs 34, 36)]

####### Distribution.

Brazos, Houston

####### Locality.

Lick Creek Park, Texas A&M University Rangeland Area

####### Time of activity.

Male (April, April 29-May 15, July); female (May – June)

####### Habitat.

(littoral: sedge meadow); (soil/woodland: pine woods [%: 88, 100], post oak woodland)

####### Method.

5 gallon bucket trap [f]; pitfall trap [mf]

####### Type.

Florida, Alachua Co.

####### Etymology.

locality (This species was named for the county in which the type specimens were found, Wallace and Exline 1978).

####### Collection.

TAMU

###### Pirata apalacheus

Taxon classificationAnimaliaAraneaeLycosidae

Gertsch, 1940

Pirata apalacheus Henderson 2007: 28–29, 40, 52, 55, 57–59, 61, 63–64, 77, 80, 84; Yantis 2005: 66, 198, 201 [Gertsch 1940: 17, mf, desc. (figs 3–4); Wallace and Exline 1978: 18, mf, desc. (figs 20–25)]

####### Distribution.

Anderson, Brazos, Houston, Trinity

####### Locality.

Lick Creek Park

####### Time of activity.

Male (April – June); female (April 26-May 5, May – July)

####### Habitat.

(littoral: sedge meadow); (soil/woodland: disturbed habitat, old field, pine woods [%: 66, 80, 83, 88, 92, 95, 99, 100], post oak woodland)

####### Method.

5 gallon bucket trap [mf]; pitfall trap [mf]

####### Type.

Florida, Alachua Co.

####### Etymology.

locality in Florida

####### Collection.

TAMU

###### Pirata davisi

Taxon classificationAnimaliaAraneaeLycosidae

Wallace & Exline, 1978

Pirata davisi Reddell and Cokendolpher 2004: 88; Wallace and Exline 1978: 87, mf, desc. (figs 181–186)
Pirata sp.; Reddell 1970: 406

####### Distribution.

Bexar, Burleson, Hidalgo, Travis

####### Locality.

5-Eagle Ranch

####### Caves.

**Bexar** (Bullis Hole)

####### Time of activity.

Male (April – May, September 25 – October 2); female (April – May, May 28 – June 4, October)

####### Habitat.

(landscape features: cave)

####### Method.

pitfall trap [mf]

####### Type.

Mexico, Tamaulipas, San Fernando

####### Etymology.

Person (collector, L. I. Davis)

####### Collection.

MCZ, TAMU, TMM, TTU

####### Note.

specimen cited in Breene et al. 1993c: 19, 47, 95, mf (figs 131A-B) lost. Delete Jackman 1997: 89, 165.

###### Pirata felix

Taxon classificationAnimaliaAraneaeLycosidae

O. P.-Cambridge, 1898

Pirata felix Dean and Sterling 1990: 402; Jackman 1997: 165 [Wallace and Exline 1978: 55, mf, desc. (figs 105–106, 109)]

####### Distribution.

Brazos

####### Time of activity.

Female (May)

####### Method.

suction trap [f]

####### Type.

Mexico, Vera Cruz

####### Etymology.

Latin, productive

####### Collection.

FSCA

###### Pirata hiteorum

Taxon classificationAnimaliaAraneaeLycosidae

Wallace & Exline, 1978

Pirata hiteorum Henderson 2007: 55, 57, 61, 77, 80, 84; Jackman 1997: 165; Wallace and Exline 1978: 89, mf, desc. (figs 192–198, 204); Yantis 2005: 198, 201

####### Distribution.

Anderson, Brazos, Burleson, Colorado, Coryell, Dallas, Houston, Madison, Trinity

####### Locality.

Attwater Prairie Chicken National Wildlife Refuge, Lick Creek Park, Texas A&M University Rangeland Area

####### Time of activity.

Male: (April – July); female (April – September)

####### Habitat.

(landscape features: under rock); (soil/woodland: old field, pine woods [%: 84, 97, 100], post oak savanna with pasture, post oak woods [%: 56], post oak woodland, upland woods)

####### Method.

5 gallon bucket trap [m]; pitfall trap [mf]

####### Type.

Arkansas, Cove Creek

####### Etymology.

Person (Named after the collectors, 0. and M. Hite, Wallace and Exline 1978).

####### Collection.

TAMU

###### Pirata sedentarius

Taxon classificationAnimaliaAraneaeLycosidae

Montgomery, 1904

Pirata sedentarius Agnew et al. 1985: 7; Gertsch 1934a: 12; Henderson 2007: 53, 63, 77, 80, 84; Jackman 1997: 165; Jones 1936: 69; Montgomery 1904: 312, mf, desc. (pl. 19, figs 28–29); Reddell 1965: 174; Reddell and Smith 1965: 20; Vogel 1970b: 14; Wallace and Exline 1978: 72, mf, desc. (figs 143–144, 146–162)
Piratula sedentaria (Montgomery, 1904); Roewer 1955: 289
Pirata sedentarias Montgomery, 1904; Vogel 1970b: 14

####### Distribution.

Brazos, Burleson, Colorado, Culberson, Dallas, Edwards, Erath, Hays, Hidalgo, Kerr, McLennan, San Saba, Travis, Uvalde, Williamson

####### Locality.

5-Eagle Ranch, Attwater Prairie Chicken National Wildlife Refuge, Guadalupe Mountains National Park, Lick Creek Park, NK Ranch

####### Caves.

**Edwards** (Devil’s Sinkhole); **San Saba** (Copperhead Cave)

####### Time of activity.

Male (April – July, October); female (April – July, October)

####### Habitat.

(landscape features: cave, under rock); (littoral: creek bank, near pond, under [rock by creek, rock by creek bank]); (soil/woodland: leaf litter, post oak woodland, upland woods)

####### Method.

pitfall trap [mf] (near pond [mf])

####### Eggs/spiderlings.

Erath [21 spiderlings] [TAMU]

####### Type.

Texas (male, female, Travis Co., Austin, no date, no collector, holotype, AMNH)

####### Etymology.

Latin, sedentary

####### Collection.

DMNS, NMSU, TAMU, TMM

###### Pirata seminolus

Taxon classificationAnimaliaAraneaeLycosidae

Gertsch & Wallace, 1935

Pirata seminolus Calixto et al. 2013: 183; Jackman 1997: 165; Platnick 1993: 505 [spelling]; Yantis 2005: 198
Pirata seminola Gertsch and Wallace, 1935; Agnew et al. 1985: 7; Breene et al. 1993c: 19, 47, 95, mf (figs 130A-B); Dean and Sterling 1990: 405; Dean et al. 1982: 255; Dondale and Redner 1990: 264, mf, desc. (figs 404–407); Wallace and Exline 1978: 22, mf, desc. (figs 32–43, 61); Young and Edwards 1990: 20

####### Distribution.

Anderson, Brazos, Burleson, Colorado, Erath, Gonzales, Henderson, Robertson, Travis, Walker

####### Locality.

5-Eagle Ranch, Attwater Prairie Chicken National Wildlife Refuge, Ellis Prison Unit, Holmes Pecan Orchard, NK Ranch, Palmetto State Park

####### Time of activity.

Male (April – September); female (April – July)

####### Habitat.

(crops: cotton); (littoral: edge of pond, swamp); (orchard: pecan); (soil/woodland: oak/celtis leaf litter, pine woods [%: 100])

####### Method.

5 gallon bucket trap [mf]; berlese funnel [f]; pitfall trap [mf] (near pond [mf]); suction trap [mf]

####### Type.

Florida, Levy Lake

####### Etymology.

Indian tribe

####### Collection.

MSU, TAMU

###### Pirata spiniger

Taxon classificationAnimaliaAraneaeLycosidae

(Simon, 1898)

Pirata spiniger Henderson 2007: 57, 77, 80, 84; Yantis 2005: 198 [Wallace and Exline 1978: 79, mf, desc. (figs 163–168)]

####### Distribution.

Brazos, Houston

####### Locality.

Lick Creek Park

####### Time of activity.

Male (May – June)

####### Habitat.

(soil/woodland: pine woods [%: 80], upland woods)

####### Method.

5 gallon bucket trap [m]; pitfall trap [m]

####### Type.

Louisiana

####### Etymology.

Latin, spine-baring

####### Collection.

TAMU

###### Pirata suwaneus

Taxon classificationAnimaliaAraneaeLycosidae

Gertsch, 1940

Pirata suwaneus [Wallace and Exline 1978: 62, mf, desc. (figs 7, 9, 125–133)]

####### Distribution.

Burleson, Colorado

####### Locality.

5-Eagle Ranch, Attwater Prairie Chicken National Wildlife Refuge

####### Time of activity.

Male (May – June); female (May – June)

####### Method.

pitfall trap [mf]

####### Type.

Florida, Port Mayaca

####### Etymology.

location

####### Collection.

TAMU

###### Pirata sylvanus

Taxon classificationAnimaliaAraneaeLycosidae

Chamberlin & Ivie, 1944

Pirata sylvanus [Wallace and Exline 1978: 106, mf, desc. (figs 12, 233–237)]

####### Distribution.

Brazos

####### Locality.

Texas A&M University Rangeland Area

####### Time of activity.

Female (July)

####### Method.

pitfall trap [f]

####### Type.

Georgia, 2 miles E Sylvania

####### Etymology.

locality (city)

####### Collection.

TAMU

##### Genus *Piratula* Roewer, 1960

###### Piratula insularis

Taxon classificationAnimaliaAraneaeLycosidae

(Emerton, 1885)

Piratula insularis Omelko et al. 2011: 216 [T], 224 (figs 71–75)
Pirata insularis Emerton, 1885; Woods and Harrel 1976: 43; Young and Edwards 1990: 20 [Dondale and Redner 1990: 255, mf (figs 380–386); Wallace and Exline 1978: 40, mf, desc. (figs 77–80, 83–86)]

####### Distribution.

Jefferson

####### Habitat.

(crops: rice)

####### Type.

New York, Long Lake

####### Etymology.

Latin, from island

##### Genus *Rabidosa* Roewer, 1960

###### Rabidosa hentzi

Taxon classificationAnimaliaAraneaeLycosidae

(Banks, 1904)

Rabidosa hentzi Yantis 2005: 201 [Brady and McKinley 1994: 154, mf, desc. (figs 5, 10, 27–30)]

####### Distribution.

Houston, Trinity

####### Time of activity.

Male (April, April 24-May 2)

####### Habitat.

(soil/woodland: pine woods [%: 85, 97])

####### Method.

5 gallon bucket trap [m]

####### Type.

Florida, Altoona

####### Etymology.

Person (arachnologist)

####### Collection.

TAMU

###### Rabidosa punctulata

Taxon classificationAnimaliaAraneaeLycosidae

(Hentz, 1844)

Rabidosa punctulata Bradley 2013: 159; Brady and McKinley 1994: 146 [T], mf, desc. (figs 3, 8, 19–22); Henderson 2007: 53, 60–61, 64, 67–68, 77, 80, 84; Jackman 1997: 165; Yantis 2005: 66, 198, 201
Lycosa punctulata Hentz, 1844; Marx 1890: 563
Hogna punctulata (Hentz, 1844); Dondale and Redner 1990: 38 [T], mf, desc. (figs 21–24)

####### Distribution.

Anderson, Archer, Blanco, Brazos, Burleson, Burnet, Clay, Comal, Coryell, Dallas, Grimes, Harris, Jasper, Leon, Madison, San Patricio, Travis, Walker, Wichita

####### Locality.

Browning Ranch, Lick Creek Park, Welder Wildlife Refuge

####### Caves.

**Comal** (Bain’s Cave)

####### Time of activity.

Male (September – November); female (March – April, September – November)

####### Habitat.

(grass: grassland); (landscape features: cave); (littoral: near water); (soil/woodland: disturbed habitat, forest, live oak woodland, pine woods [%: 60, 69], post oak savanna with pasture, post oak woods [%: 43, 70, 76, 77, 80, 90, 93, 100], post oak woodland, sandy area, upland woods)

####### Method.

5 gallon bucket trap [mf]; pitfall trap [mf]

####### Type.

Pennsylvania

####### Etymology.

Latin, black spots on venter of abdomen

####### Collection.

DMNS, MSU, TAMU, TMM

###### Rabidosa rabida

Taxon classificationAnimaliaAraneaeLycosidae

(Walckenaer, 1837)

Rabidosa rabida Barron et al. 1999: 550; Bradley 2013: 160; Brady and McKinley 1994: 142 [S], mf, desc. (figs 1, 6, 11–14); Breene et al. 1993c: 19, 47, 97, mf (figs 138A-C); Calixto et al. 2013: 183, 187; Cokendolpher and Reddell 2001b: 52; Cokendolpher et al. 2008: 29 (photo 28); Dondale and Redner 1990: 41 [T]; Henderson 2007: 58, 77, 80, 84; Irungu 2007: 31; Jackman 1997: 89, desc., 165 (photo 22h); Reddell and Cokendolpher 2004: 88; Roberts 2001: 49; Yantis 2005: 66, 198, 201
Lycosa rabida Walckenaer, 1837; Agnew et al. 1985: 4, 10; Brown 1974: 234; Bumroongsook et al. 1992: 17; Dean et al. 1982: 254; Dean et al. 1988: 287; Nyffeler et al. 1986: 197; Reddell 1965: 174; Reddell and Finch 1963: 48; Rice 1985: 139; Tugmon et al. 1990: 43; Vogel 1970b: 13; Young and Edwards 1990: 19
Hogna helluo (Walckenaer, 1837); Yantis 2005: 200 [misidentified]
Lycosa scutulata Hentz, 1844; Bishop and Crosby 1926: 209; Chamberlin 1908: 253, mf, desc. (pl. 17, fig. 9, pl. 18, fig. 1); Jones 1936: 69; Marx 1890: 563; Montgomery 1904: 289, desc.

####### Distribution.

Anderson, Archer, Atascosa, Bandera, Bastrop, Bell, Bexar, Blanco, Brazos, Burleson, Cameron, Clay, Coleman, Colorado, Comal, Comanche, Coryell (imm.), Crockett, Dallas, Denton, Erath, Falls (imm.), Floyd, Freestone (imm.), Galveston, Grayson, Grimes, Harris, Harrison, Hidalgo, Houston, Jefferson, Kendall, Kerr, Kimble, Kleberg, Leon, Llano, Lubbock, McLennan, Madison, Milam, Montague, Montgomery, Nacogdoches, Parker, Potter, Reagan, Refugio, Robertson, San Patricio, Tarrant, Taylor, Travis, Trinity, Walker, Waller, Webb, Wharton, Wichita, Williamson, Wilson, Zavala

####### Locality.

Attwater Prairie Chicken National Wildlife Refuge, Bill Haney Pecan Orchard, Browning Ranch, Camp Bullis, Ellis Prison Unit, Fort Hood, Holmes Pecan Orchard, Horne Ranch, Lick Creek Park, Texas A&M University Rangeland Area, Welder Wildlife Refuge, Wildcat Bluff Nature Center

####### Caves.

**Bell** (Keilman Cave [Fort Hood]); **Bexar** (Backhole, Linda’s First Cave, Obvious Little Cave); **Comal** (Bain’s Cave); **Williamson** (Steam Cave)

####### Time of activity.

Male (May – September, November); female (April – November)

####### Habitat.

(crops: cabbage, cotton, peanuts); (grass: grass, grassland, pasture); (landscape features: cave); (littoral: playa, sedge meadow); (orchard: pecan); (plants: bluebonnets, Indian paintbrush, miscellaneous vegetation); (soil/woodland: on ground, hackberry woodland, pine woods [%: 66, 67, 69, 73, 80, 84, 88, 95, 97, 100], post oak savanna with pasture, post oak woods [%: 41, 48, 56, 71, 74, 82, 92, 100], post oak woodland, sandy brushland, upland woods); (structures: in [house, laundry room], on floor in lab)

####### Method.

5 gallon bucket trap [mf]; boll weevil pheromone trap [f]; D-Vac suction [imm.]; pitfall trap [mf] (in sand [m], under oak [m]); suction trap [imm.]

####### Eggs/spiderlings.

Hidalgo [146, 367 spiderlings] [TAMU]

####### Type.

New York

####### Etymology.

Latin, unfavorable behavior, furious

####### Collection.

DMNS, JCC, MSU, TAMU, TMM, TTU

##### Genus *Schizocosa* Chamberlin, 1904

###### Schizocosa aulonia

Taxon classificationAnimaliaAraneaeLycosidae

Dondale, 1969

Schizocosa aulonia Dondale and Redner 1978a: 159, mf, desc. (figs 22, 71–74); Jackman 1997: 165

####### Distribution.

Coleman, Nueces, Somervell, Taylor, Tom Green

####### Locality.

Horne Ranch

####### Time of activity.

Male (July); female (July)

####### Method.

pitfall trap [mf]

####### Type.

Illinois, Waukegan

####### Etymology.

Latin, beaches and sand dunes

####### Collection.

TAMU, TTU

###### Schizocosa avida

Taxon classificationAnimaliaAraneaeLycosidae

(Walckenaer, 1837)

Schizocosa avida Agnew et al. 1985: 4, 10; Bonnet 1958: 3944; Bradley 2013: 160; Breene et al. 1993c: 19, 47, 97, mf (figs 137A-C); Calixto et al. 2013: 180, 183, 185, 187; Dean et al. 1982: 255; Dean et al. 1988: 287; Dondale and Redner 1978a: 164 [S, T], mf, desc. (figs 10–12, 51–54, 89); Jackman 1997: 165 (photo 22); Vogel 1970b: 14; Young and Edwards 1990: 20
Lycosa avida Walckenaer, 1837; Jones 1936: 69; Woods and Harrel 1976: 43
Lycosa erratica Hentz, 1844; Chamberlin 1908: 251
Lycosa lepida (Keyserling, 1877); Montgomery 1904: 287

####### Distribution.

Eastern ½ Texas; Blanco, Brazos, Burleson, Coleman, Colorado, Comanche, Coryell, Dallas, Erath, Gonzalez, Hays, Houston, Jefferson, Robertson, Tom Green, Travis, Walker, Williamson

####### Locality.

5-Eagle Ranch, Attwater Prairie Chicken National Wildlife Refuge, Bill Haney Pecan Orchard, Browning Ranch, Ellis Prison Unit, Holmes Pecan Orchard, Horne Ranch, Lick Creek Park, NK Ranch, Stiles Farm Foundation

####### Time of activity.

Male (February – October); female (March – November)

####### Habitat.

(crops: cotton, peanuts, rice); (grass: grassland, pasture, sandy-prairie grass); (littoral: near lake); (orchard: pecan); (plants: bluebonnets); (soil/woodland: edge of woods, near lake, on ground, post oak savanna with pasture, sandy area, sandy-prairie grass, under oak)

####### Method.

pitfall trap [mf] (edge of woods [m], in sand [m], under oak [m])

####### Eggs/spiderlings.

Erath [51 spiderlings in eggsac]; Williamson [17, 54, 60, 64, 73, 139, 244, 270, 435 spiderlings] [TAMU]

####### Type.

New York

####### Etymology.

Latin, greedy

####### Collection.

DMNS, MSU, TAMU

###### Schizocosa bilineata

Taxon classificationAnimaliaAraneaeLycosidae

(Emerton, 1885)

Schizocosa bilineata Agnew et al. 1985: 7; Bradley 2013: 161; Dondale and Redner 1978a: 157, mf, desc. (figs 8, 47–48); Jackman 1997: 165

####### Distribution.

East Texas; Colorado, Coryell, Erath, Hidalgo, Travis, Wichita

####### Locality.

Attwater Prairie Chicken National Wildlife Refuge

####### Time of activity.

Male (April – May); female (March – May)

####### Habitat.

(grass: grass); (soil/woodland: on field border, post oak savanna with pasture)

####### Method.

pitfall trap [mf]

####### Type.

Connecticut, New Haven

####### Etymology.

Latin, row of dark spots on sternum, each side meeting behind

####### Collection.

DMNS, MSU, TAMU

###### Schizocosa crassipes

Taxon classificationAnimaliaAraneaeLycosidae

(Walckenaer, 1837)

Schizocosa crassipes Bonnet 1958: 3946; Gertsch and Wallace 1937: 17; Vogel 1970b: 14; Yantis 2005: 66, 198, 201 [Dondale and Redner 1978a: 152, mf, desc. (figs 2, 27–30); Stratton 1991: 31 (fig. 4); Stratton 1997: 86 [table of features and key for crassipes, ocreata, rovneri, stridulans, uetzi]]

####### Distribution.

Anderson, Brazos, Dallas, Houston, Leon, Travis

####### Locality.

Lick Creek Park

####### Time of activity.

Male (May – June)

####### Habitat.

(soil/woodland: old field, pine woods [%: 83, 95], post oak woods [%: 44, 77])

####### Method.

5 gallon bucket trap [m]; pitfall trap [m]

####### Type.

Georgia

####### Etymology.

Latin, thick feet

####### Collection.

TAMU

###### Schizocosa mccooki

Taxon classificationAnimaliaAraneaeLycosidae

(Montgomery, 1904)

Schizocosa mccooki Cokendolpher et al. 2008: 9, 29; Dondale and Redner 1978a: 169 [T], mf, desc. (figs 13, 15, 59–62); Jackman 1997: 165; Roberts 2001: 49
Lycosa mccooki Montgomery, 1904; Montgomery 1904: 283, f, desc. (fig. 11); Petrunkevitch 1911: 563
Lycosa maccooki Montgomery, 1904; Roewer 1955: 276
Lycosa avida Walckenaer, 1837; Gertsch 1939b: 26 [misidentified]

####### Distribution.

Western 2/3 Texas; Carson, Crockett, Potter, Travis

####### Locality.

Chisos Basin, Chisos Mountains, Fort Lancaster, Pantex Lake, Wildcat Bluff Nature Center

####### Time of activity.

Female (June)

####### Habitat.

(grass: grassland); (littoral: near playa)

####### Method.

pitfall trap

####### Type.

Texas (female, Travis Co., Austin, no date, no collector, syntype, AMNH)

####### Etymology.

Person (described spinning habits of spiders)

####### Collection.

DMNS

###### Schizocosa ocreata

Taxon classificationAnimaliaAraneaeLycosidae

(Hentz, 1844)

Schizocosa ocreata Bradley 2013: 161; Dondale and Redner 1978a: 150, mf, desc. (figs 1, 5, 36–38); Jackman 1997: 165; Jones 1936: 69 [Stratton 1991: 30, mf (figs 3, 9–11) [compares leg I of ocreata, rovneri, stridulans]; Stratton 1997: 86 [table of features and key for crassipes, ocreata, rovneri, stridulans, uetzi]]

####### Distribution.

Eastern ½ Texas; Dallas, Palo Pinto

####### Type.

North Carolina

####### Etymology.

Latin, booted

####### Note.

some records may be *crassipes*, *rovneri*, *stridulans*, or *uetzi* based on hairs and coloration of first tibia of males [see Stratton 1997: 86].

###### Schizocosa perplexa

Taxon classificationAnimaliaAraneaeLycosidae

Bryant, 1936

Schizocosa perplexa Bonnet 1958: 3948; Bryant 1936: 91, m, desc. (fig. 2); Henderson 2007: 54, 77, 80, 84; Jones 1936: 69; Roewer 1955: 295; Vogel 1970b: 14; Yantis 2005: 198, 201

####### Distribution.

Brazos, Dallas, Leon, Madison, Walker

####### Locality.

Lick Creek Park

####### Time of activity.

Male (March – May)

####### Habitat.

(soil/woodland: disturbed habitat, pine woods [%: 66], post oak woods [%: 44, 56]); (structures: swimming pool)

####### Method.

5 gallon bucket trap [m]; pitfall trap [m]

####### Type.

Texas (male, Dallas Co., Dallas, Garland Swimming Pool, March 25, 1935, S. Jones, holotype, MCZ)

[female unknown]

####### Etymology.

Latin, very different palpus

####### Collection.

TAMU

###### Schizocosa retrorsa

Taxon classificationAnimaliaAraneaeLycosidae

(Banks, 1911)

Schizocosa retrorsa Dondale and Redner 1978a: 163, mf, desc. (figs 21, 75–78); Jackman 1997: 165

####### Distribution.

El Paso, Hardeman

####### Type.

North Carolina, Linville

####### Etymology.

Latin, backward

###### Schizocosa rovneri

Taxon classificationAnimaliaAraneaeLycosidae

Uetz & Dondale, 1979

Schizocosa rovneri Calixto et al. 2013: 183, 187; Henderson 2007: 28, 40, 53–61, 77, 80, 84; Stratton 1991: 35, mf (figs 2, 7–8, 12); Yantis 2005: 66, 198, 201 [Stratton 1997: 86 [table of features and key for crassipes, ocreata, rovneri, stridulans, uetzi]]
Schizocosa ocreata (Hentz, 1844); Agnew et al. 1985: 7, 11 [part] [misidentified]

####### Distribution.

East Texas; Anderson, Brazos, Burleson, Erath, Fort Bend, Houston, Leon, Madison, Robertson, Trinity, Walker

####### Locality.

Brazos Bend State Park, Holmes Pecan Orchard, Lick Creek Park, Texas A&M University Rangeland Area

####### Time of activity.

Male (April – June); female (April – July)

####### Habitat.

(littoral: sedge meadow); (orchard: pecan); (soil/woodland: buckeye-sycamore forest, disturbed habitat, pine woods [%: 66, 67, 69, 79, 80, 82, 83, 84, 85, 88, 92, 95, 97, 100], post oak savanna with pasture, post oak woods [%: 56, 91, 92, 96], post oak woodland, sandy area, upland woods, woods)

####### Method.

5 gallon bucket trap [mf]; flight intercept trap [m]; pitfall trap [mf] (in woods [m]); blue pan trap [m]

####### Eggs/spiderlings.

Brazos [14 spiderlings] [TAMU]

####### Type.

Illinois, Allerton Park

####### Etymology.

Person (*Schizocosa rovneri* is named in honor of Dr. J. S. Rovner in recognition of his stimulating work on the behavior of North American wolf spiders, Stratton 1991).

####### Collection.

TAMU

###### Schizocosa saltatrix

Taxon classificationAnimaliaAraneaeLycosidae

(Hentz, 1844)

Schizocosa saltatrix Agnew et al. 1985: 7, 11; Bradley 2013: 162; Chamberlin 1908: 215, mf, desc. (pl. 16, figs 2, 4); Cokendolpher and Reddell 2001b: 52; Dondale and Redner 1978a: 153 [S], mf, desc. (figs 4, 39–41, 88); Henderson 2007: 53–57, 77, 80, 84; Jackman 1997: 165; Petrunkevitch 1911: 579; Reddell 1965: 174; Reddell and Cokendolpher 2004: 88; Vogel 1970b: 14; Yantis 2005: 66, 198, 201
Lycosa relucens Montgomery, 1902; Montgomery 1904: 292.

####### Distribution.

Eastern ½ Texas; Anderson, Bell, Bexar, Brazos, Burleson, Coryell, Edwards, Erath, Grimes, Hidalgo, Houston, Jeff Davis, Kerr, Leon, Madison, Travis, Trinity, Uvalde, Val Verde, Walker

####### Locality.

Davis Mountains Resort, Fort Hood, Lick Creek Park, Santa Ana National Wildlife Refuge, Texas A&M University Rangeland Area

####### Caves.

**Bell** ([all Fort Hood] Coyote Den Cave, Keilman Cave, Lunch Counter Cave, Seven Mile Mountain Cave, Treasure Cave); **Bexar** (Ailor Hill Cave, Cave of the Bearded Tree, Cave of the Half-Snake); **Travis** (Lunsford’s Cave); **Val Verde** (Wren Cave)

####### Time of activity.

Male (March – May, September); female (March – July)

####### Habitat.

(grass: grass, sandy-prairie grass, short grass); (landscape features: cave); (littoral: edge of pond, near pond); (soil/woodland: disturbed habitat, leaf litter, old field, pine woods [%: 66, 73, 83, 85, 88, 95, 97, 99, 100], post oak savanna with pasture, post oak woods [%: 44, 56, 71, 74, 75, 77, 82, 91, 92, 94, 96], post oak woodland, sandy area, under [juniper, oak], upland woods, woods)

####### Method.

5 gallon bucket trap [mf]; carrion pitfall trap [mf]; pitfall trap [mf] (edge of pond [m], in leaves [mf], in woods [m], near pond [m], under juniper [mf], under oak [f]); yellow pan trap [m]

####### Eggs/spiderlings.

Brazos [102 spiderlings] [TAMU]

####### Type.

South Carolina

####### Etymology.

Latin, to dance

####### Collection.

DMNS, MSU, TAMU, TMM

###### Schizocosa segregata

Taxon classificationAnimaliaAraneaeLycosidae

Gertsch & Wallace, 1937

Schizocosa segregata Dondale and Redner 1978a: 158, mf, desc. (figs 23, 81–82); Jackman 1997: 165; Stratton 2005: 376

####### Distribution.

Hidalgo

####### Type.

Florida, Levy Co.

####### Etymology.

Latin, separated

###### Schizocosa stridulans

Taxon classificationAnimaliaAraneaeLycosidae

Stratton, 1984

Schizocosa stridulans Henderson 2007: 28, 40, 56–62, 64, 77, 80, 84; Yantis 2005: 66, 198, 201 [Stratton 1991: 30, mf, desc. (figs 1, 5–6, 13 [compares leg I of ocreata, rovneri, stridulans]); Stratton 1997: 86 [chart of distinguishing features and key for crassipes, ocreata, rovneri, stridulans, uetzi]]
Schizocosa ocreata (Hentz, 1844); Agnew et al. 1985: 7, 11 [part] [misidentified]
Schizocosa crassipes (Walckenaer, 1837); Yantis 2005: 198 [misidentified]

####### Distribution.

Anderson, Brazos, Erath, Houston, Leon

####### Locality.

Lick Creek Park

####### Time of activity.

Male (May – July)

####### Habitat.

(soil/woodland: disturbed habitat, old field, pine woods [%: 79, 80, 83, 84, 88, 95, 99, 100], post oak woods [%: 44, 56, 77], post oak woodland, sandy area, upland woods)

####### Method.

5 gallon bucket trap [m]; blue pan trap [m]; pitfall trap [m]

####### Type.

Illinois, Sand Ridge State Forest

####### Etymology.

Latin, sound production by males during courtship

####### Collection.

TAMU

###### Schizocosa uetzi

Taxon classificationAnimaliaAraneaeLycosidae

Stratton, 1997

Schizocosa uetzi Henderson 2007: 59–61, 77, 80, 84; Yantis 2005: 66, 198 [Stratton 1997: 85, mf, desc. (figs 1–6) [chart of distinguishing features and key for crassipes, ocreata, rovneri, stridulans, uetzi]]
Schizocosa ocreata (Hentz, 1844); Agnew et al. 1985: 7, 11 [part] [misidentified]

####### Distribution.

Anderson, Brazos, Erath, Houston, Leon, Van Zandt

####### Locality.

Lick Creek Park

####### Time of activity.

Male (April 26-May 5, May – June)

####### Habitat.

(soil/woodland: disturbed habitat, pine woods [%: 79, 80, 83, 84, 88, 95], post oak woods [%: 44, 56, 77, 82], post oak woodland, under oak, upland woods)

####### Method.

5 gallon bucket trap [m]; pitfall trap [m] (under oak [m])

####### Type.

Mississippi, 8 miles SE Oxford

####### Etymology.

Person (The specific epithet is to honor Dr. George W. Uetz, spider ecologist, educator, mentor and friend, Stratton 1997).

####### Collection.

TAMU

##### Genus *Sosippus* Simon, 1888

**Note.** Sierwald (2000) lists a collection record of Hidalgo County, Edinburg, 1 female, September–December 1933, coll. Mulaik for *Sosippus mimus* Chamberlin, 1924. This is a misprint because the same data is listed further down on p. 136 under *Sosippus texanus*.

###### Sosippus texanus

Taxon classificationAnimaliaAraneaeLycosidae

Brady, 1962

Sosippus texanus Brady 1962: 160, mf, desc. (figs 4, 10, 21–22, 37–39); Brady 1972: 35; Brady 2007: 73, f, desc. (fig. 6); Jackman 1997: 165; Sierwald 2000: 136; Vogel 1967: 108; Vogel 1970b: 14
Sosippus mimus Chamberlin, 1924; Comstock 1940: 639 [Texas record]

####### Distribution.

Aransas, Cameron, Hidalgo, Zapata

####### Locality.

Bentsen-Rio Grande Valley State Park, Goose Island State Park

####### Time of activity.

Male (June); female (March – April, June – July, September – November)

####### Habitat.

(grass: grass)

####### Method.

pitfall trap [f]

####### Type.

Texas (female, Aransas Co., Goose Island State Park, June 15, 1961, A. R. Brady, holotype, MCZ)

####### Etymology.

locality (state)

####### Collection.

TAMU

##### Genus *Tigrosa* Brady, 2012

###### Tigrosa annexa

Taxon classificationAnimaliaAraneaeLycosidae

(Chamberlin & Ivie, 1944)

Tigrosa annexa Brady 2012: 189 [T], mf, desc. (figs 1–9, 40)
Hogna annexa (Chamberlin and Ivie, 1944); Jackman 1997: 165

####### Distribution.

Brazoria, Harris, Haskell, Hidalgo, Jefferson, Palo Pinto, Victoria, Wichita

####### Locality.

Lake Wichita

####### Time of activity.

Male (June – July, December); female (February – May, July, September, December)

####### Type.

Florida, Alachua Co., Gainesville

####### Etymology.

Latin, joining

####### Collection.

MSU, TAMU

###### Tigrosa aspersa

Taxon classificationAnimaliaAraneaeLycosidae

(Hentz, 1844)

Tigrosa aspersa Brady 2012: 193 [S, T], mf, desc. (figs 10–15, 44)
Lycosa inhonesta (Keyserling, 1877); Montgomery 1904: 290
Hogna aspersa (Hentz, 1844); Dondale and Redner 1990: 49, mf, desc. (figs 39–42); Jackman 1997: 165

####### Distribution.

Bexar, Brewster, Clay, Dallas, Presidio, Travis

####### Time of activity.

Female (June)

####### Type.

Alabama

####### Etymology.

Latin, scattered

####### Collection.

MSU

###### Tigrosa georgicola

Taxon classificationAnimaliaAraneaeLycosidae

(Walckenaer, 1837)

Tigrosa georgicola Brady 2012: 196 [S, T], mf, desc. (figs 16–21, 42); Calixto et al. 2013: 183
Lycosa riparia Hentz, 1844; Bishop and Crosby 1926: 209; Chamberlin 1908: 234; Comstock 1912: 633, desc.; Comstock 1940: 645; Jones 1936: 69; Petrunkevitch 1911: 566
Allocosa sp. nr georgicola (Walckenaer, 1837); Henderson 2007: 52–53, 58, 77, 80, 83 [misidentified]; Yantis 2005: 196, 199 [misidentified]
Lycosa ripariola Bonnet, 1957; Vogel 1970b: 13
Hogna georgicola (Walckenaer, 1837); Bradley 2013: 155; Jackman 1997: 165
Hogna helluo (Walckenaer, 1837); Henderson 2007: 54, 56, 59, 77, 80, 83 [misidentified]; Yantis 2005: 197, 200 [part, misidentified]
Hogna sp. nr lenta (Hentz, 1844); Yantis 2005: 197, 200 [misidentified]
Hogna sp. nr watsoni (Gertsch, 1934); Yantis 2005: 197 [misidentified]
Hogna helluo group nr georgicola (Walckenaer, 1837); Breene et al. 1993c: 18, 47 [misidentified]
Trochosa sp. nr terricola Thorell, 1856; Henderson 2007: 58, 78, 80, 84 [misidentified]

####### Distribution.

Anderson, Angelina, Austin, Bastrop, Brazos, Caldwell, Comal, Dallas, Erath, Fort Bend, Gonzales, Grayson, Grimes, Hardin, Harris, Harrison, Hays, Houston, Jefferson, Jim Wells, Karnes, Kerr, Leon, Madison, Nacogdoches, Panola, Robertson, San Patricio, Travis, Trinity, Walker

####### Locality.

Bastrop State Park, Caddo Lake State Park, Ellis Prison Unit, Holmes Pecan Orchard, Honey Creek Ranch, Lick Creek Park, NK Ranch, Palmetto State Park, Raven Ranch, Texas A&M University Rangeland Area, Welder Wildlife Refuge, Zilker Park

####### Time of activity.

Male (March – July, September – November); female (March – December)

####### Habitat.

(crops: cotton); (littoral: sedge meadow); (orchard: pecan); (soil/woodland: disturbed habitat, forest, hackberry woodland, loblolly pine unmanaged, pine woods [%: 60, 66, 67, 69, 74, 77, 80, 82, 84, 86, 88, 95, 97, 100], post oak woods [%: 41, 44, 49, 56, 60, 71, 75, 76, 77, 80, 85, 91, 92, 94, 100], post oak woodland, upland woods); (structures: in lab)

####### Method.

5 gallon bucket trap [mf]; pitfall trap [mf] (edge of pond [f])

####### Type.

Georgia, Burke Co.

####### Etymology.

locality (state)

####### Collection.

TAMU

###### Tigrosa helluo

Taxon classificationAnimaliaAraneaeLycosidae

(Walckenaer, 1837)

Tigrosa helluo Brady 2012: 200 [S, T], mf, desc. (figs 28–33, 41)
Lycosa helluo Walckenaer, 1837; Bishop and Crosby 1926: 207; Chamberlin 1908: 226, mf, desc. (pl. 17, figs 1–2); Comstock 1912: 633, desc.; Comstock 1940: 645, desc.; Petrunkevitch 1911: 560; Vogel 1970b: 13
Alopecosa helluo (Walckenaer, 1837); Bonnet 1955: 248
Hogna helluo (Walckenaer, 1837); Bradley 2013: 155; Dondale and Redner 1990: 51; Jackman 1997: 165

####### Distribution.

Harris

####### Time of activity.

Female (November)

####### Type.

New York

####### Etymology.

Latin, a glutton

####### Note.

This species has often been misidentified for *Hogna antelucana*, *Tigrosa georgicola* and others. Cited references for this species includes: Broussard and Horner 2006: 254; Brown 1974: 234; Cokendolpher et al. 2008: 9, 29; Gertsch 1939b: 26; Jones 1936: 69; Richman et al. 2011a: 47; Taber and Fleenor 2005: 280 (fig. 12–9); Woods and Harrel 1976: 43.

##### Genus *Trochosa* C. L. Koch, 1847

###### Trochosa sepulchralis

Taxon classificationAnimaliaAraneaeLycosidae

(Montgomery, 1902)

Trochosa sepulchralis Dreyer and Brady 2008: 66 [S, T], mf, desc. (figs 2–3, 6–15)
Geolycosa sepulchralis (Montgomery, 1902); Jackman 1997: 165
Trochosa sepulchralis (Montgomery, 1902); Montgomery 1904: 307
Lycosa modesta (Keyserling, 1876); Chamberlin 1908: 268, mf [misidentified]; Comstock 1912: 639, desc.; Jones 1936: 69; Petrunkevitch 1911: 563
Alopecosa sepulchralis (Montgomery, 1902); Montgomery 1904: 307; Vogel 1970b: 12
Lycosa acompa Chamberlin, 1924; Agnew et al. 1985: 7; Gertsch and Wallace 1935: 11, m, desc. (fig. 31); Vogel 1970b: 13; Wallace 1947: 36
Varacosa acompa (Chamberlin, 1924); Breene et al. 1993c: 19, 47, 98, m (fig. 139A); Jackman 1997: 165 (photo 22); Roewer 1955: 306
Trochosa acompa (Chamberlin, 1924); Henderson 2007: 28, 40, 53–59, 78, 80, 84; Yantis 2005: 66, 198, 202

####### Distribution.

Archer, Austin, Brazos, Brown, Cameron, Clay, Colorado, Coryell, Dallas, Erath, Fort Bend, Grayson, Harrison, Hidalgo, Houston, Jasper, Jeff Davis, Kerr, Kimble, Leon, Madison, McLennan, Panola, Polk, San Jacinto, San Patricio, Taylor, Terrell, Tom Green, Travis, Trinity, Val Verde, Walker

####### Locality.

Attwater Prairie Chicken National Wildlife Refuge, Caddo Lake State Park, Camp Tonkawa, Davis Mountains Resort, Ellis Prison Unit, Lick Creek Park, Raven Ranch, Welder Wildlife Refuge

####### Time of activity.

Male (February – June, August – October, December); female (February – December)

####### Habitat.

(crops: cotton); (grass: grass); (littoral: near pond); (soil/woodland: disturbed habitat, hackberry woodland, leaf litter, on field border, pine woods [%: 66, 77, 82, 85, 97], post oak savanna with pasture, post oak woods [%: 44, 56, 96], post oak woodland, sandy area, sandy brushland, under [juniper, oak], upland woods); (structures: indoors, in house, porch)

####### Method.

5 gallon bucket trap [mf]; carrion baited pitfall trap [m]; pitfall trap [mf] (in leaves [m], in sand [m], near pond [m], under juniper [mf], under oak [mf])

####### Type.

Pennsylvania, Philadelphia

####### Etymology.

Latin, burial vault, collected Woodland Cemetery

####### Collection.

MSU, TAMU

####### Note.

Breene et al. 1993c (fig. 139B) is *Trochosa* (=*Lycosa*) *abdita* (Gertsch 1934d: 3 (fig. 6) from Florida.

###### Trochosa terricola

Taxon classificationAnimaliaAraneaeLycosidae

Thorell, 1856

Trochosa terricola Brady 1980: 177, mf, desc. (figs 1–2, 10–16, 28–31); Cokendolpher et al. 2008: 9, 30; Dondale and Redner 1990: 23, mf, desc. (figs 5–8); Jackman 1997: 165; Woods and Harrel 1976: 43; Young and Edwards 1990: 20

####### Distribution.

Carson, Jefferson, Travis, Wichita

####### Time of activity.

Female (June)

####### Habitat.

(crops: rice); (grass: grassland); (littoral: near playa)

####### Method.

pitfall trap

####### Type.

Sweden, Uppsala

####### Etymology.

Latin, earthy, -cola Latin suffix meaning inhabitant of

####### Collection.

DMNS, MSU

##### Genus *Varacosa* Chamberlin & Ivie, 1942

###### Varacosa avara

Taxon classificationAnimaliaAraneaeLycosidae

(Keyserling, 1877)

Varacosa avara Bradley 2013: 164; Dondale and Redner 1990: 93, mf, desc. (figs 108–112); Henderson 2007: 28, 40, 52–53, 55–56, 60–61, 70–75, 78, 80, 84; Jackman 1997: 165; Jiménez and Dondale 1988: 172 [T]; Yantis 2005: 66, 198, 202
Lycosa avara (Keyserling, 1877); Chamberlin 1908: 279, mf, desc. (pl. 20, figs 1–3); Comstock 1912: 640, desc.; Comstock 1940: 650; Petrunkevitch 1911: 556; Vogel 1970b: 13
Trochosa avara Keyserling, 1877; Brady 1980: 190, mf, desc. (figs 3, 19–21, 36–40); Montgomery 1904: 304, f, desc. (pl. 20, fig. 42)

####### Distribution.

Anderson, Brazos, Burleson, Coryell, Dallas, Grayson, Grimes, Hardin, Harris, Houston, Jasper, Leon, Madison, Travis, Trinity, Tyler, Walker, Wichita

####### Locality.

Big Thicket National Preserve, Kirby State Forest, Lick Creek Park, Texas A&M University Rangeland Area

####### Time of activity.

Male (January 15-February 15, February, June, September – December); female (January – June, September – December)

####### Habitat.

(grass: sandy grassland, short grass); (littoral: near water); (soil/woodland: bottomland forest, disturbed habitat, forest, hardwood litter, pine woods [%: 66, 67, 69, 73, 80, 82, 86, 88, 97, 100], post oak savanna with pasture, post oak woods [%: 41, 49, 56, 60, 74, 76, 77, 80, 84, 91, 92, 93, 94, 96, 100], post oak woodland, sandy area, upland woods)

####### Method.

5 gallon bucket trap [mf]; pitfall trap [mf]

####### Eggs/spiderlings.

Brazos [73 spiderlings] [TAMU]

####### Type.

North America

####### Etymology.

Latin, avaricious

####### Collection.

DMNS, MSU, TAMU

###### Varacosa gosiuta

Taxon classificationAnimaliaAraneaeLycosidae

(Chamberlin, 1908)

Varacosa gosiuta Broussard and Horner 2006: 254; Jiménez and Dondale 1988: 172 [T]; Richman et al. 2011a: 47; Roberts 2001: 49
Trochosa gosiuta (Chamberlin, 1908); Brady 1980: 196, mf, desc. (figs 4, 26–27, 44–46); Jackman 1997: 165; Milstead 1958: 445

####### Distribution.

Northwest Texas; Brewster, Potter, Presidio, Travis

####### Locality.

Big Bend National Park, Chihuahuan desert, Chisos Basin, Dalquest Research Site, Wildcat Bluff Nature Center

####### Time of activity.

Male (August, December); female (August, October, December)

####### Habitat.

(nest/prey: stomach of *Cnemidophorus sacki*); (soil/woodland: leaf litter under oak)

####### Method.

pitfall trap [mf]

####### Type.

Utah

####### Etymology.

referring to desert

####### Collection.

DMNS, MSU, NMSU

###### Varacosa parthenus

Taxon classificationAnimaliaAraneaeLycosidae

(Chamberlin, 1925)

Varacosa parthenus Broussard and Horner 2006: 254; Jiménez and Dondale 1988: 172 [T]; Richman et al. 2011a: 47
Trochosa parthenus Chamberlin, 1925; Brady 1980: 204, mf, desc. (figs 6–9, 32–33); Jackman 1997: 165

####### Distribution.

Brewster, Presidio, Wichita

####### Locality.

Chihuahuan desert, Dalquest Research Site

####### Time of activity.

Male (November)

####### Method.

pitfall trap [mf]

####### Type.

Florida, Bartow

####### Etymology.

Greek, “parthenos” meaning virgin

####### Collection.

MSU

###### Varacosa shenandoa

Taxon classificationAnimaliaAraneaeLycosidae

(Chamberlin & Ivie, 1942)

Varacosa shenandoa Dondale and Redner 1990: 94, mf, desc. (figs 113–117); Jackman 1997: 165; Jiménez and Dondale 1988: 172 [T]
Trochosa shenandoa Chamberlin and Ivie, 1942; Agnew et al. 1985: 4; Brady 1980: 200, mf, desc. (figs 5, 17–18, 22–25, 34–35, 41–43); Breene et al. 1993b: 647; Young and Edwards 1990: 20

####### Distribution.

Aransas, Bandera, Bastrop, Bell, Brazos, Cameron, Collin, Comanche, Coryell, Denton, Erath, Gonzales, Grayson, Hidalgo, Jasper, Jim Wells, Kendall, Kerr, Kleberg, Refugio, San Patricio, San Saba, Shelby, Travis, Victoria, Wichita, Wilbarger

####### Locality.

Bentsen-Rio Grande Valley State Park

####### Time of activity.

Male (January – February, June, October – December); female (January – February, April – June, September – December)

####### Habitat.

(crops: peanuts, sugarcane); (grass: grass); (soil/woodland: edge of woods, leaf litter, post oak savanna with pasture, sandy area)

####### Method.

pitfall trap [mf] (edge of woods [f], in sand [f])

####### Type.

Virginia, Shenandoah National Park

####### Etymology.

locality (national park)

####### Collection.

DMNS, MSU, TAMU

#### Family Mimetidae Simon, 1881

**Note.** species incorrectly reported from Texas

*Mimetus epeirodes* Emerton, 1882; Breene et al. 1993a: 169; Pamanes-Guerrero 1975: 41, 81 [misidentified, not in Texas]

##### Genus *Ero* C. L. Koch, 1836

###### Ero canionis

Taxon classificationAnimaliaAraneaeMimetidae

Chamberlin & Ivie, 1935

Ero canionis Bradley 2013: 165; Jackman 1997: 165; Rice 1986: 124 [Kaston 1948: 275, mf, desc. (figs 881–882)]

####### Distribution.

San Patricio

####### Locality.

Lake Corpus Christi State Park

####### Type.

Utah, near Salt Lake City

####### Etymology.

canyon

###### Ero pensacolae

Taxon classificationAnimaliaAraneaeMimetidae

Ivie & Barrows, 1935

Ero pensacolae Dean and Sterling 1990: 401; Gertsch and Mulaik 1940: 333; Jackman 1997: 165; Vogel 1970b: 14 [Archer 1941: 193, f, desc. (fig. 3); Ivie and Barrows 1935: 19, m, desc. (pl. 7, fig. 54)]

####### Distribution.

Brazos, Cameron (imm.), Walker

####### Time of activity.

Male (October); female (December)

####### Method.

suction trap [mf]

####### Type.

Florida, Gainesville

####### Etymology.

locality (other city, -cola Latin suffix meaning inhabitant of)

####### Collection.

SIUC

##### Genus *Mimetus* Hentz, 1832

###### Mimetus haynesi

Taxon classificationAnimaliaAraneaeMimetidae

Gertsch & Mulaik, 1940

Mimetus haynesi Gertsch and Mulaik 1940: 333, m, desc. (figs 5–6); Jackman 1997: 165; Mott 1989: 87, mf, desc. (figs 73–79); Vogel 1967: 109; Vogel 1970b: 14

####### Distribution.

Cameron, Hidalgo, Zapata

####### Locality.

Sabal Palm Audubon Sanctuary, Santa Ana National Wildlife Refuge

####### Time of activity.

Male (April, October – November); female (June, October – November)

####### Habitat.

(soil/woodland: palm forest)

####### Method.

Beating [m]

####### Type.

Texas (male, Zapata Co., 32 miles SE Laredo, April 10, 1936, Haynes, holotype, AMNH)

####### Etymology.

Person (collector)

####### Collection.

SIUC, TAMU

###### Mimetus hesperus

Taxon classificationAnimaliaAraneaeMimetidae

Chamberlin, 1923

Mimetus hesperus Agnew et al. 1985: 4, 9; Breene et al. 1993c: 20, 47, 90, mf (figs 118A–C); Calixto et al. 2013: 183; Chamberlin 1923: 5, mf, desc. (figs 2, 7–8); Chamberlin 1924b: 651; Dean and Eger 1986: 141; Dean and Sterling 1990: 401, 404; Dean et al. 1982: 254; Dean et al. 1988: 286; Gertsch and Mulaik 1940: 333; Jackman 1997: 43, desc., 165; Kagan 1942: 14; Kaston 1972: 185 (fig. 411); Kaston 1978: 176 (fig. 442); Mott 1989: 63, mf, desc. (figs 44–50); Roewer 1942: 1023; Vogel 1970b: 14; Vogel and Durden 1972: 1; Young and Edwards 1990: 20
Mimetus notius Chamberlin, 1923; Dean and Sterling 1990: 401 [misidentified]

####### Distribution.

Archer, Atascosa, Baylor, Bee, Bell, Bexar, Brazos, Brewster, Brown, Colorado, Comanche, Concho, Coryell, Culberson, Erath, Grayson, Hamilton, Harris, Hidalgo, Houston, Jackson, Jim Wells, Kenedy, Kerr, Live Oak, McLennan, Nueces, Pecos, Presidio, Robertson, Starr, Sutton, Tarrant, Taylor, Terrell, Tom Green, Travis, Walker, Wichita, Wilbarger, Young

####### Locality.

Attwater Prairie Chicken National Wildlife Refuge, Bentsen-Rio Grande Valley State Park, Big Bend National Park, Bill Haney Pecan Orchard, Ellis Prison Unit, Hagerman National Wildlife Refuge, Holmes Pecan Orchard, Lake Kickapoo, Lick Creek Park

####### Time of activity.

Male (February – December); female (February – December)

####### Habitat.

(crops: cotton, peanuts); (grass: grassland, pasture); (landscape features: under rock); (littoral: sedge meadow); (orchard: pecan); (plants: bluebonnets, Indian paintbrush, miscellaneous vegetation, roadside vegetation); (soil/woodland: post oak savanna with pasture)

####### Method.

Beating [f]; cardboard band [f]; D-Vac suction; fogging [m]; pitfall trap [mf]; suction trap [mf]; sweeping [mf]

####### Type.

California, Claremont

####### Etymology.

Greek, western

####### Collection.

DMNS, MSU, SIUC, TAMU

###### Mimetus notius

Taxon classificationAnimaliaAraneaeMimetidae

Chamberlin, 1923

Mimetus notius Agnew et al. 1985: 4; Breene et al. 1993c: 20, 47, 91, mf (figs 120A-C); Calixto et al. 2013: 183; Cutler et al. 1999: 117; Dean and Sterling 1990: 404; Gertsch and Mulaik 1940: 333; Jackman 1997: 44, desc., 165 (photo 13b); Liao et al. 1984: 410; Mott 1989: 28, mf, desc. (figs 8–17); Vogel 1970b: 14; Young and Edwards 1990: 20 [Chamberlin 1923: 7, mf, desc. (figs 4, 10); Kaston 1948: 277, mf, desc. (figs 888, 894, 907)]
Mimetus hesperus Chamberlin, 1923; Dean et al. 1982: 254 [misidentified]

####### Distribution.

Anderson, Angelina, Aransas, Atascosa, Bastrop, Bell, Bexar, Brazos, Burleson, Burleson/Lee, Cameron, Cass, Dallas, Eastland, Erath, Fannin, Freestone, Goliad, Grayson, Harris, Hidalgo, Houston, Hunt, Jack, Jim Wells, Kenedy, Kerr, Kleberg, Leon, Medina, Palo Pinto, Polk, Robertson, San Jacinto, San Patricio, Terrell, Travis, Uvalde, Walker, Washington, Wichita

####### Locality.

Adriance Pecan Orchard, Bentsen-Rio Grande Valley State Park, Ellis Prison Unit, Garner State Park, Holmes Pecan Orchard, Huntsville State Park, Lake Corpus Christi State Park, Lake Travis, Lick Creek Park, Texas A&M University Rangeland Area, Welder Wildlife Refuge, Zilker Park

####### Time of activity.

Male (February – November); female (February – December)

####### Habitat.

(crops: cotton, peanuts); (littoral: creek bank, sedge meadow); (orchard: pecan); (plants: miscellaneous vegetation, vegetation); (soil/woodland: bark of Brazil tree, juniper, post oak savanna, trees, trees/shrubs, *Juniperus ashei*, *Quercus buckleyi*, *Quercus virginiana*, *Ulmus crassifolia*)

####### Method.

Beating [mf]; cardboard band [m]; fogging [m]; suction trap [mf]; sweeping [mf]

####### Type.

Florida, Runnymeade

####### Etymology.

Latin, familiar

####### Collection.

DMNS, MSU, SIUC, TAMU

###### Mimetus puritanus

Taxon classificationAnimaliaAraneaeMimetidae

Chamberlin, 1923

Mimetus puritanus Agnew et al. 1985: 7; Bradley 2013: 165; Breene et al. 1993c: 20, 47, 91, mf (figs 119A-C); Dean and Sterling 1987: 6; Jackman 1997: 44, desc., 165; Mott 1989: 43, mf, desc. (figs 18–35) [Chamberlin 1923: 5, mf, desc. (figs 1, 6); Kaston 1948: 277, mf, desc. (figs 885–887, 893, 905)]

####### Distribution.

Anderson, Archer, Brazos, Culberson, Erath, Falls, Limestone, Marion, Montgomery, Nueces, Walker, Washington, Wichita

####### Locality.

Fort Parker State Park

####### Time of activity.

Male (April, July – September); female (January, March – April, June – August)

####### Habitat.

(crops: cotton); (nest/prey: mud dauber nest); (soil/woodland: trees/shrubs)

####### Method.

Beating [f]; D-Vac suction [f]; suction trap [m]

####### Type.

New York, Ithaca

####### Etymology.

Latin, puritan or pure

####### Collection.

MSU, SIUC, TAMU

###### Mimetus syllepsicus

Taxon classificationAnimaliaAraneaeMimetidae

Hentz, 1832

Mimetus syllepsicus Archer 1941: 186 [S]; Jackman 1997: 165; Jackman et al. 2007: 199; Mott 1989: 20, mf, desc. (figs 1–7)
Mimetus interfector Hentz, 1850; Brown 1974: 235; Marx 1890: 525 [Chamberlin 1923: 6, mf, desc. (figs 5, 9); Kaston 1948: 277, mf, desc. (figs 889, 906)]

####### Distribution.

Atascosa, Brazos, Cameron, Hunt, Limestone, Nacogdoches, Walker

####### Locality.

Fort Parker State Park, Laguna Madre, Lake Tawakoni State Park, Lick Creek Park

####### Time of activity.

Male (March – April, June – August, October); female (March, June – July, September)

####### Habitat.

(nest/prey: mud dauber nest [f]); (soil/woodland: post oak savanna); (structures: under house eave); (web: large spider web)

####### Method.

Beating [mf]; sweeping [f]

####### Type.

United States

####### Etymology.

Greek, a putting together

####### Collection.

MSU, SIUC, TAMU

#### Family Miturgidae Simon, 1886

**Note.** *Cheiracanthium* and *Strotarchus* transferred to Eutichuridae (Ramírez 2014: 340).

##### Genus *Syspira* Simon, 1895

**Note.** transferred from Clubionidae (Lehtinen 1967: 266)

###### Syspira longipes

Taxon classificationAnimaliaAraneaeMiturgidae

Simon, 1895

Syspira longipes Jackman 1997: 166; Lehtinen 1967: 266; Schoenly and Reid 1983: 256 [Simon 1895: 136, f, desc.]

####### Distribution.

El Paso

####### Locality.

Chihuahuan desert

####### Type.

Mexico

[male unknown]

####### Etymology.

Latin, feet, long + foot

##### Genus *Teminius* Keyserling, 1887

**Note.** transferred from Clubionidae (Platnick and Shadab 1989: 2)

###### Teminius affinis

Taxon classificationAnimaliaAraneaeMiturgidae

Banks, 1897

Teminius affinis Banks 1897: 193, f, desc.; Banks 1910: 9; Bradley 2013: 168; Breene et al. 1993c: 20, 47, 82, mf (figs 88A-B); Calixto et al. 2013: 183; Cokendolpher et al. 2008: 9, 30 (photo 29); Comstock 1912: 323; Irungu 2007: 31; Jackman 1997: 107, 166; Jones 1936: 69; Platnick and Shadab 1989: 7 [T], mf, desc. (figs 5–8); Reddell and Cokendolpher 2004: 88; Roewer 1955: 407; Roth 1994: 82; Ubick and Richman 2005c: 174
Syrisca affinis (Banks, 1897); Agnew et al. 1985: 4; Comstock 1940: 586; Dean and Eger 1986: 142; Dean et al. 1982: 255; Gertsch 1935b: 11 (figs 31–33); Petrunkevitch 1911: 513; Roth 1982: 13–2; Roth 1985: B-8–1; Vogel 1970b: 6; Young and Edwards 1990: 16
Drassodes sp.; Dean et al. 1982: 255 [misidentified]

####### Distribution.

Baylor, Bexar, Brazos, Brown, Burleson, Cameron, Carson, Clay, Coleman, Colorado, Coryell, Dallas, Erath, Hays, Hidalgo, Houston, Kerr, Limestone, McLennan, Medina, Montague, Robertson, San Patricio, Tom Green, Travis, Walker, Wichita, Williamson

####### Locality.

Attwater Prairie Chicken National Wildlife Refuge, Bentsen-Rio Grande Valley State Park, Camp Bullis, Camp Tonkawa, Ellis Prison Unit, Fort Parker State Park, Holmes Pecan Orchard, Horne Ranch, Laguna Madre, NK Ranch, Pantex Lake, Raven Ranch, Sabal Palm Audubon Sanctuary, Stiles Farm Foundation, Welder Wildlife Refuge

####### Caves.

**Bexar** (Backhole, Banzai Mud Dauber Cave [Camp Bullis], Hornet’s Last Laugh Pit, Power Pole 60 Feature, Strange Little Cave)

####### Time of activity.

Male (February – August, October); female (March – October, November 20-December 4, December)

####### Habitat.

(crops: cabbage, cotton, peanuts); (grass: grass, grassland, tall grass); (landscape features: cave, under rock); (littoral: near pond, playa); (orchard: pecan); (plants: Indian paintbrush); (soil/woodland: ground, in log, under oak, post oak savanna with pasture, sandy area, woods); (structures: hall, indoors, in house)

####### Method.

pitfall trap [mf] (near pond [f], in sand [m], in woods [m], under oak [m])

####### Type.

Texas (female, Brazos Co., no date, no collector, holotype, MCZ)

####### Etymology.

Latin, related to *Teminius continentalis* Keyserling, 1887 = *Orodrassus coloradensis* (Emerton, 1877)

####### Collection.

DMNS, MSU, TAMU, TMM

##### Genus *Zora* C. L. Koch, 1847

**Note.** transferred from Zoridae (Ramírez 2014: 341)

###### Zora pumila

Taxon classificationAnimaliaAraneaeZoridae

(Hentz, 1850)

Zora pumila Agnew et al. 1985: 8; Bennett 2005b: 256; Corey and Mott 1991: 57, mf, desc. (figs 1–7); Jackman 1997: 170; Roth 1985: B-47–1; Roth 1994: 195

####### Distribution.

Anderson, Angelina, Erath, Fayette

####### Locality.

Angelina National Forest

####### Time of activity.

Male (May); female (May, July)

####### Habitat.

(soil/woodland: loblolly pine unmanaged, longleaf pine unmanaged)

####### Method.

pitfall trap [mf]; suction trap [imm.]

####### Type.

United States

####### Etymology.

Latin, dwarfish

####### Collection.

TAMU

#### Family Mysmenidae Petrunkevitch, 1928

##### Genus *Mysmena* Simon, 1894

###### Mysmena incredula

Taxon classificationAnimaliaAraneaeMysmenidae

(Gertsch & Davis, 1936)

Mysmena incredula Dean et al. 1988: 286; Gertsch 1960a: 15, mf, desc. (figs 19–23, 28–29); Levi 1956a: 5, mf, desc. (figs 4–19); Lopardo and Hormiga 2015: 784 [T]; Reddell 1970: 408; Roth 1982: 30–1; Roth 1985: B-26–1; Vogel 1970b: 21
Calodipoena incredula Gertsch and Davis, 1936; Bonnet 1956: 937; Breene et al. 1993b: 647; Breene et al. 1993c: 20, 47, 99, mf (figs 143A-C); Calixto et al. 2013: 183; Dean and Sterling 1990: 401, 404; Gertsch and Davis 1936: 8, mf, desc. (figs 32–33); Henderson 2007: 54, 64, 78, 80, 84; Jackman 1997: 166; Lopardo and Coddington 2005: 177; Roewer 1942: 417; Roth 1994: 133

####### Distribution.

Blanco, Brazos, Burleson, Cameron, Colorado, Coryell, Hardeman, Hidalgo, Houston, Robertson, Walker

####### Locality.

5-Eagle Ranch, Attwater Prairie Chicken National Wildlife Refuge, Big Tree-Vine Association, Browning Ranch, Ellis Prison Unit, Holmes Pecan Orchard, Lick Creek Park, NK Ranch, Sabal Palm Audubon Sanctuary, Texas A&M University Rangeland Area

####### Caves.

**Hardeman** (Walkup Cave)

####### Time of activity.

Male (March – November, December 17-January 8); female (March – September, September 28-October 4)

####### Habitat.

(crops: cotton, sugarcane); (grass: grass, grassland, pasture); (landscape features: cave); (orchard: pecan); (soil/woodland: bottomland forest, disturbed habitat, leaf litter, post oak savanna with pasture, woods)

####### Method.

D-Vac suction [imm.]; fogging [m]; pitfall trap [mf]; suction trap [mf]; tile trap [f]

####### Type.

Texas (male, Cameron Co., May 1–2, 1936, L. I. Davis, holotype, AMNH)

####### Etymology.

Latin, tiny spider, incredible

####### Collection.

TAMU, TMM

####### Note.

A male and female were collected in a suction trap 10:00 to 12:00 hours.

#### Family Nephilidae Simon, 1894

**Note.** transferred from Tetragnathidae (Kuntner 2006: 24)

**Note.** species incorrectly reported from Texas

*Nephila fasciculata* (De Geer, 1778); Marx 1889: 551 [not in Texas]

##### Genus *Nephila* Leach, 1815

###### Nephila clavipes

Taxon classificationAnimaliaAraneaeNephilidae

(Linnaeus, 1767)

Nephila clavipes Bakkegard and Davenport 1977: 564–565; Bradley 2013: 219; Brown 1974: 233; Higgins 1987: 402; Higgins 1989: 749; Higgins 1990: 774; Higgins 1992a: 95; Higgins 1992b: 639; Higgins and Buskirk 1992: 486; Higgins and Goodnight 2010: 150; Higgins and McGuinness 1990: 287; Higgins and McGuinness 1991: 287; Higgins and Rankin 1999: 264; Higgins et al. 2001: 83; Jackman 1997: 67, desc., 168 (photo 20b); Levi 1980: 17 [S], mf, desc. (figs 23–43); Levi 2005b: 233; Marx 1889: 99; Moore 1977: 96; Roth 1982: 11–3; Roth 1985: B-6–3, B-6–7; Roth 1994: 170
Nephila wistariana McCook, 1894; McCook 1893: 252

####### Distribution.

Southeast Texas; Bee, Brazoria, Brazos, Calhoun, Chambers, Fort Bend, Galveston, Hardin, Harris, Jasper, Jefferson, Lavaca, Liberty, Matagorda, Montgomery, Nacogdoches, Tyler, Willacy

####### Locality.

Big Thicket National Preserve, Brazos Bend State Park, Lick Creek Park

####### Time of activity.

Male (August, October); female (July – November)

####### Habitat.

(grass: coastal plain grasslands, tall grass); (littoral: palmetto-cypress swamp); (soil/woodland: oak, scrub forest, wooded area); (web: in web)

####### Type.

Jamaica

####### Etymology.

Latin, club-foot

####### Collection.

DMNS, MCZ, MSU, TAMU

#### Family Nesticidae Simon, 1894

##### Genus *Eidmannella* Roewer, 1935

###### Eidmannella bullata

Taxon classificationAnimaliaAraneaeNesticidae

Gertsch, 1984

Eidmannella bullata Cokendolpher and Reddell 2001a: 26; Gertsch 1984: 62, f, desc. (figs 294–295); Gertsch 1992: 78; Jackman 1997: 166

####### Distribution.

Culberson

####### Caves.

**Culberson** (Crystal Cave, Wiggley Cave)

####### Time of activity.

Female (May – June)

####### Habitat.

(landscape features: cave)

####### Type.

Texas (female, Culberson Co., Wiggley Cave, June 27, 1967, J. Reddell, J. Fish, A. R. Smith, holotype, AMNH)

[male unknown]

####### Etymology.

Latin, inflatus (projection on epigynum)

####### Collection.

TMM

###### Eidmannella delicata

Taxon classificationAnimaliaAraneaeNesticidae

Gertsch, 1984

Eidmannella delicata Cokendolpher and Reddell 2001a: 26; Culver et al. 2003: 464; Gertsch 1984: 62, f, desc. (figs 286–287); Gertsch 1992: 78; Jackman 1997: 166
Nesticus sp.; Reddell 1965: 174 [part]

####### Distribution.

Val Verde

####### Caves.

**Val Verde** (Ladder Cave)

####### Time of activity.

Female (April, August)

####### Habitat.

(landscape features: cave)

####### Type.

Texas (female, Val Verde Co., Ladder Cave, April 2, 1965, J. Reddell, holotype, AMNH)

[male unknown]

####### Etymology.

Latin, dainty, nice

####### Collection.

TMM

###### Eidmannella nasuta

Taxon classificationAnimaliaAraneaeNesticidae

Gertsch, 1984

Eidmannella nasuta Cokendolpher and Reddell 2001a: 26; Culver et al. 2003: 464; Gertsch 1984: 62, f, desc. (figs 291–293); Gertsch 1992: 78; Jackman 1997: 166

####### Distribution.

Medina

####### Caves.

**Medina** (Davenport Cave)

####### Time of activity.

Female (July)

####### Habitat.

(landscape features: cave)

####### Type.

Texas (female, Medina Co., Davenport Cave, July 10, 1966, J. and J. Reddell, holotype, AMNH)

[male unknown]

####### Etymology.

Latin, with big nose

####### Collection.

TMM

###### Eidmannella pallida

Taxon classificationAnimaliaAraneaeNesticidae

(Emerton, 1875)

Eidmannella pallida Breene et al. 1993b: 647; Breene et al. 1993c: 20, 47, 55, mf (figs 18A-C); Calixto et al. 2013: 183; Cokendolpher and Reddell 2001a: 27; Cokendolpher and Reddell 2001b: 53; Gertsch 1984: 54 [S, T], mf, desc. (figs 249–280); Jackman 1997: 49, desc., 166; Reddell 1994: 6; Reddell and Cokendolpher 2004: 89; Roberts 2001: 48
Nesticus pallidus Emerton, 1874; Barr and Reddell 1967: 260; Bryant 1940: 321; Comstock 1912: 424; Kunath and Smith 1968: 80, 97; Reddell 1963: 20; Reddell 1965: 174 [part]; Reddell 1967: 50; Reddell 1970: 407 [part]; Reddell and Finch 1963: 40, 48; Reddell and Smith 1965: 60; Smith and Reddell 1965: 29; Vogel 1970b: 15
Nesticus mexicanus (Banks, 1898); Reddell 1965: 174; Vogel 1970b: 15
Nesticus suggerens Chamberlin, 1924; Jones 1936: 69; Vogel 1970b: 15

####### Distribution.

Widespread in caves; Bandera, Bell, Bexar, Blanco, Brazos, Brooks, Burleson, Burnet, Cameron, Childress, Clay, Collingsworth, Colorado, Comal, Comanche, Coryell, Crockett, Culberson, Dallas, Edwards, Hardeman, Hays, Hidalgo, Howard, Irion, Kendall, King, Kinney, Llano, Lubbock, Matagorda, Medina, Menard, Nueces, Potter, Reagan, Real, Robertson, San Saba, Schleicher, Starr, Stonewall, Sutton, Taylor, Terrell, Travis, Uvalde, Val Verde, Walker, Ward, Washington, Wichita, Williamson

####### Locality.

Attwater Prairie Chicken National Wildlife Refuge, Bateman Ranch, Bill Haney Pecan Orchard, Browning Ranch, Ellis Prison Unit, Fort Hood, Holmes Pecan Orchard, Laguna Atascosa National Wildlife Refuge, Sabal Palm Audubon Sanctuary, White Rock Lake, Wildcat Bluff Nature Center

####### Caves.

**Bell** (Camp 6 Cave No. 1 [Fort Hood], Figure 8 Cave [Fort Hood], Hills’ Cave, Marcelino’s Cave [Fort Hood], Rugger’s Rift Cave [Fort Hood], Sledgehammer Cave [Fort Hood], Sanford Pit Cave [Fort Hood], Talking Crows Cave [Fort Hood], Valentine Cave [Fort Hood], Viper Den Cave); **Bexar** (Alligator Lizard Cave (=Wren Cave), Black Cat Cave, Caracol Creek Coon Cave, Cave No. 189, Cave No. 194, Cave site #303 [Government Canyon Karst Fauna Region], Cave site #305, Cave site #701, Dirtwater Cave, Government Canyon Bat Cave [Government Canyon State Natural Area], Headquarters Cave, I Think It’s A Cave, Kamikazi Cricket Cave, Max and Roberts Cave [=SWCA no. 3007, 3008], Persimmon Pit, Porcupine Squeeze Cave [=Grubs Cave No. 189], Robber Baron Cave, SARA Site 4 Cave, Stealth Cave, Stevens Ranch Cave No. 1, Toad Cave, Voight’s Bat Cave, Wren Cave, Young Cave No. 1); **Blanco** (Forest View Cave, T Cave); **Burnet** (Nolan’s Cave, Snellings Cave, Waldman Cave); **Childress** (Windmill Crack Cave); **Collingsworth** (Turtle Cave); **Comal** (Bender’s Cave, Brehmmer Cave [=Heidrich’s Cave], Coreth Bat Cave, Grosser’s Cave); **Coryell** ([all Fort Hood] Chigiouxs’ Cave, Mixmaster Cave, Plateau Cave No. 1, Tippit Cave); **Crockett** (09 Well, Dudley Cave, Water Cave); **Culberson** (Porcupine Fissure, Whirlwind Cave); **Edwards** (Vance Cave); **Hardeman** (Campsey Cave); **Hays** (Grapevine Cave, McCarty Cave, Wiseman Sink); **Howard** (Cramer’s Scenic Mountain Cave); **Irion** (Corngriders Cave, Noelke Cave); **Kendall** (Behr’s Cave, Sattler’s Deep Pit); **King** (River Styx Cave); **Kinney** (Webb Cave); **Llano** (Enchanted Rock Cave); **Medina** (Valdina Farms Sinkhole); **Menard** (Neel’s Cave, Powell’s Cave, Silver Mine Cave); **Reagan** (Big Lake State Park Cave); **Real** (Bonner Fallout Shelter Cave); **San Saba** (Fern Cave); **Schleicher** (Cave Y); **Stonewall** (Aspermont Bat Cave); **Sutton** (Caverns of Sonora, Mayfield Cave); **Terrell** (Sorcerer’s Cave); **Travis** (Airman’s Cave, Austin Caverns, Brodie Sink, Broken Straw Cave, Cave X, Cotterell Cave, Driskill Cave, Flint Ridge Cave, Goat Cave, Ireland’s Cave, Jack’s Joint, Jester Pit, Kretschmarr Salamander Cave, Lundsford’s Cave, Midnight Cave, Moonmilk Cave, Plethodon Cave, Spider Cave, Spyglass Cave, Whirlpool Cave, Wooden Derrick Cave); **Uvalde** (North Well Cave, Pablo’s Cave, Rambie’s Cave, Story Cave); **Val Verde** (Airport Cave, Emerald Sink, Fawcett’s Cave, Fern Cave, Four-Mile Cave, H. T. Miers Cave, Langtry Lead Cave, Oriente Milestone Molasses Bat Cave, Seminole Sink [Seminole Canyon State Historical Park]); **Ward** (Rattlesnake Cave); **Washington** (Devil’s Den); **Williamson** (Agave Cave, Argo Cave, Ballroom Cave No. 2, The Bat Well, Bat Well Cave, Beck Creek Cave, Beck Pride Cave, Beck Ranch Cave, Brents Bad Air Cave, Brown’s Cave, Cassidy Cave, Cobb Caverns, Coffin Cave, Deliverance Cave No. 1, Do Drop In Cave, Duckworth Bat Cave, East Fork Fissure, Electro-Mag Cave, Elm Water Cave, Florence Cave No. 18, Hatchet Cave, Holler Hole Cave, Inner Space Caverns, Kiva Cave No. 1, Lorfing’s Unseen Rattler Cave, McNeil Quarry Cave, Off Campus Cave, Onion Branch Cave, Polaris Cave, Reach-Around Cave, Rockfall Cave, Sore-ped Cave, Steam Cave, Sting Cave, Texella Cave, Three-Mile Cave, Trail of Tears Cave, Turner Goat Cave, War Party Cave, Williams Cave, Williams Cave No. 1)

####### Time of activity.

Male (February, April – November); female (January – December)

####### Habitat.

(crops: cotton, sugarcane); (grass: grass); (landscape features: cave); (orchard: pecan); (soil/woodland: post oak savanna with pasture, thorn thicket)

####### Method.

Flight intercept trap [m]; pitfall trap [mf]

####### Type.

Virginia, Fountain Cave

####### Etymology.

Latin, pale cave representatives

####### Collection.

JCC, MSU, NMSU, TAMU, TMM, TTU

###### Eidmannella reclusa

Taxon classificationAnimaliaAraneaeNesticidae

Gertsch, 1984

Eidmannella reclusa Cokendolpher and Reddell 2001a: 28; Culver et al. 2003: 464; Gertsch 1984: 61, f, desc. (figs 288–290); Gertsch 1992: 78; Jackman 1997: 166
Nesticus pallidus Emerton, 1874; Reddell 1965: 174 [part]

####### Distribution.

Travis

####### Caves.

**Travis** (McDonald Cave (=Schultz), Plethodon Cave, Puzzle Pit, Stovepipe Cave, Tooth Cave, Twelve Foot Dome, Ulls Water Cave)

####### Time of activity.

Female (March- June, October)

####### Habitat.

(landscape features: cave)

####### Type.

Texas (female, Travis Co., Tooth Cave, June 9, 1967, R. Mitchell, holotype, AMNH)

[male unknown]

####### Etymology.

Latin, close off, a recluse

####### Collection.

TMM, TTU

###### Eidmannella rostrata

Taxon classificationAnimaliaAraneaeNesticidae

Gertsch, 1984

Eidmannella rostrata Cokendolpher and Reddell 2001a: 28; Culver et al. 2003: 464; Gertsch 1984: 60, mf, desc. (figs 281–283); Gertsch 1992: 78; Jackman 1997: 166; Reddell and Cokendolpher 2004: 90
Nesticus pallidus Emerton, 1874; Reddell 1965: 174 [part]; Reddell 1970: 407 [part]
Nesticus spp.; Reddell 1965: 174 [part]
Nesticus sp.; Reddell 1970: 407

####### Distribution.

Central and west central Texas; Bandera, Bexar, Blanco, Burnet, Comal, Culberson, Hays, Kendall, Kinney, Medina, Real, Terrell, Travis, Uvalde, Val Verde, Williamson

####### Locality.

Camp Bullis

####### Caves.

**Bandera** (Albino Bat Cave, Can Creek Cave No. 1, Charity Cave, Fog Fissure, Fossil Cave, Garrison Hilltop Cave, Haby Salamander Cave, Haby Water Cave); **Bexar** (Backhole, Banzai Mud Dauber Cave, Bone Pile Cave [Government Canyon State Natural Area], Braken Bat Cave, Breached Dam Cave, Bullis Hole, Caracol Creek Coon Cave, Cave of the Bearded Tree, Cave of the Half-Snake, Cave No. 18, Cave site #2101, Charley’s Cute Little Hole, Cross the Creek Cave, Eagles Nest Cave, F-150 Cave, Fair Hole, Flach’s Cave, Flying Buzzworm Cave, Game Pasture Cave No. 1, Georg’s Hole, Government Canyon Bat Cave [Government Canyon State Natural Area], Headquarters Cave, Hector Hole, Helotes Blowhole, Hitzfelder’s Bone Hole [=Hitzfeler’s Cave], Hold Me Back Cave, Isocow Cave, Isopit, Low Priority Cave, Madla’s Cave, MARS Shaft, Mattke Cave, Max and Roberts Cave [=SWCA no. 3007, 3008, 3009, 3011], Poison Ivy Pit, Robber Baron Cave, Root Canal Cave, Scenic Overlook Cave [=Cave site #2101], Stahl Cave, Sunray Cave (=Cave No. 18), Surprise Sink [Government Canyon State Natural Area], SWCA no. 3011, Up the Creek Cave, Winston’s Cave, Wurzbach Bat Cave); **Blanco** (T Cave); **Burnet** (Big Bad Wolf Cave, Longhorn Caverns); **Comal** (Bad Weather Pit, Bain’s Cave, Bender’s Cave, Camp Bullis Bad Air Cave, Camp Bullis Bat Cave, Camp Bullis Cave No. 1, Camp Bullis Cave No. 3, Ebert Cave, Grosser’s Sink [=Grosser’s-Saur’s Sink], Just Now Cave, Kappelman Cave, Klar’s Cave, Knee Deep Cave, Natural Bridge Caverns, Preserve Cave [Honey Creek Preserve], Snakeskin Pit, Strosser’s Sink, Wiley’s Cave); **Culberson** (Crystal Cave, Wiggley Cave); **Hays** (Halifax Bat Cave, Nance Bat Cave); **Kendall** (474 Cave, A Hole, Cascade Caverns, Cascade Sinkhole, Cave Without-a-Name [Century Caverns], Cole Ranch Cave No. 1, Cricket Cave, Cueva de los Tres Bobos, Forget-Me-Not Cave, Forlorn Hole, Georgia W. Cave, Glen Rose Cave, Grand Column Cave, Hal’s Cave, Jan’s Fissure, Knee Deep Cave, Pfeiffer Crawlway Cave, Pfeiffer Dirt Sink, Pfeiffer’s Water Cave, Prassel Ranch Cave, Schneider Ranch Cave, Schwarz Cave, Swaglet Cave, Two Step Cave); **King** (River Styx Cave); **Kinney** (Baker’s Crossing Cave); **Medina** (Davenport Cave, Koch Cave, Surprise Cave, Windmill Cave); **Real** (Orell Bat Cave, Orell Crevice Cave, Skeleton Cave); **Terrell** (Goode Cave); **Travis** (Airman’s Cave, Feather Sink, Five Pocket Cave, Ireland’s Cave, Jack’s Joint, Kretschmarr Salamander Cave, McDonald Cave, Midnight Cave, Schulze Cave, Tooth Cave); **Uvalde** (Barn-Sized Fissure Cave, Cave Hollow Cave, Indian Creek Cave, Maybe Stream Cave, Tampke Ranch Cave, Whitecotton Bat Cave); **Val Verde** (Cave Hollow Cave); **Williamson** (Double Dog Hole Cave, East Fork Fissure, Temples of Thor Cave)

####### Time of activity.

Male (January – June, August – October); female (January – December)

####### Habitat.

(landscape features: cave)

####### Type.

Texas (female, Kendall Co., Schneider Ranch Cave, February 27, 1972, J. Reddell, holotype, AMNH)

####### Etymology.

Latin, with beak

####### Collection.

TMM

###### Eidmannella tuckeri

Taxon classificationAnimaliaAraneaeNesticidae

Cokendolpher & Reddell, 2001

Eidmannella tuckeri Cokendolpher and Reddell 2001a: 31, f, desc. (figs 2–7); Paquin and Hedin 2005a: 179

####### Distribution.

Jeff Davis

####### Caves.

**Jeff Davis** (Phantom Lake Cave)

####### Time of activity.

Female (October)

####### Habitat.

(landscape features: cave)

####### Type.

Texas (female, Jeff Davis Co., Phantom Lake Cave, October 1996, W. Tucker, holotype, AMNH)

[male unknown]

####### Etymology.

Person (This species is named in honor of the collector, William (Bill) Tucker of Grand Prairie, Texas, Cokendolpher and Reddell 2001a).

##### Genus *Gaucelmus* Keyserling, 1884

###### Gaucelmus augustinus

Taxon classificationAnimaliaAraneaeNesticidae

Keyserling, 1884

Gaucelmus augustinus Cokendolpher and Reddell 2001a: 26; Cokendolpher and Reddell 2001b: 53; Gertsch 1979: 162; Gertsch 1984: 6 [S], mf, desc. (figs 2–11, 15–16, 23–25); Jackman 1997: 166; McKenzie and Reddell 1964: 7; Paquin and Hedin 2005a: 180; Reddell 1964: 16; Reddell 1965: 174; Reddell 1970: 406; Reddell and Cokendolpher 2004: 90; Reddell and Finch 1963: 50; Roth 1982: 31–1; Roth 1985: B-27–1; Roth 1994: 135; Smith and Reddell 1971: 21; Vogel 1970b: 14
Theridium eigenmanni Banks, 1902; Banks 1902: 97, f; Banks 1910: 19; Bonnet 1959: 4470; Eigenmann 1900: 230; Roewer 1942: 503; Ulrich 1902: 97
Theridion eigenmanni Banks, 1902; Petrunkevitch 1911: 194

####### Distribution.

Central Texas; Bandera, Bell, Bexar, Blanco, Comal, Edwards, Hays, Kendall, Kerr, Kimble, Real, Travis, Uvalde, Williamson

####### Locality.

Camp Bullis, Fort Hood

####### Caves.

**Bandera** (Garrison Hilltop Cave, Haby Salamander Cave, Haby Water Cave, Mueller Cave, Sutherland Hollow Cave); **Bell** (Adam’s Gold Mine, Camp 6 Cave No. 1 [Fort Hood]); **Bexar** (Bear Cave, Holy Smoke Cave, Lost Pot Hole, Wurzbach Bat Cave, Young Cave No. 1); **Blanco** (T Cave); **Comal** (Brehmmer Cave [=Heidrich’s Cave], Brehmmer-Heidrich Cave, Coreth Bat Cave, Dierk Cave No. 1, Ebert Cave, Fischer Pit, Little Cave); **Edwards** (700 Springs Cave); **Hays** (Bear Cave, Beaver Cave [=Wonder Cave], Boyett’s Cave, Burnett Ranch Cave, Cricket Cave, Ezell’s Cave, McGlothlin Sink, Michaelis Cave, Vogelsang’s Camp, Wimberly Bat Cave, Wiseman Sink, Wiseman Sink No. 2, Wonder Cave); **Kendall** (Cave Without A Name, Knee Deep Cave); **Kerr** (Adam Wilson’s Cave, Mingus Root Cave, Smith Cave, Wilson Ranch Cave); **Kimble** (700 Springs Cave, Fleming Bat Cave); **Real** (Orell Crevice Cave, Shellhammer Cave); **Travis** (Lost Gold Cave); **Uvalde** (Tampke Ranch Cave); **Williamson** (Bonito Sink Cave, Dynamite Cave, Short Stack Cave, Sore-ped Cave, Three-Mile Cave, Tres Amigos Cave, Whiskey Jug Cave)

####### Time of activity.

Male (February – September, December); female (January – December)

####### Habitat.

(landscape features: cave)

####### Type.

Florida, Fort St. Augustin

####### Etymology.

locality (city)

####### Collection.

TMM, TTU

#### Family Oecobiidae Blackwall, 1862

##### Genus *Oecobius* Lucas, 1846

###### Oecobius cellariorum

Taxon classificationAnimaliaAraneaeOecobiidae

(Dugès, 1836)

Oecobius cellariorum Agnew et al. 1985: 6; Jackman 1997: 45, 166; Shear 1970: 136 [S], mf, desc. (figs 3–4, 13, 28, 48–49)
Oecobius texanus Bryant, 1936; Bonnet 1958: 3135; Bryant 1936: 87, mf, desc. (figs 8a–e); Comstock 1940: 291; Gertsch and Mulaik 1940: 335; Roewer 1955: 1290; Vogel 1970b: 15

####### Distribution.

North-central and central Texas; Bastrop, Baylor, Brazos, Dallas, Erath, Kerr, Wichita, Williamson

####### Time of activity.

Male (February – April, June, September); female (February – May, July, September – November)

####### Habitat.

(structures: glue board in school, indoors, in lab, side of house)

####### Method.

glue board [m]

####### Type.

unknown

####### Etymology.

Latin, of the cellar

####### Collection.

MSU, TAMU

###### Oecobius navus

Taxon classificationAnimaliaAraneaeOecobiidae

Blackwall, 1859

Oecobius navus Wunderlich 1995a: 595 [S]
Oecobius parietalis (Hentz, 1850); Gertsch and Mulaik 1940: 335; Vogel 1970b: 15 [Texas records]
Oecobius annulipes Lucas, 1846; Brown 1974: 235; Cobb 1994: 391; Jackman 1997: 45, desc., 166; Shear 1970: 138, mf, desc. (figs 9–10, 14, 29–30, 50–51); Vincent and Frankie 1985: 380

####### Distribution.

Central, west, and south Texas; Atascosa, Bexar, Brazos, Cameron, Dallas, Erath, Fort Bend, Harris, Hidalgo, Nacogdoches, Nueces, Smith, Starr, Travis, Val Verde

####### Locality.

Comstock Railroad Tunnel, Green Island Bird Refuge

####### Time of activity.

Male (March – May, August – September, December); female (January – June, August – December)

####### Habitat.

(soil/woodland: live oak, palm tree, punkwood, *Quercus virginiana*); (structures: indoors, on brick wall, side of building, in house along window sill)

####### Method.

sweeping [m]

####### Eggs/spiderlings.

Hidalgo [3 spiders, 3 eggs in eggsac] [TAMU]

####### Type.

Portugal, Madeira Islands

####### Etymology.

Latin, referring to ship

####### Collection.

DMNS, MCZ, MSU, TAMU, TMM

###### Oecobius putus

Taxon classificationAnimaliaAraneaeOecobiidae

O. P.-Cambridge, 1876

Oecobius putus Jackman 1997: 166; Kaston 1978: 75; Shear 1970: 144, mf, desc. (figs 1–2, 17, 34, 56–57)

####### Distribution.

Brewster, El Paso

####### Locality.

Big Bend National Park

####### Time of activity.

Male (May); female (May)

####### Type.

Egypt

####### Etymology.

Latin, pure or clean

#### Family Oonopidae Simon, 1890

##### Genus *Escaphiella* Platnick & Dupérré, 2009

###### Escaphiella hespera

Taxon classificationAnimaliaAraneaeOonopidae

(Chamberlin, 1924)

Escaphiella hespera Platnick and Dupérré 2009b: 14 [S, T], mf, desc. (figs 16–128) [see note below]
Scaphiella hespera Chamberlin, 1924; Comstock 1940: 312, desc.; Gertsch and Mulaik 1940: 326; Jackman 1997: 166; Kaston 1972: 91, desc. (figs 205–206); Kaston 1978: 93, desc. (figs 223–224); Vogel 1970b: 15
Scaphiella juvenilis (Gertsch and Davis, 1936); Jackman 1997: 166
Stenoonops juvenilis Gertsch and Davis, 1936; Bonnet 1958: 4156; Gertsch and Davis 1936: 21, m, desc. (fig. 27); Gertsch and Mulaik 1940: 326; Roewer 1942: 281; Vogel 1970b: 15

####### Distribution.

Cameron, Fayette, Gonzalez, Hidalgo, Kenedy, San Patricio, Starr, Val Verde, Zapata

####### Locality.

Kenedy Ranch, Laguna Madre

####### Time of activity.

Male (January, April, June, August, October – December); female (January – February, May, July, September, November – December)

####### Habitat.

(nest/prey: nest of *Neotoma micropus* nest [m]); (soil/woodland: leaf litter)

####### Method.

pitfall trap [m]; sifting [m]

####### Type.

California

####### Etymology.

Greek, western

####### Collection.

DMNS, TAMU

####### Note.

32 miles E Laredo should be 32 miles SE Laredo in Zapata Co. based on other records from this date.

##### Genus *Noonops* Platnick & Berniker, 2013

###### Noonops furtivus

Taxon classificationAnimaliaAraneaeOonopidae

(Gertsch, 1936)

Noonops furtivus Platnick and Berniker 2013a: 17 [T], mf, desc. (figs 97–110)
Oonops furtivus Gertsch, 1936; Bonnet 1958: 3190; Gertsch 1936: 6, m, desc. (figs 19–21); Gertsch and Mulaik 1940: 325; Jackman 1997: 166; Roewer 1942: 278; Vogel 1970b: 15

####### Distribution.

Caldwell, Cameron, Hidalgo

####### Locality.

Lockhart State Park, Sabal Palm Audubon Sanctuary

####### Time of activity.

Male (February, June, September); female (February, April, September)

####### Habitat.

(littoral: dry irrigation ditch)

####### Method.

sifting [m]

####### Type.

Texas (male, Hidalgo Co., 7 miles E Edinburg, February 17, 1935, S. Mulaik, holotype, AMNH)

####### Etymology.

Latin, furtive

##### Genus *Oonopoides* Bryant, 1940

###### Oonopoides secretus

Taxon classificationAnimaliaAraneaeOonopidae

(Gertsch, 1936)

Oonopoides secretus Platnick and Berniker 2013b: 17 [T], mf, desc. (figs 104–114)
Oonops secretus Gertsch, 1936; Bonnet 1958: 3192; Gertsch 1936: 8, m, desc. (figs 14–16); Gertsch and Mulaik 1940: 325; Jackman 1997: 166; Roewer 1942: 278; Vogel 1970b: 15

####### Distribution.

Bexar, Burleson, Cameron, Gonzalez, Hidalgo, Hudspeth, Nueces, San Patricio

####### Locality.

Guadalupe Pass, Welder Wildlife Refuge

####### Time of activity.

Male (January, March 22-April 28, April, July, November – December); female (January, April, October, December)

####### Habitat.

(soil/woodland: leaf litter under cactus)

####### Method.

Berlese funnel [f]; pitfall trap [m]

####### Type.

Texas (male, Cameron Co., 15 miles SW Harlingen, November 18, 1934, S. Mulaik, holotype, AMNH)

####### Etymology.

Latin, secret

####### Collection.

DMNS, TAMU

##### Genus *Oonops* Templeton, 1835

###### Oonops stylifer

Taxon classificationAnimaliaAraneaeOonopidae

Gertsch, 1936

Oonops stylifer Bonnet 1958: 3193; Gertsch 1936: 6, m, desc. (figs 10–12); Gertsch and Mulaik 1940: 325; Jackman 1997: 166; Roewer 1942: 279; Vogel 1970b: 15

####### Distribution.

Hidalgo

####### Time of activity.

Male (June)

####### Type.

Texas (male, Hidalgo Co., Edinburg, June 1935, S. Mulaik, holotype, AMNH)

[female unknown]

####### Etymology.

Latin, embolus with long spine

##### Genus *Opopaea* Simon, 1891

###### Opopaea concolor

Taxon classificationAnimaliaAraneaeOonopidae

(Blackwall, 1859)

Opopaea concolor Platnick and Dupérré 2009a: 22 [S], mf, desc. (figs 73–104)
Opopaea devia Gertsch, 1936; Bonnet 1958: 3197; Gertsch 1936: 5, f, desc. (fig. 13); Gertsch and Mulaik 1940: 326; Jackman 1997: 166; Roewer 1942: 288; Vogel 1970b: 15

####### Distribution.

Hidalgo

####### Time of activity.

Male (May); female (March – May, December)

####### Type.

Madeira

####### Etymology.

Latin, sexes similar in color

###### Opopaea floridana

Taxon classificationAnimaliaAraneaeOonopidae

(Banks, 1896)

Opopaea floridana [Chickering 1969: 153, f, desc. (figs 20–21)]

####### Distribution.

Comal

####### Type.

Florida, Punta Gorda

[male unknown]

####### Etymology.

locality (state)

####### Collection.

MCZ

###### Opopaea meditata

Taxon classificationAnimaliaAraneaeOonopidae

Gertsch & Davis, 1936

Opopaea meditata Bonnet 1958: 3197; Comstock 1940: 312, desc.; Gertsch and Davis 1936: 20, f, desc. (figs 25–26); Gertsch and Mulaik 1940: 325; Jackman 1997: 166; Platnick and Dupérré 2009b: 4; Roewer 1942: 288
Opopaea mediata Gertsch and Davis, 1936; Vogel 1970b: 15

####### Distribution.

Bexar

####### Time of activity.

Female (December)

####### Type.

Texas (female, Bexar Co., San Antonio, December 28, 1935, L. I. Davis, holotype, AMNH)

[male unknown]

####### Etymology.

Latin, meditate

###### Opopaea sedata

Taxon classificationAnimaliaAraneaeOonopidae

Gertsch & Mulaik, 1940

Opopaea sedata Gertsch and Mulaik 1940: 325, f, desc.; Jackman 1997: 166; Platnick and Dupérré 2009b: 4; Vogel 1967: 110; Vogel 1970b: 15

####### Distribution.

Brooks

####### Time of activity.

Female (June)

####### Type.

Texas (female, Brooks Co., Encino, June 3, 1936, S. Mulaik, holotype, AMNH)

[male unknown]

####### Etymology.

Latin, quiet

##### Genus *Orchestina* Simon, 1882

###### Orchestina saltitans

Taxon classificationAnimaliaAraneaeOonopidae

Banks, 1894

Orchestina saltitans Brown 1974: 235; Jackman 1997: 166 [Petrunkevitch 1920: 158, mf, desc. (figs 1–9)]

####### Distribution.

Nacogdoches

####### Time of activity.

Male (July)

####### Habitat.

(structures: in house, on bedspread in house)

####### Type.

New York, Long Island

####### Etymology.

Latin, leaping

#### Family Oxyopidae Thorell, 1870

**Note.** species incorrectly reported from Texas

*Hamataliwa* sp.; Comstock 1912: 661 [distinct species, Travis Co., unknown]

*Oxyopes tibialis* F. O. P.-Cambridge, 1902; Milstead 1958: 445 [not in United States]

##### Genus *Hamataliwa* Keyserling, 1887

###### Hamataliwa grisea

Taxon classificationAnimaliaAraneaeOxyopidae

Keyserling, 1887

Hamataliwa grisea Brady 1964: 501, mf, desc. (figs 108–109, 115–116, 126–127, 134–135); Comstock 1940: 668; Jackman 1997: 102, desc., 166; Vincent and Frankie 1985: 380; Vogel 1970b: 15

####### Distribution.

Central, west and south Texas; Aransas, Bexar, Brazos, Brewster, Brown, Burleson, Cameron, Hidalgo, Jeff Davis, Jim Wells, Kinney, Nueces, Presidio, San Patricio, Starr, Travis, Uvalde

####### Locality.

Bentsen-Rio Grande Valley State Park, Fort Sam Houston, Goose Island State Park, Riley Estate, Russell Farm, Sabal Palm Audubon Sanctuary, Santa Ana National Wildlife Refuge, Welder Wildlife Refuge

####### Time of activity.

Male (March 3-April 4, June); female (April – October)

####### Habitat.

(nest/prey: mud dauber nest [f]); (plants: Compositae); (soil/woodland: forest, live oak, post oak savanna with pasture, sandy brushland, trees, *Quercus virginiana*, *Ulmus crassifolia*); (structures: insect emergence cage outside)

####### Method.

Beating [f]; flight intercept trap [m]; pitfall trap [f]; sweeping [m]

####### Type.

North America

####### Etymology.

Middle Latin, gray

####### Collection.

MCZ, MSU, NMSU, TAMU

###### Hamataliwa helia

Taxon classificationAnimaliaAraneaeOxyopidae

(Chamberlin, 1929)

Hamataliwa helia Brady 1964: 497 [T], mf, desc. (figs 112–114, 119–121, 124–125, 130–133); Brady 1970: 83; Brady and Santos 2005: 191; Jackman 1997: 166; Vogel 1970b: 15
Oxyopes helius Chamberlin, 1929; Bryant 1936: 92, m, desc. (fig. 7); Jones 1936: 69; Roewer 1955: 334

####### Distribution.

Brazos, Cameron, Dallas, Hidalgo, Jasper, Kaufman, Tyler

####### Locality.

Kirby State Forest, Sabal Palm Audubon Sanctuary

####### Time of activity.

Male (April – June, August, October); female (March 30-April 27, April – June, October – November)

####### Habitat.

(soil/woodland: willow)

####### Method.

pitfall trap [f]

####### Type.

Georgia, Okefenokee Swamp, Mixson’s Hammock

####### Etymology.

referring to the sun

####### Collection.

TAMU

###### Hamataliwa unca

Taxon classificationAnimaliaAraneaeOxyopidae

Brady, 1964

Hamataliwa unca Brady 1964: 499, mf, desc. (figs 110–111, 117–118, 122–123, 128–129); Brady 1970: 97; Brady and Santos 2005: 191; Jackman 1997: 166; Vogel 1967: 111; Vogel 1970b: 15
Hamataliwa unica Brady, 1964; Armstrong and Richman 2007: 396

####### Distribution.

Cameron, Hidalgo

####### Locality.

Russell Farm

####### Time of activity.

Male (“September-December”); female (June, September – October, December)

####### Method.

Boll weevil pheromone trap [m]

####### Type.

Texas (male, Hidalgo Co., Edinburg, September-December 1933, S. Mulaik, holotype, AMNH)

####### Etymology.

arbitrary combination of letters

####### Collection.

NMSU, TAMU

##### Genus *Oxyopes* Latreille, 1804

###### Oxyopes acleistus

Taxon classificationAnimaliaAraneaeOxyopidae

Chamberlin, 1929

Oxyopes acleistus Brady 1975: 198 [S]; Breene et al. 1993a: 169; Henderson 2007: 60, 78, 80, 84; Jackman 1997: 166; Yantis 2005: 197 [Brady 1964: 454, mf, desc. (figs 5–6, 18–20, 29–30, 37–38)]
Oxyopes aureus Brady, 1964; Brady 1964: 457, mf, desc. (figs 1–4, 13–17, 27–28, 35–36); Vogel 1967: 111; Vogel 1970b: 15

####### Distribution.

Widespread; Anderson, Aransas, Atascosa, Bee, Brazoria, Brazos, Brewster, Brooks, Burleson, Calhoun, Cameron, Dallas, DeWitt, Harris, Hays, Hidalgo, Kenedy, San Patricio, Starr, Travis, Walker, Williamson, Zapata

####### Locality.

Bentsen-Rio Grande Valley State Park, Goose Island State Park, Hoskins Mound, Kenedy Ranch, Lake Corpus Christi State Park, Lick Creek Park, Lower Rio Grande Valley National Wildlife Refuge, Santa Ana National Wildlife Refuge, Texas A&M University Rangeland Area, Welder Wildlife Refuge

####### Time of activity.

Male (March – November); female (January, March – November)

####### Habitat.

(grass: grass); (littoral: sand dune under live oak); (nest/prey: mud dauber nest); (orchard: citrus); (plants: Compositae, herbaceous vegetation, miscellaneous vegetation); (soil/woodland: disturbed habitat, forest, hackberry matte, *Juniperus* managed plot, mesquite thicket, pine woods [%: 100], riparian mesquite forest, trees)

####### Method.

5 gallon bucket trap [f]; beating [f]; blue pan trap [m]; D-Vac suction [mf]; flight intercept trap [mf]; flight intercept trap on ground [m]; pitfall trap [mf]; yellow pan trap [m]; sweeping [mf]

####### Type.

Florida, Sanford

####### Etymology.

without closure

####### Collection.

DMNS, MCZ, TAMU

###### Oxyopes aglossus

Taxon classificationAnimaliaAraneaeOxyopidae

Chamberlin, 1929

Oxyopes aglossus Brady 1964: 461, mf, desc. (figs 9–12, 21–22, 31–32, 39–40); Jackman 1997: 166; Kaston 1978: 199, desc.; Rapp 1984: 6; Vogel 1970b: 15

####### Distribution.

Galveston, Jasper, Tyler

####### Locality.

Kirby State Forest

####### Time of activity.

Male (May – June); female (June)

####### Habitat.

(soil/woodland: sandy area)

####### Type.

Georgia, Okefenokee Swamp, Billy’s Island

####### Etymology.

noun, without a tongue

####### Collection.

TAMU

###### Oxyopes apollo

Taxon classificationAnimaliaAraneaeOxyopidae

Brady, 1964

Oxyopes apollo Agnew et al. 1985: 4, 10; Brady 1964: 467, mf, desc. (figs 41–42, 47–50, 72–75); Brady 1969: 428; Brady 1975: 216; Breene et al. 1993c: 21, 48, 93, mf (figs 123A-C); Broussard and Horner 2006: 254; Calixto et al. 2013: 183, 187; Dean and Eger 1986: 142; Dean et al. 1982: 255; Dean et al. 1988: 287; Jackman 1997: 102, desc., 166; Kaston 1978: 199, desc.; Rapp 1984: 6; Richman et al. 2011a: 47; Roberts 2001: 49; Vogel 1967: 111; Vogel 1970b: 15; Young and Edwards 1990: 20

####### Distribution.

Widespread; Anderson, Atascosa, Bastrop, Bexar, Blanco, Brazos, Brewster, Brooks, Burleson, Cameron, Colorado, Comanche, Coryell, Dimmit, Erath, Freestone, Galveston, Garza, Hays, Hidalgo, Houston, Kaufman, Kenedy, Kerr, Kleberg, Knox, Leon, Llano, Lubbock, Maverick, Milam, Nueces, Polk, Potter, Presidio, Robertson, San Patricio, Somervell, Terrell, Uvalde, Val Verde, Van Zandt, Victoria, Walker, Wichita, Willacy, Williamson

####### Locality.

5-Eagle Ranch, Attwater Prairie Chicken National Wildlife Refuge, Big Bend National Park, Bill Haney Pecan Orchard, Browning Ranch, Chaparral Wildlife Management Area, Chihuahuan desert, Dalquest Research Site, Ellis Prison Unit, Galveston Island State Park, Garner State Park, Holmes Pecan Orchard, Santa Ana National Wildlife Refuge, Seminole Canyon State Park, Somerville Lake, Stiles Farm Foundation, Texas A&M University Rangeland Area, Welder Wildlife Refuge, Wildcat Bluff Nature Center

####### Time of activity.

Male (March – October); female (March – October)

####### Habitat.

(crops: cotton, peanuts); (grass: grass, grasses, grassland, pasture); (littoral: salt marsh area); (orchard: grapefruit, pecan); (plants: bluebonnets, Indian paintbrush, miscellaneous vegetation, *Oenothera* sp.); (soil/woodland: clay soil brushland, paloverde upland area, post oak savanna with pasture, sandy area, sandy open prairie, savanna, scrub cottonwood, woods)

####### Method.

D-Vac suction [f]; pitfall trap [mf] (in sand [m]); ramp trap [mf]; suction trap [imm.]; sweeping [mf]; tile trap [f]; yellow pan trap [f]

####### Type.

Texas (male, Brooks Co., Encino, May 12, 1952, M. Cazier, W. Gertsch, R. Schrammel, holotype, AMNH)

####### Etymology.

noun in apposition, Greek god

####### Collection.

DMNS, JCC, MSU, TAMU

###### Oxyopes cougar

Taxon classificationAnimaliaAraneaeOxyopidae

Brady, 1969

Oxyopes cougar [Brady 1969: 432, mf desc. (figs 1–4, 9–12)]

####### Distribution.

Angelina, Brazos

####### Locality.

Angelina National Forest, Lick Creek Park

####### Time of activity.

Male (May); female (September)

####### Habitat.

(soil/woodland: longleaf pine managed, post oak savanna)

####### Method.

pitfall trap [m]; sweeping [f]

####### Type.

Florida, Escambia Co., 8 miles NW Pensacola

####### Etymology.

noun in apposition, after the Cougar

####### Collection.

TAMU

###### Oxyopes felinus

Taxon classificationAnimaliaAraneaeOxyopidae

Brady, 1964

Oxyopes felinus Broussard and Horner 2006: 254; Richman et al. 2011a: 47 [Brady 1964: 476, mf, desc. (figs 56–57, 64–65); Brady 1975: 217, f desc. (figs 21–22, 39–40, 69–70, 102–103)]

####### Distribution.

Brewster, Presidio

####### Locality.

Chihuahuan desert, Dalquest Research Site

####### Method.

pitfall trap [m]

####### Type.

Arizona, Santa Catalina Mountains, Molino Basin

####### Etymology.

adjective, cat-like

####### Collection.

MSU

###### Oxyopes lynx

Taxon classificationAnimaliaAraneaeOxyopidae

Brady, 1964

Oxyopes lynx Brady 1964: 475, m, desc. (figs 58–59, 66–67); Brady 1969: 428; Jackman 1997: 166; Vogel 1967: 111; Vogel 1970b: 15

####### Distribution.

Brewster, Val Verde

####### Locality.

Seminole Canyon State Park

####### Time of activity.

Male (June – July)

####### Type.

Texas (male, Brewster Co., Marathon, June 12, 1948, M. A. Cazier, holotype, AMNH)

[female unknown]

####### Etymology.

noun in apposition after the Lynx

####### Collection.

TAMU

###### Oxyopes panther

Taxon classificationAnimaliaAraneaeOxyopidae

Brady, 1975

Oxyopes panther Broussard and Horner 2006: 254; Richman et al. 2011a: 47 [Brady 1975: 227, f, desc. (figs 15–16, 45–46)]

####### Distribution.

Brewster, Presidio

####### Locality.

Chihuahuan desert, Dalquest Research Site

####### Method.

pitfall trap [f]

####### Type.

Arizona, 12 miles W Portal

[male unknown]

####### Etymology.

noun in apposition after the Panther

####### Collection.

MSU

###### Oxyopes salticus

Taxon classificationAnimaliaAraneaeOxyopidae

Hentz, 1845

Oxyopes salticus Agnew et al. 1982: 631; Agnew et al. 1985: 4, 10; Bonnet 1958: 3240; Brady 1964: 478, mf, desc. (figs 80–86, 91–96, 104–105); Breene 1988: 15, 17, 23–26, 35–37, 39–41, 44, 47, 49; Breene et al. 1988: 180–181; Breene et al. 1989: 162; Breene et al. 1993c: 21, 48, 92, mf (figs 122A-C); Brown 1974: 235; Bumroongsook et al. 1992: 17; Calixto et al. 2013: 183, 189–190; Cokendolpher et al. 2008: 10, 30; Dean and Eger 1986: 142; Dean and Sterling 1985: 117; Dean and Sterling 1987: 6; Dean and Sterling 1990: 402, 405; Dean and Sterling 1992: 3–4; Dean et al. 1982: 255; Dean et al. 1987: 268; Dean et al. 1988: 285, 287; Glick and Noble 1961: 7; Henderson 2007: 52, 78, 81, 84; Jackman 1997: 103, desc., 166 (photo 29c); Jones 1936: 69; Kagan 1942: 41; Kagan 1943: 258; Marx 1890: 567; McDaniel et al. 1981: 104; Nuessly and Sterling 1984: 97; Nyffeler and Sterling 1994: 1295, 1298; Nyffeler et al. 1987a: 356–357; Nyffeler et al. 1987b: 1114; Nyffeler et al. 1988a: 55; Nyffeler et al. 1992a: 1181; Nyffeler et al. 1992b: 1459–1460; Nyffeler et al. 1992c: 2; Pamanes-Guerrero 1975: 16, 34, 37, 41, 59, 63, 78, 81; Rapp 1984: 6; Roberts 2001: 49; Rogers and Horner 1977: 523; Sterling et al. 1979: 979; Vincent and Frankie 1985: 380; Vogel 1970b: 16; Woods and Harrel 1976: 43; Young and Edwards 1990: 20

####### Distribution.

Widespread; Anderson, Atascosa, Austin, Bastrop, Bee, Bell, Bexar, Blanco, Bosque, Bowie, Brazoria, Brazos, Briscoe, Brown, Burleson, Burnet, Cameron, Camp, Carson, Cass, Cherokee, Collin, Colorado, Comal, Comanche, Cooke, Coryell, Cottle, Crosby, Dallas, Delta, Denton, DeWitt, Eastland, Ellis, Erath, Falls, Fayette, Floyd, Fort Bend, Franklin, Frio, Gaines, Galveston, Gillespie, Goliad, Gonzales, Grimes, Hale, Harris, Hays, Henderson, Hidalgo, Hill, Hockley, Hopkins, Houston, Howard, Hudspeth, Jasper, Jefferson, Jim Wells, Kaufman, Kendall, Kenedy, Kerr, Knox, Lamar, Lampasas, Liberty, Llano, Lubbock, Madison, Martin, McLennan, McMullen, Mills, Mitchell, Montgomery, Nacogdoches, Navarro, Newton, Nueces, Panola, Pecos, Potter, Rains, Reeves, Robertson, San Patricio, Shelby, Somervell, Starr, Taylor, Terry, Titus, Tom Green, Travis, Uvalde, Val Verde, Van Zandt, Victoria, Walker, Wharton, Wichita, Willacy, Williamson, Wise, Wood

####### Locality.

5-Eagle Ranch, Adriance Pecan Orchard, Attwater Prairie Chicken National Wildlife Refuge, Bastrop State Park, Bill Haney Pecan Orchard, Browning Ranch, Ellis Prison Unit, Falcon State Park, Galveston Island State Park, Garner State Park, Goliad State Park, Holmes Pecan Orchard, Jones State Forest, Kenedy Ranch, Lacuna Park, Lake Normangee, Lick Creek Park, Nash Prairie, Palmetto State Park, Pantex Lake, Seminole Canyon State Park, Stiles Farm Foundation, Texas A&M University Rangeland Area, Welder Wildlife Refuge, Wildcat Bluff Nature Center

####### Time of activity.

Male (March – October); female (February – November)

####### Habitat.

(crops: cotton, guar, peanuts, rice); (grass: grass, grassland, grasses and weeds, grassy and shrub area, pasture); (littoral: near playa, salt marsh area, sand dune area); (nest/prey: mud dauber nest [f]); (orchard: pecan); (plants: bluebonnets, in bush near house, clover, croton, cutleaf evening primrose, emergent vegetation, Indian paintbrush, miscellaneous vegetation, roadside vegetation, vegetation, *Aphanostephus* sp., *Coreopsis* sp., *Dalea* sp., *Eleocharis* sp., *Hedeoma* sp., *Monarda citriodora*, *Rudbeckia* sp.); (soil/woodland: brush, hackberry matte, live oak, post oak savanna with pasture, post oak woodland, savanna, trees/shrubs, under oak, *Juniperus ashei*, *Quercus buckleyi*, *Quercus virginiana*); (structures: around house, on folded sail of boat, in lab)

####### Method.

Beating/sweeping [m]; boll weevil pheromone trap [mf]; cardboard band [imm.]; D-Vac suction [mf]; fogging [mf]; pitfall trap [mf] (under oak [mf]); ramp trap [mf]; suction trap [m]; sweeping [mf]; tile trap [mf]; yellow pan trap [mf]

####### Type.

North Carolina and Alabama

####### Etymology.

Latin, jumping

####### Collection.

DMNS, MSU, TAMU

###### Oxyopes scalaris

Taxon classificationAnimaliaAraneaeOxyopidae

Hentz, 1845

Oxyopes scalaris Agnew et al. 1985: 7; Brady 1964: 484, mf, desc. (figs 87–90, 97–99, 106–107); Brown 1974: 235; Jackman 1997: 103, desc., 166; Vogel 1970b: 16; Woods and Harrel 1976: 43; Young and Edwards 1990: 20

####### Distribution.

Baylor, Brazos, Culberson, Erath, Harris, Jefferson, Llano, Lubbock, Nacogdoches, Travis, Tyler, Walker

####### Locality.

Texas A&M University Rangeland Area

####### Time of activity.

Male (March – June); female (May – August)

####### Habitat.

(crops: rice); (grass: grass); (littoral: near pond); (nest/prey: mud dauber nest [mf]); (plants: miscellaneous vegetation); (soil/woodland: juniper, pine, woods, *Juniperus ashei*, *Ulmus crassifolia*)

####### Method.

Beating [mf]; pitfall trap [m] (near pond [m]); sweeping [mf]

####### Type.

North Carolina

####### Etymology.

Latin, of ladder or scales

####### Collection.

JCC, MCZ, MSU, TAMU

###### Oxyopes tridens

Taxon classificationAnimaliaAraneaeOxyopidae

Brady, 1964

Oxyopes tridens Brady 1964: 472, mf, desc. (figs 45–46, 53–55, 62–63, 70–71); Broussard and Horner 2006: 254; Jackman 1997: 166; Knutson et al. 2010: 515; Richman et al. 2011a: 47; Vogel 1970b: 16

####### Distribution.

Brewster, Howard, La Salle, Presidio

####### Locality.

Big Bend National Park, Chaparral Wildlife Management Area, Chihuahuan desert, Chisos Mountains, Dalquest Research Site

####### Time of activity.

Female (July – August, September 11-October 10)

####### Habitat.

(soil/woodland: acacia area, saltcedar)

####### Method.

pitfall trap [mf]; yellow pan trap [f]

####### Type.

Nevada, Mercury

####### Etymology.

adjective meaning trident

####### Collection.

MSU, TAMU

##### Genus *Peucetia* Thorell, 1869

###### Peucetia longipalpis

Taxon classificationAnimaliaAraneaeOxyopidae

F. O. P.-Cambridge, 1902

Peucetia longipalpis Brady 1964: 512, mf, desc. (figs 151–155); Brady and Santos 2005: 191; Jackman 1997: 105, 166; Vogel 1970b: 16

####### Distribution.

Cameron, El Paso, Hidalgo

####### Locality.

Franklin Mountains, Sabal Palm Audubon Sanctuary

####### Time of activity.

Male (April – May, October)

####### Habitat.

(plants: miscellaneous vegetation)

####### Method.

sweeping [m]

####### Type.

Mexico, Guerrero, Amula

####### Etymology.

Latin, long palp

####### Collection.

NMSU, TAMU

###### Peucetia viridans

Taxon classificationAnimaliaAraneaeOxyopidae

(Hentz, 1832)

Peucetia viridans Agnew et al. 1982: 631; Agnew et al. 1985: 4; Bonnet 1958: 3492; Brady 1964: 506 [S], mf, desc. (figs 136–148, 158–161); Breene 1988: 23–26, 35, 39–40; Breene et al. 1988: 180–181; Breene et al. 1989: 162; Breene et al. 1993c: 21, 48, 92, mf (figs 121A-C); Brown 1974: 235; Bumroongsook et al. 1992: 17; Calixto et al. 2013: 183; Dean and Eger 1986: 142; Dean and Sterling 1987: 6; Dean et al. 1982: 255; Dean et al. 1987: 268; Dean et al. 1988: 285, 287; Jackman 1997: 104, desc., 166 (photo 29c); Jones 1936: 69; Kagan 1942: 39; Kagan 1943: 258; Killebrew and Ford 1985: 376; Nuessly and Sterling 1984: 97; Nyffeler et al. 1987a: 356–357; Nyffeler et al. 1992a: 1181; Nyffeler et al. 1992b: 1459–1460; Nyffeler et al. 1992c: 2; Pamanes-Guerrero 1975: 16, 34, 37, 42, 60, 63, 78, 81; Rapp 1984: 6; Sterling et al. 1979: 979; Taber and Fleenor 2003: 231; Taylor and Pfannenstiel 2008: 999; Vogel 1970b: 16; Young and Edwards 1990: 20
Peucetia abboti Walckenaer, 1837; Woods and Harrel 1976: 43
Puecetia viridans (Hentz, 1832); Knutson et al. 2010: 515

####### Distribution.

Widespread; Anderson, Aransas, Atascosa, Bastrop, Bexar, Brazoria, Brazos, Brown, Burleson, Cameron, Colorado, Comanche, Cooke, Coryell, Dallas, DeWitt, Erath, Fayette, Frio, Galveston, Gillespie, Hamilton, Hidalgo, Houston, Howard, Jack, Jefferson, Jim Wells, Leon, Limestone, McLennan, Montgomery, Nacogdoches, Polk, Presidio, Rains, Reeves, Robertson, San Patricio, Smith, Sutton, Travis, Val Verde, Victoria, Walker, Webb, Wichita, Willacy, Williamson, Zapata, Zavala

####### Locality.

5-Eagle Ranch, Adriance Pecan Orchard, Attwater Prairie Chicken National Wildlife Refuge, Big Bend Ranch State Park, Ellis Prison Unit, Holmes Pecan Orchard, Lick Creek Park, Ramsey Prison Farm, Sabal Palm Audubon Sanctuary, Stiles Farm Foundation

####### Time of activity.

Male (June – October); female (April, June – November)

####### Habitat.

(crops: cotton, peanuts, rice); (grass: grassland, pasture, tall grass and weeds in pastures); (nest/prey: mud dauber nest [mf]); (orchard: pecan); (plants: bluebonnets, croton, Indian paintbrush, miscellaneous vegetation, rose bush, vegetation, *Ambrosia* sp., *Lectuca* sp., *Monarda citriodora*, *Veronia* sp.); (soil/woodland: forest, open field, pine, prairie, saltcedar, sandy area, *Juniperus ashei*, *Quercus virginiana*, *Ulmus crassifolia*)

####### Method.

cardboard band [imm.]; D-Vac suction [m]; suction trap [m]; sweeping [mf]

####### Type.

North Carolina and Alabama

####### Etymology.

Latin, color, green

####### Collection.

DMNS, MSU, NMSU, TAMU

#### Family Philodromidae Thorell, 1870

**Note.** Species incorrectly reported from Texas

*Philodromus barrowsi* Gertsch, 1934; Dondale 1961: 212 [based on immature female]; Gertsch 1934b: 17; Roewer 1955: 786; Vogel 1970b: 27 [not in Texas]

*Tibellus maritimus* (Menge, 1875); Woods and Harrel 1976: 44; Young and Edwards 1990: 21 [not in Texas]

**nomen dubium**

*Philodromus abbotii* Walckenaer, 1837; Kaston 1953: 102; Kaston 1972: 246.

##### Genus *Apollophanes* O. P.-Cambridge, 1898

###### Apollophanes punctipes

Taxon classificationAnimaliaAraneaePhilodromidae

(O. P.-Cambridge, 1891)

Apollophanes punctipes Agnew et al. 1985: 8; Calixto et al. 2013: 183; Dondale and Redner 1975c: 1178, mf, desc. (figs 1–2, 4–5, 13, 18–25); Jackman 1997: 166

####### Distribution.

Brewster, Coke, Comanche, Erath, Hidalgo

####### Locality.

Anzalduas County Park, Bentsen-Rio Grande Valley State Park, Big Bend National Park, Bill Haney Pecan Orchard, Chisos Basin, Frontera Audubon, Santa Ana National Wildlife Refuge

####### Time of activity.

Male (April, June – July, September); female (June, September – November)

####### Habitat.

(orchard: grapefruit, orange, pecan, sour orange); (soil/woodland: trees/shrubs, under juniper)

####### Method.

Beating [f]; cardboard band [mf]; pitfall trap [m] (under juniper [m]); suction trap [m]; sweeping

####### Type.

Guatemala

####### Etymology.

Latin, minute black spots

####### Collection.

TAMU

###### Apollophanes texanus

Taxon classificationAnimaliaAraneaePhilodromidae

Banks, 1904

Apollophanes texanus Broussard and Horner 2006: 255; Dondale and Redner 1975c: 1181 [spelling], mf, desc. (figs 3, 6, 26–28); Jackman 1997: 166; Kaston 1953: 103 (fig. 253); Kaston 1972: 247 (fig. 562); Kaston 1978: 239 (fig. 609); Petrunkevitch 1911: 402; Richman et al. 2011a: 49; Roewer 1955: 767; Vogel 1970b: 27
Apollophanes texana Banks, 1904; Banks 1904: 113, mf, desc. (figs 12, 20); Banks 1910: 51

####### Distribution.

Central and west Texas; Bexar, Brewster, Culberson, El Paso, Hudspeth, Presidio, Terrell

####### Locality.

Big Bend National Park, Big Bend Ranch State Park, Chihuahuan desert, Dalquest Research Site, Davis Mountains, Guadalupe Mountains

####### Time of activity.

Female (March)

####### Habitat.

(landscape features: under rock)

####### Method.

pitfall trap [m]

####### Type.

Texas (male, female, Bexar Co., San Antonio, syntype, no date, no collector, MCZ)

####### Etymology.

locality (state)

####### Collection.

MCZ, MSU, NMSU

##### Genus *Ebo* Keyserling, 1884

###### Ebo evansae

Taxon classificationAnimaliaAraneaePhilodromidae

Sauer & Platnick, 1972

Ebo evansae Jackman 1997: 166; Sauer and Platnick 1972: 41, mf, desc. (figs 5–6, 17)

####### Distribution.

Brewster

####### Locality.

Big Bend National Park

####### Type.

Utah, Salt Lake City

####### Etymology.

Person (The species is named in honor of Mrs. Dana Evans, Department of Biology, Concord College, Athens, W. Va., Sauer and Platnick 1972).

###### Ebo iviei

Taxon classificationAnimaliaAraneaePhilodromidae

Sauer & Platnick, 1972

Ebo iviei [Sauer and Platnick 1972: 41, mf, desc. (figs 3–4, 16)]

####### Distribution.

Brewster

####### Type.

Utah

####### Etymology.

Person (The species is named in honor of the late Wilton Ivie, who collected the series from Utah and first recognized the species as new, Sauer and Platnick 1972).

####### Collection.

MSU

###### Ebo latithorax

Taxon classificationAnimaliaAraneaePhilodromidae

Keyserling, 1884

Ebo latithorax Dondale and Redner 1978b: 33, mf, desc. (figs 78–82); Jackman 1997: 120, 166; Kaston 1978: 235, desc. (fig. 602); Rapp 1984: 8; Sauer and Platnick 1972: 38, mf, desc. (figs 1–2, 15)

####### Distribution.

Galveston, Grayson, Harris

####### Habitat.

(soil/woodland: sandy area)

####### Method.

pitfall trap

####### Type.

Virginia, Richmond

####### Etymology.

Latin, wide thorax

###### Ebo merkeli

Taxon classificationAnimaliaAraneaePhilodromidae

Schick, 1965

Ebo merkeli Jackman 1997: 166; Sauer and Platnick 1972: 42, mf, desc. (figs 7–8, 18)

####### Distribution.

Val Verde

####### Type.

California, Borrego Valley

####### Etymology.

Person (collector, D. E. Merkel)

###### Ebo pepinensis

Taxon classificationAnimaliaAraneaePhilodromidae

Gertsch, 1933

Ebo pepinensis Cokendolpher et al. 1979: 724; Cokendolpher et al. 2008: 10, 30; Dondale and Redner 1978b: 34, mf, desc. (figs 83–86); Jackman 1997: 120, 166; Sauer and Platnick 1972: 43, mf, desc. (figs 11–12, 19)

####### Distribution.

Archer, Carson, Collin, Dallam, Lubbock, Navarro, Palo Pinto, Potter, Wichita

####### Locality.

Buffalo Lakes, Pantex Lake

####### Time of activity.

Male (April); female (April)

####### Habitat.

(grass: grass, grassland); (littoral: playa edge)

####### Method.

pitfall trap; sweeping [f]

####### Type.

Minnesota, Lake Pepin, Wacouta Beach

####### Etymology.

locality (Lake Pepin)

####### Collection.

MSU

###### Ebo punctatus

Taxon classificationAnimaliaAraneaePhilodromidae

Sauer & Platnick, 1972

Ebo punctatus Agnew et al. 1985: 5, 11; Breene et al. 1993c: 21, 48, 76, mf (figs 71A-B); Calixto et al. 2013: 183; Dean and Sterling 1987: 7; Jackman 1997: 120, 166; Sauer and Platnick 1972: 44, mf, desc. (figs 13–14, 20); Young and Edwards 1990: 20

####### Distribution.

Comanche, Coryell, Dallas, Erath, Grayson, Hale, Knox, McLennan, Martin, Robertson, San Patricio, Travis, Williamson

####### Locality.

Bill Haney Pecan Orchard, Holmes Pecan Orchard, Stiles Farm Foundation

####### Time of activity.

Male (April – October); female (June – August)

####### Habitat.

(crops: cotton, peanuts); (littoral: edge of pond); (orchard: pecan); (soil/woodland: post oak savanna with pasture)

####### Method.

pitfall trap [mf] (edge of pond [m]); suction trap [m]

####### Type.

Oklahoma, Stillwater

####### Etymology.

Latin, dark markings on abdomen

####### Collection.

DMNS, MSU, TAMU

##### Genus *Philodromus* Walckenaer, 1826

###### Philodromus alascensis

Taxon classificationAnimaliaAraneaePhilodromidae

Keyserling, 1884

Philodromus alascensis Agnew et al. 1985: 8; Cokendolpher et al. 1979: 726; Jackman 1997: 166 [Dondale and Redner 1975b: 379, mf, desc. (figs 30–40)]

####### Distribution.

Baylor, Erath

####### Time of activity.

Male (February); female (July)

####### Habitat.

(soil/woodland: brush)

####### Method.

sweeping

####### Type.

Alaska, Fort Yukon

####### Etymology.

locality (state)

####### Collection.

MSU, TAMU

###### Philodromus californicus

Taxon classificationAnimaliaAraneaePhilodromidae

Keyserling, 1884

Philodromus californicus [Dondale and Redner 1976a: 142, mf, desc. (figs 29–34, 68–70)]

####### Distribution.

Presidio

####### Locality.

Big Bend Ranch State Park

####### Time of activity.

Female (March)

####### Habitat.

(soil/woodland: cottonwood)

####### Method.

Beating [f]

####### Type.

California, San Francisco

####### Etymology.

locality (state)

####### Collection.

NMSU

###### Philodromus cespitum

Taxon classificationAnimaliaAraneaePhilodromidae

(Walckenaer, 1802)

Philodromus cespitum Agnew et al. 1985: 8; Cokendolpher et al. 1979: 726; Jackman 1997: 166; Knutson et al. 2010: 515 [Dondale and Redner 1976a: 131, mf, desc. (figs 1–2, 38–39)]

####### Distribution.

Archer, Comanche, Howard, Scurry, Wichita

####### Locality.

Lake Thomas, Proctor Lake

####### Time of activity.

Male (May – June, August – September, December); female (May – July, September, November)

####### Habitat.

(crops: *Sorghum halepense*); (orchard: *Prunus persica*); (plants: Compositae); (soil/woodland: mesquite, saltcedar, willow, bark of *Prosopis grandulosa*, *Salix nigra*)

####### Method.

sweeping [m]

####### Type.

France

####### Etymology.

Latin, tufted

####### Collection.

MSU, TAMU

###### Philodromus histrio

Taxon classificationAnimaliaAraneaePhilodromidae

(Latreille, 1819)

Philodromus histrio Cokendolpher et al. 1979: 726; Jackman 1997: 166 [Dondale and Redner 1975b: 373, mf, desc. (figs 10–25)]

####### Distribution.

Wichita

####### Time of activity.

Female (March)

####### Habitat.

(plants: *Artemesia filifolia*)

####### Type.

France

####### Etymology.

Latin, an actor

####### Collection.

MSU

###### Philodromus imbecillus

Taxon classificationAnimaliaAraneaePhilodromidae

Keyserling, 1880

Philodromus imbecillus Cokendolpher et al. 1979: 726; Dondale and Redner 1968: 7, mf, desc. (figs 1–2, 93–96, 210, 222); Dondale and Redner 1978b: 71, mf, desc. (figs 206–213); Jackman 1997: 166; Vogel 1970b: 27

####### Distribution.

Archer, Baylor, Brown, Clay, Comanche, Harris, Wichita, Young

####### Locality.

Proctor Lake

####### Time of activity.

Male (February, May – June); female (March – June)

####### Habitat.

(grass: *Cynodon dactylon*); (plants: *Thelesperma* sp., *Vicia* sp.); (soil/woodland: mesquite, willow, *Prosopis grandulosa*); (structures: wall of house)

####### Type.

Georgia

####### Etymology.

Latin, feeble

####### Collection.

MSU, TAMU

###### Philodromus infuscatus

Taxon classificationAnimaliaAraneaePhilodromidae

Keyserling, 1880

Philodromus infuscatus Jones 1936: 69; Kagan 1942: 45; Kagan 1943: 258; Platnick 1998: 814 [S]; Vogel 1970b: 27; Young and Edwards 1990: 20
Philodromus infuscatus infuscatus Keyserling, 1880; Cokendolpher et al. 1979: 726; Dondale and Redner 1969: 929, mf, desc. (figs 11–12, 48–50, 83); Dondale and Redner 1978b: 60, mf, desc. (figs 159–164); Jackman 1997: 166

####### Distribution.

Archer, Baylor, Coryell, Dallas, Denton, Grayson, Kerr, McLennan, Milam, Nacogdoches, Wichita, Wilbarger

####### Locality.

Lake Kickapoo

####### Time of activity.

Male (September – October); female (October – November)

####### Habitat.

(plants: miscellaneous vegetation); (soil/woodland: mesquite, bark and leaves of *Prosopis grandulosa*)

####### Method.

Beating [f]; light trap; sweeping [f]

####### Eggs/spiderlings.

Wichita [27–30 eggs] [Cokendolpher et al. 1979: 726]

####### Type.

Maryland, Baltimore

####### Etymology.

Latin, browned

####### Collection.

MSU, TAMU

###### Philodromus keyserlingi

Taxon classificationAnimaliaAraneaePhilodromidae

Marx, 1890

Philodromus keyserlingi Agnew et al. 1985: 5; Brown 1974: 238; Calixto et al. 2013: 183, 186–187; Cokendolpher et al. 1979: 727; Dean and Eger 1986: 142; Dean and Sterling 1990: 403, 405; Dondale 1961: 209, mf, desc. (figs 5, 12, 28, 35); Dondale and Redner 1976a: 138, mf (figs 19–21, 55–58); Jackman 1997: 166; Liao et al. 1984: 411; Vogel 1970b: 27; Young and Edwards 1990: 21

####### Distribution.

Widespread; Bandera, Brazos, Brewster, Burleson, Burnet, Comanche, Coryell, Erath, Falls, Jones, Lampasas, Montague, Nacogdoches, Nolan, Robertson, Travis, Walker, Wichita, Young

####### Locality.

Adriance Pecan Orchard, Bill Haney Pecan Orchard, Ellis Prison Unit, Holmes Pecan Orchard, Inks Lake State Park, Lake Buchanan, Lick Creek Park, Lost Maples State Park, Proctor Lake, Texas A&M University Rangeland Area

####### Time of activity.

Male (February 22-March 11, April – June, September – October, December); female (March – July)

####### Habitat.

(crops: peanuts); (nest/prey: mud dauber nest [f]); (objects: on cage outside); (orchard: pecan); (plants: Indian paintbrush, miscellaneous vegetation, roadside vegetation); (soil/woodland: cedar elm, hackberry, juniper, post oak savanna with pasture, tree, trees/shrubs, woods, under bark of *Celtis* sp., *Juniperus ashei*, *Quercus buckleyi*, *Quercus virginiana*, under bark of *Sapindus drummondii*, *Ulmus crassifolia*) ; (structures: indoors, porch)

####### Method.

Beating [mf]; cardboard band [mf]; fogging [mf]; malaise trap [m]; moth pheromone trap [m]; pitfall trap [m]; suction trap [mf]; sweeping [mf]

####### Eggs/spiderlings.

Robertson [eggsac laid May 25, 2001, hatched June 11, 113 eggs unhatched, 31 spiderlings]; [100 eggs, 104 spiderlings] [TAMU]

####### Type.

Washington D. C.

####### Etymology.

Person (arachnologist)

####### Collection.

MSU, TAMU

###### Philodromus laticeps

Taxon classificationAnimaliaAraneaePhilodromidae

Keyserling, 1880

Philodromus laticeps Dondale and Redner 1976a: 132, mf, desc. (figs 3–4, 40–41); Dondale and Redner 1978b: 48, mf, desc. (figs 106–109); Jackman 1997: 166

####### Distribution.

East Texas

####### Type.

Georgia

####### Etymology.

Latin, side of head

###### Philodromus lutulentus

Taxon classificationAnimaliaAraneaePhilodromidae

Gertsch, 1934

Philodromus lutulentus [Dondale and Redner 1976a: 134, mf, desc. (figs 5–6, 42–43)]

####### Distribution.

Archer, Lampasas

####### Type.

Georgia, Atlanta

####### Etymology.

Latin, clay yellow-lens

####### Collection.

MSU

###### Philodromus marginellus

Taxon classificationAnimaliaAraneaePhilodromidae

Banks, 1901

Philodromus marginellus Agnew et al. 1985: 8; Calixto et al. 2013: 183; Cokendolpher et al. 1979: 727; Jackman 1997: 166 [Dondale and Redner 1976a: 137, mf, desc. (figs 7–9, 44–46)]

####### Distribution.

Brazos, Comanche, Erath, Robertson, Travis, Wichita

####### Locality.

Bill Haney Pecan Orchard, Holmes Pecan Orchard, Texas A&M University Rangeland Area

####### Time of activity.

Male (April – May); female (April – July)

####### Habitat.

(orchard: pecan); (soil/woodland: juniper, *Juniperus ashei*, *Quercus virginiana*, *Ulmus crassifolia*); (structures: wooden porch near light)

####### Method.

at night; beating [mf]; cardboard band [f]; fogging [f]; pitfall trap [m]; suction trap [m]; sweeping [mf]

####### Type.

Arizona, Santa Rita Mountains

####### Etymology.

Latin, a body margined with brown

####### Collection.

TAMU

###### Philodromus marxi

Taxon classificationAnimaliaAraneaePhilodromidae

Keyserling, 1884

Philodromus marxi Banks 1910: 52; Bonnet 1958: 3577; Calixto et al. 2013: 183; Chickering 1940: 225; Dondale and Redner 1968: 12 [spelling], mf, desc. (figs 8–10, 105–107, 205); Dondale and Redner 1978b: 69, mf, desc. (figs 199–205); Jackman 1997: 166; Kaston 1978: 237, desc. (fig. 604); Marx 1890: 559; Petrunkevitch 1911: 420; Rydzak and Killebrew 1982: 7; Vogel 1970b: 27; Yantis 2005: 201
Philodromus marxii Keyserling, 1884; Keyserling 1884: 677

####### Distribution.

Angelina, Brazos, Colorado, Dallas, Hardin, Kenedy, Montague, Robertson, Smith, Trinity, Walker

####### Locality.

Holmes Pecan Orchard, Kenedy Ranch, Riley Estate

####### Time of activity.

Male (April – May); female (May)

####### Habitat.

(nest/prey: mud dauber nest [mf]); (orchard: pecan); (plants: vegetation); (soil/woodland: pine woods [%: 66]); (structures: indoors)

####### Method.

5 gallon bucket trap [m]; malaise trap [m]; pitfall trap [m]

####### Type.

Texas (female, Colorado Co., Columbus, no date, Marx collection, syntype locality unknown; others Wisconsin)

####### Etymology.

Person (collector)

####### Collection.

MSU, TAMU

###### Philodromus minutus

Taxon classificationAnimaliaAraneaePhilodromidae

Banks, 1892

Philodromus minutus Agnew et al. 1985: 5; Cokendolpher et al. 1979: 727; Dean and Eger 1986: 142; Dean and Sterling 1990: 403, 405; Dondale and Redner 1968: 54, mf, desc. (figs 78–80, 193–198, 208, 218); Dondale and Redner 1978b: 84, mf, desc. (figs 260–270); Jackman 1997: 166; Kaston 1978: 238, desc. (fig. 608); Rydzak and Killebrew 1982: 7; Vogel 1970b: 27; Young and Edwards 1990: 21

####### Distribution.

Brazos, Comal, Dallas, Denton, Erath, Fannin, Jack, Kerr, Leon, Montague, Smith, Travis, Walker, Wichita

####### Locality.

Ellis Prison Unit, Lick Creek Park

####### Time of activity.

Male (March – April, June, October); female (March – June)

####### Habitat.

(crops: peanuts); (plants: bluebonnets, Indian paintbrush); (soil/woodland: juniper, oak, *Quercus buckleyi*, *Quercus virginiana*, *Ulmus crassifolia*)

####### Method.

Beating [f]; beating/sweeping [f]; boll weevil pheromone trap [m]; suction trap [mf]; sweeping [mf]

####### Type.

New York, Ithaca

####### Etymology.

Latin, size

####### Collection.

MSU, TAMU

###### Philodromus montanus

Taxon classificationAnimaliaAraneaePhilodromidae

Bryant, 1933

Philodromus montanus Cokendolpher et al. 1979: 727; Jackman 1997: 166 [Dondale and Redner 1968: 51, mf, desc. (figs 73–77, 179–183)]

####### Distribution.

Wichita

####### Time of activity.

Male (March)

####### Habitat.

(grass: grass)

####### Type.

North Carolina, Black Mountain

####### Etymology.

Latin, montain

####### Collection.

MSU

###### Philodromus placidus

Taxon classificationAnimaliaAraneaePhilodromidae

Banks, 1892

Philodromus placidus Cokendolpher et al. 1979: 727; Dondale and Redner 1968: 32, mf, desc. (figs 34–35, 132–136, 207, 216); Jackman 1997: 166; Kaston 1978: 237, desc. (fig. 605); Rydzak and Killebrew 1982: 7; Vogel 1970b: 27

####### Distribution.

Archer, Burleson, Dallas, Montgomery, Panola, Smith, Stephens, Travis

####### Locality.

Jones State Forest, Shoshone Park

####### Time of activity.

Male (April – May); female (April – May)

####### Habitat.

(crops: cotton, peanuts); (grass: grassland, pasture); (plants: miscellaneous vegetation, *Monarda citriodora*); (soil/woodland: post oak savanna, shrub under *Populus deltoides*, *Quercus buckleyi*, *Quercus virginiana*, *Ulmus crassifolia*)

####### Method.

Beating [f]; sweeping [mf]

####### Type.

New York, Ithaca

####### Etymology.

Latin, calm

####### Collection.

MSU, TAMU

###### Philodromus praelustris

Taxon classificationAnimaliaAraneaePhilodromidae

Keyserling, 1880

Philodromus praelustris Cokendolpher et al. 1979: 727; Jackman 1997: 166 [Dondale and Redner 1976a: 145, mf, desc. (figs 35–37, 71–73)]

####### Distribution.

Kerr, Wichita

####### Time of activity.

Male (February, April, December); female (April)

####### Habitat.

(plants: vegetation); (soil/woodland: shrub, under bark, wild cherry, under bark of *Bumelia lanuginosa*); (structures: house)

####### Type.

Colorado

####### Etymology.

Latin, encircling before

####### Collection.

MSU

###### Philodromus pratariae

Taxon classificationAnimaliaAraneaePhilodromidae

(Scheffer, 1904)

Philodromus pratariae Agnew et al. 1985: 5; Breene et al. 1993c: 22, 48, 76, mf (figs 72A–C); Cokendolpher et al. 1979: 728; Cokendolpher et al. 2008: 10, 30 (fig. 11); Dean and Sterling 1987: 7; Dean and Sterling 1990: 403; Dean et al. 1982: 255; Dondale and Redner 1969: 923, mf, desc. (figs 1–2, 37–38, 81); Jackman 1997: 166; Nyffeler et al. 1992c: 2; Rydzak and Killebrew 1982: 7; Young and Edwards 1990: 21
Misumenops pratariae Scheffer, 1904; Rydzak and Killebrew 1982: 7

####### Distribution.

Brazoria, Brazos, Brown, Burleson (imm.), Cameron, Carson, Clay, Colorado, Donley, Erath, Fannin, Grayson, Houston, Kaufman, Lavaca, Llano, Polk, Rains, Smith, Somervell, Travis, Walker, Wichita, Williamson, Wise

####### Locality.

Attwater Prairie Chicken National Wildlife Refuge, Brazoria National Wildlife Refuge, Ellis Prison Unit, Lick Creek Park, Pantex Lake, Riley Estate, South Padre Island, Texas A&M University Rangeland Area, Tyler State Park

####### Time of activity.

Male (May, July – October); female (August – October)

####### Habitat.

(crops: cotton, peanuts, sorghum, *Sorghum halepense*); (grass: grass, grassland, pasture, *Panicum virgatum*); (littoral: near playa); (plants: miscellaneous vegetation, sage, vegetation, *Ambrosia* sp., *Monarda citriodora*); (soil/woodland: post oak savanna, tree)

####### Method.

Beating [m]; D-Vac suction [f]; pitfall trap; suction trap [m]; sweeping [mf]

####### Type.

Kansas, Manhattan

####### Etymology.

Latin, place, prairie

####### Collection.

DMNS, MSU, TAMU

###### Philodromus rufus quartus

Taxon classificationAnimaliaAraneaePhilodromidae

Dondale & Redner, 1968

Philodromus rufus quartus Cokendolpher et al. 1979: 728; Jackman 1997: 166 [Dondale and Redner 1968: 26, mf, desc. (figs 25–26, 121–122, 213)]
Philodromus rufus Walckenaer, 1826; Rydzak and Killebrew 1982: 7 [Texas record]

####### Distribution.

Smith, Wichita

####### Time of activity.

Male (April)

####### Habitat.

(soil/woodland: ground)

####### Type.

Canada, Ontario, Cochrane

####### Etymology.

undetermined

####### Collection.

MSU

###### Philodromus undarum

Taxon classificationAnimaliaAraneaePhilodromidae

Barnes, 1953

Philodromus undarum Dondale and Redner 1968: 15, mf, desc. (figs 11–12, 108–110); Jackman 1997: 166; Vogel 1970b: 27

####### Distribution.

Dallas

####### Type.

North Carolina, Beaufort, Carrot Island

####### Etymology.

Latin, wavy lines

###### Philodromus vulgaris

Taxon classificationAnimaliaAraneaePhilodromidae

(Hentz, 1847)

Philodromus vulgaris Agnew et al. 1985: 8; Bradley 2013: 175; Calixto et al. 2013: 183; Cokendolpher et al. 1979: 728; Dondale 1961: 205, mf, desc. (figs 16–17, 23, 32, 39); Dondale and Redner 1976a: 140, mf (figs 25–28, 63–67); Dondale and Redner 1978b: 55, mf, desc. (figs 135–143); Jackman 1997: 166; Roberts 2001: 50; Vogel 1970b: 27

####### Distribution.

North-central and central Texas; Brazos, Clay, Dallas, Denton, Erath, Lubbock, Potter, Robertson, Travis, Wichita

####### Locality.

Holmes Pecan Orchard, Wildcat Bluff Nature Center

####### Time of activity.

Male (January – May, November); female (February – June, December)

####### Habitat.

(grass: grass); (nest/prey: bird nest); (orchard: orchard, peach, pecan, under bark of *Prunus persica*); (plants: paradise); (soil/woodland: ash, chinaberry, elm, hackberry, oak, under bark of [*Celtis* sp., *Fraxinus americana*, *Salix nigra*, *Sapindus drummondii*, *Ulmus crassifolia*], *Juniperus ashei*, *Quercus buckleyi*, *Quercus virginiana*); (structures: house, wall of house)

####### Method.

Ballooning; beating [f]; cardboard band [f]; flight intercept trap [m]; light trap; malaise trap [m]; pitfall trap [m]; suction trap [m]; sweeping [mf]

####### Type.

United States

####### Etymology.

Latin, common

####### Collection.

DMNS, JCC, MSU, TAMU

##### Genus *Thanatus* C. L. Koch, 1837

###### Thanatus altimontis

Taxon classificationAnimaliaAraneaePhilodromidae

Gertsch, 1933

Thanatus altimontis Henderson 2007: 70, 78, 81, 84 [Dondale et al. 1964: 647, mf, desc. (figs 25–27, 43–44)]

####### Distribution.

Brazos

####### Locality.

Lick Creek Park

####### Time of activity.

Male (October 20-November 15, November)

####### Habitat.

(soil/woodland: disturbed habitat)

####### Method.

pitfall trap [m]

####### Type.

Wyoming Cokeville, Smith’s Fork Canyon

####### Etymology.

Latin, high mountain

####### Collection.

TAMU

###### Thanatus formicinus

Taxon classificationAnimaliaAraneaePhilodromidae

(Clerck, 1757)

Thanatus formicinus Agnew et al. 1985: 5, 11; Breene et al. 1993c: 22, 48, 77, mf (figs 73A–C); Calixto et al. 2013: 183; Cokendolpher et al. 1979: 729; Cokendolpher et al. 2008: 10, 48; Dean and Eger 1986: 142; Dean et al. 1982: 255; Dondale and Redner 1978b: 113, mf, desc. (figs 62, 69, 364–369); Dondale et al. 1964: 644, mf, desc. (figs 28–30, 35–37); Jackman 1997: 166; Nyffeler et al. 1992c: 2; Rydzak and Killebrew 1982: 7; Vogel 1970b: 27; Young and Edwards 1990: 21

####### Distribution.

Bexar, Brazos, Burleson, Carson, Clay, Colorado, Comal, Comanche, Cooke, Coryell, Culberson, Erath, Hays, Kerr, Palo Pinto, Smith, Walker, Wichita

####### Locality.

Attwater Prairie Chicken National Wildlife Refuge, Bill Haney Pecan Orchard, Ellis Prison Unit, Pantex Lake

####### Time of activity.

Male (March 29-April 5, April – August, November – December); female (March – August)

####### Habitat.

(crops: cotton, peanuts); (grass: grass, grassland); (littoral: near playa); (orchard: pecan); (plants: bluebonnets, Indian paintbrush, *Monarda citriodora*); (soil/woodland: ground, post oak savanna with pasture, sandy area)

####### Method.

pitfall trap [m] (in sand [m]); sweeping [f]

####### Type.

Sweden

####### Etymology.

Latin, relating to ants

####### Collection.

MSU, TAMU

###### Thanatus rubicellus

Taxon classificationAnimaliaAraneaePhilodromidae

Mello-Leitão, 1929

Thanatus rubicellus Cokendolpher et al. 1979: 729; Dondale et al. 1964: 648 [S], mf, desc. (figs 5–13, 45–54); Jackman 1997: 166; Roberts 2001: 50; Vogel 1970b: 27
Thanatus rubicundus Keyserling, 1880; Marx 1890: 558

####### Distribution.

Brazos, Burleson, Clay, Colorado, Coryell, Lavaca, Potter, Travis, Wichita

####### Locality.

Attwater Prairie Chicken National Wildlife Refuge, Texas A&M University Rangeland Area, Wildcat Bluff Nature Center

####### Time of activity.

Male (May – June, October); female (May – July)

####### Habitat.

(plants: miscellaneous vegetation); (soil/woodland: post oak savanna with pasture)

####### Method.

pitfall trap [mf]; sweeping [m]

####### Type.

Georgia

####### Etymology.

Latin, red cell

####### Collection.

MSU, TAMU

###### Thanatus vulgaris

Taxon classificationAnimaliaAraneaePhilodromidae

Simon, 1870

Thanatus vulgaris Brown 1974: 238; Dondale et al. 1964: 653, mf, desc. (figs 3–4, 41–42); Jackman 1997: 166

####### Distribution.

Culberson, Dallas, Denton, Presidio, Shelby

####### Time of activity.

Female (March, May)

####### Habitat.

(landscape features: under rock); (structures: warehouse)

####### Type.

Spain and Italy

####### Etymology.

Latin, common

####### Collection.

MSU

##### Genus *Tibellus* Simon, 1875

###### Tibellus duttoni

Taxon classificationAnimaliaAraneaePhilodromidae

(Hentz, 1847)

Tibellus duttoni Agnew et al. 1985: 5, 11; Breene 1988: 35–36; Breene et al. 1989: 163; Breene et al. 1993b: 648; Breene et al. 1993c: 22, 48, 77, mf (figs 74A-B); Chickering 1940: 233; Cokendolpher et al. 1979: 729; Cokendolpher et al. 2008: 10, 48 (photo 30); Dean and Eger 1986: 142; Dean et al. 1982: 255; Dean et al. 1988: 287; Gertsch 1933a: 11, mf, desc. (figs 4–6); Irungu 2007: 31; Jackman 1997: 121, desc., 166 (photo 39c); Jones 1936: 69; Kaston 1953: 104, desc.; Kaston 1972: 248, desc.; Kaston 1978: 240, desc.; Nyffeler et al. 1992c: 2; Pamanes-Guerrero 1975: 17, 34, 38, 42, 60, 81; Rapp 1984: 8; Roberts 2001: 50; Tugmon et al. 1990: 44; Vogel 1970b: 27; Young and Edwards 1990: 21

####### Distribution.

Archer, Atascosa, Bastrop, Bell, Bexar, Brazoria, Brazos, Brown, Burleson, Burnet, Cameron, Camp, Carson, Clay, Coleman, Collin, Colorado, Coryell, Dallas, Denton, DeWitt, Erath, Falls, Fayette, Galveston, Glasscock, Hays, Hidalgo, Hill, Houston, Hunt, Jim Wells, Kendall, Kerr, Knox, Lampasas, Lavaca, Leon, Llano, Lynn, McMullen, Potter, Rains, Robertson, Runnels, San Patricio, San Saba, Somervell, Starr, Sterling, Titus, Travis, Val Verde, Victoria, Walker, Wichita, Willacy, Williamson, Zapata

####### Locality.

Attwater Prairie Chicken National Wildlife Refuge, Ellis Prison Unit, Falcon State Park, Inks Lake State Park, Padre Island, Pantex Lake, Ramsey Prison Farm, Seminole Canyon State Park, South Padre Island, Stiles Farm Foundation, Texas A&M University Rangeland Area, Wildcat Bluff Nature Center

####### Time of activity.

Male (January – October, December); female (January – December)

####### Habitat.

(crops: cabbage, cotton, peanuts, sugarcane); (grass: grass, grasses, grassland, pasture, *Hordeum pusillum*); (littoral: dune, edge of pond, grass in sand dunes, playa); (nest/prey: mud dauber nest [f]); (plants: bluebonnets, Indian paintbrush, miscellaneous vegetation, roadside vegetation, *Aphanostephus* sp., *Cassia* sp., *Monarda citriodora*, *Rudbeckia* sp., *Thelesperma* sp., *Vicia* sp.); (soil/woodland: juniper, open field, under oak, post oak savanna with pasture, sedge, *Juniperus ashei*, *Prosopis grandulosa*, *Ulmus crassifolia*)

####### Method.

Beating [m]; D-Vac suction [mf]; pitfall trap [mf] (edge of pond [m], under oak [f]); sweeping [mf]

####### Type.

Georgia

####### Etymology.

Person (discoverer)

####### Collection.

DMNS, MSU, TAMU

###### Tibellus oblongus

Taxon classificationAnimaliaAraneaePhilodromidae

(Walckenaer, 1802)

Tibellus oblongus Jackman 1997: 166; Rapp 1984: 8; Rogers and Horner 1977: 523; Rydzak and Killebrew 1982: 7; Woods and Harrel 1976: 44; Young and Edwards 1990: 21 [Dondale and Redner 1978b: 99, mf, desc. (figs 63, 73, 328–332)]

####### Distribution.

South Texas; Archer, Baylor, Brewster, Brown, El Paso, Galveston, Jefferson, Montague, Smith, Wichita, Young

####### Locality.

Ascarate Lake

####### Time of activity.

Male (July); female (July)

####### Habitat.

(crops: guar, rice); (grass: grasses and marsh like vegetation, grassy and shrub area); (littoral: salt marsh area); (soil/woodland: sandy area)

####### Method.

pitfall trap; sweeping [mf]

####### Type.

unknown

####### Etymology.

Latin, shape of abdomen

####### Collection.

DMNS, MSU

##### Genus *Titanebo* Gertsch, 1933

###### Titanebo albocaudatus

Taxon classificationAnimaliaAraneaePhilodromidae

(Schick, 1965)

Titanebo albocaudatus Muster 2009: 54 [T], m (fig. 3)
Ebo albocaudatus Schick, 1965; Agnew et al. 1985: 5; Cokendolpher et al. 1979: 724; Jackman 1997: 166; Knutson et al. 2010: 515; Sauer and Platnick 1972: 52, mf, desc. (figs 24, 27); Young and Edwards 1990: 20

####### Distribution.

Andrews, Coryell, Erath, Howard, Kimble, Llano, Martin, Maverick, Taylor, Val Verde, Webb, Wichita

####### Time of activity.

Male (May – August); female (July – October)

####### Habitat.

(crops: cotton, peanuts); (grass: grass); (plants: miscellaneous vegetation, sage, *Ambrosia* sp., *Liatris mucronata*, *Prionopsis ciliata*, *Thelesperma* sp.); (soil/woodland: post oak savanna with pasture, saltcedar)

####### Method.

pitfall trap [mf]; sweeping [f]

####### Type.

California, Victorville

####### Etymology.

Latin, white area on dorsum of abdomen

####### Collection.

MSU, NMSU, TAMU

###### Titanebo mexicanus

Taxon classificationAnimaliaAraneaePhilodromidae

(Banks, 1898)

Titanebo mexicanus Muster 2009: 54 [T]
Ebo mexicanus Banks, 1898; Jackman 1997: 120, 166; Kaston 1978: 236, desc.; Sauer and Platnick 1972: 50, mf, desc. (figs 31, 33)

####### Distribution.

Western 2/3 Texas; Archer, Armstrong, Baylor, Brewster, Dallas, Hidalgo, Hudspeth, Tom Green, Val Verde, Winkler, Zapata

####### Locality.

Big Bend National Park, Palo Duro Canyon State Park

####### Time of activity.

Male (April)

####### Type.

Mexico, Hermosillo

####### Etymology.

locality (country)

####### Collection.

DMNS

###### Titanebo parabolis

Taxon classificationAnimaliaAraneaePhilodromidae

(Schick, 1965)

Titanebo parabolis Muster 2009: 54 [T]
Ebo parabolis Schick, 1965; Broussard and Horner 2006: 255 [Sauer and Platnick 1972: 56, mf, desc. (figs 37, 40)]

####### Distribution.

Brewster, Presidio

####### Locality.

Chihuahuan desert, Dalquest Research Site

####### Method.

pitfall trap [mf]

####### Type.

California, Eagle Lake

####### Etymology.

Latin, shape (parabolic)

####### Collection.

MSU

###### Titanebo redneri

Taxon classificationAnimaliaAraneaePhilodromidae

(Cokendolpher, 1978)

Titanebo redneri Calixto et al. 2013: 184; Muster 2009: 54 [T]
Ebo redneri Cokendolpher, 1978; Cokendolpher 1978a: 227, mf, desc. (figs 1–2); Cokendolpher et al. 1979: 725; Jackman 1997: 166

####### Distribution.

Archer, Comanche, Wichita

####### Locality.

Bill Haney Pecan Orchard, Lake Wichita

####### Time of activity.

Male (March, September – November); female (February, September – December)

####### Habitat.

(orchard: pecan); (soil/woodland: mesquite, *Prosopis grandulosa*, *Prosopis juliflora*)

####### Method.

cardboard band [m]

####### Eggs/spiderlings.

Archer or Wichita [13 eggs, 9 spiderlings] [Cokendolpher et al. 1979: 725]

####### Type.

Texas (male, Wichita Co., Wichita Falls, November 18, 1976, J. C. Cokendolpher, holotype, AMNH)

####### Etymology.

Person (This species is named in honor of J. H. Redner of the Biosystematics Research Institute, Ottawa, Canada, in recognition of his work on the Philodromidae and his assistance in the determinations of crab spiders from Wichita County, Texas, Cokendolpher 1978a).

####### Collection.

MSU, TAMU

###### Titanebo texanus

Taxon classificationAnimaliaAraneaePhilodromidae

Gertsch, 1933

Titanebo texanus Bonnet 1959: 4626; Gertsch 1933b: 13, m, desc. (fig. 10); Muster 2009: 54 [T]; Roewer 1955: 802; Vogel 1970b: 28
Ebo texanus (Gertsch, 1933); Jackman 1997: 166; Sauer and Platnick 1972: 55, mf, desc. (figs 32, 34)

####### Distribution.

Brooks, Maverick, Presidio, Travis

####### Time of activity.

Male (March – April, September); female (April)

####### Habitat.

(plants: *Baccharis*); (soil/woodland: saltcedar)

####### Type.

Texas (male, Travis Co., Austin, no date, no collector, holotype, AMNH)

####### Etymology.

locality (state)

####### Collection.

TAMU

#### Family Pholcidae C. L. Koch, 1850

**Note.** Species incorrectly reported from Texas

*Metagonia caudata* O. P.-Cambridge, 1895; Gertsch 1977: 105, mf, desc. (figs 1–4, 7–8, 19–20); Gertsch 1986: 41, f (figs 1–2); Jackman 1997: 166

**Remarks.** One record from Hidalgo Co. in Mexican banana bunch (April, 1, 1936)

**Type.** Mexico, Tabasco, Teapa

**Note.** Not listed here because it is not established.

##### Genus *Chisosa* Huber, 2000

###### Chisosa diluta

Taxon classificationAnimaliaAraneaePholcidae

(Gertsch & Mulaik, 1940)

Chisosa diluta Huber 2000: 125 [T], mf, desc. (figs 151, 478–489); Huber 2005: 195
Pholcophora diluta Gertsch and Mulaik, 1940; Gertsch 1982: 100, mf, desc. (figs 19–21, 28–30); Gertsch and Mulaik 1940: 320, f, desc. (figs 27–28); Jackman 1997: 166; Vogel 1967: 112; Vogel 1970b: 16

####### Distribution.

Brewster

####### Locality.

Big Bend National Park

####### Time of activity.

Male (August); female (June, August)

####### Type.

Texas (female, Brewster Co., Hot Springs, June 7–10, 1938, D. and S. Mulaik, holotype, AMNH)

####### Etymology.

Latin, tiny, diluo, dilute

##### Genus *Crossopriza* Simon, 1893

###### Crossopriza lyoni

Taxon classificationAnimaliaAraneaePholcidae

(Blackwall, 1867)

Crossopriza lyoni Edwards 1993: 1; Huber et al. 1999: 2 [S], mf, desc. (figs 1–12); Roth 1994: 145
Crossopriza stridulans Millot, 1946; Jackman 1997: 166; MacKay and Vinson 1989: 232; Roth 1985: B-33–1

####### Distribution.

Brazos, Hidalgo, McLennan, Wichita

####### Time of activity.

Male (May, July – August, October – November); female (January, May, July – October)

####### Habitat.

(structures: on house eave, in warehouse, quonset hut)

####### Type.

India

####### Etymology.

Person (collector, Captain Francis Lyon)

####### Collection.

MSU, TAMU

##### Genus *Micropholcus* Deeleman-Reinhold & Prinsen, 1987

###### Micropholcus fauroti

Taxon classificationAnimaliaAraneaePholcidae

(Simon, 1887)

Micropholcus fauroti Deeleman-Reinhold and Prinsen 1987: 73 [S, T], mf, desc. (figs 1–9); Huber 2000: 8; Huber 2003: 611; Huber 2011: 26, mf, desc. (figs 1, 30, 31, 48, 49, 83–101); Jackman 1997: 166; Roth 1994: 146
Pholcus fauroti Simon, 1887; Roth 1985: B-33–1
Pholcus unicolor Petrunkevitch, 1929; Gertsch 1937: 1; Gertsch and Mulaik 1940: 319; Roth 1982: 37–2; Vogel 1970b: 16

####### Distribution.

Cameron, Hidalgo

####### Time of activity.

Male (January – February, September); female (February – March)

####### Habitat.

(structures: high school building)

####### Type.

Gulf of Aden

####### Etymology.

Person

##### Genus *Modisimus* Simon, 1893

###### Modisimus texanus

Taxon classificationAnimaliaAraneaePholcidae

Banks, 1906

Modisimus texanus Banks 1906: 94, f, desc.; Banks 1910: 7; Bonnet 1957: 2970; Cokendolpher and Reddell 2001b: 53; Comstock 1912: 327; Comstock 1940: 341, desc.; Gertsch and Mulaik 1940: 321; Huber 1998: 58, mf, desc. (figs 182–191); Jackman 1997: 166; Petrunkevitch 1911: 160; Reddell and Cokendolpher 2004: 91; Roewer 1942: 338; Roth 1994: 145; Vogel 1970b: 16

####### Distribution.

Bandera, Bell, Bexar, Cameron, Comal, Hays, Hidalgo, Kendall, Llano, Mason, Nueces, Robertson, San Saba, Starr, Travis, Uvalde, Webb, Zapata

####### Locality.

Fort Hood, Lower Rio Grande Valley National Wildlife Refuge, Sabal Palm Audubon Sanctuary

####### Caves.

**Bell** ([all Fort Hood] Sledgehammer Cave, Viper Den Cave); **Bexar** (Boneyard Pit, Bullis Hole, Linda’s First Cave, NBC Cave, Niche Cave, Obvious Little Cave, Poison Ivy Pit, Record Fire 1 Cave, World Newt Cave, Wurzbach Bat Cave); **Comal** (Bender’s Cave, Brehmmer Cave, Just Now Cave, Klar’s Cave); **Hays** (Donaldson Cave); **Kendall** (Pfeiffer’s Water Cave); **Llano** (Enchanted Rock Cave); **San Saba** (Cobweb Fissure); **Travis** (Deer Stand Cave, Dobie Shelter, New Comanche Trail Cave, Rockpile Cave); **Uvalde** (Moss Pit Cave)

####### Time of activity.

Male (January – July, September – November); female (January – September, November – December)

####### Habitat.

(landscape features: cave); (soil/woodland: ebony-guayacan association, palm forest)

####### Method.

Flight intercept trap on ground [m]

####### Type.

Texas (female, Travis Co., Austin, March, J. H. Comstock, holotype)

####### Etymology.

locality (state)

####### Collection.

DMNS, TAMU, TMM

##### Genus *Pholcophora* Banks, 1896

###### Pholcophora texana

Taxon classificationAnimaliaAraneaePholcidae

Gertsch, 1935

Pholcophora texana Gertsch 1935a: 11, m, desc. (figs 22–24); Gertsch 1982: 100, mf, desc. (figs 16–18, 25–27); Gertsch and Mulaik 1940: 319 [see note below]; Huber 2000: 117, mf, desc. (figs 443–447); Huber 2005: 195; Jackman 1997: 166; Roewer 1942: 338; Vogel 1970b: 16

####### Distribution.

Hidalgo, Starr

####### Time of activity.

Male (November); female (January, November)

####### Habitat.

(structures: brick yard)

####### Type.

Texas (male, Starr Co., 0.5 mile E Rio Grande City, November 11, 1934, S. Mulaik, holotype, AMNH)

####### Etymology.

locality (state)

####### Note.

5 miles E Rio Grande City (Huber 2000) is 0.5 mile.

##### Genus *Pholcus* Walckenaer, 1805

###### Pholcus phalangioides

Taxon classificationAnimaliaAraneaePholcidae

(Fuesslin, 1775)

Pholcus phalangioides Gertsch and Mulaik 1940: 319; Jackman 1997: 38, desc., 166 [Huber 2011: 375, mf, desc. (figs 1760–1762, 1790–1791, 1819–1822)]
Pholcophora phalangioides (Fuesslin, 1775); Vogel 1970b: 16

####### Distribution.

Travis, Wichita

####### Time of activity.

Female (October)

####### Type.

Switzerland

####### Etymology.

Latin, daddy-long-legs like

####### Collection.

MSU

##### Genus *Physocyclus* Simon, 1893

###### Physocyclus enaulus

Taxon classificationAnimaliaAraneaePholcidae

Crosby, 1926

Physocyclus enaulus Barr and Reddell 1967: 259; Cokendolpher 1989: 475; Gertsch 1935a: 11; Gertsch 1939b: 24; Gertsch and Mulaik 1940: 320; Jackman 1997: 166; Reddell 1965: 175; Reddell 1970: 407; Reddell and Fieseler 1977: 95; Reddell and Smith 1965: 62; Valdez-Mondragón 2010: 21, mf, desc. (figs 29–41); Valdez-Mondragón 2013: 192; Vogel 1970b: 16
Physocyclus globosus Taczanowski, 1874; Jackman 1997: 166; Jones 1936: 69; Vogel 1970b: 16 [Texas records, misidentified]

####### Distribution.

Anderson, Andrews, Archer, Atascosa, Bandera, Brewster, Brown, Cass, Clay, Coryell, Cottle, Crockett, Culberson, Dallas, Edwards, El Paso, Goliad, Hidalgo, Hudspeth, Kaufman, Kinney, Live Oak, Llano, Montague, Pecos, Presidio, Real, Schleicher, Starr, Sutton, Terrell, Trinity, Uvalde, Val Verde, Victoria, Webb, Wichita, Zapata

####### Locality.

Big Bend National Park, Big Bend Ranch State Park, Chisos Basin, Chisos Mountains

####### Caves.

**Bandera** (Tucker’s Fissure Cave); **Brewster** (Javelina Hole, Lichnovsky’s Cave, O.T.L. Cave); **Crockett** (Ketchum Cave); **Culberson** (Dillahunty Swallow Cave, Grass Cave, Grassy Grotto, Harvestman Fissure, Spare Tires Cave, Windy Cave); **Edwards** (Punkin Cave, Wheat Cave No. 1); **El Paso** (Helm’s West Well); **Kinney** (Cot Cave); **Llano** (Double Door Cave); **Pecos** (Amazing Maze Cave); **Presidio** (John’s Guano Mine); **Real** (Turkey Pens Cave); **Schleicher** (Fartz Cave); **Sutton** (Alma’s Cave, Silky Cave, Word Cave); **Terrell** (Sorcerer’s Cave); **Uvalde** (Tampke Ranch Cave); **Val Verde** (Comstock Railroad Tunnel, Litter Barrel Cave, Plecotus Cave)

####### Time of activity.

Male (January – December); female (January – December)

####### Habitat.

(landscape features: cave, cave corner, under rock); (nest/prey: in animal burrow); (structures: in restroom near floor)

####### Type.

New Mexico

####### Etymology.

Greek, dwelling in dens

####### Collection.

DMNS, MSU, NMSU, TAMU, TMM

###### Physocyclus hoogstraali

Taxon classificationAnimaliaAraneaePholcidae

Gertsch & Davis, 1942

Physocyclus hoogstraali Jackman 1997: 166; Reddell 1970: 407; Valdez-Mondragón 2010: 30, mf, desc. (figs 56–62); Vogel 1970b: 16

####### Distribution.

Val Verde

####### Time of activity.

Male (April)

####### Habitat.

(landscape features: Cave near Pandale Crossing)

####### Type.

Mexico, Nuevo Leon, Sabinas Hidalgo, Bat Cave

####### Etymology.

Person (collector, H. Hoogstraal)

####### Collection.

TMM

###### Physocyclus tanneri

Taxon classificationAnimaliaAraneaePholcidae

Chamberlin, 1921

Physocyclus tanneri Valdez-Mondragón 2010: 45, mf, desc, (Figs 105–111)

####### Distribution.

Travis

####### Time of activity.

Female (February)

####### Type.

Utah, St. George

####### Etymology.

Person (V. L. Tanner)

##### Genus *Psilochorus* Simon, 1893

###### Psilochorus concolor

Taxon classificationAnimaliaAraneaePholcidae

Slowik, 2009

Psilochorus concolor Slowik 2009: 16, mf, desc. (figs 56–66, 190)

####### Distribution.

Brewster

####### Locality.

Big Bend National Park, Chisos Basin, Chisos Mountains

####### Time of activity.

Male (March, August); female (March, August)

####### Type.

Texas (male, Brewster Co., Big Bend National Park, Chisos Mountains, Cat Tail Canyon, March 20, 1977, Roth-Schroepfer, holotype, AMNH)

####### Etymology.

Latin, lack of typical coloration found in this species

###### Psilochorus imitatus

Taxon classificationAnimaliaAraneaePholcidae

Gertsch & Mulaik, 1940

Psilochorus imitatus Gertsch and Mulaik 1940: 321, mf, desc. (fig. 17); Jackman 1997: 166; Schoenly 1983: 793; Slowik 2009: 22, mf, desc. (figs 2–4, 82–91); Vogel 1967: 112; Vogel 1970b: 16

####### Distribution.

Bailey, Blanco, Brewster, Brown, Culberson, Dallam, El Paso, Hemphill, Jack, Knox, Lipscomb, Lubbock, McCulloch, Oldham, Panola, Stephens, Terrell, Tom Green, Val Verde, Wichita, Wilbarger, Winkler

####### Caves.

**Val Verde** (Emerald Sink)

####### Time of activity.

Male (May – August, December); female (February – July, October, December)

####### Habitat.

(grass: short grass); (landscape features: cave); (soil/woodland: mesquite in mesquite dunes)

####### Type.

Texas (male, Terrell Co., Sanderson, July 4, 1934, S. Mulaik, holotype, AMNH)

####### Etymology.

Latin, similar to other species

####### Collection.

DMNS, MCZ, MSU, NMSU, TMM

###### Psilochorus pallidulus

Taxon classificationAnimaliaAraneaePholcidae

Gertsch, 1935

Psilochorus pallidulus Bonnet 1958: 3823; Gertsch 1935a: 13, m, desc. (figs 28–30); Gertsch and Mulaik 1940: 321; Jackman 1997: 166; Roewer 1942: 351; Slowik 2009: 31 [S], mf, desc. (figs 99–107)
Psilochorus pallidus Gertsch, 1935; Vogel 1970b: 16
Psilochorus coahuilanus Gertsch and Davis, 1937; Gertsch and Mulaik 1940: 323; Jackman 1997: 166; Vogel 1970b: 16

####### Distribution.

Archer, Brewster, Clay, Hidalgo

####### Locality.

Big Bend National Park, Chisos Basin, Chisos Mountains

####### Time of activity.

Male (June, August – October, December); female (June, August – September, December)

####### Type.

Texas (male, Hidalgo Co., Edinburg, September-December, 1933, S. Mulaik, holotype, AMNH)

####### Etymology.

Latin, small and pallid

####### Collection.

MSU

###### Psilochorus pullulus

Taxon classificationAnimaliaAraneaePholcidae

(Hentz, 1850)

Psilochorus pullulus Gertsch 1935a: 14; Jackman 1997: 166; Jones 1936: 69; Kaston 1972: 96, desc. (fig. 217); Kaston 1978: 97, desc. (fig. 235); Slowik 2009: 33, mf, desc. (figs 118–127); Vogel 1970b: 16 [Gertsch and Mulaik 1940: 322 (records from West Texas probably redemptus, imitatus and utahensis)]

####### Distribution.

South Texas; Cameron, Dallas, Travis

####### Time of activity.

Male (August – September, November); female (June, October – November)

####### Type.

Alabama

####### Etymology.

Latin, blackish

####### Collection.

DMNS

###### Psilochorus redemptus

Taxon classificationAnimaliaAraneaePholcidae

Gertsch & Mulaik, 1940

Psilochorus redemptus Agnew et al. 1985: 3, 5; Calixto et al. 2013: 184; Gertsch and Mulaik 1940: 322, mf, desc.; Jackman 1997: 166; Slowik 2009: 35, mf, desc. (figs 128–137); Vogel 1967: 113; Vogel 1970b: 16; Young and Edwards 1990: 21

####### Distribution.

Bastrop, Bexar, Brazos, Brewster, Cameron, Comanche, Coryell, Erath, Hidalgo, Jasper, Jeff Davis, Kleberg, Llano, Panola, San Augustine, San Patricio, Starr, Travis, Val Verde, Webb, Zapata

####### Locality.

Bill Haney Pecan Orchard, Chisos Mountains, Green Island Bird Refuge, McDonald Observatory, Welder Wildlife Refuge

####### Time of activity.

Male (January – February, April – December); female (January – December)

####### Habitat.

(crops: peanuts); (objects: under rock); (orchard: pecan); (soil/woodland: juniper, under juniper)

####### Method.

pitfall trap [mf] (under juniper [m])

####### Type.

Texas (male, Hidalgo Co., Edinburg, November 7, 1934, S. Mulaik, holotype, AMNH)

####### Etymology.

Latin, redeemed

####### Collection.

DMNS, TAMU

###### Psilochorus texanus

Taxon classificationAnimaliaAraneaePholcidae

Slowik, 2009

Psilochorus texanus Slowik 2009: 41, mf, desc. (figs 158–166, 190)

####### Distribution.

Hidalgo, Kenedy

####### Time of activity.

Male (July); female (July, November)

####### Type.

Texas (male, Kenedy Co., Padre Island, seashore park, July 19, 1966, J. & W. Ivie, holotype, AMNH)

####### Etymology.

locality (The specific epithet refers to the state of the type locality, Slowik 2009).

###### Psilochorus utahensis

Taxon classificationAnimaliaAraneaePholcidae

Chamberlin, 1919

Psilochorus utahensis Jackman 1997: 166; Schoenly and Reid 1983: 256; Slowik 2009: 44, mf, desc. (figs 176–186)

####### Distribution.

Blanco, Coleman, Coryell, El Paso, Hidalgo

####### Locality.

Chihuahuan desert, Horne Ranch

####### Time of activity.

Male (April – September); female (April, June – September)

####### Habitat.

(grass: grass); (soil/woodland: post oak savanna with pasture)

####### Method.

D-Vac suction [f]; pitfall trap [mf]

####### Type.

Utah, Clear Lake

####### Etymology.

locality (state)

####### Collection.

TAMU

##### Genus *Smeringopus* Simon, 1890

###### Smeringopus pallidus

Taxon classificationAnimaliaAraneaePholcidae

(Blackwall, 1858)

Smeringopus pallidus Edwards 1993: 1; Jackman 1997: 166; Roth 1994: 146 [Kraus 1957: 219 [S], mf, desc. in German (figs 1–6)]
Smeringopus elongatus (Vinson, 1863); Brown 1974: 235; Gertsch 1979: 150; Roth 1982: 37–1; Roth 1985: B-33–1

####### Distribution.

Shelby

####### Habitat.

(structures: warehouse)

####### Type.

Brazil

####### Etymology.

Latin, pale (pallid)

##### Genus *Spermophora* Hentz, 1841

###### Spermophora senoculata

Taxon classificationAnimaliaAraneaePholcidae

(Dugès, 1836)

Spermophora senoculata Jackman 1997: 166; Platnick 1993: 130 [S]
Spermophora meridionalis Hentz, 1841; Gertsch and Mulaik 1940: 319; Jones 1936: 69; Vogel 1970b: 16 [Petrunkevitch 1910: 208, m (pl. 21, fig. 5)]

####### Distribution.

Blanco, Brazos, Cooke, Dallas, Harris, Travis

####### Locality.

Bamburger Ranch Chiroptorium

####### Time of activity.

Male (June, August); female (August, December)

####### Habitat.

(structures: on ceilings in home at night, sticky trap in garage)

####### Method.

sticky trap [f]

####### Type.

unknown

####### Etymology.

Latin, six eyes

####### Collection.

MCZ, MSU, TAMU, UNM

#### Family Phrurolithidae Banks, 1892

**Note.** raised to family (Ramirez 2014: 342)

##### Genus *Phrurolithus* C. L. Koch, 1839

**Note.** transferred from Liocranidae to Corinnidae (Bosselaers and Jocqué 2002: 265) and here (Ramírez 2014: 343)

###### Phrurolithus apertus

Taxon classificationAnimaliaAraneaeCorinnidae

Gertsch, 1935

Phrurolithus apertus Bonnet 1958: 3633; Gertsch 1935b: 1, mf, desc. (figs 1–3); Roewer 1955: 574 [T]; Vogel 1970b: 6
Phrurotimpus apertus (Gertsch, 1935); Jackman 1997: 165

####### Distribution.

Cameron

####### Time of activity.

Male (November); female (November)

####### Type.

Texas (female, Cameron Co., 15 miles SW Harlingen, November 17, 1934, S. Mulaik, holotype, AMNH)

####### Etymology.

Latin, aperture of epigynum

###### Phrurolithus callidus

Taxon classificationAnimaliaAraneaeCorinnidae

Gertsch, 1935

Phrurolithus callidus Bonnet 1958: 3633; Gertsch 1935b: 5, f, desc. (fig. 9); Roewer 1955: 575 [T]; Vogel 1970b: 6
Phrurotimpus callidus (Gertsch, 1935); Jackman 1997: 165

####### Distribution.

Cameron, Hidalgo

####### Time of activity.

Female (October – November)

####### Type.

Texas (female, Hidalgo Co., 10 miles SE Edinburg, October 20, 1934, S. Mulaik, holotype, AMNH)

[male unknown]

####### Etymology.

Latin, cunning

###### Phrurolithus emertoni

Taxon classificationAnimaliaAraneaeCorinnidae

Gertsch, 1935

Phrurolithus emertoni Henderson 2007: 60, 64, 76, 79, 82 [Gertsch 1935b: 9, f, desc. (fig. 20)]
Phrurotimpus emertoni (Gertsch, 1935); Agnew et al. 1985: 8; Jackman 1997: 165

####### Distribution.

Brazos, Erath

####### Locality.

Lick Creek Park

####### Time of activity.

Female (May 27-June 15, June, June 30-July 15)

####### Habitat.

(soil/woodland: disturbed habitat, leaf litter)

####### Method.

pitfall trap [f]

####### Type.

Georgia, Atlanta

[male unknown]

####### Etymology.

Person (collector, J. H. Emerton)

####### Collection.

TAMU

###### Phrurolithus leviculus

Taxon classificationAnimaliaAraneaeCorinnidae

Gertsch, 1936

Phrurolithus leviculus Bonnet 1958: 3637; Gertsch 1936: 18, mf, desc. (figs 17–18); Roewer 1955: 576 [T]; Vogel 1970b: 6
Phrurotimpus leviculus (Gertsch, 1936); Jackman 1997: 165

####### Distribution.

Hidalgo

####### Time of activity.

Male (June); female (June, September)

####### Type.

Texas (male, Hidalgo Co., Edinburg, June 2, 1935, S. Mulaik, holotype, AMNH)

####### Etymology.

Latin, small, smooth

##### Genus *Phruronellus* Chamberlin, 1921

**Note.** transferred from Liocranidae to Corinnidae (Bosselaers and Jocqué 2002: 265) and here (Ramírez 2014: 343)

###### Phruronellus formica

Taxon classificationAnimaliaAraneaeCorinnidae

(Banks, 1895)

Phruronellus formica Chamberlin 1921: 69 [T]
Phrurolithus formica Banks, 1895; Jackman 1997: 165 [Kaston 1948: 391, mf, desc. (figs 1391–1393)]
Scotinella formica (Banks, 1895); Agnew et al. 1985: 8

####### Distribution.

Archer, Brazos, Brown, Erath, Wichita

####### Locality.

Texas A&M University Rangeland Area

####### Time of activity.

Male (September); female (March, December)

####### Habitat.

(soil/woodland: forest litter, under bark)

####### Method.

Berlese funnel [f]

####### Type.

New York

####### Etymology.

Latin, refers to ants

####### Collection.

TAMU

##### Genus *Phrurotimpus* Chamberlin & Ivie, 1935

**Note.** transferred from Liocranidae to Corinnidae (Bosselaers and Jocqué 2002: 265) and here (Ramírez 2014: 343)

###### Phrurotimpus alarius

Taxon classificationAnimaliaAraneaeCorinnidae

(Hentz, 1847)

Phrurotimpus alarius Agnew et al. 1985: 8; Bonnet 1958: 3642; Henderson 2007: 34, 52–53, 55, 58, 64, 74, 76, 79, 82; Jackman 1997: 165; Kaston 1945: 5; Trevino 2014: 11; Vogel 1970b: 6 [Dondale and Redner 1982: 132, mf, desc. (figs 240–242)]

####### Distribution.

Anderson, Angelina, Bandera, Brazos, Burleson, Coryell, Erath, Gonzales, Houston, Robertson, Sabine, Smith, Travis, Tyler, Walker, Webb, Wichita

####### Locality.

Big Slough Wild Area, Holmes Pecan Orchard, Huntsville State Park, Lick Creek Park, Lost Maples State Park, Palmetto State Park, Tyler State Park

####### Time of activity.

Male (March – May, May 30-June 6, June 30-July 15, July 25-August 1, December); female (January, March – July)

####### Habitat.

(littoral: edge of pond); (soil/woodland: bottomland forest, celtis litter, leaf litter, in leaves, loblolly pine unmanaged, post oak savanna with pasture, post oak woodland, under oak tree, upland woods, woods)

####### Method.

Berlese funnel [f]; pitfall trap [mf] (under oak [f], edge of pond [m], in leaves [mf]); tile trap [f]

####### Type.

Alabama

####### Etymology.

Latin, wing

####### Collection.

MSU, TAMU

###### Phrurotimpus alarius tejanus

Taxon classificationAnimaliaAraneaeCorinnidae

(Chamberlin & Gertsch, 1930)

Phrurotimpus alarius tejanus [Chamberlin and Ivie 1935b: 34 [S], m (fig. 120)]
Phrurolithus tejanus Chamberlin and Gertsch, 1930; Chamberlin and Gertsch 1930: 142, m, desc. (fig. 26)

####### Distribution.

Travis

####### Type.

Texas (male, Travis Co., Austin, no date, R. V. Chamberlin, holotype)

[female unknown]

####### Etymology.

locality (state)

###### Phrurotimpus borealis

Taxon classificationAnimaliaAraneaeCorinnidae

(Emerton, 1911)

Phrurotimpus borealis Agnew et al. 1985: 4; Calixto et al. 2013: 181; Jackman 1997: 165; Trevino 2014: 11; Yantis 2005: 198, 201; Young and Edwards 1990: 16 [Dondale and Redner 1982: 133, mf, desc. (figs 243–248)]

####### Distribution.

Anderson, Bexar, Burleson, Comanche, Coryell, Dallas, Erath, Hidalgo, Houston, Webb, Wichita

####### Locality.

Bill Haney Pecan Orchard

####### Time of activity.

Male (April, May 30 – June 6, June); female (April 26 – May 2, May – June, August, December, December 17 – January 8)

####### Habitat.

(crops: peanuts); (grass: grass); (orchard: pecan); (soil/woodland: leaf litter, pine woods [%: 73, 100], post oak savanna with pasture, woods)

####### Method.

5 gallon bucket trap [mf]; pitfall trap [mf] (in dead leaves [f])

####### Type.

United States

####### Etymology.

Latin, northern

####### Collection.

MCZ, MSU, TAMU

###### Phrurotimpus certus

Taxon classificationAnimaliaAraneaeCorinnidae

Gertsch, 1941

Phrurotimpus certus Broussard and Horner 2006: 253; Henderson 2007: 65, 76, 79, 82; Richman et al. 2011a: 48 [Dondale and Redner 1982: 137, mf, desc. (figs 254–256)]

####### Distribution.

Archer, Brazos, Brewster, Burleson, Clay, Colorado, Coryell, Erath, Presidio, Robertson

####### Locality.

Attwater Prairie Chicken National Wildlife Refuge, Chihuahuan desert, Dalquest Research Site, Holmes Pecan Orchard, Lick Creek Park, Somerville Lake

####### Time of activity.

Male (March 30-April 6, April – August); female (March 29-April 5, April – September, September 28-October 4)

####### Habitat.

(crops: peanuts); (orchard: pecan); (soil/woodland: post oak savanna with pasture, upland woods)

####### Method.

pitfall trap [mf]; ramp trap [m]

####### Type.

Virginia, 5 miles E Luray

####### Etymology.

Latin, certain

####### Collection.

MSU, TAMU

##### Genus *Scotinella* Banks, 1911

**Note.** transferred from Liocranidae to Corinnidae (Bosselaers and Jocqué 2002: 265) and here (Ramírez 2014: 343)

###### Scotinella fratrella

Taxon classificationAnimaliaAraneaeCorinnidae

(Gertsch, 1935)

Scotinella fratrella Calixto et al. 2013: 181; Dondale and Redner 1982: 153 [T], mf, desc. (figs 285–288)
Phrurolithus fratrellus Gertsch, 1935; Bonnet 1958: 3637; Gertsch 1935b: 6, f, desc. (fig. 21); Roewer 1955: 575; Vogel 1970b: 6 [Penniman 1978: 127, mf, desc. (figs 1–4, 9–12, 15–16)]
Scotinella fratrellus (Gertsch, 1935); Jackman 1997: 165

####### Distribution.

Bell, Burleson, Coryell, Houston, Robertson, Walker

####### Locality.

Ellis Prison Unit, Holmes Pecan Orchard

####### Time of activity.

Male (April – October); female (April – November)

####### Habitat.

(crops: cotton); (grass: pasture); (orchard: pecan); (soil/woodland: post oak savanna with pasture, woods)

####### Method.

cardboard band [f]; pitfall trap [mf]; ramp trap [m]

####### Type.

Texas (female, Bell Co., Belton, September 1, 1933, W. Ivie, holotype, AMNH)

####### Etymology.

Latin, small brother

####### Collection.

TAMU

###### Scotinella pugnata

Taxon classificationAnimaliaAraneaeCorinnidae

(Emerton, 1890)

Scotinella pugnata Broussard and Horner 2006: 253; Richman et al. 2011a: 48 [Dondale and Redner 1982: 147, mf, desc. (figs 12, 258–265)]

####### Distribution.

Brewster, Presidio

####### Locality.

Chihuahuan desert, Dalquest Research Site

####### Method.

pitfall trap [m]

####### Type.

Massachusetts and Connecticut

####### Etymology.

Latin, fighting, fist

####### Collection.

MSU

#### Family Pisauridae Simon, 1890

**Note.** Species incorrectly reported from Texas

*Pisaurina undulata* (Keyserling, 1887) [not in Texas]

*Pelopatis undulata* (Keyserling, 1887); Comstock 1940: 624

*Pelopatis indulata* (Keyserling, 1887); Vogel 1970b: 17

##### Genus *Dolomedes* Latreille, 1804

###### Dolomedes albineus

Taxon classificationAnimaliaAraneaePisauridae

Hentz, 1845

Dolomedes albineus Agnew et al. 1985: 7; Bradley 2013: 180; Carico 1970: 66; Carico 1973: 462 [S], mf, desc. (figs 5–7, 27, 42, 57–58); Jackman 1997: 167; Yantis 2005: 196, 200
Teippus pinicola (Hentz, 1850); Roewer 1955: 140

####### Distribution.

North-central, central and southeast Texas; Anderson, Austin, Brazos, Burleson, Comal, Comanche, Dallas, Houston, Leon, Navarro, Robertson, Tarrant

####### Locality.

Benbrook-Grissom Ranch, Holmes Pecan Orchard, Lick Creek Park

####### Time of activity.

Male (May – July); female (June – August)

####### Habitat.

(littoral: near lake); (orchard: pecan); (soil/woodland: pine woods [%: 80, 100]); (structures: ground next to house, on [porch outside house, wall], side of house)

####### Method.

5 gallon bucket trap [m]; uv light [imm.]

####### Type.

Alabama

####### Etymology.

Latin, white

####### Collection.

TAMU

###### Dolomedes scriptus

Taxon classificationAnimaliaAraneaePisauridae

Hentz, 1845

Dolomedes scriptus Bradley 2013: 180; Carico 1970: 83; Carico 1973: 469, mf, desc. (figs 8–10, 22–24, 28, 43, 59–60); Dondale and Redner 1990: 342, mf, desc. (figs 575–579); Jackman 1997: 91, 167; Kaston 1978: 179 (fig. 450); McKenzie and Reddell 1964: 7; Reddell 1965: 175; Reddell 1970: 407; Vogel 1970b: 17; Woods and Harrel 1976: 43; Yantis 2005: 196, 200; Young and Edwards 1990: 21
Dolomedes pinicola Hentz, 1850; Bishop and Crosby 1936: 239 [misidentified]

####### Distribution.

Southeast and southwest Texas; Anderson, Bell, Dimmit, Harris, Hidalgo, Houston, Jefferson, Kerr, Travis, Trinity, Wichita, Young

####### Caves.

**Bell** (Adam’s Gold Mine); **Travis** (West Cave)

####### Time of activity.

Male (April, June)

####### Habitat.

(crops: rice); (landscape features: cave); (soil/woodland: pine woods [%: 74, 83, 97])

####### Method.

5 gallon bucket trap [m]

####### Type.

Alabama

####### Etymology.

Latin, written (pattern)

####### Collection.

MSU, TAMU, TMM

###### Dolomedes tenebrosus

Taxon classificationAnimaliaAraneaePisauridae

Hentz, 1844

Dolomedes tenebrosus Agnew et al. 1985: 7; Bishop 1924: 40, mf (pl. 18, fig. 2, pl. 21, figs 1–5); Bradley 2013: 181; Brown 1974: 235; Carico 1970: 37; Carico 1973: 454, mf, desc. (figs 1–2, 25, 40, 53–54); Dondale and Redner 1990: 335 (figs 558–562); Griswold 1993: 7; Jackman 1997: 90, desc., 167 (photo 23a); Kaston 1978: 179 (fig. 451); Taber and Fleenor 2005: 273 (fig. 12–1); Woods and Harrel 1976: 43; Yantis 2005: 196, 200; Young and Edwards 1990: 21

####### Distribution.

Southeast Texas; Anderson, Archer, Brown, Erath, Gonzales, Hunt, Jefferson, Leon, Liberty, Nacogdoches, Polk, Trinity, Wichita, Wood, Young

####### Locality.

Palmetto State Park

####### Time of activity.

Male (April – June); female (April, June – July)

####### Habitat.

(crops: rice); (littoral: at water edge, under rocks along creek, wetlands); (soil/woodland: oak wood, pine woods [%: 80, 97, 100])

####### Method.

5 gallon bucket trap [mf]

####### Type.

Alabama, Carolina (of 1844), Massachusetts

####### Etymology.

Latin, darkness

####### Collection.

MSU, TAMU

###### Dolomedes triton

Taxon classificationAnimaliaAraneaePisauridae

(Walckenaer, 1837)

Dolomedes triton Bonnet 1956: 1541; Breene et al. 1993c: 22, 48, 93, mf (figs 126A-C); Carico 1970: 151; Carico 1973: 481 [S], mf, desc. (figs 11, 18–19, 33–34, 37, 48, 69–70); Comstock 1940: 631; Dean et al. 1982: 254; Jackman 1997: 91, 167; Jones 1936: 69; Taber and Fleenor 2005: 273 (fig. 12–2); Young and Edwards 1990: 21
Dolomedes sexpunctatus Hentz, 1845; Brown 1974: 235; Comstock 1912: 614, desc.; Jones 1936: 69; Kaston 1953: 137, desc. (fig. 340); Montgomery 1904: 314, mf, desc. (pl. 20, fig. 34); Woods and Harrel 1976: 43
Dolomedes triton sexpunctatus Hentz, 1845; Bishop 1924: 52, mf (pl. 29, figs 1–2); Vogel 1970b: 17
Dolomedes albiclavius Bishop, 1924; Jones 1936: 69; Vogel 1970b: 16

####### Distribution.

Widespread; Archer, Bandera, Bexar, Brazos, Cameron, Dallas, Denton, Fannin, Gonzales, Harris, Hidalgo, Hood, Jackson, Jefferson, Kerr, Leon, Marion, Nacogdoches, Palo Pinto, Sabine, Travis, Wichita

####### Locality.

Mill Creek Cove, Palmetto State Park, Raven Ranch

####### Time of activity.

Male (February, June – August); female (February – April, June – September, November)

####### Habitat.

(crops: cotton, rice); (littoral: wetlands); (soil/woodland: beech bottom, on ground in woods)

####### Method.

Malaise trap [f]

####### Type.

Georgia

####### Etymology.

Greek, a triton (pattern)

####### Collection.

MSU, TAMU

###### Dolomedes vittatus

Taxon classificationAnimaliaAraneaePisauridae

Walckenaer, 1837

Dolomedes vittatus Bradley 2013: 182; Carico 1970: 113; Carico 1973: 474 [S], mf, desc. (figs 14–15, 30, 36, 45, 63–64); Dondale and Redner 1990: 339, mf, desc. (figs 569–574); Jackman 1997: 167; Kaston 1978: 180 (fig. 453)
Dolomedes urinator Hentz, 1845; Bishop 1924: 36, f (pl. 16; pl. 33, fig. 3); Montgomery 1904: 317

####### Distribution.

Northeast, central, and southeast Texas; Bandera, Bexar, Dallas, Harris, Hays, Kerr, Llano, Travis

####### Locality.

Lake Austin, Raven Ranch

####### Time of activity.

Male (August); female (June – July, October)

####### Type.

Georgia, Ogechee River Swamp

####### Etymology.

Latin, striped

####### Collection.

MCZ

##### Genus *Pisaurina* Simon, 1898

###### Pisaurina dubia

Taxon classificationAnimaliaAraneaePisauridae

(Hentz, 1847)

Pisaurina dubia Breene et al. 1993b: 648; Carico 1972: 308 [T], mf, desc. (figs 11–12, 16, 23–24); Jackman 1997: 167; Yantis 2005: 66, 198, 201
Thanatidius dubius Hentz, 1847; Bishop and Crosby 1936: 242, m, desc. (figs 4–5)

####### Distribution.

Central, southeast, and south Texas; Cameron, Guadalupe, Hidalgo, Houston, Kerr, Trinity, Wichita

####### Locality.

Sabal Palm Audubon Sanctuary

####### Time of activity.

Male (April – May); female (April, April 26-May 5, June, December)

####### Habitat.

(crops: sugarcane); (soil/woodland: pine woods [%: 66, 80, 82, 84, 97])

####### Method.

5 gallon bucket trap [mf]

####### Eggs/spiderlings.

Cameron [114 spiderlings in case] [TAMU]

####### Type.

unknown

####### Etymology.

Latin, dubious identification, mutilated when discovered

####### Collection.

DMNS, MSU, TAMU

###### Pisaurina mira

Taxon classificationAnimaliaAraneaePisauridae

(Walckenaer, 1837)

Pisaurina mira Agnew et al. 1985: 4, 9; Bishop 1924: 23, mf (pl. 5, figs 1–2, pl. 6, figs 1–2, pl. 7, fig. 1, pl. 8, figs 1–4, pl. 9, figs 1–6); Bradley 2013: 182; Brown 1974: 236; Bumroongsook et al. 1992: 17; Carico 1972: 300, mf, desc. (figs 1–6, 13, 17–18); Dean et al. 1988: 286; Dondale and Redner 1990: 326, mf, desc. (figs 550–553); Jackman 1997: 91, desc., 167 (photo 23b); Kaston 1953: 138, desc. (fig. 345); Kaston 1972: 190, desc. (fig. 426); Kaston 1978: 181, desc. (fig. 458); Montgomery 1904: 320, desc.; Woods and Harrel 1976: 43; Yantis 2005: 67, 198, 201; Young and Edwards 1990: 21

####### Distribution.

Eastern ½ Texas; Anderson, Archer, Bexar, Brazos, Erath, Grimes, Houston, Hunt, Jefferson, Kerr, Leon, Madison, Nacogdoches, Rusk, Sabine, Travis, Trinity, Upshur, Walker, Wichita

####### Locality.

Lick Creek Park

####### Time of activity.

Male (March – May); female (March – June)

####### Habitat.

(crops: cotton, peanuts, rice); (soil/woodland: beech-magnolia forest, on ground, woods by creek bank, pine woods [%: 66, 67, 69, 82, 85, 86, 95, 97], post oak woods [%: 41, 56, 71, 74, 77, 82, 84, 91, 92, 94, 96]); (structures: side of building near light)

####### Method.

5 gallon bucket trap [mf]; malaise trap [m]

####### Type.

Georgia

####### Etymology.

Latin, wonderful

####### Collection.

MSU, TAMU

##### Genus *Tinus* F. O. P.-Cambridge, 1901

###### Tinus peregrinus

Taxon classificationAnimaliaAraneaePisauridae

(Bishop, 1924)

Tinus peregrinus Bradley 2013: 182; Carico 1976: 68 [T], mf, desc. (figs 2, 10–11, 20–21); Carico 2005: 200; Comstock 1940: 633, desc. (figs 707–708); Jackman 1997: 167; Kaston 1953: 137, desc. (fig. 343); Kaston 1972: 189, desc. (fig. 424); Kaston 1978: 180, desc. (fig. 456); Roth 1982: 38–1; Roth 1985: B-34–1; Roth 1994: 148; Vogel 1970b: 17
Thaumasia peregrina Bishop, 1924; Bishop and Crosby 1936: 243, m, desc. (figs 6–7)

####### Distribution.

South Texas; Brewster, Starr

####### Time of activity.

Male (July)

####### Habitat.

(littoral: near body of water)

####### Type.

Arkansas, Hot Springs [possibly Texas]

####### Etymology.

Latin, pilgrim

#### Family Plectreuridae Simon, 1893

##### Genus *Plectreurys* Simon, 1893

###### Plectreurys tristis

Taxon classificationAnimaliaAraneaePlectreuridae

Simon, 1893

Plectreurys tristis [Gertsch 1958c: 19, mf, desc. (figs 11–13, 37–48, 85–88)]

####### Distribution.

Brewster

####### Type.

Arizona

####### Etymology.

Latin, sad

####### Collection.

MSU

###### Plectreurys

Taxon classificationAnimaliaAraneaePlectreuridae

sp.

Plectreurys data from Dave Richman

####### Distribution.

Presidio

####### Locality.

Big Bend Ranch State Park

####### Habitat.

(web: web under rock)

#### Family Prodidomidae Simon, 1884

##### Genus *Prodidomus* Hentz, 1847

###### Prodidomus rufus

Taxon classificationAnimaliaAraneaeProdidomidae

Hentz, 1847

Prodidomus rufus Bradley 2013: 184; Bryant 1935: 164, f, desc. (figs 1a-d); Bryant 1949a: 22, m, desc. (fig. 1); Comstock 1940: 319, desc.; Cooke 1964: 266, mf, desc. (figs 15, 29–30); Jackman 1997: 167; Jones 1936: 70; Platnick and Baehr 2006: 13, mf, desc. (figs 24–28); Roth 1982: 20–2; Roth 1985: B-16–2; Roth 1994: 151; Ubick 2005d: 204; Vogel 1970b: 17

####### Distribution.

Bexar, Dallas, Denton

####### Time of activity.

Male (December); female (May, November)

####### Habitat.

(landscape features: under stone by side of road); (structures: in house)

####### Type.

Alabama

####### Etymology.

Latin, red

#### Family Salticidae Blackwall, 1841

**Note.** Species incorrectly reported from Texas

*Euophrys monadnock* Emerton, 1891; Hunter 1988: 18 [not in Texas]

*Habronattus agilis* (Banks, 1893); Chickering 1944: 153; Young and Edwards 1990: 21 [not in Texas] [possibly *cognatus* or *fallax*]

*Pellenes agilis* (Banks, 1893); Peckham and Peckham 1909: 535; Petrunkevitch 1911: 680; Vogel 1970b: 18 [misidentified]

*Maevia simoni* Peckham; Bonnet 1957: 2696 [dubious quotation – see Prószyn’ski online catalog (http://salticidae.org/salticid/catalog/maevia.htm)]

*Metaphidippus manni* (Peckham and Peckham, 1901) [not in Texas]

*Metaphidippus imperialis* (Peckham and Peckham, 1888); Kaston 1972: 276

*Myrmarachne albocinctus* (C. Koch, 1846); Peckham and Peckham 1909: 371; Petrunkevitch 1911: 674 [not in Texas]

*Parnaenus cyanidens* (C. L. Koch, 1846); F. O. P.-Cambridge 1901: 288, errata; Peckham and Peckham 1909: 438 [not in United States]

*Phidippus cyanidens* C. L. Koch, 1846; Peckham and Peckham 1901a: 301

*Pelegrina verecunda* (Chamberlin and Gertsch, 1930); Maddison 1996: 299 [not in Texas, map 26 in error, misprint; map 27 for *Pelegrina tillandsiae* correct]

*Pellenes elegans* (Peckham and Peckham, 1901); Vogel 1970b: 18 [not in Texas]

*Pellenes elagus* Peckham and Peckham, 1901; Jones 1936: 69 [not in Texas]

*Habronattus elegans* (Peckham and Peckham, 1901); Milstead 1958: 446 [not in Texas]

*Phanias monticola* (Banks, 1895) [not in Texas]

*Pseudicius monticolus* (Banks, 1895); Roth 1994: 156 [not in Texas]

*Phidippus ardens* Peckham and Peckham, 1901; Hunter 1988: 18, 20 [not in Texas]

*Phidippus johnsoni* Peckham and Peckham, 1883; Kaston 1972: 270; Kaston 1978: 258; Richman and Cutler 1978: 96 [not in Texas]

*Phidippus obscurus* Peckham and Peckham, 1888; Marx 1890: 569 [*Phidippus arizonensis* or *carolinensis*]

*Phidippus purpuratus* Keyserling, 1885; Chickering 1944: 199; Kaston 1953: 112; Peckham and Peckham 1909: 423; Richman and Cutler 1978: 97; Vogel 1970b: 19 [not in Texas]

*Dendryphantes purpuratus* (Keyserling, 1884); Petrunkevitch 1911: 641 [not in Texas]

*Phidippus albomaculatus* Keyserling, 1885; Emerton 1909: 224 [not in Texas]

*Phidippus regius* C. L. Koch, 1846; Kaston 1972: 269; Kaston 1978: 257 [not in Texas]

*Phidippus miniatus* Peckham and Peckham, 1883; Marx 1890: 569; Peckham and Peckham 1888: 15; Peckham and Peckham 1909: 426; Vogel 1970b: 19 [Texas record, interception or mislabeled]

*Dendryphantes miniatus* (Peckham and Peckham, 1883); Petrunkevitch 1911: 636; Roewer 1955: 1204

*Sitticus auricomus* Bryant, 1936; Bonnet 1958: 4069; Jones 1936: 68, 70 [not described]

*Sittacus auricomus* Bryant, 1936; Vogel 1970b: 20 [not described]

*Sitticus rupicola* (C. L. Koch, 1837); Prószyn’ski 1980: 14 [not certain it occurs in North America]; Richman et al. 2011b: 72 (almost certainly erroneous)

*Wala vernalis* Peckham and Peckham, 1893; Jones 1936: 70; Vogel 1970b: 20 [not in United States]

##### Genus *Admestina* Peckham & Peckham, 1888

###### Admestina archboldi

Taxon classificationAnimaliaAraneaeSalticidae

Piel, 1992

Admestina archboldi Jackman 1997: 167; Piel 1992: 278, mf, desc. (figs 5–6, 10, 12–16); Richman et al. 2011b: 3; Richman et al. 2012a: 3; Richman et al. 2012b: 3
Admestina tibialis (C. L. Koch, 1846); Agnew et al. 1985: 8 [misidentified]

####### Distribution.

Brazos, Burleson, Erath, Travis

####### Locality.

Lick Creek Park

####### Time of activity.

Male (April, September 28-October 5, October); female (February – July)

####### Habitat.

(soil/woodland: post oak savanna with pasture, *Juniperus ashei*, *Quercus buckleyi*, *Quercus virginiana*, *Ulmus crassifolia*)

####### Method.

Beating [f]; pitfall trap [m]; suction trap [m]; sweeping [f]

####### Type.

Florida, Archbold Biological Station

####### Etymology.

locality (The species is named after the founder of the Archbold Biological Station where the holotype was collected, Piel 1992).

####### Collection.

TAMU

###### Admestina tibialis

Taxon classificationAnimaliaAraneaeSalticidae

(C. L. Koch, 1846)

Admestina tibialis Breene et al. 1993c: 22, 48, 65, mf (figs 42A-D); Chickering 1944: 143; Comstock 1912: 679, desc.; Comstock 1940: 687; Dean et al. 1982: 255; Hunter 1988: 18–20; Jackman 1997: 167; Peckham and Peckham 1909: 510; Petrunkevitch 1911: 589; Richman and Cutler 1978: 83; Richman et al. 2011b: 3; Richman et al. 2012a: 3; Richman et al. 2012b: 3; Roth 1982: 40–3; Roth 1985: B-36–2; Vogel 1970b: 17; Young and Edwards 1990: 21 [Piel 1992: 272, mf, desc. (figs 3–4, 11)]

####### Distribution.

Brazos, Ellis, Harris, Sabine, Walker

####### Locality.

Ellis Prison Unit, Lick Creek Park

####### Time of activity.

Male (June); female (March, May, June 23-July 2, December)

####### Habitat.

(crops: cotton); (grass: grassland); (plants: weed); (soil/woodland: beech-magnolia forest)

####### Method.

Beating/sweeping [f]; malaise trap [f]

####### Type.

Pennsylvania

####### Etymology.

Latin, prominent palpal tibia

####### Collection.

MCZ, TAMU

##### Genus *Anasaitis* Bryant, 1950

###### Anasaitis canosa

Taxon classificationAnimaliaAraneaeSalticidae

(Walckenaer, 1837)

Anasaitis canosa Bradley 2013: 185; Edwards 1999b: 10 [T]; Henderson 2007: 57–58, 63, 65–66, 78, 81, 84; Richman et al. 2005: 211; Richman et al. 2011b: 3; Richman et al. 2012a: 3; Richman et al. 2012b: 3; Yantis 2005: 67, 196, 199
Corythalia canosa (Walckenaer, 1837); Jackman 1997: 128, desc., 167 (photo 41a); Richman and Cutler 1978: 84 [S]
Stoides aurata Hentz, 1846 [Peckham and Peckham 1909: 527, mf (pl. 38, fig. 9, pl. 43, figs 9–9a)]
Corythalia aurata (Hentz, 1846); Kaston 1978: 253, desc. (fig. 645)

####### Distribution.

Anderson, Angelina, Bastrop, Brazos, Burleson, Fort Bend, Gonzales, Harris, Leon, Sabine, San Jacinto, Tyler, Washington

####### Locality.

5-Eagle Ranch, Angelina National Forest, Bastrop State Park, Brazos Bend State Park, Kirby State Forest, Lick Creek Park, Palmetto State Park, Sabine National Forest, Sam Houston National Forest, Texas A&M University Rangeland Area

####### Time of activity.

Male (March – December); female (March – July, September – October)

####### Habitat.

(grass: grassland, sandy-prairie grass, short grass); (littoral: sandy area by water, sedge meadow); (plants: miscellaneous vegetation); (soil/woodland: beech-magnolia forest, bottomland forest, buckeye-sycamore forest, disturbed habitat, forest, hardwood bottomland, leaf litter, oak, old field, pine woods [%: 100], post oak savanna with pasture, post oak woods [%: 56, 71, 77, 93], post oak woodland, sandy area, upland woods, woods); (structures: on wall in motel room)

####### Method.

5 gallon bucket trap [m]; beating/sweeping [m]; berlese funnel [m]; cardboard band [mf]; flight intercept trap [mf]; flight intercept trap on ground [m]; malaise trap [m]; pitfall trap [mf]; sweeping [m]

####### Type.

United States

####### Etymology.

undetermined

####### Collection.

DMNS, MCZ, MSU, TAMU

##### Genus *Attidops* Banks, 1905

###### Attidops cinctipes

Taxon classificationAnimaliaAraneaeSalticidae

(Banks, 1900)

Attidops cinctipes Richman et al. 2011b: 4; Richman et al. 2012a: 4; Richman et al. 2012b: 4 [Edwards 1999a: 13, mf, desc. (figs 12–13, 24–28)]

####### Distribution.

Sabine

####### Time of activity.

Male (April)

####### Habitat.

(soil/woodland: beech-magnolia forest)

####### Method.

Flight intercept trap/malaise trap [m]

####### Type.

Louisiana, Baton Rouge

####### Etymology.

Latin, banded legs

####### Collection.

TAMU

###### Attidops cutleri

Taxon classificationAnimaliaAraneaeSalticidae

Edwards, 1999

Attidops cutleri Edwards 1999a: 12, mf, desc. (figs 10–11, 22–23, 29–30); Richman et al. 2011b: 4; Richman et al. 2012a: 4; Richman et al. 2012b: 4

####### Distribution.

Caldwell, Travis

####### Locality.

Lockhart State Park

####### Time of activity.

Male (April, October)

####### Type.

Texas (male, Travis Co., Austin, October 18, 1967, D. Simon, holotype, FSCA)

####### Etymology.

Person (Named for Dr. Bruce Cutler, who first identified the AMNH specimens to genus, Edwards 1999a).

###### Attidops youngi

Taxon classificationAnimaliaAraneaeSalticidae

(Peckham & Peckham, 1888)

Attidops youngi Edwards 1999a: 10, mf, desc. (figs 2, 4–7, 14–17); Richman et al. 2011b: 4; Richman et al. 2012a: 4; Richman et al. 2012b: 4

####### Distribution.

Brazoria

####### Time of activity.

Male (February)

####### Type.

Pennsylvania

####### Etymology.

Person (collector, Col. J. J. Young)

##### Genus *Bagheera* Peckham & Peckham, 1896

###### Bagheera prosper

Taxon classificationAnimaliaAraneaeSalticidae

(Peckham & Peckham, 1901)

Bagheera prosper Bradley 2013: 186; Jackman 1997: 167; Jackman et al. 2007: 199; Maddison 1996: 233, mf (figs 84–85, 99) [T]; Richman et al. 2005: 216; Richman et al. 2011b: 4; Richman et al. 2012a: 4; Richman et al. 2012b: 4; Ruiz and Edwards 2013: 20, mf, desc. (figs 2, 6, 10, 13–16)
Dendryphantes prosper Peckham and Peckham, 1901; Banks 1910: 66; Peckham and Peckham 1901a: 314, m, desc. (pl. 27, figs 5–5a); Peckham and Peckham 1909: 457, m, desc.; Petrunkevitch 1911: 640; Roewer 1955: 1204
Metaphidippus prosper Peckham and Peckham, 1901; Vogel 1970b: 18

####### Distribution.

Atascosa, Bexar, Burnet, Comal, Hays, Hood, Hunt, Kimble, Llano, Randall, Runnels, Starr, Tom Green, Val Verde, Wichita

####### Locality.

Lake Ballinger, Lake Tawakoni State Park, Llano City Lake Park, Lower Rio Grande Valley National Wildlife Refuge

####### Time of activity.

Male (March – April, July – October); female (April, July)

####### Habitat.

(grass: grasses and herbs); (littoral: creek vegetation, lake edge, stream edge vegetation); (plants: on leaf, on tree, vegetation); (soil/woodland: cedar, elm, mesquite, oak, riparian mesquite forest); (web: large spider web)

####### Method.

Beating [m]; flight intercept trap [m]

####### Type.

Texas (male, Bexar Co., San Antonio, no date, no collector, syntype, MCZ)

####### Etymology.

Latin, agreeable

####### Collection.

MSU, NMSU, TAMU, WTAM

##### Genus *Bellota* Peckham & Peckham, 1892

###### Bellota micans

Taxon classificationAnimaliaAraneaeSalticidae

Peckham & Peckham, 1909

Bellota micans Banks 1910: 75; Galiano 1972: 477, f, desc. (figs 3–4, 25–26, 41, 46); Jackman 1997: 167; Peckham and Peckham 1909: 373, f, desc. (pl. 51, fig. 6); Petrunkevitch 1911: 607; Richman and Cutler 1978: 83; Richman et al. 2005: 208; Richman et al. 2011b: 5; Richman et al. 2012a: 5; Richman et al. 2012b: 5; Roewer 1955: 1036

####### Distribution.

South Texas

####### Type.

Texas (female, no locality, no date, no collector)

[male unknown]

####### Etymology.

Latin, twinkling or glowing

###### Bellota wheeleri

Taxon classificationAnimaliaAraneaeSalticidae

Peckham & Peckham, 1909

Bellota wheeleri Banks 1910: 75; Galiano 1972: 480, f, desc. (figs 1–2, 15–16, 42, 50); Jackman 1997: 167; Maddison 1996: 332, m (fig. 42); Petrunkevitch 1911: 607; Richman and Cutler 1978: 83 [spelling]; Richman et al. 2005: 208; Richman et al. 2011b: 5; Richman et al. 2012a: 5; Richman et al. 2012b: 5; Roewer 1955: 1036
Bellota wheelerii Peckham and Peckham, 1909; Peckham and Peckham 1909: 374, f, desc. (pl. 51, fig. 7)

####### Distribution.

South Texas; Hidalgo

####### Type.

Texas (female, no locality, no date, no collector)

####### Etymology.

Person (This species is named for Prof. Wm. Morton Wheeler, Peckham and Peckham 1909).

####### Collection.

MSU

##### Genus *Bredana* Gertsch, 1936

###### Bredana alternata

Taxon classificationAnimaliaAraneaeSalticidae

Gertsch, 1936

Bredana alternata Bonnet 1955: 915; Gertsch 1936: 21, f, desc. (fig. 30); Hill and Edwards 2013: 21; Jackman 1997: 167; Richman and Cutler 1978: 84; Richman et al. 2005: 210; Richman et al. 2011b: 5; Richman et al. 2012a: 5; Richman et al. 2012b: 5; Roewer 1955: 1255
Bredana alterana Gertsch, 1936; Vogel 1970b: 17

####### Distribution.

Cameron

####### Time of activity.

Female (November)

####### Type.

Texas (female, Cameron Co., 15 miles SW Harlingen, November 18, 1934, S. Mulaik, holotype, AMNH)

[male known but not described]

####### Etymology.

Latin, alternate species

###### Bredana complicata

Taxon classificationAnimaliaAraneaeSalticidae

Gertsch, 1936

Bredana complicata Bonnet 1955: 915; Gertsch 1936: 21, mf, desc. (figs 33–35); Hill and Edwards 2013: 21; Jackman 1997: 167; Richman and Cutler 1978: 84; Richman et al. 2005: 210; Richman et al. 2011b: 5; Richman et al. 2012a: 5; Richman et al. 2012b: 5; Roewer 1955: 1255; Vogel 1970b: 17

####### Distribution.

Cameron, Hidalgo

####### Locality.

Bentsen-Rio Grande Valley State Park, Sabal Palm Audubon Sanctuary

####### Time of activity.

Male (April, June, August 14-September 5); female (June, August, August 14-September 5)

####### Habitat.

(soil/woodland: cedar elm forest)

####### Method.

Lindgren flight trap [mf]

####### Type.

Texas (male, Hidalgo Co., below Weslaco, Llano Grande, April 27, 1934, S. Mulaik, holotype, AMNH)

####### Etymology.

Latin, complicated palp

####### Collection.

TAMU

##### Genus *Chalcoscirtus* Bertkau, 1880

###### Chalcoscirtus diminutus

Taxon classificationAnimaliaAraneaeSalticidae

(Banks, 1896)

Chalcoscirtus diminutus Edwards 2003: 71 [T]; Richman et al. 2011b: 6; Richman et al. 2012a: 6; Richman et al. 2012b: 6
Euophrys diminuta (Banks, 1896); Agnew et al. 1985: 8; Dean and Eger 1986: 142; Edwards 1980: 11 [S]; Jackman 1997: 167
Corythalia delicatula Gertsch and Mulaik, 1936; Bonnet 1956: 1236; Gertsch and Mulaik 1936a: 14, f, desc. (fig. 32); Richman and Cutler 1978: 84; Roewer 1955: 1101; Vogel 1970b: 17 [Chamberlin and Ivie 1944: 195, mf (figs 211–212)]

####### Distribution.

Brazos, Brooks, Burleson, Coryell, Erath, Tyler

####### Locality.

Kirby State Forest

####### Time of activity.

Male (March – April); female (May, October)

####### Habitat.

(plants: bluebonnets); (soil/woodland: post oak savanna with pasture)

####### Method.

pitfall trap [mf]; suction trap [m]; sweeping [m]

####### Type.

New York

####### Etymology.

Latin, size

####### Collection.

TAMU

##### Genus *Cheliferoides* F. O. P.-Cambridge, 1901

###### Cheliferoides longimanus

Taxon classificationAnimaliaAraneaeSalticidae

Gertsch, 1936

Cheliferoides longimanus Bonnet 1956: 1040; Gertsch 1936: 22, mf, desc. (fig. 32); Jackman 1997: 167; Richman and Cutler 1978: 84; Richman et al. 2005: 208; Richman et al. 2011b: 6; Richman et al. 2012a: 6; Richman et al. 2012b: 6; Roewer 1955: 1187; Vogel 1970b: 17
Bellota wheeleri Peckham and Peckham, 1909; Breene et al. 1993b: 648 [misidentified]

####### Distribution.

Brazoria, Cameron, Colorado, Hidalgo

####### Locality.

Attwater Prairie Chicken National Wildlife Refuge, Hoblitzelle Farms, Sabal Palm Audubon Sanctuary, Santa Ana National Wildlife Refuge

####### Time of activity.

Male (February – June, August – November); female (February – March, May – July, September – November)

####### Habitat.

(crops: sugarcane); (grass: grass); (soil/woodland: on ground, palm forest margin)

####### Method.

D-Vac suction [mf]; sifting [mf]; sweeping

####### Type.

Texas (male, Hidalgo Co., below Weslaco, Llano Grande, April 28, 1934, S. Mulaik, holotype, AMNH)

####### Etymology.

Latin, long hand (front leg)

####### Collection.

TAMU

###### Cheliferoides segmentatus

Taxon classificationAnimaliaAraneaeSalticidae

F. O. P.-Cambridge, 1901

Cheliferoides segmentatus Jackman 1997: 167; Platnick 1984: 171, mf, desc. (figs 1–6); Richman and Cutler 1978: 84; Richman et al. 2005: 208; Richman et al. 2011b: 6 (questionable); Richman et al. 2012a: 6 (questionable); Richman et al. 2012b: 6 (questionable); Roth 1982: 40–2; Roth 1985: B-36–1; Roth 1994: 153

####### Distribution.

Cameron, Comal, Frio, Starr, Uvalde

####### Locality.

Frio State Park, Laguna Atascosa National Wildlife Refuge, Lower Rio Grande Valley National Wildlife Refuge, Sabal Palm Audubon Sanctuary

####### Time of activity.

Male (March – May, September)

####### Habitat.

(soil/woodland: dense coastal brush, palm forest, riparian mesquite forest, tree trunk)

####### Method.

Flight intercept trap on ground [mf]; hanging carrion trap [m]

####### Type.

Guatemala

####### Etymology.

Latin, ornamented

####### Collection.

TAMU

##### Genus *Colonus* F. O. P-Cambridge, 1901

###### Colonus hesperus

Taxon classificationAnimalia

(Richman & Vetter, 2004)

Colonus hesperus Bustamante et al. 2015: 187 [T]
Thiodina hespera Richman and Vetter, 2004; Hill 2012: 2; Richman et al. 2011b: 75; Richman et al. 2012a: 75; Richman et al. 2012b: 75 [Richman and Vetter 2004: 426, mf, desc. (figs 2, 4, 8, 11, 14)]

####### Distribution.

Brewster, Hays

####### Time of activity.

Male (May); female (May)

####### Habitat.

(soil/woodland: *Juniperus ashei*)

####### Method.

Beating [mf]

####### Type.

California, San Diego

####### Etymology.

Greek, signifies that this species is found in the western United States

####### Collection.

MSU, TAMU

###### Colonus puerperus

Taxon classificationAnimalia

(Hentz, 1846)

Colonus puerperus Bustamante et al. 2015: 187 [T]
Thiodina puerpera (Hentz, 1846); Agnew et al. 1985: 5, 11; Breene et al. 1993c: 24, 48, 64, mf (figs 40A-C); Brown 1974: 237; Carpenter 1972: 165; Dean et al. 1982: 256; Dean et al. 1988: 287; Hill 2012: 2 (mf, color pictures); Jackman 1997: 168; Jones 1936: 69; Knutson et al. 2010: 515; Liao et al. 1984: 411; Peckham and Peckham 1909: 449; Petrunkevitch 1911: 712; Rapp 1984: 9; Richman and Vetter 2004: 424, mf, desc. (figs 2–3, 7, 10, 13); Richman et al. 2011b: 76; Richman et al. 2012a: 75; Richman et al. 2012b: 75; Vogel 1970b: 20; Wolff 1985: 13; Young and Edwards 1990: 23
Thiodina purpurea Hentz, 1846; Woods and Harrel 1976: 44

####### Distribution.

Archer, Bell, Bexar, Brazos, Burleson, Burnet, Cameron, Colorado, Comal, Dallas, Denton, DeWitt, Dimmit, Erath, Fannin, Freestone, Galveston, Gillespie, Grayson, Hamilton, Hardeman, Harris, Hays, Hidalgo, Houston, Howard, Hunt, Jefferson, Jim Wells, Kenedy, Lampasas, Live Oak, Mills, Nacogdoches, Panola, Potter, Rains, Randall, Refugio, Robertson, San Patricio, Scurry, Shackelford, Tarrant, Travis, Uvalde, Walker, Webb, Wichita, Wilbarger, Wise [West Co., not in Texas]

####### Locality.

Adriance Pecan Orchard, Attwater Prairie Chicken National Wildlife Refuge, Ellis Prison Unit, Frontera Audubon, Garner State Park, Lake Tawakoni State Park, Lake Thomas, Lake Wichita, Lick Creek Park, Medicine Mounds Ranch, Russell Farm, Sam Houston National Forest, Santa Ana National Wildlife Refuge, Stubblefield Lake, Texas A&M University Rangeland Area

####### Time of activity.

Male (January – October, December); female (January – December)

####### Habitat.

(crops: cotton, peanuts, rice, sunflower); (grass: grass, grassland, pasture); (littoral: salt marsh area, sedge meadow); (nest/prey: mud dauber nest [mf]); (orchard: grapefruit, pecan); (plants: bush, herbs, milkweed, miscellaneous vegetation, roadside vegetation, vegetation, weed, white thistle, *Baccharis*, *Dalea* sp, *Gaillardia* sp.); (soil/woodland: field, juniper, log, post oak savanna, saltcedar, trees/shrubs, willow)

####### Method.

Beating [mf]; beating/sweeping [m]; boll weevil pheromone trap [mf]; D-Vac suction [mf]; pitfall trap [m]; sweeping [mf]

####### Type.

unknown

####### Etymology.

Latin, childbearing

####### Collection.

MCZ, MSU, NMSU, TAMU, WTAM

###### Colonus sylvanus

Taxon classificationAnimalia

(Hentz, 1846)

Colonus sylvanus Bustamante et al. 2015: 187 [T]
Thiodina sylvana (Hentz, 1846); Agnew et al. 1985: 8, 11; Bradley 2013: 205; Breene et al. 1993c: 24, 48, 64, mf (figs 41A-C); Calixto et al. 2013: 184; Dean and Eger 1986: 143; Dean et al. 1982: 256; Hill 2012: 2 (mf, color pictures); Hunter 1988: 18–21; Jackman 1997: 137, desc., 168 (photo 41q); Jackman et al. 2007: 199; Peckham and Peckham 1909: 449; Petrunkevitch 1911: 712; Richman and Vetter 2004: 421, mf, desc. (figs 2–3, 5–6, 9, 12); Richman et al. 2011b: 76 [S]; Richman et al. 2012a: 76; Richman et al. 2012b: 76; Wolff 1985: 22; Young and Edwards 1990: 23
Thiodina iniquies (Walckenaer, 1837); Kaston 1978: 255; Woods and Harrel 1976: 44 [Texas record]
Plexippus puerperus Hentz, 1846; Marx 1890: 570; Peckham and Peckham 1888: 33
Thiodina silvana (Hentz, 1846); Vogel 1970b: 20
Colonus puerperus Peckham and Peckham, 1885; F. O. P.-Cambridge 1901: 246 [misidentified]

####### Distribution.

Angelina, Aransas, Archer, Bexar, Bosque, Brazos, Brown, Burleson, Caldwell, Cameron, Comal, Coryell, Dallas, Denton, Ellis, Erath, Fort Bend, Galveston, Goliad, Gonzales, Grayson, Hardin, Hidalgo, Howard, Hunt, Jasper, Jefferson, Kenedy, Leon, Liberty, McLennan, Nacogdoches, Robertson, Sabine, San Patricio, Scurry, Tarrant, Travis, Tyler, Upshur, Walker, Washington, Wichita [Fairfax, West Co., not in Texas]

####### Locality.

5-Eagle Ranch, Adriance Pecan Orchard, Anzalduas County Park, Bentsen-Rio Grande Valley State Park, Ellis Prison Unit, Estero Llano Grande State Park, Frontera Audubon, Goose Island State Park, Holmes Pecan Orchard, Kenedy Ranch, Kirby State Forest, Lacuna Park, Laguna Madre, Lake Tawakoni State Park, Lake Thomas, Landa Park Estates, Lick Creek Park, Lockhart State Park, McKelvey Park, Palmetto State Park, Resaca de la Palma State Park, Sabal Palm Audubon Sanctuary, Santa Ana National Wildlife Refuge

####### Time of activity.

Male (March – November); female (March – November)

####### Habitat.

(crops: cotton, rice); (grass: grassland); (landscape features: under rock); (littoral: sand dune under live oak, sedge meadow); (nest/prey: nest of *Neotoma micropus*); (orchard: grapefruit, orange, pecan); (plants: bluebonnets, honey suckle, Indian paintbrush, miscellaneous vegetation, vegetation, weed, *Baccharis*); (soil/woodland: beech-magnolia forest, forest, live oak area, palm forest, pine forest, post oak savanna, post oak savanna with pasture, saltcedar, trees, trees/shrubs, woods, *Quercus buckleyi*, *Quercus virginiana*, *Ulmus crassifolia*); (structures: around house); (web: large spider web)

####### Method.

Beating [mf]; beating/sweeping [mf]; cardboard band [mf]; flight intercept trap [mf]; fogging [f]; Lindgren funnel trap [f]; malaise trap [mf]; pitfall trap [m]; sweeping [mf]; yellow pan trap [f]

####### Type.

South Carolina

####### Etymology.

Latin, trees (normal habitat)

####### Collection.

MCZ, MSU, NMSU, TAMU

##### Genus *Eris* C. L. Koch, 1846

###### Eris flava

Taxon classificationAnimaliaAraneaeSalticidae

(Peckham & Peckham, 1888)

Eris flava Jackman 1997: 167; Maddison 1986: 145, mf, desc. (figs 8–13); Richman et al. 2011b: 8; Richman et al. 2012a: 8; Richman et al. 2012b: 8

####### Distribution.

Jefferson

####### Type.

New York

####### Etymology.

Latin, yellow

###### Eris floridana

Taxon classificationAnimaliaAraneaeSalticidae

(Banks, 1904)

Eris floridana Richman et al. 2011b: 9; Richman et al. 2012a: 8; Richman et al. 2012b: 9 [Kaston 1973: 120, mf (figs 63–65)]

####### Distribution.

Mason

####### Time of activity.

Female (January)

####### Habitat.

(orchard: pecan)

####### Method.

irrigation tubing [f]

####### Type.

Florida, Altoona

####### Etymology.

locality (state)

####### Collection.

TAMU

###### Eris militaris

Taxon classificationAnimaliaAraneaeSalticidae

(Hentz, 1845)

Eris militaris Breene et al. 1993c: 22, 48, 69, mf (figs 54A-C); Bumroongsook et al. 1992: 18; Calixto et al. 2013: 184–185, 187; Henderson 2007: 60–61, 78, 81, 84; Jackman 1997: 130, 167 (photo 41b); Jackman et al. 2007: 199; Lombardini et al. 2005: 1378; Maddison 1986: 141, mf (figs 2–7) [S]; Richman et al. 2011b: 9; Richman et al. 2012a: 8; Richman et al. 2012b: 9; Young and Edwards 1990: 21
Dendryphantes militaris (Hentz, 1845); Jones 1936: 69
Paraphidippus marginatus Walckenaer, 1837; Kagan 1942: 64; Kagan 1943: 258
Eris marginata (Walckenaer, 1837); Agnew et al. 1985: 5; Bonnet 1956: 1789; Dean et al. 1982: 255; Hunter 1988: 18; Liao et al. 1984: 411; Rapp 1984: 8; Vogel 1970b: 17
Eris marginatus (Walckenaer, 1837); Brown 1974: 236; Carpenter 1972: 163

####### Distribution.

North-central, central and south Texas; Archer, Brazos, Burleson, Burleson/Lee, Cameron, Clay, Comanche, Dallas, Edwards, Ellis, Erath, Galveston, Grayson, Hood, Hunt, Kerr, Lee, Martin, Mason, McLennan, Medina, Nacogdoches, Nolan, Randall, Robertson, Sabine, San Patricio, Travis, Trinity, Uvalde, Walker, Wichita

####### Locality.

Bill Haney Pecan Orchard, Brison Pecan Orchard, Ellis Prison Unit, Garner State Park, Holmes Pecan Orchard, Lake Somerville State Park [Nails Creek Unit], Lake Tawakoni State Park, Lick Creek Park, Nabor’s Lake, Proctor Lake, Sam Houston National Forest, Storey Pecan Orchard, Stubblefield Lake, Welder Wildlife Refuge, Zilker Park

####### Time of activity.

Male (January – December); female (January – December)

####### Habitat.

(crops: cotton, peanuts); (grass: grassy and shrub area); (landscape features: under rock); (littoral: creek bank, salt marsh area, sedge meadow); (orchard: pecan); (plants: honey suckle, Indian paintbrush, vegetation); (soil/woodland: ash bark, beech-magnolia forest, cottonwood, oak, old field, post oak savanna, sandy area, trees, upland woods, willow, woodland, woods, *Juniperus ashei*, *Quercus buckleyi*, *Quercus virginiana*, *Ulmus crassifolia*); (structures: at home, car window); (web: large spider web)

####### Method.

Beating [mf]; beating/sweeping [mf]; boll weevil pheromone trap [mf]; cardboard band [mf]; fogging [mf]; irrigation tubing [mf]; malaise trap [m]; pitfall trap [m]; sweeping [mf]

####### Eggs/spiderlings.

Brazos [eggsac laid March 31, 1978, hatched April 12, 9 spiders, 22 eggs infertile]; [eggsac laid March 29, 1978, hatched April 9, 66 spiderlings]; [eggsac laid April 2, 1978, hatched April 12; 23 spiderlings]; [eggsac laid March 9, 1978, hatched March 17, 38 spiderlings; second eggsac laid March 27, 1978, hatched April 12, 1 spiderling, 17 eggs infertile; eggsac laid April 2, 1978, hatched April 12, 32 spiderlings] [TAMU]

####### Type.

North Carolina and Alabama

####### Etymology.

Latin, referring to a soldier

####### Collection.

DMNS, MSU, NMSU, TAMU, TTU, WTAM

###### Eris rufa

Taxon classificationAnimaliaAraneaeSalticidae

(C. L. Koch, 1846)

Eris rufa Edwards 2004: 13 [S]
Eris pinea (Kaston, 1945); Jackman 1997: 167 [Kaston 1973: 120, mf (figs 58–62)]
Eris pineus (Kaston, 1945); Brown 1974: 236

####### Distribution.

Nacogdoches

####### Habitat.

(nest/prey: mud dauber nest [f])

####### Type.

United States

####### Etymology.

Latin, reddish

##### Genus *Ghelna* Maddison, 1996

###### Ghelna barrowsi

Taxon classificationAnimaliaAraneaeSalticidae

(Kaston, 1973)

Ghelna barrowsi Jackman 1997: 167; Maddison 1996: 239 [T]; Richman et al. 2011b: 11; Richman et al. 2012a: 11; Richman et al. 2012b: 11
Metaphidippus barrowsi Kaston, 1973; Agnew et al. 1985: 8 [Kaston 1973: 107, mf (figs 1–4)]

####### Distribution.

Erath

####### Time of activity.

Female (August)

####### Method.

sweeping

####### Type.

Virginia, Lucketts

####### Etymology.

Person (collector of specimens other than holotype, W. M. Barrows)

####### Collection.

TAMU

###### Ghelna castanea

Taxon classificationAnimaliaAraneaeSalticidae

(Hentz, 1846)

Ghelna castanea Jackman 1997: 167; Maddison 1996: 239 [T]
Metaphidippus castaneus (Hentz, 1846); Carpenter 1972: 163 [Kaston 1973: 109, mf (figs 5–7)]

####### Distribution.

Wichita

####### Habitat.

(structures: in house)

####### Type.

North Carolina

####### Etymology.

Greek, chestnut-colored

####### Collection.

MSU

###### Ghelna sexmaculata

Taxon classificationAnimaliaAraneaeSalticidae

(Banks, 1895)

Ghelna sexmaculata Jackman 1997: 167; Maddison 1996: 239 [T]; Richman et al. 2011b: 12; Richman et al. 2012a: 12; Richman et al. 2012b: 12
Metaphidippus sexmaculatus (Banks, 1895) [Kaston 1973: 109, mf (figs 12–14)]

####### Distribution.

Angelina, Brazos, Houston, Tyler

####### Locality.

Angelina National Forest, Big Slough Wild Area, Big Thicket National Preserve, Lick Creek Park

####### Time of activity.

Male (March, May, November – December); female (March)

####### Habitat.

(soil/woodland: bottomland forest litter, hardwood litter, leaf litter, loblolly pine managed, longleaf pine unmanaged)

####### Method.

Berlese funnel [m]; pitfall trap [m]

####### Type.

Canada

####### Etymology.

Latin, six white spots on male abdomen

####### Collection.

TAMU

##### Genus *Habronattus* F. O. P.-Cambridge, 1901

###### Habronattus calcaratus

Taxon classificationAnimaliaAraneaeSalticidae

(Banks, 1904)

Habronattus calcaratus Richman et al. 2011b: 14 [S]; Richman et al. 2012a: 14; Richman et al. 2012b: 14
Pellenes calcaratus Banks, 1904; Cokendolpher 1978c: 118; Richman and Cutler 1978: 93
Habronattus calcaratus agricola Griswold, 1987; Griswold 1987: 139, mf, desc. (figs 97, 190); Jackman 1997: 167; Maddison and Hedin 2003a: 19

####### Distribution.

Burleson, Clay, Coryell, Knox, Travis

####### Time of activity.

Male (March – August); female (April 25-May 4, September)

####### Habitat.

(crops: cotton); (landscape features: under rock); (soil/woodland: post oak savanna with pasture)

####### Method.

pitfall trap [m]

####### Type.

South Dakota, 1 mile S Rapid City

####### Etymology.

Latin, furnished with a spur

####### Collection.

MSU, TAMU

###### Habronattus clypeatus

Taxon classificationAnimaliaAraneaeSalticidae

(Banks, 1895)

Habronattus clypeatus [Griswold 1987: 143, mf, desc. (fig. 104); Peckham and Peckham 1909: 548, mf, desc. (pl. 44, fig. 12, pl. 45, figs 2–2c])

####### Distribution.

El Paso

####### Locality.

Tom Mays Memorial Park, Franklin Mountains

####### Time of activity.

Male (April)

####### Habitat.

(soil/woodland: on ground)

####### Type.

Colorado, Fort Collins

####### Etymology.

Latin, face (clypeus)

####### Collection.

NMSU

###### Habronattus coecatus

Taxon classificationAnimaliaAraneaeSalticidae

(Hentz, 1846)

Habronattus coecatus Agnew et al. 1985: 5; Bradley 2013: 188; Breene 1988: 35; Breene et al. 1989: 163; Breene et al. 1993b: 648; Breene et al. 1993c: 22, 48, 67, mf (figs 48A-C); Calixto et al. 2013: 184; Cokendolpher et al. 2008: 10, 48 (photo 31–32); Dean and Eger 1986: 142; Dean and Sterling 1990: 405; Dean et al. 1987: 268; Dean et al. 1988: 287; Griswold 1987: 95 [T], mf, desc. (fig. 79); Irungu 2007: 31; Jackman 1997: 130, 167; Nyffeler et al. 1992c: 3; Richman et al. 2011b: 15; Richman et al. 2012a: 15; Richman et al. 2012b: 15; Young and Edwards 1990: 21
Pellenes coecatum Hentz, 1846; Banks 1904: 119
Pellenes coecatus Hentz, 1846; Dean et al. 1982: 256; Hunter 1988: 18, 21; Richman and Cutler 1978: 93
Pellenes coronatus (Hentz, 1846); Bonnet 1958: 3461; Brown 1974: 236; Carpenter 1972: 164; Comstock 1912: 691, desc.; Comstock 1940: 699; Jones 1936: 69; Kagan 1942: 71; Kagan 1943: 258; Peckham and Peckham 1909: 545, mf, desc. (pl. 45, figs 3a-d); Petrunkevitch 1911: 682; Vogel 1970b: 18
Habronattus coronatus (Hentz, 1846); Milstead 1958: 446 [Kaston 1948: 466, mf, desc. (figs 1710, 1731–1732)]

####### Distribution.

Eastern 2/3 Texas; Anderson, Archer, Bee, Brazos, Brewster, Burleson, Cameron, Carson, Cass, Colorado, Comanche, Coryell, Dallas, Denton, Ellis, Erath, Falls, Goliad, Grayson, Hidalgo, Houston, Hunt, Jeff Davis, Kerr, Kleberg, Knox, Lubbock, Marion, McLennan, Nacogdoches, Polk, Presidio, Rains, Refugio, Robertson, San Patricio, Taylor, Travis, Victoria, Walker, Wichita, Williamson, Wilson

####### Locality.

5-Eagle Ranch, Attwater Prairie Chicken National Wildlife Refuge, Bill Haney Pecan Orchard, Black Gap Wildlife Management Area, Ellis Prison Unit, Goliad State Park, Hagerman National Wildlife Refuge, Holmes Pecan Orchard, Lake Dallas, Lick Creek Park, NK Ranch, Padre Island, Padre Island National Seashore, Riley Estate, Sheppard Air Force Base, Somerville Lake, Stiles Farm Foundation, Texas A&M University Rangeland Area, Welder Wildlife Refuge

####### Time of activity.

Male (February – December); female (March – December)

####### Habitat.

(crops: cabbage, cotton, peanuts, sugarcane); (grass: grass, grassland, pasture, tall grass); (landscape features: on rock); (littoral: brush by creek, edge of pond, near playa, near pond); (nest/prey: mud dauber nest [m], stomach of *Cnemidophorus perplexus*, stomach of *Cnemidophorus sacki*, stomach of *Cnemidophorus tigris*); (orchard: pecan); (plants: bluebonnets, Indian paintbrush, miscellaneous vegetation, roadside vegetation, *Monarda citriodora*); (soil/woodland: edge of woods, forest, sandy area, post oak savanna with pasture, woodland)

####### Method.

D-Vac suction [mf]; fogging [mf]; pitfall trap [mf] (edge of pond [m], edge of woods [f], in sand [mf], near pond [m], under juniper [f], under oak [f]); suction trap [mf]; sweeping [mf]

####### Eggs/spiderlings.

Brazos [eggsac laid May 24, 1978, hatch June 12, 37 spiderlings]; Walker [eggsac laid July 28, 1978, hatch August 11, 16 spiderlings] [TAMU]

####### Type.

Alabama

####### Etymology.

undetermined

####### Collection.

MSU, TAMU

###### Habronattus cognatus

Taxon classificationAnimaliaAraneaeSalticidae

(Peckham & Peckham, 1901)

Habronattus cognatus Agnew et al. 1985: 5; Cokendolpher et al. 2008: 10, 48; Griswold 1987: 187 [T], mf, desc.; Hedin and Maddison 2001b: 1514; Jackman 1997: 130, 167; Knutson et al. 2010: 515; Maddison and Hedin 2003a: 18; Richman et al. 2011b: 15; Richman et al. 2012a: 15; Richman et al. 2012b: 15
Pellenes cognatus Peckham and Peckham, 1901 [Peckham and Peckham 1901b: 224, f, desc. (pl. 1, fig. 19)]
Pellenes arizonensis (Banks, 1904); Carpenter 1972: 164 [Texas record]

####### Distribution.

Angelina, Blanco, Briscoe, Cameron, Carson, Clay, Comanche, Coryell, Erath, Hidalgo, Howard, Kenedy, Kerr, Potter, Randall, Travis, Ward, Wichita, Wilbarger

####### Locality.

Browning Ranch, Camp Chrysalis, Kenedy Ranch, Lake Meredith, South Padre Island

####### Time of activity.

Male (March 29-April 5, April – October); female (April – August, September 28-October 4, October)

####### Habitat.

(crops: peanuts); (grass: grass, grassland, sparse grass); (littoral: near playa, sand dune area); (plants: low weeds and annuals, miscellaneous vegetation); (soil/woodland: ground, mesquite bush, post oak savanna with pasture, saltcedar)

####### Method.

pitfall trap [mf] (edge of woods [m], in sand [mf], in sand in woods [m]); sweeping [mf]

####### Type.

Kansas

####### Etymology.

Latin, related

####### Collection.

MSU, TAMU, WTAM

###### Habronattus conjunctus

Taxon classificationAnimaliaAraneaeSalticidae

(Banks, 1898)

Habronattus conjunctus Broussard and Horner 2006: 255 [Griswold 1987: 184, mf, desc. (figs 22, 142, 233)]

####### Distribution.

Presidio, Wichita

####### Locality.

Big Bend Ranch State Park, Chihuahuan desert, Dalquest Research Site

####### Time of activity.

Female (March)

####### Habitat.

(landscape features: on rock)

####### Method.

pitfall trap [mf]

####### Type.

Mexico, Baja California Sur

####### Etymology.

Latin, connected

####### Collection.

MSU, NMSU

###### Habronattus decorus

Taxon classificationAnimaliaAraneaeSalticidae

(Blackwall, 1846)

Habronattus decorus [Kaston 1948: 468, mf (figs 1711–1712, 1733–1735); Paquin and Dupérré 2003: 106, mf (figs 2184–2186)]
Habronattus sp. nr moratus (Gertsch and Mulaik, 1936); Henderson 2007: 31, 40, 53–54, 56, 58–59, 61–63, 65, 67, 69–70, 74, 78, 81, 84 [misidentified]; Yantis 2005: 197, 200 [misidentified]

####### Distribution.

Brazos, Burleson, Houston, Hunt, Madison

####### Locality.

Lick Creek Park, Somerville Lake

####### Time of activity.

Male (February 15-March 15, March 26-April 2, April, May – June, June 30-July 15, July 26-July 2, August 15-September 17, September 28-October 5, October 20-November 15); female (July 25-August 1, August, September 28-October 5)

####### Habitat.

(grass: short grass); (soil/woodland: disturbed habitat, pine woods [%: 95%], post oak woods [%: 56%], post oak savanna with pasture, post oak woodland, upland woods)

####### Method.

pitfall trap [f]

####### Type.

Canada, Toronto

####### Etymology.

Latin, decorative

####### Collection.

FSCA, MSU, TAMU

###### Habronattus delectus

Taxon classificationAnimaliaAraneaeSalticidae

(Peckham & Peckham, 1909)

Habronattus delectus Griswold 1987: 230 [T], mf, desc. (figs 67, 146, 176, 226); Jackman 1997: 167; Maddison and Hedin 2003a: 21; Richman et al. 2011b: 16; Richman et al. 2012a: 16; Richman et al. 2012b: 16
Pellenes birgei Peckham and Peckham, 1901; Richman and Cutler 1978: 92 [Texas record]; Roewer 1955: 1133
Pellenes delectus (Peckham and Peckham, 1909); Banks 1910: 68; Chamberlin 1924b: 688; Jones 1936: 69; Peckham and Peckham 1909: 550, mf, desc. (pl. 47, figs 6–6a, pl. 49, figs 1–1a); Petrunkevitch 1911: 683; Vogel 1970b: 18
Habronattus sp.; Milstead 1958: 446 [part]

####### Distribution.

Brown, Dallas, Hidalgo, Reeves, San Patricio, Terrell, Travis, Wichita

####### Locality.

Blackstone Ranch, Welder Wildlife Refuge

####### Time of activity.

Female (June, August, December)

####### Habitat.

(crops: cotton); (grass: grass); (nest/prey: stomach of *Cnemidophorus sacki*); (soil/woodland: sandy open prairie)

####### Method.

D-Vac suction [f]; pitfall trap [f]

####### Type.

Texas (male, Travis Co., Austin, no date, no collector, lectotype, MCZ)

####### Etymology.

Latin, delighting

####### Collection.

MSU, TAMU

###### Habronattus dorotheae

Taxon classificationAnimaliaAraneaeSalticidae

(Gertsch & Mulaik, 1936)

Habronattus dorotheae Griswold 1987: 154 [T], mf, desc. (figs 36, 120, 159, 210); Jackman 1997: 167; Maddison and Hedin 2003a: 18; Richman et al. 2011b: 17; Richman et al. 2012a: 16; Richman et al. 2012b: 16
Pellenes dorotheae (Gertsch and Mulaik, 1936); Bonnet 1958: 3463; Gertsch and Mulaik 1936a: 16, mf, desc. (fig. 28); Richman and Cutler 1978: 93; Roewer 1955: 1134; Vogel 1970b: 18

####### Distribution.

Cameron, Hidalgo

####### Locality.

Bentsen-Rio Grande Valley State Park

####### Time of activity.

Male (March 26-April 1, August – September, November); female (November)

####### Habitat.

(grass: grass)

####### Method.

pitfall trap [m]

####### Type.

Texas (male, Cameron Co., 15 miles SW Harlingen, November 17, 1934, S. Mulaik, holotype, AMNH)

####### Etymology.

Person (wife of collector, Dorothea)

####### Collection.

TAMU

###### Habronattus fallax

Taxon classificationAnimaliaAraneaeSalticidae

(Peckham & Peckham, 1909)

Habronattus fallax Agnew et al. 1985: 8; Griswold 1987: 209 [T], mf, desc. (fig. 139) [see note below]; Hedin and Maddison 2001b: 1514; Jackman 1997: 167; Maddison and Hedin 2003a: 19; Richman et al. 2011b: 17; Richman et al. 2012a: 17; Richman et al. 2012b: 17
Pellenes fallax Peckham and Peckham, 1909; Banks 1910: 68; Peckham and Peckham 1909: 553, mf, desc. (pl. 47, fig. 5, pl. 49, figs 3–3a); Petrunkevitch 1911: 684; Richman and Cutler 1978: 93; Roewer 1955: 1132; Vogel 1970b: 18

####### Distribution.

Cameron, Comanche, Coryell, Erath, Hidalgo, Kerr, San Patricio, Starr, Travis, Wichita, Zapata

####### Locality.

Estero Llano Grande State Park, McKelvey Park, Nabor’s Lake, Resaca de la Palma State Park, Santa Ana National Wildlife Refuge, Welder Wildlife Refuge

####### Time of activity.

Male (March – October); female (May – June)

####### Habitat.

(crops: peanuts); (grass: grass); (soil/woodland: ground, post oak savanna with pasture, sandy area, under [juniper, oak])

####### Method.

pitfall trap [mf] (in sand [m], under juniper [mf], under oak [mf])

####### Type.

Texas (male, Travis Co., Austin, no date, no collector, holotype, MCZ)

####### Etymology.

Latin, deceptive

####### Collection.

MSU, TAMU

####### Note.

32 miles SE Laredo is in Zapata Co., not Starr Co.; 54 miles S Laredo is in Nuevo Leon, Mexico, not Starr Co.; 5 miles E Rio Grande City is in Starr Co., not Webb Co.

###### Habronattus forticulus

Taxon classificationAnimaliaAraneaeSalticidae

(Gertsch & Mulaik, 1936)

Habronattus forticulus Griswold 1987: 151 [T], mf, desc. (fig. 101); Hedin and Maddison 2001b: 1514; Jackman 1997: 130, 167; Maddison and Hedin 2003a: 20; Richman et al. 2011b: 17; Richman et al. 2012a: 17; Richman et al. 2012b: 17
Pellenes forticulus Gertsch and Mulaik, 1936; Bonnet 1958: 3464; Gertsch and Mulaik 1936a: 18, mf, desc. (figs 29–30); Richman and Cutler 1978: 93; Roewer 1955: 1134; Vogel 1970b: 19

####### Distribution.

Brewster, Hays, Hidalgo, San Patricio, Travis, Uvalde, Val Verde, Webb, Zapata

####### Locality.

Big Bend National Park

####### Time of activity.

Male (January, March – December); female (January 29-February 6, February, March – April, June, October – November)

####### Habitat.

(grass: grass)

####### Method.

pitfall trap [mf]

####### Type.

Texas (male, Hidalgo Co., Edinburg, October 11, 1934, S. Mulaik, holotype, AMNH)

####### Etymology.

Latin, strong

####### Collection.

TAMU

###### Habronattus hallani

Taxon classificationAnimaliaAraneaeSalticidae

(Richman, 1973)

Habronattus hallani Richman et al. 2011b: 18; Richman et al. 2012a: 17; Richman et al. 2012b: 17 [Griswold 1987: 211, mf, desc. (fig. 205); Richman 1973: 76, mf, desc. (figs 1–5)]

####### Distribution.

El Paso

####### Locality.

Franklin Mountains

####### Time of activity.

Male (October)

####### Type.

Arizona, Santa Catalina Mountains, Sabino Canyon

####### Etymology.

Person (Named for Mr. Joel K. Hallan who inspired my first interest in the Salticidae, Richman 1973).

####### Collection.

NMSU

###### Habronattus hirsutus

Taxon classificationAnimaliaAraneaeSalticidae

(Peckham & Peckham, 1888)

Habronattus hirsutus Broussard and Horner 2006: 255; Griswold 1987: 199, mf, desc. (figs 29, 77, 132, 235); Jackman 1997: 167; Knutson et al. 2010: 515; Richman et al. 2011a: 18; Richman et al. 2011b: 18; Richman et al. 2012a: 18; Richman et al. 2012b: 18

####### Distribution.

Brewster, Brown, Howard, Presidio, Wichita

####### Locality.

Big Bend National Park, Chihuahuan desert, Dalquest Research Site

####### Time of activity.

Male (December); female (December)

####### Habitat.

(soil/woodland: saltcedar)

####### Method.

pitfall trap [mf]

####### Type.

Oregon

####### Etymology.

Latin, hair

####### Collection.

MSU

###### Habronattus klauseri

Taxon classificationAnimaliaAraneaeSalticidae

(Peckham & Peckham, 1901)

Habronattus klauseri Platnick 2003 [spelling]
Habronattus klauserii (Peckham and Peckham, 1901); Maddison and Hedin 2003a: 21; Richman et al. 2011b: 18; Richman et al. 2012a: 18; Richman et al. 2012b: 18 [Griswold 1987: 122, mf, desc. (figs 91, 113)]
Pellenes brunneus Peckham and Peckham, 1901; Richman and Cutler 1978: 92 [Texas record]

####### Distribution.

El Paso, Lubbock, Pecos

####### Time of activity.

Male (July)

####### Habitat.

(plants: yucca); (structures: on lawn)

####### Type.

New Mexico

####### Etymology.

Person

####### Collection.

NMSU

###### Habronattus mataxus

Taxon classificationAnimaliaAraneaeSalticidae

Griswold, 1987

Habronattus mataxus Griswold 1987: 193, mf, desc. (fig. 71); Jackman 1997: 130, 167; Richman et al. 2011b: 19; Richman et al. 2012a: 18; Richman et al. 2012b: 18

####### Distribution.

Brooks, Cameron, Hidalgo, Kleberg, Nueces, Palo Pinto, Polk, San Patricio, Starr

####### Locality.

Falcon State Park, Fort Sill Recreation Area

####### Time of activity.

Male (March – May, July – October, December); female (April – May, September – October)

####### Type.

Mexico, Tamaulipas, Miramar Beach

####### Etymology.

Greek, silk, covering fine scales on first leg

####### Collection.

MSU

###### Habronattus mexicanus

Taxon classificationAnimaliaAraneaeSalticidae

(Peckham & Peckham, 1896)

Habronattus mexicanus Bodner and Maddison 2012: 234; Griswold 1987: 112, mf, desc. (figs 33, 89) [see note below]; Jackman 1997: 167; Maddison and Hedin 2003a: 21; Maddison and Hedin 2003b: 546; Richman et al. 2011b: 19; Richman et al. 2012a: 19; Richman et al. 2012b: 19
Habronattus sp. nr cockerelli Banks, 1901; Irungu 2007: 31 [misidentified]
Habronattus sp. nr conjunctus (Banks, 1898); Irungu 2007: 31 [misidentified]
Habronattus sp. nr pyrithrix (Chamberlin, 1924); Irungu 2007: 31 [misidentified]

####### Distribution.

Cameron, Hidalgo, Kleberg, Val Verde, Zapata

####### Locality.

Ramsey Nature Park

####### Time of activity.

Male (March – December); female (April 22-May 2, October – November)

####### Habitat.

(crops: cabbage)

####### Method.

pitfall trap [mf]

####### Type.

Mexico, Tamaulipas, Reynosa

####### Etymology.

locality (country)

####### Collection.

TAMU

####### Note.

32 miles E Laredo should be 32 miles SE Laredo in Zapata Co., not Webb Co. based on other records from this date.

###### Habronattus moratus

Taxon classificationAnimaliaAraneaeSalticidae

(Gertsch & Mulaik, 1936)

Habronattus moratus Griswold 1987: 93 [T], mf, desc. (figs 78, 164) [see note below]; Hedin and Maddison 2001b: 1514; Jackman 1997: 167; Maddison and Hedin 2003a: 20; Richman et al. 2011b: 19; Richman et al. 2012a: 19; Richman et al. 2012b: 19
Pellenes moratus Gertsch and Mulaik, 1936; Bonnet 1958: 3467; Gertsch and Mulaik 1936a: 17, mf, desc. (figs 26–27) [see note below]; Richman and Cutler 1978: 94; Roewer 1955: 1135; Vogel 1970b: 19

####### Distribution.

Hidalgo, Starr, Zapata

####### Locality.

Lower Rio Grande Valley National Wildlife Refuge

####### Time of activity.

Male (March – April, July); female (July, November)

####### Type.

Texas (male, Hidalgo Co., 30 miles W Edinburg, July 4, 1935, S. Mulaik, holotype, AMNH)

####### Etymology.

Latin, delay

####### Note.

32 mi E Laredo should be 32 mi SE Laredo in Zapata Co., not Hidalgo Co. (Griswold 1987: 94), based on other records from this date.

###### Habronattus orbus

Taxon classificationAnimaliaAraneaeSalticidae

Griswold, 1987

Habronattus orbus Griswold 1987: 207, mf, desc. (figs 141, 183, 200); Jackman 1997: 167; Richman et al. 2011b: 20; Richman et al. 2012a: 20; Richman et al. 2012b: 20
Habronattus n. sp.; Agnew et al. 1985: 8

####### Distribution.

Coryell, Erath

####### Time of activity.

Male (April – June); female (April, July)

####### Habitat.

(crops: peanuts); (littoral: edge of pond); (soil/woodland: edge of woods, post oak savanna with pasture, under [juniper, oak])

####### Method.

pitfall trap [mf] (edge of pond [m], under juniper [m], under oak [m])

####### Type.

Kansas, Lawrence

####### Etymology.

Latin, orphan, uncertain ancestry

####### Collection.

TAMU

###### Habronattus sugillatus

Taxon classificationAnimaliaAraneaeSalticidae

Griswold, 1987

Habronattus sugillatus Griswold 1987: 165, m, desc. (figs 167, 213); Jackman 1997: 167; Maddison and Hedin 2003a: 21; Richman et al. 2011b: 21; Richman et al. 2012a: 21; Richman et al. 2012b: 21
Habronattus sp.; Milstead 1958: 446 [part]

####### Distribution.

Brewster, Presidio, Terrell

####### Locality.

Big Bend National Park, Blackstone Ranch, Chisos Basin, Chisos Mountains, Davis Mountains

####### Time of activity.

Male (September)

####### Habitat.

(nest/prey: stomach of *Cnemidophorus perplexus*, stomach of *Cnemidophorus sacki*, stomach of *Cnemidophorus tessellatus*, stomach of *Cnemidophorus tigris*)

####### Type.

Mexico, Durango, 10 miles W Durango

[female unknown]

####### Etymology.

Latin, bruise, maculation on leg

###### Habronattus texanus

Taxon classificationAnimaliaAraneaeSalticidae

(Chamberlin, 1924)

Habronattus texanus Agnew et al. 1985: 5, 11; Calixto et al. 2013: 184; Cokendolpher et al. 2008: 10, 48; Griswold 1987: 215 [S, T], mf, desc. (figs 137, 229); Hedin and Maddison 2001b: 1514; Jackman 1997: 130, 167; Maddison and Hedin 2003a: 19; Richman et al. 2011b: 21; Richman et al. 2012a: 21; Richman et al. 2012b: 21; Young and Edwards 1990: 21
Pellenes texanus Chamberlin, 1924; Bonnet 1958: 3471; Chamberlin 1924a: 35, m, desc. (fig. 52); Richman and Cutler 1978: 94; Roewer 1955: 1137; Vogel 1970b: 19
Pellenes rutherfordi Gertsch & Mulaik, 1936; Bonnet 1958: 3470; Cokendolpher 1978c: 118; Gertsch and Mulaik 1936a: 16, m, desc.; Richman and Cutler 1978: 94; Roewer 1955: 1136; Vogel 1970b: 19
Habronattus rutherfordi (Gertsch & Mulaik, 1936) [Kaston 1948: 469, m, desc. (figs 1736–1737)]

####### Distribution.

Blanco, Cameron, Carson, Coleman, Collin, Comanche, Cooke, Coryell, Cottle, Duval, Erath, Grayson, Hamilton, Hays, Hidalgo, Palo Pinto, Robertson, San Patricio, Travis, Wichita, Williamson

####### Locality.

Bentsen-Rio Grande Valley State Park, Bill Haney Pecan Orchard, Browning Ranch, Holmes Pecan Orchard, Horne Ranch, Pantex Lake, Stiles Farm Foundation

####### Time of activity.

Male (April – November); female (April – October)

####### Habitat.

(crops: cotton, peanuts); (grass: grass, grasses, grassland); (littoral: playa); (orchard: pecan); (plants: miscellaneous vegetation); (soil/woodland: post oak savanna with pasture); (structures: indoors)

####### Method.

D-Vac suction [m]; pitfall trap [mf]; sweeping [f]

####### Eggs/spiderlings.

Hamilton [6 spiderlings] [TAMU]

####### Type.

Texas (male, Travis Co., Austin, August 1909, R. V. Chamberlin, holotype, MCZ)

####### Etymology.

locality (state)

####### Collection.

MSU, TAMU

###### Habronattus tranquillus

Taxon classificationAnimaliaAraneaeSalticidae

(Peckham & Peckham, 1901)

Habronattus tranquillus Griswold 1987: 196, mf, desc. (figs 30, 76, 131, 236); Hedin and Lowder 2009: 57; Jackman 1997: 167; Richman et al. 2011b: 22; Richman et al. 2012a: 21; Richman et al. 2012b: 21

####### Distribution.

Brown, Starr, Terrell

####### Time of activity.

Male (April – May); female (May)

####### Habitat.

(landscape features: under rock)

####### Type.

Arizona

####### Etymology.

Latin, of tranquil

####### Collection.

MSU

###### Habronattus tuberculatus

Taxon classificationAnimaliaAraneaeSalticidae

(Gertsch & Mulaik, 1936)

Habronattus tuberculatus Griswold 1987: 91 [T], mf, desc. (figs 45, 110, 153); Jackman 1997: 167; Richman et al. 2011b: 22; Richman et al. 2012a: 22; Richman et al. 2012b: 22
Pellenes tuberculatus Gertsch and Mulaik, 1936; Bonnet 1958: 3474; Gertsch and Mulaik 1936a: 14, m, desc. (fig. 25); Richman and Cutler 1978: 94; Roewer 1955: 1137; Vogel 1970b: 19

####### Distribution.

Hidalgo

####### Type.

Texas (male, Hidalgo Co., Edinburg, no date, S. Mulaik, holotype, AMNH)

####### Etymology.

Latin, tubercles

###### Habronattus virgulatus

Taxon classificationAnimaliaAraneaeSalticidae

Griswold, 1987

Habronattus virgulatus Griswold 1987: 106, mf, desc. (figs 34, 95, 193); Jackman 1997: 167; Richman et al. 2011b: 22; Richman et al. 2012a: 22; Richman et al. 2012b: 22
Habronattus sp.; Milstead 1958: 446 [part]

####### Distribution.

El Paso, Presidio, Terrell

####### Locality.

Blackstone Ranch, Franklin Mountains

####### Time of activity.

Male (March)

####### Habitat.

(nest/prey: stomach of *Cnemidophorus perplexus*, stomach of *Cnemidophorus sacki*, stomach of *Cnemidophorus tessellatus*, stomach of *Cnemidophorus tigris*); (soil/woodland: on ground)

####### Type.

Mexico, Chihuahua, 16 miles NNW Chihuahua

####### Etymology.

Latin, striped on ocular region and third patella

####### Collection.

NMSU

###### Habronattus viridipes

Taxon classificationAnimaliaAraneaeSalticidae

(Hentz, 1846)

Habronattus viridipes Bradley 2013: 189; F. O. P.-Cambridge 1901: 244, m, desc. (pl. 21, figs 4, 4a-b, 5, 5a-b); Griswold 1987: 135 [T], mf, desc. (fig. 99); Hedin and Maddison 2001b: 1514; Henderson 2007: 52, 78, 81, 84; Hunter 1988: 18, 21; Jackman 1997: 130, 167; Maddison and Hedin 2003a: 20; Richman et al. 2011b: 22; Richman et al. 2012a: 22; Richman et al. 2012b: 22 [Kaston 1948: 464, mf, desc. (figs 1703–1706, 1723–1724)]
Habrocestum viridipes (Hentz, 1846); Marx 1890: 572; Peckham and Peckham 1888: 60
Pellenes viridipes (Hentz, 1846); Peckham and Peckham 1909: 574, mf, desc. (pl. 44, fig. 10, pl. 45, figs 8–8a); Vogel 1970b: 19

####### Distribution.

Brazos, Brown, Burleson, Cameron, Colorado, Coryell, Ellis, Erath, Goliad, Hidalgo, Kenedy, Montague, Presidio, San Patricio, Travis, Van Zandt

####### Locality.

Attwater Prairie Chicken National Wildlife Refuge, Bentsen-Rio Grande Valley State Park, Lick Creek Park, South Padre Island, Welder Wildlife Refuge

####### Time of activity.

Male (March – May, July – August, October); female (January, March – April, April 26-May 2, June, July 26-August 2, August, September 28-October 4)

####### Habitat.

(grass: grassland, short grass); (landscape features: under rock); (plants: vegetation); (soil/woodland: leaf litter, live oak forest, post oak savanna, post oak savanna with pasture, post oak woodland, woods)

####### Method.

Beating [m]; beating/sweeping [mf]; pitfall trap [mf]

####### Type.

South Carolina

####### Etymology.

Latin, green

####### Collection.

MSU, TAMU

##### Genus *Hasarius* Simon, 1871

###### Hasarius adansoni

Taxon classificationAnimaliaAraneaeSalticidae

(Audouin, 1826)

Hasarius adansoni Brown 1974: 236; Hill and Edwards 2013: 50; Jackman 1997: 167; Platnick 1989: 571 [spelling]
Hasarius adansonii Audouin, 1826 [Kaston 1948: 493, mf, desc. (figs 1816, 1851–1853)]

####### Distribution.

Cameron, Nacogdoches

####### Locality.

Ramsey Nature Park

####### Time of activity.

Female (May, October)

####### Habitat.

(landscape features: in culvert); (structures: on fence)

####### Type.

Egypt

####### Etymology.

Person (honor French naturalist)

##### Genus *Hentzia* Marx, 1883

###### Hentzia alamosa

Taxon classificationAnimaliaAraneaeSalticidae

Richman, 2010

Hentzia alamosa Richman 2010: 73, mf, desc. (figs 2–10, 17); Richman et al. 2011b: 23; Richman et al. 2012a: 23; Richman et al. 2012b: 23

####### Distribution.

Presidio

####### Locality.

Big Bend Ranch State Park

####### Time of activity.

Male (March); female (March, October)

####### Habitat.

(soil/woodland: cottonwood)

####### Method.

Beating [mf]

####### Type.

Texas (female, Presidio Co., Big Bend Ranch State Park, Cuevas Amarillas, March 28, 2004, D. B. Richman, holotype, TAMU)

####### Etymology.

Spanish, alamo for cottonwood

####### Collection.

TAMU

###### Hentzia fimbriata

Taxon classificationAnimaliaAraneaeSalticidae

(F. O. P.-Cambridge, 1901)

Hentzia fimbriata [Richman 1989: 306, mf, desc. (figs 37–43)]

####### Distribution.

Presidio

####### Locality.

Big Bend Ranch State Park

####### Time of activity.

Female (October)

####### Habitat.

(soil/woodland: cottonwood)

####### Method.

Beating [f]

####### Type.

Guatemala

####### Etymology.

Latin, fringed

####### Collection.

NMSU

###### Hentzia mitrata

Taxon classificationAnimaliaAraneaeSalticidae

(Hentz, 1846)

Hentzia mitrata Bradley 2013: 189; Breene et al. 1993c: 23, 48, 73, mf (figs 63A-B); Brown 1974: 236; Dean and Sterling 1990: 405; Dean et al. 1982: 255; Jackman 1997: 131, 167; Richman 1989: 302 [T], mf, desc. (figs 29–36); Richman et al. 2011b: 24; Richman et al. 2012a: 24; Richman et al. 2012b: 24; Young and Edwards 1990: 22
Wala mitrata (Hentz, 1846); Bonnet 1959: 4805; Jones 1936: 70; Vogel 1970b: 20

####### Distribution.

Bosque, Brazos, Dallas, Fannin, Jasper, Lavaca, Nacogdoches, Sabine, Travis, Uvalde, Walker, Wichita

####### Locality.

Ellis Prison Unit, Garner State Park, Lacuna Park

####### Time of activity.

Male (March – August); female (February – July, September)

####### Habitat.

(crops: cotton); (nest/prey: mud dauber nest [m]); (plants: miscellaneous vegetation); (soil/woodland: beech-magnolia forest, *Quercus buckleyi*, *Quercus virginiana*, *Ulmus crassifolia*); (structures: kitchen floor)

####### Method.

Beating [mf]; malaise trap [f]; suction trap [mf]; sweeping [mf]

####### Type.

Alabama

####### Etymology.

Latin, miter (male carapace)

####### Collection.

MSU, TAMU

###### Hentzia palmarum

Taxon classificationAnimaliaAraneaeSalticidae

(Hentz, 1832)

Hentzia palmarum Agnew et al. 1985: 5, 11; Bradley 2013: 190; Breene 1988: 15, 17, 23–26, 35–36, 38–40; Breene et al. 1988: 180–181; Breene et al. 1989: 163; Breene et al. 1993c: 23, 48, 73, mf (figs 62A-C); Brown 1974: 236; Bryant 1940: 498; Bumroongsook et al. 1992: 17; Calixto et al. 2013: 184–187; Carpenter 1972: 164; Dean and Eger 1986: 142; Dean and Sterling 1987: 7; Dean and Sterling 1990: 403, 405; Dean et al. 1982: 255; Dean et al. 1987: 268; Dean et al. 1988: 287; Hedin and Maddison 2001a: 388; Hill and Edwards 2013: 33; Hunter 1988: 18, 20–21; Jackman 1997: 131, desc., 167 (photo 41d); Li 1990: 137, 142, 144; Liao et al. 1984: 411; Lombardini et al. 2005: 1378; Nyffeler et al. 1987a: 357; Nyffeler et al. 1992b: 1459–1460; Nyffeler et al. 1992c: 3; Pamanes–Guerrero 1975: 60, 78; Pfannenstiel 2008a: 204; Rapp 1984: 9; Richman 1989: 296 [S, T], mf, desc. (figs 16–27); Richman 2010: 76; Richman and Cutler 1978: 86; Richman et al. 2011b: 24; Richman et al. 2012a: 24; Richman et al. 2012b: 24; Rogers and Horner 1977: 523; Vincent and Frankie 1985: 380; Young and Edwards 1990: 22
Hentzia ambigua (Walckenaer, 1837); Sterling et al. 1979: 979
Wala palmarum Hentz, 1832; Bonnet 1959: 4806; Jones 1936: 70; Kagan 1942: 67; Kagan 1943: 258; Peckham and Peckham 1909: 508; Petrunkevitch 1911: 717; Vogel 1970b: 20

####### Distribution.

Eastern 2/3 Texas; Aransas, Austin, Bell, Bexar, Bosque, Brazoria, Brazos, Brown, Burleson, Cameron, Collin, Comal, Comanche, Dallas, Edwards, Ellis, Erath, Falls, Galveston, Gillespie, Goliad, Grayson, Hidalgo, Hill, Houston, Hunt, Karnes, Kenedy, Kerr, Limestone, Llano, Lubbock, Mason, McLennan, Medina, Montague, Montgomery, Motley, Nacogdoches, Nolan, Nueces, Randall, Robertson, Rockwall, Sabine, San Patricio, San Saba, Scurry, Shelby, Travis, Uvalde, Walker, Wharton, Wichita, Wilbarger, Williamson, Wood, Zavala

####### Locality.

Adriance Pecan Orchard, Bentsen-Rio Grande Valley State Park, Bill Haney Pecan Orchard, Brison Pecan Orchard, Buddy Adams Pecan Orchard, Ellis Prison Unit, Estero Llano Grande State Park, Frontera Audubon, Holmes Pecan Orchard, Jones State Forest, Lacuna Park, Lake Thomas, Lick Creek Park, McKelvey Park, Proctor Lake, Ramsey Prison Farm, Resaca de la Palma State Park, Russell Farm, Storey Pecan Orchard, Texas A&M University Rangeland Area, Welder Wildlife Refuge

####### Time of activity.

Male (January – December); female (January, March – December)

####### Habitat.

(crops: cotton, guar, peanuts, sunflower); (grass: grasses, grassland, grassy and shrub area, pasture, tall grass prairie); (littoral: salt marsh area); (nest/prey: bird nest, mud dauber nest [mf]); (objects: on cotton fleahopper emergence cage); (orchard: grapefruit, Mexican lime, orange, pecan, sour orange, tangerine); (plants: bluebonnets, bush, croton, Indian paintbrush, miscellaneous vegetation, next to cotton field, vegetation, weed, *Monarda citriodora*); (soil/woodland: beech-magnolia forest, hackberry matte, live oak, lower branches of trees, post oak savanna, sandy area, tree, trees/shrubs, willow, woods, woodland, woody annuals, *Quercus buckleyi*, *Quercus virginiana*, *Ulmus crassifolia*); (structures: on clothes line)

####### Method.

Beating [mf]; beating/sweeping [f]; boll weevil pheromone trap [f]; cardboard band [mf]; D-Vac suction [mf]; fogging [mf]; irrigation tubing [mf]; malaise trap [f]; ramp trap [m]; suction trap [mf]; sweeping [mf]

####### Eggs/spiderlings.

Robertson [12, 17 spiderlings]; Walker [eggsac August 28, 1978, hatch October 3; 28 spiderlings] [TAMU]

####### Type.

South Carolina and North Carolina

####### Etymology.

Latin, plant

####### Collection.

MSU, NMSU, TAMU, WTAM

##### Genus *Leptofreya* Edwards, 2015

###### Leptofreya ambigua

Taxon classificationAnimaliaAraneaeSalticidae

(C.L. Koch, 1846)

Leptofreya ambigua Edwards 2015: 43 [T]
Freya ambigua (C. L. Koch, 1846); Edwards and Ruiz 2013: 13, mf, desc. (figs 1–13)
Freya perelegans Simon, 1902; Richman et al. 2012a: 11; Richman et al. 2012b: 11
Freya sp.; Richman et al. 2011b: 11

####### Distribution.

Cameron, Hidalgo

####### Locality.

Bentsen-Rio Grande Valley State Park, Estero Llano Grande State Park, Laguna Atascosa National Wildlife Refuge, Lower Rio Grande Valley National Wildlife Refuge, Ramsey Nature Park, Resaca de la Palma State Park, Sabal Palm Audubon Sanctuary, Santa Ana National Wildlife Refuge

####### Time of activity.

Male (March, May, September 20-October 3, October, November); female (March, April 24-May 7, September – November)

####### Habitat.

(littoral: dense coastal brush, grass survey); (soil/woodland: cedar elm forest, ebony-guayacan association, palm forest, re-vegetated site)

####### Method.

Flight intercept trap on ground [mf]; pitfall trap [mf]

####### Type.

Suriname

####### Etymology.

Latin, doubtful

####### Collection.

FSCA, TAMU

##### Genus *Lyssomanes* Hentz, 1845

###### Lyssomanes viridis

Taxon classificationAnimaliaAraneaeSalticidae

(Walckenaer, 1837)

Lyssomanes viridis Breene et al. 1993c: 23, 48, 63, mf (figs 38A-C); Dean et al. 1982: 255; Jackman 1997: 131, desc., 167 (photo 41c); Kaston 1953: 46, desc. (fig. 98); Kaston 1972: 277, desc. (fig. 625); Kaston 1978: 265, desc. (fig. 677); Peckham and Peckham 1909: 595, mf, desc.; Richman et al. 2005: 208; Richman et al. 2011b: 26; Richman et al. 2012a: 26; Richman et al. 2012b: 26; Vogel 1970b: 17; Young and Edwards 1990: 22

####### Distribution.

Anderson, Bastrop, Brazos, Sabine, San Jacinto, Tyler, Walker

####### Locality.

Bastrop State Park, Ellis Prison Unit, Kirby State Forest, Lick Creek Park, Sam Houston National Forest

####### Time of activity.

Male (April – May, May 19-June 7); female (April – June, June 23-July 2, July 24-August 6)

####### Habitat.

(crops: cotton); (soil/woodland: beech-magnolia forest, forest understory)

####### Method.

Beating [f]; beating/sweeping [f]; flight intercept trap on ground [m]; malaise trap [f]; sweeping [m]

####### Type.

Georgia

####### Etymology.

Latin, green

####### Collection.

MCZ, MSU, TAMU

##### Genus *Maevia* C. L. Koch, 1846

###### Maevia inclemens

Taxon classificationAnimaliaAraneaeSalticidae

(Walckenaer, 1837)

Maevia inclemens Agnew et al. 1985: 5; Barnes 1955: 2 [S], mf, desc. (figs 1, 4, 7–8); Carpenter 1972: 165; Hunter 1988: 18, 20–21; Jackman 1997: 132, desc., 167; Kaston 1972: 258, desc. (fig. 581); Kaston 1978: 247, desc. (fig. 628); Richman and Cutler 1978: 87; Richman et al. 2011b: 27; Richman et al. 2012a: 26; Richman et al. 2012b: 26; Tugmon et al. 1990: 44; Vogel 1970b: 18; Young and Edwards 1990: 22
Maevia vittata (Hentz, 1846); Bonnet 1957: 2697; Jones 1936: 69

####### Distribution.

Angelina, Bandera, Bexar, Brazoria, Brazos, Brewster, Burleson, Clay, Comanche, Dallas, Ellis, Erath, Hays, Kerr, Llano, Tarrant, Travis, Wichita, Young

####### Locality.

Chisos Basin, Chisos Mountains, Grissom Ranch, Lost Maples State Park, Nabor’s Lake, Ramsey Prison Farm

####### Time of activity.

Male (April – September); female (April – October, December 16-January 26)

####### Habitat.

(crops: peanuts); (grass: grass); (plants: low succulent vegetation, weed); (soil/woodland: ground, *Juniperus* managed plot, *Juniperus* unmanaged plot, post oak savanna with pasture, upland deciduous forest, woods, woodland, *Quercus buckleyi*); (structures: house, on wall, side of house)

####### Method.

Flight intercept trap elevated [f]; flight intercept trap on ground [mf]; Lindgren flight trap [f]; pitfall trap [m]; sweeping [mf]

####### Type.

Georgia

####### Etymology.

Latin, unfavorable behavior, cruel

####### Collection.

MSU, TAMU

##### Genus *Marpissa* C. L. Koch, 1846

###### Marpissa bryantae

Taxon classificationAnimaliaAraneaeSalticidae

(Jones, 1945)

Marpissa bryantae Jackman 1997: 167; Logunov 1999: 44 (figs 86–88); Richman and Cutler 1978: 87 [T]; Richman et al. 2011b: 28; Richman et al. 2012a: 27; Richman et al. 2012b: 27
Hyctia bryantae Jones, 1945; Jones 1945: 39, f, desc. (fig. 1); Roewer 1955: 1259; Vogel 1967: 116; Vogel 1970b: 17

####### Distribution.

Denton

####### Time of activity.

Female (March)

####### Habitat.

(plants: herbs)

####### Method.

sweeping [f]

####### Type.

Texas (female, Denton Co., Denton, March 26, 1942, no collector, holotype, MCZ)

####### Etymology.

Person (arachnologist)

###### Marpissa dentoides

Taxon classificationAnimaliaAraneaeSalticidae

Barnes, 1958

Marpissa dentoides Logunov 1999: 35 [S], mf (figs 66–67, 82–83)
Marpissa obtusa Barnes, 1958; Barnes 1958: 28, f (fig. 46 [not m]); Vogel 1970b: 18 [part, West Texas records]

####### Distribution.

Brewster, Kerr, Llano

####### Locality.

Big Bend National Park, Chisos Basin, Chisos Mountains

####### Time of activity.

Female (June, September, December)

####### Type.

New York, Sea Cliff

####### Etymology.

Latin, referring to teeth

###### Marpissa formosa

Taxon classificationAnimaliaAraneaeSalticidae

(Banks, 1892)

Marpissa formosa Agnew et al. 1985: 8; Barnes 1958: 4 [S], mf, desc. (figs 4–11); Breene et al. 1993b: 648; Breene et al. 1993c: 23, 48, 66, mf (figs 45A-D); Cokendolpher 1978c: 118; Dean et al. 1982: 255; Jackman 1997: 167; Logunov 1999: 44, f (figs 70–71); Richman and Cutler 1978: 87; Richman et al. 2011b: 28; Richman et al. 2012a: 28; Richman et al. 2012b: 28; Vogel 1970b: 18; Young and Edwards 1990: 22
Hyctia bina (Hentz, 1846); Jones 1936: 70; Vogel 1970b: 17; Woods and Harrel 1976: 44 [Texas records]
Marpissa bina (Hentz, 1846); Brown 1974: 236; Carpenter 1972: 166 [Texas records]

####### Distribution.

Archer, Cameron, Cherokee, Comal, Dallas, Erath, Hardeman, Hidalgo, Jefferson, Kerr, Rusk, San Patricio, Walker, Wichita

####### Locality.

Ellis Prison Unit, Lake Striker, Lake Wichita, Medicine Mounds Ranch

####### Time of activity.

Male (March, May – August, October); female (March – October, December)

####### Habitat.

(crops: cotton, rice, sugarcane); (grass: grass); (littoral: creek bank, creek bank vegetation, pond shore); (objects: under canvas); (plants: vegetation); (structures: on table on boat pier)

####### Method.

Boll weevil pheromone trap [m]; sweeping [m]

####### Type.

New York

####### Etymology.

Latin, beautiful

####### Collection.

MCZ, MSU, TAMU, WTAM

###### Marpissa lineata

Taxon classificationAnimaliaAraneaeSalticidae

(C. L. Koch, 1846)

Marpissa lineata Agnew et al. 1985: 5; Breene et al. 1993b: 648; Breene et al. 1993c: 23, 48, 66, mf (figs 44A-C); Calixto et al. 2013: 184; Cokendolpher 1978c: 118; Dean et al. 1982: 255; Henderson 2007: 56, 78, 81, 84; Jackman 1997: 167; Richman and Cutler 1978: 87; Richman et al. 2011b: 28; Richman et al. 2012a: 28; Richman et al. 2012b: 28; Young and Edwards 1990: 22 [Barnes 1958: 23, mf, desc. (figs 35–40)]

####### Distribution.

Angelina, Archer, Blanco, Brazos, Burleson, Cameron, Colorado, Coryell, Erath, Hidalgo, Robertson, Sabine, Walker, Wichita, Willacy

####### Locality.

Angelina National Forest, Attwater Prairie Chicken National Wildlife Refuge, Browning Ranch, Ellis Prison Unit, Holmes Pecan Orchard, Lick Creek Park

####### Time of activity.

Male (March – July, September – November); female (March, May, August – October)

####### Habitat.

(crops: cotton, peanuts, sugarcane); (objects: under board); (orchard: pecan); (soil/woodland: beech-magnolia forest, hardwood bottomland, post oak savanna with pasture, sandy area on ground, under juniper, upland woods); (structures: indoors)

####### Method.

cardboard band [f]; flight intercept trap [m]; pitfall trap [mf] (under juniper [m])

####### Type.

Pennsylvania

####### Etymology.

Latin, carapace with thin black band on margin

####### Collection.

MSU, TAMU

###### Marpissa obtusa

Taxon classificationAnimaliaAraneaeSalticidae

Barnes, 1958

Marpissa obtusa Barnes 1958: 28, m, desc. (fig. 44 [not f]); Irungu 2007: 31; Jackman 1997: 167; Logunov 1999: 40, m, desc. (figs 80–81); Richman and Cutler 1978: 88; Richman et al. 2011b: 29; Richman et al. 2012a: 28; Richman et al. 2012b: 28; Vogel 1967: 117; Vogel 1970b: 18

####### Distribution.

Brewster, Hidalgo, Kerr, Matagorda

####### Locality.

Big Bend National Park, Chisos Basin, Chisos Mountains, Lower Rio Grande Valley National Wildlife Refuge

####### Time of activity.

Male (June, October, October 26-November 2)

####### Habitat.

(crops: cabbage); (soil/woodland: re-vegetated site)

####### Method.

pitfall trap [m]

####### Type.

Texas (male, Matagorda Co., Palacios, June 4, 1936, S. Mulaik, holotype, AMNH)

####### Etymology.

Latin, round form

####### Collection.

MSU, TAMU

###### Marpissa pikei

Taxon classificationAnimaliaAraneaeSalticidae

(Peckham & Peckham, 1888)

Marpissa pikei Agnew et al. 1985: 5; Armstrong and Richman 2007: 396; Barnes 1958: 15, mf, desc. (figs 16–21); Breene et al. 1993b: 648; Breene et al. 1993c: 23, 48, 65, mf (figs 43A-D); Brown 1974: 236; Carpenter 1972: 166; Cokendolpher et al. 2008: 10, 49; Dean and Eger 1986: 142; Dean and Sterling 1987: 7; Hunter 1988: 18–20; Jackman 1997: 133, desc., 167; Rapp 1984: 9; Richman et al. 2011b: 29; Richman et al. 2012a: 28; Richman et al. 2012b: 28; Roberts 2001: 51; Tugmon et al. 1990: 44; Vogel 1970b: 18; Vogel and Durden 1972: 1; Young and Edwards 1990: 22

####### Distribution.

Brazoria, Brazos, Burnet, Cameron, Carson, Colorado, Dallas, Dickens, Ellis, Erath, Fannin, Floyd, Freestone, Frio, Galveston, Hamilton, Hardeman, Henderson, Hidalgo, Jack, Jeff Davis, Kaufman, Kent, Kerr, McMullen, Milam, Nacogdoches, Nueces, Oldham, Potter, Randall, Stephens, Travis, Uvalde, Val Verde, Wichita

####### Locality.

Attwater Prairie Chicken National Wildlife Refuge, Davis Mountains, Garner State Park, Inks Lake State Park, Lick Creek Park, Medicine Mounds Ranch, Pantex Plant, Seminole Canyon State Park, South Padre Island, Wildcat Bluff Nature Center

####### Time of activity.

Male (April – December); female (April – October)

####### Habitat.

(crops: cotton, peanuts, sugarcane); (grass: Bermuda grass, grass, grassland, Johnson grass, tall grass); (landscape features: under rock); (littoral: near playa, salt marsh area); (nest/prey: mud dauber nest [f]); (plants: bluebonnets, emergent vegetation, garden, miscellaneous vegetation, roadside vegetation, vegetation, weed); (structures: ceiling of picnic table); (soil/woodland: on ground, post oak savanna, trees/shrubs)

####### Method.

Beating [mf]; boll weevil pheromone trap [imm.]; D-Vac suction [mf]; pitfall trap; sweeping [mf]

####### Type.

New York, South Carolina, Georgia, Florida

####### Etymology.

Person (collector, Nicolas G. Pike)

####### Collection.

DMNS, MSU, TAMU, WTAM

##### Genus *Menemerus* Simon, 1868

###### Menemerus bivittatus

Taxon classificationAnimaliaAraneaeSalticidae

(Dufour, 1831)

Menemerus bivittatus Barnes 1958: 44, mf, desc. (figs 71–74); Carpenter 1972: 166; Jackman 1997: 167; Richman and Cutler 1978: 88; Richman et al. 2011b: 29; Richman et al. 2012a: 29; Richman et al. 2012b: 29; Vogel 1970b: 18

####### Distribution.

Brazos, Cameron, Hidalgo, Jefferson, Kleberg, Montague, Nueces, Wichita

####### Locality.

Estero Llano Grande State Park, Resaca de la Palma State Park, Russell Farm

####### Time of activity.

Male (February, April, August – December); female (May, August, October, December)

####### Habitat.

(grass: grass); (landscape features: under stone); (nest/prey: mud dauber nest [m]); (objects: wood pile); (soil/woodland: mesquite)

####### Type.

unknown

####### Etymology.

Latin, two stripes

####### Collection.

MSU, NMSU, TAMU

##### Genus *Messua* Peckham & Peckham, 1896

###### Messua limbata

Taxon classificationAnimaliaAraneaeSalticidae

(Banks, 1898)

Messua limbata Armstrong and Richman 2007: 396; Bradley 2013: 192; Maddison 1996: 233 [T], m (figs 90, 100, 117); Richman et al. 2005: 216; Richman et al. 2011b: 30 [S]; Richman et al. 2012a: 30; Richman et al. 2012b: 30
Dendryphantes limbatus (Banks, 1898); Banks 1910: 66; Chamberlin 1924b: 682; Peckham and Peckham 1901a: 315, mf, desc.; Peckham and Peckham 1909: 458, mf, desc. (pl. 37, fig. 3); Petrunkevitch 1911: 634; Roewer 1955: 1203
Eris limbata (Banks, 1898); Breene et al. 1993b: 648; Dean and Eger 1986: 142; Jackman 1997: 167 (photo 41b); Richman and Cutler 1978: 85
Eris limbatus (Banks, 1898); Vogel 1970b: 17
Icius exornatus (Peckham and Peckham, 1909); Banks 1910: 71; Bonnet 1957: 2281; Peckham and Peckham 1909: 505, f, desc. (pl. 41, fig. 6); Petrunkevitch 1911: 661; Roewer 1955: 1222; Vogel 1970b: 17

####### Distribution.

Austin, Brazos, Cameron, Frio, Hidalgo, Kerr

####### Locality.

Bentsen-Rio Grande Valley State Park, Estero Llano Grande State Park, Frontera Audubon, Laguna Atascosa National Wildlife Refuge, Lower Rio Grande Valley National Wildlife Refuge, Resaca de la Palma State Park, Riley Estate, Russell Farm, Sabal Palm Audubon Sanctuary

####### Time of activity.

Male (January, March – April, June – August, October – November); female (February – May, July – December)

####### Habitat.

(crops: cotton, sugarcane); (grass: grass); (littoral: dense coastal brush); (nest/prey: mud dauber nest [mf]); (orchard: grapefruit, Mexican lime, orange, organic citrus grove, sour orange); (plants: bluebonnets); (soil/woodland: cedar elm forest, ebony-guayacan association, palm forest margin [resaca bank], trees)

####### Method.

Beating [f]; boll weevil pheromone trap [m]; D-Vac suction [m]; flight intercept trap elevated [m]; flight intercept trap on ground [m]; Lindgren flight trap [mf]; sweeping [m]

####### Type.

Mexico, Tepic

####### Etymology.

Latin, bordered

####### Collection.

DMNS, NMSU, TAMU

##### Genus *Metacyrba* F. O. P.-Cambridge, 1901

###### Metacyrba floridana

Taxon classificationAnimaliaAraneaeSalticidae

Gertsch, 1934

Metacyrba floridana Edwards 2006: 197, mf, desc. (figs 1–12, 34, 98); Richman et al. 2011b: 30; Richman et al. 2012a: 30; Richman et al. 2012b: 30

####### Distribution.

Dallas, Hays, Kerr, Nacogdoches

####### Locality.

White Rock Lake

####### Time of activity.

Male (August); female (September, October 27-November 11)

####### Habitat.

(soil/woodland: *Juniperus* managed plot, upland deciduous forest)

####### Method.

Flight intercept trap on ground [f]; Lindgren flight trap [f]

####### Type.

Florida, Fort Meyers

####### Etymology.

locality (state)

####### Collection.

TAMU

###### Metacyrba punctata

Taxon classificationAnimaliaAraneaeSalticidae

(Peckham & Peckham, 1894)

Metacyrba punctata Barnes 1958: 35, mf, desc. (fig. 54); Edwards 2006: 199, mf, desc. (figs 35–46, 97); Jackman 1997: 167; Richman and Cutler 1978: 88; Richman et al. 2011b: 30; Richman et al. 2012a: 30; Richman et al. 2012b: 30; Vogel 1970b: 18

####### Distribution.

Cameron

####### Locality.

McKelvey Park, Ramsey Nature Park, Resaca de la Palma State Park, Sabal Palm Audubon Sanctuary

####### Time of activity.

Male (November); female (October, December)

####### Habitat.

(soil/woodland: palm forest)

####### Method.

Beating [f]

####### Type.

Central America

####### Etymology.

Latin, six white spots on abdomen

####### Collection.

TAMU

###### Metacyrba taeniola similis

Taxon classificationAnimaliaAraneaeSalticidae

Banks, 1904

Metacyrba taeniola similis Edwards 2006: 201, mf, desc. (figs 47–67); Richman et al. 2011b: 30; Richman et al. 2012a: 30; Richman et al. 2012b: 30
Metacyrba taeniola (Hentz, 1846); Vogel 1970b: 18 [part]

####### Distribution.

Anderson, Brazos, Brewster, Cameron, Dickens, El Paso, Hays, Jeff Davis, Lubbock, Tyler, Wichita

####### Locality.

Big Bend National Park, Chisos Basin, Chisos Mountains, Davis Mountains, Engeling Wolf Management Area, Laguna Atascosa National Wildlife Refuge, Sabal Palm Audubon Sanctuary, Texas A&M University Rangeland Area

####### Time of activity.

Male (March, June – August, October); female (April – May, July – September, December)

####### Habitat.

(littoral: dense coastal brush); (plants: miscellaneous vegetation); (soil/woodland: *Juniperus* managed plot, *Juniperus* unmanaged plot, palm forest, palm tree, trees/shrubs , under bark); (structures: window of house)

####### Method.

Beating [f]; flight intercept trap [f]; flight intercept trap elevated [mf]; flight intercept trap on ground [m]

####### Eggs/spiderlings.

Brazos [eggs hatch, May 21, 1984, 18 spiderlings] [TAMU]

####### Type.

California, Los Angeles

####### Etymology.

Latin, similar to *Metacyrba taeniola* (Hentz, 1846)

####### Collection.

TAMU

###### Metacyrba taeniola taeniola

Taxon classificationAnimaliaAraneaeSalticidae

(Hentz, 1846)

Metacyrba taeniola taeniola Edwards 2006: 204, mf, desc. (figs 68–78, 99) [see note below]; Richman et al. 2011b: 30; Richman et al. 2012a: 30; Richman et al. 2012b: 30
Metacyrba taeniola (Hentz, 1846); Agnew et al. 1985: 8; Barnes 1958: 30, mf, desc. (figs 47–51); Brown 1974: 236; Carpenter 1972: 166; Hunter 1988: 18, 20; Jackman 1997: 133, desc., 167; Jones 1936: 69; Milstead 1958: 446; Peckham and Peckham 1909: 486; Petrunkevitch 1911: 673; Vogel 1970b: 18

####### Distribution.

Archer, Bastrop, Bell, Bexar, Bosque, Brazos, Brewster, Brown, Burleson, Caldwell, Cameron, Clay, Coryell, Crockett, Dallas, El Paso, Ellis, Erath, Hidalgo, Kenedy, Kerr, Nacogdoches, Presidio, San Patricio, Starr, Sutton, Travis, Uvalde, Val Verde, Waller, Wichita, Zapata

####### Locality.

Bentsen-Rio Grande Valley State Park, Big Bend National Park, Black Gap Wildlife Management Area, Chisos Basin, Chisos Mountains, Davis Mountains, Falcon Lake State Park, Laguna Atascosa National Wildlife Refuge, Lick Creek Park, Lomita Ranch, Lower Rio Grande Valley National Wildlife Refuge, Raven Ranch

####### Time of activity.

Male (January – October, December); female (January – December)

####### Habitat.

(grass: under rock in grassland); (landscape features: crevice in rocky ledge, loose stones, under rock); (nest/prey: in nest on shelter, stomach of *Cnemidophorus tigris*); (plants: flower, Indian paintbrush, miscellaneous vegetation, weeds); (soil/woodland: forest, in log, *Juniperus* managed plot, *Juniperus* unmanaged plot, oak tree, palm tree, post oak savanna with pasture, re-vegetated site, savanna with native grasses, under bark, woods, *Ulmus crassifolia*); (structures: in house, inside on window, on [table on patio, window patio], window, window screen)

####### Method.

Beating [f]; beating/sweeping [f]; cardboard band [m]; flight intercept trap on ground [mf]; Lindgren flight trap [m]; pitfall trap [mf]; suction trap [f]; sweeping [f]

####### Type.

North Carolina and Alabama

####### Etymology.

Latin, a band or ribbon

####### Collection.

MSU, NMSU, TAMU

####### Note.

32 miles SE Laredo is in Zapata Co., not Webb Co. based on other records from this date.

##### Genus *Metaphidippus* F. O. P.-Cambridge, 1901

###### Metaphidippus chera

Taxon classificationAnimaliaAraneaeSalticidae

(Chamberlin, 1924)

Metaphidippus chera Breene et al. 1993c: 23, 48, 72, mf (figs 60A-B); Calixto et al. 2013: 184; Jackman 1997: 167; Knutson et al. 2010: 515; Maddison 1996: 315 [S], mf, desc. (figs 33, 186–187, 233, 257, 514–528); Richman et al. 2011b: 31; Richman et al. 2012a: 31; Richman et al. 2012b: 31
Metaphidippus manni (Peckham and Peckham, 1901); Carpenter 1972: 163; Kaston 1978: 262, desc.; Richman and Cutler 1978: 90 [Texas records]
Metaphidippus sp. cf. manni (Peckham and Peckham, 1901); Agnew et al. 1985: 8; Dean and Sterling 1987: 7 [Texas records]

####### Distribution.

Western 2/3 Texas; Archer, Baylor, Bexar, Cameron, Clay, Dawson, Erath, Foard, Hardeman, Haskell, Hidalgo, Howard, Jones, Kerr, Martin, Presidio, Reagan, Robertson, Runnels, Scurry, Terry, Wichita, Winkler

####### Locality.

Anzalduas County Park, Bentsen-Rio Grande Valley State Park, Holmes Pecan Orchard, Lake Thomas, McKelvey Park, Medicine Mounds Ranch, Ramsey Nature Park

####### Time of activity.

Male (January – October); female (March – May, July – October)

####### Habitat.

(crops: cotton, sunflower); (grass: grasses, grassland, meadow); (orchard: pecan); (plants: low annuals and weeds, vegetation, *Baccharis*); (soil/woodland: desert brushland, mesquite, mesquite brush, mesquite brushland, saltcedar, on tree, trees/shrubs, under bark, willow)

####### Method.

Beating [m]; cardboard band [m]; suction trap

####### Type.

Mexico, Baja California, San Jose Island

####### Etymology.

Greek, widow

####### Collection.

DMNS, MSU, TAMU, WTAM

###### Metaphidippus felix

Taxon classificationAnimaliaAraneaeSalticidae

(Peckham & Peckham, 1901)

Metaphidippus felix Richman & Cutler 1978: 89 [T]; Richman et al. 2011b: 32; Richman et al. 2012a: 32; Richman et al. 2012b: 32
Dendryphantes felix Peckham & Peckham, 1901 [Peckham and Peckham 1901a: 313a, m, desc. (pl. 27, f. 6–6a)]
Bagheera felix (Peckham & Peckham, 1901); Jackman 1997: 167 [wrong generic name used]

####### Distribution.

Hidalgo

####### Locality.

Santa Ana National Wildlife Refuge

####### Time of activity.

Male (October); female (October)

####### Habitat.

(grass: native meadow)

####### Method.

sweeping [mf]

####### Type.

Mexico

####### Etymology.

Latin, fruitful

####### Collection.

NMSU

###### Metaphidippus longipalpus

Taxon classificationAnimaliaAraneaeSalticidae

F. O. P.-Cambridge, 1901

Metaphidippus longipalpus Bryant 1933: 192; Richman et al. 2011b: 32 (questionable); Richman et al. 2012a: 32 (questionable); Richman et al. 2012b: 32 (questionable) [F. O. P.-Cambridge 1901: 264, m (pl. 23, figs 12a-c)]

####### Distribution.

Cameron

####### Type.

Panama

[female unknown]

####### Etymology.

Latin, long palp

###### Metaphidippus texanus

Taxon classificationAnimaliaAraneaeSalticidae

(Banks, 1904)

Metaphidippus texanus Edwards 1980: 12 [T]; Jackman 1997: 167
Dendryphantes texanus (Banks, 1904); Petrunkevitch 1911: 642; Roewer 1955: 1216
Icius texanus Banks, 1904; Banks 1904: 116, f, desc. (fig. 15); Banks 1910:72; Bonnet 1957: 2284; Richman and Cutler 1978: 86; Roewer 1955: 1222; Vogel 1970b: 17

####### Distribution.

Hidalgo

####### Type.

Texas (female, Hidalgo Co., holotype, no date, no collector)

####### Etymology.

locality (state)

##### Genus *Mexigonus* Edwards, 2003

###### Mexigonus minutus

Taxon classificationAnimaliaAraneaeSalticidae

(F. O. P.-Cambridge, 1901)

Mexigonus minutus Edwards 2003: 70 [T], mf (figs 9–12)
Tylogonus minutus (F. O. P.-Cambridge, 1901); Jackman 1997: 168; Richman 1981: 198

####### Distribution.

Texas

####### Type.

Mexico

####### Etymology.

Latin, size

##### Genus *Naphrys* Edwards, 2003

###### Naphrys acerba

Taxon classificationAnimaliaAraneaeSalticidae

(Peckham & Peckham, 1909)

Naphrys acerba Edwards 2003: 69 [T], 73, mf (figs 5–8); Richman et al. 2011b: 34; Richman et al. 2012a: 34; Richman et al. 2012b: 34
Habrocestum acerbum Peckham and Peckham, 1909; Banks 1910: 69; Cokendolpher 1978c: 118; Cokendolpher and Reddell 2001b: 53; Hill 1979: 194; Jackman 1997: 167; Peckham and Peckham 1909: 522; Petrunkevitch 1911: 655; Richman 1981: 202, mf, desc. (figs 12–15); Richman and Cutler 1978: 85; Roewer 1955: 1120; Vincent and Frankie 1985: 380; Vogel 1970b: 17

####### Distribution.

Southern 2/3 Texas; Aransas, Bandera, Bastrop, Bell, Brazos, Brewster, Cameron, Comal, Culberson, DeWitt, Edwards, Hays, Hidalgo, Hood, Kendall, Kerr, Kimble, Llano, Mason, McLennan, Nueces, Real, San Patricio, Starr, Terrell, Travis, Uvalde, Zapata

####### Locality.

Bentsen-Rio Grande Valley State Park, Estero Llano Grande State Park, Fort Hood, Lost Maples State Park, Lower Rio Grande Valley National Wildlife Refuge, Sabal Palm Audubon Sanctuary, Texas A&M University Rangeland Area

####### Caves.

**Bell** (Road Side Sink [Fort Hood])

####### Time of activity.

Male (March – April, April 26-May 10, July – September); female (March 4-April 3, March 26-April 2, April – July)

####### Habitat.

(grass: grass); (landscape features: cave); (soil/woodland: live oak, oak-cedar scrub, riparian mesquite forest)

####### Method.

carrion pitfall trap [mf]; flight intercept trap [f]; pitfall trap [mf]

####### Type.

Texas (male, female, Travis Co., Austin, no date, no collector, syntype, MCZ)

####### Etymology.

Latin, rough

####### Collection.

MSU, NMSU, TAMU, TMM

###### Naphrys pulex

Taxon classificationAnimaliaAraneaeSalticidae

(Hentz, 1846)

Naphrys pulex Edwards 2003: 69 [T]; Richman et al. 2011b: 34; Richman et al. 2012a: 34; Richman et al. 2012b: 34
Habrocestum pulex (Hentz, 1846); Agnew et al. 1985: 8; Brown 1974: 236; Jackman 1997: 167; Richman 1981: 200, mf, desc. (figs 2, 8–11)

####### Distribution.

Angelina, Bandera, Bowie, Brazos, Burleson, Coryell, Erath, Gonzales, Hidalgo, Jasper, Nacogdoches, Newton, Sabine, San Jacinto, San Patricio, Tyler

####### Locality.

Bentsen-Rio Grande Valley State Park, Lick Creek Park, Lost Maples State Park, Palmetto State Park, Somerville Lake, Texas A&M University Rangeland Area, Welder Wildlife Refuge

####### Time of activity.

Male (April – September, November); female (May – September)

####### Habitat.

(soil/woodland: anaqua groves, clay soil brushland, hackberry woodland, leaf litter, live oak woodland, longleaf pine managed, post oak savanna with pasture, woods); (structures: side of house)

####### Method.

Blue pan trap [m]; carrion pitfall trap [m]; pitfall trap [mf] (in woods [m])

####### Type.

Alabama

####### Etymology.

Latin, flea

####### Collection.

MSU, TAMU

##### Genus *Neon* Simon, 1876

###### Neon nelli

Taxon classificationAnimaliaAraneaeSalticidae

Peckham & Peckham, 1888

Neon nelli Bradley 2013: 194; Platnick 1998: 912 [spelling]; Richman et al. 2011b: 35; Richman et al. 2012a: 35; Richman et al. 2012b: 35
Neon nellii Peckham and Peckham, 1888; Jackman 1997: 167 [Gertsch and Ivie 1955: 11, mf, desc. (figs 9–11, 15)]

####### Distribution.

Brewster

####### Locality.

Big Bend National Park

####### Time of activity.

Female (September)

####### Habitat.

(soil/woodland: mixed hardwood leaf litter)

####### Method.

Berlese funnel [f]

####### Type.

Pennsylvania and Canada

####### Etymology.

Person (collector, Philip Nell)

####### Collection.

TAMU

##### Genus *Neonella* Gertsch, 1936

###### Neonella vinnula

Taxon classificationAnimaliaAraneaeSalticidae

Gertsch, 1936

Neonella vinnula Agnew et al. 1985: 5; Bonnet 1958: 3053; Gertsch 1936: 24, mf, desc. (figs 28–29); Jackman 1997: 167; Richman and Cutler 1978: 91; Richman et al. 2005: 213; Richman et al. 2011b: 35; Richman et al. 2012a: 35; Richman et al. 2012b: 35; Roth 1982: 40–5; Roth 1985: B-36–4; Roth 1994: 156; Vogel 1970b: 18; Young and Edwards 1990: 22 [erroneously listed under cotton]

####### Distribution.

Brazos, Burleson, Cameron, Colorado, Coryell, Erath, Hidalgo

####### Locality.

Attwater Prairie Chicken National Wildlife Refuge, Riley Estate

####### Time of activity.

Male (May 29-June 5, June, December); female (March 30-April 6, April, May 29-June 5, July, September, December)

####### Habitat.

(crops: peanuts); (soil/woodland: post oak savanna with pasture)

####### Method.

suction trap [m]; pitfall trap [mf]

####### Type.

Florida, Petersburg

####### Etymology.

Latin, delightful

####### Collection.

TAMU

##### Genus *Paradamoetas* Peckham & Peckham, 1885

###### Paradamoetas formicinus

Taxon classificationAnimaliaAraneaeSalticidae

Peckham & Peckham, 1885

Paradamoetas formicinus Platnick 2000 [spelling]; Richman et al. 2005: 209, 212; Richman et al. 2011b: 36 (questionable); Richman et al. 2012a: 36 (questionable); Richman et al. 2012b: 36 (questionable); Roewer 1955: 1223
Paradamoetas formicina Peckham and Peckham, 1885; Banks 1910: 75; Cutler 1981: 210, mf, desc. (figs 1, 6–8, 11–12, 21–22); Cutler 1982: 220, f (fig. 3b); Jackman 1997: 167; Peckham and Peckham 1909: 375; Petrunkevitch 1911: 678; Richman and Cutler 1978: 91

####### Distribution.

South Texas

####### Type.

Guatemala

####### Etymology.

Latin, referring to ants

##### Genus *Paramaevia* Barnes, 1958

###### Paramaevia poultoni

Taxon classificationAnimaliaAraneaeSalticidae

(Peckham & Peckham, 1901)

Paramaevia poultoni Barnes 1955: 7 [T], mf, desc. (figs 10–12); Vogel 1970b: 18
Maevia poultoni Peckham and Peckham, 1901; Armstrong and Richman 2007: 396; Banks 1910: 71; Jackman 1997: 167; Petrunkevitch 1911: 668; Richman and Cutler 1978: 87; Richman et al. 2011b: 27; Richman et al. 2012a: 27; Richman et al. 2012b: 27
Maevia poultonii Peckham and Peckham, 1901; Comstock 1912: 694; Comstock 1940: 702; Peckham and Peckham 1901a: 344; Peckham and Peckham 1909: 451; Roewer 1955: 1015

####### Distribution.

Bexar, Cameron, Hidalgo, Kerr, Kleberg, Starr, Travis

####### Locality.

Estero Llano Grande State Park, Laguna Atascosa National Wildlife Refuge, Laguna Madre, Resaca de la Palma State Park, Russell Farm, Sabal Palm Audubon Sanctuary

####### Time of activity.

Male (February 27-March 11, March – April, September – October); female (March – May, July – August, October)

####### Habitat.

(littoral: dense coastal brush); (soil/woodland: palm forest, upland deciduous forest); (structures: inside house)

####### Method.

Boll weevil pheromone trap [m]; flight intercept trap elevated [m]; flight intercept trap on ground [mf]

####### Type.

Texas (male, female, Bexar Co., San Antonio, no date, no collector)

####### Etymology.

Person

####### Collection.

NMSU

##### Genus *Paramarpissa* F. O. P.-Cambridge, 1901

###### Paramarpissa piratica

Taxon classificationAnimaliaAraneaeSalticidae

(Peckham & Peckham, 1888)

Paramarpissa piratica Knutson et al. 2010: 515; Logunov and Cutler 1999: 1230 [T], mf, desc. (figs 11–12, 35–43); Richman et al. 2011b: 37; Richman et al. 2012a: 37; Richman et al. 2012b: 37
Eremattus piraticus Peckham and Peckham, 1888; Banks 1910: 74
Icius piraticus (Peckham and Peckham, 1888); Marx 1890: 571; Peckham and Peckham 1888: 49
Pseudicius piraticus (Peckham and Peckham, 1888); Carpenter 1972: 166; Jackman 1997: 168; Peckham and Peckham 1909: 494; Petrunkevitch 1911: 698; Richman and Cutler 1978: 98 [T]; Vogel 1970b: 20

####### Distribution.

Archer, Hidalgo, Howard, Kimble, Lubbock, Travis, Wichita

####### Time of activity.

Male (September – October); female (June, October)

####### Habitat.

(plants: bush, grapecane in vineyard); (soil/woodland: mesquite, saltcedar, low trees)

####### Method.

Beating [m]

####### Type.

Texas (male, no locality, no date, no collector, MCZ)

####### Etymology.

Latin, pirate

####### Collection.

JCC, MSU

##### Genus *Paraphidippus* F. O. P.-Cambridge, 1901

###### Paraphidippus aurantius

Taxon classificationAnimaliaAraneaeSalticidae

(Lucas, 1833)

Paraphidippus aurantius Bradley 2013: 195; Edwards 2004: 5 [T]; Richman et al. 2011b: 37 [S]; Richman et al. 2012a: 37; Richman et al. 2012b: 37
Eris aurantia (Lucas, 1833); Agnew et al. 1985: 8; Bumroongsook et al. 1992: 17; Jackman 1997: 167; Nyffeler et al. 1992c: 3 [Kaston 1973: 118, mf (figs 55–57)]
Eris chrysis (Walckenaer, 1837); Brown 1974: 236

####### Distribution.

Brazos, Burleson, Cameron, Comanche, Erath, Nacogdoches

####### Locality.

Adriance Pecan Orchard, Proctor Lake

####### Time of activity.

Male (May – July); female (March, July – August)

####### Habitat.

(nest/prey: mud dauber nest [f]); (orchard: grapefruit, pecan); (plants: *Monarda citriodora*); (structures: in lab)

####### Method.

sweeping

####### Type.

Georgia

####### Etymology.

New Latin, orange

####### Collection.

TAMU

###### Paraphidippus fartilis

Taxon classificationAnimaliaAraneaeSalticidae

(Peckham & Peckham, 1888)

Paraphidippus fartilis Edwards 2004: 5 [T]; Richman et al. 2011b: 38 [S]; Richman et al. 2012a: 38; Richman et al. 2012b: 38
Eris fartilis (Peckham and Peckham, 1888); Jackman 1997: 167; Richman and Cutler 1978: 84; Vogel 1970b: 17
Parnaeus fartilis (Peckham and Peckham 1888); Peckham and Peckham 1909: 440, f, desc. (pl. 35, fig. 7)
Dendryphantes fartilis (Peckham and Peckham 1888); Petrunkevitch 1911: 629; Roewer 1955: 1202
Phidippus fartilis (Peckham and Peckham, 1888); Banks 1910: 63

####### Distribution.

Cameron

####### Type.

Mexico

[male unknown]

####### Etymology.

undetermined

##### Genus *Parnaenus* Peckham & Peckham, 1896

###### Parnaenus

Taxon classificationAnimaliaAraneaeSalticidae

sp.

Parnaenus G. B. Edwards, pers. comm. [undescribed]; Richman et al. 2011b: 40; Richman et al. 2012a: 40; Richman et al. 2012b: 40

####### Distribution.

Cameron, Hidalgo

####### Locality.

Laguna Atascosa National Wildlife Refuge, Lower Rio Grande Valley National Wildlife Refuge, Sabal Palm Audubon Sanctuary

####### Habitat.

(littoral: dense coastal brush); (orchard: organic grapefruit grove); (soil/woodland: ebony-guayacan association)

##### Genus *Peckhamia* Simon, 1901

###### Peckhamia americana

Taxon classificationAnimaliaAraneaeSalticidae

(Peckham & Peckham, 1892)

Peckhamia americana Agnew et al. 1985: 8; Calixto et al. 2013: 184; Cokendolpher 1978c: 118; Jackman 1997: 167; Matelski 1982: 1; Peckham and Peckham 1909: 368, mf, desc. (pl. 50, fig. 4, pl. 51, fig. 1); Petrunkevitch 1911: 678; Richman et al. 2011b: 40; Richman et al. 2012a: 40; Richman et al. 2012b: 40; Tugmon et al. 1990: 43
Consingis americanus (Peckham and Peckham, 1892); Brown 1974: 236

####### Distribution.

Angelina, Bandera, Brazos, Cameron, Comanche, Erath, Hood, Hunt, Kerr, Montague, Nacogdoches, Robertson, Travis, Tyler, Wichita

####### Locality.

Bill Haney Pecan Orchard, Davy Crockett National Forest, Holmes Pecan Orchard, Kirby State Forest, Laguna Atascosa National Wildlife Refuge, Lake Tawakoni State Park, Lick Creek Park, Lost Maples State Park, Pioneer Park, Sabal Palm Audubon Sanctuary

####### Time of activity.

Male (March – July, September); female (April – July, September – October)

####### Habitat.

(littoral: sedge meadow); (orchard: organic citrus grove, pecan); (soil/woodland: dense coastal brush, palm forest, woods, *Juniperus ashei*, *Quercus buckleyi*, *Quercus virginiana*, *Ulmus crassifolia*); (structures: in lab, on car)

####### Method.

Beating [mf]; cardboard band [mf]; flight intercept trap elevated [f]; flight intercept trap on ground [m]; Lindgren funnel trap [m]; sweeping [mf]

####### Type.

United States

####### Etymology.

locality (country)

####### Collection.

MSU, TAMU

###### Peckhamia picata

Taxon classificationAnimaliaAraneaeSalticidae

(Hentz, 1846)

Peckhamia picata Agnew et al. 1985: 5; Carpenter 1972: 167; Jackman 1997: 133, desc., 167 (photo 41i); Jones 1936: 69; Kaston 1972: 256, desc. (figs 576–577); Kaston 1978: 245, desc. (figs 623–624); Matelski 1982: 1; Richman and Cutler 1978: 91; Richman et al. 2011b: 40; Richman et al. 2012a: 40; Richman et al. 2012b: 40; Vogel 1970b: 18; Young and Edwards 1990: 22 [Peckham and Peckham 1909: 369, mf, desc. (pl. 51, figs 2–2c)]

####### Distribution.

Archer, Baylor, Cameron, Clay, Dallas, Erath, Montague, Wichita

####### Locality.

Lake Amon Carter, South Padre Island

####### Time of activity.

Male (January, April, June, September – October); female (April – June, September)

####### Habitat.

(crops: peanuts); (littoral: dune, grass marsh); (objects: under decaying burlap bag); (plants: flowering annuals); (soil/woodland: leaf litter, *Quercus* sp.)

####### Method.

sweeping

####### Type.

North Carolina and Alabama

####### Etymology.

Latin, variegated

####### Collection.

MSU, NMSU, TAMU

###### Peckhamia scorpionia

Taxon classificationAnimaliaAraneaeSalticidae

(Hentz, 1846)

Peckhamia scorpionia Bonnet 1958: 3441; Jones 1936: 69; Peckham and Peckham 1909: 370, mf, desc. (pl. 50, fig. 3, pl. 51, fig. 3); Richman et al. 2011b: 40; Richman et al. 2012a: 40; Richman et al. 2012b: 40; Vogel 1970b: 18 [Chickering 1944: 185, mf, desc. (figs 86–87)]
Peckhamia scorpionea (Hentz, 1846); Petrunkevitch 1911: 679

####### Distribution.

Baylor, Dallas

####### Time of activity.

Female (April)

####### Type.

North Carolina

####### Etymology.

Greek, referring to a scorpion

####### Collection.

DMNS

##### Genus *Pelegrina* Franganillo, 1930

###### Pelegrina arizonensis

Taxon classificationAnimaliaAraneaeSalticidae

(Peckham & Peckham, 1901)

Pelegrina arizonensis Jackman 1997: 167; Maddison 1996: 297 [T], mf, desc. (figs 160–161, 217, 251, 420–425); Richman et al. 2011b: 41; Richman et al. 2012a: 41; Richman et al. 2012b: 41
Metaphidippus arizonensis Peckham and Peckham, 1901; Milstead 1958: 446

####### Distribution.

Brewster, Terrell

####### Locality.

Black Gap Wildlife Management Area, Blackstone Ranch

####### Habitat.

(nest/prey: stomach of *Cnemidophorus perplexus*, stomach of *Cnemidophorus sacki*)

####### Type.

Arizona

####### Etymology.

locality (state)

###### Pelegrina chalceola

Taxon classificationAnimaliaAraneaeSalticidae

Maddison, 1996

Pelegrina chalceola Jackman 1997: 167; Maddison 1996: 291, mf, desc. (figs 139, 211, 383–387); Richman et al. 2011b: 42; Richman et al. 2012a: 42; Richman et al. 2012b: 42
Metaphidippus insignis (Banks, 1892); Agnew et al. 1985: 8 [misidentified]; Kagan 1942: 66 [misidentified]; Kagan 1943: 258 [misidentified]; Young and Edwards 1990: 22 [misidentified, erroneously listed under cotton]
Metaphidippus cf. insignis (Banks, 1892); Cokendolpher 1978c: 118 [misidentified]

####### Distribution.

Denton, Erath, Travis

####### Locality.

Lake Dallas

####### Time of activity.

Male (February – April, August)

####### Habitat.

(soil/woodland: juniper, *Ulmus crassifolia*)

####### Method.

suction trap [m]; sweeping [m]

####### Type.

Arizona, Santa Rita Mountains, Upper Madera Canyon

####### Etymology.

arbitrary combination of letters referring to color

####### Collection.

TAMU

###### Pelegrina exigua

Taxon classificationAnimaliaAraneaeSalticidae

(Banks, 1892)

Pelegrina exigua Bradley 2013: 196; Maddison 1996: 281 [T], mf, desc. (figs 127, 146–149, 203, 243, 329–337, 342); Richman et al. 2011b: 42; Richman et al. 2012a: 42; Richman et al. 2012b: 42
Metaphidippus exiguus (Banks, 1892); Breene et al. 1993c: 23, 48, 72, mf (figs 59A–D); Dean et al. 1982: 255; Young and Edwards 1990: 22
Pelegrina exiguus (Banks, 1892); Jackman 1997: 167

####### Distribution.

Brazos, San Augustine, Walker

####### Locality.

Ellis Prison Unit, Texas A&M University Rangeland Area

####### Time of activity.

Male (January, April)

####### Habitat.

(crops: cotton)

####### Method.

Boll weevil pheromone trap [m]; pitfall trap [m]

####### Type.

New York, Ithaca

####### Etymology.

Latin, small

####### Collection.

TAMU

###### Pelegrina flavipes

Taxon classificationAnimaliaAraneaeSalticidae

(Peckham & Peckham, 1888)

Pelegrina flavipes Paquin and Dupérré 2003: 198 [new name]
Pelegrina flavipedes Maddison, 1996 [Maddison 1996: 278 (figs 142–143, 201, 241, 319–323, 338–339)]

####### Distribution.

Archer, Bandera, Fannin, Wichita

####### Locality.

Lost Maples State Park

####### Time of activity.

Female (March)

####### Type.

Canada

####### Etymology.

Latin, yellow legs

####### Collection.

MSU, TAMU

###### Pelegrina furcata

Taxon classificationAnimaliaAraneaeSalticidae

(F. O. P.-Cambridge, 1901)

Pelegrina furcata Richman et al. 2011b: 43; Richman et al. 2012a: 43; Richman et al. 2012b: 43 [Maddison 1996: 292, mf, desc. (figs 158–159, 212, 249–250, 388–402)]

####### Distribution.

Texas

####### Type.

Mexico, Orizaba

####### Etymology.

Latin, forked, referring to male embolus

###### Pelegrina galathea

Taxon classificationAnimaliaAraneaeSalticidae

(Walckenaer, 1837)

Pelegrina galathea Armstrong and Richman 2007: 396; Bradley 2013: 196; Calixto et al. 2013: 184; Cokendolpher et al. 2008: 10, 49; Jackman 1997: 134, desc., 167; Knutson et al. 2010: 515; Maddison 1996: 263 [T], mf, desc. (figs 5, 11, 13, 35, 78, 125, 130–131, 190, 236, 258–263); Richman et al. 2011b: 43; Richman et al. 2012a: 43; Richman et al. 2012b: 43
Metaphidippus galathea (Walckenaer, 1837); Agnew et al. 1985: 5, 11; Breene 1988: 15, 17, 24–26, 35–36, 38–39; Breene et al. 1988: 180–181; Breene et al. 1989: 163; Breene et al. 1993a: 169; Breene et al. 1993b: 648; Breene et al. 1993c: 23, 48, 72, mf (figs 61A-C); Brown 1974: 236; Bumroongsook et al. 1992: 17; Carpenter 1972: 163; Dean and Eger 1986: 143; Dean and Sterling 1987: 7; Dean and Sterling 1990: 403, 405; Dean et al. 1982: 256; Dean et al. 1987: 264, 268; Dean et al. 1988: 287; Hunter 1988: 18–21; Li 1990: 137, 142, 144; Nyffeler et al. 1992a: 1181; Nyffeler et al. 1992b: 1459–1460; Nyffeler et al. 1992c: 3; Pamanes-Guerrero 1975: 42, 60, 63, 81; Rapp 1984: 9; Richman and Cutler 1978: 89; Rogers and Horner 1977: 523; Tugmon et al. 1990: 44; Young and Edwards 1990: 22

####### Distribution.

Eastern 2/3 Texas; Anderson, Aransas, Archer, Atascosa, Bee, Bexar, Bosque, Brazoria, Brazos, Brown, Burleson, Cameron, Carson, Collin, Colorado, Comanche, Concho, Coryell, Dallas, Delta, Denton, Dickens, Duval, Eastland, Ellis, Erath, Falls, Fannin, Fayette, Fort Bend, Franklin, Freestone, Frio, Galveston, Gillespie, Goliad, Grayson, Hamilton, Harris, Hidalgo, Hill, Houston, Howard, Hunt, Jim Wells, Karnes, Kaufman, Kerr, Kimble, Kleberg, Leon, Llano, Madison, Mason, Matagorda, McLennan, Milam, Mitchell, Montague, Motley, Nacogdoches, Nueces, Polk, Potter, Robertson, San Jacinto, San Patricio, San Saba, Scurry, Shackelford, Starr, Taylor, Titus, Tom Green, Travis, Uvalde, Val Verde, Victoria, Walker, Wharton, Wichita, Willacy, Williamson, Wood, Young

####### Locality.

Adriance Pecan Orchard, Attwater Prairie Chicken National Wildlife Refuge, Bill Haney Pecan Orchard, Ellis Prison Unit, Falcon Lake State Park, Frontera Audubon, Garner State Park, Gorman Falls, Hoblitzelle Farms, Holmes Pecan Orchard, Lacuna Park, Laguna Atascosa National Wildlife Refuge, Lake Meredith, Lake Tawakoni State Park, Lake Thomas, Lick Creek Park, Nash Prairie, Pantex Plant, Riley Estate, Russell Farm, Sam Houston National Forest, Santa Ana National Wildlife Refuge, Seminole Canyon State Park, Storey Pecan Orchard, Stubblefield Lake, Texas A&M University Rangeland Area, Welder Wildlife Refuge

####### Time of activity.

Male (January – December); female (January – December)

####### Habitat.

(crops: alfalfa, cotton, guar, peanuts, sugarcane); (grass: grass, grassland, grassy and shrub area, pasture); (littoral: playa edge, sedge meadow); (nest/prey: mud dauber nest [mf]); (orchard: citrus, grapefruit, orange, pecan); (plants: bluebonnets, croton, Indian paintbrush, lemon horsemint, miscellaneous vegetation, roadside vegetation, sage, thistle, vegetation, weed, *Baccharis*, *Centaurea* sp., *Engelmannia* sp., *Gaillardia* sp., *Monarda citriodora*, *Rudbeckia* sp.); (soil/woodland: brush, brushy area, chaparral, field, hackberry matte, Juniper, live oak forest, mesquite, post oak savanna, post oak savanna with pasture, saltcedar, sandy area, trees/shrubs, willow, woods, *Ulmus crassifolia*); (structures: around house, on sheet on clothes line)

####### Method.

Beating [mf]; boll weevil pheromone trap [mf]; cardboard band [mf]; D-Vac suction [mf]; fogging [mf]; irrigation tubing [mf]; pitfall trap [mf]; suction trap [mf]; sweeping [mf]

####### Eggs/spiderlings.

Walker [eggsac laid May 15, 1978, hatched June 1, 18 spiderlings] [TAMU]

####### Type.

North Carolina, Raleigh

####### Etymology.

Greek, the sea-nymph Galatea

####### Collection.

MSU, NMSU, TAMU, WTAM

###### Pelegrina peckhamorum

Taxon classificationAnimaliaAraneaeSalticidae

(Kaston, 1973)

Pelegrina peckhamorum Calixto et al. 2013: 184; Jackman 1997: 167; Maddison 1996: 272, mf, desc. (figs 126, 136–137, 195, 239, 288–293); Richman et al. 2011b: 45; Richman et al. 2012a: 45; Richman et al. 2012b: 45

####### Distribution.

Leon, Robertson, Val Verde

####### Locality.

Holmes Pecan Orchard

####### Time of activity.

Male (May); female (July)

####### Habitat.

(orchard: pecan); (soil/woodland: tree)

####### Method.

Beating [m]; cardboard band [f]

####### Type.

New Jersey, Lakehurst

####### Etymology.

Person (arachnologists)

####### Collection.

TAMU

###### Pelegrina pervaga

Taxon classificationAnimaliaAraneaeSalticidae

(Peckham & Peckham, 1909)

Pelegrina pervaga Jackman 1997: 167; Maddison 1996: 276 [T], mf, desc. (figs 199, 240, 309–313); Richman et al. 2011b: 45; Richman et al. 2012a: 45; Richman et al. 2012b: 45
Metaphidippus pervagus (Peckham and Peckham, 1909); Agnew et al. 1985: 8

####### Distribution.

Erath, Hidalgo, Kerr, Kimble, Travis, Val Verde

####### Locality.

Bentsen-Rio Grande Valley State Park

####### Time of activity.

Male (February – August, October); female (February – July, October)

####### Habitat.

(plants: miscellaneous vegetation); (soil/woodland: juniper, trees, *Juniperus ashei*, *Quercus buckleyi*)

####### Method.

Beating [mf]; sweeping [mf]

####### Type.

Kansas

####### Etymology.

Latin, wandering through

####### Collection.

MSU, TAMU

###### Pelegrina proterva

Taxon classificationAnimaliaAraneaeSalticidae

(Walckenaer, 1837)

Pelegrina proterva Cokendolpher et al. 2008: 10, 69; Jackman 1997: 167; Maddison 1996: 270 [S, T], mf, desc. (figs 2–3, 6–9, 15, 34, 134–135, 194, 238, 282–287); Richman et al. 2011b: 45; Richman et al. 2012a: 45; Richman et al. 2012b: 45
Metaphidippus protervus (Walckenaer, 1837); Brown 1974: 236; Richman and Cutler 1978: 90
Dendryphantes capitatus (Hentz, 1845); Jones 1936: 69
Metaphidippus capitatus (Hentz, 1845); Bonnet 1957: 2810; Vogel 1970b: 18
Dendryphantes octavus (Hentz, 1846); Jones 1936: 70

####### Distribution.

Anderson, Brewster, Carson, Dallas, Denton, Hardin, Nacogdoches, Sabine, San Jacinto, Wichita

####### Habitat.

(grass: grassland); (nest/prey: mud dauber nest [f])

####### Type.

unknown

####### Etymology.

Latin, violent

####### Collection.

MSU

###### Pelegrina sabinema

Taxon classificationAnimaliaAraneaeSalticidae

Maddison, 1996

Pelegrina sabinema Jackman 1997: 167; Maddison 1996: 275, mf, desc. (figs 198, 304–308); Richman et al. 2011b: 46; Richman et al. 2012a: 46; Richman et al. 2012b: 46

####### Distribution.

Gonzales, Hays, Jeff Davis, Real, Scurry

####### Locality.

Lake Thomas, Palmetto State Park

####### Time of activity.

Male (May, December 16-January 26); female (May – June)

####### Habitat.

(plants: roadside vegetation); (soil/woodland: *Juniperus* unmanaged plot, willow)

####### Method.

Flight intercept trap elevated [m]; sweeping [mf]

####### Type.

Arizona, Showlow

####### Etymology.

arbitrary combination of letters

####### Collection.

TAMU

###### Pelegrina tillandsiae

Taxon classificationAnimaliaAraneaeSalticidae

(Kaston, 1973)

Pelegrina tillandsiae Jackman 1997: 167; Maddison 1996: 305, mf, desc. (figs 225, 254, 472–477); Richman et al. 2011b: 46; Richman et al. 2012a: 46; Richman et al. 2012b: 46

####### Distribution.

Harris

####### Time of activity.

Female (December)

####### Type.

North Carolina, Polluckville

####### Etymology.

habitat (Preferred habitat appears to be Spanish moss *Tillandsia*, Kaston 1973).

##### Genus *Pellenes* Simon, 1876

###### Pellenes limatus

Taxon classificationAnimaliaAraneaeSalticidae

Peckham & Peckham, 1901

Pellenes limatus Agnew et al. 1985: 5, 11; Banks 1910: 68; Broussard and Horner 2006: 255; Brown 1974: 236; Cokendolpher 1978c: 118; Cokendolpher et al. 2008: 10, 49; Jackman 1997: 167; Peckham and Peckham 1909: 561, mf, desc. (pl. 48, figs 2–2a); Petrunkevitch 1911: 684; Richman and Cutler 1978: 92 [S]; Richman et al. 2011a: 50; Richman et al. 2011b: 48; Richman et al. 2012a: 48; Richman et al. 2012b: 48; Roewer 1955: 1135; Vogel 1970b: 19; Young and Edwards 1990: 22
Pellenes townsendii Peckham and Peckham, 1901; Peckham and Peckham 1901b: 218, f, desc. (pl. 1, fig. 9)

####### Distribution.

Blanco, Brazos, Brewster, Burleson, Carson, Comanche, Coryell, Erath, Floyd, Nacogdoches, Presidio, Wichita

####### Locality.

Browning Ranch, Chihuahuan desert, Dalquest Research Site

####### Time of activity.

Male (April – October); female (June – September)

####### Habitat.

(crops: peanuts); (littoral: near playa); (nest/prey: mud dauber nest [f]); (soil/woodland: on ground, post oak savanna with pasture)

####### Method.

pitfall trap [mf]; suction trap [m]; sweeping

####### Type.

California

####### Etymology.

Latin, polished

####### Collection.

MSU, TAMU

###### Pellenes longimanus

Taxon classificationAnimaliaAraneaeSalticidae

Emerton, 1913

Pellenes longimanus Hedin and Maddison 2001b: 1514 [Kaston 1948: 462, mf, desc. (figs 1695–1697)]

####### Distribution.

Hidalgo

####### Locality.

Bentsen-Rio Grande Valley State Park

####### Type.

New Jersey

####### Etymology.

Latin, long-handed

##### Genus *Phidippus* C. L. Koch, 1846

###### Phidippus apacheanus

Taxon classificationAnimaliaAraneaeSalticidae

Chamberlin & Gertsch, 1929

Phidippus apacheanus Agnew et al. 1985: 5; Bradley 2013: 197; Carpenter 1972: 162; Cokendolpher et al. 2008: 10, 49 (photo 33); Edwards 2004: 89, mf, desc. (figs C55–56, 309–313); Hunter 1988: 18, 20; Jackman 1997: 136, 168; Knutson et al. 2010: 515; Richman et al. 2011b: 50; Richman et al. 2012a: 50; Richman et al. 2012b: 50; Roberts 2001: 51; Young and Edwards 1990: 22

####### Distribution.

Archer, Bexar, Brazos, Brown, Carson, Clay, Coryell, Crockett, Dallas, Donley, Edwards, Ellis, Erath, Floyd, Frio, Gaines, Gray, Gregg, Hardeman, Harris, Howard, Lubbock, Montague, Ochiltree, Potter, Randall, San Jacinto, Smith, Sutton, Tarrant, Taylor, Travis, Uvalde, Wichita

####### Locality.

Medicine Mounds Ranch, Pantex Plant, Wildcat Bluff Nature Center

####### Time of activity.

Male (September – October); female (March – April, September – December)

####### Habitat.

(crops: peanuts, sunflower); (grass: grass, grassland); (landscape features: under rock); (littoral: playa); (plants: bush, cactus, prickly pear, vegetation, weeds); (soil/woodland: mesquite bush, saltcedar, shrubs, trees)

####### Method.

Beating [f]; pitfall trap; sweeping

####### Type.

Utah, Black Rock

####### Etymology.

Latin, “of the Apache”

####### Collection.

JCC, MSU, TAMU, WTAM

###### Phidippus arizonensis

Taxon classificationAnimaliaAraneaeSalticidae

(Peckham & Peckham, 1883)

Phidippus arizonensis F. O. P.-Cambridge 1901: 284; Chamberlin 1924b: 681; Edwards 2004: 40 [S], mf, desc. (figs C11–12, 82–88); Jackman 1997: 168; Marx 1890: 568; Peckham and Peckham 1888: 18; Peckham and Peckham 1909: 419; Richman and Cutler 1978: 95; Richman et al. 2011b: 51; Richman et al. 2012a: 51; Richman et al. 2012b: 51; Vogel 1970b: 19
Phidippus obscurus Peckham and Peckham, 1888; Peckham and Peckham 1888: 16; Peckham and Peckham 1901a: 294; Peckham and Peckham 1909: 431; Richman and Cutler 1978: 96; Vogel 1970b: 19
Dendryphantes obscurus (Peckham and Peckham, 1888); Petrunkevitch 1911: 638; Roewer 1955: 1213
Dendryphantes tuberculatus (F. O. P.-Cambridge, 1901); Petrunkevitch 1911: 643; Roewer 1955: 1204

####### Distribution.

Atascosa, Bexar, Brewster, Cameron, Coryell, Dallas, Frio, Hays, Hidalgo, Jeff Davis, Jim Wells, Karnes, Kleberg, Nueces, Presidio, Refugio, San Patricio, Travis, Uvalde, Wichita, Williamson

####### Locality.

Anzalduas County Park, Bentsen-Rio Grande Valley State Park, Big Bend Ranch State Park, Estero Llano Grande State Park, Ramsey Nature Park, Resaca de la Palma State Park, Santa Ana National Wildlife Refuge

####### Time of activity.

Male (October – November); female (July, October)

####### Method.

Beating [m]; light trap

####### Eggs/spiderlings.

Hidalgo [eggsac collected April 15, 1980; hatched May 3, 9 spiderlings, reared male and female July 1981] [TAMU]

####### Type.

Texas (not listed, Hidalgo Co., Santa Ana National Wildlife Refuge, no date, no collector)

####### Etymology.

locality (Latin adjective derived from geographical name, the state of Arizona, Peckham and Peckham 1883).

####### Collection.

MSU, TAMU, WTAM

###### Phidippus asotus

Taxon classificationAnimaliaAraneaeSalticidae

Chamberlin & Ivie, 1933

Phidippus asotus Edwards 2004: 37, mf, desc. (figs C9–10, 65–70); Richman et al. 2011b: 51; Richman et al. 2012a: 51; Richman et al. 2012b: 51

####### Distribution.

Jeff Davis

####### Type.

Utah, Lynn, Grouse Creek

####### Etymology.

Latin, a sensualist, libertine, debaucher

###### Phidippus audax

Taxon classificationAnimaliaAraneaeSalticidae

(Hentz, 1845)

Phidippus audax Agnew et al. 1982: 631; Agnew et al. 1985: 5, 11; Armstrong and Richman 2007: 396; Bonnet 1958: 3513; Breene 1988: 15, 17, 23–26, 35–36, 38–40, 44, 49; Breene et al. 1988: 180–181; Breene et al. 1989: 163; Breene et al. 1993a: 169, Breene et al. 1993b: 648; Breene et al. 1993c: 23, 24, 48, 70, mf (figs 56A-C); Brown 1974: 236; Bumroongsook et al. 1992: 17; Calixto et al. 2013: 184, 190; Cokendolpher 1978c: 118; Comstock 1912: 681, desc.; Comstock 1940: 689; Dean and Eger 1986: 143; Dean and Sterling 1987: 7; Dean and Sterling 1990: 405; Dean et al. 1982: 256; Dean et al. 1987: 264, 268; Dean et al. 1988: 287; Edwards 2004: 72 [S], mf, desc. (figs C32–36, 237–243); Guarisco 2008b: 5; Hunter 1988: 18–21; Jackman 1997: 135, desc., 168 (photo 41l); Jackman et al. 2007: 199; Jones 1936: 70; Knutson et al. 2010: 515; Li 1990: 137, 142, 144; Liao et al. 1984: 411; Lombardini et al. 2005: 1378; McDaniel et al. 1981: 104; Nuessly and Sterling 1984: 97; Nyffeler et al. 1987a: 357; Nyffeler et al. 1987b: 1121; Nyffeler et al. 1988a: 55; Nyffeler et al. 1990b: 496; Nyffeler et al. 1992a: 1181; Nyffeler et al. 1992b: 1459–1460; Nyffeler et al. 1992c: 3; Rapp 1984: 9; Rice 1986: 124; Richman and Cutler 1978: 95; Richman et al. 2011b: 51; Richman et al. 2012a: 51; Richman et al. 2012b: 51; Roberts 2001: 51; Rogers and Horner 1977: 523; Sterling et al. 1979: 979; Taber and Fleenor 2003: 238; Taylor and Peck 1975: 90; Tugmon et al. 1990: 43–44; Vincent and Frankie 1985: 380; Vogel 1970b: 19; Woods and Harrel 1976: 44; Yantis 2005: 201; Young and Edwards 1990: 22
Phidippus variegatus (Lucas, 1833); Carpenter 1972: 163; Kagan 1942: 61; Kagan 1943: 258; Peckham and Peckham 1909: 390; Vogel 1970b: 20
Dendryphantes variegatus (Lucas, 1833); Petrunkevitch 1911: 643
Phidippus rauterbergii Peckham & Peckham, 1888; Peckham and Peckham 1888: 22; Peckham and Peckham 1901a: 295; Peckham and Peckham 1909: 429
Phidippus rauterbergi Peckham & Peckham, 1888; Banks 1910: 65; Richman and Cutler 1978: 97; Vogel 1970b: 19
Dendryphantes rautenbergi (Peckham & Peckham, 1888); Petrunkevitch 1911: 641
Dendryphantes rautbergii (Peckham & Peckham, 1888); Roewer 1955: 1204
Philaeus farneus Peckham & Peckham, 1888; Peckham and Peckham 1888: 26
Dendryphantes farneus (Peckham & Peckham, 1888); Marx 1890: 570; Petrunkevitch 1911: 629; Roewer 1955: 1210
Phidippus farneus (Peckham & Peckham, 1888); Banks 1910: 63; Peckham and Peckham 1909: 430; Richman and Cutler 1978: 96; Vogel 1970b: 19

####### Distribution.

Aransas, Archer, Atascosa, Austin, Bailey, Bastrop, Baylor, Bell, Bexar, Bosque, Brazoria, Brazos, Brooks, Brown, Burleson, Burnet, Calhoun, Cameron, Carson, Chambers, Cherokee, Clay, Collin, Colorado, Comanche, Coryell, Dalham, Dallas, Delta, Denton, Dickens, Ellis, Erath, Floyd, Fort Bend, Gaines, Galveston, Goliad, Gonzales, Gray, Grayson, Gregg, Grimes (imm.), Hansford, Hardeman, Harris, Harrison, Hidalgo, Hill, Houston, Howard, Hunt, Hutchinson, Jasper, Jeff Davis, Jefferson, Jim Hogg, Jim Wells, Karnes, Kaufman, Kenedy, Kerr, Kleberg, Lamb, Lee, Leon, Liberty, Lubbock, Lynn, Madison, Mason, McLennan, Medina, Menard, Mitchell, Montgomery, Nacogdoches, Nueces, Orange, Potter, Randall, Robertson, Runnels, Rusk, San Patricio, Tarrant, Taylor, Travis, Uvalde, Val Verde, Victoria, Walker, Washington, Wharton, Wheeler, Wichita, Wilbarger, Willacy, Williamson, Wilson, Zavala

####### Locality.

Adriance Pecan Orchard, Bentsen-Rio Grande Valley State Park, Ellis Prison Unit, Estero Llano Grande State Park, Galveston Island State Park, Holmes Pecan Orchard, Lake Corpus Christi State Park, Lake Creek, Lake Meredith National Recreation Area, Lake Somerville State Park [Nails Creek Unit], Lake Tawakoni State Park, Medicine Mounds Ranch, Nance Ranch, Palmetto State Park, Ramsey Nature Park, Ramsey Prison Farm, Resaca de la Palma State Park, Riley Estate, Rita Blanca Lake, Robert J. Baker Ranch, Russell Farm, Santa Ana National Wildlife Refuge, Stiles Ranch, Storey Pecan Orchard, Welder Wildlife Refuge, Wildcat Bluff Nature Center

####### Time of activity.

Male (January, March – December); female (February – December)

####### Habitat.

(crops: cotton, guar, peanuts, rice, soybean, sugarcane); (grass: grass, grassy and shrub area, grassland, pasture); (landscape features: bridge, culvert, overpass); (littoral: salt marsh area); (nest/prey: mud dauber nest [imm.], retreat under picnic table); (orchard: citrus, grapefruit, Mexican lime, orange tree, pecan); (plants: bluebonnets, croton, Indian paintbrush, in bush, miscellaneous vegetation, on plants, weed, *Monarda citriodora*); (soil/woodland: under Juniper logs, live oak, post oak savanna with pasture, post oak woods [%: 74, 96], saltcedar, sandy brushland, sandy open prairie, willow, woodland); (structures: garage, in house, on wall, retreat under picnic table, under house eave); (web: large spider web)

####### Method.

5 gallon bucket trap [f]; beating [m]; boll weevil pheromone trap [m]; cardboard band [mf]; fogging [mf]; irrigation tubing [imm.]; moth pheromone trap [m]; pitfall trap [mf]; suction trap [mf]; sweeping [mf]

####### Type.

Massachusetts

####### Etymology.

Latin, audacious, bold

####### Collection.

DMNS, JCC, MSU, NMSU, TAMU, TTU, WTAM

###### Phidippus bidentatus

Taxon classificationAnimaliaAraneaeSalticidae

F. O. P.-Cambridge, 1901

Phidippus bidentatus Edwards and Richman 2005: 138; Richman et al. 2011b: 52; Richman et al. 2012a: 52; Richman et al. 2012b: 52 [Edwards 2004: 71, mf, desc. (figs 232–236)]

####### Distribution.

Cameron

####### Locality.

Russell Farm

####### Time of activity.

Male (March)

####### Method.

Boll weevil pheromone trap [m]

####### Type.

Mexico, Chiapas

####### Etymology.

Latin, having two teeth

###### Phidippus californicus

Taxon classificationAnimaliaAraneaeSalticidae

Peckham & Peckham, 1901

Phidippus californicus Edwards 2004: 57, mf, desc. (figs 162–170); Richman et al. 2011b: 53; Richman et al. 2012a: 53; Richman et al. 2012b: 53

####### Distribution.

Brewster, Loving, Webb

####### Type.

California

####### Etymology.

locality (Latin adjective derived from geographic name, the state of CA, Peckham and Peckham 1901a).

###### Phidippus cardinalis

Taxon classificationAnimaliaAraneaeSalticidae

(Hentz, 1845)

Phidippus cardinalis Agnew et al. 1985: 5; Bradley 2013: 198; Breene et al. 1993c: 24, 48, 70, mf (figs 55A-C); Brown 1974: 237; Cokendolpher 1978c: 118; Dean et al. 1982: 256; Edwards 2004: 64 [T], mf, desc. (figs C47–48, 197–202); Jackman 1997: 136, desc., 168; Kaston 1978: 257, desc.; Peckham and Peckham 1909: 393; Richman and Cutler 1978: 95; Richman et al. 2011b: 53; Richman et al. 2012a: 53; Richman et al. 2012b: 53; Rogers and Horner 1977: 523; Vogel 1970b: 19; Young and Edwards 1990: 22
Dendryphantes cardinalis (Hentz, 1845); Petrunkevitch 1911: 625
Phidippus mccooki (Peckham and Peckham, 1883); Hunter 1988: 18–20 [Texas record]

####### Distribution.

Bexar, Brazos, Brown, Burleson, Comanche, Coryell, Denton, Ellis, Erath, Freestone, Grayson, Hamilton, Hardeman, Hardin, Hidalgo, Johnson, Kerr, Kleberg, Knox, Montague, Nacogdoches, Runnels, Smith, Travis, Walker, Waller, Wheeler, Wichita, Zavala

####### Locality.

Ellis Prison Unit, Lick Creek Park, Medicine Mounds Ranch, Riley Estate

####### Time of activity.

Male (April – November); female (March – June, August – November)

####### Habitat.

(crops: cotton, guar, peanuts); (grass: low grass, sandy grassland); (plants: mixed vegetation); (soil/woodland: ground in woods, post oak savanna with pasture, sandy area); (structures: on fence)

####### Method.

pitfall trap [mf]; sweeping [m]

####### Type.

southern United States

####### Etymology.

Latin, dorsal color (cardinal red)

####### Collection.

DMNS, MSU, TAMU, WTAM

###### Phidippus carneus

Taxon classificationAnimaliaAraneaeSalticidae

Peckham & Peckham, 1896

Phidippus carneus Edwards 2004: 48, mf, desc. (figs C20, 120–126); Jackman 1997: 168; Richman et al. 2011b: 54; Richman et al. 2012a: 54; Richman et al. 2012b: 54
Phidippus johnsoni (Peckham and Peckham, 1883); Richman and Cutler 1978: 96; Taber and Fleenor 2003: 238 [Texas record]
Phidippus formosus Peckham and Peckham, 1883; Carpenter 1972: 162 [Texas record]

####### Distribution.

Archer, Brewster, Culberson, El Paso, Presidio, Wichita

####### Locality.

Big Bend Ranch State Park, Franklin Mountains

####### Time of activity.

Male (September – October); female (January, March, September – October)

####### Habitat.

(plants: agave, vegetation); (soil/woodland: mesquite woodland)

####### Method.

Beating [m]

####### Type.

Central America

####### Etymology.

Latin, of the flesh, carnal

####### Collection.

MSU, NMSU, TAMU

###### Phidippus carolinensis

Taxon classificationAnimaliaAraneaeSalticidae

Peckham & Peckham, 1909

Phidippus carolinensis Agnew et al. 1985: 8; Carpenter 1972: 162; Edwards 2004: 32 [T], mf, desc. (figs C5–6, 36–42); Hunter 1988: 18–19, 21; Jackman 1997: 168; Knutson et al. 2010: 515; Peckham and Peckham 1909: 422; Richman and Cutler 1978: 95; Richman et al. 2011b: 54; Richman et al. 2012a: 54; Richman et al. 2012b: 54; Roberts 2001: 51; Vogel 1970b: 19
Dendryphantes carolinensis (Peckham and Peckham, 1909); Petrunkevitch 1911: 626; Roewer 1955: 1207

####### Distribution.

Bell, Bexar, Cameron, Cherokee, Clay, Comanche, Coryell, Dallas, Dickens, Eastland, Ellis, Erath, Frio, Gillespie, Haskell, Hidalgo, Howard, Kerr, Kimble, McLennan, Montague, Nolan, Nueces, Parker, Potter, Randall, Roberts, Runnels, Sutton, Tarrant, Taylor, Travis, Wichita [Weatherford is a city in Parker Co.]

####### Locality.

Lake Meredith, Nabor’s Lake, Wildcat Bluff Nature Center

####### Time of activity.

Male (June – September); female (April – June, August – October)

####### Habitat.

(grass: grassland); (landscape features: under rock); (plants: roadside vegetation, vegetation); (soil/woodland: mesquite bush, saltcedar, under bark, wild plum thicket, willow, woodland, woods); (structures: retreat under picnic table, window)

####### Method.

Black light trap [m]; sweeping [mf]

####### Type.

Texas (male, female, Erath Co., Stephenville [North Carolina, type mislabeled])

####### Etymology.

locality (Latin adjective from geographic name, the state of NC, Peckham and Peckham 1909).

####### Collection.

MSU, NMSU, TAMU, WTAM

###### Phidippus clarus

Taxon classificationAnimaliaAraneaeSalticidae

Keyserling, 1885

Phidippus clarus Agnew et al. 1985: 5; Breene et al. 1993c: 24, 48, 71, mf (figs 58A-C); Brown 1974: 237; Cokendolpher et al. 2008: 10, 50; Dean et al. 1982: 256; Edwards 2004: 60, mf, desc. (figs C45–46, 184–190); Hunter 1988: 18–20; Jackman 1997: 168; Nyffeler et al. 1992c: 3; Peckham and Peckham 1909: 398; Richman et al. 2011b: 54; Richman et al. 2012a: 54; Richman et al. 2012b: 54; Taber and Fleenor 2003: 239; Young and Edwards 1990: 22
Phidippus rimator (Walckenaer, 1837); Vogel 1970b: 19 [Texas record]
Phidippus coloradensis Thorell, 1877; Peckham and Peckham 1909: 399; Vogel 1970b: 19 [Texas record]

####### Distribution.

Anderson, Brazos, Burleson, Carson, Coryell, Dallas, Denton, Ellis, Erath, Falls, Grayson, Hidalgo, Hood, Hopkins, Houston, Montgomery, Nacogdoches, Randall, Tyler, Walker, Wichita

####### Locality.

Anzalduas County Park, Ellis Prison Unit, Lick Creek Park, Riley Estate, Texas A&M University Rangeland Area

####### Time of activity.

Male (April, June – September); female (May – November)

####### Habitat.

(crops: cotton, peanuts); (grass: grassland, pasture); (littoral: playa); (nest/prey: mud dauber nest [imm.]); (plants: bluebonnets, miscellaneous vegetation, mixed vegetation, weeds, *Monarda citriodora*); (soil/woodland: post oak savanna, post oak savanna with pasture)

####### Method.

Boll weevil pheromone trap [f]; pitfall trap [mf]; sweeping [mf]

####### Type.

Maryland

####### Etymology.

Latin, clear, evident

####### Collection.

MSU, TAMU, WTAM

###### Phidippus comatus

Taxon classificationAnimaliaAraneaeSalticidae

Peckham & Peckham, 1901

Phidippus comatus Cokendolpher 1978c: 118; Edwards 2004: 31, mf, desc. (figs C7, 29–35); Jackman 1997: 168; Richman and Cutler 1978: 96; Richman et al. 2011b: 55; Richman et al. 2012a: 55; Richman et al. 2012b: 55

####### Distribution.

Burleson, El Paso, Jeff Davis

####### Locality.

Franklin Mountains

####### Time of activity.

Male (July); female (May)

####### Habitat.

(soil/woodland: post oak savanna with pasture)

####### Method.

pitfall trap [m]

####### Type.

New Mexico, Las Vegas

####### Etymology.

Latin, hairy

####### Collection.

NMSU, TAMU

###### Phidippus mystaceus

Taxon classificationAnimaliaAraneaeSalticidae

(Hentz, 1846)

Phidippus mystaceus Agnew et al. 1985: 8; Carpenter 1972: 162; Comstock 1912: 684, desc.; Comstock 1940: 692; Edwards 2004: 42 [S, T], mf, desc. (figs C14–16, 89–94); Jackman 1997: 168; Peckham and Peckham 1909: 435; Richman and Cutler 1978: 96; Richman et al. 2011b: 57; Richman et al. 2012a: 57; Richman et al. 2012b: 57; Vogel 1970b: 19
Dendryphantes mystaceus (Hentz, 1845); Petrunkevitch 1911: 637; Roewer 1955: 1213
Phidippus incertus Peckham and Peckham, 1901; Bryant 1942: 698; Emerton 1909: 224; Vogel 1970b: 19

####### Distribution.

Anderson, Archer, Bastrop, Bexar, Brazos, Brown, Burnet, Clay, Colorado, Comanche, Coryell, Dallas, Denton, Erath, Frio, Grayson, Hardeman, Jones, Kerr, Kimble, Lampasas, Llano, McLennan, Motley, Potter, Sutton, Taylor, Travis, Wichita

####### Locality.

Medicine Mounds Ranch

####### Time of activity.

Male (October – November); female (March, September – November)

####### Habitat.

(crops: sunflower); (landscape features: under rock); (objects: on stake in field); (orchard: orange tree); (soil/woodland: mesquite bush, next to croton field, saltcedar shrub, trees, wild plum thicket, *Ulmus crassifolia*); (structures: side of building)

####### Method.

Beating [mf]; suction trap [m]

####### Type.

North Carolina

####### Etymology.

Latin, with moustache

####### Collection.

MSU, TAMU, WTAM

###### Phidippus octopunctatus

Taxon classificationAnimaliaAraneaeSalticidae

(Peckham & Peckham, 1883)

Phidippus octopunctatus Edwards 2004: 26, mf, desc. (figs C1, 7–11); Knutson et al. 2010: 515; Richman et al. 2011b: 58; Richman et al. 2012a: 58; Richman et al. 2012b: 58

####### Distribution.

Brewster, Howard, Jeff Davis, Montgomery

####### Habitat.

(soil/woodland: saltcedar)

####### Type.

Missouri

####### Etymology.

Latin, 8-spotted

####### Collection.

WTAM

###### Phidippus otiosus

Taxon classificationAnimaliaAraneaeSalticidae

(Hentz, 1846)

Phidippus otiosus Bradley 2013: 200; Edwards 2004: 55, mf, desc. (figs C30–31, 154–161); Jackman 1997: 168; Kaston 1972: 269, desc. (fig. 608); Kaston 1978: 257, desc. (fig. 657); Richman et al. 2011b: 58; Richman et al. 2012a: 58; Richman et al. 2012b: 58

####### Distribution.

Colorado, Montague, Newton, Panola, Rusk

####### Time of activity.

Female (March)

####### Type.

North Alabama

####### Etymology.

Latin, free, at leisure

####### Collection.

MSU

###### Phidippus phoenix

Taxon classificationAnimaliaAraneaeSalticidae

Edwards, 2004

Phidippus phoenix Edwards 2004: 51, mf, desc. (figs C19, 137–142)

####### Distribution.

Kerr

####### Locality.

Raven Ranch

####### Time of activity.

Female (June)

####### Type.

Arizona, S Wickenberg, Vulture Mountains

####### Etymology.

Latin, Greek mythology, bird arose from its own ashes

###### Phidippus pius

Taxon classificationAnimaliaAraneaeSalticidae

Scheffer, 1905

Phidippus pius Agnew et al. 1985: 5; Carpenter 1972: 162; Cokendolpher et al. 2008: 10, 50 (photo 34); Dean and Eger 1986: 143; Dean et al. 1988: 287; Edwards 2004: 58, mf, desc. (figs C43–44, 174–178); Jackman 1997: 168; Knutson et al. 2010: 515; Nyffeler et al. 1992c: 3; Richman et al. 2011b: 59; Richman et al. 2012a: 58; Richman et al. 2012b: 58; Young and Edwards 1990: 22

####### Distribution.

Archer, Brazos, Burleson (imm.), Carson, Comal, Erath (imm.), Fannin, Grayson, Grimes, Houston, Howard, Kleberg, Montague, Randall, Sutton, Travis (imm.), Uvalde (imm.), Webb, Wichita

####### Locality.

Garner State Park, Nance Ranch, Pantex Plant

####### Time of activity.

Male (April, July); female (September)

####### Habitat.

(crops: peanuts); (grass: grass, pasture); (littoral: near playa); (plants: bluebonnets, Indian paintbrush, *Monarda citriodora*); (soil/woodland: bush, saltcedar, *Quercus virginianus*, *Ulmus crassifolia*)

####### Method.

pitfall trap; sweeping [m]

####### Type.

Kansas, Manhattan

####### Etymology.

Latin, dutiful, holy, godly, devoted

####### Collection.

MSU, NMSU, TAMU, WTAM

###### Phidippus princeps

Taxon classificationAnimaliaAraneaeSalticidae

(Peckham & Peckham, 1883)

Phidippus princeps Bradley 2013: 200; Cokendolpher 1978c: 118; Edwards 2004: 69, mf, desc. (figs C37–38, 221–226); Jackman 1997: 168; Richman and Cutler 1978: 97; Richman et al. 2011b: 59; Richman et al. 2012a: 59; Richman et al. 2012b: 59

####### Distribution.

Wichita

####### Time of activity.

Male (May)

####### Habitat.

(structures: fence)

####### Type.

Pennsylvania

####### Etymology.

Latin, foremost

####### Collection.

MSU

###### Phidippus pruinosus

Taxon classificationAnimaliaAraneaeSalticidae

Peckham & Peckham, 1909

Phidippus pruinosus Banks 1910: 65; Edwards 2004: 36 [T], mf, desc. (figs C8, 59–64); Jackman 1997: 168; Jones 1936: 70; Peckham and Peckham 1909: 433; Richman and Cutler 1978: 97; Richman et al. 2011b: 59; Richman et al. 2012a: 59; Richman et al. 2012b: 59; Vogel 1970b: 19
Dendryphantes pruinosus (Peckham and Peckham, 1909); Petrunkevitch 1911: 640; Roewer 1955: 1215

####### Distribution.

Dallas, Johnson, Llano, Taylor, Travis

####### Locality.

Cleburne Lake, Lake Abilene

####### Time of activity.

Male (July, December); female (March, July, November – December)

####### Habitat.

(soil/woodland: mountain cedar)

####### Type.

Texas (female, Travis Co., Austin, no date, no collector, MCZ)

####### Etymology.

Latin, full of hoarfrost (dorsal cover of gray setae)

####### Collection.

MSU

###### Phidippus putnami

Taxon classificationAnimaliaAraneaeSalticidae

(Peckham & Peckham, 1883)

Phidippus putnami Calixto et al. 2013: 184; Edwards 2004: 28, mf, desc. (figs C2, 17–21); Jackman 1997: 168; Richman et al. 2011b: 60; Richman et al. 2012a: 60; Richman et al. 2012b: 60

####### Distribution.

Brazos, Burleson, Comanche, Denton, Grayson, Lubbock, Robertson, Tarrant, Wichita

####### Locality.

Bill Haney Pecan Orchard, Holmes Pecan Orchard, Storey Pecan Orchard

####### Time of activity.

Male (July – August); female (July – August, October)

####### Habitat.

(orchard: pecan)

####### Method.

cardboard band [f]; fogging [mf]

####### Type.

Iowa

####### Etymology.

Person (contributor)

####### Collection.

JCC, MSU, TAMU

###### Phidippus texanus

Taxon classificationAnimaliaAraneaeSalticidae

Banks, 1906

Phidippus texanus Agnew et al. 1985: 5; Banks 1906: 98, f, desc.; Banks 1910: 65; Breene et al. 1993c: 24, 48, 71, mf (figs 57A-C); Carpenter 1972: 162; Cokendolpher et al. 2008: 10, 50 (fig. 12, photo 35); Dean and Eger 1986: 143; Dean and Sterling 1987: 7; Edwards 2004: 93 [S, T], mf, desc. (figs C59, 328–333); Hunter 1988: 18–20; Jackman 1997: 168; Kagan 1942: 62; Kagan 1943: 258; Kaston 1972: 268, desc. (fig. 606); Kaston 1978: 256, desc. (fig. 655); Knutson et al. 2010: 515; Peckham and Peckham 1909: 437; Reddell 1970: 407; Richman and Cutler 1978: 97; Richman et al. 2011b: 61; Richman et al. 2012a: 61; Richman et al. 2012b: 61; Roberts 2001: 51; Tugmon et al. 1990: 44; Vogel 1970b: 19; Young and Edwards 1990: 22
Dendryphantes texanus (Banks, 1906); Petrunkevitch 1911: 642; Roewer 1955: 1216
Phidippus peritus Gertsch, 1934; Bonnet 1958: 3525; Gertsch 1934d: 14, m, desc. (fig. 18); Vogel 1970b: 19
Dendryphantes peritus (Gertsch, 1934); Roewer 1955: 1214

####### Distribution.

Archer, Atascosa, Austin, Bandera, Bastrop, Baylor, Bexar, Borden, Brazos, Brown, Burnet, Cameron, Carson, Clay, Coleman, Comanche, Coryell, Cottle, Crockett, Crosby, Dallas, DeWitt, Denton, Dickens, Duval, Eastland, Ector, Ellis, Erath, Foard, Garza, Gillespie, Grayson, Hall, Hemphill, Hidalgo, Hood, Howard, Jim Wells, Jones, Kerr, King, Kleberg, La Salle, Lampasas, Lipscomb, Live Oak, McLennan, Midland, Montague, Nolan, Nueces, Parker, Pecos, Potter, Randall, Reagan, Starr, Tarrant, Terrell, Tom Green, Travis, Val Verde, Webb, Wharton, Wheeler, Wichita, Williamson, Young

####### Locality.

Bill Haney Pecan Orchard, Horne Ranch, Lake Meredith, Matador Wildlife Management Area, Resaca de la Palma State Park, Wildcat Bluff Nature Center

####### Caves.

**Williamson** (Inner Space Caverns)

####### Time of activity.

Male (March, May – August, November); female (March, May – December)

####### Habitat.

(crops: cotton, peanuts); (grass: grassland); (landscape features: cave); (littoral: near playa); (orchard: pecan); (plants: cactus, Indian paintbrush, miscellaneous vegetation, vegetation, weed, yucca); (soil/woodland: ground, mesquite, post oak savanna with pasture, saltcedar, trees/shrubs)

####### Method.

Beating [m]; pitfall trap [mf]; sweeping [mf]

####### Eggs/spiderlings.

Erath [15 spiderlings] [TAMU]

####### Type.

Texas (female, Brazos Co., September, no collector)

####### Etymology.

locality (Latin adjective derived from geographic name, the state of Texas, Banks 1906).

####### Collection.

DMNS, MCZ, MSU, TAMU, TMM, WTAM

###### Phidippus tyrannus

Taxon classificationAnimaliaAraneaeSalticidae

Edwards, 2004

Phidippus tyrannus Edwards 2004: 90, mf, desc. (figs 314–317); Richman et al. 2011b: 61; Richman et al. 2012a: 61; Richman et al. 2012b: 61

####### Distribution.

Culberson, Floyd

####### Locality.

Montgomery Ranch

####### Time of activity.

Male (October); female (June)

####### Type.

Arizona, Skeleton Canyon

####### Etymology.

Latin, tyrant, despot

###### Phidippus vexans

Taxon classificationAnimaliaAraneaeSalticidae

Edwards, 2004

Phidippus vexans Edwards 2004: 35, mf, desc. (figs 54–58); Richman et al. 2011b: 62; Richman et al. 2012a: 62; Richman et al. 2012b: 62

####### Distribution.

Brewster, El Paso, Presidio, Wichita

####### Locality.

Crazy Cat Mountains

####### Time of activity.

Male (May, November); female (October)

####### Habitat.

(grass: on grass, *Bouteloua* sp.)

####### Type.

New Mexico, 17 miles N Las Cruces

####### Etymology.

Latin, to annoy, difficulty in collecting specimens

####### Collection.

MSU, NMSU

###### Phidippus whitmani

Taxon classificationAnimaliaAraneaeSalticidae

Peckham & Peckham, 1909

Phidippus whitmani Bradley 2013: 201; Edwards 2004: 79, mf, desc. (figs C49–51, 254–260); Richman et al. 2011b: 62; Richman et al. 2012a: 62; Richman et al. 2012b: 62

####### Distribution.

Burleson, San Jacinto, Travis

####### Time of activity.

Male (July)

####### Habitat.

(soil/woodland: post oak savanna with pasture)

####### Method.

pitfall trap [m]

####### Type.

New York

####### Etymology.

Person (Patronym for Prof. C. O. Whitman, University of Chicago, Peckham and Peckham 1909).

####### Collection.

TAMU

##### Genus *Phlegra* Simon, 1876

###### Phlegra hentzi

Taxon classificationAnimaliaAraneaeSalticidae

(Marx, 1890)

Phlegra hentzi Bradley 2013: 201; Logunov and Koponen 2002: 264 [S], mf, desc. (figs 1–2, 4–7); Richman et al. 2005: 215; Richman et al. 2011b: 63; Richman et al. 2012a: 63; Richman et al. 2012b: 63
Phlegra fasciata (Hahn, 1826); Agnew et al. 1985: 8; Carpenter 1972: 165; Jackman 1997: 136, desc., 168; Kaston 1953: 109, desc. (fig. 265); Kaston 1972: 264, desc. (fig. 597); Kaston 1978: 253, desc. (fig. 646); Petrunkevitch 1911: 694; Richman and Cutler 1978: 97; Roth 1982: 40–6; Roth 1985: B-36–5; Roth 1994: 158; Vogel 1970b: 20
Phlegra leopardus (Hentz, 1846); Peckham and Peckham 1909: 512

####### Distribution.

Archer, Bell, Brown, Burnet, Carson, Clay, Coryell, Erath, Foard, Hardeman, Kerr, Moore, Randall, Travis, Wichita

####### Locality.

Canoncita Ranch, Lake Meredith National Recreation Area, Medicine Mounds Ranch, Palo Duro Canyon State Park, Pantex Plant, Raven Ranch

####### Time of activity.

Male (April – June, August, September 28-October 4, December); female (February, April – June, August, October)

####### Habitat.

(grass: grassland); (landscape features: rocky hillside, under [rock, stone in sparse grass]); (soil/woodland: open semi-arid areas, post oak savanna with pasture)

####### Method.

pitfall trap [m]

####### Type.

Alabama

####### Etymology.

Person (honor arachnologist)

####### Collection.

MCZ, MSU, TAMU, WTAM

##### Genus *Platycryptus* Hill, 1979

###### Platycryptus californicus

Taxon classificationAnimaliaAraneaeSalticidae

(Peckham & Peckham, 1888)

Platycryptus californicus Hill 1979: 215 [T]; Richman et al. 2011b: 63; Richman et al. 2012a: 63; Richman et al. 2012b: 63
Metacyrba californica (Peckham and Peckham, 1888) [Barnes 1958: 39, mf, desc. (figs 57–58, 61, 64, 68)]

####### Distribution.

Texas

####### Type.

California

####### Etymology.

locality (state)

###### Platycryptus undatus

Taxon classificationAnimaliaAraneaeSalticidae

(De Geer, 1778)

Platycryptus undatus Bradley 2013: 201; Bumroongsook et al. 1992: 18; Calixto et al. 2013: 184; Edwards 2006: 210 (figs 107–108); Hill 1979: 215 [T]; Jackman 1997: 168 (photo 41); Richman et al. 2011b: 64; Richman et al. 2012a: 63; Richman et al. 2012b: 63; Tugmon et al. 1990: 44; Young and Edwards 1990: 22
Marpissa undata (De Geer, 1778); Bonnet 1957: 2729; Jones 1936: 70; Kagan 1942: 72; Kagan 1943: 258
Metacyrba undata (De Geer, 1778); Agnew et al. 1985: 8; Barnes 1958: 36, mf, desc. (figs 55–56, 62, 65, 67, 69); Brown 1974: 236; Carpenter 1972: 167; Hunter 1988: 18–19; Kaston 1972: 264, desc. (figs 595–596); Kaston 1978: 252, desc. (figs 643–644); Rice 1986: 124; Richman and Cutler 1978: 88; Vogel 1970b: 18

####### Distribution.

Angelina, Archer, Bastrop, Bexar, Brazoria, Brazos, Burleson, Cameron, Clay, Collin, Comanche, Coryell, Dallas, Deaf Smith, Denton, Ellis, Erath, Gaines, Harris, Hartley, Hays, Hidalgo, Houston, Jim Wells, Kerr, McLennan, Montgomery, Nacogdoches, Palo Pinto, Polk, Potter, Presidio, Randall, Robertson, Sabine, San Patricio, Tarrant, Taylor, Travis, Tyler, Victoria, Walker, Wheeler, Wichita

####### Locality.

Adriance Pecan Orchard, Bastrop State Park, Bentsen-Rio Grande Valley State Park, Bill Haney Pecan Orchard, Camp Tonkawa, Ellis Prison Unit, Holmes Pecan Orchard, Kirby State Forest, Laguna Atascosa National Wildlife Refuge, Lake Corpus Christi State Park, Lake Tanglewood, Lick Creek Park, Lomita Ranch, Resaca de la Palma State Park, Riley Estate, Somerville Lake

####### Time of activity.

Male (January – December); female (January – December)

####### Habitat.

(crops: near cotton); (grass: grassland, sandy grassland); (landscape features: bridge, culvert, underpass, walls of highway concrete bridges); (littoral: dense coastal brush, sedge meadow); (nest/prey: mud dauber nest [f]); (objects: cage outside, under board); (orchard: pecan, pecan tree trunk); (plants: flower, miscellaneous vegetation); (soil/woodland: beech-magnolia forest, juniper, on [bark, ground], tree trunk, trunk of ornamental tree, under bark, willow, woods, *Ulmus crassifolia*); (structures: indoors, on [bedroom rug, brick wall], screen door, side of building)

####### Method.

Beating [f]; cardboard band [imm.]; flight intercept trap elevated [m]; fogging [mf]; Lindgren funnel trap [f]; malaise trap [m]; moth pheromone trap [m]; pitfall trap [mf]; sweeping [mf]

####### Type.

unknown

####### Etymology.

Latin, wavy lines

####### Collection.

DMNS, MCZ, MSU, NMSU, TAMU, WTAM

##### Genus *Plexippus* C. L. Koch, 1846

###### Plexippus paykulli

Taxon classificationAnimaliaAraneaeSalticidae

(Audouin, 1826)

Plexippus paykulli Bradley 2013: 202; Hunter 1988: 18; Jackman 1997: 136, desc., 168 (photo 41o); Kaston 1953: 105, desc. (fig. 257); Kaston 1972: 250, desc. (fig. 566); Kaston 1978: 241, desc. (fig. 613); Nyffeler et al. 1990a: 92; Petrunkevitch 1911: 695; Rapp 1984: 9; Richman and Cutler 1978: 98; Richman et al. 2011b: 64; Richman et al. 2012a: 64; Richman et al. 2012b: 64; Vogel 1970b: 20
Plexippus paykullii (Audouin, 1826); Peckham and Peckham 1909: 442, mf, desc

####### Distribution.

Brazos, Cameron, Ellis, Galveston, Harris, Hidalgo, Kleberg, Nueces, Walker, Waller

####### Locality.

Ellis Prison Unit, Estero Llano Grande State Park, Galveston Island State Park, McKelvey Park, Resaca de la Palma State Park

####### Time of activity.

Male (February, April – November); female (February – April, June – October, December)

####### Habitat.

(littoral: salt marsh); (objects: wood pile); (soil/woodland: outside on ground, palm); (structures: abandoned barn, bedroom, in [bed, lab])

####### Method.

Boll weevil pheromone trap [imm.]

####### Eggs/spiderlings.

Brazos [eggsac laid October 10, 1978, hatch November 2; 14 spiderlings] [TAMU]

####### Type.

Egypt

####### Etymology.

Person

####### Collection.

MSU, NMSU, TAMU

##### Genus *Poultonella* Peckham & Peckham, 1909

###### Poultonella alboimmaculata

Taxon classificationAnimaliaAraneaeSalticidae

(Peckham & Peckham, 1883)

Poultonella alboimmaculata Carpenter 1972: 165; Cokendolpher and Horner 1978: 135, mf, desc. (figs 1–3, 6–7); Cokendolpher et al. 2008: 10, 50; Hedin and Maddison 2001a: 388; Jackman 1997: 168; Richman and Cutler 1978: 98; Richman et al. 2005: 210; Richman et al. 2011b: 65; Richman et al. 2012a: 65; Richman et al. 2012b: 65

####### Distribution.

Carson, Dickens, Donley, Nolan, Upton, Wichita, Zapata [see note below]

####### Locality.

Falcon Lake, Pantex Lake (edge), Pantex Plant

####### Time of activity.

Male (January, April – August); female (May – September)

####### Habitat.

(crops: *Helianthus* sp.); (grass: grassland, *Bromus tectorum*); (plants: low bush, sparse sage, *Asclepias aenotheroides*, *Gaillardia pulchella*, *Thelesperma* sp.); (soil/woodland: mesquite, saltcedar)

####### Method.

Ballooning [m]; beating [mf]; sweeping

####### Eggs/spiderlings.

Upton [eggsac laid late June 2013, hatched mid July, 13 spiderlings] [TAMU]

####### Type.

Iowa

####### Etymology.

Latin, cephalothorax white, dense short white hairs

####### Collection.

MSU, TAMU, WTAM

####### Note.

not Brewster Co. (mistake on map, pers. comm, N. V. Horner).

###### Poultonella nuecesensis

Taxon classificationAnimaliaAraneaeSalticidae

Cokendolpher & Horner, 1978

Poultonella nuecesensis Cokendolpher and Horner 1978: 137, mf, desc. (figs 4–5, 8–9); Jackman 1997: 168; Richman 1980: 11; Richman et al. 2005: 210; Richman et al. 2011b: 65; Richman et al. 2012a: 65; Richman et al. 2012b: 65

####### Distribution.

Nueces

####### Time of activity.

Male (April, August); female (April)

####### Habitat.

(littoral: salt-grass); (plants: low vegetation, *Gaillardia pulchella*)

####### Method.

sweeping [mf]

####### Type.

Texas (male, Nueces Co., Port Aransas, Mustang Island, August 14, 1977, W. W. Dalquest and R. M. Carpenter, holotype, AMNH)

####### Etymology.

locality (The specific name is derived from Nueces County, Texas, where the original material was collected, Cokendolpher and Horner 1978).

####### Collection.

MCZ, MSU

##### Genus *Rhetenor* Simon, 1902

###### Rhetenor texanus

Taxon classificationAnimaliaAraneaeSalticidae

Gertsch, 1936

Rhetenor texanus Gertsch 1936: 25, mf, desc. (figs 25–26); Jackman 1997: 168; Richman and Cutler 1978: 98; Richman et al. 2005: 213; Richman et al. 2011b: 65; Richman et al. 2012a: 65; Richman et al. 2012b: 65; Roewer 1955: 1017; Roth 1982: 40–5; Roth 1985: B-36–4; Roth 1994: 156; Vogel 1970b: 20

####### Distribution.

Cameron

####### Time of activity.

Male (May); female (November)

####### Type.

Texas (male, Cameron Co., Brownsville, May 25, 1934, J. N. Knull, holotype, AMNH)

####### Etymology.

locality (state)

##### Genus *Salticus* Latreille, 1804

###### Salticus austinensis

Taxon classificationAnimaliaAraneaeSalticidae

Gertsch, 1936

Salticus austinensis Carpenter 1972: 165; Gertsch 1936: 20 (new name); Horner et al. 1988: 260; Jackman 1997: 137, 168; Knutson et al. 2010: 515; Richman and Cutler 1978: 98; Richman et al. 2011b: 66; Richman et al. 2012a: 65; Richman et al. 2012b: 65; Roewer 1955: 1277; Tugmon et al. 1990: 43–44; Vogel 1970b: 20
Epiblemum albocinctum Peckham and Peckham, 1896; F. O. P.-Cambridge 1901: 300, f (pl. 29, fig. 13)
Salticus albocinctus (Peckham and Peckham, 1896); Banks 1910: 74; Jones 1936: 70; Peckham and Peckham 1909: 479, f, desc. (pl. 44, fig. 5); Petrunkevitch 1911: 700

####### Distribution.

Archer, Baylor, Carson, Clay, Dallas, Hidalgo, Howard, Lubbock, Montague, Randall, Travis, Wichita

####### Locality.

Lake McClellan

####### Time of activity.

Female (March – June)

####### Habitat.

(landscape features: concrete dam, rock-faced cliff, under rock); (plants: vegetation); (soil/woodland: open areas, saltcedar, tree trunk); (structures: outside wall of house, overhanging surface, side of building, walls of building)

####### Method.

Beating

####### Eggs/spiderlings.

Wichita [2–5 eggs/sac] [Horner et al. 1988: 260]

####### Type.

unknown

[male unknown]

####### Etymology.

after Austin, Texas

####### Collection.

JCC, MSU, NMSU, WTAM

###### Salticus peckhamae

Taxon classificationAnimaliaAraneaeSalticidae

(Cockerell, 1897)

Salticus peckhamae Broussard and Horner 2006: 255; Calixto et al. 2013: 184; Cokendolpher 1978c: 118; Hill and Edwards 2013: 51; Jackman 1997: 137, 168; Knutson et al. 2010: 515; Richman et al. 2011a: 50; Richman et al. 2011b: 66; Richman et al. 2012a: 66; Richman et al. 2012b: 66 [Peckham and Peckham 1909: 478, mf, desc. (pl. 42, figs 9–9a, pl. 44, fig. 6)]
Icius elegans (Hentz, 1846); Carpenter 1972: 165 [misidentified]

####### Distribution.

Archer, Brewster, Comanche, Howard, Presidio, Reeves, Wichita

####### Locality.

Big Bend Ranch State Park, Bill Haney Pecan Orchard, Chihuahuan desert, Dalquest Research Site

**Time of activity.** Male (October); female (June)

####### Habitat.

(crops: sunflower); (orchard: pecan); (plants: pokeberry); (soil/woodland: mesquite); (structures: bush, saltcedar, tailgate of truck)

####### Method.

cardboard band [f]; pitfall trap [f]

####### Type.

New Mexico

####### Etymology.

Person (*Icius peckhamae* is respectfully dedicated to Mrs. Elizabeth G. Peckham, who, in conjunction with her husband, has done such admirable work on the Attid spiders, Cockerell 1897).

####### Collection.

MSU, TAMU, WTAM

###### Salticus scenicus

Taxon classificationAnimaliaAraneaeSalticidae

(Clerck, 1757)

Salticus scenicus Bradley 2013: 202; Roberts 2001: 51 [Paquin and Dupérré 2003: 200, mf (figs 2242–2244)]

####### Distribution.

Coryell, Potter, Wichita

####### Locality.

Wildcat Bluff Nature Center

####### Type.

unknown

####### Etymology.

Greek, tent

####### Collection.

MSU

##### Genus *Sarinda* Peckham & Peckham, 1892

###### Sarinda hentzi

Taxon classificationAnimaliaAraneaeSalticidae

(Banks, 1913)

Sarinda hentzi Breene et al. 1993c: 24, 48, 64, mf (figs 39A-C); Brown 1974: 237; Calixto et al. 2013: 184; Cokendolpher 1978c: 118; Dean and Eger 1986: 143; Dean et al. 1982: 256; Dean et al. 1988: 287; Galiano 1965: 282, mf, desc. (pl. 2, figs 10–13; pl. 3, fig. 6; pl. 4, fig. 9; pl. 6, fig. 6; pl. 7, figs 8–9); Hunter 1988: 18–20; Jackman 1997: 168; Kaston 1972: 255, desc. (fig. 575); Kaston 1978: 245, desc. (fig. 622); Nyffeler et al. 1992c: 3; Richman and Cutler 1978: 98 [T]; Richman et al. 2011b: 66; Richman et al. 2012a: 66; Richman et al. 2012b: 66; Young and Edwards 1990: 22
Myrmarachne hentzi (Banks, 1913); Kaston 1953: 121, desc. (figs 295–296) [Kaston 1948: 449, mf, desc. (figs 1611–1612, 1625–1627)]

####### Distribution.

Angelina, Brazos, Burleson, Colorado, Comanche, Coryell, Ellis, Goliad, Hidalgo, Hill, Houston, Hunt, Kenedy, Kerr, Lavaca, Montague, Nacogdoches, Rains, Robertson, Titus, Tyler, Uvalde, Walker, Wichita, Wilbarger, Wood

####### Locality.

5-Eagle Ranch, Angelina National Forest, Attwater Prairie Chicken National Wildlife Refuge, Bill Haney Pecan Orchard, Ellis Prison Unit, Garner State Park, Holmes Pecan Orchard, Kirby State Forest, Texas A&M University Rangeland Area

####### Time of activity.

Male (March – August, October – November); female (April – August, September 25-October 2)

####### Habitat.

(crops: cotton); (grass: grass, grassland, pasture); (orchard: pecan); (plants: bluebonnets, Indian paintbrush, miscellaneous vegetation, vegetation, roadside vegetation, weed, *Monarda citriodora*); (soil/woodland: hardwood bottomland, leaf litter, post oak savanna with pasture); (structures: on patio)

####### Method.

Boll weevil pheromone trap at house [m]; cardboard band [m]; flight intercept trap on ground [mf]; pitfall trap [mf]; sweeping [mf]

####### Type.

unknown

####### Etymology.

Person (arachnologist)

####### Collection.

MSU, TAMU

##### Genus *Sassacus* Peckham & Peckham, 1895

###### Sassacus cyaneus

Taxon classificationAnimaliaAraneaeSalticidae

(Hentz, 1846)

Sassacus cyaneus Bradley 2013: 203; Richman 2008: 33 [T], mf, desc. (figs 17–22); Richman et al. 2011b: 67; Richman et al. 2012a: 67; Richman et al. 2012b: 67
Agassa cyanea (Hentz, 1846); Agnew et al. 1985: 8; Breene et al. 1993c: 22, 48, 67, mf (figs 46A-C); Brown 1974: 236; Dean and Sterling 1987: 7; Jackman 1997: 167; Jones 1936: 69; Richman et al. 2011b: 67; Vogel 1970b: 17

####### Distribution.

Blanco, Brazos, Collin, Colorado, Dallas, Fort Bend, Frio, Hale, Mitchell, Nacogdoches, San Patricio, Tom Green

####### Locality.

Attwater Prairie Chicken National Wildlife Refuge, Lick Creek Park

####### Time of activity.

Male (May, July – August); female (May – July)

####### Habitat.

(crops: cotton); (nest/prey: mud dauber nest [f]); (plants: Mexican hat, vegetation); (soil/woodland: juniper)

####### Method.

D-Vac suction [m]; sweeping [mf]

####### Type.

North Carolina and Alabama

####### Etymology.

Latin, color

####### Collection.

TAMU

###### Sassacus papenhoei

Taxon classificationAnimaliaAraneaeSalticidae

Peckham & Peckham, 1895

Sassacus papenhoei Agnew et al. 1985: 5, 11; Breene et al. 1993c: 24, 48, 68, mf (figs 49A-C); Calixto et al. 2013: 184; Carpenter 1972: 164; Cokendolpher et al. 2008: 10, 50; Dean and Sterling 1987: 7; Hunter 1988: 18–21; Jackman 1997: 168; Jones 1936: 70; Kagan 1942: 69; Kagan 1943: 258; Knutson et al. 2010: 515; Peckham and Peckham 1909: 592; Petrunkevitch 1911: 704; Richman 2008: 28, mf, desc. (figs 1–8); Richman et al. 2011b: 68; Richman et al. 2012a: 67; Richman et al. 2012b: 67; Roberts 2001: 51; Roewer 1955: 1228; Rogers and Horner 1977: 523; Vogel 1970b: 20; Young and Edwards 1990: 22

####### Distribution.

Archer, Brazos, Brewster, Brown, Burnet, Calhoun, Cameron, Carson, Collin, Comanche, Dallas, Denton, Dickens, El Paso, Ellis, Erath, Floyd, Gaines, Grayson, Hale, Howard, Jones, Kenedy, Kerr, Limestone, Martin, McLennan, Midland, Montague, Nolan, Nueces, Potter, Randall, Scurry, Somervell, Sterling, Taylor, Tom Green, Travis, Val Verde, Webb, Wichita, Wilbarger, Winkler, Wise, Yoakum

####### Locality.

Bill Haney Pecan Orchard, Lake Thomas, Pantex Lake, Pantex Plant, Seminole Canyon State Park, White Rock Lake, Wildcat Bluff Nature Center

####### Time of activity.

Male (April – September); female (May – September, November)

####### Habitat.

(crops: cotton, guar, peanuts, sunflower); (grass: grass, grassland); (littoral: near playa); (orchard: pecan); (plants: brown-eyed Susan, Compositae, flower, low vegetation, roadside vegetation, weed, *Baccharis*, *Gutierrezia*); (soil/woodland: juniper, mesquite, saltcedar, willow, woodland, *Ulmus crassifolia*)

####### Method.

cardboard band [imm.]; D-Vac suction [f]; suction trap [m]; sweeping [m]

####### Type.

Kansas

####### Etymology.

Person (We have a number of males and females sent us from Wallace, Kansas, by Mr. Papenhoe, Peckham and Peckham 1895).

####### Collection.

MCZ, MSU, NMSU, TAMU, WTAM

###### Sassacus vitis

Taxon classificationAnimaliaAraneaeSalticidae

(Cockerell, 1894)

Sassacus vitis Hill 1979: 217; Richman 2008: 35 [T], mf, desc. (figs 29–35); Richman et al. 2011b: 68; Richman et al. 2012a: 68; Richman et al. 2012b: 68;
Icius vitis (Cockerell, 1894); Peckham and Peckham 1909: 501; Petrunkevitch 1911: 662; Woods and Harrel 1976: 44
Metaphidippus vitis (Cockerell, 1894); Agnew et al. 1985: 8; Carpenter 1972: 164; Jackman 1997: 167; Jones 1936: 70; Kagan 1942: 65; Kagan 1943: 258; Richman and Cutler 1978: 91 [T]; Vogel 1970b: 18; Young and Edwards 1990: 22

####### Distribution.

Bell, Bosque, Brewster, Burnet, Cameron, Dallas, Denton, Erath, Grayson, Hidalgo, Hunt, Jefferson, Johnson, Kerr, Kimble, Llano, McLennan, Palo Pinto, Presidio, Runnels, Scurry, Travis, Wichita, Zapata

####### Locality.

Big Bend National Park, Estero Llano Grande State Park, Lacuna Park, Lake Thomas

####### Time of activity.

Male (April, July – September, November); female (June, August – September, November)

####### Habitat.

(crops: rice); (grass: grass, on ground with sparse grass); (plants: shrubs, vegetation, *Baccharis*); (soil/woodland: edge of plowed field, limbs of bushes, saltcedar, wheel-ruts of dirt roads, willow)

####### Method.

D-Vac suction [mf]; sweeping [mf]

####### Type.

New Mexico

####### Etymology.

Latin, vine

####### Collection.

MCZ, MSU, TAMU

##### Genus *Sitticus* Simon, 1901

###### Sitticus concolor

Taxon classificationAnimaliaAraneaeSalticidae

(Banks, 1895)

Sitticus concolor Maddison 1996: 270 [S]
Sitticus cf. cursor Barrows, 1919; Carpenter 1972: 165; Jackman 1997: 168
Sitticus floridanus Gertsch and Mulaik, 1936 [Kaston 1948: 459, mf, desc. (figs 1686–1688)]

####### Distribution.

Hays, Wichita

####### Locality.

Lake Wichita

####### Time of activity.

Female (January 27-February 24)

####### Habitat.

(grass: open ground in dense grass); (soil/woodland: *Juniperus* unmanaged plot)

####### Method.

Flight intercept trap on ground [f]

####### Type.

Missouri

####### Etymology.

Latin, for one color

####### Collection.

TAMU

###### Sitticus dorsatus

Taxon classificationAnimaliaAraneaeSalticidae

(Banks, 1895)

Sitticus dorsatus Agnew et al. 1985: 5; Breene et al. 1993c: 24, 48, 67, mf (figs 47A-B); Broussard and Horner 2006: 255; Dean and Sterling 1987: 7; Jackman 1997: 168; Richman 1979: 125 [S]; Richman et al. 2011a: 50 [S]; Richman et al. 2011b: 70; Richman et al. 2012a: 69; Richman et al. 2012b: 69; Young and Edwards 1990: 22
Sitticus absolutus Gertsch and Mulaik, 1936; Bonnet 1958: 4068; Cokendolpher 1978c: 118; Gertsch and Mulaik 1936a: 19, mf, desc. (figs 19–20); Prószyn’ski 1973: 79, mf, desc. (figs 17–19, 22–25, 33–35, 39, 43–44); Richman and Cutler 1978: 99; Roewer 1955: 1250
Sittacus absolutus Gertsch and Mulaik, 1936; Vogel 1970b: 20
Sitticus callidus Gertsch and Mulaik, 1936; Bonnet 1958: 4069; Gertsch and Mulaik 1936a: 20, mf, desc. (figs 17–18); Roewer 1955: 1251
Sittacus callidus Gertsch and Mulaik, 1936; Vogel 1970b: 20

####### Distribution.

Archer, Bosque, Brewster, Brown, Cameron, Coryell, Erath, Frio, Hidalgo, Kleberg, Presidio, Wichita

####### Locality.

Bentsen-Rio Grande Valley State Park, Chihuahuan desert, Dalquest Research Site, Lacuna Park

####### Time of activity.

Male (April – August, August 28-September 4, October – November); female (May – November)

####### Habitat.

(crops: cotton, peanuts); (grass: grass); (soil/woodland: juniper, leaf litter, post oak savanna with pasture, sandy area, under oak); (structures: porch)

####### Method.

pitfall trap [mf] (in sand [f], under juniper [mf], under oak [m]); suction trap [m]

####### Type.

California

####### Etymology.

Latin, dorsal markings

####### Collection.

MSU, TAMU

###### Sitticus welchi

Taxon classificationAnimaliaAraneaeSalticidae

Gertsch & Mulaik, 1936

Sitticus welchi Bonnet 1958: 4085; Gertsch and Mulaik 1936a: 21, f, desc. (fig. 31); Jackman 1997: 168; Richman and Cutler 1978: 99; Richman et al. 2011b: 72; Richman et al. 2012a: 72; Richman et al. 2012b: 72; Roewer 1955: 1251
Sittacus welchi Gertsch and Mulaik, 1936; Vogel 1970b: 20

####### Distribution.

Val Verde

####### Time of activity.

Female (August)

####### Type.

Texas (female, Val Verde Co., Langtry, August 18, 1935, S. Mulaik, holotype, AMNH)

[male unknown]

####### Etymology.

Person

##### Genus *Synageles* Simon, 1876

###### Synageles bishopi

Taxon classificationAnimaliaAraneaeSalticidae

Cutler, 1988

Synageles bishopi Cutler 1988a: 330, mf, desc. (figs 5, 10–13); Dean and Sterling 1990: 405; Jackman 1997: 168; Richman et al. 2011b: 72; Richman et al. 2012a: 72; Richman et al. 2012b: 72

####### Distribution.

Bastrop, Montgomery, Walker

####### Locality.

Ellis Prison Unit, Jones State Forest

####### Time of activity.

Male (April)

####### Method.

suction trap [m]

####### Type.

Pennsylvania, NE Jamison, Neshaminy Creek, Horseshoe Bend

####### Etymology.

Person (Named after Sherman C. Bishop, arachnologist (and herpetologist) from the eastern United States in the first half of the twentieth century, Cutler 1988a).

####### Collection.

TAMU

###### Synageles noxiosus

Taxon classificationAnimaliaAraneaeSalticidae

(Hentz, 1850)

Synageles noxiosus Calixto et al. 2013: 184; Cutler 1988a: 334 [spelling], mf, desc. (figs 1, 7, 18–24); Dean and Sterling 1990: 403, 405; Henderson 2007: 53, 78, 81, 84; Jackman 1997: 168; Richman et al. 2011b: 72; Richman et al. 2012a: 72; Richman et al. 2012b: 72
Synageles noxiosa (Hentz, 1850); Agnew et al. 1985: 8; Cokendolpher 1978c: 118; Hunter 1988: 18, 20; Richman and Cutler 1978: 100
Sarinda hentzi Banks, 1913; Dean and Sterling 1990: 403, 405 [misidentified]

####### Distribution.

Eastern 2/3 Texas; Bastrop, Brazos, Burleson, Coryell, Dallas, Ellis, Erath, Harris, Hays, Hunt, Kerr, Kleberg, Palo Pinto, Robertson, San Patricio, Shelby, Walker, Wichita, Zavala

####### Locality.

Ellis Prison Unit, Holmes Pecan Orchard, Lick Creek Park

####### Time of activity.

Male (March – May); female (March – April, April 26-May 2, June)

####### Habitat.

(grass: sandy-prairie grass); (orchard: pecan); (plants: weed); (soil/woodland: elm, juniper, post oak savanna with pasture, upland woods)

####### Method.

Beating/sweeping [m]; cardboard band [mf]; D-Vac suction [f]; pitfall trap [mf]; suction trap [m]; sweeping

####### Type.

Alabama

####### Etymology.

Latin, injurious

####### Collection.

MSU, TAMU

##### Genus *Synemosyna* Hentz, 1846

###### Synemosyna formica

Taxon classificationAnimaliaAraneaeSalticidae

Hentz, 1846

Synemosyna formica Cutler 1988b: 198; Jackman 1997: 168; Richman et al. 2005: 208; Richman et al. 2011b: 74; Richman et al. 2012a: 73; Richman et al. 2012b: 73 [Kaston 1948: 448, desc. mf (figs 1609–1610, 1623–1624); Peckham and Peckham 1909: 366, mf, desc. (pl. 50, figs 1–1c)]

####### Distribution.

Angelina, Kerr, Travis

####### Locality.

Davy Crockett National Forest

####### Time of activity.

Male (April, July)

####### Habitat.

(grass: grass)

####### Type.

North Carolina and Alabama

####### Etymology.

Latin, refers to ants

####### Collection.

MSU, TAMU

##### Genus *Talavera* Peckham & Peckham, 1909

###### Talavera minuta

Taxon classificationAnimaliaAraneaeSalticidae

(Banks, 1895)

Talavera minuta Agnew et al. 1985: 8; Jackman 1997: 168; Richman et al. 2005: 213; Richman et al. 2011b: 74; Richman et al. 2012a: 74; Richman et al. 2012b: 74; Roth 1985: B-36–4; Roth 1994: 156 [Kaston 1948: 470, mf, desc. (figs 1738–1739)]

####### Distribution.

Cameron, Coryell, Erath

####### Locality.

McKelvey Park

####### Time of activity.

Male (March 29-April 5, April, April 25-May 2, June); female (May – July)

####### Habitat.

(soil/woodland: post oak savanna with pasture, under [juniper, oak])

####### Method.

pitfall trap [mf] (under juniper [f], under oak [mf])

####### Type.

Washington

####### Etymology.

Latin, size

####### Collection.

TAMU

##### Genus *Tutelina* Simon, 1901

###### Tutelina elegans

Taxon classificationAnimaliaAraneaeSalticidae

(Hentz, 1846)

Tutelina elegans Chickering 1944: 211 [T]; Jackman 1997: 168; Richman et al. 2011b: 76; Richman et al. 2012a: 76; Richman et al. 2012b: 76
Icius elegans (Hentz, 1846) [Kaston 1948: 488, mf, desc. (figs 1809–1811, 1833–1837)]

####### Distribution.

Jones, Montague

####### Time of activity.

Male (June)

####### Habitat.

(plants: vegetation); (soil/woodland: trees/shrubs)

####### Method.

Beating [m]

####### Type.

southern states

####### Etymology.

Latin, elegant

####### Collection.

MSU, TAMU

###### Tutelina similis

Taxon classificationAnimaliaAraneaeSalticidae

(Banks, 1895)

Tutelina similis Chickering 1944: 216 [T]; Richman et al. 2011a: 50 [Paquin and Dupérré 2003: 203 (figs 2276–2278)]
Icius similis Banks, 1895 [Kaston 1948: 489 (figs 1812–1813, 1838–1840)]

####### Distribution.

Presidio

####### Type.

Washington, Olympia; Colorado, Fort Collins

####### Etymology.

Latin, similar to *Icius elegans* Hentz

##### Genus *Zygoballus* Peckham & Peckham, 1885

###### Zygoballus nervosus

Taxon classificationAnimaliaAraneaeSalticidae

(Peckham & Peckham, 1888)

Zygoballus nervosus Breene et al. 1993c: 25, 48, 69, mf (figs 53A-C); Dean and Sterling 1990: 405; Dean et al. 1982: 256; Jackman 1997: 168; Nyffeler et al. 1992c: 3; Richman et al. 2011b: 77; Richman et al. 2012a: 77; Richman et al. 2012b: 77 [Peckham and Peckham 1909: 580, mf, dec. (pl. 50, figs 8–8c, pl. 51, fig. 12)]

####### Distribution.

Burleson, Colorado, Freestone, Hidalgo, Travis, Walker

####### Locality.

Attwater Prairie Chicken National Wildlife Refuge, Bentsen-Rio Grande Valley State Park, Ellis Prison Unit, Zilker Park

####### Time of activity.

Male (March – May, August); female (March – April, June, August, October)

####### Habitat.

(crops: cotton); (plants: miscellaneous vegetation, *Monarda citriodora*)

####### Method.

Boll weevil pheromone trap [mf]; D-Vac suction [f]; suction trap [mf]; sweeping [mf]

####### Type.

New York

####### Etymology.

Latin, for nervous

####### Collection.

TAMU

###### Zygoballus rufipes

Taxon classificationAnimaliaAraneaeSalticidae

Peckham & Peckham, 1885

Zygoballus rufipes Agnew et al. 1985: 5, 11; Banks 1910: 74; Breene et al. 1993c: 25, 48, 68, 69, mf (figs 51A-B, 52A-C); Comstock 1912: 697; Comstock 1940: 705; Dean and Eger 1986: 143; Dean and Sterling 1990: 403, 405; Dean et al. 1982: 256; Dean et al. 1987: 268; Dean et al. 1988: 287; Edwards 1980: 12 [S]; Henderson 2007: 64, 70, 78, 81, 84; Hill and Edwards 2013: 35; Hunter 1988: 18–21; Jackman 1997: 138, desc., 168; Nyffeler et al. 1992c: 3; Peckham and Peckham 1909: 581, mf, desc.; Petrunkevitch 1911: 719; Rapp 1984: 9; Richman and Cutler 1978: 101; Richman et al. 2011b: 78; Richman et al. 2012a: 77; Richman et al. 2012b: 77; Roewer 1955: 1018; Vogel 1970b: 20; Young and Edwards 1990: 23
Zygoballus nervosus (Peckham and Peckham, 1888); Dean and Eger 1986: 143 [misidentified]
Zygoballus bettini Peckham and Peckham, 1888; Brown 1974: 237; Kaston 1953: 115, desc. (fig. 279); Kaston 1972: 265, desc. (fig. 599); Kaston 1978: 254, desc. (fig. 648); Pamanes-Guerrero 1975: 60; Peckham and Peckham 1909: 579, mf, desc. (pl. 50, figs 7–7e, pl. 51, fig. 10); Petrunkevitch 1911: 718; Vogel 1970b: 20

####### Distribution.

Anderson, Archer, Bastrop, Bexar, Bosque, Brazos, Burleson, Cameron, Colorado, Coryell, Ellis, Erath, Falls, Fannin, Fort Bend, Galveston, Hays, Hidalgo, Houston, Hunt, Kerr, Montgomery, Nacogdoches, Polk, San Patricio, Titus, Travis, Uvalde, Walker, Williamson, Wood

####### Locality.

Attwater Prairie Chicken National Wildlife Refuge, Bentsen-Rio Grande Valley State Park, Brazos Bend State Park, Ellis Prison Unit, Galveston Island State Park, Garner State Park, Jones State Forest, Lacuna Park, Lick Creek Park, Mansfield Dam, Reimers Ranch Park, Sabal Palm Audubon Sanctuary, Sam Houston National Forest, Santa Ana National Wildlife Refuge, Stubblefield Lake, Welder Wildlife Refuge

####### Time of activity.

Male (March – November); female (February – December)

####### Habitat.

(crops: cotton, peanuts); (grass: grass, grassland, pasture); (littoral: salt marsh, sedge meadow); (nest/prey: mud dauber nest [mf]); (plants: bluebonnets, Indian paintbrush, miscellaneous vegetation, weed, *Monarda citriodora*); (soil/woodland: buckeye-sycamore forest, disturbed habitat, hackberry matte, juniper, post oak savanna with pasture, roadside vegetation, sandy area, upland woods, woods, woodland, *Quercus virginiana*); (structures: abandoned shack)

####### Method.

Boll weevil pheromone trap [m]; flight intercept trap [f]; pitfall trap [mf] (in sand [m]); suction trap [mf]; sweeping [mf]

####### Type.

Guatemala

####### Etymology.

Latin, reddish legs

####### Collection.

MSU, TAMU

###### Zygoballus sexpunctatus

Taxon classificationAnimaliaAraneaeSalticidae

(Hentz, 1845)

Zygoballus sexpunctatus Armstrong and Richman 2007: 396; Brown 1974: 237; Cokendolpher 1978c: 118; Dean and Sterling 1990: 403, 405; Jackman 1997: 168; Kaston 1953: 115, desc. (fig. 280); Kaston 1972: 266, desc. (fig. 600); Kaston 1978: 254, desc. (fig. 649); Peckham and Peckham 1909: 583, mf, desc. (pl. 51, fig. 11); Petrunkevitch 1911: 720; Richman and Cutler 1978: 101; Richman et al. 2011b: 78; Richman et al. 2012a: 78; Richman et al. 2012b: 78; Vogel 1970b: 20
Zygoballus nervosus (Peckham and Peckham, 1888); Breene et al. 1993c: 25, 48, 69 (fig. 53); Dean and Eger 1986: 143; Dean and Sterling 1990: 405; Dean et al. 1982: 256; Young and Edwards 1990: 23 [all misidentified]
Zygoballus rufipes Peckham and Peckham, 1885; Dean and Eger 1986: 143; Dean and Sterling 1987: 7 [misidentified]

####### Distribution.

Blanco, Brazos, Burleson, Cameron, Colorado, Comal, Coryell, Fayette, Hidalgo, Lavaca, McMullen, Nacogdoches, San Patricio, Tyler, Walker, Wichita

####### Locality.

Attwater Prairie Chicken National Wildlife Refuge, Browning Ranch, Ellis Prison Unit, Estero Llano Grande State Park, Kirby State Forest, Lick Creek Park, Russell Farm, Sabal Palm Audubon Sanctuary, Texas A&M University Rangeland Area, Welder Wildlife Refuge

####### Time of activity.

Male (April – November); female (February, April – October)

####### Habitat.

(crops: cotton); (littoral: sedge meadow); (nest/prey: mud dauber nest [mf]); (orchard: organic citrus grove); (plants: bluebonnets, Indian paintbrush, miscellaneous vegetation, vegetation); (soil/woodland: hackberry matte, post oak savanna, post oak savanna with pasture)

####### Method.

Boll weevil pheromone trap [m]; D-Vac suction [m]; pitfall trap [mf]; suction trap [mf]; sweeping [mf]

####### Type.

North Carolina

####### Etymology.

Latin, spots on abdomen

####### Collection.

MSU, NMSU, TAMU

#### Family Scytodidae Blackwall, 1864

**Note.** Species incorrectly reported from Texas

*Scytodes championi* F. O. P.-Cambridge, 1899; Gertsch 1935a: 9; Jackman 1997: 168; Vogel 1970b: 21 [not in United States, misidentified]

##### Genus *Scytodes* Latreille, 1804

###### Scytodes atlacoya

Taxon classificationAnimaliaAraneaeSalticidae

Rheims, Brescovit & Durán, 2007

Scytodes atlacoya : partial data from G.B. Edwards, pers. comm. [Rheims et al. 2007: 96, mf, desc. (figs 7–8, 28–32)]
Scytodes intricata Banks, 1909; Bonnet 1958: 3984; Comstock 1940: 317, desc.; Gertsch 1935a: 9; Gertsch and Mulaik 1940: 319; Reddell and Cokendolpher 2004: 91; Roewer 1942: 330; Vogel 1970b: 21 [erroneous identification, see Brown 1974: 237; Valerio 1981: 84]
Scytodes longipes Lucas, 1844; Comstock 1912: 306 [Texas record]; Vogel 1970b: 21 [see Gertsch and Mulaik 1940: 319]
Scytodes n. sp.; Agnew et al. 1985: 6, 11; Jackman 1997: 36
Scytodes sp.; Brown 1974: 237 [undescribed species]; Yantis 2005: 202

####### Distribution.

Aransas, Archer, Bexar, Brazos, Burleson, Cameron, Collin, Coryell, Dallas, DeWitt, Erath, Fayette, Harris, Hays, Hidalgo, Houston, Jasper, Kendall, Kerr, Kleberg, Leon, Llano, Nacogdoches, Nueces, San Patricio, Starr, Travis, Washington, Webb, Wichita, Zapata

####### Locality.

Aransas National Wildlife Refuge, Arkansas Bend Park, Bentsen-Rio Grande Valley State Park, El Rancho Cima Scout Camp, Falcon State Park, Iron Wheel Mesa, Sabal Palm Audubon Sanctuary, Santa Ana National Wildlife Refuge, Welder Wildlife Refuge

####### Caves.

**Bexar** (Strange Little Cave)

####### Time of activity.

Male (February – November); female (February – December)

####### Habitat.

(landscape features: cave, under rock); (nest/prey: mud dauber nest [m]); (soil/woodland: cedar elm forest, hackberry woodland, hollow log, in [branch, dead log], *Juniperus* managed plot, *Juniperus* unmanaged plot, live oak woodland, palm forest, post oak savanna with pasture, post oak woods [93%], Red bay-liveoak forest, upland deciduous forest, yucca-*Quercus incana* association); (structures: cellar, in [house, lab, tent], garage, on house, porch, storeroom, under picnic table, window screen); (web: in webs in trees at night)

####### Method.

5 gallon bucket trap [m]; flight intercept trap on ground [mf]; pitfall trap [mf]

####### Eggs/spiderlings.

Brazos [36 spiderlings] [TAMU]

####### Type.

Mexico, Tamaulipas

####### Etymology.

noun in apposition taken from Aztec mythology; Atlacoya is believed to be the goddess of drought

####### Collection.

MSU, NMSU, TAMU

###### Scytodes dorothea

Taxon classificationAnimaliaAraneaeSalticidae

Gertsch, 1935

Scytodes dorothea Bonnet 1958: 3982; Gertsch 1935a: 9, mf, desc. (figs 10, 13); Gertsch and Mulaik 1940: 318; Jackman 1997: 168; Roewer 1942: 329; Vogel 1970b: 21
Scytodes fusca Walckenaer, 1837; Gertsch 1935a: 9 [see Gertsch and Mulaik 1940: 318]; Vogel 1970b: 21 [Texas records]

####### Distribution.

Cameron, Hidalgo, Kleberg, Nueces

####### Time of activity.

Male (September – October); female (January, March – June, September – October)

####### Type.

Texas (male, Hidalgo Co., Edinburg, October 22–25, 1934, S. Mulaik, holotype, AMNH)

####### Etymology.

Person (This fine species is named for Mrs. Stanley Mulaik [Dorothea], Gertsch 1935a).

###### Scytodes lugubris

Taxon classificationAnimaliaAraneaeSalticidae

(Thorell, 1887)

Scytodes lugubris : partial data from G.B. Edwards, pers. comm. [Rheims et al. 2007: 105, mf, desc. (figs 20–22, 90–93)]

####### Distribution.

Cameron, Hidalgo

####### Locality.

Bentsen-Rio Grande Valley State Park, Sabal Palm Audubon Sanctuary, Santa Ana National Wildlife Refuge, Valley Botanical Garden

####### Time of activity.

Male (May, August – October); female (February – March, May, August, October, November 20-December 4)

####### Habitat.

(grass: grass); (orchard: grapefruit); (soil/woodland: debris under banana trees, palm tree, palm forest, under [bark, log])

####### Method.

Beating [mf]; pitfall trap [mf]

####### Eggs/spiderlings.

Hidalgo [21, 44, 50, 60 spiderlings]; [eggsac hatch March 25, 1980, 56 spiderlings, 8 eggs unhatched] [TAMU]

####### Type.

Myanmar

####### Etymology.

Latin, dark

####### Collection.

TAMU

###### Scytodes thoracica

Taxon classificationAnimaliaAraneaeSalticidae

(Latreille, 1802)

Scytodes thoracica Gertsch and Mulaik 1940: 318; Jackman 1997: 168; Vogel 1970b: 21 [Kaston 1948: 65, mf, desc. (figs 17–21)]

####### Distribution.

Hidalgo

####### Time of activity.

Female (March – April)

####### Type.

unknown

####### Etymology.

Greek, markings on cephalothorax

###### Scytodes univittata

Taxon classificationAnimaliaAraneaeSalticidae

Simon, 1882

Scytodes univittata Rheims et al. 2007: 106 [S] [Brescovit and Rheims 2000: 323, mf, desc. (figs 11–20)]
Scytodes perfecta Banks, 1898; Gertsch 1935a: 7, f, desc. (figs 12, 17); Gertsch and Mulaik 1940: 318; Jackman 1997: 168; Kaston 1972: 86, desc. (fig. 197); Kaston 1978: 88, desc. (fig. 215); Roewer 1942: 330; Vogel 1970b: 21 [Texas record]

####### Distribution.

Brazos, Cameron, El Paso, Hidalgo, Nueces, San Patricio, Travis, Webb

####### Locality.

Franklin Mountains

####### Time of activity.

Male (March – June, August); female (March, May, October)

####### Habitat.

(landscape features: coal mine [4000 feet down]); (structures: in bathroom, bedroom, indoors, on stairway)

####### Type.

Yemen

####### Etymology.

Latin, one stripe

####### Collection.

NMSU, TAMU

###### Scytodes zapatana

Taxon classificationAnimaliaAraneaeSalticidae

Gertsch & Mulaik, 1940

Scytodes zapatana Gertsch and Mulaik 1940: 318, f, desc.; Jackman 1997: 168; Vogel 1970b: 21
Scytodes zapatan Gertsch and Mulaik, 1940; Vogel 1967: 123

####### Distribution.

Presidio, Zapata

####### Locality.

Big Bend Ranch State Park

####### Time of activity.

Female (November)

####### Type.

Texas (female, Zapata Co., 32 miles SE Laredo, November 11, 1934, S. Mulaik, holotype, AMNH)

[male unknown]

####### Etymology.

locality (county)

####### Collection.

NMSU

#### Family Segestriidae Simon, 1893

##### Genus *Ariadna* Audouin, 1826

###### Ariadna bicolor

Taxon classificationAnimaliaAraneaeSegestriidae

(Hentz, 1842)

Ariadna bicolor Agnew et al. 1985: 6, 11; Beatty 1970: 458, mf, desc. (figs 38, 42–43); Brown 1974: 237; Calixto et al. 2013: 184; Gertsch and Mulaik 1940: 323; Henderson 2007: 61–64, 78, 81, 84; Jackman 1997: 41, desc., 168; Reddell 1965: 176; Reddell and Smith 1965: 33; Vogel 1970b: 21; Yantis 2005: 196, 199

####### Distribution.

Bastrop, Brazos, Brewster, Brown, Cameron, Comal, Denton, Edwards, Erath, Hays, Hidalgo, Hood, Kerr, Lampasas, Leon, Lubbock, Madison, Matagorda, McLennan, Nacogdoches, Robertson, Sabine, Smith, Starr, Travis, Walker, Wichita, Williamson, Young

####### Locality.

Bastrop State Park, Chisos Mountains, Holmes Pecan Orchard, Lick Creek Park, Lower Rio Grande Valley National Wildlife Refuge

####### Caves.

**Edwards** (Dunbar Cave); **Lampasas** (Battery Cave)

####### Time of activity.

Male (March, May – July, September – October, October 27-November 11); female (March – May, July – September, December)

####### Habitat.

(landscape features: cave); (littoral: near water); (nest/prey: *Neotoma* rat nest litter); (orchard: pecan); (soil/woodland: beech bottom, beech-magnolia forest, *Juniperus* managed plot, leaf litter, post oak woods [%: 80, 85, 93], post oak woodland, riparian mesquite forest, upland deciduous forest, under [bark of pine tree, log, woods]); (structures: indoors)

####### Method.

5 gallon bucket trap [m]; berlese funnel [f]; cardboard band [f]; flight intercept trap [m]; flight intercept trap on ground [m]; malaise trap [f]; pitfall trap [m]; ramp trap [m]; suction trap [m]; tile trap [m]

####### Type.

Alabama

####### Etymology.

Latin, color of carapace and abdomen

####### Collection.

DMNS, JCC, MSU, TAMU, TMM, TTU

#### Family Selenopidae Simon, 1897

##### Genus *Selenops* Latreille, 1819

###### Selenops actophilus

Taxon classificationAnimaliaAraneaeSelenopidae

Chamberlin, 1924

Selenops actophilus Bradley 2013: 208; Cokendolpher 1982: 2; Crews 2011: 57, mf, desc. (figs 53–56, 198); Jackman 1997: 118, 168; Muma 1953: 14, mf, desc. (figs 19–22); Roewer 1955: 737; Vogel 1970b: 21

####### Distribution.

Brewster, Presidio, Val Verde

####### Locality.

Big Bend National Park, Chisos Basin, Seminole Canyon State Park

####### Time of activity.

Male (April – July); female (April, June – July)

####### Habitat.

(landscape features: under [bridge, rock]); (soil/woodland: running on ground)

####### Type.

Mexico, Sonora, San Carlos Bay

####### Etymology.

Greek, rocky loving

####### Collection.

NMSU

#### Family Sicariidae Keyserling, 1880

**Note.** Loxoscelidae became a synonym of Sicariidae (Platnick et al. 1991: 71).

**nomen dubium**

*Loxosceles unicolor* Keyserling, 1887; Kaston 1953: 41; Kaston 1972: 88

##### Genus *Loxosceles* Heineken & Lowe, 1832

###### Loxosceles apachea

Taxon classificationAnimaliaAraneaeSicariidae

Gertsch & Ennik, 1983

Loxosceles apachea Gertsch and Ennik 1983: 296, mf, desc. (figs 64–67, 92–96) [see note below]; Jackman 1997: 35, 168; Vetter 2008: 152; Vetter 2009: 519; Vetter 2015: 75, 78, 83
Loxosceles arizonica Gertsch and Mulaik, 1940; Gertsch 1958b: 13 [some West Texas records]

####### Distribution.

El Paso, Terrell

####### Time of activity.

Male (March, June, November – December); female (March, December)

####### Habitat.

(objects: trash pile on dry hillside)

####### Type.

Arizona, Portal

####### Etymology.

Indians (Specific name for Apache Indians, Gertsch and Ennik 1983).

####### Collection.

NMSU

####### Note.

Hudspeth Co. mistakenly listed [Gertsch and Ennik 1983: 293], should be El Paso.

###### Loxosceles blanda

Taxon classificationAnimaliaAraneaeSicariidae

Gertsch & Ennik, 1983

Loxosceles blanda Gertsch and Ennik 1983: 298 [S], mf, desc. (figs 68–71, 97–101); Jackman 1997: 35, 168; Vetter 2005: 514; Vetter 2008: 152; Vetter 2009: 519; Vetter 2015: 75, 78, 83
Loxosceles unicolor Keyserling, 1887; Kunath and Smith 1968: 51; Reddell 1965: 173; Vogel 1970b: 21 [part]
Loxosceles arizonicus Gertsch and Mulaik, 1940; Gertsch 1939b: 24; Vogel 1967: 121; Vogel 1970b: 21 [West Texas records]
Loxosceles arizonica Gertsch and Mulaik, 1940; Gertsch 1958b: 13 [some West Texas records]

####### Distribution.

Brewster, Crockett, Jeff Davis, Midland, Presidio, Terrell, Terry, Val Verde

####### Locality.

Big Bend National Park, Big Bend Ranch State Park, Chisos Basin, Chisos Mountains

####### Caves.

**Terrell** (Bendele’s Uncave); **Val Verde** (Oriente Milestone Molasses Bat Cave, Seminole Sink)

####### Time of activity.

Male (March – June, September – October); female (March, May, July, September – October)

####### Habitat.

(landscape features: cave, under [rock, rocks on trail]); (structures: in house)

####### Type.

Texas (male, Terrell Co., Sanderson, May 26, 1952, W. J. Gertsch, holotype, AMNH)

####### Etymology.

Latin, flattering

####### Collection.

MSU, NMSU, TAMU, TMM

###### Loxosceles devia

Taxon classificationAnimaliaAraneaeSicariidae

Gertsch & Mulaik, 1940

Loxosceles devia Gertsch 1958b: 11, mf (figs 7–8, 12–14, 16–20, 24–26); Gertsch and Ennik 1983: 289, 339, mf, desc. (figs 1–7, 12–15, 28–31, 42–46); Jackman 1997: 35, 168; Reddell 1965: 173; Taber and Fleenor 2003: 234; Vetter 2008: 152; Vetter 2009: 519; Vetter 2015: 75, 78, 83; Vogel 1970b: 21
Loxosceles devius Gertsch and Mulaik, 1940; Gertsch and Mulaik 1940: 316, mf, desc; Vogel 1967: 122
Loxosceles arizonicus Gertsch and Mulaik, 1940; Gertsch and Mulaik 1940: 317 [Hidalgo Co. record]; Vogel 1970b: 21 [South Texas record]

####### Distribution.

Central and south Texas; Bexar, Brewster, Brooks, Cameron, Frio, Hidalgo, Jim Wells, Kenedy, Kerr, McLennan, Nueces, Real, San Augustine, San Patricio, Starr, Terrell, Uvalde, Webb, Wilson, Zapata

####### Locality.

Bentsen-Rio Grande Valley State Park, Green Island Bird Refuge, La Mesa Ranch, Laguna Madre, Lake Corpus Christi, Raven Ranch, Sabal Palm Audubon Sanctuary

####### Caves.

**Real** (Turkey Pens Cave); **Uvalde** (Tampke Ranch Cave)

####### Time of activity.

Male (February – June, August, September 25-October 2, October – December); female (January – August, October – December)

####### Habitat.

(grass: grass); (landscape features: cave, under [rock, rock in arroya bed]); (nest/prey: nest of *Neotoma micropus* [mf]); (objects: under board of dumpsite); (structures: on floor under box in bedroom); (soil/woodland: scrub forest)

####### Method.

pitfall trap [m]

####### Type.

Texas (male, Hidalgo Co., Edinburg, December 1933, S. Mulaik, holotype, AMNH)

####### Etymology.

Latin, distinct from *Loxosceles unicolor* Keyserling, 1887 (nomen dubium)

####### Collection.

MCZ, NMSU, TAMU, TMM

###### Loxosceles reclusa

Taxon classificationAnimaliaAraneaeSicariidae

Gertsch & Mulaik, 1940

Loxosceles reclusa Agnew et al. 1985: 6; Brown 1974: 234; Cokendolpher and Reddell 2001b: 53; Cokendolpher et al. 1979: 729; Gertsch 1958b: 7, mf, desc., (figs 4–6, 9–10, 21–23, 91–93); Gertsch and Ennik 1983: 285, mf, desc. (figs 8–11, 16, 20–23, 36–41); Henderson 2007: 64, 78, 81, 85; Horner 1967: 6; Horner and Stewart 1967: 334; Jackman 1997: 34, desc., 168 (photo 7a); Kaston 1972: 87, desc. (fig. 199); Kaston 1978: 89, desc. (fig. 217); Reddell 1970: 406; Reddell and Cokendolpher 2004: 91; Roberts 2001: 48; Sandidge and Hopwood 2005: 101; Taber and Fleenor 2003: 233; Tugmon et al. 1990: 43; Vetter 2005: 514; Vetter 2008: 152; Vetter 2009: 519; Vetter 2015: 74–75, 78; Vogel 1970b: 21; Yantis 2005: 67, 197, 201
Loxosceles rufipes (Lucas, 1834); Jones 1936: 69 [Texas record]
Loxosceles reclusus Gertsch and Mulaik, 1940; Gertsch and Mulaik 1940: 317, mf, desc
Loxosceles reculsus Gertsch and Mulaik, 1940; Vogel 1967: 122

####### Distribution.

Widespread (not south or west Texas); Anderson, Archer, Bastrop, Bell, Bexar, Bowie, Brazos, Burnet, Cooke, Dallas, Denton, Erath, Grayson, Hamilton, Harrison, Hill, Houston, Jack, Leon, Llano, Lubbock, McLennan, Montague, Palo Pinto, Polk, Potter, Robertson, Shelby, Tarrant, Throckmorton, Tom Green, Travis, Uvalde, Wichita, Wilson, Wise, Young

####### Locality.

Buescher State Park, Fort Hood, Holmes Pecan Orchard, Lick Creek Park, Texas A&M University Rangeland Area, Wildcat Bluff Nature Center

####### Caves.

**Bell** (Seven Cave [Fort Hood]); **Bexar** (Roan’s Cave); **Uvalde** (Tampke Ranch Cave)

####### Time of activity.

Male (February – December); female (January, March – October, December)

####### Habitat.

(landscape features: cave); (objects: in stacks of wood or posts, outdoors under sacks, under [board, corrugated metal, rocks]); (soil/woodland: in decaying logs, pine woods [%: 69, 82, 88, 99], post oak woods [%: 77, 80, 82, 85, 90], under [bark, log], upland woods, woods); (structures: building, closet, corner of apartment, garages and closets of homes, in house, lumber yard, under miscellaneous rubbish in old barns and sheds, warehouse)

####### Method.

5 gallon bucket trap [mf]; pitfall trap [mf]

####### Type.

Texas (male, Travis Co., Austin, September 1909, no collector, holotype, AMNH)

####### Etymology.

Latin, hide

####### Collection.

DMNS, JCC, MCZ, MSU, TAMU, TMM

###### Loxosceles rufescens

Taxon classificationAnimaliaAraneaeSicariidae

(Dufour, 1820)

Loxosceles rufescens Gertsch 1958b: 31, mf, desc. (figs 60–62, 73); Gertsch 1967: 128; Gertsch and Ennik 1983: 353, mf, desc. (figs 341–343, 348–351); Jackman 1997: 35, 168; Jones 1936: 69; Petrunkevitch 1911: 118

####### Distribution.

Dallas, Galveston, Harris, Lubbock

####### Time of activity.

Female (July – August)

####### Habitat.

(structures: in building)

####### Type.

Spain, Valencia Province, Sagunto

####### Etymology.

Latin, reddish-brown

##### Family Sparassidae Bertkau, 1872

**Note.** transferred from Heteropodidae (Jäger 1999)

##### Genus *Curicaberis* Rheims, 2015

###### Curicaberis ferrugineus

Taxon classificationAnimaliaAraneaeSalticidae

(C. L. Koch, 1836)

Curicaberis ferrugineus Rheims 2015: 424, mf, desc. (figs 51–54)

####### Distribution.

Cameron

####### Time of activity.

Male (February); female (April)

####### Habitat.

(soil/woodland: palm grove)

####### Type.

Mexico, Veracruz, Pico de Orizaba

####### Etymology.

Latin, rust colored, dusky

##### Genus *Heteropoda* Latreille, 1804

###### Heteropoda venatoria

Taxon classificationAnimaliaAraneaeSalticidae

(Linnaeus, 1767)

Heteropoda venatoria Bradley 2013: 212; Broussard and Horner 2006: 255; Gertsch 1979: 206; Jackman 1997: 199, desc.; Kaston 1972: 235, desc. (fig. 530); Kaston 1978: 226, desc. (fig. 577); Richman et al. 2011a: 49 [Jäger 2014: 147, mf, (fig. 1–26)]

####### Distribution.

Brewster, Cameron, Harris, Nueces, Presidio

####### Locality.

Chihuahuan desert, Dalquest Research Site, Houston Zoo

####### Time of activity.

Male (April, June, “September/October”); female (February, June)

####### Habitat.

(structures: in bathroom, inside house)

####### Method.

pitfall trap [m]

####### Eggs/spiderlings.

Nueces [received female June 28, 2004, eggsac hatch July 4–9, 191 spiderlings] [TAMU]

####### Type.

unknown

####### Etymology.

Latin, hunter

####### Collection.

MSU, TAMU

##### Genus *Olios* Walckenaer, 1837

**Note.** Species incorrectly reported from Texas

Olios fasciculatus Simon, 1880; Gertsch 1979: 206 (West Texas) [not native to Nearctic and mislabeled, Rheims 2010: 530]

###### Olios giganteus

Taxon classificationAnimaliaAraneaeSparassidae

Keyserling, 1884

Olios giganteus Bradley 2013: 212; Rheims 2010: 535, mf, desc. (figs 13–16, 20)

####### Distribution.

Cameron

####### Type.

New Mexico, Punta del Agua

####### Etymology.

Latin, size

#### Family Symphytognathidae Hickman, 1931

##### Genus *Anapistula* Gertsch, 1941

###### Anapistula secreta

Taxon classificationAnimaliaAraneaeSymphytognathidae

Gertsch, 1941

Anapistula secreta [Forster and Platnick 1977: 22, mf, desc. (figs 19, 57–61); Gertsch 1941a: 2, f, desc. (figs 14–17)]

####### Distribution.

Travis, Williamson

####### Caves.

**Travis** (Tooth Cave); **Williamson** (Electro-Mag Cave, Shell Cave)

####### Habitat.

(landscape features: cave)

####### Type.

Panama, Barro Colorado Island

####### Etymology.

Latin, secretive

####### Collection.

TMM

####### Note.

James Reddell (pers. comm.) stated that egg sacs are laid in irregular horizontal webs in small pockets in flowstone or rocks in total darkness with one egg per sac.

#### Family Tengellidae Dahl, 1908

##### Genus *Lauricius* Simon, 1888

**Note.** transferred from Clubionidae to Tengellidae (Brignoli 1983: 534) and to Zoropsidae (Polotow et al. 2015: 152)

##### Family Tetragnathidae Menge, 1866 Genus *Azilia* Keyserling, 1881

###### Azilia affinis

Taxon classificationAnimaliaAraneaeSymphytognathidae

O. P.-Cambridge, 1893

Azilia affinis Jackman 1997: 168; Levi 1980: 72, mf, desc. (figs 290–308); Levi 2005b: 234; Roth 1982: 11–3; Roth 1985: B-6–2, B-6–9; Roth 1994: 170

####### Distribution.

East and south Texas

####### Type.

Mexico, Tabasco, Teapa

####### Etymology.

Latin, allied to *Azilia guatemalensis* O. P.-Cambridge, 1889

##### Genus *Glenognatha* Simon, 1887

###### Glenognatha foxi

Taxon classificationAnimaliaAraneaeTetragnathidae

(McCook, 1894)

Glenognatha foxi Agnew et al. 1985: 3; Breene et al. 1993c: 25, 48, 107, mf (figs 167A-C); Bumroongsook et al. 1992: 17; Calixto et al. 2013: 184; Dean and Eger 1986: 141; Dean and Sterling 1985: 117; Dean and Sterling 1987: 6; Dean and Sterling 1990: 402, 404; Dean and Sterling 1992: 3–4; Dean et al. 1988: 286; Dondale et al. 2003: 48, mf, desc. (figs 36–43); Jackman 1997: 168; Levi 1980: 68 [S], mf, desc. (figs 272–284); Liao et al. 1984: 410; Young and Edwards 1990: 15
Mimognatha foxi McCook, 1894; Kagan 1942: 38; Kagan 1943: 258

####### Distribution.

Widespread; Brazos, Burleson, Cameron, Collin, Colorado, Coryell, Delta, Denton, Erath, Fort Bend, Hidalgo, Hill, Houston, Kaufman, McLennan, Nueces, Polk, Presidio, Robertson, San Patricio, Travis (imm.), Walker, Wharton, Wichita, Williamson

####### Locality.

5-Eagle Ranch, Attwater Prairie Chicken National Wildlife Refuge, Ellis Prison Unit, Holmes Pecan Orchard, NK Ranch

####### Time of activity.

Male (January – December); female (February – December)

####### Habitat.

(crops: cotton, peanuts); (grass: grass, grassland, pasture); (orchard: pecan); (plants: bluebonnets, Indian paintbrush, miscellaneous vegetation, *Baccharis*); (soil/woodland: post oak savanna with pasture, *Juniperus ashei*)

####### Method.

cardboard band [m]; D-Vac suction [mf]; fogging [mf]; pitfall trap [mf]; ramp trap [mf]; suction trap [mf]; sweeping [mf]; tile trap [mf]

####### Type.

unknown

####### Etymology.

Person

####### Collection.

MCZ, MSU, TAMU

##### Genus *Leucauge* White, 1841

###### Leucauge venusta

Taxon classificationAnimaliaAraneaeTetragnathidae

(Walckenaer, 1841)

Leucauge venusta Agnew et al. 1985: 7; Breene et al. 1993b: 648; Brown 1974: 232; Cokendolpher and Reddell 2001b: 54; Dean and Eger 1986: 141; Dean and Sterling 1990: 404; Dondale et al. 2003: 51, mf, desc. (figs 44–51); Jackman 1997: 66, desc., 168; Kaston 1953: 197, desc. (fig. 483); Kaston 1972: 141, desc. (fig. 315); Kaston 1978: 135, desc. (fig. 338); Levi 1980: 25 [T], mf, desc. (figs 44–59); Reddell 1970: 404; Reddell and Cokendolpher 2004: 91; Roth 1994: 170; Taber and Fleenor 2003: 236; Vogel 1970b: 5
Argyroepeira venusta (Walckenaer, 1841); McCook 1893: 242

####### Distribution.

Eastern ½ Texas; Archer, Bastrop, Bell, Brazos, Cameron, Comal, Edwards, Erath, Grimes, Hidalgo, Houston, Hunt, Montgomery, Nacogdoches, Red River, Travis, Upshur, Walker

####### Locality.

Buescher State Park, Ellis Prison Unit, Fort Hood, Lake Tawakoni State Park, Lick Creek Park, Sam Houston National Forest, Santa Ana National Wildlife Refuge, Stubblefield Lake

####### Caves.

**Bell** ([all Fort Hood] C. B. Cave, Keilman Cave, Violet Cave); **Comal** (Little Gem Cave No. 1); **Edwards** (Devil’s Sinkhole); **Travis** (La Crosse Cave [questionable])

####### Time of activity.

Male (April); female (April – November)

####### Habitat.

(crops: sugarcane); (grass: grasses, pasture); (landscape features: cave); (littoral: creek bank); (nest/prey: mud dauber nest [f] *Chalybion californicum*); (plants: Indian paintbrush); (soil/woodland: oak, palm forest margin [resaca bank], trees/shrubs, *Quercus buckleyi*); (web: web between shrub)

####### Method.

Beating [mf]; beating/sweeping [f]; suction trap [f]; sweeping [mf]

####### Type.

Georgia

####### Etymology.

Latin, elegant or charming

####### Collection.

DMNS, MSU, TAMU, TMM

##### Genus *Metellina* Chamberlin & Ivie, 1941

###### Metellina mimetoides

Taxon classificationAnimaliaAraneaeTetragnathidae

Chamberlin & Ivie, 1941

Metellina mimetoides Bradley 2013: 218; Dondale et al. 2003: 109, mf, desc. (figs 235–239); Levi 1980: 36 [T], mf, desc. (figs 87–94)
Meta mimetoides Chamberlin and Ivie, 1941; Jackman 1997: 168
Meta sp.; Reddell 1965: 170; Reddell 1970: 404; Vogel 1970b: 5

####### Distribution.

North and southwest Texas; Bandera, Collingsworth, Hardeman, King, Medina, San Saba, Uvalde, Wheeler

####### Caves.

**Bandera** (Tucker’s Fissure, Garrison Hilltop Cave); **Collingsworth** (Bumpas Cave); **Hardeman** (Walkup Cave); **King** (River Styx Cave); **Medina** (Davenport Cave); **San Saba** (Davenport Cave [questionable], Wedge Cave [questionable]); **Uvalde** (Tampke Ranch Cave); **Wheeler** (Big Mouth Cave)

####### Habitat.

(landscape features: cave)

####### Type.

California, Mount Diablo

####### Etymology.

Greek-Latin, mimic-like

####### Collection.

TMM

##### Genus *Pachygnatha* Sundevall, 1823

###### Pachygnatha autumnalis

Taxon classificationAnimaliaAraneaeTetragnathidae

Marx, 1884

Pachygnatha autumnalis [Levi 1980: 58, mf, desc. (figs 155, 158–159, 202–213)]

####### Distribution.

Colorado

####### Locality.

Attwater Prairie Chicken National Wildlife Refuge

####### Time of activity.

Male (May, August)

####### Habitat.

(soil/woodland: post oak savanna with pasture)

####### Method.

pitfall trap [m]

####### Type.

Pennsylvania, Harrisburg

####### Etymology.

collected in autumn (November)

####### Collection.

TAMU

###### Pachygnatha tristriata

Taxon classificationAnimaliaAraneaeTetragnathidae

C. L. Koch, 1845

Pachygnatha tristriata Dondale et al. 2003: 98, mf, desc. (figs 195–204); Henderson 2007: 54, 61–63, 78, 81, 85; Jackman 1997: 168; Kaston 1953: 198, desc. (fig. 485); Kaston 1972: 168, desc. (fig. 371); Kaston 1978: 161, desc. (fig. 400); Levi 1980: 60 [S], mf, desc. (figs 238–250); McCook 1893: 270; Petrunkevitch 1911: 384

####### Distribution.

Southeast Texas; Brazos, Montgomery

####### Locality.

Lick Creek Park

####### Time of activity.

Male (June); female (April, May 27-June 15, June)

####### Habitat.

(littoral: near river); (soil/woodland: disturbed habitat, post oak woodland, upland woods)

####### Method.

pitfall trap [mf]

####### Type.

Pennsylvania

####### Etymology.

Greek, for three stripes

####### Collection.

MCZ, TAMU

##### Genus *Tetragnatha* Latreille, 1804

###### Tetragnatha caudata

Taxon classificationAnimaliaAraneaeTetragnathidae

Emerton, 1884

Tetragnatha caudata Jackman 1997: 168; Levi 1981a: 310 [T], mf, desc. (figs 140–148)
Eucta lacerta (Walckenaer, 1837); McCook 1893: 266
Eucta caudata Emerton, 1884; Marx 1890: 552

####### Distribution.

North Texas; Jefferson

####### Time of activity.

Male (July)

####### Type.

Massachusetts, Malden

####### Etymology.

Latin, shape of abdomen (tail)

####### Collection.

MCZ

####### Note.

Dondale et al. 2003 (page 60) labels map 6 as *caudata* but is actually *Tetragnatha pallescens* (see map 8).

###### Tetragnatha elongata

Taxon classificationAnimaliaAraneaeTetragnathidae

Walckenaer, 1841

Tetragnatha elongata Agnew et al. 1985: 7; Brown 1974: 237; Dondale et al. 2003: 83, mf, desc. (figs 144–156); Jackman 1997: 168; Jones 1936: 70; Levi 1981a: 300, mf, desc. (figs 74–89); McKenzie and Reddell 1964: 7; Reddell 1965: 170; Seeley 1928: 109; Vogel 1970b: 21

####### Distribution.

Eastern ½ Texas; Archer, Bell, Brown, Burleson, Clay, Comanche, Dallas, Erath, Harrison, Kerr, Lee, Nacogdoches (imm.), Travis, Wichita

####### Locality.

Proctor Lake

####### Caves.

**Bell** (Adam’s Gold Mine)

####### Time of activity.

Male (March – June, October – November); female (March – May, August – September)

####### Habitat.

(grass: grass); (landscape features: cave); (littoral: brush pile by creek); (web: web in grass by creek)

####### Method.

sweeping [m]

####### Type.

Carolina’s (of 1841)

####### Etymology.

Latin, long jaws

####### Collection.

DMNS, MSU, TAMU, TMM

###### Tetragnatha extensa

Taxon classificationAnimaliaAraneaeTetragnathidae

(Linnaeus, 1758)

Tetragnatha extensa [Levi 1981a: 298, mf, desc. (figs 56–64)]

####### Distribution.

Jack, Kerr

####### Type.

Sweden

####### Etymology.

Latin, stretched out

####### Collection.

MSU

###### Tetragnatha guatemalensis

Taxon classificationAnimaliaAraneaeTetragnathidae

O. P.-Cambridge, 1889

Tetragnatha guatemalensis Agnew et al. 1985: 7; Bradley 2013: 220; Dondale et al. 2003: 81, mf, desc. (figs 134–143); Guarisco 2008b: 5; Jackman 1997: 168; Jackman et al. 2007: 199; Knutson et al. 2010: 516; Levi 1981a: 296 [S], mf, desc. (figs 46–55); Rapp 1984: 5; Rice 1986: 124
Tetragnatha seneca Seeley, 1928; Brown 1974: 237
Tetragnatha laudativa Gertsch and Mulaik, 1936; Bonnet 1959: 4337; Gertsch and Mulaik 1936b: 15, mf, desc. (figs 33–35); Roewer 1942: 993; Vogel 1970b: 22

####### Distribution.

North-central and south Texas; Archer, Cameron, Clay, Comanche, Dallas, Galveston, Hidalgo, Hood, Howard, Hunt, Kerr, Kleberg, Lee, Limestone, Nacogdoches, San Patricio, Travis, Wharton, Wichita

####### Locality.

Arkansas Bend Park, Frontera Audubon, Galveston Island State Park, Lake Corpus Christi State Park, Lake Limestone, Lake Rayburn, Lake Somerville State Park [Nails Creek Unit], Lake Tawakoni State Park, Lakeside Park South, Proctor Lake, Sabal Palm Audubon Sanctuary, Starnes Island

####### Time of activity.

Male (June – December); female (March, June – December)

####### Habitat.

(grass: grass, grassy and shrub area); (littoral: salt marsh); (orchard: grapefruit, orange); (soil/woodland: saltcedar, under log); (structures: outside house); (web: large spider web, on communal web, trees overhanging town lake)

####### Method.

Beating [mf]; D-Vac suction [mf]

####### Type.

Guatemala

####### Etymology.

locality (country)

####### Collection.

MSU, NMSU, TAMU, TTU

###### Tetragnatha laboriosa

Taxon classificationAnimaliaAraneaeTetragnathidae

Hentz, 1850

Tetragnatha laboriosa Agnew et al. 1982: 631; Agnew et al. 1985: 4, 9; Bonnet 1959: 4335; Breene 1988: 23–26; Breene et al. 1988: 180–181; Breene et al. 1993a: 169; Breene et al. 1993c: 25, 48, 108, mf (figs 168A-C); Bumroongsook et al. 1992: 17; Calixto et al. 2013: 184, 190; Cokendolpher et al. 2008: 10, 52 (fig. 13, photo 36); Dean and Eger 1986: 141; Dean and Sterling 1985: 117; Dean and Sterling 1987: 6; Dean and Sterling 1990: 402, 404; Dean and Sterling 1992: 3–4; Dean et al. 1982: 254; Dean et al. 1987: 268; Dean et al. 1988: 286; Dondale et al. 2003: 74, mf, desc. (figs 106–114); Jackman 1997: 68, 168, desc. (photo 20d); Jones 1936: 70; Kagan 1942: 33; Kagan 1943: 258; Knutson and Gilstrap 1989: 514; Knutson et al. 2010: 515; Levi 1981a: 308, mf, desc. (figs 16–22, 120–128); Liao et al. 1984: 410; McCook 1893: 262; Milstead 1958: 445; Nyffeler and Sterling 1994: 1295, 1298; Nyffeler et al. 1987b: 1119; Nyffeler et al. 1987c: 372; Nyffeler et al. 1988a: 55; Nyffeler et al. 1989: 374, 377; Nyffeler et al. 1992b: 1459–1460; Nyffeler et al. 1992c: 2; Rapp 1984: 5; Roberts 2001: 48; Rogers and Horner 1977: 523; Seeley 1928: 123; Vogel 1970b: 22; Woods and Harrel 1976: 43; Young and Edwards 1990: 15

####### Distribution.

Widespread; Archer, Atascosa, Bastrop, Baylor, Bee, Bell, Bexar, Brazos, Briscoe, Brown, Burleson, Cameron, Carson, Castro (imm.), Clay, Collin, Colorado, Comanche, Coryell, Dallas, Delta, Erath, Fayette, Floyd, Fort Bend, Frio, Gaines, Galveston, Gillespie, Hale, Harris, Hidalgo, Hill, Hockley, Houston, Howard, Jefferson, Kaufman, Kerr, Lubbock (imm.), Martin (imm.), McLennan, Mitchell, Montague, Nueces, Pecos, Potter, Presidio, Reeves, Robertson, San Patricio, Scurry, Terrell, Terry, Tom Green, Travis, Victoria (imm.), Walker, Wharton, Wichita, Willacy, Williamson, Wood

####### Locality.

Big Bend Ranch State Park, Bill Haney Pecan Orchard, Blackstone Ranch, Ellis Prison Unit, Galveston Island State Park, Holmes Pecan Orchard, Lake Thomas, Lick Creek Park, Mansfield Dam Park, Pantex Lake, Proctor Lake, Santa Ana National Wildlife Refuge, Stiles Farm Foundation, Texas A&M University Rangeland Area, Wildcat Bluff Nature Center

####### Time of activity.

Male (February – October); female (February – November)

####### Habitat.

(crops: corn, cotton, guar, peanuts, rice); (grass: grass, grassland, grassy and shrub area, pasture); (littoral: creek bank, near falls, near playa, salt marsh); (nest/prey: stomach of *Cnemidophorus sacki*); (orchard: citrus, pecan); (plants: bluebonnets, clover, croton, emergent vegetation, geranium, Indian paintbrush, miscellaneous vegetation, roadside vegetation, vegetation, *Baccharis*, *Monarda citriodora*); (soil/woodland: brush, juniper, post oak savanna with pasture, saltcedar, shrub, willow, *Juniperus ashei*, *Quercus buckleyi*, *Ulmus crassifolia*); (structures: around house, in camper)

####### Method.

Beating [mf]; beating/sweeping [mf]; boll weevil pheromone trap [mf]; D-Vac suction [mf]; fogging [m]; pitfall trap [mf]; suction trap [mf]; sweeping [mf]

####### Type.

United States

####### Etymology.

Latin, toiling

####### Collection.

DMNS, MSU, NMSU, TAMU

###### Tetragnatha nitens

Taxon classificationAnimaliaAraneaeTetragnathidae

(Audouin, 1826)

Tetragnatha nitens Jackman 1997: 168; Levi 1981a: 291, mf, desc. (figs 23–34)

####### Distribution.

Baylor, Clay, Hidalgo, Hudspeth, Travis, Wichita

####### Locality.

Indio Mountains

####### Time of activity.

Male (June); female (August)

####### Habitat.

(plants: vegetation)

####### Type.

Egypt

####### Etymology.

Latin, glittering

####### Collection.

DMNS, MSU, NMSU

###### Tetragnatha pallescens

Taxon classificationAnimaliaAraneaeTetragnathidae

F. O. P.-Cambridge, 1903

Tetragnatha pallescens Armstrong and Richman 2007: 396; Dondale et al. 2003: 64, mf, desc. (figs 70–78); Jackman 1997: 168; Jones 1936: 69; Levi 1981a: 308 [S, T], mf, desc. (figs 129–139); Rapp 1984: 5; Seeley 1928: 131; Vogel 1970b: 22
Eugnatha pallida (Banks, 1892); McCook 1893: 265
Eugnatha pallescens (F. O. P.-Cambridge, 1903); Petrunkevitch 1911: 340

####### Distribution.

Eastern ½ Texas; Bexar, Brown, Cameron, Dallas, Fannin, Galveston, Titus, Victoria, Wichita

####### Locality.

Galveston Island State Park, Russell Farm, South Padre Island

####### Time of activity.

Male (May, July – September); female (June, September – October)

####### Habitat.

(grass: grass, grassland, grassy and shrub area); (littoral: salt marsh); (plants: miscellaneous vegetation)

####### Method.

Boll weevil pheromone trap [m]; sweeping [m]

####### Type.

New York, Ithaca

####### Etymology.

Latin, pale

####### Collection.

MSU, NMSU, TAMU

###### Tetragnatha straminea

Taxon classificationAnimaliaAraneaeTetragnathidae

Emerton, 1884

Tetragnatha straminea Dondale et al. 2003: 67, mf, desc. (figs 79–87); Jackman 1997: 168; Kaston 1953: 201, desc. (fig. 493); Kaston 1972: 172, desc. (fig. 379); Kaston 1978: 163, desc. (fig. 408); Levi 1981a: 312, mf, desc. (figs 149–157); Woods and Harrel 1976: 43; Young and Edwards 1990: 15

####### Distribution.

Northeast Texas; Dallas, Jefferson

####### Habitat.

(crops: rice)

####### Type.

Connecticut, New Haven

####### Etymology.

Latin, swollen

####### Collection.

MCZ

###### Tetragnatha vermiformis

Taxon classificationAnimaliaAraneaeTetragnathidae

Emerton, 1884

Tetragnatha vermiformis Jackman 1997: 168 [Levi 1981a: 316, mf, desc. (figs 176–184)]

####### Distribution.

Wichita

####### Time of activity.

Male (July)

####### Habitat.

(littoral: vegetation near water)

####### Type.

Massachusetts, Beverly

####### Etymology.

Latin, worm-like

####### Collection.

MSU

###### Tetragnatha versicolor

Taxon classificationAnimaliaAraneaeTetragnathidae

Walckenaer, 1841

Tetragnatha versicolor Agnew et al. 1985: 7; Dondale et al. 2003: 76, mf, desc. (figs 115–123); Jackman 1997: 168; Levi 1981a: 302 [S], mf, desc. (figs 90–109); Vogel 1970b: 22; Woods and Harrel 1976: 43; Young and Edwards 1990: 15
Tetragnatha extensa Linnaeus, 1758; Jones 1936: 70; McCook 1889: 155; Seeley 1928: 113; Vogel 1970b: 21 [Texas records]
Tetragnatha limnocharis (Seeley, 1928); Jones 1936: 69; Vogel 1970b: 22

####### Distribution.

North-central and central Texas; Brown, Comanche, Dallas, Houston, Jack, Jefferson, Travis, Wichita

####### Locality.

Proctor Lake

####### Time of activity.

Male (June – August)

####### Habitat.

(crops: rice); (plants: miscellaneous vegetation); (soil/woodland: cottonwood, willow)

####### Method.

sweeping [m]

####### Type.

Georgia

####### Etymology.

Latin, changed color

####### Collection.

DMNS, MSU, TAMU

###### Tetragnatha viridis

Taxon classificationAnimaliaAraneaeTetragnathidae

Walckenaer, 1841

Tetragnatha viridis Dondale et al. 2003: 78, mf, desc. (figs 124–133); Jackman 1997: 168; Levi 1981a: 304, mf, desc. (figs 110–119)

####### Distribution.

Harris

####### Habitat.

(soil/woodland: pine)

####### Type.

Georgia

####### Etymology.

Latin, green

#### Family Theridiidae Sundevall, 1833

**Note.** Species incorrectly reported from Texas

*Hentziectypus conjuncta* (Gertsch & Mulaik, 1936) [not in Texas]

*Achaearanea conjuncta* (Gertsch and Mulaik, 1936); Vogel 1970b: 22

##### Genus *Anelosimus* Simon, 1891

###### Anelosimus studiosus

Taxon classificationAnimaliaAraneaeTheridiidae

(Hentz, 1850)

Anelosimus studiosus Agnarsson 2006: 505, mf (figs 49A–F, 50, 51); Agnew et al. 1985: 6; Bradley 2013: 223; Breene et al. 1993c: 26, 48, 56, mf (figs 20A-C); Dean and Sterling 1990: 404; Dean et al. 1982: 254; Jackman 1997: 53, desc., 169; Kaston 1978: 109, desc.; Levi 1956b: 418, mf, desc. (figs 21–23, 37–39); Vogel 1970b: 22; Young and Edwards 1990: 23

####### Distribution.

Aransas, Archer, Bee, Brazos, Cameron, Comanche, Coryell, Dallas, Denton, Erath, Hidalgo, Jefferson, Jim Wells, Kleberg, La Salle, Liberty, Montague, Newton, Nueces, Travis, Walker, Zapata

####### Locality.

Ellis Prison Unit, Frontera Audubon, Goose Island State Park, Proctor Lake, Texas A&M University Rangeland Area

####### Time of activity.

Male (March – September); female (March – December)

####### Habitat.

(crops: cotton); (orchard: orange, sour orange, Valley lemon); (soil/woodland: juniper, shrub, trees/shrubs, willow, yaupon holly, *Quercus buckleyi*, *Quercus virginiana*, *Ulmus crassifolia*)

####### Method.

Beating [m]; boll weevil pheromone trap [mf]; suction trap [mf]; sweeping [m]

####### Type.

Alabama

####### Etymology.

Latin, eager

####### Collection.

MCZ, MSU, TAMU

##### Genus *Argyrodes* Simon, 1864

###### Argyrodes elevatus

Taxon classificationAnimaliaAraneaeTheridiidae

Taczanowski, 1873

Argyrodes elevatus Agnarsson 2004: 513; Agnew et al. 1985: 6; Dean and Sterling 1990: 404; Exline and Levi 1962: 134, mf, desc. (figs 128–132, 154); Jackman 1997: 169; Jackman et al. 2007: 199; Knutson et al. 2010: 516; Nyffeler et al. 1987c: 370; Vogel 1970b: 22

####### Distribution.

Archer, Bastrop, Brazos, Burleson, Cameron, Comal, Comanche, Denton, DeWitt, Erath, Hidalgo, Hood, Houston, Howard, Hunt, Navarro, San Patricio, Starr, Travis, Walker

####### Locality.

Bill Haney Pecan Orchard, Ellis Prison Unit, Lake Dallas, Lake Tawakoni State Park, Lick Creek Park, Texas A&M University Rangeland Area

####### Time of activity.

Male (April – September); female (April, June – September)

####### Habitat.

(crops: cotton); (grass: pasture); (orchard: pecan); (plants: miscellaneous vegetation, vegetation); (soil/woodland: saltcedar, shrubs, woods); (web: *Araneus* sp. web [mf], *Araneus bicentenarius* web [mf], *Argiope aurantia* web, bowl and doily web [f], *Neoscona crucifera* web [f], large spider web)

####### Method.

Beating [mf]; fogging [f]; suction trap [m]; sweeping [mf]

####### Type.

French Guiana, Uassa

####### Etymology.

Latin, elevated

####### Collection.

DMNS, MSU, TAMU

###### Argyrodes pluto

Taxon classificationAnimaliaAraneaeTheridiidae

Banks, 1906

Argyrodes pluto Exline and Levi 1962: 143, mf, desc. (figs 138–142); Jackman 1997: 169; Levi and Randolph 1975: 36; Vogel 1970b: 23

####### Distribution.

Brewster, Travis

####### Locality.

Chisos Mountains

####### Type.

Virginia, Falls Church

####### Etymology.

Greek, god of the underworld

##### Genus *Asagena* Sundevall, 1833

###### Asagena americana

Taxon classificationAnimaliaAraneaeTheridiidae

Emerton, 1882

Asagena americana Wunderlich 2008: 199 [T]
Steatoda americana (Emerton, 1882); Agnew et al. 1985: 6; Brown 1974: 238; Henderson 2007: 54, 75, 78, 81, 85; Jackman 1997: 59, desc., 169; Levi 1957b: 400, mf, desc. (figs 66–69); Levi and Randolph 1975: 40; Vogel 1970b: 24; Yantis 2005: 198

####### Distribution.

Baylor, Bexar, Brazos, Erath, Leon, Nacogdoches, Titus, Travis, Wichita

####### Locality.

Lick Creek Park, Texas A&M University Rangeland Area

####### Time of activity.

Male (March 15-April 15, April – May, July – August); female (July)

####### Habitat.

(littoral: near pond, pond, sedge meadow); (nest/prey: mud dauber nest [f]); (soil/woodland: post oak woods [%: 82], post oak woodland, woods)

####### Method.

5 gallon bucket trap [m]; pitfall trap [m] (near pond [m])

####### Type.

Massachusetts, Boston

####### Etymology.

locality (country)

####### Collection.

DMNS, MSU, TAMU

###### Asagena fulva

Taxon classificationAnimaliaAraneaeTheridiidae

(Keyserling, 1884)

Asagena fulva Wunderlich 2008: 199 [T]
Lithyphantes fulvus Keyserling, 1884; Comstock 1912: 362; Comstock 1940: 377, desc.; Fox 1940: 41; Marx 1890: 522; Milstead 1958: 446
Steatoda fulva (Keyserling, 1884); Agnew et al. 1985: 3; Gertsch 1960b: 45, mf, desc. (figs 62, 64–65, 70–71); Jackman 1997: 169; Kaston 1972: 119, desc.; Kaston 1978: 115, desc.; Levi 1957b: 391 [T], mf, desc. (figs 32–33, 45–47, 52); Levi and Randolph 1975: 41; Vogel 1970b: 24; Young and Edwards 1990: 23

####### Distribution.

Brewster, Brown, Colorado, Comanche, Culberson, Dallam, El Paso, Erath, Hidalgo, Howard, Hudspeth, Knox, Llano, Martin, Somervell, Starr, Wichita

####### Locality.

Black Gap Wildlife Management Area

####### Time of activity.

Male (March, July – September); female (July, September)

####### Habitat.

(crops: cotton, peanuts); (grass: grass); (nest/prey: stomach of *Cnemidophorus tigris*)

####### Method.

pitfall trap [mf]

####### Type.

Utah, Spring Lake

####### Etymology.

Latin, tawny-yellow

####### Collection.

MSU, TAMU

##### Genus *Chrosiothes* Simon, 1894

###### Chrosiothes jocosus

Taxon classificationAnimaliaAraneaeTheridiidae

(Gertsch & Davis, 1936)

Chrosiothes jocosus Jackman 1997: 169; Levi 1964d: 82 [T]; Levi and Randolph 1975: 37
Dipoena jocosa Gertsch and Davis, 1936; Bonnet 1956: 1507; Gertsch and Davis 1936: 7, mf, desc. (fig. 20); Roewer 1942: 424
Theridiotis jocosa (Gertsch and Davis, 1936); Levi 1954a: 180 [T], mf, desc. (figs 1–5, 10, 19, 26–27)
Chrosiothes jocosa (Gertsch and Davis, 1936); Vogel 1970b: 23

####### Distribution.

Cameron, Hidalgo, Llano, Starr, Travis, Uvalde

####### Locality.

Falcon State Park, Garner State Park

####### Time of activity.

Male (January, June – August); female (January, March – April, July – August, October, December)

####### Habitat.

(soil/woodland: *Juniperus ashei*)

####### Method.

Beating [m]

####### Type.

Texas (male, Travis Co., Austin, August 1935, L. I. Davis, holotype, AMNH)

####### Etymology.

Latin, full of fun

####### Collection.

TAMU

###### Chrosiothes minusculus

Taxon classificationAnimaliaAraneaeTheridiidae

(Gertsch, 1936)

Chrosiothes minusculus Breene et al. 1993b: 648; Jackman 1997: 169; Levi 1964d: 82 [T]; Levi and Randolph 1975: 37; Vogel 1970b: 23
Episinus minusculus Gertsch, 1936; Bonnet 1956: 1721; Gertsch 1936: 9, m, desc. (fig. 9); Roewer 1942: 450
Theridiotis minuscula (Gertsch, 1936); Levi 1954a: 182 [T], mf, desc. (figs 11, 16–18, 21, 28–29)

####### Distribution.

Cameron, Hidalgo

####### Locality.

Big Tree-Vine Association

####### Time of activity.

Male (February – March, September); female (July, September)

####### Habitat.

(crops: sugarcane)

####### Type.

Texas (male, Hidalgo Co., 5 miles S San Juan, February 22, 1935, S. Mulaik, holotype, AMNH)

####### Etymology.

Latin, small size

####### Collection.

TAMU

##### Genus *Chrysso* O. P.-Cambridge, 1882

###### Chrysso albomaculata

Taxon classificationAnimaliaAraneaeTheridiidae

O. P.-Cambridge, 1882

Chrysso albomaculata Jackman 1997: 169; Levi 1957c: 61 [T], mf, desc. (figs 1–4, 18–19, 25–27); Levi and Randolph 1975: 37; Vogel 1970b: 23
Steatoda albomaculata (O. P.-Cambridge, 1882); F. O. P.-Cambridge 1902: 385
Theridion albomaculatum (O. P.-Cambridge, 1882); Petrunkevitch 1911: 190; Roewer 1942: 501

####### Distribution.

Newton

####### Type.

Amazon

####### Etymology.

Latin, white spots on abdomen

##### Genus *Coleosoma* O. P.-Cambridge, 1882

###### Coleosoma acutiventer

Taxon classificationAnimaliaAraneaeTheridiidae

(Keyserling, 1884)

Coleosoma acutiventer Breene et al. 1993a: 169; Breene et al. 1993c: 26, 48, 60, mf (figs 30A-D); Dean and Sterling 1987: 6; Jackman 1997: 169; Levi 1959a: 4, mf, desc. (figs 6–11); Levi and Randolph 1975: 37; Vogel 1970b: 23

####### Distribution.

Cameron, Hidalgo, Wharton

####### Locality.

Big Tree-Vine Association

####### Time of activity.

Male (July, October)

####### Habitat.

(crops: cotton, sugarcane); (orchard: citrus)

####### Method.

D-Vac suction [m]

####### Type.

Peru

####### Etymology.

Latin, shape of abdomen

####### Collection.

TAMU

##### Genus *Crustulina* Menge, 1868

###### Crustulina altera

Taxon classificationAnimaliaAraneaeTheridiidae

Gertsch & Archer, 1942

Crustulina altera Jackman 1997: 169 [Levi 1957b: 372, mf, desc. (figs 4–6, 8–10)]

####### Distribution.

Gonzalez, Montague, Sabine, Tyler

####### Locality.

Big Thicket National Preserve

####### Time of activity.

Male (April); female (March, May, December)

####### Habitat.

(plants: vegetation); (soil/woodland: leaf litter)

####### Type.

Connecticut, Norwalk

####### Etymology.

Latin, alternate

####### Collection.

DMNS, MSU, TAMU

###### Crustulina sticta

Taxon classificationAnimaliaAraneaeTheridiidae

(O. P.-Cambridge, 1861)

Crustulina sticta Jackman 1997: 169; Levi 1957b: 370, mf, desc. (figs 1–3, 7); Levi and Randolph 1975: 37; Vogel 1970b: 23

####### Distribution.

Hidalgo, Matagorda

####### Type.

England

####### Etymology.

Greek, dappled

##### Genus *Cryptachaea* Archer, 1946

###### Cryptachaea canionis

Taxon classificationAnimaliaAraneaeTheridiidae

(Chamberlin & Gertsch, 1929)

Cryptachaea canionis Yoshida 2008: 39 [T]
Achaearanea canionis (Chamberlin and Gertsch, 1929) [Levi 1955a: 24, mf, desc. (figs 60–68)]

####### Distribution.

Culberson

####### Caves.

**Culberson** (Brooks Cave, Canyon Cave, Straight Cave)

####### Habitat.

(landscape features: cave)

####### Type.

Utah, Zion National Park

####### Etymology.

canyon

####### Collection.

TMM

###### Cryptachaea insulsa

Taxon classificationAnimaliaAraneaeTheridiidae

(Gertsch & Mulaik, 1936)

Cryptachaea insulsa Yoshida 2008: 39 [T]
Theridion insulsum Gertsch & Mulaik, 1936; Gertsch and Mulaik 1936b: 11, f, desc. (figs 25–26)
Theridium insulsum Gertsch & Mulaik, 1936; Bonnet 1959: 4483
Achaearanea insulsa (Gertsch & Mulaik, 1936); Jackman 1997: 168; Levi 1955a: 19 [T], mf, desc. (figs 41–45); Levi 1959b: 61; Levi 1963b: 192
Achaearanea insula (Gertsch & Mulaik, 1936); Levi and Randolph 1975: 36; Vogel 1970b: 22

####### Distribution.

Cameron, Hidalgo, Starr

####### Locality.

Big Tree-Vine Association, Santa Ana National Wildlife Refuge

####### Time of activity.

Male (October); female (February, April – May, September – November)

####### Eggs/spiderlings.

Hidalgo [16 spiderlings] [TAMU]

####### Type.

Texas (female, Cameron Co., Brownsville, November 30, 1934, S. Mulaik, holotype, AMNH)

####### Etymology.

Latin, boring

####### Collection.

TAMU

###### Cryptachaea porteri

Taxon classificationAnimaliaAraneaeTheridiidae

(Banks, 1896)

Cryptachaea porteri Bradley 2013: 225; Yoshida 2008: 39 [T]
Theridium porteri Banks, 1896; Banks 1910: 20
Theridion redemptum Gertsch and Mulaik, 1936; Gertsch and Mulaik 1936b: 13, mf, desc. (figs 14–15); Roewer 1942: 505
Theridium redemptum Gertsch and Mulaik, 1936; Bonnet 1959: 4518
Achaearanea porteri (Banks, 1896); Agnew et al. 1985: 6; Barr and Reddell 1967: 261; Brown 1974: 237; Cokendolpher and Reddell 2001b: 54; Jackman 1997: 52, desc., 168; Kaston 1978: 106, desc.; Kunath and Smith 1968: 19, 30; Levi 1955a: 30 [S], mf, desc. (figs 71–75, 80–82); Levi 1963b: 215; McKenzie and Reddell 1964: 15, 22; Rapp 1984: 3; Reddell 1964: 41; Reddell 1965: 176; Reddell 1967: 11, 15, 23, 27, 34, 50; Reddell 1970: 408; Reddell 1973: 29, 46; Reddell 1994: 6; Reddell and Cokendolpher 2004: 92; Reddell and Finch 1963: 28, 41, 48, 53, 54; Reddell and Smith 1965: 20, 33, 46, 61, 62, 66; Smith and Reddell 1971: 21, 24, 29, 41; Vogel 1970b: 22

####### Distribution.

Widespread in caves; Atascosa, Bandera, Bell, Bexar, Blanco, Brown, Burnet, Childress, Clay, Collingsworth, Comal, Comanche, Coryell, Crockett, Culberson, Denton, Edwards, Erath, Galveston, Hardeman, Harrison, Hays, Irion, Kendall, Kerr, Kimble, King, Kinney, Lampasas, Llano, Mason, Medina, Menard, Nacogdoches, Pecos, Randall, Real, San Saba, Schleicher, Stonewall, Sutton, Terrell, Travis, Uvalde, Val Verde, Wheeler, Wichita, Williamson

####### Locality.

Bill Haney Pecan Orchard, Camp Bullis, Fort Hood, Galveston Island State Park

####### Caves.

**Bandera** (Haby Swallow Cave, Keese Cave); **Bell** (Cub Cave [Fort Hood], Gnarla Cave [Fort Hood], Hill’s Cave, Lunch Counter Cave [Fort Hood], Nolan Creek Cave [Fort Hood], Rugger’s Rift Cave [Fort Hood], Sanford Pit Cave [Fort Hood], Streak Cave [Fort Hood]); **Bexar** (40 mm Cave, Assassin Cave, Banzai Mud Dauber Cave, Bear Cave, Boneyard Pit, Bunny Hole [Camp Bullis], Cave site #301, Cave site #306, Cave of the Bee Spirits, Charley’s Hammer Hole, Cross the Creek Cave, Dirtwater Cave, Dos Viboras Cave, Eagles Nest Cave, Goat Cave, Government Canyon Bat Cave, Hairy Tooth Cave, Headquarters Cave, Hogan’s Cave, Holy Smoke Cave, Isocow Cave, Isopit, Kamikazi Cricket Cave, Lithic Ridge Cave, Mattke Cave, Phil’s Friggin Line Cave [Cave, site #803], Poor Boy Baculum Cave, Porcupine Parlor Cave, Raging Cajun Cave, Rattlesnake Cave, Robber Baron Cave, Scorpion Cave, Stevens Ranch Cave No. 1, Strange Little Cave, Tall Tales Cave, Three Fingers Cave, Tin Pot, Unknown Cave, Up the Creek Cave, Valley of Death Cave, Well Done Cave, World Newt Cave, Wurzbach Bat Cave, Young Cave No. 1); **Blanco** (Davis Blowout Cave); **Burnet** (Beaver Creek Bat Cave, Duncan’s Flea Cave, Huber Mine, Longhorn Caverns, Nolan’s Cave, Pie Cave, Snelling’s Cave, Taylor Water Cave); **Childress** (Black Hand Cave); **Collingsworth** (Bumpas Cave, Turtle Cave); **Comal** (Kappelman Cave, Little Bear Creek Cave, Natural Bridge Caverns); **Coryell** (Fossil Spring Cave [Fort Hood], Oxygen Bottle Cave, Plateau Cave No. 2, Rocket River Cave System (Double Tree Cave) [Fort Hood], Saltpeter Cave [Fort Hood]); **Crockett** (Dudley Cave); **Culberson** (Dillahunty Swallow Cave, Gyp Joint, Plateau Cave); **Edwards** (Blue Elm Cave, Cueva de la Cola Blanca, Devil’s Sinkhole, Dunbar Cave, Green Cave, Hughes Cave, Jacoby Cave, Midnight Cave, 3-Bounce Pit, Wheat Cave, Wheat Cave No. 1, Wyatt Cave); **Hardeman** (Walkup Cave); **Hays** (Boggus Cave, Donaldson Cave, Ezell’s Cave, Fern Cave, Ladder Cave, McCarty Cave, McGlothlin Sink); **Irion** (Arden Cave); **Kendall** (Jan’s Fissure, Swaglet Cave); **Kerr** (East Trap Cave, Mingus Swallow Cave, Old Morris Cave, Pinto Ranch Cave, Seven Room Cave, Stowers Cave); **Kimble** (Flemming Bat Cave, Garter Snake Cave, The Hole, Live Dog Cave, Lizard Cave, Top Dog Cave); **King** (River Styx Cave); **Kinney** (Cot Cave, Kickapoo Caverns); **Lampasas** (Battery Cave, Dead Goat Cave, Jackson Flea Cave, Jackson One-Bat Cave); **Llano** (Enchanted Rock Cave, Miller’s Cave); **Mason** (Kothmann Cave, Zesch Ranch Cave); **Medina** (Boehme’s Cave, Davenport Cave, Haby Bat Cave, Lutz Cave, Ney Cave, Valdina Farms Sinkhole); **Menard** (Kearney’s Dead Goat Cave, Powell’s Cave); **Pecos** (Ess Cave); **Randall** (Catarina Cave, Confusion Cave); **Real** (Orell Bat Cave, Red Arrow Cave, Turkey Pens Cave); **San Saba** (Bremer Cave, Chimneyer’s Delight Cave, Cicurina Cave, Fence Line Fissure, Wedge Cave); **Schleicher** (Cave Y); **Stonewall** (Aspermont Bat Cave); **Sutton** (Felton Cave, Silky Cave, Word Cave); **Terrell** (Blackstone Cave); **Travis** (Balcones Sink, Beckett’s Cave, Broken Lid Cave, Cave X, Cave Y, Central Sink, Cold Cave, Cotterell Cave, Dead Dog Cave No. 1, Deer Stand Cave, Driskill Cave, Feather Sink, Gallifer Cave, Get Down Cave, Goat Cave, Grove Sinks Cave, Jack’s Joint, Kretschmarr Double Pit, Kretschmarr Fluted Sink, LaCrosse Cave, Lost Gold Cave, McDonald Cave, Midden Sink, No Rent Cave, Outhouse Hole Sink, Pickle Pit, Salamander Cave, Schulze Cave, Seider Springs Cave, Singletary Cave, Slumberger Sink, Spanish Wells, Stark’s North Mine, Stoneworks Sink, Substations Sink, Tardus Hole, Three-Holer Cave, Tooth Cave, Weldon Cave, Weldon West Cave, Whirlpool Cave, Wildflower Cave); **Uvalde** (Big Foot Cave, Burial Cave, Cement Tank Cave, Crom Cave, Davy Crockett Cave, Frio Bat Cave, Frio King Cave, Maybe Stream Cave, North Well Cave, Pablo’s Cave, Picture Cave No. 1, Sandtleben Cave, Tampke Ranch Cave, Whitecotton Bat Cave); **Val Verde** (Fawcett’s Cave, Four-Mile Cave); **Wheeler** (Big Mouth Cave); **Williamson** (Beck Sewer Cave, Bev’s Grotto, Coon Scat Cave, Elm Bat Cave, Elm Cave, Formation Forest Cave, Good Friday Cave, Grimace Cave, Jug Cave, Ku Klux Klan Cave, Lorfing’s Unseen Rattler Cave, Man-With-A-Spear Cave, Marigold Cave, Sore-ped Cave, Steam Cave, Susana Cave, T.W.A.S. A Cave, Three-Mile Cave, Two Hole Cave, Walsh Ranch Cave, Williams Cave, Wolf Cave)

####### Time of activity.

Male (January – August, October – November); female (January – December)

####### Habitat.

(landscape features: cave); (littoral: salt marsh); (nest/prey: mud dauber nest [f]); (orchard: pecan); (soil/woodland: woods); (structures: bathroom, indoors)

####### Method.

Fogging [m]; pitfall trap [m] (in woods [m])

####### Type.

Indiana, Porter’s Cave

####### Etymology.

locality (name of Porter’s cave)

####### Collection.

MSU, TAMU, TMM

##### Genus *Dipoena* Thorell, 1869

###### Dipoena abdita

Taxon classificationAnimaliaAraneaeTheridiidae

Gertsch & Mulaik, 1936

Dipoena abdita Agnew et al. 1985: 6; Bonnet 1956: 1502; Breene et al. 1993a: 169; Breene et al. 1994: 8; Calixto et al. 2013: 184; Gertsch and Mulaik 1936b: 6, f, desc. (fig. 28); Jackman 1997: 169; Levi 1953: 37, mf, desc. (figs 77–82, 108–109); Reddell and Cokendolpher 2004: 93; Roewer 1942: 423; Vogel 1970b: 23

####### Distribution.

Bexar, Burleson, Cameron, Colorado, Comanche, Coryell, Erath, Hays, Hidalgo, Llano, Robertson, San Patricio, Starr

####### Locality.

Attwater Prairie Chicken National Wildlife Refuge, Bill Haney Pecan Orchard, Holmes Pecan Orchard

####### Caves.

**Bexar** (Firing Line 11 Cave)

####### Time of activity.

Male (May, June 20-July 2, July – October); female (January – February, April – July, October, December)

####### Habitat.

(crops: cotton, watermelon); (landscape features: cave); (orchard: citrus, pecan); (soil/woodland: post oak savanna with pasture)

####### Method.

pitfall trap [m]; suction trap [mf]

####### Type.

Texas (female, Hidalgo Co., Edinburg, December 7, 1935, S. Mulaik, holotype, AMNH)

####### Etymology.

Latin, hidden

####### Collection.

TAMU, TMM

###### Dipoena buccalis

Taxon classificationAnimaliaAraneaeTheridiidae

Keyserling, 1886

Dipoena buccalis [Levi 1953: 27, mf, desc. (figs 6, 16–18, 33–34, 98–101)]

####### Distribution.

Travis

####### Type.

“Philadelphia, Fortress Monroe and Atlantic City” collected by Marx

####### Etymology.

Latin, mouth or cheek

####### Collection.

DMNS

###### Dipoena cathedralis

Taxon classificationAnimaliaAraneaeTheridiidae

Levi, 1953

Dipoena cathedralis Jackman 1997: 169; Levi 1953: 15, m, desc. (figs 19–22); Levi and Randolph 1975: 37; Vogel 1967: 130

####### Distribution.

Brewster

####### Type.

Texas (male, Brewster Co., 25 miles S Alpine, no date, no collector, holotype, AMNH)

[female unknown]

####### Etymology.

referring to a cathedral

###### Dipoena nigra

Taxon classificationAnimaliaAraneaeTheridiidae

(Emerton, 1882)

Dipoena nigra Agnew et al. 1985: 6; Jackman 1997: 169; Levi 1953: 21, mf, desc. (figs 30–32, 37–46, 91–97); Vogel 1970b: 23

####### Distribution.

Brazos, Colorado, Erath, Travis

####### Locality.

Attwater Prairie Chicken National Wildlife Refuge, Lick Creek Park

####### Time of activity.

Male (March – June, September); female (May, July – August)

####### Habitat.

(soil/woodland: *Quercus buckleyi*, *Ulmus crassifolia*)

####### Method.

Beating/sweeping [f]; pitfall trap [m]; suction trap [m]; sweeping [mf]

####### Type.

Maine, Portland; Massachusetts, Beverly and Holyoke

####### Etymology.

Latin, color brown to black

####### Collection.

TAMU

##### Genus *Emertonella* Bryant, 1945

###### Emertonella taczanowskii

Taxon classificationAnimaliaAraneaeTheridiidae

(Keyserling, 1886)

Emertonella taczanowskii Yoshida 2002: 17 [T]
Euryopis taczanowskii (Keyserling, 1886); Jackman 1997: 169; Levi 1967: 178 [S], mf, desc. (figs 37–41); Levi and Randolph 1975: 39; Vogel 1970b: 24
Euryopis nigripes Banks, 1929; Levi 1954b: 24, mf, desc. (figs 38–52); Vogel 1970b: 23
Euryopis dentatus Gertsch and Mulaik, 1936; Gertsch and Mulaik 1936b: 6, mf, desc. (figs 10–11)
Euryopis dentata Gertsch and Mulaik, 1936; Bonnet 1956: 1821; Roewer 1942: 454

####### Distribution.

Hidalgo, Starr

####### Locality.

Frontera Audubon

####### Time of activity.

Male (September); female (September)

####### Habitat.

(orchard: grapefruit)

####### Type.

Peru, Tumbes

####### Etymology.

Person (arachnologist)

####### Collection.

TAMU

##### Genus *Enoplognatha* Pavesi, 1880

###### Enoplognatha caricis

Taxon classificationAnimaliaAraneaeTheridiidae

(Fickert, 1876)

Enoplognatha caricis Wunderlich 1976: 99 [S]
Enoplognatha tecta (Keyserling, 1884); Jackman 1997: 169; Levi 1957a: 13, 113, mf, desc. (figs 11, 25, 28–29, 34–37); Levi and Randolph 1975: 38; Vogel 1970b: 23

####### Distribution.

Travis

####### Type.

unknown

####### Etymology.

Latin, sedge

###### Enoplognatha marmorata

Taxon classificationAnimaliaAraneaeTheridiidae

(Hentz, 1850)

Enoplognatha marmorata Jackman 1997: 169; Levi 1957a: 11, 113, mf, desc. (figs 24, 26–27, 30–33); Levi and Randolph 1975: 38; Vogel 1970b: 23

####### Distribution.

Wilbarger

####### Type.

Alabama

####### Etymology.

Greek, of marble

##### Genus *Euryopis* Menge, 1868

###### Euryopis lineatipes

Taxon classificationAnimaliaAraneaeTheridiidae

O. P.-Cambridge, 1893

Euryopis lineatipes Jackman 1997: 169; Levi 1954b: 36, mf, desc. (figs 60, 73, 76, 90–91, 104, 125–126); Levi and Randolph 1975: 39; Vogel 1970b: 23

####### Distribution.

Cameron, Hidalgo, Presidio, San Patricio

####### Locality.

Lake Corpus Christi State Park, Santa Ana National Wildlife Refuge

####### Time of activity.

Male (August – September); female (November)

####### Habitat.

(grass: grass)

####### Method.

Beating [f]; pitfall trap [m]

####### Type.

Guatemala

####### Etymology.

Latin, black transverse lines

####### Collection.

TAMU

###### Euryopis mulaiki

Taxon classificationAnimaliaAraneaeTheridiidae

Levi, 1954

Euryopis mulaiki Jackman 1997: 169; Levi 1954b: 19, mf, desc. (figs 17–18, 27–28, 32); Levi and Randolph 1975: 38; Vogel 1967: 134; Vogel 1970b: 23

####### Distribution.

Kleberg

####### Time of activity.

Male (October)

####### Type.

Texas (male, Kleberg Co., Kingsville, October 1934, S. Mulaik, holotype, USNM)

####### Etymology.

Person (collector)

###### Euryopis quinquemaculata

Taxon classificationAnimaliaAraneaeTheridiidae

Banks, 1900

Euryopis quinquemaculata Agnew et al. 1985: 6; Jackman 1997: 169; Levi 1954b: 46 [S], f, desc. (figs 133–136); Levi 1963a: 131, mf, desc. (figs 11–16); Levi and Randolph 1975: 38; Vogel 1970b: 23
Mufila texana Bryant, 1949; Bryant 1949b: 67, m, desc. (figs 1a-b); Vogel 1967: 135
Euryopis bryantae Levi, 1954; Levi 1954b: 47, m (fig. 137); Vogel 1970b: 23

####### Distribution.

Comanche, Dallas, Erath, Kerr, Uvalde, Wichita

####### Locality.

Garner State Park, Nabor’s Lake

####### Time of activity.

Male (April – May, July); female (July, September)

####### Habitat.

(soil/woodland: woods); (structures: outside house)

####### Method.

pitfall trap [m]; sweeping [f]

####### Type.

Washington D. C.; Virginia, Falls Church

####### Etymology.

Latin, five white spots on abdomen

####### Collection.

MSU, TAMU

###### Euryopis spinigera

Taxon classificationAnimaliaAraneaeTheridiidae

O. P.-Cambridge, 1895

Euryopis spinigera Agnew et al. 1985: 6; Henderson 2007: 66–69, 71–72, 78, 81, 85; Jackman 1997: 169; Levi 1954b: 20 [S], mf, desc. (figs 23–24, 30, 33, 36); Levi and Randolph 1975: 39; Vogel 1970b: 23
Euryopis deridens Gertsch and Mulaik, 1936; Bonnet 1956: 1821; Gertsch and Mulaik 1936b: 7, f, desc. (fig. 13); Roewer 1942: 454

####### Distribution.

Bell, Bexar, Brazos, Cameron, Erath, Gonzalez, Hidalgo, Jeff Davis, Llano

####### Locality.

Bentsen-Rio Grande Valley State Park, Davis Mountains, Lick Creek Park, Sabal Palm Audubon Sanctuary, Santa Ana National Wildlife Refuge, South Padre Island

####### Time of activity.

Male (January – April, June – October, November 15-December 21, December); female (February – March, August 15-September 17, September 17-October 20, October, December)

####### Habitat.

(grass: grass); (littoral: dune); (soil/woodland: bottomland forest, disturbed habitat, leaf litter, next to woods, post oak woodland, upland woods)

####### Method.

Berlese funnel [mf]; D-Vac suction [m]; pitfall trap [mf]

####### Type.

Guatemala

####### Etymology.

Latin, spines on abdomen

####### Collection.

DMNS, MSU, TAMU

###### Euryopis texana

Taxon classificationAnimaliaAraneaeTheridiidae

Banks, 1908

Euryopis texana Agnew et al. 1985: 3, 9; Banks 1908: 207, m, desc.; Banks 1910: 22; Broussard and Horner 2006: 255; Calixto et al. 2013: 184; Jackman 1997: 169; Levi 1954b: 34, mf, desc. (figs 57–58, 72, 87–89, 103, 122–124); Levi and Randolph 1975: 39; Milstead 1958: 445; Petrunkevitch 1911: 178; Richman et al. 2011a: 46; Roewer 1942: 454; Vogel 1970b: 24; Young and Edwards 1990: 23

####### Distribution.

Bastrop, Brazos, Brewster, Burleson, Comal, Comanche, Erath, Gray, Hidalgo, Kerr, Nueces, Presidio, Randall, San Patricio, Scurry, Val Verde, Wheeler

####### Locality.

Bill Haney Pecan Orchard, Black Gap Wildlife Management Area, Chihuahuan desert, Dalquest Research Site, Lake Corpus Christi State Park, Lake Thomas, Seminole Canyon State Park

####### Time of activity.

Male (March, July – August); female (July – October)

####### Habitat.

(crops: peanuts); (grass: grass); (nest/prey: stomach of *Cnemidophorus perplexus*); (orchard: pecan); (plants: over grazed mixed prairie); (soil/woodland: saltcedar)

####### Method.

cardboard band [f]; pitfall trap [mf]

####### Type.

Texas (male, Brazos Co., no date, no collector, cotype, MCZ)

####### Etymology.

locality (state)

####### Collection.

DMNS, MSU, TAMU

##### Genus *Faiditus* Keyserling, 1884

###### Faiditus americanus

Taxon classificationAnimaliaAraneaeTheridiidae

(Taczanowski, 1874)

Faiditus americanus Agnarsson 2004: 478 [T]
Argyrodes americanus (Taczanowski, 1874); Exline and Levi 1962: 161 [S], mf, desc. (figs 236–247); Jackman 1997: 169; Levi and Randolph 1975: 36; Vogel 1970b: 22
Argyrodes argenteola O. P.-Cambridge, 1894; Petrunkevitch 1911: 166
Argyrodina argenteola (O. P.-Cambridge, 1894); Roewer 1942: 436

####### Distribution.

Cameron, Hidalgo

####### Type.

French Guiana, Uassa

####### Etymology.

locality (country)

###### Faiditus cancellatus

Taxon classificationAnimaliaAraneaeTheridiidae

(Hentz, 1850)

Faiditus cancellatus Agnarsson 2004: 479 [T]
Argyrodes cancellatus (Hentz, 1850); Exline and Levi 1962: 180 [S], mf, desc. (figs 323–336); Fox 1940: 39; Jackman 1997: 169; Levi and Randolph 1975: 36; Vogel 1970b: 22
Argyrodes larvatus Keyserling, 1884; Marx 1890: 524

####### Distribution.

Brazos, Colorado, Denton, Liberty, Robertson, San Augustine, Tyler, Walker, Wood

####### Locality.

Lick Creek Park, Sam Houston National Forest, Stubblefield Lake

####### Time of activity.

Male (April – June, August); female (March – April)

####### Habitat.

(soil/woodland: shrubs, tree)

####### Method.

Beating [mf]; beating/sweeping [f]

####### Type.

Alabama

####### Etymology.

Latin, grating or bars

####### Collection.

TAMU

###### Faiditus caudatus

Taxon classificationAnimaliaAraneaeTheridiidae

(Taczanowski, 1874)

Faiditus caudatus Agnarsson 2004: 479 [T]
Argyrodes caudatus (Taczanowski, 1874); Exline and Levi 1962: 176, mf, desc. (figs 300–322); Jackman 1997: 169; Levi and Randolph 1975: 36; Vogel 1970b: 22

####### Distribution.

Hidalgo

####### Time of activity.

Male (April, October)

####### Type.

French Guiana, Uassa

####### Etymology.

Latin, posterior abdomen

###### Faiditus davisi

Taxon classificationAnimaliaAraneaeTheridiidae

(Exline & Levi, 1962)

Faiditus davisi Agnarsson 2004: 479 [T]
Argyrodes davisi Exline and Levi, 1962; Exline and Levi 1962: 191, mf, desc. (figs 370–374); Jackman 1997: 169; Levi and Randolph 1975: 36; Vogel 1967: 128

####### Distribution.

Cameron

####### Locality.

Big Tree-Vine Association

####### Time of activity.

Male (September)

####### Type.

Texas (male, Cameron Co., Big Tree-Vine Association, September 1936, L. I. Davis, holotype, AMNH)

####### Etymology.

Person (The species is named after the collector, Exline and Levi 1962).

###### Faiditus globosus

Taxon classificationAnimaliaAraneaeTheridiidae

(Keyserling, 1884)

Faiditus globosus Agnarsson 2004: 479 [T]
Argyrodes globosus Keyserling, 1884; Exline and Levi 1962: 164, mf, desc. (figs 248–260); Jackman 1997: 169; Levi and Randolph 1975: 36; Vogel 1970b: 22

####### Distribution.

Tyler

####### Type.

Florida, Crescent City

####### Etymology.

Latin, round form

###### Faiditus subdolus

Taxon classificationAnimaliaAraneaeTheridiidae

(O. P.-Cambridge, 1898)

Faiditus subdolus Agnarsson 2004: 479 [T]
Argyrodes subdolus O. P.-Cambridge, 1898; Exline and Levi 1962: 190, mf, desc. (figs 365–369); Jackman 1997: 169; Levi and Randolph 1975: 36; Vogel 1970b: 23

####### Distribution.

Bell, Hidalgo, Leon, Sutton, Travis

####### Type.

Guatemala, near Guatemala, San Antonio

####### Etymology.

Latin, “below a trap”

##### Genus *Hentziectypus* Archer, 1946

###### Hentziectypus florendidus

Taxon classificationAnimaliaAraneaeTheridiidae

(Levi, 1959)

Hentziectypus florendidus Yoshida 2008: 38 [T]
Achaearanea florens O. P.-Cambridge, 1896; Levi 1955a: 15 [Texas record]
Achaearanea florendida Levi, 1959; Jackman 1997: 168; Levi 1959b: 65, mf, desc., syn. (figs 17, 20–21); Levi 1963b: 233; Levi and Randolph 1975: 35; Vogel 1970b: 22 [Texas records]

####### Distribution.

Hidalgo

####### Time of activity.

Male (April)

####### Type.

Panama

####### Etymology.

Latin, glittering

###### Hentziectypus globosus

Taxon classificationAnimaliaAraneaeTheridiidae

(Hentz, 1850)

Hentziectypus globosus Bradley 2013: 227; Yoshida 2008: 38 [T]
Achaearanea globosa (Hentz, 1850); Agnew et al. 1985: 6; Breene et al. 1993b: 648; Breene et al. 1993c: 25, 48, 58, mf (figs 26A-C); Bumroongsook et al. 1992: 17; Dean and Eger 1986: 141; Dean and Sterling 1987: 6; Dean and Sterling 1990: 401, 404; Dean et al. 1982: 254; Dean et al. 1988: 286; Jackman 1997: 52, desc., 168; Levi 1955a: 9, mf, desc. (figs 19–25); Levi 1963b: 203; Vogel 1970b: 22; Young and Edwards 1990: 23
Achaearanea globosus (Hentz, 1850); Kaston 1972: 109, desc. (fig. 242); Kaston 1978: 106, desc. (fig. 260)

####### Distribution.

Bexar, Brazos, Cameron, Erath, Gonzalez, Hidalgo, Houston, Montgomery, Nueces, Robertson, San Patricio, Uvalde, Walker, Willacy

####### Locality.

Ellis Prison Unit, Garner State Park, Holmes Pecan Orchard, Jones State Forest, Palmetto State Park, Sam Houston National Forest, Stubblefield Lake

####### Time of activity.

Male (April, June – November); female (April, June – November)

####### Habitat.

(crops: cotton, sugarcane); (grass: grassland, pasture); (orchard: pecan); (plants: Indian paintbrush)

####### Method.

D-Vac suction [m]; fogging [m]; suction trap [m]; sweeping [m]

####### Type.

Alabama

####### Etymology.

Latin, round form

####### Collection.

TAMU

###### Hentziectypus schullei

Taxon classificationAnimaliaAraneaeTheridiidae

(Gertsch & Mulaik, 1936)

Hentziectypus schullei Yoshida 2008: 38 [T]
Theridion schullei Gertsch and Mulaik, 1936; Gertsch and Mulaik 1936b: 15, f, desc. (fig. 22); Roewer 1942: 505
Theridium schullei Gertsch and Mulaik, 1936; Bonnet 1959: 4524
Achaearanea schullei (Gertsch and Mulaik, 1936); Breene et al. 1993a: 169; Breene et al. 1993b: 648; Jackman 1997: 168; Levi 1955a: 17 [S], mf, desc. (figs 32–38); Levi 1963b: 203; Levi and Randolph 1975: 36; Vogel 1970b: 22
Theridion credulum Gertsch and Davis, 1936; Gertsch and Davis 1936: 11, m, desc. (fig. 17); Roewer 1942: 502
Theridium credulum Gertsch and Davis, 1936; Bonnet 1959: 4463

####### Distribution.

Bell, Bexar, Cameron, Coryell, Dallas, Hays, Hidalgo, Llano, Starr

####### Time of activity.

Male (March, June, August); female (April – November)

####### Habitat.

(crops: sugarcane); (grass: grass); (orchard: citrus); (soil/woodland: in log, post oak savanna with pasture, woods)

####### Method.

pitfall trap [m]

####### Type.

Texas (female, Hidalgo Co., Edinburg, October 15, 1935, Schulle, holotype, AMNH)

####### Etymology.

Person (collector)

####### Collection.

TAMU

##### Genus *Latrodectus* Walckenaer, 1805

###### Latrodectus geometricus

Taxon classificationAnimaliaAraneaeTheridiidae

C. L. Koch, 1841

Latrodectus geometricus Brown et al. 2008: 960; Jackman 1997: 55, desc., 169 [Levi 1959c: 21, mf, desc. (figs 8–10, 25–28, 37, 39–51, 80–83); Levi 1967: 185, mf (figs 57–59)]

####### Distribution.

Aransas, Cameron, Hidalgo, Nueces, San Patricio

####### Time of activity.

Female (March, August, October – November)

####### Habitat.

(structures: in autos at Aransas Auto-Plex, eave of building, ice chest, in house, refinery equipment)

####### Type.

Colombia

####### Etymology.

Greek, land measuring

####### Collection.

TAMU

###### Latrodectus hesperus

Taxon classificationAnimaliaAraneaeTheridiidae

Chamberlin & Ivie, 1935

Latrodectus hesperus Cokendolpher 1993: 39–40; Cokendolpher et al. 2008: 11, 53 (fig. 14, photo 40–46); Jackman 1997: 55, desc., 169; Kaston 1970: 39 [S], mf, desc. (figs 5b, 8–11, 13e, f, h); Kaston 1972: 102, desc. (figs 231–233); Kaston 1978: 101, desc. (figs 249–251); Knutson et al. 2010: 516; Roberts 2001: 48; Zhang et al. 2004: 349
Latrodectus mactans hesperus Chamberlin and Ivie, 1935; Chamberlin and Ivie 1935a: 15, mf, desc. (figs 1, 4, 6–14, 21, 23–33); Keegan 1955: 148; Levi 1969: 72
Latrodectus mactans texanus Chamberlin and Ivie, 1935; Chamberlin and Ivie 1935a: 14, mf, desc. (figs 3, 15–18); Comstock 1940: 374; Keegan 1955: 148; Vogel 1970b: 24

####### Distribution.

Bastrop, Brewster, Carson, Culberson, Floyd, Garza, Howard, Johnson, Kent, Loving, Lubbock, Potter, Presidio

####### Locality.

Big Bend Ranch State Park, Wildcat Bluff Nature Center

####### Caves.

**Culberson** (Gully Cave, Jack Rabbit Cave)

####### Time of activity.

Female (August – September)

####### Habitat.

(grass: grassland); (landscape features: cave, sheltered rock face); (soil/woodland: saltcedar)

####### Method.

pitfall trap

####### Type.

Utah, Salt Lake City

####### Etymology.

Greek, western

####### Collection.

DMNS, JCC, NMSU, TAMU, TMM

###### Latrodectus mactans

Taxon classificationAnimaliaAraneaeTheridiidae

(Fabricius, 1775)

Latrodectus mactans Agnew et al. 1985: 3, 5; Breene and Sweet 1985: 332; Breene et al. 1993b: 648; Breene et al. 1993c: 27, 48, 56–57, mf (figs 23A-B, 24A-B); Brown 1974: 238; Calixto et al. 2013: 184; Cokendolpher 1993: 39; Dean and Sterling 1987: 6; Dean and Sterling 1990: 401, 404; Dean and Sterling 1992: 3–4; Dean et al. 1982: 254; Dean et al. 1988: 285–286; Gertsch 1939b: 24; Jackman 1997: 54, 56, desc., 169 (photo 18f); Jones 1936: 69; Kagan 1942: 17; Kagan 1943: 258; Kaston 1970: 37 [S], mf, desc. (figs 2, 3, 4a, 4c, 5, 12, 13a-d, g, i, k); Kaston 1972: 101, desc. (figs 225–227); Kaston 1978: 100, desc. (figs 243–245); Levi 1959c: 24, mf, desc. (figs 1, 5–7, 15, 19–21, 53–67, 72–79); Levi 1967: 185; Milstead 1958: 445; Nyffeler and Sterling 1994: 1295, 1298; Nyffeler et al. 1986: 200; Nyffeler et al. 1987c: 370; Nyffeler et al. 1988a: 55; Nyffeler et al. 1992a: 1181; Reddell 1964: 37; Reddell 1965: 177; Reddell 1967: 34, 54; Reddell 1970: 408; Reddell 1973: 41; Reddell and Cokendolpher 2004: 93; Rogers and Horner 1977: 523; Taber and Fleenor 2003: 237; Vogel 1970b: 24; Young and Edwards 1990: 23; Zhang et al. 2004: 349 [Levi 1967: 185, mf (figs 60–62)]
Latrodectus mactans mactans Fabricius, 1775; Chamberlin and Ivie 1935a: 13, mf, desc. (figs 2, 5, 19–20, 22); Keegan 1955: 148; Vogel 1970b: 24
Theridium lineamentum McCook, 1879; Banks 1910: 24

####### Distribution.

Widespread; Bailey, Bexar, Brazos, Brewster, Brown, Burnet, Cameron, Childress, Clay, Comal, Comanche, Coryell, Dallam, Dallas, Edwards, Erath, Frio, Hardeman, Harris, Hidalgo, Houston, Jack, Kimble, Lamar, Limestone, Lubbock, McLennan, Medina, Nacogdoches, Presidio, Randall, Robertson, San Jacinto, San Patricio, San Saba, Shelby, Stonewall, Sutton, Terrell, Travis, Walker, Webb, Wichita, Williamson, Wise

####### Locality.

Bentsen-Rio Grande Valley State Park, Bill Haney Pecan Orchard, Blackstone Ranch, Camp Arrowmoon, Chisos Basin, Chisos Mountains, Ellis Prison Unit, Holmes Pecan Orchard, Inks Lake State Park, La Mota Mountains, Lick Creek Park, Muleshoe National Wildlife Refuge, Stiles Farm Foundation, Welder Wildlife Refuge

####### Caves.

**Bexar** (Strange Little Cave); **Childress** (Black Hand Cave); **Comal** (Little Brehmmer-Heidrich Cave); **Edwards** (Punkin Cave); **Hardeman** (Short Cave); **Medina** (Ney Cave, Weynand Cave); **Randall** (Big Rock Cave); **San Saba** (Dove Cave); **Stonewall** (Aspermont Bat Cave)

####### Time of activity.

Male (March – September, November); female (February – December)

####### Habitat.

(crops: cotton, guar, peanuts, sugarcane); (grass: grassland); (landscape features: cave, under stones); (nest/prey: mud dauber nest [mf], stomach of *Cnemidophorus perplexus*, stomach of *Cnemidophorus sacki*, stomach of *Cnemidophorus tessellatus*); (orchard: pecan); (plants: vegetation); (soil/woodland: clay soil brushland, hackberry woodland, live oak woodland, post oak savanna, post oak savanna with pasture, sandy brushland, sandy open prairie, tree, *Quercus virginiana*); (structures: barn, base of building, roof, storeroom, under porch, top of cellar doorway near entrance, warehouse)

####### Method.

Beating [m]; beating/sweeping [m]; cardboard band [imm.]; D-Vac suction [m]; pitfall trap [mf]; suction trap [m]; sweeping [m]

####### Type.

America

####### Etymology.

Latin, unfavorable behavior, dangerous

####### Collection.

DMNS, JCC, MSU, TAMU, TMM, TTU

###### Latrodectus variolus

Taxon classificationAnimaliaAraneaeTheridiidae

Walckenaer, 1837

Latrodectus variolus Jackman 1997: 58, desc., 169; Kaston 1972: 101, desc. (figs 228–230); Kaston 1978: 101, desc. (figs 246–248); McCrone and Levi 1964: 13 (figs 3, 8–13) [Kaston 1970: 38 [S], mf, desc. (figs 4b, 5a, 6a-f, 7, 14a, b, f)]
Latrodectus curacaviensis (Muller, 1776); Levi 1959c: 38, mf, desc. (figs 2–4, 16–18, 35–36, 52, 68–71); Vogel 1970b: 24

####### Distribution.

Brewster, Hunt, Pecos, Starr, Terrell, Webb

####### Locality.

Chisos Basin, Chisos Mountains

####### Type.

Georgia

####### Etymology.

Latin, variable form

####### Collection.

MSU

##### Genus *Neopisinus* Marques, Buckup & Rodrigues, 2011

###### Neopisinus cognatus

Taxon classificationAnimaliaAraneaeTheridiidae

(O. P.-Cambridge, 1893)

Neopisinus cognatus Marques et al. 2011: 374 [T]
Episinus cognatus O. P.-Cambridge, 1893; Jackman 1997: 169; Levi 1955b: 71, mf, desc. (figs 8–10, 21–22, 33, 41); Levi 1964b: 13; Levi 2005c: 239; Levi and Randolph 1975: 38; Vogel 1970b: 23

####### Distribution.

Hidalgo

####### Locality.

Lower Rio Grande Valley National Wildlife Refuge

####### Time of activity.

Male (February 28-March 14, April); female (August – September)

####### Habitat.

(orchard: orange); (soil/woodland: ebony-guayacan association)

####### Method.

Flight intercept trap on ground [m]

####### Type.

Mexico, Tabasco, Teapa

####### Etymology.

Latin, related

####### Collection.

TAMU

##### Genus *Neospintharus* Exline, 1950

###### Neospintharus furcatus

Taxon classificationAnimaliaAraneaeTheridiidae

(O. P.-Cambridge, 1894)

Neospintharus furcatus Agnarsson 2004: 479, 514 [T]
Argyrodes furcatus (O. P.-Cambridge, 1894); Bumroongsook et al. 1992: 18; Cokendolpher and Reddell 2001b: 54; Exline and Levi 1962: 116, mf, desc. (figs 84–88); Jackman 1997: 169; Levi and Randolph 1975: 36; Vogel 1970b: 22

####### Distribution.

Anderson, Atascosa, Bastrop, Bell, Bexar, Brazos, Cameron, Frio, Harris, Harrison, Henderson, Hidalgo, Jasper, Kleberg, Montgomery, Newton, Polk, Starr, Travis

####### Locality.

Fort Hood, Jones State Forest, Lick Creek Park, Santa Ana National Wildlife Refuge, Zilker Park

####### Caves.

**Bell** ([all Fort Hood] Coyote Den Cave, Talking Crows Cave)

####### Time of activity.

Male (April, June – July, September – November); female (April, October)

####### Habitat.

(landscape features: cave); (soil/woodland: bottomland forest, *Juniperus ashei*, *Ulmus crassifolia*); (web: web of *Tidarren sisyphoides*)

####### Method.

Beating [m]; flight intercept trap [m]; sweeping [m]

####### Type.

Mexico, Tabasco, Teapa

####### Etymology.

Latin, end of abdomen fish-tail or furcate termination

####### Collection.

DMNS, TAMU, TMM

###### Neospintharus trigonum

Taxon classificationAnimaliaAraneaeTheridiidae

(Hentz, 1850)

Neospintharus trigonum Agnarsson 2004: 479 [T]; Bradley 2013: 229
Argyrodes trigonum (Hentz, 1850); Breene et al. 1993c: 26, 48, 56, mf (figs 21A-C); Dean et al. 1982: 254; Exline and Levi 1962: 122, mf, desc. (figs 66–78); Fox 1940: 40; Jackman 1997: 53, desc., 169; Levi and Randolph 1975: 36; Vogel 1970b: 23; Young and Edwards 1990: 23

####### Distribution.

Brazos, Harris, Hunt, Polk, Travis, Walker

####### Locality.

Ellis Prison Unit, Lick Creek Park, Sam Houston National Forest, Stubblefield Lake

####### Time of activity.

Male (April, July); female (April, August)

####### Habitat.

(crops: cotton); (soil/woodland: bottomland forest)

####### Method.

Flight intercept trap [imm.]; suction trap [m]

####### Type.

Alabama

####### Etymology.

Greek, abdomen viewed sideways appears three-sided

####### Collection.

DMNS, MSU, TAMU

##### Genus *Nesticodes* Archer, 1950

###### Nesticodes rufipes

Taxon classificationAnimaliaAraneaeTheridiidae

(Lucas, 1846)

Nesticodes rufipes Jackman 1997: 169; Levi 2005c: 242; Platnick 1989: 198 [T]
Theridion rufipes Lucas, 1846; Jackman 1997: 169; Levi 1957a: 56, 116, mf, desc. (figs 188–193); Levi and Randolph 1975: 45; Vogel 1970b: 25

####### Distribution.

Hays

####### Type.

Algiers, Oran

####### Etymology.

Latin, reddish legs

##### Genus *Parasteatoda* Archer, 1946

###### Parasteatoda tepidariorum

Taxon classificationAnimaliaAraneaeTheridiidae

(C. L. Koch, 1841)

Parasteatoda tepidariorum Saaristo 2006: 70 [T] (figs 60–63)
Achaearanea tepidariorum (C. L. Koch, 1841); Agnew et al. 1985: 6; Brown 1974: 237; Bumroongsook et al. 1992: 18; Dean and Sterling 1990: 401; Jackman 1997: 52, desc., 168 (photo 18a); Levi 1955a: 32, mf (figs 69–70, 83–84); Levi 1963b: 215; Levi and Randolph 1975: 36; Nyffeler et al. 1988a: 55; Reddell and Cokendolpher 2004: 93; Rice 1986: 124; Vogel 1970b: 22

####### Distribution.

Eastern ½ Texas; Angelina, Aransas, Bexar, Brazos, Clay, Erath, Fort Bend, Galveston, Harris, Harrison, Hays, Henderson, Hidalgo, Houston, Jefferson, Jim Wells, Kerr, Llano, Lubbock, Montgomery, Nacogdoches, Robertson, San Patricio, Titus, Travis, Wichita

####### Locality.

Lake Corpus Christi State Park, Lick Creek Park

####### Caves.

**Bexar** (Robber Barron Cave); **Hays** (Ezell’s Cave); **Kerr** (Seven Room Cave); **Llano** (Enchanted Rock Cave)

####### Time of activity.

Male (January, April, September – December); female (March – April, June, August – December)

####### Habitat.

(landscape features: cave); (littoral: near water); (nest/prey: mud dauber nest [mf]); (structures: attached garage, barn, in [garage, house], indoors, storage area, window screen)

####### Method.

suction trap [mf]

####### Type.

Germany, Bavaria

####### Etymology.

Latin, warm water referring to a Roman bath

####### Collection.

DMNS, JCC, MSU, TAMU, TMM

##### Genus *Paratheridula* Levi, 1957

###### Paratheridula perniciosa

Taxon classificationAnimaliaAraneaeTheridiidae

(Keyserling, 1886)

Paratheridula perniciosa [Levi 1967: 176, mf, desc. (figs 1–4)]

####### Distribution.

Travis

####### Time of activity.

Female (November)

####### Type.

Brazil, Blumenau

####### Etymology.

Latin, rapid or swift

####### Collection.

DMNS

##### Genus *Pholcomma* Thorell, 1869

###### Pholcomma hirsutum

Taxon classificationAnimaliaAraneaeTheridiidae

Emerton, 1882

Pholcomma hirsutum Platnick 1998: 279 [spelling]
Pholcomma hirsuta Emerton, 1882 [Levi 1957d: 110, mf, desc. (figs 19–27)]

####### Distribution.

Wichita

####### Type.

Connecticut, Hamden, Mt. Carmel

####### Etymology.

Latin, hairy

####### Collection.

MSU

##### Genus *Phoroncidia* Westwood, 1835

###### Phoroncidia americana

Taxon classificationAnimaliaAraneaeTheridiidae

(Emerton, 1882)

Phoroncidia americana Guarisco 2008a: 153; Jackman 1997: 169 [Levi 1964c: 74 [T]]
Oronota americana (Emerton, 1882) [Levi 1955c: 334, mf, desc. (figs 1–8)]

####### Distribution.

Sabine, Travis (imm.)

####### Time of activity.

Female (August 25-September 10)

####### Habitat.

(soil/woodland: beech-magnolia forest, *Juniperus ashei*, *Quercus buckleyi*, *Quercus virginiana*, *Ulmus crassifolia*)

####### Method.

Flight intercept trap [f]

####### Type.

Massachusetts, Beverly and Danvers; Connecticut, New Haven

####### Etymology.

locality (country)

####### Collection.

TAMU

##### Genus *Phycosoma* O. P.-Cambridge, 1879

###### Phycosoma lineatipes

Taxon classificationAnimaliaAraneaeTheridiidae

(Bryant, 1933)

Phycosoma lineatipes Fitzgerald and Sirvid 2004: 10 [T]
Dipoena alta Keyserling, 1886; Jackman 1997: 169; Levi 1963a: 159, f (figs 138–139); Levi and Randolph 1975: 37; Vogel 1970b: 23 [see note below]
Dipoena lineatipes Bryant, 1933; Levi 1953: 12, mf, desc. (figs 11–15, 120–121); Roberts 1979: 202, 205, mf, desc. (figs 58–87, 108–110); Vogel 1970b: 23

####### Distribution.

Brazos, Harris, Travis

####### Locality.

Lick Creek Park

####### Time of activity.

Male (March – July, October); female (March – August)

####### Habitat.

(littoral: sedge meadow); (soil/woodland: *Juniperus ashei*, *Quercus buckleyi*, *Quercus virginiana*, *Ulmus crassifolia*)

####### Method.

Beating [mf]; sweeping [mf]

####### Type.

Florida, Royal Palm Park

####### Etymology.

Latin, striped legs

####### Collection.

TAMU

####### Note.

Texas record is *Dipoena cathedralis* Levi, 1953.

##### Genus *Platnickina* Koçak & Kemal, 2008

###### Platnickina alabamensis

Taxon classificationAnimaliaAraneaeTheridiidae

(Gertsch & Archer, 1942)

Platnickina alabamensis Bradley 2013: 231; Koçak and Kemal 2008: 3 [T]
Theridion alabamense Gertsch and Archer, 1942; Agnew et al. 1985: 6; Jackman 1997: 169; Kaston 1978: 108, desc. (fig. 268); Levi 1957a: 58, 116, mf, desc. (figs 202–203, 206–208); Levi and Randolph 1975: 41

####### Distribution.

Brazos, Burleson, Erath, Goliad, Matagorda

####### Locality.

Adriance Pecan Orchard

####### Time of activity.

Male (March, May, July)

####### Habitat.

(orchard: pecan); (soil/woodland: trees)

####### Method.

sweeping [m]

####### Type.

Massachusetts, Wellesley

####### Etymology.

locality (range of distribution)

####### Collection.

TAMU

###### Platnickina antoni

Taxon classificationAnimaliaAraneaeTheridiidae

(Keyserling, 1884)

Platnickina antoni Koçak and Kemal 2008: 3 [T]
Theridium antonii Keyserling, 1884; Banks 1910: 19; Marx 1890: 519
Theridion antoni Keyserling, 1884; Petrunkevitch 1911: 191
Theridion antonii Keyserling, 1884; Fox 1940: 42; Jackman 1997: 169; Levi 1957a: 60, mf, desc. (figs 196–197, 205, 215–216, 219–220); Levi and Randolph 1975: 43; Roewer 1942: 501

####### Distribution.

Bexar

####### Type.

Texas (male, Bexar Co., San Antonio, no date, no collector, holotype, USNM)

####### Etymology.

locality (city)

###### Platnickina mneon

Taxon classificationAnimaliaAraneaeTheridiidae

(Bösenberg & Strand, 1906)

Platnickina mneon Koçak and Kemal 2008: 3 [T]
Theridion adamsoni (Berland, 1934); Levi and Randolph 1975: 41; Vogel 1970b: 24
Coleosoma adamsoni (Berland, 1934); Jackman 1997: 169
Theridion hobbsi Gertsch and Archer, 1942; Levi 1957a: 62, 116, mf, desc. (figs 198–199, 209, 213–214)
Keijia mneon (Bösenberg and Strand, 1906); Yoshida 2001: 172 [S]

####### Distribution.

Jefferson

####### Type.

Japan

####### Etymology.

Greek, mindful

###### Platnickina punctosparsa

Taxon classificationAnimaliaAraneaeTheridiidae

(Emerton, 1882)

Platnickina punctosparsa Koçak and Kemal 2008: 3 [T]
Theridion punctosparsum Emerton, 1882; Jackman 1997: 169; Vogel 1970b: 25 [Levi 1957a: 60, mf, desc. (figs 194–195, 204, 217–218, 220–221)]
Theridion punctisparsum Emerton, 1882; Jones 1936: 69

####### Distribution.

Dallas

####### Type.

Massachusetts, Salem

####### Etymology.

Latin, white spot on abdomen

##### Genus *Rhomphaea* L. Koch, 1872

###### Rhomphaea fictilium

Taxon classificationAnimaliaAraneaeTheridiidae

(Hentz, 1850)

Rhomphaea fictilium Agnarsson 2004: 480 [T]
Argyrodes fictilium (Hentz, 1850); Dean et al. 1988: 286; Exline and Levi 1962: 103, mf, desc. (figs 6–7, 26–28); Jackman 1997: 169; Kaston 1978: 103, desc. (fig. 253C); Levi and Randolph 1975: 36; Vogel 1970b: 22
Rhomphea fictilum (Hentz, 1850); Knutson et al. 2010: 516

####### Distribution.

Dallas, Denton, Erath (imm.), Houston, Howard, Travis

####### Time of activity.

Female (September)

####### Habitat.

(grass: pasture); (soil/woodland: saltcedar)

####### Method.

D-Vac suction [f]; suction trap [imm.]

####### Type.

Alabama

####### Etymology.

Latin, to make

####### Collection.

NMSU, TAMU

###### Rhomphaea projiciens

Taxon classificationAnimaliaAraneaeTheridiidae

O. P.-Cambridge, 1896

Rhomphaea projiciens Agnarsson 2004: 480 [T]
Argyrodes projiciens O. P.-Cambridge, 1896; Exline and Levi 1962: 106, mf, desc. (figs 8–10, 29–31); Jackman 1997: 169; Levi and Randolph 1975: 36; Vogel 1970b: 23
Rhomphea projiciens (O. P.-Cambridge, 1896); Knutson et al. 2010: 516

####### Distribution.

Brazos, Cameron, Hidalgo, Howard, Kerr, Liberty, Travis, Tyler, Walker, Washington

####### Locality.

Ellis Prison Unit, Lick Creek Park, Sabal Palm Audubon Sanctuary

####### Time of activity.

Male (June – August, October); female (February, May, July – October)

####### Habitat.

(crops: cotton); (soil/woodland: forest, palm forest margin [resaca bank], post oak savanna, saltcedar, trees, trees/shrubs, *Juniperus ashei*, *Quercus buckleyi*, *Ulmus crassifolia*)

####### Method.

Beating [mf]; pitfall trap [m]; sweeping [m]

####### Type.

Mexico, Tabasco, Teapa

####### Etymology.

Latin, abdomen and clypeus project forward

####### Collection.

MSU, NMSU, TAMU

##### Genus *Spintharus* Hentz, 1850

###### Spintharus flavidus

Taxon classificationAnimaliaAraneaeTheridiidae

Hentz, 1850

Spintharus flavidus Bradley 2013: 232; Jackman 1997: 169; Kaston 1978: 102, desc. (fig. 252); Levi 1963d: 225, mf (figs 1, 2k-u, w, 3–9); Levi and Randolph 1975: 40; Vogel 1970b: 24

####### Distribution.

Hardin

####### Type.

Alabama

####### Etymology.

Latin, of golden yellow

##### Genus *Steatoda* Sundevall, 1833

###### Steatoda alamosa

Taxon classificationAnimaliaAraneaeTheridiidae

Gertsch, 1960

Steatoda alamosa Gertsch 1960b: 41 [S, part], mf, desc. (figs 53, 59–60, 68–69); Levi and Randolph 1975: 41; Vogel 1967: 136; Vogel 1970b: 24
Lithyphantes pulcher Keyserling, 1884; Milstead 1958: 445
Steatoda pulcher (Keyserling, 1884); Jackman 1997: 169; Levi 1957b: 393 [T], m, desc. (fig. 40) [male misidentified]
Steatoda pulchra (Keyserling, 1884); Vogel 1970b: 24

####### Distribution.

Archer, Brewster, Brown, Concho, Culberson, Hunt, Jeff Davis, Kendall, Kerr, McCulloch, Presidio, Terrell

####### Locality.

Big Bend National Park, Chisos Basin, Chisos Mountains, Davis Mountains, La Mota Mountains, Mount Locke Observatory, Raven Ranch

####### Time of activity.

Male (March, May); female (March, May, July – September, December)

####### Habitat.

(grass: grass); (landscape features: under rock); (nest/prey: stomach of *Cnemidophorus perplexus*, stomach of *Cnemidophorus tigris*)

####### Type.

Texas (male, Brewster Co., Chisos Mountains, Chisos Basin, May 28, 1952, W. J. Gertsch, holotype, AMNH)

####### Etymology.

Spanish, cottonwood

####### Collection.

MSU

###### Steatoda borealis

Taxon classificationAnimaliaAraneaeTheridiidae

(Hentz, 1850)

Steatoda borealis Jackman 1997: 169; Kaston 1978: 113, desc. (fig. 281); Levi 1957b: 422, mf, desc. (figs 116–118, 148–154); Levi and Randolph 1975: 40; Vogel 1970b: 24

####### Distribution.

Jeff Davis, Knox, McLennan

####### Time of activity.

Female (September)

####### Type.

United States

####### Etymology.

Latin, northern

####### Collection.

MSU

###### Steatoda mexicana

Taxon classificationAnimaliaAraneaeTheridiidae

Levi, 1957

Steatoda mexicana Jackman 1997: 169; Levi 1957b: 417, mf, desc. (figs 98–103, 124–128); Levi and Randolph 1975: 41; Vogel 1970b: 24

####### Distribution.

Brewster, Walker

####### Locality.

Big Bend National Park, Chisos Mountains

####### Time of activity.

Female (October)

####### Type.

Mexico, Guerrero, Omiltemi

####### Etymology.

locality (country, new name)

###### Steatoda punctulata

Taxon classificationAnimaliaAraneaeTheridiidae

(Marx, 1898)

Steatoda punctulata Gertsch 1960b: 11 [S, part], mf, desc. (figs 3–5, 11–13, 26–27) [see note below]; Levi 1959d: 109; Levi and Randolph 1975: 41; Vogel 1970b: 24
Steatoda medialis Levi, 1957; Levi 1957b: 388, mf, desc. (figs 34, 44, 55) [part]; Vogel 1970b: 24 [part]

####### Distribution.

Brewster, Hidalgo, Kerr, Llano, Starr, Terrell, Travis, Webb, Zapata

####### Locality.

Enchanted Rock, Raven Ranch

####### Time of activity.

Male (March – April, July – August, November); female (February – August, October – December)

####### Type.

Mexico, Baja California

####### Etymology.

Latin, markings on abdomen

####### Collection.

MSU

####### Note.

32 miles E Laredo and 32 miles SW Laredo should be 32 miles SE Laredo in Zapata Co. based on other records from this date.

###### Steatoda quadrimaculata

Taxon classificationAnimaliaAraneaeTheridiidae

(O. P.-Cambridge, 1896)

Steatoda quadrimaculata Brown 1974: 238; Dean and Eger 1986: 141; Irungu 2007: 31; Jackman 1997: 169; Levi 1957b: 385, mf, desc. (figs 28–31); Levi and Randolph 1975: 41; Vogel 1970b: 24

####### Distribution.

Chambers, Hidalgo, Nacogdoches, Starr, Victoria

####### Locality.

Bentsen-Rio Grande Valley State Park

####### Time of activity.

Male (April, May 25-June 8, August, October, November); female (July)

####### Habitat.

(crops: cabbage); (nest/prey: mud dauber nest [f]); (plants: Indian paintbrush); (soil/woodland: ground); (structures: on house, patio)

####### Method.

pitfall trap [m]; sweeping [m]

####### Type.

Guatemala, Antigua

####### Etymology.

Latin, four white spots forming quadrangle on abdomen

####### Collection.

TAMU

###### Steatoda transversa

Taxon classificationAnimaliaAraneaeTheridiidae

(Banks, 1898)

Steatoda transversa Agnew et al. 1985: 3, 9; Calixto et al. 2013: 184; Dean and Eger 1986: 141; Irungu 2007: 31; Jackman 1997: 169; Levi 1957b: 383 [spelling], mf, desc. (figs 23–27); Levi and Randolph 1975: 41; Young and Edwards 1990: 23
Steatoda transversus (Banks, 1898); Vogel 1970b: 24

####### Distribution.

Brazos, Burleson, Colorado, Comanche, Coryell, Erath, Hays, Hidalgo

####### Locality.

Attwater Prairie Chicken National Wildlife Refuge, Bill Haney Pecan Orchard, Somerville Lake

####### Time of activity.

Male (April – September); female (June, September)

####### Habitat.

(crops: cabbage, peanuts); (littoral: near pond); (orchard: pecan); (plants: Indian paintbrush); (structures: lawn)

####### Method.

pitfall trap [mf] (near pond [m]); suction trap [f]; sweeping [m]

####### Type.

Mexico, Baja California, El Taste

####### Etymology.

Latin, abdomen with transverse band

####### Collection.

MSU, TAMU

###### Steatoda triangulosa

Taxon classificationAnimaliaAraneaeTheridiidae

(Walckenaer, 1802)

Steatoda triangulosa Agnew et al. 1985: 3; Bradley 2013: 233; Breene et al. 1993a: 169; Breene et al. 1993c: 27, 48, 56, mf (figs 22A-C); Brown 1974: 238; Dean et al. 1982: 254; Horner and Russell 1986: 142; Jackman 1997: 59, desc., 169 (photo 18i); Levi 1957b: 407 [T], mf, desc. (figs 75–76, 80–82); Levi 1962: 25; Levi and Randolph 1975: 41; MacKay and Vinson 1989: 232; Rice 1986: 124; Roberts 2001: 48; Tugmon et al. 1990: 43–44; Vogel 1970b: 24; Young and Edwards 1990: 23
Teutana triangulosa Walckenaer, 1802; Jones 1936: 69

####### Distribution.

Anderson, Archer, Atascosa, Bexar, Brazos, Clay, Dallas, Erath, Fort Bend, Haskell, Hidalgo, Kerr, Lubbock, McLennan, Montague, Nacogdoches, Nueces, Potter, Robertson, San Patricio, Shelby, Taylor, Travis, Walker, Wichita

####### Locality.

Ellis Prison Unit, Lake Corpus Christi State Park, Riley Estate, Wildcat Bluff Nature Center

####### Time of activity.

Male (January – April, June, August – December); female (January – December)

####### Habitat.

(crops: cotton, peanuts); (landscape features: culvert, under wooden bridge); (nest/prey: mud dauber nest [mf]); (objects: water meter housing); (orchard: citrus); (soil/woodland: bark of Brazil tree); (structures: around house, behind old boards in [attic, lab, warehouse], house, indoors, on [house by door, wall in lab])

####### Type.

France, Paris

####### Etymology.

Latin, markings on abdomen

####### Collection.

DMNS, JCC, MSU, TAMU

###### Steatoda variata

Taxon classificationAnimaliaAraneaeTheridiidae

Gertsch, 1960

Steatoda variata Broussard and Horner 2006: 255; Gertsch 1960b: 24 [S, part], mf, desc. (figs 23–25, 34–44); Levi and Randolph 1975: 41; Richman et al. 2011a: 46; Vogel 1970b: 24
Steatoda medialis (Banks, 1898); Agnew et al. 1985: 3 [misidentified]; Jackman 1997: 169; Levi 1957b: 388, m, desc. (fig. 35); Vogel 1970b: 24 [part]; Young and Edwards 1990: 23 [Texas records, male misidentified, see punctulata]

####### Distribution.

El Paso, Erath, Hudspeth, Jeff Davis, Maverick, Presidio, Reeves, Wise

####### Locality.

Big Bend Ranch State Park, Chihuahuan desert, Dalquest Research Site, Davis Mountains, Fort Hancock, La Mota Mountains

####### Time of activity.

Male (June); female (May – June, August – October)

####### Habitat.

(crops: peanuts); (plants: vegetation)

####### Method.

Beating [f]; pitfall trap [mf]

####### Type.

Arizona

####### Etymology.

Latin, variable

####### Collection.

MSU, TAMU

###### Steatoda variata china

Taxon classificationAnimaliaAraneaeTheridiidae

Gertsch, 1960

Steatoda variata china Gertsch 1960b: 29, mf, desc; Levi and Randolph 1975: 41; Vogel 1967: 137; Vogel 1970b: 24

####### Distribution.

Starr

####### Time of activity.

Male (April)

####### Type.

Mexico, Nuevo Leon, China

####### Etymology.

locality (town)

##### Genus *Stemmops* O. P.-Cambridge, 1894

###### Stemmops bicolor

Taxon classificationAnimaliaAraneaeTheridiidae

O. P.-Cambridge, 1894

Stemmops bicolor Jackman 1997: 169; Levi 1955c: 338, mf, desc. (figs 14, 17–18, 35–36); Levi and Randolph 1975: 41; Vogel 1970b: 24

####### Distribution.

Cameron, Hidalgo, Kenedy, Starr

####### Locality.

Big Tree-Vine Association, Santa Ana National Wildlife Refuge

####### Time of activity.

Male (January, January 30-February 2, March 3-April 4, May, September – October); female (February, March 3-April 4, September)

####### Habitat.

(soil/woodland: forest, oak savanna)

####### Method.

carrion trap [m]; flight intercept trap [mf]

####### Type.

Mexico, Tabasco, Teapa

####### Etymology.

Latin, two colors

####### Collection.

TAMU

##### Genus *Theridion* Walckenaer, 1805

###### Theridion australe

Taxon classificationAnimaliaAraneaeTheridiidae

Banks, 1899

Theridion australe Agnew et al. 1985: 3, 9; Breene 1988: 23–24, 35; Breene et al. 1988: 180; Breene et al. 1989: 162; Breene et al. 1993b: 648; Breene et al. 1993c: 28, 48, 60, mf (figs 31A-B); Breene et al. 1994: 8; Brown 1974: 238; Bumroongsook et al. 1992: 18; Calixto et al. 2013: 185; Dean and Sterling 1987: 6; Dean and Sterling 1990: 401, 404; Dean et al. 1982: 254; Dean et al. 1987: 268; Dean et al. 1988: 286; Jackman 1997: 60, desc., 169; Levi 1957a: 41, 115, mf, desc. (figs 131–132, 148–151); Li 1990: 137, 142, 144; Liao et al. 1984: 410; Nyffeler et al. 1988a: 55; Nyffeler et al. 1988b: 215; Vincent and Frankie 1985: 380; Vogel 1970b: 24; Young and Edwards 1990: 24

####### Distribution.

Archer, Bee, Brazos, Burleson, Cameron, Collin, Colorado, Comanche, Coryell, Crosby, Delta, Erath, Hays, Hidalgo, Houston, Hunt, Kaufman, Kenedy, Nacogdoches, Nueces, Robertson, San Patricio, Travis, Victoria, Walker, Wilbarger

####### Locality.

Adriance Pecan Orchard, Attwater Prairie Chicken National Wildlife Refuge, Bill Haney Pecan Orchard, Ellis Prison Unit, Holmes Pecan Orchard, South Padre Island, Storey Pecan Orchard

####### Time of activity.

Male (January, April – October); female (January, May – September, November – December)

####### Habitat.

(crops: cotton, peanuts, sugarcane, watermelon); (grass: grass, grassland, pasture); (landscape features: under rock); (nest/prey: mud dauber nest [f]); (orchard: grapefruit, pecan); (plants: miscellaneous vegetation, orchid, ornamental bush); (soil/woodland: live oak, post oak savanna with pasture)

####### Method.

cardboard band [m]; D-Vac suction [mf]; pitfall trap [mf]; suction trap [mf]; sweeping [mf]

####### Eggs/spiderlings.

Nueces [13 spiderlings] [TAMU]

####### Type.

Louisiana, Shreveport

####### Etymology.

Latin, southern

####### Collection.

MSU, TAMU

###### Theridion cameronense

Taxon classificationAnimaliaAraneaeTheridiidae

Levi, 1957

Theridion cameronense Jackman 1997: 169; Levi 1957a: 40, f, desc. (figs 114–115); Levi 1959b: 81; Levi and Randolph 1975: 43; Vogel 1967: 139

####### Distribution.

Cameron

####### Type.

Texas (female, Cameron Co., Harlingen, no date, no collector, holotype, AMNH)

[male unknown]

####### Etymology.

locality (county)

###### Theridion cinctipes

Taxon classificationAnimaliaAraneaeTheridiidae

Banks, 1898

Theridion cinctipes Agnew et al. 1985: 3; Dean and Sterling 1990: 404; Jackman 1997: 169; Levi 1957a: 29 [T], m, desc. (figs 87–88, 99); Levi 1959b: 80; Levi and Randolph 1975: 43; Petrunkevitch 1911: 193; Roewer 1942: 502; Young and Edwards 1990: 24
Theridium cinctipes Banks, 1898; Banks 1898a: 186, m, desc.; Banks 1910: 19; Bonnet 1959: 4460

####### Distribution.

Brazos, Brown, Comanche, Coryell, Erath, Jasper, Walker

####### Locality.

Ellis Prison Unit

####### Time of activity.

Male (June, June 28-July 5, August)

####### Habitat.

(crops: peanuts); (soil/woodland: post oak savanna with pasture)

####### Method.

pitfall trap [m]; suction trap [m]

####### Type.

Texas (male, Brown Co., Brownwood, no date, no collector, holotype, MCZ)

[female unknown]

####### Etymology.

Latin, markings on dorsum

####### Collection.

TAMU

###### Theridion cynicum

Taxon classificationAnimaliaAraneaeTheridiidae

Gertsch & Mulaik, 1936

Theridion cynicum Gertsch and Mulaik 1936b: 10, m, desc. (fig. 12); Jackman 1997: 169; Levi 1957a: 39 [T], 115, mf, desc. (figs 126–128); Levi 1959b: 81; Levi and Randolph 1975: 43; Roewer 1942: 502; Vogel 1970b: 25; Vogel and Durden 1972: 1
Theridium cynicum Gertsch and Mulaik, 1936; Bonnet 1959: 4464

####### Distribution.

Hidalgo, Jim Wells, Kleberg, Llano, Travis

####### Locality.

Bentsen-Rio Grande Valley State Park

####### Time of activity.

Male (May, July, September); female (August, October)

####### Type.

Texas (male, Hidalgo Co., Edinburg, May 27, 1935, S. Mulaik, holotype, AMNH)

####### Etymology.

Greek, snarling or dog-like

####### Collection.

DMNS

###### Theridion differens

Taxon classificationAnimaliaAraneaeTheridiidae

Emerton, 1882

Theridion differens Jackman 1997: 169; Levi 1957a: 32, 114, mf, desc. (figs 100–101, 104–106); Marx 1890: 519; Vogel 1970b: 25

####### Distribution.

Brown, Burleson, Cherokee, Denton, Fannin, Freestone, Henderson, Hunt, Navarro, Titus

####### Time of activity.

Male (May, August); female (August)

####### Habitat.

(plants: miscellaneous vegetation); (soil/woodland: shrubs, trees)

####### Method.

Beating [mf]; sweeping [mf]

####### Type.

Massachusetts, Saugus

####### Etymology.

Latin, difference in size and color of sexes greater than other species

####### Collection.

MSU, TAMU

###### Theridion dilutum

Taxon classificationAnimaliaAraneaeTheridiidae

Levi, 1957

Theridion dilutum Agnew et al. 1985: 6; Jackman 1997: 169; Knutson et al. 2010: 516; Levi 1957a: 37, 115, mf, desc. (figs 112–113, 123–125); Levi and Randolph 1975: 43; Vogel 1967: 141; Vogel 1970b: 25

####### Distribution.

Concho, Coryell, Dickens, Erath, Foard, Hamilton, Hidalgo, Howard, Kimble, Llano, Menard, Scurry, Travis, Uvalde, Val Verde

####### Locality.

Frontera Audubon, Garner State Park, Lake Thomas, Seminole Canyon State Park

####### Time of activity.

Male (April – July); female (May – August)

####### Habitat.

(orchard: grapefruit, orange); (plants: roadside vegetation); (soil/woodland: juniper, post oak savanna with pasture, saltcedar, trees/shrubs, under oak, *Juniperus ashei*, *Quercus buckleyi*, *Quercus virginiana*, *Ulmus crassifolia*); (structures: greenhouse)

####### Method.

Beating [mf]; pitfall trap [m] (under oak [m]); sweeping [mf]

####### Type.

Texas (male, Hidalgo Co., S of Pharr, April 5, 1936, S. Mulaik, holotype, AMNH)

####### Etymology.

Latin, diluted

####### Collection.

MSU, NMSU, TAMU

###### Theridion dividuum

Taxon classificationAnimaliaAraneaeTheridiidae

Gertsch & Archer, 1942

Theridion dividuum Agnew et al. 1985: 6; Dean and Sterling 1990: 401; Jackman 1997: 169 [Levi 1957a: 25, mf, desc. (figs 67–68, 71–74)]

####### Distribution.

Brazos, Coryell, Erath, Uvalde

####### Locality.

Garner State Park

####### Time of activity.

Male (April, June – September); female (July – August)

####### Habitat.

(soil/woodland: post oak savanna with pasture)

####### Method.

pitfall trap [m]; suction trap [mf]

####### Type.

Alabama, Pea River Project

####### Etymology.

Latin, divided

####### Collection.

TAMU

###### Theridion dulcineum

Taxon classificationAnimaliaAraneaeTheridiidae

Gertsch & Archer, 1942

Theridion dulcineum [Levi 1957a: 26, mf, desc. (figs 69–70, 75–76)]

####### Distribution.

Gonzales

####### Time of activity.

Female (October)

####### Type.

Alabama, Cypress Creek

####### Etymology.

Latin, sweet

####### Collection.

DMNS

###### Theridion flavonotatum

Taxon classificationAnimaliaAraneaeTheridiidae

Becker, 1879

Theridion flavonotatum Breene et al. 1993c: 28, 48, 63, mf (figs 37A-B); Brown 1974: 238; Jackman 1997: 169; Levi 1957a: 34, 114, mf, desc. (figs 102–103, 107–109); Levi and Randolph 1975: 43; Vogel 1970b: 25

####### Distribution.

Angelina, Brazos, Coryell, Hidalgo, Houston, Lavaca, Marion, Montgomery, Nacogdoches, Polk, Shelby, Travis, Tyler, Walker, Wichita

####### Locality.

Ellis Prison Unit, Jones State Forest, Lick Creek Park, Sam Houston National Forest, Stubblefield Lake

####### Time of activity.

Male (April – May, July – August); female (April – August)

####### Habitat.

(crops: cotton); (littoral: sedge meadow); (plants: miscellaneous vegetation, vegetation); (soil/woodland: bottomland forest, post oak savanna with pasture, shrubs, trees, *Quercus virginiana*, *Ulmus crassifolia*); (structures: abandoned shack)

####### Method.

Beating [mf]; flight intercept trap [m]; pitfall trap [f]; suction trap [m]; sweeping [mf]

####### Type.

Mississippi, Pascagoula

####### Etymology.

Latin, yellow spots

####### Collection.

MSU, TAMU

###### Theridion frondeum

Taxon classificationAnimaliaAraneaeTheridiidae

Hentz, 1850

Theridion frondeum [Levi 1957a: 81, mf, desc. (figs 288–289, 298–299)]

####### Distribution.

Bexar, Brazoria

####### Locality.

Ramsey Prison Farm

####### Time of activity.

Female (August)

####### Type.

Alabama

####### Etymology.

Latin, referring to a leaf (pattern?)

####### Collection.

MSU, TAMU

###### Theridion glaucescens

Taxon classificationAnimaliaAraneaeTheridiidae

Becker, 1879

Theridion glaucescens Breene et al. 1993c: 28, 48, 63, mf (figs 36A-C); Dean and Sterling 1990: 404; Dean et al. 1982: 254; Guarisco 2008b: 5; Jackman 1997: 169; Jackman et al. 2007: 199; Kaston 1972: 111, desc. (fig. 248); Kaston 1978: 108, desc. (fig. 266); Levi 1957a: 44, 115, mf, desc. (figs 152–153, 155–156); Levi and Randolph 1975: 43; Vogel 1970b: 25; Young and Edwards 1990: 24

####### Distribution.

Burnet, Fannin, Hays, Hunt, Nacogdoches, Walker, Washington

####### Locality.

Ellis Prison Unit, Inks Lake State Park, Lake Tawakoni State Park

####### Time of activity.

Male (April, June – August); female (June, August – September)

####### Habitat.

(crops: cotton); (soil/woodland: trees, trees/shrubs); (web: large spider web)

####### Method.

Beating [mf]; suction trap [m]

####### Type.

Mississippi, Pascagoula

####### Etymology.

Greek, silvery

####### Collection.

MSU, TAMU

###### Theridion goodnightorum

Taxon classificationAnimaliaAraneaeTheridiidae

Levi, 1957

Theridion goodnightorum Jackman 1997: 169; Knutson et al. 2010: 516; Levi 1957a: 41, 115, mf, desc. (figs 129–130, 145–147); Levi and Randolph 1975: 43; Vogel 1970b: 25

####### Distribution.

Crockett, Howard, Hutchinson, Lubbock, Wichita

####### Locality.

Johnson Ranch

####### Time of activity.

Male (August); female (April, August)

####### Habitat.

(soil/woodland: saltcedar, under log)

####### Method.

Beating [m]

####### Type.

Colorado, Blanca

####### Etymology.

Person (collectors, C. and M. Goodnight)

####### Collection.

JCC, MSU, NMSU, TAMU

###### Theridion hidalgo

Taxon classificationAnimaliaAraneaeTheridiidae

Levi, 1957

Theridion hidalgo Agnew et al. 1985: 3, 9; Breene et al. 1993c: 28, 48, 62, mf (figs 35A-C); Bumroongsook et al. 1992: 18; Dean and Sterling 1987: 6; Dean and Sterling 1990: 401; Jackman 1997: 60, desc., 169; Levi 1957a: 43, 115, mf, desc. (figs 133–134, 139–141); Levi 1959b: 83; Levi and Randolph 1975: 43; Vogel 1967: 142; Young and Edwards 1990: 24
Theridion hildalgo Levi, 1957; Vincent and Frankie 1985: 380

####### Distribution.

Andrews, Brazos, Cameron, Collin, Comanche, Coryell, Ellis, Erath, Falls, Gregg, Hidalgo, Kendall, Kerr, Kimble, Kleberg, Llano, McLennan, Mills, Real, Robertson, Scurry, Shackelford, Starr, Sutton, Travis, Uvalde, Val Verde, Walker, Zapata

####### Locality.

Bill Haney Pecan Orchard, Ellis Prison Unit, Garner State Park, Holmes Pecan Orchard, Lake Thomas, Seminole Canyon State Park

####### Time of activity.

Male (February – August, October); female (March – October)

####### Habitat.

(crops: cotton, peanuts); (orchard: grapefruit, orange, sour orange); (plants: roadside vegetation, *Baccharis*); (soil/woodland: juniper, live oak, post oak savanna with pasture, trees, trees/shrubs, *Juniperus ashei*, *Quercus buckleyi*, *Quercus virginiana*, *Ulmus crassifolia*)

####### Method.

Beating [mf]; cardboard band [f]; D-Vac suction [m]; fogging [mf]; suction trap [mf]; sweeping [mf]

####### Type.

Texas (male, Starr Co., 5 miles W Rio Grande City, April 10, 1936, S. Mulaik, holotype, AMNH)

####### Etymology.

undetermined (not county)

####### Collection.

MSU, TAMU

###### Theridion kawea

Taxon classificationAnimaliaAraneaeTheridiidae

Levi, 1957

Theridion kawea [Levi 1957a: 48, f, desc. (figs 118–119)]

####### Distribution.

Presidio

####### Time of activity.

Female (April, September)

####### Habitat.

(plants: *Baccharis*); (soil/woodland: saltcedar, willow)

####### Type.

California, Kawea River, 5 miles E Three Rivers

[male known but not described, deposited at TAMU]

####### Etymology.

locality (river)

####### Collection.

TAMU

###### Theridion llano

Taxon classificationAnimaliaAraneaeTheridiidae

Levi, 1957

Theridion llano Agnew et al. 1985: 3, 9; Dean and Sterling 1990: 401; Jackman 1997: 169; Levi 1957a: 28, mf, desc. (figs 77–80); Levi and Randolph 1975: 45; Reddell 1965: 177; Vogel 1967: 143; Vogel 1970b: 25; Young and Edwards 1990: 24

####### Distribution.

Brazos, Coryell, Dickens, Erath, Hardeman, Hidalgo, Llano, Starr, Val Verde

####### Locality.

Seminole Canyon State Park

####### Caves.

**Hardeman** (Campsey Cave)

####### Time of activity.

Male (June – September); female (April – May, July – August)

####### Habitat.

(crops: peanuts); (landscape features: cave); (soil/woodland: post oak savanna with pasture, trees/shrubs)

####### Method.

Beating [f]; pitfall trap [m]; suction trap [m]

####### Type.

Texas (male, Llano Co., Llano, July 9, 1936, L. I. Davis, holotype, AMNH)

####### Etymology.

locality (city)

####### Collection.

MSU, TAMU, TMM

###### Theridion murarium

Taxon classificationAnimaliaAraneaeTheridiidae

Emerton, 1882

Theridion murarium Agnew et al. 1985: 3; Breene et al. 1993c: 28, 48, 61, mf (figs 32A–C); Bumroongsook et al. 1992: 17; Calixto et al. 2013: 185; Dean and Eger 1986: 141; Dean and Sterling 1990: 401, 404; Dean et al. 1982: 254; Dean et al. 1988: 286; Jackman 1997: 60, desc., 169; Kagan 1942: 16; Kagan 1943: 258; Levi 1957a: 22, 113, mf, desc. (figs 12, 57–58, 61–63); Li 1990: 137, 142, 144; Vincent and Frankie 1985: 380; Vogel 1970b: 25; Young and Edwards 1990: 24

####### Distribution.

Widespread; Angelina, Archer, Bandera, Brazos, Brown, Burleson, Burnet, Cameron, Coke, Comal, Comanche, Concho, Coryell, Crockett, Dallas, Eastland, Edwards, Erath, Falls, Gaines, Gillespie, Hall, Hidalgo, Houston, Kerr, Kimble, Lamar, Liberty, McLennan, Medina, Mitchell, Navarro, Panola, Pecos, Robertson, Shelby, Sutton, Travis, Uvalde, Walker, Wichita, Young

####### Locality.

Adriance Pecan Orchard, Bill Haney Pecan Orchard, Ellis Prison Unit, Garner State Park, Holmes Pecan Orchard, Inks Lake State Park, Lost Maples State Park, Proctor Lake, Vinson Pecan Farm

####### Time of activity.

Male (March – August); female (April – September, November)

####### Habitat.

(crops: cotton, peanuts); (grass: grass, grassland, pasture); (orchard: orange, pecan); (plants: bluebonnets, Indian paintbrush, miscellaneous vegetation); (soil/woodland: elm, juniper, live oak, oak, post oak savanna with pasture, trees/shrubs, woods, *Juniperus ashei*, *Quercus buckleyi*, *Quercus virginiana*, *Ulmus crassifolia*)

####### Method.

Beating [mf]; cardboard band [mf]; fogging [mf]; irrigation tubing [f]; suction trap [m]; sweeping [mf]; tile trap [f]

####### Eggs/spiderlings.

Robertson [eggsac hatch August 18, 2001, 48 spiderlings] [TAMU]

####### Type.

Massachusetts, Salem

####### Etymology.

Latin, mouse-like

####### Collection.

MSU, TAMU

###### Theridion myersi

Taxon classificationAnimaliaAraneaeTheridiidae

Levi, 1957

Theridion myersi Breene et al. 1993b: 648; Jackman 1997: 169 [Levi 1957a: 31, mf, desc. (figs 95–98)]

####### Distribution.

Cameron, Hidalgo, Willacy

####### Time of activity.

Male (March 26-April 2, August, October – November, November 20-December 4); female (January 29-February 6, March 26-April 2, April, August – December)

####### Habitat.

(crops: sugarcane); (grass: grass); (orchard: orange, sour orange, Valley lemon)

####### Method.

D-Vac suction [mf]; pitfall trap [mf]

####### Type.

Florida, Fort Myers

####### Etymology.

locality (city)

####### Collection.

TAMU

###### Theridion positivum

Taxon classificationAnimaliaAraneaeTheridiidae

Chamberlin, 1924

Theridion positivum Calixto et al. 2013: 185; Dean and Sterling 1990: 401; Jackman 1997: 169; Levi 1957a: 68 [S], 117, mf, desc. (figs 237–239, 243–246); Levi 1959b: 114; Levi 1963c: 565; Levi 2005c: 240; Levi and Randolph 1975: 45; Vogel 1970b: 25
Theridion detractum Gertsch and Mulaik, 1936; Gertsch and Mulaik 1936b: 14, f, desc. (fig. 27); Roewer 1942: 502
Theridium detractum Gertsch and Mulaik, 1936; Bonnet 1959: 4468

####### Distribution.

Brazos, Burleson, Cameron, Edwards, Hidalgo, Jim Wells, Kleberg, Medina, Robertson, Starr, Travis, Uvalde, Zapata

####### Locality.

Frontera Audubon, Garner State Park, Holmes Pecan Orchard, Vinson Pecan Farm

####### Time of activity.

Male (April, June – October); female (July – November)

####### Habitat.

(orchard: grapefruit, Mexican lime, orange, pecan, sour orange, tangerine); (soil/woodland: post oak savanna with pasture, *Ulmus crassifolia*)

####### Method.

cardboard band [mf]; fogging [m]; pitfall trap [m]; suction trap [m]; sweeping [m]

####### Type.

Mexico, Gulf of California, Pond Island

####### Etymology.

Latin, positive

####### Collection.

TAMU

###### Theridion rabuni

Taxon classificationAnimaliaAraneaeTheridiidae

Chamberlin & Ivie, 1944

Theridion rabuni Agnew et al. 1985: 3, 9; Breene et al. 1993c: 28, 48, 62, mf (figs 34A-B); Dean and Sterling 1987: 6; Dean and Sterling 1990: 401; Jackman 1997: 169; Levi 1957a: 28, 114, mf, desc. (figs 81–86); Levi and Randolph 1975: 45; Nyffeler et al. 1988a: 55; Young and Edwards 1990: 24

####### Distribution.

Brazos, Colorado, Comanche, Coryell, Crockett, Dallam, Erath, Floyd, Hale, Hidalgo, Hockley, Houston, Lubbock, Terry

####### Locality.

Attwater Prairie Chicken National Wildlife Refuge, Frontera Audubon

####### Time of activity.

Male (April – September); female (June – September, November)

####### Habitat.

(crops: cotton, peanuts); (orchard: grapefruit); (soil/woodland: juniper, post oak savanna with pasture)

####### Method.

Beating [f]; D-Vac suction [mf]; pitfall trap [mf]; suction trap [mf]

####### Type.

Georgia, Tallulah Falls

####### Etymology.

undetermined

####### Collection.

MSU, TAMU

###### Theridion submissum

Taxon classificationAnimaliaAraneaeTheridiidae

Gertsch & Davis, 1936

Theridion submissum Gertsch and Davis 1936: 10, m, desc. (fig. 21); Jackman 1997: 169; Levi 1957a: 38, 115, m, desc. (figs 116–117); Levi 1959b: 84 [T], f, desc. (figs 89–90); Levi and Randolph 1975: 45; Roewer 1942: 505; Vogel 1970b: 25
Theridium submissum Gertsch and Davis, 1936; Bonnet 1959: 4535

####### Distribution.

Brewster

####### Locality.

Big Bend National Park, Chisos Mountains

####### Time of activity.

Male (July)

####### Type.

Texas (male, Brewster Co., Chisos Mountains, July 1935, L. I. Davis, holotype, AMNH)

####### Etymology.

Latin, submissive

##### Genus *Theridula* Emerton, 1882

###### Theridula opulenta

Taxon classificationAnimaliaAraneaeTheridiidae

(Walckenaer, 1841)

Theridula opulenta Dean and Sterling 1990: 404; Jackman 1997: 169; Kaston 1972: 106; Kaston 1978: 104; Levi 1954c: 334, mf, desc. (figs 9–13); Levi 1966: 126; Levi and Randolph 1975: 47; Vogel 1970b: 25

####### Distribution.

East Texas; Bowie, Harrison, Jasper, Newton, Polk, Walker

####### Locality.

Ellis Prison Unit

####### Time of activity.

Male (May – July); female (May)

####### Method.

suction trap [m]

####### Type.

Georgia

####### Etymology.

Latin, magnificent

####### Collection.

TAMU

##### Genus *Thymoites* Keyserling, 1884

###### Thymoites expulsus

Taxon classificationAnimaliaAraneaeTheridiidae

(Gertsch & Mulaik, 1936)

Thymoites expulsus Agnew et al. 1985: 3; Breene et al. 1993b: 648; Breene et al. 1993c: 28, 48, 60, mf (figs 29A-B); Dean and Eger 1986: 141; Dean and Sterling 1987: 6; Jackman 1997: 169; Levi 1964a: 469 [T]; Levi and Randolph 1975: 47; Vogel 1970b: 25; Young and Edwards 1990: 24
Theridion expulsum Gertsch and Mulaik, 1936; Gertsch and Mulaik 1936b: 9, mf, desc. (figs 16–17); Roewer 1942: 503
Theridium expulsum Gertsch and Mulaik, 1936; Bonnet 1959: 4471
Paidisca expulsa (Gertsch and Mulaik, 1936); Levi 1957a: 109 [T], 120, mf, desc. (figs 400, 416–417)

####### Distribution.

Cameron, Colorado, Erath, Hidalgo, Llano, Nueces, Travis, Uvalde, Val Verde, Wharton

####### Locality.

Attwater Prairie Chicken National Wildlife Refuge, Bentsen-Rio Grande Valley State Park, Garner State Park, Piper’s Lake, Seminole Canyon State Park

####### Time of activity.

Male (March – August); female (March – April, June – August)

####### Habitat.

(crops: cotton, peanuts, sugarcane); (grass: grass); (orchard: grapefruit); (plants: bluebonnets); (soil/woodland: *Juniperus ashei*)

####### Method.

Beating [m]; D-Vac suction [mf]; suction trap [mf]; sweeping [mf]

####### Type.

Texas (male, Hidalgo Co., Edinburg, March and April, 1934, S. Mulaik, holotype, AMNH)

####### Etymology.

Latin, driven out

####### Collection.

TAMU

###### Thymoites illudens

Taxon classificationAnimaliaAraneaeTheridiidae

(Gertsch & Mulaik, 1936)

Thymoites illudens Jackman 1997: 169; Levi and Randolph 1975: 47 [T]; Vogel 1970b: 25
Paidisca illudens Gertsch and Mulaik, 1936; Bonnet 1958: 3299; Gertsch and Mulaik 1936b: 3, mf, desc. (figs 20–21); Levi 1957a: 110, 120, mf, desc. (figs 396, 399, 414–415); Roewer 1942: 392
Sphyrotinus illudens (Gertsch and Mulaik, 1936); Levi 1959b: 145 [T]

####### Distribution.

Cameron, Hidalgo

####### Locality.

Bentsen-Rio Grande Valley State Park

####### Time of activity.

Male (January, April, December); female (November – December)

####### Habitat.

(nest/prey: nest of *Neotoma micropus*)

####### Type.

Texas (male, Cameron Co., Brownsville, January 5, 1928, F. E. Lutz, holotype, AMNH)

####### Etymology.

Latin, deceiving

####### Collection.

TAMU

###### Thymoites marxi

Taxon classificationAnimaliaAraneaeTheridiidae

(Crosby, 1906)

Thymoites marxi Jackman 1997: 169; Levi and Randolph 1975: 47 [T]; Vogel 1970b: 25
Paidisca marxi (Crosby, 1906); Gertsch and Mulaik 1936b: 5 [see note below]; Levi 1957a: 111, 120, mf, desc. (figs 393–395, 401, 418–419)

####### Distribution.

Frio, Harris, Hidalgo, Jasper, Starr, Zapata [not Webb]

####### Time of activity.

Male (July, November); female (February, July)

####### Type.

Tennessee, Beersheba; Washington D. C.

####### Etymology.

Person (from Marx collection)

####### Note.

32 miles E Laredo should be 32 miles SE Laredo in Zapata Co. based on other records from this date.

###### Thymoites missionensis

Taxon classificationAnimaliaAraneaeTheridiidae

(Levi, 1957)

Thymoites missionensis Jackman 1997: 169; Levi and Randolph 1975: 47 [T]
Paidisca missionensis Levi, 1957; Levi 1957a: 102, mf, desc. (figs 380–383); Vogel 1967: 135
Sphyrotinus missionensis (Levi, 1957); Levi 1959b: 157 [T]

####### Distribution.

Hidalgo

####### Locality.

Santa Ana National Wildlife Refuge

####### Time of activity.

Female (March, May)

####### Type.

Mexico, Nuevo Leon, 76 miles N Monterrey

####### Etymology.

locality (city, Mission, Texas)

####### Collection.

TAMU

###### Thymoites pallidus

Taxon classificationAnimaliaAraneaeTheridiidae

(Emerton, 1913)

Thymoites pallidus Agnew et al. 1985: 6; Dean and Sterling 1990: 401; Jackman 1997: 169; Levi 1964a: 470 [T]; Vogel 1970b: 25
Theridion edinburgensis Gertsch and Mulaik, 1936; Gertsch and Mulaik 1936b: 9, mf, desc. (figs 18–19); Roewer 1942: 502
Theridium edinburgense Gertsch and Mulaik, 1936; Bonnet 1959: 4470
Paidisca pallida (Emerton, 1913); Levi 1957a: 99 [S], 120, mf, desc. (figs 358–366)

####### Distribution.

Brazos, Erath, Hidalgo, Panola, Starr, Travis

####### Time of activity.

Male (March, May, July – August); female (March)

####### Habitat.

(orchard: orange); (soil/woodland: *Juniperus ashei*)

####### Method.

suction trap [m]

####### Type.

Rhode Island, Buttonwoods or Providence

####### Etymology.

Latin, pale (pallid)

####### Collection.

TAMU

###### Thymoites unimaculatus

Taxon classificationAnimaliaAraneaeTheridiidae

(Emerton, 1882)

Thymoites unimaculatus Breene et al. 1993c: 29, 48, 59, mf (figs 28A-C); Jackman 1997: 169; Levi and Randolph 1975: 47 [T]
Paidisca unimaculata (Emerton, 1882); Levi 1957a: 106, 120, mf, desc. (figs 388–392, 406–413)
Thymoites unimaculatum (Emerton, 1882); Kaston 1978: 107, desc. (fig. 262)
Thymoites unimaculata (Emerton, 1882); Vogel 1970b: 26

####### Distribution.

Brazos, Henderson, McMullen, Walker

####### Locality.

Ellis Prison Unit, Lick Creek Park, Sam Houston National Forest, Stubblefield Lake

####### Time of activity.

Male (July); female (March – July)

####### Habitat.

(crops: cotton); (plants: miscellaneous vegetation); (soil/woodland: tree)

####### Method.

Beating [f]; beating/sweeping [f]; sweeping [m]

####### Type.

Massachusetts, Danvers

####### Etymology.

Latin, white abdomen with black spot in center of dorsum

####### Collection.

TAMU

##### Genus *Tidarren* Chamberlin & Ivie, 1934

###### Tidarren haemorrhoidale

Taxon classificationAnimaliaAraneaeTheridiidae

(Bertkau, 1880)

Tidarren haemorrhoidale Breene et al. 1993a: 169; Breene et al. 1993b: 648; Breene et al. 1993c: 29, 48, 59, mf (figs 27A-C); Bumroongsook et al. 1992: 18; Calixto et al. 2013: 185; Dean and Sterling 1990: 401; Dean et al. 1988: 285; Jackman 1997: 61, desc., 169; Levi 1969: 71 [S]; Nyffeler et al. 1988a: 55; Nyffeler et al. 1988b: 215
Tidarren fordum (Keyserling, 1884); Levi 1957c: 73 [S], mf, desc. (figs 49–57, 61–64); Vogel 1970b: 26
Theridium fordum Keyserling, 1884; Banks 1898b: 236; Banks 1910: 19
Theridion fordum Keyserling, 1884; Petrunkevitch 1911: 196
Theridium elevatum Banks, 1897; Banks 1897: 195, f, desc.; Banks 1898b: 237
Steatoda elevata Banks, 1897; F. O. P.-Cambridge 1902: 387
Theridion texanum Banks, 1910; Banks 1910: 20; Petrunkevitch 1911: 208; Roewer 1942: 499
Theridium texanum Banks, 1910; Banks 1910: 20; Bonnet 1959: 4541
Tidarren sisyphoides (Walckenaer, 1841); Dean et al. 1982: 254 [misidentified]

####### Distribution.

Brazos, Cameron, Hidalgo, Houston, Hunt, Lee, Liberty, Presidio, Robertson, Travis, Walker, Willacy

####### Locality.

Ellis Prison Unit, Holmes Pecan Orchard, Lake Somerville State Park [Nails Creek Unit], Lake Tawakoni State Park

####### Time of activity.

Male (May, July – September); female (June – November)

####### Habitat.

(crops: cotton, sugarcane); (nest/prey: mud dauber nest [f]); (orchard: citrus, pecan); (plants: *Baccharis*); (soil/woodland: *Juniperus ashei*); (structures: barn); (web: large spider web)

####### Method.

Beating [mf]; cardboard band [m]; fogging [f]; suction trap [m]

####### Type.

Brazil, Rio de Janeiro

####### Etymology.

Latin, referring to blood – a hemorrhage

####### Collection.

TAMU

###### Tidarren sisyphoides

Taxon classificationAnimaliaAraneaeTheridiidae

(Walckenaer, 1841)

Tidarren sisyphoides Agnew et al. 1985: 6; Brown 1974: 238; Cokendolpher and Reddell 2001b: 55; Dean and Sterling 1990: 401; Jackman 1997: 169; Levi 1957c: 70 [S], mf, desc. (figs 41–45, 58–60); Levi and Randolph 1975: 47; Liao et al. 1984: 410; Reddell and Cokendolpher 2004: 93; Taber and Fleenor 2003: 237; Vogel 1970b: 26; Young and Edwards 1990: 24
Steatoda forda Keyserling, 1884; F. O. P.-Cambridge 1902: 382
Tidarren fordum Keyserling, 1881; Chamberlin and Ivie 1934: 5, mf (pl. 1)

####### Distribution.

Bell, Bexar, Blanco, Brazos, Cameron, Erath, Hidalgo, Montgomery, Nacogdoches, Newton, Panola, Polk, San Saba, Travis, Walker, Wharton, Williamson, Wise

####### Locality.

Bamburger Ranch Chiroptorium, Ellis Prison Unit, Fort Hood

####### Caves.

**Bell** ([all Fort Hood] Camp 6 Cave No. 1, Coyote Den Cave); **Bexar** (B. J. Pit, Bone Pile Cave, Buzzard Egg Cave, Cave of the Skinny Snake, Eagles Nest Cave, Haz Mat Pit, John Wagner Ranch Cave No. 3, Logan’s Cave, Lost Mine Trail Cave, Winston’s Cave, World Newt Cave); **San Saba** (Blue Haw Cave, Cobweb Fissure, Crevice Cave, Gorman Cave, Wedge Cave); **Travis** (Get Down Cave); **Williamson** (Jug Cave)

####### Time of activity.

Male (July – September); female (March – July, September, November)

####### Habitat.

(crops: cotton); (landscape features: cave, under bridge); (structures: by door outside, in curled leaf under covered bridge over creek); (web: web on dead limb, web 5” from ground)

####### Method.

suction trap [m]

####### Type.

Georgia

####### Etymology.

resemble *Theridion sisyphum* Walckenaer, 1805 = *Parasteatoda lunata* (Clerck, 1757)

####### Collection.

MSU, TAMU, TMM, TTU, USNM

##### Genus *Wamba* O. P.-Cambridge, 1896

###### Wamba crispulus

Taxon classificationAnimaliaAraneaeTheridiidae

(Simon, 1895)

Wamba crispulus Calixto et al. 2013: 185; Levi 2005c: 240, 243; Wunderlich 1995b: 611 [T]
Theridion crispulum Simon, 1895; Agnew et al. 1985: 3; Breene et al. 1993a: 169; Breene et al. 1993c: 28, 48, 61, mf (figs 33A-C); Bumroongsook et al. 1992: 17; Dean and Sterling 1987: 6; Dean and Sterling 1990: 401, 404; Dean et al. 1988: 286; Levi 1959b: 113 [S]; Levi and Randolph 1975: 43; Li 1990: 137, 142, 144; Vogel 1970b: 25; Young and Edwards 1990: 24
Theridion crispulus Simon, 1895; Jackman 1997: 169
Theridion intervallatum Emerton, 1915; Levi 1957a: 64, 117, mf, desc. (figs 222–224, 229–231); Vogel 1970b: 25
Theridion realisticum Gertsch and Mulaik, 1936; Gertsch and Mulaik 1936b: 11, mf, desc. (figs 23–24); Roewer 1942: 505
Theridium realisticum Gertsch and Mulaik, 1936; Bonnet 1959: 4518

####### Distribution.

South, southeast and north Texas; Bastrop, Brazos, Burleson, Cameron, Comanche, Dallas, Erath, Harris, Hidalgo, Houston, Jim Wells, Kenedy, Kleberg, Liberty, Marion, Nueces, Red River, Robertson, Starr, Sutton, Travis, Uvalde, Walker

####### Locality.

Adriance Pecan Orchard, Bastrop State Park, Bill Haney Pecan Orchard, Ellis Prison Unit, Frontera Audubon, Garner State Park, Holmes Pecan Orchard, Lick Creek Park, Sabal Palm Audubon Sanctuary, Texas A&M University Rangeland Area

####### Time of activity.

Male (January – October, December); female (February – December)

####### Habitat.

(crops: cotton, peanuts); (littoral: sandy area); (orchard: citrus, grapefruit, orange, pecan, sour orange, tangerine, Valley lemon); (soil/woodland: juniper, palm forest margin [resaca bank], post oak savanna, sedge meadow, trees, trees/shrubs, *Juniperus ashei*, *Quercus buckleyi*, *Quercus virginiana*, *Ulmus crassifolia*)

####### Method.

Beating [mf]; cardboard band [mf]; D-Vac suction [m]; fogging [mf]; pitfall trap [mf]; suction trap [mf]; sweeping [mf]

####### Type.

Venezuela

####### Etymology.

Latin, to curl

####### Collection.

MSU, TAMU

##### Genus *Yunohamella* Yoshida, 2007

###### Yunohamella lyrica

Taxon classificationAnimaliaAraneaeTheridiidae

(Walckenaer, 1841)

Yunohamella lyrica Bradley 2013: 236; Calixto et al. 2013: 185; Yoshida 2007: 69 [T]
Theridion lyricum Walckenaer, 1841; Agnew et al. 1985: 6; Dean and Eger 1986: 141; Dean and Sterling 1990: 404; Jackman 1997: 169; Kaston 1978: 108, desc. (fig. 267); Levi 1957a: 89, 119, mf, desc. (figs 322–323, 329–331); Levi and Randolph 1975: 45; Liao et al. 1984: 410; Vogel 1970b: 25

####### Distribution.

Brazos, Burleson, Comal, Denton, Erath, Montgomery, Nacogdoches, Orange, Robertson, Travis, Tyler, Walker

####### Locality.

Adriance Pecan Orchard, Brison Pecan Orchard, Ellis Prison Unit, Holmes Pecan Orchard, Jones State Forest, Lick Creek Park

####### Time of activity.

Male (March – September); female (April – September)

####### Habitat.

(littoral: sandy area by water, sedge meadow); (orchard: pecan); (plants: Indian paintbrush, miscellaneous vegetation); (soil/woodland: bottomland forest, sandy area, trees, *Juniperus ashei*, *Quercus buckleyi*, *Quercus virginiana*, *Ulmus crassifolia*)

####### Method.

Beating [mf]; cardboard band [mf]; flight intercept trap [m]; fogging [f]; pitfall trap [f]; suction trap [m]; sweeping [mf]

####### Type.

Georgia

####### Etymology.

Greek, lyre

####### Collection.

MSU, TAMU

####### Note.

A male was collected in a suction trap 10:00 to 12:00 hours.

#### Family Thomisidae Sundevall, 1833

**Note.** Species incorrectly reported from Texas

*Xysticus luctans* (C. L. Koch, 1845); Petrunkevitch 1911: 440 [not in Texas]

*Xysticus triguttatus* Keyserling, 1880; Chickering 1940: 216 [not in Texas]

##### Genus *Bassaniana* Strand, 1928

###### Bassaniana floridana

Taxon classificationAnimaliaAraneaeThomisidae

(Banks, 1896)

Bassaniana floridana Jackman 1997: 169; Ono 1988: 74 [T]
Coriarachne floridana Banks, 1896; Bowling and Sauer 1975: 188, mf, desc. (figs 4, 10, 13)

####### Distribution.

Sabine, Trinity, Walker

####### Time of activity.

Male (April, April 26-May 5)

####### Habitat.

(soil/woodland: pine [%: 66])

####### Method.

5 gallon bucket trap [m]

####### Type.

Florida, Punta Gorda

####### Etymology.

locality (state)

####### Collection.

TAMU

###### Bassaniana utahensis

Taxon classificationAnimaliaAraneaeThomisidae

(Gertsch, 1932)

Bassaniana utahensis Jackman 1997: 169; Ono 1988: 74 [T]
Coriarachne utahensis (Gertsch, 1932); Bowling and Sauer 1975: 192, mf, desc. (figs 8, 11–12, 15, 18)

####### Distribution.

South Texas

####### Type.

Utah, Salt Lake City

####### Etymology.

locality (state)

###### Bassaniana versicolor

Taxon classificationAnimaliaAraneaeThomisidae

(Keyserling, 1880)

Bassaniana versicolor Calixto et al. 2013: 185; Jackman 1997: 169 (photo 40); Ono 1988: 74 [T]; Yantis 2005: 199
Coriarachne versicolor Keyserling, 1880; Agnew et al. 1985: 8; Bowling and Sauer 1975: 189 [S], mf, desc. (figs 6–7, 19–21); Cokendolpher et al. 1979: 729; Dean and Eger 1986: 142; Dean and Sterling 1990: 403; Gertsch 1939a: 405, mf, desc. (figs 254–255, 269); Gertsch 1953: 458, mf (figs 60–61, 64); Jones 1936: 69; Rapp 1984: 7; Vogel 1970b: 26
Coriarachne aemula O. P.-Cambridge, 1898; Gertsch 1953: 459, mf (figs 67–68); Roewer 1955: 832

####### Distribution.

Eastern ½ Texas; Anderson, Baylor, Brazos, Cameron, Childress, Dallas, Erath, Galveston, Hays, Hidalgo, Kerr, Knox, Leon, Lubbock, Robertson, Travis, Wichita

####### Locality.

Holmes Pecan Orchard

####### Time of activity.

Male (February, April – May, October – November, November 12-December 15); female (February – May, July – November)

####### Habitat.

(objects: on croton cage); (orchard: pecan); (plants: Indian paintbrush); (soil/woodland: *Juniperus* managed plot, mesquite, post oak woods [%: 75], sandy area, under bark, under bark associated by many *Loxosceles reclusa* Gertsch and Mulaik, 1940, upland deciduous forest); (structures: in house, indoors, on house)

####### Method.

5 gallon bucket trap [mf]; cardboard band [mf]; flight intercept trap elevated [mf]; flight intercept trap on ground [m]; light trap; Lindgren funnel trap [m]; pitfall trap [f]; suction trap [mf]; sweeping [m]

####### Type.

California, Mariposa; Massachusetts, Boston; Illinois, Peoria; Georgia

####### Etymology.

Latin, changed color

####### Collection.

DMNS, JCC, MSU, TAMU

##### Genus *Bucranium* O. P.-Cambridge, 1881

**Note.** previously in Family Aphantochilidae

###### Bucranium

Taxon classificationAnimaliaAraneaeThomisidae

sp.

Bucranium Teixeira et al. 2014: 73
Majella sp.; Gertsch 1953: 417
Majellula sp.; Jackman 1997: 169; Roth 1985: B-5–1; Roth 1994: 187
Majella affinis Cambridge, 1896; Gertsch and Mulaik 1940: 307

####### Distribution.

Cameron, Hidalgo

####### Locality.

Laguna Atascosa National Wildlife Refuge, Lower Rio Grande Valley National Wildlife Refuge, Santa Ana National Wildlife Refuge

####### Habitat.

(orchard: grapefruit); (soil/woodland: dense coastal brush, ebony-guayacan association)

####### Collection.

TAMU, TTU

####### Note.

Specimens of the undescribed male of *Majellula affinis* (O. P.-Cambridge, 1896) deposited at TAMU, TTU.

##### Genus *Mecaphesa* Simon, 1900

###### Mecaphesa asperata

Taxon classificationAnimaliaAraneaeThomisidae

(Hentz, 1847)

Mecaphesa asperata Calixto et al. 2013: 185; Knutson et al. 2010: 516; Lehtinen and Marusik 2008: 194 [T]
Misumenops asperatus (Hentz, 1847); Agnew et al. 1985: 4; Bonnet 1957: 2953; Breene et al. 1993c: 30, 48, 79, mf (figs 79A-C); Cokendolpher et al. 1979: 730; Dean and Eger 1986: 142; Dean et al. 1982: 255; Dean et al. 1988: 287; Gertsch 1939a: 328, mf, desc. (figs 34–35, 56–57, 69, 72–73); Jackman 1997: 124, desc., 169 (photo 40c); Jones 1936: 69; Pamanes-Guerrero 1975: 16, 34, 38, 42, 60, 63, 79, 81; Rapp 1984: 7; Rydzak and Killebrew 1982: 7; Vogel 1970b: 26; Woods and Harrel 1976: 44; Young and Edwards 1990: 24
Misumenops asparatus (Hentz, 1847); Glick and Noble 1961: 7
Misumenops prosper (Hentz); Pamanes-Guerrero 1975: 42, 60, 79, 81 [misidentified]

####### Distribution.

Archer, Bastrop, Brazos, Brown, Burleson, Burnet, Cameron, Clay, Culberson, Dallas, Erath, Galveston, Haskell, Hidalgo, Houston, Howard, Jefferson, Montague, Robertson, Smith, Travis, Walker, Wichita

####### Locality.

Adriance Pecan Orchard, Bentsen-Rio Grande Valley State Park, Ellis Prison Unit, Galveston Island State Park, Holmes Pecan Orchard, Inks Lake State Park, Lick Creek Park

####### Time of activity.

Male (February – June, October, December); female (March – June, August, October – November)

####### Habitat.

(crops: cotton, peanuts, rice); (grass: grasses, grassland, pasture); (littoral: salt marsh); (orchard: pecan); (plants: bluebonnets, flower, Indian paintbrush, miscellaneous vegetation, vegetation, *Hedeoma* sp.); (soil/woodland: juniper, pricklyash, saltcedar, sandy area, *Juniperus ashei*, *Quercus buckleyi*, *Quercus virginiana*, *Ulmus crassifolia*)

####### Method.

Beating [mf]; cardboard band [f]; suction trap [m]; sweeping [mf]

####### Type.

Alabama

####### Etymology.

Latin, rough

####### Collection.

DMNS, MSU, TAMU

###### Mecaphesa californica

Taxon classificationAnimaliaAraneaeThomisidae

(Banks, 1896)

Mecaphesa californica Lehtinen and Marusik 2008: 194 [T]
Misumenops californicus (Banks, 1896); Armstrong and Richman 2007: 396; Cokendolpher et al. 1979: 730; Gertsch 1939a: 326, mf, desc. (figs 52–53, 67); Jackman 1997: 169; Vogel 1970b: 26

####### Distribution.

Brooks, Cameron, Hidalgo, San Patricio, Travis, Uvalde, Wichita

####### Locality.

Bentsen-Rio Grande Valley State Park, Garner State Park, Russell Farm, Welder Wildlife Refuge, Zilker Park

####### Time of activity.

Male (March, May, July, October, December); female (October, December)

####### Habitat.

(grass: grass); (plants: miscellaneous vegetation); (soil/woodland: *Juniperus ashei*, *Ulmus crassifolia*)

####### Method.

Beating [m]; boll weevil pheromone trap [m]; sweeping [m]

####### Type.

California, Los Angeles

####### Etymology.

locality (state)

####### Collection.

DMNS, MSU, NMSU, TAMU

###### Mecaphesa carletonica

Taxon classificationAnimaliaAraneaeThomisidae

(Dondale & Redner, 1976)

Mecaphesa carletonica Lehtinen and Marusik 2008: 194 [T]
Misumenops carletonicus Dondale and Redner, 1976; Agnew et al. 1985: 8, 11; Jackman 1997: 169 [Dondale and Redner 1976b: 1007, mf, desc. (figs 1–5)]

####### Distribution.

Erath, Hidalgo

####### Time of activity.

Male (March, June)

####### Habitat.

(orchard: grapefruit); (soil/woodland: brush, woods)

####### Method.

sweeping [m]

####### Type.

Canada, Ontario, Carleton Co., Fitzroy Township

####### Etymology.

locality (county)

####### Collection.

TAMU

###### Mecaphesa celer

Taxon classificationAnimaliaAraneaeThomisidae

(Hentz, 1847)

Mecaphesa celer Calixto et al. 2013: 185; Knutson et al. 2010: 516; Lehtinen and Marusik 2008: 194 [T]
Misumenops celer (Hentz, 1847); Agnew et al. 1985: 4, 10; Bonnet 1957: 2954; Breene 1988: 15, 17, 23–26, 35, 39–40, 44; Breene et al. 1988: 180–181; Breene et al. 1989: 162; Breene et al. 1993c: 30, 48, 79, mf (figs 80A-B); Broussard and Horner 2006: 255; Brown 1974: 238; Cokendolpher et al. 1979: 731; Cokendolpher et al. 2008: 11, 53 (fig. 15, photo 47–48); Dean and Eger 1986: 142; Dean and Sterling 1987: 6; Dean and Sterling 1990: 403, 405; Dean et al. 1982: 255; Dean et al. 1987: 264, 268; Dean et al. 1988: 287; Gertsch 1939a: 322 [S], mf, desc. (figs 30–31, 50–51, 68); Jackman 1997: 125, desc., 169; Jones 1936: 69; Kagan 1942: 51; Kagan 1943: 258; Knutson and Gilstrap 1989: 514; Liao et al. 1984: 410; McDaniel et al. 1981: 104; Milstead 1958: 446; Nuessly and Sterling 1984: 97; Nyffeler et al. 1987b: 1121; Nyffeler et al. 1992c: 2; Pamanes-Guerrero 1975: 16, 34, 42, 79, 81; Rapp 1984: 7; Reddell 1965: 177; Richman et al. 2011a: 48; Roberts 2001: 51; Rogers and Horner 1977: 523; Rydzak and Killebrew 1982: 7; Vogel 1970b: 26; Woods and Harrel 1976: 44; Young and Edwards 1990: 24
Misumenops spinosus Keyserling, 1880; Jones 1936: 69

####### Distribution.

Widespread; Archer, Atascosa, Bastrop, Bee, Bexar, Borden, Bosque, Brazoria, Brazos, Burleson, Burnet, Cameron, Carson, Castro, Chambers, Collin, Colorado, Comal, Comanche, Coryell, Crosby, Dallas, Delta, Dickens, Duval, Eastland, Ector, Ellis, Erath, Falls, Fannin, Fayette, Fisher, Floyd, Frio, Gaines, Galveston, Gillespie, Goliad, Gonzales, Hale, Harris, Hays, Henderson, Hidalgo, Hill, Hockley, Houston, Howard, Jefferson, Kaufman, Kendall, Kent, Kerr, King, Kinney, Knox, Lavaca, Limestone, Lubbock, Martin, McLennan, Midland, Milam, Mitchell, Nacogdoches, Newton, Nueces, Pecos, Polk, Potter, Presidio, Reagan, Reeves, Refugio, Robertson, San Patricio, Schleicher, Scurry, Smith, Sterling, Sutton, Terry, Titus, Tom Green, Travis, Upshur, Upton, Uvalde, Val Verde, Victoria, Walker, Wharton, Wichita, Williamson, Yoakum

####### Locality.

Attwater Prairie Chicken National Wildlife Refuge, Bentsen-Rio Grande Valley State Park, Bill Haney Pecan Orchard, Chihuahuan desert, Dalquest Research Site, Ellis Prison Unit, Galveston Island State Park, Garner State Park, Holmes Pecan Orchard, Lacuna Park, Lake Buchanan, Lake Meredith, Lake Thomas, Lick Creek Park, Nash Prairie, Palmetto State Park, Pantex Lake, Proctor Lake, Ramsey Prison Farm, Riley Estate, Sam Houston National Forest, Santa Ana National Wildlife Refuge, Seminole Canyon State Park, South Padre Island, Stiles Farm Foundation, Stubblefield Lake, Texas A&M University Rangeland Area, Wildcat Bluff Nature Center

####### Caves.

**Sutton** (Felton Cave)

####### Time of activity.

Male (February – December); female (January – December)

####### Habitat.

(crops: corn, cotton, guar, *Helianthus annuus*, peanuts, rice, sugarcane); (grass: *Bromus tectorum*, grass, grassland, pasture); (landscape features: cave); (littoral: near playa, salt marsh area); (nest/prey: mud dauber nest [f], stomach of *Cnemidophorus perplexus*); (objects: in croton cage, lawn mower); (orchard: peach orchard, pecan); (plants: bluebonnets, next to cotton field, among croton, emergent vegetation, Indian paintbrush, miscellaneous vegetation, pigeon pea, pink evening primrose, roadside vegetation, sage, thistle, vegetation, yellow horsemint, *Achillea millefolium*, *Aphanostephus* sp., *Asclepias* sp., *Aster* sp., *Baccharis*, *Borrichia frutescens*, *Cassia* sp., *Coreopsis* sp., *Dalea* sp., *Engelmannia* sp., *Euphorbia* sp., *Gaillardia pulchella*, *Melilotus officinalis*, *Monarda citriodora*, *Oenothera speciosa*, *Prionopsis ciliata*, *Rudbeckia* sp., *Thelesperma* sp., *Vicia* sp.); (soil/woodland: brush, on ground, post oak savanna with pasture, saltcedar, sandy area, trees/shrubs, willow, *Prosopis grandulosa*, *Quercus buckleyi*, *Quercus virginiana*, *Tamarix gallica*, *Ulmus crassifolia*)

####### Method.

Ballooning; beating [m]; boll weevil pheromone trap [mf]; D-Vac suction [mf]; light trap; pitfall trap [mf]; suction trap [mf]; sweeping [mf]

####### Eggs/spiderlings.

Brazos [eggsac laid May 22, 1978, hatch June 1, 91 spiderlings] [TAMU]

####### Type.

South Carolina

####### Etymology.

Latin, swift

####### Collection.

DMNS, JCC, MSU, TAMU, TMM

###### Mecaphesa coloradensis

Taxon classificationAnimaliaAraneaeThomisidae

(Gertsch, 1933)

Mecaphesa coloradensis Knutson et al. 2010: 516; Lehtinen and Marusik 2008: 194 [T]
Misumenops coloradensis Gertsch, 1933; Breene et al. 1993c: 30, 48, 79, mf (figs 81A-B); Cokendolpher et al. 2008: 11, 68; Dean and Sterling 1987: 6; Gertsch 1939a: 331, mf, desc. (figs 60–61, 66); Jackman 1997: 169; Reddell and Fieseler 1977: 95; Vogel 1970b: 26

####### Distribution.

Brewster, Carson, Culberson, Howard, Presidio, Reeves, Travis

####### Locality.

Big Bend National Park, Big Bend Ranch State Park

####### Caves.

**Presidio** (John’s Guano Mine)

####### Time of activity.

Male (July – October); female (March – April, September)

####### Habitat.

(crops: cotton); (grass: grassland); (plants: vegetation); (soil/woodland: saltcedar, scrub cottonwood, willow, *Quercus buckleyi*, *Quercus virginiana*)

####### Method.

Beating [m]; D-Vac suction [m]; sweeping [f]

####### Type.

Colorado

####### Etymology.

locality (state)

####### Collection.

TAMU, TMM

###### Mecaphesa dubia

Taxon classificationAnimaliaAraneaeThomisidae

(Keyserling, 1880)

Mecaphesa dubia Calixto et al. 2013: 185; Knutson et al. 2010: 516; Lehtinen and Marusik 2008: 194 [T]
Misumenops dubius (Keyserling, 1880); Armstrong and Richman 2007: 396; Breene et al. 1993a: 169; Breene et al. 1993b: 648; Breene et al. 1993c: 30, 48, 79, mf (figs 82A-B); Cokendolpher et al. 1979: 731; Dean and Sterling 1987: 6; Dean et al. 1988: 287; Gertsch 1939a: 325 [S], mf, desc. (figs 48–49, 64); Glick 1957: 5; Jackman 1997: 169; Kagan 1942: 49; Kagan 1943: 258; Nyffeler et al. 1992c: 2; Vogel 1970b: 26; Young and Edwards 1990: 24
Misumena dubia Keyserling, 1880; Marx 1890: 556

####### Distribution.

Archer, Atascosa, Bastrop, Bee, Bell, Bexar, Borden, Brazos, Brewster, Brooks, Burleson, Burnet, Callahan, Cameron, Colorado, Comanche, Concho, Coryell, Dimmit, Erath, Falls, Frio, Goliad, Gonzales, Hamilton, Hays, Hidalgo, Hill, Houston, Howard, Jones, Kendall, Kenedy, Kerr, Kimble, Kinney, Knox, Lee, McLennan, McMullen, Nolan, Nueces, Presidio, Robertson, San Patricio, Scurry, Starr, Travis, Uvalde, Val Verde, Victoria, Ward, Webb, Wharton, Wichita, Willacy, Williamson, Zapata

####### Locality.

Adriance Pecan Orchard, Attwater Prairie Chicken National Wildlife Refuge, Bentsen-Rio Grande Valley State Park, Big Bend National Park, Bill Haney Pecan Orchard, Falcon State Park, Garner State Park, Goliad State Park, Holmes Pecan Orchard, Lake Thomas, Pollito Lake, Russell Farm, Sabal Palm Audubon Sanctuary, Santa Ana National Wildlife Refuge, Seminole Canyon State Park, South Padre Island, Stiles Farm Foundation, Welder Wildlife Refuge, Zilker Park

####### Caves.

**Burnet** (Beaver Creek Bat Cave)

####### Time of activity.

Male (February – December); female (February – December)

####### Habitat.

(crops: cotton, *Sorghum halepense*, sugarcane); (grass: grass, grassland, pasture, *Panicum virgatum*); (landscape features: cave, rocky hillside); (littoral: dune vegetation, grass marsh); (nest/prey: mud dauber nest); (orchard: citrus, grapefruit, pecan, Valley lemon); (plants: bluebonnets, Compositae, miscellaneous vegetation, pink evening primrose, roadside vegetation, *Baccharis*, *Centaurea* sp., *Coreopsis* sp., *Dalea* sp., *Euphorbia* sp., *Gaillardia* sp., *Liatris mucronata*, *Monarda citriodora*, *Prionopsis ciliata*, *Thelesperma* sp., Xanthium sp. cf. italicum, *Xanthocephalum dracunculoides*); (soil/woodland: brushy area, chaparral, hackberry matte, juniper, saltcedar, scrub cottonwood, trees, trees/shrubs, willow, *Prosopis grandulosa*)

####### Method.

Beating [mf]; boll weevil pheromone trap [m]; D-Vac suction [mf]; pitfall trap [f]; suction trap [m]; sweeping [mf]

####### Type.

Mexico

####### Etymology.

Latin, uncertain affinity

####### Collection.

DMNS, MSU, NMSU, TAMU, TMM

##### Genus *Misumena* Latreille, 1804

###### Misumena vatia

Taxon classificationAnimaliaAraneaeThomisidae

(Clerck, 1757)

Misumena vatia Brown 1974: 238; Jackman 1997: 123, desc., 169; Jones 1936: 69; Roewer 1955: 837 [S]; Vogel 1970b: 26; Woods and Harrel 1976: 44; Young and Edwards 1990: 24
Misumena calycina (Linnaeus, 1758); Gertsch 1939a: 314, mf, desc. (figs 3–4, 26–27, 38–39, 86, 96–97)

####### Distribution.

Cameron, Dallas, Jefferson, Nacogdoches, Val Verde

####### Caves.

**Val Verde** (Fawcett’s Cave)

####### Time of activity.

Female (June)

####### Habitat.

(crops: rice); (landscape features: cave); (nest/prey: mud dauber nest [f])

####### Type.

unknown

####### Etymology.

Latin, bow-legged

####### Collection.

TMM

##### Genus *Misumenoides* F. O. P.-Cambridge, 1900

###### Misumenoides formosipes

Taxon classificationAnimaliaAraneaeThomisidae

(Walckenaer, 1837)

Misumenoides formosipes Agnew et al. 1985: 4; Breene et al. 1993c: 29, 48, 78, mf (figs 77A-C); Cokendolpher et al. 1979: 730; Cokendolpher et al. 2008: 11, 68; Dean and Eger 1986: 142; Dean and Sterling 1987: 6; Dean et al. 1982: 255; Dean et al. 1988: 287; Jackman 1997: 124, desc., 169 (photo 40b); Knutson et al. 2010: 516; Liao et al. 1984: 410; Nuessly and Sterling 1984: 97; Nyffeler et al. 1992c: 2; Platnick 1993: 711 [S]; Rapp 1984: 8; Rogers and Horner 1977: 523; Rydzak and Killebrew 1982: 7; Taber and Fleenor 2003: 241; Wilson and Pitts 2007: 226; Young and Edwards 1990: 24
Misumenoides aleatorius (Hentz, 1847); Gertsch 1939a: 309, mf, desc. (figs 5–6, 28–29, 40–41, 87, 94–95); Kagan 1942: 53; Kagan 1943: 258

####### Distribution.

Anderson, Bastrop, Brazoria, Brazos, Burleson, Burleson/Lee, Carson, Collin (imm.), Colorado, Culberson, Erath (imm.), Galveston, Gillespie, Hidalgo, Houston (imm.), Howard, Knox, Limestone, Marion (imm.), McLennan, Orange, Palo Pinto, Presidio, San Patricio (imm.), Scurry, Smith, Travis, Van Zandt, Victoria, Walker, Ward, Wichita

####### Locality.

Attwater Prairie Chicken National Wildlife Refuge, Big Bend Ranch State Park, Ellis Prison Unit, Galveston Island State Park, Guadalupe Mountains National Park, Lake Thomas, Lick Creek Park, Monahans Sandhills State Park, Nash Prairie, Welder Wildlife Refuge

####### Time of activity.

Male (May – June, August – September); female (May – November)

####### Habitat.

(crops: cotton, guar, *Helianthus annuus*, peanuts); (grass: grassland, pasture, grassy and shrub area); (littoral: salt marsh area); (plants: bluebonnets, croton, Indian paintbrush, miscellaneous vegetation, vegetation, *Euphorbia* sp., *Monarda citriodora*, *Prionopsis ciliata*); (soil/woodland: hackberry matte, post oak savanna, post oak savanna with pasture, saltcedar, sandy area, trees/shrubs, willow, *Prosopis grandulosa*)

####### Method.

Beating [pen m]; boll weevil pheromone trap [imm.]; light trap [f]; pitfall trap [m]; suction trap [imm.]; sweeping [mf]

####### Eggs/spiderlings.

Brazos [195, 199 eggs]; Walker [eggsac laid October 9, 1978, hatch November 2, 304 spiderlings, 215 unhatched eggs] [TAMU]

####### Type.

Georgia

####### Etymology.

Latin, referring to beautiful

####### Collection.

DMNS, MSU, NMSU, TAMU

##### Genus *Misumessus* Banks, 1904

###### Misumessus oblongus

Taxon classificationAnimaliaAraneaeThomisidae

(Keyserling, 1880)

Misumessus oblongus Calixto et al. 2013: 185; Lehtinen and Marusik 2008: 195 [T]
Misumenops oblongus (Keyserling, 1880); Agnew et al. 1985: 5; Breene et al. 1993c: 30, 48, 78, mf (figs 78A-C); Brown 1974: 238; Bumroongsook et al. 1992: 17; Cokendolpher et al. 1979: 732; Dean and Sterling 1987: 6; Dean and Sterling 1990: 403; Dean et al. 1982: 255; Gertsch 1939a: 319, mf, desc. (figs 44–45, 62–63); Jackman 1997: 125, desc., 169; Jones 1936: 69; Liao et al. 1984: 410; Rapp 1984: 8; Rydzak and Killebrew 1982: 7; Vincent and Frankie 1985: 380; Vogel 1970b: 26; Woods and Harrel 1976: 44; Young and Edwards 1990: 24

####### Distribution.

Archer, Brazos, Burleson, Comanche, Dallas, Delta, Erath, Fannin, Frio, Galveston, Hill, Jefferson, Johnson, Kerr, Llano, Nacogdoches, Polk, Presidio, Robertson, Smith, Travis, Walker, Wharton, Wichita, Young

####### Locality.

Adriance Pecan Orchard, Ellis Prison Unit, Galveston Island State Park, Holmes Pecan Orchard

####### Time of activity.

Male (April – September); female (May – October)

####### Habitat.

(crops: cotton, peanuts, rice); (littoral: salt marsh); (nest/prey: mud dauber nest [mf]); (orchard: pecan); (plants: vegetation, *Baccharis*); (soil/woodland: cedar elm, live oak, sandy area, willow, woods, *Quercus buckleyi*, *Quercus virginiana*, *Ulmus crassifolia*); (structures: on ground under clothes line)

####### Method.

Ballooning [m]; beating [m]; cardboard band [m]; D-Vac suction [m]; fogging [mf]; suction trap [m]; sweeping [m]

####### Type.

Maryland, Baltimore; Illinois, Peoria

####### Etymology.

Latin, oblong

####### Collection.

DMNS, MSU, TAMU

##### Genus *Modysticus* Gertsch, 1953

###### Modysticus modestus

Taxon classificationAnimaliaAraneaeThomisidae

(Scheffer, 1904)

Modysticus modestus Cokendolpher et al. 2008: 11, 53; Marusik et al. 2005: 153 [T]
Ozyptila modesta (Scheffer, 1904); Roberts 2001: 51; Yantis 2005: 201 [Dondale and Redner 1975a: 142, mf, desc. (figs 7–8, 50–52)]

####### Distribution.

Carson, Potter, Trinity

####### Locality.

Wildcat Bluff Nature Center

####### Time of activity.

Male (April 26-May 5)

####### Habitat.

(grass: grassland); (littoral: near playa); (soil/woodland: pine woods [%: 66])

####### Method.

5 gallon bucket trap [f]; pitfall trap

####### Type.

Kansas, Manhattan

####### Etymology.

Latin, calm

####### Collection.

TAMU

##### Genus *Ozyptila* Simon, 1864

**Note.** Dondale and Redner 1975a: 157 [spelling of genus]

###### Ozyptila americana

Taxon classificationAnimaliaAraneaeThomisidae

Banks, 1895

Ozyptila americana Bradley 2013: 241; Dondale and Redner 1975a: 157 [S], mf, desc. (figs 30, 33, 95–96); Dondale and Redner 1978b: 164, mf, desc. (figs 508–511); Jackman 1997: 169
Oxyptila americana Banks, 1895; Kaston 1978: 231; Rydzak and Killebrew 1982: 7
Oxyptila barrowsi Gertsch, 1939; Gertsch 1953: 466, f, (fig. 80)

####### Distribution.

Dallas, Gonzales

####### Locality.

Palmetto State Park

####### Type.

New York, Ithaca

####### Etymology.

locality (country)

###### Ozyptila hardyi

Taxon classificationAnimaliaAraneaeThomisidae

Gertsch, 1953

Ozyptila hardyi Dondale and Redner 1975a: 143, f, desc. (figs 45–46); Jackman 1997: 169; Roewer 1955: 883; Vogel 1967: 151
Oxyptila hardyi Gertsch, 1953; Gertsch 1953: 471, f (fig. 83); Vogel 1970b: 26

####### Distribution.

Cameron

####### Locality.

Laguna Madre

####### Time of activity.

Female (August)

####### Habitat.

(nest/prey: nest of *Neotoma micropus*)

####### Type.

Texas (female, Cameron Co., Laguna Madre, 25 miles SW Harlingen, August 22, 1945, Hardy and Wooley, holotype, AMNH)

[male unknown]

####### Etymology.

Person (collector)

###### Ozyptila monroensis

Taxon classificationAnimaliaAraneaeThomisidae

Keyserling, 1884

Ozyptila monroensis Dondale and Redner 1975a: 148, mf , desc. (figs 15–16, 61–63); Dondale and Redner 1978b: 160, mf, desc. (figs 498–502); Jackman 1997: 169
Oxyptila monroensis Keyserling, 1884; Kaston 1978: 231

####### Distribution.

Bandera, Houston, Kerr

####### Locality.

Big Slough Wild Area, Lost Maples State Park, Raven Ranch

####### Time of activity.

Male (April – May); female (April – May)

####### Habitat.

(soil/woodland: leaf litter, mixed hardwood leaf litter)

####### Method.

Berlese funnel [mf]; carrion pitfall trap [mf]

####### Type.

Virginia, Fort Monroe

####### Etymology.

locality (county)

####### Collection.

TAMU

###### Ozyptila praticola

Taxon classificationAnimaliaAraneaeThomisidae

(C. L. Koch, 1837)

Ozyptila praticola [Dondale and Redner 1975a: 144, mf, desc. (figs 9, 12, 53–54)]

####### Distribution.

Brown

####### Type.

Europe

####### Etymology.

Latin, referring to a meadow, -cola Latin suffix meaning inhabitant of)

####### Collection.

MSU

##### Genus *Synema* Simon, 1864

**Note.** Platnick 1993: 718 [spelling of genus]

###### Synema parvulum

Taxon classificationAnimaliaAraneaeThomisidae

(Hentz, 1847)

Synema parvulum Jackman 1997: 169 [Gertsch 1939a: 334, mf, desc. (figs 80–81, 88)]
Synema parvula (Hentz, 1847); Breene et al. 1993c: 30, 48, 80, mf (figs 83A-C); Dean et al. 1982: 255; Rydzak and Killebrew 1982: 7; Young and Edwards 1990: 24

####### Distribution.

Brazos, Smith, Walker

####### Locality.

Ellis Prison Unit, Lick Creek Park

####### Time of activity.

Male (April – May); female (June, August)

####### Habitat.

(crops: cotton); (plants: Indian paintbrush); (soil/woodland: bottomland forest, forest litter, tree)

####### Method.

Beating [m]; berlese funnel [imm.]

####### Type.

southern states

####### Etymology.

Latin, small

####### Collection.

TAMU

###### Synema viridans

Taxon classificationAnimaliaAraneaeThomisidae

(Banks, 1896)

Synema viridans Armstrong and Richman 2007: 396; Dean and Eger 1986: 142; Dean and Sterling 1990: 405; Gertsch 1939a: 335, mf, desc. (figs 84–85, 89); Jackman 1997: 169; Roewer 1955: 894; Vogel 1970b: 26

####### Distribution.

Brazos, Cameron, Hidalgo, Uvalde, Walker

####### Locality.

Bentsen-Rio Grande Valley State Park, Ellis Prison Unit, Garner State Park, Lick Creek Park, Russell Farm, Sabal Palm Audubon Sanctuary

####### Time of activity.

Male (February – April); female (March – July)

####### Habitat.

(plants: bluebonnets, Indian paintbrush); (soil/woodland: palm forest margin [resaca bank], tree)

####### Method.

Beating [f]; boll weevil pheromone trap [m]; suction trap [m]; sweeping [mf]

####### Type.

Florida, Punta Gorda

####### Etymology.

Latin, color (green)

####### Collection.

NMSU, TAMU

##### Genus *Tmarus* Simon, 1875

###### Tmarus angulatus

Taxon classificationAnimaliaAraneaeThomisidae

(Walckenaer, 1837)

Tmarus angulatus Agnew et al. 1985: 5; Brown 1974: 238; Gertsch 1939a: 305, mf, desc. (figs 11, 21–22, 25); Jackman 1997: 170; Vogel 1970b: 26; Young and Edwards 1990: 24

####### Distribution.

Archer, Brown, Burleson, Cameron, Eastland, Erath, Hidalgo, Kimble, Lavaca, Llano, Montgomery, Nacogdoches, Rockwall, Travis, Wichita

####### Time of activity.

Male (February – August); female (March – June, August, November – December)

####### Habitat.

(crops: peanuts); (nest/prey: mud dauber nest [f]); (plants: miscellaneous vegetation); (soil/woodland: brush, cedar, shrubs, trees, *Juniperus ashei*, *Quercus buckleyi*, *Quercus virginiana*, *Ulmus crassifolia*); (structures: driveway)

####### Method.

Beating [m]; suction trap [m]; sweeping [mf]

####### Type.

Georgia

####### Etymology.

Latin, angle of abdomen

####### Collection.

MSU, TAMU

###### Tmarus floridensis

Taxon classificationAnimaliaAraneaeThomisidae

Keyserling, 1884

Tmarus floridensis Brown 1974: 238; Gertsch 1939a: 304, mf, desc. (figs 15–16, 23); Jackman 1997: 170; Marx 1890: 558; Vogel 1970b: 26

####### Distribution.

Brazos, Freestone, Harris, Liberty, Nacogdoches, Walker

####### Locality.

Lick Creek Park

####### Time of activity.

Male (May – July); female (June, August)

####### Habitat.

(nest/prey: mud dauber nest [f])

####### Method.

Beating [m]; beating/sweeping [m]

####### Type.

Florida

####### Etymology.

locality (state)

####### Collection.

TAMU

###### Tmarus rubromaculatus

Taxon classificationAnimaliaAraneaeThomisidae

Keyserling, 1880

Tmarus rubromaculatus Gertsch 1939a: 307, mf, desc. (figs 17–18, 24); Jackman 1997: 170

####### Distribution.

Bandera, Brazos, Brown, Burleson, Jasper, Kerr, Travis, Walker

####### Locality.

Lost Maples State Park

####### Time of activity.

Male (March – April, August); female (March, May – July)

####### Habitat.

(grass: grass); (soil/woodland: tree, *Quercus buckleyi*)

####### Method.

sweeping [mf]

####### Type.

Georgia

####### Etymology.

Latin, red-spotted

####### Collection.

MSU, TAMU

###### Tmarus unicus

Taxon classificationAnimaliaAraneaeThomisidae

Gertsch, 1936

Tmarus unicus Bonnet 1959: 4648; Gertsch 1936: 14, imm. f, desc.; Gertsch 1939a: 302, imm. f, desc. (figs 12–14); Jackman 1997: 170; Roewer 1955: 825; Vogel 1970b: 26

####### Distribution.

Hidalgo

####### Type.

Texas (immature female, Hidalgo Co., Edinburg, March 3, 1934, S. Mulaik, holotype, AMNH)

[male, female unknown]

####### Etymology.

Latin, for unique

##### Genus *Xysticus* C. L. Koch, 1835

###### Xysticus apachecus

Taxon classificationAnimaliaAraneaeThomisidae

Gertsch, 1933

Xysticus apachecus Agnew et al. 1985: 8; Gertsch 1933a: 22, f, desc. (fig. 24); Gertsch 1939a: 356, mf, desc. (figs 144–145, 174); Gertsch 1953: 423; Jackman 1997: 170
Xysticus apacheus Gertsch, 1933; Vogel 1970b: 26

####### Distribution.

Bexar, Colorado, Coryell, Erath, Kerr, Kimble, Travis

####### Locality.

Attwater Prairie Chicken National Wildlife Refuge

####### Time of activity.

Male (April – May); female (February, April – May, November)

####### Habitat.

(landscape features: under rock); (soil/woodland: cedar, juniper, post oak savanna with pasture, upland deciduous forest)

####### Method.

Flight intercept trap on ground [m]; pitfall trap [m]; sweeping [m]

####### Eggs/spiderlings.

Erath [eggsac hatch May 5, 1983, 217 spiderlings] [TAMU]

####### Type.

Utah, Blanding

####### Etymology.

Indians

####### Collection.

MSU, TAMU

###### Xysticus aprilinus

Taxon classificationAnimaliaAraneaeThomisidae

Bryant, 1930

Xysticus aprilinus Bonnet 1959: 4852; Gertsch 1939a: 381, mf, desc. (figs 204–205); Gertsch 1953: 445; Jackman 1997: 170; Roewer 1955: 916; Vogel 1970b: 26

####### Distribution.

El Paso, Reeves

####### Type.

Texas (female, El Paso Co., no date, no collector, holotype, MCZ)

####### Etymology.

Latin, seasons (month collected)

###### Xysticus auctificus

Taxon classificationAnimaliaAraneaeThomisidae

Keyserling, 1880

Xysticus auctificus Agnew et al. 1985: 8, 11; Breene et al. 1993c: 30, 48, 80, mf (figs 84A-B); Brown 1974: 238; Cokendolpher et al. 1979: 732; Dean and Eger 1986: 142; Dean et al. 1982: 255; Gertsch 1939a: 361, mf, desc. (figs 176–177, 188); Gertsch 1953: 431; Jackman 1997: 126, 170 (photo 40f); Nyffeler et al. 1992c: 2; Roberts 2001: 51; Vogel 1970b: 26; Young and Edwards 1990: 24

####### Distribution.

Atascosa, Bee, Bexar, Bosque, Brazos, Brown, Burleson, Cameron, Cass, Colorado, Coryell, Dallas, Erath, Fayette, Gillespie, Gonzales, Hays, Kendall, Kerr, Lampasas, Leon, Montague, Nacogdoches, Navarro, Nueces, Palo Pinto, Potter, Robertson, San Patricio, Somervell, Travis, Victoria, Walker, Wichita, Wise

####### Locality.

Attwater Prairie Chicken National Wildlife Refuge, Ellis Prison Unit, Holmes Pecan Orchard, Lacuna Park, Texas A&M University Rangeland Area, Welder Wildlife Refuge, Wildcat Bluff Nature Center

####### Time of activity.

Male (March – June, August); female (January, April – July)

####### Habitat.

(crops: cotton); (littoral: near pond); (nest/prey: mud dauber nest [mf]); (plants: bluebonnets, Indian paintbrush, miscellaneous vegetation, roadside vegetation, *Aphonostephus* sp., *Gaillardia pulchella*, *Monarda citriodora*, *Thelesperma* sp.); (soil/woodland: cedar litter, edge of woods, post oak savanna with pasture, sandy area, savanna, woods); (structures: brick wall)

####### Method.

Ballooning [f]; light trap; pitfall trap [mf] (in sand [m], in woods [f], near pond [m]); sweeping [mf]; tile trap [f]; yellow pan trap [m]

####### Eggs/spiderlings.

North-central Texas [58 eggs; 117 eggs] [Cokendolpher et al. 1979: 732]

####### Type.

Colorado

####### Etymology.

Latin, augmentation

####### Collection.

MSU, TAMU

###### Xysticus coloradensis

Taxon classificationAnimaliaAraneaeThomisidae

Bryant, 1930

Xysticus coloradensis Gertsch 1939a: 380, mf, desc. (figs 199, 206–207); Roberts 2001: 51; Roewer 1955: 917; Vogel 1970b: 26

####### Distribution.

El Paso, Potter

####### Locality.

Wildcat Bluff Nature Center

####### Time of activity.

Female (April)

####### Type.

Colorado, Fort Collins

####### Etymology.

locality (state)

###### Xysticus concursus

Taxon classificationAnimaliaAraneaeThomisidae

Gertsch, 1934

Xysticus concursus Agnew et al. 1985: 5; Bonnet 1959: 4862; Gertsch 1934b: 9, f, desc. (fig. 13); Gertsch 1939a: 381, mf, desc. (figs 198, 208–209); Gertsch 1953: 445; Jackman 1997: 170; Roewer 1955: 917; Vogel 1970b: 27; Young and Edwards 1990: 25

####### Distribution.

Childress, Coryell, Dickens, Erath, Hidalgo

####### Time of activity.

Male (July); female (July – September)

####### Habitat.

(crops: peanuts); (soil/woodland: post oak savanna with pasture)

####### Method.

pitfall trap [f]

####### Type.

Texas (female, Hidalgo Co., Edinburg, no date, S. Mulaik, holotype, AMNH)

####### Etymology.

Latin, resemble two other species (*Xysticus gulosus* Keyserling, 1880 and *Xysticus ontariensis* Emerton, 1919 = *Xysticus pellax* O. P.-Cambridge, 1894, Gertsch 1934b)

####### Collection.

TAMU

###### Xysticus elegans

Taxon classificationAnimaliaAraneaeThomisidae

Keyserling, 1880

Xysticus elegans Bonnet 1959: 4870; Breene et al. 1993c: 30, 48, 81, mf (figs 86A-C); Calixto et al. 2013: 185; Dean et al. 1982: 255; Gertsch 1939a: 372 [S], mf, desc. (figs 156–157, 192); Jackman 1997: 170; Young and Edwards 1990: 25
Xysticus limbatus Keyserling, 1880; Banks 1913: 177; Comstock 1912: 536; Marx 1890: 555

####### Distribution.

Hill, Jack, Montague, Robertson, Walker, Wichita

####### Locality.

Ellis Prison Unit, Holmes Pecan Orchard

####### Time of activity.

Female (March, November – December)

####### Habitat.

(crops: cotton); (orchard: pecan); (soil/woodland: shrub); (structures: homeowner bitten in shower)

####### Method.

cardboard band [f]

####### Type.

Georgia

####### Etymology.

Latin, elegant

####### Collection.

MSU, TAMU

###### Xysticus ellipticus

Taxon classificationAnimaliaAraneaeThomisidae

Turnbull, Dondale & Redner, 1965

Xysticus ellipticus Dondale and Redner 1978b: 228, mf, desc. (figs 690–693); Jackman 1997: 170; Turnbull et al. 1965: 1258 [new name], mf (figs 68, 71, 148, 151)
Synema obscurum Keyserling, 1880; Gertsch 1939a: 339, mf, desc. (figs 78–79, 93); Roewer 1955: 894; Vogel 1970b: 26

####### Distribution.

Jeff Davis

####### Type.

New Hampshire

####### Etymology.

Latin, epigynum elliptical

###### Xysticus emertoni

Taxon classificationAnimaliaAraneaeThomisidae

Keyserling, 1880

Xysticus emertoni Comstock 1940: 549, mf, desc. (figs 590, 601); Dondale and Redner 1978b: 206, mf, desc. (figs 419–421, 620–624); Gertsch 1939a: 374, mf, desc. (figs 158–159, 197); Gertsch 1953: 436; Jackman 1997: 170; Turnbull et al. 1965: 1249; Vogel 1970b: 27

####### Distribution.

Texas

####### Type.

Georgia

####### Etymology.

Person (arachnologist)

###### Xysticus ferox

Taxon classificationAnimaliaAraneaeThomisidae

(Hentz, 1847)

Xysticus ferox Agnew et al. 1985: 8, 11; Bradley 2013: 243; Calixto et al. 2013: 185; Cokendolpher et al. 1979: 732; Dean and Eger 1986: 142; Dondale and Redner 1978b: 212, mf, desc. (figs 640–644); Gertsch 1953: 446; Henderson 2007: 54, 78, 81, 85; Jackman 1997: 126, 170 (photo 40f); Kaston 1972: 244, desc. (fig. 555); Kaston 1978: 234, desc. (fig. 600); Reddell and Cokendolpher 2004: 94; Roberts 2001: 51; Turnbull et al. 1965: 1251; Vogel 1970b: 27; Yantis 2005: 67, 199, 202 [Gertsch 1939a: 385 [S], mf, desc. (figs 212–213, 225, 233)]
Xysticus transversatus Walckenaer, 1837; Rydzak and Killebrew 1982: 7
Xysticus stomachosus Keyserling, 1880; Jones 1936: 69

####### Distribution.

Anderson, Angelina, Bandera, Bastrop, Baylor, Bexar, Brazos, Burleson, Coryell, Dallas, Erath, Fort Bend, Goliad, Hays, Hood, Houston, Kerr, Leon, Madison, Montague, Potter, Robertson, San Patricio, Smith, Travis, Trinity, Uvalde, Walker, Wichita

####### Locality.

Angelina National Forest, Brazos Bend State Park, Goliad State Park, Holmes Pecan Orchard, Lick Creek Park, Lost Maples State Park, Texas A&M University Rangeland Area, Welder Wildlife Refuge, Wildcat Bluff Nature Center

####### Caves.

**Bexar** (Cave of the Bearded Tree)

####### Time of activity.

Male (January, March – May, August, October, December); female (March – August)

####### Habitat.

(landscape features: cave); (littoral: sedge meadow); (nest/prey: mud dauber nest); (orchard: pecan); (plants: bluebonnets, Indian paintbrush); (soil/woodland: buckeye-sycamore forest, disturbed habitat, edge of woods, field border, hardwood bottomland, *Juniperus* managed plot, leaf litter, live oak woodland, old field, pine woods [%: 66, 67, 80, 83, 84, 85, 95, 97, 99], post oak savanna with pasture, post oak woods [%: 41, 56, 77, 82, 92, 94, 96], riparian woodland, sandy area, under [juniper, oak], upland woods, woods); (structures: on bedroom floor, dark corner in house, in garage)

####### Method.

5 gallon bucket trap [mf]; blue pan trap [f]; cardboard band [f]; carrion pitfall trap [mf]; flight intercept trap [mf]; flight intercept trap elevated [f]; flight intercept trap on ground [mf]; pitfall trap [mf] (edge of woods [f], in leaves [mf], in woods [mf], under juniper [f], under oak [f]); ramp trap [f]; tile trap [f]; sweeping [mf]

####### Type.

United States

####### Etymology.

Latin, fierce

####### Collection.

DMNS, MSU, TAMU, TMM

###### Xysticus fraternus

Taxon classificationAnimaliaAraneaeThomisidae

Banks, 1895

Xysticus fraternus Dean and Eger 1986: 142; Henderson 2007: 35, 52–55, 57, 75, 78, 81, 85; Jackman 1997: 170; Yantis 2005: 67, 199, 202 [Gertsch 1939a: 384, mf, desc. (figs 214–215, 224)]

####### Distribution.

Angelina, Brazos, Houston, Hunt, Leon, Madison, Sabine, Smith, Travis, Tyler, Walker

####### Locality.

Angelina National Forest, Big Slough Wild Area, Big Thicket National Preserve, Huntsville State Park, Lick Creek Park, Tyler State Park

####### Time of activity.

Male (March – May); female (March – May)

####### Habitat.

(plants: bluebonnets, Indian paintbrush); (soil/woodland: beech magnolia forest, bottomland hardwood, disturbed habitat, hardwood litter, leaf litter, loblolly pine managed, pine woods [%: 88], post oak woods [%: 49, 71, 84, 91, 92, 96], post oak woodland, sedge, upland woods)

####### Method.

5 gallon bucket trap [mf]; blue pan trap [mf]; flight intercept trap [f]; flight intercept trap/malaise trap [mf]; malaise trap [mf]; pitfall trap [mf]; sweeping [mf]

####### Type.

New York, Long Island

####### Etymology.

Latin, brotherly

####### Collection.

DMNS, MSU, TAMU

###### Xysticus funestus

Taxon classificationAnimaliaAraneaeThomisidae

Keyserling, 1880

Xysticus funestus Agnew et al. 1985: 5, 10; Bonnet 1959: 4875; Breene et al. 1993c: 30, 48, 81, mf (figs 87A-C); Brown 1974: 238; Calixto et al. 2013: 185; Cokendolpher et al. 1979: 732; Dean et al. 1982: 255; Dondale and Redner 1978b: 211, mf, desc. (figs 635–639); Gertsch 1939a: 367 [S], mf, desc. (figs 162–163, 175); Gertsch 1953: 433; Jackman 1997: 126, 170; Kagan 1942: 48; Kagan 1943: 258; Kaston 1972: 245, desc. (fig. 556); Kaston 1978: 234, desc. (fig. 601); Milstead 1958: 446; Nyffeler et al. 1988a: 55; Reddell and Cokendolpher 2004: 94; Roewer 1955: 914 [S]; Turnbull et al. 1965: 1247; Vogel 1970b: 27; Yantis 2005: 67, 199, 202; Young and Edwards 1990: 25
Xysticus tumefactus (Walckenaer, 1837); Rapp 1984: 8
Xysticus nervosus Banks, 1892; Jones 1936: 69

####### Distribution.

Anderson, Archer, Baylor, Bexar, Brazos, Burleson, Cameron, Colorado, Comal, Comanche, Coryell, Dallas, Erath, Fort Bend, Galveston, Grimes, Hamilton, Harris, Henderson, Hidalgo, Houston, Jeff Davis, Kerr, Kimble, Lampasas, Lavaca, Leon, Madison, McLennan, Nacogdoches, Parker, Presidio, Robertson, Runnels, Sabine, Travis, Victoria, Walker, Wichita

####### Locality.

Anzalduas County Park, Attwater Prairie Chicken National Wildlife Refuge, Bentsen-Rio Grande Valley State Park, Bill Haney Pecan Orchard, Davis Mountains Resort, Ellis Prison Unit, Holmes Pecan Orchard, Lick Creek Park, Riley Estate, Sabal Palm Audubon Sanctuary, Santa Ana National Wildlife Refuge, Zilker Park

####### Caves.

**Bexar** (Lone Gunman Pit)

####### Time of activity.

Male (January – July, September – December); female (January – December)

####### Habitat.

(crops: cotton, peanuts); (grass: grass); (landscape features: cave); (littoral: salt marsh area, sedge meadow); (nest/prey: mud dauber nest [f]; stomach of *Cnemidophorus sacki*); (orchard: pecan); (plants: bluebonnets, miscellaneous vegetation); (soil/woodland: beech-magnolia forest, leaf litter, pine woods [%: 60, 67, 69, 73, 82, 88], post oak savanna with pasture, post oak woods [%: 74, 80, 84, 96], tree, upland deciduous forest, woods); (structures: house wall, indoors, on floor in building)

####### Method.

5 gallon bucket trap [mf]; beating [f]; cardboard band [mf]; D-Vac suction [f]; flight intercept trap [f]; flight intercept trap on ground [m]; fogging [m]; malaise trap [mf]; pitfall trap [mf] (in leaves [f]); suction trap [mf]; sweeping [mf]

####### Type.

Maryland, Baltimore

####### Etymology.

Latin, deadly

####### Collection.

DMNS, MSU, TAMU, TMM

###### Xysticus furtivus

Taxon classificationAnimaliaAraneaeThomisidae

Gertsch, 1936

Xysticus furtivus Bonnet 1959: 4876; Gertsch 1936: 15, mf, desc; Gertsch 1939a: 388, mf, desc. (figs 218–219, 227); Gertsch 1953: 450; Jackman 1997: 170; Roewer 1955: 919; Vogel 1970b: 27

####### Distribution.

Hidalgo, Kenedy, Milam

####### Locality.

Kenedy Ranch

####### Time of activity.

Male (March 25-April 18, April); female (March 1-April 2, April)

####### Habitat.

(littoral: sand dune area); (soil/woodland: oak savanna)

####### Method.

pitfall trap [mf]

####### Type.

Texas (male, Hidalgo Co., Edinburg, no date, S. Mulaik, holotype, AMNH)

####### Etymology.

Latin, concealed

####### Collection.

TAMU

###### Xysticus gulosus

Taxon classificationAnimaliaAraneaeThomisidae

Keyserling, 1880

Xysticus gulosus Agnew et al. 1985: 5; Bonnet 1959: 4877; Cokendolpher et al. 1979: 732; Gertsch 1939a: 353, mf, desc. (figs 140–141, 165); Jackman 1997: 170; Jones 1936: 69; Rapp 1984: 8; Vogel 1970b: 27; Young and Edwards 1990: 25

####### Distribution.

Brazos, Coryell, Dallas, El Paso, Erath, Fannin, Galveston, Kerr, Sutton, Travis, Wichita

####### Locality.

Zilker Park

####### Time of activity.

Male (March, October); female (February, April 26-May 2, July, October)

####### Habitat.

(crops: peanuts); (plants: miscellaneous vegetation); (soil/woodland: post oak savanna with pasture, sandy area)

####### Method.

pitfall trap [f]

####### Type.

Georgia

####### Etymology.

Latin, gluttonous

####### Collection.

MSU, TAMU

###### Xysticus lassanus

Taxon classificationAnimaliaAraneaeThomisidae

Chamberlin, 1925

Xysticus lassanus Agnew et al. 1985: 8; Broussard and Horner 2006: 255; Chamberlin 1925: 218, desc; Gertsch 1939a: 360, m, desc. (figs 124–125); Gertsch 1953: 431 [S]; Jackman 1997: 170; Milstead 1958: 446; Richman et al. 2011a: 49; Roewer 1955: 920; Vogel 1970b: 27
Xysticus coloradensis Bryant, 1930; Gertsch 1939a: 380, f (fig. 199)

####### Distribution.

Brewster, El Paso, Erath, Presidio, Roberts

####### Locality.

Chihuahuan desert, Dalquest Research Site, La Mota Mountains

####### Time of activity.

Female (April)

####### Habitat.

(nest/prey: stomach of *Cnemidophorus tessellatus*, *Geococcyx californicus*)

####### Method.

pitfall trap [mf]

####### Type.

Texas (male, Roberts Co., no date, no collector, holotype, MCZ)

####### Etymology.

Latin, faint

####### Collection.

MSU, TAMU

###### Xysticus locuples

Taxon classificationAnimaliaAraneaeThomisidae

Keyserling, 1880

Xysticus locuples Jackman 1997: 170 [Turnbull et al. 1965: 1245, mf (figs 30, 33, 112, 115, 168)]

####### Distribution.

Travis

####### Time of activity.

Female (March)

####### Habitat.

(soil/woodland: *Juniperus ashei*)

####### Method.

sweeping [f]

####### Type.

Colorado

####### Etymology.

Latin, substantial

####### Collection.

TAMU

###### Xysticus nevadensis

Taxon classificationAnimaliaAraneaeThomisidae

(Keyserling, 1880)

Xysticus nevadensis Dondale and Redner 1975a: 134 [T]; Jackman 1997: 170
Oxyptila nevadensis Keyserling, 1880; Gertsch 1953: 467, f, desc. (fig. 84); Vogel 1970b: 26 [not Hidalgo Co. record] [Gertsch 1939a: 347, mf, desc. (figs 112–113, 132)]

####### Distribution.

Kerr

####### Locality.

Raven Ranch

####### Time of activity.

Female (December)

####### Type.

Nevada

####### Etymology.

locality (state)

###### Xysticus paiutus

Taxon classificationAnimaliaAraneaeThomisidae

Gertsch, 1933

Xysticus paiutus [Gertsch 1953: 441, mf (figs 42–44)]

####### Distribution.

Hays, Knox

####### Time of activity.

Male (April, July – August); female (August)

####### Habitat.

(crops: cotton); (soil/woodland: *Juniperus* managed plot, post oak savanna with pasture)

####### Method.

Flight intercept trap [m]; pitfall trap [mf]

####### Type.

Utah, St. George

####### Etymology.

Indian tribe

####### Collection.

TAMU

###### Xysticus pellax

Taxon classificationAnimaliaAraneaeThomisidae

O. P.-Cambridge, 1894

Xysticus pellax Agnew et al. 1985: 5, 10; Cokendolpher et al. 1979: 732; Cokendolpher et al. 2008: 11, 56; Gertsch 1953: 421, m (figs 1–2); Jackman 1997: 170; Vogel 1970b: 27; Yantis 2005: 67, 199, 202; Young and Edwards 1990: 25 [Dondale and Redner 1978b: 181, mf, desc. (figs 547–553)]

####### Distribution.

Brazos, Brewster, Brown, Burleson, Carson, Comanche, Coryell, Erath, Jeff Davis, Leon, Madison, Polk, Rains, Travis, Wichita

####### Locality.

Pantex Lake

####### Time of activity.

Male (July, September – November); female (April – May, July, September – November)

####### Habitat.

(crops: peanuts); (grass: grassland); (littoral: near playa); (plants: miscellaneous vegetation, *Thelesperma* sp.); (soil/woodland: ground, pine woods [%: 77], post oak savanna with pasture, post oak woods [%: 43, 48, 75, 76, 85, 93])

####### Method.

5 gallon bucket trap [mf]; pitfall trap [mf]; sweeping [m]

####### Type.

Mexico, Guerrero, Amula

####### Etymology.

Latin, deceitful

####### Collection.

DMNS, MSU, TAMU

###### Xysticus punctatus

Taxon classificationAnimaliaAraneaeThomisidae

Keyserling, 1880

Xysticus punctatus Jackman 1997: 170 [Gertsch 1939a: 393 [S], mf, desc. (figs 236–237, 265)]
Xysticus formosus Banks, 1892; Brown 1974: 238

####### Distribution.

Nacogdoches

####### Time of activity.

Female (May)

####### Habitat.

(soil/woodland: falling from tree)

####### Type.

North Carolina

####### Etymology.

Latin, spotted with puncture-like spots

###### Xysticus robinsoni

Taxon classificationAnimaliaAraneaeThomisidae

Gertsch, 1953

Xysticus robinsoni Agnew et al. 1985: 8; Cokendolpher and Horner 1980: 109, f, desc. (figs 1–3); Cokendolpher and Reddell 2001b: 55; Cokendolpher et al. 1979: 732; Cokendolpher et al. 2008: 11, 56 (fig. 16); Gertsch 1953: 441, m, desc. (fig. 46); Jackman 1997: 126, 170; Knutson and Gilstrap 1989: 514; Roewer 1955: 915; Vogel 1967: 157; Vogel 1970b: 27
Xysticus orizaba Banks, 1898; Gertsch 1939a: 378 [Texas record]

####### Distribution.

Archer, Bell, Bosque, Brazos, Brown, Carson, Castro, Coryell, Erath, Fannin, Floyd, Jeff Davis, Lubbock, Montague, Palo Pinto, Taylor, Wichita

####### Locality.

Fort Hood, Lacuna Park, McDonald Observatory, Pantex Lake

####### Caves.

**Bell** (Keilman Cave [Fort Hood])

####### Time of activity.

Male (February – April, July – August); female (February – June, August)

####### Habitat.

(crops: corn, cotton, peanuts); (landscape features: cave); (littoral: near playa); (plants: Indian paintbrush, *Gaillardia pulchella*); (soil/woodland: edge of woods, ground litter, leaf litter, post oak savanna with pasture); (structures: outside house)

####### Method.

pitfall trap [mf] (edge of woods [m]); sweeping [mf]

####### Type.

Texas (male, Brazos Co., February 23, 1935, J. H. Robinson, holotype, AMNH)

####### Etymology.

Person (collector)

####### Collection.

MSU, TAMU, TMM

###### Xysticus texanus

Taxon classificationAnimaliaAraneaeThomisidae

Banks, 1904

Xysticus texanus Agnew et al. 1985: 5; Banks 1904: 112, f, desc; Banks 1910: 48; Breene et al. 1993c: 31, 80, mf (figs 85A-B); Brown 1974: 239; Calixto et al. 2013: 185; Cokendolpher et al. 1979: 733; Cokendolpher et al. 2008: 11, 56 (photo 49); Dean and Eger 1986: 142; Dean et al. 1982: 255; Gertsch 1939a: 375, mf, desc. (figs 186–187, 193); Gertsch 1953: 439; Jackman 1997: 126, 170; Petrunkevitch 1911: 441; Roberts 2001: 51; Roewer 1955: 915; Vogel 1970b: 27; Young and Edwards 1990: 25

####### Distribution.

Archer, Bexar, Brazos, Cameron, Carson, Comanche, Coryell, Dickens, Erath, Hidalgo, Llano, Lubbock, Nacogdoches, Palo Pinto, Potter, San Patricio, Walker, Wichita

####### Locality.

Bill Haney Pecan Orchard, Ellis Prison Unit, Pantex Lake, Robert J. Baker Ranch, Welder Wildlife Refuge, Wildcat Bluff Nature Center

####### Time of activity.

Male (April, July – September); female (April – May, July, September)

####### Habitat.

(crops: cotton, peanuts, sunflower); (littoral: near playa); (nest/prey: mud dauber nest [imm.]); (orchard: pecan); (plants: Indian paintbrush, miscellaneous vegetation, *Catalpa speciosa*); (soil/woodland: clay soil brushland, post oak savanna with pasture, redbud, *Albizzia julibrissin*); (structures: garage, indoors, on house)

####### Method.

Ballooning [imm.]; pitfall trap [m]; suction trap [pen f]; sweeping [f]

####### Type.

Texas (female, Bexar Co., San Antonio, no date, no collector, holotype, MCZ)

####### Etymology.

locality (state)

####### Collection.

JCC, MSU, TAMU, TTU

#### Family Titanoecidae Lehtinen, 1967

**Note.** raised to family (Lehtinen 1967: 270)

##### Genus *Titanoeca* Thorell, 1870

###### Titanoeca americana

Taxon classificationAnimaliaAraneaeTitanoecidae

Emerton, 1888

Titanoeca americana Agnew et al. 1985: 6; Brown 1974: 231; Cokendolpher et al. 2008: 11, 56 (photo 50); Henderson 2007: 55, 78, 81, 85; Jackman 1997: 100, desc., 170; Leech 1972: 100 [S], mf, desc. (figs 181–182, 377, 380)
Titanoeca americana anopla Chamberlin, 1947; Chamberlin 1947: 21, f (fig. 35); Roewer 1955: 1374; Vogel 1967: 19; Vogel 1970b: 3

####### Distribution.

Brazos, Cameron, Carson, Clay, Colorado, Coryell, Erath, Hays, Hidalgo, Jack, Jeff Davis, Jim Wells, Kenedy, Lubbock, Montague, San Patricio, Shelby (imm.)

####### Locality.

Attwater Prairie Chicken National Wildlife Refuge, Kenedy Ranch, Lick Creek Park, Pantex Lake

####### Time of activity.

Male (March – June, August); female (March, May – June)

####### Habitat.

(grass: grass, grassland); (landscape features: under rock); (littoral: near [playa, pond], sand dune area); (soil/woodland: leaf litter, post oak savanna with pasture, post oak woodland, sandy area, under [juniper, live oak, oak])

####### Method.

pitfall trap [mf] (in sand [m], near pond [m], under juniper [m], under oak [m]); yellow pan trap [m]

####### Type.

New Hampshire, Mount Monadnock

####### Etymology.

locality (country)

####### Collection.

JCC, MSU, TAMU

###### Titanoeca nigrella

Taxon classificationAnimaliaAraneaeTitanoecidae

(Chamberlin, 1919)

Titanoeca nigrella Jackman 1997: 170; Leech 1972: 96, mf, desc. (figs 177–178, 375, 379); Yantis 2005: 67, 198, 202

####### Distribution.

Archer, Houston, Jeff Davis, Leon, Nueces, San Patricio, Tarrant, Taylor, Travis, Trinity, Uvalde, Walker, Wichita

####### Locality.

Davis Mountains

####### Time of activity.

Male (March – May); female (March, September 27-October 6)

####### Habitat.

(landscape features: under rock); (soil/woodland: pine woods [%: 66, 82, 85, 86, 97], post oak woods [%: 71, 82, 91, 92, 93]); (structures: house)

####### Method.

5 gallon bucket trap [mf]

####### Type.

California, Claremont

####### Etymology.

Latin, color black

####### Collection.

MSU, TAMU

###### Titanoeca nivalis

Taxon classificationAnimaliaAraneaeTitanoecidae

Simon, 1874

Titanoeca nivalis Marusik 1995: 126 [S]
Titanoeca silvicola (Chamberlin and Ivie, 1947); Chamberlin 1947: 22, m (fig. 36); Vogel 1970b: 3 [Leech 1972: 98, mf, desc. (figs 179–180, 376, 381)]

####### Distribution.

Texas

####### Type.

Alps

####### Etymology.

Latin, referring to snow (as in snow white)

##### Family Trachelidae Simon, 1897

**Note.** raised to family (Ramírez 2014: 342)

##### Genus *Meriola* Banks, 1895

**Note.** transferred from Corinnidae (Ramírez 2014: 342)

###### Meriola decepta

Taxon classificationAnimaliaAraneaeTitanoecidae

Banks, 1895

Meriola decepta Calixto et al. 2013: 181, 188, 189; Cokendolpher et al. 2008: 8, 16 (fig. 4); Irungu 2007: 30; Knutson et al. 2010: 515; Pfannenstiel 2008a: 204; Platnick and Ewing 1995: 8 [T]
Meriola deceptus Banks, 1895; Jackman 1997: 162
Trachelas deceptus (Banks, 1895); Agnew et al. 1985: 4, 10; Breene et al. 1993a: 169; Breene et al. 1993c: 14, 47, 85, mf (figs 96A-B); Dean and Eger 1986: 142; Dean and Sterling 1987: 6; Dean and Sterling 1990: 403; Dean et al. 1982: 255; Kaston 1978: 213, desc.; Platnick and Shadab 1974b: 29 [S], mf, desc. (figs 103–106); Young and Edwards 1990: 16
Meriola inornata (Banks, 1901); Brown 1974: 233

####### Distribution.

Bastrop, Bexar, Blanco, Brazos, Brooks, Burleson, Caldwell, Cameron, Carson, Clay, Collingsworth, Comanche, Coryell, Dallas, Delta, Denton, Erath, Floyd, Goliad, Gonzales, Grayson, Hidalgo, Houston, Howard, Jeff Davis, Jim Wells, Kerr, Kleberg, Llano, Nacogdoches, Robertson, San Patricio, Taylor, Tom Green, Travis, Walker, Webb, Wichita, Williamson

####### Locality.

5-Eagle Ranch, Adriance Pecan Orchard, Bastrop State Park, Bill Haney Pecan Orchard, Browning Ranch, Ellis Prison Unit, Holmes Pecan Orchard, Lake Corpus Christi State Park, Palmetto State Park, Proctor Lake, Stiles Farm Foundation, Texas A&M University Rangeland Area, Welder Wildlife Refuge

####### Time of activity.

Male (January, March – November); female (February – December, December 22 – January 12)

####### Habitat.

(crops: cabbage, cotton, peanuts); (grass: grass, pasture); (landscape features: under rock); (littoral: near pond, playa); (orchard: citrus, pecan); (plants: emergent vegetation, Indian paintbrush, vegetation); (soil/woodland: forest litter, leaf litter, post oak savanna with pasture, saltcedar, sandy area, savanna, trees, woods); (structures: building at night, house, indoors)

####### Method.

Beating [m]; berlese funnel [f]; cardboard band [mf]; D-Vac suction [f]; fogging [f]; malaise trap [m]; pitfall trap [mf]; suction trap [mf]; sweeping [f]; yellow pan trap [m]

####### Type.

New York, Long Island, Sea Cliff

####### Etymology.

Latin, deceiving

####### Collection.

DMNS, MCZ, MSU, TAMU

##### Genus *Trachelas* L. Koch, 1872

**Note.** transferred from Corinnidae (Ramírez 2014: 342)

###### Trachelas mexicanus

Taxon classificationAnimaliaAraneaeCorinnidae

Banks, 1898

Trachelas mexicanus Agnew et al. 1985: 8; Calixto et al. 2013: 181, 185, 187; Jackman 1997: 162; Lombardini et al. 2005: 1378; Platnick and Shadab 1974a: 12, mf, desc. (figs 18–21, 46); Roberts 2001: 50

####### Distribution.

Bexar, Brazos, Brewster, Burleson, Cameron, Comanche, Erath, Goliad, Hale, Hays, Hidalgo, Howard, Hunt, Lubbock, Medina, Potter, Presidio, Robertson, Travis, Val Verde, Washington, Wichita

####### Locality.

Adriance Pecan Orchard, Bentsen-Rio Grande Valley State Park, Big Bend National Park, Bill Haney Pecan Orchard, Chinati Mountains, Frontera Audubon, Goliad State Park, Holmes Pecan Orchard, Lick Creek Park, Riley Estate, Somerville Lake, Storey Pecan Orchard, Wildcat Bluff Nature Center

####### Time of activity.

Male (January – December); female (January – December)

####### Habitat.

(crops: peanuts); (grass: grass); (orchard: grapefruit, orange, pecan, sour orange); (soil/woodland: *Juniperus* unmanaged plot, old field, post oak savanna with pasture, saltcedar, sandy area, trees/shrubs, under bark, *Quercus buckleyi*, *Quercus virginiana*, *Ulmus crassifolia*); (structures: in house)

####### Method.

Beating [mf]; cardboard band [mf]; flight intercept trap on ground [f]; irrigation tubing [f]; pitfall trap [mf]; sweeping [m]

####### Type.

Mexico, Nayarit

####### Etymology.

locality (country)

####### Collection.

DMNS, JCC, MSU, NMSU, TAMU

###### Trachelas similis

Taxon classificationAnimaliaAraneaeCorinnidae

F. O. P.-Cambridge, 1899

Trachelas similis Calixto et al. 2013: 181; Jackman 1997: 162; Kaston 1978: 213; Platnick and Shadab 1974a: 23, mf, desc. (figs 52–55); Rapp 1984: 7

####### Distribution.

Angelina, Brazos, Dallas, Fannin, Galveston, Gonzales, Hardin, Houston, Hunt, Jefferson, Liberty, Robertson, Rusk, Sabine, Walker

####### Locality.

Holmes Pecan Orchard, Lick Creek Park, Sam Houston National Forest, White Rock Lake

####### Time of activity.

Male (March, May, July – September, November – December); female (March, July – November)

####### Habitat.

(grass: grassy and shrub area); (littoral: sedge meadow); (orchard: pecan); (soil/woodland: beech magnolia forest, damp hardwood forest, bottomland forest, magnolia litter, sandy area, wooded area)

####### Method.

Berlese funnel [m]; cardboard band [mf]; flight intercept trap [mf]; malaise trap [f]; pitfall trap [m]; sweeping [f]

####### Type.

Mexico, Veracruz, Orizaba

####### Etymology.

Latin, similar to *Trachelas bulbosus* F. O. P.-Cambridge, 1899

####### Collection.

MCZ, MSU, TAMU

###### Trachelas tranquillus

Taxon classificationAnimaliaAraneaeCorinnidae

(Hentz, 1847)

Trachelas tranquillus Brown 1974: 233; Jackman 1997: 162; Trevino 2014: 11 [Platnick and Shadab 1974a: 8, mf, desc. (figs 1–9, 42–44)]

####### Distribution.

Bexar, Clay, Kerr, Nacogdoches, Palo Pinto, Tarrant, Travis, Webb, Wichita

####### Time of activity.

Male (April, July); female (February, April, July)

####### Habitat.

(plants: vegetation); (structures: in house)

####### Type.

New York, Long Island, Greenport

####### Etymology.

Latin, quiet, calm

####### Collection.

DMNS, MCZ, MSU

###### Trachelas volutus

Taxon classificationAnimaliaAraneaeCorinnidae

Gertsch, 1935

Trachelas volutus Breene et al. 1993a: 169; Breene et al. 1993c: 14, 47, 85, mf (figs 97A-B); Bumroongsook et al. 1992: 17; Chamberlin and Ivie 1935b: 41; Dean et al. 1982: 255; Gertsch 1935b: 13, mf, desc. (figs 27–28); Jackman 1997: 162; Liao et al. 1984: 410; Pfannenstiel 2008a: 204; Platnick and Shadab 1974a: 10, mf, desc. (figs 14–17, 45); Reddell and Cokendolpher 2004: 77; Roewer 1955: 589; Vincent and Frankie 1985: 380; Vogel 1970b: 6; Young and Edwards 1990: 16

####### Distribution.

Eastern 2/3 Texas; Bastrop, Bell, Bexar, Brazos, Brooks, Brown, Burleson, Caldwell, Cameron, Comanche, Dallas, Harris, Hidalgo, Kerr, Kleberg, La Salle, Llano, Lubbock, Medina, Nueces, San Patricio, Starr, Travis, Wichita

####### Locality.

Adriance Pecan Orchard, Bastrop State Park, Bill Haney Pecan Orchard, Lick Creek Park, Raven Ranch, Sabal Palm Audubon Sanctuary, Texas A&M University Rangeland Area, Vinson Pecan Farm

####### Caves.

**Bexar** (Surprise Sink)

####### Time of activity.

Male (January, March – July, October – November); female (January – August, September 25-October 2, October – December)

####### Habitat.

(crops: cotton); (grass: grass); (landscape features: cave); (orchard: citrus, pecan); (soil/woodland: live oak, old field, sandy area, tree bark, under bark, *Quercus buckleyi*, *Quercus virginiana*, *Ulmus crassifolia*)

####### Method.

Beating [f]; cardboard band [m]; irrigation tubing [f]; pitfall trap [mf]; sweeping [f]

####### Type.

Texas (male, Hidalgo Co., Edinburg, January 15, 1934, S. Mulaik, holotype, AMNH)

####### Etymology.

Latin, spiral

####### Collection.

DMNS, JCC, MCZ, MSU, TAMU, TMM, TTU

#### Family Uloboridae Thorell, 1869

**Note.** Species incorrectly reported from Texas

*Uloborus diversus* Marx, 1898; Kaston 1972: 75; Kaston 1978: 77 [not in Texas]

##### Genus *Hyptiotes* Walckenaer, 1837

###### Hyptiotes cavatus

Taxon classificationAnimaliaAraneaeUloboridae

(Hentz, 1847)

Hyptiotes cavatus Dondale et al. 2003: 36, mf, desc. (figs 19–24); Gertsch and Mulaik 1940: 334; Jackman 1997: 47, desc., 170; Kaston 1972: 76, desc. (fig. 172); Kaston 1978: 77, desc. (fig. 190); Muma and Gertsch 1964: 13, mf, desc. (figs 6–11, 13–17); Vogel 1970b: 28

####### Distribution.

East Texas; Panola, San Augustine, Travis, Tyler

####### Caves.

**Travis** (Dobie Shelter)

####### Habitat.

(landscape features: cave)

####### Type.

Alabama

####### Etymology.

Latin, caves

####### Collection.

TMM

###### Hyptiotes puebla

Taxon classificationAnimaliaAraneaeUloboridae

Muma & Gertsch, 1964

Hyptiotes puebla Jackman 1997: 170; Muma and Gertsch 1964: 14, mf, desc. (figs 18, 20, 23, 33–34); Vogel 1970b: 28

####### Distribution.

Brewster

####### Locality.

Big Bend National Park, Chisos Basin

####### Time of activity.

Female (September)

####### Type.

New Mexico, Camp Mary White

####### Etymology.

Spanish, house

##### Genus *Miagrammopes* O. P.-Cambridge, 1870

###### Miagrammopes mexicanus

Taxon classificationAnimaliaAraneaeUloboridae

O. P.-Cambridge, 1893

Miagrammopes mexicanus Bradley 2013: 246; Gertsch 1979: 144; Jackman 1997: 170; Muma and Gertsch 1964: 4 [S], f, desc. (figs 5, 12); Opell 2005: 253; Roth 1982: 50–1; Roth 1985: B-46–1; Roth 1994: 31; Vogel 1970b: 28
Miagrammopes lineatus O. P.-Cambridge, 1894; Bryant 1933: 171; Gertsch and Mulaik 1940: 334; Vogel 1970b: 28

####### Distribution.

Cameron

####### Locality.

Sabal Palm Audubon Sanctuary

####### Time of activity.

Female (February)

####### Habitat.

(soil/woodland: palm forest margin [resaca bank])

####### Type.

Mexico, Guerrero, Amula

[male known but not described, deposited at TAMU]

####### Etymology.

locality (country)

####### Collection.

TAMU

##### Genus *Octonoba* Opell, 1979

###### Octonoba sinensis

Taxon classificationAnimaliaAraneaeUloboridae

(Simon, 1880)

Octonoba sinensis Yoshida 1980: 58 [S]
Uloborus octonarius Muma, 1945 [Muma and Gertsch 1964: 38, mf, desc. (figs 35, 87–90)]
Octonoba octonaria (Muma, 1945); Opell 1979: 515 [T]
Octonoba octonarius (Muma, 1945); Peaslee and Peck 1983: 53

####### Distribution.

North-central Texas

####### Type.

China

####### Etymology.

New Latin, China

##### Genus *Philoponella* Mello-Leitão, 1917

###### Philoponella oweni

Taxon classificationAnimaliaAraneaeUloboridae

(Chamberlin, 1924)

Philoponella oweni Breene et al. 1993a: 169; Jackman 1997: 170; Lehtinen 1967: 258 [T]; Opell 1979: 536, mf, desc. (figs 255–258)
Uloborus oweni Chamberlin, 1924 [Muma and Gertsch 1964: 34, mf, desc. (figs 77–81)]

####### Distribution.

Hidalgo, Hudspeth, Wichita

####### Locality.

Bentsen-Rio Grande Valley State Park, Santa Ana National Wildlife Refuge

####### Time of activity.

Male (May); female (May, September – October)

####### Habitat.

(orchard: citrus)

####### Type.

Mexico, Baja California, Gulf of California, Marques Bay, Carmen Island

####### Etymology.

Person (collector, Virgil Owen)

####### Collection.

MSU, TAMU

###### Philoponella semiplumosa

Taxon classificationAnimaliaAraneaeUloboridae

(Simon, 1893)

Philoponella semiplumosa Jackman 1997: 170; Opell 1979: 534 [S], mf, desc. (figs 245–254); Opell 1983: 65
Uloborus variegatus O. P.-Cambridge, 1898; Gertsch and Mulaik 1940: 334; Muma and Gertsch 1964: 33, mf, desc. (figs 72–76); Roewer 1955: 1345; Vogel 1970b: 28

####### Distribution.

South Texas; Cameron, Hidalgo, Live Oak, Starr

####### Locality.

Bentsen-Rio Grande Valley State Park, Lake Corpus Christi, Piper’s Lake, Santa Ana National Wildlife Refuge

####### Time of activity.

Male (March – April, August); female (March, May – August, October – December)

####### Type.

Venezuela

####### Etymology.

Latin, half-feather

####### Collection.

DMNS, TAMU

##### Genus *Uloborus* Latreille, 1806

###### Uloborus campestratus

Taxon classificationAnimaliaAraneaeUloboridae

Simon, 1893

Uloborus campestratus [Opell 1979: 506 [S], mf, desc. (figs 148–156)]
Uloborus cinereus Muma & Gertsch, 1964 [Muma and Gertsch 1964: 28, mf, desc. (figs 52–56)]

####### Distribution.

Galveston, Wichita

####### Type.

Venezuela

####### Etymology.

Latin, referring to a field

####### Collection.

MSU

###### Uloborus glomosus

Taxon classificationAnimaliaAraneaeUloboridae

(Walckenaer, 1841)

Uloborus glomosus Agnew et al. 1985: 3; Breene et al. 1993a: 169; Breene et al. 1993b: 648; Breene et al. 1993c: 31, 48, 52, mf (figs 8A-C); Brown 1974: 239; Bumroongsook et al. 1992: 18; Dean and Eger 1986: 141; Dean and Sterling 1990: 401; Dean et al. 1982: 254; Dean et al. 1988: 286; Dondale et al. 2003: 41, mf, desc. (figs 31–35); Jackman 1997: 48, desc., 170 (photo 16b); Kaston 1972: 74, desc. (fig. 169); Kaston 1978: 76, desc. (fig. 187); Muma and Gertsch 1964: 22 [S], mf, desc. (figs 40–41, 44–45, 66–70); Nyffeler and Sterling 1994: 1295, 1298; Nyffeler et al. 1987c: 372; Nyffeler et al. 1988a: 55; Nyffeler et al. 1989: 374, 377; Rapp 1984: 3; Rice 1986: 124; Vogel 1970b: 28; Young and Edwards 1990: 25
Uloborus americanus Walckenaer, 1841; Bonnet 1959: 4759; Gertsch and Mulaik 1940: 335; Jones 1936: 69; Vogel 1970b: 28 [Texas records]
Uloborus mammeatus Hentz, 1850; McCook 1889: 176
Uloborus plumipes Emerton, 1888; Banks 1898b: 234

####### Distribution.

Eastern ½ Texas; Anderson, Archer, Atascosa, Bowie, Brazos, Brewster, Burleson, Cameron, Coryell, Dallas, Erath, Galveston, Goliad, Harris, Hidalgo, Houston, Hunt, Kerr, Lubbock, Montgomery, Nacogdoches, Newton, Polk, Presidio, Sabine, San Patricio, Starr, Travis, Tyler, Uvalde, Walker, Washington, Wichita, Zapata

####### Locality.

Adriance Pecan Orchard, Bentsen-Rio Grande Valley State Park, Big Bend National Park, Big Bend Ranch State Park, Brison Pecan Orchard, Chisos Mountains, Ellis Prison Unit, Frontera Audubon, Garner State Park, Kirby State Forest, La Gringa Resaca, Lake Corpus Christi State Park, Lake Tawakoni State Park, Lick Creek Park, Texas A&M University Rangeland Area

####### Time of activity.

Male (March 20-April 29, April – October); female (March – November)

####### Habitat.

(crops: cotton, peanuts, sugarcane); (grass: grass, grassland, grassy and shrub area, pasture); (littoral: woods); (nest/prey: mud dauber nest); (orchard: citrus, grapefruit, peach tree, pecan); (plants: bluebonnets, bush, miscellaneous vegetation); (soil/woodland: beech-magnolia forest, post oak savanna with pasture, woods, *Juniperus ashei*, *Ulmus crassifolia*); (web: web near creek); (structures: porch)

####### Method.

Beating [mf]; beating/sweeping [f]; D-Vac suction [mf]; flight intercept trap on ground [m]; malaise trap [m]; suction trap [m]; sweeping [mf]

####### Eggs/spiderlings.

Brazos [36 spiderlings] [TAMU]

####### Type.

Georgia

####### Etymology.

Latin, referring to a rounded body

####### Collection.

DMNS, JCC, MSU, TAMU

###### Uloborus segregatus

Taxon classificationAnimaliaAraneaeUloboridae

Gertsch, 1936

Uloborus segregatus Bonnet 1959: 4768; Gertsch 1936: 4, mf, desc. (fig. 7); Gertsch and Mulaik 1940: 335; Jackman 1997: 170; Muma and Gertsch 1964: 26, mf, desc. (figs 57–61); Opell 1979: 505, mf, desc. (figs 140–147); Opell 1983: 64; Roewer 1955: 1345; Vogel 1970b: 28

####### Distribution.

Hidalgo

####### Locality.

Bentsen-Rio Grande Valley State Park, Piper’s Lake

####### Time of activity.

Male (March, June, August – September); female (March, May – October)

####### Habitat.

(soil/woodland: punkwood)

####### Type.

Texas (male, Hidalgo Co., Edinburg, September 16, 1935, S. Mulaik, holotype, AMNH)

####### Etymology.

Latin, separated

####### Collection.

TAMU

#### Family Zorocratidae Dahl, 1913

##### Genus *Zorocrates* Simon, 1888

**Note.** transferred from Tengellidae to Zorocratidae (Griswold et al. 1999: 59) and to Zoropsidae (Polotow et al. 2015: 152)

###### Family Zoropsidae Bertkau, 1882

**Note.** family revalidated (Polotow et al. 2015: 141)

###### Genus *Lauricius* Simon, 1888

**Note.** transferred from Tengellidae (Polotow et al. 2015: 152)

####### Lauricius hooki

Taxon classificationAnimaliaAraneaeTengellidae

Gertsch, 1941

Lauricius hooki [Edwards 1958: 372, mf, desc. (figs 4–6, 18, 204)]

######## Distribution.

Brown

######## Type.

Arizona, White Mountains

######## Etymology.

Person (collector, Luther Hook)

######## Collection.

MSU

###### Genus *Zorocrates* Simon, 1888

**Note.** transferred from Zorocratidae (Polotow et al. 2015: 152)

####### Zorocrates aemulus

Taxon classificationAnimaliaAraneaeZorocratidae

Gertsch, 1935

Zorocrates aemulus Bonnet 1959: 4990; Comstock 1940: 302, desc.; Gertsch 1935a: 23, mf, desc. (figs 31–32) [see note below]; Jackman 1997: 168; Platnick and Ubick 2007: 38, mf, desc. (figs 103–107); Reddell 1965: 177; Roewer 1955: 1284; Vogel 1970b: 28

######## Distribution.

Hidalgo, Kerr, Starr, Uvalde, Val Verde, Wichita, Zapata

######## Locality.

Raven Ranch

######## Caves.

**Uvalde** (Burial Cave); **Val Verde** (Wren Cave)

######## Time of activity.

Male (January – February, April – May, November); female (January, August, October – November)

######## Habitat.

(landscape features: cave, under rock); (soil/woodland: woods); (structures: brick yard)

######## Method.

pitfall trap [m] (in woods [m])

######## Type.

Texas (male, Starr Co., 0.5 mile E Rio Grande City, November 11, 1934, S. Mulaik, holotype, AMNH)

######## Etymology.

Latin, emulating or rivaling

######## Collection.

TAMU, TMM

######## Note.

32 miles E Laredo should be 32 miles SE Laredo in Zapata Co. based on other records from this date.

####### Zorocrates alternatus

Taxon classificationAnimaliaAraneaeZorocratidae

Gertsch & Davis, 1936

Zorocrates alternatus Bonnet 1959: 4990; Gertsch and Davis 1936: 14, mf, desc. (figs 18–19); Jackman 1997: 168; Platnick and Ubick 2007: 23, mf, desc. (figs 53–57); Roewer 1955: 1284; Vogel 1970b: 28

######## Distribution.

Cameron

######## Locality.

Sabal Palm Audubon Sanctuary

######## Time of activity.

Male (“January – March”, November – December); female (April – May, December)

######## Habitat.

(soil/woodland: palm forest, palm grove)

######## Method.

carrion trap [f]

######## Type.

Texas (male, Cameron Co., E Harlingen, January-March, 1936, L. I. Davis, holotype, AMNH)

######## Etymology.

Latin, resembles *Zorocrates aemulus* Gertsch, 1935 in appearance but differs

######## Collection.

TAMU

####### Zorocrates karli

Taxon classificationAnimaliaAraneaeZorocratidae

Gertsch & Riechert, 1976

Zorocrates karli [Platnick and Ubick 2007: 37, mf, desc. (figs 98–102)]

######## Distribution.

Brewster, Presidio

######## Type.

New Mexico, Lincoln Co.

######## Etymology.

Person (Named for the late Karl Riechert, father of the second author, Gertsch and Riechert 1976).

######## Collection.

MSU

####### Zorocrates terrell

Taxon classificationAnimaliaAraneaeZorocratidae

Platnick & Ubick, 2007

Zorocrates terrell Platnick and Ubick 2007: 29, mf, desc. (figs 73–77)

######## Distribution.

Terrell

######## Type.

Texas (female, Terrell Co., 10 miles SE Sanderson, no date, no collector, holotype, AMNH)

######## Etymology.

locality (The specific name is a noun in apposition taken from the type locality, Platnick and Ubick 2007).

####### Zorocrates unicolor

Taxon classificationAnimaliaAraneaeZorocratidae

(Banks, 1901)

Zorocrates unicolor Bradley 2013: 250; Platnick and Ubick 2007: 8 [S], mf, desc. (figs 6–10)
Zorocrates isolatus Gertsch and Davis, 1936; Bonnet 1959: 4990; Gertsch 1939b: 25; Gertsch and Davis 1936: 16, imm. f, desc.; Jackman 1997: 168; Ramirez 2014: 374; Roewer 1955: 1284; Vogel 1970b: 28
Zorocrates sp.; Griswold et al. 2005: 93

######## Distribution.

Brewster

######## Locality.

Big Bend National Park, Chisos Basin, Chisos Mountains

######## Time of activity.

Male (August – September); female (May, August – September)

######## Type.

Arizona, Santa Rita Mountains

######## Etymology.

Latin, one color

## Footnotes

1
*Elaver dorotheae* (Gertsch, 1935) is a synonym of *Elaver wheeleri* (Roewer, 1933) see Saturnino and Bonaldo (2015).

## Appendix

### Contents

Number of species by county (total of 254) 564

Localities with county (number of species) 565

List of spiders in caves by county 570

List of spiders in caves 601

Spiders in parks 606

National forests 606

National wildlife refuges 607

National (other areas) 614

State forests 618

State parks 619

Wildlife management areas 636

Other 637

Prairie study 650

Table A1. Number of spiders at 3 sites by year. 651

Table A2. Number of species at three sites by year. 651

Table A3. Species and measurement ranges in millimeters by sex (male, female). 652

Data for Barr, Burleson Co. 656

Data for C3, Coryell Co. 666

Data for Pruitt, Coryell Co. 677

Attwater Prairie Chicken National Wildlife Refuge (Colorado Co.), 2006–2009 687

Table A4. Number of species. 687

Golden Cheeked Warbler Project 690

Table A5. Sex collected by tree species 690

Table A6. Number of specimens by family 693

Species from various elevations in Texas 693

### Number of species by county (total of 254).

**Table:** Number of species by county (total of 254).

No. of species	No. of counties	
0	2	Cochran, Sherman
1–25	163	Andrews-5, Aransas-23, Armstrong-2, Austin-8, Bailey-5, Borden-8, Bosque-18, Bowie-8, Briscoe-10, Brooks-18, Caldwell-20, Calhoun-6, Callahan-1, Camp-3, Cass-5, Castro-4, Chambers-4, Cherokee-11, Childress-9, Coke-3, Coleman-15, Collingsworth-6, Concho-9, Cooke-12, Cottle-6, Crane-1, Crockett-16, Crosby-6, Dallam-12, Dawson-1, Deaf Smith-2, DeWitt-16, Delta-18, Dickens-16, Dimmit-6, Donley-3, Duval-10, Eastland-10, Ector-6, Ellis-25, Falls-17, Fisher-2, Floyd-20, Foard-4, Franklin-2, Freestone-15, Gaines-9, Garza-9, Gillespie-22, Glasscock-2, Gray-4, Gregg-3, Guadalupe-3, Hale-10, Hall-2, Hamilton-11, Hansford-1, Hardin-15, Harrison-14, Hartley-2, Haskell-9, Hemphill-3, Henderson-16, Hill-13, Hockley-4, Hood-13, Hopkins-4, Hudspeth-18, Hutchinson-6, Irion-4, Jack-14, Jackson-2, Jasper-24, Jim Hogg-1, Johnson-6, Jones-8, Karnes-6, Kaufman-20, Kent-5, King-7, Kinney-11, La Salle-10, Lamar-3, Lamb-1, Lampasas-15, Lavaca-13, Lee-12, Liberty-20, Limestone-13, Lipscomb-4, Live Oak-5, Loving-2, Lynn-5, Marion-7, Martin-14, Mason-12, Matagorda-12, Maverick-7, McCulloch-6, McMullen-6, Medina-24, Menard-8, Midland-8, Milam-8, Mills-3, Mitchell-8, Moore-1, Morris-2, Motley-5, Navarro-11, Newton-12, Nolan-11, Ochiltree-1, Oldham-4, Orange-9, Palo Pinto-23, Panola-16, Parker-8, Parmer-3, Pecos-20, Rains-11, Reagan-9, Real-16, Red River-5, Reeves-19, Refugio-9, Roberts-3, Rockwall-2, Runnels-14, Rusk-11, San Augustine-8, San Jacinto-14, San Saba-25, Schleicher-6, Scurry-25, Shackelford-3, Shelby-20, Somervell-10, Stephens-5, Sterling-4, Stonewall-3, Swisher-3, Tarrant-21, Terry-7, Throckmorton-3, Titus-13, Upshur-4, Upton-5, Van Zandt-9, Waller-7, Ward-10, Washington-19, Wharton-21, Wheeler-10, Wilbarger-19, Willacy-22, Wilson-9, Winkler-6, Wise-10, Wood-10, Yoakum-2, Young-20, Zavala-9
26–49	38	Angelina-47, Atascosa-34, Bandera-46, Baylor-39, Bee-24, Blanco-37, Brazoria-27, Burnet-33, Collin-32, Culberson-43, Edwards-30, El Paso-37, Fannin-35, Fayette-28, Fort Bend-31, Frio-28, Goliad-31, Gonzales-37, Grayson-45, Grimes-26, Hardeman-30, Jim Wells-38, Kendall-32, Kimble-37, Knox-28, Madison-35, McLennan-35, Montgomery-41, Polk-33, Randall-32, Smith-26, Sutton-39, Taylor-29, Tom Green-33, Trinity-34, Tyler-49, Victoria-34
50–99	30	Anderson-67, Bastrop-75, Bell-65, Brown-87, Carson-64, Clay-77, Comal-56, Denton-56, Galveston-67, Harris-83, Hays-88, Howard-60, Hunt-69, Jeff Davis-58, Jefferson-52, Kenedy-50, Kleberg-52, Leon-67, Llano-75, Lubbock-58, Montague-51, Nueces-72, Potter-60, Sabine-50, Starr-87, Terrell-50, Uvalde-71, Val Verde-81, Webb-89, Williamson-94, Zapata-55
100–199	14	Archer-119, Bexar-134, Brewster-163, Burleson-183, Colorado-115, Comanche-137, Coryell-174, Dallas-174, Houston-131, Kerr-160, Nacogdoches-117, Presidio-124, Robertson-128, San Patricio-138
200+	7	Brazos-323, Cameron-268, Erath-265, Hidalgo-340, Travis-314, Walker-200, Wichita-282

### Localities with County (number of species)

5-Eagle Ranch (36) Burleson

Adriance Pecan Orchard (32) Burleson

Amistad National Recreational Area (1) Val Verde

Angelina National Forest (24) Angelina

Anzalduas County Park (7) Hidalgo

Aransas National Wildlife Refuge (1) Aransas

Arkansas Bend Park (2) Travis

Ascarate Lake (1) El Paso

Attwater Prairie Chicken National Wildlife Refuge (102) Colorado

Bamburger Ranch Chiroptorium (2) Blanco

Bastrop State Park (21) Bastrop

Bateman Ranch (1) King

Benbrook-Grissom Ranch (1) Tarrant

Bentsen-Rio Grande Valley State Park (79) Hidalgo

Big Bend National Park (69) Brewster

Big Bend Ranch State Park (34) Presidio

Big Creek Scenic Area (1) San Jacinto

Big Slough Wild Area (8) Houston

Big Thicket National Preserve (11) Tyler

Big Tree-Vine Association (6) Cameron

Bill Haney Pecan Orchard (92) Comanche

Black Gap Wildlife Management Area (8) Brewster

Blackstone Ranch (10) Terrell

Brazoria National Wildlife Refuge (1) Brazoria

Brazos Bend State Park (8) Fort Bend

Brison Pecan Orchard (6) Burleson

Browning Ranch (18) Blanco

Buddy Adams Pecan Orchard (2) San Saba

Buescher State Park (6) Bastrop

Buffalo Lakes (2) Lubbock

Buffalo Lake (1) Wichita

Caddo Lake State Park (2) Harrison

Caine’s Ranch (1) Travis

Camp Arrowmoon (1) Robertson

Camp Bullis (18) Bexar, Comal

Camp Chrysalis (1) Kerr

Camp Tonkawa (3) McLennan

Canoncita Ranch (4) Randall

Caprock Canyons State Park (2) Briscoe

Chaparral Wildlife Management Area (5) Dimmit

Chihuahuan desert (49) Brewster

Chinati Mountains (1) Presidio

Chisos Basin (38) Brewster

Chisos Mountains (42) Brewster

Chisos Pass (1) Brewster

Cleburne Lake (1) Johnson

Comstock Railroad Tunnel (1) Val Verde

Corpus Christi Botanical Gardens (1) Nueces

Corpus Christi State Park (1) San Patricio

Crazy Cat Mountains (1) El Paso

Dalquest Research Site (48) Presidio

Davis Mountains (11) Jeff Davis

Davis Mountains Resort (6) Jeff Davis

Davy Crockett National Forest (2) Angelina

Decker’s Prairie (2) Montgomery

El Rancho Cima Scout Camp (1) Hays

Ellis Prison Unit (149) Walker

Ellison Brite Ranch (1) Val Verde

Enchanted Rock (1) Llano

Engeling Wolf Management Area (1) Anderson

Estero Llano Grande State Park (13) Hidalgo

Falcon State Park (14) Starr/Zapata

Flat Creek Ranch (1) Blanco

Fort Hancock (1) Hudspeth

Fort Hood (30) Bell

Fort Lancaster (1) Crockett

Fort Parker State Park (3) Limestone

Fort Sam Houston (2) Bexar

Fort Sill Recreation Area (1) Palo Pinto

Franklin Mountains (7) El Paso

Fresnos Resaca (1) Cameron

Frio State Park (1) Frio

Frontera Audubon (28) Hidalgo

Galveston Island State Park (20) Galveston

Garner State Park (30) Uvalde

Goliad State Park (9) Goliad

Goose Island State Park (9) Aransas

Gorman Falls (1) San Saba

Green Island Bird Refuge (9) Cameron

Grissom Ranch (1) Tarrant

Guadalupe Mountains (2) Culberson

Guadalupe Mountains National Park (5) Culberson

Guadalupe Pass (2) Hudspeth

Hagerman National Wildlife Refuge (2) Grayson

Hoblitzelle Farms (4) Hidalgo

Holmes Pecan Orchard (119) Robertson

Honey Creek Ranch (1) Comal

Horne Ranch (12) Coleman

Hoskins Mound (1) Brazoria

Houston Zoo (1) Harris

Huntsville State Park (6) Walker

Indio Mountains (1) Hudspeth

Indio Mountain Research Station (1) Hudspeth

Inks Lake State Park (9) Burnet

Iron Wheel Mesa (1) Hays

Johnson Ranch (3) Hutchinson

Jones State Forest (15) Montgomery

Kenedy Ranch (22) Kenedy

Kirby State Forest (29) Tyler

La Gringa Resaca (3) Cameron

La Mesa Ranch (2) Webb

La Mota Mountains (8) Presidio

Lackland Air Force Base (1) Bexar

Lacuna Park (13) Bosque

Laguna Atascosa National Wildlife Refuge (22) Cameron

Laguna Madre (13) Cameron

Lake Abilene (1) Taylor

Lake Amon Carter (1) Montague

Lake Arrowhead State Park (2) Clay

Lake Austin (1) Travis

Lake Ballinger (1) Runnels

Lake Buchanan (4) Burnet

Lake Corpus Christi (2) San Patricio

Lake Corpus Christi Dam (1) San Patricio

Lake Corpus Christi State Park (19) San Patricio

Lake Creek (1) Delta

Lake Dallas (5) Denton

Lake Grapevine (1) Dallas

Lake Kickapoo (3) Archer

Lake Limestone (1) Limestone

Lake McClellan (1) Carson

Lake McKenzie Park (1) Briscoe

Lake Meredith (6) Potter

Lake Meredith National Recreation Area (7) Hutchinson, Moore, Potter

Lake Normangee (1) Madison

Lake Rayburn (2) Nacogdoches

Lake Somerville State Park (9) Lee

Lake Striker (2) Cherokee

Lake Tanglewood (2) Randall

Lake Tawakoni State Park (26) Hunt

Lake Texoma (1) Grayson

Lake Thomas (24) Scurry

Lake Travis (4) Travis

Lake Wichita (8) Wichita

Lakeside Park South (3) Dallas

Landa Park Estates (1) Comal

Lick Creek Park (179) Brazos

Llano City Lake Park (1) Llano

Lockhart State Park (4) Caldwell

Lomita Ranch (2) Hidalgo

Lost Maples State Park (18) Bandera

Love Creek Ranch (1) Bandera

Lower Rio Grande Valley National Wildlife Refuge (21) Cameron, Hidalgo

Mansfield Dam (1) Travis

Mansfield Dam Park (1) Travis

Marneldo Ranch (1) Uvalde

McDonald Observatory (2) Jeff Davis

McKelvey Park (7) Cameron

Matador Wildlife Management Area (3) Cottle

Medicine Mounds Ranch (18) Hardeman

Mill Creek Cove (1) Sabine

Mo Ranch (1) Kerr

Monahans Sandhills State Park (2) Ward

Montgomery Ranch (1) Floyd

Moon Rocks Ranch (1) Burnet

Mount Barker (2) Travis

Mount Locke Observatory (2) Jeff Davis

Muleshoe National Wildlife Refuge (1) Bailey

Nabor’s Lake (11) Comanche

Nance Ranch (2) Randall

Nash Prairie (6) Brazoria

NK Ranch (27) Brazos

Padre Island (3) Cameron

Padre Island National Seashore (2) Kenedy

Palmetto State Park (35) Gonzales

Palo Duro Canyon (4) Randall

Palo Duro Canyon State Park (9) Randall

Pantex Lake (20) Carson

Pantex Lake (edge) (7) Carson

Pantex Plant (21) Carson

Parson’s Slough (1) Kaufman

Pedernales Falls State Park (2) Blanco

Perkins Scout Reservation (1) Wichita

Pioneer Park (1) Nacogdoches

Piper’s Lake (3) Hidalgo

Pollito Lake (1) San Patricio

Proctor Lake (24) Comanche

Ramsey Nature Park (7) Cameron

Ramsey Prison Farm (10) Brazoria

Raven Ranch (38) Kerr

Reimers Ranch Park (1) Travis

Resaca de la Palma State Park (20) Cameron

Riley Estate (24) Brazos

Rita Blanca Lake (1) Dallam

Robert J. Baker Ranch (2) Dickens

Russell Farm (28) Cameron

Sabal Palm Audubon Sanctuary (61) Cameron

Sabine National Forest (1) Sabine

Sam Houston National Forest (19) Walker

Sam Houston State Park (2) Walker

Santa Ana National Wildlife Refuge (53) Hidalgo

Sattler and Hoffman Ranch (1) Medina

Seminole Canyon State Park (20) Val Verde

Sheppard Air Force Base (1) Wichita

Shipp Farm (1) Wichita

Shoshone Park (1) Archer

Signal Peak (1) Hudspeth

Somerville Lake (17) Burleson

South Padre Island (18) Cameron

Starnes Island (1) Travis

Stetz Pecan Orchard (1) Brazos

Stiles Farm Foundation (40) Williamson

Stiles Ranch (1) Wheeler

Stockton Plateau (1) Terrell

Storey Pecan Orchard (10) Burleson

Stubblefield Lake (17) Walker

Stubblefield Lake Recreation Area (2) Walker

Texas A&M University Rangeland Area (77) Brazos

Thurmond Lake (1) Brazoria

Tom Mays Memorial Park (1) El Paso

Travis Park (1) Travis

Tyler State Park (8) Smith

Valley Botanical Garden (1) Hidalgo

Vinson Pecan Farm (3) Medina

W. J. Wagoneer Estate (1) Wilbarger

Welder Wildlife Refuge (54) San Patricio

White Rock Lake (9) Dallas

Wildcat Bluff Nature Center (50) Potter

Williams Lake (1) Matagorda

Zilker Park (15) Travis

### List of Spiders in Caves by County

Bandera, Bell, Bexar, Blanco, Brewster, Burnet, Childress, Collingsworth, Comal, Coryell, Crockett, Culberson, Edwards, El Paso, Gillespie, Hardeman, Hays, Howard, Irion, Jeff Davis, Kendall, Kerr, Kimble, King, Kinney, Lampasas, Llano, Mason, Medina, Menard, Pecos, Presidio, Randall, Reagan, Real, San Saba, Schleicher, Stonewall, Sutton, Terrell, Travis, Uvalde, Val Verde, Ward, Washington, Wheeler, Williamson

Note: caves with ? in front of name are questionable records.

**Table:** List of Spiders in Caves by County

**Bandera**	
Albino Bat Cave	*Eidmannella rostrata* Gertsch
Bob Clark Cave	*Agyneta llanoensis* (Gertsch & Davis)
Can Creek Cave No. 1	*Eidmannella rostrata* Gertsch
Charity Cave	*Eidmannella rostrata* Gertsch
Emmett Wilson Cave	*Cicurina varians* Gertsch & Mulaik
Fog Fissure	*Cicurina mckenziei* Gertsch, *Cicurina varians* Gertsch & Mulaik, *Eidmannella rostrata* Gertsch
Fossil Cave	*Cicurina bandera* Gertsch, *Cicurina varians* Gertsch & Mulaik, *Eidmannella rostrata* Gertsch
Garrison Hilltop Cave	*Cicurina varians* Gertsch & Mulaik, *Eidmannella rostrata* Gertsch, *Gaucelmus augustinus* Keyserling, *Metellina mimetoides* Chamberlin & Ivie
Haby Salamander Cave	*Eidmannella rostrata* Gertsch, *Gaucelmus augustinus* Keyserling
Haby Swallow Cave	*Cryptachaea porteri* (Banks)
Haby Water Cave	*Eidmannella rostrata* Gertsch, *Gaucelmus augustinus* Keyserling
Harvestman Cave [Hill Country State Natural Area]	*Tayshaneta valverdae* (Gertsch)
Keese Cave	*Cryptachaea porteri* (Banks)
Melanie’s Cave [Hill Country State Natural Area]	*Tayshaneta valverdae* (Gertsch)
Mueller Cave	*Gaucelmus augustinus* Keyserling
Station “C” Cave No. 1	*Cicurina varians* Gertsch & Mulaik
Station “C” Cave	*Cicurina sprousei* Gertsch
Sutherland Hollow Cave	*Cicurina obscura* Gertsch, *Gaucelmus augustinus* Keyserling
Tucker’s Fissure	*Metellina mimetoides* Chamberlin & Ivie
Tucker’s Fissure Cave	*Physocyclus enaulus* Crosby
**Bell**	
Adam’s Gold Mine	*Cicurina varians* Gertsch & Mulaik, *Gaucelmus augustinus* Keyserling, *Dolomedes scriptus* Hentz, *Tetragnatha elongata* Walckenaer
Afternoon Cave [Fort Hood]	*Agyneta llanoensis* (Gertsch & Davis)
Awesome Entrance Cave	*Agyneta llanoensis* (Gertsch & Davis)
Big Crevice [Fort Hood]	*Hahnia flaviceps* Emerton, *Agyneta llanoensis* (Gertsch & Davis), *Mermessus albulus* (Zorsch & Crosby)
Black Cave	*Cicurina varians* Gertsch & Mulaik
Blue Bottle Sink	*Agyneta llanoensis* (Gertsch & Davis)
Blue Green Hole Cave	*Agyneta llanoensis* (Gertsch & Davis)
Boca Verde Cave	*Agyneta llanoensis* (Gertsch & Davis)
Born Again Cave	*Agyneta llanoensis* (Gertsch & Davis)
Buchanan Cave	*Cicurina caliga* Cokendolpher & Reddell, *Cicurina hoodensis* Cokendolpher & Reddell, *Agyneta llanoensis* (Gertsch & Davis)
Bumelia Well Cave	*Agyneta llanoensis* (Gertsch & Davis)
C. B. Cave	*Agyneta llanoensis* (Gertsch & Davis), *Leucauge venusta* (Walckenaer)
Camp 6 Cave No. 1 [Fort Hood]	*Cicurina hoodensis* Cokendolpher & Reddell, *Cicurina varians* Gertsch & Mulaik, *Tayshaneta paraconcinna* (Cokendolpher & Reddell), *Agyneta llanoensis* (Gertsch & Davis), *Eidmannella pallida* (Emerton), *Gaucelmus augustinus* Keyserling, *Tidarren sisyphoides* (Walckenaer)
Canyon Side Sink [Fort Hood]	*Hypsosinga funebris* (Keyserling)
Cellular Cave	*Agyneta llanoensis* (Gertsch & Davis)
Chupacabra Pit Cave	*Agyneta llanoensis* (Gertsch & Davis)
Cicurina Cave	*Agyneta llanoensis* (Gertsch & Davis)
Copperdead Cave	*Agyneta llanoensis* (Gertsch & Davis)
Corkscrew Cave	*Agyneta llanoensis* (Gertsch & Davis)
Coyote Den Cave	*Schizocosa saltatrix* (Hentz), *Neospintharus furcatus* (O. P.-Cambridge), *Tidarren sisyphoides* (Walckenaer)
Craggy Rock Cave	*Agyneta llanoensis* (Gertsch & Davis)
Cub Cave [Fort Hood]	*Gnaphosa fontinalis* Keyserling, *Cryptachaea porteri* (Banks)
Deceiving Sink	*Agyneta llanoensis* (Gertsch & Davis)
Deep in Dis Bear Cave	*Agyneta llanoensis* (Gertsch & Davis)
Dual Sinks Cave	*Agyneta llanoensis* (Gertsch & Davis)
Dying Oak Cave	*Agyneta llanoensis* (Gertsch & Davis)
Endless Pit Cave	*Agyneta llanoensis* (Gertsch & Davis)
Estes Cave	*Agyneta llanoensis* (Gertsch & Davis)
Falling Hat Cave	*Agyneta llanoensis* (Gertsch & Davis)
Falling Turtle Cave	*Agyneta llanoensis* (Gertsch & Davis)
Fellers Cave [Fort Hood]	*Agyneta llanoensis* (Gertsch & Davis), *Erigone autumnalis* Emerton
Figure 8 Cave [Fort Hood]	*Cicurina varians* Gertsch & Mulaik, *Agyneta llanoensis* (Gertsch & Davis), *Mermessus albulus* (Zorsch & Crosby), *Tayshaneta paraconcinna* (Cokendolpher & Reddell), *Eidmannella pallida* (Emerton)
Fire Break Cave	*Agyneta llanoensis* (Gertsch & Davis)
Fools Cave [Fort Hood]	*Cicurina varians* Gertsch & Mulaik, *Agyneta llanoensis* (Gertsch & Davis), *Mermessus albulus* (Zorsch & Crosby)
Forbidden Chasm Cave	*Agyneta llanoensis* (Gertsch & Davis)
Forgotten Cave	*Agyneta llanoensis* (Gertsch & Davis)
Forgotten Sink	*Agyneta llanoensis* (Gertsch & Davis)
Geocache Cave	*Agyneta llanoensis* (Gertsch & Davis)
Gnarla Cave	*Cicurina varians* Gertsch & Mulaik, *Agyneta llanoensis* (Gertsch & Davis), *Cryptachaea porteri* (Banks)
Green Carpet Cave	*Agyneta llanoensis* (Gertsch & Davis)
Hammer Crack Cave	*Agyneta llanoensis* (Gertsch & Davis)
Hidden Pit Cave [Fort Hood]	*Mermessus albulus* (Zorsch & Crosby), *Tayshaneta paraconcinna* (Cokendolpher & Reddell)
Hidey Ho Cave	*Agyneta llanoensis* (Gertsch & Davis)
Hill’s Cave	*Cicurina varians* Gertsch & Mulaik, *Eidmannella pallida* (Emerton), *Cryptachaea porteri* (Banks)
Hope Well Sink	*Agyneta llanoensis* (Gertsch & Davis)
Humpty Cave	*Agyneta llanoensis* (Gertsch & Davis)
Jagged Walls Cave [Fort Hood]	*Cicurina varians* Gertsch & Mulaik, *Hahnia flaviceps* Emerton, *Agyneta llanoensis* (Gertsch & Davis)
Keilman Cave [Fort Hood]	*Mermessus albulus* (Zorsch & Crosby), *Mermessus maculatus* (Banks), *Rabidosa rabida* (Walckenaer), *Schizocosa saltatrix* (Hentz), *Leucauge venusta* (Walckenaer), *Xysticus robinsoni* Gertsch
L. Z. Sid Cave	*Agyneta llanoensis* (Gertsch & Davis)
Legless Visitor Cave	*Agyneta llanoensis* (Gertsch & Davis)
Leopard Frog Cave	*Agyneta llanoensis* (Gertsch & Davis)
Long Joint Sink [Fort Hood]	*Agyneta llanoensis* (Gertsch & Davis), *Neriene radiata* (Walckenaer)
Lost Chasm Cave	*Agyneta llanoensis* (Gertsch & Davis)
Lucky Rock Cave	*Agyneta llanoensis* (Gertsch & Davis)
Lunch Counter Cave [Fort Hood]	*Schizocosa saltatrix* (Hentz), *Cryptachaea porteri* (Banks)
Marcelino’s Cave [Fort Hood]	*Agyneta llanoensis* (Gertsch & Davis), *Eidmannella pallida* (Emerton)
Medusa Cave [Fort Hood]	*Argiope aurantia* Lucas
Moffatt Pit Cave [Fort Hood]	*Cicurina varians* Gertsch & Mulaik
Molly Hatchet Cave	*Agyneta llanoensis* (Gertsch & Davis)
Newby Cave [Fort Hood]	*Drassyllus texamans* Chamberlin
Nolan Creek Cave [Fort Hood]	*Cicurina varians* Gertsch & Mulaik, *Agyneta llanoensis* (Gertsch & Davis), *Cryptachaea porteri* (Banks)
Owl Mountain Cave	*Agyneta llanoensis* (Gertsch & Davis)
Peep in the Deep Cave [Fort Hood]	*Cicurina hoodensis* Cokendolpher & Reddell, *Agyneta llanoensis* (Gertsch & Davis), *Mermessus albulus* (Zorsch & Crosby), *Tayshaneta paraconcinna* (Cokendolpher & Reddell)
Plasma Cave	*Mermessus maculatus* (Banks)
Plethodon Cave	*Agyneta llanoensis* (Gertsch & Davis)
Plethodon Pit Cave	*Agyneta llanoensis* (Gertsch & Davis)
Poison Ivy Pit	*Mermessus albulus* (Zorsch & Crosby)
Price Pit Cave [Fort Hood]	*Cicurina varians* Gertsch & Mulaik, *Drassyllus aprilinus* (Banks), *Drassyllus gynosaphes* Chamberlin, *Hahnia flaviceps* Emerton, *Mermessus albulus* (Zorsch & Crosby)
Raining Rattler Cave	*Agyneta llanoensis* (Gertsch & Davis)
Road Side Sink [Fort Hood]	*Agelenopsis aperta* (Gertsch), *Argiope aurantia* Lucas, *Agyneta llanoensis* (Gertsch & Davis), *Naphrys acerba* (Peckham & Peckham)
Root Sink [Fort Hood]	*Cicurina varians* Gertsch & Mulaik
Rugger’s Rift Cave [Fort Hood]	*Cicurina varians* Gertsch & Mulaik, *Agyneta llanoensis* (Gertsch & Davis), *Eidmannella pallida* (Emerton), *Cryptachaea porteri* (Banks)
Rusty Cans Cave	*Agyneta llanoensis* (Gertsch & Davis)
Soldiers Cave	*Mermessus albulus* (Zorsch & Crosby)
Sparta Cave [Fort Hood]	*Cicurina varians* Gertsch & Mulaik, *Cryptachaea porteri* (Banks)
Sanford Pit Cave [Fort Hood]	*Agyneta llanoensis* (Gertsch & Davis), *Eidmannella pallida* (Emerton), *Cryptachaea porteri* (Banks)
Seven Cave [Fort Hood]	*Argiope aurantia* Lucas, *Loxosceles reclusa* Gertsch & Mulaik
Seven Mile Mountain Cave	*Cicurina troglobia* Cokendolpher, *Agyneta llanoensis* (Gertsch & Davis), *Schizocosa saltatrix* (Hentz)
Skeeter Cave	*Agyneta llanoensis* (Gertsch & Davis)
Sledgehammer Cave [Fort Hood]	*Cicurina varians* Gertsch & Mulaik, *Agyneta llanoensis* (Gertsch & Davis), *Modisimus texanus* Banks, *Eidmannella pallida* (Emerton)
Sleepy Hollow Cave	*Agyneta llanoensis* (Gertsch & Davis)
Sleepy Hollow Pit	*Agyneta llanoensis* (Gertsch & Davis)
Slotsky Pit Cave	*Agyneta llanoensis* (Gertsch & Davis)
Soldiers Cave	*Agyneta llanoensis* (Gertsch & Davis)
Southern Cross Cave	*Agyneta llanoensis* (Gertsch & Davis)
Sparta Cave [Fort Hood]	*Cicurina varians* Gertsch & Mulaik
Stand-Off Sink	*Agyneta llanoensis* (Gertsch & Davis)
Stone Eyes Sink	*Agyneta llanoensis* (Gertsch & Davis)
Streak Cave [Fort Hood]	*Cicurina caliga* Cokendolpher & Reddell, *Agyneta llanoensis* (Gertsch & Davis)
Talking Crows Cave [Fort Hood]	*Cicurina hoodensis* Cokendolpher & Reddell, *Cicurina varians* Gertsch & Mulaik, *Tayshaneta paraconcinna* (Cokendolpher & Reddell), *Agyneta llanoensis* (Gertsch & Davis), *Eidmannella pallida* (Emerton), *Neospintharus furcatus* (O. P.-Cambridge)
Treasure Cave [Fort Hood]	*Tenuiphantes sabulosus* (Keyserling), *Schizocosa saltatrix* (Hentz)
Tres Dedos Cave [Fort Hood]	*Cicurina varians* Gertsch & Mulaik
Thumbs Up Cave	*Agyneta llanoensis* (Gertsch & Davis)
Tinaja Cave	*Agyneta llanoensis* (Gertsch & Davis)
Tony’s Can Cave	*Agyneta llanoensis* (Gertsch & Davis)
Treasure Cave [Fort Hood]	*Cicurina hoodensis* Cokendolpher & Reddell, *Agyneta llanoensis* (Gertsch & Davis)
Tres Dedos Cave [Fort Hood]	*Cicurina varians* Gertsch & Mulaik
Triple J Cave [Fort Hood]	*Cicurina caliga* Cokendolpher & Reddell, *Cicurina hoodensis* Cokendolpher & Reddell, *Agyneta llanoensis* (Gertsch & Davis)
Tweedledum Cave	*Agyneta llanoensis* (Gertsch & Davis)
Valentine Cave [Fort Hood]	*Cicurina varians* Gertsch & Mulaik, *Agyneta llanoensis* (Gertsch & Davis), *Eidmannella pallida* (Emerton)
Vine Cave	*Agyneta llanoensis* (Gertsch & Davis)
Violet Cave	*Agyneta llanoensis* (Gertsch & Davis), *Leucauge venusta* (Walckenaer)
Viper Den Cave [Fort Hood])	*Cicurina varians* Gertsch & Mulaik, *Agyneta llanoensis* (Gertsch & Davis), *Mermessus albulus* (Zorsch & Crosby), *Eidmannella pallida* (Emerton), *Modisimus texanus* Banks
Weep Hole Cave	*Agyneta llanoensis* (Gertsch & Davis)
West Corral Cave No. 1	*Agyneta llanoensis* (Gertsch & Davis)
West Corral Cave No. 2	*Agyneta llanoensis* (Gertsch & Davis)
West Corral Cave No. 4	*Agyneta llanoensis* (Gertsch & Davis)
West Corral Sink	*Agyneta llanoensis* (Gertsch & Davis)
**Bexar**	
40 mm Cave	*Cryptachaea porteri* (Banks)
Ailor Hill Cave	*Schizocosa saltatrix* (Hentz)
Alligator Lizard Cave (=Wren Cave)	*Eidmannella pallida* (Emerton)
Assassin Cave	*Cicurina varians* Gertsch & Mulaik, *Cryptachaea porteri* (Banks)
B-52 Cave [Camp Bullis]	*Cicurina puentecilla* Gertsch, *Cicurina varians* Gertsch & Mulaik, *Agyneta llanoensis* (Gertsch & Davis)
B. J. Pit	*Tidarren sisyphoides* (Walckenaer)
Backhole [Camp Bullis]	*Drassyllus gynosaphes* Chamberlin, *Mermessus maculatus* (Banks), *Agyneta serrata* (Emerton), *Rabidosa rabida* (Walckenaer), *Teminius affinis* Banks, *Eidmannella rostrata* Gertsch
Banzai Mud Dauber Cave	*Cicurina varians* Gertsch & Mulaik, *Eidmannella rostrata* Gertsch, *Teminius affinis* Banks, *Cryptachaea porteri* (Banks)
Bear Cave	*Cicurina varians* Gertsch & Mulaik, *Gaucelmus augustinus* Keyserling, *Cryptachaea porteri* (Banks)
Bexar (=Bear) Cave	*Agyneta llanoensis* (Gertsch & Davis)
Black Cat Cave	*Cicurina pampa* Chamberlin & Ivie, *Cicurina puentecilla* Gertsch, *Cicurina varians* Gertsch & Mulaik, *Agyneta llanoensis* (Gertsch & Davis), *Eidmannella pallida* (Emerton)
Bob Wire Cave	*Mermessus albulus* (Zorsch & Crosby)
Bone Pile Cave [Government Canyon State Natural Area]	*Eidmannella rostrata* Gertsch, *Tidarren sisyphoides* (Walckenaer)
Boneyard Pit [Camp Bullis]	*Cicurina varians* Gertsch & Mulaik, *Modisimus texanus* Banks, *Cryptachaea porteri* (Banks)
Braken Bat Cave	*Cicurina venii* Gertsch, *Eidmannella rostrata* Gertsch
Breached Dam Cave	*Cicurina varians* Gertsch & Mulaik, *Tayshaneta sprousei* Ledford et al., *Eidmannella rostrata* Gertsch
Bullis Hole	*Pirata davisi* Wallace & Exline, *Eidmannella rostrata* Gertsch, *Modisimus texanus* Banks
Bunny Hole [Camp Bullis]	*Cicurina varians* Gertsch & Mulaik, *Agyneta llanoensis* (Gertsch & Davis), *Cryptachaea porteri* (Banks)
Buzzard Egg Cave	*Tidarren sisyphoides* (Walckenaer)
Cannonball Cave [Camp Bullis]	*Agyneta llanoensis* (Gertsch & Davis)
Caracol Creek Coon Cave	*Cicurina loftini* Cokendolpher, *Cicurina varians* Gertsch & Mulaik, *Tayshaneta whitei* Ledford et al., *Eidmannella pallida* (Emerton), *Eidmannella rostrata* Gertsch
Cave No. 18	*Tayshaneta madla* Ledford et al., *Eidmannella rostrata* Gertsch
Cave No. 189	*Tayshaneta madla* Ledford et al., *Eidmannella pallida* (Emerton)
Cave No. 194	*Mermessus albulus* (Zorsch & Crosby), *Eidmannella pallida* (Emerton)
Cave of the Bearded Tree	*Camptocosa parallela* (Banks), *Schizocosa saltatrix* (Hentz), *Eidmannella rostrata* Gertsch, *Xysticus ferox* (Hentz)
Cave of the Bee Spirits	*Cryptachaea porteri* (Banks)
Cave of the Half-Snake	*Agelenopsis aperta* (Gertsch), *Camptocosa parallela* (Banks), *Schizocosa saltatrix* (Hentz), *Eidmannella rostrata* Gertsch
Cave of the Skinny Snake	*Tidarren sisyphoides* (Walckenaer)
Cave site #301	*Cryptachaea porteri* (Banks)
Cave site #303 [Government Canyon Karst Fauna Region]	*Eidmannella pallida* (Emerton)
Cave site #305	*Eidmannella pallida* (Emerton)
Cave site #306	*Cryptachaea porteri* (Banks)
Cave site #602	*Agyneta llanoensis* (Gertsch & Davis)
Cave site #603	*Agyneta llanoensis* (Gertsch & Davis)
Cave site #701	*Eidmannella pallida* (Emerton)
Cave site #801	*Tayshaneta whitei* Ledford et al.
Cave site #2101	*Eidmannella rostrata* Gertsch
Cave With A View	*Tegenaria domestica* (Clerck)
Charley’s Cute Little Hole	*Eidmannella rostrata* Gertsch
Charley’s Hammer Hole	*Cryptachaea porteri* (Banks)
Cherry Hollow Cave (20b) (=Cave No. 19)	*Cicurina pampa* Chamberlin & Ivie
Christmas Cave	*Cicurina madla* Gertsch, *Agyneta llanoensis* (Gertsch & Davis)
Constant Sorrow Cave [Camp Bullis]	*Cicurina varians* Gertsch & Mulaik, *Agyneta sandia* Dupérré, *Tayshaneta sprousei* Ledford et al.
Cross the Creek Cave [Camp Bullis]	*Cicurina pampa* Chamberlin & Ivie, *Cicurina varians* Gertsch & Mulaik, *Eidmannella rostrata* Gertsch, *Cryptachaea porteri* (Banks)
Crownridge Canyon Cave	*Falconina gracilis* (Keyserling)
Dangerfield Cave [Camp Bullis]	*Cicurina varians* Gertsch & Mulaik, *Agyneta llanoensis* (Gertsch & Davis)
Dirtwater Cave	*Cicurina varians* Gertsch & Mulaik, *Eidmannella pallida* (Emerton), *Cryptachaea porteri* (Banks)
Dogleg Cave [Camp Bullis]	*Cicurina varians* Gertsch & Mulaik, *Agyneta llanoensis* (Gertsch & Davis)
Dos Viboras Cave	*Cryptachaea porteri* (Banks)
Droll Cave	*Neoantistea mulaiki* Gertsch, *Agyneta llanoensis* (Gertsch & Davis)
Eagles Nest Cave [Camp Bullis]	*Agyneta llanoensis* (Gertsch & Davis)
Elm Springs Cave	*Agyneta llanoensis* (Gertsch & Davis)
Elm Water Hole Cave	*Agyneta llanoensis* (Gertsch & Davis)
F-150 Cave	*Eidmannella rostrata* Gertsch
Fair Hole	*Eidmannella rostrata* Gertsch
Firing Line 11 Cave	*Dipoena abdita* Gertsch & Mulaik
Flach’s Cave	*Eidmannella rostrata* Gertsch
Flying Buzzworm Cave [Camp Bullis]	*Agyneta llanoensis* (Gertsch & Davis), *Eidmannella rostrata* Gertsch
Forked Pit	*Agyneta llanoensis* (Gertsch & Davis)
Friesenhahn Cave	*Cicurina varians* Gertsch & Mulaik
Eagles Nest Cave	*Cicurina bullis* Cokendolpher, *Cicurina varians* Gertsch & Mulaik, *Mermessus albulus* (Zorsch & Crosby), *Eidmannella rostrata* Gertsch, *Cryptachaea porteri* (Banks), *Tidarren sisyphoides* (Walckenaer)
Elm Springs Cave (=Grubbs Cave ES)	*Cicurina neovespera* Cokendolpher
Elm Water Hole Cave	*Mermessus albulus* (Zorsch & Crosby)
Game Pasture Cave No. 1	*Agyneta llanoensis* (Gertsch & Davis), *Eidmannella rostrata* Gertsch
Georg’s Hole	*Eidmannella rostrata* Gertsch
Get a Rope Cave [Camp Bullis]	*Leptoctenus byrrhus* Simon, *Agyneta sandia* Dupérré
Glinn’s Gloat Hole [Camp Bullis]	*Cicurina varians* Gertsch & Mulaik
Goat Cave	*Cryptachaea porteri* (Banks)
Goat Cave [Government Canyon State Natural Area]	*Cicurina varians* Gertsch & Mulaik
Government Canyon Bat Cave	*Cicurina varians* Gertsch & Mulaik, *Cicurina vespera* Gertsch, *Tayshaneta microps* (Gertsch), *Agyneta llanoensis* (Gertsch & Davis), *Eidmannella pallida* (Emerton), *Eidmannella rostrata* Gertsch, *Cryptachaea porteri* (Banks)
Hairy Tooth Cave	*Agyneta llanoensis* (Gertsch & Davis), *Cryptachaea porteri* (Banks)
Han’s Grotto	*Cicurina varians* Gertsch & Mulaik
Haz Mat Pit	*Mermessus maculatus* (Banks), *Tidarren sisyphoides* (Walckenaer)
Headquarters Cave [Camp Bullis]	*Cicurina madla* Gertsch, *Cicurina varians* Gertsch & Mulaik, *Eidmannella pallida* (Emerton), *Eidmannella rostrata* Gertsch, *Cryptachaea porteri* (Banks)
Hector Hole	*Eidmannella rostrata* Gertsch
Hector’s Hole [Camp Bullis]	*Cicurina varians* Gertsch & Mulaik
Helotes Blowhole	*Cicurina madla* Gertsch, *Eidmannella rostrata* Gertsch
Hilger Hole [Camp Bullis]	*Cicurina bullis* Cokendolpher, *Cicurina varians* Gertsch & Mulaik
Hill’s and Dale’s Pit	*Tayshaneta bullis* (Cokendolpher)
Hills and Dales Pit	*Cicurina madla* Gertsch, *Cicurina varians* Gertsch & Mulaik
Hitzfelder’s Bone Hole (=Hitzfelder Cave)	*Cicurina varians* Gertsch & Mulaik, *Eidmannella rostrata* Gertsch
Hogan’s Cave	*Cryptachaea porteri* (Banks)
Hold Me Back Cave	*Cicurina varians* Gertsch & Mulaik, *Eidmannella rostrata* Gertsch
Holy Smoke Cave	*Gaucelmus augustinus* Keyserling, *Cryptachaea porteri* (Banks)
Hornet’s Last Laugh Pit	*Cicurina varians* Gertsch & Mulaik, *Teminius affinis* Banks
I Think It’s A Cave	*Eidmannella pallida* (Emerton)
Isocow Cave [Camp Bullis]	*Cicurina bullis* Cokendolpher, *Cicurina varians* Gertsch & Mulaik, *Eidmannella rostrata* Gertsch, *Cryptachaea porteri* (Banks)
Isopit	*Cicurina varians* Gertsch & Mulaik, *Eidmannella rostrata* Gertsch, *Cryptachaea porteri* (Banks)
John Wagner Ranch Cave No. 3	*Tidarren sisyphoides* (Walckenaer)
Kamikazi Cricket Cave	*Cicurina varians* Gertsch & Mulaik, *Mermessus maculatus* (Banks), *Eidmannella pallida* (Emerton), *Cryptachaea porteri* (Banks)
Karst Feature 471-4	*Cicurina pampa* Chamberlin & Ivie
Kick Start Cave	*Wulfila tantillus* Chickering
King Toad Cave	*Agyneta llanoensis* (Gertsch & Davis)
La Cantera Cave No. 3	*Agyneta llanoensis* (Gertsch & Davis)
La Cantera Sink (=Grubbs Cave No. 23)	*Cicurina neovespera* Cokendolpher
Leon Hill Cave [Camp Bullis]	*Mermessus albulus* (Zorsch & Crosby)
Linda’s First Cave	*Rabidosa rabida* (Walckenaer), *Modisimus texanus* Banks
Linda’s First Cave Find	*Agyneta llanoensis* (Gertsch & Davis)
Lithic Ridge Cave	*Tayshaneta whitei* Ledford et al., *Cryptachaea porteri* (Banks)
Logan’s Cave	*Agelenopsis aperta* (Gertsch), *Cicurina madla* Gertsch, *Tidarren sisyphoides* (Walckenaer)
Lone Gunman Pit [Camp Bullis]	*Cicurina varians* Gertsch & Mulaik, *Agyneta llanoensis* (Gertsch & Davis), *Xysticus funestus* Keyserling
Lost Mine Trail Cave	*Tidarren sisyphoides* (Walckenaer)
Lost Pot Hole	*Gaucelmus augustinus* Keyserling
Lost Pothole (=Lost Pot)	*Cicurina madla* Gertsch
Low Priority Cave [Camp Bullis]	*Cicurina varians* Gertsch & Mulaik, *Agyneta llanoensis* (Gertsch & Davis), *Eidmannella rostrata* Gertsch
Madla’s Cave	*Cicurina madla* Gertsch, *Cicurina varians* Gertsch & Mulaik, *Tayshaneta madla* Ledford et al., *Mermessus maculatus* (Banks), *Eidmannella rostrata* Gertsch
Madla’s Drop	*Tayshaneta madla* Ledford et al.
Madla’s Drop Cave	*Cicurina madla* Gertsch, *Mermessus maculatus* (Banks)
MARS Pit [Camp Bullis]	*Cicurina platypus* Cokendolpher
MARS Shaft [Camp Bullis]	*Cicurina varians* Gertsch & Mulaik, *Eidmannella rostrata* Gertsch
Mastodon Pit	*Agyneta sandia* Dupérré
Mattke Cave	*Cicurina varians* Gertsch & Mulaik, *Eidmannella rostrata* Gertsch, *Cryptachaea porteri* (Banks)
Max and Roberts Cave (=SWCA no. 3007, 3008, 3009, 3011)	*Agyneta llanoensis* (Gertsch & Davis), *Eidmannella pallida* (Emerton), *Eidmannella rostrata* Gertsch
Max and Roberts Cave	*Cicurina varians* Gertsch & Mulaik
Meusebach Flats Cave	*Agyneta llanoensis* (Gertsch & Davis)
NBC Cave	*Modisimus texanus* Banks
Niche Cave	*Cicurina varians* Gertsch & Mulaik, *Modisimus texanus* Banks
Obvious Little Cave	*Neoantistea mulaiki* Gertsch, *Agyneta llanoensis* (Gertsch & Davis), *Rabidosa rabida* (Walckenaer), *Modisimus texanus* Banks
One Formation Cave [Government Canyon State Natural Area]	*Cicurina varians* Gertsch & Mulaik
Peace Pipe Cave [Camp Bullis]	*Cicurina varians* Gertsch & Mulaik, *Agyneta llanoensis* (Gertsch & Davis)
Persimmon Pit	*Mermessus maculatus* (Banks), *Eidmannella pallida* (Emerton)
Phil’s Friggin Line Cave (Cave, site #803)	*Cryptachaea porteri* (Banks)
Platypus Pit [Camp Bullis]	*Cicurina bullis* Cokendolpher, *Cicurina platypus* Cokendolpher, *Cicurina varians* Gertsch & Mulaik
Plethodon Pit (Stone Oak Karst Region)	*Agyneta llanoensis* (Gertsch & Davis)
Poison Ivy Pit	*Eidmannella rostrata* Gertsch, *Modisimus texanus* Banks
Poor Boy Baculum Cave	*Cryptachaea porteri* (Banks)
Porcupine Parlor Cave [Camp Bullis]	*Agyneta llanoensis* (Gertsch & Davis)
Porcupine Squeeze Cave (=Grubs Cave No. 189)	*Cicurina pampa* Chamberlin & Ivie, *Cicurina varians* Gertsch & Mulaik, *Eidmannella pallida* (Emerton), *Cryptachaea porteri* (Banks)
Power Pole 60 Feature	*Teminius affinis* Banks
Raging Cajun Cave (=Rajin’ Cajun Cave)	*Cicurina varians* Gertsch & Mulaik, *Agyneta llanoensis* (Gertsch & Davis), *Cryptachaea porteri* (Banks)
Rattlesnake Cave	*Agyneta llanoensis* (Gertsch & Davis), *Cryptachaea porteri* (Banks)
Record Fire 1 Cave	*Modisimus texanus* Banks
Record Fire 1 Pit [Camp Bullis]	*Mermessus albulus* (Zorsch & Crosby)
Roan’s Cave	*Loxosceles reclusa* Gertsch & Mulaik
Robber Baron Cave	*Metaltella simoni* (Keyserling), *Cicurina baronia* Gertsch, *Cicurina varians* Gertsch & Mulaik, *Eidmannella pallida* (Emerton), *Eidmannella rostrata* Gertsch, *Cryptachaea porteri* (Banks)
Robber Barron Cave	*Parasteatoda tepidariorum* (C. L. Koch)
Robbers Cave	*Cicurina madla* Gertsch, *Cicurina varians* Gertsch & Mulaik
Root Canal Cave [Camp Bullis]	*Cicurina bullis* Cokendolpher, *Cicurina varians* Gertsch & Mulaik, *Agyneta llanoensis* (Gertsch & Davis), *Eidmannella rostrata* Gertsch
Root Toupee Cave [Camp Bullis]	*Agyneta llanoensis* (Gertsch & Davis)
SARA Site 4 Cave	*Eidmannella pallida* (Emerton)
SBC Cave	*Cicurina loftini* Cokendolpher
Scenic Overlook Cave (=Cave site #2101)	*Eidmannella rostrata* Gertsch
Scorpion Cave	*Tayshaneta madla* Ledford et al., *Cryptachaea porteri* (Banks)
Some Monk Chanted Evening Cave	*Cicurina varians* Gertsch & Mulaik
Stahl Cave	*Cicurina brunsi* Cokendolpher, *Eidmannella rostrata* Gertsch
Stealth Cave	*Eidmannella pallida* (Emerton)
Stevens Ranch Cave No. 1	*Cicurina varians* Gertsch & Mulaik, *Mermessus maculatus* (Banks), *Eidmannella pallida* (Emerton), *Cryptachaea porteri* (Banks
Stevens Ranch Trash Hole Cave	*Agyneta llanoensis* (Gertsch & Davis)
Stone Oak Parkway Pit	*Cicurina pampa* Chamberlin & Ivie, *Cicurina varians* Gertsch & Mulaik, *Hahnia flaviceps* Emerton, *Mermessus maculatus* (Banks)
Strange Little Cave [Camp Bullis]	*Cicurina varians* Gertsch & Mulaik, *Agyneta llanoensis* (Gertsch & Davis), *Teminius affinis* Banks, *Scytodes atlacoya* Rheims, Brescovit & Durán, *Cryptachaea porteri* (Banks), *Latrodectus mactans* (Fabricius)
Sunless City Cave	*Cicurina varians* Gertsch & Mulaik
Sunray Cave (=Cave No. 18)	*Eidmannella rostrata* Gertsch
Surprise Sink [Government Canyon State Natural Area]	*Trachelas volutus* Gertsch, *Eidmannella rostrata* Gertsch
SWCA Cave 3	*Agyneta llanoensis* (Gertsch & Davis)
SWCA no. 3011	*Eidmannella rostrata* Gertsch
Tall Tales Cave	*Cicurina varians* Gertsch & Mulaik, *Cryptachaea porteri* (Banks)
Three Fingers Cave	*Cryptachaea porteri* (Banks)
Tin Pot	*Cryptachaea porteri* (Banks)
Tin Pot Cave [Camp Bullis]	*Agyneta llanoensis* (Gertsch & Davis)
Toad Cave	*Mermessus albulus* (Zorsch & Crosby), *Eidmannella pallida* (Emerton
Twin Pits	*Cicurina varians* Gertsch & Mulaik
Unknown Cave	*Cryptachaea porteri* (Banks)
Up the Creek Cave [Camp Bullis]	*Leptoctenus byrrhus* Simon, *Cicurina pampa* Chamberlin & Ivie, *Cicurina varians* Gertsch & Mulaik, *Tayshaneta bullis* (Cokendolpher), *Mermessus albulus* (Zorsch & Crosby), *Eidmannella rostrata* Gertsch, *Cryptachaea porteri* (Banks)
Valley of Death Cave	*Cryptachaea porteri* (Banks)
Vera Cruz Shaft [Camp Bullis]	*Cicurina pampa* Chamberlin & Ivie, *Cicurina varians* Gertsch & Mulaik, *Zelotes pseustes* Chamberlin
Voight’s Bat Cave	*Eidmannella pallida* (Emerton)
Well Done Cave	*Cicurina varians* Gertsch & Mulaik, *Cryptachaea porteri* (Banks)
Winston’s Cave [Camp Bullis]	*Cicurina varians* Gertsch & Mulaik, *Eidmannella rostrata* Gertsch, *Tidarren sisyphoides* (Walckenaer)
World Newt Cave	*Modisimus texanus* Banks, *Cryptachaea porteri* (Banks), *Tidarren sisyphoides* (Walckenaer)
Wren Cave	*Eidmannella pallida* (Emerton)
Wurzbach Bat Cave	*Cicurina varians* Gertsch & Mulaik, *Agyneta llanoensis* (Gertsch & Davis), *Agyneta serrata* (Emerton), *Eidmannella rostrata* Gertsch, *Gaucelmus augustinus* Keyserling, *Modisimus texanus* Banks, *Cryptachaea porteri* (Banks)
Yellow Ball Cave [Camp Bullis]	*Agyneta llanoensis* (Gertsch & Davis)
Young Cave No. 1	*Tayshaneta madla* Ledford et al., *Eidmannella pallida* (Emerton), *Gaucelmus augustinus* Keyserling, *Cryptachaea porteri* (Banks)
**Blanco**	
Davis Blowout Cave	*Cicurina varians* Gertsch & Mulaik, *Urozelotes rusticus* (L. Koch), *Cryptachaea porteri* (Banks)
Forest View Cave	*Eidmannella pallida* (Emerton)
Llewellyn Cave	*Cicurina varians* Gertsch & Mulaik
T Cave	*Cicurina varians* Gertsch & Mulaik, *Eidmannella pallida* (Emerton), *Eidmannella rostrata* Gertsch, *Gaucelmus augustinus* Keyserling
Wells Sink	*Agyneta llanoensis* (Gertsch & Davis)
**Brewster**	
400 Foot Cave	*Tayshaneta vidrio* Ledford et al., *Mermessus antraeus* (Crosby)
Javelina Hole	*Physocyclus enaulus* Crosby
Lichnovsky’s Cave	*Physocyclus enaulus* Crosby
O.T.L. Cave	*Araneus gemma* (McCook), *Araneus illaudatus* (Gertsch & Mulaik), *Cicurina varians* Gertsch & Mulaik, *Drassyllus prosaphes* Chamberlin, *Drassyllus texamans* Chamberlin, *Physocyclus enaulus* Crosby
Split Tank Cave	*Cicurina varians* Gertsch & Mulaik
**Burnet**	
Beaver Creek Bat Cave	*Cicurina varians* Gertsch & Mulaik, *Cryptachaea porteri* (Banks), *Mecaphesa dubia* (Keyserling)
Big Bad Wolf Cave	*Eidmannella rostrata* Gertsch
Cricket City Sink	*Agyneta llanoensis* (Gertsch & Davis)
Crossing Cave	*Cicurina varians* Gertsch & Mulaik
Duncan’s Flea Cave	*Cicurina varians* Gertsch & Mulaik, *Cryptachaea porteri* (Banks)
Eckhardt Root Cave	*Agyneta llanoensis* (Gertsch & Davis)
Fenceline Sink	*Cicurina varians* Gertsch & Mulaik, *Agyneta llanoensis* (Gertsch & Davis)
Huber Mine	*Cryptachaea porteri* (Banks)
Longhorn Caverns	*Cicurina varians* Gertsch & Mulaik, *Agyneta llanoensis* (Gertsch & Davis), *Eidmannella rostrata* Gertsch, *Cryptachaea porteri* (Banks)
Marble Falls Cave No. 3	*Cicurina varians* Gertsch & Mulaik
Nolan’s Cave	*Eidmannella pallida* (Emerton), *Cryptachaea porteri* (Banks)
Persimon Sink	*Cicurina varians* Gertsch & Mulaik
Pie Cave	*Cicurina varians* Gertsch & Mulaik, *Agyneta llanoensis* (Gertsch & Davis), *Cryptachaea porteri* (Banks)
Porcupine Cave	*Cicurina varians* Gertsch & Mulaik
Railroad Cave	*Agyneta llanoensis* (Gertsch & Davis)
Resurrection Well	*Agyneta llanoensis* (Gertsch & Davis)
Shin Oak Sink	*Cicurina varians* Gertsch & Mulaik
Simon Says Sink No. 2	*Cicurina varians* Gertsch & Mulaik
Simons 1174 Sink	*Cicurina varians* Gertsch & Mulaik
Simons Pretty Pit	*Agyneta llanoensis* (Gertsch & Davis)
Simons Rattlesnake Well	*Cicurina varians* Gertsch & Mulaik
Simons Squeeze-Down Pit	*Cicurina varians* Gertsch & Mulaik
Simons Squirm-Around Cave	*Cicurina varians* Gertsch & Mulaik
Simons Water Cave	*Agyneta llanoensis* (Gertsch & Davis)
Snake Pit Sink	*Cicurina varians* Gertsch & Mulaik
Snelling’s Cave	*Cicurina varians* Gertsch & Mulaik, *Eidmannella pallida* (Emerton), *Cryptachaea porteri* (Banks)
Taylor Water Cave	*Agyneta llanoensis* (Gertsch & Davis), *Cryptachaea porteri* (Banks)
Tree Ladder Sink	*Cicurina varians* Gertsch & Mulaik
Wagon Trail Cave	*Cicurina varians* Gertsch & Mulaik
Waldman Cave	*Eidmannella pallida* (Emerton)
Washout Cave	*Agyneta llanoensis* (Gertsch & Davis)
**Childress**	
Black Hand Cave	*Cicurina varians* Gertsch & Mulaik, *Islandiana unicornis* Ivie, *Cryptachaea porteri* (Banks), *Latrodectus mactans* (Fabricius
Buzzard Wall Cave	*Cicurina varians* Gertsch & Mulaik
Windmill Crack Cave	*Agyneta llanoensis* (Gertsch & Davis), *Eidmannella pallida* (Emerton)
**Collingsworth**	
Bumpas Cave	*Metellina mimetoides* Chamberlin & Ivie, *Cryptachaea porteri* (Banks)
Turtle Cave	*Eidmannella pallida* (Emerton), *Cryptachaea porteri* (Banks)
**Comal**	
Bad Weather Pit	*Agyneta llanoensis* (Gertsch & Davis), *Eidmannella rostrata* Gertsch
Bain’s Cave	*Rabidosa punctulata* (Hentz), *Rabidosa rabida* (Walckenaer), *Eidmannella rostrata* Gertsch
Bear Creek Cave	*Cicurina varians* Gertsch & Mulaik
Bender’s Cave	*Eidmannella pallida* (Emerton), *Eidmannella rostrata* Gertsch, *Modisimus texanus* Banks
Bracken Bat Cave	*Cicurina varians* Gertsch & Mulaik, *Scotophaeus blackwalli* (Thorell)
Brehmmer Cave (=Heidrich’s Cave)	*Cicurina joya* Gertsch, *Tayshaneta coeca* (Chamberlin & Ivie), *Eidmannella pallida* (Emerton), *Gaucelmus augustinus* Keyserling, *Modisimus texanus* Banks
Brehmmer-Heidrich Cave	*Cicurina varians* Gertsch & Mulaik, *Gaucelmus augustinus* Keyserling
Camp Bullis Bad Air Cave	*Mermessus maculatus* (Banks), *Eidmannella rostrata* Gertsch
Camp Bullis Bat Cave	*Eidmannella rostrata* Gertsch
Camp Bullis Cave No. 1 [Camp Bullis]	*Agyneta llanoensis* (Gertsch & Davis), *Eidmannella rostrata* Gertsch
Camp Bullis Cave No. 3	*Cicurina varians* Gertsch & Mulaik, *Eidmannella rostrata* Gertsch
Coreth Bat Cave	*Cicurina varians* Gertsch & Mulaik, *Tayshaneta coeca* (Chamberlin & Ivie), *Eidmannella pallida* (Emerton), *Gaucelmus augustinus* Keyserling
Deepwater Cave	*Cicurina varians* Gertsch & Mulaik
Dierk Cave No. 1	*Gaucelmus augustinus* Keyserling
Ebert Cave	*Cicurina varians* Gertsch & Mulaik, *Agyneta llanoensis* (Gertsch & Davis), *Agyneta serrata* (Emerton), *Eidmannella rostrata* Gertsch, *Gaucelmus augustinus* Keyserling
Fischer Cave	*Cicurina varians* Gertsch & Mulaik
Fischer Pit	*Gaucelmus augustinus* Keyserling
Fisher’s Pit	*Agyneta llanoensis* (Gertsch & Davis)
Grosser’s Cave	*Eidmannella pallida* (Emerton)
Grosser’s Sink (=Grosser’s-Saur’s Sink)	*Eidmannella rostrata* Gertsch
Heidrich’s Cave	*Cicurina joya* Gertsch
Hitzfielder’s Cave	*Cicurina varians* Gertsch & Mulaik
Just Now Cave	*Eidmannella rostrata* Gertsch, *Modisimus texanus* Banks
Kappelman Cave	*Cicurina reclusa* Gertsch, *Cicurina varians* Gertsch & Mulaik, *Eidmannella rostrata* Gertsch, *Cryptachaea porteri* (Banks)
Kappelman Salamander Cave	*Cicurina reclusa* Gertsch, *Cicurina varians* Gertsch & Mulaik, *Agyneta llanoensis* (Gertsch & Davis)
Klar’s Cave	*Cicurina varians* Gertsch & Mulaik, *Agyneta llanoensis* (Gertsch & Davis), *Eidmannella rostrata* Gertsch, *Modisimus texanus* Banks
Knee Deep Cave	*Eidmannella rostrata* Gertsch
Lewis Cave	*Cicurina varians* Gertsch & Mulaik
Little Bear Creek Cave	*Cryptachaea porteri* (Banks)
Little Brehmmer-Heidrich Cave	*Latrodectus mactans* (Fabricius)
Little Cave	*Gaucelmus augustinus* Keyserling
Little Gem Cave No. 1	*Leucauge venusta* (Walckenaer)
Little Gem Cave	*Cicurina varians* Gertsch & Mulaik
Natural Bridge Caverns	*Cicurina puentecilla* Gertsch, *Cicurina varians* Gertsch & Mulaik, *Tayshaneta coeca* (Chamberlin & Ivie), *Eidmannella rostrata* Gertsch, *Cryptachaea porteri* (Banks)
Preserve Cave [Honey Creek Preserve]	*Eidmannella rostrata* Gertsch
Snake Skin Pit [Camp Bullis]	*Agyneta llanoensis* (Gertsch & Davis)
Snakeskin Pit	*Eidmannella rostrata* Gertsch
Startzville Bat Cave	*Cicurina varians* Gertsch & Mulaik
Strosser’s Sink	*Eidmannella rostrata* Gertsch
Washington Cave	*Cicurina varians* Gertsch & Mulaik, *Mermessus albulus* (Zorsch & Crosby), *Mermessus maculatus* (Banks)
Wiley’s Cave	*Cicurina varians* Gertsch & Mulaik, *Eidmannella rostrata* Gertsch
**Coryell**	
Big Red Cave [Fort Hood]	*Cicurina coryelli* Gertsch, *Agyneta llanoensis* (Gertsch & Davis)
Brokeback Cave [Fort Hood]	*Argiope aurantia* Lucas, *Cicurina varians* Gertsch & Mulaik
Chigiouxs’ Cave [Fort Hood]	*Cicurina varians* Gertsch & Mulaik, *Agyneta llanoensis* (Gertsch & Davis), *Mermessus maculatus* (Banks), *Eidmannella pallida* (Emerton)
Copperhead Cave	*Agyneta llanoensis* (Gertsch & Davis)
Copperhead Cave No. 2 [Fort Hood]	*Cicurina varians* Gertsch & Mulaik
Copperhead Sink No. 2	*Mermessus albulus* (Zorsch & Crosby), *Mermessus maculatus* (Banks)
Cornelius Cave	*Agyneta llanoensis* (Gertsch & Davis)
Diamond Cave	*Cicurina varians* Gertsch & Mulaik, *Agyneta llanoensis* (Gertsch & Davis
Dionne Cave	*Agyneta llanoensis* (Gertsch & Davis)
Egypt Cave [Fort Hood]	*Cicurina coryelli* Gertsch, *Cicurina varians* Gertsch & Mulaik, *Agyneta llanoensis* (Gertsch & Davis)
Formation Cave	*Agyneta llanoensis* (Gertsch & Davis)
Fossil Spring Cave [Fort Hood]	*Erigone autumnalis* Emerton, *Cryptachaea porteri* (Banks)
Gann Cave [Fort Hood]	*Cicurina varians* Gertsch & Mulaik
Ingram Cave	*Agyneta llanoensis* (Gertsch & Davis)
Keyhole Cave	*Agyneta llanoensis* (Gertsch & Davis)
Lucky Day Cave	*Agyneta llanoensis* (Gertsch & Davis)
Mixmaster Cave [Fort Hood]	*Argiope aurantia* Lucas, *Cicurina mixmaster* Cokendolpher & Reddell, *Cicurina varians* Gertsch & Mulaik, *Eidmannella pallida* (Emerton)
New Cave	*Agyneta llanoensis* (Gertsch & Davis)
Oxygen Bottle Cave	*Cicurina varians* Gertsch & Mulaik, *Cryptachaea porteri* (Banks)
Plateau Cave No. 1	*Eidmannella pallida* (Emerton)
Plateau Cave No. 2	*Agyneta llanoensis* (Gertsch & Davis), *Mermessus maculatus* (Banks), *Cryptachaea porteri* (Banks)
Porter Cave [Fort Hood]	*Hahnia flaviceps* Emerton, *Agyneta llanoensis* (Gertsch & Davis), *Mermessus albulus* (Zorsch & Crosby), *Mermessus maculatus* (Banks)
Rocket River C System (B. R.’s Secret Cave)	*Mermessus albulus* (Zorsch & Crosby)
Rocket River Cave System (Double Tree Cave) [Fort Hood]	*Cicurina varians* Gertsch & Mulaik, *Cryptachaea porteri* (Banks)
Rocket River Cave System (Rocket River Cave) [Fort Hood]	*Cicurina varians* Gertsch & Mulaik
Runoff Cave [Fort Hood]	*Cicurina varians* Gertsch & Mulaik, *Mermessus maculatus* (Banks)
Saltpeter Cave [Fort Hood]	*Cicurina varians* Gertsch & Mulaik, *Cryptachaea porteri* (Banks)
Shell Mountain Bat Cave [Fort Hood]	*Cicurina varians* Gertsch & Mulaik
Sperry Cave	*Agyneta llanoensis* (Gertsch & Davis)
Tippit Cave [Fort Hood]	*Cicurina coryelli* Gertsch, *Cicurina varians* Gertsch & Mulaik, *Agyneta llanoensis* (Gertsch & Davis), *Eidmannella pallida* (Emerton)
Wagontop Spring Cave	*Agyneta llanoensis* (Gertsch & Davis)
**Crockett**	
09 Well	*Eidmannella pallida* (Emerton)
Dudley Cave	*Cicurina varians* Gertsch & Mulaik, *Eidmannella pallida* (Emerton), *Cryptachaea porteri* (Banks)
Ketchum Cave	*Cicurina varians* Gertsch & Mulaik, *Physocyclus enaulus* Crosby
Water Cave	*Eidmannella pallida* (Emerton)
**Culberson**	
Border Cave	*Mermessus antraeus* (Crosby)
Brooks Cave	*Cryptachaea canionis* (Chamberlin & Gertsch)
Canyon Cave	*Cryptachaea canionis* (Chamberlin & Gertsch)
Crystal Cave	*Eidmannella bullata* Gertsch, *Eidmannella rostrata* Gertsch
Cutoff Cave	*Mermessus antraeus* (Crosby)
Decent Cave	*Cicurina varians* Gertsch & Mulaik
Dillahunty Swallow Cave	*Physocyclus enaulus* Crosby, *Cryptachaea porteri* (Banks)
East Mill Cave	*Cicurina varians* Gertsch & Mulaik
Granado Cave	*Neoanagraphis chamberlini* Gertsch & Mulaik
Grass Cave	*Physocyclus enaulus* Crosby
Grassy Grotto	*Physocyclus enaulus* Crosby
Gully Cave	*Latrodectus hesperus* Chamberlin & Ivie
Gyp Joint	*Mermessus antraeus* (Crosby), *Cryptachaea porteri* (Banks)
Harvestman Fissure	*Physocyclus enaulus* Crosby
Hully Gully Cave	*Camptocosa parallela* (Banks), *Camptocosa texana* Dondale, Jiménez & Nieto
Jack Rabbit Cave	*Latrodectus hesperus* Chamberlin & Ivie
New Cave	*Mermessus antraeus* (Crosby)
Olive’s Cave	*Mermessus antraeus* (Crosby)
Plateau Cave	*Masoncus conspectus* (Gertsch & Davis), *Cryptachaea porteri* (Banks)
Porcupine Fissure	*Eidmannella pallida* (Emerton)
Spare Tires Cave	*Physocyclus enaulus* Crosby
Straight Cave	*Cryptachaea canionis* (Chamberlin & Gertsch)
Whirlwind Cave	*Eidmannella pallida* (Emerton)
Wiggley Cave	*Eidmannella bullata* Gertsch, *Eidmannella rostrata* Gertsch
Windy Cave	*Physocyclus enaulus* Crosby
**Edwards**	
3-Bounce Pit	*Cicurina rainesi* Gertsch, *Cicurina varians* Gertsch & Mulaik, *Cryptachaea porteri* (Banks)
700 Springs Cave	*Gaucelmus augustinus* Keyserling
Blue Elm Cave	*Cryptachaea porteri* (Banks)
Cueva de la Cola Blanca	*Cryptachaea porteri* (Banks)
Deep Cave	*Cicurina varians* Gertsch & Mulaik
Devil’s Sinkhole	*Cicurina varians* Gertsch & Mulaik, *Mermessus maculatus* (Banks), *Hogna carolinensis* (Walckenaer), *Pirata sedentarius* Montgomery, *Leucauge venusta* (Walckenaer), *Cryptachaea porteri* (Banks)
Dunbar Cave	*Cicurina gruta* Gertsch, *Cicurina varians* Gertsch & Mulaik, *Ariadna bicolor* (Hentz), *Cryptachaea porteri* (Banks)
Green Cave	*Cryptachaea porteri* (Banks)
Hughes Cave	*Cicurina varians* Gertsch & Mulaik, *Cryptachaea porteri* (Banks)
Jacoby Cave	*Cicurina varians* Gertsch & Mulaik, *Cryptachaea porteri* (Banks)
Jenkins Skylight Stream Cave	*Agyneta llanoensis* (Gertsch & Davis)
Killer Frog Cave	*Agyneta llanoensis* (Gertsch & Davis)
Midnight Cave	*Cryptachaea porteri* (Banks)
Punkin Cave	*Euagrus chisoseus* Gertsch, *Cicurina varians* Gertsch & Mulaik, *Hogna antelucana* (Montgomery), *Physocyclus enaulus* Crosby, *Latrodectus mactans* (Fabricius)
Vance Cave	*Eidmannella pallida* (Emerton)
Wheat Cave	*Cryptachaea porteri* (Banks)
Wheat Cave No. 1	*Physocyclus enaulus* Crosby, *Cryptachaea porteri* (Banks)
Wyatt Cave	*Agyneta llanoensis* (Gertsch & Davis), *Cryptachaea porteri* (Banks)
**El Paso**	
Helm’s West Well	*Physocyclus enaulus* Crosby
**Gillespie**	
Cave Creek Mosquito Cave	*Cicurina varians* Gertsch & Mulaik, *Agyneta llanoensis* (Gertsch & Davis)
**Hardeman**	
Campsey Cave	*Drassyllus texamans* Chamberlin, *Eidmannella pallida* (Emerton), *Theridion llano* Levi
Short Cave	*Latrodectus mactans* (Fabricius)
Walkup Cave	*Cicurina varians* Gertsch & Mulaik, *Agyneta spicula* Dupérré, *Mysmena incredula* (Gertsch & Davis), *Metellina mimetoides* Chamberlin & Ivie, *Cryptachaea porteri* (Banks)
**Hays**	
Bear Cave	*Gaucelmus augustinus* Keyserling
Beaver Cave (=Wonder Cave)	*Gaucelmus augustinus* Keyserling
Boggus Cave	*Cicurina varians* Gertsch & Mulaik, *Cryptachaea porteri* (Banks)
Boyett’s Cave	*Cicurina russelli* Gertsch, *Cicurina varians* Gertsch & Mulaik, *Agyneta llanoensis* (Gertsch & Davis), *Gaucelmus augustinus* Keyserling
Burnett Ranch Cave	*Tayshaneta archambaulti* Ledford et al., *Gaucelmus augustinus* Keyserling
Cathy’s Cave	*Tayshaneta oconnorae* Ledford et al., *Gaucelmus augustinus* Keyserling
Cricket Cave	*Gaucelmus augustinus* Keyserling
Donaldson Cave	*Cicurina varians* Gertsch & Mulaik, *Modisimus texanus* Banks, *Cryptachaea porteri* (Banks)
Ezell’s Cave	*Tegenaria pagana* C. L. Koch), *Argiope aurantia* Lucas, *Cicurina ezelli* Gertsch, *Cicurina varians* Gertsch & Mulaik, *Gnaphosa fontinalis* Keyserling, *Mermessus maculatus* (Banks), *Gaucelmus augustinus* Keyserling, *Cryptachaea porteri* (Banks), *Parasteatoda tepidariorum* (C. L. Koch)
Fern Cave	*Argiope aurantia* Lucas, *Cicurina ubicki* Gertsch, *Tayshaneta oconnorae* Ledford et al., *Cryptachaea porteri* (Banks)
Freeman Crawl	*Tayshaneta coeca* (Chamberlin & Ivie)
Grapevine Cave	*Cicurina ezelli* Gertsch, *Tayshaneta archambaulti* Ledford et al., *Eidmannella pallida* (Emerton)
Hackberry Cave	*Tayshaneta coeca* (Chamberlin & Ivie)
Halifax Bat Cave	*Cicurina varians* Gertsch & Mulaik, *Eidmannella rostrata* Gertsch
Hunter Uncave	*Cicurina varians* Gertsch & Mulaik
Ladder Cave	*Cryptachaea porteri* (Banks)
McCarty Cave	*Cicurina varians* Gertsch & Mulaik, *Agyneta llanoensis* (Gertsch & Davis), *Tayshaneta coeca* (Chamberlin & Ivie), *Eidmannella pallida* (Emerton), *Cryptachaea porteri* (Banks)
McGlothlin Cave	*Cicurina ubicki* Gertsch
McGlothlin Sink	*Tayshaneta coeca* (Chamberlin & Ivie), *Gaucelmus augustinus* Keyserling, *Cryptachaea porteri* (Banks)
Michaelis Cave	*Gaucelmus augustinus* Keyserling
Morton’s Cave	*Cicurina varians* Gertsch & Mulaik
Nance Bat Cave	*Cicurina varians* Gertsch & Mulaik, *Eidmannella rostrata* Gertsch
Pulpit Cave	*Tayshaneta bullis* (Cokendolpher)
Root Beard Cave	*Tayshaneta coeca* (Chamberlin & Ivie)
Taylor Bat Cave	*Agyneta llanoensis* (Gertsch & Davis)
Vogelsang’s Camp	*Gaucelmus augustinus* Keyserling
Wimberly Bat Cave	*Cicurina varians* Gertsch & Mulaik, *Agyneta llanoensis* (Gertsch & Davis), *Mermessus albulus* (Zorsch & Crosby), *Gaucelmus augustinus* Keyserling
Wiseman Sink	*Eidmannella pallida* (Emerton), *Gaucelmus augustinus* Keyserling
Wiseman Sink No. 2	*Gaucelmus augustinus* Keyserling
Wiseman’s Sink	*Tayshaneta coeca* (Chamberlin & Ivie)
Wiseman’s Sink No. 2	*Tayshaneta coeca* (Chamberlin & Ivie)
Wonder Cave	*Gaucelmus augustinus* Keyserling
**Howard**	
Cramer’s Scenic Mountain Cave	*Eidmannella pallida* (Emerton)
**Irion**	
Arden Cave	*Cicurina varians* Gertsch & Mulaik, *Agyneta llanoensis* (Gertsch & Davis), *Cryptachaea porteri* (Banks)
Corngriders Cave	*Eidmannella pallida* (Emerton)
Murphy Wells Cave	*Agyneta llanoensis* (Gertsch & Davis)
Noelke Cave	*Eidmannella pallida* (Emerton)
**Jeff Davis**	
Bloys Camp Cave	*Cicurina varians* Gertsch & Mulaik, *Jalapyphantes puebla* Gertsch & Davis
Phantom Lake Cave	*Eidmannella tuckeri* Cokendolpher & Reddell
**Kendall**	
474 Cave	*Agyneta llanoensis* (Gertsch & Davis), *Eidmannella rostrata* Gertsch
A Hole	*Eidmannella rostrata* Gertsch
Behr’s Cave	*Agyneta llanoensis* (Gertsch & Davis), *Eidmannella pallida* (Emerton)
Cascade Caverns	*Cicurina varians* Gertsch & Mulaik, *Eidmannella rostrata* Gertsch
Cascade Sinkhole	*Eidmannella rostrata* Gertsch
Cave Without A Name	*Gaucelmus augustinus* Keyserling
Cave Without-a-Name [Century Caverns]	*Eidmannella rostrata* Gertsch
Cave-Without-A-Name–Dead Man’s Cave System	*Cicurina varians* Gertsch & Mulaik
Century Caverns	*Cicurina varians* Gertsch & Mulaik
Charley’s Downclimb Cave	*Agyneta llanoensis* (Gertsch & Davis)
Cole Ranch Cave No. 1	*Eidmannella rostrata* Gertsch
Covered Hole	*Agyneta llanoensis* (Gertsch & Davis)
Cricket Cave	*Cicurina varians* Gertsch & Mulaik, *Eidmannella rostrata* Gertsch
Cueva de los Tres Bobos	*Argiope aurantia* Lucas, *Eidmannella rostrata* Gertsch
Forget-Me-Not Cave	*Cicurina varians* Gertsch & Mulaik, *Eidmannella rostrata* Gertsch
Forlorn Hole	*Eidmannella rostrata* Gertsch
Georgia W. Cave	*Eidmannella rostrata* Gertsch
Gertrude’s Unknown Cave	*Cicurina varians* Gertsch & Mulaik
Glen Rose Cave	*Eidmannella rostrata* Gertsch
Grand Column Cave	*Eidmannella rostrata* Gertsch
Hal’s Cave	*Eidmannella rostrata* Gertsch
Jan’s Fissure	*Eidmannella rostrata* Gertsch, *Cryptachaea porteri* (Banks)
Knee Deep Cave	*Eidmannella rostrata* Gertsch, *Gaucelmus augustinus* Keyserling
Kohl Ranch Cave No. 1	*Cicurina varians* Gertsch & Mulaik
Pfeiffer Crawlway Cave	*Cicurina varians* Gertsch & Mulaik, *Eidmannella rostrata* Gertsch
Pfeiffer Dirt Sink	*Eidmannella rostrata* Gertsch
Pfeiffer’s Water Cave	*Agyneta llanoensis* (Gertsch & Davis), *Eidmannella rostrata* Gertsch, *Modisimus texanus* Banks
Prassel Ranch Cave	*Eidmannella rostrata* Gertsch
Sattler’s Deep Pit	*Agyneta llanoensis* (Gertsch & Davis), *Eidmannella pallida* (Emerton)
Schneider Ranch Cave	*Cicurina varians* Gertsch & Mulaik, *Eidmannella rostrata* Gertsch
Schroeder Bat Cave	*Agyneta llanoensis* (Gertsch & Davis)
Schwarz Cave	*Cicurina varians* Gertsch & Mulaik, *Eidmannella rostrata* Gertsch
Swaglet Cave	*Cicurina varians* Gertsch & Mulaik, *Eidmannella rostrata* Gertsch, *Cryptachaea porteri* (Banks)
Two Step Cave	*Eidmannella rostrata* Gertsch
**Kerr**	
Adam Wilson’s Cave	*Gaucelmus augustinus* Keyserling
East Trap Cave	*Cicurina varians* Gertsch & Mulaik, *Cryptachaea porteri* (Banks)
Goat Trap Cave	*Cicurina varians* Gertsch & Mulaik
Mingus Root Cave	*Cicurina varians* Gertsch & Mulaik, *Gaucelmus augustinus* Keyserling
Mingus Swallow Cave	*Cryptachaea porteri* (Banks)
Old Morris Cave	*Cicurina varians* Gertsch & Mulaik, *Cryptachaea porteri* (Banks)
Pinto Ranch Cave	*Cryptachaea porteri* (Banks)
Secrest Cave	*Cicurina varians* Gertsch & Mulaik
Seiker’s Cave	*Agyneta llanoensis* (Gertsch & Davis)
Seven Room Cave	*Cicurina varians* Gertsch & Mulaik, *Cryptachaea porteri* (Banks), *Parasteatoda tepidariorum* (C. L. Koch)
Smith Cave	*Gaucelmus augustinus* Keyserling
Stowers Cave	*Cicurina stowersi* Gertsch, *Cicurina varians* Gertsch & Mulaik, *Cryptachaea porteri* (Banks)
Water Pond Pasture Cave	*Cicurina pastura* Gertsch
Wilson Ranch Cave	*Agyneta llanoensis* (Gertsch & Davis), *Gaucelmus augustinus* Keyserling
**Kimble**	
700 Springs Cave	*Gaucelmus augustinus* Keyserling
Fleming Bat Cave	*Cicurina varians* Gertsch & Mulaik, *Mermessus antraeus* (Crosby), *Gaucelmus augustinus* Keyserling
Flemming Bat Cave	*Cicurina caverna* Gertsch, *Cryptachaea porteri* (Banks)
Garter Snake Cave	*Cicurina varians* Gertsch & Mulaik, *Drassyllus dromeus* Chamberlin, *Cryptachaea porteri* (Banks)
Live Dog Cave	*Cicurina varians* Gertsch & Mulaik, *Cryptachaea porteri* (Banks)
Lizard Cave	*Cicurina varians* Gertsch & Mulaik, *Cryptachaea porteri* (Banks)
Llewelyn Rose Cave	*Cicurina varians* Gertsch & Mulaik
The Hole	*Cicurina varians* Gertsch & Mulaik, *Cryptachaea porteri* (Banks)
Top Dog Cave	*Cicurina varians* Gertsch & Mulaik, *Cryptachaea porteri* (Banks)
**King**	
River Styx Cave	*Cicurina varians* Gertsch & Mulaik, *Scylaceus* sp., *Eidmannella pallida* (Emerton), *Eidmannella rostrata* Gertsch, *Metellina mimetoides* Chamberlin & Ivie, *Cryptachaea porteri* (Banks)
**Kinney**	
Bader Cave	*Cicurina varians* Gertsch & Mulaik
Baker’s Crossing Cave	*Eidmannella rostrata* Gertsch
Cot Cave	*Physocyclus enaulus* Crosby, *Cryptachaea porteri* (Banks)
Cricket Siphon Cave	*Cicurina varians* Gertsch & Mulaik
Kelley Cave	*Agyneta llanoensis* (Gertsch & Davis)
Kickapoo Caverns	*Cryptachaea porteri* (Banks)
Rattlesnake Cave	*Cicurina varians* Gertsch & Mulaik
Webb Cave	*Cicurina varians* Gertsch & Mulaik, *Agyneta llanoensis* (Gertsch & Davis), *Eidmannella pallida* (Emerton)
**Lampasas**	
Battery Cave	*Agyneta llanoensis* (Gertsch & Davis), *Ariadna bicolor* (Hentz), *Cryptachaea porteri* (Banks)
Dead Goat Cave	*Cryptachaea porteri* (Banks)
Enough Cave	*Cicurina varians* Gertsch & Mulaik, *Mermessus maculatus* (Banks)
Jackson Flea Cave	*Cryptachaea porteri* (Banks)
Jackson One-Bat Cave	*Cryptachaea porteri* (Banks)
**Llano**	
Double Door Cave	*Physocyclus enaulus* Crosby
Enchanted Rock Cave	*Eidmannella pallida* (Emerton), *Modisimus texanus* Banks, *Cryptachaea porteri* (Banks), *Parasteatoda tepidariorum* (C. L. Koch)
Miller’s Cave	*Cicurina varians* Gertsch & Mulaik, *Cryptachaea porteri* (Banks)
**Mason**	
Kothmann Cave	*Cicurina varians* Gertsch & Mulaik, *Agyneta llanoensis* (Gertsch & Davis), *Cryptachaea porteri* (Banks)
Mill Creek Cavern	*Cicurina varians* Gertsch & Mulaik, *Agyneta llanoensis* (Gertsch & Davis)
Zesch Ranch Cave	*Agyneta llanoensis* (Gertsch & Davis), *Cryptachaea porteri* (Banks)
**Medina**	
Boehme’s Cave	*Cicurina medina* Gertsch, *Cryptachaea porteri* (Banks)
Coontop Tip	*Cicurina varians* Gertsch & Mulaik
Davenport Cave	*Metellina mimetoides* Chamberlin & Ivie, *Eidmannella nasuta* Gertsch, *Eidmannella rostrata* Gertsch, *Cryptachaea porteri* (Banks)
Haby Bat Cave	*Leptoctenus byrrhus* Simon, *Cicurina varians* Gertsch & Mulaik, *Agyneta llanoensis* (Gertsch & Davis), *Cryptachaea porteri* (Banks)
Koch Cave	*Cicurina varians* Gertsch & Mulaik, *Agyneta llanoensis* (Gertsch & Davis), *Eidmannella rostrata* Gertsch
Lutz Cave	*Cicurina varians* Gertsch & Mulaik, *Cryptachaea porteri* (Banks)
Medina Dam Cave	*Tayshaneta whitei* Ledford et al.
Ney Cave	*Cicurina varians* Gertsch & Mulaik, *Urozelotes rusticus* (L. Koch), *Frontinella communis* (Hentz), *Latrodectus mactans* (Fabricius), *Cryptachaea porteri* (Banks)
Nisbet Cave	*Tayshaneta whitei* Ledford et al.
Surprise Cave	*Eidmannella rostrata* Gertsch
Valdina Farms Sinkhole	*Cicurina varians* Gertsch & Mulaik, *Eidmannella pallida* (Emerton), *Cryptachaea porteri* (Banks)
Weynand Cave	*Cicurina varians* Gertsch & Mulaik, *Latrodectus mactans* (Fabricius)
Windmill Cave	*Eidmannella rostrata* Gertsch
**Menard**	
Celery Creek Cave	*Cicurina varians* Gertsch & Mulaik
Kearney’s Dead Goat Cave	*Cicurina varians* Gertsch & Mulaik, *Cryptachaea porteri* (Banks)
Neel Cave and Powell’s Cave	*Cicurina varians* Gertsch & Mulaik
Neel’s Cave	*Eidmannella pallida* (Emerton)
Powell’s Cave	*Cicurina menardia* Gertsch, *Eidmannella pallida* (Emerton), *Cryptachaea porteri* (Banks)
Silver Mine Cave	*Eidmannella pallida* (Emerton)
**Pecos**	
Amazing Maze Cave	*Cicurina mirifica* Gertsch, *Cicurina varians* Gertsch & Mulaik, *Physocyclus enaulus* Crosby
Ess Cave	*Cryptachaea porteri* (Banks)
**Presidio**	
John’s Guano Mine	*Physocyclus enaulus* Crosby; *Mecaphesa coloradensis* (Gertsch)
**Randall**	
Big Rock Cave	*Latrodectus mactans* (Fabricius)
Catarina Cave	*Cryptachaea porteri* (Banks)
Confusion Cave	*Cryptachaea porteri* (Banks)
**Reagan**	
Big Lake State Park Cave	*Eidmannella pallida* (Emerton)
**Real**	
Bonner Fallout Shelter Cave	*Eidmannella pallida* (Emerton)
Cave of the Lakes	*Cicurina varians* Gertsch & Mulaik
Haby Cave	*Cicurina varians* Gertsch & Mulaik
Orell Bat Cave	*Cicurina varians* Gertsch & Mulaik, *Eidmannella rostrata* Gertsch, *Cryptachaea porteri* (Banks)
Orell Crevice Cave	*Cicurina orellia* Gertsch, *Kukulcania arizonica* (Chamberlin & Ivie), *Eidmannella rostrata* Gertsch, *Gaucelmus augustinus* Keyserling
Ramsey Bat Cave	*Cicurina orellia* Gertsch, *Cicurina sheari* Gertsch, *Cicurina varians* Gertsch & Mulaik
Red Arrow Cave	*Agyneta llanoensis* (Gertsch & Davis), *Cryptachaea porteri* (Banks)
Section 6 Cave	*Cicurina varians* Gertsch & Mulaik
Shellhammer Cave	*Gaucelmus augustinus* Keyserling
Skeleton Cave	*Cicurina varians* Gertsch & Mulaik, *Eidmannella rostrata* Gertsch
Tucker Hollow Cave	*Cicurina varians* Gertsch & Mulaik
Turkey Pens Cave	*Physocyclus enaulus* Crosby, *Loxosceles devia* Gertsch & Mulaik, *Cryptachaea porteri* (Banks)
Wilson Cave	*Cicurina varians* Gertsch & Mulaik
**San Saba**	
?Davenport Cave	*Metellina mimetoides* Chamberlin & Ivie
?Wedge Cave	*Metellina mimetoides* Chamberlin & Ivie
Blue Haw Cave	*Tidarren sisyphoides* (Walckenaer)
Bremer Cave	*Tegenaria pagana* C. L. Koch), *Cryptachaea porteri* (Banks)
Chimneyer’s Delight Cave	*Cryptachaea porteri* (Banks)
Cicurina Cave	*Cicurina varians* Gertsch & Mulaik, *Cryptachaea porteri* (Banks)
Cobweb Fissure	*Modisimus texanus* Banks, *Tidarren sisyphoides* (Walckenaer)
Copperhead Cave	*Dictyna bellans* Chamberlin, *Pirata sedentarius* Montgomery
Crevice Cave	*Tidarren sisyphoides* (Walckenaer)
Dove Cave	*Agelenopsis aleenae* Chamberlin & Ivie, *Latrodectus mactans* (Fabricius)
Fence Line Fissure	*Cryptachaea porteri* (Banks)
Fern Cave	*Eidmannella pallida* (Emerton)
Gorman Cave	*Cicurina sansaba* Gertsch, *Cicurina varians* Gertsch & Mulaik, *Agyneta llanoensis* (Gertsch & Davis), *Tidarren sisyphoides* (Walckenaer)
Harrell’s Cave	*Cicurina varians* Gertsch & Mulaik, *Agyneta llanoensis* (Gertsch & Davis)
Lemon’s Cave	*Cicurina varians* Gertsch & Mulaik, *Agyneta llanoensis* (Gertsch & Davis)
Lemons Ranch Cave	*Cicurina sansaba* Gertsch
Puberty Pit	*Cicurina varians* Gertsch & Mulaik
Springdale Ranch Cave	*Cicurina varians* Gertsch & Mulaik
Upper Cave	*Cicurina varians* Gertsch & Mulaik
Wedge Cave	*Cryptachaea porteri* (Banks), *Tidarren sisyphoides* (Walckenaer)
Whiteface Cave	*Cicurina machete* Gertsch, *Cicurina varians* Gertsch & Mulaik, *Agyneta llanoensis* (Gertsch & Davis)
**Schleicher**	
Cave Y	*Cicurina varians* Gertsch & Mulaik, *Agyneta llanoensis* (Gertsch & Davis), *Eidmannella pallida* (Emerton), *Cryptachaea porteri* (Banks)
Fartz Cave	*Physocyclus enaulus* Crosby
**Stonewall**	
Aspermont Bat Cave	*Eidmannella pallida* (Emerton), *Cryptachaea porteri* (Banks), *Latrodectus mactans* (Fabricius)
**Sutton**	
Alma’s Cave	*Physocyclus enaulus* Crosby
Caverns of Sonora (=Mayfield Cave)	*Cicurina barri* Gertsch, *Eidmannella pallida* (Emerton)
Felton Cave	*Metepeira labyrinthea* (Hentz), *Cicurina suttoni* Gertsch, *Cicurina varians* Gertsch & Mulaik, *Cryptachaea porteri* (Banks), *Mecaphesa celer* (Hentz)
Felton Cave Root	*Agyneta llanoensis* (Gertsch & Davis)
Harrison Cave	*Cicurina varians* Gertsch & Mulaik, *Agyneta llanoensis* (Gertsch & Davis)
Mayfield Cave	*Eidmannella pallida* (Emerton)
Silky Cave	*Cicurina varians* Gertsch & Mulaik, *Physocyclus enaulus* Crosby, *Cryptachaea porteri* (Banks)
Word Cave	*Physocyclus enaulus* Crosby, *Cryptachaea porteri* (Banks)
**Terrell**	
Bendele’s Uncave	*Loxosceles blanda* Gertsch & Ennik
Blackstone Cave	*Cryptachaea porteri* (Banks)
Goode Cave	*Cicurina varians* Gertsch & Mulaik, *Agyneta llanoensis* (Gertsch & Davis), *Hogna carolinensis* (Walckenaer), *Eidmannella rostrata* Gertsch
Longley Cave	*Leptoctenus byrrhus* Simon, *Cicurina varians* Gertsch & Mulaik
Pasotex Pit	*Cicurina varians* Gertsch & Mulaik, *Agyneta llanoensis* (Gertsch & Davis)
Sorcerer’s Cave	*Eidmannella pallida* (Emerton), *Physocyclus enaulus* Crosby
The Crack	*Agyneta llanoensis* (Gertsch & Davis)
Wizard’s Well	*Cicurina varians* Gertsch & Mulaik, *Cicurina venefica* Gertsch
**Travis**	
?La Crosse Cave	*Leucauge venusta* (Walckenaer)
3-Holer Cave	*Mermessus albulus* (Zorsch & Crosby)
9K-2 Cave (=Moonmilk Cave)	*Tayshaneta devia* (Gertsch)
Adobe Springs Cave	*Cicurina varians* Gertsch & Mulaik
Airman’s Cave	*Cicurina bandida* Gertsch, *Cicurina varians* Gertsch & Mulaik, *Eidmannella pallida* (Emerton), *Eidmannella rostrata* Gertsch
Amber Cave	*Cicurina travisae* Gertsch, *Cicurina varians* Gertsch & Mulaik, *Agyneta llanoensis* (Gertsch & Davis)
Armadillo Ranch Sink	*Agyneta llanoensis* (Gertsch & Davis)
Arrow Cave	*Cicurina varians* Gertsch & Mulaik
Austin Caverns	*Eucteniza relata* (O. P.-Cambridge), *Eidmannella pallida* (Emerton)
Backhole	*Agyneta micaria* (Emerton)
Backyard Cave	*Cicurina buwata* Chamberlin & Ivie
Balcones Sink	*Cryptachaea porteri* (Banks)
Bandit Cave	*Cicurina bandida* Gertsch, *Cicurina varians* Gertsch & Mulaik
Beckett’s Cave	*Cicurina varians* Gertsch & Mulaik, *Cryptachaea porteri* (Banks
Bee Creek Cave	*Cicurina varians* Gertsch & Mulaik
Beer Bottle Cave	*Cicurina varians* Gertsch & Mulaik
Blowing Sink	*Cicurina bandida* Gertsch
Brew Pot Sink	*Cicurina varians* Gertsch & Mulaik
Brewpot Sink	*Tayshaneta devia* (Gertsch)
Brodie Sink	*Eidmannella pallida* (Emerton)
Broken Arrow Cave	*Cicurina travisae* Gertsch, *Cicurina varians* Gertsch & Mulaik, *Agyneta llanoensis* (Gertsch & Davis)
Broken Lid Cave	*Cryptachaea porteri* (Banks)
Broken Straw Cave	*Eidmannella pallida* (Emerton)
Cave site #401	*Agyneta llanoensis* (Gertsch & Davis)
Cave X	*Cicurina bandida* Gertsch, *Eidmannella pallida* (Emerton), *Cryptachaea porteri* (Banks)
Cave Y	*Cicurina varians* Gertsch & Mulaik, *Cryptachaea porteri* (Banks)
Ceiling Slot Cave	*Agyneta llanoensis* (Gertsch & Davis)
Central Sink	*Cryptachaea porteri* (Banks)
Chuck’s Joint	*Agyneta llanoensis* (Gertsch & Davis)
Cold Cave	*Cicurina varians* Gertsch & Mulaik, *Cryptachaea porteri* (Banks)
Coon Slide Cave	*Agyneta llanoensis* (Gertsch & Davis)
Cortaña Cave	*Tayshaneta myopica* (Gertsch)
Cotterell Cave	*Cicurina buwata* Chamberlin & Ivie, *Cicurina travisae* Gertsch, *Agyneta llanoensis* (Gertsch & Davis), *Eidmannella pallida* (Emerton), *Cryptachaea porteri* (Banks)
County Line Bat Cave	*Tayshaneta concinna* (Gertsch)
Dead Dog Cave No. 1	*Cicurina varians* Gertsch & Mulaik, *Cryptachaea porteri* (Banks)
Deer Stand Cave	*Modisimus texanus* Banks, *Cryptachaea porteri* (Banks)
District Park Cave	*Tayshaneta sandersi* Ledford et al., *Mermessus albulus* (Zorsch & Crosby)
Dobie Shelter	*Orthonops lapanus* Gertsch & Mulaik, *Modisimus texanus* Banks, *Hyptiotes cavatus* (Hentz)
Driskill Cave	*Cicurina bandida* Gertsch, *Cicurina varians* Gertsch & Mulaik, *Agyneta llanoensis* (Gertsch & Davis), *Eidmannella pallida* (Emerton), *Cryptachaea porteri* (Banks)
Feather Sink	*Eidmannella rostrata* Gertsch, *Cryptachaea porteri* (Banks)
Five Pocket Cave	*Falconina gracilis* (Keyserling), *Eidmannella rostrata* Gertsch
Flint Ridge Cave	*Cicurina bandida* Gertsch, *Eidmannella pallida* (Emerton)
Fossil Cave	*Cicurina varians* Gertsch & Mulaik
Fossil Garden Cave	*Cicurina buwata* Chamberlin & Ivie
Gallifer Cave	*Cicurina buwata* Chamberlin & Ivie, *Cicurina varians* Gertsch & Mulaik, *Tayshaneta myopica* (Gertsch), *Cryptachaea porteri* (Banks)
GCWA Cave	*Agyneta llanoensis* (Gertsch & Davis)
Geode Cave	*Tayshaneta myopica* (Gertsch)
Get Down Cave	*Cicurina bandida* Gertsch, *Cryptachaea porteri* (Banks), *Tidarren sisyphoides* (Walckenaer)
Goat Cave	*Cicurina varians* Gertsch & Mulaik, *Eidmannella pallida* (Emerton), *Cryptachaea porteri* (Banks)
Grove Sinks Cave	*Cicurina varians* Gertsch & Mulaik, *Cryptachaea porteri* (Banks)
Hammett’s Crossing	*Tayshaneta devia* (Gertsch)
Hideout Cave	*Cicurina varians* Gertsch & Mulaik
Hole in the Road	*Cicurina varians* Gertsch & Mulaik
Ireland’s Cave	*Cicurina bandida* Gertsch, *Cicurina varians* Gertsch & Mulaik, *Eidmannella pallida* (Emerton), *Eidmannella rostrata* Gertsch
Jack’s Joint	*Agyneta llanoensis* (Gertsch & Davis), *Eidmannella pallida* (Emerton), *Eidmannella rostrata* Gertsch, *Cryptachaea porteri* (Banks)
Jack’s Joint Cave	*Agyneta llanoensis* (Gertsch & Davis)
Jest John Cave	*Cicurina varians* Gertsch & Mulaik, *Agyneta llanoensis* (Gertsch & Davis)
Jester Estate’s Cave	*Tayshaneta myopica* (Gertsch)
Jester Pit	*Eidmannella pallida* (Emerton)
Jollyville Plateau Cave	*Agyneta llanoensis* (Gertsch & Davis)
Ken Harrell Cave	*Cicurina varians* Gertsch & Mulaik
Kretschmarr Cave	*Cicurina travisae* Gertsch
Kretschmarr Double Pit	*Cicurina travisae* Gertsch, *Agyneta llanoensis* (Gertsch & Davis), *Cryptachaea porteri* (Banks)
Kretschmarr Fluted Sink	*Cicurina varians* Gertsch & Mulaik, *Cryptachaea porteri* (Banks)
Kretschmarr Salamander Cave	*Cicurina varians* Gertsch & Mulaik, *Eidmannella pallida* (Emerton), *Eidmannella rostrata* Gertsch
Kretschmarr Sink	*Cicurina varians* Gertsch & Mulaik
LaCrosse Cave	*Cicurina varians* Gertsch & Mulaik, *Cryptachaea porteri* (Banks)
Lost Gold Cave	*Cicurina bandida* Gertsch, *Cicurina varians* Gertsch & Mulaik, *Tayshaneta concinna* (Gertsch), *Gaucelmus augustinus* Keyserling, *Cryptachaea porteri* (Banks)
Lost Oasis Cave	*Cicurina bandida* Gertsch
Lundsford’s Cave	*Eidmannella pallida* (Emerton)
Lunsford Cave	*Agyneta llanoensis* (Gertsch & Davis), *Camptocosa parallela* (Banks)
Lunsford’s Cave	*Cicurina varians* Gertsch & Mulaik, *Agyneta llanoensis* (Gertsch & Davis), *Schizocosa saltatrix* (Hentz)
MacDonald Cave (=Schultz Cave)	*Tayshaneta devia* (Gertsch)
Maple Run Cave	*Cicurina bandida* Gertsch, *Cicurina varians* Gertsch & Mulaik
McDonald Cave	*Cicurina varians* Gertsch & Mulaik, *Eidmannella rostrata* Gertsch, *Cryptachaea porteri* (Banks)
McDonald Cave (=Schulze Cave)	*Cicurina travisae* Gertsch, *Eidmannella reclusa* Gertsch
McNeil Bat Cave	*Cicurina buwata* Chamberlin & Ivie, *Cicurina varians* Gertsch & Mulaik, *Tayshaneta myopica* (Gertsch)
Midden Sink	*Agyneta llanoensis* (Gertsch & Davis), *Cryptachaea porteri* (Banks)
Midnight Cave	*Eidmannella pallida* (Emerton), *Eidmannella rostrata* Gertsch
Moonmilk Cave	*Eidmannella pallida* (Emerton)
Moss Pit	*Cicurina varians* Gertsch & Mulaik, *Mermessus albulus* (Zorsch & Crosby)
New Comanche Trail Cave	*Cicurina varians* Gertsch & Mulaik, *Tayshaneta myopica* (Gertsch), *Modisimus texanus* Banks
Night Sink	*Cicurina varians* Gertsch & Mulaik
No Rent Cave	*Cicurina varians* Gertsch & Mulaik, *Agyneta llanoensis* (Gertsch & Davis), *Mermessus albulus* (Zorsch & Crosby), *Cryptachaea porteri* (Banks)
North Root Cave	*Cicurina travisae* Gertsch
Northwoods Cave	*Cicurina varians* Gertsch & Mulaik
Outhouse Hole Sink	*Cryptachaea porteri* (Banks)
Pickle Pit	*Cicurina travisae* Gertsch, *Cryptachaea porteri* (Banks)
Pickle Pit Cave	*Cicurina travisae* Gertsch
Pisarowicz Cave	*Cicurina travisae* Gertsch
Plethodon Cave	*Eidmannella pallida* (Emerton), *Eidmannella reclusa* Gertsch
Puzzle Pit	*Eidmannella reclusa* Gertsch
Rockpile Cave	*Modisimus texanus* Banks
Rolling Rock Cave	*Cicurina varians* Gertsch & Mulaik, *Agyneta llanoensis* (Gertsch & Davis)
Root Cave	*Agelenopsis aperta* (Gertsch), *Cicurina travisae* Gertsch, *Tayshaneta myopica* (Gertsch)
Salamander Cave	*Cicurina travisae* Gertsch, *Cryptachaea porteri* (Banks)
Schulze Cave	*Cicurina varians* Gertsch & Mulaik, *Eidmannella rostrata* Gertsch, *Cryptachaea porteri* (Banks)
Seibert Sink (Stinkin Sink)	*Tayshaneta concinna* (Gertsch)
Seider Springs Cave	*Cryptachaea porteri* (Banks)
Singletary Cave	*Cryptachaea porteri* (Banks)
Slaughter Creek Cave	*Tayshaneta sandersi* Ledford et al.
Slumberger Sink	*Cryptachaea porteri* (Banks)
Spanish Wells	*Cicurina varians* Gertsch & Mulaik, *Cryptachaea porteri* (Banks)
Spider Cave	*Cicurina travisae* Gertsch, *Eidmannella pallida* (Emerton)
Spyglass Cave	*Eidmannella pallida* (Emerton)
Stark’s North Mine	*Cicurina varians* Gertsch & Mulaik, *Tayshaneta concinna* (Gertsch), *Cryptachaea porteri* (Banks)
Steiner Telephone Pole Cave	*Tayshaneta myopica* (Gertsch)
Stoneworks Sink	*Cryptachaea porteri* (Banks)
Stovepipe Cave	*Cicurina varians* Gertsch & Mulaik, *Tayshaneta devia* (Gertsch), *Eidmannella reclusa* Gertsch
Substations Sink	*Cryptachaea porteri* (Banks)
Tardus Hole	*Cryptachaea porteri* (Banks)
Three-Holer Cave	*Cicurina varians* Gertsch & Mulaik, *Agyneta flax* Dupérré, *Cryptachaea porteri* (Banks)
Tight Pit	*Tayshaneta myopica* (Gertsch)
Tooth Cave	*Cicurina travisae* Gertsch, *Cicurina varians* Gertsch & Mulaik, *Tayshaneta myopica* (Gertsch), *Eidmannella reclusa* Gertsch, *Eidmannella rostrata* Gertsch, *Anapistula secreta* Gertsch, *Cryptachaea porteri* (Banks)
Tooth Cave surface	*Tayshaneta devia* (Gertsch)
Twelve Foot Dome	*Eidmannella reclusa* Gertsch
Twin Dig Pit	*Cicurina varians* Gertsch & Mulaik
Two Trunks Cave	*Agyneta llanoensis* (Gertsch & Davis)
Ulls Water Cave	*Eidmannella reclusa* Gertsch
Wade Sink	*Cicurina varians* Gertsch & Mulaik, *Mermessus albulus* (Zorsch & Crosby)
Weldon Cave	*Cicurina varians* Gertsch & Mulaik, *Agyneta llanoensis* (Gertsch & Davis), *Cryptachaea porteri* (Banks)
Weldon West Cave	*Cicurina varians* Gertsch & Mulaik, *Cryptachaea porteri* (Banks)
West Cave	*Dolomedes scriptus* Hentz
Whirlpool Cave	*Cicurina varians* Gertsch & Mulaik, *Tayshaneta sandersi* Ledford et al., *Eidmannella pallida* (Emerton), *Cryptachaea porteri* (Banks)
Wildflower Cave	*Cryptachaea porteri* (Banks)
Windmill Cave	*Agyneta llanoensis* (Gertsch & Davis)
Wooden Derrick Cave	*Eidmannella pallida* (Emerton)
**Uvalde**	
?Indian Creek Cave	*Cicurina varians* Gertsch & Mulaik
Barn-Sized Fissure Cave	*Agyneta llanoensis* (Gertsch & Davis), *Eidmannella rostrata* Gertsch
BFS Cave	*Cicurina varians* Gertsch & Mulaik
Big Foot Cave	*Cryptachaea porteri* (Banks)
Big Fucking Snake Cave	*Tayshaneta valverdae* (Gertsch)
Burial Cave	*Cicurina varians* Gertsch & Mulaik, *Cryptachaea porteri* (Banks), *Zorocrates aemulus* Gertsch
Carson Cave	*Cicurina varians* Gertsch & Mulaik
Cave Hollow Cave	*Eidmannella rostrata* Gertsch
Cement Tank Cave	*Cryptachaea porteri* (Banks)
Crom Cave	*Cryptachaea porteri* (Banks)
Davy Crockett Cave	*Cryptachaea porteri* (Banks)
Frio Bat Cave	*Scotophaeus blackwalli* (Thorell), *Cryptachaea porteri* (Banks)
Frio King Cave	*Cryptachaea porteri* (Banks)
Frio Queen Cave	*Cicurina watersi* Gertsch
Grape Hollow Cave	*Cicurina varians* Gertsch & Mulaik
Indian Creek Cave	*Eidmannella rostrata* Gertsch
Maybe Stream Cave	*Cicurina varians* Gertsch & Mulaik, *Eidmannella rostrata* Gertsch, *Cryptachaea porteri* (Banks)
Moss Pit Cave	*Modisimus texanus* Banks
North Well Cave	*Cicurina serena* Gertsch, *Eidmannella pallida* (Emerton), *Cryptachaea porteri* (Banks)
Pablo’s Cave	*Cicurina pablo* Gertsch, *Eidmannella pallida* (Emerton), *Cryptachaea porteri* (Banks)
Picture Cave No. 1	*Cicurina serena* Gertsch, *Cicurina varians* Gertsch & Mulaik, *Cryptachaea porteri* (Banks)
Rambie’s Cave	*Cicurina uvalde* Gertsch, *Cicurina varians* Gertsch & Mulaik, *Eidmannella pallida* (Emerton)
Sandtleben Cave (=Davy Crockett Cave)	*Cicurina selecta* Gertsch, *Cryptachaea porteri* (Banks), *Cicurina varians* Gertsch & Mulaik
Story Cave	*Eidmannella pallida* (Emerton)
Tampke Ranch Cave	*Cicurina varians* Gertsch & Mulaik, *Agyneta llanoensis* (Gertsch & Davis), *Eidmannella rostrata* Gertsch, *Gaucelmus augustinus* Keyserling, *Physocyclus enaulus* Crosby, *Loxosceles devia* Gertsch & Mulaik, *Loxosceles reclusa* Gertsch & Mulaik, *Metellina mimetoides* Chamberlin & Ivie, *Cryptachaea porteri* (Banks)
West Holler Cave	*Cicurina varians* Gertsch & Mulaik
Whitecotton Bat Cave	*Agyneta llanoensis* (Gertsch & Davis), *Eidmannella rostrata* Gertsch, *Cryptachaea porteri* (Banks)
**Val Verde**	
Airport Cave	*Eidmannella pallida* (Emerton)
Arledge Bat Cave	*Cicurina varians* Gertsch & Mulaik
Cave 8	*Ctenus valverdiensis* Peck
Cave No. 8	*Cicurina delrio* Gertsch
Cave Hollow Cave	*Cicurina varians* Gertsch & Mulaik, *Eidmannella rostrata* Gertsch
Centipede Cave	*Cicurina varians* Gertsch & Mulaik
Diablo Cave	*Ctenus valverdiensis* Peck, *Leptoctenus byrrhus* Simon, *Cicurina delrio* Gertsch
East Gypsum Cave	*Ctenus valverdiensis* Peck
Emerald Sink	*Cicurina varians* Gertsch & Mulaik, *Tayshaneta emeraldae* Ledford et al., *Eidmannella pallida* (Emerton), *Psilochorus imitatus* Gertsch & Mulaik
Fawcett’s Cave	*Cicurina patei* Gertsch, *Eidmannella pallida* (Emerton), *Cryptachaea porteri* (Banks), *Misumena vatia* (Clerck)
Fawcett’s Cave [Devil’s River State Natural Area]	*Tayshaneta fawcetti* Ledford et al.
Fern Cave	*Cicurina varians* Gertsch & Mulaik, *Hogna antelucana* (Montgomery), *Hogna carolinensis* (Walckenaer), *Eidmannella pallida* (Emerton)
Four-Mile Cave	*Eulaira suspecta* Gertsch & Mulaik, *Eidmannella pallida* (Emerton), *Cryptachaea porteri* (Banks)
H. T. Miers Cave	*Cicurina varians* Gertsch & Mulaik, *Agyneta llanoensis* (Gertsch & Davis), *Mermessus maculatus* (Banks), *Eidmannella pallida* (Emerton)
Ladder Cave	*Ctenus valverdiensis* Peck, *Leptoctenus byrrhus* Simon, *Eidmannella delicata* Gertsch
Langtry East Gypsum Cave	*Ctenus valverdiensis* Peck, *Leptoctenus byrrhus* Simon
Langtry Lead Cave	*Cicurina varians* Gertsch & Mulaik, *Eidmannella pallida* (Emerton)
Langtry Quarry Cave	*Cicurina varians* Gertsch & Mulaik
Litter Barrel Cave	*Cicurina varians* Gertsch & Mulaik, *Physocyclus enaulus* Crosby
Litterbarrel Cave	*Tayshaneta grubbsi* Ledford et al.
Marshall Bat Cave	*Filistatinella crassipalpis* (Gertsch)
Oriente Milestone Molasses Bat Cave	*Cicurina porteri* Gertsch, *Cicurina varians* Gertsch & Mulaik, *Tayshaneta valverdae* (Gertsch), *Eidmannella pallida* (Emerton), *Loxosceles blanda* Gertsch & Ennik
Plecotus Cave	*Physocyclus enaulus* Crosby
Popcorn Ball Cave	*Masoncus conspectus* (Gertsch & Davis)
Powers Ranch Bat Cave	*Agyneta llanoensis* (Gertsch & Davis)
Robertson Mill Dirt Cave	*Cicurina varians* Gertsch & Mulaik
Seminole Canyon Cave	*Cicurina holsingeri* Gertsch
Seminole Sink [Seminole Canyon State Historical Park]	*Eidmannella pallida* (Emerton), *Loxosceles blanda* Gertsch & Ennik
Sunset Cave	*Cicurina delrio* Gertsch
Tarantula Cave	*Ctenus valverdiensis* Peck
Twin Tree Cave	*Cicurina varians* Gertsch & Mulaik
Unnamed Cave No. 8	*Leptoctenus byrrhus* Simon
Wren Cave	*Agyneta llanoensis* (Gertsch & Davis), *Camptocosa parallela* (Banks), *Schizocosa saltatrix* (Hentz), *Zorocrates aemulus* Gertsch
**Ward**	
Rattlesnake Cave	*Eidmannella pallida* (Emerton)
**Washington**	
Devil’s Den	*Eidmannella pallida* (Emerton)
**Wheeler**	
Big Mouth Cave	*Islandiana unicornis* Ivie, *Cryptachaea porteri* (Banks), *Metellina mimetoides* Chamberlin & Ivie
Small Mouth Cave	*Cicurina varians* Gertsch & Mulaik
**Williamson**	
?Ballroom Cave No. 2	*Cicurina varians* Gertsch & Mulaik
?Bone Cave	*Cicurina varians* Gertsch & Mulaik
?Chinaberry Cave	*Cicurina varians* Gertsch & Mulaik
A. J. & B. L. Wilcox Cave	*Agyneta llanoensis* (Gertsch & Davis)
Agave Cave	*Eidmannella pallida* (Emerton)
Argo Cave	*Eidmannella pallida* (Emerton)
Avant Ranch Cave	*Agyneta llanoensis* (Gertsch & Davis)
Avery Ranch Cave	*Agyneta llanoensis* (Gertsch & Davis), *Mermessus albulus* (Zorsch & Crosby)
Avery Stairstep Cave	*Agyneta llanoensis* (Gertsch & Davis)
Ballroom #2 Cave	*Agyneta llanoensis* (Gertsch & Davis)
Ballroom Cave No. 2	*Eidmannella pallida* (Emerton)
Bat Well	*Cicurina varians* Gertsch & Mulaik
Bat Well Cave	*Agyneta llanoensis* (Gertsch & Davis), *Eidmannella pallida* (Emerton)
Beck Bat Cave	*Cicurina varians* Gertsch & Mulaik, *Agyneta llanoensis* (Gertsch & Davis)
Beck Creek Cave	*Agyneta llanoensis* (Gertsch & Davis), *Eidmannella pallida* (Emerton)
Beck Crevice Cave	*Agyneta llanoensis* (Gertsch & Davis), *Mermessus albulus* (Zorsch & Crosby)
Beck Horse Cave	*Cicurina varians* Gertsch & Mulaik, *Agyneta llanoensis* (Gertsch & Davis), *Mermessus albulus* (Zorsch & Crosby)
Beck Pride Cave	*Agyneta llanoensis* (Gertsch & Davis), *Eidmannella pallida* (Emerton)
Beck Ranch Cave	*Cicurina varians* Gertsch & Mulaik, *Agyneta llanoensis* (Gertsch & Davis), *Eidmannella pallida* (Emerton)
Beck Rattlesnake Cave	*Agyneta llanoensis* (Gertsch & Davis)
Beck Sewer Cave	*Agyneta llanoensis* (Gertsch & Davis), *Cryptachaea porteri* (Banks)
Beck’s Sewer Cave	*Cicurina buwata* Chamberlin & Ivie *Cicurina varians* Gertsch & Mulaik
Behren’s Ranch Cave	*Agyneta llanoensis* (Gertsch & Davis)
Bev’s Grotto	*Cicurina buwata* Chamberlin & Ivie, *Cryptachaea porteri* (Banks)
Blowhole Cave	*Agyneta llanoensis* (Gertsch & Davis)
Blue Wasp Cave	*Cicurina varians* Gertsch & Mulaik
Bonito Sink Cave	*Gaucelmus augustinus* Keyserling
Boyd’s Void Cave	*Agyneta llanoensis* (Gertsch & Davis)
Brents Bad Air Cave	*Eidmannella pallida* (Emerton)
Broken Knife Sink	*Cicurina varians* Gertsch & Mulaik
Broken Plate Cave	*Agyneta llanoensis* (Gertsch & Davis)
Brown’s Cave	*Cicurina browni* Gertsch, *Agyneta llanoensis* (Gertsch & Davis), *Eidmannella pallida* (Emerton)
Buttercup Blow Hole Cave	*Agyneta llanoensis* (Gertsch & Davis)
Buttercup River Cave	*Cicurina buwata* Chamberlin & Ivie
Cassidy Cave	*Eidmannella pallida* (Emerton)
Cat Cave	*Agyneta llanoensis* (Gertsch & Davis)
Cat Hollow Bat Cave	*Agyneta llanoensis* (Gertsch & Davis)
Cat Hollow Cave No. 3	*Agyneta llanoensis* (Gertsch & Davis)
Cave Coral Cave	*Agyneta llanoensis* (Gertsch & Davis)
Chagas Cave	*Agyneta llanoensis* (Gertsch & Davis)
Clan Cave	*Agyneta llanoensis* (Gertsch & Davis)
Cobb Cavern (=Cobb’s Caverns)	*Cicurina varians* Gertsch & Mulaik, *Tayshaneta anopica* (Gertsch), *Agyneta llanoensis* (Gertsch & Davis), *Eidmannella pallida* (Emerton)
Coffin Cave	*Cicurina varians* Gertsch & Mulaik, *Eidmannella pallida* (Emerton)
Coon Scat Cave	*Cryptachaea porteri* (Banks)
Core Barrel Cave	*Cicurina varians* Gertsch & Mulaik, *Mermessus albulus* (Zorsch & Crosby)
Corn Cobb’s Cave	*Tayshaneta anopica* (Gertsch)
Cricket Cave	*Cicurina varians* Gertsch & Mulaik, *Agyneta llanoensis* (Gertsch & Davis)
Dead Ash Cave	*Cicurina varians* Gertsch & Mulaik
Deliverance Cave No. 1	*Eidmannella pallida* (Emerton)
Desert Dune Cave	*Cicurina varians* Gertsch & Mulaik
Dion Cave	*Agyneta llanoensis* (Gertsch & Davis)
Do Drop In Cave	*Eidmannella pallida* (Emerton)
Double Dog Hole Cave	*Eidmannella rostrata* Gertsch
Double Nickel Cave	*Agyneta llanoensis* (Gertsch & Davis)
Duckworth Bat Cave	*Agyneta llanoensis* (Gertsch & Davis), *Eidmannella pallida* (Emerton)
Dynamite Cave	*Gaucelmus augustinus* Keyserling
East Fork Fissure	*Eidmannella pallida* (Emerton), *Eidmannella rostrata* Gertsch
Electro-Mag Cave	*Eidmannella pallida* (Emerton), *Anapistula secreta* Gertsch
Elm Bat Cave	*Cicurina varians* Gertsch & Mulaik, *Cryptachaea porteri* (Banks)
Elm Cave	*Cicurina varians* Gertsch & Mulaik, *Cryptachaea porteri* (Banks)
Elm Water Cave	*Cicurina varians* Gertsch & Mulaik, *Eidmannella pallida* (Emerton)
Feature No. 1	*Agyneta llanoensis* (Gertsch & Davis)
Fern Bluff Cave	*Cicurina varians* Gertsch & Mulaik
Fern Cave	*Agyneta llanoensis* (Gertsch & Davis)
Fissure F-8 [The Sanctuary]	*Tayshaneta paraconcinna* (Cokendolpher & Reddell)
Flat Rock Cave	*Tayshaneta coeca* (Chamberlin & Ivie)
Flint Wash Cave	*Cicurina varians* Gertsch & Mulaik
Florence Cave No. 18	*Eidmannella pallida* (Emerton)
Formation Forest Cave	*Cryptachaea porteri* (Banks)
Fortune 500 Cave	*Agyneta llanoensis* (Gertsch & Davis)
Four-Corners Cave	*Cicurina varians* Gertsch & Mulaik
Goat Cave	*Tayshaneta myopica* (Gertsch)
Godwin’s Goat Grave Cave (=Lift Station Cave)	*Agyneta llanoensis* (Gertsch & Davis)
Good Friday Cave	*Cicurina buwata* Chamberlin & Ivie, *Cryptachaea porteri* (Banks)
Grimace Cave	*Agyneta llanoensis* (Gertsch & Davis), *Cryptachaea porteri* (Banks)
Hatchet Cave	*Agyneta llanoensis* (Gertsch & Davis), *Eidmannella pallida* (Emerton)
Holler Hole Cave	*Agyneta llanoensis* (Gertsch & Davis), *Eidmannella pallida* (Emerton)
Hook Cave	*Agyneta llanoensis* (Gertsch & Davis)
Ilex Cave	*Agyneta llanoensis* (Gertsch & Davis)
Inner Space Caverns	*Eidmannella pallida* (Emerton), *Phidippus texanus* Banks
Joker Cave	*Agyneta llanoensis* (Gertsch & Davis)
Jug Cave	*Cicurina varians* Gertsch & Mulaik, *Agyneta llanoensis* (Gertsch & Davis), *Cryptachaea porteri* (Banks), *Tidarren sisyphoides* (Walckenaer)
Killian Caver	*Agyneta llanoensis* (Gertsch & Davis)
Kiva Cave No. 1	*Eidmannella pallida* (Emerton)
Ku Klux Klan Cave	*Cryptachaea porteri* (Banks)
LakeLine Cave	*Agyneta llanoensis* (Gertsch & Davis)
LakeLine Mall Well Trap No. 3	*Agyneta llanoensis* (Gertsch & Davis)
Leaning Tree Cave	*Agyneta llanoensis* (Gertsch & Davis)
Life Station Cave	*Cicurina varians* Gertsch & Mulaik
Little Lake Cave	*Hogna carolinensis* (Walckenaer)
Lizard’s Lounge Cave [F-11]	*Tayshaneta paraconcinna* (Cokendolpher & Reddell)
Lobo’s Lair	*Mermessus albulus* (Zorsch & Crosby)
Lorfing’s Unseen Rattler Cave	*Cicurina varians* Gertsch & Mulaik, *Eidmannella pallida* (Emerton), *Cryptachaea porteri* (Banks)
Man-With-A-Spear Cave	*Cicurina varians* Gertsch & Mulaik, *Agyneta llanoensis* (Gertsch & Davis), *Cryptachaea porteri* (Banks)
Marigold Cave	*Cicurina buwata* Chamberlin & Ivie, *Cicurina varians* Gertsch & Mulaik, *Cryptachaea porteri* (Banks)
Maverick Cave	*Agyneta llanoensis* (Gertsch & Davis)
Mayfield Cave	*Agyneta llanoensis* (Gertsch & Davis)
McNeil Bat Cave	*Tayshaneta myopica* (Gertsch)
McNeil Quarry Cave	*Cicurina buwata* Chamberlin & Ivie, *Cicurina varians* Gertsch & Mulaik, *Eidmannella pallida* (Emerton)
Medicine Man Cave	*Agyneta llanoensis* (Gertsch & Davis)
Millennium Cave	*Agyneta llanoensis* (Gertsch & Davis)
Mongo Cave	*Agyneta llanoensis* (Gertsch & Davis)
Mosquito Cave	*Cicurina varians* Gertsch & Mulaik
Muscle Sink	*Cicurina varians* Gertsch & Mulaik
Mustard Cave	*Agyneta llanoensis* (Gertsch & Davis)
Near Miss Cave	*Agyneta llanoensis* (Gertsch & Davis)
O’Connor Cave	*Agyneta llanoensis* (Gertsch & Davis)
Off Campus Cave	*Agyneta llanoensis* (Gertsch & Davis), *Eidmannella pallida* (Emerton)
On Campus Cave	*Tayshaneta paraconcinna* (Cokendolpher & Reddell)
Onion Branch Cave	*Eidmannella pallida* (Emerton)
Paleospring Cave	*Agyneta llanoensis* (Gertsch & Davis)
Pemmican Cave	*Agyneta llanoensis* (Gertsch & Davis)
Polaris Cave	*Eidmannella pallida* (Emerton)
Prairie Flats Cave	*Agyneta llanoensis* (Gertsch & Davis)
Prairie’s Flats Cave	*Tayshaneta coeca* (Chamberlin & Ivie)
Price Is Right Cave	*Agyneta llanoensis* (Gertsch & Davis)
Prospectors Cave	*Agyneta llanoensis* (Gertsch & Davis)
Pussy Cat Cave	*Cicurina varians* Gertsch & Mulaik
Raccoon Cave	*Cicurina varians* Gertsch & Mulaik, *Agyneta llanoensis* (Gertsch & Davis)
Ramsel’s Corral Cave	*Cicurina varians* Gertsch & Mulaik
Rattlesnake Filled Cave	*Cicurina buwata* Chamberlin & Ivie, *Cicurina varians* Gertsch & Mulaik, *Cicurina vibora* Gertsch, *Agyneta llanoensis* (Gertsch & Davis)
Reach-Around Cave	*Eidmannella pallida* (Emerton)
Rock Ridge Cave	*Agyneta llanoensis* (Gertsch & Davis)
Rockfall Cave	*Agyneta llanoensis* (Gertsch & Davis), *Eidmannella pallida* (Emerton)
Rootin Tootin Cave	*Agyneta llanoensis* (Gertsch & Davis)
Salamander Squeeze Cave	*Agyneta llanoensis* (Gertsch & Davis)
Salt Lick Cave [The Sanctuary]	*Tayshaneta paraconcinna* (Cokendolpher & Reddell)
Scoot Over Cave	*Tayshaneta paraconcinna* (Cokendolpher & Reddell)
Serta Cave	*Tayshaneta paraconcinna* (Cokendolpher & Reddell)
Shell Cave	*Anapistula secreta* Gertsch
Short Stack Cave	*Tayshaneta paraconcinna* (Cokendolpher & Reddell), *Gaucelmus augustinus* Keyserling
Snowmelt Cave	*Agyneta llanoensis* (Gertsch & Davis)
Sore-ped Cave	*Eidmannella pallida* (Emerton), *Gaucelmus augustinus* Keyserling, *Cryptachaea porteri* (Banks)
Squeeze-Down Cave	*Agyneta llanoensis* (Gertsch & Davis)
Steam Cave	*Argiope aurantia* Lucas, *Cicurina varians* Gertsch & Mulaik, *Rabidosa rabida* (Walckenaer), *Eidmannella pallida* (Emerton), *Cryptachaea porteri* (Banks)
Steiner Telephone Pole Cave	*Tayshaneta myopica* (Gertsch)
Stepstone Cave	*Agyneta llanoensis* (Gertsch & Davis)
Sting Cave	*Eidmannella pallida* (Emerton)
Sunless City Cave	*Cicurina varians* Gertsch & Mulaik, *Cicurina vibora* Gertsch
Susana Cave	*Cicurina varians* Gertsch & Mulaik, *Mermessus albulus* (Zorsch & Crosby), *Cryptachaea porteri* (Banks)
T.W.A.S. A Cave	*Cicurina buwata* Chamberlin & Ivie, *Cryptachaea porteri* (Banks)
Temples of Thor Cave	*Cicurina varians* Gertsch & Mulaik, *Cicurina vibora* Gertsch, *Eidmannella rostrata* Gertsch
Terrell’s Cave	*Cicurina varians* Gertsch & Mulaik
Testudo Cave	*Cicurina travisae* Gertsch, *Agyneta llanoensis* (Gertsch & Davis), *Mermessus albulus* (Zorsch & Crosby)
Testudo Tube	*Cicurina buwata* Chamberlin & Ivie
Texella Cave	*Cicurina varians* Gertsch & Mulaik, *Agyneta llanoensis* (Gertsch & Davis), *Eidmannella pallida* (Emerton)
Texella Cave Karst Park	*Mermessus albulus* (Zorsch & Crosby)
The Abyss	*Agyneta llanoensis* (Gertsch & Davis)
The Bat Well	*Eidmannella pallida* (Emerton)
The Chimney	*Cicurina varians* Gertsch & Mulaik, *Agyneta llanoensis* (Gertsch & Davis)
Thin Roof Cave	*Agyneta llanoensis* (Gertsch & Davis)
Three-Mile Cave	*Cicurina varians* Gertsch & Mulaik, *Eidmannella pallida* (Emerton), *Gaucelmus augustinus* Keyserling, *Cryptachaea porteri* (Banks)
Three Miles Cave (=Three Mile Bat Cave)	*Agelenopsis aperta* (Gertsch), *Tayshaneta paraconcinna* (Cokendolpher & Reddell)
Trail of Tears Cave	*Eidmannella pallida* (Emerton)
Tres Amigos Cave	*Gaucelmus augustinus* Keyserling
Turner Goat Cave	*Eidmannella pallida* (Emerton)
Twin Springs Cave (=Whitney West Cave)	*Tayshaneta paraconcinna* (Cokendolpher & Reddell)
Two Hole Cave	*Agyneta llanoensis* (Gertsch & Davis), *Cryptachaea porteri* (Banks)
Underline Cave	*Cicurina buwata* Chamberlin & Ivie, *Agyneta llanoensis* (Gertsch & Davis)
Valley Cave	*Agyneta serrata* (Emerton)
Vault Cave	*Agyneta llanoensis* (Gertsch & Davis)
Velcro Cave	*Agyneta llanoensis* (Gertsch & Davis)
Venom Cave	*Agyneta llanoensis* (Gertsch & Davis)
Venturi Cave	*Mermessus albulus* (Zorsch & Crosby)
Village Idiot Cave	*Tayshaneta devia* (Gertsch), *Agyneta llanoensis* (Gertsch & Davis)
Walsh Ranch Cave	*Cicurina varians* Gertsch & Mulaik, *Cryptachaea porteri* (Banks)
War Party Cave	*Eidmannella pallida* (Emerton)
Water Tank Cave	*Agyneta llanoensis* (Gertsch & Davis)
Water Tower Cave	*Agyneta llanoensis* (Gertsch & Davis)
Waterfall Canyon Cave	*Agyneta llanoensis* (Gertsch & Davis)
Whiskey Jug Cave	*Gaucelmus augustinus* Keyserling
White Wall Cave	*Agyneta llanoensis* (Gertsch & Davis)
Wild Card Cave	*Agyneta llanoensis* (Gertsch & Davis)
Williams Cave	*Neoscona domiciliorum* (Hentz), *Cicurina varians* Gertsch & Mulaik, *Eidmannella pallida* (Emerton), *Cryptachaea porteri* (Banks)
Williams Cave No. 1	*Eidmannella pallida* (Emerton)
Wolf Cave	*Cicurina varians* Gertsch & Mulaik, *Cryptachaea porteri* (Banks)
Wolf’s Rattlesnake Cave	*Cicurina varians* Gertsch & Mulaik
Zapata Cave	*Agyneta llanoensis* (Gertsch & Davis)

### List of spiders in caves

Dipluridae

*Euagrus chisoseus* Gertsch, 1939

Euctenizidae

*Eucteniza relata* (O.P.-Cambridge, 1895)

Agelenidae

*Agelenopsis aleenae* Chamberlin & Ivie, 1935

*Agelenopsis aperta* (Gertsch, 1934)

*Tegenaria domestica* (Clerck, 1757)

*Tegenaria pagana* C. L. Koch, 1840)

Amphinectidae

*Metaltella simoni* (Keyserling, 1878)

Anyphaenidae

*Wulfila tantillus* Chickering, 1940

Araneidae

*Araneus gemma* (McCook, 1888)

*Araneus illaudatus* (Gertsch & Mulaik, 1936)

*Argiope aurantia* Lucas, 1833

*Hypsosinga funebris* (Keyserling, 1892)

*Metepeira labyrinthea* (Hentz, 1847)

*Neoscona domiciliorum* (Hentz, 1847)

Caponiidae

*Orthonops lapanus* Gertsch & Mulaik, 1940

Corinnidae

*Falconina gracilis* (Keyserling, 1891)

Ctenidae

*Ctenus valverdiensis* Peck, 1981

*Leptoctenus byrrhus* Simon, 1888

Dictynidae

*Cicurina bandera* Gertsch, 1992

*Cicurina bandida* Gertsch, 1992

*Cicurina baronia* Gertsch, 1992

*Cicurina barri* Gertsch, 1992

*Cicurina browni* Gertsch, 1992

*Cicurina brunsi* Cokendolpher, 2004

*Cicurina bullis* Cokendolpher, 2004

*Cicurina buwata* Chamberlin & Ivie, 1940

*Cicurina caliga* Cokendolpher & Reddell, 2001

*Cicurina caverna* Gertsch, 1992

*Cicurina coryelli* Gertsch, 1992

*Cicurina delrio* Gertsch, 1992

*Cicurina ezelli* Gertsch, 1992

*Cicurina gruta* Gertsch, 1992

*Cicurina holsingeri* Gertsch, 1992

*Cicurina hoodensis* Cokendolpher & Reddell, 2001

*Cicurina joya* Gertsch, 1992

*Cicurina loftini* Cokendolpher, 2004

*Cicurina machete* Gertsch, 1992

*Cicurina madla* Gertsch, 1992

*Cicurina mckenziei* Gertsch, 1992

*Cicurina medina* Gertsch, 1992

*Cicurina menardia* Gertsch, 1992

*Cicurina mirifica* Gertsch, 1992

*Cicurina mixmaster* Cokendolpher & Reddell, 2001

*Cicurina neovespera* Cokendolpher, 2004

*Cicurina obscura* Gertsch, 1992

*Cicurina orellia* Gertsch, 1992

*Cicurina pablo* Gertsch, 1992

*Cicurina pampa* Chamberlin & Ivie, 1940

*Cicurina pastura* Gertsch, 1992

*Cicurina patei* Gertsch, 1992

*Cicurina platypus* Cokendolpher, 2004

*Cicurina porteri* Gertsch, 1992

*Cicurina puentecilla* Gertsch, 1992

*Cicurina rainesi* Gertsch, 1992

*Cicurina reclusa* Gertsch, 1992

*Cicurina russelli* Gertsch, 1992

*Cicurina sansaba* Gertsch, 1992

*Cicurina selecta* Gertsch, 1992

*Cicurina serena* Gertsch, 1992

*Cicurina sheari* Gertsch, 1992

*Cicurina sprousei* Gertsch, 1992

*Cicurina stowersi* Gertsch, 1992

*Cicurina suttoni* Gertsch, 1992

*Cicurina travisae* Gertsch, 1992

*Cicurina troglobia* Cokendolpher, 2004

*Cicurina ubicki* Gertsch, 1992

*Cicurina uvalde* Gertsch, 1992

*Cicurina varians* Gertsch & Mulaik, 1940

*Cicurina venefica* Gertsch, 1992

*Cicurina venii* Gertsch, 1992

*Cicurina vespera* Gertsch, 1992

*Cicurina vibora* Gertsch, 1992

*Cicurina watersi* Gertsch, 1992

*Dictyna bellans* Chamberlin, 1919

Filistatidae

*Filistatinella crassipalpis* (Gertsch, 1935)

*Kukulcania arizonica* (Chamberlin & Ivie, 1935)

Gnaphosidae

*Drassyllus aprilinus* (Banks, 1904)

*Drassyllus dromeus* Chamberlin, 1922

*Drassyllus gynosaphes* Chamberlin, 1936

*Drassyllus prosaphes* Chamberlin, 1936

*Drassyllus texamans* Chamberlin, 1936

*Gnaphosa fontinalis* Keyserling, 1887

*Scotophaeus blackwalli* (Thorell, 1871)

*Urozelotes rusticus* (L. Koch, 1872)

*Zelotes pseustes* Chamberlin, 1922

Hahniidae

*Hahnia flaviceps* Emerton, 1913

*Neoantistea mulaiki* Gertsch, 1946

Leptonetidae

*Tayshaneta anopica* (Gertsch, 1974)

*Tayshaneta archambaulti* Ledford et al., 2012

*Tayshaneta bullis* (Cokendolpher, 2004)

*Tayshaneta coeca* (Chamberlin & Ivie, 1942)

*Tayshaneta concinna* (Gertsch, 1974)

*Tayshaneta devia* (Gertsch, 1974)

*Tayshaneta emeraldae* Ledford et al., 2012

*Tayshaneta fawcetti* Ledford et al., 2012

*Tayshaneta grubbsi* Ledford et al., 2012

*Tayshaneta madla* Ledford et al., 2012

*Tayshaneta microps* (Gertsch, 1974)

*Tayshaneta myopica* (Gertsch, 1974)

*Tayshaneta oconnorae* Ledford et al., 2012

*Tayshaneta paraconcinna* (Cokendolpher & Reddell, 2001)

*Tayshaneta sandersi* Ledford et al., 2012

*Tayshaneta sprousei* Ledford et al., 2012

*Tayshaneta valverdae* (Gertsch, 1974)

*Tayshaneta vidrio* Ledford et al., 2012

*Tayshaneta whitei* Ledford et al., 2012

Linyphiidae

*Agyneta flax* Dupérré, 2013

*Agyneta llanoensis* (Gertsch & Davis, 1936)

*Agyneta micaria* (Emerton 1882)

*Agyneta sandia* Dupérré, 2013

*Agyneta serrata* (Emerton 1909)

*Agyneta spicula* Dupérré, 2013

*Erigone autumnalis* Emerton, 1882

*Eulaira suspecta* Gertsch & Mulaik, 1936

*Frontinella communis* (Hentz, 1850)

*Islandiana unicornis* Ivie, 1965

*Jalapyphantes puebla* Gertsch & Davis, 1946

*Masoncus conspectus* (Gertsch & Davis, 1936)

*Mermessus albulus* (Zorsch & Crosby, 1934)

*Mermessus antraeus* (Crosby, 1926)

*Mermessus maculatus* (Banks, 1892)

*Neriene radiata* (Walckenaer, 1841)

*Scylaceus* sp.

*Tenuiphantes sabulosus* (Keyserling, 1886)

Liocranidae

*Neoanagraphis chamberlini* Gertsch & Mulaik, 1936

Lycosidae

*Camptocosa parallela* (Banks, 1898)

*Camptocosa texana* Dondale, Jiménez & Nieto, 2005

*Hogna antelucana* (Montgomery, 1904)

*Hogna carolinensis* (Walckenaer, 1805)

*Pirata davisi* Wallace & Exline, 1978

*Pirata sedentarius* Montgomery, 1904

*Rabidosa punctulata* (Hentz, 1844)

*Rabidosa rabida* (Walckenaer, 1837)

*Schizocosa saltatrix* (Hentz, 1844)

Miturgidae

*Teminius affinis* Banks, 1897

Mysmenidae

*Mysmena incredula* (Gertsch & Davis, 1936)

Nesticidae

*Eidmannella bullata* Gertsch, 1984

*Eidmannella delicata* Gertsch, 1984

*Eidmannella nasuta* Gertsch, 1984

*Eidmannella pallida* (Emerton, 1875)

*Eidmannella reclusa* Gertsch, 1984

*Eidmannella rostrata* Gertsch, 1984

*Eidmannella tuckeri* Cokendolpher & Reddell, 2001

*Gaucelmus augustinus* Keyserling, 1884

Pholcidae

*Modisimus texanus* Banks, 1906

*Physocyclus enaulus* Crosby, 1926

*Psilochorus imitatus* Gertsch & Mulaik, 1940

Pisauridae

*Dolomedes scriptus* Hentz, 1845

Salticidae

*Naphrys acerba* (Peckham & Peckham, 1909)

*Phidippus texanus* Banks, 1906

Scytodidae

*Scytodes atlacoya* Rheims, Brescovit & Durán, 2007

Segestriidae

*Ariadna bicolor* (Hentz, 1842)

Sicariidae

*Loxosceles blanda* Gertsch & Ennik, 1983

*Loxosceles devia* Gertsch & Mulaik, 1940

*Loxosceles reclusa* Gertsch & Mulaik, 1940

Symphytognathidae

*Anapistula secreta* Gertsch, 1941

Tetragnathidae

*Leucauge venusta* (Walckenaer, 1841)

*Metellina mimetoides* Chamberlin & Ivie, 1941

*Tetragnatha elongata* Walckenaer, 1841

Theridiidae

*Cryptachaea canionis* (Chamberlin & Gertsch, 1929)

*Cryptachaea porteri* (Banks, 1896)

*Dipoena abdita* Gertsch & Mulaik, 1936

*Latrodectus hesperus* Chamberlin & Ivie, 1935

*Latrodectus mactans* (Fabricius, 1775)

*Neospintharus furcatus* (O. P.-Cambridge, 1894)

*Parasteatoda tepidariorum* (C. L. Koch, 1841)

*Theridion llano* Levi, 1957

*Tidarren sisyphoides* (Walckenaer, 1841)

Thomisidae

*Mecaphesa celer* (Hentz, 1847)

*Mecaphesa dubia* (Keyserling, 1880)

*Misumena vatia* (Clerck, 1757)

*Xysticus ferox* (Hentz, 1847)

*Xysticus funestus* Keyserling, 1880

*Xysticus robinsoni* Gertsch, 1953

Trachelidae

*Trachelas volutus* Gertsch, 1935

Uloboridae

*Hyptiotes cavatus* (Hentz, 1847)

Zoropsidae

*Zorocrates aemulus* Gertsch, 1935

### Spiders in Parks

#### National Forests

**Angelina National Forest, Angelina County (24 spp.)**

Anyphaenidae

*Hibana gracilis* (Hentz, 1847)

Gnaphosidae

*Cesonia bilineata* (Hentz, 1847)

*Drassyllus aprilinus* (Banks, 1904)

*Drassyllus dixinus* Chamberlin, 1922

*Drassyllus dromeus* Chamberlin, 1922

*Drassyllus gynosaphes* Chamberlin, 1936

*Drassyllus prosaphes* Chamberlin, 1936

*Gnaphosa fontinalis* Keyserling, 1887

*Litopyllus temporarius* Chamberlin, 1922

*Sergiolus ocellatus* (Walckenaer, 1837)

*Synaphosus paludis* (Chamberlin & Gertsch, 1940)

*Talanites exlineae* (Platnick & Shadab, 1976)

*Zelotes duplex* Chamberlin, 1922

*Zelotes hentzi* Barrows, 1945

*Zelotes lymnophilus* Chamberlin, 1936

Hahniidae

*Neoantistea oklahomensis* Opell & Beatty, 1976

Miturgidae

*Zora pumila* (Hentz, 1850)

Oxyopidae

*Oxyopes cougar* Brady, 1969

Salticidae

*Anasaitis canosa* (Walckenaer, 1837)

*Ghelna sexmaculata* (Banks, 1895)

*Marpissa lineata* (C. L. Koch, 1846)

*Sarinda hentzi* (Banks, 1913)

Thomisidae

*Xysticus ferox* (Hentz, 1847)

*Xysticus fraternus* Banks, 1895

**Davy Crockett National Forest, Angelina County (2 spp.)**

Salticidae

*Peckhamia americana* (Peckham & Peckham, 1892)

*Synemosyna formica* Hentz, 1846

**Sabine National Forest, Sabine County (1 sp.)**

Salticidae

*Anasaitis canosa* (Walckenaer, 1837)

**Sam Houston National Forest, Walker County (19 spp.)**

Anyphaenidae

*Wulfila saltabundus* (Hentz, 1847)

Araneidae

*Mangora placida* (Hentz, 1847)

Linyphiidae

*Erigone autumnalis* Emerton, 1882

*Frontinella communis* (Hentz, 1850)

*Neriene radiata* (Walckenaer, 1841)

Salticidae

*Anasaitis canosa* (Walckenaer, 1837)

*Colonus puerperus* (Hentz, 1846)

*Eris militaris* (Hentz, 1845)

*Lyssomanes viridis* (Walckenaer, 1837)

*Pelegrina galathea* (Walckenaer, 1837)

*Zygoballus rufipes* Peckham & Peckham, 1885

Tetragnathidae

*Leucauge venusta* (Walckenaer, 1841)

Theridiidae

*Faiditus cancellatus* (Hentz, 1850)

*Hentziectypus globosus* (Hentz, 1850)

*Neospintharus trigonum* (Hentz, 1850)

*Theridion flavonotatum* Becker, 1879

*Thymoites unimaculatus* (Emerton, 1882)

Thomisidae

*Mecaphesa celer* (Hentz, 1847)

Trachelidae

*Trachelas similis* F. O. P.-Cambridge, 1899

#### National Wildlife Refuges

**Aransas National Wildlife Refuge, Aransas County (1 sp.)**

Scytodidae

*Scytodes atlacoya* Rheims, Brescovit & Durán, 2007

**Attwater Prairie Chicken National Wildlife Refuge, Colorado County (102 spp.)**

Amphinectidae

*Metaltella simoni* (Keyserling, 1878)

Anyphaenidae

*Hibana gracilis* (Hentz, 1847)

*Hibana velox* (Becker, 1879)

Araneidae

*Acanthepeira cherokee* Levi, 1976

*Acanthepeira stellata* (Walckenaer, 1805)

*Argiope aurantia* Lucas, 1833

*Eustala anastera* (Walckenaer, 1841)

*Eustala cepina* (Walckenaer, 1841)

*Gea heptagon* (Hentz, 1850)

*Kaira hiteae* Levi, 1977

*Larinia directa* (Hentz, 1847)

*Neoscona arabesca* (Walckenaer, 1841)

Clubionidae

*Clubiona abboti* L. Koch, 1866

*Clubiona catawba* Gertsch, 1941

*Clubiona kiowa* Gertsch, 1941

Corinnidae

*Castianeira crocata* (Hentz, 1847)

*Castianeira longipalpa* (Hentz, 1847)

*Falconina gracilis* (Keyserling, 1891)

Eutichuridae

*Cheiracanthium inclusum* (Hentz, 1847)

Gnaphosidae

*Camillina pulchra* (Keyserling, 1891)

*Cesonia bilineata* (Hentz, 1847)

*Cesonia sincera* Gertsch & Mulaik, 1936

*Drassyllus creolus* Chamberlin & Gertsch, 1940

*Drassyllus texamans* Chamberlin, 1936

*Gnaphosa sericata* (L. Koch, 1866)

*Micaria deserticola* Gertsch, 1933

*Micaria gertschi* Barrows & Ivie, 1942

*Micaria longipes* Emerton, 1890

*Micaria nanella* Gertsch, 1935

*Micaria vinnula* Gertsch & Davis, 1936

*Sergiolus capulatus* (Walckenaer, 1837)

*Zelotes hentzi* Barrows, 1945

*Zelotes laccus* (Barrows, 1919)

*Zelotes lasalanus* Chamberlin, 1928

Hahniidae

*Neoantistea mulaiki* Gertsch, 1946

Linyphiidae

*Agyneta chiricahua* Dupérré, 2013

*Agyneta regina* (Chamberlin & Ivie, 1944)

*Agyneta serrata* (Emerton, 1909)

*Ceraticelus similis* (Banks, 1892)

*Ceratinops latus* (Emerton, 1882)

*Ceratinopsis laticeps* Emerton, 1882

*Erigone autumnalis* Emerton, 1882

*Grammonota texana* (Banks, 1899)

*Mermessus bryantae* (Ivie & Barrows, 1935)

*Mermessus denticulatus* (Banks, 1898)

*Mermessus trilobatus* (Emerton, 1882)

*Tennesseellum formicum* (Emerton, 1882)

*Tutaibo anglicanus* (Hentz, 1850)

*Walckenaeria spiralis* (Emerton, 1882)

Lycosidae

*Hogna antelucana* (Montgomery, 1904)

*Pardosa delicatula* Gertsch & Wallace, 1935

*Pardosa milvina* (Hentz, 1844)

*Pardosa pauxilla* Montgomery, 1904

*Pardosa saxatilis* (Hentz, 1844)

*Pirata hiteorum* Wallace & Exline, 1978

*Pirata sedentarius* Montgomery, 1904

*Pirata seminolus* Gertsch & Wallace, 1935

*Pirata suwaneus* Gertsch, 1940

*Rabidosa rabida* (Walckenaer, 1837)

*Schizocosa avida* (Walckenaer, 1837)

*Schizocosa bilineata* (Emerton, 1885)

*Trochosa sepulchralis* (Montgomery, 1902)

Mimetidae

*Mimetus hesperus* Chamberlin, 1923

Miturgidae

*Teminius affinis* Banks, 1897

Mysmenidae

*Mysmena incredula* (Gertsch & Davis, 1936)

Nesticidae

*Eidmannella pallida* (Emerton, 1875)

Oxyopidae

*Oxyopes apollo* Brady, 1964

*Oxyopes salticus* Hentz, 1845

*Peucetia viridans* (Hentz, 1832)

Philodromidae

*Philodromus pratariae* (Scheffer, 1904)

*Thanatus formicinus* (Clerck, 1757)

*Thanatus rubicellus* Mello-Leitão, 1929

*Tibellus duttoni* (Hentz, 1847)

Phrurolithidae

*Phrurotimpus certus* Gertsch, 1941

Salticidae

*Cheliferoides longimanus* Gertsch, 1936

*Colonus puerperus* (Hentz, 1846)

*Habronattus coecatus* (Hentz, 1846)

*Habronattus viridipes* (Hentz, 1846)

*Marpissa lineata* (C. L. Koch, 1846)

*Marpissa pikei* (Peckham & Peckham, 1888)

*Neonella vinnula* Gertsch, 1936

*Pelegrina galathea* (Walckenaer, 1837)

*Sarinda hentzi* (Banks, 1913)

*Sassacus cyaneus* (Hentz, 1846)

*Zygoballus nervosus* (Peckham & Peckham, 1888)

*Zygoballus rufipes* Peckham & Peckham, 1885

*Zygoballus sexpunctatus* (Hentz, 1845)

Tetragnathidae

*Glenognatha foxi* (McCook, 1894)

*Pachygnatha autumnalis* Marx, 1884

Theridiidae

*Dipoena abdita* Gertsch & Mulaik, 1936

*Dipoena nigra* (Emerton, 1882)

*Steatoda transversa* (Banks, 1898)

*Theridion australe* Banks, 1899

*Theridion rabuni* Chamberlin & Ivie, 1944

*Thymoites expulsus* (Gertsch & Mulaik, 1936)

Thomisidae

*Mecaphesa celer* (Hentz, 1847)

*Mecaphesa dubia* (Keyserling, 1880)

*Misumenoides formosipes* (Walckenaer, 1837)

*Xysticus apachecus* Gertsch, 1933

*Xysticus auctificus* Keyserling, 1880

*Xysticus funestus* Keyserling, 1880

Titanoecidae

*Titanoeca americana* Emerton, 1888

**Brazoria National Wildlife Refuge, Brazoria County (1 sp.)**

Philodromidae

*Philodromus pratariae* (Scheffer, 1904)

**Hagerman National Wildlife Refuge, Grayson County (2 spp.)**

Mimetidae

*Mimetus hesperus* Chamberlin, 1923

Salticidae

*Habronattus coecatus* (Hentz, 1846)

**Laguna Atascosa National Wildlife Refuge, Cameron County (22 spp.)**

Anyphaenidae

*Hibana futilis* (Banks, 1898)

Araneidae

*Acacesia hamata* (Hentz, 1847)

*Cyclosa walckenaeri* (O. P.-Cambridge, 1889)

*Ocrepeira georgia* (Levi, 1976)

Caponiidae

*Tarsonops systematicus* Chamberlin, 1924

Corinnidae

*Castianeira crocata* (Hentz, 1847)

*Castianeira cubana* (Banks, 1926)

Filistatidae

*Kukulcania arizonica* (Chamberlin & Ivie, 1935)

Gnaphosidae

*Drassyllus dromeus* Chamberlin, 1922

*Eilica bicolor* Banks, 1896

Nesticidae

*Eidmannella pallida* (Emerton, 1875)

Salticidae

*Cheliferoides segmentatus* F. O. P.-Cambridge, 1901

*Leptofreya ambigua* (C. L. Koch, 1846)

*Messua limbata* (Banks, 1898)

*Metacyrba taeniola similis* Banks, 1904

*Metacyrba taeniola taeniola* (Hentz, 1846)

*Paramaevia poultoni* (Peckham & Peckham, 1901)

*Parnaenus* sp.

*Peckhamia americana* (Peckham & Peckham, 1892)

*Pelegrina galathea* (Walckenaer, 1837)

*Platycryptus undatus* (De Geer, 1778)

Thomisidae

*Bucranium* sp.

**Lower Rio Grande Valley National Wildlife Refuge, Cameron/Hidalgo Counties (21 spp.)**

Araneidae

*Gasteracantha cancriformis* (Linnaeus, 1758)

*Wagneriana tauricornis* (O. P.-Cambridge, 1889)

Ctenidae

*Leptoctenus byrrhus* Simon, 1888

Eutichuridae

*Cheiracanthium inclusum* (Hentz, 1847)

Gnaphosidae

*Cesonia bilineata* (Hentz, 1847)

*Drassyllus dromeus* Chamberlin, 1922

*Eilica bicolor* Banks, 1896

Oxyopidae

*Oxyopes acleistus* Chamberlin, 1929

Pholcidae

*Modisimus texanus* Banks, 1906

Salticidae

*Bagheera prosper* (Peckham & Peckham, 1901)

*Cheliferoides segmentatus* F. O. P.-Cambridge, 1901

*Habronattus moratus* (Gertsch & Mulaik, 1936)

*Leptofreya ambigua* (C. L. Koch, 1846)

*Marpissa obtusa* Barnes, 1958

*Messua limbata* (Banks, 1898)

*Metacyrba taeniola taeniola* (Hentz, 1846)

*Naphrys acerba* (Peckham & Peckham, 1909)

*Parnaenus* sp.

Segestriidae

*Ariadna bicolor* (Hentz, 1842)

Theridiidae

*Neopisinus cognatus* O. P.-Cambridge, 1893

Thomisidae

*Bucranium* sp.

**Muleshoe National Wildlife Refuge, Bailey County (1 sp.)**

Theridiidae

*Latrodectus mactans* (Fabricius, 1775)

**Santa Ana National Wildlife Refuge, Hidalgo County (53 spp.)**

Agelenidae

*Agelenopsis naevia* (Walckenaer, 1841)

Anyphaenidae

*Wulfila bryantae* Platnick, 1974

Araneidae

*Acacesia hamata* (Hentz, 1847)

*Araneus pegnia* (Walckenaer, 1841)

*Eriophora ravilla* (C. L. Koch, 1844)

*Larinia directa* (Hentz, 1847)

*Mastophora alvareztoroi* Ibarra & Jiménez, 2003

*Mastophora cornigera* (Hentz, 1850)

*Mastophora leucabulba* (Gertsch, 1955)

*Mecynogea lemniscata* (Walckenaer, 1841)

*Metepeira minima* Gertsch, 1936

*Neoscona arabesca* (Walckenaer, 1841)

*Scoloderus nigriceps* (O. P.-Cambridge, 1895)

Filistatidae

*Kukulcania arizonica* (Chamberlin & Ivie, 1935)

*Kukulcania hibernalis* (Hentz, 1842)

Gnaphosidae

*Callilepis imbecilla* (Keyserling, 1887)

*Drassyllus texamans* Chamberlin, 1936

*Eilica bicolor* Banks, 1896

*Herpyllus ecclesiasticus* Hentz, 1832

*Zelotes pseustes* Chamberlin, 1922

Linyphiidae

*Grammonota texana* (Banks, 1899)

Lycosidae

*Schizocosa saltatrix* (Hentz, 1844)

Mimetidae

*Mimetus haynesi* Gertsch & Mulaik, 1940

Oxyopidae

*Hamataliwa grisea* Keyserling, 1887

*Oxyopes acleistus* Chamberlin, 1929

*Oxyopes apollo* Brady, 1964

Philodromidae

*Apollophanes punctipes* (O. P.-Cambridge, 1891)

Salticidae

*Cheliferoides longimanus* Gertsch, 1936

*Colonus puerperus* (Hentz, 1846)

*Colonus sylvanus* (Hentz, 1846)

*Habronattus fallax* (Peckham & Peckham, 1909)

*Leptofreya ambigua* (C. L. Koch, 1846)

*Metaphidippus felix* (Peckham & Peckham, 1901)

*Pelegrina galathea* (Walckenaer, 1837)

*Phidippus arizonensis* (Peckham & Peckham, 1883)

*Phidippus audax* (Hentz, 1845)

*Zygoballus rufipes* Peckham & Peckham, 1885

Scytodidae

*Scytodes atlacoya* Rheims, Brescovit & Durán, 2007

*Scytodes lugubris* (Thorell, 1887)

Tetragnathidae

*Leucauge venusta* (Walckenaer, 1841)

*Tetragnatha laboriosa* Hentz, 1850

Theridiidae

*Cryptachaea insulsa* (Gertsch & Mulaik, 1936)

*Euryopis lineatipes* O. P.-Cambridge, 1893

*Euryopis spinigera* O. P.-Cambridge, 1895

*Neospintharus furcatus* (O. P.-Cambridge, 1894)

*Stemmops bicolor* O. P.-Cambridge, 1894

*Thymoites missionensis* (Levi, 1957)

Thomisidae

*Bucranium* sp.

*Mecaphesa celer* (Hentz, 1847)

*Mecaphesa dubia* (Keyserling, 1880)

*Xysticus funestus* Keyserling, 1880

Uloboridae

*Philoponella oweni* (Chamberlin, 1924)

*Philoponella semiplumosa* (Simon, 1893)

#### National (other areas)

**Amistad National Recreational Area, Val Verde County (1 sp.)**

Agelenidae

*Agelenopsis aperta* (Gertsch, 1934)

**Big Bend National Park, Brewster County (69 spp.)**

Dipluridae

*Euagrus chisoseus* Gertsch, 1939

Euctenizidae

*Entychides arizonicus* Gertsch & Wallace, 1936

Theraphosidae

*Aphonopelma echinum* (Chamberlin, 1940)

*Aphonopelma steindachneri* (Ausserer, 1875)

Anyphaenidae

*Hibana incursa* (Chamberlin, 1919)

Araneidae

*Cyclosa berlandi* Levi, 1999

*Mangora fascialata* Franganillo, 1936

*Metepeira arizonica* Chamberlin & Ivie, 1942

Caponiidae

*Orthonops lapanus* Gertsch & Mulaik, 1940

Clubionidae

*Elaver chisosa* (Roddy, 1966)

Corinnidae

*Septentrinna bicalcarata* (Simon, 1896)

Ctenidae

*Leptoctenus byrrhus* Simon, 1888

Dictynidae

*Lathys delicatula* (Gertsch & Mulaik, 1936)

Diguetidae

*Diguetia albolineata* (O. P.-Cambridge, 1895)

*Diguetia canities* (McCook, 1889)

*Diguetia imperiosa* Gertsch & Mulaik, 1940

Gnaphosidae

*Callilepis chisos* Platnick, 1975

*Cesonia sincera* Gertsch & Mulaik, 1936

*Drassyllus antonito* Platnick & Shadab, 1982

*Drassyllus aprilinus* (Banks, 1904)

*Drassyllus notonus* Chamberlin, 1928

*Herpyllus bubulcus* Chamberlin, 1922

*Herpyllus cockerelli* (Banks, 1901)

*Herpyllus gertschi* Platnick & Shadab, 1977

*Micaria deserticola* Gertsch, 1933

*Micaria langtry* Platnick & Shadab, 1988

*Micaria nye* Platnick & Shadab, 1988

*Scopoides cambridgei* (Gertsch & Davis, 1940)

*Synaphosus syntheticus* (Chamberlin, 1924)

*Trachyzelotes lyonneti* (Audouin, 1826)

Hahniidae

*Hahnia arizonica* Chamberlin & Ivie, 1942

Leptonetidae

*Chisoneta chisosea* (Gertsch, 1974)

Lycosidae

*Camptocosa parallela* (Banks, 1898)

*Hogna carolinensis* (Walckenaer, 1805)

*Pardosa vadosa* Barnes, 1959

*Varacosa gosiuta* (Chamberlin, 1908)

Mimetidae

*Mimetus hesperus* Chamberlin, 1923

Oecobiidae

*Oecobius putus* O. P.-Cambridge, 1876

Oxyopidae

*Oxyopes apollo* Brady, 1964

*Oxyopes tridens* Brady, 1964

Philodromidae

*Apollophanes punctipes* (O. P.-Cambridge, 1891)

*Apollophanes texanus* Banks, 1904

*Ebo evansae* Sauer & Platnick, 1972

*Titanebo mexicanus* (Banks, 1898)

Pholcidae

*Chisosa diluta* (Gertsch & Mulaik, 1940)

*Physocyclus enaulus* Crosby, 1926

*Psilochorus concolor* Slowik, 2009

*Psilochorus pallidulus* Gertsch, 1935

Plectreuridae

*Plectreurys tristis* Simon, 1893

Salticidae

*Habronattus forticulus* (Gertsch & Mulaik, 1936)

*Habronattus hirsutus* (Peckham & Peckham, 1888)

*Habronattus sugillatus* Griswold, 1987

*Marpissa dentoides* Barnes, 1958

*Marpissa obtusa* Barnes, 1958

*Metacyrba taeniola similis* Banks, 1904

*Metacyrba taeniola taeniola* (Hentz, 1846)

*Neon nelli* Peckham & Peckham, 1888

*Sassacus vitis* (Cockerell, 1894)

Selenopidae

*Selenops actophilus* Chamberlin, 1924

Sicariidae

*Loxosceles blanda* Gertsch & Ennik, 1983

Theridiidae

*Steatoda alamosa* Gertsch, 1960

*Steatoda mexicana* Levi, 1957

*Theridion submissum* Gertsch & Davis, 1936

Thomisidae

*Mecaphesa coloradensis* (Gertsch, 1933)

*Mecaphesa dubia* (Keyserling, 1880)

Trachelidae

*Trachelas mexicanus* Banks, 1898

Uloboridae

*Hyptiotes puebla* Muma & Gertsch, 1964

*Uloborus glomosus* (Walckenaer, 1841)

Zoropsidae

*Zorocrates unicolor* (Banks, 1901)

**Big Thicket National Preserve, Tyler County (11 spp.)**

Dictynidae

*Lathys delicatula* (Gertsch & Mulaik, 1936)

Gnaphosidae

*Drassyllus aprilinus* (Banks, 1904)

*Drassyllus covensis* Exline, 1962

*Talanites exlineae* (Platnick & Shadab, 1976)

Hahniidae

*Hahnia flaviceps* Emerton, 1913

Linyphiidae

*Walckenaeria spiralis* (Emerton, 1882)

Lycosidae

*Varacosa avara* (Keyserling, 1877)

Nephilidae

*Nephila clavipes* (Linnaeus, 1767)

Salticidae

*Ghelna sexmaculata* (Banks, 1895)

Theridiidae

*Crustulina altera* Gertsch & Archer, 1942

Thomisidae

*Xysticus fraternus* Banks, 1895

**Guadalupe Mountains National Park, Culberson County (5 spp.)**

Dipluridae

*Euagrus chisoseus* Gertsch, 1939

Dictynidae

*Mallos blandus* Chamberlin & Gertsch, 1958

Lycosidae

*Pardosa xerophila* Vogel, 1964

*Pirata sedentarius* Montgomery, 1904

Thomisidae

*Misumenoides formosipes* (Walckenaer, 1837)

**Lake Meredith National Recreation Area, Hutchinson, Moore, Potter Counties (7 spp.)**

Gnaphosidae

*Drassyllus lepidus* (Banks, 1899)

*Gnaphosa fontinalis* Keyserling, 1887

*Haplodrassus signifer* (C. L. Koch, 1839)

*Herpyllus bubulcus* Chamberlin, 1922

*Zelotes gertschi* Platnick & Shadab, 1983

Salticidae

*Phidippus audax* (Hentz, 1845)

*Phlegra hentzi* (Marx, 1890)

**Padre Island National Seashore, Kenedy County (2 spp.)**

Gnaphosidae

*Sergiolus lowelli* Chamberlin & Woodbury, 1929

Salticidae

*Habronattus coecatus* (Hentz, 1846)

#### State Forests

**Jones State Forest, Montgomery County (15 spp.)**

Araneidae

*Hypsosinga rubens* (Hentz, 1847)

*Mangora placida* (Hentz, 1847)

*Mangora spiculata* (Hentz, 1847)

Dictynidae

*Emblyna sublata* (Hentz, 1850)

Linyphiidae

*Frontinella communis* (Hentz, 1850)

*Neriene radiata* (Walckenaer, 1841)

Oxyopidae

*Oxyopes salticus* Hentz, 1845

Philodromidae

*Philodromus placidus* Banks, 1892

Salticidae

*Hentzia palmarum* (Hentz, 1832)

*Synageles bishopi* Cutler, 1988

*Zygoballus rufipes* Peckham & Peckham, 1885

Theridiidae

*Hentziectypus globosus* (Hentz, 1850)

*Neospintharus furcatus* (O. P.-Cambridge, 1894)

*Theridion flavonotatum* Becker, 1879

*Yunohamella lyrica* (Walckenaer, 1841)

**Kirby State Forest, Tyler County (29 spp.)**

Agelenidae

*Agelenopsis kastoni* Chamberlin & Ivie, 1941

Araneidae

*Verrucosa arenata* (Walckenaer, 1841)

Ctenidae

*Anahita punctulata* (Hentz, 1844)

Gnaphosidae

*Cesonia bilineata* (Hentz, 1847)

*Drassyllus aprilinus* (Banks, 1904)

*Drassyllus dixinus* Chamberlin, 1922

*Drassyllus dromeus* Chamberlin, 1922

*Drassyllus ellipes* Chamberlin & Gertsch, 1940

*Drassyllus eremitus* Chamberlin, 1922

*Litopyllus temporarius* Chamberlin, 1922

*Sergiolus bicolor* Banks, 1900

*Sergiolus capulatus* (Walckenaer, 1837)

*Sergiolus cyaneiventris* Simon, 1893

*Talanites exlineae* (Platnick & Shadab, 1976)

*Zelotes duplex* Chamberlin, 1922

*Zelotes hentzi* Barrows, 1945

Hahniidae

*Neoantistea oklahomensis* Opell & Beatty, 1976

Lycosidae

*Varacosa avara* (Keyserling, 1877)

Oxyopidae

*Hamataliwa helia* (Chamberlin, 1929)

*Oxyopes aglossus* Chamberlin, 1929

Salticidae

*Anasaitis canosa* (Walckenaer, 1837)

*Chalcoscirtus diminutus* (Banks, 1896)

*Colonus sylvanus* (Hentz, 1846)

*Lyssomanes viridis* (Walckenaer, 1837)

*Peckhamia americana* (Peckham & Peckham, 1892)

*Platycryptus undatus* (De Geer, 1778)

*Sarinda hentzi* (Banks, 1913)

*Zygoballus sexpunctatus* (Hentz, 1845)

Uloboridae

*Uloborus glomosus* (Walckenaer, 1841)

#### State Parks

**Bastrop State Park, Bastrop County (21 spp.)**

Dipluridae

*Euagrus chisoseus* Gertsch, 1939

Euctenizidae

*Eucteniza relata* (O. P.-Cambridge, 1895)

Anyphaenidae

*Hibana cambridgei* (Bryant, 1931)

*Lupettiana mordax* (O. P.-Cambridge, 1896)

Araneidae

*Mangora gibberosa* (Hentz, 1847)

*Mangora placida* (Hentz, 1847)

Gnaphosidae

*Cesonia bilineata* (Hentz, 1847)

*Herpyllus ecclesiasticus* Hentz, 1832

Linyphiidae

*Agyneta flax* Dupérré, 2013

*Agyneta sandia* Dupérré, 2013

*Erigone autumnalis* Emerton, 1882

*Neriene radiata* (Walckenaer, 1841)

Lycosidae

*Tigrosa georgicola* (Walckenaer, 1837)

Oxyopidae

*Oxyopes salticus* Hentz, 1845

Salticidae

*Anasaitis canosa* (Walckenaer, 1837)

*Lyssomanes viridis* (Walckenaer, 1837)

*Platycryptus undatus* (De Geer, 1778)

Segestriidae

*Ariadna bicolor* (Hentz, 1842)

Theridiidae

*Wamba crispulus* (Simon, 1895)

Trachelidae

*Meriola decepta* Banks, 1895

*Trachelas volutus* Gertsch, 1935

**Bentsen-Rio Grande Valley State Park, Hidalgo County (79 spp.)**

Anyphaenidae

*Hibana arunda* (Platnick, 1974)

*Hibana futilis* (Banks, 1898)

*Hibana gracilis* (Hentz, 1847)

*Wulfila tantillus* Chickering, 1940

Araneidae

*Araneus detrimentosus* (O. P.-Cambridge, 1889)

*Araneus pegnia* (Walckenaer, 1841)

*Eriophora edax* (Blackwall, 1863)

*Larinia directa* (Hentz, 1847)

*Mecynogea lemniscata* (Walckenaer, 1841)

*Metazygia zilloides* (Banks, 1898)

*Micrathena sagittata* (Walckenaer, 1841)

*Neoscona arabesca* (Walckenaer, 1841)

*Neoscona utahana* (Chamberlin, 1919)

*Ocrepeira georgia* (Levi, 1976)

Corinnidae

*Castianeira longipalpa* (Hentz, 1847)

Ctenidae

*Leptoctenus byrrhus* Simon, 1888

Dictynidae

*Dictyna bellans* Chamberlin, 1919

*Dictyna volucripes* Keyserling, 1881

Filistatidae

*Kukulcania hibernalis* (Hentz, 1842)

Gnaphosidae

*Callilepis chisos* Platnick, 1975

*Callilepis gertschi* Platnick, 1975

*Drassyllus inanus* Chamberlin & Gertsch, 1940

*Drassyllus prosaphes* Chamberlin, 1936

*Gnaphosa sericata* (L. Koch, 1866)

*Nodocion floridanus* (Banks, 1896)

*Trachyzelotes lyonneti* (Audouin, 1826)

*Zelotes pseustes* Chamberlin, 1922

Hersiliidae

*Neotama mexicana* (O. P.-Cambridge, 1893)

Lycosidae

*Allocosa absoluta* (Gertsch, 1934)

*Arctosa littoralis* (Hentz, 1844)

*Pardosa delicatula* Gertsch & Wallace, 1935

*Sosippus texanus* Brady, 1962

*Varacosa shenandoa* (Chamberlin & Ivie, 1942)

Mimetidae

*Mimetus hesperus* Chamberlin, 1923

*Mimetus notius* Chamberlin, 1923

Miturgidae

*Teminius affinis* Banks, 1897

Oxyopidae

*Hamataliwa grisea* Keyserling, 1887

*Oxyopes acleistus* Chamberlin, 1929

Philodromidae

*Apollophanes punctipes* (O. P.-Cambridge, 1891)

Salticidae

*Bredana complicata* Gertsch, 1936)

*Colonus sylvanus* (Hentz, 1846)

*Habronattus dorotheae* (Gertsch & Mulaik, 1936)

*Habronattus texanus* (Chamberlin, 1924)

*Habronattus viridipes* (Hentz, 1846)

*Hentzia palmarum* (Hentz, 1832)

*Leptofreya ambigua* (C. L. Koch, 1846)

*Messua limbata* (Banks, 1898)

*Metacyrba taeniola taeniola* (Hentz, 1846)

*Metaphidippus chera* (Chamberlin, 1924)

*Naphrys acerba* (Peckham & Peckham, 1909)

*Naphrys pulex* (Hentz, 1846)

*Pelegrina pervaga* (Peckham & Peckham, 1909)

*Pellenes longimanus* Emerton, 1913

*Phidippus arizonensis* (Peckham & Peckham, 1883)

*Phidippus audax* (Hentz, 1845)

*Platycryptus undatus* (De Geer, 1778)

*Sitticus dorsatus* (Banks, 1895)

*Zygoballus nervosus* (Peckham & Peckham, 1888)

*Zygoballus rufipes* Peckham & Peckham, 1885

Scytodidae

*Scytodes atlacoya* Rheims, Brescovit & Durán, 2007

*Scytodes lugubris* (Thorell, 1887)

Sicariidae

*Loxosceles devia* Gertsch & Mulaik, 1940

Theridiidae

*Euryopis spinigera* O. P.-Cambridge, 1895

*Latrodectus mactans* (Fabricius, 1775)

*Steatoda quadrimaculata* (O. P.-Cambridge, 1896)

*Theridion cynicum* Gertsch & Mulaik, 1936

*Thymoites expulsus* (Gertsch & Mulaik, 1936)

*Thymoites illudens* (Gertsch & Mulaik, 1936)

Thomisidae

*Mecaphesa asperata* (Hentz, 1847)

*Mecaphesa californica* (Banks, 1896)

*Mecaphesa celer* (Hentz, 1847)

*Mecaphesa dubia* (Keyserling, 1880)

*Synema viridans* (Banks, 1896)

*Xysticus funestus* Keyserling, 1880

Trachelidae

*Trachelas mexicanus* Banks, 1898

Uloboridae

*Philoponella oweni* (Chamberlin, 1924)

*Philoponella semiplumosa* (Simon, 1893)

*Uloborus glomosus* (Walckenaer, 1841)

*Uloborus segregatus* Gertsch, 1936

**Big Bend Ranch State Park, Presidio County (34 spp.)**

Dipluridae

*Euagrus chisoseus* Gertsch, 1939

Anyphaenidae

*Hibana incursa* (Chamberlin, 1919)

Araneidae

*Eustala anastera* (Walckenaer, 1841)

*Neoscona crucifera* (Lucas, 1838)

Caponiidae

*Orthonops lapanus* Gertsch & Mulaik, 1940

Dictynidae

*Mallos pallidus* (Banks, 1904)

Diguetidae

*Diguetia canities* (McCook, 1889)

*Diguetia imperiosa* Gertsch & Mulaik, 1940

Gnaphosidae

*Gnaphosa saxosa* Platnick & Shadab, 1975

*Herpyllus propinquus* (Keyserling, 1887)

*Scopoides cambridgei* (Gertsch & Davis, 1940)

*Zelotes anglo* Gertsch & Riechert, 1976

Linyphiidae

*Frontinella communis* (Hentz, 1850)

Lycosidae

*Hogna tigana* (Gertsch & Wallace, 1935)

*Pardosa falcifera* F. O. P.-Cambridge, 1902

Oxyopidae

*Peucetia viridans* (Hentz, 1832)

Philodromidae

*Apollophanes texanus* Banks, 1904

*Philodromus californicus* Keyserling, 1884

Pholcidae

*Physocyclus enaulus* Crosby, 1926

Plectreuridae

*Plectreurys* sp.

Salticidae

*Habronattus conjunctus* (Banks, 1898)

*Hentzia alamosa* Richman, 2010

*Hentzia fimbriata* (F. O. P.-Cambridge, 1901

*Phidippus arizonensis* (Peckham & Peckham, 1883)

*Phidippus carneus* Peckham & Peckham, 1896

*Salticus peckhamae* (Cockerell, 1897)

Scytodidae

*Scytodes zapatana* Gertsch & Mulaik, 1940

Sicariidae

*Loxosceles blanda* Gertsch & Ennik, 1983

Tetragnathidae

*Tetragnatha laboriosa* Hentz, 1850

Theridiidae

*Latrodectus hesperus* Chamberlin & Ivie, 1935

*Steatoda variata* Gertsch, 1960

Thomisidae

*Mecaphesa coloradensis* (Gertsch, 1933)

*Misumenoides formosipes* (Walckenaer, 1837)

Uloboridae

*Uloborus glomosus* (Walckenaer, 1841)

**Brazos Bend State Park, Fort Bend County (8 spp.)**

Corinnidae

*Falconina gracilis* (Keyserling, 1891)

Gnaphosidae

*Cesonia bilineata* (Hentz, 1847)

*Sergiolus capulatus* (Walckenaer, 1837)

Lycosidae

*Schizocosa rovneri* Uetz & Dondale, 1979

Nephilidae

*Nephila clavipes* (Linnaeus, 1767)

Salticidae

*Anasaitis canosa* (Walckenaer, 1837)

*Zygoballus rufipes* Peckham & Peckham, 1885

Thomisidae

*Xysticus ferox* (Hentz, 1847)

**Buescher State Park, Bastrop County (6 spp.)**

Araneidae

*Micrathena gracilis* (Walckenaer, 1805)

*Verrucosa arenata* (Walckenaer, 1841)

Eutichuridae

*Cheiracanthium inclusum* (Hentz, 1847)

Linyphiidae

*Frontinella communis* (Hentz, 1850)

Sicariidae

*Loxosceles reclusa* Gertsch & Mulaik, 1940

Tetragnathidae

*Leucauge venusta* (Walckenaer, 1841)

**Caddo Lake State Park, Harrison County (2 spp.)**

Lycosidae

*Tigrosa georgicola* (Walckenaer, 1837)

*Trochosa sepulchralis* (Montgomery, 1902)

**Caprock Canyons State Park, Briscoe County (2 spp.)**

Agelenidae

*Agelenopsis aleenae* Chamberlin & Ivie, 1935

*Agelenopsis spatula* Chamberlin & Ivie, 1935

**Corpus Christi State Park, San Patricio County (1 sp.)**

Lycosidae

*Hogna tigana* (Gertsch & Wallace, 1935)

**Estero Llano Grande State Park, Hidalgo County (13 spp.)**

Salticidae

*Colonus sylvanus* (Hentz, 1846)

*Habronattus fallax* (Peckham & Peckham, 1909)

*Hentzia palmarum* (Hentz, 1832)

*Leptofreya ambigua* (C. L. Koch, 1846)

*Menemerus bivittatus* (Dufour, 1831)

*Messua limbata* (Banks, 1898)

*Naphrys acerba* (Peckham & Peckham, 1909)

*Paramaevia poultoni* (Peckham & Peckham, 1901)

*Phidippus arizonensis* (Peckham & Peckham, 1883)

*Phidippus audax* (Hentz, 1845)

*Plexippus paykulli* (Audouin, 1826)

*Sassacus vitis* (Cockerell, 1894)

*Zygoballus sexpunctatus* (Hentz, 1845)

**Falcon State Park, Starr/Zapata Counties (14 spp.)**

Theraphosidae

*Aphonopelma anax* (Chamberlin, 1940)

Araneidae

*Araneus detrimentosus* (O. P.-Cambridge, 1889)

Gnaphosidae

*Callilepis gertschi* Platnick, 1975

*Cesonia bilineata* (Hentz, 1847)

*Gnaphosa sericata* (L. Koch, 1866)

Oxyopidae

*Oxyopes salticus* Hentz, 1845

Philodromidae

*Tibellus duttoni* (Hentz, 1847)

Salticidae

*Habronattus mataxus* Griswold, 1987

*Metacyrba taeniola taeniola* (Hentz, 1846)

*Pelegrina galathea* (Walckenaer, 1837)

*Poultonella alboimmaculata* (Peckham & Peckham, 1883)

Scytodidae

*Scytodes atlacoya* Rheims, Brescovit & Durán, 2007

Theridiidae

*Chrosiothes jocosus* (Gertsch & Davis, 1936)

Thomisidae

*Mecaphesa dubia* (Keyserling, 1880)

**Fort Parker State Park, Limestone County (3 spp.)**

Mimetidae

*Mimetus puritanus* Chamberlin, 1923

*Mimetus syllepsicus* Hentz, 1832

Miturgidae

*Teminius affinis* Banks, 1897

**Frio State Park, Frio County (1 sp.)**

Salticidae

*Cheliferoides segmentatus* F. O. P.-Cambridge, 1901

**Galveston Island State Park, Galveston County (20 spp.)**

Araneidae

*Acanthepeira stellata* (Walckenaer, 1805)

*Araniella displicata* (Hentz, 1847)

*Eustala anastera* (Walckenaer, 1841)

*Larinioides cornutus* (Clerck, 1757)

*Neoscona arabesca* (Walckenaer, 1841)

*Verrucosa arenata* (Walckenaer, 1841)

Gnaphosidae

*Sergiolus ocellatus* (Walckenaer, 1837)

Oxyopidae

*Oxyopes apollo* Brady, 1964

*Oxyopes salticus* Hentz, 1845

Salticidae

*Phidippus audax* (Hentz, 1845)

*Plexippus paykulli* (Audouin, 1826)

*Zygoballus rufipes* Peckham & Peckham, 1885

Tetragnathidae

*Tetragnatha guatemalensis* O. P.-Cambridge, 1889

*Tetragnatha laboriosa* Hentz, 1850

*Tetragnatha pallescens* F. O. P.-Cambridge, 1903

Theridiidae

*Cryptachaea porteri* (Banks, 1896)

Thomisidae

*Mecaphesa asperata* (Hentz, 1847)

*Mecaphesa celer* (Hentz, 1847)

*Misumenoides formosipes* (Walckenaer, 1837)

*Misumessus oblongus* (Keyserling, 1880)

**Garner State Park, Uvalde County (30 spp.)**

Araneidae

*Hypsosinga funebris* (Keyserling, 1892)

*Kaira alba* (Hentz, 1850)

*Mangora fascialata* Franganillo, 1936

*Mangora gibberosa* (Hentz, 1847)

Mimetidae

*Mimetus notius* Chamberlin, 1923

Oxyopidae

*Oxyopes apollo* Brady, 1964

*Oxyopes salticus* Hentz, 1845

Salticidae

*Colonus puerperus* (Hentz, 1846)

*Eris militaris* (Hentz, 1845)

*Hentzia mitrata* (Hentz, 1846)

*Marpissa pikei* (Peckham & Peckham, 1888)

*Pelegrina galathea* (Walckenaer, 1837)

*Phidippus pius* Scheffer, 1905

*Sarinda hentzi* (Banks, 1913)

*Zygoballus rufipes* Peckham & Peckham, 1885

Theridiidae

*Chrosiothes jocosus* (Gertsch & Davis, 1936)

*Euryopis quinquemaculata* Banks, 1900

*Hentziectypus globosus* (Hentz, 1850)

*Theridion dilutum* Levi, 1957

*Theridion dividuum* Gertsch & Archer, 1942

*Theridion hidalgo* Levi, 1957

*Theridion murarium* Emerton, 1882

*Theridion positivum* Chamberlin, 1924

*Thymoites expulsus* (Gertsch & Mulaik, 1936)

*Wamba crispulus* (Simon, 1895)

Thomisidae

*Mecaphesa californica* (Banks, 1896)

*Mecaphesa celer* (Hentz, 1847)

*Mecaphesa dubia* (Keyserling, 1880)

*Synema viridans* (Banks, 1896)

Uloboridae

*Uloborus glomosus* (Walckenaer, 1841)

**Goliad State Park, Goliad County (9 spp.)**

Araneidae

*Cyclosa turbinata* (Walckenaer, 1841)

*Metazygia wittfeldae* (McCook, 1894)

Corinnidae

*Castianeira longipalpa* (Hentz, 1847)

*Falconina gracilis* (Keyserling, 1891)

Oxyopidae

*Oxyopes salticus* Hentz, 1845

Salticidae

*Habronattus coecatus* (Hentz, 1846)

Thomisidae

*Mecaphesa dubia* (Keyserling, 1880)

*Xysticus ferox* (Hentz, 1847)

Trachelidae

*Trachelas mexicanus* Banks, 1898

**Goose Island State Park, Aransas County (9 spp.)**

Araneidae

*Allocyclosa bifurca* (McCook, 1887)

*Hypsosinga rubens* (Hentz, 1847)

*Micrathena gracilis* (Walckenaer, 1805)

Gnaphosidae

*Callilepis imbecilla* (Keyserling, 1887)

Lycosidae

*Sosippus texanus* Brady, 1962

Oxyopidae

*Hamataliwa grisea* Keyserling, 1887

*Oxyopes acleistus* Chamberlin, 1929

Salticidae

*Colonus sylvanus* (Hentz, 1846)

Theridiidae

*Anelosimus studiosus* (Hentz, 1850)

**Huntsville State Park, Walker County (6 spp.)**

Araneidae

*Mastophora phrynosoma* Gertsch, 1955

Clubionidae

*Elaver excepta* (L. Koch, 1866)

Gnaphosidae

*Drassyllus covensis* Exline, 1962

Mimetidae

*Mimetus notius* Chamberlin, 1923

Phrurolithidae

*Phrurotimpus alarius* (Hentz, 1847)

Thomisidae

*Xysticus fraternus* Banks, 1895

**Inks Lake State Park, Burnet County (9 spp.)**

Dipluridae

*Euagrus chisoseus* Gertsch, 1939

Araneidae

*Larinioides cornutus* (Clerck, 1757)

Philodromidae

*Philodromus keyserlingi* Marx, 1890

*Tibellus duttoni* (Hentz, 1847)

Salticidae

*Marpissa pikei* (Peckham & Peckham, 1888)

Theridiidae

*Latrodectus mactans* (Fabricius, 1775)

*Theridion glaucescens* Becker, 1879

*Theridion murarium* Emerton, 1882

Thomisidae

*Mecaphesa asperata* (Hentz, 1847)

**Lake Arrowhead State Park, Clay County (2 spp.)**

Agelenidae

*Agelenopsis aleenae* Chamberlin & Ivie, 1935

*Agelenopsis oklahoma* (Gertsch, 1936)

**Lake Corpus Christi State Park, San Patricio County (19 spp.)**

Anyphaenidae

*Anyphaena lacka* Platnick, 1974

Araneidae

*Acacesia hamata* (Hentz, 1847)

*Metazygia wittfeldae* (McCook, 1894)

*Metazygia zilloides* (Banks, 1898)

*Neoscona arabesca* (Walckenaer, 1841)

Filistatidae

*Kukulcania hibernalis* (Hentz, 1842)

Gnaphosidae

*Nodocion floridanus* (Banks, 1896)

Mimetidae

*Ero canionis* Chamberlin & Ivie, 1935

*Mimetus notius* Chamberlin, 1923

Oxyopidae

*Oxyopes acleistus* Chamberlin, 1929

Salticidae

*Phidippus audax* (Hentz, 1845)

*Platycryptus undatus* (De Geer, 1778)

Tetragnathidae

*Tetragnatha guatemalensis* O. P.-Cambridge, 1889

Theridiidae

*Euryopis lineatipes* O. P.-Cambridge, 1893

*Euryopis texana* Banks, 1908

*Parasteatoda tepidariorum* (C. L. Koch, 1841)

*Steatoda triangulosa* (Walckenaer, 1802)

Trachelidae

*Meriola decepta* Banks, 1895

Uloboridae

*Uloborus glomosus* (Walckenaer, 1841)

**Lake Somerville State Park, Lee County (9 spp.)**

Araneidae

*Araneus detrimentosus* (O. P.-Cambridge, 1889)

*Larinioides cornutus* (Clerck, 1757)

*Metazygia wittfeldae* (McCook, 1894)

*Metazygia zilloides* (Banks, 1898)

Gnaphosidae

*Cesonia bilineata* (Hentz, 1847)

Salticidae

*Eris militaris* (Hentz, 1845)

*Phidippus audax* (Hentz, 1845)

Tetragnathidae

*Tetragnatha guatemalensis* O. P.-Cambridge, 1889

Theridiidae

*Tidarren haemorrhoidale* (Bertkau, 1880)

**Lake Tawakoni State Park, Hunt County (26 spp.)**

Agelenidae

*Agelenopsis emertoni* Chamberlin & Ivie, 1935

*Barronopsis texana* (Gertsch, 1934)

Araneidae

*Argiope aurantia* Lucas, 1833

*Eustala emertoni* (Banks, 1904)

*Larinioides cornutus* (Clerck, 1757)

*Mecynogea lemniscata* (Walckenaer, 1841)

*Metazygia wittfeldae* (McCook, 1894)

*Neoscona arabesca* (Walckenaer, 1841)

*Neoscona crucifera* (Lucas, 1838)

Clubionidae

*Elaver excepta* (L. Koch, 1866)

Dictynidae

*Emblyna sublata* (Hentz, 1850)

*Phantyna segregata* (Gertsch & Mulaik, 1936)

Mimetidae

*Mimetus syllepsicus* Hentz, 1832

Salticidae

*Bagheera prosper* (Peckham & Peckham, 1901)

*Colonus puerperus* (Hentz, 1846)

*Colonus sylvanus* (Hentz, 1846)

*Eris militaris* (Hentz, 1845)

*Peckhamia americana* (Peckham & Peckham, 1892)

*Pelegrina galathea* (Walckenaer, 1837)

*Phidippus audax* (Hentz, 1845)

Tetragnathidae

*Leucauge venusta* (Walckenaer, 1841)

*Tetragnatha guatemalensis* O. P.-Cambridge, 1889

Theridiidae

*Argyrodes elevatus* Taczanowski, 1873

*Theridion glaucescens* Becker, 1879

*Tidarren haemorrhoidale* (Bertkau, 1880)

Uloboridae

*Uloborus glomosus* (Walckenaer, 1841)

**Lockhart State Park, Caldwell County (4 spp.)**

Linyphiidae

*Agyneta micaria* (Emerton, 1882)

Oonopidae

*Noonops furtivus* (Gertsch, 1936)

Salticidae

*Attidops cutleri* Edwards, 1999

*Colonus sylvanus* (Hentz, 1846)

**Lost Maples State Park, Bandera County (18 spp.)**

Araneidae

*Cyclosa turbinata* (Walckenaer, 1841)

*Ocrepeira georgia* (Levi, 1976)

Ctenidae

*Leptoctenus byrrhus* Simon, 1888

Dictynidae

*Cicurina varians* Gertsch & Mulaik, 1940

*Dictyna formidolosa* Gertsch & Ivie, 1936

Gnaphosidae

*Drassyllus aprilinus* (Banks, 1904)

*Drassyllus texamans* Chamberlin, 1936

Philodromidae

*Philodromus keyserlingi* Marx, 1890

Phrurolithidae

*Phrurotimpus alarius* (Hentz, 1847)

Salticidae

*Maevia inclemens* (Walckenaer, 1837)

*Naphrys acerba* (Peckham & Peckham, 1909)

*Naphrys pulex* (Hentz, 1846)

*Peckhamia americana* (Peckham & Peckham, 1892)

*Pelegrina flavipes* (Peckham & Peckham, 1888)

Theridiidae

*Theridion murarium* Emerton, 1882

Thomisidae

*Ozyptila monroensis* Keyserling, 1884

*Tmarus rubromaculatus* Keyserling, 1880

*Xysticus ferox* (Hentz, 1847)

**Monahans Sandhills State Park, Ward County (2 spp.)**

Lycosidae

*Hogna coloradensis* (Banks, 1894)

Thomisidae

*Misumenoides formosipes* (Walckenaer, 1837)

**Palmetto State Park, Gonzales County (35 spp.)**

Anyphaenidae

*Anyphaena pectorosa* L. Koch, 1866

*Wulfila albens* (Hentz, 1847)

Araneidae

*Araneus bicentenarius* (McCook, 1888)

*Araneus marmoreus* Clerck, 1757

*Argiope aurantia* Lucas, 1833

*Gasteracantha cancriformis* (Linnaeus, 1758)

*Mangora gibberosa* (Hentz, 1847)

*Mangora maculata* (Keyserling, 1865)

*Mangora placida* (Hentz, 1847)

*Mecynogea lemniscata* (Walckenaer, 1841)

*Micrathena gracilis* (Walckenaer, 1805)

*Micrathena sagittata* (Walckenaer, 1841)

*Verrucosa arenata* (Walckenaer, 1841)

Eutichuridae

*Cheiracanthium inclusum* (Hentz, 1847)

Filistatidae

*Kukulcania hibernalis* (Hentz, 1842)

Gnaphosidae

*Drassyllus aprilinus* (Banks, 1904)

*Drassyllus gynosaphes* Chamberlin, 1936

*Synaphosus paludis* (Chamberlin & Gertsch, 1940)

Hahniidae

*Hahnia flaviceps* Emerton, 1913

Linyphiidae

*Mermessus denticulatus* (Banks, 1898)

Lycosidae

*Pirata seminolus* Gertsch & Wallace, 1935

*Tigrosa georgicola* (Walckenaer, 1837)

Oxyopidae

*Oxyopes salticus* Hentz, 1845

Phrurolithidae

*Phrurotimpus alarius* (Hentz, 1847)

Pisauridae

*Dolomedes tenebrosus* Hentz, 1844

*Dolomedes triton* (Walckenaer, 1837)

Salticidae

*Anasaitis canosa* (Walckenaer, 1837)

*Colonus sylvanus* (Hentz, 1846)

*Naphrys pulex* (Hentz, 1846)

*Pelegrina sabinema* Maddison, 1996

*Phidippus audax* (Hentz, 1845)

Theridiidae

*Hentziectypus globosus* (Hentz, 1850)

Thomisidae

*Mecaphesa celer* (Hentz, 1847)

*Ozyptila americana* Banks, 1895

Trachelidae

*Meriola decepta* Banks, 1895

**Palo Duro Canyon State Park, Randall County (9 spp.)**

Agelenidae

*Agelenopsis aperta* (Gertsch, 1934)

Araneidae

*Neoscona oaxacensis* (Keyserling, 1864)

Gnaphosidae

*Drassyllus lepidus* (Banks, 1899)

*Drassyllus texamans* Chamberlin, 1936

*Zelotes gertschi* Platnick & Shadab, 1983

Lycosidae

*Arctosa littoralis* (Hentz, 1844)

*Hogna antelucana* (Montgomery, 1904)

Philodromidae

*Titanebo mexicanus* (Banks, 1898)

Salticidae

*Phlegra hentzi* (Marx, 1890)

**Pedernales Falls State Park, Blanco County (2 spp.)**

Dipluridae

*Euagrus chisoseus* Gertsch, 1939

Leptonetidae

*Tayshaneta paraconcinna* (Cokendolpher & Reddell, 2001)

**Resaca de la Palma State Park, Cameron County (20 spp.)**

Anyphaenidae

*Hibana arunda* (Platnick, 1974)

Araneidae

*Araneus miniatus* (Walckenaer, 1841)

*Araneus pegnia* (Walckenaer, 1841)

*Eriophora ravilla* (C. L. Koch, 1844)

*Micrathena sagittata* (Walckenaer, 1841)

*Neoscona arabesca* (Walckenaer, 1841)

Gnaphosidae

*Eilica bicolor* Banks, 1896

Salticidae

*Colonus sylvanus* (Hentz, 1846)

*Habronattus fallax* (Peckham & Peckham, 1909)

*Hentzia palmarum* (Hentz, 1832)

*Leptofreya ambigua* (C. L. Koch, 1846)

*Menemerus bivittatus* (Dufour, 1831)

*Messua limbata* (Banks, 1898)

*Metacyrba punctata* (Peckham & Peckham, 1894)

*Paramaevia poultoni* (Peckham & Peckham, 1901)

*Phidippus arizonensis* (Peckham & Peckham, 1883)

*Phidippus audax* (Hentz, 1845)

*Phidippus texanus* Banks, 1906

*Platycryptus undatus* (De Geer, 1778)

*Plexippus paykulli* (Audouin, 1826)

**Sam Houston State Park, Walker County (2 spp.)**

Anyphaenidae

*Hibana gracilis* (Hentz, 1847)

Araneidae

*Acanthepeira stellata* (Walckenaer, 1805)

**Seminole Canyon State Park, Val Verde County (20 spp.)**

Araneidae

*Colphepeira catawba* (Banks, 1911)

*Hypsosinga funebris* (Keyserling, 1892)

*Metepeira comanche* Levi, 1977

Dictynidae

*Cicurina holsingeri* Gertsch, 1992

Diguetidae

*Diguetia albolineata* (O. P.-Cambridge, 1895)

Oxyopidae

*Oxyopes apollo* Brady, 1964

*Oxyopes lynx* Brady, 1964

*Oxyopes salticus* Hentz, 1845

Philodromidae

*Tibellus duttoni* (Hentz, 1847)

Salticidae

*Marpissa pikei* (Peckham & Peckham, 1888)

*Pelegrina galathea* (Walckenaer, 1837)

*Sassacus papenhoei* Peckham & Peckham, 1895

Selenopidae

*Selenops actophilus* Chamberlin, 1924

Theridiidae

*Euryopis texana* Banks, 1908

*Theridion dilutum* Levi, 1957

*Theridion hidalgo* Levi, 1957

*Theridion llano* Levi, 1957

*Thymoites expulsus* (Gertsch & Mulaik, 1936)

Thomisidae

*Mecaphesa celer* (Hentz, 1847)

*Mecaphesa dubia* (Keyserling, 1880)

**Tyler State Park, Smith County (8 spp.)**

Dictynidae

*Lathys delicatula* (Gertsch & Mulaik, 1936)

Gnaphosidae

*Callilepis chisos* Platnick, 1975

*Gnaphosa fontinalis* Keyserling, 1887

*Talanites exlineae* (Platnick & Shadab, 1976)

Lycosidae

*Gladicosa pulchra* (Keyserling, 1877)

Philodromidae

*Philodromus pratariae* (Scheffer, 1904)

Phrurolithidae

*Phrurotimpus alarius* (Hentz, 1847)

Thomisidae

*Xysticus fraternus* Banks, 1895

#### Wildlife Management Areas

**Black Gap Wildlife Management Area, Brewster County (8 spp.)**

Diguetidae

*Diguetia canities* (McCook, 1889)

Gnaphosidae

*Callilepis imbecilla* (Keyserling, 1887)

Lycosidae

*Allocosa retenta* (Gertsch & Wallace, 1935)

Salticidae

*Habronattus coecatus* (Hentz, 1846)

*Metacyrba taeniola taeniola* (Hentz, 1846)

*Pelegrina arizonensis* (Peckham & Peckham, 1901)

Theridiidae

*Asagena fulva* (Keyserling, 1884)

*Euryopis texana* Banks, 1908

**Chaparral Wildlife Management Area, Dimmit County (5 spp.)**

Gnaphosidae

*Gnaphosa sericata* (L. Koch, 1866)

*Zelotes lasalanus* Chamberlin, 1928

*Zelotes pseustes* Chamberlin, 1922

Oxyopidae

*Oxyopes apollo* Brady, 1964

*Oxyopes tridens* Brady, 1964

**Engeling Wolf Management Area, Anderson County (1 sp.)**

Salticidae

*Metacyrba taeniola similis* Banks, 1904

**Matador Wildlife Management Area, Cottle County (3 spp.)**

Gnaphosidae

*Gnaphosa sericata* (L. Koch, 1866)

*Zelotes gertschi* Platnick & Shadab, 1983

Salticidae

*Phidippus texanus* Banks, 1906

#### Other

**Dalquest Research Site, Presidio County (48 spp.)**

Agelenidae

*Agelenopsis naevia* (Walckenaer, 1841)

Anyphaenidae

*Anyphaena rita* Platnick, 1974

Caponiidae

*Orthonops lapanus* Gertsch & Mulaik, 1940

Corinnidae

*Castianeira amoena* (C. L. Koch, 1841)

*Castianeira nanella* Gertsch, 1933

*Castianeira occidens* Reiskind, 1969

Ctenidae

*Leptoctenus byrrhus* Simon, 1888

Dictynidae

*Cicurina varians* Gertsch & Mulaik, 1940

Filistatidae

*Kukulcania hibernalis* (Hentz, 1842)

Gnaphosidae

*Callilepis chisos* Platnick, 1975

*Callilepis gertschi* Platnick, 1975

*Cesonia sincera* Gertsch & Mulaik, 1936

*Drassyllus broussardi* Platnick & Horner, 2007

*Drassyllus prosaphes* Chamberlin, 1936

*Gnaphosa fontinalis* Keyserling, 1887

*Haplodrassus chamberlini* Platnick & Shadab, 1975

*Herpyllus bubulcus* Chamberlin, 1922

*Micaria emertoni* Gertsch, 1935

*Micaria imperiosa* Gertsch, 1935

*Micaria langtry* Platnick & Shadab, 1988

*Micaria longipes* Emerton, 1890

*Micaria nye* Platnick & Shadab, 1988

*Scopoides cambridgei* (Gertsch & Davis, 1940)

*Sergiolus stella* Chamberlin, 1922

*Zelotes lasalanus* Chamberlin, 1928

Liocranidae

*Neoanagraphis chamberlini* Gertsch & Mulaik, 1936

Lycosidae

*Alopecosa aculeata* (Clerck, 1757)

*Hogna carolinensis* (Walckenaer, 1805)

*Varacosa gosiuta* (Chamberlin, 1908)

*Varacosa parthenus* (Chamberlin, 1925)

Oxyopidae

*Oxyopes apollo* Brady, 1964

*Oxyopes felinus* Brady, 1964

*Oxyopes panther* Brady, 1975

*Oxyopes tridens* Brady, 1964

Philodromidae

*Apollophanes texanus* Banks, 1904

*Titanebo parabolis* (Schick, 1965)

Phrurolithidae

*Phrurotimpus certus* Gertsch, 1941

*Scotinella pugnata* (Emerton, 1890)

Salticidae

*Habronattus conjunctus* (Banks, 1898)

*Habronattus hirsutus* (Peckham & Peckham, 1888)

*Pellenes limatus* Peckham & Peckham, 1901

*Salticus peckhamae* (Cockerell, 1897)

*Sitticus dorsatus* (Banks, 1895)

Sparassidae

*Heteropoda venatoria* (Linnaeus, 1767)

Theridiidae

*Euryopis texana* Banks, 1908

*Steatoda variata* Gertsch, 1960

Thomisidae

*Mecaphesa celer* (Hentz, 1847)

*Xysticus lassanus* Chamberlin, 1925

**Fort Sill Recreation Area, Palo Pinto County (1 sp.)**

Salticidae

*Habronattus mataxus* Griswold, 1987

**Frontera Audubon, Hidalgo County (28 spp.)**

Anyphaenidae

*Hibana arunda* (Platnick, 1974)

*Hibana futilis* (Banks, 1898)

*Wulfila bryantae* Platnick, 1974

Araneidae

*Eriophora edax* (Blackwall, 1863)

*Eriophora ravilla* (C. L. Koch, 1844)

*Eustala anastera* (Walckenaer, 1841)

*Kaira altiventer* O. P.-Cambridge, 1889

*Mastophora cornigera* (Hentz, 1850)

*Metazygia zilloides* (Banks, 1898)

*Micrathena sagittata* (Walckenaer, 1841)

*Neoscona domiciliorum* (Hentz, 1847)

Clubionidae

*Elaver texana* (Gertsch, 1933)

Dictynidae

*Phantyna segregata* (Gertsch & Mulaik, 1936)

Philodromidae

*Apollophanes punctipes* (O. P.-Cambridge, 1891)

Salticidae

*Colonus puerperus* (Hentz, 1846)

*Colonus sylvanus* (Hentz, 1846)

*Hentzia palmarum* (Hentz, 1832)

*Messua limbata* (Banks, 1898)

*Pelegrina galathea* (Walckenaer, 1837)

Tetragnathidae

*Tetragnatha guatemalensis* O. P.-Cambridge, 1889

Theridiidae

*Anelosimus studiosus* (Hentz, 1850)

*Emertonella taczanowskii* (Keyserling, 1886)

*Theridion dilutum* Levi, 1957

*Theridion positivum* Chamberlin, 1924

*Theridion rabuni* Chamberlin & Ivie, 1944

*Wamba crispulus* (Simon, 1895)

Trachelidae

*Trachelas mexicanus* Banks, 1898

Uloboridae

*Uloborus glomosus* (Walckenaer, 1841)

**Green Island Bird Refuge, Cameron County (9 spp.)**

Gnaphosidae

*Callilepis gertschi* Platnick, 1975

*Gnaphosa clara* (Keyserling, 1887)

*Micaria nanella* Gertsch, 1935

*Micaria triangulosa* Gertsch, 1935

*Trachyzelotes lyonneti* (Audouin, 1826)

Hahniidae

*Neoantistea mulaiki* Gertsch, 1946

Oecobiidae

*Oecobius navus* Blackwall, 1859

Pholcidae

*Psilochorus redemptus* Gertsch & Mulaik, 1940

Sicariidae

*Loxosceles devia* Gertsch & Mulaik, 1940

**Stubblefield Lake Recreation Area, Walker County (2 spp.)**

Araneidae

*Neoscona crucifera* (Lucas, 1838)

*Verrucosa arenata* (Walckenaer, 1841)

**Wildcat Bluff Nature Center, Potter County (50 spp.)**

Theraphosidae

*Aphonopelma hentzi* (Girard, 1852)

Araneidae

*Acanthepeira stellata* (Walckenaer, 1805)

*Argiope aurantia* Lucas, 1833

*Argiope trifasciata* (Forskål, 1775)

*Larinioides cornutus* (Clerck, 1757)

*Metepeira labyrinthea* (Hentz, 1847)

*Neoscona crucifera* (Lucas, 1838)

*Neoscona oaxacensis* (Keyserling, 1864)

Corinnidae

*Castianeira crocata* (Hentz, 1847)

Dictynidae

*Cicurina varians* Gertsch & Mulaik, 1940

Gnaphosidae

*Drassodes saccatus* (Emerton, 1890)

*Drassyllus lepidus* (Banks, 1899)

*Gnaphosa fontinalis* Keyserling, 1887

*Gnaphosa sericata* (L. Koch, 1866)

*Haplodrassus signifer* (C. L. Koch, 1839)

*Herpyllus ecclesiasticus* Hentz, 1832

*Zelotes gertschi* Platnick & Shadab, 1983

Lycosidae

*Alopecosa kochi* (Keyserling, 1877)

*Hesperocosa unica* (Gertsch & Wallace, 1935)

*Hogna antelucana* (Montgomery, 1904)

*Hogna carolinensis* (Walckenaer, 1805)

*Pardosa falcifera* F. O. P.-Cambridge, 1902

*Pardosa mercurialis* Montgomery, 1904

*Rabidosa rabida* (Walckenaer, 1837)

*Schizocosa mccooki* (Montgomery, 1904)

*Varacosa gosiuta* (Chamberlin, 1908)

Nesticidae

*Eidmannella pallida* (Emerton, 1875)

Oxyopidae

*Oxyopes apollo* Brady, 1964

*Oxyopes salticus* Hentz, 1845

Philodromidae

*Philodromus vulgaris* (Hentz, 1847)

*Thanatus rubicellus* Mello-Leitão, 1929

*Tibellus duttoni* (Hentz, 1847)

Salticidae

*Marpissa pikei* (Peckham & Peckham, 1888)

*Phidippus apacheanus* Chamberlin & Gertsch, 1929

*Phidippus audax* (Hentz, 1845)

*Phidippus carolinensis* Peckham & Peckham, 1909

*Phidippus texanus* Banks, 1906

*Salticus scenicus* (Clerck, 1757)

*Sassacus papenhoei* Peckham & Peckham, 1895

Sicariidae

*Loxosceles reclusa* Gertsch & Mulaik, 1940

Tetragnathidae

*Tetragnatha laboriosa* Hentz, 1850

Theridiidae

*Latrodectus hesperus* Chamberlin & Ivie, 1935

*Steatoda triangulosa* (Walckenaer, 1802)

Thomisidae

*Mecaphesa celer* (Hentz, 1847)

*Modysticus modestus* (Scheffer, 1904)

*Xysticus auctificus* Keyserling, 1880

*Xysticus coloradensis* Bryant, 1930

*Xysticus ferox* (Hentz, 1847)

*Xysticus texanus* Banks, 1904

Trachelidae

*Trachelas mexicanus* Banks, 1898

**Lick Creek Park, Brazos County (179 spp.)**

Euctenizidae

*Entychides arizonicus* Gertsch & Wallace, 1936

*Myrmekiaphila comstocki* Bishop & Crosby, 1926

Agelenidae

*Agelenopsis aperta* (Gertsch, 1934)

*Agelenopsis emertoni* Chamberlin & Ivie, 1935

*Agelenopsis naevia* (Walckenaer, 1841)

*Agelenopsis oklahoma* (Gertsch, 1936)

*Barronopsis texana* (Gertsch, 1934)

Amphinectidae

*Metaltella simoni* (Keyserling, 1878)

Anyphaenidae

*Anyphaena fraterna* (Banks, 1896)

*Anyphaena maculata* (Banks, 1896)

*Hibana futilis* (Banks, 1898)

*Lupettiana mordax* (O. P.-Cambridge, 1896)

*Wulfila albens* (Hentz, 1847)

Araneidae

*Acacesia hamata* (Hentz, 1847)

*Acanthepeira cherokee* Levi, 1976

*Araneus bicentenarius* (McCook, 1888)

*Araneus marmoreus* Clerck, 1757

*Araneus miniatus* (Walckenaer, 1841)

*Argiope aurantia* Lucas, 1833

*Argiope trifasciata* (Forskål, 1775)

*Eriophora ravilla* (C. L. Koch, 1844)

*Eustala anastera* (Walckenaer, 1841)

*Eustala emertoni* (Banks, 1904)

*Gasteracantha cancriformis* (Linnaeus, 1758)

*Gea heptagon* (Hentz, 1850)

*Hypsosinga rubens* (Hentz, 1847)

*Kaira alba* (Hentz, 1850)

*Larinia directa* (Hentz, 1847)

*Mangora gibberosa* (Hentz, 1847)

*Mangora maculata* (Keyserling, 1865)

*Mangora placida* (Hentz, 1847)

*Mastophora cornigera* (Hentz, 1850)

*Mecynogea lemniscata* (Walckenaer, 1841)

*Metepeira labyrinthea* (Hentz, 1847)

*Micrathena gracilis* (Walckenaer, 1805)

*Neoscona arabesca* (Walckenaer, 1841)

*Verrucosa arenata* (Walckenaer, 1841)

Clubionidae

*Clubiona abboti* L. Koch, 1866

*Clubiona catawba* Gertsch, 1941

*Elaver excepta* (L. Koch, 1866)

Corinnidae

*Castianeira amoena* (C. L. Koch, 1841)

*Castianeira trilineata* (Hentz, 1847)

*Falconina gracilis* (Keyserling, 1891)

Dictynidae

*Cicurina dorothea* Gertsch, 1992

*Emblyna sublata* (Hentz, 1850)

Eutichuridae

*Cheiracanthium inclusum* (Hentz, 1847)

Gnaphosidae

*Cesonia bilineata* (Hentz, 1847)

*Drassyllus aprilinus* (Banks, 1904)

*Drassyllus dixinus* Chamberlin, 1922

*Drassyllus orgilus* Chamberlin, 1922

*Drassyllus rufulus* (Banks, 1892)

*Gnaphosa sericata* (L. Koch, 1866)

*Litopyllus temporarius* Chamberlin, 1922

*Micaria vinnula* Gertsch & Davis, 1936

*Sergiolus capulatus* (Walckenaer, 1837)

*Synaphosus paludis* (Chamberlin & Gertsch, 1940)

*Talanites exlineae* (Platnick & Shadab, 1976)

*Zelotes aiken* Platnick & Shadab, 1983

*Zelotes duplex* Chamberlin, 1922

*Zelotes hentzi* Barrows, 1945

*Zelotes pseustes* Chamberlin, 1922

Hahniidae

*Hahnia flaviceps* Emerton, 1913

*Neoantistea agilis* (Keyserling, 1887)

*Neoantistea oklahomensis* Opell & Beatty, 1976

Linyphiidae

*Agyneta chiricahua* Dupérré, 2013

*Agyneta llanoensis* (Gertsch & Davis, 1936)

*Agyneta micaria* (Emerton, 1882)

*Agyneta parva* (Banks, 1896)

*Agyneta regina* (Chamberlin & Ivie, 1944)

*Agyneta serrata* (Emerton, 1909)

*Ceratinops crenatus* (Emerton, 1882)

*Erigone autumnalis* Emerton, 1882

*Frontinella communis* (Hentz, 1850)

*Mermessus maculatus* (Banks, 1892)

*Neriene radiata* (Walckenaer, 1841)

*Styloctetor purpurescens* (Keyserling, 1886)

*Tenuiphantes sabulosus* (Keyserling, 1886)

*Walckenaeria spiralis* (Emerton, 1882)

Lycosidae

*Allocosa noctuabunda* (Montgomery, 1904)

*Arctosa littoralis* (Hentz, 1844)

*Hogna antelucana* (Montgomery, 1904)

*Pardosa atlantica* Emerton, 1913

*Pardosa delicatula* Gertsch & Wallace, 1935

*Pardosa milvina* (Hentz, 1844)

*Pardosa pauxilla* Montgomery, 1904

*Pirata alachuus* Gertsch & Wallace, 1935

*Pirata apalacheus* Gertsch, 1940

*Pirata hiteorum* Wallace & Exline, 1978

*Pirata sedentarius* Montgomery, 1904

*Pirata spiniger* (Simon, 1898)

*Rabidosa punctulata* (Hentz, 1844)

*Rabidosa rabida* (Walckenaer, 1837)

*Schizocosa avida* (Walckenaer, 1837)

*Schizocosa crassipes* (Walckenaer, 1837)

*Schizocosa perplexa* Bryant, 1936

*Schizocosa rovneri* Uetz & Dondale, 1979

*Schizocosa saltatrix* (Hentz, 1844)

*Schizocosa stridulans* Stratton, 1984

*Schizocosa uetzi* Stratton, 1997

*Tigrosa georgicola* (Walckenaer, 1837)

*Trochosa sepulchralis* (Montgomery, 1902)

*Varacosa avara* (Keyserling, 1877)

Mimetidae

*Mimetus hesperus* Chamberlin, 1923

*Mimetus notius* Chamberlin, 1923

*Mimetus syllepsicus* Hentz, 1832

Mysmenidae

*Mysmena incredula* (Gertsch & Davis, 1936)

Nephilidae

*Nephila clavipes* (Linnaeus, 1767)

Oxyopidae

*Oxyopes acleistus* Chamberlin, 1929

*Oxyopes cougar* Brady, 1969

*Oxyopes salticus* Hentz, 1845

*Peucetia viridans* (Hentz, 1832)

Philodromidae

*Philodromus keyserlingi* Marx, 1890

*Philodromus minutus* Banks, 1892

*Philodromus pratariae* (Scheffer, 1904)

*Thanatus altimontis* Gertsch, 1933

Phrurolithidae

*Phrurolithus emertoni* Gertsch, 1935

*Phrurotimpus alarius* (Hentz, 1847)

*Phrurotimpus certus* Gertsch, 1941

Pisauridae

*Dolomedes albineus* Hentz, 1845

*Pisaurina mira* (Walckenaer, 1837)

Salticidae

*Admestina archboldi* Piel, 1992

*Admestina tibialis* (C. L. Koch, 1846)

*Anasaitis canosa* (Walckenaer, 1837)

*Colonus puerperus* (Hentz, 1846)

*Colonus sylvanus* (Hentz, 1846)

*Eris militaris* (Hentz, 1845)

*Ghelna sexmaculata* (Banks, 1895)

*Habronattus coecatus* (Hentz, 1846)

*Habronattus decorus* (Blackwall, 1846)

*Habronattus viridipes* (Hentz, 1846)

*Hentzia palmarum* (Hentz, 1832)

*Lyssomanes viridis* (Walckenaer, 1837)

*Marpissa lineata* (C. L. Koch, 1846)

*Marpissa pikei* (Peckham & Peckham, 1888)

*Metacyrba taeniola taeniola* (Hentz, 1846)

*Naphrys pulex* (Hentz, 1846)

*Peckhamia americana* (Peckham & Peckham, 1892)

*Pelegrina galathea* (Walckenaer, 1837)

*Phidippus cardinalis* (Hentz, 1845)

*Phidippus clarus* Keyserling, 1885

*Platycryptus undatus* (De Geer, 1778)

*Sassacus cyaneus* (Hentz, 1846)

*Synageles noxiosus* (Hentz, 1850)

*Zygoballus rufipes* Peckham & Peckham, 1885

*Zygoballus sexpunctatus* (Hentz, 1845)

Segestriidae

*Ariadna bicolor* (Hentz, 1842)

Sicariidae

*Loxosceles reclusa* Gertsch & Mulaik, 1940

Tetragnathidae

*Leucauge venusta* (Walckenaer, 1841)

*Pachygnatha tristriata* C. L. Koch, 1845

*Tetragnatha laboriosa* Hentz, 1850

Theridiidae

*Argyrodes elevatus* Taczanowski, 1873

*Asagena americana* Emerton, 1882

*Dipoena nigra* (Emerton, 1882)

*Euryopis spinigera* O. P.-Cambridge, 1895

*Faiditus cancellatus* (Hentz, 1850)

*Latrodectus mactans* (Fabricius, 1775)

*Neospintharus furcatus* (O. P.-Cambridge, 1894)

*Neospintharus trigonum* (Hentz, 1850)

*Parasteatoda tepidariorum* (C. L. Koch, 1841)

*Phycosoma lineatipes* (Bryant, 1933)

*Rhomphaea projiciens* O. P.-Cambridge, 1896

*Theridion flavonotatum* Becker, 1879

*Thymoites unimaculatus* (Emerton, 1882)

*Wamba crispulus* (Simon, 1895)

*Yunohamella lyrica* (Walckenaer, 1841)

Thomisidae

*Mecaphesa asperata* (Hentz, 1847)

*Mecaphesa celer* (Hentz, 1847)

*Misumenoides formosipes* (Walckenaer, 1837)

*Synema parvulum* (Hentz, 1847)

*Synema viridans* (Banks, 1896)

*Tmarus floridensis* Keyserling, 1884

*Xysticus ferox* (Hentz, 1847)

*Xysticus fraternus* Banks, 1895

*Xysticus funestus* Keyserling, 1880

Titanoecidae

*Titanoeca americana* Emerton, 1888

Trachelidae

*Trachelas mexicanus* Banks, 1898

*Trachelas similis* F. O. P.-Cambridge, 1899

*Trachelas volutus* Gertsch, 1935

Uloboridae

*Uloborus glomosus* (Walckenaer, 1841)

**Sabal Palm Audubon Sanctuary, Cameron County (61 spp.)**

Atypidae

*Sphodros paisano* Gertsch & Platnick, 1980

Anyphaenidae

*Hibana arunda* (Platnick, 1974)

*Wulfila bryantae* Platnick, 1974

Araneidae

*Acacesia hamata* (Hentz, 1847)

*Araneus pegnia* (Walckenaer, 1841)

*Argiope blanda* O. P.-Cambridge, 1898

*Eriophora edax* (Blackwall, 1863)

*Eriophora ravilla* (C. L. Koch, 1844)

*Eustala anastera* (Walckenaer, 1841)

*Eustala bifida* F. O. P.-Cambridge, 1904

*Gasteracantha cancriformis* (Linnaeus, 1758)

*Larinia directa* (Hentz, 1847)

*Metazygia zilloides* (Banks, 1898)

*Micrathena sagittata* (Walckenaer, 1841)

*Ocrepeira ectypa* (Walckenaer, 1841)

*Wagneriana tauricornis* (O. P.-Cambridge, 1889)

Clubionidae

*Elaver mulaiki* (Gertsch, 1935)

Corinnidae

*Mazax pax* Reiskind, 1969

Ctenidae

*Leptoctenus byrrhus* Simon, 1888

Filistatidae

*Kukulcania hibernalis* (Hentz, 1842)

Hersiliidae

*Neotama mexicana* (O. P.-Cambridge, 1893)

Linyphiidae

*Agyneta flax* Dupérré, 2013

*Agyneta serrata* (Emerton, 1909)

Mimetidae

*Mimetus haynesi* Gertsch & Mulaik, 1940

Miturgidae

*Teminius affinis* Banks, 1897

Mysmenidae

*Mysmena incredula* (Gertsch & Davis, 1936)

Nesticidae

*Eidmannella pallida* (Emerton, 1875)

Oonopidae

*Noonops furtivus* (Gertsch, 1936)

Oxyopidae

*Hamataliwa grisea* Keyserling, 1887

*Hamataliwa helia* (Chamberlin, 1929)

*Peucetia longipalpis* F. O. P.-Cambridge, 1902

*Peucetia viridans* (Hentz, 1832)

Pholcidae

*Modisimus texanus* Banks, 1906

Pisauridae

*Pisaurina dubia* (Hentz, 1847)

Salticidae

*Bredana complicata* Gertsch, 1936

*Cheliferoides longimanus* Gertsch, 1936

*Cheliferoides segmentatus* F. O. P.-Cambridge, 1901

*Colonus sylvanus* (Hentz, 1846)

*Leptofreya ambigua* (C. L. Koch, 1846)

*Messua limbata* (Banks, 1898)

*Metacyrba punctata* (Peckham & Peckham, 1894)

*Metacyrba taeniola similis* Banks, 1904

*Naphrys acerba* (Peckham & Peckham, 1909)

*Paramaevia poultoni* (Peckham & Peckham, 1901)

*Parnaenus* sp.

*Peckhamia americana* (Peckham & Peckham, 1892)

*Zygoballus rufipes* Peckham & Peckham, 1885

*Zygoballus sexpunctatus* (Hentz, 1845)

Scytodidae

*Scytodes atlacoya* Rheims, Brescovit & Durán, 2007

*Scytodes lugubris* (Thorell, 1887)

Sicariidae

*Loxosceles devia* Gertsch & Mulaik, 1940

Tetragnathidae

*Tetragnatha guatemalensis* O. P.-Cambridge, 1889

Theridiidae

*Euryopis spinigera* O. P.-Cambridge, 1895

*Rhomphaea projiciens* O. P.-Cambridge, 1896

*Wamba crispulus* (Simon, 1895)

Thomisidae

*Mecaphesa dubia* (Keyserling, 1880)

*Synema viridans* (Banks, 1896)

*Xysticus funestus* Keyserling, 1880

Trachelidae

*Trachelas volutus* Gertsch, 1935

Uloboridae

*Miagrammopes mexicanus* O. P.-Cambridge, 1893

Zoropsidae

*Zorocrates alternatus* Gertsch & Davis, 1936

**We lder Wildlife Refuge, San Patricio County (54 spp.)**

Agelenidae

*Agelenopsis emertoni* Chamberlin & Ivie, 1935

Araneidae

*Araneus miniatus* (Walckenaer, 1841)

*Argiope aurantia* Lucas, 1833

*Larinia directa* (Hentz, 1847)

*Mangora gibberosa* (Hentz, 1847)

*Mastophora cornigera* (Hentz, 1850)

*Neoscona arabesca* (Walckenaer, 1841)

*Neoscona crucifera* (Lucas, 1838)

*Neoscona utahana* (Chamberlin, 1919)

Corinnidae

*Falconina gracilis* (Keyserling, 1891)

Eutichuridae

*Strotarchus piscatorius* (Hentz, 1847)

*Strotarchus planeticus* Edwards, 1958

Filistatidae

*Kukulcania hibernalis* (Hentz, 1842)

Gnaphosidae

*Drassyllus creolus* Chamberlin & Gertsch, 1940

*Drassyllus lepidus* (Banks, 1899)

*Drassyllus texamans* Chamberlin, 1936

*Gnaphosa sericata* (L. Koch, 1866)

*Micaria nanella* Gertsch, 1935

*Trachyzelotes lyonneti* (Audouin, 1826)

*Zelotes pseustes* Chamberlin, 1922

Linyphiidae

*Florinda coccinea* (Hentz, 1850)

Lycosidae

*Hogna antelucana* (Montgomery, 1904)

*Rabidosa punctulata* (Hentz, 1844)

*Rabidosa rabida* (Walckenaer, 1837)

*Tigrosa georgicola* (Walckenaer, 1837)

*Trochosa sepulchralis* (Montgomery, 1902)

Mimetidae

*Mimetus notius* Chamberlin, 1923

Miturgidae

*Teminius affinis* Banks, 1897

Oonopidae

*Oonopoides secretus* (Gertsch, 1936)

Oxyopidae

*Hamataliwa grisea* Keyserling, 1887

*Oxyopes acleistus* Chamberlin, 1929

*Oxyopes apollo* Brady, 1964

*Oxyopes salticus* Hentz, 1845

Pholcidae

*Psilochorus redemptus* Gertsch & Mulaik, 1940

Salticidae

*Eris militaris* (Hentz, 1845)

*Habronattus coecatus* (Hentz, 1846)

*Habronattus delectus* (Peckham & Peckham, 1909)

*Habronattus fallax* (Peckham & Peckham, 1909)

*Habronattus viridipes* (Hentz, 1846)

*Hentzia palmarum* (Hentz, 1832)

*Naphrys pulex* (Hentz, 1846)

*Pelegrina galathea* (Walckenaer, 1837)

*Phidippus audax* (Hentz, 1845)

*Zygoballus rufipes* Peckham & Peckham, 1885

*Zygoballus sexpunctatus* (Hentz, 1845)

Scytodidae

*Scytodes atlacoya* Rheims, Brescovit & Durán, 2007

Theridiidae

*Latrodectus mactans* (Fabricius, 1775)

Thomisidae

*Mecaphesa californica* (Banks, 1896)

*Mecaphesa dubia* (Keyserling, 1880)

*Misumenoides formosipes* (Walckenaer, 1837)

*Xysticus auctificus* Keyserling, 1880

*Xysticus ferox* (Hentz, 1847)

*Xysticus texanus* Banks, 1904

Trachelidae

*Meriola decepta* Banks, 1895

### Prairie study

In a dissertation by Calixto (2008), a large number of pitfall traps (60 traps per site per week) were used to study ants at three sites in two counties. Spiders were retained (26,287 total, 63.1% adults) and each was measured. A total of 177 species in 29 families were recorded. The sizes of adults (in mm) are included here except those where the abdomen was missing or could not be identified to species. They were measured from the front of the cephalothorax (excluding the eyes) to the end of the abdomen (excluding the spinnerets). The number in brackets [] is the number of specimens of each size. The habitat is post oak savanna with pasture. This data is previously unpublished.

Barr Site, Burleson Co., 4.5 mi. SW Snook, 30.4339°N, 96.5114°W

C3 Site, Coryell Co., 7.7 mi. E Gatesville, 31.4269°N, 97.6123°W

Pruitt Site – Coryell Co., 4.8 mi. N Gatesville, 31.5069°N, 97.7249°W

**Table A1.** Number of spiders at 3 sites by year.

Location	Year	Number weeks	Number spiders	Number immatures	Number adults	Number adults listed here	Number adults unidentified	% adults
Barr Burleson Co.	2006 2007	21 21	5,068 6,054	2,356 2,125	2,712 3,929	2,687 3,798	25 131	53.5 64.9
C3 Coryell Co.	2006 2007	19 19	3,886 4,264	1,525 1,152	2,361 3,112	2,307 3,023	54 89	60.8 73.0
Pruitt Coryell Co.	2006 2007	20 18	3,170 3,845	1,217 1,312	1,953 2,533	1,869 2,411	84 122	61.6 65.9
Total			26,287	9,687	16,600	16,095	505	63.2

**Table A2.** Number of species at three sites by year. First number, 2006, second number, 2007.

Number of species	Barr	Barr Total	C3	C3 Total	Pruitt	Pruitt Total	Total
Euctenizidae	0, 0	0	0, 1	1	0, 1	1	1
Agelenidae	1, 1	1	0, 0	0	0, 0	0	1
Anyphaenidae	0, 0	0	0, 0	0	2, 0	2	2
Araneidae	1, 1	1	1, 1	2	0, 0	0	3
Clubionidae	0, 1	1	0, 0	0	0, 0	0	1
Corinnidae	4, 4	4	5, 4	5	5, 5	6	7
Dictynidae	3, 3	4	2, 4	5	1, 1	1	6
Eutichuridae	0, 1	1	0, 1	1	0, 0	0	1
Gnaphosidae	16, 14	20	21, 18	24	16, 18	20	32
Hahniidae	5, 4	5	3, 3	4	4, 2	4	5
Linyphiidae	10, 9	11	10, 13	15	7, 11	12	17
Lycosidae	8, 7	11	11, 9	14	4, 5	7	17
Mimetidae	0, 0	0	0, 1	1	0, 0	0	1
Miturgidae	1, 1	1	1, 1	1	1, 1	1	1
Mysmenidae	1, 0	1	0, 1	1	1, 1	1	1
Nesticidae	1, 0	1	0, 0	0	0, 0	0	1
Oonopidae	1, 0	1	0, 0	0	0, 0	0	1
Oxyopidae	3, 2	3	2, 2	2	2, 2	2	3
Philodromidae	2, 2	3	3, 0	3	3, 2	5	6
Pholcidae	0, 0	0	0, 0	0	1, 1	1	1
Phrurolithidae	3, 3	3	2, 3	3	2, 4	4	4
Salticidae	15, 14	22	16, 20	21	10, 9	14	32
Scytodidae	0, 0	0	1, 0	1	0, 0	0	1
Tetragnathidae	1, 3	3	0, 1	1	0, 2	2	3
Theridiidae	1, 1	2	6, 5	9	1, 7	7	13
Thomisidae	5, 3	5	6, 5	8	2, 7	8	12
Titanoecidae	0, 0	0	1, 1	1	1, 1	1	1
Trachelidae	1, 2	2	1, 1	1	1, 1	1	2
Uloboridae	0, 0	0	0, 0	0	1, 0	1	1
# species # families	83, 76 20, 19	106 22	93, 95 17, 20	122 22	65, 83 19, 20	101 21	177 29

**Table A3.** Species and measurement ranges in millimeters by sex (male, female).

Family/species	Number males	Male size	Number females	Female size
Euctenizidae				
*Myrmekiaphila comstocki* Bishop & Crosby, 1926	2	12.5, 15.0		
Agelenidae				
*Agelenopsis emertoni* Chamberlin & Ivie, 1935	5	8.3–10.0	2	7.3–8.5
Anyphaenidae				
*Hibana futilis* (Banks, 1898)	1	9.7		
*Hibana gracilis* (Hentz, 1847)	1	8.5		
Araneidae				
*Argiope trifasciata* (Forskål, 1775)	1	4.3		
*Hypsosinga funebris* (Keyserling, 1892)	6	2.7–3.5	2	3.9, 4.4
*Mangora fascialata* Franganillo, 1936	1	2.4		
Clubionidae				
*Clubiona catawba* Gertsch, 1941			3	3.4–3.9
Corinnidae				
*Castianeira alteranda* Gertsch, 1942			2	9.1, 9.8
*Castianeira amoena* (C. L. Koch, 1841)			1	8.3
*Castianeira crocata* (Hentz, 1847)			9	8.3–10.7
*Castianeira descripta* (Hentz, 1847)	61	5.7–8.4	18	6.6–10.1
*Castianeira longipalpa* (Hentz, 1847)	19	5.3–7.4		
*Castianeira trilineata* (Hentz, 1847)	2	6.3, 6.6		
*Falconina gracilis* (Keyserling, 1891)	358	4.1–8.1	430	2.9–9.8
Dictynidae				
*Dictyna annexa* Gertsch & Mulaik, 1936	2	1.9	1	2.1
*Dictyna formidolosa* Gertsch & Ivie, 1936	10	1.7–2.4		
*Dictyna volucripes* Keyserling, 1881	1	1.2		
*Emblyna consulta* Keyserling, 1881	1	1.7		
*Lathys delicatula* (Gertsch & Mulaik, 1936)	4	1.1–1.6		
*Phantyna segregata* (Gertsch & Mulaik, 1936)	175	1.6–3.1	37	1.9–3.2
Eutichuridae				
*Cheiracanthium inclusum* (Hentz, 1847)	2	6.1, 6.3		
Gnaphosidae				
*Callilepis gertschi* Platnick, 1975	2	2.8, 2.9		
*Camillina pulchra* (Keyserling, 1891)	10	2.7–3.7	10	3.0–5.7
*Cesonia bilineata* (Hentz, 1847)	22	3.9–5.8	1	6.1
*Drassyllus antonito* Platnick & Shadab, 1982	10	1.9–2.4		
*Drassyllus aprilinus* (Banks, 1904)	8	3.9–4.4		
*Drassyllus dixinus* Chamberlin, 1922	2	3.5, 3.7	1	3.5
*Drassyllus inanus* Chamberlin & Gertsch, 1940	218	1.4–2.9	76	1.7–3.5
*Drassyllus lepidus* (Banks, 1899)	64	2.4–4.8	67	2.7–6.0
*Drassyllus notonus* Chamberlin, 1928	1	2.4	11	2.2–4.4
*Drassyllus orgilus* Chamberlin, 1922			1	6.8
*Drassyllus texamans* Chamberlin, 1936	10	3.5–4.0	10	3.0–4.1
*Gnaphosa altudona* Chamberlin, 1922	23	2.8–4.4	4	3.7–4.8
*Gnaphosa fontinalis* Keyserling, 1887	1	7.5	1	8.7
*Gnaphosa sericata* (L. Koch, 1866)	93	4.3–6.4	25	4.1–9.6
*Haplodrassus signifer* (C. L. Koch, 1839)	3	5.6–7.1		
*Litopyllus temporarius* Chamberlin, 1922	1	6.2		
*Micaria deserticola* Gertsch, 1933	23	2.0–4.9	2	2.1, 2.8
*Micaria longipes* Emerton, 1890			1	4.9
*Micaria nanella* Gertsch, 1935	164	1.3–3.5	50	1.9–2.7
*Micaria nye* Platnick & Shadab, 1988	18	1.8–2.5	7	2.0–2.9
*Nodocion floridanus* (Banks, 1896)	1	5.3		
*Synaphosus paludis* (Chamberlin & Gertsch, 1940)	11	3.1–5.6	11	4.0–6.7
*Talanites captiosus* (Gertsch & Davis, 1936)	14	3.1–4.5	7	3.7–4.8
*Talanites exlineae* (Platnick & Shadab, 1976)	4	3.3–5.1	3	3.4–4.3
*Zelotes aiken* Platnick & Shadab, 1983	36	3.8–6.5	11	4.4–6.2
*Zelotes anglo* Gertsch & Riechert, 1976	9	5.0–9.1	8	6.8–9.7
*Zelotes gertschi* Platnick & Shadab, 1983	40	3.9–7.2	21	4.0–9.2
*Zelotes hentzi* Barrows, 1945	1	5.9	4	7.0–7.7
*Zelotes lasalanus* Chamberlin, 1928			2	5.7, 6.3
*Zelotes lymnophilus* Chamberlin, 1936	17	3.1–4.1		
*Zelotes pseustes* Chamberlin, 1922	5	6.0–7.2	1	5
*Zelotes tuobus* Chamberlin, 1919	2	7.3, 8.0	2	5.0, 8.0
Hahniidae				
*Hahnia cinerea* Emerton, 1890	7	1.6–1.9	77	1.7–2.4
*Hahnia flaviceps* Emerton, 1913	2	1.5, 1.8	7	1.6–2.0
*Neoantistea agilis* (Keyserling, 1887)	9	2.3–3.7	2	3.8, 4.6
*Neoantistea mulaiki* Gertsch, 1946	243	2.3–5.1	106	2.8–7.2
*Neoantistea oklahomensis* Opell & Beatty, 1976	1	2.8	11	2.7–4.4
Linyphiidae				
*Agyneta chiricahua* Dupérré, 2013	123	1.2–1.9	75	1.6–2.6
*Agyneta crista* Dupérré, 2013	151	1.2–2.1	71	1.0–2.0
*Agyneta flax* Dupérré, 2013	1	1.8	2	1.8
*Agyneta micaria* (Emerton, 1882)	2	1.3, 1.7	1	1.3
*Agyneta parva* (Banks, 1898)	1	1.4	3	1.7–2.2
*Agyneta regina* (Chamberlin & Ivie, 1944)			11	1.3–1.9
*Agyneta sandia* Dupérré, 2013			2	1.5
*Agyneta serrata* (Emerton, 1909)	134	1.0–1.8	14	1.1–1.4
*Ceraticelus laetus* (O. P.-Cambridge, 1874)	1	1.6		
*Ceratinella brunnea* Emerton, 1882	244	1.0–3.3	75	1.1–2.0
*Ceratinops crenatus* (Emerton, 1882)	312	0.9–2.8	97	1.5–2.5
*Ceratinops latus* (Emerton, 1882)	4	1.4–1.5	1	1.5
*Erigone autumnalis* Emerton, 1882	798	0.9–1.9	152	0.8–2.0
*Grammonota texana* (Banks, 1899)	16	2.2–3.0	9	2.7–3.7
*Mermessus denticulatus* (Banks, 1898)	3	1.7–2.7	3	1.7–2.8
*Tennesseellum formicum* (Emerton, 1882)	2.273	1.1–2.9	73	1.3–2.2
*Walckenaeria puella* Millidge, 1983			9	1.2–2.2
Lycosidae				
*Allocosa funerea* (Hentz, 1844)			1	4.9
*Hogna antelucana* (Montgomery, 1904)	4	10.3–13.8	3	18.1–22.8
*Hogna carolinensis* (Walckenaer, 1805)			2	26.0, 28.0
*Hogna frondicola* Emerton, 1885			1	9.9
*Pardosa delicatula* Gertsch & Wallace, 1935	3	4.0–4.5		
*Pardosa mercurialis* Montgomery, 1904			1	8.4
*Pardosa milvina* (Hentz, 1844)	4	3.5–4.8		
*Pardosa pauxilla* Montgomery, 1904	109	3.7–5.3	46	4.0–7.2
*Pirata hiteorum* Wallace & Exline, 1978	2	2.4, 2.9	1	3.1
*Rabidosa punctulata* (Hentz, 1844)			2	13.2, 14.0
*Schizocosa avida* (Walckenaer, 1837)	248	5.2–15.5	189	6.8–17.0
*Schizocosa bilineata* (Emerton, 1885)			1	12.3
*Schizocosa rovneri* Uetz & Dondale, 1979	3	6.6–7.4		
*Schizocosa saltatrix* (Hentz, 1844)	22	6.8–10.3	8	7.8–12.2
*Trochosa sepulchralis* (Montgomery, 1902)	1	9.4		
*Varacosa avara* (Keyserling, 1877)			4	8.9–10.8
*Varacosa shenandoa* (Chamberlin & Ivie, 1942)			5	9.3–12.1
Mimetidae				
*Mimetus hesperus* Chamberlin, 1923			1	5.2
Miturgidae				
*Teminius affinis* Banks, 1897	12	5.9–12.8	13	9.5–16.2
Mysmenidae				
*Mysmena incredula* (Gertsch & Davis, 1936)	3	0.6–0.8	3	0.7–1.1
Nesticidae				
*Eidmannella pallida* (Emerton, 1875)	3	1.6–1.8	1	2
Oonopidae				
*Oonopoides secretus* (Gertsch, 1936)	1	1.4		
Oxyopidae				
*Hamataliwa grisea* Keyserling, 1887			1	7.9
*Oxyopes apollo* Brady, 1964	3.844	3.0–6.7	739	3.7–7.7
*Oxyopes salticus* Hentz, 1845	111	3.0–5.8	176	3.6–8.1
Philodromidae				
*Ebo punctatus* Sauer & Platnick, 1972	1	1.9	2	2.8, 3.1
*Philodromus keyserlingi* Marx, 1890	1	4.5		
*Thanatus formicinus* (Clerck, 1757)	6	4.1–6.3		
*Thanatus rubicellus* Mello-Leitão, 1929	3	5.0–5.1	3	5.2–6.7
*Tibellus duttoni* (Hentz, 1847)	3	6.0–8.0	1	8.8
*Titanebo albocaudatus* (Schick, 1965)			1	3.2
Pholcidae				
*Psilochorus utahensis* Chamberlin, 1919	21	1.3–2.4	12	1.6–2.5
Phrurolithidae				
*Phrurotimpus alarius* (Hentz, 1847)	56	1.7–2.4	34	2.0–3.5
*Phrurotimpus borealis* (Emerton, 1911)	4	2.4–2.7	4	2.7–3.3
*Phrurotimpus certus* Gertsch, 1941	35	1.5–2.7	71	1.9–3.7
*Scotinella fratrella* (Gertsch, 1935)	30	1.3–1.7	15	1.3–2.2
Salticidae				
*Admestina archboldi* Piel, 1992	1	2.9		
*Anasaitis canosa* (Walckenaer, 1837)	7	3.6–4.9		
*Chalcoscirtus diminutus* (Banks, 1896)	6	1.9–2.4		
*Colonus sylvanus* (Hentz, 1846)	1	6.2		
*Habronattus calcaratus* (Banks, 1904)	67	4.4–5.6	2	6.0, 6.3
*Habronattus coecatus* (Hentz, 1846)	500	2.3–6.2	306	4.5–9.1
*Habronattus cognatus* (Peckham & Peckham, 1901)	98	4.4–5.9	43	5.0–7.8
*Habronattus decorus* (Blackwall, 1846)	18	4.6–6.1	12	5.2–8.3
*Habronattus fallax* (Peckham & Peckham, 1909)	8	4.4–5.1		
*Habronattus orbus* Griswold, 1987			1	5.6
*Habronattus texanus* (Chamberlin, 1924)	285	3.1–5.2	102	3.8–7.7
*Habronattus viridipes* (Hentz, 1846)			22	5.2–7.4
*Maevia inclemens* (Walckenaer, 1837)	1	5.8		
*Marpissa lineata* (C. L. Koch, 1846)	6	3.1–4.3	2	3.5, 3.9
*Metacyrba taeniola taeniola* (Hentz, 1846)	2	4.3, 5.6	1	5.4
*Naphrys pulex* (Hentz, 1846)	4	3.8–4.0	2	3.6, 4.1
*Neonella vinnula* Gertsch, 1936	1	1.4	3	1.3–1.9
*Pelegrina galathea* (Walckenaer, 1837)	1	4.9		
*Pellenes limatus* Peckham & Peckham, 1901	35	4.5–7.6	10	5.8–8.6
*Phidippus audax* (Hentz, 1845)	1	10.1		
*Phidippus cardinalis* (Hentz, 1845)	3	11.1–12.6	1	13
*Phidippus clarus* Keyserling, 1885			4	12.6–15.7
*Phidippus comatus* Peckham & Peckham, 1901	1	8.6		
*Phidippus texanus* Banks, 1906	4	9.5–14.4	1	16.5
*Phidippus whitmani* Peckham & Peckham, 1909	1	7.7		
*Phlegra hentzi* (Marx, 1890)	5	5.9–6.7		
*Sarinda hentzi* (Banks, 1913)	27	3.3–6.5	9	3.7–7.0
*Sitticus dorsatus* (Banks, 1895)	2	2.6, 2.9	1	3
*Synageles noxiosus* (Hentz, 1850)	3	2.6–3.1	1	3
*Talavera minuta* (Banks, 1895)	3	2.2–2.5		
*Zygoballus rufipes* Peckham & Peckham, 1885	4	3.6–4.2		
*Zygoballus sexpunctatus* (Hentz, 1845)			1	3.3
Scytodidae				
*Scytodes atlacoya* Rheims, Brescovit & Durán, 2007	1	7		
Tetragnathidae				
*Glenognatha foxi* (McCook, 1894)	554	1.3–2.2	446	1.4–2.7
*Pachygnatha autumnalis* Marx, 1884	1	4.1		
*Tetragnatha laboriosa* Hentz, 1850	4	4.7–5.8	1	5
Theridiidae				
*Dipoena abdita* Gertsch & Mulaik, 1936	2	1.5, 1.7		
*Hentziectypus schullei* (Gertsch & Mulaik, 1936)	1	1		
*Latrodectus mactans* (Fabricius, 1775)	1	4.5		
*Theridion australe* Banks, 1899	1	2.4	1	2.9
*Theridion cinctipes* Banks, 1898	5	1.3–1.5		
*Theridion dilutum* Levi, 1957	3	2.0–2.4		
*Theridion dividuum* Gertsch & Archer, 1942	4	1.1–1.4		
*Theridion flavonotatum* Becker, 1879			1	2.1
*Theridion hidalgo* Levi, 1957			1	2.1
*Theridion llano* Levi, 1957	1	1.6	1	1.6
*Theridion murarium* Emerton, 1882	3	1.2–1.6		
*Theridion positivum* Chamberlin, 1924	1	1.6		
*Theridion rabuni* Chamberlin & Ivie, 1944	3	1.6–1.8	2	1.5, 2.1
Thomisidae				
*Mecaphesa celer* (Hentz, 1847)	4	2.9–3.4	1	6.2
*Misumenoides formosipes* (Walckenaer, 1837)	1	3.5		
*Xysticus apachecus* Gertsch, 1933	1	4		
*Xysticus auctificus* Keyserling, 1880	8	3.6–5.2	2	5.4, 7.1
*Xysticus concursus* Gertsch, 1934			1	7
*Xysticus ferox* (Hentz, 1847)	5	4.0–5.6	3	6.9–7.0
*Xysticus funestus* Keyserling, 1880	2	4.2, 5.6	4	6.5–9.0
*Xysticus gulosus* Keyserling, 1880			1	8.3
*Xysticus paiutus* Gertsch, 1933	1	5.3		
*Xysticus pellax* O. P.-Cambridge, 1894	26	3.9–6.0	4	6.5–7.5
*Xysticus robinsoni* Gertsch, 1953	2	4.7, 5.2		
*Xysticus texanus* Banks, 1904	4	4.6–6.3		
Titanoecidae				
*Titanoeca americana* Emerton, 1888	15	5.2–6.7	2	5.3, 5.7
Trachelidae				
*Meriola decepta* Banks, 1895	13	2.7–4.9	28	3.6–5.0
*Trachelas mexicanus* Banks, 1898	1	5.4		
Uloboridae				
*Uloborus glomosus* (Walckenaer, 1841)	1	2.9		
**Total adults, males and females**	**12,084**		**4,011**	

#### Barr (Burleson Co.)

**Agelenidae**

*Agelenopsis emertoni* Chamberlin & Ivie, 1935

3m, 1f 2006 m (8.3, 9.2, 10.0); f (7.3)

2m, 1f 2007 m (8.7, 9.3); f (8.5)

**Araneidae**

*Hypsosinga funebris* (Keyserling, 1892)

3m, 1f 2006 m (2.7, 2.8, 3.0); f (3.9)

3m, 1f 2007 m (3.2, 3.3, 3.5); f (4.4)

**Clubionidae**

*Clubiona catawba* Gertsch, 1941

3f 2007 f (3.4, 3.7, 3.9)

**Corinnidae**

*Castianeira crocata* (Hentz, 1847)

1f 2006 f (8.3)

1f 2007 f (10.7)

*Castianeira descripta* (Hentz, 1847)

21m, 5f 2006 m (6.7, 6.8, 6.9 [2], 7.0 [2], 7.1, 7.2 [3], 7.3, 7.4, 7.5, 7.6, 7.7, 7.8 [2], 7.9, 8.0, 8.4 [2]); f (8.6, 8.7, 9.2, 9.4, 9.8)

3m, 1f 2007 m (7.0, 7.5, 8.2); f (10.1)

*Castianeira longipalpa* (Hentz, 1847)

9m 2006 m (5.3, 6.1, 6.4, 6.5, 6.6, 6.7, 7.2, 7.3, 7.4)

2m 2007 m (6.5, 7.0)

*Falconina gracilis* (Keyserling, 1891)

101m, 76f 2006 m (4.9, 5.1, 5.2, 5.3, 5.4 [2], 5.5 [4], 5.6 [3], 5.7 [2], 5.8 [3], 5.9, 6.0 [10], 6.1 [6], 6.2 [7], 6.3 [3], 6.4 [6], 6.5 [8], 6.6 [10], 6.7 [3], 6.8 [4], 6.9 [6], 7.0 [5], 7.1 [5], 7.2 [2], 7.3 [2], 7.4, 7.5 [3], 7.9); f (5.3, 5.4, 5.5, 5.6 [2], 5.8 [6], 6.0, 6.2 [2], 6.3 [3], 6.4 [2], 6.5 [3], 6.6 [2], 6.7, 6.8 [6], 6.9 [3], 7.0 [5], 7.1 [2], 7.2 [7], 7.5 [3], 7.6 [2], 7.7 [4], 7.8, 7.9, 8.0 [2], 8.2 [3], 8.3, 8.4, 8.5 [3], 8.8, 9.0 [4], 9.4, 9.8)

31m, 51f 2007 m (4.5, 5.2, 5.3, 5.4, 5.6 [3], 5.7 [2], 5.8 [7], 6.2 [2], 6.3 [2], 6.4 [2], 6.6, 6.7 [2], 6.8 [3], 7.0, 7.8, 7.9); f (5.1, 5.5, 6.0 [3], 6.1 [3], 6.3 [4], 6.4 [3], 6.6, 6.7 [3], 6.8 [2], 6.9 [3], 7.0 [2], 7.1 [2], 7.2 [2], 7.4 [2], 7.5 [3], 7.6 [2], 7.7, 7.8 [2], 7.9, 8.0, 8.1, 8.2, 8.4 [2], 8.5, 8.7, 8.9, 9.0 [2])

**Dictynidae**

*Dictyna annexa* Gertsch & Mulaik, 1936

1f 2007 f (2.1)

*Dictyna formidolosa* Gertsch & Ivie, 1936

3m 2006 m (1.9 [2], 2.2)

7m 2007 m (1.7, 1.9 [2], 2.0 [2], 2.1, 2.4)

*Lathys delicatula* (Gertsch & Mulaik, 1936)

3m 2006 m (1.2, 1.4, 1.6)

*Phantyna segregata* (Gertsch & Mulaik, 1936)

71m, 23f 2006 m (1.6, 1.8 [2], 1.9, 2.0 [8], 2.1 [6], 2.2 [16], 2.3 [13], 2.4 [11], 2.5 [6], 2.6 [3], 2.7 [2], 2.8, 3.1); f (1.9, 2.0, 2.1 [2], 2.2 [2], 2.3 [5], 2.4 [4], 2.5 [3], 2.6 [2], 2.7, 3.0, 3.2)

66m, 8f 2007 m (1.9 [4], 2.0 [3], 2.1 [2], 2.2 [8], 2.3 [7], 2.4 [12], 2.5 [3], 2.6 [4], 2.7 [6], 2.8 [4], 2.9 [6], 3.0 [4], 3.1 [3]); f (2.2, 2.3, 2.4, 2.6, 2.7 [2], 2.8, 2.9)

**Eutichuridae**

*Cheiracanthium inclusum* (Hentz, 1847)

1m 2007 m (6.3)

**Gnaphosidae**

*Camillina pulchra* (Keyserling, 1891)

9m, 7f 2006 m (2.8, 2.9, 3.0 [2], 3.1 [2], 3.3, 3.5, 3.7); f (3.2, 3.6, 3.7, 3.8, 4.0, 4.3, 5.7)

1f 2007 f (3.9)

*Cesonia bilineata* (Hentz, 1847)

12m, 1f 2006 m (3.9 [2], 4.2, 4.3, 4.4, 4.5, 4.6, 4.7, 4.8, 4.9, 5.1, 5.2); f (6.1)

4m 2007 m (3.9, 4.0, 4.1, 4.3)

*Drassyllus aprilinus* (Banks, 1904)

1m 2006 m (3.9)

3m 2007 m (4.1 [2], 4.4)

*Drassyllus inanus* Chamberlin & Gertsch, 1940

48m, 8f 2006 m (1.4, 1.7, 1.8 [2], 1.9 [2], 2.0 [5], 2.1 [6], 2.2 [13], 2.3 [9], 2.4 [6], 2.5 [3]); f (2.2, 2.3 [2], 2.4, 2.5 [2], 2.8, 3.0)

48m, 21f 2007 m (1.9, 2.0 [4], 2.1 [4], 2.2 [5], 2.3 [6], 2.4 [10], 2.5 [9], 2.6 [4], 2.7 [4], 2.8); f (2.2, 2.3, 2.4 [3], 2.5 [3], 2.6 [4], 2.7 [4], 2.8, 2.9 [2], 3.0, 3.5)

*Drassyllus lepidus* (Banks, 1899)

11m, 9f 2006 m (2.4, 2.8 [2], 3.0, 3.3 [2], 3.6 [2], 3.7, 3.8, 3.9); f (2.8, 3.0, 3.4, 3.5, 3.8 [2], 3.9, 4.2, 4.8)

18m, 24f 2007 m (2.9 [2], 3.1, 3.2 [2], 3.4, 3.7, 3.8 [2], 3.9 [2], 4.1 [3], 4.2 [2], 4.3, 4.8); f (3.3, 3.7, 3.8, 3.9, 4.2, 4.4, 4.5, 4.6, 4.8 [2], 4.9 [5], 5.1 [4], 5.2, 5.6, 5.7, 5.8, 6.0)

*Drassyllus notonus* Chamberlin, 1928

5f 2006 f (2.2 [2], 2.3, 2.4, 3.9)

*Drassyllus orgilus* Chamberlin, 1922

1f 2007 f (6.8)

*Drassyllus texamans* Chamberlin, 1936

1f 2006 f (3.0)

4m, 3f 2007 m (3.6, 3.7, 3.8 [2]); f (3.3, 3.5, 3.8)

*Gnaphosa sericata* (L. Koch, 1866)

1f 2006 f (6.7)

*Litopyllus temporarius* Chamberlin, 1922

1m 2006 m (6.2)

*Micaria deserticola* Gertsch, 1933

3m 2007 m (2.0, 2.2, 2.3)

*Micaria nanella* Gertsch, 1935

6m 2006 m (1.7, 1.9 [2], 2.0, 2.2, 2.3)

15m 2007 m (1.3, 1.8, 1.9, 2.0, 2.1 [4], 2.2 [3], 2.3 [2], 2.5, 2.6)

*Micaria nye* Platnick & Shadab, 1988

1m 2007 m (2.3)

*Nodocion floridanus* (Banks, 1896)

1m 2006 m (5.3)

*Synaphosus paludis* (Chamberlin & Gertsch, 1940)

7m, 7f 2006 m (3.1, 3.7, 3.9, 4.0, 4.9, 5.0, 5.6); f (4.0, 4.6, 4.7, 5.1, 6.1, 6.4, 6.7)

4m, 4f 2007 m (3.1, 4.4, 4.5, 5.0); f (4.4, 5.0 [2], 5.4)

*Talanites exlineae* (Platnick & Shadab, 1976)

3m, 3f 2007 m (3.3, 4.5, 5.1); f (3.4, 3.8, 4.3)

*Zelotes aiken* Platnick & Shadab, 1983

2m 2006 m (5.4, 5.6)

3m 2007 m (4.7, 5.1, 5.5)

*Zelotes anglo* Gertsch & Riechert, 1976

1m, 3f 2006 m (7.2); f (6.8, 7.0, 9.0)

*Zelotes hentzi* Barrows, 1945

1m, 1f 2006 m (5.9); f (7.4)

2f 2007 f (7.0, 7.7)

*Zelotes pseustes* Chamberlin, 1922

1m 2006 m (7.0)

**Hahniidae**

*Hahnia cinerea* Emerton, 1890

2f 2006 f (1.9 [2])

*Hahnia flaviceps* Emerton, 1913

1f 2006 f (2.0)

1m, 2f 2007 m (1.8); f (1.9, 2.0)

*Neoantistea agilis* (Keyserling, 1887)

4m, 1f 2006 m (2.5, 2.7 [3]); f (4.6)

1f 2007 f (3.8)

*Neoantistea mulaiki* Gertsch, 1946

82m, 41f 2006 m (2.3, 2.6 [4], 2.8 [6], 2.9 [7], 3.0 [9], 3.1 [11], 3.2 [7], 3.3 [4], 3.4 [5], 3.5 [4], 3.6 [6], 3.7 [7], 3.8 [3], 3.9 [3], 4.0 [2], 4.1 [2], 4.6); f (3.1 [2], 3.2, 3.3 [2], 3.4 [2], 3.5 [4], 3.6 [6], 3.7 [2], 3.8 [4], 3.9 [3], 4.0 [2], 4.1 [2], 4.2 [4], 4.3 [5], 4.7, 7.2)

161m, 65f 2007 m (2.4, 2.5, 2.7 [2], 2.8 [4], 2.9 [3], 3.0, 3.1 [9], 3.2 [8], 3.3 [17], 3.4 [16], 3.5 [6], 3.6 [13], 3.7 [13], 3.8 [6], 3.9 [9], 4.0 [4], 4.1 [10], 4.2 [9], 4.3 [5], 4.4 [4], 4.5 [5], 4.6 [5], 4.7 [3], 4.8 [4], 4.9, 5.0, 5.1); f (2.8, 2.9, 3.1, 3.3 [3], 3.4 [3], 3.5 [4], 3.6, 3.7 [3], 3.8 [3], 3.9 [5], 4.0 [2], 4.1 [5], 4.2 [4], 4.3 [6], 4.4 [4], 4.5 [3], 4.6 [3], 4.7, 4.8 [3], 4.9 [6], 5.0 [2], 5.1)

*Neoantistea oklahomensis* Opell & Beatty, 1976

8f 2006 f (2.7, 2.9, 3.1, 3.5 [2], 3.6, 4.0, 4.4)

1f 2007 f (3.2)

**Linyphiidae**

*Agyneta chiricahua* Dupérré, 2013

1m, 2f 2006 m (1.4); f (1.4, 1.5)

1m, 1f 2007 m (1.6); f (1.6)

*Agyneta crista* Dupérré, 2013

2m, 2f 2006 m (1.4, 1.5); f (1.5, 1.6)

1m, 1f 2007 m (1.8); f (1.2)

*Agyneta micaria* (Emerton, 1882)

1m 2006 m (1.3)

*Agyneta parva* (Banks, 1898)

1f 2006 f (1.7)

1m 2007 m (1.4)

*Agyneta sandia* Dupérré, 2013

1f 2006 f (1.5)

*Agyneta serrata* (Emerton, 1909)

8m 2006 m (1.1 [3], 1.2, 1.3 [2], 1.5, 1.6)

24m, 10f 2007 m (1.1, 1.2 [3], 1.3 [5], 1.4 [9], 1.5 [5], 1.7); f (1.2 [2], 1.3 [4], 1.4 [4])

*Ceratinella brunnea* Emerton, 1882

122m, 55f 2006 m (1.0 [4], 1.1 [16], 1.2 [22], 1.3 [46], 1.4 [32], 1.5, 3.3); f (1.1, 1.2, 1.3 [5], 1.4 [19], 1.5 [14], 1.6 [8], 1.7 [5], 1.8, 2.0)

117m, 18f 2007 m (1.2 [8], 1.3 [30], 1.4 [48], 1.5 [31]); f (1.2, 1.4 [2], 1.5 [2], 1.6 [7], 1.7 [3], 1.8 [2], 1.9)

*Erigone autumnalis* Emerton, 1882

25m, 7f 2006 m (0.9 [2], 1.0 [3], 1.1 [6], 1.2 [5], 1.3 [3], 1.4 [3], 1.5, 1.6 [2]); f (1.1 [3], 1.2 [4])

454m, 78f 2007 m (0.9, 1.0 [3], 1.1 [7], 1.2 [25], 1.3 [46], 1.4 [73], 1.5 [107], 1.6 [101], 1.7 [65], 1.8 [21], 1.9 [5]); f (0.8, 0.9, 1.0 [4], 1.1 [15], 1.2 [22], 1.3 [13], 1.4 [8], 1.5 [6], 1.6 [6], 1.7 [2])

*Grammonota texana* (Banks, 1899)

1m 2006 m (2.4)

15m, 9f 2007 m (2.2, 2.3, 2.4 [2], 2.5 [3], 2.6 [3], 2.7 [2], 2.8, 2.9, 3.0); f (2.7 [2], 2.8 [2], 3.1, 3.3, 3.4, 3.7 [2])

*Mermessus denticulatus* (Banks, 1898)

3m, 2f 2007 m (1.7, 1.8, 2.7); f (1.7, 1.9)

*Tennesseellum formicum* (Emerton, 1882)

116m, 4f 2006 m (1.3 [4], 1.4 [5], 1.5 [6], 1.6 [18], 1.7 [25], 1.8 [26], 1.9 [19], 2.0 [12], 2.1); f (1.6 [2], 1.7, 1.9)

994m, 30f 2007 m (1.1 [2], 1.2, 1.3 [3], 1.4 [11], 1.5 [32], 1.6 [39], 1.7 [84], 1.8 [128], 1.9 [197], 2.0 [191], 2.1 [175], 2.2 [95], 2.3 [32], 2.4 [3], 2.9); f (1.3 [2], 1.4 [2], 1.5 [2], 1.6 [6], 1.7 [7], 1.8 [2], 1.9 [6], 2.0 [2], 2.1)

**Lycosidae**

*Hogna antelucana* (Montgomery, 1904)

3m 2007 m (10.3, 11.2, 13.8)

*Hogna carolinensis* (Walckenaer, 1805)

2f 2007 f (26.0, 28.0)

*Pardosa delicatula* Gertsch & Wallace, 1935

2m 2006 m (4.3, 4.5)

*Pardosa milvina* (Hentz, 1844)

2m 2006 m (3.5, 3.7)

*Pardosa pauxilla* Montgomery, 1904

13m, 7f 2006 m (3.7, 3.9, 4.0 [2], 4.1 [2], 4.2 [2], 4.5 [4], 4.6); f (4.6, 5.0, 5.3, 5.4, 5.6 [2], 6.1)

16m, 8f 2007 m (4.1, 4.2, 4.3 [3], 4.4 [2], 4.5 [4], 4.9 [3], 5.0, 5.3); f (4.9, 5.1, 5.7 [3], 6.7 [2], 6.9)

*Pirata hiteorum* Wallace & Exline, 1978

1m 2006 m (2.4)

*Rabidosa punctulata* (Hentz, 1844)

1f 2006 f (13.2)

*Schizocosa avida* (Walckenaer, 1837)

33m, 25f 2006 m (5.2, 5.8, 6.1, 7.2, 7.4, 7.5 [2], 7.6, 7.7, 7.8, 8.2, 8.3 [2], 8.4 [2], 8.5, 8.7 [2], 8.9, 9.0, 9.5 [2], 9.6, 9.8, 10.0, 10.1, 10.2, 10.8, 10.9, 11.0, 11.5 [2], 12.2); f (9.0 [2], 9.1, 9.8, 10.6, 10.9, 11.0 [2], 11.2, 11.6, 12.1, 12.3, 12.4 [2], 12.6, 12.7 [2], 12.8, 12.9, 13.1, 13.2, 13.3, 13.7, 13.9, 14.0)

28m, 26f 2007 m (6.9, 7.0 [2], 7.2, 7.9 [3], 8.1 [4], 8.2 [3], 8.3 [2], 8.4, 8.5, 8.7, 9.0 [2], 9.1 [2], 9.2, 9.4 [2], 9.5 [2]); f (7.0, 7.6, 7.8, 7.9 [2], 8.2, 8.4, 9.3, 9.4, 10.0 [2], 10.1, 10.2 [2], 10.3, 11.2, 11.3 [3], 11.5, 12.0, 12.2, 12.5 [3], 14.2)

*Schizocosa rovneri* Uetz & Dondale, 1979

2m 2006 m (6.6, 7.3)

1m 2007 m (7.4)

*Schizocosa saltatrix* (Hentz, 1844)

3m 2006 m (6.8, 7.0, 7.4)

3m, 1f 2007 m (7.5, 7.6, 8.7); f (8.8)

*Varacosa avara* (Keyserling, 1877)

3f 2007 f (8.9, 10.6, 10.8)

**Miturgidae**

*Teminius affinis* Banks, 1897

3m, 3f 2006 m (5.9, 7.6, 9.1); f (9.5, 11.6, 15.4)

2m 2007 m (7.4, 8.9)

**Mysmenidae**

*Mysmena incredula* (Gertsch & Davis, 1936)

1m 2006 m (0.6)

**Nesticidae**

*Eidmannella pallida* (Emerton, 1875)

3m, 1f 2006 m (1.6, 1.7, 1.8); f (2.0)

**Oonopidae**

*Oonopoides secretus* (Gertsch, 1936)

1m 2006 m (1.4)

**Oxyopidae**

*Hamataliwa grisea* Keyserling, 1887

1f 2006 f (7.9)

*Oxyopes apollo* Brady, 1964

901m, 210f 2006 m (3.3 [2], 3.5 [3], 3.6 [8], 3.7 [9], 3.8 [21], 3.9 [20], 4.0 [53], 4.1 [52], 4.2 [46], 4.3 [68], 4.4 [108], 4.5 [108], 4.6 [78], 4.7 [66], 4.8 [75], 4.9 [64], 5.0 [70], 5.1 [24], 5.2 [15], 5.3 [7], 5.4 [4]); f (4.2, 4.4, 4.5 [2], 4.6 [3], 4.7 [5], 4.8 [6], 4.9 [3], 5.0 [10], 5.1 [3], 5.2 [5], 5.3 [6], 5.4 [7], 5.5 [15], 5.6 [6], 5.7 [13], 5.8 [10], 5.9 [14], 6.0 [25], 6.1 [16], 6.2 [12], 6.3 [6], 6.4 [7], 6.5 [9], 6.6 [8], 6.7, 6.8 [4], 6.9 [4], 7.0 [5], 7.1, 7.2, 7.6)

461m, 148f 2007 m (3.6 [2], 3.7 [3], 3.8 [5], 3.9 [8], 4.0 [15], 4.1 [26], 4.2 [25], 4.3 [36], 4.4 [49], 4.5 [58], 4.6 [57], 4.7 [30], 4.8 [51], 4.9 [43], 5.0 [24], 5.1 [11], 5.2 [8], 5.3 [4], 5.4 [3], 5.5 [2], 6.7); f (4.1, 4.3, 4.7, 4.9 [2], 5.0, 5.1 [2], 5.2 [4], 5.3 [5], 5.4 [6], 5.5 [6], 5.6 [5], 5.7 [2], 5.8 [11], 5.9 [7], 6.0 [17], 6.1 [15], 6.2 [12], 6.3 [5], 6.4 [9], 6.5 [9], 6.6 [4], 6.7 [3], 6.8 [6], 6.9 [4], 7.0 [2], 7.1 [3], 7.2 [2], 7.4 [2], 7.7)

*Oxyopes salticus* Hentz, 1845

26m, 42f 2006 m (3.0, 3.4, 3.5 [2], 3.8 [2], 4.0 [3], 4.1 [3], 4.2 [3], 4.3 [2], 4.5, 4.6 [3], 4.7, 4.9, 5.1, 5.4, 5.8); f (3.7, 3.9, 4.4 [2], 4.5 [2], 4.6 [2], 4.7, 4.9 [4], 5.0 [3], 5.1 [2], 5.2, 5.4 [2], 5.5, 5.6, 5.7 [3], 5.8, 5.9 [5], 6.0 [4], 6.2, 6.5, 6.6, 6.7, 6.8, 7.0)

6m, 15f 2007 m (4.2, 4.3, 5.1, 5.2, 5.4, 5.5); f (5.1, 5.2, 5.7 [2], 5.8, 5.9 [2], 6.0 [2], 6.1, 6.2, 6.7, 6.8, 6.9, 7.5)

**Philodromidae**

*Thanatus formicinus* (Clerck, 1757)

1m 2007 m (6.3)

*Thanatus rubicellus* Mello-Leitão, 1929

1m 2006 m (5.0)

*Tibellus duttoni* (Hentz, 1847)

1m 2006 m (8.0)

2m 2007 m (6.0, 6.8)

**Phrurolithidae**

*Phrurotimpus alarius* (Hentz, 1847)

8m, 6f 2006 m (1.8, 1.9, 2.0 [4], 2.1, 2.2); f (2.0, 2.2, 2.4, 2.5, 2.6, 3.5)

37m, 28f 2007 m (1.7, 1.8, 1.9 [2], 2.0 [11], 2.1 [10], 2.2 [7], 2.3 [3], 2.4 [2]); f (2.3, 2.4 [2], 2.5 [6], 2.6 [3], 2.7 [3], 2.8 [5], 2.9 [5], 3.0 [3])

*Phrurotimpus borealis* (Emerton, 1911)

1f 2006 f (2.7)

1m 2007 m (2.7)

*Phrurotimpus certus* Gertsch, 1941

13m, 9f 2006 m (1.5 [2], 1.6, 1.7 [3], 1.8 [3], 1.9, 2.0 [2], 2.2); f (1.9, 2.0, 2.2 [4], 2.3 [2], 2.4)

4m, 7f 2007 m (1.7, 1.8, 1.9 [2]); f (2.2, 2.4, 2.5, 2.7 [2], 2.8 [2])

**Salticidae**

*Admestina archboldi* Piel, 1992

1m 2007 m (2.9)

*Anasaitis canosa* (Walckenaer, 1837)

2m 2006 m (4.1, 4.7)

5m 2007 m (3.6, 4.4, 4.6, 4.8, 4.9)

*Chalcoscirtus diminutus* (Banks, 1896)

2m 2006 m (2.1, 2.2)

3m 2007 m (2.2, 2.3, 2.4)

*Colonus sylvanus* (Hentz, 1846)

1m 2007 m (6.2)

*Habronattus calcaratus* (Banks, 1904)

2f 2006 f (6.0, 6.3)

*Habronattus coecatus* (Hentz, 1846)

225m, 131f 2006 m (3.8, 3.9, 4.0 [3], 4.1, 4.2 [2], 4.3 [4], 4.4 [5], 4.5 [4], 4.6 [17], 4.7 [7], 4.8 [26], 4.9 [13], 5.0 [19], 5.1 [25], 5.2 [19], 5.3 [19], 5.4 [21], 5.5 [20], 5.6 [12], 5.7 [2], 5.8 [3], 6.2); f (5.0, 5.3 [2], 5.5 [2], 5.6 [2], 5.7 [4], 5.8 [3], 5.9 [2], 6.0 [5], 6.1 [8], 6.2 [4], 6.3 [6], 6.4 [6], 6.5 [10], 6.6 [5], 6.7 [10], 6.8 [10], 6.9 [4], 7.0 [6], 7.1 [3], 7.2 [6], 7.3 [4], 7.4 [6], 7.5 [5], 7.6 [4], 7.7, 8.0 [5], 8.1, 8.2 [3], 8.5 [2], 9.1)

108m, 87f 2007 m (3.9, 4.2 [2], 4.4, 4.5 [6], 4.6 [3], 4.7 [3], 4.8 [10], 4.9 [10], 5.0 [9], 5.1 [10], 5.2 [14], 5.3 [7], 5.4 [6], 5.5 [5], 5.6 [9], 5.7 [2], 5.8 [7], 5.9 [2], 6.1); f (5.0, 5.2, 5.3 [2], 5.4, 5.5, 5.7, 5.8 [4], 5.9 [3], 6.0 [2], 6.1 [2], 6.2 [6], 6.3, 6.4 [4], 6.5 [3], 6.6 [6], 6.7 [3], 6.8 [5], 6.9 [4], 7.0 [5], 7.1 [4], 7.2 [4], 7.3 [2], 7.4 [3], 7.5 [2], 7.6 [2], 7.7, 7.8 [3], 7.9 [2], 8.0 [4], 8.1 [2], 8.4, 8.5, 8.8)

*Habronattus decorus* (Blackwall, 1846)

9m, 4f 2006 m (5.3, 5.5 [3], 5.8, 5.9, 6.0 [3]); f (6.4, 6.8, 6.9, 7.9)

8m, 5f 2007 m (5.6 [2], 5.7 [2], 5.8 [2], 5.9, 6.1); f (5.4, 6.4 [2], 6.6, 7.4)

*Habronattus viridipes* (Hentz, 1846)

1f 2007 f (7.4)

*Maevia inclemens* (Walckenaer, 1837)

1m 2006 m (5.8)

*Marpissa lineata* (C. L. Koch, 1846)

3m, 2f 2006 m (3.1, 4.0, 4.1); f (3.5, 3.9)

*Metacyrba taeniola taeniola* (Hentz, 1846)

1m 2007 m (4.3)

*Naphrys pulex* (Hentz, 1846)

3m, 2f 2007 m (3.8, 3.9, 4.0); f (3.6, 4.1)

*Neonella vinnula* Gertsch, 1936

2f 2006 f (1.3, 1.5)

1f 2007 f (1.9)

*Pellenes limatus* Peckham & Peckham, 1901

7m 2006 m (5.4, 6.0 [2], 6.4, 6.9, 7.5, 7.6)

4m, 5f 2007 m (5.5, 6.0, 6.3, 7.1); f (6.2, 6.9, 7.8, 8.6 [2])

*Phidippus audax* (Hentz, 1845)

1m 2006 m (10.1)

*Phidippus cardinalis* (Hentz, 1845)

1f 2006 f (13.0)

*Phidippus clarus* Keyserling, 1885

3f 2007 f (12.6, 15.0, 15.7)

*Phidippus comatus* Peckham & Peckham, 1901

1m 2006 m (8.6)

*Phidippus whitmani* Peckham & Peckham, 1909

1m 2006 m (7.7)

*Sarinda hentzi* (Banks, 1913)

8m, 1f 2006 m (3.4, 3.6, 3.7 [2], 4.0, 4.2, 4.3, 4.8); f (5.2)

17m, 5f 2007 m (3.3, 3.5, 3.6, 3.7 [2], 3.8, 4.0 [2], 4.2, 4.3, 4.4, 4.6, 4.7, 4.8, 5.1, 5.2, 5.3); f (3.7, 4.4, 4.8, 5.6, 6.0)

*Synageles noxiosus* (Hentz, 1850)

1m 2006 m (2.6)

*Zygoballus rufipes* Peckham & Peckham, 1885

1m 2007 m (3.9)

**Tetragnathidae**

*Glenognatha foxi* (McCook, 1894)

1f 2006 f (2.1)

221m, 161f 2007 m (1.4 [3], 1.5 [12], 1.6 [24], 1.7 [33], 1.8 [26], 1.9 [50], 2.0 [53], 2.1 [14], 2.2 [6]); f (1.4 [2], 1.5, 1.6 [8], 1.7 [18], 1.8 [12], 1.9 [17], 2.0 [20], 2.1 [22], 2.2 [31], 2.3 [13], 2.4 [11], 2.5 [2], 2.6 [2], 2.7 [2])

*Pachygnatha autumnalis* Marx, 1884

1m 2007 m (4.1)

*Tetragnatha laboriosa* Hentz, 1850

3m, 1f 2007 m (4.9, 5.2, 5.8); f (5.0)

**Theridiidae**

*Theridion positivum* Chamberlin, 1924

1m 2007 m (1.6)

*Theridion rabuni* Chamberlin & Ivie, 1944

1f 2006 f (1.5)

**Thomisidae**

*Misumenoides formosipes* (Walckenaer, 1837)

1m 2006 m (3.5)

*Xysticus auctificus* Keyserling, 1880

1m 2006 m (3.9)

*Xysticus ferox* (Hentz, 1847)

1m 2006 m (4.0)

1m, 3f 2007 m (5.1); f (6.9 [2], 7.0)

*Xysticus funestus* Keyserling, 1880

1m 2006 m (4.2)

1f 2007 f (9.0)

*Xysticus pellax* O. P.-Cambridge, 1894

3m 2006 m (4.4, 4.6, 4.9)

1m, 1f 2007 m (4.9); f (6.6)

**Trachelidae**

*Meriola decepta* Banks, 1895

2f 2006 f (4.0, 4.4)

1m 2007 m (3.5)

*Trachelas mexicanus* Banks, 1898

1m 2007 m (5.4)

#### C3, Coryell Co.

**Euctenizidae**

*Myrmekiaphila comstocki* Bishop & Crosby, 1926

1m 2007 m (12.5)

**Araneidae**

*Argiope trifasciata* (Forskål, 1775)

1m 2006 m (4.3)

*Mangora fascialata* Franganillo, 1936

1m 2007 m (2.4)

**Corinnidae**

*Castianeira amoena* (C. L. Koch, 1841)

1f 2006 f (8.3)

*Castianeira crocata* (Hentz, 1847)

2f 2006 f (8.5, 9.5)

3f 2007 f (9.5 [2], 9.9)

*Castianeira descripta* (Hentz, 1847)

13m, 8f 2006 m (5.7, 6.0, 6.2, 6.4, 6.6, 6.7, 6.8, 6.9, 7.5, 7.6 [2], 7.8 [2]); f (7.8, 8.0, 8.2, 8.9 [2], 9.2 [2], 9.4)

11m, 2f 2007 m (6.8, 7.0 [2], 7.1, 7.2, 7.3, 7.4, 7.5, 7.6, 7.8, 7.9); f (6.6, 6.8)

*Castianeira longipalpa* (Hentz, 1847)

1m 2006 m (7.2)

3m 2007 m (6.9, 7.2 [2])

*Falconina gracilis* (Keyserling, 1891)

50m, 61f 2006 m (4.1, 4.3, 4.5, 4.9, 5.2 [4], 5.5 [2], 5.7 [2], 5.8 [2], 5.9 [2], 6.0 [5], 6.1 [2], 6.2, 6.3 [2], 6.4 [5], 6.5 [3], 6.6 [2], 6.7 [3], 6.8 [2], 6.9 [3], 7.0, 7.1, 7.2, 7.3 [2], 7.4); f (4.7, 4.9, 5.1 [2], 5.2, 5.3, 5.5 [2], 5.6, 5.8, 5.9 [3], 6.0 [2], 6.2, 6.3 [2], 6.4, 6.5 [3], 6.6 [2], 6.7 [2], 6.8 [2], 7.0 [3], 7.1 [2], 7.2 [2], 7.3 [3], 7.5 [4], 7.7 [2], 7.8 [3], 7.9, 8.0 [3], 8.1, 8.2, 8.3 [3], 8.4, 8.5 [2], 8.7, 9.1)

3m, 13f 2007 m (4.7, 5.5, 7.1); f (5.0, 5.1, 6.0, 6.1, 6.4 [2], 7.0 [2], 7.1, 7.3, 7.4, 7.9, 8.5)

**Dictynidae**

*Dictyna annexa* Gertsch & Mulaik, 1936

1m 2006 m (1.9)

1m 2007 m (1.9)

*Dictyna volucripes* Keyserling, 1881

1m 2007 m (1.2)

*Emblyna consulta* Keyserling, 1881

1m 2006 m (1.7)

*Lathys delicatula* (Gertsch & Mulaik, 1936)

1m 2007 m (1.1)

*Phantyna segregata* (Gertsch & Mulaik, 1936)

9m 2007 m (1.8 [3], 1.9 [2], 2.0, 2.1, 2.2 [2])

**Eutichuridae**

*Cheiracanthium inclusum* (Hentz, 1847)

1m 2007 m (6.1)

**Gnaphosidae**

*Callilepis gertschi* Platnick, 1975

2m 2006 m (2.8, 2.9)

*Camillina pulchra* (Keyserling, 1891)

1f 2006 f (3.5)

*Drassyllus antonito* Platnick & Shadab, 1982

5m 2006 m (2.1, 2.2, 2.3, 2.4 [2])

1m 2007 m (2.4)

*Drassyllus dixinus* Chamberlin, 1922

1m 2006 m (3.5)

1m, 1f 2007 m (3.7); f (3.5)

*Drassyllus inanus* Chamberlin & Gertsch, 1940

33m, 7f 2006 m (1.6, 1.9 [3], 2.0 [5], 2.1 [2], 2.2 [7], 2.3 [7], 2.4 [4], 2.5 [3], 2.9); f (2.0 [2], 2.1, 2.2, 2.3, 2.4, 2.5)

5m, 1f 2007 m (2.2 [2], 2.3, 2.4, 2.8); f (2.0)

*Drassyllus lepidus* (Banks, 1899)

8m, 11f 2006 m (3.3, 3.5, 3.6, 3.7 [2], 3.8, 4.0, 4.3); f (2.7, 2.8, 3.0, 3.7, 3.8, 4.3 [2], 4.4, 4.5 [2], 4.9)

11m, 15f 2007 m (2.8, 3.1, 3.2, 3.9 [2], 4.0 [3], 4.2 [2], 4.6); f (3.6, 3.7, 4.2 [2], 4.3 [2], 4.5, 4.6 [2], 4.7, 4.8, 4.9 [2], 5.1, 5.2)

*Drassyllus notonus* Chamberlin, 1928

1m, 1f 2006 m (2.4); f (4.4)

*Drassyllus texamans* Chamberlin, 1936

1f 2006 f (3.6)

6m, 2f 2007 m (3.5 [2], 3.7 [2], 3.8, 4.0); f (3.5, 4.1)

*Gnaphosa altudona* Chamberlin, 1922

7m, 1f 2006 m (3.6, 3.8, 3.9, 4.0, 4.1, 4.2, 4.4); f (3.7)

1m, 2f 2007 m (4.3); f (3.8, 4.4)

*Gnaphosa fontinalis* Keyserling, 1887

1f 2006 f (8.7)

*Gnaphosa sericata* (L. Koch, 1866)

70m, 16f 2006 m (4.3, 4.4, 4.6, 4.7 [3], 4.8, 4.9, 5.0 [4], 5.1 [5], 5.2 [4], 5.3 [8], 5.4 [8], 5.5 [5], 5.6 [6], 5.7 [5], 5.8 [7], 5.9 [3], 6.0 [3], 6.1 [2], 6.2, 6.4); f (4.1, 4.6, 5.1, 5.3, 5.5, 5.6, 5.8, 6.4 [3], 6.6, 6.7, 7.0, 7.1, 7.5, 9.6)

23m, 6f 2007 m (4.4, 4.6, 4.7, 4.8, 4.9, 5.0, 5.1 [2], 5.2 [2], 5.3, 5.4 [3], 5.5, 5.6 [2], 5.7, 5.8, 5.9 [4]); f (5.1, 6.1, 6.3, 6.4, 6.5, 7.0)

*Haplodrassus signifer* (C. L. Koch, 1839)

3m 2006 m (5.6, 6.3, 7.1)

*Micaria deserticola* Gertsch, 1933

3m 2006 m (4.6, 4.8, 4.9)

12m, 2f 2007 m (2.0, 2.2 [3], 2.3 [2], 2.4 [3], 2.5 [3]); f (2.1, 2.8)

*Micaria nanella* Gertsch, 1935

50m, 25f 2006 m (1.5, 1.6, 1.7, 1.8 [7], 1.9 [12], 2.0 [10], 2.1 [13], 2.2 [3], 2.3, 2.5); f (2.0 [3], 2.1 [2], 2.2 [3], 2.3, 2.4 [7], 2.5 [2], 2.6 [4], 2.7 [3])

37m, 7f 2007 m (1.8 [2], 1.9 [6], 2.0 [5], 2.1 [9], 2.2 [6], 2.3 [4], 2.4 [4], 3.5); f (2.1 [2], 2.4 [2], 2.5 [2], 2.6)

*Micaria nye* Platnick & Shadab, 1988

14m, 7f 2006 m (1.8, 1.9 [2], 2.1 [3], 2.2, 2.3 [4], 2.4, 2.5 [2]); f (2.0, 2.4, 2.5, 2.7 [2], 2.9 [2])

3m 2007 m (2.1, 2.3, 2.4)

*Talanites captiosus* (Gertsch & Davis, 1936)

12m, 7f 2007 m (3.1, 3.5 [2], 3.6 [2], 3.7 [3], 3.8, 3.9, 4.3, 4.5); f (3.7, 4.0, 4.1, 4.2, 4.4, 4.5, 4.8)

*Talanites exlineae* (Platnick & Shadab, 1976)

1m 2006 m (4.4)

*Zelotes aiken* Platnick & Shadab, 1983

5m 2006 m (4.5, 5.1 [2], 5.2, 6.0)

7m, 1f 2007 m (3.8, 4.8, 5.3, 5.6, 5.7, 5.9, 6.5); f (4.9)

*Zelotes anglo* Gertsch & Riechert, 1976

1m, 1f 2006 m (8.1); f (8.4)

1m 2007 m (5.0)

*Zelotes gertschi* Platnick & Shadab, 1983

18m, 8f 2006 m (3.9, 4.1, 4.5, 4.9 [3], 5.2, 5.3 [2], 5.5 [2], 5.6, 5.8 [2], 6.0, 6.3, 7.1, 7.2); f (4.8, 5.0, 5.7, 5.9, 6.1, 6.2, 7.0, 9.2)

7m, 4f 2007 m (5.1, 5.4, 5.5 [2], 5.7 [3]); f (5.7, 5.8 [2], 5.9)

*Zelotes hentzi* Barrows, 1945

1f 2007 f (7.5)

*Zelotes lasalanus* Chamberlin, 1928

2f 2007 f (5.7, 6.3)

*Zelotes lymnophilus* Chamberlin, 1936

11m 2006 m (3.1 [2], 3.2 [2], 3.6 [2], 3.7, 3.9 [2], 4.0, 4.1)

6m 2007 m (3.2, 3.5 [2], 3.7 [3])

*Zelotes pseustes* Chamberlin, 1922

3m 2006 m (6.3, 6.5, 7.2)

1m 2007 m (6.0)

**Hahniidae**

*Hahnia cinerea* Emerton, 1890

3f 2006 f (1.8, 1.9, 2.1)

1m, 7f 2007 m (1.6); f (1.9 [2], 2.2 [3], 2.3 [2])

*Hahnia flaviceps* Emerton, 1913

2f 2006 f (1.8, 1.9)

*Neoantistea agilis* (Keyserling, 1887)

3m 2006 m (2.3, 2.5, 2.9)

1m 2007 m (3.7)

*Neoantistea oklahomensis* Opell & Beatty, 1976

1m, 1f 2007 m (2.8); f (3.6)

**Linyphiidae**

*Agyneta chiricahua* Dupérré, 2013

12m, 6f 2006 m (1.2, 1.3 [2], 1.4 [6], 1.6 [3]); f (1.1, 1.2, 1.4, 1.5, 1.6, 1.7)

23m, 29f 2007 m (1.4 [5], 1.5 [7], 1.6 [2], 1.7 [5], 1.8 [4]); f (1.1, 1.2 [2], 1.3 [2], 1.4 [3], 1.5 [4], 1.6 [4], 1.7 [4], 1.8 [3], 1.9 [3], 2.0, 2.3, 2.6)

*Agyneta crista* Dupérré, 2013

9m, 3f 2006 m (1.3, 1.5 [6], 1.6 [2]); f (1.5, 1.6 [2])

64m, 40f 2007 m (1.2, 1.4 [3], 1.5 [6], 1.6 [14], 1.7 [18], 1.8 [7], 1.9 [13], 2.0 [2]); f (1.0 [2], 1.1 [5], 1.2 [12], 1.3 [3], 1.4 [7], 1.5 [5], 1.6 [3], 1.8 [2], 2.0)

*Agyneta flax* Dupérré, 2013

1f 2007 f (1.8)

*Agyneta micaria* (Emerton, 1882)

1f 2006 f (1.3)

*Agyneta parva* (Banks, 1898)

1f 2006 f (1.7)

1f 2007 f (2.2)

*Agyneta regina* (Chamberlin & Ivie, 1944)

1f 2007 f (1.8)

*Agyneta sandia* Dupérré, 2013

1f 2006 f (1.5)

*Agyneta serrata* (Emerton, 1909)

7m, 3f 2006 m (1.2, 1.3 [4], 1.4 [2]); f (1.2, 1.4 [2])

61m 2007 m (1.0, 1.2 [8], 1.3 [12], 1.4 [14], 1.5 [10], 1.6 [9], 1.7 [6], 1.8)

*Ceraticelus laetus* (O. P.-Cambridge, 1874)

1m 2007 m (1.6)

*Ceratinella brunnea* Emerton, 1882

5m, 2f 2007 m (1.2, 1.3 [2], 1.4, 1.5); f (1.5, 1.6)

*Ceratinops crenatus* (Emerton, 1882)

5m, 6f 2006 m (1.7, 1.8, 2.0 [2], 2.1); f (1.9 [2], 2.0 [2], 2.1 [2])

269m, 82f 2007 m (0.9, 1.5, 1.6 [5], 1.7 [6], 1.8 [22], 1.9 [86], 2.0 [83], 2.1 [56], 2.2 [7], 2.3, 2.8); f (1.5, 1.6 [2], 1.8 [8], 1.9 [12], 2.0 [16], 2.1 [24], 2.2 [12], 2.3 [5], 2.4, 2.5)

*Ceratinops latus* (Emerton, 1882)

1m 2007 m (1.5)

*Erigone autumnalis* Emerton, 1882

13m, 9f 2006 m (1.0, 1.1, 1.2 [3], 1.4 [5], 1.5, 1.6 [2]); f (1.0, 1.1 [5], 1.3, 1.4, 1.5)

128m, 31f 2007 m (1.0 [2], 1.1 [5], 1.2 [16], 1.3 [30], 1.4 [21], 1.5 [26], 1.6 [16], 1.7 [11], 1.8); f (0.9 [2], 1.0 [7], 1.1 [8], 1.2 [9], 1.3 [3], 1.4, 2.0)

*Tennesseellum formicum* (Emerton, 1882)

136m, 10f 2006 m (1.4 [3], 1.5 [8], 1.6 [33], 1.7 [34], 1.8 [39], 1.9 [15], 2.0 [4]); f (1.4, 1.6 [6], 1.7, 1.8, 2.0)

458m, 22f 2007 m (1.1, 1.3, 1.4 [14], 1.5 [14], 1.6 [47], 1.7 [54], 1.8 [66], 1.9 [90], 2.0 [85], 2.1 [52], 2.2 [24], 2.3 [9], 2.5); f (1.5 [2], 1.6 [3], 1.7 [6], 1.8 [2], 1.9 [2], 2.0 [2], 2.1 [3], 2.2 [2])

*Walckenaeria puella* Millidge, 1983

2f 2006 f (1.6 [2])

4f 2007 f (1.5, 1.6, 2.1, 2.2)

**Lycosidae**

*Allocosa funerea* (Hentz, 1844)

1f 2006 f (4.9)

*Hogna antelucana* (Montgomery, 1904)

1m 2006 m (12.5)

1f 2007 f (22.8)

*Hogna frondicola* Emerton, 1885

1f 2006 f (9.9)

*Pardosa mercurialis* Montgomery, 1904

1f 2006 f (8.4)

*Pardosa milvina* (Hentz, 1844)

1m 2007 m (4.4)

*Pardosa pauxilla* Montgomery, 1904

1m 2006 m (4.7)

10m, 7f 2007 m (3.8, 4.2 [2], 4.3 [2], 4.4, 4.5 [2], 4.7, 4.9); f (4.8, 5.0 [2], 5.3, 5.9, 6.2, 6.8)

*Pirata hiteorum* Wallace & Exline, 1978

1m 2006 m (2.9)

1f 2007 f (3.1)

*Rabidosa punctulata* (Hentz, 1844)

1f 2006 f (14.0)

*Schizocosa avida* (Walckenaer, 1837)

8m, 2f 2006 m (6.4, 6.8, 7.2, 7.8, 8.2, 8.7, 9.8, 10.4); f (8.5, 11.2)

39m, 31f 2007 m (6.2, 6.3, 6.5, 6.6 [2], 6.9, 7.1, 7.4, 7.5 [3], 7.6, 7.7, 7.9 [2], 8.0 [2], 8.1 [2], 8.5, 8.6, 8.9, 9.0, 9.1, 9.2 [4], 9.5 [2], 9.6, 9.7, 9.9, 10.0, 10.1 [2], 10.5, 10.6, 15.5); f (7.6, 7.9, 8.0 [2], 8.3, 8.5 [2], 8.6, 8.9, 9.1, 9.6, 9.9 [2], 10.0 [2], 10.1, 10.3, 10.4, 10.5, 10.7 [3], 11.1, 11.6, 12.3, 12.4, 13.1, 13.7, 14.6, 15.0, 17.0)

*Schizocosa bilineata* (Emerton, 1885)

1f 2007 f (12.3)

*Schizocosa saltatrix* (Hentz, 1844)

6m, 4f 2006 m (7.4, 7.7, 7.9, 8.4, 8.6, 8.8); f (7.8, 8.4, 11.3, 11.7)

9m, 3f 2007 m (7.3, 7.8, 8.1, 8.6, 8.8, 9.0, 9.4, 9.8, 10.3); f (10.2, 11.8, 12.2)

*Trochosa sepulchralis* (Montgomery, 1902)

1m 2007 m (9.4)

*Varacosa avara* (Keyserling, 1877)

1f 2006 f (9.0)

*Varacosa shenandoa* (Chamberlin & Ivie, 1942)

1f 2006 f (10.0)

3f 2007 f (9.3, 11.0, 12.1)

**Mimetidae**

*Mimetus hesperus* Chamberlin, 1923

1f 2007 f (5.2)

**Miturgidae**

*Teminius affinis* Banks, 1897

2m, 1f 2006 m (9.1, 9.7); f (15.9)

4m, 4f 2007 m (8.6, 9.2, 9.5, 12.0); f (11.3, 13.2, 13.6, 15.6)

**Mysmenidae**

*Mysmena incredula* (Gertsch & Davis, 1936)

1m, 1f 2007 m (0.6); f (0.9)

**Oxyopidae**

*Oxyopes apollo* Brady, 1964

992m, 156f 2006 m (3.4, 3.5 [2], 3.6 [9], 3.7 [13], 3.8 [20], 3.9 [39], 4.0 [104], 4.1 [97], 4.2 [145], 4.3 [148], 4.4 [128], 4.5 [102], 4.6 [73], 4.7 [48], 4.8 [26], 4.9 [23], 5.0 [8], 5.1 [3], 5.2 [3]); f (3.7, 4.2, 4.3 [2], 4.6 [4], 4.7 [5], 4.8 [4], 4.9 [9], 5.0 [14], 5.1 [12], 5.2 [10], 5.3 [16], 5.4 [8], 5.5 [6], 5.6 [9], 5.7 [12], 5.8 [11], 5.9 [6], 6.0 [5], 6.1 [11], 6.2 [2], 6.3 [2], 6.4 [3], 6.5, 6.6, 6.9)

681m, 153f 2007 m (3.3, 3.4, 3.5 [2], 3.6 [4], 3.7 [8], 3.8 [18], 3.9 [24], 4.0 [67], 4.1 [66], 4.2 [74], 4.3 [81], 4.4 [105], 4.5 [82], 4.6 [60], 4.7 [37], 4.8 [21], 4.9 [12], 5.0 [13], 5.1 [5]); f (4.5, 4.6 [5], 4.7, 4.8 [7], 4.9 [3], 5.0 [8], 5.1 [6], 5.2 [6], 5.3 [11], 5.4 [6], 5.5 [11], 5.6 [8], 5.7 [5], 5.8 [19], 5.9 [12], 6.0 [15], 6.1 [8], 6.2 [10], 6.3, 6.4 [4], 6.5 [2], 6.6 [3], 6.8)

*Oxyopes salticus* Hentz, 1845

32m, 29f 2006 m (3.0, 3.4, 3.5, 3.6 [4], 3.7 [4], 3.8, 3.9 [8], 4.0 [2], 4.1, 4.2, 4.4 [2], 4.5 [2], 4.6, 5.1 [2], 5.3); f (3.6, 3.9, 4.0, 4.2, 4.3, 4.4 [4], 4.5 [2], 4.6 [3], 4.7 [3], 4.9, 5.0, 5.1, 5.2 [2], 5.3 [3], 5.4, 5.5 [2], 7.0])

13m, 32f 2007 m (4.2 [2], 4.4, 4.8, 4.9, 5.0 [2], 5.1, 5.2, 5.3 [3], 5.7); f (5.0, 5.1 [3], 5.3, 5.5 [3], 5.6 [2], 5.7 [2], 5.8 [4], 5.9, 6.0 [2], 6.2, 6.3 [4], 6.4, 6.5, 6.6, 6.7 [3], 6.9, 7.8)

**Philodromidae**

*Philodromus keyserlingi* Marx, 1890

1m 2006 m (4.5)

*Thanatus formicinus* (Clerck, 1757)

1m 2006 m (4.1)

*Thanatus rubicellus* Mello-Leitão, 1929

1m 2006 m (5.0)

**Phrurolithidae**

*Phrurotimpus alarius* (Hentz, 1847)

2m 2007 m (2.0, 2.1)

*Phrurotimpus certus* Gertsch, 1941

2m, 26f 2006 m (1.9, 2.0); f (2.0 [2], 2.1 [3], 2.2 [4], 2.3 [4], 2.4 [3], 2.5 [2], 2.6 [6], 2.7 [2])

6m, 26f 2007 m (1.8 [2], 1.9 [2], 2.0 [2]); f (2.1 [2], 2.2 [4], 2.3 [9], 2.4 [5], 2.6 [2], 2.7, 2.8 [3])

*Scotinella fratrella* (Gertsch, 1935)

21m, 13f 2006 m (1.3 [6], 1.4 [5], 1.5 [5], 1.6 [3], 1.7 [2]); f (1.3, 1.5 [4], 1.6, 1.8 [4], 1.9 [2], 2.2)

8m, 2f 2007 m (1.5 [4], 1.6 [2], 1.7 [2]); f (1.7, 1.9)

**Salticidae**

*Habronattus calcaratus* (Banks, 1904)

28m 2006 m (4.4, 4.5, 4.6 [2], 4.7 [3], 4.8 [3], 4.9 [3], 5.0 [3], 5.1 [6], 5.2 [2], 5.3, 5.5, 5.6 [2])

39m 2007 m (4.4, 4.5 [2], 4.6 [2], 4.7 [2], 4.8 [5], 4.9 [4], 5.0 [7], 5.1 [5], 5.2 [2], 5.3 [3], 5.4, 5.5 [3], 5.6 [2])

*Habronattus coecatus* (Hentz, 1846)

32m, 12f 2006 m (3.8 [2], 4.1, 4.2, 4.3 [2], 4.5, 4.6 [9], 4.7 [3], 4.8, 4.9, 5.0 [4], 5.1 [4], 5.3, 5.4, 5.6); f (5.5, 5.6, 6.2, 6.4, 6.8 [3], 6.9, 7.0, 7.2, 7.5 [2])

22m, 20f 2007 m (4.3, 4.4 [2], 4.6 [2], 4.7, 4.8 [4], 4.9 [4], 5.0 [2], 5.1 [2], 5.2, 5.4, 5.5 [2]); f (5.6, 5.7, 6.0 [2], 6.2 [2], 6.5, 6.8, 6.9, 7.1, 7.4, 7.5, 7.8 [2], 8.0, 8.1, 8.4, 8.5 [2], 8.6)

*Habronattus cognatus* (Peckham & Peckham, 1901)

63m, 29f 2006 m (4.4, 4.5 [3], 4.6 [6], 4.7 [4], 4.8 [2], 4.9 [4], 5.0 [12], 5.1 [8], 5.2 [6], 5.3 [4], 5.4 [7], 5.5 [2], 5.6 [4]); f (5.0, 5.1, 5.4 [2], 5.5 [5], 5.6 [3], 5.7 [2], 5.8 [2], 6.0, 6.2 [2], 6.3 [2], 6.4 [2], 6.6, 6.8, 7.1, 7.4, 7.6, 7.8)

33m, 13f 2007 m (4.4, 4.5 [2], 4.6 [2], 4.7 [3], 4.9, 5.0 [6], 5.1 [2], 5.2 [6], 5.3 [3], 5.4, 5.6 [2], 5.7, 5.8 [2], 5.9); f (5.5, 5.7, 6.0, 6.2 [2], 6.4 [2], 6.5, 6.8, 7.0, 7.1, 7.2, 7.4)

*Habronattus decorus* (Blackwall, 1846)

1m 2006 m (4.6)

2f 2007 f (5.2, 8.3)

*Habronattus fallax* (Peckham & Peckham, 1909)

3m 2006 m (5.0 [2], 5.1)

5m 2007 m (4.4, 4.7, 4.8, 4.9, 5.1)

*Habronattus orbus* Griswold, 1987

1f 2006 f (5.6)

*Habronattus texanus* (Chamberlin, 1924)

52m, 20f 2006 m (3.2, 3.4 [2], 3.5, 3.6 [2], 3.7 [5], 3.8 [10], 3.9 [6], 4.0 [3], 4.1 [6], 4.2 [4], 4.4 [4], 4.5 [2], 4.6 [4], 4.7, 5.2); f (4.5, 4.7 [2], 4.9, 5.1, 5.3 [2], 5.4, 5.5 [3], 5.9, 6.0, 6.1 [4], 6.2, 6.4 [2])

30m, 11f 2007 m (3.6 [3], 3.7 [2], 3.8 [4], 4.0, 4.1 [2], 4.2 [8], 4.3 [2], 4.4 [3], 4.5 [4], 4.6); f (5.1, 5.3, 5.4 [2], 5.5 [3], 5.6, 5.7, 6.7, 7.4)

*Habronattus viridipes* (Hentz, 1846)

12f 2006 f (5.2 [2], 5.4, 5.6, 5.8 [2], 5.9 [2], 6.0, 6.1, 6.2, 6.8)

8f 2007 f (5.6, 5.8, 5.9, 6.2, 6.3, 6.5, 6.9, 7.0)

*Marpissa lineata* (C. L. Koch, 1846)

1m 2007 m (4.2)

*Metacyrba taeniola taeniola* (Hentz, 1846)

1f 2006 f (5.4)

1m 2007 m (5.6)

*Neonella vinnula* Gertsch, 1936

1m 2007 m (1.4)

*Pellenes limatus* Peckham & Peckham, 1901

3m, 1f 2006 m (5.2, 5.3, 5.8); f (7.6)

4m, 1f 2007 m (4.5, 5.6, 5.9, 6.6); f (6.7)

*Phidippus cardinalis* (Hentz, 1845)

3m 2007 m (11.1, 11.8, 12.6)

*Phidippus clarus* Keyserling, 1885

1f 2007 f (12.7)

*Phidippus texanus* Banks, 1906

2m 2006 m (10.9, 11.8)

1m, 1f 2007 m (14.4); f (16.5)

*Phlegra hentzi* (Marx, 1890)

1m 2006 m (6.6)

4m 2007 m (5.9, 6.3 [2], 6.7)

*Sarinda hentzi* (Banks, 1913)

1m, 1f 2007 m (6.5); f (7.0)

*Sitticus dorsatus* (Banks, 1895)

1m 2006 m (2.9)

1m, 1f 2007 m (2.6); f (3.0)

*Synageles noxiosus* (Hentz, 1850)

1m, 1f 2006 m (3.1); f (3.0)

1m 2007 m (2.8)

*Talavera minuta* (Banks, 1895)

1m 2006 m (2.2)

2m 2007 m (2.3, 2.5)

*Zygoballus rufipes* Peckham & Peckham, 1885

1m 2006 m (4.1)

1m 2007 m (3.6)

**Scytodidae**

*Scytodes atlacoya* Rheims, Brescovit & Durán, 2007

1m 2006 m (7.0)

**Tetragnathidae**

*Glenognatha foxi* (McCook, 1894)

141m, 121f 2007 m (1.3, 1.4, 1.5 [5], 1.6 [9], 1.7 [23], 1.8 [41], 1.9 [36], 2.0 [19], 2.1 [6]); f (1.5, 1.6 [5], 1.7 [6], 1.8 [18], 1.9 [13], 2.0 [23], 2.1 [22], 2.2 [11], 2.3 [9], 2.4 [9], 2.5 [2], 2.6 [2])

**Theridiidae**

*Hentziectypus schullei* (Gertsch & Mulaik, 1936)

1m 2007 m (1.0)

*Latrodectus mactans* (Fabricius, 1775)

1m 2006 m (4.5)

*Theridion australe* Banks, 1899

1m 2006 m (2.4)

*Theridion cinctipes* Banks, 1898

2m 2006 m (1.5 [2])

3m 2007 m (1.3, 1.4, 1.5)

*Theridion dilutum* Levi, 1957

1m 2006 m (2.0)

*Theridion dividuum* Gertsch & Archer, 1942

4m 2007 m (1.1, 1.2, 1.3, 1.4)

*Theridion llano* Levi, 1957

1m 2006 m (1.6)

1f 2007 f (1.6)

*Theridion murarium* Emerton, 1882

1m 2007 m (1.3)

*Theridion rabuni* Chamberlin & Ivie, 1944

2m, 1f 2006 m (1.6, 1.8); f (2.1)

**Thomisidae**

*Mecaphesa celer* (Hentz, 1847)

1m, 1f 2006 m (3.3); f (6.2)

*Xysticus apachecus* Gertsch, 1933

1m 2006 m (4.0)

*Xysticus auctificus* Keyserling, 1880

4m 2007 m (3.8, 4.5 [2], 5.2)

*Xysticus ferox* (Hentz, 1847)

1m 2006 m (5.5)

2m 2007 m (5.0, 5.6)

*Xysticus funestus* Keyserling, 1880

2f 2006 f (6.5, 8.0)

1m 2007 m (5.6)

*Xysticus pellax* O. P.-Cambridge, 1894

4m 2006 m (4.4, 4.5, 4.6, 4.9)

10m, 3f 2007 m (3.9, 4.4, 4.6, 5.0, 5.1, 5.2, 5.3 [2], 6.0 [2]); f (6.5 [2], 7.5)

*Xysticus robinsoni* Gertsch, 1953

2m 2006 m (4.7, 5.2)

*Xysticus texanus* Banks, 1904

3m 2007 m (5.4, 5.6, 6.3)

**Titanoecidae**

*Titanoeca americana* Emerton, 1888

3m, 1f 2006 m (6.2, 6.5, 6.6); f (5.3)

7m 2007 m (5.3, 5.4, 6.0 [3], 6.2, 6.7)

**Trachelidae**

*Meriola decepta* Banks, 1895

6m, 16f 2006 m (3.4, 3.7, 4.3, 4.4, 4.5, 4.9); f (3.6, 3.7 [2], 3.8, 3.9 [2], 4.1, 4.3, 4.4, 4.5, 4.6, 4.7 [3], 4.8, 5.0)

1m, 1f 2007 m (2.7); f (3.7)

#### Pruitt, Coryell Co.

**Euctenizidae**

*Myrmekiaphila comstocki* Bishop & Crosby, 1926

1m 2007 m (15.0)

**Anyphaenidae**

*Hibana futilis* (Banks, 1898)

1m 2006 m (9.7)

*Hibana gracilis* (Hentz, 1847)

1m 2006 m (8.5)

**Corinnidae**

*Castianeira alteranda* Gertsch, 1942

2f 2007 f (9.1, 9.8)

*Castianeira crocata* (Hentz, 1847)

2f 2006 f (9.3, 9.9)

*Castianeira descripta* (Hentz, 1847)

8m, 1f 2006 m (5.9, 6.2, 6.3, 6.5, 6.9, 7.0, 7.2, 7.4); f (7.4)

5m, 1f 2007 m (6.6, 7.4, 7.9, 8.0, 8.4); f (9.5)

*Castianeira longipalpa* (Hentz, 1847)

1m 2006 m (6.8)

3m 2007 m (6.3, 7.1, 7.4)

*Castianeira trilineata* (Hentz, 1847)

1m 2006 m (6.6)

1m 2007 m (6.3)

*Falconina gracilis* (Keyserling, 1891)

170m, 220f 2006 m (4.3 [2], 4.5 [3], 4.6 [3], 4.7, 4.8 [3], 5.0, 5.1 [2], 5.2 [6], 5.3 [4], 5.4 [2], 5.5 [9], 5.6 [7], 5.7 [10], 5.8 [9], 5.9 [13], 6.0 [18], 6.1 [8], 6.2 [6], 6.3 [11], 6.4 [7], 6.5 [14], 6.6 [3], 6.7 [4], 6.8 [10], 6.9 [4], 7.0 [3], 7.2 [2], 7.3, 7.4 [2], 7.6, 8.1); f (2.9, 3.6, 4.3, 4.4, 4.6 [4], 4.8 [2], 4.9 [5], 5.0 [3], 5.2 [2], 5.3 [7], 5.4 [5], 5.5 [4], 5.6 [5], 5.7 [8], 5.8 [10], 5.9 [9], 6.0 [14], 6.1 [4], 6.2 [6], 6.3 [10], 6.4 [8], 6.5 [7], 6.6 [4], 6.7 [6], 6.8 [10], 6.9 [8], 7.0 [13], 7.1 [8], 7.2 [11], 7.3 [5], 7.4 [4], 7.5 [5], 7.6 [3], 7.7 [3], 7.8 [2], 7.9 [2], 8.0 [4], 8.2 [2], 8.3 [4], 8.4, 8.5 [3], 8.7, 8.8, 9.0, 9.2, 9.4)

3m, 9f 2007 m (4.8, 6.6, 7.1); f (5.8, 5.9, 6.0 [2], 6.2, 6.3, 6.4, 7.5, 7.8)

**Dictynidae**

*Phantyna segregata* (Gertsch & Mulaik, 1936)

1f 2006 f (2.1)

29m, 5f 2007 m (1.9 [4], 2.0 [4], 2.1 [5], 2.2 [5], 2.3 [2], 2.4 [3], 2.5 [3], 2.6, 2.7, 3.0); f (2.2 [3], 2.4, 2.5)

**Gnaphosidae**

*Camillina pulchra* (Keyserling, 1891)

1m, 1f 2006 m (2.7); f (3.0)

*Cesonia bilineata* (Hentz, 1847)

3m 2006 m (4.9, 5.4, 5.5)

3m 2007 m (5.1, 5.3, 5.8)

*Drassyllus antonito* Platnick & Shadab, 1982

2m 2006 m (2.1, 2.2)

2m 2007 m (1.9, 2.4)

*Drassyllus aprilinus* (Banks, 1904)

2m 2006 m (4.1, 4.4)

2m 2007 m (4.1, 4.2)

*Drassyllus inanus* Chamberlin & Gertsch, 1940

66m, 34f 2006 m (1.6 [2], 1.7, 1.8 [3], 1.9 [6], 2.0 [9], 2.1 [12], 2.2 [12], 2.3 [9], 2.4 [7], 2.5 [4], 2.6); f (1.7, 1.8 [2], 1.9, 2.0 [2], 2.1 [7], 2.2 [8], 2.3 [5], 2.4 [5], 2.5, 2.8, 3.0)

18m, 5f 2007 m (1.7, 2.0 [2], 2.1, 2.2 [3], 2.3 [2], 2.4 [2], 2.5 [5], 2.6 [2]); f (2.5, 2.6 [2], 2.7, 2.8)

*Drassyllus lepidus* (Banks, 1899)

10m, 6f 2006 m (2.7, 3.0, 3.1, 3.3, 3.5, 3.6 [2], 3.7, 4.0, 4.4); f (3.3, 3.5 [2], 3.7, 3.8, 4.0)

6m, 2f 2007 m (3.1, 3.9, 4.0 [2], 4.1, 4.2); f (5.3 [2])

*Drassyllus notonus* Chamberlin, 1928

4f 2006 f (2.2 [2], 2.4, 2.5)

1f 2007 f (2.9)

*Drassyllus texamans* Chamberlin, 1936

2f 2006 f (3.2, 3.6)

1f 2007 f (3.1)

*Gnaphosa altudona* Chamberlin, 1922

13m, 1f 2006 m (2.8, 3.6, 3.7, 3.8 [2], 3.9 [2], 4.0 [2], 4.1, 4.2, 4.3 [2]); f (4.8)

2m 2007 m (3.9, 4.4)

*Gnaphosa fontinalis* Keyserling, 1887

1m 2007 m (7.5)

*Gnaphosa sericata* (L. Koch, 1866)

1f 2006 f (6.6)

1f 2007 f (5.7)

*Micaria deserticola* Gertsch, 1933

2m 2006 m (3.5, 4.2)

3m 2007 m (2.0, 2.2, 3.7)

*Micaria longipes* Emerton, 1890

1f 2007 f (4.9)

*Micaria nanella* Gertsch, 1935

34m, 11f 2006 m (1.6 [2], 1.7 [4], 1.8 [8], 1.9 [6], 2.0 [9], 2.1 [2], 2.2 [2], 2.3); f (1.9, 2.0, 2.1 [3], 2.2, 2.3 [3], 2.4, 2.7)

22m, 7f 2007 m (1.7, 1.8, 1.9, 2.0 [6], 2.1 [4], 2.2 [4], 2.3 [3], 2.5 [2]); f (1.9, 2.4 [4], 2.5, 2.6)

*Talanites captiosus* (Gertsch & Davis, 1936)

2m 2007 m (3.2, 3.4)

*Zelotes aiken* Platnick & Shadab, 1983

9m, 9f 2006 m (4.9, 5.2, 5.3, 5.5, 5.6 [2], 6.2, 6.3 [2]); f (4.4, 4.8 [2], 5.0, 5.5 [2], 5.8, 5.9, 6.2)

10m, 1f 2007 m (4.4, 4.6 [3], 4.7, 4.9, 5.0, 5.6 [2], 6.0); f (5.2)

*Zelotes anglo* Gertsch & Riechert, 1976

4m, 2f 2006 m (6.0, 7.8, 8.0, 8.2); f (7.1, 9.7)

2m, 2f 2007 m (8.2, 9.1); f (7.5, 8.5)

*Zelotes gertschi* Platnick & Shadab, 1983

10m, 7f 2006 m (3.9, 4.3 [2], 4.4, 4.5, 5.0, 5.3, 5.7 [2], 5.8); f (4.0, 4.3, 4.6, 4.8, 5.1, 5.4, 5.8)

5m, 2f 2007 m (4.2, 4.9, 5.0, 5.1, 5.8); f (6.1 [2])

*Zelotes pseustes* Chamberlin, 1922

1f 2007 f (5.0)

*Zelotes tuobus* Chamberlin, 1919

2m, 2f 2006 m (7.3, 8.0); f (5.0, 8.0)

**Hahniidae**

*Hahnia cinerea* Emerton, 1890

4m, 50f 2006 m (1.6, 1.7 [3]); f (1.7 [3], 1.8 [10], 1.9 [13], 2.0 [10], 2.1 [11], 2.2 [2], 2.3)

2m, 15f 2007 m (1.7, 1.9); f (1.9 [2], 2.0 [4], 2.1 [5], 2.2 [3], 2.4)

*Hahnia flaviceps* Emerton, 1913

1f 2006 f (1.9)

1m, 1f 2007 m (1.5); f (1.6)

*Neoantistea agilis* (Keyserling, 1887)

1m 2006 m (2.9)

*Neoantistea oklahomensis* Opell & Beatty, 1976

1f 2006 f (3.3)

**Linyphiidae**

*Agyneta chiricahua* Dupérré, 2013

10m 2006 m (1.2 [5], 1.3 [2], 1.4 [3])

76m, 37f 2007 m (1.2 [5], 1.3 [10], 1.4 [25], 1.5 [18], 1.6 [5], 1.7 [7], 1.8 [3], 1.9 [3]); f (1.2 [3], 1.3 [5], 1.4 [9], 1.5 [11], 1.6 [7], 1.8, 1.9)

*Agyneta crista* Dupérré, 2013

10m, 1f 2006 m (1.3 [2], 1.4 [3], 1.5, 1.6 [3], 1.9); f (1.2])

65m, 24f 2007 m (1.4 [3], 1.5 [5], 1.6 [20], 1.7 [9], 1.8 [14], 1.9 [11], 2.0 [2], 2.1); f (1.1 [3], 1.2 [3], 1.3 [3], 1.4 [9], 1.5 [3], 1.7, 1.8 [2])

*Agyneta flax* Dupérré, 2013

1m, 1f 2007 m (1.8); (f 1.8)

*Agyneta micaria* (Emerton, 1882)

1m 2007 m (1.7)

*Agyneta regina* (Chamberlin & Ivie, 1944)

1f 2006 f (1.3)

9f 2007 f (1.3, 1.4 [2], 1.5, 1.6, 1.8, 1.9 [3])

*Agyneta serrata* (Emerton, 1909)

3m 2006 m (1.2, 1.3, 1.4)

31m, 1f 2007 m (1.0, 1.2 [7], 1.3 [10], 1.4 [11], 1.5 [2]); f (1.1])

*Ceratinops crenatus* (Emerton, 1882)

38m, 9f 2007 m (1.4, 1.8 [5], 1.9 [10], 2.0 [12], 2.1 [9], 2.3); f (1.8, 1.9, 2.0 [3], 2.1 [2], 2.2, 2.4)

*Ceratinops latus* (Emerton, 1882)

3m, 1f 2007 m (1.4, 1.5 [2]); f (1.5)

*Erigone autumnalis* Emerton, 1882

1m 2006 m (1.2)

177m, 27f 2007 m (1.0, 1.1 [10], 1.2 [19], 1.3 [28], 1.4 [40], 1.5 [43], 1.6 [26], 1.7 [9], 1.9); f (0.8, 0.9, 1.0 [4], 1.1 [4], 1.2 [10], 1.3 [6], 1.4)

*Mermessus denticulatus* (Banks, 1898)

1f 2007 f (2.8)

*Tennesseellum formicum* (Emerton, 1882)

53m, 1f 2006 m (1.4 [4], 1.5 [11], 1.6 [10], 1.7 [8], 1.8 [9], 1.9 [8], 2.0 [2], 2.1); f (2.0)

516m, 6f 2007 m (1.4 [5], 1.5 [14], 1.6 [29], 1.7 [58], 1.8 [73], 1.9 [102], 2.0 [99], 2.1 [61], 2.2 [57], 2.3 [15], 2.4 [2], 2.5); f (1.4 [2], 1.6, 1.7 [2], 1.8)

*Walckenaeria puella* Millidge, 1983

3f 2006 f (1.2, 1.4, 1.8)

**Lycosidae**

*Hogna antelucana* (Montgomery, 1904)

2f 2007 f (18.1, 18.6)

*Pardosa delicatula* Gertsch & Wallace, 1935

1m 2006 m (4.0)

*Pardosa milvina* (Hentz, 1844)

1m 2007 m (4.8)

*Pardosa pauxilla* Montgomery, 1904

6m, 2f 2006 m (3.7, 4.0 [2], 4.1, 4.3 [2]); f (4.0 [2])

63m, 22f 2007 m (3.9 [4], 4.0, 4.1 [2], 4.2 [3], 4.3 [3], 4.4 [11], 4.5 [6], 4.6 [2], 4.7 [5], 4.8 [6], 4.9 [4], 5.0 [9], 5.1 [3], 5.2, 5.3 [3]); f (4.6, 4.8, 5.2 [3], 5.3, 5.4, 5.5, 5.6, 5.7 [3], 5.9 [2], 6.0 [2], 6.1 [4], 6.3, 7.2)

*Schizocosa avida* (Walckenaer, 1837)

18m, 20f 2006 m (5.2, 7.5, 7.6 [2], 8.0 [2], 8.2, 8.5, 8.6, 8.7, 8.9, 9.1, 9.4, 9.6, 9.7, 10.0, 10.9, 12.4); f (7.7, 8.0 [2], 8.1, 8.3 [2], 8.4, 8.6, 8.7, 9.2, 9.3, 9.4 [2], 9.5, 9.6, 10.2 [2], 10.8, 11.2, 11.4)

122m, 85f 2007 m (6.2, 6.3, 6.4, 6.6, 6.7 [3], 6.8 [3], 6.9, 7.0 [3], 7.2 [5], 7.3 [3], 7.4 [2], 7.5 [2], 7.6, 7.7 [2], 7.8 [3], 7.9 [2], 8.1 [6], 8.2 [5], 8.3 [2], 8.4 [3], 8.5 [5], 8.6 [4], 8.7 [2], 8.8 [5], 8.9 [10], 9.0, 9.1 [5], 9.2 [2], 9.3 [2], 9.4 [5], 9.5 [4], 9.6, 9.7 [2], 9.8 [4], 9.9, 10.0, 10.1 [2], 10.2 [2], 10.3 [2], 10.4, 10.5 [2], 10.6 [3], 10.7 [2], 10.8, 10.9, 11.0, 12.4); f (6.8, 7.0, 7.3, 7.5, 7.7, 7.8, 8.0 [2], 8.1, 8.2 [4], 8.3, 8.4 [2], 8.6 [2], 8.9 [2], 9.0 [3], 9.1 [2], 9.2 [4], 9.3 [6], 9.4, 9.5, 9.6, 9.7 [3], 9.9, 10.0 [4], 10.1 [3], 10.2, 10.3, 10.6 [2], 10.7, 10.8, 10.9, 11.1, 11.2 [3], 11.4, 11.6, 11.7 [4], 11.9, 12.1, 12.3 [3], 12.9 [2], 13.3 [2], 13.7, 13.8, 14.0, 14.3, 14.4 [2], 14.5, 15.0, 15.3, 15.4)

*Schizocosa saltatrix* (Hentz, 1844)

1m 2006 m (7.8)

*Varacosa shenandoa* (Chamberlin & Ivie, 1942)

1f 2007 f (9.8)

**Miturgidae**

*Teminius affinis* Banks, 1897

3f 2006 f (15.5, 16.0, 16.2)

1m, 2f 2007 m (12.8); f (10.6, 13.7)

**Mysmenidae**

*Mysmena incredula* (Gertsch & Davis, 1936)

2f 2006 f (0.7, 1.1)

1m 2007 m (0.8)

**Oxyopidae**

*Oxyopes apollo* Brady, 1964

585m, 53f 2006 m (3.0, 3.2, 3.4 [2], 3.5 [4], 3.6 [14], 3.7 [10], 3.8 [11], 3.9 [35], 4.0 [77], 4.1 [87], 4.2 [94], 4.3 [71], 4.4 [74], 4.5 [51], 4.6 [34], 4.7 [11], 4.8 [5], 4.9 [2], 5.0); f (4.1, 4.2, 4.3, 4.4 [2], 4.5 [3], 4.6 [2], 4.7 [4], 4.8, 4.9 [2], 5.0 [2], 5.1 [5], 5.2 [5], 5.3, 5.4 [5], 5.5 [5], 5.6 [4], 5.8 [2], 5.9, 6.0 [2], 6.2, 6.4 [2], 6.8)

224m, 19f 2007 m (3.6 [5], 3.7 [7], 3.8 [12], 3.9 [13], 4.0 [33], 4.1 [24], 4.2 [23], 4.3 [34], 4.4 [24], 4.5 [16], 4.6 [15], 4.7 [10], 4.8 [6], 4.9 [2]); f (4.9 [2], 5.0 [2], 5.1 [2], 5.3, 5.4, 5.5, 5.6, 5.7, 5.8 [2], 6.0, 6.1 [2], 6.4, 6.5, 7.0)

*Oxyopes salticus* Hentz, 1845

17m, 20f 2006 m (3.4, 3.6 [6], 3.7, 3.8 [3], 3.9, 4.0 [2], 4.1 [2], 4.4); f (3.6, 4.1 [3], 4.2 [2], 4.4, 4.5, 4.7 [2], 4.8, 4.9, 5.0 [3], 5.1, 5.2, 5.5, 5.7, 5.8)

17m, 38f 2007 m (4.0, 4.1, 4.3, 4.4, 4.5, 4.7, 4.8 [2], 4.9 [3], 5.0 [4], 5.2, 5.7); f (4.5, 4.6, 4.7, 4.9, 5.0, 5.2, 5.4 [3], 5.5 [2], 5.7 [2], 5.8 [2], 5.9 [3], 6.0 [2], 6.1, 6.2, 6.3 [2], 6.4, 6.5, 6.6 [3], 6.7 [2], 6.9 [2], 7.0 [2], 7.2, 7.4, 8.1)

**Philodromidae**

*Ebo punctatus* Sauer & Platnick, 1972

1m, 2f 2007 m (1.9); f (2.8, 3.1)

*Thanatus formicinus* (Clerck, 1757)

4m 2006 m (4.6, 4.9, 5.5, 6.0)

*Thanatus rubicellus* Mello-Leitão, 1929

1m, 3f 2006 m (5.1); f (5.2, 6.3, 6.7)

*Tibellus duttoni* (Hentz, 1847)

1f 2007 f (8.8)

*Titanebo albocaudatus* (Schick, 1965)

1f 2006 f (3.2)

**Pholcidae**

*Psilochorus utahensis* Chamberlin, 1919

20m, 12f 2006 m (1.3 [2], 1.5 [2], 1.6 [3], 1.7 [2], 1.8, 1.9 [4], 2.0, 2.1 [2], 2.2, 2.3, 2.4); f (1.6 [2], 1.7, 1.9 [3], 2.0 [2], 2.1, 2.2, 2.3, 2.5)

1m 2007 m (2.1)

**Phrurolithidae**

*Phrurotimpus alarius* (Hentz, 1847)

8m 2006 m (1.9 [2], 2.0 [3], 2.1 [2], 2.2)

1m 2007 m (1.8)

*Phrurotimpus borealis* (Emerton, 1911)

3m, 3f 2007 m (2.4 [2], 2.5); f (3.0, 3.3 [2])

*Phrurotimpus certus* Gertsch, 1941

1m 2006 m (1.9)

9m, 3f 2007 m (1.8 [2], 1.9, 2.2 [2], 2.5, 2.6 [2], 2.7); f (2.0, 2.2, 3.7)

*Scotinella fratrella* (Gertsch, 1935)

1m 2007 m (1.4)

**Salticidae**

*Chalcoscirtus diminutus* (Banks, 1896)

1m 2007 m (1.9)

*Habronattus coecatus* (Hentz, 1846)

69m, 27f 2006 m (2.3, 3.8, 3.9 [3], 4.0 [2], 4.1 [2], 4.2 [9], 4.4 [4], 4.5 [6], 4.6 [10], 4.7 [3], 4.8 [3], 4.9 [7], 5.0 [4], 5.1 [4], 5.2 [2], 5.3 [4], 5.4, 5.5, 5.6, 5.9); f (4.6, 4.7, 4.9, 5.1 [2], 5.2, 5.3, 5.6, 5.7, 5.9, 6.0 [2], 6.1, 6.2 [3], 6.3 [3], 6.5 [2], 6.6 [2], 6.8, 7.0 [2], 7.2)

44m, 29f 2007 m (4.3 [2], 4.5 [7], 4.6 [5], 4.7 [2], 4.8 [7], 4.9 [2], 5.0 [6], 5.1 [3], 5.2 [2], 5.5 [2], 5.6 [4], 5.8, 6.1); f (4.5, 5.5, 5.8, 6.1, 6.4, 6.5 [2], 6.6, 6.7, 7.1 [5], 7.2 [2], 7.3 [2], 7.4, 7.5, 7.7, 7.8, 8.0 [2], 8.1, 8.2 [2], 8.3, 8.8)

*Habronattus cognatus* (Peckham & Peckham, 1901)

2m, 1f 2007 m (5.1, 5.3); f (6.8)

*Habronattus decorus* (Blackwall, 1846)

1f 2006 f (6.7)

*Habronattus texanus* (Chamberlin, 1924)

134m, 46f 2006 m (3.1 [2], 3.2 [2], 3.3 [3], 3.4 [11], 3.5 [14], 3.6 [16], 3.7 [13], 3.8 [14], 3.9 [15], 4.0 [14], 4.1 [14], 4.2 [8], 4.3 [4], 4.5 [2], 4.6, 4.7); f (3.8, 3.9, 4.1 [2], 4.2, 4.3 [2], 4.4 [2], 4.5 [3], 4.6 [2], 4.7 [7], 4.8 [4], 4.9 [4], 5.0 [2], 5.1, 5.2 [3], 5.3 [2], 5.4 [3], 5.6 [2], 6.0, 6.3, 6.4, 6.6)

69m, 25f 2007 m (3.4 [2], 3.5 [4], 3.6 [2], 3.7 [3], 3.8 [8], 3.9 [12], 4.0 [7], 4.1 [9], 4.2 [5], 4.3 [5], 4.4 [4], 4.5 [4], 4.6 [2], 4.7, 5.0); f (4.5, 4.8, 4.9 [2], 5.2, 5.3 [4], 5.4, 5.5 [2], 5.6 [4], 5.8, 5.9, 6.0, 6.1 [2], 6.2, 6.4, 7.0, 7.7)

*Habronattus viridipes* (Hentz, 1846)

1f 2007 f (5.8)

*Marpissa lineata* (C. L. Koch, 1846)

2m 2006 m (3.3, 4.3)

*Naphrys pulex* (Hentz, 1846)

1m 2006 m (3.8)

*Pelegrina galathea* (Walckenaer, 1837)

1m 2007 m (4.9)

*Pellenes limatus* Peckham & Peckham, 1901

6m, 3f 2006 m (4.5 [2], 4.7, 4.8, 5.7, 6.2); f (5.8, 6.0, 6.3)

11m 2007 m (4.4, 4.7, 5.1 [3], 5.2, 5.5, 5.7, 5.8, 6.0 [2])

*Phidippus texanus* Banks, 1906

1m 2006 m (9.5)

*Sarinda hentzi* (Banks, 1913)

1m, 1f 2006 m (4.1); f (4.3)

1f 2007 f (4.6)

*Zygoballus rufipes* Peckham & Peckham, 1885

1f 2006 f (2.9)

1m, 3f 2007 m (4.2); f (3.8, 4.0, 4.2)

*Zygoballus sexpunctatus* (Hentz, 1845)

1f 2006 f (3.3)

**Tetragnathidae**

*Glenognatha foxi* (McCook, 1894)

192m, 163f 2007 m (1.3 [2], 1.4 [8], 1.5 [19], 1.6 [29], 1.7 [31], 1.8 [38], 1.9 [41], 2.0 [15], 2.1 [6], 2.2 [3]); f (1.5, 1.6 [14], 1.7 [14], 1.8 [23], 1.9 [21], 2.0 [22], 2.1 [29], 2.2 [20], 2.3 [9], 2.4 [5], 2.5 [3], 2.6 [2])

*Tetragnatha laboriosa* Hentz, 1850

1m 2007 m (4.7)

**Theridiidae**

*Dipoena abdita* Gertsch & Mulaik, 1936

2m 2007 m (1.5, 1.7)

*Theridion australe* Banks, 1899

1f 2007 f (2.9)

*Theridion dilutum* Levi, 1957

1m 2006 m (2.4)

1m 2007 m (2.0)

*Theridion flavonotatum* Becker, 1879

1f 2007 f (2.1)

*Theridion hidalgo* Levi, 1957

1f 2007 f (2.1)

*Theridion murarium* Emerton, 1882

2m 2007 m (1.2, 1.6)

*Theridion rabuni* Chamberlin & Ivie, 1944

1m 2007 m (1.8)

**Thomisidae**

*Mecaphesa celer* (Hentz, 1847)

3m 2007 m (2.9, 3.0, 3.4)

*Xysticus auctificus* Keyserling, 1880

3m 2006 m (3.6, 4.0, 4.7)

2f 2007 f (5.4, 7.1)

*Xysticus concursus* Gertsch, 1934

1f 2007 f (7.0)

*Xysticus funestus* Keyserling, 1880

1f 2006 f (8.0)

*Xysticus gulosus* Keyserling, 1880

1f 2007 f (8.3)

*Xysticus paiutus* Gertsch, 1933

1m 2007 m (5.3)

*Xysticus pellax* O. P.-Cambridge, 1894

8m 2007 m (4.6, 4.9, 5.2, 5.3, 5.4, 5.6 [2], 6.0)

*Xysticus texanus* Banks, 1904

1m 2007 m (4.6)

**Titanoecidae**

*Titanoeca americana* Emerton, 1888

2m 2006 m (5.2, 5.5)

3m, 1f 2007 m (5.4, 5.8, 5.9); f (5.7)

**Trachelidae**

*Meriola decepta* Banks, 1895

5m 2006 m (3.0, 3.3, 3.4, 3.6, 3.8)

9f 2007 f (3.6, 3.7, 3.8, 3.9, 4.0, 4.1, 4.3, 4.5, 4.7)

**Uloboridae**

*Uloborus glomosus* (Walckenaer, 1841)

1m 2006 m (2.9)

#### Attwater Prairie Chicken National Wildlife Refuge (Colorado Co.), 2006–2009

Numbers represent number of species, “x” equals presence. Pitfall traps and sweep net samples were made to determine prey available for the Attwater prairie chicken, an endangered animal. This data is previously unpublished.

**Table A4.** Number of species.

Species	Pitfall Trap	Sweep Net
**Amphinectidae**	1	0
*Metaltella simoni* (Keyserling, 1877)	x	
**Anyphaenidae**	0	2
*Hibana gracilis* (Hentz, 1847)		x
*Hibana velox* (Becker, 1879)		x
**Araneidae**	2	9
*Acanthepeira cherokee* Levi, 1976		x
*Acanthepeira stellata* (Walckenaer, 1805)		x
*Argiope aurantia* Lucas, 1833		x
*Eustala anastera* (Walckenaer, 1841)		x
*Eustala cepina* (Walckenaer, 1841)		x
*Eustala emertoni* (Banks, 1904)		x
*Gea heptagon* (Hentz, 1850)	x	
*Kaira hiteae* Levi, 1977		x
*Larinia directa* (Hentz, 1847)		x
*Neoscona arabesca* (Walckenaer, 1841)	x	x
**Clubionidae**	3	2
*Clubiona abboti* L. Koch, 1866	x	
*Clubiona catawba* Gertsch, 1941	x	x
*Clubiona kiowa* Gertsch, 1941	x	x
**Corinnidae**	3	0
*Castianeira crocata* (Hentz, 1847)	x	
*Castianeira longipalpa* (Hentz, 1847)	x	
*Falconina gracilis* (Keyserling, 1891)	x	
**Eutichuridae**	0	1
*Cheiracanthium inclusum* (Hentz, 1847)		x
**Gnaphosidae**	14	2
*Camillina pulchra* (Keyserling, 1891)	x	
*Cesonia bilineata* (Hentz, 1847)	x	
*Cesonia sincera* Gertsch & Mulaik, 1936		x
*Drassyllus creolus* Chamberlin & Gertsch, 1940	x	
*Drassyllus lepidus* (Banks, 1899)	x	
*Drassyllus texamans* Chamberlin, 1936	x	
*Gnaphosa sericata* (L. Koch, 1866)	x	
*Micaria deserticola* Gertsch, 1933	x	
*Micaria gertschi* Barrows & Ivie, 1942	x	
*Micaria longipes* Emerton, 1890	x	
*Micaria nanella* Gertsch, 1935	x	
*Micaria vinnula* Gertsch & Davis, 1936	x	
*Sergiolus capulatus* (Walckenaer, 1837)		x
*Zelotes hentzi* Barrows, 1945	x	
*Zelotes laccus* (Barrows, 1919)	x	
*Zelotes lasalanus* Chamberlin, 1928	x	
**Hahniidae**	1	0
*Neoantistea mulaiki* Gertsch, 1946	x	
**Linyphiidae**	14	4
*Agyneta chiricahua* Dupérré, 2013	x	
*Agyneta regina* (Chamberlin & Ivie, 1944)	x	
*Agyneta serrata* (Emerton, 1909)	x	
*Ceraticelus similis* (Banks, 1892)	x	x
*Ceratinops latus* (Emerton, 1882)	x	
*Ceratinopsis laticeps* Emerton, 1882	x	
*Erigone autumnalis* Emerton, 1882	x	x
*Grammonota texana* (Banks, 1899)	x	x
*Mermessus bryantae* (Ivie & Barrows, 1935)	x	
*Mermessus denticulatus* (Banks, 1898)	x	
*Mermessus trilobatus* (Emerton, 1882)	x	
*Tennesseellum formicum* (Emerton, 1882)	x	
*Tutaibo anglicanus* (Hentz, 1850)	x	x
*Walckenaeria spiralis* (Emerton, 1882)	x	
**Lycosidae**	13	1
*Hogna antelucana* (Montgomery, 1904)	x	
*Pardosa delicatula* Gertsch & Wallace, 1935	x	
*Pardosa milvina* (Hentz, 1844)	x	
*Pardosa pauxilla* Montgomery, 1904	x	
*Pardosa saxatilis* (Hentz, 1844)	x	
*Pirata hiteorum* Wallace & Exline, 1978	x	
*Pirata sedentarius* Montgomery, 1904	x	
*Pirata seminolus* Gertsch & Wallace, 1935	x	
*Pirata suwaneus* Gertsch, 1940	x	
*Rabidosa rabida* (Walckenaer, 1837)	x	x
*Schizocosa avida* (Walckenaer, 1837)	x	
*Schizocosa bilineata* (Emerton, 1885)	x	
*Trochosa sepulchralis* (Montgomery, 1902)	x	
**Mimetidae**	0	1
*Mimetus hesperus* Chamberlin, 1923		x
**Miturgidae**	1	0
*Teminius affinis* Banks, 1897	x	
**Mysmenidae**	1	0
*Mysmena incredula* (Gertsch & Davis, 1936)	x	
**Nesticidae**	1	0
*Eidmannella pallida* (Emerton, 1875)	x	
**Oxyopidae**	1	3
*Oxyopes apollo* Brady, 1964		x
*Oxyopes salticus* Hentz, 1845	x	x
*Peucetia viridans* (Hentz, 1832)		x
**Philodromidae**	1	4
*Philodromus pratariae* (Scheffer, 1904)		x
*Thanatus formicinus* (Clerck, 1757)		x
*Thanatus rubicellus* Mello-Leitão, 1929	x	x
*Tibellus duttoni* (Hentz, 1847)		x
**Pholcidae**	1	0
*Psilochorus pullulus* (Hentz, 1850)	x	
**Phrurolithidae**	1	0
*Phrurotimpus certus* Gertsch, 1941	x	
**Salticidae**	7	10
*Cheliferoides longimanus* Gertsch, 1936		x
*Colonus puerperus* (Hentz, 1846)		x
*Habronattus coecatus* (Hentz, 1846)	x	x
*Habronattus cognatus* (Peckham & Peckham, 1901)	x	
*Habronattus viridipes* (Hentz, 1846)	x	
*Marpissa lineata* (C. L. Koch, 1846)	x	
*Marpissa pikei* (Peckham & Peckham, 1888)		x
*Neonella vinnula* Gertsch, 1936	x	
*Pelegrina galathea* (Walckenaer, 1837)	x	x
*Sarinda hentzi* (Banks, 1913)	x	x
*Sassacus cyaneus* (Hentz, 1846)		x
*Zygoballus nervosus* (Peckham & Peckham, 1888)		x
*Zygoballus rufipes* Peckham & Peckham, 1885		x
*Zygoballus sexpunctatus* (Hentz, 1845)		x
**Tetragnathidae**	2	1
*Glenognatha foxi* (McCook, 1894)	x	
*Pachygnatha autumnalis* Marx, 1884	x	
*Tetragnatha laboriosa* Hentz, 1850		x
**Theridiidae**	2	4
*Dipoena abdita* Gertsch & Mulaik, 1936	x	
*Dipoena nigra* (Emerton, 1882)	x	
*Steatoda transversa* (Banks, 1898)		x
*Theridion australe* Banks, 1899		x
*Theridion rabuni* Chamberlin & Ivie, 1944		x
*Thymoites expulsus* (Gertsch & Mulaik, 1936)		x
**Thomisidae**	3	6
*Mecaphesa celer* (Hentz, 1847)		x
*Mecaphesa dubia* (Keyserling, 1880)		x
*Misumenoides formosipes* (Walckenaer, 1837)	x	x
*Xysticus apachecus* Gertsch, 1933		x
*Xysticus auctificus* Keyserling, 1880	x	x
*Xysticus funestus* Keyserling, 1880	x	x
**Titanoecidae**	1	
*Titanoeca americana* Emerton, 1888	x	0
Total	73	50

#### Golden Cheeked Warbler Project

Quinn (2000) studied the potential prey of the golden-cheeked warbler, an endangered bird, at two locations near Austin (Travis Co.) in 1993–1994. A total of 12,107 spiders (674 males, 687 females, and 10,746 immatures) were collected from four types of trees mainly by sweeping and beating. This data is previously unpublished.

**Table A5.** Sex collected by tree species.

	*Juniperus ashei*	*Quercus buckleyi*	*Quercus virginiana*	*Ulmus crassifolia*
Anyphaenidae				
*Anyphaena fraterna* (Banks, 1896)		f		f
*Anyphaena pectorosa* L. Koch, 1866			m	m
*Hibana cambridgei* (Bryant, 1931)	mf	mf	mf	mf
*Hibana gracilis* (Hentz, 1847)	mf	mf	mf	mf
*Lupettiana mordax* (O. P.-Cambridge, 1896)	mf	mf	mf	mf
*Wulfila tantillus* Chickering, 1940			m	m
Araneidae				
*Acacesia hamata* (Hentz, 1847)	imm	imm	imm	imm
*Araneus cingulatus* (Walckenaer, 1841)			mf	f
*Araneus cochise* Levi, 1973	mf		mf	
*Araneus detrimentosus* (O. P.-Cambridge, 1889)			m	
*Araneus miniatus* (Walckenaer, 1841)	f	m	mf	mf
*Araneus nashoba* Levi, 1973		f	mf	
*Araneus pegnia* (Walckenaer, 1841)	m	m		
*Araniella displicata* (Hentz, 1847)		m	f	f
*Cyclosa turbinata* (Walckenaer, 1841)		m	f	mf
*Eustala anastera* (Walckenaer, 1841)	m		m	m
*Eustala cepina* (Walckenaer, 1841)		m	f	
*Eustala emertoni* (Banks, 1904)	f	f	f	f
*Gea heptagon* (Hentz, 1850)			m	
*Hypsosinga rubens* (Hentz, 1847)	f			f
*Kaira alba* (Hentz, 1850)			m	
*Mangora maculata* (Keyserling, 1865)		m		
*Mangora placida* (Hentz, 1847)	mf	mf	m	mf
*Mastophora cornigera* (Hentz, 1850)		m	m	m
*Micrathena gracilis* (Walckenaer, 1805)		m		
*Ocrepeira georgia* (Levi, 1976)		m		f
Corinnidae				
*Castianeira amoena* (C. L. Koch, 1841)				f
Dictynidae				
*Dictyna annexa* Gertsch & Mulaik, 1936				f
*Dictyna bellans* Chamberlin, 1919				m
*Dictyna bostoniensis* Emerton, 1888	f			f
*Emblyna callida* (Gertsch & Ivie, 1936)	mf			m
*Emblyna melva* (Chamberlin & Gertsch, 1958)		m	f	m
*Emblyna reticulata* (Gertsch & Ivie, 1936)	mf		m	
*Mallos* sp.	m	f		m
Eutichuridae				
*Cheiracanthium inclusum* (Hentz, 1847)			f	
Linyphiidae				
*Agyneta micaria* (Emerton 1882)		m		
*Agyneta sandia* Dupérré, 2013			f	
*Agyneta serrata* (Emerton, 1909)			m	
*Agyneta tuberculata* Dupérré, 2013	m			
*Erigone autumnalis* Emerton, 1882	f	mf	mf	mf
*Frontinella communis* (Hentz, 1850)	m	m		
*Styloctetor purpurescens* (Keyserling, 1886)	mf	mf	mf	mf
*Tutaibo anglicanus* (Hentz, 1850)	f		f	f
Lycosidae				
*Pardosa pauxilla* Montgomery, 1904	m			
Mimetidae				
*Mimetus notius* Chamberlin, 1923	mf	f	mf	f
Oecobiidae				
*Oecobius navus* Blackwall, 1859			m	
Oxyopidae				
*Hamataliwa grisea* Keyserling, 1887			mf	f
*Oxyopes salticus* Hentz, 1845	f	f	mf	
*Oxyopes scalaris* Hentz, 1845	mf			f
*Peucetia viridans* (Hentz, 1832)	imm		imm	imm
Philodromidae				
*Philodromus keyserlingi* Marx, 1890	mf	mf	f	f
*Philodromus marginellus* Banks, 1901	mf		mf	m
*Philodromus minutus* Banks, 1892		f	f	f
*Philodromus placidus* Banks, 1892		f	f	f
*Philodromus vulgaris* (Hentz, 1847)	f	f	mf	m
*Tibellus duttoni* (Hentz, 1847)				m
Salticidae				
*Admestina archboldi* Piel, 1992	f	f	f	f
*Colonus sylvanus* (Hentz, 1846)		mf	f	mf
*Eris militaris* (Hentz, 1845)	mf	f	mf	mf
*Hentzia mitrata* (Hentz, 1846)		mf	mf	mf
*Hentzia palmarum* (Hentz, 1832)		m	mf	mf
*Maevia inclemens* (Walckenaer, 1837)		m		
*Peckhamia americana* (Peckham & Peckham, 1892)	m	mf	f	f
*Pelegrina galathea* (Walckenaer, 1837)				m
*Pelegrina pervaga* (Peckham & Peckham, 1909)	mf	f		
*Phidippus mystaceus* (Hentz, 1846)				f
*Zygoballus rufipes* Peckham & Peckham, 1885			f	
Tetragnathidae				
*Leucauge venusta* (Walckenaer, 1841)		m		
*Tetragnatha laboriosa* Hentz, 1850	m	mf		m
Theridiidae				
*Anelosimus studiosus* (Hentz, 1850)		m	m	m
*Chrosiothes jocosus* (Gertsch & Davis, 1936)	m			
*Dipoena nigra* (Emerton, 1882)		m		mf
*Latrodectus mactans* (Fabricius, 1775)			m	
*Neospintharus furcatus* (O. P.-Cambridge, 1894)	m			m
*Phoroncidia americana* (Emerton, 1882)	imm	imm	imm	imm
*Phycosoma lineatipes* (Bryant, 1933)	mf	mf	mf	f
*Rhomphaea projiciens* O. P.-Cambridge, 1896	m	m		m
*Theridion dilutum* Gertsch & Archer, 1942	m	m	m	m
*Theridion flavonotatum* Becker, 1879			f	f
*Theridion hidalgo* Levi, 1957	mf	mf	mf	mf
*Theridion murarium* Emerton, 1882	mf		mf	mf
*Theridion positivum* Chamberlin, 1924				m
*Thymoites expulsus* (Gertsch & Mulaik, 1936)	mf			
*Tidarren haemorrhoidale* (Bertkau, 1880)	m			
*Wamba crispulus* (Simon, 1895)	mf	mf	mf	mf
*Yunohamella lyrica* (Walckenaer, 1841)	mf	mf	mf	mf
Thomisidae				
*Mecaphesa asperata* (Hentz, 1847)	f	mf	mf	m
*Mecaphesa californica* (Banks, 1896)	m			m
*Mecaphesa celer* (Hentz, 1847)		mf	mf	mf
*Mecaphesa coloradensis* (Gertsch, 1933)		f	f	
*Misumessus oblongus* (Keyserling, 1880)		m	m	m
*Tmarus angulatus* (Walckenaer, 1837)	f	mf	mf	m
*Tmarus rubromaculatus* Keyserling, 1880		mf		
*Xysticus locuples* Keyserling, 1880	f			
Trachelidae				
*Trachelas mexicanus* Banks, 1898		mf	f	mf
*Trachelas volutus* Gertsch, 1935		f	f	f
Uloboridae				
*Uloborus glomosus* (Walckenaer, 1841)	m			f

**Table A6.** Number of specimens by family.

Family	Number Male	Number Female	Number Immature
Anyphaenidae	66	64	2,461
Araneidae	75	71	1,536
Corinnidae	0	1	0
Dictynidae	17	18	66
Eutichuridae	0	2	21
Gnaphosidae	0	0	56
Hahniidae	0	0	1
Linyphiidae	77	145	364
Lycosidae	1	0	20
Mimetidae	6	14	216
Oecobiidae	1	0	0
Oxyopidae	7	21	787
Philodromidae	15	37	906
Salticidae	95	146	1,468
Tetragnathidae	4	1	129
Theridiidae	191	127	1,041
Thomisidae	115	26	1,591
Trachelidae	2	12	66
Uloboridae	2	2	17
Total	674	687	10,746

### Species from Various Elevations in Texas Counties

Atascosa

106 meters *Eucteniza relata* (O. P.-Cambridge, 1895)

140 meters *Eucteniza relata* (O. P.-Cambridge, 1895)

Bastrop

125 meters *Eucteniza relata* (O. P.-Cambridge, 1895)

168 meters *Eucteniza relata* (O. P.-Cambridge, 1895)

Bell

221 meters *Eucteniza relata* (O. P.-Cambridge, 1895)

Bexar

198 meters *Eucteniza relata* (O. P.-Cambridge, 1895)

199 meters *Eucteniza relata* (O. P.-Cambridge, 1895)

Blanco

450 meters *Agelenopsis aperta* (Gertsch, 1934)

Brewster (Big Bend National Park)

5400 feet *Zorocrates unicolor* (Banks, 1901)

5600 feet *Euagrus chisoseus* Gertsch, 1939

5900 feet *Zorocrates unicolor* (Banks, 1901)

6000 feet *Entychides arizonicus* Gertsch & Wallace, 1936; *Euagrus chisoseus* Gertsch, 1939; *Orthonops lapanus* Gertsch & Mulaik, 1940; *Zorocrates unicolor* (Banks, 1901)

7000 feet *Euagrus chisoseus* Gertsch, 1939

Brewster

1212 meters *Eucteniza ronnewtoni* Bond & Godwin, 2013

1235 meters *Agelenopsis aperta* (Gertsch, 1934)

Cameron (Laguna Atascosa National Wildlife Refuge)

2 meters *Hibana futilis* (Banks, 1898); *Drassyllus dromeus* Chamberlin, 1922; *Eilica bicolor* Banks, 1896; *Eidmannella pallida* (Emerton, 1875)

Cameron (Sabal Palm Audubon Sanctuary)

3 meters *Miagrammopes mexicanus* O. P.-Cambridge, 1893; *Zorocrates alternatus* Gertsch & Davis, 1936

Cameron (Lower Rio Grande Valley National Wildlife Refuge)

5 meters *Cesonia bilineata* (Hentz, 1847); *Oxyopes acleistus* Chamberlin, 1929

Crosby

3200 feet *Aphonopelma arnoldi* Smith, 1995

Culberson

3500 feet *Phidippus tyrannus* Edwards, 2004

5200 feet *Euagrus chisoseus* Gertsch, 1939

5470 feet *Pardosa xerophila* Vogel, 1964; *Pirata sedentarius* Montgomery, 1904; *Misumenoides formosipes* (Walckenaer, 1837)

1760 meters *Mallos blandus* Chamberlin & Gertsch, 1904

Dimmit

166 meters *Eucteniza relata* (O. P.-Cambridge, 1895)

Duval

186 meters *Eucteniza relata* (O. P.-Cambridge, 1895)

El Paso

5300 feet *Habronattus virgulatus* Griswold, 1987

Hays

1340 feet *Anyphaena dixiana* (Chamberlin & Woodbury, 1929); *Hibana cambridgei* (Bryant, 1931); *Orthonops lapanus* Gertsch & Mulaik, 1940; *Cesonia bilineata* (Hentz, 1847); *Drassyllus aprilinus* (Banks, 1904); *Drassyllus dromeus* Chamberlin, 1922; *Drassyllus orgilus* Chamberlin, 1922; *Drassyllus texamans* Chamberlin, 1936; *Gnaphosa fontinalis* Keyserling, 1887; *Herpyllus ecclesiasticus* Hentz, 1832; *Sergiolus cyaneiventris* Simon, 1893; *Zelotes aiken* Platnick & Shadab, 1983; *Zelotes lasalanus* Chamberlin, 1928; *Hahnia flaviceps* Emerton, 1913; *Oxyopes acleistus* Chamberlin, 1929; *Metacyrba floridana* Gertsch, 1934; *Ariadna bicolor* (Hentz, 1842); *Bassaniana versicolor* (Keyserling, 1880); *Xysticus ferox* (Hentz, 1847); *Xysticus paiutus* Gertsch, 1933

Hidalgo (Santa Ana National Wildlife Refuge)

10 meters *Wulfila bryantae* Platnick, 1974; *Drassyllus texamans* Chamberlin, 1936; *Eilica bicolor* Banks, 1896; *Zelotes pseustes* Chamberlin, 1922; *Hamataliwa grisea* Keyserling, 1887; *Oxyopes acleistus* Chamberlin, 1929; *Stemmops bicolor* O. P.-Cambridge, 1894; *Mecaphesa dubia* (Keyserling, 1880)

29 meters *Eucteniza relata* (O. P.-Cambridge, 1895)

43 meters *Eucteniza relata* (O. P.-Cambridge, 1895)

Houston

122 meters *Eucteniza relata* (O. P.-Cambridge, 1895)

Jeff Davis (Davis Mountains Resort)

5800 feet *Xysticus funestus* Keyserling, 1880

6180 feet *Schizocosa saltatrix* (Hentz, 1844); *Trochosa sepulchralis* (Montgomery, 1902)

6240 feet *Pardosa falcifera* F. O. P.-Cambridge, 1892; *Pardosa vadosa* Barnes, 1959; *Schizocosa saltatrix* (Hentz, 1844)

Jeff Davis

4850 feet *Pardosa falcifera* F. O. P.-Cambridge, 1892

5800 feet *Euagrus chisoseus* Gertsch, 1939

1500 meters *Lathys delicatula* (Gertsch & Mulaik, 1936); *Drassyllus aprilinus* (Banks, 1904)

1524 meters *Lathys delicatula* (Gertsch & Mulaik, 1936); *Drassyllus aprilinus* (Banks, 1904)

Kenedy

20 meters *Stemmops bicolor* O. P.-Cambridge, 1894; *Xysticus furtivus* Gertsch, 1936

154 meters *Eucteniza relata* (O. P.-Cambridge, 1895)

Kendall

429 meters *Eucteniza relata* (O. P.-Cambridge, 1895)

Kerr

1960 feet *Anyphaena dixiana* (Chamberlin & Woodbury, 1929); *Anyphaena fraterna* (Banks, 1896); *Orthonops lapanus* Gertsch & Mulaik, 1940; *Elaver excepta* (L. Koch, 1866); *Leptoctenus byrrhus* Simon, 1888; *Cesonia bilineata* (Hentz, 1847); *Drassyllus aprilinus* (Banks, 1904); *Gnaphosa fontinalis* Keyserling, 1887; *Haplodrassus signifer* (C. L. Koch, 1839); *Ariadna bicolor* (Hentz, 1842); *Bassaniana versicolor* (Keyserling, 1880); *Xysticus apachecus* Gertsch, 1933; *Xysticus ferox* (Hentz, 1847); *Xysticus funestus* Keyserling, 1880

546 meters *Eucteniza relata* (O. P.-Cambridge, 1895)

Kleberg

18 meters *Eucteniza relata* (O. P.-Cambridge, 1895)

La Salle

110 meters *Eucteniza relata* (O. P.-Cambridge, 1895)

Midland

848 meters *Eucteniza relata* (O. P.-Cambridge, 1895)

Nueces

7 meters *Poultonella nuecesensis* Cokendolpher & Horner, 1978

21 meters *Eucteniza relata* (O. P.-Cambridge, 1895)

Pecos

970 meters *Agelenopsis aperta* (Gertsch, 1934)

Presidio (Big Bend Ranch State Park)

3591 feet *Hentzia alamosa* Richman, 2010

Presidio (Dalquest Research Site)

1267 meters *Drassyllus broussardi* Platnick & Horner, 2007

Presidio

4360 feet *Argiope trifasciata* (Forskål, 1775); *Micaria longipes* Emerton, 1890

Randall

630 meters *Agelenopsis aperta* (Gertsch, 1934)

Sabine

58 meters *Eucteniza relata* (O. P.-Cambridge, 1895)

San Patricio (Welder Wildlife Refuge)

5 meters *Xysticus ferox* (Hentz, 1847)

20 meters *Micaria nanella* Gertsch, 1935; *Oxyopes salticus* Hentz, 1845; *Xysticus auctificus* Keyserling, 1880

San Patricio

10 meters *Eucteniza relata* (O. P.-Cambridge, 1895)

Starr (Lower Rio Grande Valley National Wildlife Refuge)

20 meters *Cesonia bilineata* (Hentz, 1847); *Drassyllus dromeus* Chamberlin, 1922; *Eilica bicolor* Banks, 1896; *Oxyopes acleistus* Chamberlin, 1929; *Ariadna bicolor* (Hentz, 1842)

Starr

200 feet *Euagrus comstocki* Gertsch, 1935

58 meters *Eucteniza relata* (O. P.-Cambridge, 1895)

135 meters *Eucteniza relata* (O. P.-Cambridge, 1895)

Sutton

2200 feet *Araneus pegnia* (Walckenaer, 1841); *Phidippus apacheanus* Chamberlin & Gertsch, 1929; *Phidippus mystaceus* (Hentz, 1846)

647 meters *Eucteniza relata* (O. P.-Cambridge, 1895)

Taylor

2300 feet *Phidippus pruinosus* Peckham & Peckham, 1909

Tom Green

560 meters *Agelenopsis aperta* (Gertsch, 1934)

Travis

148 meters *Eucteniza relata* (O. P.-Cambridge, 1895)

152 meters *Eucteniza relata* (O. P.-Cambridge, 1895)

153 meters *Eucteniza relata* (O. P.-Cambridge, 1895)

168 meters *Eucteniza relata* (O. P.-Cambridge, 1895)

183 meters *Eucteniza relata* (O. P.-Cambridge, 1895)

Uvalde (Garner State Park)

1400 feet *Hypsosinga funebris* (Keyserling, 1892); *Kaira alba* (Hentz, 1850); *Mangora fascialata* Franganillo, 1936; *Mangora gibberosa* (Hentz, 1847); *Mimetus notius* Chamberlin, 1923; *Oxyopes apollo* Brady, 1964; *Oxyopes salticus* Hentz, 1845; *Colonus puerperus* (Hentz, 1846); *Eris militaris* (Hentz, 1845); *Hentzia mitrata* (Hentz, 1846); *Marpissa pikei* (Peckham & Peckham, 1888); *Pelegrina galathea* (Walckenaer, 1837); *Phidippus pius* Scheffer, 1905; *Sarinda hentzi* (Banks, 1913); *Zygoballus rufipes* Peckham & Peckham, 1885; *Chrosiothes jocosus* (Gertsch & Davis, 1936); *Euryopis quinquemaculata* Banks, 1900; *Hentziectypus globosus* (Hentz, 1850); *Theridion dilutum* Levi, 1957; *Theridion dividuum* Gertsch & Archer, 1942; *Theridion hidalgo* Levi, 1957; *Theridion murarium* Emerton, 1882; *Theridion positivum* Chamberlin, 1924; *Thymoites expulsus* (Gertsch & Mulaik, 1936); *Wamba crispulus* (Simon, 1895); *Mecaphesa celer* (Hentz, 1847); *Mecaphesa dubia* (Keyserling, 1880); *Synema viridans* (Banks, 1896); *Uloborus glomosus* (Walckenaer, 1841)

Val Verde (Seminole Canyon State Park)

1400 feet *Colphepeira catawba* (Banks, 1911); *Hypsosinga funebris* (Keyserling, 1892); *Metepeira comanche* Levi, 1977; *Diguetia albolineata* (O. P.-Cambridge, 1895); *Oxyopes apollo* Brady, 1964; *Oxyopes lynx* Brady, 1964; *Oxyopes salticus* Hentz, 1845; *Tibellus duttoni* (Hentz, 1847); *Marpissa pikei* (Peckham & Peckham, 1888); *Pelegrina galathea* (Walckenaer, 1837); *Sassacus papenhoei* Peckham & Peckham, 1895; *Euryopis texana* Banks, 1908; *Theridion dilutum* Levi, 1957; *Theridion hidalgo* Levi; *Theridion llano* Levi, 1957; *Thymoites expulsus* (Gertsch & Mulaik, 1936); *Mecaphesa celer* (Hentz, 1847); *Mecaphesa dubia* (Keyserling, 1880)

Val Verde

180 meters *Agelenopsis aperta* (Gertsch, 1934)

396 meters *Eucteniza ronnewtoni* Bond & Godwin, 2013

Ward

808 meters *Eucteniza relata* (O. P.-Cambridge, 1895)

Webb

213 meters *Eucteniza relata* (O. P.-Cambridge, 1895)

Wichita

300 meters *Titanebo redneri* (Cokendolpher, 1978)

Zapata

350 meters *Eucteniza relata* (O. P.-Cambridge, 1895)
